# An Unusual Benzoisoquinoline-9-one Derivative and Other Related Compounds with Antiproliferative Activity from Hawaiian Endophytic Fungus *Peyronellaea* sp. FT431

**DOI:** 10.3390/molecules24010196

**Published:** 2019-01-07

**Authors:** Chunshun Li, Ariel M. Sarotti, Xiaohua Wu, Baojun Yang, James Turkson, Yongfei Chen, Qingsong Liu, Shugeng Cao

**Affiliations:** 1 Department of Pharmaceutical Sciences, Daniel K. Inouye College of Pharmacy, University of Hawai'i at Hilo, 200 West Kawili Street, Hilo, HI 96720, USA; chunshun@hawaii.edu (C.L.); xiaohua3@hawaii.edu (X.W.); 2 Cancer Biology Program, University of Hawaii Cancer Center, 701 Ilalo Street, Honolulu, HI 96813, USA; byang@cc.hawaii.edu (B.Y.); jturkson@cc.hawaii.edu (J.T.); 3 Instituto de Química Rosario (CONICET), Facultad de Ciencias Bioquímicas y Farmacéuticas, Universidad Nacional de Rosario, Suipacha 531, Rosario 2000, Argentina; sarotti@iquir-conicet.gov.ar; 4 High Magnetic Field Laboratory, Key Laboratory of High Magnetic Field and Ion Beam Physical Biology, Hefei Institute of Physical Science, Chinese Academy of Sciences, Hefei 230031, China; Chenyf@hmfl.ac.cn (Y.C.); qsliu97@hmfl.ac.cn (Q.L.)

**Keywords:** endophytic fungi, *Peyronellaea*, benzoisoquinoline-9-one, antiproliferative, Hawaii

## Abstract

A new polyketide containing the benzoisoquinoline-9-one moiety, peyronetide A (**1**), and three other new derivatives peyronetides B–D (**2**–**4**), as well as one known compound (**5**) were purified from the cultured broth of the endophytic fungus *Peyronellaea* sp. FT431, which was isolated from the Hawaiian indigenous plant, *Verbena* sp. The structures of the new compounds were determined through the analysis of HRMS and NMR spectroscopic data. Compounds **1**, **2**, and **5** showed cytotoxic activities against TK-10 (human kidney adenocarcinoma cells), cisplatin sensitive A2780S (human ovarian carcinoma cells), and cisplatin resistant A2780CisR cell lines, with IC_50_ values between 6.7 to 29.2 μM.

## 1. Introduction

Endophytic fungi are wonderful producers of various secondary metabolites, which have attracted great interest in the past decades to identify structurally unique and biologically active small molecules [1,2,3,4,5,6]. Our previous investigation of Hawaiian endophytic fungi had led to the identification of many new and/or bioactive compounds [7,8,9,10,11,12,13,14,15,16,17,18], including verbenanone from *Peyronellaea* sp. FT431 [13]. The crude extract of FT431 showed antiproliferative activity at 20 μg/mL against the A2780 cancer cell line, but verbenanone was inactive, so we decided to study FT431 further to identify the antiproliferative compounds.

**Figure 1:**
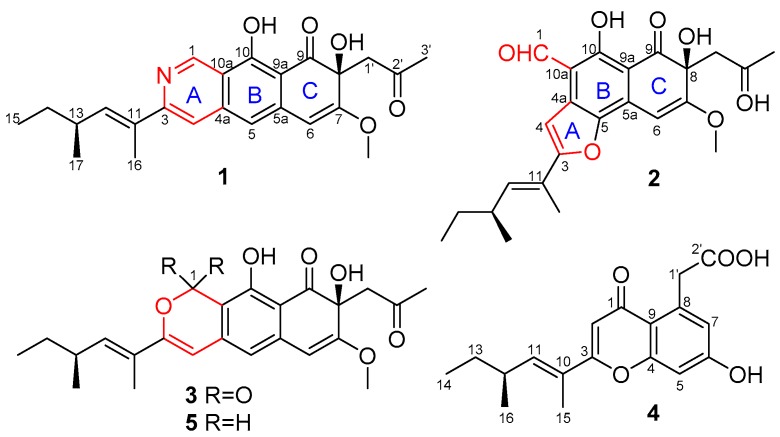
Structures of compounds **1**–**5**.

The fermented whole broth (4.5 L) was filtered through filter paper to separate the supernatant from the mycelia. The latter was extracted with 80% acetone/H_2_O (×3), and the extract was concentrated under reduced pressure to afford an aqueous solution. The aqueous solution was passed through HP-20 eluted with MeOH-H_2_O (10%, 50%, 90%, 100%) to afford four fractions (Fr. A–D). The active fraction (Fr. C) was further separated by preparative HPLC and semi-preparative HPLC to get compounds **1**–**5** (Figure 1). Three of them (**1**, **2**, and **5**) showed antiproliferative activity against different cancer cell lines. Herein, we report the isolation, structure elucidation, and bioactivities of these isolated compounds.

## 2. Results and Discussions

**Figure 2:**
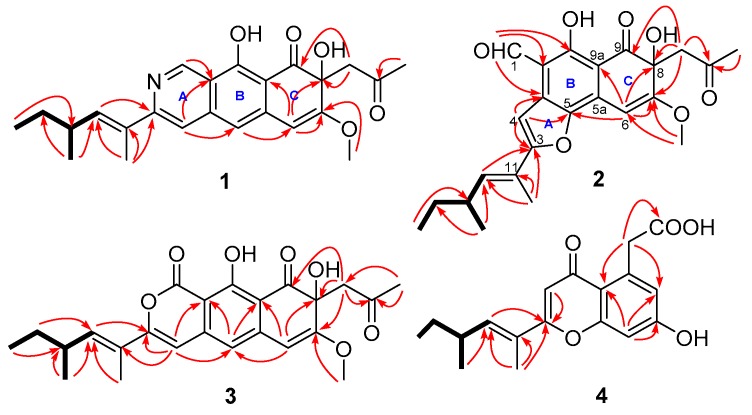
dqfCOSY (bolds) and selected HMBC (single head arrows, red) correlations of compounds **1**–**4**.

Compound **1** was isolated as a brown solid. Its molecular formula, C_24_H_27_NO_5_, was determined by HRESIMS (High-Resolution Electrospray Ionization Mass Spectrometry) at *m*/*z* 410.1964 [M + H]^+^ (calcd 410.1968), requiring 12 degrees of unsaturation. A comprehensive analysis of the 1D and 2D NMR spectra indicated the presence of four methyls, one methoxy group, two methylenes, six methines (five olefinic or aromatic), and eleven carbons with no hydrogen connected, including two ketones (δ_C_ 205.7, 202.8) (Table 1). The spin systems, C-12‒C-13(C-17)‒C-14‒C-15, were established by the ^1^H-^1^H COSY spectrum as shown in Figure 2, which was also verified by the corresponding HMBC correlations from H_3_-17 to C-12 and C-14, and from H_3_-15 to C-13. Meanwhile, HMBC correlations from the singlet methyl H_3_-16 (δ_H_ 2.16) to C-11 and C-12, C-3, as well as from H-12 to C-3 implied that the side-chain CH_3_-CH_2_-CH(CH_3_)-CH=C(CH_3_)- was connected to the ring system at C-3. HMBC correlations from the methyl group H_3_-3′ to the ketone C-2′ (δ_C_ 205.7) and methylene C-1′ (δ_C_ 50.6), and from H_2_-1′ to the oxygenated aromatic carbon C-7 (δ_C_ 161.6), an oxygenated quaternary C-8 (δ_C_ 72.8), and a ketone C-9 (δ_C_ 202.8) indicated the presence of another side-chain C-1′−C-3′, which was connected to C-8. The only methoxy group was assigned at 7-position by an HMBC correlation from the methoxy group to C-7. In addition, the specific de-shielded aromatic methine resonating at δ_H_ 9.43/δ_C_ 148.4 implied that it should be a nitrogenated atom. HMBC correlations from H-1 to C-4a, C-10, and C-10a, and from the aromatic proton H-4 to C-3, C-5, and C-10a suggested the presence of an isoquinoline ring system (rings A and B). Moreover, the HMBC correlations from H-5 to C-6, and from H-6 to the oxygenated olefinic carbon C-7, C-8, and C-9a established the naphthalen-1-(2*H*)-one rings B and C, and rings A–C were linearly aligned to form a benzoisoquinoline-9-one moiety (rings A–C) as shown. Literature research indicated that compound **1** had a similar ring system to the compound *O*-dihydroquinone (**5**) that was obtained as an epimer mixture from a marine ascomycete strain, which was the only report of this type of compound [19]. In spite of this, the presence of the nitrogen atom at 2-position in compound **1** instead of the oxygen in that of the reported compound was unusual. The configuration of the double bond C11(12) on the side-chain was determined to be E by the NOE correlations between H_3_-16 and H_3_-17. Hence, the planar structure of **1** was determined as shown.

Compound **2** was isolated as a brown solid. Its molecular formula was determined to be C_24_H_26_O_7_ by HRESIMS at *m*/*z* 427.1751 [M + H]*^+^* (calcd 427.1757), with 12 degrees of unsaturation. A comprehensive analysis of the 1D and 2D NMR spectra indicated the presence of four methyls, one methoxy group, two methylenes, five methines (including one aldehyde), and twelve carbons with no hydrogen connected, including two ketones (δ_C_ 206.5, 202.6) and four oxygenated aromatic carbons (Table 1). The ^1^H-^1^H COSY implied that **2** had the same spin system as **1**, which was verified by HMBC correlations as shown in Figure 2. The similarity of the NMR data of **2** to those of **1** implied that both had similar moieties. The key HMBC correlations from H_3_-3′ and H_2_-1′ to the ketone at δ_C_ 206.5, from H_2_-1′ to C-7, C-8, and C-9, and from H-6 to C-8, C-9a, and C-5, as well as from the methoxy group to C-7 implied the presence of ring C and the same substituents at 7- and 8-positions as those of **1**. Moreover, the HMBC correlations from the proton of the aldehyde proton H-1 (δ_H_ 10.52 ppm) to C-10, C-10a, and C-4a placed the aldehyde group at C-10a, which implied that ring A in the molecule of **1** was opened in **2**. A combined analysis of the observed HMBC correlations from H-4 to C-4a, and to the two oxygenated carbons C-3 and C-5 suggested the formation of a furan ring (ring A) as shown. The side-chain at 3-position was the same as that of **1**. The configuration of the double bond was assigned as E at 11(12)-position by NOESY spectrum. Hence, the planar structure of **2** was determined as shown.

Compound **3** was isolated as a brown solid. The positive HRESIMS quasi-molecular ion peak at *m*/*z* 427.1760 [M + H]^+^ (calcd 427.1757) suggested the molecular formula of **3** as C_24_H_26_O_7_, which was same as that of compound **2**. A comprehensive comparison of the NMR data (Table 2) of **3** with those of **1** indicated that the main difference was the absence of the nitrogenated methine (-N=CH-) and the presence of a lactone carbonyl group (-O-CO-) at δ_C_ 164.8 in **3**. The configuration of the double bond at C-11 was assigned as E by NOESY spectrum. Hence, the planar structure of **3** was determined as shown. 

Compound **4**, a brown solid, was determined to have a molecular formula of C_18_H_20_O_5_ by HRESIMS at *m*/*z* 317.1390 [M + H]^+^ (calcd 317.1389), which was six carbons less than those of compounds **1**–**3**. ^1^H-^1^H COSY and HMBC spectra suggested that **4** had the same side-chain (C-10–C-16) as compounds **1**–**3** (C-11–C-17). A comprehensive analysis of the NMR data (Table 2), and especially the HMBC spectrum, implied that **4** was a chromone derivative, which has the same ring system as that of 2-methyl-5-carboxymethyl-7-hydroxychromone [20]. The main difference between **4** and 2-methyl-5-carboxymethyl-7-hydroxychromone was the long side-chain instead of a methyl group in the molecule of **4**. HMBC correlations from H-2 to C-3 and C-10, and from the olefinic H-10 and single methyl H_3_-15 to C-3 confirmed the position of the side-chain. The E configuration of the double bond was determined by NOESY spectrum. Hence, the planar structure of **4** was determined as shown.

The known compound **5** (*O*-dihydroquinone) was identified by comparison of its physical data with reported values in the literature [19].

We tried to determine the configuration of the new compounds including chemical reactions and crystallization, but it was unsuccessful. Then we purchased both (*S*)-(+)- and (*R*)-(−)- 2-methylbutanoic acids, and compared the optical rotation of **4** with those of (*S*)-(+)- and (*R*)-(−)- 2-methylbutanoic acids. Compound **4** showed a positive sign of optical rotation, indicating that **4** should also have an S configuration at 13-position. Hence, assuming a 13-S configuration in compounds **1**–**3**, the relative configuration of the 8-position remained unknown. To solve this task, we relied on GIAO ^13^C-NMR calculations, a strategy that has been extensively employed in recent publications to settle structural and stereochemical issues of complex organic molecules [21,22,23,24,25]. Several strategies have been developed to determine the most likely stereostructure among several candidates, including DP4, [22] and DP4+, an updated version of DP4 including scaled and unscaled NMR shifts computed at higher levels of theory [23]. The capacity of these methodologies to discriminate among candidates featuring rigid structures and contiguous or near-by stereocenters is often excellent [24], but when two or more steroclusters are separated the determination of the relative configuration becomes much more challenging [25]. In any case, we decided to explore this approach to suggest a sound stereochemical assignment of the new natural products herein reported and to validate our assignment of the planar structure of **1** discussed above. Initially, we carried out preliminary DP4 calculations of the two possible diastereoisomers of **1** (**1a** = **1**–**8S,13S** and **1b = 1**–**8S,13R** [equivalent to **1**–**8R,13S**], see structures of **1a** and **1b** in Supplementary Materials) at the affordable B3LYP/6-31G**//MMFF level of theory [21b]. As shown in the Supporting Information, compound **1a** displayed a slightly better fit between experimental and calculated NMR data, and was identified by DP4 as the most probable candidate (55% for **1a** and 45% for **1b**). Most of the calculated shifts agreed well with our experimental values, providing further evidence of our proposed connectivity analysis. However, we noticed alarmingly high errors (defined as Δδ = abs[δ_exp_ − δ_calc_]) in the signals assigned to C-8 (Δδ = 8.2 ppm), C-1′ (Δδ = 9.9 ppm), and C-2′ (Δδ = 9.1 ppm). After a detailed examination of the computational data, we noticed that such discrepancies arose from the conformations bearing intramolecular H-bonding between the OH group at C-8 with the ketone oxygen at C-2′, which in turn represented > 93% of the corresponding Boltzmann distributions according to the B3LYP/6-31G** energies. However, since the experimental NMR data were collected in acetone-*d*_6_, the real conformational landscape of the system might be shifted toward more flexible structures. Hence, following a similar approach recently employed in a related situation [14], we recomputed the NMR shifts by neglecting all conformations featuring intramolecular H-bonding between C_8_-OH and C_2′_=O. In excellent agreement with our hypothesis, a much better fit was computed for C-8 (Δδ = 2.5 ppm), C-1′ (Δδ = 1.9 ppm), C-2′ (Δδ = 2.2 ppm), and H-1′ (Δδ = 0.3 ppm). Nevertheless, the slight preference to **1a** (52%) remained almost constant. We next refined the computational results by performing full geometry optimizations at the B3LYP/6-31G* level of theory followed by NMR calculations at the PCM/mPW1PW91/6-31+G** level, the recommended method for DP4+ calculations [21c–e]. Here again, the conformational preferences of **1a** and **1b** were considerably shifted toward intramolecular H-bonded structures. As expected, strong deviations from the experimental values were computed for the ^13^C-NMR resonances of C-8, C-1′, and C-2′ (Δδ = 5.1 − 11.8 ppm). Since the Boltzmann distributions hardly changed upon performing full geometry optimizations in water, we decided to recompute the NMR data by removing all the conformations showing H-bonding. In this reduced system, a much better agreement between experimental and calculated NMR data was observed, with a slight preference toward **1a** (CMAE = 1.5 ppm for **1a** and 1.6 ppm for **1b**). As a result, the DP4+ values identified **1a** as the most probable candidate (69%), in line with the previous DP4 results. From a biogenetic point of view, **2** and **3** should have the same configuration as **1** at the corresponding chiral centers. However, given the separation of the two stereocenters, the other relative configurations cannot be completely ruled out.

**Figure 3:**
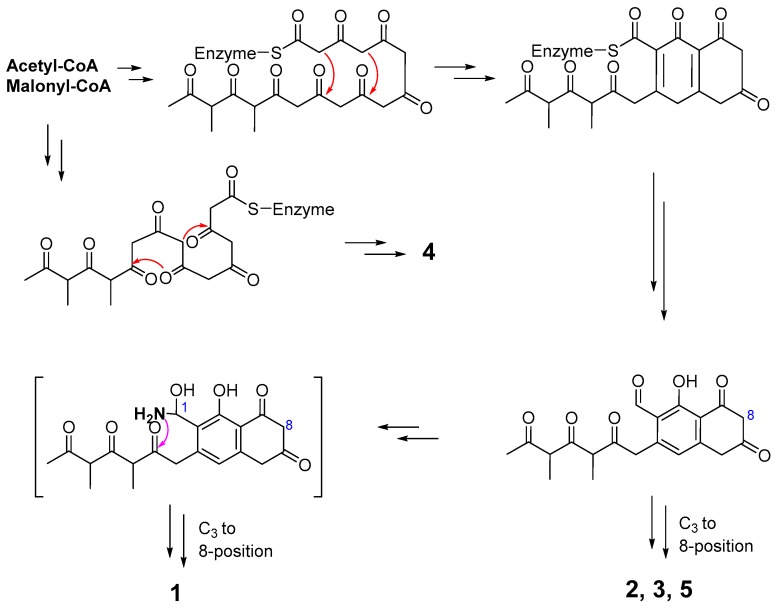
Proposed biosynthesis of compounds **1**–**5**.

Biogenetically, all the new compounds could be derived from acetyl CoA and malonyl CoA. However, it is worthy to investigate how the nitrogen atom was introduced [26] into 1-position of compound **1**, and the C_3_ side-chain to 8-position of compounds **1**–**3** and **5** (Figure 3). 

**Figure 4:**
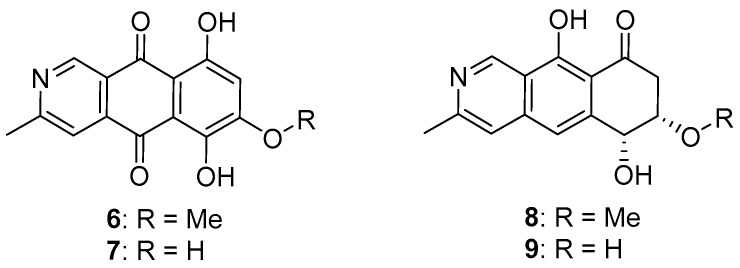
Some known natural azaanthraquinones and benzo-isoquinoline-9-one derivatives.

Natural azaanthraquinone derivatives were not rare, for example, bostrycoidin (**6**) and tolypocladin (**7**) [27,28]. However, natural products containing a benzoisoquinoline-9-one moiety are very uncommon. To the best of our knowledge, pyrenolines A (**8**) [29,30] and B (**9**) [30] were the only two known benzoisoquinoline-9-one derivatives (Figure 4) that were isolated from the culture fluid of *Pyrenophora teres*, a pathogen of barley.

All the compounds were tested against ovarian cancer cell lines A2780S and A2780CisR, and renal cancer cell TK-10. Compounds **1**, **2**, and **5** were active, and compound **5** showed moderate activities against those cell lines (Table 3, see anti-proliferative data in Supplementary Materials). 

Cisplatin had IC_50_ values of 0.36, 1.1, and 13.2 μM against A2780S, A2780CisR, and TK-10, respectively.

## 3. Materials and Methods

### 3.1. General Experimental Procedures

Optical rotation was measured with a Rudolph Research Analytical AutoPol IV Automatic Polarimeter (Hackettstown, NJ, USA). UV and IR spectra were obtained with a Shimadzu UV-1800 spectrophotometer (Kyoto, Japan) and a Thermo Fisher Scientific Nicolet iS50 FTIR spectrometer (Madison, WI, USA), respectively. NMR spectra including 1D and 2D experiments were recorded in acetone-*d*_6_ or MeOH-*d*_4_ on a Bruker 400 MHz NMR (Fällanden, Switzerland). High resolution mass spectra were obtained on a Waters Micromass Q-Tof Ultima ESI-TOF mass spectrometer (Milford, MA, USA), or an Agilent Technologies 6530 Accurate-Mass Q-TOF LC/MS (Santa Clara, CA, USA). HPLC was carried out on a Thermo Fisher Scientific Ultimate 3000 LC system (Germering, Germany), and all solvents were HPLC grade. Column chromatography used a Diaion HP-20 (Alfa Aesar, Ward Hill, MA, USA).

### 3.2. Isolation and Identification of Fungal Strain 

The fungal strain was isolated on PDA medium from a healthy leaf of a Hawaiian indigenous plant, *Verbena* sp., which was collected in the Lyon Botanical Garden in 2014. The strain FT431 was identified as *Peyronellaea* sp. based on the analysis of the DNA sequence of the nuclear ribosomal internal transcribed spacer, which has been deposited in GenBank with the accession no. KY971272. A voucher specimen was deposited at the Daniel K. Inouye College of Pharmacy, University of Hawaii at Hilo, USA (accession no. FT431).

### 3.3. Cultivation

The fungus was grown under static conditions at room temperature for 30 days in a 1 L conical flask containing a liquid medium (300 mL/flask) composed of mannitol (20 g/L), sucrose (20 g/L), monosodium glutamate (5 g/L), KH_2_PO_4_ (0.5 g/L), and MgSO_4_.

### 3.4. Isolation of Compounds ***1**–**5***

The whole fermented broth (4.5 L) was filtered through filter paper to separate the supernatant from the mycelia. The mycelia were extracted by 80% acetone/H_2_O three times, and the extracts were condensed under vacuum to get an aqueous solution. The solution was passed through a Diaion HP-20 column (Alfa Aesar, Ward Hill, MA, USA), eluted with MeOH-H_2_O (10%, 50%, 90%, and 100% methanol in H_2_O) to afford four fractions (Fr. A‒D). Fraction C (517.8 mg) was separated with a preparative HPLC column (C18 column, 5 µ, 100.0 × 21.2 mm; 10 mL/min; 10–100% methanol in H_2_O in 40 min) to generate 40 sub-fractions (C1‒40). C35 (27.4 mg) was subjected to the semi-preparative HPLC (C18 column, 5 µ, 250.0 × 10.0 mm; 4 mL/min; with 0.1% formic acid in 75% methanol in H_2_O) to obtain compounds **4** (7.12 mg, *t*_R_ 31.5 min) and **5** (1.56 mg, *t*_R_ 33.5 min). Fraction D (347.2 mg) was separated with a preparative HPLC column (C18 column, 5 µ, 100.0 × 21.2 mm; 10 mL/min; 30–100% methanol in H_2_O in 30 min) to generate 30 sub-fractions (D1‒30). D20 (8.47 mg) was subjected to the semi-preparative HPLC (C18 column, 5 µ, 250.0 × 10.0 mm; 3 mL/min; with 0.1% formic acid in 58% methanol in H_2_O) to afford compound **1** (1.34 mg, *t*_R_ 35.0 min). D26 (18.28 mg) was subjected to the semi-preparative HPLC (C18 column, 5 µ, 250.0 × 10.0 mm; 3 mL/min; with 0.1% formic acid in 75% methanol in H_2_O) to afford compounds **2** (8.51 mg, *t*_R_ 20.8 min) and **3** (1.38 mg, *t*_R_ 25.6 min).

### 3.5. Charaterization of Compounds ***1**–**4***

Peyronetide A (**1**): Brown solid; ${\left[\alpha \right]}_{D}^{25}$ + 73.3 (*c* = 0.06, MeOH); UV (MeOH) *λ*_max_ (log *e*) 298 (4.21), 417 (3.56) nm; IR ν_max_3388, 2959, 2927, 2871, 1710, 1586, 1478, 1461, 1452, 1383, 1354, 1316, 1280, 1232, 1166, 1200, 1067, 873 cm^−1^; ^1^H(acetone-*d*_6_, 400 MHz) and ^13^C-NMR (acetone-*d*_6_, 100 MHz) data, see Table 1; positive HRESIMS *m*/*z* 410.1964 [M + H]^+^ (calcd for C_24_H_28_NO_5_, 410.1968).

Peyronetide B (**2**): Brown solid; ${\left[\alpha \right]}_{D}^{25}$ + 68.8 (*c* = 0.08, MeOH); UV (MeOH) *λ*_max_ (log *e*) 246 (4.13), 254 (4.11), 296 (4.26), 399 (4.22) nm; IR ν_max_3393, 2960, 2926, 2871, 1711, 1646, 1626, 1560, 1529, 1455, 1404, 1377, 1323, 1261, 1212, 1182, 1149, 1097, 1027, 991, 831 cm^−1^; ^1^H(acetone-*d*_6_, 400 MHz) and ^13^C-NMR (acetone-*d*_6_, 100 MHz) data, see Table 1; positive HRESIMS *m*/*z* 427.1751 [M + H]^+^ (calcd for C_24_H_27_O_7_, 427.1757).

Peyronetide C (**3**): Brown solid; ${\left[\alpha \right]}_{D}^{25}$ + 79.1 (*c* = 0.09, MeOH); UV (MeOH) *λ*_max_ (log *e*) 285 (4.57), 394 (4.20) nm; IR ν_max_3400, 2960, 2927, 2872, 1709, 1651, 1611, 1538, 1489, 1455, 1403, 1363, 1335, 1278, 1215, 1170, 1073, 1004, 869, 821, 780 cm^−1^; ^1^H(acetone-*d*_6_, 400 MHz) and ^13^C-NMR (acetone-*d*_6_, 100 MHz) data, see Table 1; positive HRESIMS *m*/*z* 427.1760 [M + H]^+^ (calcd for C_24_H_27_O_7_, 427.1757).

Peyronetide D (**4**): Brown solid; ${\left[\alpha \right]}_{D}^{25}$ + 45.0 (*c* = 0.02, MeOH); UV (MeOH) *λ*_max_ (log *e*) 215 (3.99), 238 (3.83), 257 (3.77), 304 (3.74) nm; IR ν_max_3382, 2959, 2928, 2872, 2360, 2342, 1617, 1578, 1506, 1452, 1384, 1340, 1315, 1280, 1163, 1110 cm^−1^; ^1^H(acetone-*d*_6_, 400 MHz) and ^13^C-NMR (acetone-*d*_6_, 100 MHz) data, see Table 1; positive HRESIMS *m*/*z* 317.1390 [M + H]^+^ (calcd for C_18_H_21_O_5_, 317.1389).

### 3.6. Anti-Proliferative Activity

The viability of A2780 and TK-10 (from the NCI) and the cisplatin-resistant, A2780CisR [31], was determined using the CyQuant cell proliferation assay kit, according to the manufacturer’s instructions (Life Technologies, Eugene, OR, USA). Briefly, cells in 96-well plates, seeded 24 h prior, were treated with or without compounds for 72 h, and subjected to CyQuant cell viability assay (Life Technologies, Eugene, OR, USA) [32,33,34]. Each cell line was cultured in 96-well plates at 6000 cells per well with the following conditions: 0 (no treatment, vehicle (DMSO)) and increasing concentrations of compounds for 72 h. Cisplatin was used as a positvie control. Viable cells were analyzed by subjecting the plates to the CyQuant, as previously reported [32,33,34]. Relative viability of the treated cells was normalized to the DMSO-treated control cells. All experiments were performed in triplicate.

### 3.7. DP4+ Calculations

All of the quantum mechanical calculations were performed using Gaussian 09 [35]. The conformational search was done in the gas phase using the MMFF (Merck Molecular Force Field) force field (implemented in Macromodel) [36]. All conformers within 10 kcal/mol from the global minima (more than 900 different structures) were kept for further calculations. After an exhaustive exploration of the conformational space of the two possible diastereoisomers of **1**, namely, **1a** = **1**–**8*S*,13*S*** and **1b** = **1**–**8*S*,13*R*** (equivalent to **1–8*R*,13*S***), we were able to locate more than 900 unique conformations for both compounds. In order to narrow down the number of geometries for B3LYP/6-31G* optimizations, a previous HF/3-21G geometry optimization stage was carried out, and all confomers within 6 kcal/mol from the global minima were submitted to full geometry optimizations at the B3LYP/6-31G* level. The isotropic magnetic shielding constants (σ) were computed using the gauge including the atomic orbitals (GIAO) method [37,38,39,40], at the B3LYP/6-31G**//MMFF level (for DP4 calculations) [22] and PCM/mPW1PW91/6-31+G**//B3LYP/6-31G* level (for DP4+ calculations) [23] using methanol as the solvent. The unscaled chemical shifts (δ_u_) were computed using TMS (Tetramethylsilane) as a reference standard according to δ_u_ = σ_0_ − σ_x_, where σ_x_ is the Boltzmann averaged shielding tensor (over all significantly populated conformations) and σ_0_ is the shielding tensor of the TMS computed at the same level of theory employed for σ_x_. The scaled chemical shifts (δ_s_) were calculated as δ_s_ = (δ_u_ − b)/m, where m and b are the slope and intercept, respectively, deduced from a linear regression calculation on a plot of δ_u_ against δ_exp_. The DP4+ calculations were run by the Excel spreadsheet available for free at sarotti-nmr.weebly.com or as part of the Supporting Information of the original paper [23], and the DP4 calculations were done according to the original reference [22]. 

## 4. Conclusions

In conclusion, five compounds (**1**–**5**) including four new ones (**1**–**4**) were isolated from a Hawaiian plant-asssociated endophytic fungus *Peyronellaea* sp. FT431. Compound **1** is a unique benzoisoquinoline-9-one derivative with two side-chains, 1,3-dimethyl-1-pentene and 2-propanone at 3- and 8-positions, respectively, which were diagonal to each other in the benzoisoquinoline-9-one. Compounds **1**–**5** were evaluated for their antiproliferative activity, and compound **5** was the most active one with IC_50_ values of 7.1, 6.7, and 8.5 μM against A2780S, A2780CisR, and TK-10, respectively. The results indicated that Hawaiian fungi are a good resource of new and bioactive compounds.

## Figures and Tables

**Table 1:** NMR spectroscopic data for **1** and **2** in acetone-*d*_6._

No.	1		2	
	δ_H_, *J* (Hz) ^a^	δ_C_^b^	δ_H_, *J* (Hz) ^a^	δ_C_^b^
1	9.43, s	148.4	10.52, s	187.6
3		158.5		166.87
4	7.61, s	115.0	7.42, s	104.2
4a		143.3		112.2
5	7.05, s	114.7		144.2
5a		139.0		136.3
6	6.00, s	98.9	6.33, s	90.7
7		161.6		166.92
8		72.8		73.2
9		202.8		202.6
9a		107.7		106.4
10		164.7		
10a		117.9		130.0
11		133.3		124.6
12	6.70, d, 10	140.1		141.0
13	2.60, m	35.7	2.64, m	35.5
14	1.49, m;1.41, m	31.0	1.49, m;1.42, m	30.8
15	0.92, t, 7.4	12.4	0.92, t, 7.4	12.4
16	2.16, d, 1.3	14.5	1.45, d, 1.4	13.6
17	1.07, d, 6.7	20.8	1.10, d, 6.6	20.5
1′	3.52, s	50.6	3.59, d, 5.5	51.3
2′		205.7		206.5
3′	2.09, s	29.6	2.13, s	29.8
7-MeO	3.79, s	56.2	3.91, s	56.9

^a^ Spectra recorded at 400 MHz. ^b^ Spectra recorded at 100 MHz. Data based on ^1^H, ^13^C, HSQC, and HMBC experiments.

**Table 2:** NMR spectroscopic data for compounds **3** (acetone-*d*_6_) and **4** (MeOH-*d*_4_).

No.	3 ^a^		4 ^b^	
	δ_H_, *J* (Hz)	δ_C_	δ_H_, *J* (Hz)	δ_C_
1		164.8		181.6
2			6.16, s	107.4
3		158.0		164.3
4	6.56	101.6		160.3
4a				
5	6.77, s	114.1	6.78, d, 2.0	102.1
5a				
6	5.89, s	97.1		163.1
7		163.1	6.69, d, 2.6	118.5
8		72.1		140.2
9		199.9		115.4
9a		109.1		
10		159.1		126.8
10a		104.4		
11		125.6		142.8
12	6.34, d, 9.7	140.3	2.59, m	35.8
13	2.58, m	34.7	1.52, m; 1.42, m	30.6
14	1.48, m; 1.41, m	29.8	0.92, t, 7.6	12.0
15	0.89, t, 7.4	11.4	1.97, s	12.7
16	1.98, s	11.9	1.08, d, 6.7	20.3
17	1.05, d, 6.6	19.6		
1′	3.46, br.d, 5.9	50.6	4.11, s	42.8
2′		205.7		176.9
3′	2.10, s	29.0		
7-MeO	3.79, s	56.2		107.4

^a^ Spectra recorded at 400 MHz. ^b^ Spectra recorded at 100 MHz. Data based on ^1^H, ^13^C, HSQC, and HMBC experiments.

**Table 3:** Antiproliferative activities of compounds **1**, **2**, and **5** against different cell lines.

Compounds	IC_50_ (μM)		
	A2780S	A2780CisR	TK-10
**1**	24.1 ± 0.8	28.3 ± 7.2	29.2 ± 2.9
**2**	21.5 ± 0.3	27.2 ± 1.3	22.7 ± 1.3
**5**	7.1 ± 0.8	6.7 ± 1.2	8.5 ± 0.9

## Supplementary Materials

The supplementary materials (NMR [including ^1^H, ^13^C, COSY, HSQC, HMBC, and NOESY], HRESIMS, IR spectra, anti-proliferative, and NMR calculation data) are available online.
