# Supplementary material for: An Unusual Benzoisoquinoline-9-one Derivative and Other Related Compounds with Antiproliferative Activity from Hawaiian Endophytic Fungus Peyronellaea sp. FT431
Source: Molecules. 2019 Jan 7;24(1):196. doi: 10.3390/molecules24010196 (PMC6337129; doi:10.3390/molecules24010196)
Supplement: Supplementary file 1 [file molecules-24-00196-s001.pdf]

Supporting Information

An Unusual Benzoisoquinoline-9-one Derivative and Other Related Compounds with Antiproliferative Activity from Hawaiian Endophytic Fungus Peyronellaea sp. FT431

Chunshun Li[1,2], Ariel M. Sarotti[3], Xiaohua Wu[1], Baojun Yang[2], James Turkson[2], Yongfei Chen[4], Qingsong Liu[4], and Shugeng Cao[1,2,*]

[1] Department of Pharmaceutical Sciences, Daniel K. Inouye College of Pharmacy, University of Hawai'i at Hilo, 200 West Kawili Street, Hilo, HI 96720, USA; chunshun@hawaii.edu (C.L.); xiaohua3@hawaii.edu(X.W.); scao@hawaii.edu (S.C.)

[2] Cancer Biology Program, University of Hawaii Cancer Center, 701 Ilalo Street, Honolulu, Hawaii 96813, USA; byang@cc.hawaii.edu (B.Y.); jturkson@cc.hawaii.edu (J.T.)

[3] Instituto de Química Rosario (CONICET), Facultad de Ciencias Bioquímicas y Farmacéuticas, Universidad Nacional de Rosario, Suipacha 531, Rosario 2000, Argentina; sarotti@iquir-conicet.gov.ar(A.M.S.)

[4] High Magnetic Field Laboratory, Key Laboratory of High Magnetic Field and Ion Beam Physical Biology, Hefei Institute of Physical Science, Chinese Academy of Sciences, Hefei 230031, People's Republic of China; Chenyf@hmfl.ac.cn (F.C.); qsliu97@hmfl.ac.cn (Q.L.)

*Corresponding Author

E-mail: scao@hawaii.edu; Tel: 808 9818017; Fax: 808 9332874.

Content

- Figure S1. ^{1}H NMR spectrum of compound **1** (in acetone-d_6)……S3
- Figure S2. ^{13}C NMR spectrum of compound **1**……S4
- Figure S3. ^{1}H-^{1}H COSY of compound **1**……S5
- Figure S4. HSQC spectrum of compound **1** ……S6
- Figure S5. HMBC spectrum of compound **1**……S7
- Figure S6. NOESY spectrum of compound **1**……S8
- Figure S7. ^{1}H NMR spectrum of compound **2** (in acetone-d_6)……S9
- Figure S8. ^{13}C NMR spectrum of compound **2**……S10
- Figure S9. ^{1}H-^{1}H COSY of compound **2**……S11
- Figure S10. HSQC spectrum of compound **2** ……S12
- Figure S11. HMBC spectrum of compound **2** ……S13
- Figure S12. NOESY spectrum of compound **2**……S14
- Figure S13. ^{1}H NMR data of compound **3** (in acetone-d_6)……S15
- Figure S14. ^{13}C NMR spectrum of compound **3**……S16
- Figure S15. HSQC spectrum of compound **3** ……S17
- Figure S16. HMBC spectrum of compound **3** ……S18
- Figure S17. NOESY spectrum of compound **3**……S19
- Figure S18. ^{1}H NMR data of compound **4** (in methanol-d_4)……S20
- Figure S19. ^{13}C NMR spectrum of compound **4**……S21
- Figure S20. ^{1}H-^{1}H COSY of compound **4**……S22
- Figure S21. HSQC spectrum of compound **4** ……S23
- Figure S22. HMBC spectrum of compound **4** ……S24
- Figure S23. NOESY spectrum of compound **4**……S25
- Figure S24. HRESIMS spectrum of compound **1**……S26
- Figure S25. HRESIMS spectrum of compound **2**……S27
- Figure S26. HRESIMS spectrum of compound **3**……S28
- Figure S27. HRESIMS spectrum of compound **4**……S29
- Figure S28. IR spectrum of compound **1**……S30
- Figure S29. IR spectrum of compound **2**……S31
- Figure S30. IR spectrum of compound **3**……S32
- Figure S31. IR spectrum of compound **4**……S33
- Anti-proliferative data of compounds **1**, **2**, and **5**……S34
- Figure S32. Structures of isomers **1a** and **1b**……S37
- Table S1 and Table S2……S38
- Table S3 and Table S4……S39
- Cartesian coordinates……S40

^{1}H NMR spectrum of compound **1** (Acetone-d_6)

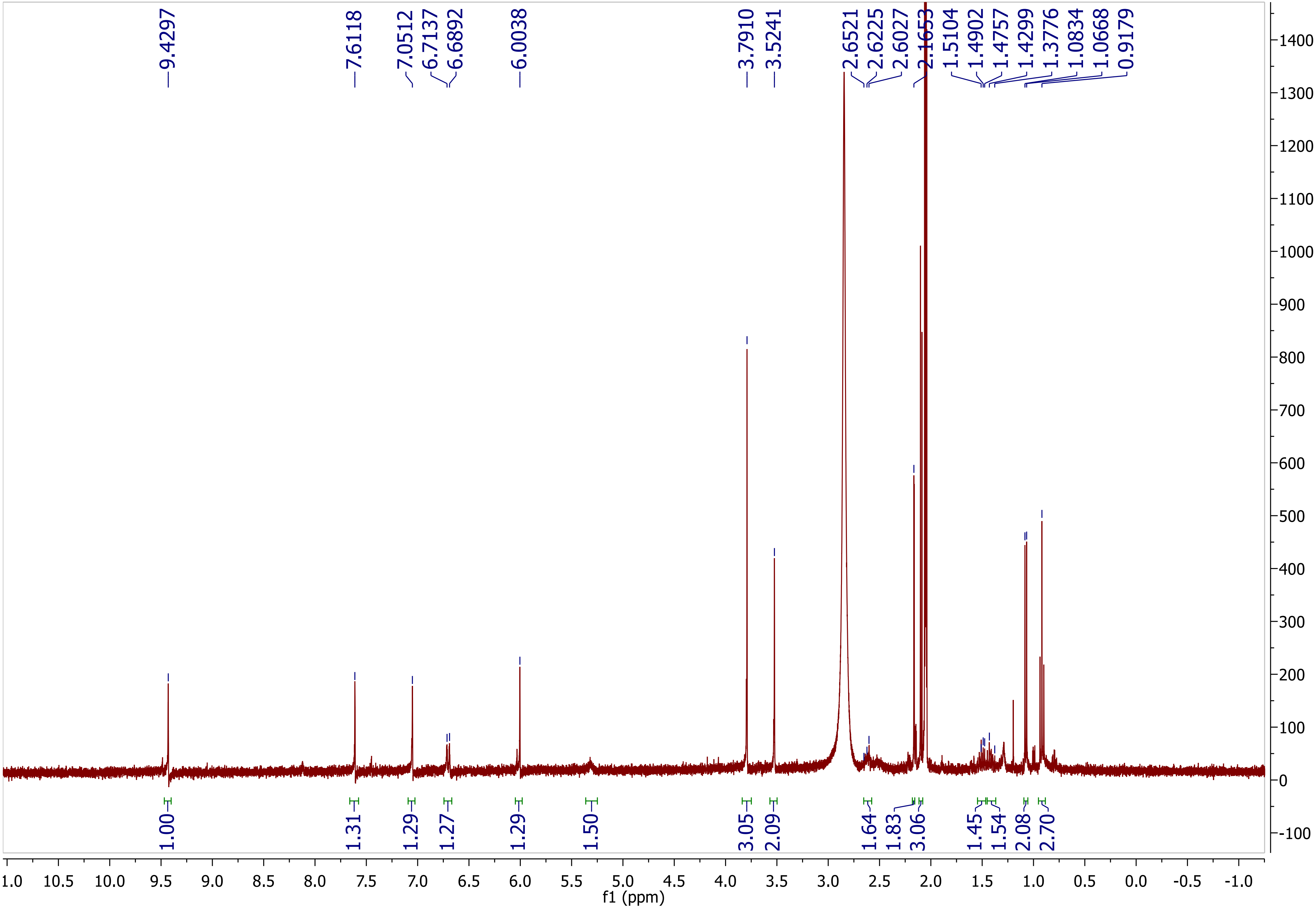

^{13}C NMR spectrum of compound **1**

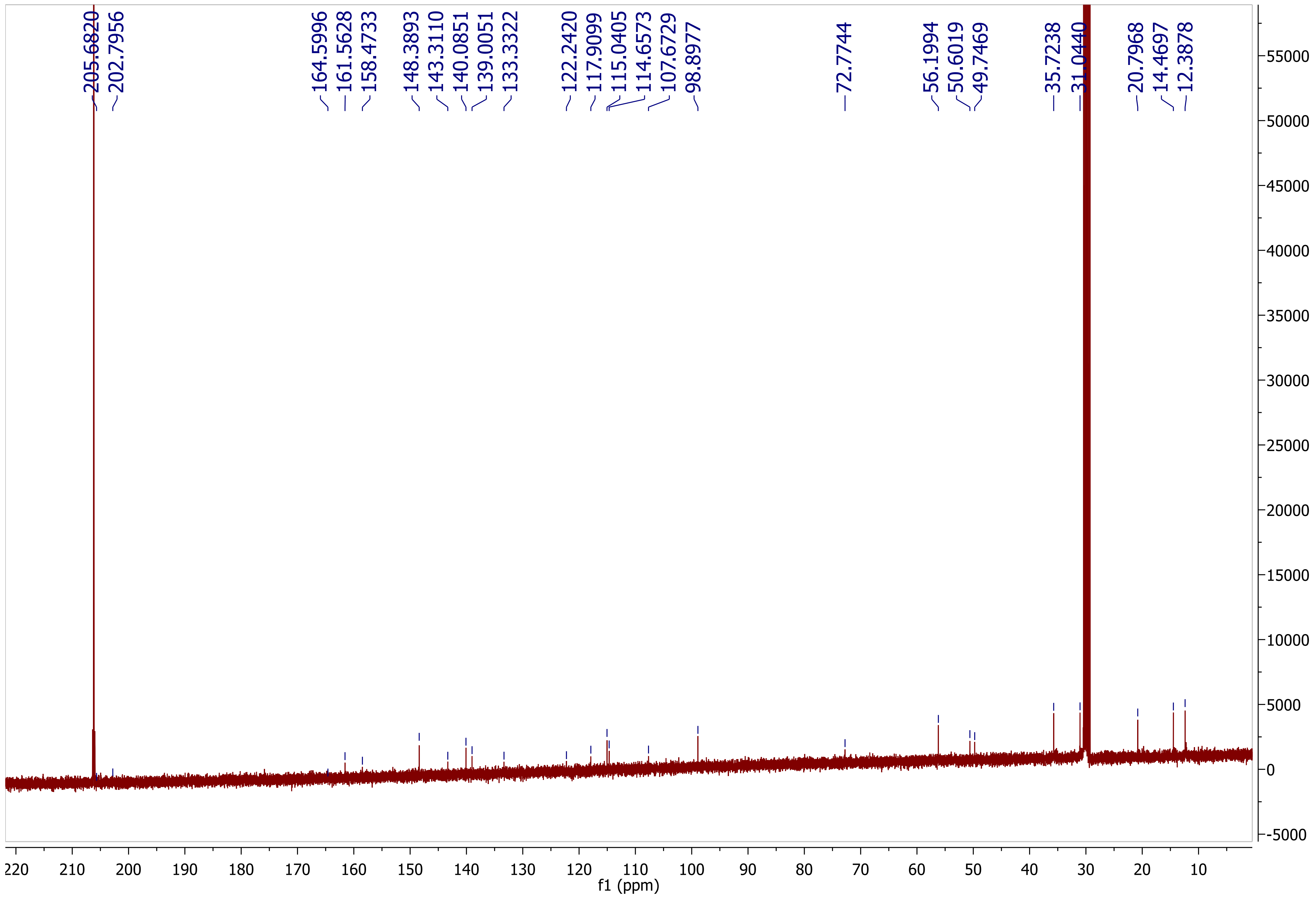

^{1}H-^{1}H COSY of compound **1**

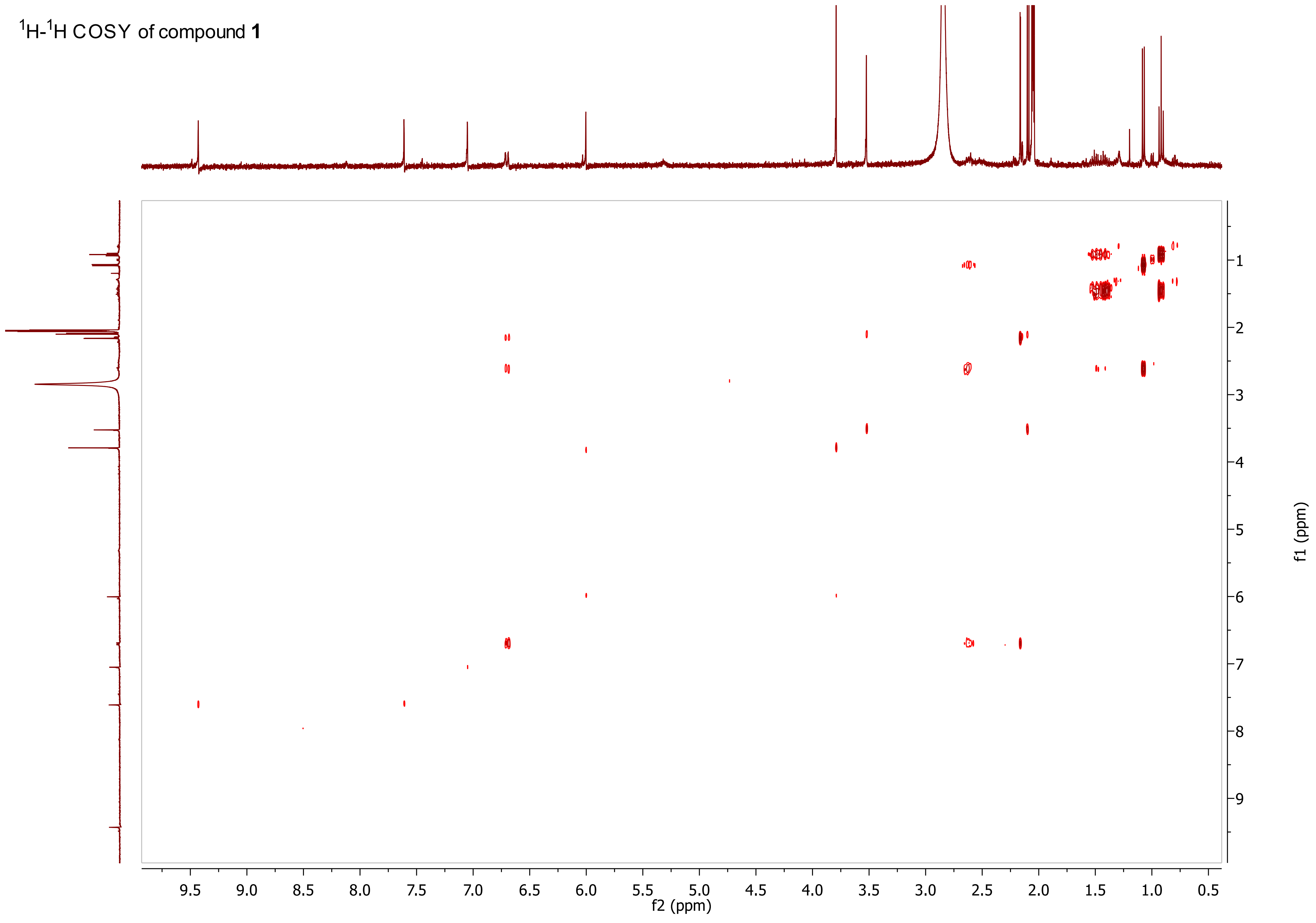

HSQC spectrum of compound **1**

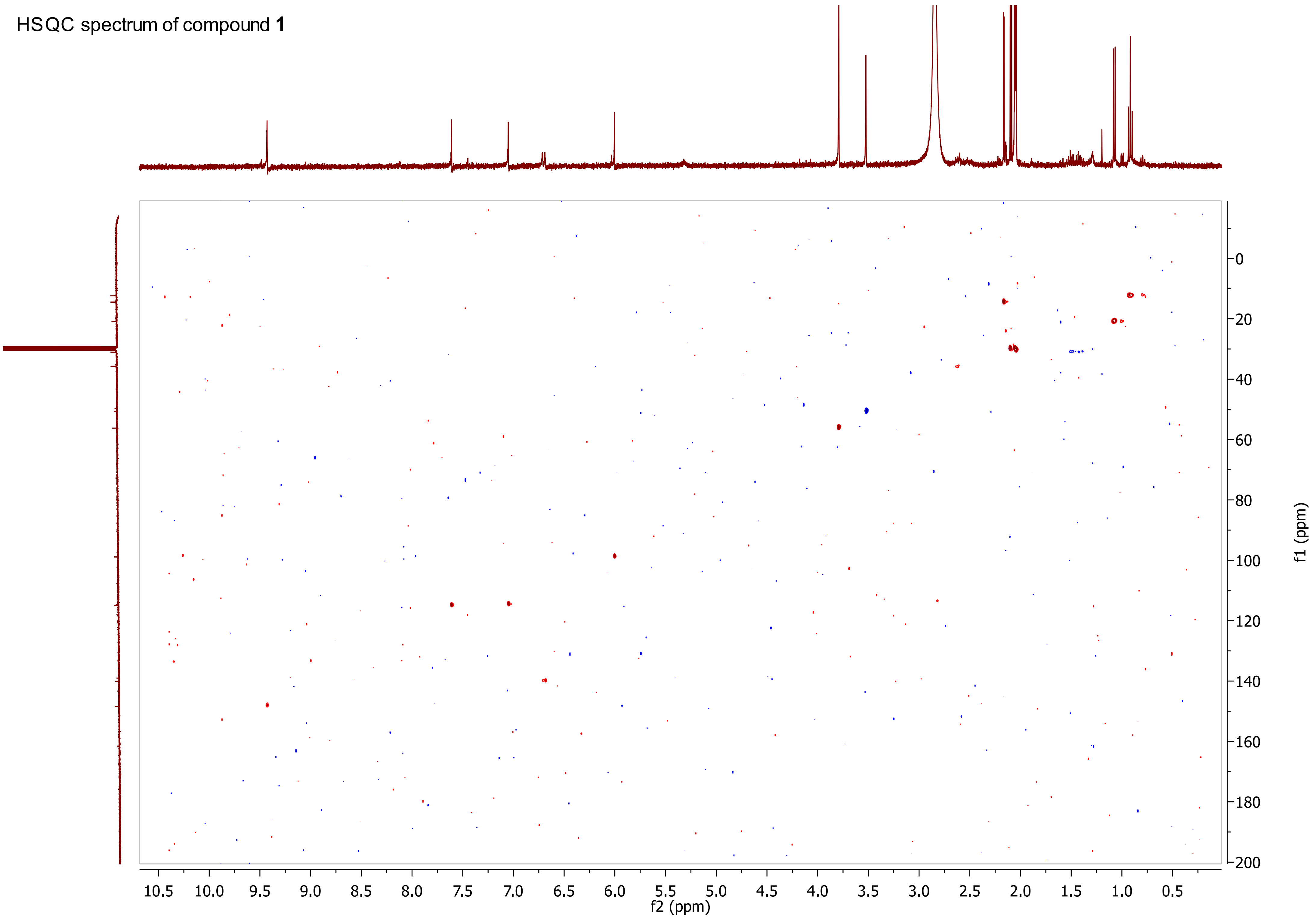

HMBC spectrum of compound **1**

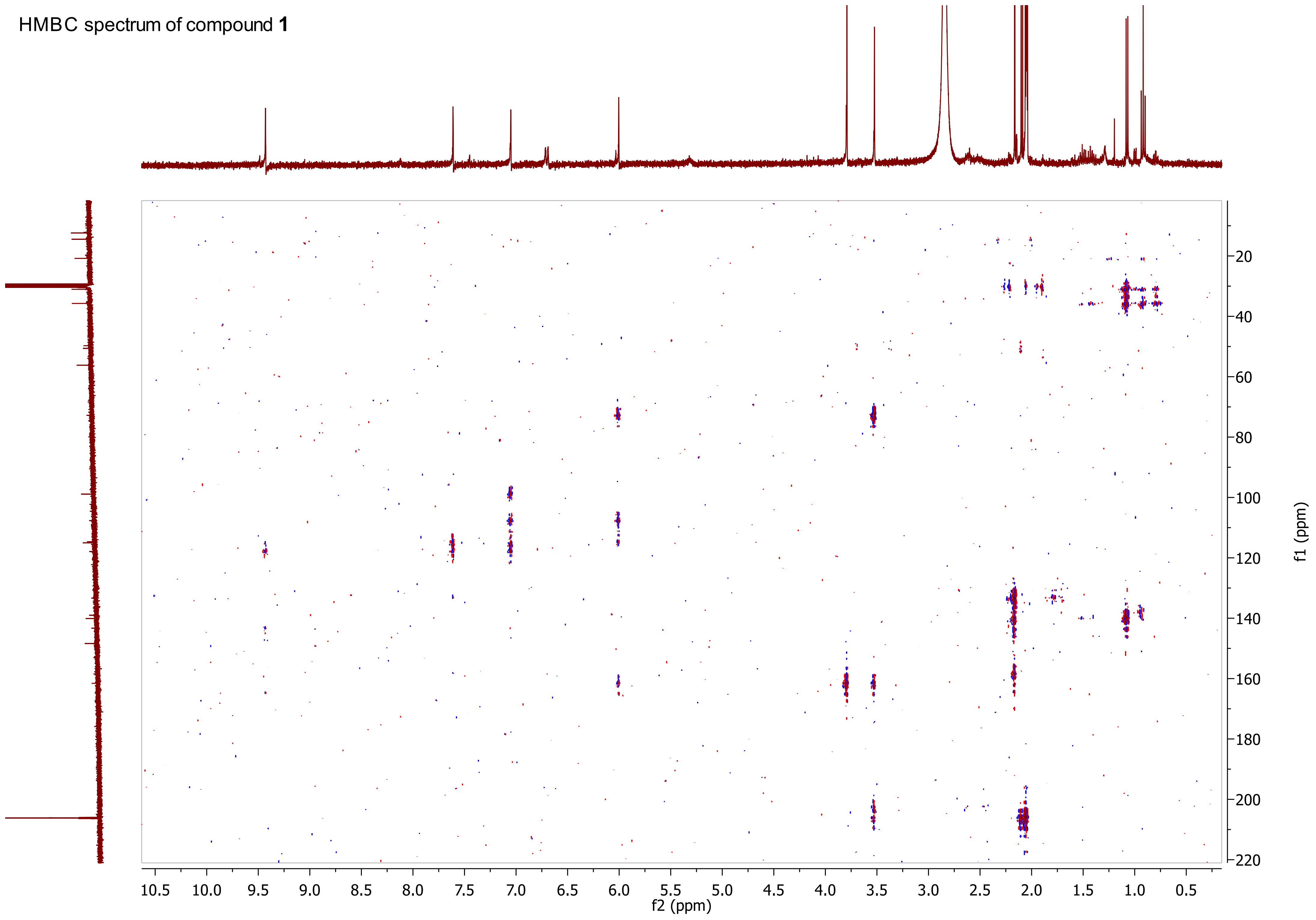

NOESY spectrum of compound **1**

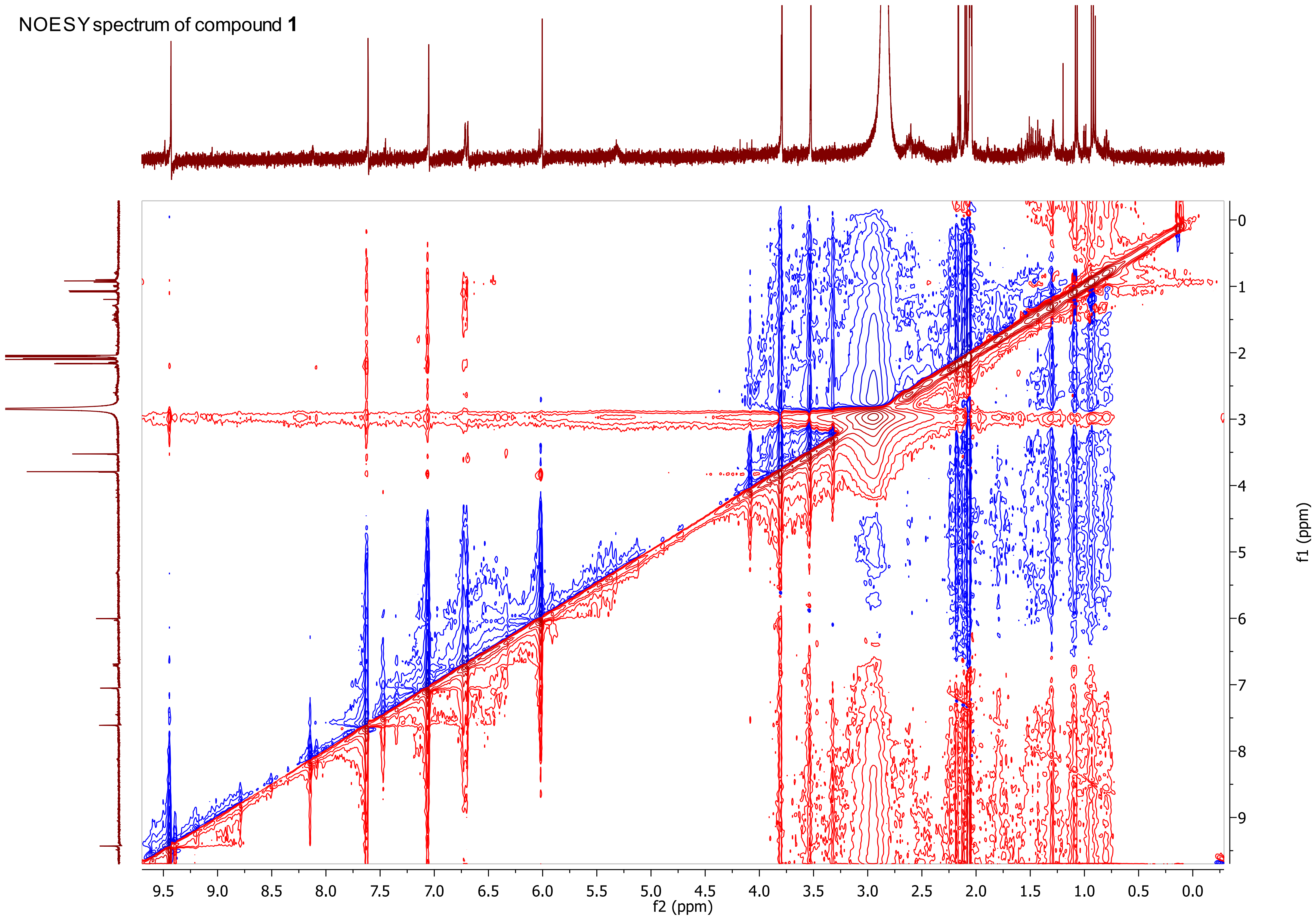

^{1}H NMR spectrum of compound **2** (in Acetone-d_6)

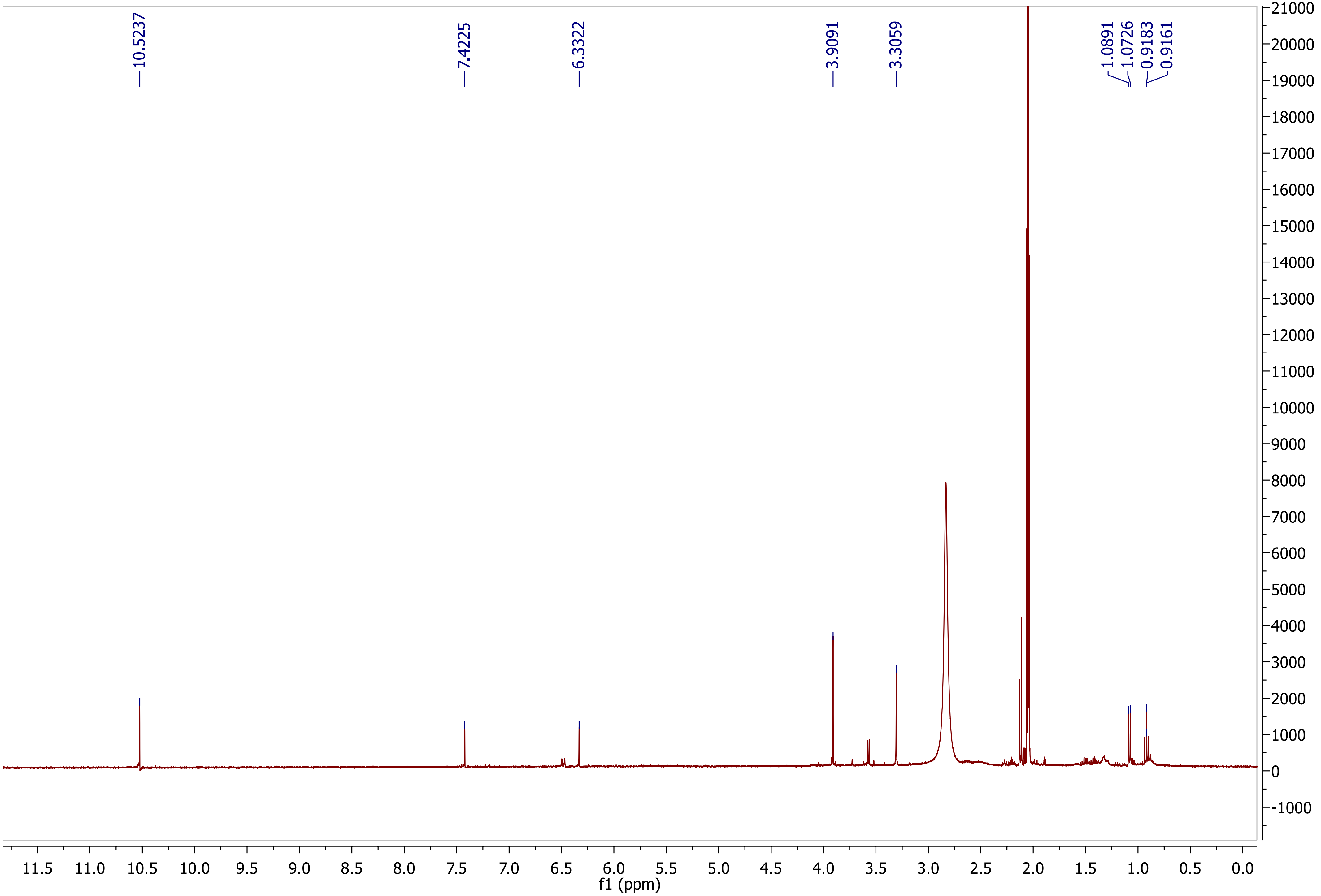

^{13}C NMR spectrum of compound **2**

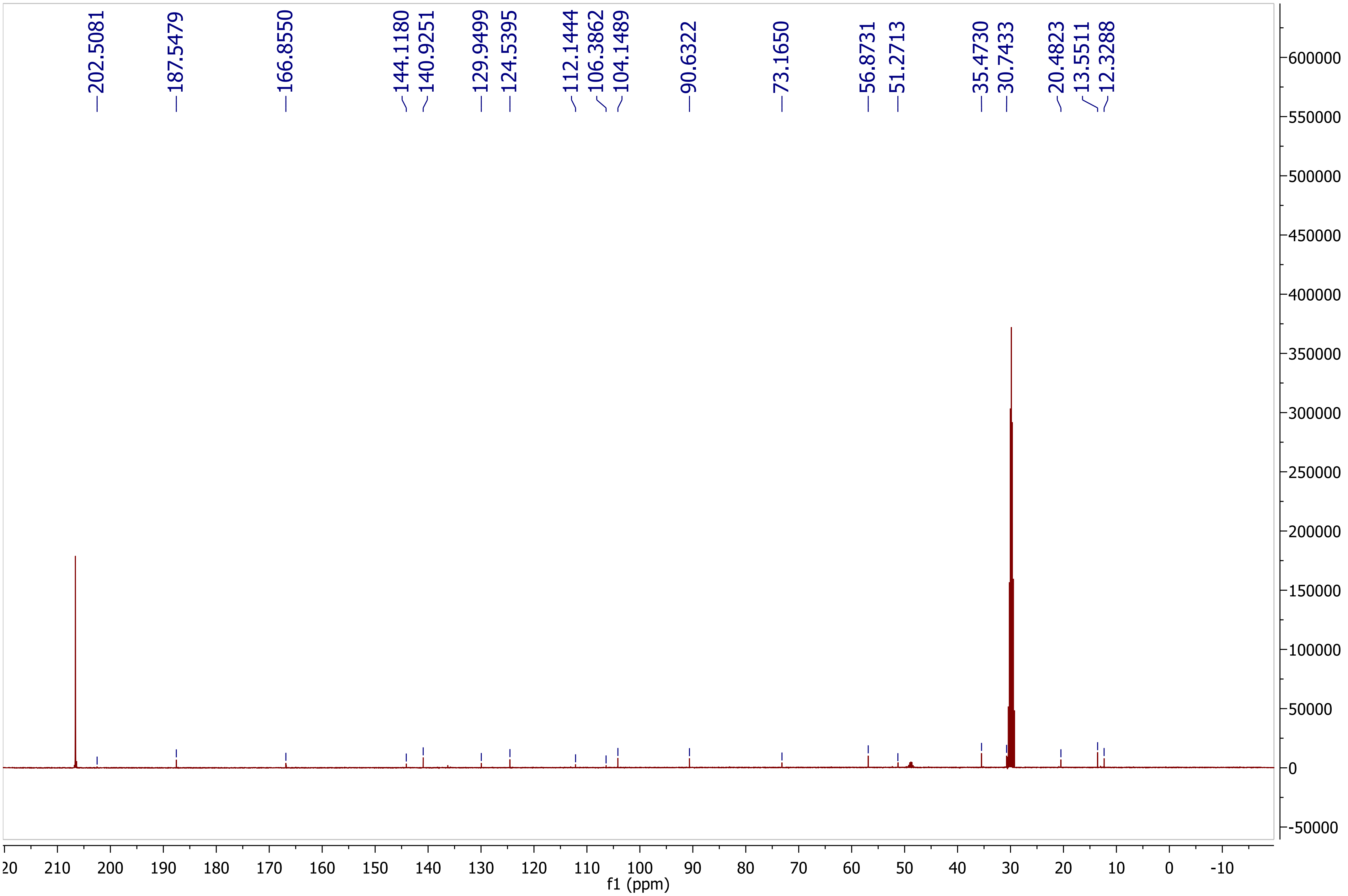

^{1}H-^{1}H COSY spectrum of compound 2

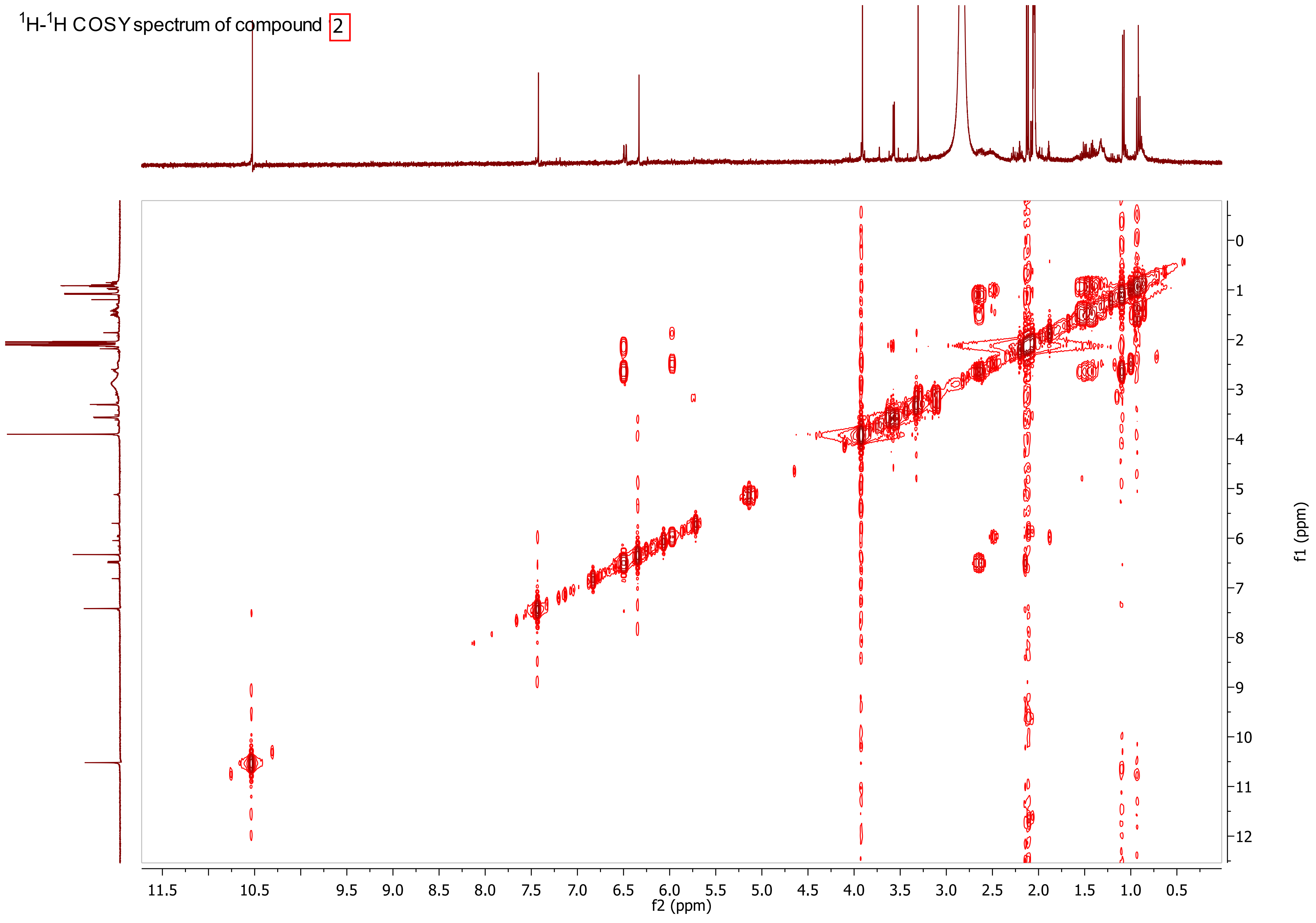

HSQC spectrum of compound **2**

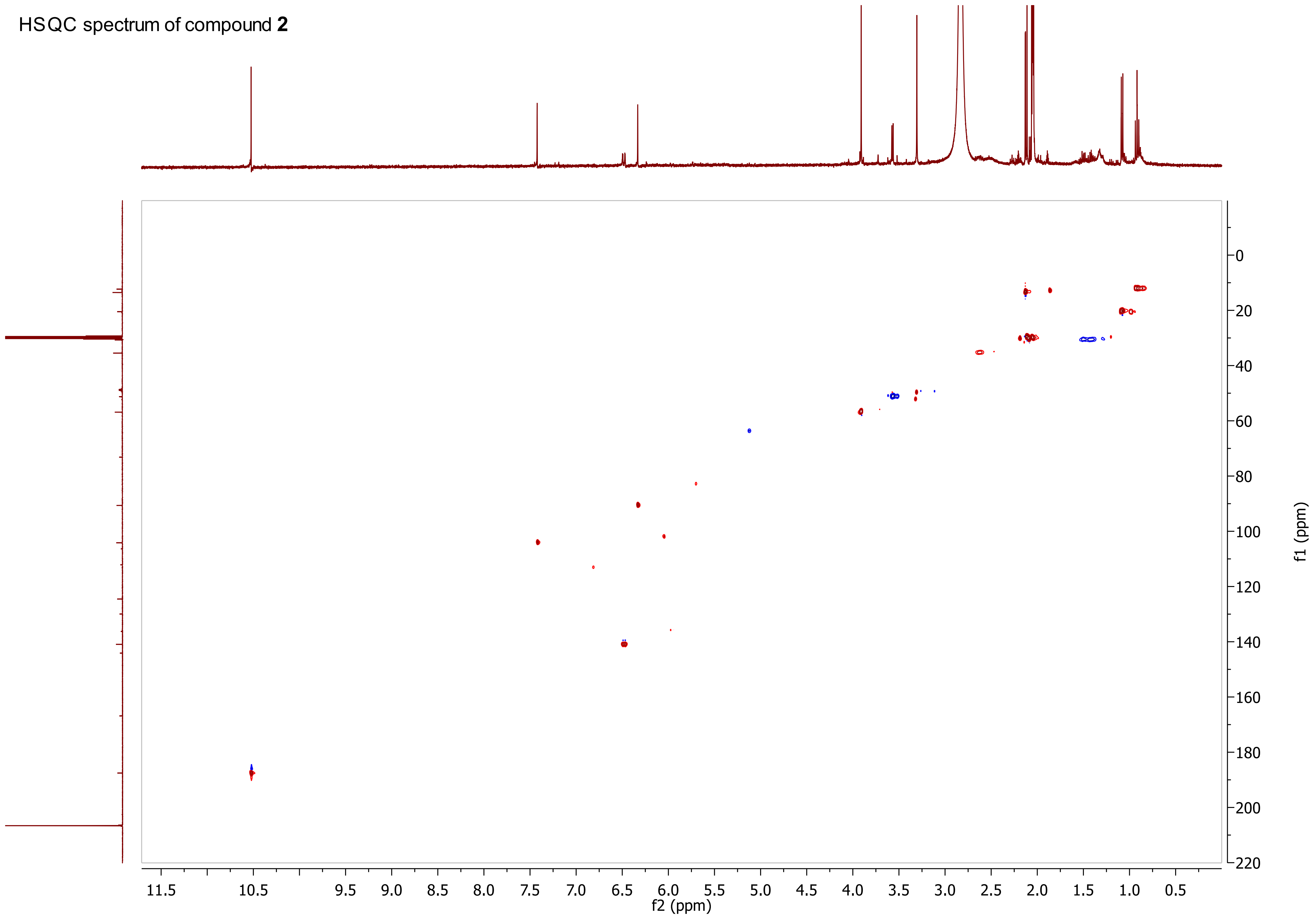

HMBC spectrum of compound **2**

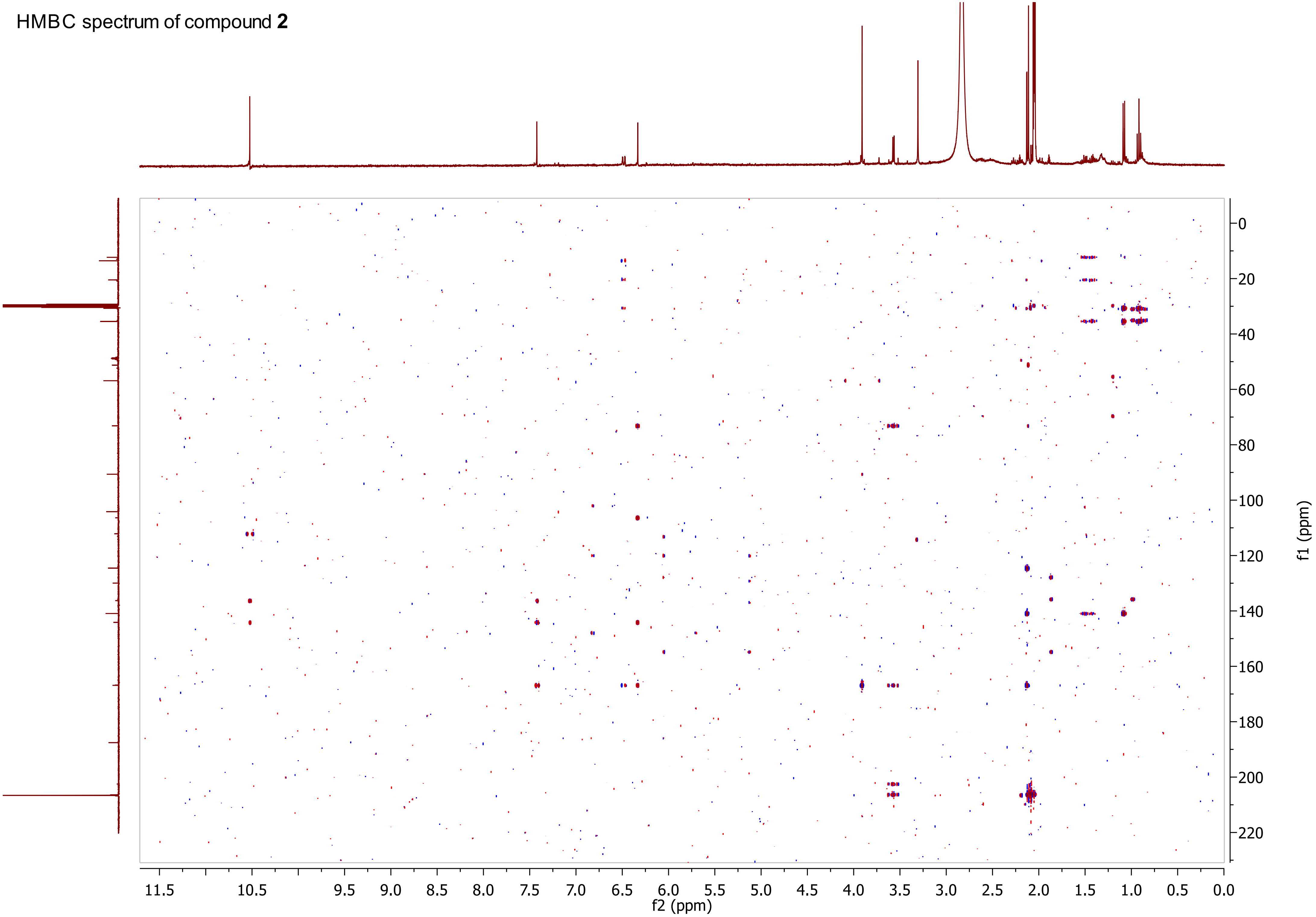

NOESY spectrum of compound **2**

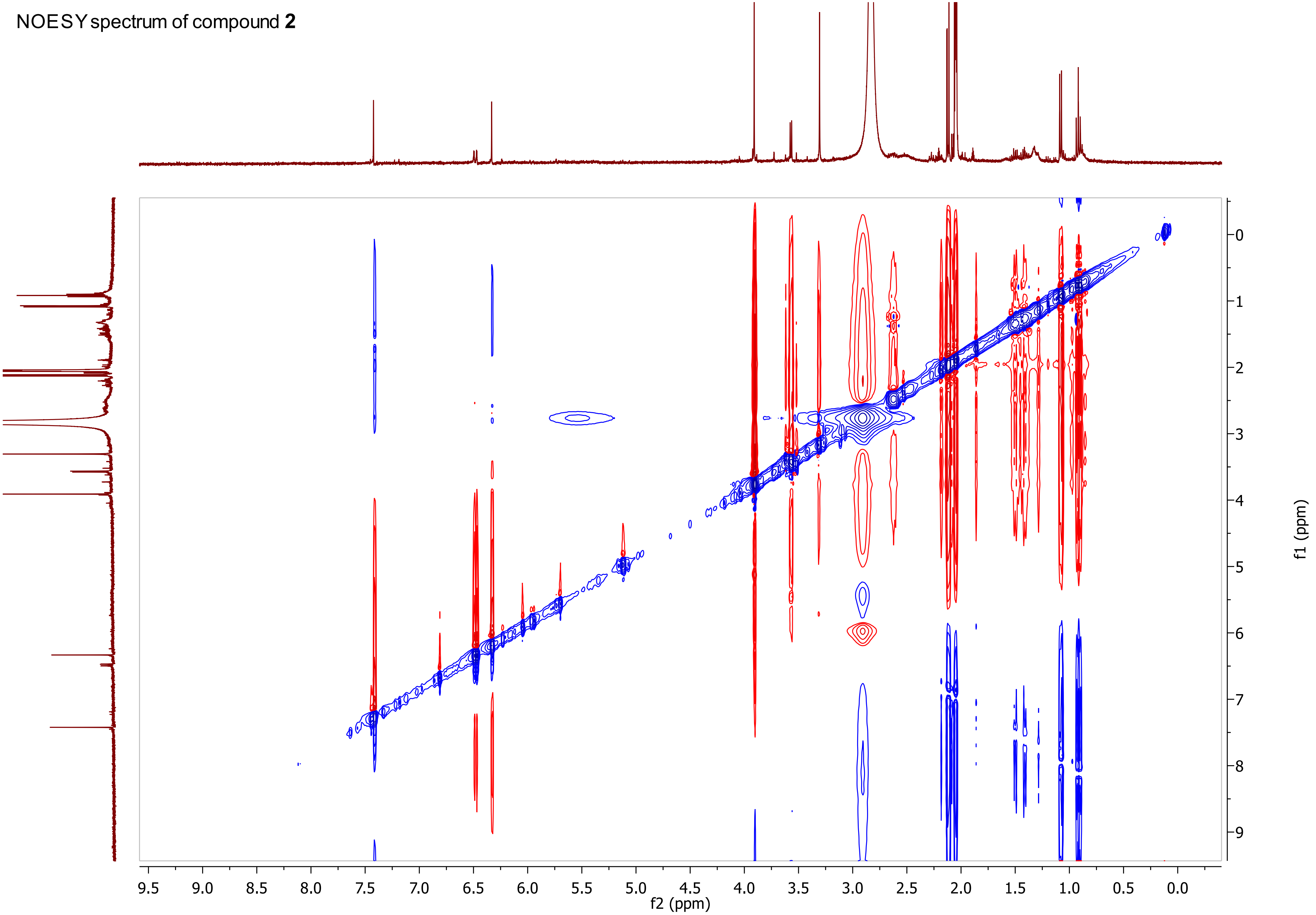

^{1}H NMR spectrum of compound 3 (in acetone-d_6)

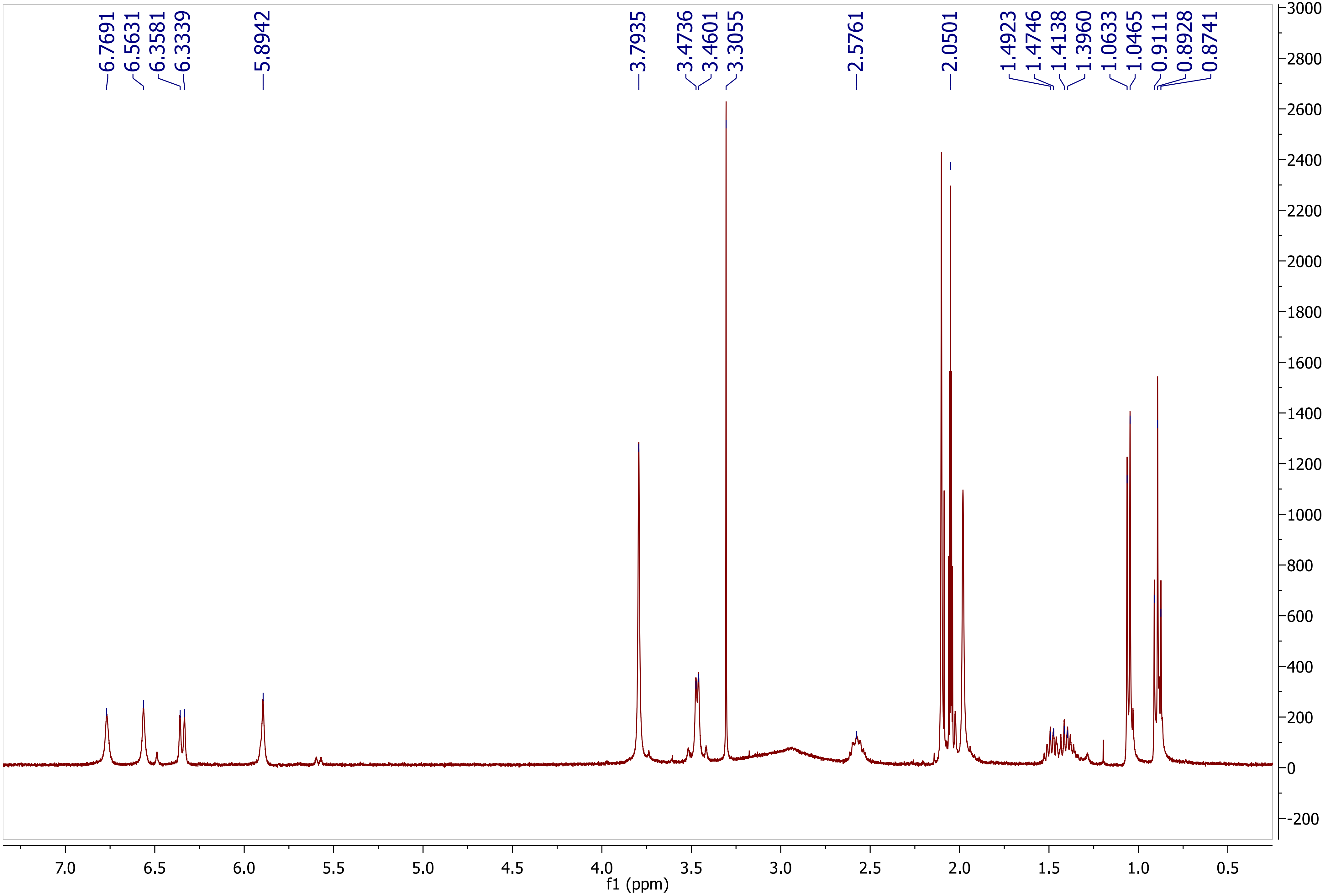

^{13}C NMR spectrum of compound **3**

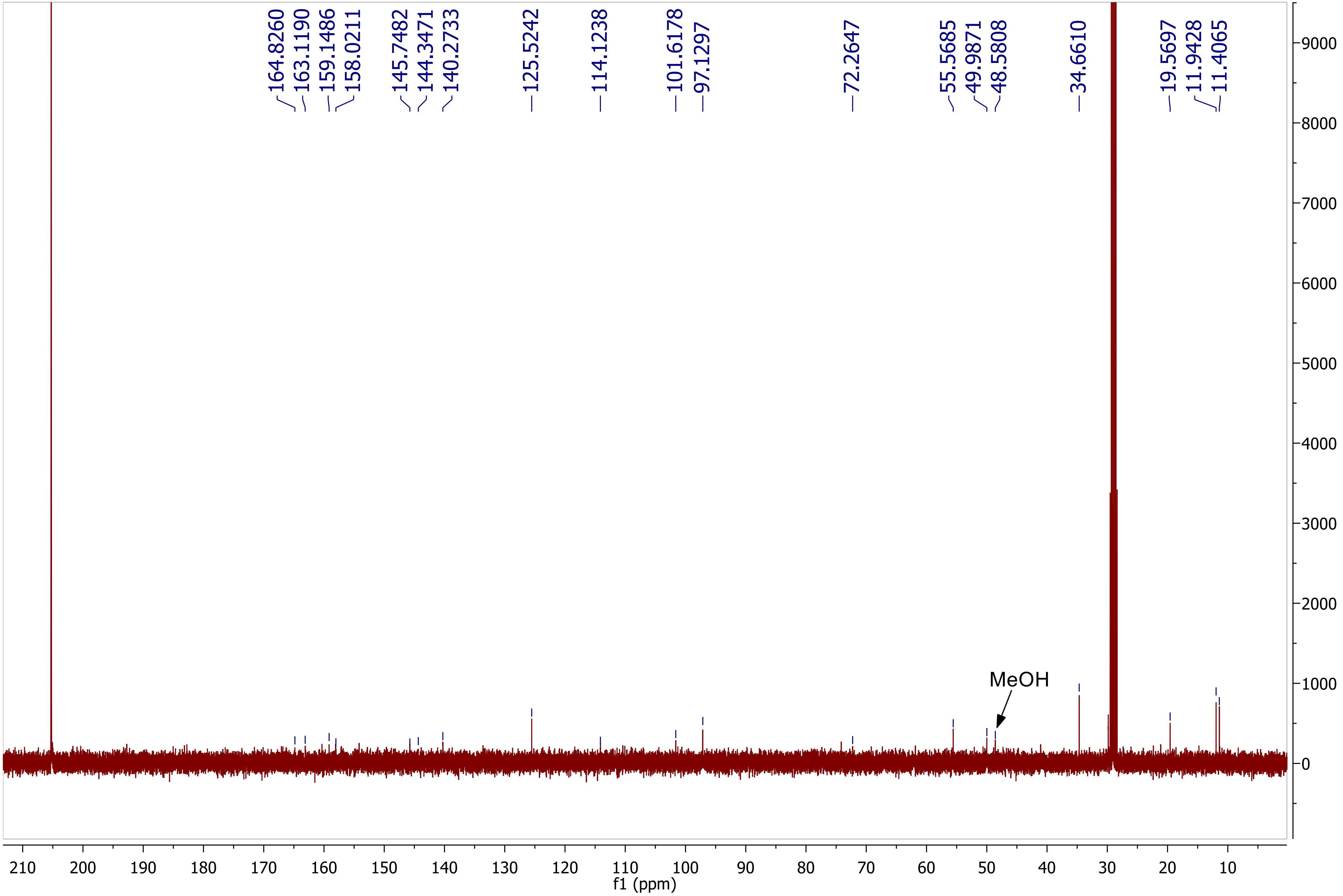

HSQC spectrum of compound **3**

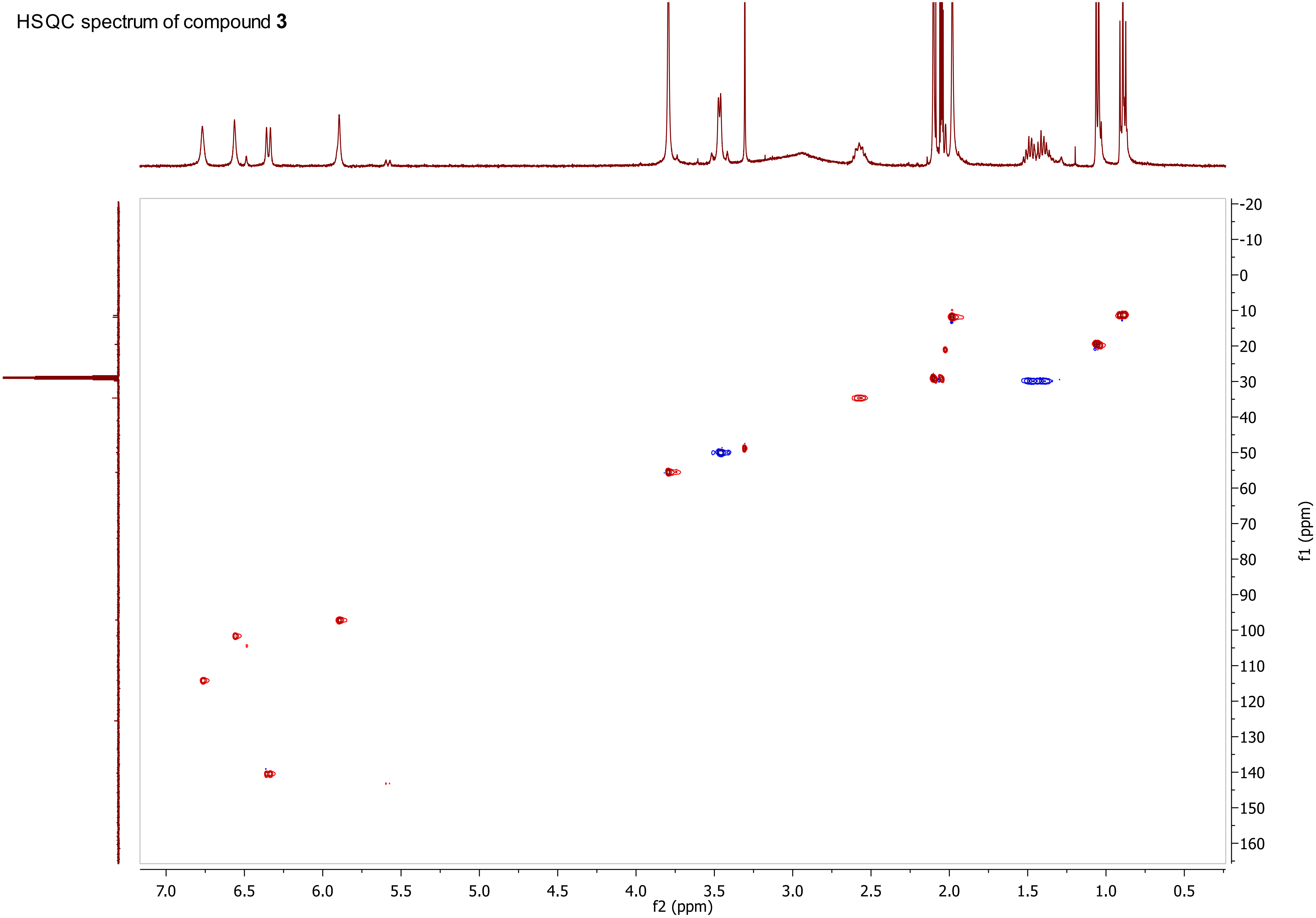

HMBC spectrum of compound **3**

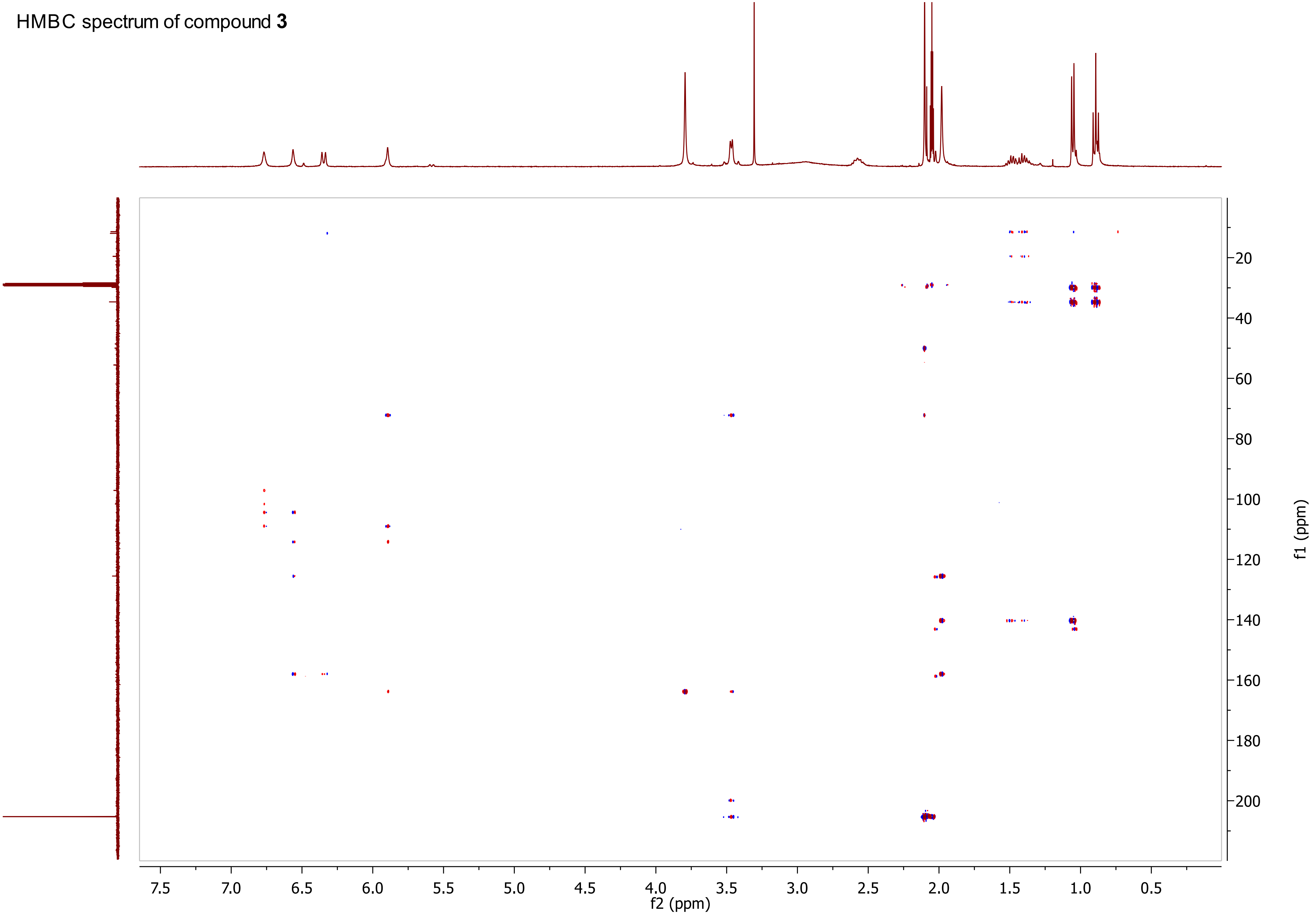

NOESY spectrum of compound **3**

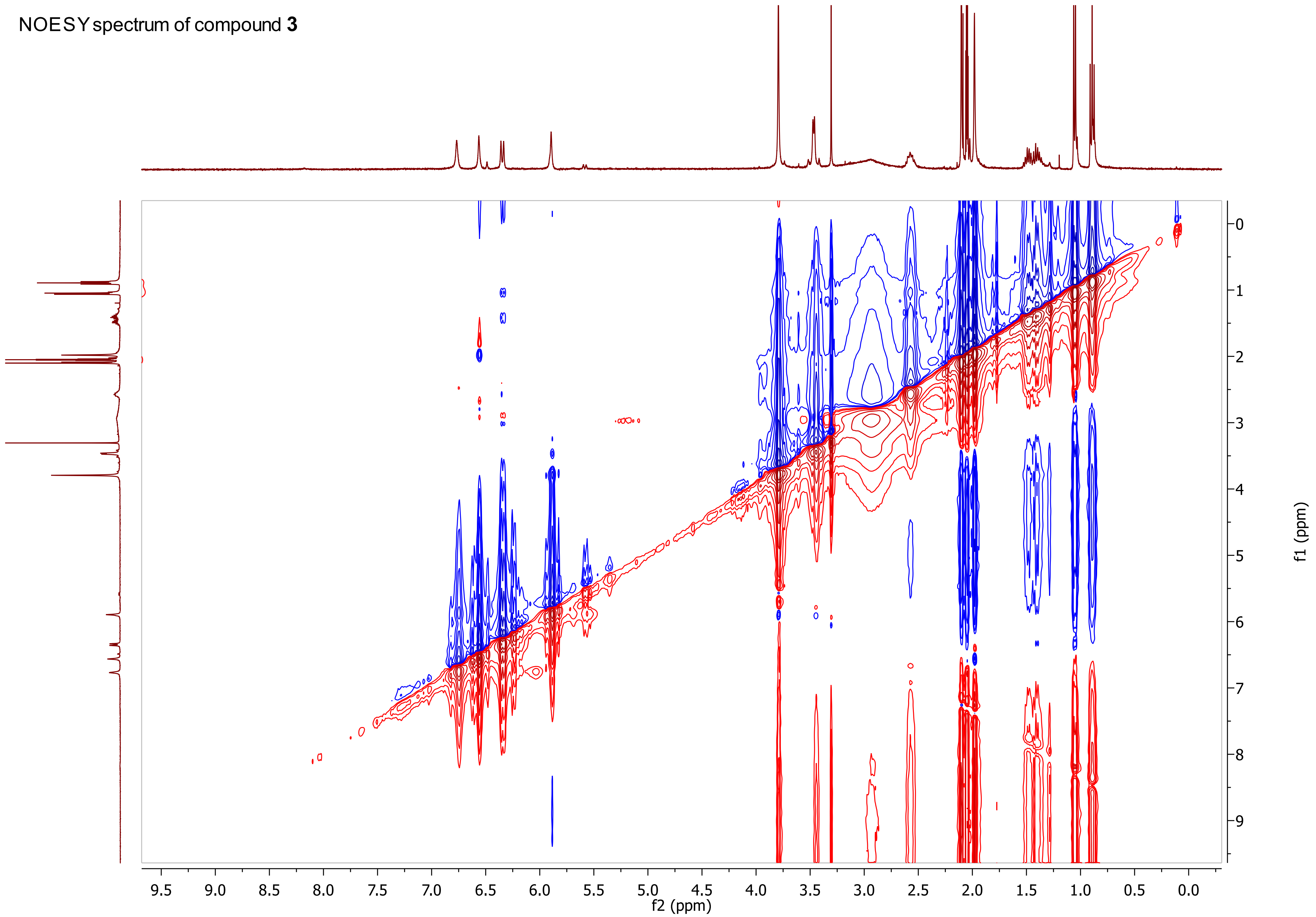

^{1}H NMR spectrum of compound 4 in (MeOH-d_4)

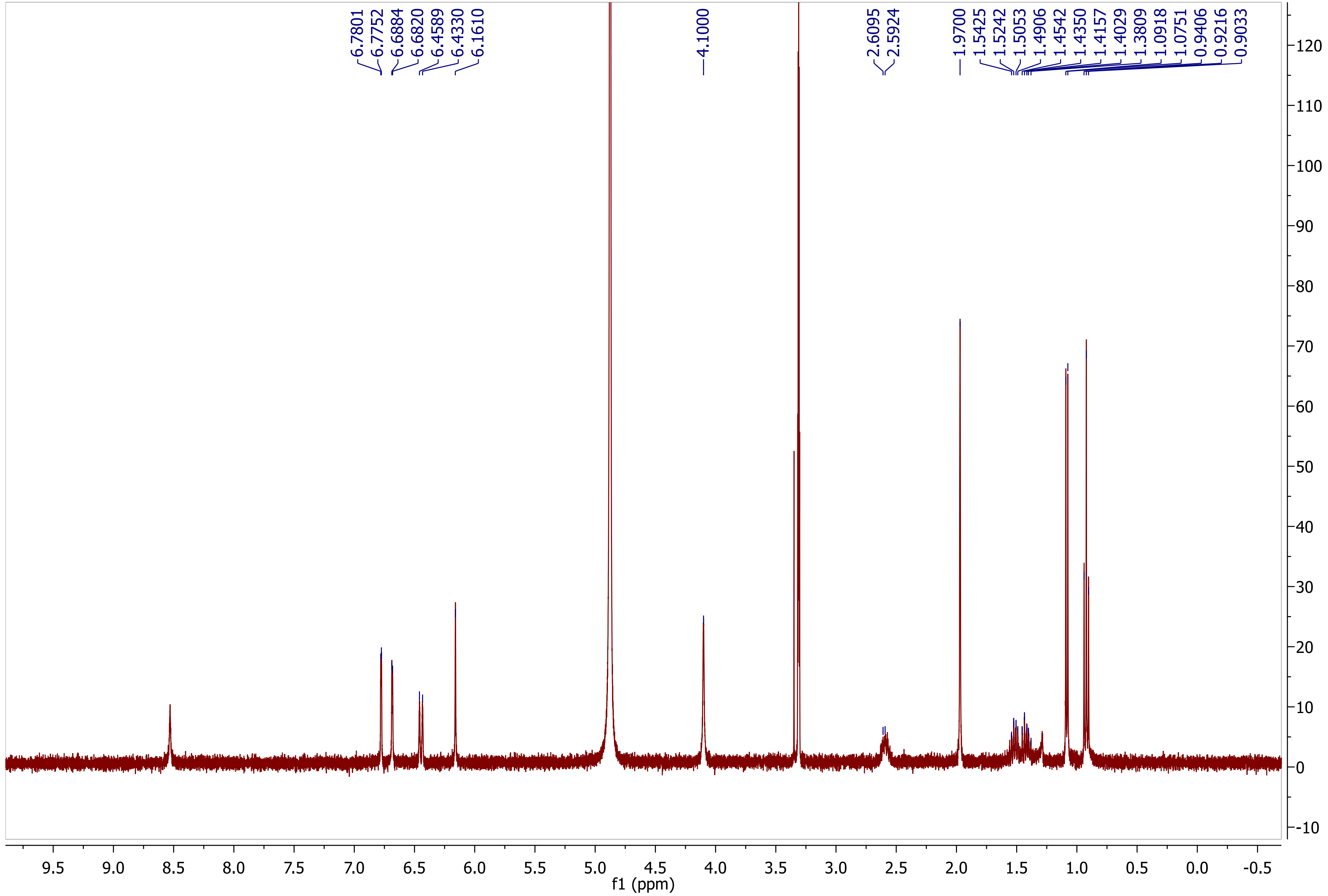

^{13}CNMR spectrum of compound 4

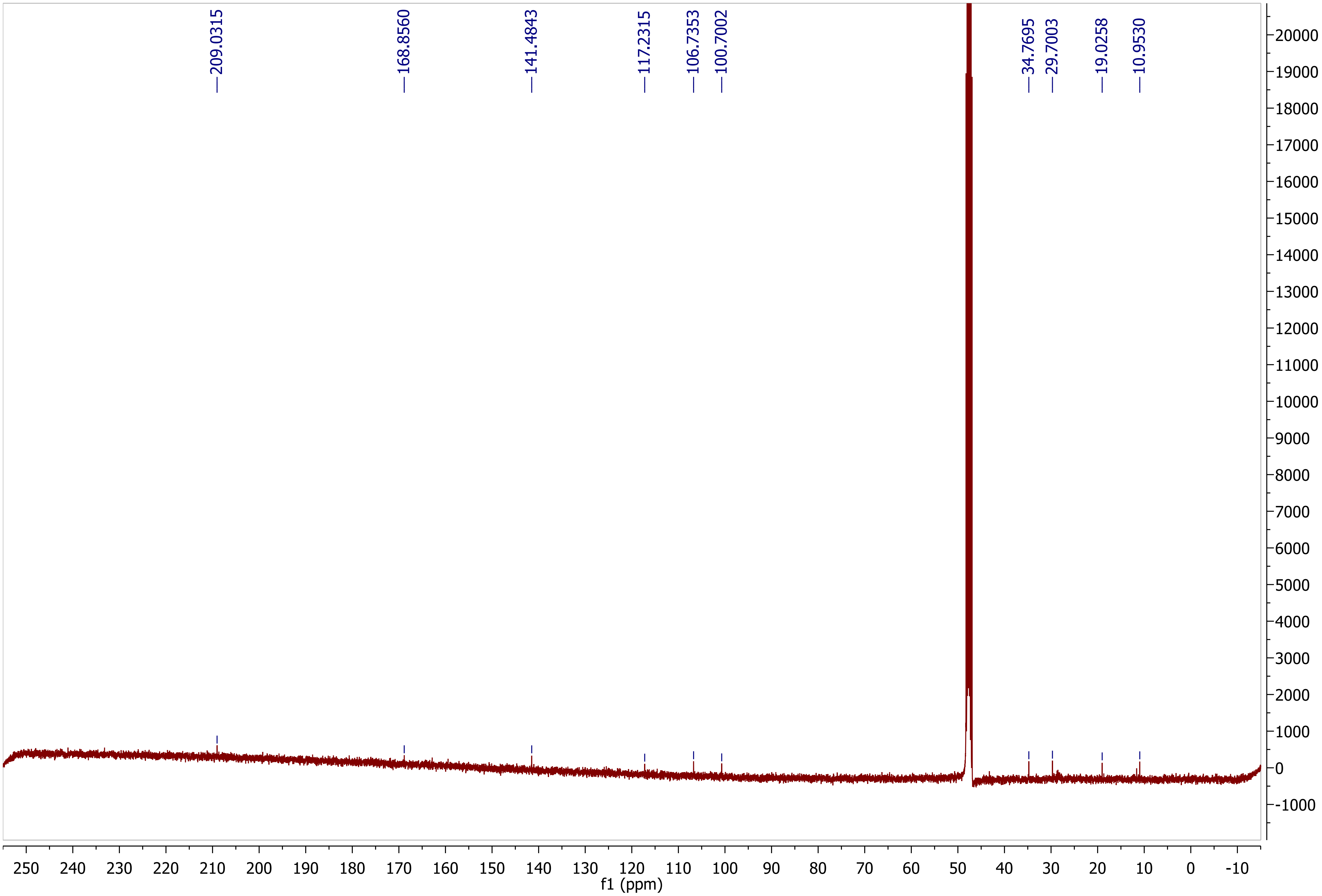

^1H-^1H COSY spectrum of compound 4

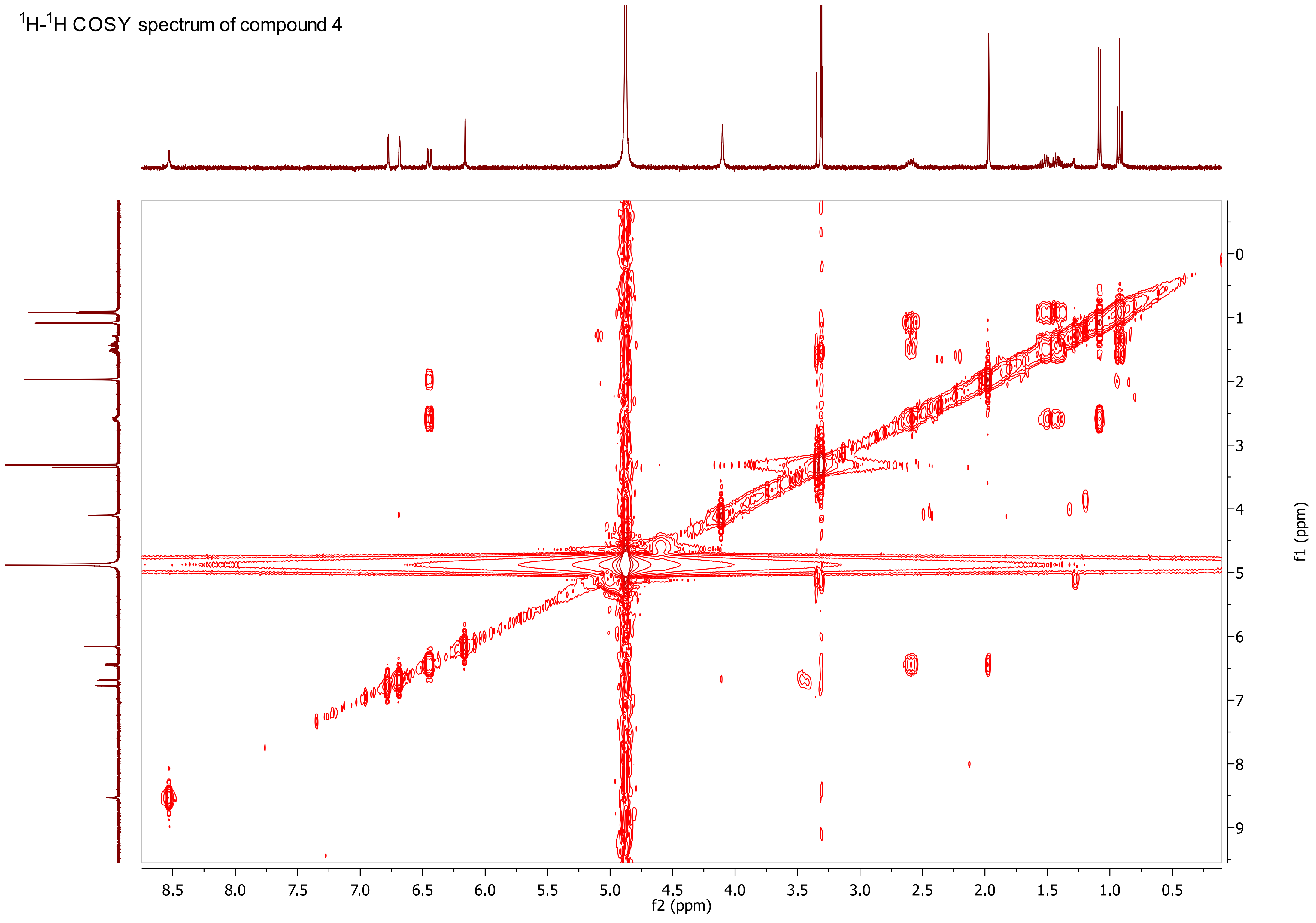

HSQC spectrum of compound 4

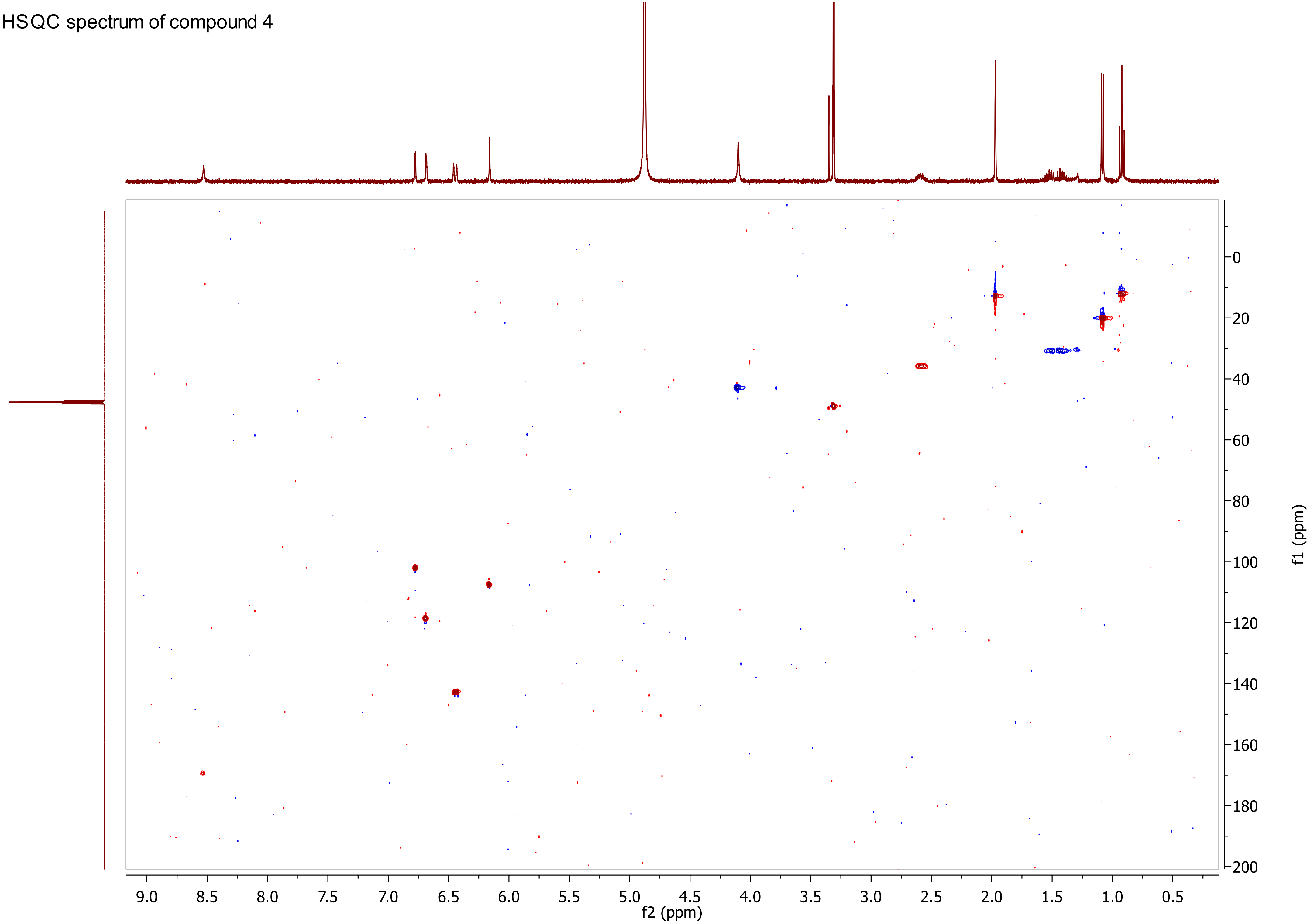

HMBC spectrum of compound 4

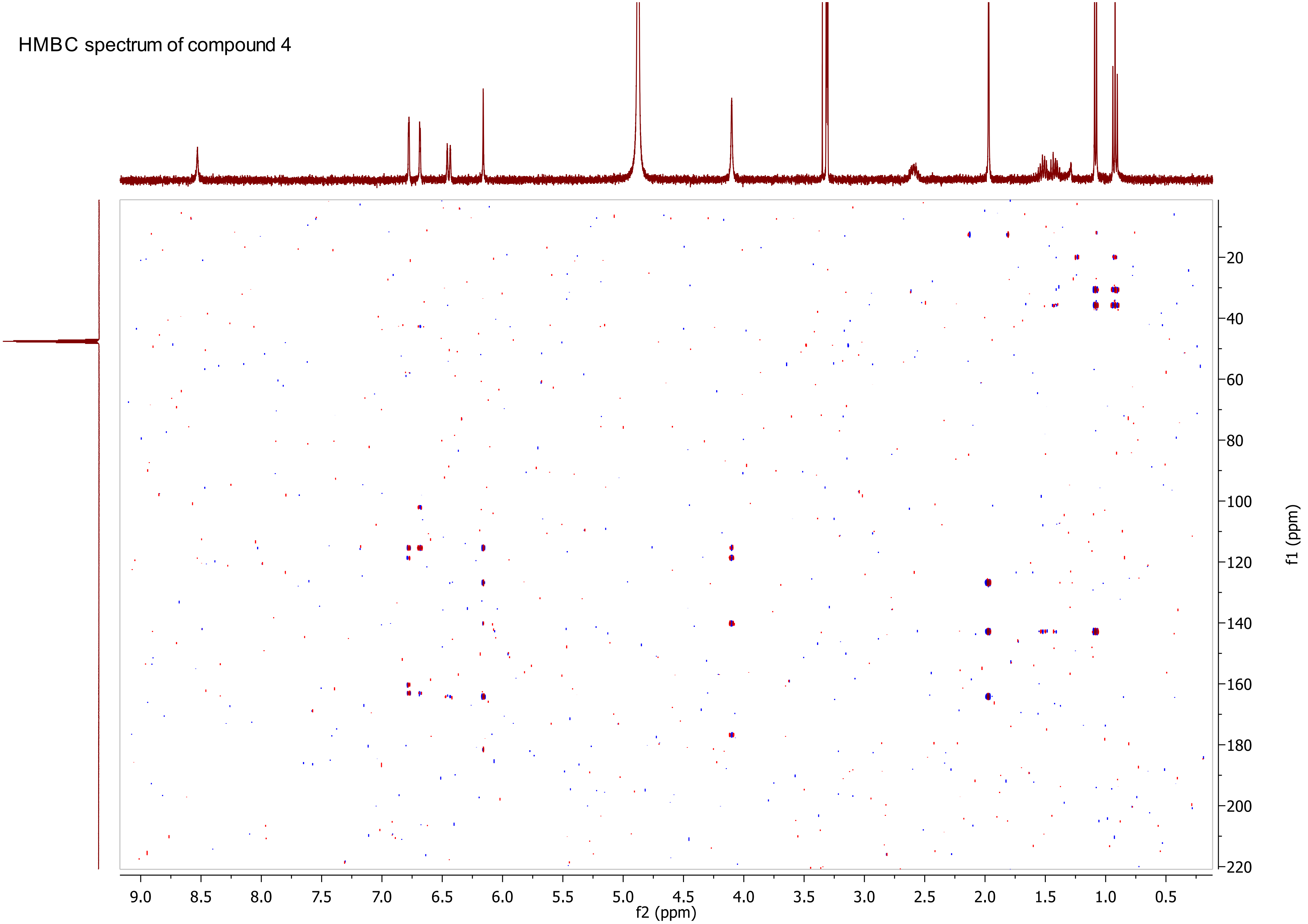

NOESY spectrum of compound 4

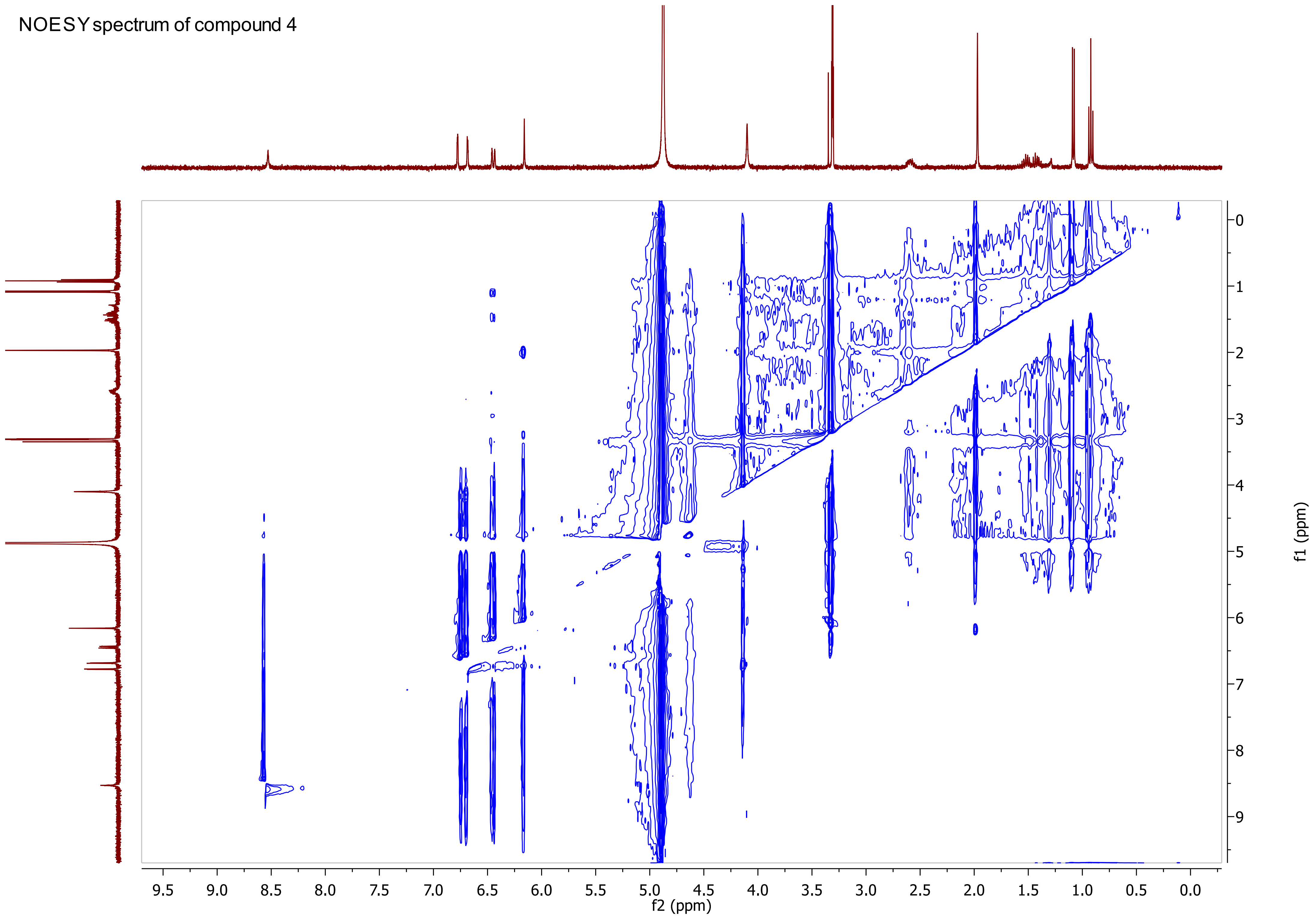

HRESIMS of compound **1**

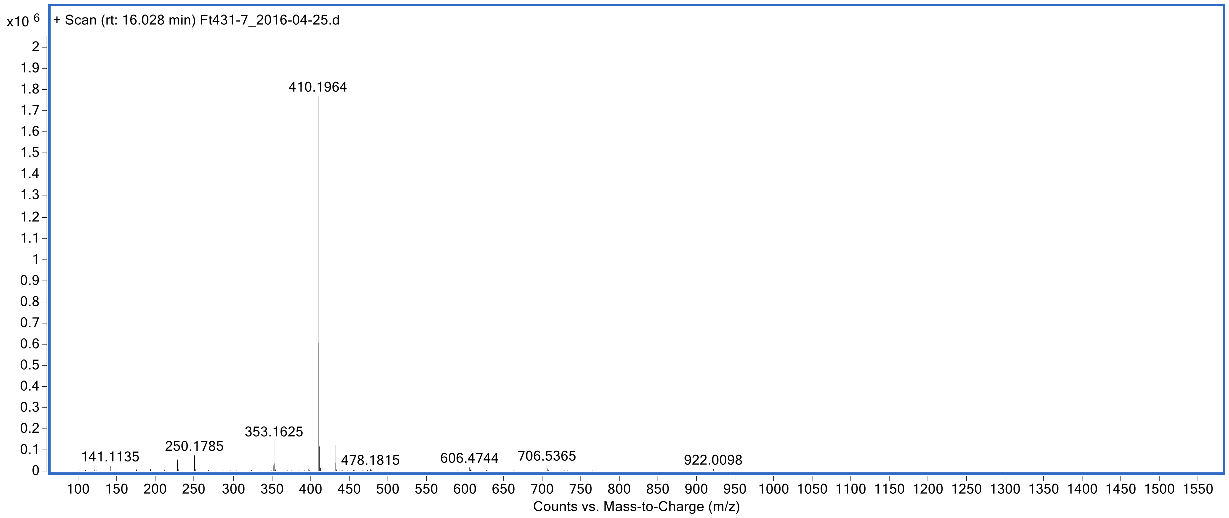

HRESIMS of compound **2**

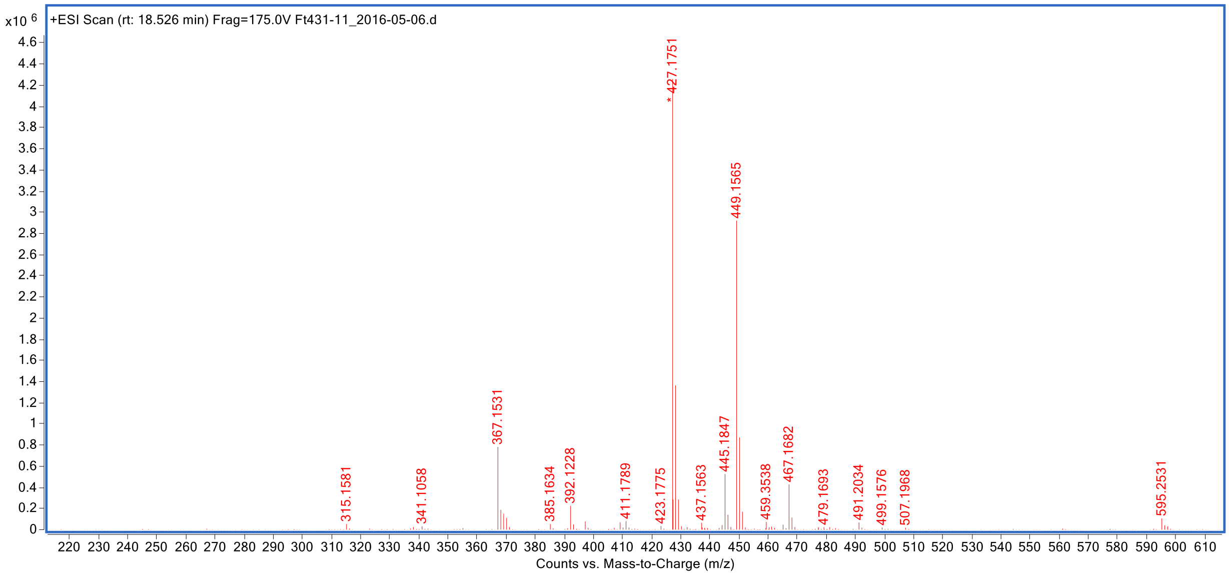

HRESIMS of compound **3**

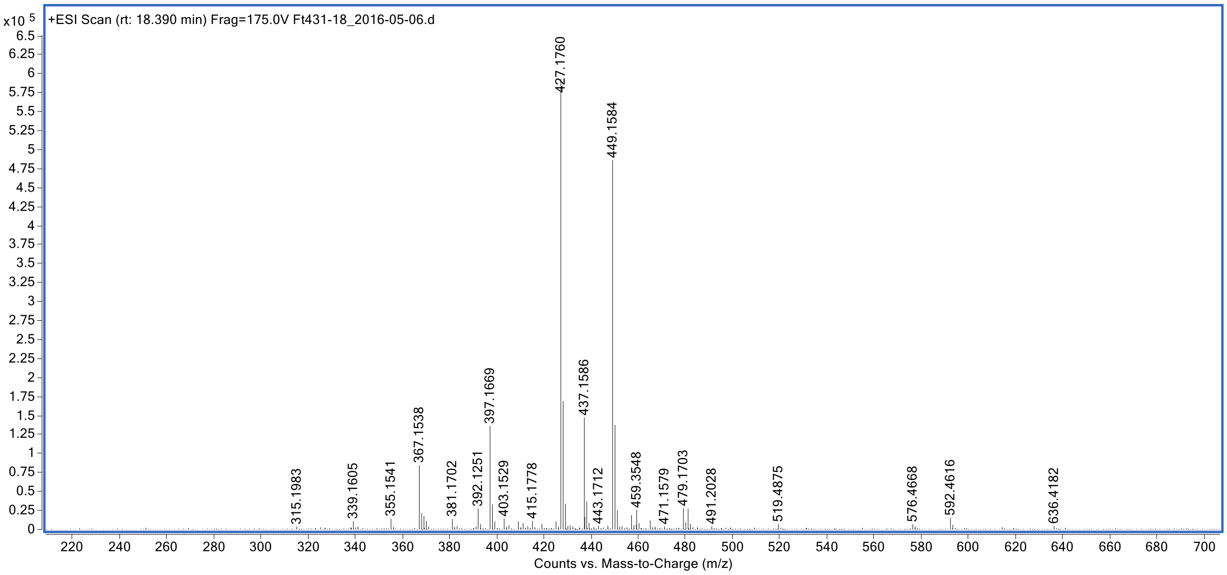

HRESIMS of compound **4**

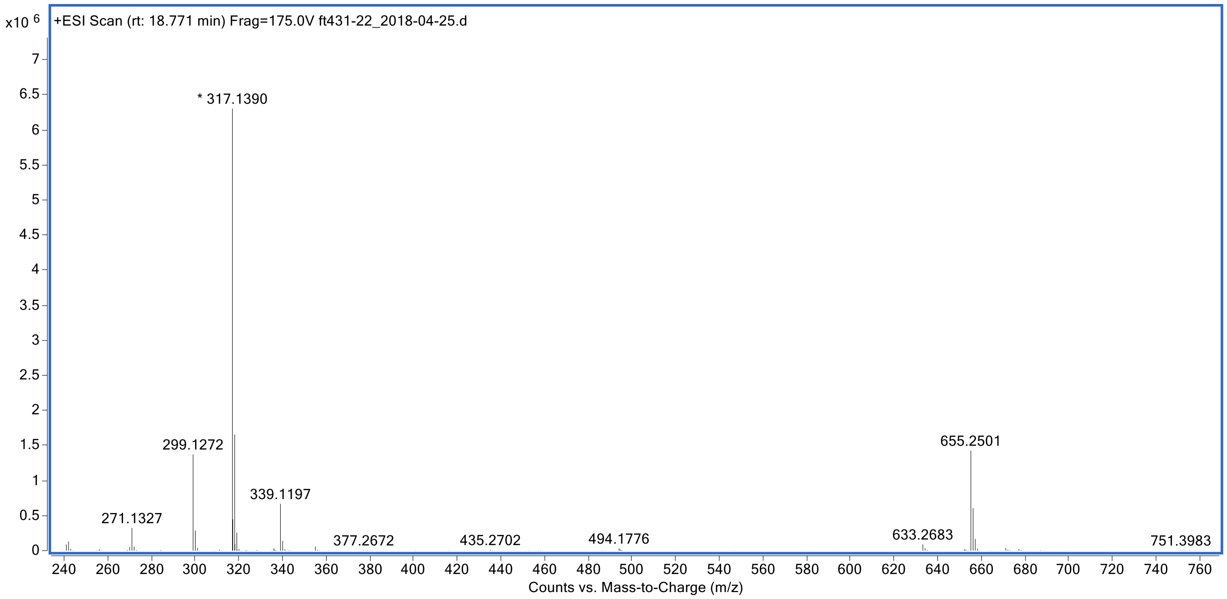

IR of compound **1**

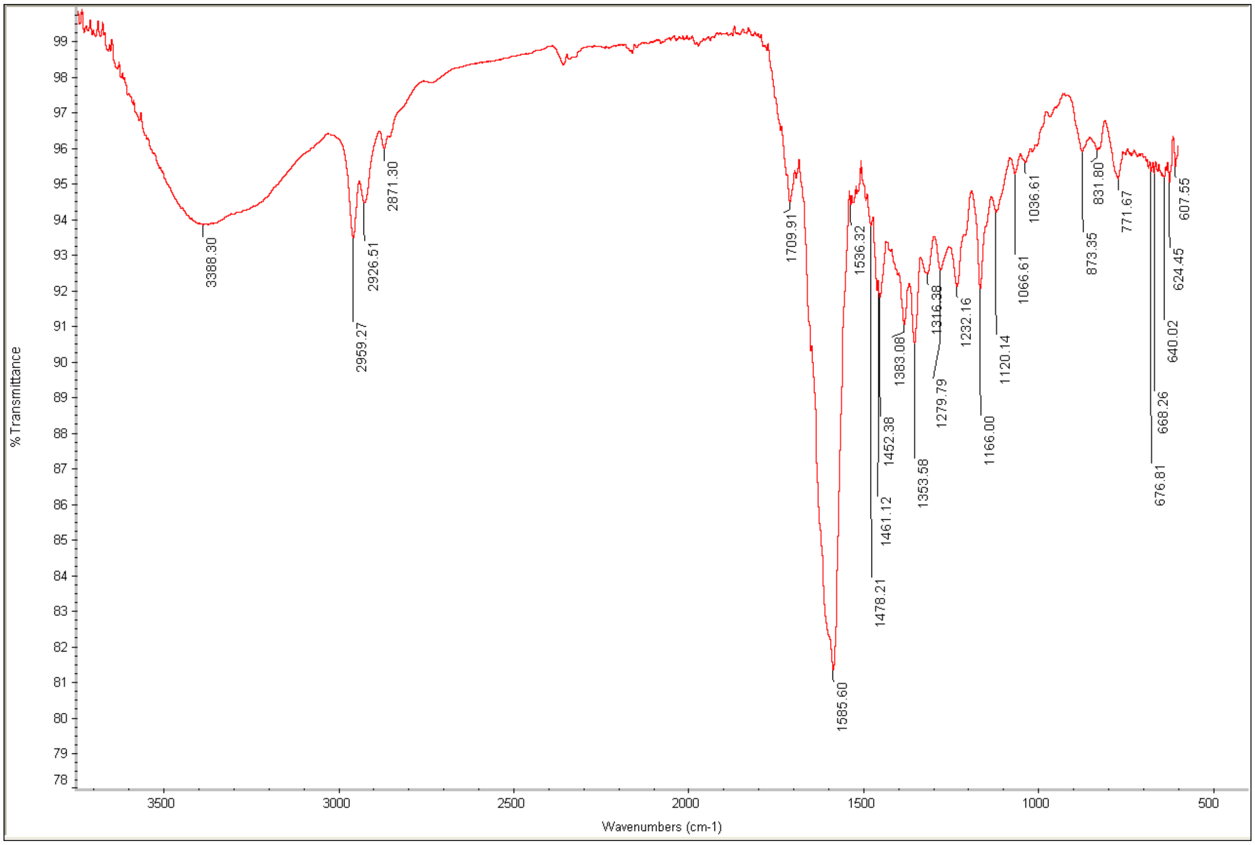

IR of compound **2**

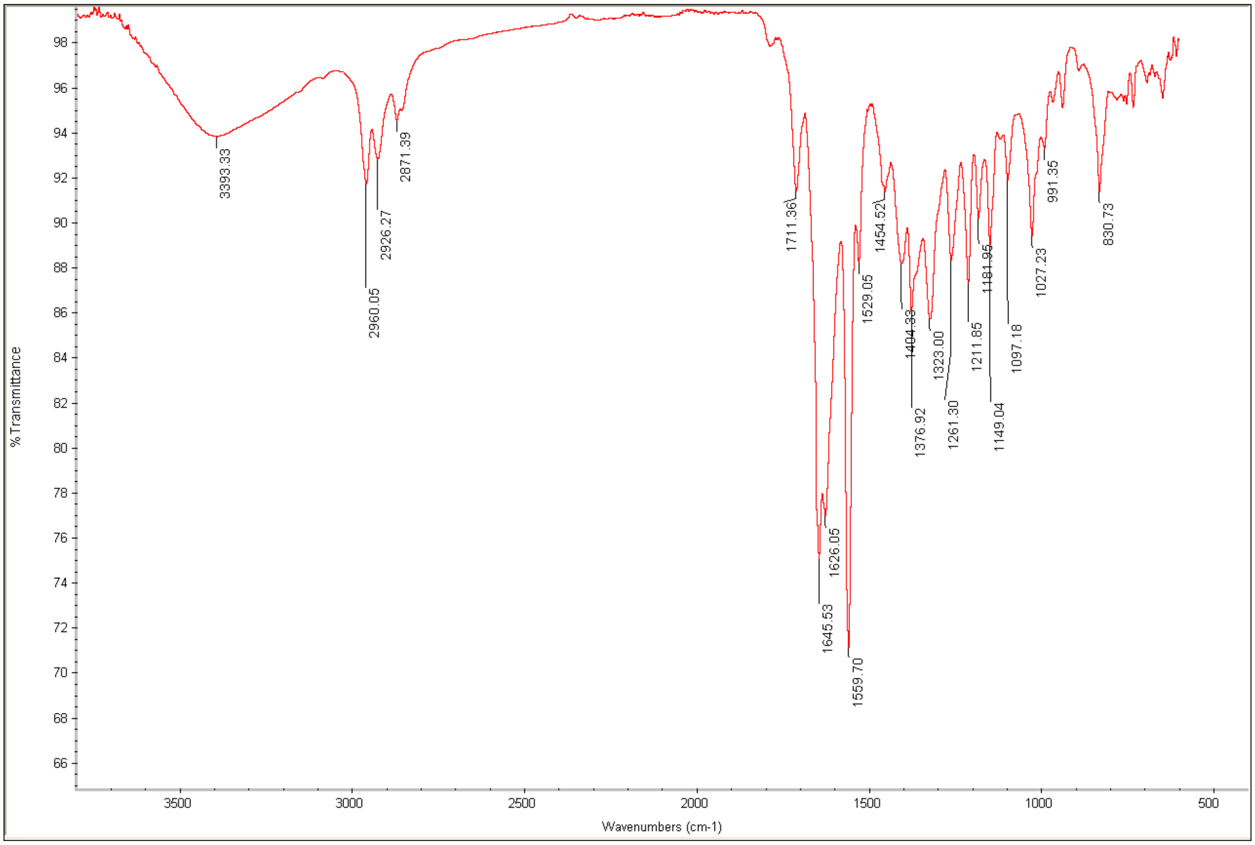

IR of compound **3**

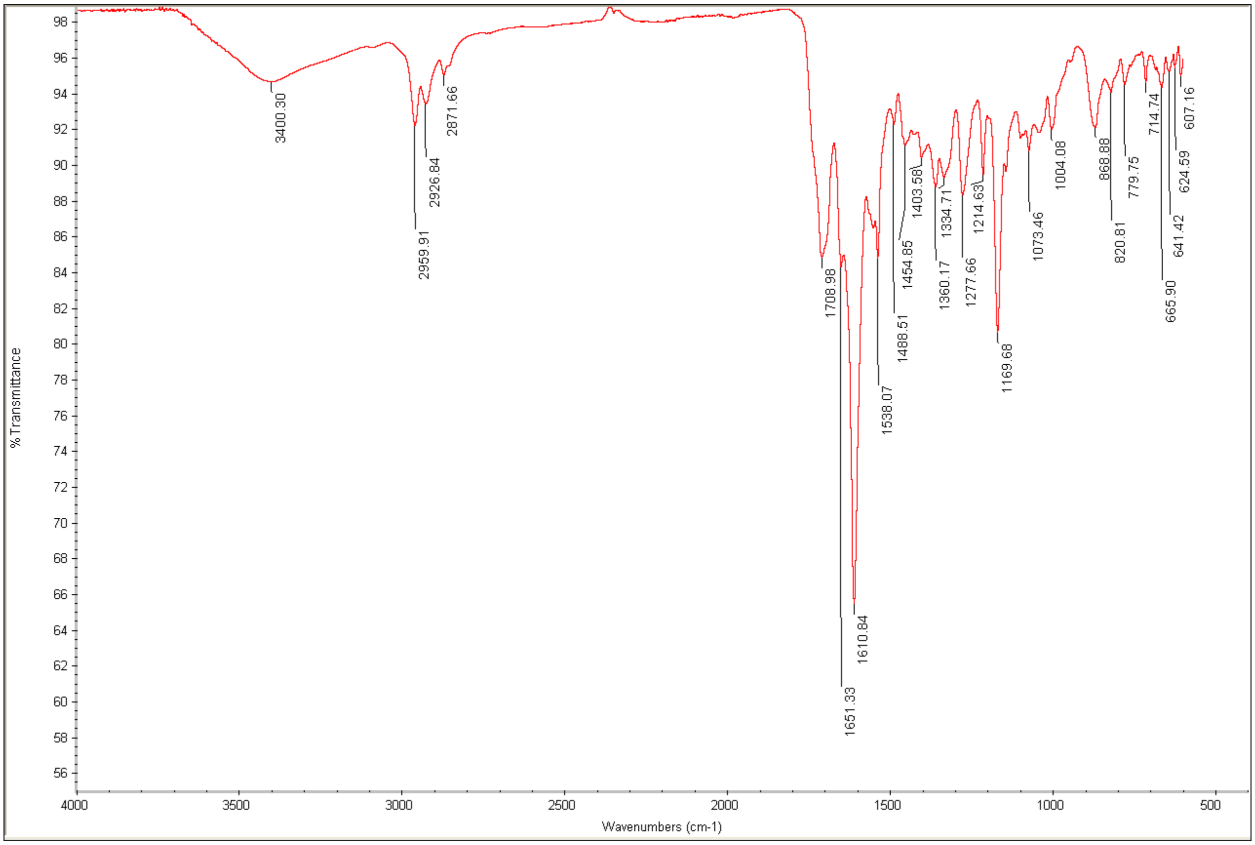

IR of compound **4**

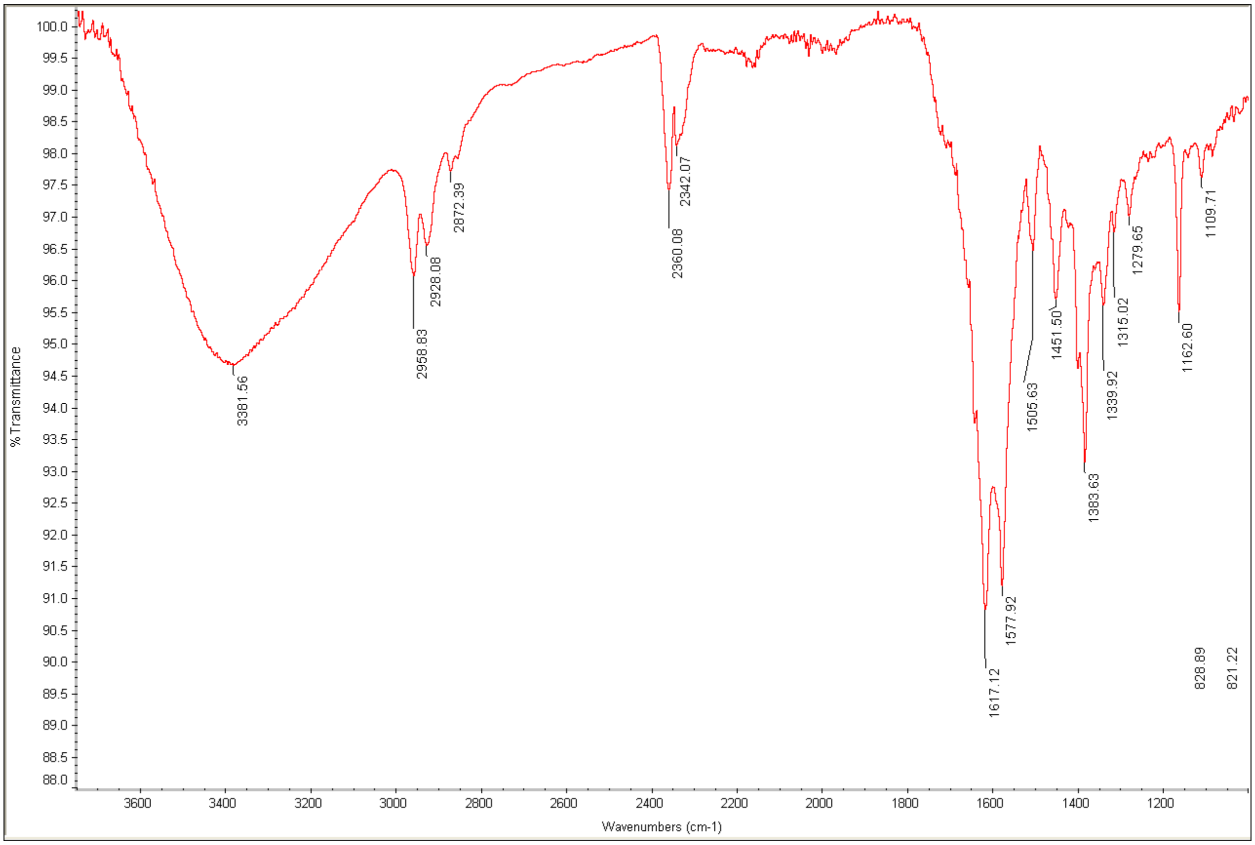

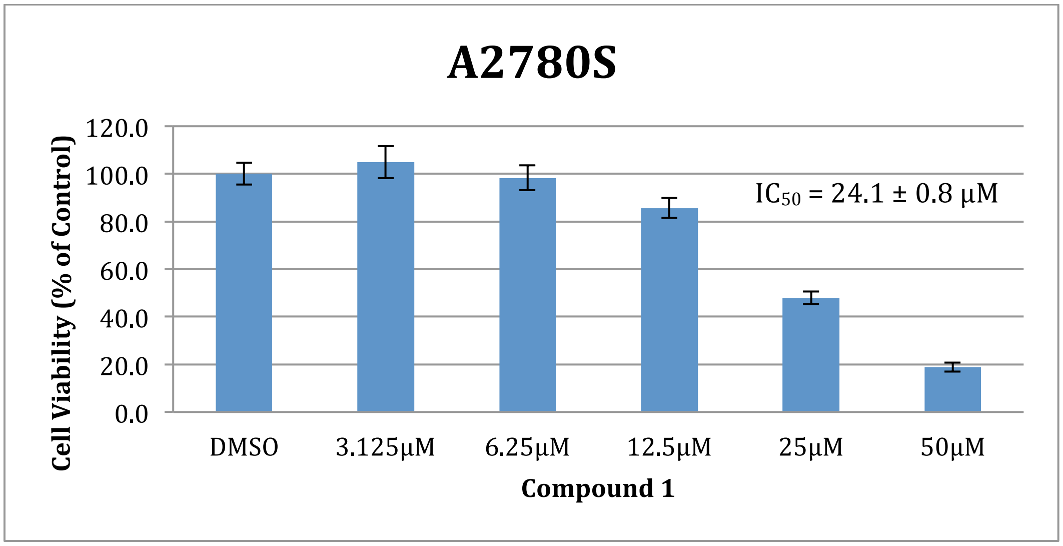
A2780S
120.0
100.0
80.0
60.0
40.0
20.0
0.0
Cell Viability (% of Control)
IC50 = 24.1 ± 0.8 μM
DMSO
3.125μM
6.25μM
12.5μM
25μM
50μM
Compound 1

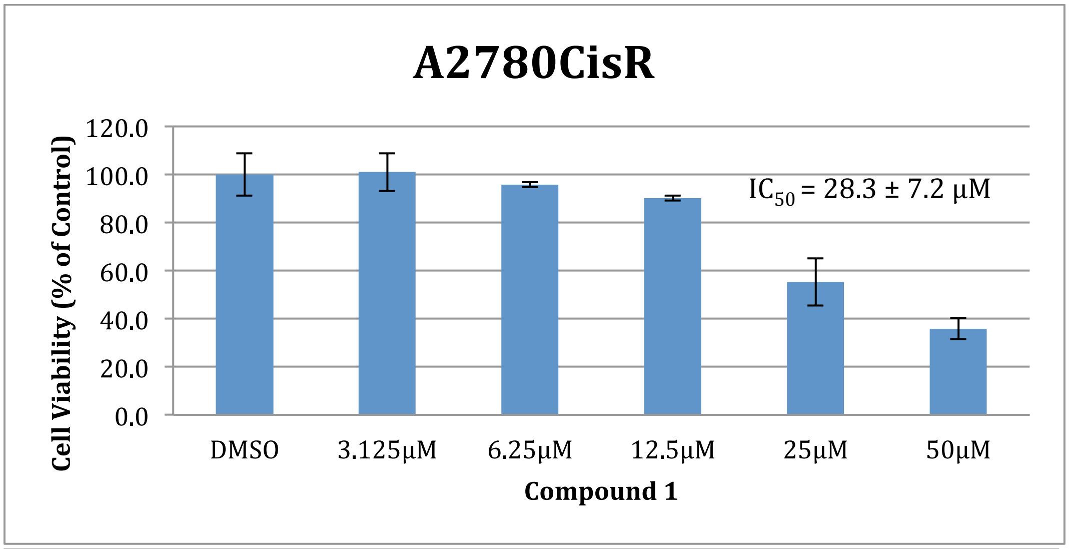
A2780CisR
120.0
100.0
80.0
60.0
40.0
20.0
0.0
Cell Viability (% of Control)
IC50 = 28.3 ± 7.2 μM
DMSO
3.125μM
6.25μM
12.5μM
25μM
50μM
Compound 1

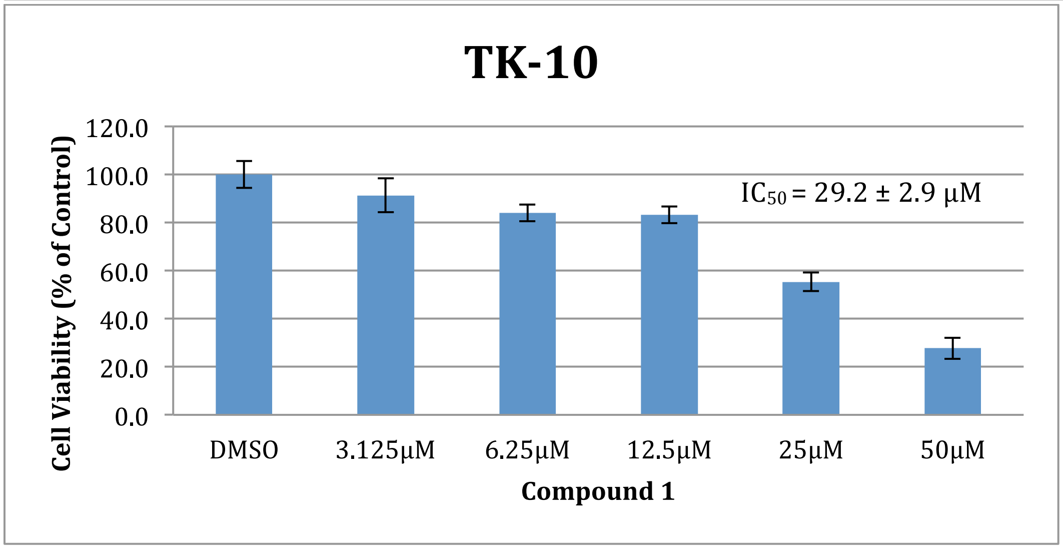
TK-10
120.0
100.0
80.0
60.0
40.0
20.0
0.0
Cell Viability (% of Control)
IC50 = 29.2 ± 2.9 μM
DMSO
3.125μM
6.25μM
12.5μM
25μM
50μM
Compound 1

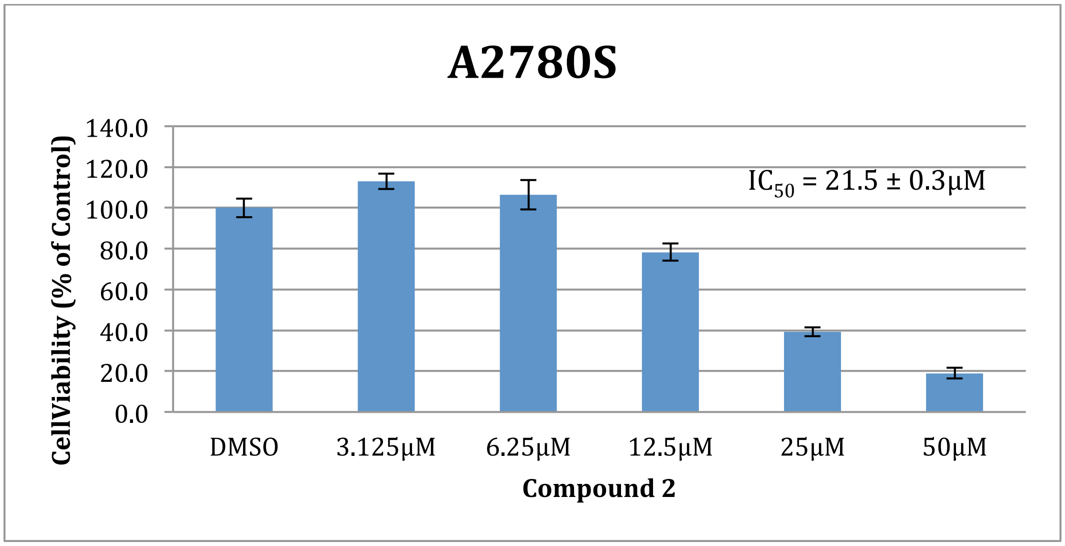
A2780S
140.0
120.0
100.0
80.0
60.0
40.0
20.0
0.0
CellViability (% of Control)
IC50 = 21.5 ± 0.3µM
DMSO
3.125µM
6.25µM
12.5µM
25µM
50µM
Compound 2

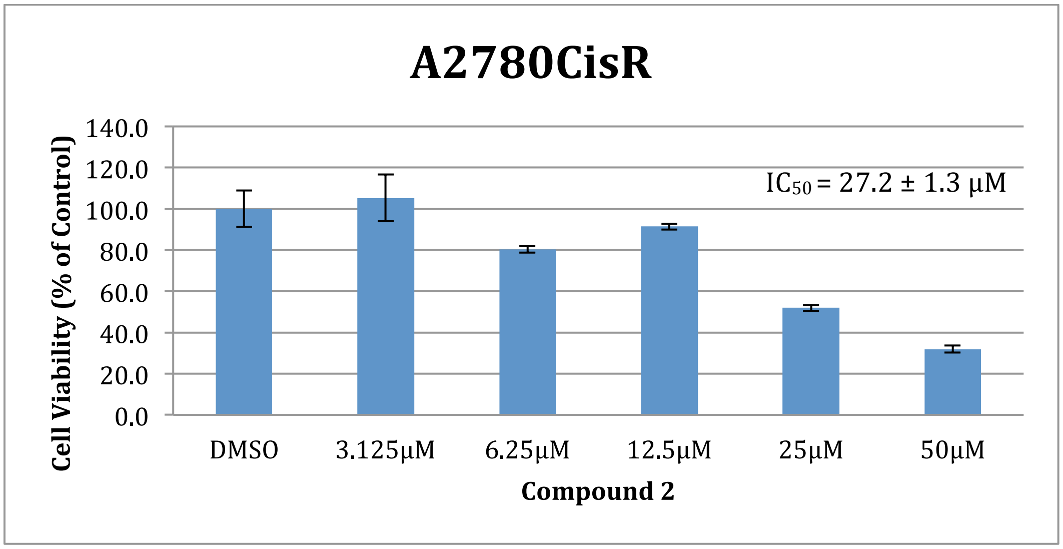
A2780CisR
140.0
120.0
100.0
80.0
60.0
40.0
20.0
0.0
Cell Viability (% of Control)
IC50 = 27.2 ± 1.3 µM
DMSO
3.125µM
6.25µM
12.5µM
25µM
50µM
Compound 2

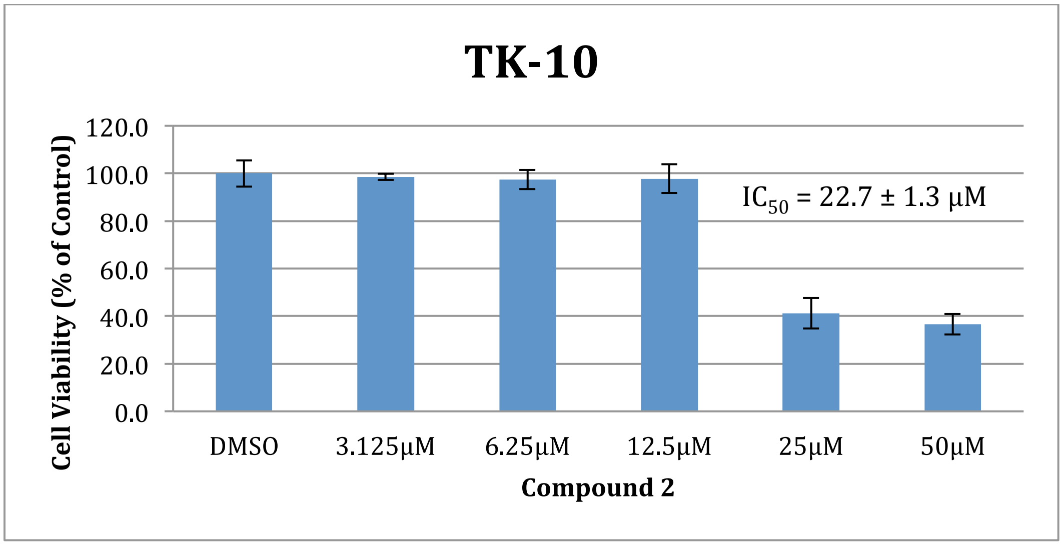
TK-10
120.0
100.0
80.0
60.0
40.0
20.0
0.0
Cell Viability (% of Control)
IC50 = 22.7 ± 1.3 µM
DMSO
3.125µM
6.25µM
12.5µM
25µM
50µM
Compound 2

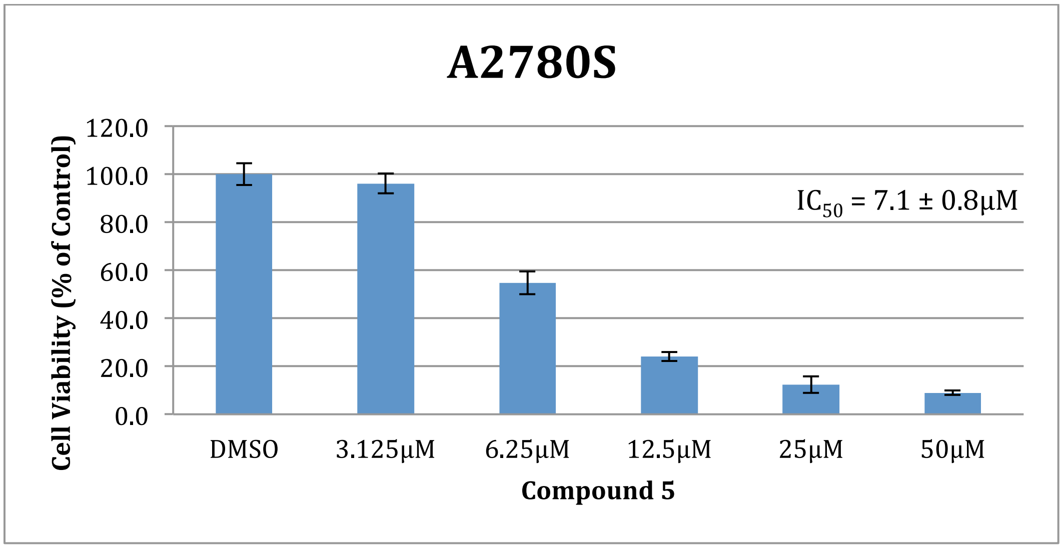
A2780S
Cell Viability (% of Control)
120.0
100.0
80.0
60.0
40.0
20.0
0.0
IC$_{50}$ = 7.1 ± 0.8μM
DMSO
3.125μM
6.25μM
12.5μM
25μM
50μM
Compound 5

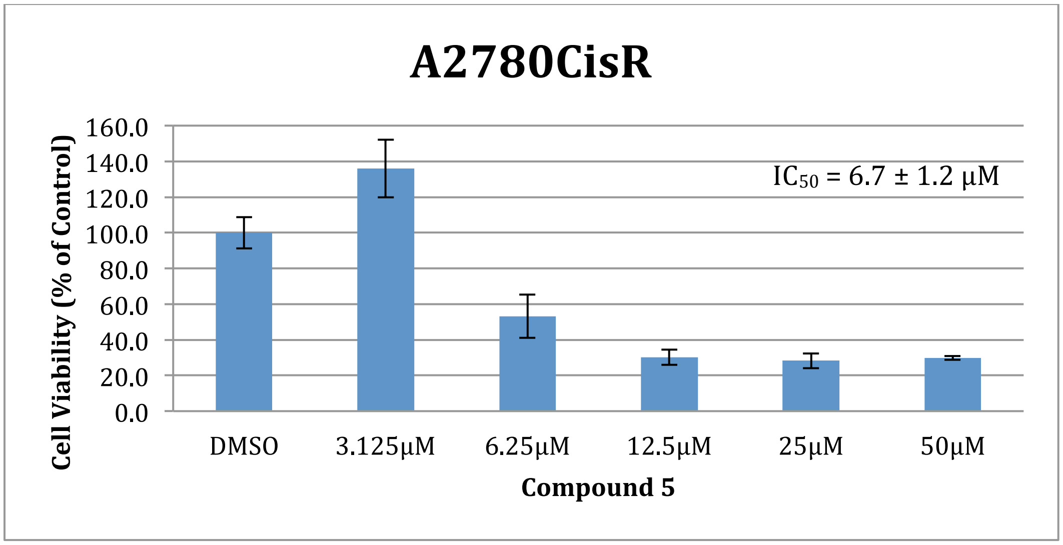
A2780CisR
Cell Viability (% of Control)
160.0
140.0
120.0
100.0
80.0
60.0
40.0
20.0
0.0
IC$_{50}$ = 6.7 ± 1.2 μM
DMSO
3.125μM
6.25μM
12.5μM
25μM
50μM
Compound 5

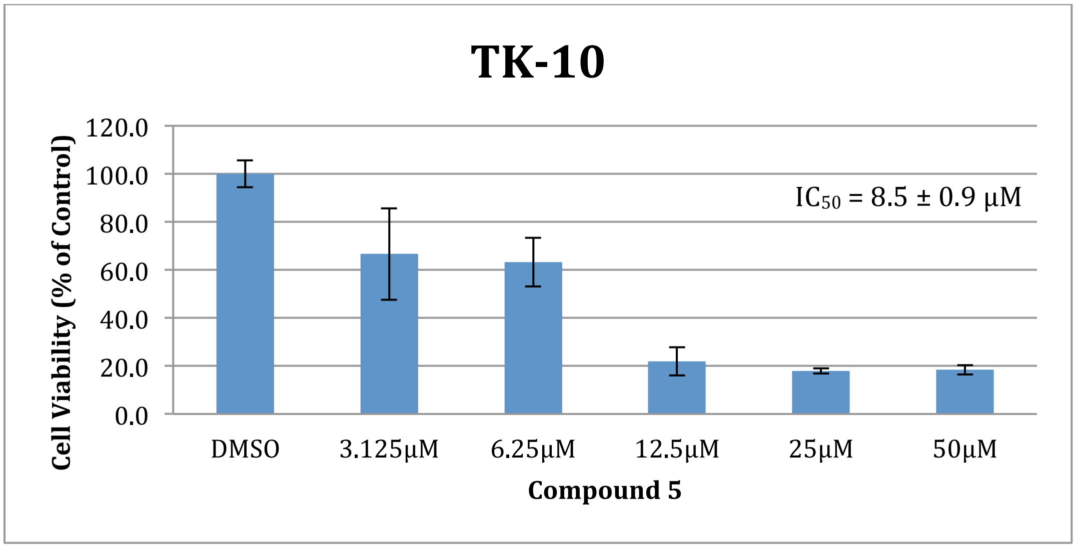
TK-10
Cell Viability (% of Control)
120.0
100.0
80.0
60.0
40.0
20.0
0.0
IC$_{50}$ = 8.5 ± 0.9 μM
DMSO
3.125μM
6.25μM
12.5μM
25μM
50μM
Compound 5

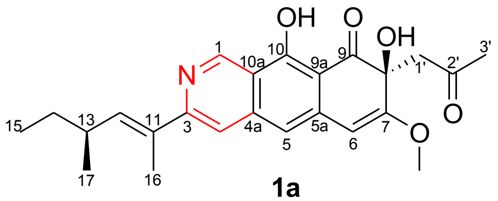

1a

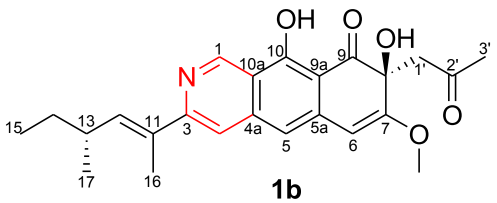

1b

Figure S32. Structures of Isomers **1a** and **1b**

Table S1. NMR Boltzmann averaged isotropic magnetic shielding values (σ), unscaled (δu) and scaled (δs) chemical shifts calculated at the B3LYP/6-31G**//MMFF level of theory for all significantly populated conformers of **1a** and **1b**.

Atom	δexp	σ (ppm)		δu (ppm)		δs (ppm)	
		**1a**	**1b**	**1a**	**1b**	**1a**	**1b**
C-1	148.4	42.2118	42.2116	150.1	150.1	151.9	151.9
C-3	158.5	34.9649	35.0431	157.3	157.3	159.3	159.2
C-4	115.0	81.7147	81.7271	110.6	110.6	111.5	111.5
C-4a	143.3	58.9351	58.9378	133.4	133.4	134.8	134.8
C-5	114.7	77.7316	77.7284	114.6	114.6	115.6	115.6
C-5a	133.3	58.2568	58.2566	134.0	134.0	135.5	135.5
C-6	98.9	91.6402	91.6084	100.7	100.7	101.4	101.4
C-7	161.6	33.3127	33.3489	159.0	158.9	161.0	160.9
C-8	72.8	111.5648	111.5082	80.7	80.8	81.0	81.1
C-9	202.8	-5.9091	-5.9655	198.2	198.3	201.1	201.1
C-9a	107.7	83.1269	83.1626	109.2	109.1	110.1	110.0
C-10	164.7	33.8476	33.8619	158.4	158.4	160.4	160.4
C-10a	117.9	82.8353	82.8469	109.5	109.4	110.4	110.4
C-11	133.3	59.9030	60.0194	132.4	132.3	133.8	133.7
C-12	140.1	53.3124	53.1967	139.0	139.1	140.5	140.7
C-13	35.7	153.0835	153.1712	39.2	39.1	38.5	38.5
C-14	31.0	159.2510	159.3453	33.0	32.9	32.2	32.2
C-15	12.4	177.2822	177.3138	15.0	15.0	13.8	13.8
C-16	14.5	175.1937	175.1952	17.1	17.1	15.9	16.0
C-17	20.8	169.6919	169.7194	22.6	22.6	21.6	21.6
C-1'	50.6	151.0257	150.9943	41.3	41.3	40.7	40.7
C-2'	205.7	-19.3088	-19.3085	211.6	211.6	214.8	214.8
C-3'	29.6	161.1608	161.2008	31.1	31.1	30.3	30.3
C-7-OCH_3	56.2	138.2133	138.1958	54.1	54.1	53.7	53.8

Table S2. NMR Boltzmann averaged isotropic magnetic shielding values (σ), unscaled (δu) and scaled (δs) chemical shifts calculated at the B3LYP/6-31G**//MMFF level of theory for all significantly populated conformers of **1a** and **1b** after removing all conformations showing intramolecular H-bonding between the OH group at C-8 and the ketone oxygen at C-2’

Atom	δexp	σ (ppm)		δu (ppm)		δs (ppm)	
		**1a**	**1b**	**1a**	**1b**	**1a**	**1b**
C-1	148.4	41.8929	41.9035	150.4	150.4	152.8	152.8
C-3	158.5	34.8855	34.8556	157.4	157.4	160.1	160.2
C-4	115	81.8390	81.8551	110.5	110.4	111.4	111.3
C-4a	143.4	58.7759	58.7552	133.5	133.5	135.3	135.3
C-5	114.7	76.6227	76.6477	115.7	115.6	116.8	116.7
C-5a	133.3	57.3767	57.3674	134.9	134.9	136.8	136.8
C-6	98.9	89.9390	89.9439	102.4	102.3	103.0	102.9
C-7	161.6	35.8929	35.8832	156.4	156.4	159.1	159.1
C-8	72.8	116.5285	116.5232	75.8	75.8	75.3	75.3
C-9	202.8	-6.4993	-6.5115	198.8	198.8	203.1	203.1
C-9a	107.7	82.7682	82.7684	109.5	109.5	110.4	110.4
C-10	164.7	32.4614	32.4558	159.8	159.8	162.6	162.6
C-10a	117.9	82.6554	82.6414	109.6	109.7	110.5	110.5
C-11	133.3	60.0065	59.9716	132.3	132.3	134.0	134.1
C-12	140.1	52.9746	53.0253	139.3	139.3	141.3	141.3
C-13	35.7	153.1028	152.9862	39.2	39.3	37.4	37.5
C-14	31.0	159.3249	159.2369	33.0	33.1	30.9	31.0
C-15	12.4	177.2554	177.2752	15.0	15.0	12.3	12.2
C-16	14.5	175.2243	175.2256	17.1	17.1	14.4	14.4
C-17	20.8	169.6797	169.6579	22.6	22.6	20.2	20.2
C-1'	50.6	138.4813	138.4841	53.8	53.8	52.5	52.5
C-2'	205.7	-11.1067	-11.1166	203.4	203.4	207.9	207.9
C-3'	29.6	161.3022	161.2923	31.0	31.0	28.9	28.8
C-7-OCH_3	56.2	138.4603	138.4739	53.8	53.8	52.6	52.5

Table S3. NMR Boltzmann averaged isotropic magnetic shielding values (σ), unscaled (δu) and scaled (δs) chemical shifts calculated at the PCM/mPW1PW91/6-31+G**//B3LYP/6-31G* level of theory for all significantly populated conformers of **1a** and **1b**.

Atom	δexp	σ (ppm)		δu (ppm)		δs (ppm)	
		**1a**	**1b**	**1a**	**1b**	**1a**	**1b**
C-1	148.4	48.7989	48.6680	148.0	148.1	148.0	147.7
C-3	158.5	41.1099	40.3361	155.7	156.4	155.7	156.0
C-4	115	84.2639	83.9254	112.5	112.9	112.1	112.3
C-4a	143.4	54.8453	54.7100	141.9	142.1	141.9	141.6
C-5	114.7	84.3272	84.3967	112.5	112.4	112.1	111.8
C-5a	133.3	59.0246	59.3811	137.8	137.4	137.6	136.9
C-6	98.9	98.5402	98.5207	98.2	98.3	97.7	97.7
C-7	161.6	36.1169	37.2528	160.7	159.5	160.8	159.1
C-8	72.8	118.1014	116.2493	78.7	80.5	77.9	79.9
C-9	202.8	-2.1825	-3.1225	199.0	199.9	199.5	199.6
C-9a	107.7	91.0737	91.4428	105.7	105.3	105.2	104.8
C-10	164.7	32.3689	31.8074	164.4	165.0	164.6	164.6
C-10a	117.9	81.4411	81.4448	115.3	115.3	115.0	114.8
C-11	133.3	65.0331	64.3948	131.7	132.4	131.6	131.9
C-12	140.1	53.9260	54.2015	142.9	142.6	142.8	142.1
C-13	35.7	157.3424	157.5050	39.4	39.3	38.3	38.5
C-14	31.0	163.4556	163.4081	33.3	33.4	32.1	32.6
C-15	12.4	181.7341	182.1508	15.0	14.6	13.6	13.8
C-16	14.5	181.0072	181.0866	15.8	15.7	14.4	14.9
C-17	20.8	173.6222	174.4094	23.2	22.4	21.8	21.6
C-1'	50.6	151.2833	155.2875	45.5	41.5	44.4	40.7
C-2'	205.7	-17.7883	-20.9308	214.6	217.7	215.3	217.5
C-3'	29.6	162.6543	161.5472	34.1	35.2	32.9	34.5
C-7-OCH_3	56.2	141.4706	141.3485	55.3	55.4	54.3	54.7

Table S4. NMR Boltzmann averaged isotropic magnetic shielding values (σ), unscaled (δu) and scaled (δs) chemical shifts calculated at the PCM/mPW1PW91/6-31+G**//B3LYP/6-31G* level of theory for all significantly populated conformers of **1a** and **1b** after removing all conformations showing intramolecular H-bonding between the OH group at C-8 and the ketone oxygen at C-2'

Atom	δexp	σ (ppm)		δu (ppm)		δs (ppm)	
		**1a**	**1b**	**1a**	**1b**	**1a**	**1b**
C-1	148.4	48.7054	48.8694	148.1	147.9	148.7	148.3
C-3	158.5	40.4308	38.3606	156.3	158.4	157.1	159.0
C-4	115	84.1773	83.9061	112.6	112.9	112.5	112.6
C-4a	143.4	54.5169	55.2770	142.3	141.5	142.8	141.8
C-5	114.7	85.0927	84.4066	111.7	112.4	111.6	112.1
C-5a	133.3	59.6550	59.7340	137.1	137.0	137.5	137.2
C-6	98.9	98.1396	98.6079	98.6	98.2	98.3	97.6
C-7	161.6	36.3178	36.5264	160.5	160.3	161.3	160.9
C-8	72.8	121.1635	120.2459	75.6	76.5	74.8	75.5
C-9	202.8	-2.5831	-2.4111	199.4	199.2	201.0	200.5
C-9a	107.7	90.6125	90.4436	106.2	106.3	106.0	105.9
C-10	164.7	33.0466	32.5233	163.7	164.3	164.7	164.9
C-10a	117.9	81.5849	81.3125	115.2	115.5	115.2	115.2
C-11	133.3	64.5876	62.1867	132.2	134.6	132.5	134.7
C-12	140.1	54.2110	55.2122	142.6	141.6	143.1	141.8
C-13	35.7	157.4888	158.2155	39.3	38.6	37.8	36.9
C-14	31.0	163.6469	163.5325	33.1	33.2	31.5	31.4
C-15	12.4	182.1309	183.1161	14.6	13.7	12.6	11.5
C-16	14.5	180.7408	180.1904	16.0	16.6	14.0	14.5
C-17	20.8	174.6066	174.5998	22.2	22.2	20.3	20.2
C-1'	50.6	143.2349	142.2998	53.5	54.5	52.3	53.1
C-2'	205.7	-11.0063	-10.8775	207.8	207.7	209.6	209.2
C-3'	29.6	164.8823	163.9638	31.9	32.8	30.2	31.0
C-7-OCH_3	56.2	141.3265	141.4370	55.5	55.3	54.2	54.0

Cartesian Coordinates of all conformations found for each isomer at the MMFF level of theory, with SCF energies computed at the B3LYP/6-31G level.**

1a_c001
MMFF Geometry
C -1.569074 -2.501497 -0.061078
C -2.310989 0.098175 -0.327008
N -2.893180 -2.213504 -0.032818
C -0.547956 -1.558617 -0.219280
C -0.944481 -0.217154 -0.359060
C -3.249795 -0.917125 -0.155383
C   0.035541 0.776775 -0.531739
C   1.402748 0.459499 -0.560511
C   1.793134 -0.874702 -0.412177
C   0.824690 -1.880377 -0.250097
C   3.234954 -1.210094 -0.446435
C   4.276075 -0.080735 -0.519561
C   3.703830 1.307136 -0.733424
C   2.378692 1.536217 -0.752097
O   1.167740 -3.206648 -0.122238
O   3.578023 -2.398343 -0.454944
O   5.057698 -0.403193 -1.694830
C   5.226190 -0.160896 0.682200
C   4.585909 0.305189 1.969279
C   5.493836 0.949240 2.982753
O   3.392718 0.121615 2.207969
O   4.679506 2.280270 -0.874770
C   4.276641 3.601284 -1.214803
C -4.706511 -0.644257 -0.121879
C -5.538665 -1.629258 -0.897846
C -5.191629 0.400022 0.583439
C -6.640587 0.800177 0.759148
C -6.920732 2.184783 0.146209
C -6.744302 2.218503 -1.366858
C -6.982754 0.808885 2.252681
H -1.338766 -3.558851 0.051007
H -2.640387 1.125339 -0.448830
H -0.277955 1.814270 -0.642073
H   1.973511 2.531891 -0.891940
H   2.145395 -3.284089 -0.196352
H   5.265021 -1.355569 -1.634715
H   6.116855 0.450845 0.489503
H   5.590782 -1.183224 0.840010
H   4.915309 1.240162 3.863911
H   6.267730 0.239110 3.283971
H   5.948519 1.844198 2.550754
H   3.726756 3.613254 -2.161426
H   3.684180 4.047346 -0.409693
H   5.179594 4.205803 -1.341674
H -5.655153 -2.561911 -0.335836
H -6.535704 -1.249980 -1.134574
H -5.068404 -1.861435 -1.860647
H -4.492588 1.032267 1.131794
H -7.310543 0.075380 0.286361
H -7.952208 2.478984 0.377372
H -6.269073 2.942629 0.599339
H -7.376138 1.467686 -1.851914
H -7.028398 3.201730 -1.755510
H -5.704938 2.035278 -1.655595
H -6.804662 -0.174802 2.701569
H -8.038501 1.057593 2.406135
H -6.382046 1.543970 2.800417
SCF Energy (B3LYP/6-31G**)= -1361.71051590
Number of imaginary frequencies = 0

1a_c002
MMFF Geometry
C   1.595308 2.466820 -0.049250
C   2.341887 -0.130880 -0.322610
N   2.920736 2.183539 -0.043223
C   0.574859 1.520274 -0.188453
C   0.974037 0.179644 -0.332465
C   3.279826 0.888055 -0.168910
C -0.004524 -0.818355 -0.486762
C -1.371831 -0.504409 -0.494405
C -1.766828 0.828965 -0.340732
C -0.799400 1.837843 -0.196386
C -3.212581 1.160363 -0.354664
C -4.256768 0.029547 -0.420706
C -3.665245 -1.342478 -0.657168
C -2.347418 -1.580296 -0.661393
O -1.144547 3.163184 -0.065274
O -3.560375 2.347404 -0.362167
O -5.061728 0.376376 -1.570738
C -5.174858 0.080172 0.805381
C -4.486257 -0.406851 2.059046
C -5.283296 -1.295123 2.975739
O -3.339975 -0.058418 2.340753
O -4.532306 -2.428054 -0.693956
C -5.253906 -2.501115 -1.923851
C   4.737897 0.620383 -0.159171
C   5.553696 1.607313 -0.949871
C   5.238290 -0.421359 0.539198
C   6.691290 -0.816400 0.691395
C   6.966012 -2.200739 0.075403
C   6.764749 -2.236772 -1.434508
C   7.058090 -0.822255 2.179084
H   1.363348 3.523553 0.065156
H   2.672541 -1.157299 -0.447645
H   0.308590 -1.855799 -0.599584
H -1.981743 -2.597822 -0.765500
H -2.123965 3.236793 -0.112848
H -5.240803 1.335250 -1.498013
H -6.067676 -0.532249 0.625967
H -5.542634 1.095671 0.996027
H -4.672747 -1.577899 3.837704
H -6.167619 -0.758224 3.327365
H -5.578216 -2.201919 2.441850
H -6.138755 -1.859660 -1.884510
H -4.626267 -2.246784 -2.785200
H -5.597093 -3.532689 -2.047450
H   5.675983 2.541129 -0.391033
H   6.548107 1.231326 -1.202371
H   5.066941 1.836509 -1.905159
H   4.550599 -1.055505 1.099641
H   7.350872 -0.089868 0.206833
H   8.002163 -2.491168 0.289822
H   6.324517 -2.960285 0.540045
H   7.385919 -1.484357 -1.930736
H   7.045756 -3.219465 -1.826740
H   5.720139 -2.057415 -1.706242
H   6.884073 0.161329 2.629793
H   8.117069 -1.067202 2.315354
H   6.469019 -1.558755 2.737454
SCF Energy (B3LYP/6-31G**)= -1361.70481780
Number of imaginary frequencies = 0

1a_c003
MMFF Geometry
C -1.635440 -2.282848 -0.042720
C -2.239425 0.333558 0.368586
N -2.923074 -1.965122 0.236526
C -0.586367 -1.362492 -0.137412
C -0.911011 -0.011890 0.078824
C -3.213252 -0.661019 0.431693
C   0.101275 0.961353 -0.000284
C   1.430315 0.613422 -0.289049
C   1.749623 -0.731425 -0.498578
C   0.747404 -1.714479 -0.430301
C   3.150481 -1.098200 -0.807730
C   4.244290 -0.017916 -0.771469
C   3.740194 1.400263 -0.586857
C   2.441182 1.671853 -0.368654
O   1.018453 -3.045790 -0.647463
O   3.412214 -2.263475 -1.129177
O   4.845635 -0.086695 -2.086663
C   5.333975 -0.404710 0.236681
C   4.895799 -0.226577 1.671950
C   5.965357 0.139388 2.666106
O   3.734387 -0.422831 2.028317
O   4.750540 2.346399 -0.641110

C 4.393755 3.723007 -0.615589
C -4.631202 -0.356796 0.739287
C -5.280761 -1.326771 1.689137
C -5.247278 0.696674 0.161486
C -6.691247 1.120598 0.321809
C -7.412844 1.189375 -1.038150
C -7.528142 -0.166463 -1.724079
C -6.739728 2.486145 1.012000
H -1.459743 -3.345720 -0.194474
H -2.508872 1.368064 0.557130
H -0.156215 2.006332 0.168738
H 2.086624 2.685947 -0.223742
H 1.971008 -3.136699 -0.874786
H 4.997220 -1.033650 -2.270825
H 6.230115 0.204733 0.063034
H 5.649087 -1.447506 0.109160
H 5.524820 0.238244 3.662060
H 6.726157 -0.644540 2.689970
H 6.414975 1.095033 2.385223
H 3.728904 3.972363 -1.448899
H 3.939615 3.990087 0.343895
H 5.310194 4.309808 -0.728142
H -4.601232 -1.580284 2.511338
H -6.181362 -0.922987 2.158142
H -5.553823 -2.250696 1.168462
H -4.684006 1.315260 -0.537746
H -7.242677 0.414781 0.950607
H -8.425270 1.585184 -0.888841
H -6.898621 1.888257 -1.710006
H -8.033970 -0.890809 -1.077754
H -8.110225 -0.071723 -2.646401
H -6.545798 -0.567795 -1.990908
H -6.252199 2.447483 1.992645
H -7.776142 2.804088 1.169094
H -6.238597 3.257412 0.415983
SCF Energy (B3LYP/6-31G**)= -1361.71058237
Number of imaginary frequencies = 0

1a_c004
MMFF Geometry
C -1.659852 -2.244851 -0.209893
C -2.273779 0.327019 0.413710
N -2.952671 -1.951464 0.072090
C -0.610080 -1.320488 -0.206696
C -0.940225 0.007083 0.118769
C -3.247551 -0.668772 0.373674
C 0.071591 0.983372 0.141419
C 1.404332 0.659830 -0.154059
C 1.731301 -0.663003 -0.471406
C 0.729208 -1.647498 -0.504784
C 3.139683 -1.002710 -0.789607
C 4.235741 0.071473 -0.654578
C 3.706132 1.460667 -0.375367
C 2.416160 1.714470 -0.120823
O 1.005076 -2.955462 -0.830297
O 3.408420 -2.135502 -1.207503
O 4.863429 0.082960 -1.957123
C 5.298204 -0.375928 0.355243
C 4.807325 -0.288700 1.781572
C 5.767196 0.259422 2.802752
O 3.688208 -0.687708 2.102685
O 4.627750 2.486301 -0.201211
C 5.190050 2.922815 -1.438930
C -4.671094 -0.391447 0.681013
C -5.336683 -1.439728 1.531358
C -5.277629 0.708739 0.186126
C -6.724490 1.118357 0.357086
C -7.421762 1.306225 -1.004325
C -7.523907 0.015539 -1.807933
C -6.786183 2.418489 1.162867
H -1.480489 -3.290991 -0.448622
H -2.547269 1.341695 0.685892
H -0.187671 2.010660 0.395433
H 2.104814 2.721086 0.143204
H 1.963336 -3.029924 -1.037986
H 4.989737 -0.854463 -2.206297
H 6.197435 0.243789 0.247078
H 5.622934 -1.407581 0.173343
H 5.290759 0.272225 3.786905
H 6.656053 -0.374577 2.846403
H 6.044088 1.281728 2.533469
H 6.030750 2.283036 -1.721554
H 4.443149 2.961144 -2.239683
H 5.578685 3.934633 -1.289317
H -4.671789 -1.764009 2.340555
H -6.245942 -1.077593 2.017359
H -5.599498 -2.314847 0.927705
H -4.702379 1.385560 -0.446225
H -7.286585 0.360835 0.911928
H -8.436953 1.688398 -0.839109
H -6.896101 2.060624 -1.603338
H -8.040712 -0.762001 -1.236427
H -8.089498 0.190948 -2.728700
H -6.536692 -0.361858 -2.091209
H -6.316201 2.293939 2.144930
H -7.825445 2.722435 1.328611
H -6.275010 3.238388 0.645479
SCF Energy (B3LYP/6-31G**)= -1361.70493532
Number of imaginary frequencies = 0

1a_c005
MMFF Geometry
C -1.838945 -1.634899 -0.314418
C -2.058944 1.058869 -0.063531
N -3.078086 -1.106576 -0.165835
C -0.654561 -0.894732 -0.350700
C -0.780794 0.498390 -0.222027
C -3.184270 0.236152 -0.038777
C 0.375010 1.299603 -0.258051
C 1.652059 0.737430 -0.413819
C 1.772592 -0.650128 -0.533125
C 0.626430 -1.462940 -0.509454
C 3.118772 -1.243386 -0.701113
C 4.361821 -0.341889 -0.618486
C 4.072863 1.144876 -0.544315
C 2.819188 1.623299 -0.453403
O 0.701319 -2.830127 -0.643942
O 3.219565 -2.451781 -0.944622
O 5.047782 -0.578792 -1.871279
C 5.293749 -0.829991 0.498070
C 4.776156 -0.504252 1.880051
C 5.807622 -0.258092 2.948600
O 3.573475 -0.492996 2.140426
O 5.219917 1.921460 -0.553722
C 5.082143 3.335310 -0.624465
C -4.562081 0.779295 0.142386
C -4.685963 2.087328 0.876051
C -5.617529 0.074664 -0.322785
C -7.085074 0.435678 -0.265058
C -7.875555 -0.548642 0.616684
C -7.466679 -0.503493 2.083706
C -7.656948 0.440212 -1.686880
H -1.823003 -2.718637 -0.407428
H -2.164735 2.134864 0.025863
H 0.272396 2.379730 -0.159922
H 2.617429 2.686294 -0.387157
H 1.642972 -3.078611 -0.781079
H 5.063118 -1.546959 -1.997928
H 6.284958 -0.374660 0.376330
H 5.451256 -1.914179 0.445919
H 5.310451 -0.034646 3.896578
H 6.425650 -1.150578 3.072860
H 6.428520 0.596482 2.668431
H 4.532770 3.632311 -1.523682
H 4.600710 3.725500 0.277842
H 6.085144 3.768139 -0.683721
H -4.438312 2.924444 0.215125
H -5.688733 2.260825 1.274060
H -4.016282 2.114225 1.743406
H -5.421277 -0.878633 -0.818697
H -7.228305 1.444827 0.133369
H -8.944875 -0.311307 0.552016

H -7.758703 -1.574282 0.244214
H -7.566078 0.509158 2.487245
H -8.108919 -1.166475 2.672341
H -6.432687 -0.832477 2.224280
H -7.116075 1.148151 -2.324976
H -8.710873 0.738891 -1.678869
H -7.592745 -0.550458 -2.151304
SCF Energy (B3LYP/6-31G**)= -1361.71155950
Number of imaginary frequencies = 0

1a_c006
MMFF Geometry
C -1.851935 -1.578096 -0.425112
C -2.090780 1.085088 0.042684
N -3.096285 -1.068676 -0.255364
C -0.671041 -0.832899 -0.380195
C -0.807261 0.544755 -0.139651
C -3.211854 0.258616 -0.019770
C 0.343947 1.351334 -0.089824
C 1.624871 0.808238 -0.270986
C 1.757340 -0.565350 -0.500782
C 0.615545 -1.381687 -0.562969
C 3.111438 -1.137880 -0.697558
C 4.353202 -0.240950 -0.533402
C 4.034204 1.227536 -0.359692
C 2.787177 1.693045 -0.213096
O 0.699746 -2.732827 -0.808013
O 3.223279 -2.321203 -1.040040
O 5.057821 -0.402821 -1.785662
C 5.263401 -0.788585 0.571340
C 4.701724 -0.544298 1.952645
C 5.671280 -0.106685 3.017127
O 3.513192 -0.736502 2.207912
O 5.093839 2.105974 -0.166869
C 5.798168 2.376677 -1.379028
C -4.595474 0.779425 0.181904
C -4.738442 2.022930 1.017332
C -5.639356 0.110623 -0.356519
C -7.109588 0.459698 -0.294048
C -7.908877 -0.596275 0.491622
C -7.524113 -0.668787 1.964059
C -7.658344 0.577382 -1.720114
H -1.828691 -2.650504 -0.606135
H -2.203578 2.149651 0.218822
H 0.236094 2.419485 0.095823
H 2.623409 2.749460 -0.020618
H 1.645504 -2.967955 -0.939009
H 5.049369 -1.361355 -1.980452
H 6.254548 -0.322819 0.500177
H 5.431421 -1.866852 0.462030
H 5.140612 0.037638 3.962262
H 6.436509 -0.874758 3.152831
H 6.132582 0.840113 2.725459
H 6.542992 1.598587 -1.568197
H 5.120862 2.483779 -2.233616
H 6.332256 3.322782 -1.249286
H -4.484104 2.912019 0.431030
H -5.748628 2.159656 1.410881
H -4.083577 1.981651 1.895369
H -5.429850 -0.798350 -0.924892
H -7.264798 1.432533 0.182537
H -8.978289 -0.358895 0.428673
H -7.780387 -1.587767 0.039117
H -7.635582 0.307307 2.446761
H -8.172188 -1.380053 2.486180
H -6.490756 -1.003834 2.094628
H -7.111072 1.337058 -2.289565
H -8.713877 0.870065 -1.705390
H -7.581202 -0.372002 -2.262293
SCF Energy (B3LYP/6-31G**)= -1361.70586943
Number of imaginary frequencies = 0

1a_c007
MMFF Geometry
C -1.812026 -1.775608 -0.427284
C -2.093897 0.921257 -0.549809
N -3.067231 -1.264704 -0.439080
C -0.640412 -1.016031 -0.474954
C -0.798493 0.378353 -0.537886
C -3.204473 0.079828 -0.499882
C 0.343713 1.198060 -0.588598
C 1.637814 0.653722 -0.574918
C 1.789369 -0.734229 -0.506397
C 0.657931 -1.566655 -0.463609
C 3.153797 -1.309240 -0.496508
C 4.372271 -0.372939 -0.438371
C 4.054651 1.103472 -0.575549
C 2.790199 1.557219 -0.639749
O 0.764820 -2.937435 -0.414274
O 3.292282 -2.535754 -0.575006
O 5.155270 -0.747863 -1.597024
C 5.224251 -0.692653 0.796696
C 4.597383 -0.209381 2.084067
C 5.540021 0.196482 3.185429
O 3.378324 -0.197248 2.251694
O 5.186433 1.901890 -0.598180
C 5.031793 3.291993 -0.856340
C -4.600845 0.604678 -0.524621
C -4.814694 1.941446 -1.181565
C -5.596383 -0.137077 0.009489
C -7.065332 0.206144 0.116232
C -7.568566 0.082246 1.567867
C -6.911240 1.077308 2.516527
C -7.867527 -0.722269 -0.798786
H -1.771592 -2.861497 -0.376229
H -2.223215 1.997864 -0.584040
H 0.216578 2.278936 -0.637354
H 2.566942 2.614606 -0.724267
H 1.718065 -3.177055 -0.448460
H 5.196414 -1.723528 -1.597453
H 6.214289 -0.230749 0.691308
H 5.402987 -1.770360 0.894644
H 4.968280 0.524233 4.058046
H 6.161762 -0.656616 3.467552
H 6.165952 1.025115 2.845013
H 4.548507 3.460770 -1.824101
H 4.476173 3.778856 -0.048604
H 6.029289 3.739623 -0.894931
H -4.226305 2.023924 -2.102694
H -5.852510 2.108017 -1.480954
H -4.525075 2.753017 -0.505994
H -5.341250 -1.107497 0.441132
H -7.254461 1.234568 -0.206886
H -8.652249 0.253034 1.588960
H -7.403885 -0.935002 1.945166
H -7.054387 2.104835 2.167286
H -7.355203 0.994039 3.513742
H -5.837339 0.891657 2.614268
H -7.539632 -0.626118 -1.839971
H -8.934008 -0.474816 -0.762780
H -7.756382 -1.772404 -0.505401
SCF Energy (B3LYP/6-31G**)= -1361.71165558
Number of imaginary frequencies = 0

1a_c008
MMFF Geometry
C 1.824552 1.751475 -0.380876
C 2.122885 -0.940996 -0.553464
N 3.082866 1.249710 -0.421319
C 0.657421 0.984794 -0.423381
C 0.824265 -0.407510 -0.512647
C 3.228344 -0.092713 -0.506635
C -0.312328 -1.234712 -0.559968
C -1.608274 -0.698631 -0.518641
C -1.770502 0.687559 -0.422459
C -0.644337 1.526663 -0.382253
C -3.140861 1.253956 -0.384867
C -4.358083 0.310843 -0.336441
C -4.013371 -1.150423 -0.521632
C -2.754596 -1.604089 -0.575800
O -0.759401 2.895577 -0.307775
O -3.289509 2.480590 -0.442574

O -5.158690 0.730370 -1.465099
C -5.185156 0.573296 0.926846
C -4.515887 0.041180 2.172653
C -5.395451 -0.651076 3.178530
O -3.313315 0.204353 2.377335
O -5.046851 -2.077250 -0.453929
C -5.841557 -2.087563 -1.640099
C 4.627805 -0.607162 -0.561716
C 4.840027 -1.933132 -1.240772
C 5.626914 0.133981 -0.033417
C 7.099703 -0.200372 0.044678
C 7.625634 -0.093382 1.489599
C 6.990621 -1.106305 2.434549
C 7.880559 0.746471 -0.869917
H 1.777720 2.835997 -0.310248
H 2.258631 -2.016037 -0.607589
H -0.180813 -2.314102 -0.628266
H -2.566903 -2.672140 -0.638433
H -1.714527 3.129266 -0.312882
H -5.174272 1.708060 -1.438914
H -6.174505 0.109284 0.825617
H -5.371102 1.643872 1.075617
H -4.791777 -0.988871 4.025400
H -6.156179 0.045239 3.539592
H -5.867634 -1.520819 2.714971
H -6.605644 -1.306586 -1.592988
H -5.232322 -1.982622 -2.544728
H -6.355603 -3.052281 -1.687777
H 4.238127 -2.006434 -2.153911
H 5.874138 -2.088738 -1.558305
H 4.565984 -2.756036 -0.572447
H 5.372272 1.096315 0.416217
H 7.290595 -1.222774 -0.296012
H 8.710654 -0.256816 1.490738
H 7.460190 0.917268 1.883911
H 7.135060 -2.127779 2.068490
H 7.450173 -1.033962 3.425534
H 5.917213 -0.929611 2.552270
H 7.536441 0.662679 -1.906927
H 8.949149 0.506064 -0.854670
H 7.767038 1.791560 -0.559898
SCF Energy (B3LYP/6-31G**)= -1361.70595731
Number of imaginary frequencies = 0

1a_c009
MMFF Geometry
C -1.461258 -2.541711 0.039652
C -2.238647 0.019330 -0.425117
N -2.789988 -2.274853 0.023512
C -0.452189 -1.595401 -0.167405
C -0.866937 -0.274278 -0.409996
C -3.164712 -0.996786 -0.198176
C 0.100597 0.721261 -0.635788
C 1.472905 0.425972 -0.617248
C 1.881190 -0.887440 -0.366784
C 0.925656 -1.895251 -0.150485
C 3.328464 -1.199940 -0.351089
C 4.353019 -0.061190 -0.486247
C 3.763343 1.298004 -0.809148
C 2.435384 1.502677 -0.867913
O 1.286870 -3.203023 0.078169
O 3.690154 -2.379785 -0.268326
O 5.161850 -0.453335 -1.621230
C 5.281186 -0.039345 0.734983
C 4.609278 0.506406 1.973717
C 5.487546 1.236362 2.954570
O 3.414766 0.320133 2.203535
O 4.726213 2.274980 -1.002303
C 4.309317 3.561373 -1.443202
C -4.626142 -0.747893 -0.209283
C -5.429579 -1.804048 -0.919403
C -5.142542 0.338211 0.404769
C -6.604110 0.716255 0.529090
C -6.874335 1.978586 -0.309393
C -8.343776 2.374953 -0.352013
C -6.948028 0.914688 2.008589
H -1.216677 -3.584317 0.231358
H -2.581339 1.029552 -0.625979
H -0.226889 1.742791 -0.825734
H 2.017437 2.478836 -1.085921
H 2.266851 -3.268979 0.027329
H 5.382845 -1.395350 -1.489372
H 6.165697 0.572105 0.514952
H 5.658632 -1.041493 0.972091
H 4.887849 1.579527 3.802137
H 6.266550 0.562725 3.319667
H 5.936354 2.105814 2.467812
H 3.777473 3.496503 -2.397952
H 3.694706 4.053592 -0.682837
H 5.204970 4.170525 -1.596832
H -5.555786 -2.685418 -0.281874
H -6.421492 -1.453292 -1.215580
H -4.931886 -2.114838 -1.845568
H -4.465321 1.027388 0.910183
H -7.244146 -0.090751 0.158694
H -6.288879 2.824065 0.073962
H -6.538529 1.807008 -1.340464
H -8.703962 2.689500 0.631707
H -8.485231 3.215399 -1.039269
H -8.965121 1.544318 -0.701188
H -6.671563 0.031990 2.596693
H -8.022345 1.071619 2.147245
H -6.422887 1.778636 2.431321
SCF Energy (B3LYP/6-31G**)= -1361.70941424
Number of imaginary frequencies = 0

1a_c010
MMFF Geometry
C -1.505105 -2.391101 -0.213921
C -2.197191 0.179582 0.326538
N -2.804886 -2.129584 0.067418
C -0.485508 -1.434147 -0.249051
C -0.855654 -0.107760 0.033710
C -3.138947 -0.847389 0.327036
C 0.125337 0.899990 0.016558
C 1.467557 0.610131 -0.275367
C 1.832112 -0.711037 -0.551204
C 0.861610 -1.727693 -0.545251
C 3.246906 -1.016356 -0.863675
C 4.304831 0.094777 -0.759159
C 3.753453 1.484570 -0.506410
C 2.444369 1.702892 -0.288601
O 1.177569 -3.036491 -0.828513
O 3.549153 -2.154253 -1.242536
O 4.920272 0.114239 -2.069477
C 5.397044 -0.309515 0.239344
C 4.940031 -0.220942 1.677057
C 5.987930 0.126158 2.700611
O 3.782306 -0.472426 2.009953
O 4.733140 2.463903 -0.500189
C 4.331810 3.825234 -0.406871
C -4.568786 -0.605153 0.635336
C -5.193584 -1.642917 1.528642
C -5.213597 0.458003 0.108733
C -6.672557 0.828309 0.279018
C -7.336776 0.932338 -1.107683
C -8.838930 1.173250 -1.043745
C -6.767000 2.135630 1.068792
H -1.293728 -3.438431 -0.418905
H -2.501576 1.193666 0.565973
H -0.167359 1.925947 0.236994
H 2.055878 2.696199 -0.094920
H 2.134632 -3.085011 -1.049821
H 5.104095 -0.816453 -2.301082
H 6.274583 0.336390 0.107540
H 5.746879 -1.333637 0.061096
H 5.535200 0.158578 3.695473
H 6.773402 -0.633374 2.691907
H 6.409033 1.109026 2.474884
H 3.667013 4.096677 -1.233293
H 3.860379 4.027049 0.560191
H 5.229802 4.445978 -0.478631

H -4.511264 -1.918387 2.341444
H -6.108517 -1.291271 2.011854
H -5.435210 -2.546288 0.958747
H -4.663825 1.131609 -0.549325
H -7.204410 0.056203 0.843406
H -6.873280 1.735946 -1.694141
H -7.164654 0.000403 -1.661930
H -9.070990 2.154259 -0.619352
H -9.266731 1.140025 -2.051004
H -9.334960 0.406605 -0.440308
H -6.227690 2.058498 2.019926
H -7.806854 2.380491 1.306667
H -6.341296 2.975496 0.508171
SCF Energy (B3LYP/6-31G**)= -1361.70947016
Number of imaginary frequencies = 0

1a_c011
MMFF Geometry
C 1.488706 2.504284 0.094096
C 2.269608 -0.046114 -0.421304
N 2.818152 2.243671 0.052840
C 0.480375 1.556933 -0.111573
C 0.897182 0.241197 -0.380687
C 3.194776 0.970609 -0.193560
C -0.068836 -0.755412 -0.606560
C -1.440663 -0.464894 -0.563452
C -1.852958 0.842868 -0.285458
C -0.898455 1.851004 -0.068314
C -3.303576 1.150029 -0.245762
C -4.331547 0.012229 -0.394476
C -3.723697 -1.323432 -0.761621
C -2.402713 -1.537919 -0.808946
O -1.261182 3.153599 0.185689
O -3.669378 2.327109 -0.142052
O -5.162353 0.445973 -1.495590
C -5.227954 -0.061155 0.846417
C -4.509475 -0.645634 2.040238
C -5.276245 -1.624713 2.887642
O -3.363691 -0.304408 2.332592
O -4.574745 -2.415814 -0.880127
C -5.317317 -2.391801 -2.099429
C 4.656936 0.728418 -0.230844
C 5.445078 1.799156 -0.936203
C 5.187188 -0.364993 0.357921
C 6.651985 -0.738691 0.453963
C 6.914494 -1.986371 -0.408557
C 8.384709 -2.375691 -0.479826
C 7.019260 -0.959118 1.924709
H 1.242924 3.542722 0.305789
H 2.613117 -1.051875 -0.642369
H 0.257835 -1.773440 -0.816236
H -2.023619 -2.536000 -1.009042
H -2.242302 3.214783 0.161212
H -5.354580 1.391530 -1.335065
H -6.114499 -0.670110 0.628279
H -5.607551 0.927215 1.132554
H -4.645980 -1.972418 3.710796
H -6.162088 -1.135868 3.300244
H -5.567012 -2.485507 2.280369
H -6.210916 -1.771193 -1.988541
H -4.709305 -2.051825 -2.945168
H -5.647033 -3.413975 -2.308346
H 5.577143 2.670976 -0.286838
H 6.433900 1.457563 -1.252721
H 4.932165 2.122197 -1.849787
H 4.520607 -1.065159 0.862446
H 7.283014 0.076839 0.086795
H 6.338394 -2.840342 -0.029879
H 6.562291 -1.799881 -1.431536
H 8.761146 -2.704274 0.493162
H 8.519061 -3.204498 -1.182471
H 8.997276 -1.536951 -0.825115
H 6.748231 -0.087069 2.530951
H 8.096197 -1.113613 2.044493
H 6.504145 -1.831921 2.341546
SCF Energy (B3LYP/6-31G**)= -1361.70371578
Number of imaginary frequencies = 0

1a_c012
MMFF Geometry
C -1.532209 -2.336314 -0.403414
C -2.231500 0.174194 0.363777
N -2.836464 -2.099099 -0.121170
C -0.511332 -1.382812 -0.331849
C -0.885570 -0.087522 0.067560
C -3.173875 -0.846084 0.252135
C 0.095579 0.915483 0.161196
C 1.440799 0.650167 -0.135753
C 1.811617 -0.640976 -0.526834
C 0.840568 -1.651242 -0.631523
C 3.233184 -0.918807 -0.846443
C 4.294274 0.177685 -0.633752
C 3.719316 1.531262 -0.279408
C 2.419432 1.730720 -0.026907
O 1.159948 -2.928597 -1.030512
O 3.541098 -2.016073 -1.327349
O 4.935727 0.285724 -1.925113
C 5.358669 -0.297269 0.361343
C 4.849560 -0.310117 1.783887
C 5.780839 0.204427 2.848108
O 3.739676 -0.760841 2.066282
O 4.607111 2.571759 -0.032791
C 5.169483 3.098046 -1.235054
C -4.608577 -0.630564 0.557812
C -5.249347 -1.746572 1.338361
C -5.243407 0.479326 0.123610
C -6.704618 0.835685 0.303941
C -7.345198 1.072892 -1.077487
C -8.847956 1.310665 -1.016047
C -6.810987 2.062225 1.212805
H -1.318258 -3.360505 -0.701463
H -2.538826 1.162136 0.692353
H -0.197811 1.917551 0.472391
H 2.074503 2.710007 0.292379
H 2.122282 -2.961833 -1.229886
H 5.093431 -0.630944 -2.227855
H 6.239648 0.354416 0.301770
H 5.716832 -1.306041 0.122716
H 5.293346 0.144170 3.825060
H 6.688127 -0.404223 2.865486
H 6.029312 1.248807 2.643607
H 6.032461 2.501715 -1.544293
H 4.430691 3.161789 -2.041688
H 5.525274 4.110287 -1.020631
H -4.581202 -2.099269 2.132891
H -6.172095 -1.439957 1.837134
H -5.481980 -2.591459 0.681633
H -4.681869 1.211025 -0.458063
H -7.246770 0.014828 0.783333
H -6.870988 1.927380 -1.577011
H -7.164744 0.197427 -1.714916
H -9.086092 2.247493 -0.504200
H -9.258710 1.374522 -2.028909
H -9.354942 0.491447 -0.496649
H -6.287914 1.893549 2.161174
H -7.854448 2.286143 1.455472
H -6.374962 2.950454 0.741818
SCF Energy (B3LYP/6-31G**)= -1361.70379954
Number of imaginary frequencies = 0

1a_c013
MMFF Geometry
C -1.538646 -2.577095 0.049101
C -2.319089 -0.017710 -0.419541
N -2.867699 -2.311800 0.033092
C -0.530730 -1.629962 -0.159905
C -0.947073 -0.309715 -0.404499
C -3.243908 -1.034526 -0.190426
C 0.019183 0.686584 -0.632342
C 1.391833 0.392914 -0.613880
C 1.801741 -0.919614 -0.361421
C 0.847467 -1.928182 -0.143067

C 3.249384 -1.230410 -0.345814
C 4.272561 -0.090697 -0.483271
C 3.681177 1.267281 -0.808160
C 2.352958 1.470320 -0.866711
O 1.210292 -3.235157 0.087583
O 3.612476 -2.409697 -0.261263
O 5.081376 -0.483770 -1.617945
C 5.201208 -0.065769 0.737535
C 4.529178 0.481235 1.975650
C 5.407000 1.213826 2.954936
O 3.334979 0.293946 2.206271
O 4.642835 2.245054 -1.003317
C 4.224266 3.530240 -1.446143
C -4.705394 -0.787205 -0.201502
C -5.509573 -1.845465 -0.907742
C -5.223668 0.302442 0.404660
C -6.686473 0.672430 0.523478
C -7.075259 1.788875 -0.464973
C -6.367052 3.123001 -0.262454
C -7.019551 1.039298 1.973086
H -1.292782 -3.619109 0.242381
H -2.662942 0.991805 -0.622094
H -0.309577 1.707418 -0.823829
H 1.933790 2.445635 -1.086148
H 2.190329 -3.300057 0.036448
H 5.303521 -1.425309 -1.484628
H 6.084917 0.546346 0.516133
H 5.579917 -1.067087 0.976131
H 4.807255 1.557684 3.802187
H 6.186941 0.541699 3.320816
H 5.854592 2.082999 2.466563
H 3.692102 3.463195 -2.400564
H 3.609399 4.022989 -0.686327
H 5.119148 4.140178 -1.601140
H -5.631447 -2.726893 -0.269482
H -6.503717 -1.495583 -1.198602
H -5.015529 -2.155175 -1.836167
H -4.546686 0.994196 0.905977
H -7.309935 -0.196492 0.284114
H -6.880785 1.444852 -1.489041
H -8.157366 1.958871 -0.399238
H -5.282190 3.023974 -0.362220
H -6.704251 3.840068 -1.018319
H -6.589294 3.551081 0.719171
H -6.847265 0.184197 2.636773
H -8.072233 1.326397 2.068013
H -6.406931 1.869125 2.341236
SCF Energy (B3LYP/6-31G**)= -1361.70841454
Number of imaginary frequencies = 0

1a_c014
MMFF Geometry
C 1.565660 2.540263 0.098855
C 2.349650 -0.008790 -0.418409
N 2.895443 2.281451 0.056891
C 0.558481 1.591690 -0.106932
C 0.976897 0.276670 -0.377025
C 3.273581 1.009035 -0.190324
C 0.012148 -0.721106 -0.603106
C -1.360041 -0.432450 -0.559231
C -1.773965 0.874594 -0.280262
C -0.820721 1.883882 -0.062910
C -3.224976 1.179788 -0.239762
C -4.251486 0.040701 -0.388728
C -3.642007 -1.293922 -0.756944
C -2.320757 -1.506612 -0.804965
O -1.185085 3.185836 0.192034
O -3.592310 2.356313 -0.135183
O -5.083344 0.474001 -1.489223
C -5.147262 -0.034637 0.852504
C -4.427486 -0.618872 2.045663
C -5.192571 -1.599502 2.892796
O -3.282039 -0.276277 2.337735
O -4.491643 -2.387370 -0.875750
C -5.234773 -2.363610 -2.094716
C 4.735808 0.768686 -0.228544
C 5.523915 1.840883 -0.931827
C 5.268560 -0.327395 0.352972
C 6.734636 -0.692524 0.442765
C 7.112202 -1.792421 -0.568329
C 6.413057 -3.132585 -0.374371
C 7.092605 -1.079343 1.881210
H 1.318582 3.578251 0.311245
H 2.694316 -1.013976 -0.640401
H 0.340104 -1.738566 -0.813538
H -1.940413 -2.504063 -1.005829
H -2.166297 3.245717 0.168016
H -5.276770 1.419201 -1.328039
H -6.033082 -0.644650 0.634377
H -5.528063 0.953049 1.139401
H -4.561485 -1.946857 3.715469
H -6.078897 -1.112106 3.306073
H -5.482434 -2.460318 2.285122
H -6.129154 -1.744267 -1.983064
H -4.627582 -2.022306 -2.940510
H -5.563210 -3.386098 -2.304113
H 5.652068 2.713437 -0.282695
H 6.514833 1.499949 -1.243569
H 5.013752 2.161840 -1.847629
H 4.602746 -1.029676 0.854679
H 7.350445 0.182653 0.206395
H 6.899779 -1.434128 -1.583903
H 8.195944 -1.958491 -0.522515
H 5.326316 -3.036986 -0.455232
H 6.741095 -3.836837 -1.146149
H 6.652912 -3.574166 0.597074
H 6.927392 -0.234965 2.560246
H 8.147896 -1.363068 1.954933
H 6.489549 -1.917292 2.246744
SCF Energy (B3LYP/6-31G**)= -1361.70270905
Number of imaginary frequencies = 0

1a_c015
MMFF Geometry
C -1.759789 -1.673546 -0.165346
C -1.993197 1.028610 -0.058059
N -3.000458 -1.144691 -0.031980
C -0.580283 -0.930033 -0.254510
C -0.713471 0.467317 -0.199993
C -3.113212 0.202314 0.023683
C 0.437053 1.271649 -0.292022
C 1.715714 0.708778 -0.431151
C 1.843283 -0.682535 -0.476519
C 0.702330 -1.499062 -0.396337
C 3.191127 -1.276732 -0.626925
C 4.429559 -0.365372 -0.606840
C 4.132502 1.121643 -0.610062
C 2.877023 1.597500 -0.531420
O 0.783977 -2.871102 -0.457491
O 3.296561 -2.495979 -0.805790
O 5.103534 -0.666080 -1.852429
C 5.376248 -0.787224 0.524200
C 4.871482 -0.389894 1.892124
C 5.912792 -0.080614 2.934397
O 3.671601 -0.371064 2.164695
O 5.274714 1.902770 -0.674064
C 5.127720 3.309944 -0.819700
C -4.491974 0.747483 0.189165
C -4.617574 2.093277 0.850890
C -5.549387 0.013609 -0.223201
C -7.017101 0.379936 -0.171461
C -7.717620 -0.474181 0.900341
C -9.176413 -0.096583 1.118304
C -7.632046 0.189562 -1.561754
H -1.738332 -2.760627 -0.199886
H -2.104690 2.107291 -0.026178
H 0.329011 2.354934 -0.251363
H 2.669612 2.661417 -0.520580
H 1.725587 -3.121549 -0.591311
H 5.123310 -1.639589 -1.926671
H 6.363358 -0.333809 0.367128
H 5.539682 -1.871779 0.529070

H 5.424429 0.191170 3.874294
H 6.537459 -0.961694 3.099985
H 6.525522 0.760877 2.601590
H 4.567017 3.554815 -1.727590
H 4.653606 3.745837 0.065397
H 6.127419 3.744346 -0.913295
H -4.393736 2.894799 0.139339
H -5.615196 2.274709 1.259308
H -3.931885 2.177120 1.701942
H -5.356085 -0.967995 -0.661624
H -7.140737 1.436571 0.086739
H -7.661016 -1.538253 0.637496
H -7.190468 -0.359305 1.856479
H -9.784877 -0.317420 0.236612
H -9.589997 -0.667613 1.955893
H -9.276222 0.967869 1.352677
H -7.065991 0.743395 -2.319596
H -8.661518 0.560037 -1.592863
H -7.643750 -0.866166 -1.855346
SCF Energy (B3LYP/6-31G**)= -1361.71037205
Number of imaginary frequencies = 0

1a_c016
MMFF Geometry
C 1.773944 1.639349 -0.191904
C 2.025248 -1.057225 -0.008407
N 3.019745 1.121488 -0.063096
C 0.597564 0.887230 -0.240468
C 0.740251 -0.507490 -0.146865
C 3.141404 -0.222689 0.030977
C -0.406054 -1.321009 -0.196895
C -1.688483 -0.768609 -0.333885
C -1.827526 0.620936 -0.417156
C -0.690554 1.445423 -0.378519
C -3.183149 1.203870 -0.567377
C -4.420665 0.287815 -0.514418
C -4.093864 -1.189075 -0.494880
C -2.845461 -1.660821 -0.384364
O -0.781021 2.814537 -0.478813
O -3.299070 2.416398 -0.782774
O -5.112557 0.578797 -1.750324
C -5.346545 0.709280 0.631851
C -4.798461 0.321776 1.985588
C -5.776715 -0.232402 2.985872
O -3.614108 0.492049 2.273734
O -5.149703 -2.088822 -0.409333
C -5.838867 -2.232215 -1.651582
C 4.525862 -0.755070 0.190814
C 4.669591 -2.080574 0.888897
C 5.572478 -0.027477 -0.258594
C 7.042968 -0.384014 -0.218839
C 7.754449 0.504229 0.817415
C 9.218603 0.141129 1.023752
C 7.635716 -0.229976 -1.623204
H 1.745565 2.724816 -0.257727
H 2.143374 -2.133909 0.053979
H -0.293157 -2.402283 -0.125176
H -2.676814 -2.730855 -0.303952
H -1.726822 3.057187 -0.594932
H -5.108312 1.552692 -1.841496
H -6.333744 0.248432 0.499821
H -5.520495 1.792129 0.636414
H -5.255511 -0.474055 3.916284
H -6.548482 0.512585 3.193954
H -6.228478 -1.145087 2.589370
H -6.586714 -1.442412 -1.765195
H -5.151522 -2.243678 -2.504788
H -6.368059 -3.189650 -1.629870
H 4.439294 -2.903459 0.204302
H 5.674511 -2.244864 1.286527
H 3.997828 -2.143487 1.752763
H 5.366650 0.940020 -0.721915
H 7.176834 -1.432147 0.067386
H 7.687505 1.560034 0.525294
H 7.242487 0.413758 1.784366
H 9.812358 0.340096 0.126955
H 9.641349 0.738124 1.838348
H 9.328320 -0.915628 1.286767
H 7.061620 -0.808348 -2.356262
H 8.666802 -0.595458 -1.659460
H 7.636662 0.816993 -1.946857
SCF Energy (B3LYP/6-31G**)= -1361.70469735
Number of imaginary frequencies = 0

1a_c017
MMFF Geometry
C -1.740749 -1.690079 -0.579047
C -2.007957 1.005538 -0.406510
N -2.993138 -1.174379 -0.530970
C -0.565057 -0.935958 -0.547779
C -0.715552 0.457803 -0.458105
C -3.123078 0.169487 -0.444673
C 0.431059 1.272179 -0.423245
C 1.722144 0.722827 -0.473273
C 1.866161 -0.665113 -0.556662
C 0.730230 -1.491326 -0.600826
C 3.227389 -1.244912 -0.614041
C 4.451217 -0.326877 -0.458557
C 4.141436 1.157336 -0.433222
C 2.879353 1.621977 -0.443363
O 0.829625 -2.859876 -0.701280
O 3.358820 -2.456311 -0.825970
O 5.228835 -0.577597 -1.653789
C 5.304894 -0.783520 0.731482
C 4.684368 -0.439920 2.065919
C 5.632359 -0.161229 3.201672
O 3.465874 -0.439729 2.238034
O 5.277576 1.947549 -0.372723
C 5.129949 3.358278 -0.477580
C -4.516651 0.701348 -0.408136
C -4.724494 2.101801 -0.918205
C -5.515668 -0.088894 0.043861
C -6.984175 0.248634 0.188227
C -7.435560 -0.048948 1.631698
C -8.867500 0.381270 1.920047
C -7.784962 -0.544619 -0.846474
H -1.706208 -2.775274 -0.646559
H -2.131428 2.080145 -0.323057
H 0.309806 2.352594 -0.353701
H 2.661749 2.683435 -0.411587
H 1.781421 -3.099334 -0.764567
H 5.264550 -1.547626 -1.760490
H 6.297185 -0.318070 0.673586
H 5.477905 -1.866405 0.710995
H 5.064920 0.072615 4.106712
H 6.250138 -1.043194 3.387189
H 6.261927 0.696242 2.951284
H 4.644878 3.633884 -1.419577
H 4.579332 3.757253 0.380224
H 6.129809 3.802230 -0.470645
H -4.136763 2.279325 -1.826263
H -5.761885 2.304065 -1.196650
H -4.430040 2.835296 -0.160471
H -5.265150 -1.102653 0.364554
H -7.155097 1.313911 0.004384
H -7.336314 -1.119947 1.850045
H -6.774291 0.476604 2.333021
H -9.587618 -0.206454 1.343555
H -9.096111 0.234838 2.980700
H -9.014966 1.440115 1.685478
H -7.400921 -0.368755 -1.857921
H -8.838176 -0.247034 -0.845382
H -7.737454 -1.621939 -0.651511
SCF Energy (B3LYP/6-31G**)= -1361.71035725
Number of imaginary frequencies = 0

1a_c018
MMFF Geometry
C -1.754420 -1.608663 -0.692164
C -2.037715 1.062318 -0.300033
N -3.009951 -1.103263 -0.621412
C -0.583003 -0.855648 -0.580057

C -0.742067 0.525614 -0.377232
C -3.147936 0.228281 -0.425193
C 0.399148 1.338980 -0.257138
C 1.692095 0.799638 -0.334393
C 1.846600 -0.577057 -0.529152
C 0.715723 -1.400636 -0.658737
C 3.213767 -1.145642 -0.616725
C 4.436634 -0.239192 -0.379277
C 4.099754 1.230335 -0.255095
C 2.843451 1.691547 -0.207703
O 0.823087 -2.755688 -0.870920
O 3.355051 -2.334217 -0.928792
O 5.230579 -0.418932 -1.574347
C 5.266859 -0.762530 0.797868
C 4.605338 -0.497658 2.130285
C 5.492649 -0.033893 3.253779
O 3.402607 -0.693894 2.302419
O 5.138861 2.117754 -0.001283
C 5.929200 2.370443 -1.163254
C -4.544639 0.750036 -0.366098
C -4.751200 2.188513 -0.757055
C -5.546918 -0.079631 -0.000103
C -7.019164 0.239087 0.148845
C -7.491998 -0.183195 1.553847
C -8.930429 0.215596 1.854787
C -7.799496 -0.464996 -0.963121
H -1.713636 -2.684403 -0.848672
H -2.167450 2.125802 -0.128996
H 0.273669 2.409632 -0.099011
H 2.661751 2.750206 -0.046113
H 1.776810 -2.987916 -0.929094
H 5.240565 -1.380782 -1.751973
H 6.258510 -0.292489 0.790477
H 5.447119 -1.841552 0.720017
H 4.894106 0.123489 4.155274
H 6.250654 -0.793772 3.458572
H 5.968153 0.910867 2.978788
H 6.688893 1.592972 -1.283163
H 5.316006 2.458800 -2.066979
H 6.448677 3.321461 -1.011911
H -4.150765 2.445350 -1.637410
H -5.785188 2.410678 -1.032945
H -4.471497 2.855084 0.065339
H -5.296601 -1.116277 0.236518
H -7.192388 1.315614 0.054536
H -7.391006 -1.268658 1.680875
H -6.844546 0.282365 2.308431
H -9.638405 -0.322852 1.218250
H -9.175166 -0.022373 2.895059
H -9.079319 1.290177 1.709773
H -7.400288 -0.201391 -1.949320
H -8.854042 -0.172318 -0.953575
H -7.749811 -1.554923 -0.860772
SCF Energy (B3LYP/6-31G**)= -1361.70475052
Number of imaginary frequencies = 0

1a_c019
MMFF Geometry
C -1.589990 -2.364495 -0.246288
C -2.251909 0.178346 0.441545
N -2.881963 -2.109586 0.074498
C -0.564092 -1.413748 -0.252033
C -0.918585 -0.101740 0.107099
C -3.201748 -0.840906 0.407100
C 0.069380 0.899201 0.122888
C 1.403334 0.616414 -0.210856
C 1.752430 -0.690792 -0.562734
C 0.774639 -1.700074 -0.590346
C 3.158411 -0.988423 -0.919280
C 4.226500 0.108862 -0.778212
C 3.690683 1.487010 -0.442001
C 2.387855 1.702044 -0.186645
O 1.075058 -2.994230 -0.947995
O 3.444531 -2.106815 -1.363161
O 4.815909 0.193058 -2.097904
C 5.335276 -0.354274 0.175472
C 4.907581 -0.338310 1.624799
C 5.978169 -0.052135 2.643709
O 3.754845 -0.599459 1.967424
O 4.677616 2.458330 -0.404360
C 4.288443 3.815447 -0.231811
C -4.623605 -0.602240 0.757880
C -5.239218 -1.685025 1.603549
C -5.265287 0.490541 0.290799
C -6.703649 0.894438 0.532139
C -7.596015 0.594997 -0.688531
C -7.248267 1.354787 -1.962907
C -6.768692 2.366344 0.951549
H -1.390666 -3.401036 -0.509836
H -2.543787 1.180497 0.739841
H -0.211215 1.914041 0.402562
H 2.010744 2.686306 0.066557
H 2.027156 -3.037274 -1.190809
H 4.988157 -0.725366 -2.381531
H 6.214833 0.291998 0.059920
H 5.673827 -1.369863 -0.063156
H 5.545540 -0.069040 3.647857
H 6.757606 -0.815201 2.579393
H 6.402051 0.938492 2.461247
H 3.609418 4.134173 -1.029275
H 3.837839 3.969264 0.753827
H 5.189464 4.433297 -0.289129
H -4.623867 -1.873176 2.490823
H -6.239731 -1.438394 1.964111
H -5.324613 -2.618593 1.036695
H -4.720431 1.171361 -0.363753
H -7.118206 0.325668 1.370573
H -7.559046 -0.480613 -0.904401
H -8.638078 0.819252 -0.427545
H -6.231981 1.133195 -2.301323
H -7.934107 1.064820 -2.765903
H -7.342113 2.435693 -1.824859
H -6.228039 2.523727 1.891928
H -7.806495 2.679029 1.108947
H -6.324065 3.030139 0.202419
SCF Energy (B3LYP/6-31G**)= -1361.70906848
Number of imaginary frequencies = 0

1a_c020
MMFF Geometry
C -1.616883 -2.299154 -0.480594
C -2.287141 0.151566 0.480014
N -2.914084 -2.077540 -0.156136
C -0.589476 -1.357702 -0.360488
C -0.948655 -0.093366 0.139055
C -3.237760 -0.853755 0.313171
C 0.039661 0.896165 0.285710
C 1.377352 0.647570 -0.056292
C 1.733315 -0.613298 -0.547214
C 0.754815 -1.609281 -0.705580
C 3.146820 -0.873060 -0.914244
C 4.217920 0.200720 -0.643716
C 3.657492 1.527364 -0.180843
C 2.363901 1.713327 0.111178
O 1.059236 -2.855619 -1.202358
O 3.439206 -1.933790 -1.479430
O 4.834672 0.399568 -1.936318
C 5.298851 -0.349592 0.293021
C 4.817482 -0.463593 1.720703
C 5.772148 -0.031507 2.800757
O 3.710827 -0.929061 1.991623
O 4.555684 2.543544 0.122906
C 5.097463 3.153384 -1.048950
C -4.665315 -0.651563 0.663865
C -5.297757 -1.821779 1.368960
C -5.296673 0.490005 0.314139
C -6.738334 0.869133 0.575116
C -7.609146 0.716366 -0.687571
C -7.237057 1.616969 -1.859197
C -6.808002 2.282965 1.160657
H -1.414552 -3.299963 -0.856128
H -2.582437 1.114381 0.885429

H -0.242078 1.874192 0.674485
H 2.030696 2.668265 0.507399
H 2.017310 -2.878292 -1.422636
H 4.981393 -0.493349 -2.307770
H 6.182097 0.301072 0.263398
H 5.646707 -1.339841 -0.025174
H 5.303448 -0.160528 3.780241
H 6.676215 -0.643539 2.755966
H 6.022375 1.023934 2.667633
H 5.950929 2.577512 -1.417648
H 4.343473 3.278563 -1.834039
H 5.462982 4.145911 -0.768804
H -4.698385 -2.112985 2.239172
H -6.303904 -1.614169 1.738312
H -5.375099 -2.683763 0.697269
H -4.738926 1.239540 -0.247958
H -7.169104 0.208961 1.334673
H -7.570371 -0.327223 -1.025688
H -8.655305 0.913116 -0.420848
H -6.215283 1.431906 -2.202992
H -7.908906 1.424517 -2.702311
H -7.331270 2.675040 -1.598752
H -6.284101 2.328429 2.122348
H -7.847850 2.579589 1.334751
H -6.348598 3.027063 0.501278
SCF Energy (B3LYP/6-31G**)= -1361.70329469
Number of imaginary frequencies = 0

1a_c021
MMFF Geometry
C -1.859330 -1.540748 -0.253436
C -2.016177 1.156485 0.007143
N -3.084251 -0.985551 -0.086119
C -0.659487 -0.826859 -0.304543
C -0.753062 0.568340 -0.170688
C -3.159016 0.358822 0.045861
C 0.419581 1.343914 -0.221031
C 1.681570 0.754099 -0.396141
C 1.769685 -0.635448 -0.520528
C 0.606260 -1.422798 -0.482709
C 3.099916 -1.257838 -0.708911
C 4.363646 -0.384252 -0.641611
C 4.108729 1.108323 -0.559725
C 2.867346 1.614072 -0.449987
O 0.648976 -2.790930 -0.621584
O 3.170519 -2.467489 -0.956765
O 5.026536 -0.632785 -1.904531
C 5.300135 -0.895908 0.460491
C 4.809337 -0.562648 1.850431
C 5.860902 -0.342367 2.904945
O 3.610973 -0.525498 2.127756
O 5.272441 1.859400 -0.583385
C 5.165010 3.276139 -0.648700
C -4.521647 0.931887 0.246855
C -4.608862 2.246888 0.973729
C -5.601832 0.246916 -0.190098
C -7.055683 0.655265 -0.106822
C -7.787768 -0.061669 1.044047
C -7.867486 -1.579143 0.928693
C -7.741929 0.432218 -1.458386
H -1.868719 -2.624307 -0.349378
H -2.096739 2.234322 0.100569
H 0.342286 2.425769 -0.118823
H 2.690109 2.681078 -0.378294
H 1.582881 -3.059781 -0.772584
H 5.018661 -1.600701 -2.033760
H 6.299368 -0.462244 0.325906
H 5.432871 -1.983162 0.403457
H 5.382160 -0.110615 3.860386
H 6.460669 -1.248660 3.018271
H 6.496605 0.499030 2.618174
H 4.609781 3.587697 -1.539354
H 4.705037 3.674367 0.261258
H 6.176413 3.686866 -0.721015
H -4.357978 3.075285 0.303158
H -5.602924 2.438405 1.386816
H -3.924673 2.267449 1.829807
H -5.436996 -0.716339 -0.677177
H -7.127132 1.731259 0.089107
H -7.296406 0.188713 1.993103
H -8.809113 0.333395 1.114439
H -6.873508 -2.034943 0.898664
H -8.393261 -1.989745 1.797133
H -8.418840 -1.885891 0.035298
H -7.275066 1.049304 -2.234676
H -8.800256 0.709316 -1.405949
H -7.680045 -0.610716 -1.786900
SCF Energy (B3LYP/6-31G**)= -1361.70953588
Number of imaginary frequencies = 0

1a_c022
MMFF Geometry
C 1.871533 1.501876 -0.302026
C 2.048776 -1.179737 0.079821
N 3.102394 0.961235 -0.130518
C 0.675176 0.780405 -0.300066
C 0.779539 -0.607006 -0.103796
C 3.187309 -0.375244 0.062323
C -0.388481 -1.390618 -0.098585
C -1.655059 -0.815224 -0.280918
C -1.755911 0.567700 -0.466516
C -0.596915 1.361669 -0.483905
C -3.094789 1.174550 -0.664583
C -4.356981 0.298739 -0.549541
C -4.070700 -1.181035 -0.420321
C -2.835882 -1.677047 -0.270435
O -0.649721 2.721663 -0.684760
O -3.177092 2.370671 -0.968850
O -5.038125 0.516754 -1.806267
C -5.273349 0.828239 0.558935
C -4.738834 0.526571 1.939678
C -5.733930 0.074213 2.973999
O -3.550852 0.685340 2.219326
O -5.150903 -2.043117 -0.273224
C -5.841215 -2.258661 -1.504361
C 4.556507 -0.931352 0.266587
C 4.663329 -2.211648 1.050636
C 5.625390 -0.261192 -0.218501
C 7.082797 -0.657745 -0.140644
C 7.828950 0.114129 0.964757
C 7.897708 1.625269 0.780038
C 7.745937 -0.492190 -1.511866
H 1.873011 2.579947 -0.447387
H 2.136972 -2.251704 0.221783
H -0.305334 -2.466361 0.052763
H -2.696704 -2.742454 -0.111010
H -1.588297 2.980678 -0.822192
H -5.007087 1.480843 -1.968661
H -6.272496 0.385849 0.457095
H -5.417662 1.912809 0.483286
H -5.221433 -0.112445 3.921745
H -6.485427 0.853170 3.123686
H -6.209710 -0.852545 2.643774
H -6.566965 -1.459358 -1.678642
H -5.152716 -2.351411 -2.351652
H -6.396421 -3.197213 -1.414570
H 4.406136 -3.070823 0.422527
H 5.665187 -2.379312 1.455164
H 3.993590 -2.196800 1.918182
H 5.446952 0.678157 -0.745771
H 7.163819 -1.723435 0.102240
H 7.354391 -0.095843 1.931990
H 8.853642 -0.271981 1.036174
H 6.900662 2.074012 0.745749
H 8.434872 2.077327 1.620466
H 8.432816 1.894372 -0.135104
H 7.270380 -1.146049 -2.251932
H 8.806606 -0.761089 -1.464275
H 7.672564 0.534553 -1.885869
SCF Energy (B3LYP/6-31G**)= -1361.70383846
Number of imaginary frequencies = 0

1a_c023
MMFF Geometry
C -1.562528 -2.482508 -0.211414
C -2.325747 0.122688 -0.306028
N -2.889022 -2.207755 -0.166858
C -0.549087 -1.522814 -0.305731
C -0.956649 -0.178981 -0.357328
C -3.256405 -0.909236 -0.203430
C 0.015688 0.831323 -0.462260
C 1.386049 0.527854 -0.512159
C 1.789775 -0.809063 -0.452778
C 0.826733 -1.829919 -0.354418
C 3.236282 -1.134672 -0.502307
C 4.268297 0.012075 -0.588230
C 3.674368 1.409410 -0.665988
C 2.348304 1.625645 -0.630552
O 1.177707 -3.159771 -0.309061
O 3.564642 -2.325670 -0.495690
O 5.003733 -0.204886 -1.812961
C 5.289779 -0.150373 0.546184
C 4.711754 0.065782 1.927386
C 5.703272 0.143538 3.058865
O 3.502481 0.145667 2.139400
O 4.635374 2.399035 -0.781459
C 4.216706 3.757837 -0.832892
C -4.715335 -0.651162 -0.153868
C -5.538887 -1.590255 -0.993390
C -5.209663 0.340540 0.617825
C -6.662113 0.715944 0.817946
C -6.953798 2.135381 0.296961
C -6.776961 2.269883 -1.210445
C -7.005049 0.623638 2.308475
H -1.323517 -3.543065 -0.168425
H -2.663547 1.152953 -0.359317
H -0.306908 1.871273 -0.501488
H 1.931122 2.625192 -0.681266
H 2.159718 -3.223603 -0.349259
H 4.444945 0.104378 -2.545585
H 6.120650 0.552969 0.406984
H 5.732128 -1.154487 0.524285
H 5.174432 0.303632 4.002541
H 6.262445 -0.793236 3.120444
H 6.385360 0.980769 2.891690
H 3.599001 3.943214 -1.717402
H 3.687361 4.038894 0.083200
H 5.111516 4.382473 -0.910093
H -5.648001 -2.558510 -0.493547
H -6.538872 -1.204518 -1.206027
H -5.066157 -1.755251 -1.968757
H -4.516449 0.941213 1.207497
H -7.325626 0.018095 0.297897
H -7.987857 2.404991 0.545887
H -6.308849 2.867345 0.799542
H -7.402140 1.547203 -1.744399
H -7.069258 3.274063 -1.533990
H -5.735937 2.114840 -1.509525
H -6.818747 -0.385852 2.691983
H -8.062961 0.852730 2.476834
H -6.410922 1.326256 2.903919
SCF Energy (B3LYP/6-31G**)= -1361.70825032
Number of imaginary frequencies = 0

1a_c024
MMFF Geometry
C -1.626807 -2.271558 -0.161984
C -2.254505 0.310726 0.400556
N -2.918281 -1.981164 0.129144
C -0.585166 -1.338541 -0.197648
C -0.922174 -0.006357 0.096221
C -3.220287 -0.693579 0.400368
C 0.082597 0.977179 0.079254
C 1.415918 0.658248 -0.225815
C 1.749727 -0.668159 -0.514036
C 0.753428 -1.661389 -0.503421
C 3.157128 -1.010582 -0.834717
C 4.230430 0.100779 -0.814950
C 3.709455 1.494722 -0.503703
C 2.413468 1.730561 -0.236774
O 1.034235 -2.977179 -0.792538
O 3.417502 -2.181284 -1.131370
O 4.784649 0.143198 -2.148695
C 5.382188 -0.334704 0.101867
C 5.004224 -0.416141 1.564416
C 6.139305 -0.613626 2.534969
O 3.839570 -0.357377 1.956604
O 4.700171 2.461020 -0.535043
C 4.354195 3.807006 -0.230251
C -4.641839 -0.419872 0.719654
C -5.286855 -1.450665 1.606561
C -5.264640 0.661497 0.203924
C -6.712796 1.062945 0.383573
C -7.429870 1.208205 -0.972759
C -7.531052 -0.104388 -1.740191
C -6.775468 2.383618 1.155146
H -1.441418 -3.322301 -0.374861
H -2.533306 1.329825 0.649660
H -0.184602 2.007646 0.311304
H 2.049084 2.726378 -0.010318
H 1.997126 -3.054555 -0.984594
H 4.151589 0.620455 -2.710905
H 6.224984 0.361160 0.002471
H 5.761681 -1.319569 -0.198826
H 5.749149 -0.654082 3.555737
H 6.650792 -1.553494 2.313518
H 6.837116 0.223826 2.458270
H 3.636933 4.200414 -0.957608
H 3.966056 3.889591 0.790019
H 5.264299 4.410669 -0.294390
H -4.608194 -1.748077 2.414652
H -6.192325 -1.083092 2.095545
H -5.550576 -2.343521 1.029881
H -4.704194 1.325850 -0.454297
H -7.260560 0.315711 0.966008
H -8.446181 1.585826 -0.804003
H -6.919113 1.950827 -1.598772
H -8.033109 -0.870802 -1.141173
H -8.110463 0.041364 -2.657531
H -6.544334 -0.480639 -2.026702
H -6.291301 2.289510 2.133701
H -7.815127 2.682866 1.326836
H -6.278683 3.193762 0.609167
SCF Energy (B3LYP/6-31G**)= -1361.70834991
Number of imaginary frequencies = 0

1a_c025
MMFF Geometry
C -1.838662 -1.464522 -0.871207
C -2.040312 1.172209 -0.259268
N -3.078155 -0.932163 -0.741016
C -0.644946 -0.755705 -0.714915
C -0.761529 0.607846 -0.398140
C -3.175607 0.382196 -0.435304
C 0.404629 1.376276 -0.228598
C 1.682029 0.810123 -0.366143
C 1.792220 -0.548578 -0.675742
C 0.636511 -1.327421 -0.856422
C 3.139023 -1.145331 -0.825420
C 4.384244 -0.296393 -0.519728
C 4.111043 1.170953 -0.251865
C 2.860886 1.662703 -0.187541
O 0.702651 -2.663076 -1.179759
O 3.241697 -2.308555 -1.232904
O 5.162114 -0.367441 -1.738744
C 5.219718 -0.963273 0.580605
C 4.600390 -0.827413 1.952397
C 5.548590 -0.762577 3.119874
O 3.381326 -0.824688 2.120409
O 5.265981 1.911622 -0.060972
C 5.153800 3.323739 0.066649
C -4.556820 0.935641 -0.314551
C -4.723644 2.405194 -0.592667
C -5.573114 0.106017 0.011672

C -7.036976 0.436667 0.200370
C -7.490768 0.222312 1.659421
C -7.445232 -1.213840 2.167003
C -7.882764 -0.361248 -0.794986
H -1.830574 -2.524473 -1.115821
H -2.137325 2.221433 -0.001134
H 0.309699 2.433404 0.016973
H 2.669368 2.709762 0.017505
H 1.648586 -2.912895 -1.280072
H 5.174490 -1.307466 -2.002974
H 6.223499 -0.519840 0.601271
H 5.366071 -2.032184 0.383117
H 4.982030 -0.665940 4.050164
H 6.143401 -1.678319 3.159214
H 6.200487 0.108173 3.014373
H 4.680946 3.762131 -0.818130
H 4.608415 3.590548 0.977447
H 6.164257 3.735155 0.147734
H -4.137802 2.705822 -1.469032
H -5.754663 2.686089 -0.818820
H -4.397393 2.998830 0.267702
H -5.336965 -0.948060 0.170761
H -7.217831 1.494200 -0.014949
H -6.875463 0.845126 2.321578
H -8.519988 0.588966 1.763726
H -6.430408 -1.620681 2.142484
H -7.788991 -1.249910 3.206187
H -8.098167 -1.868149 1.582411
H -7.630348 -0.083728 -1.824855
H -8.949024 -0.158333 -0.648573
H -7.725632 -1.440882 -0.698367
SCF Energy (B3LYP/6-31G**)= -1361.70934925
Number of imaginary frequencies = 0

1a_c026
MMFF Geometry
C -1.850028 -1.267437 -1.087618
C -2.069848 1.219459 -0.022203
N -3.093108 -0.766319 -0.886646
C -0.661114 -0.597584 -0.788507
C -0.787333 0.688793 -0.237542
C -3.199680 0.473104 -0.354457
C 0.372877 1.415724 0.084724
C 1.652608 0.882613 -0.131287
C 1.774318 -0.401344 -0.673246
C 0.624248 -1.135950 -1.007365
C 3.127568 -0.961901 -0.906894
C 4.371169 -0.177173 -0.447446
C 4.069700 1.219092 0.050533
C 2.824826 1.683491 0.217525
O 0.699413 -2.393358 -1.560523
O 3.241125 -2.033664 -1.513922
O 5.165480 -0.063665 -1.650371
C 5.183460 -1.005398 0.554079
C 4.523021 -1.075181 1.911302
C 5.416663 -0.937329 3.114231
O 3.315153 -1.279333 2.029955
O 5.129063 1.985765 0.521314
C 5.930174 2.508670 -0.538618
C -4.584525 0.996248 -0.160824
C -4.750558 2.491971 -0.171957
C -5.604137 0.121846 -0.007798
C -7.072062 0.413507 0.211181
C -7.550345 -0.058168 1.600229
C -7.508736 -1.561889 1.843818
C -7.898016 -0.193688 -0.925443
H -1.834874 -2.267572 -1.515253
H -2.173910 2.206366 0.416188
H 0.272894 2.412675 0.512672
H 2.668320 2.669356 0.645850
H 1.647423 -2.626299 -1.678503
H 5.152767 -0.947649 -2.068958
H 6.186295 -0.573777 0.665399
H 5.337693 -2.032391 0.201460
H 4.818366 -1.001913 4.027240
H 6.155054 -1.742751 3.115425
H 5.916205 0.034352 3.089921
H 6.671184 1.769548 -0.855759
H 5.323098 2.841091 -1.388021
H 6.472044 3.375893 -0.149391
H -4.151837 2.945414 -0.970408
H -5.778553 2.809657 -0.359870
H -4.439243 2.920557 0.786419
H -5.367859 -0.943738 -0.036854
H -7.252558 1.492466 0.185033
H -6.948701 0.436459 2.373739
H -8.582446 0.283719 1.750181
H -6.492263 -1.957550 1.764908
H -7.870300 -1.783135 2.853619
H -8.149234 -2.101378 1.140323
H -7.628641 0.263348 -1.884567
H -8.967272 -0.020122 -0.763928
H -7.739271 -1.273226 -1.020490
SCF Energy (B3LYP/6-31G**)= -1361.70366586
Number of imaginary frequencies = 0

1a_c027
MMFF Geometry
C -1.834102 -1.585291 -0.473904
C -2.076009 1.063756 0.065545
N -3.077842 -1.084982 -0.275081
C -0.655381 -0.836664 -0.425300
C -0.793104 0.532909 -0.147819
C -3.195018 0.235077 -0.003624
C 0.356773 1.340894 -0.092398
C 1.639146 0.808685 -0.304901
C 1.773451 -0.556312 -0.573612
C 0.631206 -1.374723 -0.637701
C 3.125795 -1.124281 -0.795637
C 4.360720 -0.201014 -0.695762
C 4.051452 1.261494 -0.418573
C 2.795181 1.705835 -0.242959
O 0.714292 -2.720258 -0.913833
O 3.213443 -2.323918 -1.077581
O 5.007968 -0.260407 -1.986258
C 5.358581 -0.809085 0.299854
C 4.869217 -0.816738 1.731309
C 5.884042 -1.192567 2.779307
O 3.705601 -0.562851 2.039885
O 5.185084 2.054859 -0.377804
C 5.040825 3.441934 -0.095960
C -4.577520 0.745217 0.229529
C -4.714256 1.963779 1.101852
C -5.625709 0.088234 -0.314992
C -7.096238 0.429965 -0.224594
C -7.882271 -0.650927 0.540284
C -7.479124 -0.764056 2.005222
C -7.662914 0.586304 -1.639893
H -1.809317 -2.652693 -0.682672
H -2.190587 2.122793 0.271097
H 0.244878 2.402843 0.124001
H 2.581709 2.749421 -0.040405
H 1.663007 -2.954404 -1.036761
H 4.501779 0.307477 -2.591158
H 6.307654 -0.259213 0.263681
H 5.594363 -1.844689 0.023559
H 5.420999 -1.160826 3.769464
H 6.247316 -2.205633 2.590256
H 6.714522 -0.482682 2.756642
H 4.449234 3.938820 -0.871464
H 4.600757 3.596038 0.894502
H 6.038777 3.890154 -0.094685
H -4.471020 2.870250 0.537883
H -5.719712 2.084937 1.512272
H -4.047703 1.900306 1.969711
H -5.420308 -0.803614 -0.911372
H -7.248743 1.388229 0.281489
H -8.953157 -0.415756 0.497249
H -7.756101 -1.628615 0.058067
H -7.587860 0.197437 2.516977
H -8.118344 -1.492315 2.514679
H -6.443130 -1.098907 2.113491

H -7.125227 1.363933 -2.194035
H -8.719152 0.874571 -1.603904
H -7.589306 -0.346922 -2.209900
SCF Energy (B3LYP/6-31G**)= -1361.70927778
Number of imaginary frequencies = 0

1a_c028
MMFF Geometry
C -1.808241 -1.675717 -0.691052
C -2.110620 1.003758 -0.381920
N -3.067254 -1.178328 -0.625822
C -0.642345 -0.909736 -0.613163
C -0.811145 0.475038 -0.453351
C -3.214938 0.157383 -0.470773
C 0.325133 1.299581 -0.368750
C 1.623971 0.769630 -0.440174
C 1.788596 -0.609708 -0.594364
C 0.660707 -1.445418 -0.684198
C 3.158163 -1.175060 -0.667870
C 4.374932 -0.230911 -0.543270
C 4.034508 1.243914 -0.400267
C 2.765219 1.683717 -0.355807
O 0.774691 -2.806794 -0.849366
O 3.276084 -2.391161 -0.850910
O 5.120082 -0.376862 -1.772607
C 5.297551 -0.750820 0.568145
C 4.699231 -0.659216 1.954577
C 5.632968 -0.942842 3.102221
O 3.513322 -0.398940 2.153426
O 5.155791 2.052702 -0.330956
C 4.980067 3.454567 -0.163254
C -4.615301 0.669140 -0.415204
C -4.838114 2.093401 -0.846540
C -5.606403 -0.156842 -0.012631
C -7.078296 0.153573 0.143132
C -7.584084 -0.208690 1.553376
C -6.936933 0.623487 2.653767
C -7.870901 -0.619408 -0.913753
H -1.759595 -2.755633 -0.813366
H -2.248120 2.070902 -0.243141
H 0.189109 2.373133 -0.241986
H 2.529151 2.736518 -0.248323
H 1.731750 -3.037067 -0.881092
H 4.657947 0.138344 -2.454956
H 6.242517 -0.192770 0.563656
H 5.561557 -1.800813 0.388654
H 5.094614 -0.844218 4.048939
H 6.017579 -1.962131 3.017742
H 6.457204 -0.225461 3.090100
H 4.447169 3.886417 -1.016369
H 4.463056 3.674268 0.776350
H 5.971648 3.914467 -0.117725
H -4.248900 2.328772 -1.740358
H -5.876642 2.299178 -1.117724
H -4.555608 2.786298 -0.047159
H -5.344917 -1.182716 0.256416
H -7.274763 1.219445 -0.008978
H -8.669126 -0.051608 1.597984
H -7.412321 -1.272546 1.760678
H -7.087314 1.693076 2.475901
H -7.382660 0.375724 3.622484
H -5.861845 0.432323 2.723950
H -7.541233 -0.352626 -1.924185
H -8.939387 -0.389005 -0.841891
H -7.752189 -1.702443 -0.794794
SCF Energy (B3LYP/6-31G**)= -1361.70932576
Number of imaginary frequencies = 0

1a_c029
MMFF Geometry
C -1.611755 -2.325980 -0.343157
C -2.561359 0.208088 -0.094938
N -2.953222 -2.142139 -0.398287
C -0.671409 -1.305727 -0.167250
C -1.175024 0.001848 -0.041388
C -3.414681 -0.880111 -0.265844
C -0.279909 1.071472 0.138196
C 1.105078 0.859272 0.184338
C 1.603663 -0.438687 0.051609
C 0.721500 -1.519206 -0.110534
C 3.069550 -0.650091 0.118712
C 4.018431 0.555894 -0.104844
C 3.325841 1.864284 0.253517
C 2.001206 1.988159 0.409527
O 1.172706 -2.813798 -0.218143
O 3.474574 -1.800130 0.310823
O 4.335472 0.642197 -1.496414
C 5.283179 0.361224 0.757778
C 6.122965 -0.815624 0.301446
C 6.835151 -1.604626 1.366171
O 6.258871 -1.076427 -0.896009
O 4.122815 2.977073 0.499516
C 4.626188 3.537984 -0.715382
C -4.886888 -0.722288 -0.337808
C -5.546885 -1.553947 -1.404548
C -5.531633 0.094005 0.523197
C -7.021635 0.342755 0.617050
C -7.368816 1.809247 0.300544
C -7.052483 2.204525 -1.136577
C -7.504314 -0.018109 2.025814
H -1.297442 -3.362059 -0.450280
H -2.970878 1.210612 -0.018109
H -0.671999 2.082752 0.241996
H 1.572093 2.946901 0.685797
H 2.144716 -2.820844 -0.065848
H 4.945219 -0.102419 -1.697387
H 5.003514 0.211444 1.807729
H 5.950967 1.228996 0.705931
H 7.389620 -2.427192 0.906175
H 7.535812 -0.954445 1.895486
H 6.104747 -2.021383 2.064040
H 5.448570 2.934293 -1.110069
H 3.835743 3.652592 -1.465325
H 5.021775 4.531478 -0.484180
H -5.630544 -2.597305 -1.082572
H -6.547217 -1.198163 -1.662867
H -4.967193 -1.523863 -2.334653
H -4.950047 0.628449 1.274867
H -7.573406 -0.296833 -0.078771
H -8.440437 1.970793 0.472200
H -6.836367 2.484168 0.982670
H -7.562899 1.545189 -1.845782
H -7.387871 3.229299 -1.325921
H -5.977812 2.164540 -1.338318
H -7.279678 -1.064504 2.261301
H -8.588359 0.115620 2.109297
H -7.029321 0.609355 2.788627
SCF Energy (B3LYP/6-31G**)= -1361.70802270
Number of imaginary frequencies = 0

1a_c030
MMFF Geometry
C 1.679616 -2.202519 -0.002125
C 2.512484 0.311721 -0.609962
N 3.010828 -1.990439 -0.140909
C 0.694041 -1.221135 -0.148875
C 1.137286 0.075724 -0.466458
C 3.414506 -0.735459 -0.433179
C 0.193760 1.104005 -0.639646
C -1.178828 0.863272 -0.486749
C -1.616263 -0.422436 -0.160381
C -0.687699 -1.464398 -0.005204
C -3.071753 -0.665840 -0.017822
C -4.021775 0.539793 0.202899
C -3.420842 1.815576 -0.372669
C -2.130814 1.946086 -0.709055
O -1.081624 -2.749306 0.286288
O -3.462006 -1.836626 -0.039983
O -4.162867 0.765624 1.607711
C -5.378802 0.233542 -0.465145
C -6.117131 -0.903953 0.212657
C -6.933831 -1.812174 -0.665699

O -6.091983 -1.040787 1.437822
O -4.276693 2.881940 -0.626329
C -4.640150 3.558299 0.579663
C 4.877955 -0.547716 -0.577517
C 5.575477 -1.657762 -1.316652
C 5.486723 0.531863 -0.041660
C 6.965434 0.854396 -0.050108
C 7.509010 1.044895 1.379284
C 7.440084 -0.224050 2.220186
C 7.197566 2.123537 -0.874020
H 1.413089 -3.229025 0.240706
H 2.875502 1.301028 -0.870913
H 0.538106 2.105761 -0.894806
H -1.769840 2.878005 -1.134351
H -2.064433 -2.789028 0.262873
H -4.718759 0.035304 1.960212
H -5.229666 -0.020151 -1.521763
H -6.061307 1.089932 -0.415797
H -7.399776 -2.591957 -0.057028
H -7.715734 -1.233241 -1.162992
H -6.285279 -2.286459 -1.406396
H -5.386663 2.984604 1.136552
H -3.765368 3.765579 1.205877
H -5.092895 4.514569 0.301196
H 4.991553 -1.970292 -2.190380
H 6.554851 -1.362179 -1.700761
H 5.714272 -2.526445 -0.664404
H 4.883153 1.259158 0.502121
H 7.542592 0.049857 -0.516191
H 8.557383 1.364351 1.326118
H 6.962666 1.845678 1.893585
H 7.973054 -1.046587 1.732691
H 7.903798 -0.052232 3.196874
H 6.405956 -0.536648 2.393756
H 6.838816 1.995213 -1.901480
H 8.265226 2.363588 -0.922981
H 6.678967 2.986339 -0.440606
SCF Energy (B3LYP/6-31G**)= -1361.70814096
Number of imaginary frequencies = 0

1a_c031
MMFF Geometry
C -1.456660 -2.527437 -0.095646
C -2.253612 0.050261 -0.409768
N -2.787408 -2.270194 -0.097776
C -0.454807 -1.562856 -0.246342
C -0.879682 -0.233632 -0.411389
C -3.172041 -0.984334 -0.243729
C 0.080666 0.779977 -0.577330
C 1.455788 0.494434 -0.577393
C 1.876529 -0.827378 -0.405236
C 0.925788 -1.851880 -0.244808
C 3.328058 -1.133818 -0.401763
C 4.345776 0.018209 -0.558458
C 3.734427 1.395918 -0.757239
C 2.405083 1.594058 -0.763852
O 1.293958 -3.168564 -0.088151
O 3.672502 -2.315477 -0.295089
O 5.108318 -0.283063 -1.748276
C 5.346630 -0.039068 0.604177
C 4.738346 0.276079 1.952966
C 5.706008 0.457513 3.093190
O 3.524116 0.353982 2.135311
O 4.683895 2.387932 -0.932290
C 4.247761 3.731968 -1.098497
C -4.635313 -0.746410 -0.241009
C -5.430733 -1.765795 -1.011403
C -5.160123 0.298075 0.435001
C -6.624647 0.656662 0.580776
C -6.904984 1.963593 -0.182699
C -8.377515 2.350166 -0.202520
C -6.969852 0.765708 2.069238
H -1.204087 -3.577575 0.035379
H -2.604037 1.067981 -0.550352
H -0.255279 1.808608 -0.704468
H 1.975341 2.580068 -0.901008
H 2.277357 -3.220407 -0.104731
H 4.560021 -0.040844 -2.513342
H 6.170390 0.663732 0.425571
H 5.803041 -1.034951 0.669789
H 5.156364 0.683166 4.011230
H 6.276604 -0.462925 3.238881
H 6.379773 1.289300 2.873369
H 3.645305 3.837889 -2.006302
H 3.696483 4.076007 -0.217501
H 5.135289 4.362161 -1.207878
H -5.550163 -2.683536 -0.426023
H -6.425302 -1.406041 -1.287172
H -4.930746 -2.018622 -1.953827
H -4.488408 0.961840 0.980190
H -7.258366 -0.132378 0.163749
H -6.326147 2.789857 0.249494
H -6.568027 1.855122 -1.221932
H -8.739988 2.603927 0.797779
H -8.525727 3.228139 -0.839606
H -8.992351 1.536444 -0.599736
H -6.686306 -0.147588 2.604913
H -8.045350 0.905784 2.216515
H -6.451482 1.607643 2.541804
SCF Energy (B3LYP/6-31G**)= -1361.70718940
Number of imaginary frequencies = 0

1a_c032
MMFF Geometry
C -1.497552 -2.371017 -0.321941
C -2.211512 0.162465 0.349112
N -2.800539 -2.133926 -0.033761
C -0.485174 -1.405862 -0.302646
C -0.866712 -0.099422 0.047041
C -3.145519 -0.869893 0.291659
C 0.107055 0.914186 0.086907
C 1.452896 0.650507 -0.215939
C 1.830985 -0.650521 -0.559776
C 0.866070 -1.673263 -0.606305
C 3.251768 -0.934679 -0.878367
C 4.289634 0.206990 -0.795195
C 3.722125 1.568669 -0.427597
C 2.416600 1.752268 -0.166156
O 1.190866 -2.965280 -0.951186
O 3.551702 -2.082107 -1.224475
O 4.856687 0.327150 -2.118768
C 5.444399 -0.234354 0.115013
C 5.053324 -0.393953 1.567642
C 6.183421 -0.600644 2.542087
O 3.883224 -0.388833 1.947799
O 4.682524 2.565295 -0.403055
C 4.291482 3.884750 -0.041946
C -4.578288 -0.654949 0.606392
C -5.198614 -1.743663 1.440305
C -5.229263 0.430064 0.134813
C -6.691730 0.779628 0.318695
C -7.351462 0.953343 -1.063165
C -8.855674 1.179109 -0.992655
C -6.799302 2.041670 1.177429
H -1.277204 -3.404792 -0.579826
H -2.524514 1.160098 0.640827
H -0.194625 1.924326 0.362063
H 2.018925 2.725088 0.100647
H 2.157724 -3.004276 -1.134756
H 4.215186 0.809916 -2.666521
H 6.266125 0.490817 0.057536
H 5.857640 -1.192389 -0.225267
H 5.783673 -0.699547 3.555125
H 6.726307 -1.513327 2.284609
H 6.855596 0.260413 2.511914
H 3.570233 4.288748 -0.759488
H 3.889921 3.908799 0.976187
H 5.182971 4.518314 -0.067562
H -4.517195 -2.057454 2.239866
H -6.117814 -1.425015 1.938089
H -5.431534 -2.616849 0.821811
H -4.682382 1.142118 -0.483899

H -7.219738 -0.025805 0.838340
H -6.891994 1.790912 -1.603456
H -7.170004 0.054010 -1.666149
H -9.096953 2.133999 -0.516935
H -9.279371 1.197079 -2.002033
H -9.348028 0.377286 -0.433554
H -6.263040 1.917377 2.125287
H -7.841919 2.265505 1.423771
H -6.377977 2.913748 0.664729
SCF Energy (B3LYP/6-31G**)= -1361.70719529
Number of imaginary frequencies = 0

1a_c033
MMFF Geometry
C 1.776894 2.536252 0.128368
C 2.436655 -0.063662 -0.310588
N 3.089651 2.201970 0.147348
C 0.727687 1.640314 -0.100100
C 1.081536 0.297514 -0.329234
C 3.406024 0.906081 -0.062305
C 0.072277 -0.650363 -0.579682
C -1.281000 -0.285334 -0.588197
C -1.626033 1.045908 -0.344825
C -0.632424 2.009395 -0.117755
C -3.058350 1.409851 -0.338850
C -4.111768 0.296230 -0.187300
C -3.614496 -1.027602 -0.747886
C -2.302887 -1.285120 -0.906282
O -0.942142 3.331216 0.096136
O -3.379388 2.601035 -0.401658
O -5.254153 0.741435 -0.937511
C -4.486840 0.240218 1.297885
C -5.346949 -0.946710 1.671480
C -6.796683 -0.926183 1.261561
O -4.873705 -1.890662 2.308133
O -4.633084 -1.916082 -1.041846
C -4.298938 -3.230487 -1.468767
C 4.852780 0.584381 -0.037144
C 5.723915 1.594297 -0.734531
C 5.297612 -0.522570 0.595421
C 6.731578 -0.980770 0.752878
C 6.973907 -2.326233 0.044139
C 6.811462 -2.245834 -1.468731
C 7.058526 -1.107412 2.244482
H 1.581404 3.591097 0.309472
H 2.734669 -1.090020 -0.501959
H 0.351629 -1.686242 -0.770516
H -1.946426 -2.235901 -1.287247
H -1.901608 3.464335 -0.074374
H -5.258357 1.718358 -0.892058
H -5.027000 1.145650 1.602708
H -3.585563 0.194740 1.923831
H -7.326674 -1.742628 1.760568
H -7.252640 0.018141 1.569423
H -6.890879 -1.055142 0.182258
H -3.765669 -3.206951 -2.424387
H -3.714639 -3.753534 -0.704897
H -5.231379 -3.783117 -1.616900
H 5.865325 2.479983 -0.106180
H 6.710251 1.200104 -0.990899
H 5.270566 1.910198 -1.681486
H 4.574167 -1.170021 1.091832
H 7.428725 -0.245912 0.338564
H 7.993244 -2.669294 0.261382
H 6.294198 -3.093198 0.436664
H 7.471468 -1.482967 -1.893758
H 7.068180 -3.207354 -1.924880
H 5.781310 -2.009014 -1.751433
H 6.907111 -0.153177 2.761557
H 8.104338 -1.400294 2.387667
H 6.429557 -1.860134 2.733378
SCF Energy (B3LYP/6-31G**)= -1361.70948639
Number of imaginary frequencies = 0

1a_c034
MMFF Geometry
C -1.425367 -2.270715 -0.723378
C -2.348876 0.246843 -0.279145
N -2.765570 -2.092610 -0.629257
C -0.474448 -1.251668 -0.610537
C -0.964316 0.048475 -0.383416
C -3.212890 -0.840061 -0.397905
C -0.058918 1.120584 -0.276894
C 1.323752 0.914336 -0.374342
C 1.803402 -0.382583 -0.580948
C 0.915269 -1.458508 -0.718708
C 3.265488 -0.577312 -0.681603
C 4.181215 0.497134 -0.049010
C 3.566792 1.888092 -0.193312
C 2.238063 2.060212 -0.326352
O 1.357904 -2.735106 -0.967691
O 3.686481 -1.592897 -1.238030
O 5.446291 0.477159 -0.723539
C 4.451386 0.206764 1.429051
C 4.697167 -1.261062 1.690106
C 6.055203 -1.822156 1.363070
O 3.791558 -1.973531 2.127226
O 4.500295 2.906894 -0.147464
C 4.049883 4.253395 -0.240595
C -4.684634 -0.688973 -0.304850
C -5.454136 -1.483661 -1.325391
C -5.234419 0.088824 0.652298
C -6.706149 0.322968 0.916856
C -7.089657 1.798362 0.699050
C -6.932114 2.252596 -0.746801
C -7.032229 -0.096981 2.353909
H -1.121312 -3.300400 -0.900241
H -2.751460 1.242592 -0.121374
H -0.444569 2.127036 -0.118208
H 1.786582 3.042898 -0.408614
H 2.332868 -2.713384 -1.099474
H 5.304435 0.862807 -1.605548
H 3.596882 0.498502 2.054016
H 5.319258 0.766156 1.799595
H 6.833255 -1.164747 1.758987
H 6.165164 -2.804042 1.832107
H 6.165812 -1.929217 0.282283
H 3.555732 4.434472 -1.200472
H 3.388425 4.502740 0.595268
H 4.926096 4.905926 -0.183785
H -5.499956 -2.539081 -1.036857
H -6.477386 -1.124824 -1.460206
H -4.978537 -1.414254 -2.310800
H -4.576198 0.596423 1.357978
H -7.328343 -0.292362 0.259642
H -8.136786 1.945349 0.992047
H -6.488243 2.449442 1.346131
H -7.514612 1.618320 -1.422532
H -7.288869 3.281673 -0.857359
H -5.885550 2.228263 -1.065125
H -6.780467 -1.150272 2.521395
H -8.101201 0.025591 2.559536
H -6.479082 0.503072 3.085468
SCF Energy (B3LYP/6-31G**)= -1361.70087255
Number of imaginary frequencies = 0

1a_c035
MMFF Geometry
C -1.532297 -2.565306 -0.072411
C -2.333810 0.008464 -0.406343
N -2.863504 -2.310428 -0.076002
C -0.532175 -1.600175 -0.231019
C -0.959421 -0.273026 -0.406287
C -3.250374 -1.026426 -0.231734
C -0.000955 0.740958 -0.580544
C 1.374660 0.457842 -0.578919
C 1.797796 -0.861846 -0.396596
C 0.848927 -1.886741 -0.227781
C 3.249865 -1.165691 -0.391310
C 4.265488 -0.013133 -0.557413
C 3.651634 1.361902 -0.766714
C 2.321941 1.557644 -0.774346

O 1.219481 -3.201509 -0.060986
O 3.596434 -2.345872 -0.275541
O 5.028101 -0.322356 -1.745144
C 5.266891 -0.059557 0.605234
C 4.658572 0.265057 1.951760
C 5.626352 0.457120 3.090141
O 3.444277 0.342235 2.133976
O 4.599285 2.354186 -0.949892
C 4.160717 3.696114 -1.126414
C -4.713827 -0.790915 -0.230449
C -5.509416 -1.817806 -0.990728
C -5.241117 0.261349 0.431436
C -6.707013 0.612199 0.569173
C -7.105537 1.775323 -0.359783
C -6.408612 3.103034 -0.087863
C -7.042779 0.900145 2.035886
H -1.277825 -3.613948 0.066681
H -2.685991 1.024467 -0.554910
H -0.338776 1.767967 -0.715585
H 1.890407 2.541794 -0.919034
H 2.202963 -3.251745 -0.077551
H 4.479084 -0.087087 -2.511861
H 6.089342 0.643274 0.420809
H 5.725082 -1.054091 0.678449
H 5.076664 0.688976 4.006608
H 6.198635 -0.461139 3.242800
H 6.298556 1.288352 2.863550
H 3.557725 3.793881 -2.034778
H 3.609174 4.046052 -0.247912
H 5.047091 4.326994 -1.241069
H -5.623936 -2.732054 -0.398968
H -6.506438 -1.461513 -1.263381
H -5.012995 -2.074977 -1.933811
H -4.570021 0.931646 0.968520
H -7.323156 -0.248316 0.284738
H -6.908441 1.486824 -1.400364
H -8.189028 1.932473 -0.285447
H -5.322976 3.018547 -0.192469
H -6.752082 3.855647 -0.805374
H -6.634211 3.477455 0.914706
H -6.863039 0.013101 2.654113
H -8.097836 1.172941 2.145489
H -6.437131 1.714830 2.446896
SCF Energy (B3LYP/6-31G**)= -1361.70619227
Number of imaginary frequencies = 0

1a_c036
MMFF Geometry
C -1.495130 -2.131096 -0.384985
C -2.283120 0.340358 0.427161
N -2.803662 -1.948118 -0.083501
C -0.512358 -1.139164 -0.310784
C -0.931551 0.136413 0.112437
C -3.185669 -0.715090 0.311958
C 0.009178 1.179086 0.204234
C 1.359350 0.966839 -0.105305
C 1.770539 -0.306655 -0.510109
C 0.843187 -1.350870 -0.632343
C 3.199215 -0.508286 -0.832423
C 4.221921 0.480708 -0.224911
C 3.631942 1.886889 -0.137210
C 2.302722 2.089249 -0.065902
O 1.213212 -2.600167 -1.068146
O 3.512164 -1.464174 -1.544174
O 5.381944 0.516562 -1.066838
C 4.683393 0.030817 1.163133
C 4.922078 -1.459542 1.233645
C 6.206813 -1.999959 0.664914
O 4.065411 -2.202896 1.715703
O 4.590565 2.882910 -0.115627
C 4.168837 4.237252 -0.003809
C -4.623783 -0.560828 0.636901
C -5.211606 -1.712768 1.406895
C -5.307621 0.527429 0.223039
C -6.779240 0.820397 0.421209
C -7.492891 1.057521 -0.923933
C -7.506730 -0.175032 -1.819874
C -6.929446 2.050646 1.319738
H -1.244849 -3.141963 -0.700398
H -2.626972 1.310452 0.772268
H -0.323438 2.167689 0.518689
H 1.871386 3.080795 0.018031
H 2.160590 -2.576555 -1.333291
H 5.131491 0.995684 -1.875852
H 3.930629 0.265192 1.927517
H 5.608799 0.536250 1.465763
H 7.049407 -1.398670 1.015325
H 6.352728 -3.027654 1.009499
H 6.165863 -1.992407 -0.425986
H 3.553134 4.526698 -0.861403
H 3.635105 4.403762 0.937345
H 5.062184 4.868678 -0.001388
H -4.522251 -2.049255 2.190230
H -6.141417 -1.451246 1.917926
H -5.416120 -2.556899 0.739875
H -4.784049 1.287111 -0.357821
H -7.284604 -0.013342 0.918319
H -8.531884 1.354061 -0.732828
H -7.023837 1.888829 -1.465381
H -7.965409 -1.026732 -1.307531
H -8.086581 0.026803 -2.726246
H -6.496404 -0.460166 -2.128425
H -6.448344 1.888175 2.290814
H -7.986930 2.267665 1.505609
H -6.479132 2.940113 0.864450
SCF Energy (B3LYP/6-31G**)= -1361.70101515
Number of imaginary frequencies = 0

1a_c037
MMFF Geometry
C -1.829277 -2.311901 0.206566
C -2.359330 0.350375 0.343938
N -3.104916 -1.930009 0.456137
C -0.756336 -1.435829 0.014458
C -1.042453 -0.059854 0.089227
C -3.359258 -0.605055 0.514008
C -0.005641 0.872973 -0.097088
C 1.309908 0.457090 -0.344335
C 1.588911 -0.909957 -0.405814
C 0.564598 -1.854196 -0.243464
C 2.984327 -1.331347 -0.649232
C 4.121802 -0.317906 -0.421505
C 3.655102 1.111797 -0.649810
C 2.354646 1.453723 -0.589744
O 0.806580 -3.204720 -0.323639
O 3.213988 -2.499698 -0.978125
O 5.136594 -0.655373 -1.381923
C 4.668866 -0.575226 0.987082
C 5.647063 0.473124 1.469030
C 7.034427 0.468970 0.881325
O 5.317847 1.291672 2.330378
O 4.691611 1.994948 -0.892775
C 4.401511 3.381758 -1.011844
C -4.766730 -0.231899 0.791789
C -5.437943 -1.080181 1.838267
C -5.357164 0.772775 0.109713
C -6.788479 1.250321 0.228936
C -7.514833 1.198880 -1.129261
C -7.670097 -0.216530 -1.672028
C -6.796578 2.680297 0.775308
H -1.685169 -3.389481 0.164618
H -2.600519 1.406033 0.422663
H -0.233692 1.937501 -0.049390
H 2.022774 2.476265 -0.732725
H 1.728275 -3.344176 -0.637342
H 5.079766 -1.621170 -1.524979
H 5.176198 -1.546890 1.041744
H 3.851211 -0.613897 1.719289
H 7.011584 0.807430 -0.155651
H 7.674644 1.143553 1.457123
H 7.455730 -0.537756 0.939915
H 3.761958 3.573858 -1.879142

H 3.945711 3.766609 -0.093903
H 5.346470 3.910871 -1.166376
H -4.761155 -1.267096 2.680328
H -6.324128 -0.604776 2.265769
H -5.739728 -2.044580 1.416180
H -4.781628 1.300700 -0.651112
H -7.355587 0.628093 0.928116
H -8.515455 1.635442 -1.019102
H -6.985369 1.810815 -1.870512
H -8.192001 -0.856627 -0.953581
H -8.254058 -0.201066 -2.597892
H -6.700286 -0.669825 -1.898426
H -6.305320 2.729138 1.753634
H -7.823220 3.040942 0.901246
H -6.277818 3.372312 0.102088
SCF Energy (B3LYP/6-31G**)= -1361.70958492
Number of imaginary frequencies = 0

1a_c038
MMFF Geometry
C 1.882054 -1.516236 0.155504
C 2.343364 1.129044 -0.247531
N 3.167764 -1.090183 0.116719
C 0.764152 -0.692706 0.002783
C 1.015079 0.674755 -0.205079
C 3.395050 0.228489 -0.083902
C -0.068898 1.555871 -0.369853
C -1.392857 1.097228 -0.319581
C -1.639172 -0.260534 -0.104415
C -0.567553 -1.155653 0.043240
C -3.043565 -0.734251 -0.069852
C -4.182066 0.291998 0.165519
C -3.759650 1.683598 -0.287371
C -2.490258 2.034675 -0.531797
O -0.768631 -2.503248 0.229152
O -3.242739 -1.946419 -0.190247
O -4.432337 0.390074 1.569841
C -5.435904 -0.169204 -0.607003
C -6.021381 -1.452889 -0.052186
C -6.635446 -2.409520 -1.037656
O -6.041765 -1.672952 1.160882
O -4.757883 2.621986 -0.526035
C -5.287787 3.143240 0.695234
C 4.823757 0.655956 -0.135648
C 5.133562 1.900091 -0.923622
C 5.761090 -0.091351 0.488783
C 7.250195 0.156817 0.581545
C 8.052568 -0.938882 -0.144305
C 7.814876 -0.961074 -1.649091
C 7.658406 0.213597 2.057625
H 1.770051 -2.585742 0.320204
H 2.543925 2.185104 -0.394828
H 0.125763 2.614643 -0.538056
H -2.258561 3.039654 -0.872319
H -1.730101 -2.692180 0.140336
H -4.884429 -0.440684 1.838359
H -5.191902 -0.319203 -1.665780
H -6.246409 0.566451 -0.547477
H -7.004993 -3.293818 -0.511256
H -7.470618 -1.923011 -1.547198
H -5.880790 -2.723367 -1.763047
H -5.964112 2.422420 1.163777
H -4.491619 3.434514 1.389228
H -5.869223 4.037182 0.450748
H 4.878661 2.794900 -0.346326
H 6.185748 1.974839 -1.208790
H 4.571759 1.916892 -1.864679
H 5.435428 -0.992457 1.013360
H 7.517563 1.124174 0.145283
H 9.123571 -0.776672 0.030610
H 7.812957 -1.926329 0.270258
H 8.039587 0.012684 -2.095733
H 8.463517 -1.706730 -2.120016
H 6.779988 -1.222125 -1.889901
H 7.108238 0.999844 2.586792
H 8.727180 0.433252 2.155124
H 7.463585 -0.736824 2.567508
SCF Energy (B3LYP/6-31G**)= -1361.70913921
Number of imaginary frequencies = 0

1a_c039
MMFF Geometry
C -1.854266 -1.565108 -0.498146
C -2.371143 1.073692 -0.120635
N -3.144220 -1.153115 -0.547012
C -0.758671 -0.730173 -0.265910
C -1.038487 0.633455 -0.069926
C -3.398781 0.162605 -0.360696
C 0.021140 1.524498 0.178503
C 1.350210 1.080036 0.220285
C 1.626081 -0.273521 0.014658
C 0.577521 -1.178847 -0.214098
C 3.034479 -0.732217 0.077543
C 4.175497 0.307591 -0.069652
C 3.706689 1.691355 0.361048
C 2.419328 2.027332 0.516532
O 0.805934 -2.523155 -0.392073
O 3.237890 -1.943027 0.204325
O 4.523758 0.418493 -1.451980
C 5.376286 -0.146028 0.787058
C 6.013739 -1.419427 0.266918
C 6.566739 -2.376717 1.287288
O 6.122628 -1.630411 -0.943046
O 4.675037 2.638330 0.676526
C 5.285032 3.173975 -0.500321
C -4.828856 0.580247 -0.438283
C -5.101823 2.003617 -0.843318
C -5.797974 -0.323601 -0.172908
C -7.295828 -0.114727 -0.166505
C -7.921792 -0.567149 1.167410
C -7.452522 0.258049 2.359475
C -7.918010 -0.892013 -1.329015
H -1.719325 -2.632636 -0.658443
H -2.595725 2.122732 0.041597
H -0.196622 2.579918 0.339281
H 2.152912 3.027250 0.846402
H 1.760757 -2.702441 -0.236584
H 5.002960 -0.405472 -1.693022
H 5.059357 -0.306276 1.824806
H 6.180850 0.598494 0.790107
H 6.982405 -3.253242 0.782735
H 7.358240 -1.885206 1.858059
H 5.765973 -2.703724 1.955148
H 6.000739 2.463605 -0.924193
H 4.537272 3.462186 -1.247410
H 5.837919 4.072001 -0.209050
H -4.438066 2.313080 -1.658881
H -6.117128 2.152397 -1.219446
H -4.954773 2.680086 0.005024
H -5.497003 -1.340851 0.087176
H -7.547173 0.941846 -0.301863
H -9.013642 -0.481780 1.099917
H -7.700866 -1.625567 1.355053
H -7.655753 1.322319 2.203597
H -7.980075 -0.057954 3.265181
H -6.380337 0.133290 2.538743
H -7.502469 -0.562740 -2.288042
H -9.001302 -0.733623 -1.365101
H -7.739079 -1.969258 -1.235544
SCF Energy (B3LYP/6-31G**)= -1361.70910327
Number of imaginary frequencies = 0

1a_c040
MMFF Geometry
C -1.755424 -1.641239 -0.312901
C -2.009834 1.035507 0.057486
N -3.000504 -1.136826 -0.133640
C -0.581357 -0.883825 -0.324897
C -0.725565 0.499917 -0.134714
C -3.123791 0.197174 0.053751
C 0.419284 1.316844 -0.143560
C 1.703033 0.779922 -0.334833

C 1.843896 -0.598710 -0.516486
C 0.706652 -1.426545 -0.515733
C 3.197760 -1.171500 -0.715693
C 4.427321 -0.236153 -0.688489
C 4.110931 1.239255 -0.502810
C 2.853506 1.686275 -0.342960
O 0.796096 -2.786543 -0.705532
O 3.290714 -2.386257 -0.920704
O 5.061874 -0.374634 -1.979227
C 5.439315 -0.772646 0.333652
C 4.964516 -0.691016 1.767704
C 5.992419 -0.992285 2.826981
O 3.802383 -0.424904 2.071529
O 5.239494 2.040540 -0.525146
C 5.088659 3.441987 -0.331708
C -4.507036 0.713372 0.267018
C -4.644950 1.985542 1.059076
C -5.557505 0.016589 -0.220545
C -7.028138 0.365266 -0.138248
C -7.725454 -0.596322 0.840576
C -9.187742 -0.252882 1.089424
C -7.637302 0.309719 -1.542934
H -1.725480 -2.719691 -0.452888
H -2.129738 2.104909 0.195297
H 0.302354 2.389826 0.005170
H 2.634999 2.739418 -0.205790
H 1.745092 -3.022298 -0.823098
H 4.545725 0.149939 -2.614079
H 6.384209 -0.220434 0.252155
H 5.679330 -1.822439 0.122148
H 5.539210 -0.899627 3.817861
H 6.360639 -2.013184 2.699738
H 6.817784 -0.280259 2.749910
H 4.485894 3.884208 -1.131331
H 4.657587 3.656916 0.651351
H 6.083502 3.895474 -0.369803
H -4.425318 2.855616 0.431719
H -5.645106 2.117911 1.479962
H -3.962307 1.989277 1.916673
H -5.355442 -0.915238 -0.753492
H -7.160586 1.390046 0.223310
H -7.659950 -1.628519 0.473400
H -7.202151 -0.573336 1.805412
H -9.791779 -0.389297 0.187810
H -9.599565 -0.907410 1.864459
H -9.296365 0.782212 1.428143
H -7.073132 0.940311 -2.239741
H -8.669462 0.673961 -1.540989
H -7.640058 -0.711612 -1.940137
SCF Energy (B3LYP/6-31G**)= -1361.70805240
Number of imaginary frequencies = 0

1a_c041
MMFF Geometry
C 1.752797 2.545185 0.167871
C 2.404517 -0.046476 -0.330001
N 3.063962 2.204500 0.187581
C 0.701623 1.659770 -0.089884
C 1.051162 0.320990 -0.349723
C 3.376174 0.912652 -0.051047
C 0.040050 -0.616078 -0.632333
C -1.310832 -0.244320 -0.640264
C -1.652038 1.082428 -0.363897
C -0.656279 2.035818 -0.107940
C -3.085915 1.450914 -0.353634
C -4.120749 0.320508 -0.149240
C -3.644387 -0.966936 -0.813100
C -2.337390 -1.223777 -1.005382
O -0.963913 3.353202 0.133780
O -3.379545 2.643808 -0.451956
O -5.358763 0.738938 -0.735044
C -4.339773 0.189298 1.362463
C -5.130486 -1.040326 1.748313
C -6.621467 -1.017087 1.535228
O -4.565084 -2.033493 2.211736
O -4.678536 -1.813220 -1.168831
C -4.362132 -3.146230 -1.554976
C 4.821318 0.583894 -0.023275
C 5.701950 1.606682 -0.689277
C 5.256687 -0.540080 0.585410
C 6.687478 -1.008252 0.742360
C 6.929423 -2.337271 0.003119
C 6.778672 -2.219656 -1.508526
C 7.002696 -1.172299 2.232843
H 1.560257 3.596083 0.373672
H 2.699726 -1.069142 -0.544015
H 0.316183 -1.647736 -0.848596
H -1.989489 -2.145981 -1.457996
H -1.921083 3.493090 -0.051227
H -5.259344 0.655858 -1.699605
H -4.868635 1.064180 1.761483
H -3.383249 0.139004 1.899214
H -7.079787 -1.842674 2.087254
H -7.035742 -0.080148 1.915870
H -6.853267 -1.128388 0.474544
H -3.802448 -3.660981 -0.767248
H -5.302664 -3.683651 -1.707716
H -3.810825 -3.156711 -2.500411
H 5.842474 2.476084 -0.038391
H 6.688467 1.214465 -0.947974
H 5.257038 1.947679 -1.631517
H 4.526801 -1.196022 1.060810
H 7.390815 -0.266693 0.351086
H 7.945641 -2.689969 0.219539
H 6.243549 -3.110482 0.371951
H 7.445072 -1.449671 -1.910094
H 7.034730 -3.171007 -1.985874
H 5.751682 -1.971533 -1.793032
H 6.851442 -0.230165 2.771696
H 8.046159 -1.473156 2.376635
H 6.366906 -1.933824 2.698741
SCF Energy (B3LYP/6-31G**)= -1361.70344975
Number of imaginary frequencies = 0

1a_c042
MMFF Geometry
C -1.737864 -1.594392 -0.772599
C -2.024525 1.055638 -0.261008
N -2.993919 -1.096907 -0.664755
C -0.567533 -0.842446 -0.642099
C -0.728206 0.527174 -0.377593
C -3.133772 0.224042 -0.408790
C 0.412850 1.337160 -0.235884
C 1.708511 0.807466 -0.352580
C 1.865058 -0.557136 -0.611046
C 0.732314 -1.377904 -0.758859
C 3.231245 -1.122334 -0.732794
C 4.453588 -0.196514 -0.542519
C 4.121721 1.265044 -0.287255
C 2.855016 1.706731 -0.204365
O 0.838277 -2.723506 -1.026714
O 3.341863 -2.321750 -1.007522
O 5.193194 -0.253124 -1.782518
C 5.377648 -0.803496 0.522591
C 4.785179 -0.813678 1.914512
C 5.721777 -1.187843 3.033522
O 3.601472 -0.563080 2.137461
O 5.247632 2.060550 -0.161909
C 5.080161 3.446648 0.111797
C -4.531191 0.737544 -0.309543
C -4.747522 2.190844 -0.634683
C -5.526054 -0.111133 0.032271
C -6.997413 0.194993 0.213250
C -7.451154 -0.290140 1.604180
C -8.887030 0.089486 1.940076
C -7.789257 -0.462856 -0.918710
H -1.695537 -2.662241 -0.976206
H -2.155757 2.109978 -0.041643
H 0.283139 2.398775 -0.027951
H 2.625076 2.749619 -0.016788
H 1.793944 -2.955626 -1.079768
H 4.731240 0.314454 -2.422096

H 6.325603 -0.251540 0.556183
H 5.635263 -1.838295 0.263306
H 5.187615 -1.158162 3.987260
H 6.100527 -2.199809 2.870781
H 6.549838 -0.475818 3.072032
H 4.546332 3.944354 -0.704044
H 4.567970 3.597476 1.067483
H 6.074389 3.896722 0.187673
H -4.158959 2.488232 -1.510278
H -5.785614 2.420781 -0.887861
H -4.459820 2.821971 0.212542
H -5.269502 -1.156246 0.220110
H -7.175415 1.273932 0.168287
H -7.344922 -1.379714 1.682333
H -6.795855 0.144523 2.370330
H -9.601120 -0.423390 1.289407
H -9.117894 -0.194680 2.971890
H -9.041344 1.168788 1.844073
H -7.403337 -0.154838 -1.897297
H -8.844578 -0.175000 -0.883308
H -7.734626 -1.556013 -0.864772
SCF Energy (B3LYP/6-31G**)= -1361.70817330
Number of imaginary frequencies = 0

1a_c043
MMFF Geometry
C -1.806513 -2.316630 0.218113
C -2.326387 0.349670 0.318052
N -3.078442 -1.926151 0.473585
C -0.732802 -1.447409 0.001715
C -1.013474 -0.068950 0.056878
C -3.327672 -0.599586 0.513229
C 0.023988 0.857704 -0.156126
C 1.335090 0.432573 -0.408158
C 1.609432 -0.937169 -0.447612
C 0.583720 -1.874995 -0.262028
C 3.004889 -1.366989 -0.692227
C 4.135008 -0.353209 -0.399791
C 3.679588 1.064870 -0.726416
C 2.381053 1.417099 -0.696443
O 0.822241 -3.226915 -0.325281
O 3.198811 -2.526548 -1.062002
O 5.263783 -0.698911 -1.210544
C 4.535663 -0.548682 1.067074
C 5.453519 0.534713 1.586584
C 6.904170 0.484773 1.183977
O 5.019679 1.435988 2.307901
O 4.724432 1.917163 -1.033209
C 4.462913 3.314799 -1.099262
C -4.731038 -0.217223 0.799212
C -5.394194 -1.046499 1.865906
C -5.325211 0.778434 0.107230
C -6.753737 1.262491 0.233434
C -7.494104 1.191935 -1.116312
C -7.659328 -0.231375 -1.634944
C -6.751785 2.700960 0.757098
H -1.666058 -3.395245 0.192271
H -2.563501 1.407189 0.382607
H -0.200209 1.923601 -0.125455
H 2.051029 2.428516 -0.907317
H 1.738833 -3.370691 -0.655691
H 5.060118 -0.413098 -2.118282
H 5.041456 -1.511679 1.212504
H 3.653517 -0.564695 1.720630
H 7.482518 1.154036 1.827395
H 7.289745 -0.529753 1.312184
H 7.019485 0.803599 0.146566
H 3.809281 3.548503 -1.945454
H 4.035602 3.679299 -0.159433
H 5.416097 3.827475 -1.258494
H -4.709360 -1.222395 2.703826
H -6.274322 -0.561286 2.294899
H -5.703525 -2.016409 1.462313
H -4.755816 1.292201 -0.667725
H -7.315607 0.653275 0.948130
H -8.492180 1.633468 -1.002916
H -6.970343 1.790315 -1.872531
H -8.175852 -0.858299 -0.901157
H -8.252676 -0.228657 -2.554946
H -6.693302 -0.691380 -1.863978
H -6.250399 2.763675 1.729477
H -7.775955 3.066922 0.887736
H -6.237779 3.380519 0.067738
SCF Energy (B3LYP/6-31G**)= -1361.70355203
Number of imaginary frequencies = 0

1a_c044
MMFF Geometry
C -1.581024 -2.345592 -0.348924
C -2.266406 0.153953 0.462798
N -2.876615 -2.116778 -0.023003
C -0.562689 -1.387778 -0.301632
C -0.929409 -0.098852 0.120940
C -3.208101 -0.869326 0.372065
C 0.050945 0.906660 0.192153
C 1.388908 0.651965 -0.150646
C 1.752344 -0.631914 -0.567087
C 0.780627 -1.646224 -0.645557
C 3.164789 -0.906654 -0.928161
C 4.210880 0.224223 -0.808555
C 3.658900 1.568413 -0.361517
C 2.359946 1.745039 -0.064904
O 1.090777 -2.920794 -1.061499
O 3.450938 -2.036626 -1.337578
O 4.752218 0.408196 -2.135559
C 5.380861 -0.268177 0.054829
C 5.017793 -0.498908 1.505168
C 6.165787 -0.760017 2.445017
O 3.855527 -0.507234 1.908591
O 4.625509 2.557776 -0.306202
C 4.249588 3.859241 0.128707
C -4.633329 -0.659819 0.727664
C -5.244288 -1.789622 1.513177
C -5.281556 0.450738 0.314659
C -6.724174 0.830421 0.569773
C -7.609030 0.586623 -0.668612
C -7.262105 1.413095 -1.901020
C -6.802903 2.278456 1.063389
H -1.372084 -3.366191 -0.662736
H -2.567520 1.137211 0.810660
H -0.239222 1.903102 0.523834
H 1.973446 2.705113 0.258491
H 2.053545 -2.955275 -1.266252
H 4.102875 0.921207 -2.645258
H 6.205585 0.454899 0.017375
H 5.781546 -1.209860 -0.341498
H 5.785669 -0.907919 3.459641
H 6.698044 -1.661205 2.131055
H 6.842308 0.098160 2.444679
H 3.516628 4.302498 -0.552891
H 3.868507 3.833861 1.154649
H 5.144147 4.488877 0.117057
H -4.631690 -2.018525 2.392744
H -6.248479 -1.569768 1.880760
H -5.319475 -2.693315 0.898477
H -4.739398 1.168714 -0.301189
H -7.137533 0.216396 1.376269
H -7.562504 -0.476259 -0.938833
H -8.653940 0.789114 -0.401295
H -6.242653 1.217033 -2.245614
H -7.942215 1.159061 -2.720885
H -7.365256 2.484788 -1.708519
H -6.267391 2.391930 2.012989
H -7.843850 2.574475 1.231861
H -6.360640 2.983109 0.351083
SCF Energy (B3LYP/6-31G**)= -1361.70674128
Number of imaginary frequencies = 0

1a_c045
MMFF Geometry
C -1.683713 -2.188668 -0.280896
C -2.216970 0.464593 -0.518402

N -2.982539 -1.801921 -0.299856
C -0.589135 -1.322837 -0.376470
C -0.877834 0.047331 -0.502086
C -3.235224 -0.480101 -0.407969
C 0.180265 0.967324 -0.612407
C 1.519868 0.547999 -0.592778
C 1.802643 -0.814564 -0.458752
C 0.755644 -1.746953 -0.358766
C 3.215463 -1.257476 -0.441927
C 4.340378 -0.209018 -0.447220
C 3.883205 1.222084 -0.652950
C 2.580914 1.551199 -0.720324
O 0.992747 -3.097616 -0.246880
O 3.468649 -2.467908 -0.463216
O 5.144645 -0.566958 -1.596655
C 5.230163 -0.384146 0.790111
C 4.572740 0.104078 2.060233
C 5.482714 0.657546 3.124134
O 3.360111 0.006978 2.245919
O 4.934104 2.121066 -0.732639
C 4.646018 3.475107 -1.059104
C -4.668243 -0.098140 -0.426472
C -5.523073 -0.983115 -1.290155
C -5.100538 0.946571 0.312524
C -6.501198 1.492947 0.499239
C -7.563913 0.479395 0.966143
C -7.189343 -0.237319 2.257355
C -6.969730 2.265340 -0.736689
H -1.538063 -3.262377 -0.183210
H -2.463852 1.515842 -0.629587
H -0.049717 2.027530 -0.712029
H 2.257699 2.577221 -0.854135
H 1.964110 -3.247446 -0.283012
H 5.277356 -1.533285 -1.549576
H 6.171469 0.162009 0.647628
H 5.510204 -1.434012 0.939591
H 4.890866 0.974352 3.987337
H 6.187710 -0.114989 3.440518
H 6.021130 1.523643 2.731333
H 4.139046 3.546932 -2.026800
H 4.055148 3.949170 -0.268965
H 5.596254 4.011822 -1.135581
H -5.736800 -1.928820 -0.780931
H -6.474200 -0.520959 -1.559053
H -5.019025 -1.206816 -2.237754
H -4.367211 1.483061 0.916829
H -6.421081 2.236963 1.305807
H -8.507256 1.014182 1.136601
H -7.775977 -0.264475 0.192300
H -6.979641 0.479518 3.057593
H -8.016197 -0.875916 2.584632
H -6.310606 -0.875269 2.124287
H -6.228708 3.015016 -1.036159
H -7.906496 2.793213 -0.526493
H -7.147058 1.607763 -1.592648
SCF Energy (B3LYP/6-31G**)= -1361.70432984
Number of imaginary frequencies = 0

1a_c046
MMFF Geometry
C -1.597949 -2.406503 -0.041878
C -2.286526 0.221493 -0.036305
N -2.909732 -2.094867 0.096118
C -0.564279 -1.474613 -0.183610
C -0.932899 -0.117928 -0.177779
C -3.240796 -0.786089 0.089619
C 0.061687 0.867151 -0.313855
C 1.416060 0.525378 -0.453920
C 1.778815 -0.824778 -0.454364
C 0.795239 -1.820710 -0.326877
C 3.206771 -1.185671 -0.606709
C 4.271541 -0.076586 -0.637348
C 3.724970 1.337062 -0.685110
C 2.407563 1.594679 -0.602179
O 1.110247 -3.159966 -0.340231
O 3.517005 -2.374760 -0.746413
O 4.973944 -0.302279 -1.883048
C 5.288025 -0.290387 0.491565
C 4.736666 0.064714 1.853049
C 5.718395 0.593561 2.864367
O 3.555528 -0.117983 2.146179
O 4.716358 2.298524 -0.793069
C 4.329796 3.654284 -0.981427
C -4.684365 -0.485474 0.250364
C -5.360590 -1.304604 1.314238
C -5.274957 0.438345 -0.538287
C -6.719530 0.891315 -0.593137
C -7.156249 1.748812 0.609243
C -6.306463 2.999279 0.800035
C -7.701374 -0.255928 -0.850070
H -1.388572 -3.474078 -0.034106
H -2.592476 1.262774 -0.010533
H -0.230041 1.916777 -0.308033
H 2.021624 2.607452 -0.623147
H 2.079208 -3.250675 -0.482318
H 5.159247 -1.260156 -1.924918
H 6.181843 0.318065 0.303112
H 5.633963 -1.330475 0.530653
H 5.200280 0.811225 3.802422
H 6.491200 -0.156121 3.050595
H 6.168418 1.516452 2.490120
H 3.726161 3.768742 -1.887592
H 3.797424 4.033254 -0.103278
H 5.239643 4.249074 -1.104976
H -4.740441 -1.356744 2.216832
H -6.316884 -0.885353 1.630037
H -5.541011 -2.324912 0.959460
H -4.660662 0.935028 -1.291067
H -6.790705 1.537798 -1.480536
H -8.197403 2.062859 0.461968
H -7.139352 1.164778 1.535345
H -6.301492 3.612558 -0.106639
H -6.711172 3.606414 1.616225
H -5.272304 2.748103 1.054566
H -7.367112 -0.881729 -1.684928
H -8.688360 0.142261 -1.111382
H -7.834864 -0.898411 0.024198
SCF Energy (B3LYP/6-31G**)= -1361.70439269
Number of imaginary frequencies = 0

1a_c047
MMFF Geometry
C 1.554427 -2.379896 0.018790
C 2.512578 0.161635 0.121333
N 2.895521 -2.209927 0.112952
C 0.618979 -1.341621 -0.030151
C 1.126527 -0.029772 0.024541
C 3.361140 -0.943192 0.155825
C 0.236316 1.058939 -0.024829
C -1.148167 0.860095 -0.113500
C -1.645669 -0.443936 -0.152255
C -0.772523 -1.541020 -0.130182
C -3.109105 -0.628141 -0.247106
C -4.018142 0.521245 0.252181
C -3.380058 1.873785 -0.079015
C -2.048203 2.010013 -0.228477
O -1.234119 -2.833502 -0.206221
O -3.528917 -1.703901 -0.679446
O -4.105494 0.476354 1.679868
C -5.414428 0.386447 -0.389708
C -6.130447 -0.876399 0.047849
C -7.056221 -1.519152 -0.948811
O -6.002075 -1.321737 1.190612
O -4.290168 2.914790 -0.157821
C -3.797352 4.245094 -0.264812
C 4.832682 -0.801997 0.265721
C 5.477142 -1.769288 1.221876
C 5.490056 0.117812 -0.472625
C 6.981716 0.370687 -0.514804
C 7.328415 1.781465 -0.004103
C 6.994936 1.985781 1.468538
C 7.481248 0.196092 -1.952788

H 1.236224 -3.419722 -0.016261
H 2.925764 1.163615 0.183643
H 0.633139 2.072925 0.006601
H -1.583800 2.967649 -0.433422
H -2.199196 -2.810485 -0.395545
H -4.673791 -0.291682 1.907906
H -5.329393 0.384989 -1.483027
H -6.076928 1.209416 -0.095878
H -7.495396 -2.421647 -0.514891
H -7.856745 -0.821616 -1.206573
H -6.494837 -1.798169 -1.843901
H -3.158080 4.497058 0.587510
H -3.266755 4.389279 -1.211289
H -4.655843 4.923150 -0.254415
H 5.562665 -2.761534 0.766468
H 6.474843 -1.454378 1.537159
H 4.885636 -1.859614 2.140573
H 4.919142 0.748578 -1.154528
H 7.523116 -0.357010 0.097598
H 8.402502 1.959823 -0.139968
H 6.806241 2.542305 -0.598050
H 7.494776 1.236923 2.091107
H 7.330380 2.975429 1.794991
H 5.917713 1.923987 1.650164
H 7.257078 -0.809375 -2.326475
H 8.566578 0.335243 -2.004760
H 7.017423 0.920255 -2.632249
SCF Energy (B3LYP/6-31G**)= -1361.71322098
Number of imaginary frequencies = 0

1a_c048
MMFF Geometry
C 1.702301 2.157978 -0.248784
C 2.246008 -0.490530 -0.515352
N 3.002725 1.778549 -0.291960
C 0.610871 1.287121 -0.333626
C 0.905184 -0.080746 -0.474637
C 3.260771 0.458892 -0.413984
C -0.148899 -1.006029 -0.575276
C -1.488896 -0.592843 -0.532368
C -1.779183 0.767526 -0.381824
C -0.735810 1.704375 -0.290785
C -3.196345 1.203773 -0.342241
C -4.321858 0.151831 -0.350164
C -3.841955 -1.262398 -0.590813
C -2.546216 -1.596158 -0.644723
O -0.977963 3.052814 -0.164613
O -3.456876 2.412946 -0.352536
O -5.146145 0.543828 -1.471641
C -5.182537 0.283284 0.911065
C -4.480139 -0.238808 2.142774
C -5.301382 -1.055582 3.103538
O -3.300824 0.027558 2.372984
O -4.786466 -2.281878 -0.577301
C -5.561526 -2.315604 -1.775981
C 4.695399 0.085007 -0.458078
C 5.531949 0.981890 -1.327319
C 5.144785 -0.963571 0.265084
C 6.551018 -1.503982 0.425264
C 7.615556 -0.488736 0.884265
C 7.257443 0.214914 2.187277
C 7.004241 -2.263271 -0.824416
H 1.552641 3.230052 -0.139962
H 2.496457 -1.539670 -0.638276
H 0.082993 -2.064825 -0.685908
H -2.260137 -2.638842 -0.749065
H -1.950556 3.197451 -0.173841
H -5.251861 1.513996 -1.405339
H -6.124159 -0.264071 0.776439
H -5.467314 1.325064 1.101953
H -4.678040 -1.372693 3.944134
H -6.128858 -0.451134 3.482818
H -5.683528 -1.944233 2.595055
H -6.394997 -1.610653 -1.710109
H -4.953069 -2.117563 -2.665417
H -5.983639 -3.320580 -1.871060
H 5.748502 1.924428 -0.813436
H 6.481248 0.527252 -1.614847
H 5.012058 1.210800 -2.265063
H 4.423825 -1.509323 0.875995
H 6.487417 -2.255268 1.226548
H 8.564245 -1.019865 1.035398
H 7.811592 0.262838 0.113646
H 7.064038 -0.509838 2.984484
H 8.085941 0.855146 2.507120
H 6.373393 0.849230 2.073409
H 6.262583 -3.014348 -1.118754
H 7.946938 -2.787861 -0.633388
H 7.164719 -1.597470 -1.677338
SCF Energy (B3LYP/6-31G**)= -1361.69861920
Number of imaginary frequencies = 0

1a_c049
MMFF Geometry
C 2.010309 1.695505 -0.053205
C 2.183044 -1.010532 -0.206172
N 3.237602 1.127628 0.026563
C 0.814772 0.990166 -0.208412
C 0.916100 -0.410231 -0.288533
C 3.321209 -0.220822 -0.048529
C -0.251624 -1.177656 -0.456587
C -1.514595 -0.573779 -0.529512
C -1.608113 0.816286 -0.434390
C -0.453678 1.599309 -0.290741
C -2.948637 1.435441 -0.494026
C -4.189226 0.556696 -0.245981
C -3.945028 -0.888109 -0.654760
C -2.703528 -1.397346 -0.760142
O -0.513713 2.970601 -0.222168
O -3.044477 2.651609 -0.688028
O -5.230710 1.121028 -1.060143
C -4.566801 0.729491 1.229552
C -5.629639 -0.233679 1.709971
C -7.051587 0.007833 1.274186
O -5.336733 -1.175208 2.450287
O -5.110229 -1.600818 -0.873120
C -5.024643 -2.992082 -1.153469
C 4.688061 -0.808744 0.060798
C 4.783262 -2.215678 0.586527
C 5.759928 -0.059560 -0.280838
C 7.220978 -0.450572 -0.265110
C 8.016012 0.373281 0.764649
C 7.588771 0.111049 2.203421
C 7.809874 -0.247183 -1.665287
H 2.014765 2.781246 0.014971
H 2.271137 -2.089539 -0.279377
H -0.168957 -2.261613 -0.531997
H -2.528881 -2.432535 -1.032226
H -1.431902 3.258992 -0.424895
H -5.054487 2.081262 -1.118206
H -4.931398 1.745640 1.427038
H -3.688609 0.587582 1.873616
H -7.721621 -0.641116 1.845498
H -7.326212 1.046564 1.474290
H -7.169294 -0.214540 0.212537
H -4.496806 -3.168771 -2.095970
H -4.546385 -3.529072 -0.328006
H -6.043138 -3.376771 -1.260560
H 4.530224 -2.937400 -0.197060
H 5.778519 -2.465243 0.962310
H 4.103312 -2.363493 1.433473
H 5.585191 0.961592 -0.627045
H 7.343628 -1.511154 -0.024820
H 9.082200 0.130210 0.674573
H 7.919696 1.445731 0.552752
H 7.667476 -0.953022 2.447845
H 8.234110 0.664867 2.892865
H 6.558412 0.432428 2.382887
H 7.265735 -0.839552 -2.409530
H 8.858817 -0.561787 -1.693101
H 7.766823 0.803894 -1.972893
SCF Energy (B3LYP/6-31G**)= -1361.71064053

Number of imaginary frequencies = 0

1a_c050
MMFF Geometry
C -1.623409 -2.372779 -0.081052
C -2.318743 0.252896 -0.020832
N -2.938225 -2.067477 0.041526
C -0.589696 -1.435624 -0.181353
C -0.962149 -0.079987 -0.147735
C -3.272445 -0.759572 0.062831
C 0.031401 0.910639 -0.241846
C 1.387422 0.574044 -0.368579
C 1.756102 -0.775360 -0.395475
C 0.773128 -1.775840 -0.309498
C 3.189550 -1.130027 -0.535632
C 4.257032 -0.019210 -0.532770
C 3.688440 1.382052 -0.571187
C 2.379181 1.643511 -0.467676
O 1.091359 -3.113686 -0.351055
O 3.505492 -2.314275 -0.702396
O 4.984560 -0.243087 -1.762131
C 5.243120 -0.231351 0.621078
C 4.640927 0.117182 1.962304
C 5.510586 0.879919 2.924867
O 3.505461 -0.243327 2.271324
O 4.579621 2.447888 -0.533477
C 5.231285 2.651427 -1.787503
C -4.719375 -0.466271 0.206236
C -5.411893 -1.314503 1.236284
C -5.298513 0.476975 -0.567782
C -6.742953 0.928583 -0.635516
C -7.202216 1.753166 0.581399
C -6.358707 2.999714 0.819764
C -7.717615 -0.213258 -0.939548
H -1.411604 -3.439785 -0.096138
H -2.627455 1.292655 0.026376
H -0.260857 1.959862 -0.213921
H 2.034533 2.672932 -0.429798
H 2.063858 -3.199659 -0.468518
H 5.142714 -1.207054 -1.812733
H 6.139558 0.381032 0.460606
H 5.595071 -1.268966 0.669354
H 4.958417 1.070051 3.849353
H 6.401565 0.291767 3.157594
H 5.794232 1.837915 2.482056
H 6.100124 1.993211 -1.876052
H 4.549015 2.512047 -2.633444
H 5.593286 3.683714 -1.810637
H -4.807317 -1.389225 2.147812
H -6.374509 -0.905609 1.546401
H -5.583699 -2.325444 0.851670
H -4.672435 0.994955 -1.296112
H -6.800365 1.598181 -1.506623
H -8.241408 2.069078 0.424568
H -7.199871 1.144868 1.491901
H -6.339585 3.636772 -0.070172
H -6.778762 3.584298 1.644619
H -5.328495 2.743801 1.085340
H -7.367630 -0.816146 -1.784759
H -8.700871 0.189899 -1.207226
H -7.864645 -0.878885 -0.084989
SCF Energy (B3LYP/6-31G**)= -1361.69869776
Number of imaginary frequencies = 0

1a_c051
MMFF Geometry
C -1.435213 -2.279402 -0.686897
C -2.365361 0.240690 -0.272149
N -2.775945 -2.103500 -0.597454
C -0.486754 -1.257117 -0.582697
C -0.980118 0.044310 -0.370687
C -3.226649 -0.849375 -0.381159
C -0.077333 1.119474 -0.272164
C 1.306111 0.915856 -0.364260
C 1.787705 -0.382028 -0.557157
C 0.903672 -1.461935 -0.685210
C 3.247233 -0.570966 -0.660799
C 4.174626 0.513355 -0.078842
C 3.549425 1.898922 -0.190328
C 2.218198 2.063686 -0.312415
O 1.350085 -2.740163 -0.917799
O 3.696004 -1.585143 -1.202782
O 5.382902 0.488432 -0.860778
C 4.547476 0.244486 1.381063
C 4.795206 -1.219177 1.655234
C 6.176005 -1.760140 1.398440
O 3.876712 -1.945991 2.036646
O 4.471138 2.926804 -0.102332
C 4.023002 4.264632 -0.278443
C -4.698948 -0.700642 -0.293315
C -5.464169 -1.508056 -1.307051
C -5.252806 0.086503 0.653787
C -6.725683 0.320571 0.911947
C -7.111656 1.792621 0.676570
C -6.951338 2.230887 -0.773896
C -7.054580 -0.083852 2.352807
H -1.128692 -3.310201 -0.852368
H -2.770365 1.237328 -0.126405
H -0.465351 2.126594 -0.123579
H 1.762258 3.046144 -0.365883
H 2.324893 -2.720817 -1.042068
H 5.361413 -0.331879 -1.393131
H 3.742575 0.555164 2.060567
H 5.443432 0.803683 1.677477
H 6.251487 -2.773501 1.802824
H 6.371248 -1.790633 0.324769
H 6.917776 -1.133523 1.899770
H 3.560439 4.397304 -1.261808
H 3.334196 4.553541 0.521733
H 4.895834 4.921965 -0.224572
H -5.508394 -2.560378 -1.007176
H -6.487867 -1.152985 -1.448280
H -4.986269 -1.448311 -2.291980
H -4.597454 0.603579 1.355254
H -7.344929 -0.303417 0.260132
H -8.159834 1.940716 0.965230
H -6.513243 2.452190 1.317808
H -7.530793 1.587836 -1.443925
H -7.309924 3.257909 -0.896937
H -5.903912 2.205159 -1.089263
H -6.801094 -1.134666 2.532782
H -8.124322 0.038810 2.554331
H -6.504533 0.525543 3.078962
SCF Energy (B3LYP/6-31G**)= -1361.70542308
Number of imaginary frequencies = 0

1a_c052
MMFF Geometry
C 1.634414 -2.196572 -0.378599
C 2.467265 0.385128 -0.547992
N 2.966268 -1.963209 -0.468053
C 0.648559 -1.204935 -0.367356
C 1.091286 0.128119 -0.459237
C 3.369741 -0.676628 -0.541018
C 0.146970 1.171363 -0.469910
C -1.225945 0.907709 -0.371222
C -1.656953 -0.415815 -0.261453
C -0.732688 -1.470264 -0.278748
C -3.109673 -0.668473 -0.159660
C -4.008123 0.473815 0.373951
C -3.485896 1.824998 -0.123881
C -2.191103 2.006886 -0.448208
O -1.133439 -2.783540 -0.214960
O -3.523839 -1.789010 -0.465049
O -3.916369 0.527995 1.801034
C -5.464126 0.233758 -0.075704
C -6.054727 -1.023983 0.531072
C -7.062456 -1.775296 -0.295686
O -5.763284 -1.380576 1.675017
O -4.451176 2.817633 -0.156771
C -4.044498 4.156345 -0.414624
C 4.834038 -0.466330 -0.637892

C 5.540616 -1.434799 -1.547737
C 5.435510 0.507061 0.079020
C 6.913557 0.827000 0.139575
C 7.443494 0.771821 1.585618
C 7.369427 -0.621707 2.197729
C 7.150675 2.217908 -0.454218
H 1.367659 -3.249340 -0.313519
H 2.831065 1.404189 -0.635584
H 0.492132 2.201027 -0.555218
H -1.805057 2.966132 -0.773109
H -2.114471 -2.814949 -0.280731
H -4.411716 -0.245434 2.149300
H -5.514685 0.157586 -1.168507
H -6.126733 1.046566 0.244947
H -7.397671 -2.662376 0.248720
H -7.923460 -1.132792 -0.494929
H -6.602531 -2.093383 -1.234557
H -3.319357 4.497325 0.331371
H -3.642158 4.253200 -1.427978
H -4.929055 4.795993 -0.341937
H 4.965378 -1.595117 -2.467216
H 6.522858 -1.077928 -1.866996
H 5.675224 -2.401339 -1.050763
H 4.825351 1.130969 0.732806
H 7.496655 0.113756 -0.451109
H 8.491649 1.096158 1.597492
H 6.890758 1.473188 2.223442
H 7.908571 -1.349081 1.582484
H 7.823749 -0.618324 3.193748
H 6.334388 -0.959754 2.305820
H 6.801701 2.266072 -1.491879
H 8.218245 2.463281 -0.451533
H 6.626314 2.994168 0.114718
SCF Energy (B3LYP/6-31G**)= -1361.71342223
Number of imaginary frequencies = 0

1a_c053
MMFF Geometry
C -1.854543 -1.509115 -0.361252
C -2.033546 1.157050 0.114496
N -3.084387 -0.978144 -0.155638
C -0.660149 -0.784247 -0.350205
C -0.765359 0.594411 -0.105465
C -3.170264 0.350430 0.084160
C 0.401538 1.379403 -0.088706
C 1.669153 0.815442 -0.307779
C 1.771166 -0.558364 -0.543805
C 0.611553 -1.354152 -0.569093
C 3.107838 -1.159765 -0.772590
C 4.362819 -0.260199 -0.715685
C 4.088034 1.214921 -0.470894
C 2.844123 1.689592 -0.286857
O 0.662634 -2.707627 -0.812226
O 3.166372 -2.367664 -1.025294
O 4.987616 -0.365392 -2.014301
C 5.364140 -0.863766 0.279199
C 4.898094 -0.825350 1.717923
C 5.921948 -1.195902 2.758995
O 3.745097 -0.539587 2.038259
O 5.238145 1.985278 -0.468149
C 5.126849 3.381684 -0.219378
C -4.537936 0.895125 0.325151
C -4.638167 2.145246 1.157198
C -5.611043 0.240320 -0.171391
C -7.068366 0.629563 -0.061289
C -7.799173 -0.184872 1.023635
C -7.866621 -1.688308 0.783799
C -7.747727 0.513096 -1.429610
H -1.855001 -2.581688 -0.543265
H -2.122983 2.223090 0.294779
H 0.314895 2.448545 0.102380
H 2.655332 2.742147 -0.107535
H 1.604242 -2.964494 -0.944087
H 4.483320 0.197537 -2.625382
H 6.323524 -0.334850 0.214120
H 5.574202 -1.910653 0.025608
H 5.475835 -1.129716 3.755203
H 6.261293 -2.220744 2.590013
H 6.766298 -0.504288 2.705234
H 4.532988 3.871192 -0.997834
H 4.706195 3.569693 0.773661
H 6.133636 3.808914 -0.245203
H -4.391512 3.027819 0.557970
H -5.635173 2.294554 1.580483
H -3.957143 2.100647 2.014885
H -5.436917 -0.678636 -0.734873
H -7.148937 1.685279 0.221965
H -7.313365 -0.009585 1.992189
H -8.823826 0.195447 1.121711
H -6.869014 -2.132680 0.720867
H -8.392471 -2.172722 1.613285
H -8.412173 -1.924787 -0.134183
H -7.282769 1.195302 -2.150576
H -8.808380 0.777029 -1.359281
H -7.676448 -0.498856 -1.842324
SCF Energy (B3LYP/6-31G**)= -1361.70724776
Number of imaginary frequencies = 0

1a_c054
MMFF Geometry
C 1.991376 1.848987 -0.145494
C 2.222633 -0.808336 -0.656209
N 3.235353 1.317282 -0.215725
C 0.806582 1.129067 -0.315426
C 0.938033 -0.246034 -0.579187
C 3.348271 -0.006762 -0.470774
C -0.218489 -1.026208 -0.765111
C -1.498552 -0.461201 -0.679475
C -1.620867 0.902509 -0.404889
C -0.479695 1.700337 -0.238382
C -2.977672 1.478635 -0.299695
C -4.175881 0.534680 -0.085261
C -3.923063 -0.839882 -0.685906
C -2.678998 -1.289978 -0.933794
O -0.569950 3.049541 0.007113
O -3.117360 2.705552 -0.332476
O -5.283583 1.159609 -0.754978
C -4.457875 0.511874 1.421250
C -5.462822 -0.536786 1.843816
C -6.916013 -0.289682 1.531797
O -5.098703 -1.552153 2.441121
O -5.081764 -1.559513 -0.915857
C -4.979795 -2.902824 -1.370450
C 4.734613 -0.551329 -0.554964
C 4.932890 -1.784354 -1.394581
C 5.736444 0.085345 0.091240
C 7.197055 -0.300394 0.164058
C 7.682375 -0.395095 1.623976
C 6.992900 -1.501067 2.413680
C 8.029544 0.731836 -0.600256
H 1.972634 2.916980 0.060768
H 2.332456 -1.870872 -0.846875
H -0.113471 -2.089163 -0.980938
H -2.496900 -2.277470 -1.343479
H -1.506797 3.329726 -0.098493
H -5.136806 2.125099 -0.702478
H -4.832740 1.483648 1.767125
H -3.535342 0.319940 1.985405
H -7.530796 -1.023917 2.060400
H -7.201428 0.707950 1.874860
H -7.098869 -0.385680 0.460464
H -4.512967 -2.945965 -2.359565
H -4.432849 -3.519494 -0.649999
H -5.993015 -3.305876 -1.458330
H 4.356591 -1.721655 -2.324867
H 5.971611 -1.927829 -1.702637
H 4.618138 -2.678182 -0.846014
H 5.494452 0.989135 0.654805
H 7.370875 -1.276081 -0.300311
H 8.762171 -0.589900 1.632407
H 7.532084 0.561289 2.140703
H 7.121096 -2.471169 1.923027

H 7.424474 -1.570006 3.417405
H 5.921514 -1.308691 2.525062
H 7.714357 0.791836 -1.648055
H 9.090479 0.459472 -0.588175
H 7.934497 1.731582 -0.161298
SCF Energy (B3LYP/6-31G**)= -1361.71067105
Number of imaginary frequencies = 0

1a_c055
MMFF Geometry
C -1.723604 -1.399737 -0.658831
C -2.104042 1.147613 0.203176
N -2.992168 -0.988887 -0.417314
C -0.585403 -0.606262 -0.498952
C -0.793944 0.711827 -0.053908
C -3.178712 0.279541 0.014307
C 0.311538 1.566888 0.114213
C 1.617165 1.123612 -0.136959
C 1.816500 -0.193450 -0.561156
C 0.725908 -1.050915 -0.761479
C 3.201562 -0.640342 -0.821044
C 4.350395 0.136374 -0.135446
C 4.015453 1.623089 -0.032266
C 2.741334 2.058160 -0.017878
O 0.889781 -2.335432 -1.220451
O 3.376748 -1.620922 -1.546308
O 5.540608 -0.017094 -0.920068
C 4.651993 -0.419075 1.258289
C 4.617967 -1.929239 1.294227
C 5.813700 -2.678515 0.769969
O 3.619139 -2.517389 1.712833
O 5.133102 2.431016 0.065626
C 4.953210 3.836300 0.197456
C -4.587497 0.687855 0.287182
C -4.794762 1.779125 1.302380
C -5.595570 0.053425 -0.351644
C -7.082449 0.308078 -0.242777
C -7.819674 -0.897635 0.368752
C -7.425394 -1.173429 1.814383
C -7.642706 0.612381 -1.636386
H -1.643245 -2.430191 -0.998657
H -2.275551 2.166087 0.535308
H 0.144383 2.591969 0.443013
H 2.489056 3.108768 0.076034
H 1.839099 -2.476670 -1.437709
H 5.420804 0.515263 -1.725692
H 3.914287 -0.070412 1.993203
H 5.635552 -0.094242 1.619570
H 5.756382 -3.723081 1.089004
H 5.831259 -2.639652 -0.320891
H 6.730560 -2.245898 1.178027
H 4.444447 4.248947 -0.679596
H 4.408938 4.075921 1.116539
H 5.943075 4.297650 0.261453
H -4.593426 2.760123 0.859641
H -5.808800 1.797455 1.709153
H -4.133699 1.640839 2.165730
H -5.339444 -0.745608 -1.051122
H -7.288462 1.186662 0.376337
H -8.900693 -0.712092 0.338331
H -7.639192 -1.799746 -0.229535
H -7.587940 -0.290213 2.440237
H -8.031516 -1.990474 2.218812
H -6.374761 -1.467628 1.896537
H -7.140054 1.478920 -2.080614
H -8.712565 0.841355 -1.581566
H -7.516130 -0.237270 -2.317053
SCF Energy (B3LYP/6-31G**)= -1361.70193143
Number of imaginary frequencies = 0

1a_c056
MMFF Geometry
C 1.504578 -2.137863 0.344528
C 2.299629 0.342448 -0.432786
N 2.814355 -1.953516 0.049700
C 0.523667 -1.143404 0.279612
C 0.946572 0.136781 -0.125772
C 3.199974 -0.715947 -0.327980
C 0.007750 1.182001 -0.208755
C -1.343922 0.968772 0.093555
C -1.757104 -0.308817 0.481714
C -0.833364 -1.356785 0.593642
C -3.182946 -0.507504 0.804324
C -4.211762 0.496288 0.249016
C -3.617633 1.895001 0.131888
C -2.287491 2.091284 0.051753
O -1.207597 -2.611119 1.010552
O -3.524413 -1.466224 1.502853
O -5.301441 0.535372 1.188712
C -4.771398 0.070818 -1.110566
C -5.011416 -1.417009 -1.198031
C -6.328231 -1.948634 -0.699462
O -4.132530 -2.165985 -1.626959
O -4.572182 2.894288 0.067857
C -4.143291 4.249654 0.042409
C 4.639596 -0.559885 -0.645322
C 5.228771 -1.702696 -1.427723
C 5.323413 0.521915 -0.214836
C 6.796200 0.815308 -0.403437
C 7.504876 1.034057 0.947436
C 7.513473 -0.209953 1.827467
C 6.951653 2.056808 -1.285445
H 1.251768 -3.152181 0.646392
H 2.646225 1.316515 -0.763775
H 0.342697 2.173690 -0.510894
H -1.857202 3.080562 -0.058406
H -2.156057 -2.592894 1.267756
H -5.184168 -0.224458 1.793424
H -4.075577 0.320730 -1.922657
H -5.715171 0.582608 -1.335648
H -6.428433 -2.999722 -0.984592
H -6.374885 -1.869637 0.388381
H -7.149116 -1.388944 -1.154544
H -3.555394 4.492011 0.933594
H -3.578331 4.462689 -0.870630
H -5.034053 4.884768 0.042692
H 4.542102 -2.027922 -2.218139
H 6.161081 -1.436111 -1.931533
H 5.429230 -2.555706 -0.770845
H 4.798652 1.274952 0.373625
H 7.302354 -0.012687 -0.909268
H 8.545025 1.331536 0.764243
H 7.034849 1.858997 1.497709
H 7.972982 -1.055643 1.305993
H 8.090029 -0.020645 2.738631
H 6.501544 -0.497582 2.128355
H 6.474151 1.907552 -2.260410
H 8.010162 2.274677 -1.464360
H 6.500791 2.940987 -0.820502
SCF Energy (B3LYP/6-31G**)= -1361.70560380
Number of imaginary frequencies = 0

1a_c057
MMFF Geometry
C -1.519026 -2.349861 -0.295741
C -2.488833 0.186392 -0.188116
N -2.860690 -2.178652 -0.379426
C -0.588080 -1.315379 -0.157322
C -1.102060 -0.006884 -0.104168
C -3.332551 -0.914975 -0.317041
C -0.216658 1.076787 0.034916
C 1.168848 0.877191 0.111744
C 1.677797 -0.422109 0.051140
C 0.805192 -1.515703 -0.069769
C 3.143865 -0.619297 0.150035
C 4.088107 0.580924 -0.119465
C 3.381741 1.900466 0.163102
C 2.054148 2.022346 0.293421
O 1.266352 -2.810749 -0.106125
O 3.553455 -1.755402 0.405070
O 4.425335 0.600207 -1.508830
C 5.341065 0.438562 0.770158

C 6.195221 -0.753406 0.385348
C 6.896521 -1.483315 1.498355
O 6.350714 -1.072434 -0.795512
O 4.167692 3.029860 0.364923
C 4.685492 3.533270 -0.868951
C -4.804776 -0.772811 -0.417348
C -5.445418 -1.667666 -1.444195
C -5.468970 0.084503 0.387165
C -6.964697 0.316877 0.447756
C -7.280462 1.727488 -0.081400
C -8.771736 2.018906 -0.178121
C -7.447272 0.112860 1.887233
H -1.196462 -3.387697 -0.346430
H -2.905654 1.188592 -0.167175
H -0.616808 2.089118 0.082472
H 1.614729 2.990508 0.515280
H 2.236013 -2.803178 0.060448
H 5.042850 -0.149045 -1.663523
H 5.046724 0.339160 1.822074
H 6.003889 1.307499 0.684888
H 7.463147 -2.323698 1.088065
H 7.584943 -0.802563 2.004801
H 6.158488 -1.870136 2.205372
H 5.517600 2.916674 -1.221002
H 3.905610 3.604683 -1.635135
H 5.071092 4.539882 -0.681818
H -5.542658 -2.688017 -1.058695
H -6.437195 -1.322062 -1.746820
H -4.845646 -1.699570 -2.361436
H -4.903401 0.669505 1.112928
H -7.493990 -0.413640 -0.172161
H -6.809977 2.488039 0.554807
H -6.845687 1.844198 -1.082728
H -9.240056 2.050425 0.809837
H -8.933100 2.993160 -0.650850
H -9.282043 1.261605 -0.781469
H -7.139758 -0.867890 2.268045
H -8.539313 0.154957 1.947918
H -7.041651 0.877188 2.559658
SCF Energy (B3LYP/6-31G**)= -1361.70693568
Number of imaginary frequencies = 0

1a_c058
MMFF Geometry
C -1.694349 -1.390026 -1.003545
C -2.136843 1.156709 -0.169800
N -2.977221 -0.978131 -0.858733
C -0.570858 -0.598181 -0.755767
C -0.811364 0.718876 -0.323984
C -3.194866 0.290533 -0.442692
C 0.279062 1.570186 -0.063712
C 1.599590 1.124976 -0.212887
C 1.829220 -0.190360 -0.626993
C 0.756206 -1.044514 -0.916484
C 3.229572 -0.639234 -0.779052
C 4.322156 0.130902 -0.000436
C 3.983052 1.617834 0.084527
C 2.712707 2.056015 0.000285
O 0.953337 -2.327641 -1.366149
O 3.459544 -1.616270 -1.493715
O 5.570352 -0.020996 -0.689436
C 4.511490 -0.433150 1.409525
C 4.472919 -1.943459 1.433611
C 5.705651 -2.691625 1.001565
O 3.443276 -2.532456 1.767865
O 5.091043 2.422401 0.275601
C 4.904002 3.827213 0.402368
C -4.620086 0.710105 -0.308006
C -4.915990 2.177627 -0.459222
C -5.567608 -0.227662 -0.086008
C -7.054335 -0.028636 0.106800
C -7.545217 -0.682140 1.413624
C -6.946494 -0.046705 2.662598
C -7.801576 -0.621860 -1.090045
H -1.589352 -2.419881 -1.338435
H -2.331898 2.166648 0.175188
H 0.088455 2.593920 0.256406
H 2.455945 3.106695 0.080136
H 1.916480 -2.469896 -1.509216
H 5.515589 0.516061 -1.498970
H 3.718201 -0.087528 2.085622
H 5.463675 -0.112097 1.849632
H 5.622252 -3.737778 1.309454
H 5.809476 -2.647011 -0.084265
H 6.587791 -2.262475 1.483187
H 4.467164 4.246945 -0.509408
H 4.289056 4.062068 1.277040
H 5.886575 4.285673 0.547753
H -4.334692 2.611889 -1.280800
H -5.962393 2.378452 -0.702486
H -4.675746 2.715763 0.463604
H -5.253471 -1.271613 -0.016916
H -7.305955 1.035152 0.160612
H -8.637163 -0.592110 1.473021
H -7.318469 -1.755849 1.413999
H -7.152284 1.028017 2.692497
H -7.381907 -0.499554 3.559157
H -5.863091 -0.192845 2.709114
H -7.482894 -0.148221 -2.025424
H -8.880933 -0.464386 -0.989544
H -7.626528 -1.699977 -1.180766
SCF Energy (B3LYP/6-31G**)= -1361.70200130
Number of imaginary frequencies = 0

1a_c059
MMFF Geometry
C 1.559752 -2.297883 0.150391
C 2.460779 0.161859 -0.572308
N 2.896818 -2.126714 0.011246
C 0.600676 -1.300055 -0.050897
C 1.079094 -0.031847 -0.427626
C 3.334504 -0.898066 -0.338398
C 0.163412 1.010443 -0.657347
C -1.215827 0.812387 -0.503060
C -1.688195 -0.444407 -0.118035
C -0.787723 -1.500857 0.094228
C -3.150312 -0.643528 0.025444
C -4.070315 0.595160 0.180539
C -3.433241 1.825778 -0.451709
C -2.138251 1.906960 -0.784534
O -1.216363 -2.759661 0.444679
O -3.570430 -1.803712 0.057131
O -4.214116 0.892512 1.571692
C -5.430674 0.291391 -0.481837
C -6.202159 -0.792688 0.244816
C -7.036593 -1.721473 -0.594500
O -6.188015 -0.870414 1.475321
O -4.259795 2.899959 -0.762994
C -4.613078 3.643149 0.406126
C 4.803245 -0.755087 -0.481690
C 5.474995 -1.916439 -1.163892
C 5.437029 0.334089 0.003525
C 6.924764 0.619158 -0.013592
C 7.414393 0.856881 1.428180
C 8.924033 1.024179 1.535841
C 7.192721 1.819416 -0.924270
H 1.265411 -3.304282 0.440645
H 2.850644 1.127740 -0.878376
H 0.534976 1.989541 -0.958186
H -1.750820 2.807633 -1.251798
H -2.199707 -2.775483 0.416006
H -4.790761 0.194548 1.955057
H -5.281674 -0.017061 -1.523806
H -6.091198 1.166235 -0.479033
H -7.526160 -2.458661 0.047782
H -7.800287 -1.147708 -1.124972
H -6.395959 -2.247523 -1.306578
H -5.377496 3.116353 0.984645
H -3.737090 3.858320 1.027976
H -5.039351 4.595952 0.078358
H 4.885616 -2.256600 -2.023552
H 6.462006 -1.663316 -1.558839

H 5.590955 -2.755772 -0.470123
H 4.849055 1.103424 0.505071
H 7.478016 -0.235901 -0.413996
H 6.927917 1.742894 1.855812
H 7.120663 0.004725 2.054972
H 9.263636 1.937712 1.039386
H 9.219281 1.091263 2.587989
H 9.446993 0.171451 1.091662
H 6.773637 1.654196 -1.923535
H 8.266570 1.989988 -1.049135
H 6.748980 2.736738 -0.521147
SCF Energy (B3LYP/6-31G**)= -1361.70706867
Number of imaginary frequencies = 0

1a_c060
MMFF Geometry
C 1.609482 2.437396 -0.266309
C 2.374041 -0.169264 -0.280500
N 2.936879 2.165111 -0.236951
C 0.595482 1.474587 -0.307212
C 1.004004 0.129630 -0.317809
C 3.305149 0.866203 -0.232729
C 0.031892 -0.884746 -0.367523
C -1.338153 -0.582749 -0.405284
C -1.744551 0.755775 -0.386363
C -0.781509 1.779708 -0.341594
C -3.194184 1.079179 -0.434492
C -4.227884 -0.071656 -0.506907
C -3.620199 -1.459705 -0.514994
C -2.301824 -1.679954 -0.447631
O -1.133165 3.110028 -0.338916
O -3.526309 2.268187 -0.476544
O -4.925890 0.107197 -1.758079
C -5.271422 0.119829 0.601837
C -4.713858 -0.083236 1.993933
C -5.715241 -0.371116 3.081267
O -3.515028 0.026989 2.248406
O -4.471380 -2.554916 -0.425276
C -5.236162 -2.770820 -1.610070
C 4.765100 0.610980 -0.200377
C 5.572371 1.520265 -1.087146
C 5.274447 -0.352608 0.596754
C 6.730645 -0.720100 0.783813
C 7.014475 -2.156853 0.307904
C 6.810753 -2.344382 -1.190416
C 7.100182 -0.575259 2.263767
H 1.370306 3.498721 -0.256702
H 2.711697 -1.200796 -0.302086
H 0.352462 -1.926196 -0.372000
H -1.927357 -2.698212 -0.388047
H -2.115647 3.173128 -0.348310
H -4.254020 0.153174 -2.460394
H -6.104780 -0.578589 0.455030
H -5.707409 1.125771 0.560577
H -5.196872 -0.500291 4.035418
H -6.413172 0.465305 3.166418
H -6.255159 -1.292120 2.847905
H -6.111693 -2.115770 -1.626401
H -4.631978 -2.636650 -2.513998
H -5.594543 -3.804407 -1.592027
H 5.689083 2.505703 -0.623942
H 6.568934 1.127942 -1.303759
H 5.082173 1.650263 -2.059177
H 4.592660 -0.932702 1.219502
H 7.383960 -0.040678 0.227703
H 8.053130 -2.417067 0.547526
H 6.379451 -2.870979 0.847428
H 7.425455 -1.640645 -1.760607
H 7.098294 -3.359187 -1.483653
H 5.764358 -2.200438 -1.475998
H 6.919701 0.447014 2.614827
H 8.161194 -0.797808 2.421032
H 6.517603 -1.256763 2.894137
SCF Energy (B3LYP/6-31G**)= -1361.70144271
Number of imaginary frequencies = 0

1a_c061
MMFF Geometry
C -1.669006 -2.216630 -0.374041
C -2.304943 0.286891 0.467145
N -2.965799 -1.956663 -0.077990
C -0.625676 -1.289378 -0.282597
C -0.967345 0.002156 0.154579
C -3.271680 -0.707430 0.332917
C 0.037988 0.978049 0.270363
C 1.375047 0.690917 -0.045127
C 1.715318 -0.595882 -0.475843
C 0.718497 -1.580523 -0.596933
C 3.128703 -0.900887 -0.818850
C 4.198251 0.212207 -0.695195
C 3.662538 1.544489 -0.211375
C 2.376674 1.744292 0.101349
O 1.003402 -2.855354 -1.029426
O 3.394596 -2.026287 -1.253116
O 4.716184 0.407573 -2.028676
C 5.368102 -0.306945 0.151501
C 5.008939 -0.521380 1.605816
C 6.157081 -0.572571 2.578990
O 3.850335 -0.696023 1.981641
O 4.570027 2.557623 0.075113
C 5.182097 3.109657 -1.089307
C -4.699083 -0.467201 0.653168
C -5.363305 -1.590991 1.402222
C -5.309836 0.668855 0.253689
C -6.760648 1.052476 0.450013
C -7.450024 1.356965 -0.894228
C -7.537850 0.142697 -1.810705
C -6.836710 2.274933 1.368293
H -1.480778 -3.237091 -0.701043
H -2.587089 1.271849 0.825945
H -0.229720 1.976261 0.615772
H 2.062956 2.701633 0.508421
H 1.971586 -2.919197 -1.196387
H 3.956264 0.575493 -2.612709
H 6.207751 0.397455 0.100159
H 5.745675 -1.258652 -0.242698
H 5.775510 -0.728464 3.591856
H 6.820987 -1.400277 2.318049
H 6.703243 0.373568 2.551676
H 6.015226 2.482603 -1.418867
H 4.458647 3.254006 -1.899189
H 5.589248 4.088761 -0.819775
H -4.701568 -1.982040 2.184035
H -6.278029 -1.278962 1.912130
H -5.616453 -2.410454 0.721268
H -4.735197 1.402977 -0.311646
H -7.321247 0.244864 0.930770
H -8.468916 1.716458 -0.702948
H -6.925562 2.165190 -1.419551
H -8.053124 -0.686056 -1.314785
H -8.098417 0.396196 -2.716230
H -6.546170 -0.201667 -2.119332
H -6.372508 2.066002 2.338710
H -7.879131 2.556339 1.552923
H -6.327716 3.140991 0.930013
SCF Energy (B3LYP/6-31G**)= -1361.70154661
Number of imaginary frequencies = 0

1a_c062
MMFF Geometry
C -1.461082 -2.189609 -0.842131
C -2.398184 0.286684 -0.227698
N -2.803029 -2.021196 -0.756358
C -0.514474 -1.180531 -0.639021
C -1.011711 0.098575 -0.324215
C -3.257317 -0.789672 -0.440273
C -0.111554 1.161002 -0.122332
C 1.272427 0.964800 -0.214232
C 1.760257 -0.312975 -0.509941
C 0.877656 -1.377303 -0.739505
C 3.223760 -0.496472 -0.613625
C 4.152964 0.544453 0.042283

C 3.508551 1.917532 0.034625
C 2.181321 2.099501 -0.044674
O 1.326470 -2.632555 -1.072031
O 3.677678 -1.471400 -1.220427
O 5.348706 0.590309 -0.755743
C 4.541315 0.171843 1.474931
C 4.789544 -1.307569 1.643381
C 6.178697 -1.821979 1.377609
O 3.867394 -2.064706 1.948733
O 4.344347 2.993028 0.295230
C 4.691506 3.679455 -0.907954
C -4.730767 -0.648208 -0.359504
C -5.482223 -1.367139 -1.447401
C -5.297442 0.056853 0.643060
C -6.773655 0.269321 0.900749
C -7.157259 1.756166 0.784784
C -6.978259 2.314569 -0.621551
C -7.121109 -0.254449 2.298158
H -1.151944 -3.202931 -1.090031
H -2.805346 1.267682 -0.002973
H -0.500658 2.152313 0.107886
H 1.758140 3.096441 0.037212
H 2.303687 -2.606505 -1.173282
H 5.373307 -0.240740 -1.272455
H 3.744694 0.433324 2.184292
H 5.440840 0.709142 1.799643
H 6.243086 -2.871372 1.678830
H 6.408246 -1.743744 0.313179
H 6.902512 -1.249980 1.963359
H 5.180019 3.013666 -1.626150
H 3.807915 4.140632 -1.361936
H 5.395006 4.476020 -0.648636
H -5.529504 -2.440961 -1.237795
H -6.504295 -1.001137 -1.571445
H -4.991709 -1.224822 -2.417547
H -4.651588 0.512998 1.393919
H -7.384042 -0.297366 0.190832
H -8.209185 1.880130 1.071171
H -6.567586 2.359211 1.486880
H -7.548603 1.730387 -1.350701
H -7.335727 3.348508 -0.662594
H -5.926797 2.314788 -0.924282
H -6.869475 -1.316817 2.392559
H -8.193448 -0.148493 2.495310
H -6.580876 0.291494 3.080019
SCF Energy (B3LYP/6-31G**)= -1361.70039855
Number of imaginary frequencies = 0

1a_c063
MMFF Geometry
C 1.990087 1.708109 -0.027995
C 2.149290 -0.994005 -0.253929
N 3.213636 1.131181 0.045126
C 0.792189 1.013932 -0.211622
C 0.886312 -0.384708 -0.329818
C 3.290360 -0.215160 -0.065840
C -0.283842 -1.141033 -0.529790
C -1.542096 -0.527728 -0.594593
C -1.628927 0.860337 -0.458981
C -0.471727 1.632705 -0.286679
C -2.968940 1.487972 -0.506089
C -4.195056 0.596353 -0.202600
C -3.970219 -0.818012 -0.726319
C -2.734915 -1.331491 -0.873229
O -0.526980 3.001881 -0.183075
O -3.035795 2.698229 -0.728899
O -5.336754 1.175468 -0.844486
C -4.426478 0.662803 1.311502
C -5.429224 -0.353626 1.808927
C -6.891246 -0.076503 1.575144
O -5.055075 -1.382128 2.377283
O -5.145695 -1.489144 -1.009210
C -5.085147 -2.889895 -1.254805
C 4.653014 -0.813602 0.038094
C 4.736510 -2.233631 0.529399
C 5.731413 -0.062297 -0.277265
C 7.190104 -0.461932 -0.260945
C 7.982281 0.331538 0.794552
C 7.543236 0.036170 2.223314
C 7.790177 -0.227252 -1.651444
H 1.999574 2.791549 0.070334
H 2.232457 -2.071061 -0.355731
H -0.206493 -2.222670 -0.636560
H -2.567226 -2.343492 -1.225442
H -1.441912 3.298061 -0.395469
H -5.258867 0.979179 -1.794521
H -4.781907 1.656432 1.612665
H -3.493004 0.491531 1.863641
H -7.491513 -0.743351 2.200745
H -7.122470 0.954325 1.854957
H -7.145698 -0.248987 0.527922
H -4.550234 -3.098534 -2.186597
H -4.627173 -3.417062 -0.411547
H -6.110095 -3.255835 -1.365795
H 4.485953 -2.934271 -0.273868
H 5.727404 -2.497844 0.906717
H 4.048981 -2.398717 1.366985
H 5.564767 0.968224 -0.598889
H 7.305087 -1.528843 -0.046214
H 9.047718 0.084645 0.705997
H 7.893477 1.409450 0.608687
H 7.614252 -1.034070 2.441786
H 8.186667 0.569004 2.930841
H 6.513417 0.358915 2.403428
H 7.248126 -0.797850 -2.413997
H 8.837530 -0.547074 -1.679667
H 7.755174 0.831360 -1.933138
SCF Energy (B3LYP/6-31G**)= -1361.70454393
Number of imaginary frequencies = 0

1a_c064
MMFF Geometry
C -1.580105 -2.405423 -0.259293
C -2.565683 0.126620 -0.214817
N -2.922404 -2.244500 -0.352761
C -0.656069 -1.362280 -0.140862
C -1.178191 -0.056096 -0.120361
C -3.402138 -0.982670 -0.321680
C -0.300056 1.035976 -0.002650
C 1.086270 0.846780 0.084839
C 1.603372 -0.450412 0.056665
C 0.737986 -1.551897 -0.042606
C 3.070151 -0.636185 0.166508
C 4.008286 0.563272 -0.126680
C 3.292648 1.884627 0.122060
C 1.963766 2.001292 0.243716
O 1.207168 -2.844561 -0.046856
O 3.485487 -1.763513 0.449651
O 4.351654 0.552386 -1.514632
C 5.258061 0.449344 0.771476
C 6.121173 -0.745932 0.418202
C 6.821869 -1.445464 1.550922
O 6.283929 -1.091313 -0.754235
O 4.070804 3.023237 0.301009
C 4.591095 3.501060 -0.941957
C -4.874500 -0.851775 -0.431775
C -5.506869 -1.774712 -1.438774
C -5.548119 0.024271 0.344230
C -7.045197 0.243652 0.385997
C -7.455756 1.525322 -0.364601
C -6.902930 2.826610 0.204163
C -7.536625 0.238641 1.836993
H -1.251014 -3.442142 -0.284491
H -2.988574 1.126510 -0.219134
H -0.706573 2.046641 0.019627
H 1.517475 2.971609 0.441097
H 2.176006 -2.827131 0.123725
H 4.974405 -0.196423 -1.649173
H 4.959588 0.372565 1.824122
H 5.915969 1.320147 0.668934
H 7.395438 -2.291624 1.162749
H 7.503848 -0.748899 2.044417

H 6.083018 -1.820327 2.263505
H 5.428514 2.881614 -1.275944
H 3.814257 3.549846 -1.712993
H 4.969721 4.514109 -0.776587
H -5.596106 -2.787040 -1.030886
H -6.501760 -1.443267 -1.748089
H -4.906543 -1.821587 -2.354956
H -4.988890 0.630970 1.056353
H -7.556756 -0.591495 -0.105802
H -7.140716 1.443435 -1.412899
H -8.550984 1.592149 -0.378216
H -5.809050 2.836688 0.200904
H -7.242920 3.671094 -0.404502
H -7.250149 2.997719 1.227096
H -7.338498 -0.731004 2.308048
H -8.616778 0.414486 1.880840
H -7.043073 1.005232 2.443783
SCF Energy (B3LYP/6-31G**)= -1361.70594173
Number of imaginary frequencies = 0

1a_c065
MMFF Geometry
C 1.525652 -2.078433 0.508707
C 2.334273 0.315865 -0.492000
N 2.839811 -1.922720 0.216821
C 0.546553 -1.095374 0.334458
C 0.976853 0.140161 -0.185883
C 3.232005 -0.727080 -0.272813
C 0.040908 1.172169 -0.382892
C -1.314275 0.988310 -0.079507
C -1.737195 -0.246907 0.423964
C -0.815439 -1.278764 0.647705
C -3.169707 -0.416940 0.748283
C -4.195938 0.542126 0.112554
C -3.577932 1.906840 -0.124743
C -2.255321 2.096317 -0.250231
O -1.195678 -2.488658 1.176112
O -3.521148 -1.315858 1.518452
O -5.271843 0.679535 1.057156
C -4.770737 0.007673 -1.201273
C -5.016536 -1.481266 -1.164733
C -6.348055 -1.961534 -0.653455
O -4.133148 -2.268623 -1.506153
O -4.458108 2.945550 -0.388434
C -4.648313 3.765467 0.765208
C 4.676221 -0.601739 -0.582953
C 5.275686 -1.814721 -1.242185
C 5.354621 0.517230 -0.249936
C 6.830113 0.792005 -0.445201
C 7.519536 1.142018 0.887885
C 7.514793 -0.010226 1.885081
C 6.998919 1.941687 -1.441776
H 1.267656 -3.059252 0.902749
H 2.686161 1.253731 -0.910515
H 0.379439 2.130346 -0.775819
H -1.862875 3.077110 -0.502619
H -2.149593 -2.450244 1.409454
H -5.212621 -0.086740 1.663421
H -4.082819 0.185912 -2.038632
H -5.714505 0.504333 -1.457991
H -6.436653 -3.038545 -0.821684
H -6.431000 -1.761645 0.416587
H -7.154054 -1.460385 -1.195130
H -5.023899 3.186555 1.614630
H -3.718234 4.275335 1.038165
H -5.392815 4.527304 0.516215
H 4.600280 -2.215445 -2.007229
H 6.215449 -1.597780 -1.755871
H 5.465847 -2.599891 -0.502829
H 4.821955 1.323722 0.254632
H 7.342947 -0.080981 -0.860569
H 8.562379 1.421033 0.691434
H 7.042191 2.016321 1.348306
H 7.981206 -0.902377 1.455196
H 8.078368 0.267501 2.781587
H 6.498489 -0.267945 2.198087
H 6.535317 1.697678 -2.404261
H 8.060010 2.141902 -1.625942
H 6.541992 2.866640 -1.071775
SCF Energy (B3LYP/6-31G**)= -1361.70052048
Number of imaginary frequencies = 0

1a_c066
MMFF Geometry
C 1.970411 1.868290 -0.076313
C 2.188654 -0.762921 -0.713941
N 3.211525 1.332586 -0.165453
C 0.782540 1.164892 -0.287742
C 0.907010 -0.196977 -0.617539
C 3.317948 0.021657 -0.483193
C -0.252852 -0.959958 -0.849706
C -1.529086 -0.391254 -0.742893
C -1.644698 0.958476 -0.400114
C -0.500156 1.740370 -0.190218
C -3.001545 1.536000 -0.268957
C -4.179131 0.564993 -0.022284
C -3.951605 -0.749986 -0.760012
C -2.715575 -1.190529 -1.058937
O -0.585968 3.076843 0.117869
O -3.116693 2.762446 -0.306837
O -5.377192 1.187798 -0.499435
C -4.309059 0.404159 1.496828
C -5.248871 -0.708534 1.902738
C -6.730127 -0.453184 1.804567
O -4.810336 -1.794140 2.289995
O -5.124672 -1.416985 -1.062060
C -5.042366 -2.765492 -1.510229
C 4.701514 -0.527033 -0.585162
C 4.897655 -1.719925 -1.481297
C 5.703115 0.071845 0.096562
C 7.160924 -0.325975 0.159665
C 7.636468 -0.493239 1.616286
C 6.935507 -1.631335 2.348115
C 8.004299 0.736366 -0.549466
H 1.956608 2.925099 0.181689
H 2.293429 -1.815683 -0.954897
H -0.153174 -2.011247 -1.118366
H -2.544609 -2.134470 -1.564961
H -1.521019 3.366510 0.009668
H -5.358809 1.132795 -1.470922
H -4.669191 1.330637 1.961881
H -3.336114 0.188623 1.957795
H -7.268938 -1.223415 2.363863
H -6.968784 0.518276 2.244653
H -7.050353 -0.483489 0.761632
H -4.566995 -2.819751 -2.494593
H -4.513275 -3.389596 -0.782706
H -6.061995 -3.149835 -1.607113
H 4.327762 -1.609317 -2.411066
H 5.937436 -1.855273 -1.789449
H 4.573905 -2.636811 -0.977878
H 5.462993 0.949265 0.701159
H 7.331905 -1.279446 -0.349720
H 8.715020 -0.694920 1.621895
H 7.488580 0.438313 2.177182
H 7.061053 -2.577695 1.812474
H 7.360336 -1.750768 3.349978
H 5.864597 -1.437847 2.462119
H 7.696080 0.848249 -1.595080
H 9.063509 0.457158 -0.544030
H 7.912400 1.714597 -0.063846
SCF Energy (B3LYP/6-31G**)= -1361.70464096
Number of imaginary frequencies = 0

1a_c067
MMFF Geometry
C -1.740419 -1.936662 -0.256209
C -2.145317 0.704292 0.224740
N -3.006767 -1.519355 -0.013488
C -0.616703 -1.106410 -0.280368
C -0.838667 0.258481 -0.032274
C -3.203749 -0.203053 0.228089

C 0.251876 1.146946 -0.050255
C 1.557931 0.699064 -0.304660
C 1.774433 -0.661317 -0.542626
C 0.693644 -1.559581 -0.538388
C 3.151113 -1.133744 -0.814582
C 4.326969 -0.148336 -0.709572
C 3.933225 1.301194 -0.501518
C 2.653485 1.672745 -0.319676
O 0.862542 -2.901996 -0.788005
O 3.328870 -2.307733 -1.160744
O 4.968298 -0.232057 -2.004887
C 5.344201 -0.649580 0.323586
C 4.870189 -0.474671 1.747874
C 5.930216 -0.234061 2.789392
O 3.683697 -0.577235 2.057853
O 5.018259 2.162329 -0.494608
C 4.773583 3.562490 -0.443200
C -4.606804 0.218586 0.510280
C -4.777996 1.399569 1.424098
C -5.624747 -0.486276 -0.032215
C -7.122691 -0.283636 0.052392
C -7.640220 1.123313 -0.305177
C -7.205228 1.594229 -1.687282
C -7.672485 -0.755029 1.401080
H -1.648886 -3.005165 -0.438822
H -2.327888 1.758297 0.406163
H 0.074763 2.204798 0.140570
H 2.377411 2.708454 -0.157968
H 1.812348 -3.065384 -0.984071
H 5.048805 -1.183140 -2.211241
H 6.292340 -0.110973 0.198780
H 5.578379 -1.710894 0.176055
H 5.463478 -0.125106 3.772272
H 6.617778 -1.082923 2.814325
H 6.472178 0.685479 2.554806
H 4.161705 3.886105 -1.291429
H 4.308183 3.842051 0.507290
H 5.738048 4.074778 -0.508233
H -4.634126 2.336165 0.875511
H -5.762500 1.431551 1.893834
H -4.059453 1.364299 2.251156
H -5.363546 -1.346175 -0.654471
H -7.552893 -0.959838 -0.701447
H -8.737639 1.108598 -0.282456
H -7.335370 1.865164 0.438887
H -7.505364 0.877800 -2.458556
H -7.673698 2.556051 -1.919764
H -6.121386 1.731292 -1.744386
H -7.342039 -1.775414 1.625673
H -8.768008 -0.758364 1.388911
H -7.353623 -0.109821 2.224979
SCF Energy (B3LYP/6-31G**)= -1361.70456718
Number of imaginary frequencies = 0

1a_c068
MMFF Geometry
C -1.571249 -2.454276 -0.303880
C -2.328041 0.154638 -0.290196
N -2.897711 -2.178366 -0.266504
C -0.554549 -1.494138 -0.339758
C -0.959039 -0.147827 -0.336075
C -3.262023 -0.878514 -0.248270
C 0.015711 0.864347 -0.381698
C 1.384739 0.558871 -0.426473
C 1.787039 -0.781790 -0.419345
C 0.821224 -1.803260 -0.382989
C 3.235926 -1.109913 -0.471217
C 4.274131 0.036833 -0.453115
C 3.670539 1.422297 -0.509631
C 2.351507 1.654401 -0.476390
O 1.168903 -3.134554 -0.396966
O 3.563387 -2.296028 -0.576595
O 5.084095 -0.121355 -1.638176
C 5.228063 -0.155014 0.732630
C 4.577541 0.087296 2.076063
C 5.494884 0.467221 3.208221
O 3.370832 -0.067336 2.261278
O 4.571600 2.476513 -0.464388
C 4.711797 3.108186 -1.736944
C -4.721039 -0.619242 -0.207389
C -5.534679 -1.517255 -1.099808
C -5.224165 0.337823 0.601462
C -6.678474 0.707686 0.798289
C -6.959968 2.150012 0.338108
C -6.761878 2.352085 -1.159077
C -7.042324 0.548968 2.278228
H -1.335187 -3.516345 -0.305280
H -2.662886 1.187232 -0.300629
H -0.302461 1.906436 -0.377311
H 1.976799 2.673470 -0.450679
H 2.150524 -3.200749 -0.432149
H 4.478735 -0.202697 -2.394655
H 6.083928 0.524980 0.632154
H 5.645585 -1.169513 0.742279
H 4.911820 0.616217 4.121260
H 6.219668 -0.333109 3.375904
H 6.010258 1.399766 2.965352
H 5.031672 2.394709 -2.502940
H 3.778443 3.594845 -2.038573
H 5.482641 3.878991 -1.645889
H -5.652485 -2.506894 -0.645933
H -6.530921 -1.119809 -1.308411
H -5.048848 -1.639053 -2.075088
H -4.538052 0.909480 1.227246
H -7.336108 0.035857 0.238059
H -7.996841 2.410851 0.584667
H -6.320586 2.856765 0.882173
H -7.381035 1.655970 -1.733785
H -7.047586 3.370649 -1.440847
H -5.717108 2.207946 -1.450451
H -6.863451 -0.477332 2.618186
H -8.102007 0.773041 2.442148
H -6.455110 1.222339 2.913024
SCF Energy (B3LYP/6-31G**)= -1361.70113947
Number of imaginary frequencies = 0

1a_c069
MMFF Geometry
C -1.758945 -1.869409 -0.452205
C -2.177940 0.697283 0.326967
N -3.030269 -1.483379 -0.184868
C -0.636699 -1.042013 -0.359842
C -0.866222 0.284848 0.042115
C -3.234259 -0.204383 0.205772
C 0.221807 1.169924 0.147077
C 1.531442 0.754326 -0.136861
C 1.757540 -0.569866 -0.527442
C 0.678999 -1.462450 -0.646192
C 3.141703 -1.006992 -0.832606
C 4.317992 -0.038172 -0.606468
C 3.896573 1.371450 -0.254697
C 2.625094 1.716780 -0.015217
O 0.854774 -2.766483 -1.047852
O 3.328262 -2.131667 -1.312564
O 4.980721 -0.001872 -1.891113
C 5.311546 -0.632127 0.397964
C 4.790310 -0.589854 1.815598
C 5.766913 -0.198198 2.891477
O 3.631252 -0.903807 2.085292
O 4.893983 2.304262 0.003546
C 5.525211 2.764615 -1.191576
C -4.642928 0.181090 0.511047
C -4.832213 1.248382 1.552331
C -5.650008 -0.456693 -0.126441
C -7.149619 -0.265793 -0.044197
C -7.664422 1.172809 -0.245694
C -7.207531 1.800278 -1.556697
C -7.720747 -0.889968 1.231603
H -1.662042 -2.909381 -0.756292
H -2.365589 1.722833 0.627470
H 0.041373 2.198474 0.457839
H 2.390182 2.728512 0.302744

H 1.809374 -2.909290 -1.235614
H 5.036583 -0.930100 -2.194548
H 6.260994 -0.083933 0.348923
H 5.555743 -1.674507 0.159945
H 5.266179 -0.203574 3.863564
H 6.593050 -0.913005 2.913243
H 6.142788 0.809143 2.695825
H 6.318178 2.074520 -1.493146
H 4.807043 2.912686 -2.005729
H 5.991282 3.729695 -0.971135
H -5.824712 1.225794 2.005484
H -4.127962 1.117608 2.382089
H -4.680845 2.242187 1.118396
H -5.376420 -1.238704 -0.839473
H -7.565602 -0.850636 -0.878173
H -8.762029 1.155117 -0.243316
H -7.373778 1.823958 0.584029
H -7.493057 1.177529 -2.410439
H -7.674323 2.782309 -1.684502
H -6.123212 1.943467 -1.579324
H -7.391660 -1.929310 1.342472
H -8.815903 -0.892369 1.200634
H -7.417261 -0.344047 2.129733
SCF Energy (B3LYP/6-31G**)= -1361.69886068
Number of imaginary frequencies = 0

1a_c070
MMFF Geometry
C -1.835370 -1.366003 -0.994695
C -2.057374 1.196176 -0.127266
N -3.078884 -0.856499 -0.819085
C -0.647056 -0.668324 -0.764155
C -0.774293 0.656200 -0.315797
C -3.186728 0.420397 -0.385395
C 0.386220 1.410637 -0.066367
C 1.668595 0.869501 -0.255122
C 1.791788 -0.450803 -0.696778
C 0.639346 -1.215287 -0.953766
C 3.143787 -1.028191 -0.895417
C 4.388307 -0.167681 -0.582108
C 4.092271 1.253482 -0.130325
C 2.836745 1.711345 0.012912
O 0.712450 -2.514229 -1.402120
O 3.225209 -2.181524 -1.330734
O 5.129471 -0.073777 -1.818908
C 5.294218 -0.936673 0.389876
C 4.698100 -1.121031 1.767999
C 5.622236 -1.666966 2.825035
O 3.520408 -0.873593 2.023894
O 5.237311 1.996179 0.100978
C 5.103699 3.335992 0.560627
C -4.572220 0.950178 -0.216547
C -4.747788 2.439344 -0.344212
C -5.584485 0.085371 0.018005
C -7.051516 0.385700 0.231399
C -7.510295 0.022751 1.659151
C -7.456526 -1.456790 2.020142
C -7.887153 -0.313714 -0.843460
H -1.819162 -2.396951 -1.341726
H -2.162472 2.213950 0.233269
H 0.282459 2.437057 0.284041
H 2.632359 2.724499 0.340657
H 1.662174 -2.760806 -1.487094
H 4.683464 0.586835 -2.375033
H 6.255536 -0.418294 0.497362
H 5.526646 -1.932772 -0.007825
H 5.086523 -1.753782 3.774403
H 5.976160 -2.656581 2.526020
H 6.467619 -0.987653 2.959232
H 4.584498 3.953111 -0.179559
H 4.592995 3.368347 1.528387
H 6.108626 3.746582 0.696092
H -4.161300 2.831179 -1.183318
H -5.779886 2.735634 -0.544205
H -4.427924 2.944282 0.573291
H -5.342107 -0.977983 0.070419
H -7.238978 1.458267 0.122415
H -6.902538 0.580063 2.383708
H -8.542624 0.370042 1.794398
H -6.438647 -1.852155 1.960166
H -7.804681 -1.599446 3.048642
H -8.102013 -2.053496 1.369387
H -7.632053 0.067530 -1.838920
H -8.955468 -0.133542 -0.682951
H -7.722853 -1.396538 -0.854956
SCF Energy (B3LYP/6-31G**)= -1361.70707417
Number of imaginary frequencies = 0

1a_c071
MMFF Geometry
C 1.668369 2.578602 0.185547
C 2.365744 0.018373 -0.402876
N 2.986854 2.267740 0.165406
C 0.631157 1.677175 -0.073723
C 1.004359 0.355232 -0.380233
C 3.322357 0.990908 -0.118737
C 0.007537 -0.596198 -0.664789
C -1.351836 -0.256018 -0.631851
C -1.715730 1.053823 -0.311908
C -0.735263 2.021798 -0.049582
C -3.153838 1.390717 -0.263283
C -4.185008 0.251551 -0.154678
C -3.674476 -1.031206 -0.793393
C -2.361492 -1.255918 -0.986531
O -1.064176 3.324456 0.239875
O -3.496543 2.577496 -0.256924
O -5.347611 0.715196 -0.861401
C -4.533607 0.109585 1.331228
C -5.366489 -1.111062 1.654473
C -6.823152 -1.095148 1.269684
O -4.866135 -2.078832 2.231880
O -4.682355 -1.921202 -1.118075
C -4.332777 -3.204655 -1.620188
C 4.774971 0.695662 -0.133030
C 5.618217 1.760905 -0.780922
C 5.251336 -0.437437 0.426019
C 6.699248 -0.868372 0.539699
C 6.936524 -2.091750 -0.363951
C 8.392987 -2.532185 -0.417896
C 7.024050 -1.157075 2.008626
H 1.457752 3.618531 0.426165
H 2.677892 -0.990793 -0.653708
H 0.301515 -1.615159 -0.915379
H -1.995138 -2.178365 -1.423632
H -2.028586 3.448945 0.092274
H -5.368008 1.688065 -0.763562
H -5.084099 0.987472 1.692846
H -3.621106 0.047232 1.939202
H -7.330306 -1.946547 1.732714
H -7.290088 -0.177039 1.635018
H -6.933523 -1.168108 0.186682
H -3.816419 -3.120472 -2.581660
H -3.726500 -3.756925 -0.894994
H -5.257874 -3.765537 -1.782656
H 5.768154 2.602493 -0.096504
H 6.600380 1.393694 -1.089394
H 5.138285 2.136768 -1.692221
H 4.548944 -1.130821 0.889487
H 7.367656 -0.063513 0.217935
H 6.321327 -2.937417 -0.030886
H 6.615154 -1.854230 -1.386504
H 8.734602 -2.910616 0.549817
H 8.513633 -3.338527 -1.148607
H 9.043286 -1.704321 -0.717392
H 6.770600 -0.298913 2.641694
H 8.091643 -1.355570 2.145933
H 6.468264 -2.025265 2.380417
SCF Energy (B3LYP/6-31G**)= -1361.70843978
Number of imaginary frequencies = 0

1a_c072
MMFF Geometry

C -1.810731 -1.746675 -0.096386
C -2.084052 0.952541 -0.191962
N -3.060242 -1.228562 -0.013168
C -0.641283 -0.993698 -0.229919
C -0.794933 0.401912 -0.277949
C -3.192287 0.116958 -0.060573
C 0.345227 1.214953 -0.412203
C 1.633339 0.662842 -0.496440
C 1.781052 -0.726176 -0.442377
C 0.650893 -1.551990 -0.316864
C 3.138853 -1.309333 -0.535760
C 4.363996 -0.380474 -0.566813
C 4.046091 1.098154 -0.676738
C 2.783189 1.559603 -0.645303
O 0.752814 -2.923506 -0.278560
O 3.263543 -2.536441 -0.627060
O 5.055958 -0.757075 -1.781506
C 5.303846 -0.708432 0.600491
C 4.777824 -0.224665 1.932069
C 5.802500 0.173530 2.960544
O 3.574808 -0.206238 2.190469
O 5.177902 1.889860 -0.782175
C 5.012951 3.281534 -1.025046
C -4.583549 0.651428 0.017071
C -4.840280 1.951016 -0.693168
C -5.523553 -0.061982 0.676416
C -6.986888 0.229529 0.933034
C -7.890161 0.027350 -0.297680
C -7.816743 -1.380014 -0.877346
C -7.230632 1.592710 1.588252
H -1.773019 -2.832920 -0.050932
H -2.210584 2.029854 -0.214136
H 0.221405 2.296688 -0.449023
H 2.560743 2.618520 -0.710651
H 1.699355 -3.168681 -0.384470
H 5.091007 -1.732963 -1.787056
H 6.286083 -0.252215 0.422413
H 5.482749 -1.787434 0.682582
H 5.299513 0.502414 3.874113
H 6.438805 -0.683592 3.193754
H 6.405848 0.999504 2.575777
H 4.459757 3.455724 -1.953618
H 4.522237 3.769585 -0.176995
H 6.007509 3.723303 -1.137102
H -4.325032 1.978336 -1.660296
H -5.895914 2.109406 -0.919315
H -4.495017 2.796125 -0.088625
H -5.211705 -1.009164 1.124271
H -7.305602 -0.510513 1.682223
H -8.930965 0.224601 -0.011151
H -7.645624 0.747705 -1.085566
H -8.057933 -2.130404 -0.117785
H -8.535117 -1.485764 -1.696692
H -6.822176 -1.598879 -1.277693
H -6.551874 1.751834 2.433376
H -8.255046 1.648323 1.973752
H -7.105929 2.424038 0.889584
SCF Energy (B3LYP/6-31G**)= -1361.70471306
Number of imaginary frequencies = 0

1a_c073
MMFF Geometry
C 1.843856 -1.545686 -0.135745
C 2.296603 1.130769 -0.050089
N 3.127890 -1.115699 -0.087192
C 0.723996 -0.711179 -0.145532
C 0.969873 0.673021 -0.101289
C 3.350810 0.218195 -0.043513
C -0.116741 1.567581 -0.116270
C -1.438690 1.103666 -0.158983
C -1.674637 -0.272081 -0.186500
C -0.604724 -1.178146 -0.199304
C -3.076761 -0.737282 -0.232483
C -4.174036 0.214886 0.302390
C -3.822428 1.664731 -0.044925
C -2.548227 2.056663 -0.238764
O -0.808277 -2.535825 -0.266426
O -3.294230 -1.875010 -0.655170
O -4.201918 0.157782 1.732023
C -5.538772 -0.190255 -0.291863
C -5.980320 -1.567002 0.164772
C -6.797145 -2.380093 -0.802283
O -5.728761 -1.975821 1.300681
O -4.919542 2.508877 -0.088850
C -4.698527 3.909266 -0.207379
C 4.778046 0.652012 -0.005363
C 5.088831 2.018437 -0.553899
C 5.713520 -0.192890 0.482474
C 7.200764 0.038549 0.631233
C 8.013539 -0.906630 -0.272786
C 7.788021 -0.658561 -1.759048
C 7.596867 -0.169872 2.096950
H 1.734942 -2.627643 -0.167594
H 2.494241 2.196473 -0.002159
H 0.075768 2.639515 -0.093794
H -2.285940 3.085703 -0.455536
H -1.765373 -2.701535 -0.422017
H -4.601811 -0.705505 1.976317
H -5.492738 -0.178023 -1.387449
H -6.338001 0.489094 0.027883
H -7.037417 -3.349574 -0.357273
H -7.726322 -1.852142 -1.029734
H -6.223265 -2.547068 -1.717144
H -4.091848 4.283062 0.623779
H -4.238469 4.151123 -1.170703
H -5.671544 4.407608 -0.165360
H 4.826112 2.794020 0.172995
H 6.142823 2.145925 -0.812473
H 4.534002 2.203033 -1.481037
H 5.387428 -1.174523 0.833673
H 7.467541 1.069298 0.378281
H 9.082412 -0.775621 -0.062306
H 7.774767 -1.953162 -0.044564
H 8.012211 0.380231 -2.021415
H 8.443548 -1.305653 -2.350713
H 6.756204 -0.874850 -2.051800
H 7.039168 0.506944 2.754048
H 8.663891 0.031552 2.241591
H 7.401955 -1.196997 2.425980
SCF Energy (B3LYP/6-31G**)= -1361.71430300
Number of imaginary frequencies = 0

1a_c074
MMFF Geometry
C -1.811256 -1.661253 -0.185058
C -2.325083 1.002701 -0.309370
N -3.099695 -1.264489 -0.321175
C -0.716231 -0.798216 -0.102080
C -0.993947 0.579276 -0.164812
C -3.352439 0.063142 -0.385749
C 0.065240 1.501615 -0.072425
C 1.392515 1.071895 0.062505
C 1.661579 -0.297375 0.105541
C 0.617186 -1.230800 0.043932
C 3.068733 -0.726192 0.248829
C 4.175301 0.255381 -0.208091
C 3.763002 1.694651 0.115683
C 2.468651 2.053014 0.220627
O 0.850581 -2.582845 0.125978
O 3.285874 -1.858818 0.685145
O 4.303157 0.203089 -1.632470
C 5.505849 -0.116139 0.478472
C 6.013206 -1.479819 0.052019
C 6.782210 -2.274319 1.072271
O 5.851113 -1.891817 -1.098935
O 4.832166 2.566579 0.237240
C 4.567210 3.960279 0.343947
C -4.780553 0.460085 -0.553625
C -5.046053 1.779897 -1.225950
C -5.754211 -0.375248 -0.128557
C -7.251451 -0.167231 -0.175198
C -7.889692 -0.355278 1.215166

C -7.427956 0.682222 2.231324
C -7.866221 -1.151835 -1.172855
H -1.677045 -2.740034 -0.143390
H -2.549351 2.063559 -0.347246
H -0.153002 2.568268 -0.107450
H 2.165230 3.074244 0.419974
H 1.798647 -2.723991 0.347228
H 4.741197 -0.648912 -1.849458
H 5.384136 -0.108112 1.568287
H 6.307378 0.584418 0.215238
H 7.077505 -3.236079 0.643929
H 7.679578 -1.723303 1.363732
H 6.151191 -2.458498 1.945244
H 4.010271 4.322125 -0.526392
H 4.035200 4.185881 1.273578
H 5.527544 4.483629 0.371090
H -4.373124 1.927808 -2.078462
H -6.057088 1.854112 -1.634353
H -4.906323 2.605930 -0.520928
H -5.458051 -1.324413 0.323579
H -7.498593 0.844247 -0.512405
H -8.980702 -0.283093 1.123142
H -7.673320 -1.358491 1.603768
H -7.626876 1.697238 1.872933
H -7.963869 0.546008 3.176161
H -6.357646 0.592789 2.440424
H -7.441822 -1.012672 -2.173527
H -8.948719 -1.001944 -1.247965
H -7.691155 -2.191507 -0.873449
SCF Energy (B3LYP/6-31G**)= -1361.71430945
Number of imaginary frequencies = 0

1a_c075
MMFF Geometry
C -1.328531 -2.316804 -0.669035
C -2.276627 0.215941 -0.396171
N -2.671492 -2.143072 -0.614263
C -0.386408 -1.286003 -0.597232
C -0.888892 0.021753 -0.458849
C -3.131498 -0.882217 -0.468105
C 0.007611 1.104831 -0.397389
C 1.393286 0.903181 -0.452821
C 1.885044 -0.400236 -0.571180
C 1.006541 -1.488869 -0.663193
C 3.350104 -0.590018 -0.628823
C 4.245461 0.526894 -0.042303
C 3.626015 1.902262 -0.282618
C 2.299481 2.056485 -0.454000
O 1.462074 -2.774816 -0.825824
O 3.789125 -1.633934 -1.114375
O 5.524963 0.475878 -0.687344
C 4.485245 0.327235 1.455900
C 4.734115 -1.120563 1.809479
C 6.102263 -1.690267 1.545569
O 3.823538 -1.812250 2.269079
O 4.552199 2.928735 -0.277922
C 4.095880 4.263972 -0.461015
C -4.605965 -0.737762 -0.414804
C -5.349723 -1.604080 -1.395570
C -5.182920 0.095771 0.477233
C -6.664207 0.324705 0.696919
C -7.029285 1.750262 0.245197
C -8.522034 2.043027 0.309758
C -6.998009 0.077110 2.171321
H -1.014606 -3.352996 -0.777362
H -2.688153 1.216470 -0.306777
H -0.387376 2.116145 -0.307467
H 1.844032 3.029206 -0.604506
H 2.439522 -2.753937 -0.937867
H 5.399961 0.807141 -1.593670
H 3.615649 0.649519 2.043952
H 5.341502 0.914056 1.810709
H 6.207932 -2.641565 2.074770
H 6.236900 -1.860866 0.475725
H 6.867593 -1.004735 1.917881
H 3.621605 4.383847 -1.440316
H 3.414959 4.557950 0.344150
H 4.966740 4.925074 -0.424687
H -5.409511 -2.634841 -1.030690
H -6.366361 -1.250768 -1.585997
H -4.846437 -1.610016 -2.369604
H -4.545097 0.659086 1.158924
H -7.255468 -0.387328 0.112530
H -6.494812 2.491827 0.852629
H -6.698574 1.897385 -0.791285
H -8.887163 2.044432 1.340806
H -8.728679 3.030876 -0.114663
H -9.092770 1.303689 -0.260843
H -6.655352 -0.914332 2.489253
H -8.078091 0.116314 2.344225
H -6.524373 0.821301 2.821498
SCF Energy (B3LYP/6-31G**)= -1361.69981313
Number of imaginary frequencies = 0

1a_c076
MMFF Geometry
C 1.810268 -1.538002 0.029369
C 2.274170 1.118292 -0.289355
N 3.096682 -1.114399 -0.013456
C 0.692908 -0.706672 -0.078798
C 0.945187 0.666447 -0.243227
C 3.325180 0.209713 -0.172244
C -0.138214 1.555662 -0.361812
C -1.462774 1.099158 -0.308058
C -1.710317 -0.264529 -0.136104
C -0.639561 -1.167108 -0.034888
C -3.115581 -0.735337 -0.097396
C -4.247258 0.285298 0.189627
C -3.826957 1.690502 -0.221277
C -2.560108 2.046330 -0.471942
O -0.842269 -2.519746 0.107552
O -3.320370 -1.942102 -0.256443
O -4.476911 0.335683 1.599980
C -5.513596 -0.145647 -0.579972
C -6.095126 -1.446063 -0.061135
C -6.726413 -2.366561 -1.069862
O -6.098714 -1.707666 1.143825
O -4.825511 2.639255 -0.413004
C -5.336040 3.119615 0.833036
C 4.753992 0.635421 -0.229012
C 5.058031 1.905361 -0.977219
C 5.699367 -0.134882 0.353865
C 7.189784 0.116918 0.434994
C 7.924682 -0.876612 -0.482516
C 9.424298 -0.627576 -0.567942
C 7.639951 0.004064 1.895118
H 1.697190 -2.612215 0.158979
H 2.476173 2.178270 -0.402840
H 0.057407 2.619059 -0.496355
H -2.330125 3.061796 -0.781066
H -1.805525 -2.702896 0.026306
H -4.927744 -0.502591 1.846341
H -5.285390 -0.259796 -1.646714
H -6.320788 0.589725 -0.483427
H -7.091165 -3.267438 -0.568838
H -7.567284 -1.860550 -1.550201
H -5.983314 -2.657318 -1.816495
H -6.007848 2.384944 1.286314
H -4.528999 3.384664 1.524980
H -5.918062 4.023022 0.627902
H 4.825817 2.780333 -0.361300
H 6.104690 1.978144 -1.284018
H 4.475581 1.963139 -1.903987
H 5.382133 -1.057189 0.845649
H 7.426050 1.135029 0.109392
H 7.753807 -1.906637 -0.144301
H 7.510479 -0.807269 -1.496868
H 9.918077 -0.814654 0.389953
H 9.873859 -1.298360 -1.307283
H 9.635088 0.402142 -0.873228
H 7.046046 0.661942 2.540057
H 8.687649 0.299367 2.009818

H   7.534392 -1.020011 2.270648
SCF Energy (B3LYP/6-31G**)= -1361.70794542
Number of imaginary frequencies = 0

1a_c077
MMFF Geometry
C   1.824491 1.717659 -0.059080
C   2.115520 -0.978457 -0.187503
N   3.078838 1.207776 -0.001371
C   0.658375 0.957894 -0.182484
C   0.821463 -0.436317 -0.247929
C   3.219721 -0.136285 -0.064686
C -0.314168 -1.256590 -0.373387
C -1.605747 -0.711813 -0.433216
C -1.764900 0.676177 -0.360517
C -0.638989 1.508368 -0.243040
C -3.130222 1.251678 -0.429900
C -4.354294 0.316870 -0.464812
C -4.007055 -1.148104 -0.612076
C -2.750980 -1.610314 -0.568530
O -0.749689 2.878579 -0.188328
O -3.265486 2.478702 -0.508457
O -5.065614 0.731923 -1.653335
C -5.271297 0.595371 0.731166
C -4.700414 0.069548 2.027583
C -5.657102 -0.609249 2.970248
O -3.515434 0.227268 2.320247
O -5.049223 -2.067643 -0.614249
C -5.753308 -2.082971 -1.856301
C   4.615896 -0.661371 -0.014223
C   4.869919 -1.953832 -0.738366
C   5.561754 0.053574 0.635046
C   7.031074 -0.229646 0.865383
C   7.912553 -0.012701 -0.378551
C   7.819964 1.398088 -0.947040
C   7.294783 -1.595615 1.506919
H   1.779928 2.803030 -0.000971
H   2.248956 -2.054630 -0.222734
H -0.185506 -2.337344 -0.423022
H -2.566684 -2.680049 -0.608431
H -1.700107 3.118324 -0.266664
H -5.076238 1.709880 -1.636196
H -6.253617 0.136915 0.560194
H -5.460243 1.668348 0.857113
H -5.120551 -0.943653 3.862438
H -6.438126 0.094475 3.268076
H -6.098996 -1.479956 2.479438
H -6.513261 -1.296727 -1.872618
H -5.077583 -1.989665 -2.713751
H -6.269148 -3.044738 -1.934463
H   4.340002 -1.977084 -1.697641
H   5.922901 -2.103714 -0.981957
H   4.539470 -2.805762 -0.135116
H   5.250797 0.994968 1.095517
H   7.357134 0.507363 1.614405
H   8.959259 -0.204667 -0.110526
H   7.660050 -0.729279 -1.167361
H   8.068478 2.144876 -0.186291
H   8.524013 1.514494 -1.777302
H   6.817466 1.612778 -1.329463
H   6.631262 -1.765143 2.362062
H   8.325819 -1.646930 1.874964
H   7.163995 -2.422952 0.804637
SCF Energy (B3LYP/6-31G**)= -1361.69902369
Number of imaginary frequencies = 0

1a_c078
MMFF Geometry
C -1.732700 -1.412138 -0.623689
C -2.122510 1.148578 0.193115
N -3.003005 -1.000857 -0.392731
C -0.597013 -0.613085 -0.473906
C -0.810455 0.711878 -0.052083
C -3.194329 0.274535 0.015984
C   0.292358 1.572357 0.106205
C   1.600114 1.128886 -0.133379
C   1.802762 -0.194104 -0.535690
C   0.716286 -1.058617 -0.724646
C   3.186822 -0.636362 -0.791370
C   4.344433 0.159157 -0.157684
C   3.999903 1.638537 -0.031637
C   2.722625 2.065668 -0.012941
O   0.885370 -2.350663 -1.159568
O   3.391868 -1.627531 -1.497924
O   5.473110 0.022253 -1.040488
C   4.746555 -0.383580 1.215775
C   4.715043 -1.891688 1.274451
C   5.941893 -2.639886 0.826989
O   3.696137 -2.480015 1.639223
O   5.110335 2.451749 0.107872
C   4.925844 3.861004 0.147322
C -4.605117 0.683700 0.277287
C -4.818373 1.794218 1.270177
C -5.609665 0.033864 -0.351556
C -7.097568 0.286299 -0.251694
C -7.832996 -0.909280 0.381514
C -7.441881 -1.155362 1.833354
C -7.654900 0.561442 -1.652522
H -1.648850 -2.447899 -0.945935
H -2.297454 2.172323 0.506844
H   0.121814 2.602055 0.418533
H   2.467706 3.113312 0.102775
H   1.835021 -2.496794 -1.366545
H   5.257400 -0.694249 -1.670469
H   4.063315 -0.028701 1.998952
H   5.752424 -0.051835 1.501207
H   5.839347 -3.697095 1.087597
H   6.059873 -2.550458 -0.254631
H   6.823251 -2.243651 1.337294
H   4.439195 4.218788 -0.765770
H   4.358268 4.155121 1.035891
H   5.913114 4.328262 0.208911
H -4.617924 2.766823 0.808903
H -5.833778 1.818106 1.673216
H -4.159870 1.674508 2.138246
H -5.349414 -0.777817 -1.034725
H -7.307815 1.176301 0.349414
H -8.914457 -0.727540 0.344543
H -7.648283 -1.822477 -0.198380
H -7.608671 -0.260456 2.441227
H -8.046760 -1.966042 2.252197
H -6.390637 -1.444811 1.924109
H -7.153517 1.420515 -2.112410
H -8.725553 0.788339 -1.605077
H -7.524049 -0.301089 -2.315965
SCF Energy (B3LYP/6-31G**)= -1361.70651885
Number of imaginary frequencies = 0

1a_c079
MMFF Geometry
C -1.370301 -2.213322 -0.506965
C -2.237298 0.210951 0.364671
N -2.687129 -2.075343 -0.217829
C -0.416685 -1.197176 -0.391471
C -0.876696 0.053590 0.062287
C -3.108494 -0.865242 0.207408
C   0.033271 1.118756 0.196311
C   1.392631 0.952899 -0.101118
C   1.844342 -0.296541 -0.536528
C   0.948183 -1.362003 -0.700823
C   3.282032 -0.449260 -0.845369
C   4.268959 0.549659 -0.196337
C   3.638804 1.935895 -0.076481
C   2.303651 2.099328 -0.017169
O   1.358463 -2.587515 -1.167059
O   3.630512 -1.375208 -1.579827
O   5.437991 0.641812 -1.021354
C   4.725400 0.072822 1.184355
C   5.004787 -1.411792 1.215557
C   6.311166 -1.899904 0.648838
O   4.163280 -2.192110 1.664782
O   4.568830 2.956972 -0.013743

C 4.107994 4.295362 0.130971
C -4.554322 -0.760491 0.518197
C -5.117535 -1.949355 1.249714
C -5.263570 0.320590 0.128791
C -6.745935 0.567883 0.319667
C -7.401422 0.817584 -1.052538
C -8.916972 0.948746 -0.985481
C -6.936306 1.745486 1.278185
H -1.087887 -3.207378 -0.847659
H -2.612357 1.160784 0.732853
H -0.330795 2.088311 0.534352
H 1.843716 3.075750 0.089224
H 2.308058 -2.530110 -1.418774
H 5.184388 1.136964 -1.819663
H 3.956849 0.264177 1.945001
H 5.632436 0.594670 1.513575
H 7.132150 -1.285947 1.027445
H 6.481442 -2.932695 0.965912
H 6.283701 -1.862010 -0.441853
H 3.495236 4.592310 -0.726167
H 3.558048 4.419853 1.069283
H 4.983219 4.950885 0.163293
H -4.426247 -2.289549 2.029768
H -6.058257 -1.726988 1.759473
H -5.293369 -2.779400 0.557367
H -4.754014 1.112266 -0.421146
H -7.230333 -0.307442 0.763183
H -6.987159 1.722627 -1.514957
H -7.161216 -0.016217 -1.725140
H -9.218951 1.844947 -0.435961
H -9.331225 1.026177 -1.995969
H -9.366360 0.075777 -0.501844
H -6.402806 1.573965 2.220214
H -7.992674 1.886950 1.527005
H -6.562405 2.680325 0.845552
SCF Energy (B3LYP/6-31G**)= -1361.69986099
Number of imaginary frequencies = 0

1a_c080
MMFF Geometry
C -1.701664 -2.444060 0.053932
C -2.318241 0.190190 0.314768
N -2.990463 -2.115604 0.311880
C -0.656428 -1.525915 -0.087835
C -0.987276 -0.164992 0.050651
C -3.287865 -0.803774 0.431223
C 0.020000 0.808382 -0.082822
C 1.349762 0.447148 -0.339734
C 1.673121 -0.905733 -0.464587
C 0.678864 -1.888946 -0.355487
C 3.082862 -1.270098 -0.717327
C 4.185625 -0.232975 -0.432616
C 3.674401 1.190422 -0.595633
C 2.363245 1.487324 -0.529120
O 0.964803 -3.225617 -0.498480
O 3.352109 -2.413309 -1.100122
O 5.216584 -0.491175 -1.400369
C 4.732168 -0.540248 0.966127
C 5.673057 0.514399 1.505230
C 7.063371 0.582914 0.928581
O 5.312357 1.279556 2.402312
O 4.683309 2.116925 -0.788084
C 4.349313 3.497879 -0.842727
C -4.708304 -0.489698 0.716720
C -5.356215 -1.407349 1.718560
C -5.327075 0.528482 0.081242
C -6.774306 0.955677 0.216092
C -7.444340 0.929807 -1.171478
C -8.938856 1.218181 -1.128409
C -6.827612 2.341905 0.862057
H -1.522583 -3.513215 -0.038516
H -2.593750 1.232585 0.442380
H -0.242580 1.861544 0.014148
H 1.999404 2.504440 -0.625415
H 1.892396 -3.320274 -0.811516
H 5.191827 -1.450280 -1.590016
H 5.270278 -1.496701 0.977978
H 3.911768 -0.640274 1.689397
H 7.677958 1.249108 1.540914
H 7.516610 -0.411480 0.942046
H 7.035900 0.970019 -0.091121
H 3.709154 3.710992 -1.704653
H 3.875775 3.823309 0.089120
H 5.277615 4.063749 -0.964528
H -4.676304 -1.614108 2.553464
H -6.257233 -0.979492 2.165054
H -5.627524 -2.359047 1.249626
H -4.763905 1.112337 -0.647414
H -7.323582 0.263377 0.861505
H -6.962712 1.652980 -1.842001
H -7.301486 -0.060378 -1.623631
H -9.141105 2.244969 -0.810381
H -9.373894 1.089744 -2.124870
H -9.451884 0.534462 -0.444845
H -6.284523 2.351538 1.814118
H -7.858773 2.639844 1.076014
H -6.382543 3.104898 0.213483
SCF Energy (B3LYP/6-31G**)= -1361.70846960
Number of imaginary frequencies = 0

1a_c081
MMFF Geometry
C -1.794873 -1.516089 -0.478290
C -2.286140 1.115944 -0.026662
N -3.081737 -1.092475 -0.504220
C -0.690228 -0.696510 -0.234473
C -0.956797 0.663705 -0.000063
C -3.323559 0.219898 -0.280968
C 0.112504 1.539236 0.261726
C 1.438131 1.083031 0.279298
C 1.700749 -0.266832 0.035560
C 0.642580 -1.157248 -0.207053
C 3.105772 -0.738469 0.073104
C 4.254064 0.295543 -0.057415
C 3.800845 1.671235 0.413913
C 2.517828 2.013457 0.590281
O 0.858077 -2.498206 -0.422475
O 3.300186 -1.953817 0.166070
O 4.590533 0.440026 -1.439561
C 5.458825 -0.190267 0.775746
C 6.080782 -1.454648 0.216330
C 6.635082 -2.442986 1.205930
O 6.176789 -1.634524 -0.999734
O 4.779952 2.601653 0.745061
C 5.383579 3.163164 -0.422980
C -4.750759 0.651282 -0.334786
C -5.014707 2.086503 -0.702756
C -5.725642 -0.251212 -0.085960
C -7.222972 -0.029751 -0.064191
C -7.792150 -0.529471 1.278284
C -9.274692 -0.231355 1.456834
C -7.852518 -0.741432 -1.263476
H -1.670352 -2.580080 -0.667870
H -2.500465 2.162144 0.165130
H -0.094920 2.591786 0.452243
H 2.262842 3.006461 0.948822
H 1.812751 -2.689294 -0.280737
H 5.060582 -0.381167 -1.706679
H 5.150106 -0.375245 1.811854
H 6.269594 0.547346 0.790810
H 7.435879 -1.973157 1.781968
H 5.837767 -2.780968 1.872453
H 7.038737 -3.309251 0.674616
H 6.089387 2.458420 -0.872091
H 4.631431 3.477046 -1.155170
H 5.946621 4.048671 -0.113409
H -4.354402 2.410435 -1.515517
H -6.031489 2.252462 -1.067776
H -4.855746 2.740870 0.160615
H -5.430990 -1.277995 0.142306
H -7.456420 1.036648 -0.142513
H -7.632792 -1.610373 1.382303

H -7.246951 -0.051411 2.102634
H -9.885949 -0.790004 0.742264
H -9.596265 -0.520324 2.462705
H -9.479322 0.836356 1.330176
H -7.383964 -0.419213 -2.200532
H -8.920389 -0.515165 -1.342661
H -7.741634 -1.829101 -1.188884
SCF Energy (B3LYP/6-31G**)= -1361.70789525
Number of imaginary frequencies = 0

1a_c082
MMFF Geometry
C -1.703953 -1.409227 -0.967610
C -2.155914 1.150076 -0.178820
N -2.988339 -0.998965 -0.832637
C -0.583321 -0.610011 -0.730628
C -0.828803 0.713528 -0.321943
C -3.210713 0.276238 -0.439423
C 0.258458 1.572127 -0.072253
C 1.580846 1.128943 -0.210748
C 1.814007 -0.191991 -0.603162
C 0.745449 -1.055023 -0.880401
C 3.213685 -0.633833 -0.752197
C 4.318382 0.157234 -0.025386
C 3.965416 1.636100 0.082041
C 2.690751 2.064114 0.002911
O 0.947745 -2.345382 -1.306033
O 3.473084 -1.621157 -1.446158
O 5.512518 0.024773 -0.818159
C 4.611752 -0.393841 1.372080
C 4.576743 -1.902301 1.418888
C 5.835646 -2.647471 1.065229
O 3.532622 -2.493125 1.698342
O 5.061713 2.447598 0.313242
C 4.874950 3.856672 0.347987
C -4.637471 0.693973 -0.315789
C -4.936947 2.157887 -0.493112
C -5.583051 -0.242457 -0.080258
C -7.070835 -0.044120 0.105038
C -7.563656 -0.676582 1.421452
C -6.970088 -0.018381 2.661061
C -7.813174 -0.659628 -1.083575
H -1.595344 -2.444195 -1.284976
H -2.354571 2.165379 0.147996
H 0.064248 2.600198 0.231492
H 2.427875 3.111242 0.104616
H 1.910567 -2.490959 -1.439479
H 5.346863 -0.688596 -1.466578
H 3.868898 -0.043864 2.101327
H 5.591900 -0.063690 1.737608
H 5.713668 -3.706233 1.310729
H 6.038517 -2.551615 -0.003167
H 6.673700 -2.254009 1.645797
H 4.461456 4.221157 -0.597922
H 4.239503 4.145007 1.191357
H 5.854435 4.322773 0.490054
H -4.354610 2.579514 -1.320516
H -5.983226 2.351590 -0.742607
H -4.700684 2.712522 0.420931
H -5.266327 -1.284179 0.007596
H -7.325390 1.019755 0.140043
H -8.655996 -0.588460 1.476306
H -7.334104 -1.749522 1.440741
H -7.178774 1.056142 2.672085
H -7.406814 -0.457059 3.564004
H -5.886440 -0.160819 2.713034
H -7.493125 -0.201136 -2.026007
H -8.893217 -0.503347 -0.988736
H -7.635048 -1.738663 -1.155436
SCF Energy (B3LYP/6-31G**)= -1361.70658327
Number of imaginary frequencies = 0

1a_c083
MMFF Geometry
C 1.638805 -2.294471 0.148528
C 2.523637 0.143756 -0.660385
N 2.973541 -2.124282 -0.012793
C 0.674382 -1.305735 -0.070864
C 1.144404 -0.048613 -0.492304
C 3.403824 -0.906129 -0.404931
C 0.222989 0.983945 -0.742190
C -1.153624 0.787397 -0.564337
C -1.617549 -0.458130 -0.134984
C -0.711493 -1.505384 0.098046
C -3.077161 -0.656292 0.033168
C -3.998596 0.584345 0.161945
C -3.373041 1.796523 -0.515795
C -2.082605 1.870587 -0.867438
O -1.132087 -2.753885 0.492341
O -3.493577 -1.815971 0.105762
O -4.125580 0.923889 1.545055
C -5.366385 0.257110 -0.473402
C -6.125552 -0.806022 0.295921
C -6.967969 -1.762121 -0.503817
O -6.095577 -0.845930 1.527949
O -4.206487 2.858645 -0.849292
C -4.547013 3.636502 0.300908
C 4.870898 -0.760537 -0.572983
C 5.537432 -1.944296 -1.221911
C 5.500922 0.345384 -0.121167
C 6.978393 0.665243 -0.193671
C 7.679754 0.446665 1.161430
C 7.203651 1.337433 2.302525
C 7.178697 2.086783 -0.728259
H 1.351099 -3.292285 0.473062
H 2.906779 1.100807 -1.000954
H 0.587994 1.954235 -1.077608
H -1.703655 2.757433 -1.366990
H -2.115668 -2.772977 0.476707
H -4.695370 0.236633 1.956952
H -5.229740 -0.082809 -1.507242
H -6.029272 1.130031 -0.488988
H -7.447284 -2.480434 0.166952
H -7.739933 -1.206737 -1.041828
H -6.334953 -2.308225 -1.207563
H -5.302549 3.125856 0.905009
H -3.663820 3.872777 0.904645
H -4.980085 4.577762 -0.050448
H 5.040224 -2.192212 -2.166700
H 6.589760 -1.774796 -1.457976
H 5.493959 -2.821303 -0.566822
H 4.909915 1.108116 0.386643
H 7.471484 0.002997 -0.912443
H 7.556299 -0.601216 1.464094
H 8.758077 0.602389 1.030033
H 6.140458 1.190671 2.513593
H 7.757737 1.097100 3.216029
H 7.373146 2.395636 2.084122
H 6.779566 2.176281 -1.745153
H 8.243559 2.340194 -0.766090
H 6.673128 2.836402 -0.110212
SCF Energy (B3LYP/6-31G**)= -1361.70657703
Number of imaginary frequencies = 0

1a_c084
MMFF Geometry
C -1.862862 -1.465317 -0.689955
C -2.124980 1.056782 0.278646
N -3.112033 -1.005479 -0.435435
C -0.687990 -0.734852 -0.494562
C -0.836418 0.569704 0.004476
C -3.239335 0.250756 0.050354
C 0.308465 1.358055 0.216860
C 1.594989 0.869253 -0.058770
C 1.741641 -0.432878 -0.548348
C 0.604435 -1.229996 -0.768835
C 3.101585 -0.951124 -0.848624
C 4.331185 -0.038840 -0.616217
C 3.996727 1.338326 -0.079849
C 2.746645 1.733149 0.189598
O 0.697809 -2.510203 -1.264454
O 3.201462 -2.082045 -1.335272

O 4.941628 0.133570 -1.913225
C 5.355373 -0.786694 0.247926
C 4.891852 -1.011697 1.670817
C 5.964226 -1.308687 2.685595
O 3.702899 -1.004717 1.987730
O 5.041899 2.171357 0.301921
C 5.795041 2.670329 -0.802188
C -4.627998 0.714121 0.338421
C -4.785570 1.771811 1.397282
C -5.663439 0.155240 -0.326697
C -7.136697 0.476198 -0.208711
C -7.932418 -0.716717 0.352536
C -7.555788 -1.069662 1.786045
C -7.677925 0.863439 -1.589204
H -1.830362 -2.483209 -1.072499
H -2.246997 2.066588 0.656117
H 0.191195 2.368398 0.607809
H 2.573553 2.708007 0.637131
H 1.649656 -2.726642 -1.392670
H 4.250235 0.453754 -2.518403
H 6.300292 -0.229649 0.273402
H 5.592143 -1.766758 -0.184662
H 5.511062 -1.449327 3.670816
H 6.491684 -2.223043 2.403280
H 6.662535 -0.469691 2.737225
H 6.530193 1.930489 -1.130972
H 5.147527 2.972277 -1.632656
H 6.342485 3.553839 -0.460459
H -4.534723 2.759064 0.995629
H -5.799084 1.823322 1.802483
H -4.135546 1.565931 2.255525
H -5.443670 -0.625708 -1.058134
H -7.302216 1.337709 0.445533
H -9.003242 -0.478543 0.328814
H -7.793611 -1.601345 -0.281888
H -7.677638 -0.205866 2.447251
H -8.201413 -1.872511 2.156332
H -6.520661 -1.416949 1.856999
H -7.133194 1.722272 -1.997324
H -8.735759 1.140963 -1.525951
H -7.590249 0.037104 -2.303852
SCF Energy (B3LYP/6-31G**)= -1361.70242366
Number of imaginary frequencies = 0

1a_c085
MMFF Geometry
C -1.500814 -2.389015 -0.108511
C -2.201401 0.234060 -0.010593
N -2.813688 -2.088327 0.042652
C -0.471697 -1.448050 -0.219806
C -0.846487 -0.094093 -0.166336
C -3.150764 -0.781552 0.082464
C 0.143400 0.899623 -0.270236
C 1.499020 0.569115 -0.424643
C 1.867874 -0.778533 -0.472438
C 0.889072 -1.782764 -0.377564
C 3.297125 -1.127394 -0.639916
C 4.356806 -0.013039 -0.634264
C 3.803752 1.398891 -0.632104
C 2.485369 1.647440 -0.537782
O 1.210093 -3.119302 -0.437825
O 3.612438 -2.309511 -0.821224
O 5.057630 -0.192343 -1.888368
C 5.376594 -0.261041 0.484628
C 4.826476 0.044300 1.858626
C 5.807907 0.542427 2.885708
O 3.646793 -0.153843 2.147551
O 4.790556 2.368048 -0.708691
C 4.397476 3.727715 -0.849360
C -4.595572 -0.495236 0.255066
C -5.268785 -1.355857 1.287763
C -5.194734 0.454692 -0.495372
C -6.648339 0.884501 -0.540038
C -6.998046 1.791043 0.653964
C -8.380946 2.420290 0.550289
C -7.626019 -0.277737 -0.738345
H -1.286625 -3.455247 -0.138181
H -2.511830 1.272422 0.051499
H -0.153027 1.947079 -0.227590
H 2.094814 2.658550 -0.523005
H 2.179156 -3.200604 -0.584821
H 5.247157 -1.147344 -1.963671
H 6.267263 0.357646 0.315585
H 5.727303 -1.300262 0.487099
H 5.290770 0.725186 3.831719
H 6.584490 -0.209672 3.044441
H 6.252959 1.479751 2.542689
H 3.791442 3.870627 -1.749866
H 3.865227 4.073686 0.042380
H 5.304365 4.330589 -0.954041
H -4.622571 -1.495074 2.162515
H -6.191377 -0.912866 1.667310
H -5.505752 -2.341429 0.873524
H -4.585081 0.991781 -1.223641
H -6.738322 1.500682 -1.446956
H -6.938546 1.232878 1.595027
H -6.258306 2.599069 0.724294
H -9.169937 1.664376 0.604610
H -8.536854 3.121230 1.376726
H -8.492294 2.973194 -0.387708
H -7.250271 -0.990029 -1.481270
H -8.590195 0.090768 -1.105456
H -7.821818 -0.821806 0.189485
SCF Energy (B3LYP/6-31G**)= -1361.70319497
Number of imaginary frequencies = 0

1a_c086
MMFF Geometry
C -1.751996 -2.608300 0.186958
C -2.447435 -0.045832 -0.393864
N -3.070218 -2.296168 0.168869
C -0.714157 -1.707129 -0.070772
C -1.086380 -0.384011 -0.373359
C -3.404712 -1.018252 -0.111385
C -0.088967 0.567285 -0.656244
C 1.270113 0.225765 -0.625454
C 1.633085 -1.085277 -0.309391
C 0.651965 -2.053066 -0.048804
C 3.070926 -1.423624 -0.262983
C 4.103246 -0.285706 -0.152231
C 3.593318 0.999252 -0.786995
C 2.280369 1.225696 -0.978331
O 0.979939 -3.356808 0.236813
O 3.412542 -2.610730 -0.260148
O 5.264774 -0.748503 -0.861274
C 4.453331 -0.148093 1.333735
C 5.287649 1.070895 1.659527
C 6.743944 1.054667 1.273360
O 4.788728 2.037561 2.240015
O 4.601724 1.889199 -1.110172
C 4.252875 3.174339 -1.608464
C -4.856828 -0.721506 -0.123742
C -5.703281 -1.784076 -0.771953
C -5.332454 0.413352 0.432379
C -6.780973 0.838561 0.543346
C -7.140667 1.917576 -0.496355
C -6.386001 3.235065 -0.365354
C -7.088511 1.287558 1.975385
H -1.542125 -3.649083 0.424522
H -2.758787 0.964309 -0.641847
H -0.382244 1.587190 -0.903799
H 1.914470 2.149664 -1.412591
H 1.944102 -3.481777 0.088010
H 5.284367 -1.721651 -0.766083
H 5.003331 -1.027471 1.692470
H 3.541443 -0.086538 1.942709
H 6.853392 1.130478 0.190460
H 7.252320 1.904324 1.738245
H 7.210353 0.135128 1.635760
H 3.735568 3.093254 -2.569692
H 3.647766 3.725186 -0.881215
H 5.178341 3.734810 -1.770243

H -5.850068 -2.628653 -0.090568
H -6.687161 -1.414171 -1.072765
H -5.228239 -2.155610 -1.687527
H -4.628639 1.105615 0.894403
H -7.435515 -0.019318 0.351917
H -6.967112 1.517064 -1.503506
H -8.215780 2.127497 -0.431246
H -5.306043 3.094208 -0.467990
H -6.705541 3.925126 -1.153346
H -6.584844 3.718342 0.595445
H -6.939198 0.460822 2.679353
H -8.130031 1.614708 2.063623
H -6.445013 2.113149 2.297498
SCF Energy (B3LYP/6-31G**)= -1361.70741687
Number of imaginary frequencies = 0

1a_c087
MMFF Geometry
C -1.837913 -1.486754 -0.963084
C -2.157205 1.080952 -0.143103
N -3.099934 -1.012299 -0.825657
C -0.676740 -0.749946 -0.716572
C -0.854548 0.577460 -0.293197
C -3.256148 0.267408 -0.415427
C 0.275794 1.371019 -0.028641
C 1.576473 0.865982 -0.181155
C 1.751774 -0.457899 -0.597495
C 0.629658 -1.261192 -0.867308
C 3.127340 -0.993590 -0.767977
C 4.341425 -0.075208 -0.483736
C 3.975774 1.326286 -0.038453
C 2.711297 1.736907 0.115459
O 0.752405 -2.563822 -1.293184
O 3.256879 -2.146083 -1.193137
O 5.050758 0.035242 -1.736438
C 5.291384 -0.785321 0.490085
C 4.718562 -0.942618 1.881909
C 5.708010 -1.193524 2.989149
O 3.508779 -0.918889 2.105389
O 4.993939 2.172927 0.384179
C 5.833335 2.617703 -0.680165
C -4.659579 0.757317 -0.286390
C -4.880309 2.238845 -0.430342
C -5.654625 -0.132483 -0.074748
C -7.130419 0.140283 0.111636
C -7.660693 -0.494972 1.412068
C -7.037441 0.102400 2.667809
C -7.900139 -0.407788 -1.092475
H -1.782824 -2.522282 -1.291937
H -2.301251 2.100401 0.199061
H 0.135464 2.398718 0.305316
H 2.510727 2.731861 0.503319
H 1.709870 -2.788594 -1.338263
H 4.409774 0.328228 -2.407307
H 6.235231 -0.230591 0.562493
H 5.554166 -1.785120 0.122578
H 5.179578 -1.287080 3.941937
H 6.250806 -2.120985 2.791288
H 6.404690 -0.354198 3.055373
H 6.586650 1.861081 -0.916697
H 5.253837 2.882824 -1.571241
H 6.358742 3.514390 -0.338163
H -4.276704 2.647080 -1.249173
H -5.914749 2.494174 -0.673538
H -4.614255 2.759356 0.495517
H -5.393986 -1.191227 -0.009475
H -7.328505 1.215039 0.170449
H -8.747014 -0.350287 1.466410
H -7.488397 -1.578742 1.407275
H -7.188926 1.185952 2.702990
H -7.499975 -0.332939 3.559446
H -5.963054 -0.098486 2.718999
H -7.552965 0.054436 -2.023413
H -8.970719 -0.196673 -0.996555
H -7.779146 -1.492833 -1.188645
SCF Energy (B3LYP/6-31G**)= -1361.70258471
Number of imaginary frequencies = 0

1a_c088
MMFF Geometry
C -1.392151 -2.379336 -0.618989
C -2.355349 0.153430 -0.405974
N -2.736151 -2.212400 -0.568536
C -0.456198 -1.341522 -0.571286
C -0.966496 -0.033891 -0.463818
C -3.203644 -0.951246 -0.452068
C -0.076531 1.055692 -0.427725
C 1.310335 0.861081 -0.478150
C 1.809908 -0.441802 -0.565704
C 0.937958 -1.537541 -0.632215
C 3.276094 -0.624120 -0.618613
C 4.164576 0.511624 -0.058389
C 3.536998 1.877226 -0.331117
C 2.209621 2.019436 -0.506315
O 1.401220 -2.824212 -0.764453
O 3.721498 -1.676529 -1.079385
O 5.444563 0.453095 -0.701830
C 4.405079 0.348718 1.444139
C 4.662495 -1.088842 1.831736
C 6.034113 -1.656393 1.581554
O 3.755925 -1.774967 2.307316
O 4.457031 2.909046 -0.350418
C 3.992798 4.236852 -0.564955
C -4.678744 -0.814208 -0.402562
C -5.418635 -1.708456 -1.361007
C -5.262139 0.041259 0.464174
C -6.745313 0.261488 0.671142
C -7.227466 1.564828 0.005208
C -6.611463 2.848392 0.548775
C -7.079832 0.215472 2.165428
H -1.072010 -3.415894 -0.702876
H -2.772785 1.153380 -0.340394
H -0.477595 2.066457 -0.361702
H 1.748415 2.985604 -0.679751
H 2.378561 -2.800127 -0.876784
H 5.317868 0.762185 -1.615723
H 3.533385 0.679538 2.024292
H 5.257698 0.948843 1.785188
H 6.145304 -2.594322 2.133017
H 6.170112 -1.851313 0.516045
H 6.795207 -0.957715 1.937766
H 3.518123 4.330815 -1.546889
H 3.309878 4.545614 0.232951
H 4.859680 4.903824 -0.544041
H -5.469113 -2.731217 -0.972994
H -6.439041 -1.366751 -1.554265
H -4.918907 -1.731846 -2.336560
H -4.627671 0.625956 1.130157
H -7.310027 -0.558370 0.212940
H -7.025923 1.512662 -1.072542
H -8.317630 1.633643 0.110000
H -5.524079 2.856921 0.429578
H -7.010321 3.710540 0.003949
H -6.847321 2.990531 1.607264
H -6.837170 -0.767612 2.584975
H -8.148409 0.391573 2.328852
H -6.521152 0.963566 2.737862
SCF Energy (B3LYP/6-31G**)= -1361.69882474
Number of imaginary frequencies = 0

1a_c089
MMFF Geometry
C -1.527202 -2.351925 -0.175854
C -2.233968 0.264611 0.013629
N -2.842960 -2.058813 -0.035120
C -0.497921 -1.406372 -0.233266
C -0.876245 -0.055660 -0.132934
C -3.182917 -0.754925 0.050892
C 0.112742 0.942797 -0.181635
C 1.469855 0.618850 -0.328198
C 1.844346 -0.725986 -0.421451
C 0.865999 -1.733856 -0.381424

C 3.278912 -1.066991 -0.582503
C 4.341540 0.047176 -0.528696
C 3.766724 1.446079 -0.497631
C 2.456643 1.696318 -0.378035
O 1.189937 -3.066672 -0.488657
O 3.599533 -2.240334 -0.807316
O 5.066455 -0.113603 -1.769420
C 5.331901 -0.216174 0.610851
C 4.732106 0.064271 1.969009
C 5.601214 0.783434 2.965030
O 3.599141 -0.315915 2.263274
O 4.653349 2.512839 -0.410726
C 5.300447 2.779827 -1.655177
C -4.630899 -0.477321 0.210945
C -5.319555 -1.379538 1.196896
C -5.219240 0.501628 -0.510191
C -6.672553 0.931670 -0.561204
C -7.043026 1.788929 0.662566
C -8.424998 2.420685 0.561812
C -7.645135 -0.222418 -0.822248
H -1.310809 -3.415976 -0.243347
H -2.546904 1.299479 0.111769
H -0.183982 1.988116 -0.102111
H 2.107616 2.721132 -0.289388
H 2.162455 -3.142478 -0.612766
H 5.228655 -1.073266 -1.867107
H 6.225190 0.407286 0.477819
H 5.688511 -1.253317 0.607845
H 5.050918 0.926073 3.899131
H 6.495443 0.188743 3.166585
H 5.879354 1.763023 2.568376
H 6.171890 2.130595 -1.777873
H 4.616321 2.678522 -2.505027
H 5.657866 3.813643 -1.629239
H -4.687571 -1.553286 2.075858
H -6.248978 -0.953121 1.578751
H -5.548080 -2.347832 0.739423
H -4.598526 1.068626 -1.205719
H -6.748670 1.583919 -1.443845
H -6.998025 1.193239 1.581162
H -6.305745 2.594076 0.777553
H -9.213632 1.662529 0.572658
H -8.595402 3.087524 1.413230
H -8.521880 3.010949 -0.354781
H -7.256258 -0.903806 -1.587081
H -8.603740 0.159818 -1.189908
H -7.855242 -0.803688 0.079527
SCF Energy (B3LYP/6-31G**)= -1361.69748706
Number of imaginary frequencies = 0

1a_c090
MMFF Geometry
C 1.642924 2.588051 0.225413
C 2.333353 0.039613 -0.421060
N 2.960226 2.271926 0.205549
C 0.603837 1.697676 -0.062268
C 0.973294 0.381604 -0.399048
C 3.292125 1.000870 -0.107137
C -0.025303 -0.558300 -0.714874
C -1.382698 -0.212622 -0.680897
C -1.743269 1.090753 -0.328414
C -0.760819 2.048075 -0.038054
C -3.183247 1.430619 -0.275095
C -4.193568 0.271313 -0.114676
C -3.705297 -0.969629 -0.854136
C -2.397341 -1.190774 -1.081135
O -1.088238 3.344593 0.278813
O -3.500278 2.621208 -0.304631
O -5.448949 0.696508 -0.657037
C -4.384023 0.055697 1.391202
C -5.145385 -1.207625 1.723512
C -6.639998 -1.201633 1.536254
O -4.554037 -2.213031 2.123868
O -4.729798 -1.815464 -1.237755
C -4.395804 -3.119741 -1.699754
C 4.743566 0.699698 -0.119306
C 5.595513 1.776775 -0.735479
C 5.211498 -0.448660 0.415195
C 6.656825 -0.888396 0.527997
C 6.895027 -2.090821 -0.403117
C 8.349979 -2.536099 -0.458068
C 6.970672 -1.213542 1.991681
H 1.434836 3.622614 0.490339
H 2.643125 -0.964458 -0.694186
H 0.265838 -1.571515 -0.990499
H -2.040518 -2.080758 -1.588046
H -2.050859 3.475760 0.117173
H -5.364697 0.666776 -1.626151
H -4.921794 0.897827 1.845019
H -3.417614 -0.004757 1.908857
H -7.073579 -2.064027 2.050714
H -7.064669 -0.294362 1.973153
H -6.888004 -1.260794 0.475046
H -3.860813 -3.069359 -2.653244
H -3.813281 -3.664832 -0.949906
H -5.328856 -3.666211 -1.865696
H 5.744397 2.600782 -0.029769
H 6.578169 1.412953 -1.046387
H 5.123216 2.176793 -1.640463
H 4.503170 -1.149876 0.857367
H 7.330685 -0.078961 0.229961
H 6.274135 -2.941560 -0.094426
H 6.581440 -1.827533 -1.421768
H 8.683591 -2.939032 0.502518
H 8.472133 -3.325245 -1.207074
H 9.005676 -1.704112 -0.733398
H 6.716582 -0.369685 2.643445
H 8.036499 -1.419851 2.131171
H 6.408840 -2.087979 2.338937
SCF Energy (B3LYP/6-31G**)= -1361.70240342
Number of imaginary frequencies = 0

1a_c091
MMFF Geometry
C 1.903337 -1.429006 0.047031
C 2.305543 1.234210 -0.296662
N 3.179522 -0.976032 -0.000259
C 0.767008 -0.624889 -0.068448
C 0.987376 0.752092 -0.245813
C 3.377436 0.351504 -0.171394
C -0.116389 1.614775 -0.372360
C -1.430002 1.128203 -0.313852
C -1.645828 -0.239181 -0.129021
C -0.554410 -1.115689 -0.019738
C -3.039787 -0.742071 -0.085393
C -4.194794 0.254695 0.192434
C -3.807252 1.665350 -0.231783
C -2.549031 2.048092 -0.486225
O -0.725657 -2.471270 0.135436
O -3.216552 -1.954702 -0.233028
O -4.425367 0.312980 1.602333
C -5.450896 -0.212710 -0.572646
C -6.002042 -1.521332 -0.041424
C -6.611932 -2.465660 -1.041248
O -5.999410 -1.771617 1.165941
O -4.827567 2.588850 -0.432050
C -5.348946 3.068903 0.809608
C 4.796100 0.809262 -0.232863
C 5.070717 2.085041 -0.982757
C 5.761831 0.060310 0.344591
C 7.244224 0.351824 0.416506
C 8.039806 -0.462108 -0.622242
C 7.982889 -1.975820 -0.455421
C 7.754918 0.127236 1.843461
H 1.815281 -2.504299 0.186689
H 2.482580 2.297473 -0.420436
H 0.054482 2.681114 -0.516949
H -2.342705 3.065671 -0.804945
H -1.684417 -2.677470 0.056274
H -4.856604 -0.533173 1.856707
H -5.220243 -0.331548 -1.638348
H -6.274914 0.504619 -0.482727

H -6.955627 -3.370008 -0.531663
H -7.464374 -1.983823 -1.526029
H -5.862381 -2.746104 -1.785372
H -6.003481 2.323133 1.269978
H -4.548184 3.359070 1.498764
H -5.951787 3.956608 0.596216
H 4.811746 2.955206 -0.370706
H 6.117532 2.184765 -1.282204
H 4.492809 2.124530 -1.913276
H 5.466557 -0.867767 0.838321
H 7.424025 1.410990 0.199565
H 7.679477 -0.210196 -1.627993
H 9.091254 -0.150083 -0.586582
H 6.958121 -2.351965 -0.526243
H 8.567341 -2.458061 -1.246040
H 8.404333 -2.290613 0.503500
H 7.255706 0.807104 2.543424
H 8.831400 0.319731 1.904479
H 7.572607 -0.894794 2.192283
SCF Energy (B3LYP/6-31G**)= -1361.70714528
Number of imaginary frequencies = 0

1a_c092
MMFF Geometry
C -1.744781 -1.325435 -0.731503
C -2.156989 1.136823 0.337804
N -3.020107 -0.942992 -0.479514
C -0.614453 -0.543514 -0.483061
C -0.839780 0.730747 0.069115
C -3.222571 0.283166 0.055290
C 0.256542 1.573051 0.332433
C 1.568651 1.159657 0.067414
C 1.785352 -0.115687 -0.465724
C 0.704483 -0.958538 -0.757634
C 3.176517 -0.528955 -0.748668
C 4.331131 0.213053 -0.046190
C 3.955675 1.658462 0.219482
C 2.683643 2.076755 0.308411
O 0.883754 -2.199760 -1.318680
O 3.392668 -1.453378 -1.538247
O 5.449609 0.184382 -0.950033
C 4.751956 -0.453415 1.265640
C 4.730267 -1.960603 1.184711
C 5.973171 -2.656429 0.698908
O 3.708896 -2.586639 1.470593
O 4.994901 2.515632 0.548795
C 5.371916 3.321651 -0.568149
C -4.638757 0.659942 0.335635
C -4.871006 1.663190 1.433012
C -5.631374 0.074899 -0.370985
C -7.121575 0.312128 -0.268221
C -7.861347 -0.943471 0.229200
C -7.490498 -1.334839 1.654215
C -7.659498 0.726804 -1.642023
H -1.652164 -2.322517 -1.156806
H -2.340281 2.122437 0.752700
H 0.078849 2.564434 0.747973
H 2.461648 3.103986 0.582572
H 1.837828 -2.326186 -1.517688
H 5.279223 -0.541332 -1.584505
H 4.075697 -0.179249 2.086422
H 5.758650 -0.141256 1.569611
H 5.862435 -3.736324 0.831826
H 6.130084 -2.443653 -0.360304
H 6.834760 -2.323584 1.282923
H 5.672117 2.709993 -1.424573
H 4.557983 3.996566 -0.853669
H 6.229069 3.931324 -0.267707
H -4.667191 2.678121 1.076232
H -5.892445 1.643833 1.820744
H -4.225250 1.457088 2.294417
H -5.357872 -0.662392 -1.129158
H -7.344185 1.135896 0.416968
H -8.942876 -0.761651 0.194722
H -7.664497 -1.792535 -0.437633
H -7.669757 -0.506767 2.347287
H -8.098160 -2.185413 1.979456
H -6.439534 -1.629358 1.730799
H -7.154921 1.629432 -2.004724
H -8.731636 0.944997 -1.587772
H -7.515497 -0.063502 -2.387622
SCF Energy (B3LYP/6-31G**)= -1361.70147137
Number of imaginary frequencies = 0

1a_c093
MMFF Geometry
C 1.780998 2.502265 0.114053
C 2.461068 -0.097489 -0.293836
N 3.097295 2.181644 0.118454
C 0.737665 1.592537 -0.084930
C 1.102270 0.249778 -0.298499
C 3.423912 0.885727 -0.075084
C 0.099979 -0.712286 -0.519474
C -1.255981 -0.360061 -0.514640
C -1.613850 0.972035 -0.285755
C -0.626499 1.948196 -0.087887
C -3.052162 1.324912 -0.266692
C -4.099923 0.205487 -0.099918
C -3.577112 -1.103720 -0.651767
C -2.271176 -1.372402 -0.797457
O -0.945420 3.270365 0.110006
O -3.383936 2.512826 -0.337527
O -5.243935 0.629373 -0.857722
C -4.469380 0.164176 1.386286
C -5.326848 -1.015296 1.788800
C -6.754304 -1.052223 1.307712
O -4.876702 -1.895783 2.524833
O -4.519636 -2.088859 -0.901389
C -4.818674 -2.183494 -2.294466
C 4.874131 0.579000 -0.065763
C 5.725030 1.586865 -0.790513
C 5.339144 -0.513745 0.576914
C 6.779736 -0.955286 0.720795
C 7.025674 -2.308844 0.028911
C 6.841131 -2.252945 -1.482527
C 7.128927 -1.056109 2.209323
H 1.577519 3.557768 0.281973
H 2.766552 -1.123891 -0.473030
H 0.385647 -1.748835 -0.697275
H -1.947723 -2.354003 -1.132048
H -1.909433 3.391562 -0.041587
H -5.279684 1.604749 -0.785847
H -5.010229 1.072571 1.681344
H -3.563431 0.130081 2.006356
H -6.794144 -1.251334 0.235966
H -7.293287 -1.847133 1.831352
H -7.242275 -0.100470 1.532524
H -5.167532 -1.227245 -2.696927
H -3.947319 -2.534645 -2.857194
H -5.620454 -2.917619 -2.416181
H 5.866057 2.483418 -0.177677
H 6.711713 1.198786 -1.054750
H 5.255317 1.883692 -1.735661
H 4.629404 -1.160896 1.093182
H 7.463503 -0.219904 0.285635
H 8.051392 -2.638457 0.236736
H 6.359365 -3.076488 0.442517
H 7.487321 -1.490082 -1.928286
H 7.101128 -3.218658 -1.927832
H 5.804752 -2.030657 -1.754051
H 6.975157 -0.095711 2.714147
H 8.179565 -1.336411 2.341977
H 6.514562 -1.807549 2.718346
SCF Energy (B3LYP/6-31G**)= -1361.70263502
Number of imaginary frequencies = 0

1a_c094
MMFF Geometry
C -1.717099 -1.271685 -1.088954
C -2.188157 1.178541 -0.016317
N -3.004566 -0.881386 -0.926716
C -0.602228 -0.503940 -0.744886

C -0.857887 0.763312 -0.189787
C -3.236489 0.339437 -0.391716
C 0.222459 1.588849 0.173652
C 1.547292 1.166523 0.002700
C 1.792860 -0.101148 -0.536192
C 0.730122 -0.927606 -0.925306
C 3.197865 -0.523753 -0.719226
C 4.301591 0.193028 0.083978
C 3.919923 1.637207 0.347545
C 2.648689 2.067220 0.346256
O 0.940849 -2.160991 -1.492651
O 3.465782 -1.435692 -1.507449
O 5.486095 0.169192 -0.731564
C 4.613827 -0.501850 1.411311
C 4.586375 -2.006993 1.299660
C 5.857832 -2.705432 0.898938
O 3.540773 -2.628534 1.492664
O 4.938383 2.477149 0.772306
C 5.408069 3.299379 -0.296655
C -4.666233 0.738099 -0.241407
C -4.967105 2.212453 -0.248033
C -5.612723 -0.220545 -0.133203
C -7.103657 -0.047053 0.051701
C -7.614396 -0.831917 1.276027
C -7.041292 -0.325078 2.593923
C -7.826390 -0.517723 -1.212755
H -1.601001 -2.262991 -1.521931
H -2.394137 2.148915 0.423163
H 0.022252 2.574271 0.593110
H 2.415582 3.091483 0.622283
H 1.906174 -2.293584 -1.620756
H 5.358646 -0.543148 -1.390793
H 3.878089 -0.236638 2.182363
H 5.596368 -0.205073 1.798365
H 5.728411 -3.786510 1.002012
H 6.098719 -2.474529 -0.140524
H 6.673796 -2.391681 1.554650
H 5.768139 2.700321 -1.138784
H 4.624851 3.987907 -0.631348
H 6.244873 3.894430 0.080640
H -4.374535 2.729800 -1.011378
H -6.010325 2.433448 -0.487409
H -4.744971 2.654972 0.728570
H -5.294513 -1.265022 -0.164644
H -7.361699 1.004860 0.208952
H -8.707675 -0.751993 1.324342
H -7.382193 -1.899326 1.171224
H -7.253092 0.740322 2.729325
H -7.490282 -0.868348 3.431622
H -5.958154 -0.471634 2.645081
H -7.493539 0.049804 -2.089082
H -8.908164 -0.374882 -1.116458
H -7.644203 -1.580402 -1.409554
SCF Energy (B3LYP/6-31G**)= -1361.70152375
Number of imaginary frequencies = 0

1a_c095
MMFF Geometry
C 1.677628 2.450452 0.061970
C 2.285726 -0.189160 0.290035
N 2.963258 2.114894 0.326816
C 0.631876 1.536970 -0.102888
C 0.958168 0.172867 0.018422
C 3.256384 0.800784 0.430233
C -0.049458 -0.796597 -0.140414
C -1.375352 -0.427551 -0.403203
C -1.694927 0.928843 -0.508453
C -0.699588 1.907710 -0.377450
C -3.105238 1.300189 -0.763416
C -4.199548 0.265912 -0.412425
C -3.699604 -1.151067 -0.671784
C -2.390007 -1.458816 -0.634301
O -0.982920 3.246299 -0.505839
O -3.339495 2.432939 -1.188758
O -5.343960 0.533782 -1.230671
C -4.597599 0.521053 1.045960
C -5.476089 -0.564487 1.625575
C -6.929995 -0.582033 1.232098
O -5.008434 -1.414065 2.387368
O -4.717612 -2.051217 -0.927935
C -4.410565 -3.441132 -0.926605
C 4.673276 0.479212 0.724939
C 5.313230 1.380701 1.746415
C 5.296096 -0.531009 0.080770
C 6.741042 -0.963305 0.223628
C 7.424760 -0.918858 -1.156778
C 8.918179 -1.211131 -1.103222
C 6.785110 -2.358845 0.849940
H 1.501537 3.521285 -0.016231
H 2.557842 -1.233856 0.405334
H 0.209927 -1.851760 -0.058620
H -2.028097 -2.468178 -0.797266
H -1.905717 3.343459 -0.835973
H -5.136442 0.209911 -2.124651
H -5.133986 1.473081 1.147308
H -3.712545 0.598390 1.691152
H -7.482076 -1.236934 1.912317
H -7.348037 0.424495 1.312786
H -7.040976 -0.955731 0.212685
H -3.754684 -3.695243 -1.765140
H -3.965827 -3.744233 0.026847
H -5.347271 -3.991926 -1.052931
H 4.625502 1.576956 2.577445
H 6.208779 0.944238 2.195577
H 5.591348 2.338446 1.294074
H 4.738891 -1.102851 -0.661812
H 7.285375 -0.281626 0.884354
H 6.948261 -1.631193 -1.842398
H 7.288417 0.078075 -1.595926
H 9.115160 -2.242856 -0.798125
H 9.363273 -1.069263 -2.093400
H 9.425866 -0.538505 -0.404826
H 6.232658 -2.381042 1.796387
H 7.813494 -2.662134 1.069666
H 6.344863 -3.111389 0.186046
SCF Energy (B3LYP/6-31G**)= -1361.70242876
Number of imaginary frequencies = 0

1a_c096
MMFF Geometry
C -1.563988 -2.293335 -0.057927
C -2.164785 0.318314 -0.503011
N -2.872183 -1.941198 -0.097806
C -0.491934 -1.411519 -0.230926
C -0.815515 -0.063369 -0.463660
C -3.158708 -0.638941 -0.309226
C 0.218617 0.870388 -0.655565
C 1.568376 0.486882 -0.612762
C 1.885885 -0.853192 -0.372163
C 0.863105 -1.799854 -0.189694
C 3.309404 -1.258647 -0.330828
C 4.407714 -0.186809 -0.428827
C 3.913740 1.211541 -0.745047
C 2.603265 1.502241 -0.828504
O 1.134514 -3.130974 0.028206
O 3.592633 -2.460326 -0.257005
O 5.214059 -0.616129 -1.552063
C 5.308692 -0.240193 0.811641
C 4.646580 0.332233 2.043596
C 5.548532 0.991441 3.052651
O 3.437843 0.220735 2.245838
O 4.941367 2.126646 -0.904654
C 4.617658 3.442643 -1.336350
C -4.601238 -0.294089 -0.348617
C -5.437360 -1.269300 -1.129970
C -5.053958 0.798263 0.304508
C -6.465176 1.334203 0.447371
C -7.428909 0.327595 1.103842
C -8.757824 0.950008 1.513701
C -6.986592 1.920349 -0.866893
H -1.391033 -3.351877 0.124245
H -2.438321 1.350885 -0.696494

H -0.038365 1.913226 -0.838054
H 2.253743 2.505992 -1.041609
H 2.109094 -3.259433 -0.003307
H 5.371149 -1.572019 -1.428721
H 6.235220 0.315609 0.618573
H 5.615740 -1.267501 1.042609
H 4.953920 1.361979 3.892238
H 6.274424 0.264281 3.424469
H 6.062866 1.836102 2.587550
H 4.103495 3.424101 -2.302739
H 4.019676 3.964044 -0.582200
H 5.553728 3.994556 -1.462945
H -5.578945 -2.196594 -0.564577
H -6.424334 -0.879138 -1.381582
H -4.955617 -1.515358 -2.083526
H -4.330441 1.395817 0.861649
H -6.380856 2.181424 1.144084
H -7.638170 -0.513381 0.435342
H -6.957319 -0.093905 2.000819
H -9.331366 1.279712 0.642520
H -9.365715 0.215077 2.051322
H -8.603582 1.808802 2.174466
H -6.232400 2.559498 -1.339951
H -7.870949 2.541956 -0.692290
H -7.267164 1.143786 -1.583851
SCF Energy (B3LYP/6-31G**)= -1361.70349347
Number of imaginary frequencies = 0

1a_c097
MMFF Geometry
C 1.831687 2.295043 0.061371
C 2.386210 -0.344426 0.398077
N 3.114396 1.942951 0.318428
C 0.763050 1.399200 -0.043914
C 1.062199 0.035174 0.133414
C 3.380673 0.628445 0.476157
C 0.031137 -0.917077 0.037577
C -1.290450 -0.530508 -0.220814
C -1.584678 0.826628 -0.383633
C -0.565496 1.787368 -0.311722
C -2.989331 1.220383 -0.639590
C -4.120379 0.217617 -0.333171
C -3.623651 -1.206491 -0.463573
C -2.330011 -1.547087 -0.366777
O -0.818818 3.126009 -0.493041
O -3.232248 2.360277 -1.049335
O -5.137443 0.468827 -1.315622
C -4.663196 0.574704 1.054219
C -5.638934 -0.430053 1.625929
C -6.999009 -0.541730 0.987048
O -5.338515 -1.104318 2.613220
O -4.593143 -2.190755 -0.575263
C -4.734028 -2.633186 -1.925611
C 4.795464 0.288343 0.759246
C 5.476204 1.221563 1.724092
C 5.383221 -0.763265 0.149429
C 6.820038 -1.220088 0.282750
C 7.524281 -1.272345 -1.087013
C 7.659489 0.096227 -1.743381
C 6.848341 -2.601786 0.941178
H 1.678033 3.365029 -0.061925
H 2.637142 -1.388959 0.555738
H 0.267184 -1.973290 0.165450
H -2.034832 -2.592096 -0.397397
H -1.748134 3.236107 -0.795117
H -5.117762 1.430311 -1.497187
H -5.172909 1.546653 1.037643
H -3.840315 0.670760 1.775354
H -7.648211 -1.153501 1.620182
H -7.444900 0.451988 0.896518
H -6.925641 -1.011857 0.005484
H -4.970800 -1.802888 -2.598386
H -3.827999 -3.146805 -2.264044
H -5.562372 -3.346818 -1.959939
H 4.811387 1.469889 2.559795
H 6.372992 0.787758 2.173173
H 5.763239 2.151561 1.222085
H 4.799942 -1.354177 -0.557260
H 7.393160 -0.540189 0.920638
H 8.530012 -1.691939 -0.958909
H 6.988052 -1.945079 -1.768291
H 8.187581 0.795156 -1.086958
H 8.228758 0.010836 -2.674571
H 6.682581 0.523434 -1.989167
H 6.373104 -2.575677 1.928250
H 7.879727 -2.944273 1.078525
H 6.324538 -3.348837 0.333917
SCF Energy (B3LYP/6-31G**)= -1361.70270768
Number of imaginary frequencies = 0

1a_c098
MMFF Geometry
C -1.834435 -1.471031 -0.726336
C -2.077645 1.041167 0.272456
N -3.079620 -1.008070 -0.458239
C -0.654708 -0.748551 -0.530572
C -0.793311 0.551138 -0.015967
C -3.197411 0.243065 0.042728
C 0.356652 1.332303 0.196224
C 1.639028 0.840045 -0.092120
C 1.775758 -0.458842 -0.595161
C 0.633192 -1.246944 -0.819466
C 3.131532 -0.983072 -0.905865
C 4.371126 -0.111667 -0.593957
C 4.043904 1.280182 -0.101209
C 2.796247 1.701266 0.145494
O 0.716862 -2.520938 -1.332522
O 3.220557 -2.087085 -1.452125
O 5.093883 0.036923 -1.835296
C 5.310874 -0.876494 0.346642
C 4.766397 -1.032089 1.748812
C 5.778552 -1.248775 2.842543
O 3.559736 -1.037852 1.990261
O 5.131808 2.080110 0.218108
C 5.349587 3.093754 -0.763304
C -4.581746 0.709801 0.345590
C -4.727092 1.754296 1.419165
C -5.624219 0.164918 -0.320140
C -7.095017 0.491785 -0.188544
C -7.893337 -0.704330 0.362102
C -7.509466 -1.078001 1.788420
C -7.642956 0.899881 -1.560353
H -1.809465 -2.484690 -1.120523
H -2.192427 2.047171 0.662083
H 0.246149 2.339407 0.597114
H 2.626072 2.689021 0.563732
H 1.665321 -2.733005 -1.489384
H 4.457460 0.333131 -2.508047
H 6.277632 -0.359649 0.403628
H 5.525295 -1.880096 -0.041221
H 5.267319 -1.345215 3.804387
H 6.339980 -2.165125 2.644913
H 6.456054 -0.392634 2.889978
H 5.504002 2.660355 -1.756474
H 4.516725 3.804276 -0.785304
H 6.255302 3.640712 -0.485542
H -4.474393 2.745538 1.028657
H -5.737563 1.805110 1.831994
H -4.072143 1.534298 2.270132
H -5.413079 -0.607581 -1.063014
H -7.251908 1.345460 0.477991
H -8.963041 -0.460408 0.348362
H -7.763138 -1.581240 -0.284740
H -7.622654 -0.222361 2.461662
H -8.156879 -1.882350 2.152273
H -6.475721 -1.431456 1.848187
H -7.096381 1.761219 -1.960654
H -8.698918 1.181930 -1.486706
H -7.564075 0.082573 -2.286315
SCF Energy (B3LYP/6-31G**)= -1361.70214649
Number of imaginary frequencies = 0

1a_c099
MMFF Geometry
C 1.585515 2.258360 0.030032
C 2.194759 -0.334135 -0.506869
N 2.894994 1.915896 -0.040683
C 0.515978 1.376047 -0.155303
C 0.844147 0.037739 -0.435827
C 3.185878 0.622680 -0.297347
C -0.186623 -0.896203 -0.642370
C -1.536457 -0.521111 -0.568796
C -1.860476 0.808867 -0.279493
C -0.840633 1.755062 -0.081427
C -3.287979 1.205448 -0.207520
C -4.387712 0.134200 -0.335731
C -3.872236 -1.235475 -0.718537
C -2.568491 -1.531485 -0.795158
O -1.116064 3.076864 0.182803
O -3.577523 2.402650 -0.093706
O -5.213698 0.622224 -1.417582
C -5.259726 0.112721 0.924380
C -4.553190 -0.519032 2.101132
C -5.360899 -1.450862 2.963643
O -3.382209 -0.250858 2.368744
O -4.791951 -2.272479 -0.820379
C -5.557936 -2.198482 -2.023076
C 4.629684 0.288270 -0.368580
C 5.448896 1.293564 -1.129408
C 5.098060 -0.822018 0.241874
C 6.514164 -1.353755 0.346932
C 7.481628 -0.363291 1.022204
C 8.819817 -0.990659 1.392414
C 7.019787 -1.893435 -0.993151
H 1.409357 3.309391 0.248870
H 2.471075 -1.358495 -0.736846
H 0.071480 -1.931892 -0.860987
H -2.256653 -2.550552 -1.005336
H -2.091779 3.198990 0.179833
H -5.343225 1.577380 -1.251163
H -6.186949 -0.439300 0.724620
H -5.570821 1.121855 1.220541
H -4.735725 -1.839686 3.772153
H -6.205232 -0.908909 3.396444
H -5.718092 -2.290013 2.361536
H -6.408593 -1.523916 -1.891496
H -4.948499 -1.894232 -2.881304
H -5.955038 -3.197524 -2.226600
H 6.434352 0.917911 -1.407644
H 4.952088 1.567623 -2.067472
H 5.593335 2.202775 -0.536060
H 4.386066 -1.442041 0.789285
H 6.444701 -2.223795 1.016641
H 7.676453 0.500349 0.378704
H 7.020713 0.025780 1.939160
H 9.382530 -1.288204 0.502789
H 9.431286 -0.270005 1.945094
H 8.679991 -1.871519 2.026812
H 6.262434 -2.521366 -1.476078
H 7.910065 -2.515011 -0.851668
H 7.285572 -1.092152 -1.688207
SCF Energy (B3LYP/6-31G**)= -1361.69779043
Number of imaginary frequencies = 0

1a_c100
MMFF Geometry
C 1.929903 1.715791 0.045739
C 2.117060 -0.985388 -0.164960
N 3.159054 1.151908 0.125625
C 0.739360 1.008608 -0.137208
C 0.848169 -0.389210 -0.247113
C 3.249736 -0.194151 0.021965
C -0.314093 -1.158040 -0.444121
C -1.579119 -0.558447 -0.516741
C -1.680237 0.828739 -0.391951
C -0.531081 1.613641 -0.219171
C -3.023023 1.442957 -0.451750
C -4.261872 0.553311 -0.235956
C -4.006617 -0.880978 -0.673967
C -2.761689 -1.382111 -0.777741
O -0.598362 2.982797 -0.121018
O -3.122685 2.662666 -0.619869
O -5.297672 1.130761 -1.048167
C -4.655311 0.691724 1.239057
C -5.718395 -0.286648 1.687124
C -7.136953 -0.042027 1.242123
O -5.428579 -1.242971 2.409476
O -5.166111 -1.593976 -0.920007
C -5.071024 -2.978296 -1.230090
C 4.617918 -0.778451 0.131203
C 4.716195 -2.196634 0.625439
C 5.690900 -0.018006 -0.180568
C 7.152129 -0.412955 -0.162182
C 7.855386 0.292037 1.011448
C 9.305714 -0.134130 1.193360
C 7.783957 -0.065006 -1.513871
H 1.928468 2.799783 0.137753
H 2.211269 -2.062078 -0.261351
H -0.225478 -2.239677 -0.542565
H -2.579330 -2.410226 -1.070778
H -1.515798 3.271415 -0.326807
H -5.125440 2.092833 -1.083230
H -5.026748 1.701592 1.455141
H -3.783074 0.539602 1.888856
H -7.809686 -0.706474 1.792049
H -7.418556 0.990772 1.462233
H -7.242751 -0.241423 0.174672
H -4.532749 -3.131697 -2.170764
H -4.598665 -3.531230 -0.411787
H -6.086524 -3.365151 -1.356147
H 4.486446 -2.901973 -0.179892
H 5.706906 -2.443041 1.016242
H 4.021299 -2.371619 1.454889
H 5.518198 1.012669 -0.498269
H 7.256317 -1.495141 -0.033984
H 7.818387 1.381026 0.880024
H 7.316906 0.070436 1.942154
H 9.926433 0.182157 0.350123
H 9.719929 0.323382 2.097679
H 9.386186 -1.220762 1.296828
H 7.216740 -0.512530 -2.338164
H 8.807630 -0.446475 -1.581738
H 7.815496 1.018288 -1.676265
SCF Energy (B3LYP/6-31G**)= -1361.70945057
Number of imaginary frequencies = 0

1a_c101
MMFF Geometry
C -1.460588 -2.396227 0.005769
C -2.439338 0.130688 -0.205222
N -2.801702 -2.242129 -0.112393
C -0.534915 -1.348384 0.027841
C -1.053046 -0.044258 -0.082963
C -3.277907 -0.982245 -0.209366
C -0.172989 1.053626 -0.062851
C 1.211840 0.870940 0.051551
C 1.720033 -0.426100 0.145966
C 0.856778 -1.531169 0.153697
C 3.183586 -0.593219 0.267033
C 4.089339 0.544891 -0.263100
C 3.435141 1.903013 0.008443
C 2.100252 2.032556 0.135033
O 1.328444 -2.815611 0.284479
O 3.606781 -1.648111 0.745077
O 4.196460 0.447238 -1.686800
C 5.477892 0.447076 0.401853
C 6.210690 -0.824695 0.021687
C 7.128353 -1.421087 1.054030
O 6.101724 -1.313765 -1.105100
O 4.335057 2.954533 0.060018
C 3.829343 4.283348 0.110298
C -4.749256 -0.859855 -0.342953
C -5.374413 -1.873841 -1.263084
C -5.425847 0.084234 0.345731

C -6.922955 0.315089 0.360722
C -7.236933 1.651019 -0.336590
C -8.727878 1.921768 -0.484766
C -7.422285 0.281918 1.808535
H -1.133958 -3.431056 0.084242
H -2.859976 1.125905 -0.310555
H -0.578085 2.062050 -0.137689
H 1.624844 2.992920 0.297600
H 2.290633 -2.776680 0.485531
H 4.774428 -0.323580 -1.878204
H 5.378030 0.485882 1.493229
H 6.137176 1.264468 0.086043
H 7.581177 -2.335173 0.660260
H 7.919236 -0.707069 1.295938
H 6.557286 -1.671440 1.951467
H 3.199536 4.497269 -0.759293
H 3.284727 4.458103 1.043569
H 4.681997 4.968368 0.085729
H -5.473294 -2.841270 -0.759610
H -6.363422 -1.571298 -1.616608
H -4.763252 -2.012381 -2.162730
H -4.870915 0.754140 1.003016
H -7.442266 -0.486640 -0.173744
H -6.776721 2.484230 0.209779
H -6.790423 1.649538 -1.339553
H -9.208259 2.068666 0.486848
H -8.886710 2.831920 -1.072199
H -9.228318 1.095514 -0.999365
H -7.116182 -0.644956 2.307192
H -8.515115 0.325796 1.851035
H -7.027374 1.122889 2.389651
SCF Energy (B3LYP/6-31G**)= -1361.71223890
Number of imaginary frequencies = 0

1a_c102
MMFF Geometry
C -1.648503 -1.453598 -0.549954
C -2.035626 1.123270 0.215925
N -2.917106 -1.040742 -0.311960
C -0.513512 -0.648169 -0.432450
C -0.725529 0.685112 -0.037053
C -3.106864 0.242413 0.071977
C 0.376418 1.552255 0.086482
C 1.682142 1.106796 -0.160279
C 1.885167 -0.224700 -0.534755
C 0.797799 -1.095407 -0.690556
C 3.270260 -0.673667 -0.790877
C 4.421097 0.135638 -0.147642
C 4.078317 1.623356 -0.099187
C 2.801827 2.051541 -0.089015
O 0.964833 -2.395931 -1.100719
O 3.444185 -1.680837 -1.479059
O 5.604464 -0.041723 -0.937564
C 4.739637 -0.363248 1.263608
C 4.714980 -1.871022 1.358769
C 5.909965 -2.633520 0.852169
O 3.723837 -2.447935 1.810004
O 5.192032 2.440714 -0.044089
C 5.005058 3.849050 0.034609
C -4.515076 0.653956 0.341839
C -4.720643 1.783831 1.314381
C -5.526916 -0.010704 -0.259075
C -7.013481 0.250688 -0.145011
C -7.668341 -0.879811 0.668699
C -9.147277 -0.648289 0.946738
C -7.610678 0.378500 -1.550118
H -1.565319 -2.496039 -0.850249
H -2.210153 2.153044 0.509476
H 0.206372 2.588448 0.376704
H 2.544217 3.103592 -0.033636
H 1.912804 -2.540241 -1.321808
H 5.473590 0.458056 -1.762113
H 4.007093 0.009737 1.991714
H 5.724729 -0.019063 1.602072
H 5.862019 -3.665127 1.212275
H 5.916589 -2.637213 -0.239498
H 6.828185 -2.180177 1.233815
H 4.485276 4.224269 -0.852733
H 4.468392 4.121358 0.949051
H 5.992731 4.318065 0.070638
H -4.541206 2.748208 0.827945
H -5.728451 1.802505 1.737415
H -4.043744 1.691235 2.171541
H -5.276069 -0.840867 -0.923258
H -7.198535 1.201692 0.364689
H -7.549731 -1.840491 0.151431
H -7.152632 -0.977549 1.632947
H -9.738397 -0.675951 0.026852
H -9.530936 -1.431185 1.608978
H -9.308922 0.317478 1.435734
H -7.074143 1.133261 -2.136581
H -8.659558 0.688411 -1.507267
H -7.560603 -0.570193 -2.096350
SCF Energy (B3LYP/6-31G**)= -1361.70069612
Number of imaginary frequencies = 0

1a_c103
MMFF Geometry
C 1.512005 -2.318459 -0.224657
C 2.414906 0.225988 -0.537711
N 2.849859 -2.126619 -0.323062
C 0.553404 -1.301530 -0.272797
C 1.032300 0.011699 -0.438814
C 3.288276 -0.857626 -0.467611
C 0.116650 1.077764 -0.511699
C -1.263072 0.857317 -0.402524
C -1.730015 -0.445703 -0.219134
C -0.834679 -1.524001 -0.173619
C -3.189219 -0.652847 -0.107739
C -4.057198 0.541772 0.357314
C -3.497693 1.847872 -0.214797
C -2.197920 1.976000 -0.544847
O -1.271022 -2.820228 -0.036644
O -3.633150 -1.777338 -0.350315
O -3.966304 0.674500 1.779299
C -5.518454 0.315944 -0.082566
C -6.143944 -0.888862 0.592790
C -7.170360 -1.658475 -0.193139
O -5.864125 -1.187585 1.756050
O -4.435626 2.862734 -0.307045
C -3.992352 4.173154 -0.639209
C 4.757994 -0.692936 -0.572481
C 5.438045 -1.729175 -1.426336
C 5.385235 0.304039 0.087835
C 6.872543 0.588513 0.131081
C 7.348375 0.590074 1.596867
C 8.856652 0.738172 1.744388
C 7.146763 1.920322 -0.570949
H 1.216672 -3.358243 -0.100780
H 2.806287 1.228227 -0.682076
H 0.489761 2.091218 -0.654254
H -1.785462 2.904449 -0.922477
H -2.252439 -2.828824 -0.103605
H -4.483014 -0.064224 2.169362
H -5.569302 0.179161 -1.169434
H -6.159274 1.163261 0.189324
H -7.530400 -2.503795 0.399705
H -8.013289 -1.005322 -0.431253
H -6.717728 -2.041725 -1.110980
H -3.259431 4.536283 0.088505
H -3.585897 4.201350 -1.655154
H -4.859341 4.839550 -0.605749
H 4.857179 -1.926738 -2.334991
H 6.428185 -1.415496 -1.766150
H 5.549136 -2.669178 -0.875615
H 4.791181 0.982011 0.701535
H 7.431004 -0.190368 -0.397125
H 6.856371 1.395132 2.157546
H 7.050387 -0.352312 2.074807
H 9.199195 1.720061 1.405420
H 9.142027 0.634297 2.796271
H 9.385230 -0.031354 1.173106

H 6.737235 1.918769 -1.587681
H 8.221415 2.109131 -0.656469
H 6.697673 2.760256 -0.028985
SCF Energy (B3LYP/6-31G**)= -1361.71221382
Number of imaginary frequencies = 0

1a_c104
MMFF Geometry
C -1.339002 -2.323095 -0.634809
C -2.293230 0.209971 -0.388004
N -2.682376 -2.152015 -0.583926
C -0.399231 -1.289607 -0.570810
C -0.904952 0.018298 -0.445766
C -3.145503 -0.890725 -0.451143
C -0.010975 1.103930 -0.391379
C 1.375342 0.905406 -0.442319
C 1.868819 -0.397965 -0.548780
C 0.994289 -1.489921 -0.631944
C 3.331348 -0.581967 -0.610518
C 4.239488 0.541224 -0.073670
C 3.608417 1.913315 -0.279135
C 2.279130 2.060939 -0.438579
O 1.453307 -2.776259 -0.779885
O 3.797748 -1.622749 -1.082680
O 5.464335 0.479535 -0.827277
C 4.582665 0.360759 1.406969
C 4.833403 -1.082551 1.771440
C 6.222650 -1.627429 1.575763
O 3.911419 -1.792589 2.175366
O 4.521782 2.951290 -0.232093
C 4.069404 4.273247 -0.495546
C -4.620392 -0.749108 -0.402236
C -5.360195 -1.626403 -1.376199
C -5.201039 0.091869 0.480366
C -6.683271 0.319926 0.694515
C -7.050320 1.740070 0.227596
C -8.543811 2.030339 0.285959
C -7.019777 0.086563 2.170622
H -1.022803 -3.359546 -0.733120
H -2.706973 1.210517 -0.309260
H -0.408165 2.115152 -0.310163
H 1.818486 3.035244 -0.559000
H 2.430414 -2.757089 -0.884579
H 5.459211 -0.370586 -1.311054
H 3.761534 0.704647 2.050085
H 5.468648 0.942863 1.688904
H 6.295661 -2.614868 2.040262
H 6.440970 -1.719127 0.510048
H 6.949702 -0.967179 2.055002
H 3.627184 4.344915 -1.494484
H 3.361901 4.603325 0.271715
H 4.936880 4.938966 -0.461911
H -5.418537 -2.653741 -1.001558
H -6.377187 -1.277155 -1.572151
H -4.854825 -1.640605 -2.349067
H -4.565888 0.663474 1.157646
H -7.271770 -0.399235 0.116094
H -6.518717 2.488863 0.828645
H -6.717651 1.877393 -0.809604
H -8.911195 2.041410 1.316146
H -8.751580 3.013407 -0.148886
H -9.111762 1.284069 -0.278364
H -6.675758 -0.900891 2.499314
H -8.100312 0.125250 2.340785
H -6.549106 0.838286 2.814255
SCF Energy (B3LYP/6-31G**)= -1361.70435728
Number of imaginary frequencies = 0

1a_c105
MMFF Geometry
C 1.916818 1.798217 -0.272678
C 2.136656 -0.894179 -0.552536
N 3.158476 1.256973 -0.294943
C 0.728974 1.071580 -0.381954
C 0.854511 -0.321683 -0.526229
C 3.265713 -0.084592 -0.434959
C -0.305315 -1.109843 -0.646235
C -1.582907 -0.533918 -0.611594
C -1.699370 0.848885 -0.455530
C -0.554795 1.653033 -0.356422
C -3.053675 1.437910 -0.402488
C -4.256423 0.521202 -0.109604
C -4.009102 -0.901039 -0.589367
C -2.766786 -1.376281 -0.795595
O -0.639229 3.018741 -0.228055
O -3.187751 2.658065 -0.540910
O -5.360440 1.091086 -0.832403
C -4.540395 0.629328 1.392823
C -5.550632 -0.374632 1.902385
C -7.002292 -0.148856 1.567888
O -5.191886 -1.336452 2.585415
O -5.170770 -1.632663 -0.758444
C -5.074335 -3.010543 -1.095610
C 4.649759 -0.640521 -0.470471
C 4.843142 -1.940579 -1.203586
C 5.654560 0.045190 0.118618
C 7.115165 -0.338959 0.222674
C 7.549970 -0.292067 1.700742
C 8.970190 -0.790680 1.931055
C 7.944120 0.599029 -0.657164
H 1.902659 2.880068 -0.159080
H 2.241947 -1.969648 -0.650992
H -0.204855 -2.187878 -0.769661
H -2.588664 -2.396151 -1.118517
H -1.574666 3.292934 -0.358875
H -5.209352 2.056858 -0.862900
H -4.911262 1.628903 1.653213
H -3.619448 0.482553 1.972902
H -7.621072 -0.832153 2.156699
H -7.283588 0.875850 1.823376
H -7.184247 -0.335872 0.508490
H -4.606481 -3.140692 -2.076584
H -4.531088 -3.565345 -0.323885
H -6.089264 -3.415185 -1.150135
H 4.266815 -1.954081 -2.135883
H 5.881323 -2.112878 -1.498710
H 4.525232 -2.784103 -0.582071
H 5.416437 0.996283 0.600183
H 7.271237 -1.362335 -0.132512
H 7.465187 0.729910 2.091817
H 6.869801 -0.913637 2.297697
H 9.708364 -0.129498 1.468159
H 9.185456 -0.825510 3.003965
H 9.103623 -1.798902 1.526572
H 7.572268 0.599612 -1.688264
H 8.992170 0.285749 -0.692713
H 7.911435 1.630124 -0.287493
SCF Energy (B3LYP/6-31G**)= -1361.70949987
Number of imaginary frequencies = 0

1a_c106
MMFF Geometry
C 1.727493 2.617260 0.222286
C 2.415284 0.064670 -0.410381
N 3.044416 2.299345 0.205587
C 0.687565 1.726767 -0.062044
C 1.055681 0.408520 -0.391691
C 3.374953 1.026277 -0.100118
C 0.056271 -0.531712 -0.703907
C -1.300709 -0.184124 -0.673273
C -1.660008 1.121494 -0.327846
C -0.676665 2.079027 -0.041212
C -3.099608 1.463472 -0.277944
C -4.111610 0.306294 -0.112817
C -3.624076 -0.939023 -0.845375
C -2.316143 -1.163000 -1.069711
O -1.002783 3.377562 0.268662
O -3.415070 2.654301 -0.313906
O -5.365805 0.730335 -0.658814
C -4.304106 0.098593 1.393914
C -5.067499 -1.162034 1.731751
C -6.561882 -1.155061 1.542709

O -4.477927 -2.166156 2.137911
O -4.649219 -1.785487 -1.225881
C -4.316368 -3.092535 -1.680821
C   4.825779 0.723067 -0.109241
C   5.681459 1.795721 -0.728075
C   5.292457 -0.425862 0.425151
C   6.738262 -0.860006 0.536853
C   7.100390 -1.917949 -0.523454
C   6.338986 -3.234481 -0.426170
C   7.033817 -1.341245 1.960922
H   1.520435 3.653436 0.481659
H   2.723990 -0.941184 -0.678204
H   0.346440 -1.546688 -0.974027
H -1.959877 -2.056013 -1.571664
H -1.965047 3.509133 0.105237
H -5.280461 0.695549 -1.627664
H -4.841313 0.943718 1.842807
H -3.338383 0.039525 1.913006
H -6.997187 -2.014258 2.061048
H -6.985883 -0.245025 1.974478
H -6.808725 -1.219311 0.481525
H -3.780199 -3.047712 -2.633926
H -3.735425 -3.634536 -0.927514
H -5.249930 -3.638647 -1.845066
H   5.827324 2.624402 -0.027252
H   6.665720 1.427954 -1.030257
H   5.214439 2.189515 -1.638458
H   4.582364 -1.124643 0.867339
H   7.397934 -0.001215 0.368524
H   6.935632 -1.494979 -1.522872
H   8.174081 -2.134161 -0.455523
H   5.260404 -3.086482 -0.533136
H   6.660930 -3.908815 -1.226699
H   6.528993 -3.739313 0.525289
H   6.883277 -0.529233 2.681569
H   8.073232 -1.675006 2.049213
H   6.384427 -2.170638 2.260669
SCF Energy (B3LYP/6-31G**)= -1361.70139379
Number of imaginary frequencies = 0

1a_c107
MMFF Geometry
C -1.380318 -2.221285 -0.467665
C -2.253459 0.211780 0.372588
N -2.698172 -2.081965 -0.184263
C -0.428349 -1.202620 -0.360771
C -0.891599 0.052711 0.076938
C -3.122696 -0.867339 0.224913
C   0.016682 1.120384 0.202916
C   1.377299 0.953568 -0.088247
C   1.830542 -0.299955 -0.508802
C   0.937770 -1.369094 -0.663580
C   3.265132 -0.449598 -0.818866
C   4.258848 0.564992 -0.220902
C   3.624613 1.942697 -0.069964
C   2.288652 2.099840 -0.001088
O   1.351781 -2.599598 -1.112662
O   3.641529 -1.377184 -1.541082
O   5.358197 0.662195 -1.144990
C   4.813792 0.114478 1.132522
C   5.094092 -1.368145 1.178903
C   6.431076 -1.848189 0.681682
O   4.231335 -2.153517 1.574159
O   4.550109 2.965482 0.035895
C   4.083343 4.307170 0.096014
C -4.569806 -0.760795 0.529071
C -5.134499 -1.941679 1.272213
C -5.278771 0.314874 0.124464
C -6.762087 0.562835 0.306865
C -7.412651 0.795806 -1.070614
C -8.928574 0.926153 -1.010783
C -6.957254 1.751346 1.250845
H -1.095736 -3.218766 -0.796182
H -2.630897 1.165549 0.728039
H -0.349360 2.092978 0.530022
H   1.829676 3.073163 0.132839
H   2.302376 -2.547594 -1.357051
H   5.269398 -0.082089 -1.773422
H   4.101603 0.320703 1.942690
H   5.740180 0.645031 1.384745
H   6.557092 -2.904205 0.936780
H   6.488532 -1.735345 -0.402654
H   7.230548 -1.280052 1.163627
H   3.499646 4.559887 -0.795054
H   3.501762 4.477114 1.007653
H   4.956001 4.966258 0.125973
H -4.445876 -2.271892 2.058885
H -6.077479 -1.714530 1.775649
H -5.306639 -2.779977 0.588939
H -4.767986 1.100762 -0.432663
H -7.247265 -0.307762 0.758756
H -6.997559 1.695821 -1.542016
H -7.169076 -0.045540 -1.732523
H -9.233520 1.828394 -0.472908
H -9.339096 0.991344 -2.023655
H -9.378903 0.058422 -0.518672
H -6.427130 1.591397 2.196807
H -8.014694 1.894590 1.494031
H -6.582674 2.681464 0.808730
SCF Energy (B3LYP/6-31G**)= -1361.70446091
Number of imaginary frequencies = 0

1a_c108
MMFF Geometry
C -1.629150 -1.345170 -0.980708
C -2.048206 1.189172 -0.098514
N -2.908140 -0.926663 -0.821148
C -0.498480 -0.566124 -0.725096
C -0.726857 0.744599 -0.268182
C -3.114116 0.335795 -0.380979
C   0.371255 1.583115 0.001163
C   1.687612 1.131110 -0.163629
C   1.905224 -0.178135 -0.602770
C   0.824342 -1.019089 -0.901489
C   3.301357 -0.634328 -0.770980
C   4.403940 0.113614 0.015141
C   4.076272 1.601183 0.128941
C   2.808732 2.050028 0.059947
O   1.009493 -2.295248 -1.375424
O   3.520112 -1.599887 -1.504514
O   5.647091 -0.034818 -0.683665
C   4.597041 -0.477272 1.413553
C   4.547506 -1.987450 1.410528
C   5.772261 -2.736572 0.957939
O   3.515453 -2.574935 1.739977
O   5.191210 2.394091 0.328166
C   5.015228 3.797692 0.481415
C -4.535516 0.763517 -0.231195
C -4.820968 2.235595 -0.357419
C -5.489524 -0.170829 -0.022372
C -6.975441 0.036697 0.178633
C -7.414345 -0.655716 1.483938
C -8.869504 -0.394979 1.848913
C -7.725978 -0.496913 -1.043277
H -1.533610 -2.369520 -1.334766
H -2.233932 2.194106 0.265721
H   0.189991 2.602236 0.340831
H   2.560164 3.100923 0.160269
H   1.970744 -2.441874 -1.526567
H   5.591734 0.517220 -1.483018
H   3.810124 -0.138228 2.100344
H   5.554018 -0.171163 1.853915
H   5.682907 -3.787503 1.247330
H   5.870310 -2.673033 -0.127485
H   6.660222 -2.322626 1.442166
H   4.576374 4.237056 -0.420086
H   4.406951 4.021094 1.363717
H   6.001950 4.246296 0.629422
H -4.239572 2.678816 -1.174149
H -5.866813 2.447813 -0.593672
H -4.573212 2.757062 0.572936
H -5.183065 -1.218405 0.023909

H -7.206064 1.101866 0.279013
H -7.255021 -1.739360 1.413979
H -6.786634 -0.299286 2.311145
H -9.552874 -0.847444 1.124610
H -9.093234 -0.828646 2.828999
H -9.076216 0.678646 1.899128
H -7.348907 -0.039522 -1.965259
H -8.794430 -0.267500 -0.983298
H -7.617887 -1.583260 -1.138418
SCF Energy (B3LYP/6-31G**)= -1361.70078621
Number of imaginary frequencies = 0

1a_c109
MMFF Geometry
C -1.760149 -1.332452 -0.702166
C -2.176382 1.150563 0.316075
N -3.036255 -0.946364 -0.459846
C -0.630934 -0.544457 -0.468045
C -0.858392 0.740327 0.057644
C -3.240735 0.290298 0.049473
C 0.236928 1.588343 0.305831
C 1.550080 1.171194 0.050872
C 1.769151 -0.113385 -0.456363
C 0.689049 -0.963236 -0.732110
C 3.160259 -0.529552 -0.732714
C 4.320623 0.237451 -0.065183
C 3.937584 1.683561 0.195765
C 2.663019 2.091652 0.283377
O 0.869882 -2.215782 -1.267219
O 3.374647 -1.476464 -1.496476
O 5.410015 0.196180 -1.003684
C 4.783210 -0.408312 1.243444
C 4.751937 -1.916849 1.195253
C 5.978246 -2.629009 0.691276
O 3.736418 -2.531453 1.523316
O 4.942844 2.568645 0.560821
C 5.665327 3.061921 -0.567233
C -4.657732 0.671298 0.319983
C -4.892493 1.697525 1.395370
C -5.648820 0.070189 -0.375216
C -7.139408 0.307893 -0.279425
C -7.878440 -0.937615 0.243795
C -7.508998 -1.298002 1.677323
C -7.676022 0.692494 -1.662458
H -1.665885 -2.337848 -1.107009
H -2.361037 2.144293 0.710519
H 0.057940 2.587403 0.702104
H 2.438472 3.118565 0.557997
H 1.824403 -2.345293 -1.461959
H 5.236063 -0.557892 -1.603921
H 4.136922 -0.115021 2.081655
H 5.800957 -0.094936 1.506716
H 5.860152 -3.706558 0.836288
H 6.115960 -2.427639 -0.372823
H 6.854270 -2.298573 1.254823
H 6.503782 2.400235 -0.800532
H 5.024368 3.196287 -1.445853
H 6.078828 4.038238 -0.296814
H -4.688930 2.704790 1.017351
H -5.914516 1.685624 1.781856
H -4.247930 1.510348 2.261974
H -5.373542 -0.682760 -1.117175
H -7.363814 1.145870 0.387706
H -8.960124 -0.757793 0.204069
H -7.679798 -1.800523 -0.404478
H -7.690055 -0.455499 2.352299
H -8.116147 -2.142106 2.019913
H -6.457813 -1.589637 1.761517
H -7.171968 1.587726 -2.043743
H -8.748467 0.910594 -1.614235
H -7.530199 -0.113411 -2.390805
SCF Energy (B3LYP/6-31G**)= -1361.70075973
Number of imaginary frequencies = 0

1a_c110
MMFF Geometry
C 1.446511 -2.204917 0.486736
C 2.293411 0.192069 -0.474264
N 2.757369 -2.074740 0.168172
C 0.489496 -1.193503 0.359124
C 0.939058 0.043168 -0.141071
C 3.169461 -0.878050 -0.301123
C 0.025352 1.103187 -0.289704
C -1.327802 0.945514 0.038837
C -1.769444 -0.290458 0.520557
C -0.868930 -1.349838 0.699677
C -3.200702 -0.434721 0.861637
C -4.201244 0.543391 0.201849
C -3.575272 1.925783 0.027870
C -2.241721 2.088250 -0.062181
O -1.268546 -2.560943 1.210662
O -3.533752 -1.338218 1.630416
O -5.354235 0.659726 1.046158
C -4.683669 0.024597 -1.154670
C -4.961718 -1.460471 -1.135453
C -6.256307 -1.932063 -0.529080
O -4.128032 -2.253479 -1.576972
O -4.507627 2.943856 -0.047782
C -4.051369 4.277550 -0.241918
C 4.609429 -0.778256 -0.644703
C 5.160531 -1.991529 -1.345582
C 5.319020 0.315513 -0.292194
C 6.785630 0.589747 -0.545967
C 7.637419 0.371339 0.720023
C 7.326001 1.293586 1.892509
C 6.960225 1.996179 -1.127472
H 1.172068 -3.188469 0.862625
H 2.660009 1.130639 -0.878289
H 0.381629 2.062283 -0.663941
H -1.785161 3.061309 -0.206996
H -2.213184 -2.496608 1.478816
H -5.085907 1.179093 1.824004
H -3.930201 0.193324 -1.935494
H -5.597557 0.535603 -1.481940
H -6.431344 -2.974118 -0.811338
H -6.207873 -1.861067 0.559227
H -7.085218 -1.330446 -0.910214
H -3.422539 4.600793 0.593800
H -3.519814 4.373878 -1.194041
H -4.927884 4.931195 -0.277165
H 4.547281 -2.239311 -2.219558
H 6.181400 -1.857195 -1.708391
H 5.171335 -2.856444 -0.673291
H 4.814111 1.103130 0.267580
H 7.170689 -0.098045 -1.305579
H 7.521818 -0.667547 1.055069
H 8.696256 0.492639 0.458402
H 6.291933 1.182047 2.231230
H 7.978265 1.050969 2.738055
H 7.497670 2.342715 1.635448
H 6.445177 2.081789 -2.091207
H 8.019600 2.216971 -1.296456
H 6.553168 2.770108 -0.468039
SCF Energy (B3LYP/6-31G**)= -1361.69938702
Number of imaginary frequencies = 0

1a_c111
MMFF Geometry
C -1.788525 -2.415594 0.047668
C -2.372511 0.211414 0.425827
N -3.068990 -2.085119 0.342157
C -0.736314 -1.502914 -0.076085
C -1.050244 -0.145645 0.123296
C -3.350946 -0.776661 0.519450
C -0.035323 0.822438 0.010890
C 1.285749 0.458913 -0.284700
C 1.592535 -0.890892 -0.470032
C 0.590066 -1.867906 -0.382578
C 2.993601 -1.258523 -0.763055
C 4.112166 -0.244681 -0.456937
C 3.613145 1.189112 -0.552870
C 2.306595 1.495923 -0.450398

O 0.859244 -3.200416 -0.584680
O 3.243870 -2.387851 -1.196578
O 5.122814 -0.473471 -1.453098
C 4.680442 -0.613851 0.917976
C 5.641933 0.408740 1.481913
C 7.022323 0.486959 0.883084
O 5.305483 1.140341 2.415662
O 4.628162 2.112721 -0.726148
C 4.307834 3.497969 -0.718730
C -4.762809 -0.456606 0.844365
C -5.403523 -1.412200 1.815523
C -5.376595 0.590766 0.252432
C -6.801186 1.062343 0.449614
C -7.711586 0.642431 -0.721204
C -7.352908 1.230929 -2.080343
C -6.821574 2.575616 0.686857
H -1.622505 -3.481860 -0.091005
H -2.634575 1.250506 0.600542
H -0.285033 1.873313 0.155145
H 1.951839 2.519634 -0.498800
H 1.780052 -3.291415 -0.918167
H 5.084595 -1.423808 -1.680821
H 5.208550 -1.575236 0.881277
H 3.872138 -0.735103 1.651545
H 7.654795 1.121669 1.510463
H 7.465208 -0.511565 0.848020
H 6.980650 0.915405 -0.119460
H 3.654622 3.752221 -1.559448
H 3.854526 3.789881 0.234045
H 5.239725 4.059181 -0.834524
H -4.788568 -1.509672 2.717537
H -6.394831 -1.096103 2.146026
H -5.517963 -2.404320 1.365219
H -4.818057 1.172772 -0.481106
H -7.224484 0.612102 1.353184
H -7.706717 -0.452080 -0.804418
H -8.744749 0.926909 -0.484681
H -6.346018 0.940474 -2.393840
H -8.053097 0.865246 -2.838809
H -7.415183 2.322860 -2.074775
H -6.268857 2.830634 1.598371
H -7.848884 2.934817 0.810099
H -6.364902 3.130290 -0.139761
SCF Energy (B3LYP/6-31G**)= -1361.70796139
Number of imaginary frequencies = 0

1a_c112
MMFF Geometry
C -1.732799 -1.288534 -1.057620
C -2.207880 1.184385 -0.040550
N -3.020934 -0.896475 -0.905192
C -0.619166 -0.511805 -0.729912
C -0.876958 0.766995 -0.203624
C -3.254847 0.335789 -0.397938
C 0.202206 1.601273 0.142560
C 1.527994 1.177195 -0.018187
C 1.776005 -0.100757 -0.528800
C 0.714107 -0.937255 -0.899648
C 3.181125 -0.524056 -0.705236
C 4.292595 0.222077 0.061731
C 3.901046 1.666868 0.317602
C 2.626465 2.084178 0.314463
O 0.926367 -2.183102 -1.438825
O 3.446422 -1.460046 -1.466472
O 5.449980 0.187103 -0.791812
C 4.649367 -0.449197 1.390492
C 4.612539 -1.956439 1.313733
C 5.869651 -2.669181 0.893407
O 3.570959 -2.568888 1.551759
O 4.881362 2.537468 0.773971
C 5.696242 3.038855 -0.285427
C -4.685220 0.735945 -0.257829
C -4.987771 2.209453 -0.297302
C -5.630710 -0.221188 -0.129371
C -7.122016 -0.045390 0.050253
C -7.633043 -0.803637 1.291121
C -7.061797 -0.267169 2.598049
C -7.842985 -0.544695 -1.204188
H -1.615031 -2.289084 -1.468274
H -2.415267 2.164144 0.376909
H 0.000586 2.595157 0.540969
H 2.388913 3.108195 0.588963
H 1.891973 -2.317286 -1.562939
H 5.316985 -0.555709 -1.416096
H 3.942513 -0.165235 2.181708
H 5.645858 -0.148004 1.736345
H 5.734120 -3.748097 1.010288
H 6.090200 -2.451068 -0.153305
H 6.701652 -2.354767 1.528316
H 6.546900 2.373996 -0.457564
H 5.129902 3.187960 -1.211648
H 6.092245 4.009013 0.029661
H -4.395196 2.710471 -1.071459
H -6.031052 2.423897 -0.542296
H -4.766917 2.673726 0.669443
H -5.311253 -1.265718 -0.137425
H -7.381420 1.009434 0.184007
H -8.726459 -0.723941 1.336628
H -7.399517 -1.872829 1.210121
H -7.274948 0.800713 2.709705
H -7.510977 -0.792342 3.447110
H -5.978542 -0.411297 2.653451
H -7.509937 0.003741 -2.092513
H -8.925015 -0.401027 -1.112086
H -7.659390 -1.611246 -1.377313
SCF Energy (B3LYP/6-31G**)= -1361.70079075
Number of imaginary frequencies = 0

1a_c113
MMFF Geometry
C -1.707978 -1.829230 -0.262189
C -2.039211 0.864728 -0.206566
N -2.969731 -1.343628 -0.167942
C -0.553659 -1.044999 -0.334863
C -0.737301 0.347555 -0.304821
C -3.130111 -0.000778 -0.140419
C 0.386338 1.191056 -0.374920
C 1.687332 0.672199 -0.471531
C 1.864884 -0.714180 -0.495344
C 0.751605 -1.569708 -0.434479
C 3.236373 -1.261994 -0.601845
C 4.441042 -0.307097 -0.561038
C 4.092143 1.168332 -0.590359
C 2.819061 1.600276 -0.551285
O 0.883258 -2.938669 -0.473643
O 3.389342 -2.478832 -0.761787
O 5.157710 -0.597960 -1.784929
C 5.371825 -0.682073 0.599355
C 4.817077 -0.287483 1.948569
C 5.818537 0.072252 3.013441
O 3.610527 -0.309654 2.189612
O 5.207557 1.988659 -0.633084
C 5.015298 3.388249 -0.797813
C -4.532474 0.499891 -0.051554
C -4.813054 1.823182 -0.706940
C -5.463455 -0.261342 0.565789
C -6.932683 -0.002545 0.831495
C -7.781613 -0.288866 -0.420255
C -9.280880 -0.246368 -0.156717
C -7.214858 1.362041 1.467666
H -1.646783 -2.915240 -0.278567
H -2.190199 1.938501 -0.167880
H 0.239171 2.270230 -0.351229
H 2.574231 2.656240 -0.558851
H 1.836342 -3.157131 -0.579346
H 5.214350 -1.570925 -1.846144
H 6.346111 -0.195571 0.462542
H 5.573335 -1.759978 0.621838
H 5.295953 0.336933 3.936815
H 6.470394 -0.783189 3.206402
H 6.408675 0.931765 2.685904
H 4.471170 3.604151 -1.722974

H 4.502367 3.815834 0.069515
H 6.001313 3.856778 -0.869350
H -5.864893 1.946511 -0.971828
H -4.527500 2.649170 -0.047481
H -4.261732 1.921318 -1.649294
H -5.138383 -1.224428 0.967912
H -7.221629 -0.743411 1.591726
H -7.547012 0.424550 -1.218348
H -7.530156 -1.284206 -0.809017
H -9.611739 0.761019 0.112688
H -9.828374 -0.544827 -1.056601
H -9.557951 -0.931504 0.650602
H -6.473745 1.601994 2.238123
H -8.196104 1.361547 1.955004
H -7.221659 2.172345 0.733812
SCF Energy (B3LYP/6-31G**)= -1361.70356500
Number of imaginary frequencies = 0

1a_c114
MMFF Geometry
C 1.505195 2.486848 -0.089719
C 2.302246 -0.092744 -0.389176
N 2.836224 2.232223 -0.112474
C 0.502821 1.518943 -0.212759
C 0.928014 0.188490 -0.370744
C 3.221124 0.945426 -0.250423
C -0.032071 -0.829213 -0.508567
C -1.406292 -0.544861 -0.490268
C -1.829072 0.778395 -0.324745
C -0.878309 1.805984 -0.190652
C -3.283316 1.083972 -0.313118
C -4.303646 -0.067981 -0.486700
C -3.678802 -1.438702 -0.650169
C -2.356708 -1.645597 -0.629565
O -1.246554 3.123572 -0.043083
O -3.631125 2.265961 -0.225102
O -5.027698 0.229814 -1.699835
C -5.328070 -0.007957 0.654310
C -4.741414 -0.346511 2.007551
C -5.718071 -0.760333 3.076582
O -3.539406 -0.245968 2.250794
O -4.514251 -2.549547 -0.659946
C -5.298836 -2.652208 -1.847014
C 4.684786 0.710462 -0.269765
C 5.465185 1.723523 -1.063455
C 5.223014 -0.326192 0.407754
C 6.690450 -0.680695 0.532606
C 6.960596 -1.994615 -0.222505
C 8.433350 -2.378709 -0.263076
C 7.060633 -0.774373 2.016104
H 1.252947 3.537837 0.034767
H 2.652058 -1.111517 -0.524039
H 0.301419 -1.859798 -0.627245
H -1.968378 -2.658988 -0.683068
H -2.229750 3.173043 -0.028365
H -4.370043 0.358271 -2.405356
H -6.155229 -0.699356 0.450346
H -5.777400 0.990388 0.725838
H -5.179924 -0.980454 4.002726
H -6.424699 0.052503 3.260796
H -6.250745 -1.659822 2.758156
H -6.182690 -2.011779 -1.779453
H -4.713831 -2.416060 -2.742631
H -5.643773 -3.687162 -1.930395
H 5.592242 2.647418 -0.489468
H 6.455868 1.363010 -1.351925
H 4.949200 1.965733 -2.000025
H 4.561868 -0.985935 0.970543
H 7.315577 0.105317 0.097297
H 6.390669 -2.817601 0.227419
H 6.606153 -1.897045 -1.256991
H 8.812942 -2.621901 0.733492
H 8.572652 -3.262678 -0.893850
H 9.039878 -1.567852 -0.678505
H 6.784258 0.143668 2.547395
H 8.138711 -0.911042 2.146788
H 6.551873 -1.612522 2.505542
SCF Energy (B3LYP/6-31G**)= -1361.70042273
Number of imaginary frequencies = 0

1a_c115
MMFF Geometry
C -1.884424 -1.391561 -0.591967
C -2.323251 1.223717 -0.008298
N -3.161899 -0.939846 -0.602762
C -0.764410 -0.609274 -0.300615
C -1.003863 0.742501 0.001904
C -3.377766 0.364169 -0.313334
C 0.081931 1.580530 0.313770
C 1.397562 1.095925 0.314771
C 1.633315 -0.244975 0.003475
C 0.558232 -1.099081 -0.289972
C 3.027867 -0.747705 0.024040
C 4.198395 0.266965 -0.047357
C 3.771210 1.626060 0.491588
C 2.494580 1.986030 0.678654
O 0.746874 -2.431536 -0.572792
O 3.196143 -1.970080 0.055565
O 4.546413 0.474811 -1.418524
C 5.387490 -0.286371 0.765961
C 5.986247 -1.533408 0.145618
C 6.513567 -2.582646 1.086045
O 6.085984 -1.652826 -1.077564
O 4.767541 2.517261 0.875087
C 5.390088 3.124867 -0.259474
C -4.796120 0.827725 -0.352901
C -5.023541 2.284864 -0.653019
C -5.788086 -0.066431 -0.143102
C -7.282038 0.169863 -0.124579
C -7.886288 -0.083275 1.272402
C -7.808952 -1.517441 1.781749
C -7.955205 -0.672514 -1.210835
H -1.781095 -2.446832 -0.835376
H -2.516431 2.263113 0.235666
H -0.104535 2.626126 0.556967
H 2.258255 2.964581 1.086350
H 1.696422 -2.649907 -0.436106
H 5.000783 -0.341446 -1.725002
H 5.068517 -0.517529 1.789615
H 6.213448 0.432089 0.823011
H 6.902252 -3.428974 0.513071
H 7.320459 -2.160035 1.689504
H 5.705230 -2.937265 1.730276
H 6.083726 2.429179 -0.740530
H 4.649271 3.491771 -0.978448
H 5.969661 3.981174 0.098029
H -4.360187 2.626475 -1.456058
H -6.037117 2.499850 -0.998740
H -4.838528 2.894315 0.237715
H -5.505349 -1.104238 0.044133
H -7.503621 1.215485 -0.359180
H -7.391849 0.572006 2.001156
H -8.941755 0.217347 1.257324
H -6.774424 -1.859691 1.874469
H -8.267119 -1.582619 2.774327
H -8.347634 -2.207076 1.125687
H -7.604097 -0.372043 -2.204717
H -9.041958 -0.538417 -1.188359
H -7.741907 -1.740750 -1.096762
SCF Energy (B3LYP/6-31G**)= -1361.70686749
Number of imaginary frequencies = 0

1a_c116
MMFF Geometry
C -2.027713 -1.591202 -0.061639
C -2.140896 1.121762 -0.113969
N -3.241038 -0.999509 0.051651
C -0.818133 -0.907360 -0.203504
C -0.888639 0.496927 -0.231426
C -3.295039 0.352314 0.026823
C 0.294427 1.243900 -0.383624
C 1.542696 0.615238 -0.491756

C 1.605756 -0.779113 -0.448371
C 0.435398 -1.541071 -0.321281
C 2.931336 -1.425250 -0.544593
C 4.193767 -0.583934 -0.277689
C 3.978276 0.879958 -0.630930
C 2.747616 1.420135 -0.704652
O 0.465144 -2.914951 -0.303393
O 2.997845 -2.635306 -0.783809
O 5.214342 -1.140980 -1.122760
C 4.581590 -0.819082 1.186523
C 5.670485 0.101989 1.690569
C 7.082342 -0.154871 1.231379
O 5.406068 1.021995 2.467750
O 5.157089 1.574173 -0.835358
C 5.100197 2.976344 -1.063870
C -4.647017 0.966016 0.170952
C -4.708038 2.359614 0.736369
C -5.741645 0.250627 -0.171023
C -7.188614 0.688639 -0.123597
C -7.917883 0.126195 1.111822
C -8.020520 -1.392750 1.180087
C -7.893151 0.315999 -1.432107
H -2.056035 -2.678347 -0.033125
H -2.205212 2.204423 -0.146931
H 0.235507 2.331462 -0.418585
H 2.593739 2.468229 -0.936961
H 1.374589 -3.216011 -0.526126
H 5.015949 -2.094289 -1.213956
H 4.925060 -1.849633 1.342994
H 3.713090 -0.681441 1.844505
H 7.772341 0.457635 1.818930
H 7.335376 -1.206087 1.390567
H 7.194772 0.103653 0.177374
H 4.567396 3.199165 -1.993716
H 4.642172 3.493161 -0.214405
H 6.126028 3.342014 -1.167532
H -4.452459 3.098149 -0.030506
H -5.694847 2.614304 1.132351
H -4.014678 2.471608 1.577865
H -5.596599 -0.766219 -0.541411
H -7.242460 1.781333 -0.057539
H -7.412371 0.480820 2.019307
H -8.932633 0.542637 1.143953
H -7.033541 -1.864219 1.195540
H -8.542288 -1.688156 2.096446
H -8.586212 -1.795731 0.335197
H -7.426223 0.828261 -2.281192
H -8.946730 0.613879 -1.403301
H -7.849966 -0.759620 -1.633696
SCF Energy (B3LYP/6-31G**)= -1361.70862597
Number of imaginary frequencies = 0

1a_c117
MMFF Geometry
C 1.723460 1.791747 -0.288483
C 2.069916 -0.898097 -0.163713
N 2.989103 1.315058 -0.200838
C 0.572255 1.000386 -0.321904
C 0.764048 -0.390239 -0.256525
C 3.157073 -0.025783 -0.138375
C -0.355328 -1.241293 -0.286396
C -1.658851 -0.730621 -0.378905
C -1.846561 0.654512 -0.436608
C -0.737215 1.516470 -0.415599
C -3.224599 1.193353 -0.540484
C -4.428845 0.235240 -0.468045
C -4.052417 -1.229875 -0.479979
C -2.786339 -1.660628 -0.411024
O -0.876386 2.883147 -0.490164
O -3.386941 2.404284 -0.733742
O -5.163921 0.520389 -1.680146
C -5.336256 0.608529 0.709274
C -4.737840 0.220649 2.041392
C -5.667744 -0.383947 3.058435
O -3.552876 0.429367 2.300239
O -5.074726 -2.166025 -0.379087
C -5.794083 -2.314182 -1.603521
C 4.563374 -0.516862 -0.057159
C 4.840337 -1.858199 -0.676474
C 5.500198 0.267017 0.521850
C 6.974743 0.023138 0.771712
C 7.802254 0.274512 -0.501701
C 9.305709 0.246914 -0.261027
C 7.273769 -1.319862 1.445039
H 1.656393 2.876597 -0.333286
H 2.226828 -1.969711 -0.097415
H -0.204306 -2.319047 -0.234593
H -2.579843 -2.725379 -0.351399
H -1.832615 3.095249 -0.577041
H -5.195136 1.495091 -1.756220
H -6.310700 0.116305 0.597288
H -5.546368 1.684647 0.735064
H -5.112872 -0.620670 3.970464
H -6.459781 0.329222 3.299255
H -6.097130 -1.306287 2.659423
H -6.570740 -1.548436 -1.683593
H -5.131469 -2.289877 -2.475808
H -6.290295 -3.289121 -1.581977
H 4.275458 -1.987105 -1.607018
H 5.888641 -1.985088 -0.953382
H 4.568606 -2.665066 0.011866
H 5.176624 1.240205 0.900119
H 7.272070 0.788414 1.504022
H 7.558598 -0.464225 -1.273609
H 7.539691 1.256243 -0.916830
H 9.645825 -0.750181 0.033926
H 9.837356 0.519924 -1.178325
H 9.592135 0.957876 0.520278
H 6.546213 -1.539187 2.234335
H 8.262635 -1.299930 1.916302
H 7.272894 -2.152430 0.736514
SCF Energy (B3LYP/6-31G**)= -1361.69789882
Number of imaginary frequencies = 0

1a_c118
MMFF Geometry
C -1.519591 -2.448037 0.020063
C -2.515606 0.068968 -0.225481
N -2.861260 -2.305171 -0.105735
C -0.601624 -1.393272 0.033586
C -1.128673 -0.094379 -0.095123
C -3.346089 -1.050053 -0.219770
C -0.256733 1.010082 -0.084403
C 1.128858 0.838903 0.038194
C 1.646025 -0.453169 0.150396
C 0.790787 -1.564353 0.167660
C 3.110201 -0.608122 0.279811
C 4.010010 0.530144 -0.260023
C 3.344779 1.886606 -0.007662
C 2.008437 2.007898 0.111567
O 1.271179 -2.843652 0.315906
O 3.538921 -1.654089 0.772324
O 4.124138 0.416221 -1.681969
C 5.396278 0.450457 0.412077
C 6.139962 -0.820388 0.050404
C 7.057349 -1.397642 1.093811
O 6.039538 -1.323710 -1.070888
O 4.236804 2.945217 0.035180
C 3.721245 4.270808 0.067306
C -4.817450 -0.939841 -0.361306
C -5.432984 -1.969728 -1.270277
C -5.504466 0.011274 0.307105
C -7.002858 0.225566 0.306538
C -7.409134 1.416382 -0.583142
C -6.869081 2.774528 -0.151581
C -7.512785 0.374572 1.743439
H -1.185810 -3.479441 0.112338
H -2.942879 1.059804 -0.344743
H -0.668814 2.014545 -0.173108
H 1.525357 2.966640 0.260518
H 2.232161 -2.795280 0.520650
H 4.708527 -0.352590 -1.861586

H 5.291306 0.501609 1.502464
H 6.051005 1.268802 0.089332
H 7.518533 -2.313047 0.713022
H 7.841951 -0.675015 1.330553
H 6.484146 -1.641390 1.991705
H 3.093755 4.469691 -0.807514
H 3.171251 4.452742 0.996041
H 4.569006 4.961697 0.038211
H -5.523473 -2.932433 -0.756318
H -6.425084 -1.679026 -1.626046
H -4.820616 -2.112261 -2.168423
H -4.956794 0.694327 0.956223
H -7.504582 -0.660631 -0.098381
H -7.080360 1.223507 -1.612507
H -8.504368 1.475278 -0.617341
H -5.775302 2.790254 -0.142478
H -7.204777 3.546440 -0.852067
H -7.230085 3.053186 0.842530
H -7.316658 -0.537419 2.318909
H -8.594135 0.548149 1.754773
H -7.030238 1.204928 2.269848
SCF Energy (B3LYP/6-31G**)= -1361.71120716
Number of imaginary frequencies = 0

1a_c119
MMFF Geometry
C -1.543034 -2.300531 -0.536432
C -2.261934 0.145030 0.402701
N -2.850581 -2.090432 -0.248123
C -0.528166 -1.349048 -0.390085
C -0.912667 -0.087935 0.097425
C -3.197805 -0.869384 0.211779
C 0.062513 0.909976 0.269698
C 1.411336 0.674716 -0.038584
C 1.794208 -0.581988 -0.519472
C 0.827870 -1.588171 -0.697281
C 3.220196 -0.831749 -0.854909
C 4.255487 0.304656 -0.667942
C 3.675260 1.598954 -0.134862
C 2.380162 1.748570 0.167627
O 1.155082 -2.834417 -1.180206
O 3.524122 -1.929222 -1.333442
O 4.785096 0.572440 -1.984149
C 5.428562 -0.217635 0.172338
C 5.056602 -0.505249 1.610718
C 6.192825 -0.566367 2.597216
O 3.898660 -0.728704 1.961926
O 4.549311 2.623869 0.208122
C 5.160501 3.243136 -0.922454
C -4.635654 -0.684036 0.522268
C -5.275236 -1.857236 1.215118
C -5.274027 0.451212 0.165749
C -6.738471 0.785634 0.361606
C -7.371352 1.120275 -1.003108
C -8.875833 1.344932 -0.934835
C -6.857875 1.941531 1.357291
H -1.321168 -3.300079 -0.904379
H -2.576958 1.105030 0.799743
H -0.238384 1.884247 0.653965
H 2.033639 2.678042 0.611061
H 2.126820 -2.863571 -1.335804
H 4.028411 0.744104 -2.571287
H 6.248157 0.511757 0.163547
H 5.838578 -1.140235 -0.257078
H 5.802597 -0.776923 3.596809
H 6.883678 -1.362866 2.310389
H 6.711818 0.395094 2.618631
H 6.015631 2.654794 -1.266719
H 4.443925 3.402127 -1.735698
H 5.535670 4.220680 -0.605090
H -4.610200 -2.263606 1.986276
H -6.202749 -1.592422 1.728777
H -5.499105 -2.653113 0.496993
H -4.713110 1.226465 -0.357020
H -7.279011 -0.071075 0.775628
H -6.898839 2.011646 -1.435238
H -7.181657 0.294964 -1.701816
H -9.122739 2.240356 -0.357259
H -9.280305 1.480841 -1.943110
H -9.381458 0.487101 -0.480526
H -6.340070 1.706416 2.294339
H -7.904180 2.141517 1.608466
H -6.423900 2.864238 0.955826
SCF Energy (B3LYP/6-31G**)= -1361.70041138
Number of imaginary frequencies = 0

1a_c120
MMFF Geometry
C 1.909094 1.727305 0.067498
C 2.083503 -0.968898 -0.211415
N 3.134635 1.154670 0.141416
C 0.716430 1.031487 -0.142300
C 0.818444 -0.364025 -0.287831
C 3.218821 -0.188770 0.004251
C -0.345972 -1.121503 -0.515078
C -1.606433 -0.512794 -0.580299
C -1.701296 0.871816 -0.417462
C -0.549623 1.645836 -0.217720
C -3.043763 1.494113 -0.465539
C -4.268594 0.590883 -0.192744
C -4.031675 -0.811495 -0.742712
C -2.792494 -1.316199 -0.887365
O -0.612499 3.012359 -0.086969
O -3.114172 2.708327 -0.664440
O -5.406500 1.177674 -0.834393
C -4.515657 0.625477 1.319948
C -5.518503 -0.405447 1.786379
C -6.979394 -0.130347 1.543319
O -5.145202 -1.443557 2.337564
O -5.200988 -1.482132 -1.051162
C -5.131227 -2.877306 -1.324494
C 4.582950 -0.783226 0.108757
C 4.670020 -2.212995 0.570571
C 5.662125 -0.021717 -0.177976
C 7.121019 -0.425106 -0.158849
C 7.819969 0.248584 1.035555
C 9.266672 -0.189776 1.217360
C 7.764031 -0.049481 -1.497792
H 1.912372 2.808572 0.187732
H 2.173087 -2.043239 -0.334493
H -0.262344 -2.200378 -0.642965
H -2.616387 -2.320050 -1.258295
H -1.526644 3.308581 -0.302665
H -5.318036 1.001125 -1.787371
H -4.878880 1.611136 1.637552
H -3.587012 0.447303 1.878016
H -7.582763 -0.812516 2.149105
H -7.218384 0.893512 1.841587
H -7.222390 -0.282687 0.490269
H -4.585906 -3.064491 -2.254808
H -4.679294 -3.419402 -0.487465
H -6.153230 -3.245620 -1.453361
H 4.442800 -2.898180 -0.252676
H 5.656426 -2.473680 0.963016
H 3.967845 -2.403491 1.390418
H 5.497130 1.017103 -0.472467
H 7.218427 -1.510541 -0.055065
H 7.789791 1.340514 0.929174
H 7.273880 0.008506 1.957201
H 9.894906 0.142476 0.385911
H 9.677110 0.244353 2.134832
H 9.340529 -1.278945 1.296144
H 7.200106 -0.474640 -2.336065
H 8.786071 -0.434966 -1.567550
H 7.802543 1.037090 -1.634807
SCF Energy (B3LYP/6-31G**)= -1361.70334741
Number of imaginary frequencies = 0

1a_c121
MMFF Geometry
C -1.402108 -2.384813 -0.586499
C -2.371838 0.147309 -0.397409

N -2.746530 -2.220877 -0.539776
C -0.468686 -1.344397 -0.545740
C -0.982428 -0.037112 -0.450509
C -3.217346 -0.959783 -0.435625
C -0.095176 1.054957 -0.420584
C 1.292343 0.863840 -0.466707
C 1.793841 -0.438561 -0.543465
C 0.926057 -1.537479 -0.602020
C 3.257493 -0.614930 -0.600704
C 4.158537 0.525704 -0.089279
C 3.519134 1.888881 -0.325947
C 2.189024 2.024688 -0.488985
O 1.393010 -2.823998 -0.720648
O 3.730435 -1.663269 -1.049042
O 5.384004 0.454460 -0.841032
C 4.502291 0.380977 1.395146
C 4.761765 -1.052127 1.792309
C 6.154407 -1.592742 1.609353
O 3.844015 -1.758486 2.212022
O 4.426099 2.933245 -0.302264
C 3.965714 4.246086 -0.595720
C -4.692872 -0.825960 -0.390381
C -5.428572 -1.730276 -1.342571
C -5.280161 0.035811 0.467431
C -6.764287 0.254563 0.669064
C -7.247894 1.550638 -0.009867
C -6.636015 2.840536 0.523262
C -7.102053 0.221484 2.162963
H -1.079526 -3.421266 -0.661244
H -2.791649 1.146896 -0.341635
H -0.498603 2.065298 -0.362411
H 1.722454 2.993164 -0.631572
H 2.370018 -2.801205 -0.825538
H 5.384267 -0.406432 -1.305397
H 3.678835 0.734303 2.030122
H 5.384581 0.974763 1.663997
H 6.233316 -2.568927 2.096140
H 6.373676 -1.707234 0.546041
H 6.877210 -0.917333 2.073662
H 3.523414 4.292370 -1.596121
H 3.255928 4.589124 0.163709
H 4.829077 4.917711 -0.577003
H -5.477442 -2.749739 -0.945771
H -6.449377 -1.392731 -1.540965
H -4.926693 -1.760964 -2.316817
H -4.648524 0.628168 1.129334
H -7.326108 -0.570757 0.217153
H -7.043822 1.489051 -1.086644
H -8.338443 1.617922 0.091871
H -5.548390 2.850459 0.406391
H -7.035611 3.696739 -0.030327
H -6.874551 2.991832 1.579880
H -6.858111 -0.757156 2.592048
H -8.171389 0.396639 2.322388
H -6.546352 0.976067 2.729751
SCF Energy (B3LYP/6-31G**)= -1361.70337589
Number of imaginary frequencies = 0

1a_c122
MMFF Geometry
C -1.745809 -1.342660 -0.530169
C -2.061490 1.239718 0.249753
N -3.001574 -0.898761 -0.280399
C -0.589882 -0.566838 -0.418190
C -0.764920 0.769581 -0.015415
C -3.155924 0.387104 0.110432
C 0.359645 1.607902 0.103211
C 1.651684 1.130578 -0.155722
C 1.817909 -0.203933 -0.537529
C 0.707596 -1.046052 -0.688527
C 3.189120 -0.686741 -0.806467
C 4.365099 0.090191 -0.168815
C 4.060612 1.585893 -0.111229
C 2.795530 2.046277 -0.089173
O 0.838340 -2.348537 -1.105579
O 3.332059 -1.694882 -1.500336
O 5.537416 -0.113558 -0.968784
C 4.681800 -0.422995 1.237715
C 4.619598 -1.930073 1.326541
C 5.790868 -2.720333 0.807259
O 3.617653 -2.483701 1.783060
O 5.195126 2.374473 -0.061347
C 5.044610 3.786728 0.024916
C -4.551208 0.832557 0.393286
C -4.721614 1.970615 1.363267
C -5.585704 0.188353 -0.191252
C -7.061717 0.490124 -0.057672
C -7.759805 -0.480113 0.914829
C -7.749363 -1.946817 0.501239
C -7.722615 0.500810 -1.439878
H -1.691517 -2.385528 -0.835539
H -2.207173 2.272210 0.549344
H 0.218248 2.646755 0.399236
H 2.565170 3.104264 -0.027218
H 1.780593 -2.515862 -1.334740
H 5.412877 0.393083 -1.790120
H 3.964632 -0.034850 1.973166
H 5.677910 -0.105439 1.569905
H 5.719537 -3.752009 1.163270
H 5.788925 -2.719258 -0.284432
H 6.723238 -2.292150 1.183621
H 4.527672 4.179030 -0.856681
H 4.522136 4.068422 0.944717
H 6.044131 4.230365 0.055213
H -4.515265 2.928151 0.873993
H -5.728313 2.019957 1.787073
H -4.046155 1.860138 2.219397
H -5.360892 -0.649168 -0.854878
H -7.197794 1.500385 0.344972
H -7.291742 -0.395333 1.904052
H -8.803120 -0.164541 1.041651
H -6.730132 -2.331358 0.401523
H -8.257226 -2.549841 1.261277
H -8.274423 -2.101123 -0.445725
H -7.286471 1.284403 -2.070071
H -8.795782 0.702398 -1.354642
H -7.596908 -0.451397 -1.966149
SCF Energy (B3LYP/6-31G**)= -1361.69989278
Number of imaginary frequencies = 0

1a_c123
MMFF Geometry
C -1.366134 -2.242704 -0.773184
C -2.326198 0.264231 -0.347624
N -2.710258 -2.076202 -0.726747
C -0.428155 -1.217040 -0.619462
C -0.937168 0.077646 -0.402451
C -3.176412 -0.828609 -0.504548
C -0.045755 1.156213 -0.254421
C 1.340652 0.961863 -0.304141
C 1.839807 -0.330282 -0.502428
C 0.966574 -1.412527 -0.677552
C 3.305901 -0.512329 -0.563131
C 4.216855 0.575682 0.039824
C 3.567139 1.941532 -0.074178
C 2.241137 2.110417 -0.193075
O 1.427388 -2.684924 -0.914876
O 3.776448 -1.523570 -1.092953
O 5.428905 0.574021 -0.734565
C 4.576532 0.302991 1.502153
C 4.827264 -1.160241 1.775540
C 6.223804 -1.683754 1.574021
O 3.902032 -1.900082 2.112342
O 4.392871 3.036775 0.130201
C 4.762433 3.642132 -1.109282
C -4.651981 -0.691396 -0.463308
C -5.378884 -1.493383 -1.509211
C -5.244315 0.081059 0.472554
C -6.729256 0.292385 0.684983
C -7.091965 1.743449 0.321111
C -8.586341 2.028446 0.382713
C -7.083540 -0.050744 2.135271

H -1.047757 -3.268785 -0.945320
H -2.741673 1.256009 -0.198242
H -0.443638 2.158682 -0.100003
H 1.812259 3.108271 -0.187697
H 2.406402 -2.660422 -0.997418
H 5.467751 -0.289931 -1.193168
H 3.764103 0.607443 2.175530
H 5.466810 0.865995 1.808267
H 6.286181 -2.709985 1.946934
H 6.475337 -1.676457 0.511711
H 6.932790 -1.069428 2.134501
H 5.268649 2.931979 -1.770458
H 3.886702 4.066674 -1.611652
H 5.457067 4.458273 -0.890026
H -5.440459 -2.545954 -1.213432
H -6.393882 -1.130789 -1.690726
H -4.861803 -1.434378 -2.474208
H -4.618087 0.600627 1.198379
H -7.309941 -0.381538 0.047233
H -6.568476 2.445227 0.982942
H -6.746786 1.957979 -0.698761
H -8.966291 1.962417 1.406264
H -8.789745 3.041270 0.020087
H -9.146625 1.326435 -0.242754
H -6.742598 -1.059994 2.393389
H -8.166111 -0.024958 2.294596
H -6.621502 0.650765 2.839038
SCF Energy (B3LYP/6-31G**)= -1361.69933808
Number of imaginary frequencies = 0

1a_c124
MMFF Geometry
C -1.403870 -2.152318 -0.630658
C -2.287823 0.192617 0.421856
N -2.725496 -2.038152 -0.353582
C -0.453020 -1.149667 -0.416236
C -0.922239 0.059959 0.130653
C -3.155233 -0.866566 0.162041
C -0.016089 1.109792 0.368570
C 1.347420 0.968839 0.079875
C 1.809014 -0.241114 -0.450266
C 0.917420 -1.289716 -0.714677
C 3.249747 -0.365718 -0.758401
C 4.242023 0.601668 -0.082633
C 3.585712 1.943254 0.182109
C 2.257048 2.095465 0.293608
O 1.336037 -2.474787 -1.269660
O 3.634833 -1.234220 -1.547158
O 5.326818 0.791980 -1.007689
C 4.812460 0.046708 1.224577
C 5.097173 -1.433948 1.151852
C 6.447525 -1.866111 0.647085
O 4.229873 -2.252515 1.459443
O 4.435018 2.996564 0.486043
C 4.619688 3.851972 -0.642455
C -4.606323 -0.786900 0.455410
C -5.181435 -2.031760 1.076029
C -5.309354 0.323425 0.145035
C -6.794821 0.555661 0.330481
C -7.426768 0.919595 -1.027148
C -8.943283 1.046444 -0.975321
C -7.001943 1.649497 1.380419
H -1.115205 -3.115231 -1.046963
H -2.669454 1.109490 0.860343
H -0.384643 2.048041 0.782037
H 1.835933 3.058742 0.566425
H 2.291736 -2.405753 -1.488184
H 5.295743 0.041125 -1.634977
H 4.108788 0.184641 2.056437
H 5.739468 0.560144 1.507971
H 6.561651 -2.944669 0.787769
H 6.539868 -1.635309 -0.415943
H 7.232821 -1.359409 1.213440
H 5.021713 3.306020 -1.501543
H 3.680540 4.345144 -0.915037
H 5.340744 4.625541 -0.362551
H -4.503688 -2.435862 1.837247
H -6.131116 -1.851677 1.585863
H -5.344521 -2.801530 0.314388
H -4.790725 1.157792 -0.328034
H -7.286490 -0.353038 0.691136
H -7.004902 1.859552 -1.405377
H -7.174832 0.144473 -1.762695
H -9.254892 1.894079 -0.358268
H -9.340180 1.208286 -1.982777
H -9.400643 0.136723 -0.573877
H -6.484615 1.399460 2.313860
H -8.062463 1.770925 1.622061
H -6.620963 2.616716 1.033668
SCF Energy (B3LYP/6-31G**)= -1361.69938183
Number of imaginary frequencies = 0

1a_c125
MMFF Geometry
C 2.007935 1.660820 -0.131885
C 2.209408 -1.047524 -0.115656
N 3.242757 1.112081 -0.035872
C 0.818240 0.934776 -0.223559
C 0.934713 -0.466977 -0.216582
C 3.340643 -0.237521 -0.026146
C -0.225836 -1.256157 -0.317796
C -1.495425 -0.670763 -0.410277
C -1.606033 0.722867 -0.400825
C -0.458295 1.524798 -0.324291
C -2.956229 1.326159 -0.481627
C -4.190424 0.452740 -0.177275
C -3.912572 -0.999060 -0.504561
C -2.676779 -1.516707 -0.565874
O -0.531763 2.897082 -0.341639
O -3.066359 2.528105 -0.745033
O -5.236444 0.950199 -1.026401
C -4.565199 0.712054 1.285372
C -5.622580 -0.217936 1.838042
C -7.032226 -0.074433 1.325463
O -5.340444 -1.038649 2.713521
O -5.017618 -1.826395 -0.629334
C -5.325284 -2.082382 -2.000054
C 4.715591 -0.802955 0.099311
C 4.834355 -2.172713 0.711433
C 5.773823 -0.065759 -0.305155
C 7.239287 -0.439764 -0.286804
C 8.039654 0.455814 0.676819
C 7.636168 0.280680 2.135548
C 7.805633 -0.319221 -1.705742
H 2.001144 2.748676 -0.132248
H 2.308481 -2.128088 -0.121154
H -0.133485 -2.342004 -0.324733
H -2.536176 -2.580492 -0.735214
H -1.456881 3.164008 -0.541299
H -5.094367 1.915150 -1.107029
H -4.933865 1.737098 1.419990
H -3.681996 0.615189 1.931101
H -7.706962 -0.669625 1.947602
H -7.340829 0.972160 1.387867
H -7.105058 -0.427209 0.295840
H -5.494080 -1.153977 -2.554712
H -4.530525 -2.666955 -2.475306
H -6.246486 -2.671393 -2.032581
H 4.577965 -2.945223 -0.020968
H 5.837782 -2.387698 1.086860
H 4.168668 -2.273536 1.576416
H 5.582506 0.929545 -0.712418
H 7.377420 -1.481687 0.018037
H 9.107126 0.218655 0.586192
H 7.928133 1.511651 0.399156
H 7.730454 -0.764922 2.445407
H 8.285035 0.883690 2.778866
H 6.604918 0.602016 2.309898
H 7.257602 -0.963116 -2.402842
H 8.857570 -0.624009 -1.729461
H 7.746234 0.709788 -2.078366
SCF Energy (B3LYP/6-31G**)= -1361.70379946

Number of imaginary frequencies = 0

1a_c126
MMFF Geometry
C 1.895066 1.821089 -0.224548
C 2.102485 -0.859680 -0.609125
N 3.133955 1.274149 -0.261061
C 0.704332 1.105781 -0.369520
C 0.823197 -0.281770 -0.568325
C 3.234992 -0.061472 -0.452721
C -0.339767 -1.058471 -0.728275
C -1.613626 -0.476834 -0.677390
C -1.723692 0.899827 -0.464492
C -0.575955 1.693305 -0.328534
C -3.078142 1.492896 -0.390305
C -4.260061 0.554669 -0.053843
C -4.037650 -0.825386 -0.663073
C -2.803307 -1.297522 -0.917415
O -0.656252 3.053351 -0.148891
O -3.187998 2.710672 -0.544634
O -5.455093 1.134365 -0.589344
C -4.391776 0.539409 1.473531
C -5.336660 -0.525725 1.982234
C -6.816733 -0.274612 1.858583
O -4.903081 -1.571477 2.471348
O -5.213351 -1.513124 -0.901716
C -5.136509 -2.898445 -1.219651
C 4.616355 -0.623171 -0.501292
C 4.807718 -1.894969 -1.282838
C 5.620921 0.033805 0.120066
C 7.078840 -0.361865 0.218294
C 7.504315 -0.374613 1.699762
C 8.920463 -0.889344 1.919448
C 7.918227 0.605083 -0.619291
H 1.885603 2.897677 -0.068107
H 2.202973 -1.930993 -0.748952
H -0.244408 -2.130935 -0.895759
H -2.636031 -2.285973 -1.331297
H -1.589977 3.335355 -0.285151
H -5.436252 0.987240 -1.551191
H -4.748253 1.507382 1.848073
H -3.420100 0.364535 1.953981
H -7.359256 -0.985948 2.487912
H -7.051523 0.735260 2.204126
H -7.136335 -0.402509 0.822884
H -4.660668 -3.047978 -2.193873
H -4.610624 -3.452824 -0.435533
H -6.157708 -3.285965 -1.280728
H 4.237493 -1.869266 -2.218625
H 5.846894 -2.061502 -1.577764
H 4.481266 -2.760100 -0.696444
H 5.384604 0.966846 0.636626
H 7.232004 -1.371519 -0.175344
H 7.422204 0.631876 2.129648
H 6.817150 -1.015218 2.267886
H 9.664966 -0.214663 1.487180
H 9.128610 -0.966879 2.991527
H 9.051379 -1.881828 1.477004
H 7.553053 0.647613 -1.651899
H 8.964877 0.287830 -0.660403
H 7.888400 1.621237 -0.210113
SCF Energy (B3LYP/6-31G**)= -1361.70341185
Number of imaginary frequencies = 0

1a_c127
MMFF Geometry
C -1.663406 -1.935715 -0.177002
C -2.070819 0.734219 0.095762
N -2.932200 -1.500301 0.016192
C -0.538204 -1.110458 -0.249283
C -0.761412 0.269401 -0.108905
C -3.131066 -0.169292 0.153910
C 0.330728 1.153291 -0.179727
C 1.639391 0.686638 -0.381624
C 1.856931 -0.688021 -0.512378
C 0.774765 -1.582734 -0.454332
C 3.236385 -1.180548 -0.728962
C 4.412274 -0.190645 -0.683459
C 4.018054 1.270734 -0.591677
C 2.736577 1.655752 -0.455925
O 0.944852 -2.940359 -0.598665
O 3.416916 -2.377592 -0.982277
O 5.070742 -0.373098 -1.959893
C 5.414805 -0.612322 0.398458
C 4.922046 -0.329242 1.798790
C 5.968423 -0.010723 2.833175
O 3.731343 -0.407189 2.099655
O 5.104251 2.129302 -0.635701
C 4.861112 3.529448 -0.694303
C -4.538004 0.272534 0.385351
C -4.718625 1.530757 1.187855
C -5.550185 -0.481696 -0.098851
C -7.051084 -0.280845 -0.040809
C -7.509887 1.076768 -0.606805
C -9.013522 1.148592 -0.843652
C -7.604829 -0.583816 1.353385
H -1.571217 -3.015092 -0.276332
H -2.253181 1.799168 0.194209
H 0.152762 2.222705 -0.072405
H 2.459989 2.700917 -0.377261
H 1.896940 -3.118749 -0.768880
H 5.152530 -1.337175 -2.092134
H 6.365367 -0.085283 0.245796
H 5.649285 -1.681912 0.335301
H 5.488743 0.173000 3.798536
H 6.654196 -0.855651 2.931886
H 6.514986 0.887963 2.536633
H 4.261170 3.787895 -1.572875
H 4.383453 3.880802 0.225793
H 5.827168 4.034770 -0.785121
H -4.549464 2.414358 0.563697
H -5.714449 1.616010 1.625502
H -4.021737 1.560256 2.033451
H -5.281616 -1.391781 -0.641613
H -7.468371 -1.049047 -0.708651
H -7.232259 1.898789 0.060045
H -7.003925 1.261570 -1.563019
H -9.570494 1.090099 0.095970
H -9.272162 2.098227 -1.323240
H -9.348594 0.337775 -1.498051
H -7.195989 -1.522643 1.744052
H -8.693764 -0.694089 1.325823
H -7.371491 0.208257 2.070649
SCF Energy (B3LYP/6-31G**)= -1361.70382235
Number of imaginary frequencies = 0

1a_c128
MMFF Geometry
C 1.988795 1.814107 -0.130992
C 2.246700 -0.842312 -0.633575
N 3.238331 1.297886 -0.216549
C 0.810839 1.078869 -0.281704
C 0.956347 -0.295770 -0.541816
C 3.364566 -0.025896 -0.467039
C -0.192008 -1.091521 -0.709217
C -1.476748 -0.541382 -0.609540
C -1.615051 0.822892 -0.337256
C -0.481475 1.634875 -0.189009
C -2.979661 1.386019 -0.217947
C -4.171619 0.431754 -0.000442
C -3.887349 -0.926694 -0.605246
C -2.648441 -1.383593 -0.840291
O -0.584150 2.983987 0.052297
O -3.132421 2.611569 -0.248009
O -5.283555 1.040581 -0.675437
C -4.449951 0.408907 1.505888
C -5.449214 -0.638468 1.945104
C -6.892940 -0.448878 1.557618
O -5.092513 -1.594863 2.636258
O -4.981084 -1.753783 -0.807350
C -5.378487 -1.767133 -2.178819
C 4.756514 -0.553367 -0.567539

C 4.958383 -1.790330 -1.400539
C 5.759452 0.100806 0.059217
C 7.225745 -0.265935 0.113793
C 7.733152 -0.343311 1.567212
C 7.069209 -1.451799 2.375059
C 8.033971 0.770833 -0.670173
H 1.959604 2.882573 0.071505
H 2.366983 -1.904375 -0.820753
H -0.078061 -2.154412 -0.921157
H -2.498146 -2.393909 -1.210094
H -1.526164 3.252300 -0.034749
H -5.167654 2.008247 -0.585951
H -4.827861 1.380382 1.849657
H -3.522790 0.223830 2.064915
H -7.512438 -1.166306 2.103605
H -7.216230 0.559609 1.827668
H -7.029054 -0.613637 0.487942
H -5.604993 -0.759702 -2.541924
H -4.606917 -2.229105 -2.803715
H -6.287231 -2.370792 -2.258725
H 4.369003 -1.741564 -2.323433
H 5.994512 -1.923838 -1.721501
H 4.661795 -2.683648 -0.841123
H 5.514173 1.005632 0.619666
H 7.405321 -1.242872 -0.345713
H 8.815348 -0.524419 1.561486
H 7.578152 0.615037 2.078886
H 7.202667 -2.423941 1.889878
H 7.515998 -1.507595 3.372926
H 5.997175 -1.272094 2.500480
H 7.703040 0.818815 -1.713728
H 9.098352 0.511983 -0.671384
H 7.932514 1.772635 -0.237381
SCF Energy (B3LYP/6-31G**)= -1361.70387615
Number of imaginary frequencies = 0

1a_c129
MMFF Geometry
C -1.764913 -2.420782 0.051642
C -2.339477 0.212411 0.402726
N -3.041672 -2.083512 0.354667
C -0.712295 -1.512045 -0.094171
C -1.021180 -0.151165 0.090998
C -3.318888 -0.772367 0.518857
C -0.006016 0.813823 -0.045569
C 1.310647 0.442735 -0.348780
C 1.613211 -0.911150 -0.517488
C 0.609690 -1.884432 -0.409322
C 3.014380 -1.285756 -0.814585
C 4.126355 -0.278654 -0.440825
C 3.637403 1.153148 -0.630328
C 2.332204 1.472445 -0.555059
O 0.875747 -3.219177 -0.599688
O 3.228199 -2.401712 -1.291911
O 5.252415 -0.523135 -1.291224
C 4.548449 -0.599791 0.997624
C 5.449463 0.450981 1.606023
C 6.895955 0.470341 1.186187
O 5.005398 1.272085 2.411879
O 4.660399 2.052905 -0.867266
C 4.368828 3.444578 -0.801095
C -4.726590 -0.445201 0.854628
C -5.358722 -1.387205 1.844547
C -5.344620 0.595913 0.256105
C -6.766139 1.072778 0.462521
C -7.689793 0.639970 -0.693122
C -7.344572 1.210574 -2.063332
C -6.781157 2.588959 0.680841
H -1.602232 -3.489099 -0.075041
H -2.597750 1.254066 0.567399
H -0.252197 1.867141 0.085798
H 1.978532 2.491498 -0.668175
H 1.791179 -3.311674 -0.950992
H 5.032040 -0.159264 -2.166547
H 5.076049 -1.560731 1.048322
H 3.674670 -0.695460 1.655600
H 7.467727 1.089951 1.882998
H 7.304248 -0.542954 1.216179
H 6.992208 0.885984 0.181624
H 5.309078 3.990612 -0.921620
H 3.700422 3.740959 -1.615519
H 3.945158 3.711270 0.172615
H -4.734261 -1.474726 2.741034
H -6.345748 -1.064835 2.181738
H -5.480048 -2.384625 1.407963
H -4.792816 1.167425 -0.490612
H -7.180592 0.634815 1.376168
H -7.687861 -0.455510 -0.762561
H -8.719833 0.929476 -0.449198
H -6.341635 0.914178 -2.383862
H -8.053512 0.836761 -2.809608
H -7.404747 2.302610 -2.070885
H -6.218251 2.854338 1.583116
H -7.806417 2.951745 0.810488
H -6.332312 3.132251 -0.157539
SCF Energy (B3LYP/6-31G**)= -1361.70194833
Number of imaginary frequencies = 0

1a_c130
MMFF Geometry
C -1.681788 -1.890884 -0.285161
C -2.102794 0.752333 0.168444
N -2.955078 -1.472992 -0.082918
C -0.558307 -1.060200 -0.280920
C -0.788812 0.306155 -0.047372
C -3.160706 -0.155445 0.145376
C 0.300571 1.195769 -0.037929
C 1.612337 0.746595 -0.252457
C 1.839209 -0.615781 -0.475146
C 0.759513 -1.514552 -0.497186
C 3.225670 -1.089054 -0.707184
C 4.402018 -0.101586 -0.586698
C 3.980575 1.341126 -0.415396
C 2.707310 1.715073 -0.235878
O 0.936222 -2.858224 -0.733922
O 3.414686 -2.264319 -1.043466
O 5.080452 -0.223825 -1.857770
C 5.381475 -0.569572 0.495183
C 4.843131 -0.353300 1.890379
C 5.807749 0.165568 2.922344
O 3.679916 -0.630085 2.181989
O 4.977557 2.297053 -0.260840
C 5.624733 2.606661 -1.495342
C -4.572742 0.265676 0.384599
C -4.770353 1.464925 1.269270
C -5.574599 -0.455746 -0.166482
C -7.076917 -0.263129 -0.118217
C -7.531756 1.129037 -0.596714
C -9.031835 1.213310 -0.851328
C -7.651095 -0.662707 1.242983
H -1.584415 -2.960438 -0.458279
H -2.290040 1.807310 0.339088
H 0.119476 2.254903 0.142315
H 2.471578 2.758469 -0.047098
H 1.892606 -3.024394 -0.890849
H 5.137234 -1.182302 -2.044579
H 6.333085 -0.032905 0.391250
H 5.625437 -1.633610 0.389596
H 5.295191 0.279985 3.881464
H 6.631361 -0.542487 3.041713
H 6.189073 1.140803 2.609606
H 6.419239 1.883646 -1.700232
H 4.917019 2.655011 -2.330410
H 6.090986 3.590771 -1.388888
H -4.593957 2.390000 0.710642
H -5.773392 1.517651 1.695395
H -4.087362 1.437177 2.126187
H -5.294395 -1.325382 -0.766601
H -7.481091 -0.984620 -0.843733
H -7.267206 1.903988 0.129198
H -7.011884 1.380312 -1.530036
H -9.602892 1.089088 0.073331

H -9.286421 2.193006 -1.268567
H -9.354045 0.448532 -1.564931
H -7.244861 -1.625077 1.574619
H -8.739051 -0.773734 1.190754
H -7.431918 0.078795 2.016559
SCF Energy (B3LYP/6-31G**)= -1361.69813335
Number of imaginary frequencies = 0

1a_c131
MMFF Geometry
C -1.580795 -2.525212 -0.059124
C -2.382127 0.049181 -0.390063
N -2.912265 -2.273228 -0.085570
C -0.580033 -1.557099 -0.193336
C -1.007450 -0.229397 -0.367577
C -3.299273 -0.988869 -0.239109
C -0.049119 0.788275 -0.517232
C 1.325584 0.506587 -0.494840
C 1.750605 -0.813830 -0.313146
C 0.801587 -1.841409 -0.167108
C 3.205380 -1.116676 -0.297137
C 4.223768 0.034898 -0.484122
C 3.596596 1.402441 -0.664431
C 2.274131 1.607237 -0.646962
O 1.172076 -3.156467 -0.003472
O 3.555224 -2.296901 -0.194697
O 4.948954 -0.276242 -1.693225
C 5.247722 -0.009538 0.658022
C 4.659788 0.344295 2.006792
C 5.635174 0.772738 3.071216
O 3.457839 0.244560 2.250655
O 4.430095 2.514559 -0.687218
C 5.215096 2.604264 -1.875060
C -4.763097 -0.756588 -0.262139
C -5.543018 -1.780643 -1.042167
C -5.304289 0.290683 0.396440
C -6.773095 0.637809 0.510441
C -7.157749 1.805389 -0.418772
C -6.468513 3.132940 -0.127181
C -7.135118 0.916727 1.972647
H -1.326764 -3.574182 0.078123
H -2.733569 1.065667 -0.537552
H -0.384374 1.816758 -0.648501
H 1.884044 2.619221 -0.712882
H 2.155351 -3.204018 0.012313
H 4.291881 -0.414371 -2.397459
H 6.073766 0.680807 0.446123
H 5.698771 -1.006152 0.741818
H 5.096173 1.003073 3.994374
H 6.343149 -0.036555 3.265575
H 6.166413 1.669260 2.742202
H 6.100042 1.966260 -1.799343
H 4.630960 2.356288 -2.768043
H 5.558254 3.638744 -1.970765
H -5.665691 -2.698654 -0.457914
H -6.535982 -1.424940 -1.329976
H -5.029728 -2.031096 -1.978000
H -4.644204 0.959448 0.948941
H -7.382336 -0.222317 0.210391
H -6.941845 1.523211 -1.457352
H -8.242707 1.959923 -0.362552
H -5.381036 3.051248 -0.213231
H -6.800968 3.888927 -0.846331
H -6.712421 3.501191 0.873384
H -6.964356 0.026545 2.588906
H -8.192510 1.186760 2.065274
H -6.538495 1.730284 2.398811
SCF Energy (B3LYP/6-31G**)= -1361.69941308
Number of imaginary frequencies = 0

1a_c132
MMFF Geometry
C 1.772362 -1.547281 -0.243895
C 2.227456 1.126828 -0.110902
N 3.057206 -1.118549 -0.206296
C 0.652813 -0.712609 -0.219649
C 0.899908 0.670389 -0.151072
C 3.281230 0.214149 -0.139223
C -0.186426 1.565192 -0.131165
C -1.509068 1.102383 -0.163032
C -1.745986 -0.272495 -0.214907
C -0.676776 -1.178233 -0.262527
C -3.148845 -0.736513 -0.248841
C -4.237397 0.204696 0.322042
C -3.890542 1.661269 -0.001053
C -2.619297 2.056927 -0.206388
O -0.881909 -2.534245 -0.354096
O -3.373244 -1.865385 -0.691193
O -4.243406 0.118518 1.750476
C -5.611245 -0.188120 -0.259352
C -6.046330 -1.573825 0.175893
C -6.878202 -2.366986 -0.794880
O -5.777584 -2.005696 1.299266
O -4.987845 2.506255 -0.010970
C -4.768082 3.908742 -0.104347
C 4.708694 0.647357 -0.113011
C 5.013243 2.024784 -0.637216
C 5.652722 -0.206548 0.341399
C 7.141456 0.032222 0.475386
C 7.887269 -0.793384 -0.588164
C 9.386497 -0.529674 -0.618813
C 7.580894 -0.320113 1.900201
H 1.662489 -2.628361 -0.295935
H 2.426445 2.191282 -0.043993
H 0.006862 2.636424 -0.089842
H -2.359930 3.090136 -0.406174
H -1.841347 -2.696651 -0.498332
H -4.639870 -0.749519 1.983256
H -5.581991 -0.153603 -1.355039
H -6.405198 0.484671 0.086355
H -7.112040 -3.345293 -0.366055
H -7.810531 -1.834416 -0.997291
H -6.318462 -2.515374 -1.721628
H -4.148579 4.265475 0.724848
H -4.322738 4.170108 -1.069489
H -5.740131 4.406233 -0.037285
H 4.773384 2.784526 0.113859
H 6.061775 2.150200 -0.919667
H 4.437151 2.234464 -1.545861
H 5.335412 -1.198576 0.670616
H 7.376164 1.090861 0.325096
H 7.717895 -1.865693 -0.426901
H 7.480555 -0.557813 -1.580320
H 9.873680 -0.871772 0.298793
H 9.844345 -1.067318 -1.455470
H 9.595536 0.536974 -0.747328
H 6.979483 0.220091 2.640442
H 8.626510 -0.045174 2.070872
H 7.476518 -1.392570 2.099788
SCF Energy (B3LYP/6-31G**)= -1361.71308980
Number of imaginary frequencies = 0

1a_c133
MMFF Geometry
C -1.505153 -2.315793 -0.552230
C -2.215627 0.133008 0.384866
N -2.810453 -2.103975 -0.255066
C -0.488625 -1.364606 -0.415963
C -0.868730 -0.101646 0.070502
C -3.153469 -0.881430 0.203881
C 0.107973 0.896800 0.231536
C 1.454646 0.659387 -0.084251
C 1.833336 -0.600581 -0.562088
C 0.864806 -1.605734 -0.732583
C 3.257805 -0.854868 -0.902432
C 4.307533 0.252584 -0.647785
C 3.723587 1.565371 -0.175942
C 2.423893 1.738383 0.099152
O 1.187354 -2.852728 -1.216714
O 3.549999 -1.934191 -1.426722
O 4.954444 0.506848 -1.913581
C 5.404030 -0.285130 0.280298

C 4.937050 -0.505433 1.701580
C 5.998465 -0.480000 2.769453
O 3.762943 -0.748076 1.979029
O 4.639303 2.572817 0.092154
C 4.624385 3.583678 -0.915820
C -4.588857 -0.694258 0.524456
C -5.223736 -1.864727 1.226207
C -5.229358 0.440017 0.168666
C -6.692177 0.775738 0.374222
C -7.335087 1.105692 -0.986940
C -8.838931 1.331207 -0.908269
C -6.803709 1.935285 1.366570
H -1.286575 -3.316577 -0.918797
H -2.527333 1.094310 0.781309
H -0.190499 1.872835 0.612944
H 2.071874 2.684132 0.500666
H 2.154479 -2.876489 -1.399190
H 4.254905 0.654161 -2.572593
H 6.250482 0.413637 0.295830
H 5.802958 -1.237221 -0.091179
H 5.541059 -0.648964 3.748247
H 6.729259 -1.269346 2.577534
H 6.488976 0.496527 2.775654
H 4.832703 3.163450 -1.904816
H 3.667362 4.115665 -0.924107
H 5.411886 4.305134 -0.679241
H -4.553064 -2.268770 1.993699
H -6.147189 -1.597639 1.745968
H -5.453439 -2.662970 0.512565
H -4.672026 1.213074 -0.361110
H -7.229940 -0.079244 0.795369
H -6.865469 1.995300 -1.425809
H -7.150920 0.277779 -1.684047
H -9.081184 2.228817 -0.332118
H -9.250858 1.463628 -1.913987
H -9.341481 0.475234 -0.447098
H -6.279021 1.703355 2.300579
H -7.848035 2.136609 1.624813
H -6.372387 2.856353 0.958538
SCF Energy (B3LYP/6-31G**)= -1361.70006370
Number of imaginary frequencies = 0

1a_c134
MMFF Geometry
C 1.753277 -1.607691 0.187171
C 2.240196 1.064047 0.215722
N 3.038557 -1.193396 0.298853
C 0.648647 -0.759208 0.083795
C 0.912433 0.622382 0.096905
C 3.277928 0.138176 0.315719
C -0.157060 1.530167 -0.018180
C -1.480807 1.082570 -0.127166
C -1.735865 -0.290039 -0.120956
C -0.681302 -1.210111 -0.036116
C -3.139560 -0.737834 -0.237653
C -4.252637 0.247553 0.194583
C -3.858031 1.678962 -0.181808
C -2.568384 2.046664 -0.309823
O -0.901189 -2.566484 -0.069767
O -3.348332 -1.886948 -0.633022
O -4.368509 0.242966 1.620938
C -5.584689 -0.160883 -0.467666
C -6.074314 -1.514187 0.009544
C -6.843116 -2.351089 -0.976382
O -5.898697 -1.884693 1.172555
O -4.937200 2.535264 -0.324237
C -4.687705 3.927095 -0.480913
C 4.703131 0.555265 0.458643
C 4.959371 1.899049 1.085849
C 5.682733 -0.284496 0.056260
C 7.179413 -0.059933 0.087509
C 7.760346 -0.303621 -1.319271
C 9.243178 0.025056 -1.428182
C 7.802323 -0.978433 1.140809
H 1.630023 -2.688576 0.183657
H 2.453684 2.127808 0.215392
H 0.050283 2.599560 -0.021587
H -2.277274 3.063472 -0.546615
H -1.849469 -2.724800 -0.278104
H -4.795859 -0.605484 1.870681
H -5.471811 -0.189094 -1.558097
H -6.391366 0.540115 -0.221912
H -7.124888 -3.300508 -0.512845
H -7.748509 -1.819586 -1.279049
H -6.217224 -2.558742 -1.847775
H -4.127616 4.324311 0.371801
H -4.165563 4.125996 -1.422163
H -5.653650 4.439397 -0.517946
H 4.290353 2.067989 1.937549
H 5.972165 1.996251 1.485179
H 4.806803 2.700319 0.355400
H 5.393054 -1.252644 -0.358483
H 7.408601 0.974439 0.362125
H 7.605405 -1.347364 -1.621197
H 7.219761 0.316592 -2.046209
H 9.850916 -0.654102 -0.823423
H 9.573278 -0.073728 -2.467396
H 9.443260 1.051761 -1.106055
H 7.325634 -0.834621 2.117295
H 8.868793 -0.768455 1.268746
H 7.695700 -2.034142 0.866960
SCF Energy (B3LYP/6-31G**)= -1361.71313877
Number of imaginary frequencies = 0

1a_c135
MMFF Geometry
C -1.657887 -1.462574 -0.514796
C -2.053749 1.125319 0.207981
N -2.928137 -1.049411 -0.286770
C -0.525199 -0.652535 -0.406980
C -0.741788 0.686419 -0.033715
C -3.122361 0.239526 0.075297
C 0.357732 1.558024 0.080464
C 1.665484 1.112527 -0.155348
C 1.871477 -0.223770 -0.509044
C 0.788003 -1.100479 -0.653915
C 3.255364 -0.668271 -0.761759
C 4.414780 0.156818 -0.170612
C 4.063292 1.638122 -0.097483
C 2.783906 2.059027 -0.082079
O 0.959940 -2.407264 -1.041060
O 3.458881 -1.684576 -1.432121
O 5.535359 -0.007791 -1.058984
C 4.833569 -0.331072 1.218357
C 4.811044 -1.835990 1.334720
C 6.037516 -2.594331 0.903596
O 3.799121 -2.415259 1.731982
O 5.170514 2.461807 -0.000330
C 4.978581 3.870566 -0.012692
C -4.532484 0.651769 0.334170
C -4.743767 1.799048 1.284919
C -5.540973 -0.026799 -0.256781
C -7.028549 0.232559 -0.151388
C -7.682410 -0.884362 0.681647
C -9.162692 -0.651818 0.951553
C -7.622466 0.332426 -1.560148
H -1.571474 -2.509383 -0.798349
H -2.231466 2.159429 0.483966
H 0.184570 2.597980 0.355085
H 2.524322 3.108985 -0.003747
H 1.908281 -2.556246 -1.251975
H 5.317369 -0.748878 -1.659026
H 4.156192 0.049858 1.994356
H 5.840366 0.016526 1.480722
H 5.943473 -3.641350 1.205310
H 6.144188 -2.545608 -0.181791
H 6.921704 -2.174353 1.389432
H 4.480877 4.190747 -0.933750
H 4.418254 4.195402 0.869758
H 5.963798 4.344945 0.021023
H -4.565053 2.754650 0.781205
H -5.752930 1.823221 1.704418

H -4.069385 1.723974 2.145767
H -5.286198 -0.868274 -0.905006
H -7.217551 1.192399 0.339957
H -7.559821 -1.854205 0.182732
H -7.168936 -0.962621 1.648861
H -9.751348 -0.698319 0.030847
H -9.545914 -1.423271 1.627337
H -9.328256 0.322462 1.421997
H -7.086480 1.077579 -2.159263
H -8.672295 0.640172 -1.525752
H -7.568396 -0.626168 -2.088417
SCF Energy (B3LYP/6-31G**)= -1361.70530523
Number of imaginary frequencies = 0

1a_c136
MMFF Geometry
C 2.007740 1.603091 -0.054429
C 2.106830 -1.109415 -0.155152
N 3.216950 1.002616 0.057383
C 0.795916 0.928458 -0.218313
C 0.858923 -0.475888 -0.271589
C 3.263792 -0.348792 0.008917
C -0.326462 -1.213977 -0.448735
C -1.569629 -0.576048 -0.554245
C -1.625732 0.818380 -0.483001
C -0.452686 1.571517 -0.334371
C -2.950462 1.473278 -0.572317
C -4.199487 0.624409 -0.240943
C -4.002486 -0.817194 -0.697270
C -2.778035 -1.364617 -0.808030
O -0.477355 2.944901 -0.293920
O -2.987417 2.673247 -0.850786
O -5.321653 1.199106 -0.920159
C -4.443125 0.765411 1.266118
C -5.473501 -0.204247 1.799176
C -6.926562 0.094807 1.538138
O -5.128330 -1.213798 2.417638
O -5.190463 -1.473890 -0.961180
C -5.159925 -2.885438 -1.141970
C 4.611175 -0.972431 0.153020
C 4.660199 -2.374953 0.697064
C 5.712203 -0.257931 -0.169675
C 7.156341 -0.704706 -0.118412
C 7.879512 -0.165475 1.130864
C 7.990024 1.351635 1.223340
C 7.872585 -0.315898 -1.415810
H 2.041298 2.689411 -0.005617
H 2.165989 -2.191613 -0.206929
H -0.273057 -2.300842 -0.505245
H -2.630471 -2.395228 -1.111977
H -1.383250 3.251608 -0.528905
H -5.239614 0.957798 -1.859415
H -4.778320 1.779573 1.518005
H -3.518918 0.598599 1.834960
H -7.547704 -0.528928 2.187384
H -7.136536 1.142340 1.768252
H -7.175352 -0.119727 0.497346
H -4.621476 -3.148602 -2.057784
H -4.721917 -3.383586 -0.270921
H -6.191968 -3.232856 -1.246561
H 4.407043 -3.100059 -0.083309
H 5.642393 -2.641036 1.097021
H 3.959349 -2.496334 1.531020
H 5.575434 0.765476 -0.524888
H 7.203674 -1.798577 -0.068831
H 7.365330 -0.531188 2.029031
H 8.891682 -0.588072 1.164000
H 7.005576 1.828362 1.238863
H 8.506621 1.629939 2.147945
H 8.564166 1.764396 0.388937
H 7.409169 -0.812405 -2.276105
H 8.924276 -0.620065 -1.383890
H 7.836779 0.762923 -1.601075
SCF Energy (B3LYP/6-31G**)= -1361.70252439
Number of imaginary frequencies = 0

1a_c137
MMFF Geometry
C -1.429348 -2.314429 -0.708237
C -2.404631 0.199449 -0.366661
N -2.774498 -2.154463 -0.668863
C -0.497605 -1.278720 -0.587299
C -1.014491 0.019383 -0.413686
C -3.248207 -0.903033 -0.488346
C -0.129677 1.107524 -0.300269
C 1.257909 0.919862 -0.341934
C 1.764931 -0.375141 -0.496926
C 0.898327 -1.467736 -0.637290
C 3.232153 -0.550391 -0.549887
C 4.135863 0.562285 0.017934
C 3.478273 1.919760 -0.141795
C 2.151463 2.076771 -0.267744
O 1.366949 -2.744508 -0.832000
O 3.709367 -1.575735 -1.045538
O 5.348957 0.542287 -0.754563
C 4.495144 0.340079 1.488878
C 4.754132 -1.111833 1.810642
C 6.154014 -1.633390 1.628143
O 3.832819 -1.845662 2.170550
O 4.297265 3.026020 0.027329
C 4.664946 3.592374 -1.231006
C -4.724390 -0.773087 -0.453480
C -5.446791 -1.614128 -1.471527
C -5.323658 0.031158 0.450662
C -6.810458 0.236010 0.646993
C -7.286816 1.573100 0.047069
C -6.683238 2.826040 0.670201
C -7.167221 0.106379 2.131185
H -1.104705 -3.343717 -0.846117
H -2.826051 1.193214 -0.250700
H -0.533677 2.112158 -0.179449
H 1.716711 3.071699 -0.295767
H 2.345914 -2.716932 -0.914158
H 5.393514 -0.336067 -1.184386
H 3.680013 0.661724 2.150900
H 5.381665 0.918145 1.777309
H 6.221934 -2.646386 2.034750
H 6.406966 -1.659606 0.566468
H 6.858587 -0.996725 2.168910
H 5.176234 2.863828 -1.867817
H 3.787417 3.994928 -1.748121
H 5.354465 4.419402 -1.037964
H -5.499299 -2.657201 -1.142307
H -6.465470 -1.264985 -1.660554
H -4.932619 -1.581316 -2.439271
H -4.701217 0.579852 1.157449
H -7.365544 -0.558552 0.135551
H -7.068956 1.581272 -1.028736
H -8.378649 1.633348 0.138970
H -5.594224 2.843822 0.568139
H -7.076710 3.716102 0.168015
H -6.935394 2.908677 1.731248
H -6.927680 -0.897868 2.499241
H -8.238700 0.270555 2.287887
H -6.619641 0.822927 2.752607
SCF Energy (B3LYP/6-31G**)= -1361.69827785
Number of imaginary frequencies = 0

1a_c138
MMFF Geometry
C 1.592521 -2.312470 -0.243100
C 2.478488 0.228392 -0.622935
N 2.928143 -2.116189 -0.361541
C 0.628113 -1.301709 -0.302252
C 1.098225 0.009746 -0.502955
C 3.358787 -0.848921 -0.538858
C 0.176325 1.069465 -0.588572
C -1.200864 0.844764 -0.458165
C -1.658899 -0.456130 -0.240431
C -0.757538 -1.528750 -0.181693
C -3.115600 -0.667888 -0.107146
C -3.984189 0.532505 0.341613

C -3.438193 1.828302 -0.265889
C -2.143071 1.955426 -0.614111
O -1.185517 -2.823743 -0.010881
O -3.556546 -1.799652 -0.319455
O -3.877242 0.697214 1.759130
C -5.449338 0.289820 -0.075763
C -6.060620 -0.902733 0.633498
C -7.092248 -1.694612 -0.122933
O -5.765581 -1.174183 1.799678
O -4.382365 2.836264 -0.369440
C -3.949797 4.141130 -0.735821
C 4.826978 -0.675637 -0.665874
C 5.502467 -1.731744 -1.499514
C 5.449920 0.338175 -0.026932
C 6.927224 0.666828 -0.029605
C 7.616267 0.221602 1.275373
C 7.127240 0.905041 2.546595
C 7.129148 2.158708 -0.312801
H 1.304137 -3.350735 -0.092892
H 2.862875 1.229172 -0.794173
H 0.542536 2.081304 -0.757959
H -1.739873 2.877267 -1.017083
H -2.167597 -2.838645 -0.065975
H -4.385528 -0.035174 2.171569
H -5.512271 0.128686 -1.158654
H -6.091271 1.139823 0.184889
H -7.440924 -2.528308 0.492725
H -7.941275 -1.051032 -0.365412
H -6.648494 -2.095932 -1.037380
H -3.210239 4.523929 -0.025100
H -3.555484 4.148724 -1.756901
H -4.819756 4.803855 -0.706838
H 5.014771 -1.816351 -2.477419
H 6.556551 -1.523800 -1.693223
H 5.454980 -2.707128 -1.002987
H 4.852210 1.002912 0.597428
H 7.428661 0.136788 -0.845675
H 7.492492 -0.862576 1.394289
H 8.695405 0.398235 1.182858
H 6.062457 0.723703 2.719287
H 7.673244 0.513338 3.411297
H 7.296229 1.985101 2.512912
H 6.739438 2.419469 -1.303517
H 8.193706 2.415679 -0.296643
H 6.615951 2.791959 0.418727
SCF Energy (B3LYP/6-31G**)= -1361.71175964
Number of imaginary frequencies = 0

1a_c139
MMFF Geometry
C -1.639162 -1.362570 -0.946699
C -2.067234 1.184329 -0.106152
N -2.919633 -0.945380 -0.796497
C -0.511169 -0.576498 -0.700806
C -0.744289 0.740702 -0.265278
C -3.130114 0.323620 -0.377501
C 0.350857 1.586153 -0.005368
C 1.669041 1.135835 -0.159996
C 1.889948 -0.178983 -0.579129
C 0.813321 -1.028472 -0.866784
C 3.285369 -0.628333 -0.744721
C 4.400113 0.140253 -0.009183
C 4.059004 1.619369 0.128762
C 2.787204 2.058366 0.065245
O 1.003410 -2.311846 -1.318371
O 3.533353 -1.603982 -1.459069
O 5.588732 0.014192 -0.811236
C 4.697139 -0.439896 1.375688
C 4.650951 -1.948675 1.393608
C 5.902164 -2.696264 1.018417
O 3.603962 -2.536966 1.667619
O 5.162717 2.418017 0.369238
C 4.986863 3.827514 0.432225
C -4.553025 0.749878 -0.238285
C -4.841863 2.219045 -0.388876
C -5.505227 -0.183460 -0.017055
C -6.992192 0.023574 0.176656
C -7.432970 -0.649210 1.491561
C -8.889741 -0.386317 1.848471
C -7.738111 -0.531121 -1.038673
H -1.540198 -2.392000 -1.284570
H -2.256380 2.194615 0.241231
H 0.166175 2.609594 0.319242
H 2.532867 3.105273 0.188627
H 1.964335 -2.462028 -1.460134
H 5.414022 -0.685266 -1.472310
H 3.961069 -0.098530 2.115814
H 5.681801 -0.124206 1.741858
H 5.773537 -3.758629 1.244160
H 6.099764 -2.581305 -0.049083
H 6.746419 -2.320367 1.601631
H 4.570841 4.213299 -0.504080
H 4.358375 4.104246 1.284648
H 5.970644 4.283436 0.577604
H -4.259456 2.650592 -1.211118
H -5.887616 2.424753 -0.631216
H -4.597880 2.756007 0.533629
H -5.196306 -1.229356 0.046748
H -7.225664 1.089623 0.259586
H -7.270842 -1.733431 1.439162
H -6.808372 -0.278218 2.314711
H -9.570046 -0.851844 1.129579
H -9.115087 -0.804997 2.834684
H -9.099180 0.687457 1.881159
H -7.359646 -0.087422 -1.966756
H -8.807275 -0.303424 -0.985190
H -7.627129 -1.618565 -1.116344
SCF Energy (B3LYP/6-31G**)= -1361.70537309
Number of imaginary frequencies = 0

1a_c140
MMFF Geometry
C 1.456238 -2.213488 0.446898
C 2.309759 0.193893 -0.481534
N 2.768305 -2.081407 0.134482
C 0.500901 -1.199390 0.328040
C 0.953948 0.042676 -0.155492
C 3.183799 -0.879390 -0.318166
C 0.041946 1.105373 -0.295880
C -1.312656 0.946148 0.025951
C -1.756050 -0.294690 0.492151
C -0.858978 -1.357981 0.661621
C -3.184270 -0.436266 0.833787
C -4.190610 0.559235 0.224949
C -3.561311 1.932375 0.021040
C -2.227125 2.088538 -0.077906
O -1.262598 -2.574940 1.154884
O -3.545549 -1.342274 1.590299
O -5.272315 0.682971 1.166523
C -4.770563 0.068163 -1.103654
C -5.049581 -1.415423 -1.100569
C -6.376212 -1.881594 -0.563977
O -4.193356 -2.211475 -1.488596
O -4.490102 2.950765 -0.097566
C -4.026469 4.290463 -0.206488
C 4.625276 -0.777163 -0.654654
C 5.178033 -1.982424 -1.367876
C 5.334552 0.311490 -0.286051
C 6.802449 0.587326 -0.530453
C 7.648752 0.353144 0.736398
C 7.333306 1.261770 1.918402
C 6.980737 2.000368 -1.094548
H 1.179450 -3.201080 0.810130
H 2.678939 1.137064 -0.872367
H 0.400359 2.068100 -0.658676
H -1.772157 3.057833 -0.249452
H -2.208462 -2.516531 1.415638
H -5.170577 -0.042256 1.814956
H -4.074211 0.250794 -1.933024
H -5.702336 0.590181 -1.353842
H -6.505519 -2.944858 -0.785056
H -6.413276 -1.736517 0.517410

H -7.185488 -1.328780 -1.047376
H -3.426294 4.570117 0.665380
H -3.462568 4.433663 -1.133695
H -4.900473 4.947622 -0.239431
H 4.568133 -2.218978 -2.247291
H 6.200519 -1.844795 -1.724851
H 5.185195 -2.855415 -0.706057
H 4.828130 1.092925 0.281046
H 7.190058 -0.091811 -1.296528
H 7.530800 -0.689516 1.058653
H 8.708782 0.476502 0.480630
H 6.297739 1.147230 2.251493
H 7.981829 1.008552 2.763718
H 7.506999 2.313699 1.674476
H 6.469796 2.097857 -2.059340
H 8.041007 2.222136 -1.256496
H 6.571628 2.766831 -0.427708
SCF Energy (B3LYP/6-31G**)= -1361.70396563
Number of imaginary frequencies = 0

1a_c141
MMFF Geometry
C -1.423085 -2.153113 -0.602055
C -2.305914 0.205289 0.420613
N -2.745048 -2.034116 -0.328659
C -0.471332 -1.149092 -0.398035
C -0.939992 0.067379 0.133378
C -3.174225 -0.855830 0.172020
C -0.032615 1.118402 0.360646
C 1.331388 0.972563 0.075701
C 1.792446 -0.243165 -0.439496
C 0.899576 -1.293987 -0.692321
C 3.232429 -0.372207 -0.747180
C 4.229358 0.612253 -0.101418
C 3.570289 1.953014 0.169271
C 2.242005 2.098125 0.285507
O 1.317293 -2.486575 -1.232004
O 3.615709 -1.257615 -1.518549
O 5.286718 0.788419 -1.060518
C 4.836765 0.074549 1.196789
C 5.109516 -1.409331 1.144709
C 6.444757 -1.859883 0.616366
O 4.245012 -2.215972 1.489174
O 4.387807 3.021201 0.512148
C 4.938874 3.666970 -0.635606
C -4.625737 -0.770818 0.461801
C -5.203625 -2.007448 1.096120
C -5.326712 0.336677 0.137012
C -6.812175 0.573138 0.317023
C -7.441207 0.921790 -1.045960
C -8.957630 1.051333 -0.998355
C -7.019631 1.679650 1.353529
H -1.134874 -3.121269 -1.006318
H -2.686997 1.127917 0.847364
H -0.400332 2.061695 0.763345
H 1.822211 3.059955 0.566595
H 2.273499 -2.421394 -1.449360
H 5.254734 0.014673 -1.659995
H 4.160242 0.232276 2.047510
H 5.775136 0.586962 1.442179
H 6.549594 -2.938804 0.761390
H 6.518909 -1.636741 -0.449720
H 7.246387 -1.358261 1.164051
H 5.870472 3.180701 -0.936898
H 4.232994 3.698717 -1.473195
H 5.178313 4.697139 -0.355014
H -4.527846 -2.403291 1.863403
H -6.154047 -1.820108 1.601942
H -5.366251 -2.786047 0.343408
H -4.806079 1.164700 -0.344949
H -7.305746 -0.330536 0.687568
H -7.017354 1.856604 -1.434569
H -7.189048 0.137638 -1.771794
H -9.269146 1.906666 -0.391970
H -9.352512 1.201745 -2.008369
H -9.416972 0.147077 -0.586956
H -6.504307 1.440007 2.290799
H -8.080408 1.805398 1.591815
H -6.636687 2.642160 0.996000
SCF Energy (B3LYP/6-31G**)= -1361.69868590
Number of imaginary frequencies = 0

1a_c142
MMFF Geometry
C -1.785279 -1.571566 -0.453561
C -2.058884 1.045801 0.211509
N -3.035739 -1.093638 -0.242243
C -0.614640 -0.814766 -0.358647
C -0.769036 0.538762 -0.016486
C -3.168639 0.210391 0.092517
C 0.371261 1.354875 0.088608
C 1.659067 0.845914 -0.140640
C 1.811773 -0.504099 -0.473640
C 0.679139 -1.329814 -0.585853
C 3.173001 -1.044701 -0.724639
C 4.397179 -0.102564 -0.615274
C 4.056512 1.326176 -0.242736
C 2.805863 1.741348 -0.008846
O 0.778335 -2.659146 -0.926914
O 3.277548 -2.224705 -1.074560
O 4.993573 -0.081184 -1.929980
C 5.435865 -0.735144 0.320655
C 4.988038 -0.792743 1.765034
C 6.072623 -0.959431 2.796467
O 3.802194 -0.756576 2.091471
O 5.098382 2.206062 0.026299
C 5.836622 2.575621 -1.137179
C -4.557957 0.695552 0.337205
C -4.715748 1.871269 1.263363
C -5.596476 0.054405 -0.243447
C -7.069955 0.387652 -0.148955
C -7.779448 -0.678591 0.704710
C -9.247430 -0.369026 0.964248
C -7.654551 0.486607 -1.561674
H -1.748023 -2.627419 -0.712971
H -2.185827 2.092494 0.467344
H 0.249393 2.403660 0.358022
H 2.629028 2.761228 0.321556
H 1.730660 -2.882532 -1.038594
H 4.293713 0.159895 -2.561532
H 6.376329 -0.172331 0.269772
H 5.676561 -1.757966 0.005033
H 5.630210 -0.985203 3.796169
H 6.604888 -1.897095 2.619155
H 6.764415 -0.115317 2.740684
H 6.574669 1.807185 -1.383556
H 5.178607 2.772143 -1.990712
H 6.379999 3.497340 -0.908616
H -4.488689 2.806343 0.740940
H -5.723894 1.953595 1.677886
H -4.048740 1.781019 2.128489
H -5.380782 -0.811727 -0.873033
H -7.213521 1.365606 0.321604
H -7.702699 -1.663475 0.226533
H -7.272973 -0.761341 1.675235
H -9.835177 -0.406250 0.042540
H -9.669425 -1.106682 1.654514
H -9.366813 0.621774 1.413707
H -7.081464 1.192217 -2.174178
H -8.688314 0.845472 -1.537570
H -7.645600 -0.484342 -2.069682
SCF Energy (B3LYP/6-31G**)= -1361.70124233
Number of imaginary frequencies = 0

1a_c143
MMFF Geometry
C -2.014341 -1.647780 -0.531019
C -2.170758 1.063108 -0.484061
N -3.243127 -1.078683 -0.494503
C -0.809472 -0.941455 -0.544857
C -0.902179 0.461575 -0.520145
C -3.318957 0.272171 -0.471298

C 0.276210 1.230930 -0.537488
C 1.540075 0.625134 -0.565708
C 1.623824 -0.768745 -0.577466
C 0.460505 -1.551802 -0.582748
C 2.963920 -1.391578 -0.588581
C 4.184680 -0.545544 -0.180280
C 3.973705 0.929675 -0.485779
C 2.744258 1.455462 -0.639863
O 0.512834 -2.924619 -0.620127
O 3.071414 -2.588884 -0.872869
O 5.281464 -1.049728 -0.960614
C 4.453594 -0.841095 1.299569
C 5.482039 0.069900 1.932464
C 6.930951 -0.147872 1.581167
O 5.139494 0.950300 2.724856
O 5.153047 1.648826 -0.558646
C 5.090722 3.059239 -0.727129
C -4.690722 0.860396 -0.447364
C -4.844845 2.242616 -1.022563
C -5.711167 0.133058 0.059845
C -7.167220 0.519248 0.197962
C -7.596948 0.619886 1.676451
C -7.560259 -0.680091 2.470779
C -8.040977 -0.454286 -0.596735
H -2.025731 -2.735457 -0.549239
H -2.250407 2.144770 -0.452455
H 0.201288 2.318045 -0.530108
H 2.592028 2.510789 -0.837983
H 1.442867 -3.202945 -0.777965
H 5.108775 -2.000917 -1.108562
H 4.799529 -1.873253 1.439131
H 3.531407 -0.743895 1.888041
H 7.560120 0.444572 2.251717
H 7.186815 -1.202260 1.712401
H 7.125929 0.161405 0.553190
H 4.634145 3.317956 -1.687722
H 4.553784 3.529345 0.103039
H 6.114992 3.443504 -0.726666
H -4.272109 2.345074 -1.951585
H -5.876396 2.486871 -1.285733
H -4.495019 2.996820 -0.309907
H -5.486008 -0.868672 0.431152
H -7.338738 1.511767 -0.229707
H -6.961854 1.356628 2.185189
H -8.619390 1.016749 1.717925
H -6.551254 -1.099439 2.515967
H -7.885460 -0.494028 3.499857
H -8.231921 -1.430938 2.044996
H -7.803929 -0.400779 -1.665523
H -9.101756 -0.208286 -0.479475
H -7.895667 -1.492700 -0.280165
SCF Energy (B3LYP/6-31G**)= -1361.70841100
Number of imaginary frequencies = 0

1a_c144
MMFF Geometry
C -1.541609 -2.545463 -0.093314
C -2.335872 0.034229 -0.398948
N -2.872373 -2.289091 -0.113127
C -0.538244 -1.579358 -0.222290
C -0.961986 -0.248745 -0.383605
C -3.255778 -1.002243 -0.253906
C -0.001091 0.767258 -0.529063
C 1.372806 0.481444 -0.512274
C 1.794040 -0.842366 -0.340952
C 0.842413 -1.868527 -0.203430
C 3.248234 -1.150182 -0.327106
C 4.269320 0.007946 -0.423809
C 3.647224 1.367149 -0.653149
C 2.324601 1.579596 -0.671867
O 1.209328 -3.186408 -0.055173
O 3.594737 -2.334891 -0.287055
O 5.105578 -0.273871 -1.566910
C 5.201401 -0.028439 0.793842
C 4.520120 0.358457 2.087233
C 5.408430 0.884175 3.183665
O 3.312321 0.205873 2.266971
O 4.531925 2.434333 -0.714484
C 4.689669 2.915228 -2.049370
C -4.718913 -0.765118 -0.269913
C -5.504677 -1.779480 -1.056714
C -5.254541 0.277687 0.400198
C -6.721856 0.628297 0.522462
C -7.105949 1.805794 -0.394387
C -6.411552 3.128374 -0.092630
C -7.078069 0.894529 1.988454
H -1.290421 -3.596384 0.033911
H -2.684773 1.052975 -0.536345
H -0.334177 1.797320 -0.652469
H 1.934893 2.588352 -0.772414
H 2.192311 -3.239943 -0.064087
H 4.517075 -0.453279 -2.319783
H 6.049468 0.649108 0.630420
H 5.632919 -1.027870 0.930052
H 4.804727 1.129073 4.061943
H 6.141522 0.121614 3.457584
H 5.914655 1.790574 2.842470
H 5.036086 2.122668 -2.720158
H 3.755852 3.347315 -2.423875
H 5.447027 3.704418 -2.034465
H -5.628352 -2.702436 -0.480519
H -6.497443 -1.417991 -1.337925
H -4.995285 -2.022934 -1.996514
H -4.590465 0.939084 0.956763
H -7.334816 -0.227047 0.216380
H -6.894431 1.532761 -1.436309
H -8.190219 1.963178 -0.333031
H -5.324633 3.044102 -0.183130
H -6.744034 3.892151 -0.803489
H -6.650935 3.487924 0.912184
H -6.908043 -0.001961 2.595707
H -8.134285 1.166979 2.087216
H -6.477448 1.702157 2.420241
SCF Energy (B3LYP/6-31G**)= -1361.69906463
Number of imaginary frequencies = 0

1a_c145
MMFF Geometry
C -1.581538 -2.401674 -0.110109
C -2.271857 0.226270 -0.065003
N -2.894598 -2.093741 0.023432
C -0.547605 -1.466362 -0.228121
C -0.917244 -0.109929 -0.202286
C -3.224649 -0.784839 0.038144
C 0.077399 0.877974 -0.314965
C 1.433010 0.539467 -0.450818
C 1.796864 -0.810262 -0.470943
C 0.813223 -1.808926 -0.367212
C 3.226206 -1.167553 -0.618817
C 4.290254 -0.057365 -0.623222
C 3.742883 1.356488 -0.651658
C 2.424692 1.611768 -0.574156
O 1.129520 -3.147535 -0.400637
O 3.538453 -2.353933 -0.775977
O 5.002030 -0.261934 -1.867251
C 5.298542 -0.289095 0.509496
C 4.736850 0.043028 1.872558
C 5.710680 0.555598 2.899769
O 3.553705 -0.145216 2.153848
O 4.734238 2.320301 -0.736456
C 4.347950 3.678717 -0.905220
C -4.668558 -0.488481 0.190017
C -5.337313 -1.254033 1.295363
C -5.260997 0.399553 -0.637084
C -6.705624 0.841754 -0.768820
C -6.933024 2.240523 -0.161755
C -6.747702 2.331073 1.347400
C -7.786002 -0.158581 -0.350418
H -1.371074 -3.469029 -0.119319
H -2.581134 1.266183 -0.026157
H -0.215233 1.927152 -0.293877
H 2.038085 2.624478 -0.581241

H 2.099572 -3.235142 -0.537105
H 5.188422 -1.218859 -1.923580
H 6.193219 0.323032 0.337758
H 5.645048 -1.329441 0.533927
H 5.185472 0.757354 3.837436
H 6.482622 -0.196611 3.079260
H 6.162801 1.484842 2.544237
H 3.750871 3.807711 -1.813773
H 3.808850 4.042749 -0.024858
H 5.258187 4.276107 -1.012248
H -4.621178 -1.558182 2.068041
H -6.079446 -0.643137 1.814692
H -5.817255 -2.157253 0.905887
H -4.632902 0.873937 -1.394449
H -6.854000 0.960175 -1.853218
H -6.248597 2.955458 -0.636778
H -7.947260 2.577877 -0.409960
H -5.730382 2.061426 1.645488
H -6.927922 3.358071 1.682059
H -7.449982 1.683588 1.879588
H -7.587985 -1.150859 -0.769771
H -8.762205 0.165573 -0.729522
H -7.886611 -0.255143 0.732399
SCF Energy (B3LYP/6-31G**)= -1361.70102160
Number of imaginary frequencies = 0

1a_c146
MMFF Geometry
C -1.724357 -1.190436 -1.059011
C -2.082596 1.303392 -0.044479
N -2.992912 -0.750515 -0.875645
C -0.575365 -0.453034 -0.764698
C -0.772273 0.836988 -0.239385
C -3.168756 0.491569 -0.369229
C 0.345635 1.633874 0.071695
C 1.650781 1.159932 -0.118669
C 1.836946 -0.129316 -0.626111
C 0.736206 -0.927497 -0.966713
C 3.221740 -0.609170 -0.820224
C 4.342123 0.070100 0.002226
C 4.050078 1.557046 0.194265
C 2.793582 2.038934 0.150901
O 0.890685 -2.181113 -1.507136
O 3.417135 -1.539903 -1.603667
O 5.581141 -0.071092 -0.705364
C 4.521544 -0.597648 1.367479
C 4.435914 -2.103982 1.285480
C 5.642271 -2.857382 0.792393
O 3.390206 -2.683118 1.585320
O 5.183687 2.311666 0.432928
C 5.041295 3.709105 0.659733
C -4.580421 0.944342 -0.195436
C -4.827275 2.427809 -0.249540
C -5.551883 0.020408 -0.022041
C -7.034605 0.238118 0.183158
C -7.495079 -0.224820 1.581150
C -7.375405 -1.717941 1.861346
C -7.820297 -0.439602 -0.942047
H -1.653437 -2.196761 -1.466512
H -2.243860 2.292076 0.372031
H 0.188864 2.637825 0.464566
H 2.570189 3.088752 0.306498
H 1.848111 -2.342461 -1.667300
H 5.538731 0.523261 -1.474599
H 3.743204 -0.276363 2.072343
H 5.485722 -0.337936 1.821721
H 5.527881 -3.919603 1.026462
H 5.741512 -2.739398 -0.288360
H 6.540014 -2.490703 1.296283
H 4.612721 4.205406 -0.216821
H 4.438840 3.900405 1.553500
H 6.038492 4.125726 0.829433
H -4.245601 2.891482 -1.054692
H -5.869028 2.683326 -0.455852
H -4.549775 2.898646 0.699338
H -5.258303 -1.031312 -0.021085
H -7.272138 1.305010 0.130148
H -6.924844 0.319435 2.345064
H -8.544659 0.065353 1.718231
H -6.339009 -2.060972 1.796581
H -7.730528 -1.933730 2.874607
H -7.982462 -2.307349 1.168329
H -7.570263 0.008087 -1.910768
H -8.898225 -0.319844 -0.789570
H -7.603411 -1.511029 -1.010157
SCF Energy (B3LYP/6-31G**)= -1361.69971827
Number of imaginary frequencies = 0

1a_c147
MMFF Geometry
C -1.452549 -2.268407 -0.601480
C -2.375169 0.273777 -0.324109
N -2.791630 -2.085528 -0.500589
C -0.502210 -1.243164 -0.574804
C -0.991908 0.070258 -0.432967
C -3.238269 -0.819963 -0.352943
C -0.088722 1.149346 -0.416831
C 1.290829 0.935352 -0.515978
C 1.771378 -0.373410 -0.637385
C 0.885974 -1.457074 -0.689219
C 3.236193 -0.567288 -0.735547
C 4.145307 0.535756 -0.133226
C 3.530710 1.901352 -0.422352
C 2.210759 2.071117 -0.590319
O 1.329807 -2.745794 -0.858470
O 3.665936 -1.600312 -1.250383
O 5.440743 0.470883 -0.735790
C 4.326542 0.316304 1.373288
C 4.505543 -1.143402 1.725851
C 5.854952 -1.768603 1.491911
O 3.556775 -1.796628 2.162377
O 4.365414 2.979772 -0.680257
C 5.159829 3.371130 0.436155
C -4.708962 -0.664357 -0.250321
C -5.490788 -1.530936 -1.200532
C -5.247103 0.179630 0.656021
C -6.715471 0.430918 0.923347
C -7.104757 1.886415 0.605083
C -6.967965 2.235366 -0.871790
C -7.020964 0.115391 2.391314
H -1.149033 -3.307690 -0.709512
H -2.777495 1.277946 -0.232686
H -0.471400 2.165719 -0.331423
H 1.807184 3.049310 -0.836786
H 2.300521 -2.730015 -1.015218
H 5.319859 0.677385 -1.680925
H 3.452772 0.672520 1.934562
H 5.195424 0.854980 1.767532
H 5.900083 -2.736434 1.999288
H 6.018006 -1.919389 0.423066
H 6.636908 -1.127425 1.906314
H 4.564902 3.418282 1.354622
H 6.012545 2.698468 0.561497
H 5.551658 4.372365 0.233827
H -5.530210 -2.563079 -0.836678
H -6.516634 -1.183975 -1.346259
H -5.029114 -1.531571 -2.194966
H -4.580170 0.737627 1.313865
H -7.345465 -0.230776 0.320887
H -8.148042 2.053182 0.901188
H -6.495759 2.583009 1.195163
H -7.558467 1.553479 -1.491918
H -7.328185 3.253461 -1.051290
H -5.925827 2.189119 -1.201985
H -6.764925 -0.922853 2.630793
H -8.087238 0.251507 2.602356
H -6.458951 0.767132 3.069980
SCF Energy (B3LYP/6-31G**)= -1361.69391672
Number of imaginary frequencies = 0

1a_c148
MMFF Geometry

C 1.866931 -1.444145 -0.193242
C 2.258153 1.242065 -0.110748
N 3.141059 -0.984294 -0.159804
C 0.727874 -0.636020 -0.188846
C 0.941918 0.753504 -0.146375
C 3.333421 0.354405 -0.117803
C -0.165384 1.622461 -0.147477
C -1.476570 1.127843 -0.174666
C -1.680670 -0.253019 -0.200530
C -0.590138 -1.133853 -0.227008
C -3.072009 -0.750796 -0.230019
C -4.184671 0.175191 0.318851
C -3.871201 1.633050 -0.031422
C -2.608922 2.054729 -0.240253
O -0.762745 -2.495874 -0.292848
O -3.267957 -1.893045 -0.651032
O -4.193973 0.116623 1.748666
C -5.546649 -0.261347 -0.259275
C -5.950417 -1.648291 0.201457
C -6.759634 -2.479671 -0.756399
O -5.675721 -2.051781 1.333907
O -4.988163 2.451383 -0.061404
C -4.801329 3.856617 -0.181390
C 4.750241 0.821435 -0.095607
C 5.024152 2.199834 -0.634313
C 5.715313 -0.005140 0.365278
C 7.195697 0.276855 0.493920
C 8.002931 -0.361933 -0.652709
C 7.952069 -1.883314 -0.724847
C 7.696773 -0.166086 1.872386
H 1.782945 -2.528334 -0.224646
H 2.431315 2.312082 -0.064201
H 0.002313 2.698583 -0.126395
H -2.373350 3.089736 -0.459281
H -1.717523 -2.683793 -0.437026
H -4.570636 -0.755905 1.997014
H -5.514128 -0.247408 -1.355325
H -6.357598 0.398976 0.070657
H -6.971839 -3.454753 -0.309363
H -7.703552 -1.973425 -0.972203
H -6.193076 -2.632674 -1.678240
H -4.193557 4.243996 0.642720
H -4.358691 4.109699 -1.149977
H -5.785132 4.332088 -0.127229
H 4.756931 2.962482 0.104483
H 6.072551 2.348838 -0.906523
H 4.452521 2.382605 -1.551502
H 5.420842 -1.000087 0.705426
H 7.372019 1.357546 0.446539
H 7.648916 0.042723 -1.609662
H 9.052568 -0.055533 -0.560198
H 6.929687 -2.247458 -0.861896
H 8.544739 -2.234007 -1.576303
H 8.367843 -2.342541 0.176532
H 7.189079 0.394285 2.665867
H 8.771832 0.018359 1.971483
H 7.516768 -1.230709 2.055788
SCF Energy (B3LYP/6-31G**)= -1361.71228986
Number of imaginary frequencies = 0

1a_c149
MMFF Geometry
C -1.768518 -1.413158 -0.996870
C -2.071900 1.104497 -0.028994
N -3.027666 -0.942328 -0.824856
C -0.602738 -0.696367 -0.715509
C -0.772320 0.605248 -0.215591
C -3.175947 0.312335 -0.340931
C 0.362981 1.377788 0.087202
C 1.660547 0.877143 -0.101915
C 1.827676 -0.421346 -0.594585
C 0.700534 -1.203333 -0.903243
C 3.199889 -0.951862 -0.803857
C 4.419582 -0.056172 -0.474748
C 4.062612 1.318901 0.052424
C 2.800846 1.725062 0.237300
O 0.815148 -2.479845 -1.403908
O 3.321950 -2.078556 -1.295227
O 5.122086 0.123168 -1.723324
C 5.372101 -0.824807 0.450949
C 4.806710 -1.059447 1.834900
C 5.801486 -1.377439 2.919908
O 3.598362 -1.043758 2.066695
O 5.086883 2.135809 0.516761
C 5.921996 2.637587 -0.525374
C -4.576526 0.799992 -0.176141
C -4.791203 2.288264 -0.237014
C -5.574989 -0.095995 -0.011283
C -7.050183 0.173057 0.195599
C -7.527173 -0.555281 1.467582
C -8.969840 -0.241966 1.840718
C -7.819965 -0.269035 -1.050640
H -1.719827 -2.428315 -1.384925
H -2.209586 2.103218 0.371897
H 0.229056 2.385178 0.480417
H 2.606855 2.696904 0.682820
H 1.771349 -2.705487 -1.467600
H 4.478486 0.456741 -2.372380
H 6.318752 -0.278927 0.549348
H 5.628399 -1.802904 0.025096
H 5.278206 -1.523421 3.868941
H 6.339100 -2.294173 2.665877
H 6.502168 -0.546085 3.029958
H 6.670632 1.892783 -0.809487
H 5.338480 2.955735 -1.396243
H 6.453271 3.511055 -0.135674
H -4.189053 2.738617 -1.034579
H -5.825528 2.560722 -0.461785
H -4.519178 2.755844 0.714962
H -5.318928 -1.157950 -0.010440
H -7.230437 1.242414 0.343179
H -7.419297 -1.641085 1.349437
H -6.886788 -0.266547 2.311310
H -9.670835 -0.628467 1.095361
H -9.218177 -0.707841 2.799874
H -9.125447 0.836982 1.938596
H -7.417623 0.210615 -1.950361
H -8.876558 0.008131 -0.982755
H -7.763210 -1.353961 -1.194168
SCF Energy (B3LYP/6-31G**)= -1361.70134866
Number of imaginary frequencies = 0

1a_c150
MMFF Geometry
C -1.520556 -2.090251 -0.339006
C -2.307089 0.407139 0.392802
N -2.825766 -1.900429 -0.027441
C -0.540320 -1.093608 -0.313253
C -0.958864 0.196109 0.069429
C -3.207140 -0.654707 0.327312
C -0.022213 1.245618 0.110563
C 1.322982 1.023154 -0.205263
C 1.734583 -0.263893 -0.569415
C 0.811298 -1.313964 -0.645521
C 3.165142 -0.468286 -0.892775
C 4.186291 0.522669 -0.275357
C 3.589394 1.926140 -0.288068
C 2.265987 2.141456 -0.248821
O 1.181644 -2.574648 -1.045415
O 3.484211 -1.426358 -1.597867
O 5.386820 0.519895 -1.052625
C 4.558008 0.090421 1.147889
C 4.728424 -1.407334 1.269610
C 6.010784 -2.017540 0.769891
O 3.822764 -2.098336 1.738374
O 4.421507 3.014956 -0.507834
C 5.371041 3.231353 0.532458
C -4.641837 -0.493022 0.663813
C -5.216637 -1.618020 1.481885
C -5.334123 0.577632 0.219225
C -6.804140 0.873643 0.424380
C -7.534550 1.059998 -0.919756

C -7.555247 -0.204211 -1.770306
C -6.947447 2.135199 1.279580
H -1.270947 -3.111068 -0.621006
H -2.649995 1.388420 0.705864
H -0.352421 2.246521 0.386064
H 1.869497 3.151653 -0.301374
H 2.122086 -2.554042 -1.332191
H 5.149004 0.859206 -1.935147
H 3.780340 0.379831 1.866824
H 5.490835 0.552672 1.489526
H 6.087336 -3.047725 1.129091
H 6.024554 -2.019513 -0.321694
H 6.863986 -1.455088 1.157174
H 4.905538 3.159727 1.521357
H 6.207514 2.532559 0.446942
H 5.769095 4.243884 0.417261
H -4.517037 -1.923764 2.268730
H -6.141347 -1.340988 1.993997
H -5.425987 -2.486417 0.848356
H -4.819873 1.317397 -0.394828
H -7.300916 0.057088 0.957379
H -8.572107 1.360468 -0.726990
H -7.074596 1.872419 -1.496529
H -8.005109 -1.038037 -1.222005
H -8.146515 -0.036877 -2.676328
H -6.547794 -0.497611 -2.080502
H -6.454269 2.009282 2.250025
H -8.003309 2.355952 1.470217
H -6.505394 3.008815 0.787030
SCF Energy (B3LYP/6-31G**)= -1361.69400220
Number of imaginary frequencies = 0

1a_c151
MMFF Geometry
C -1.607152 -2.365215 -0.165828
C -2.303894 0.258856 -0.046545
N -2.923020 -2.063879 -0.045384
C -0.573245 -1.425088 -0.235505
C -0.946536 -0.070503 -0.171807
C -3.256092 -0.756618 0.006899
C 0.047031 0.922475 -0.235237
C 1.404077 0.589466 -0.360354
C 1.773663 -0.758791 -0.416937
C 0.790660 -1.761471 -0.362066
C 3.208282 -1.109440 -0.555307
C 4.275114 0.001380 -0.518106
C 3.706056 1.402946 -0.527061
C 2.395947 1.661363 -0.426730
O 1.110003 -3.097791 -0.433611
O 3.526072 -2.289218 -0.748197
O 5.011617 -0.192716 -1.747187
C 5.252959 -0.237987 0.637429
C 4.640924 0.078075 1.982243
C 5.503312 0.817594 2.969159
O 3.503380 -0.289977 2.274264
O 4.596360 2.467907 -0.457438
C 5.256944 2.701634 -1.701493
C -4.703064 -0.467802 0.143687
C -5.389081 -1.271181 1.210955
C -5.283087 0.446872 -0.662932
C -6.726100 0.891301 -0.804342
C -6.966618 2.268341 -0.154184
C -6.807300 2.307888 1.360130
C -7.811498 -0.124173 -0.438726
H -1.394381 -3.431391 -0.205808
H -2.615759 1.296546 0.021387
H -0.245991 1.970617 -0.184234
H 2.050484 2.689457 -0.366697
H 2.083368 -3.180510 -0.546118
H 5.170653 -1.155136 -1.819719
H 6.150194 0.378404 0.498159
H 5.605117 -1.276330 0.663365
H 4.944389 0.985330 3.893921
H 6.392790 0.224174 3.194128
H 5.789819 1.786016 2.551590
H 6.126772 2.046065 -1.799471
H 4.580878 2.582258 -2.555436
H 5.618520 3.734321 -1.697317
H -6.141385 -0.679313 1.737627
H -5.860273 -2.161548 0.783093
H -4.685710 -1.599999 1.985250
H -4.643018 0.947835 -1.392660
H -6.856141 1.046260 -1.886487
H -6.275591 2.999944 -0.592883
H -7.977130 2.612499 -0.408166
H -5.794687 2.029720 1.666306
H -6.995261 3.322682 1.726277
H -7.517281 1.641724 1.857936
H -7.604328 -1.101388 -0.887966
H -8.781695 0.211262 -0.823393
H -7.930492 -0.257523 0.638305
SCF Energy (B3LYP/6-31G**)= -1361.69531298
Number of imaginary frequencies = 0

1a_c152
MMFF Geometry
C -1.754898 -1.351069 -0.499150
C -2.080144 1.241715 0.241065
N -3.012576 -0.907269 -0.259452
C -0.601397 -0.570515 -0.395129
C -0.781433 0.771257 -0.012734
C -3.171799 0.384069 0.111204
C 0.340567 1.614152 0.098333
C 1.634923 1.137200 -0.149575
C 1.804544 -0.201773 -0.512339
C 0.698252 -1.050006 -0.654220
C 3.174905 -0.679556 -0.777707
C 4.359017 0.112554 -0.190417
C 4.045598 1.601798 -0.106305
C 2.777398 2.054742 -0.079135
O 0.834322 -2.358432 -1.049990
O 3.347855 -1.696735 -1.455288
O 5.468657 -0.075055 -1.087914
C 4.775333 -0.393870 1.192649
C 4.715643 -1.898397 1.300633
C 5.919438 -2.684884 0.856225
O 3.692296 -2.454283 1.702017
O 5.173947 2.396711 -0.012596
C 5.017584 3.809911 -0.015552
C -4.569274 0.829769 0.382896
C -4.745826 1.984030 1.332469
C -5.600284 0.172346 -0.193052
C -7.077558 0.471925 -0.068632
C -7.775306 -0.483261 0.918894
C -7.759311 -1.956948 0.531105
C -7.734815 0.456378 -1.452532
H -1.697051 -2.398065 -0.789188
H -2.229346 2.278308 0.524437
H 0.195726 2.656522 0.380108
H 2.544995 3.110437 0.007058
H 1.777053 -2.530059 -1.268698
H 5.227714 -0.806901 -1.690516
H 4.113394 -0.000533 1.975749
H 5.792450 -0.073313 1.449584
H 5.801153 -3.730949 1.152675
H 6.019498 -2.632510 -0.229622
H 6.817422 -2.290189 1.337935
H 4.521524 4.147937 -0.931104
H 4.471998 4.143600 0.872811
H 6.014689 4.259082 0.013634
H -4.540407 2.933497 0.827331
H -5.754004 2.038062 1.752153
H -4.072888 1.890181 2.192558
H -5.371234 -0.675673 -0.841693
H -7.217762 1.488665 0.315866
H -7.310150 -0.379724 1.907703
H -8.819907 -0.168726 1.037430
H -6.738656 -2.340030 0.440791
H -8.267355 -2.548127 1.300273
H -8.281387 -2.129438 -0.414371
H -7.299343 1.230126 -2.095229
H -8.808802 0.656172 -1.373634

H -7.604869 -0.504520 -1.961697
SCF Energy (B3LYP/6-31G**)= -1361.70449196
Number of imaginary frequencies = 0

1a_c153
MMFF Geometry
C -1.670770 -1.390307 -0.615138
C -2.088174 1.116300 0.343325
N -2.945999 -1.004313 -0.367995
C -0.543080 -0.590942 -0.414737
C -0.771115 0.706165 0.080175
C -3.150934 0.243957 0.111786
C 0.322291 1.566377 0.292861
C 1.634331 1.149051 0.033738
C 1.853954 -0.148315 -0.441982
C 0.775710 -1.010290 -0.683395
C 3.244868 -0.565868 -0.719827
C 4.401624 0.214858 -0.064415
C 4.019469 1.668745 0.137899
C 2.745703 2.083040 0.220486
O 0.957407 -2.275079 -1.188210
O 3.459092 -1.524588 -1.467944
O 5.511146 0.151044 -0.977451
C 4.839902 -0.387514 1.272459
C 4.827196 -1.896937 1.261639
C 6.069651 -2.607040 0.795753
O 3.812840 -2.515187 1.586751
O 5.056356 2.546509 0.416420
C 5.416902 3.302122 -0.740485
C -4.566393 0.625614 0.388068
C -4.795872 1.678131 1.439012
C -5.563617 0.002255 -0.278055
C -7.053627 0.246001 -0.169884
C -7.713952 -0.945950 0.545810
C -9.198823 -0.744271 0.815341
C -7.628538 0.475306 -1.571390
H -1.575935 -2.405414 -0.994879
H -2.273904 2.119070 0.713702
H 0.142330 2.574871 0.663860
H 2.519799 3.120523 0.449060
H 1.910238 -2.404839 -1.390993
H 5.339115 -0.604312 -1.575857
H 4.170139 -0.079663 2.086598
H 5.847543 -0.055536 1.551247
H 5.967283 -3.680255 0.979690
H 6.214516 -2.442683 -0.273745
H 6.934887 -2.242274 1.354777
H 5.712462 2.653227 -1.570718
H 4.595778 3.958155 -1.048535
H 6.273047 3.930223 -0.477463
H -5.810989 1.660136 1.844202
H -4.133531 1.524484 2.298770
H -4.613922 2.677244 1.029727
H -5.296182 -0.773966 -0.998538
H -7.253626 1.155104 0.406278
H -7.580224 -1.864506 -0.039777
H -7.213501 -1.112709 1.508630
H -9.774539 -0.706309 -0.113900
H -9.587898 -1.576723 1.410595
H -9.375150 0.181231 1.372391
H -7.087653 1.274877 -2.090624
H -8.680089 0.775223 -1.523206
H -7.562973 -0.429521 -2.186112
SCF Energy (B3LYP/6-31G**)= -1361.70029229
Number of imaginary frequencies = 0

1a_c154
MMFF Geometry
C -1.626169 -2.265533 -0.595957
C -2.317738 0.114720 0.514662
N -2.927159 -2.071419 -0.269172
C -0.605083 -1.328280 -0.407595
C -0.975420 -0.100931 0.168699
C -3.261482 -0.882436 0.275802
C 0.006581 0.881184 0.386916
C 1.348339 0.663314 0.037743
C 1.717207 -0.560141 -0.531683
C 0.743883 -1.550139 -0.756101
C 3.135532 -0.790907 -0.910405
C 4.178464 0.328251 -0.669618
C 3.613422 1.586294 -0.041838
C 2.324945 1.718975 0.294893
O 1.057065 -2.762895 -1.325785
O 3.426089 -1.855419 -1.465563
O 4.683695 0.680341 -1.975515
C 5.365500 -0.250842 0.111765
C 5.020160 -0.631094 1.535198
C 6.174874 -0.759399 2.493369
O 3.868357 -0.874266 1.893424
O 4.497721 2.584393 0.350420
C 5.089395 3.274843 -0.748874
C -4.692718 -0.710908 0.627734
C -5.323989 -1.928274 1.249232
C -5.327869 0.448012 0.349155
C -6.773575 0.801611 0.623525
C -7.634410 0.727185 -0.652972
C -7.259053 1.704414 -1.760390
C -6.855612 2.173719 1.299719
H -1.415236 -3.239476 -1.032596
H -2.621395 1.047497 0.979780
H -0.283268 1.828927 0.839877
H 1.990479 2.618266 0.804832
H 2.025513 -2.784204 -1.501827
H 3.916522 0.891950 -2.535370
H 6.187499 0.475528 0.134798
H 5.763737 -1.144359 -0.384960
H 5.803129 -1.033992 3.484430
H 6.857160 -1.537081 2.141723
H 6.697715 0.197342 2.567550
H 5.935555 2.708197 -1.147415
H 4.357932 3.488504 -1.535916
H 5.474233 4.228583 -0.375586
H -4.729726 -2.273013 2.103235
H -6.334139 -1.750647 1.623254
H -5.391243 -2.744569 0.521696
H -4.770366 1.235952 -0.157855
H -7.205946 0.090918 1.335061
H -7.587238 -0.291841 -1.058269
H -8.683567 0.900590 -0.381864
H -6.233779 1.547600 -2.107792
H -7.923687 1.563977 -2.619343
H -7.361213 2.742681 -1.432079
H -6.338854 2.158951 2.266224
H -7.898379 2.452780 1.485038
H -6.395843 2.961799 0.693867
SCF Energy (B3LYP/6-31G**)= -1361.69993800
Number of imaginary frequencies = 0

1a_c155
MMFF Geometry
C -1.653055 -1.240520 -1.046251
C -2.100058 1.203867 0.049738
N -2.936710 -0.843060 -0.871564
C -0.530640 -0.483013 -0.703990
C -0.773851 0.781296 -0.136718
C -3.156654 0.374832 -0.324941
C 0.314564 1.596667 0.225609
C 1.635290 1.167015 0.041626
C 1.868521 -0.097782 -0.509364
C 0.797527 -0.913976 -0.897508
C 3.269326 -0.528141 -0.706018
C 4.383551 0.174709 0.094999
C 4.013423 1.619164 0.373110
C 2.745107 2.057563 0.384540
O 0.995945 -2.144020 -1.476427
O 3.525485 -1.435319 -1.503587
O 5.561977 0.149748 -0.729268
C 4.700680 -0.533099 1.414315
C 4.662415 -2.037060 1.290568
C 5.926294 -2.740549 0.874980
O 3.614112 -2.653259 1.486031
O 5.040486 2.448841 0.797330

C 5.507947 3.276705 -0.268252
C -4.582666 0.782066 -0.161659
C -4.873211 2.258628 -0.157361
C -5.535563 -0.170627 -0.055997
C -7.025678 0.012790 0.137314
C -7.483457 -0.798158 1.365611
C -8.945862 -0.576761 1.727602
C -7.752692 -0.408596 -1.141356
H -1.546673 -2.228982 -1.488137
H -2.296452 2.171969 0.498534
H 0.123949 2.579914 0.654535
H 2.520823 3.081041 0.670605
H 1.959420 -2.281935 -1.612588
H 5.425048 -0.556296 -1.393351
H 3.972277 -0.269389 2.192812
H 5.687926 -0.245997 1.796664
H 5.790426 -3.821561 0.970148
H 6.161255 -2.502702 -0.164272
H 6.749006 -2.437585 1.527324
H 5.857952 2.682221 -1.117833
H 4.726950 3.973120 -0.591627
H 6.351389 3.863093 0.107820
H -4.281137 2.776655 -0.920649
H -5.916175 2.488133 -0.390123
H -4.642776 2.693601 0.820685
H -5.224887 -1.216988 -0.100392
H -7.263133 1.063442 0.331157
H -7.317775 -1.870259 1.199134
H -6.871658 -0.517318 2.232886
H -9.614606 -0.962870 0.952982
H -9.184191 -1.099456 2.659632
H -9.158541 0.487026 1.872679
H -7.362167 0.132877 -2.010688
H -8.823105 -0.189130 -1.079169
H -7.637757 -1.481231 -1.334041
SCF Energy (B3LYP/6-31G**)= -1361.70033699
Number of imaginary frequencies = 0

1a_c156
MMFF Geometry
C 1.674474 2.539737 0.205400
C 2.390239 -0.014436 -0.387301
N 2.995720 2.242504 0.167683
C 0.643014 1.627389 -0.038147
C 1.025974 0.308591 -0.347343
C 3.340517 0.968430 -0.117991
C 0.035962 -0.653824 -0.616905
C -1.325321 -0.326456 -0.567182
C -1.701067 0.980831 -0.243551
C -0.726648 1.958597 0.004164
C -3.144439 1.306471 -0.178304
C -4.170797 0.160261 -0.070968
C -3.635938 -1.099662 -0.717817
C -2.328400 -1.335124 -0.901639
O -1.063796 3.258745 0.295624
O -3.496753 2.490488 -0.163778
O -5.334118 0.613984 -0.780566
C -4.514597 0.012919 1.414663
C -5.345821 -1.205660 1.750157
C -6.780329 -1.235118 1.289985
O -4.869097 -2.125547 2.418121
O -4.566255 -2.082149 -1.018465
C -4.886994 -2.088352 -2.409915
C 4.795793 0.688005 -0.150796
C 5.619722 1.760771 -0.810955
C 5.290895 -0.439381 0.403510
C 6.744415 -0.855571 0.498962
C 6.982268 -2.077833 -0.406047
C 8.442273 -2.503674 -0.478305
C 7.091139 -1.138821 1.963935
H 1.456626 3.577876 0.447254
H 2.709109 -1.021107 -0.639865
H 0.335523 -1.670974 -0.868544
H -1.994571 -2.286279 -1.306578
H -2.032068 3.372996 0.167615
H -5.384621 1.581569 -0.642614
H -5.065203 0.889912 1.778634
H -3.597958 -0.046896 2.016816
H -7.297424 -2.072606 1.767279
H -7.279953 -0.309220 1.585920
H -6.834829 -1.362609 0.208019
H -5.258154 -1.113504 -2.741575
H -4.019554 -2.385705 -3.008545
H -5.678573 -2.826550 -2.568256
H 5.769771 2.604930 -0.129734
H 6.601579 1.403158 -1.131443
H 5.124353 2.130264 -1.716581
H 4.601648 -1.139263 0.876925
H 7.400448 -0.044508 0.167459
H 6.379996 -2.929154 -0.063884
H 6.645285 -1.845067 -1.424659
H 8.800207 -2.877221 0.485398
H 8.561551 -3.309838 -1.209437
H 9.080257 -1.669753 -0.787333
H 6.837287 -0.282318 2.599084
H 8.162370 -1.326362 2.087632
H 6.549002 -2.012009 2.344082
SCF Energy (B3LYP/6-31G**)= -1361.70162412
Number of imaginary frequencies = 0

1a_c157
MMFF Geometry
C 1.479193 -2.145255 0.619665
C 2.344772 0.161217 -0.527770
N 2.795617 -2.041875 0.314798
C 0.524970 -1.149569 0.388768
C 0.984639 0.040157 -0.207419
C 3.216926 -0.889193 -0.247685
C 0.074791 1.082276 -0.464397
C -1.283277 0.952714 -0.146230
C -1.735651 -0.237991 0.433155
C -0.839843 -1.277938 0.716791
C -3.170560 -0.350484 0.771617
C -4.174590 0.594401 0.081527
C -3.522620 1.925487 -0.240711
C -2.196148 2.072385 -0.381575
O -1.248744 -2.442988 1.319356
O -3.541543 -1.191215 1.596400
O -5.242190 0.817274 1.019229
C -4.769101 -0.004134 -1.195380
C -5.053105 -1.481147 -1.067069
C -6.394221 -1.894419 -0.523359
O -4.191925 -2.310645 -1.362329
O -4.376865 2.968747 -0.564798
C -4.540485 3.862292 0.537081
C 4.663030 -0.817790 -0.572188
C 5.226658 -2.086466 -1.154967
C 5.366266 0.303481 -0.303253
C 6.837227 0.557013 -0.553249
C 7.665585 0.452377 0.742464
C 7.332528 1.473695 1.823309
C 7.022220 1.907146 -1.252956
H 1.197838 -3.093227 1.073576
H 2.718651 1.062291 -1.004198
H 0.436180 2.005494 -0.916243
H -1.779655 3.025357 -0.694702
H -2.200266 -2.365561 1.552790
H -5.200019 0.088150 1.671027
H -4.080706 0.104636 -2.044174
H -5.700907 0.500352 -1.479114
H -6.511361 -2.977027 -0.625063
H -6.467073 -1.627401 0.532680
H -7.189487 -1.406497 -1.092286
H -4.926985 3.346349 1.421468
H -3.596287 4.363451 0.775510
H -5.266187 4.626654 0.244192
H 4.629213 -2.410274 -2.014919
H 6.253856 -1.982554 -1.509810
H 5.225413 -2.889643 -0.409933
H 4.851208 1.136291 0.176191
H 7.236404 -0.194258 -1.242144
H 7.543972 -0.553190 1.165487

H 8.729044 0.552035 0.490629
H 6.292427 1.390573 2.151562
H 7.969177 1.307598 2.698723
H 7.508734 2.496428 1.478069
H 6.525033 1.906682 -2.229784
H 8.084485 2.114166 -1.421227
H 6.602890 2.735340 -0.671777
SCF Energy (B3LYP/6-31G**)= -1361.69886741
Number of imaginary frequencies = 0

1a_c158
MMFF Geometry
C -1.756159 -1.581004 -0.497309
C -2.011970 1.027019 0.210192
N -3.002819 -1.099980 -0.271007
C -0.581005 -0.831872 -0.397623
C -0.726154 0.517073 -0.033597
C -3.126762 0.199229 0.085046
C 0.418839 1.326188 0.075872
C 1.702713 0.813998 -0.167593
C 1.846106 -0.532845 -0.520166
C 0.708449 -1.350170 -0.640986
C 3.203444 -1.078791 -0.782950
C 4.438214 -0.167940 -0.585665
C 4.103429 1.268613 -0.251968
C 2.854503 1.705595 -0.042085
O 0.798476 -2.673829 -1.005829
O 3.297017 -2.237358 -1.200245
O 5.147865 -0.156873 -1.843303
C 5.393510 -0.812859 0.426412
C 4.863709 -0.811108 1.842764
C 5.888031 -0.893422 2.943513
O 3.659483 -0.798443 2.095828
O 5.187221 2.108143 -0.037568
C 5.386789 3.004426 -1.130692
C -4.511943 0.687661 0.345909
C -4.658023 1.847967 1.293149
C -5.557181 0.062236 -0.239790
C -7.028255 0.401703 -0.131109
C -7.738780 -0.675419 0.707915
C -9.203561 -0.362519 0.981180
C -7.620216 0.528413 -1.538532
H -1.725984 -2.632864 -0.773343
H -2.132119 2.070180 0.483065
H 0.303265 2.371682 0.360069
H 2.679603 2.733397 0.262180
H 1.747240 -2.895183 -1.147254
H 4.502523 0.055484 -2.538864
H 6.356178 -0.285498 0.413849
H 5.613077 -1.852587 0.153674
H 5.386786 -0.883050 3.915341
H 6.455608 -1.822041 2.846160
H 6.558389 -0.032312 2.885664
H 5.535627 2.461389 -2.069320
H 4.547534 3.701356 -1.225164
H 6.290238 3.586509 -0.926741
H -4.429437 2.790779 0.785509
H -5.663173 1.928094 1.715320
H -3.986136 1.739501 2.152389
H -5.349740 -0.794081 -0.885388
H -7.163796 1.372065 0.357221
H -7.670124 -1.652218 0.212243
H -7.227323 -0.777800 1.673944
H -9.796671 -0.380504 0.062335
H -9.625713 -1.109810 1.660905
H -9.314979 0.620926 1.448484
H -7.046720 1.241501 -2.141924
H -8.651847 0.892357 -1.502289
H -7.619430 -0.433567 -2.063402
SCF Energy (B3LYP/6-31G**)= -1361.70091992
Number of imaginary frequencies = 0

1a_c159
MMFF Geometry
C -1.882053 -1.443188 -0.471386
C -2.083352 1.161219 0.266361
N -3.118214 -0.939081 -0.238065
C -0.691604 -0.719692 -0.364550
C -0.808598 0.627502 0.015308
C -3.215245 0.358369 0.132802
C 0.353180 1.410767 0.134234
C 1.625813 0.875246 -0.118097
C 1.741239 -0.468650 -0.488715
C 0.586842 -1.261525 -0.615051
C 3.086297 -1.037092 -0.764181
C 4.335209 -0.130151 -0.638196
C 4.034194 1.296183 -0.224550
C 2.796306 1.736897 0.029611
O 0.649250 -2.583164 -0.992755
O 3.157880 -2.209400 -1.146743
O 4.923307 -0.088235 -1.956141
C 5.363253 -0.814617 0.272640
C 4.923578 -0.900107 1.718141
C 6.010218 -1.122849 2.736749
O 3.741245 -0.842298 2.054058
O 5.100294 2.141254 0.060681
C 5.840232 2.523301 -1.097674
C -4.589995 0.871857 0.400809
C -4.711908 2.033035 1.350619
C -5.650479 0.265996 -0.178178
C -7.111526 0.637708 -0.057320
C -7.857800 -0.282292 0.928132
C -7.913179 -1.754904 0.539881
C -7.765285 0.654738 -1.442860
H -1.873972 -2.492183 -0.759556
H -2.181067 2.203398 0.551667
H 0.260361 2.454592 0.432897
H 2.648177 2.751588 0.388850
H 1.594699 -2.827886 -1.117382
H 4.225801 0.187939 -2.575814
H 6.317652 -0.275040 0.230217
H 5.575246 -1.834294 -0.072375
H 5.573862 -1.164518 3.738575
H 6.516763 -2.068734 2.530172
H 6.723328 -0.295651 2.698879
H 6.556452 1.743116 -1.370163
H 5.181957 2.759952 -1.940760
H 6.408840 3.424126 -0.848141
H -4.458095 2.971387 0.846709
H -5.717454 2.136864 1.767236
H -4.047126 1.905461 2.212708
H -5.461414 -0.592012 -0.826495
H -7.202624 1.660023 0.327166
H -7.390614 -0.201926 1.918138
H -8.886000 0.083109 1.044159
H -6.912331 -2.187596 0.451977
H -8.451549 -2.320605 1.307580
H -8.440722 -1.901356 -0.406951
H -7.290760 1.406326 -2.084206
H -8.828368 0.906978 -1.366571
H -7.681372 -0.311267 -1.952029
SCF Energy (B3LYP/6-31G**)= -1361.70045185
Number of imaginary frequencies = 0

1a_c160
MMFF Geometry
C 1.706506 2.413844 -0.119512
C 2.344663 -0.184040 0.361485
N 3.001426 2.116046 0.144932
C 0.665166 1.481492 -0.163482
C 1.007551 0.139524 0.088290
C 3.309337 0.821363 0.374575
C 0.005563 -0.847435 0.056664
C -1.329278 -0.516478 -0.210662
C -1.666409 0.819593 -0.447621
C -0.676755 1.813299 -0.440232
C -3.084476 1.155347 -0.712126
C -4.181688 0.137944 -0.338338
C -3.642390 -1.275591 -0.392244
C -2.338161 -1.570759 -0.288598
O -0.972578 3.131102 -0.694978
O -3.365564 2.262072 -1.183728

O -5.213659 0.301659 -1.323715
C -4.724368 0.557155 1.031615
C -5.664192 -0.442378 1.668888
C -7.025148 -0.631547 1.050456
O -5.335424 -1.049691 2.690020
O -4.581966 -2.293542 -0.438474
C -4.719722 -2.816500 -1.760054
C 4.736109 0.541174 0.663842
C 5.393480 1.547334 1.569969
C 5.351562 -0.526286 0.111437
C 6.803381 -0.932596 0.260675
C 7.451651 -1.027379 -1.134221
C 8.948455 -1.303375 -1.089534
C 6.875495 -2.255348 1.026746
H 1.519021 3.469893 -0.301981
H 2.628751 -1.209759 0.576104
H 0.275000 -1.886977 0.242171
H -2.011201 -2.606389 -0.262531
H -1.907171 3.195555 -0.993947
H -5.225003 1.251344 -1.559881
H -5.263887 1.510675 0.964636
H -3.899217 0.719172 1.738206
H -7.650216 -1.225530 1.723553
H -7.502131 0.341404 0.907825
H -6.945030 -1.154559 0.096563
H -4.987200 -2.033568 -2.476683
H -3.800985 -3.320944 -2.077368
H -5.525947 -3.555690 -1.745800
H 4.725295 1.823767 2.394111
H 6.304124 1.165403 2.038058
H 5.651216 2.455111 1.014316
H 4.780895 -1.175643 -0.553137
H 7.358193 -0.182731 0.832889
H 6.964286 -1.809888 -1.729790
H 7.295525 -0.082225 -1.670526
H 9.162121 -2.296665 -0.684489
H 9.367171 -1.262250 -2.100312
H 9.467667 -0.558846 -0.477960
H 6.347305 -2.182642 1.984445
H 7.911719 -2.527902 1.249884
H 6.425276 -3.075379 0.456003
SCF Energy (B3LYP/6-31G**)= -1361.70159283
Number of imaginary frequencies = 0

1a_c161
MMFF Geometry
C -1.678758 -2.141487 -0.480098
C -2.233240 0.517769 -0.457327
N -2.980636 -1.764893 -0.464499
C -0.591067 -1.261711 -0.488235
C -0.890804 0.111216 -0.479692
C -3.244069 -0.441065 -0.442409
C 0.160294 1.045048 -0.496797
C 1.503850 0.636519 -0.517763
C 1.800022 -0.729554 -0.518325
C 0.757608 -1.674305 -0.508012
C 3.217714 -1.166183 -0.537546
C 4.338161 -0.102262 -0.526034
C 3.857874 1.340036 -0.546968
C 2.552215 1.658944 -0.543886
O 1.002326 -3.028413 -0.523883
O 3.452109 -2.378252 -0.585364
O 5.100068 -0.313126 -1.735552
C 5.300557 -0.404167 0.631270
C 4.689549 -0.214752 2.002165
C 5.640881 -0.274967 3.168674
O 3.483014 -0.050071 2.177746
O 4.896483 2.254729 -0.576114
C 4.586835 3.643365 -0.567147
C -4.680094 -0.070542 -0.426155
C -5.526993 -0.874077 -1.373282
C -5.121680 0.893562 0.410467
C -6.527048 1.407561 0.647306
C -7.581585 0.344602 1.011607
C -7.201981 -0.491382 2.227436
C -7.001101 2.292815 -0.508217
H -1.524513 -3.218481 -0.487206
H -2.488533 1.572851 -0.464521
H -0.078859 2.107997 -0.489700
H 2.216118 2.689793 -0.553711
H 1.977228 -3.167985 -0.534997
H 4.594947 0.077263 -2.468406
H 6.188182 0.237123 0.558606
H 5.664102 -1.437793 0.569221
H 5.090970 -0.122549 4.101515
H 6.122931 -1.255175 3.197523
H 6.391706 0.512915 3.070437
H 4.018976 3.922898 -1.460277
H 4.046957 3.917772 0.344805
H 5.529717 4.197995 -0.580286
H -5.733306 -1.866607 -0.958955
H -6.481690 -0.395667 -1.597353
H -5.020407 -1.000263 -2.337387
H -4.393539 1.374290 1.065804
H -6.453773 2.070101 1.522638
H -8.529496 0.852431 1.231891
H -7.786850 -0.322063 0.168654
H -6.998870 0.145787 3.094023
H -8.023703 -1.165630 2.489669
H -6.317840 -1.106058 2.034212
H -6.266138 3.074174 -0.732083
H -7.942389 2.789971 -0.249032
H -7.172321 1.720339 -1.424442
SCF Energy (B3LYP/6-31G**)= -1361.70204297
Number of imaginary frequencies = 0

1a_c162
MMFF Geometry
C 1.993491 1.671854 -0.507698
C 2.136289 -1.040373 -0.535989
N 3.219158 1.095479 -0.479100
C 0.785514 0.972261 -0.549679
C 0.870917 -0.431800 -0.564209
C 3.288202 -0.255841 -0.492875
C -0.310880 -1.194885 -0.613243
C -1.570632 -0.581460 -0.631960
C -1.647333 0.813476 -0.602036
C -0.480607 1.590389 -0.578940
C -2.987171 1.443230 -0.598680
C -4.187079 0.585970 -0.134207
C -4.001743 -0.867270 -0.557205
C -2.780853 -1.398074 -0.754378
O -0.528209 2.963770 -0.579984
O -3.070612 2.632294 -0.911773
O -5.373392 1.118275 -0.734391
C -4.305231 0.775390 1.382570
C -5.269474 -0.190589 2.033120
C -6.744504 0.077161 1.886290
O -4.855403 -1.172731 2.653412
O -5.194944 -1.552284 -0.695896
C -5.152612 -2.968866 -0.828583
C 4.656948 -0.851413 -0.475536
C 4.808545 -2.218273 -1.086889
C 5.677262 -0.143211 0.058324
C 7.130468 -0.540085 0.196032
C 7.549012 -0.683666 1.674179
C 7.512466 0.593979 2.503958
C 8.014450 0.450845 -0.565157
H 2.010056 2.759588 -0.494878
H 2.210648 -2.122846 -0.533961
H -0.241548 -2.282035 -0.638215
H -2.639934 -2.436169 -1.035119
H -1.456460 3.247504 -0.746668
H -5.366679 0.845150 -1.668472
H -4.636148 1.792501 1.628150
H -3.333468 0.642341 1.876070
H -7.297351 -0.531904 2.607250
H -6.953075 1.129065 2.096912
H -7.076335 -0.178508 0.878472
H -4.689178 -3.256845 -1.777366
H -4.632406 -3.427368 0.018703
H -6.182898 -3.336631 -0.831831

H 4.242341 -2.292513 -2.022573
H 5.840815 -2.460696 -1.348916
H 4.449594 -2.989713 -0.397600
H 5.454232 0.849163 0.455240
H 7.300501 -1.521184 -0.257776
H 6.906907 -1.431191 2.157762
H 8.569313 -1.086370 1.711913
H 6.505072 1.016688 2.553564
H 7.829348 0.377955 3.529773
H 8.190596 1.353137 2.103946
H 7.784880 0.428102 -1.636670
H 9.073202 0.196559 -0.447244
H 7.871684 1.480785 -0.220956
SCF Energy (B3LYP/6-31G**)= -1361.70235914
Number of imaginary frequencies = 0

1a_c163
MMFF Geometry
C -1.738386 -1.420862 -1.026928
C -2.024363 1.092271 -0.042178
N -2.994311 -0.944837 -0.845786
C -0.567665 -0.711668 -0.746914
C -0.728177 0.587833 -0.238303
C -3.133885 0.307446 -0.353311
C 0.412401 1.353620 0.062310
C 1.706526 0.847267 -0.134787
C 1.864288 -0.450646 -0.634163
C 0.731916 -1.224097 -0.944570
C 3.232837 -0.989558 -0.848963
C 4.456625 -0.134682 -0.443024
C 4.110695 1.258371 0.033290
C 2.853412 1.693138 0.191506
O 0.837749 -2.497070 -1.456062
O 3.348395 -2.091691 -1.394050
O 5.268983 0.011778 -1.627930
C 5.316998 -0.915560 0.558384
C 4.670661 -1.071727 1.916523
C 5.598448 -1.303709 3.079719
O 3.449644 -1.066133 2.069615
O 5.181678 2.043390 0.435956
C 5.483118 3.058609 -0.521388
C -4.531173 0.800967 -0.178418
C -4.738959 2.290548 -0.229926
C -5.533070 -0.091116 -0.013260
C -7.005847 0.183831 0.202862
C -7.479648 -0.549180 1.473351
C -8.918817 -0.231032 1.855791
C -7.784273 -0.247741 -1.041694
H -1.696674 -2.433864 -1.421389
H -2.155260 2.089147 0.365455
H 0.284827 2.359927 0.460122
H 2.664876 2.680549 0.602630
H 1.792551 -2.719482 -1.545681
H 4.686023 0.317255 -2.343612
H 6.283039 -0.410662 0.688150
H 5.547273 -1.919671 0.181075
H 5.017739 -1.399114 4.001390
H 6.163570 -2.224929 2.918642
H 6.279229 -0.454834 3.180648
H 5.705766 2.627719 -1.502619
H 4.662329 3.778959 -0.601343
H 6.371937 3.593689 -0.174506
H -4.138625 2.742460 -1.027977
H -5.773070 2.569208 -0.447983
H -4.459920 2.751489 0.723257
H -5.282120 -1.154283 -0.019653
H -7.180178 1.253215 0.357215
H -7.377608 -1.634822 1.348718
H -6.833467 -0.268132 2.315252
H -9.625560 -0.610094 1.112043
H -9.164352 -0.700965 2.813687
H -9.068726 0.848094 1.960369
H -7.384354 0.234903 -1.940894
H -8.839150 0.034095 -0.966729
H -7.733488 -1.332123 -1.191435
SCF Energy (B3LYP/6-31G**)= -1361.70104147
Number of imaginary frequencies = 0

1a_c164
MMFF Geometry
C -1.590312 -2.392960 -0.156855
C -2.301172 0.224896 -0.011532
N -2.905061 -2.099972 -0.006570
C -0.564234 -1.446117 -0.245548
C -0.944436 -0.095450 -0.167921
C -3.247295 -0.795740 0.057200
C 0.042522 0.902868 -0.247848
C 1.400481 0.580567 -0.404570
C 1.777053 -0.763581 -0.477725
C 0.799124 -1.772151 -0.402055
C 3.210480 -1.109284 -0.641001
C 4.260934 0.022470 -0.697287
C 3.694893 1.431631 -0.624993
C 2.378427 1.667367 -0.491577
O 1.122812 -3.107311 -0.481532
O 3.511989 -2.302412 -0.749980
O 4.916679 -0.105932 -1.978436
C 5.344589 -0.250335 0.355336
C 4.855141 -0.137952 1.782344
C 5.914154 -0.168163 2.853308
O 3.662667 -0.056574 2.074007
O 4.669324 2.410466 -0.717697
C 4.279022 3.775961 -0.631892
C -4.693639 -0.516360 0.230584
C -5.365098 -1.398502 1.246078
C -5.290531 0.444555 -0.507380
C -6.738882 0.887597 -0.540578
C -7.185221 1.673866 0.706140
C -6.346531 2.919055 0.967502
C -7.710382 -0.251988 -0.862661
H -1.371831 -3.457703 -0.205374
H -2.615953 1.260618 0.070454
H -0.258537 1.948017 -0.183540
H 1.981512 2.674839 -0.435291
H 2.098839 -3.184704 -0.588377
H 4.321795 0.271468 -2.647988
H 6.181244 0.447366 0.222545
H 5.762185 -1.256867 0.225347
H 5.446325 -0.076900 3.837499
H 6.456410 -1.115604 2.805108
H 6.602247 0.668949 2.712161
H 3.614287 4.045094 -1.458920
H 3.811384 3.989037 0.334719
H 5.181506 4.389083 -0.712005
H -4.746304 -1.495235 2.145921
H -6.325466 -1.005200 1.582227
H -5.536291 -2.399135 0.835134
H -4.679180 0.987152 -1.230178
H -6.813912 1.581359 -1.391197
H -8.228739 1.986751 0.573895
H -7.165102 1.039892 1.598729
H -6.345070 3.581349 0.095976
H -6.757983 3.476890 1.814907
H -5.310751 2.662977 1.210286
H -7.369136 -0.828043 -1.729872
H -8.700210 0.151666 -1.104081
H -7.840129 -0.942726 -0.025408
SCF Energy (B3LYP/6-31G**)= -1361.70215924
Number of imaginary frequencies = 0

1a_c165
MMFF Geometry
C -1.766056 -1.288192 -0.577770
C -2.115529 1.231992 0.372193
N -3.029499 -0.870772 -0.321480
C -0.618027 -0.514989 -0.390961
C -0.810898 0.789411 0.099676
C -3.200767 0.384257 0.153969
C 0.304461 1.624304 0.298863
C 1.603916 1.174699 0.030467
C 1.788509 -0.129678 -0.440934
C 0.688031 -0.966838 -0.668947

C 3.166638 -0.581476 -0.728733
C 4.347278 0.174485 -0.087023
C 4.001543 1.637960 0.111645
C 2.738745 2.082819 0.203206
O 0.835219 -2.237891 -1.169249
O 3.351501 -1.548427 -1.474083
O 5.446972 0.080131 -1.009257
C 4.782758 -0.432046 1.248884
C 4.734119 -1.940774 1.245361
C 5.955256 -2.682345 0.772194
O 3.708238 -2.533233 1.582124
O 5.061373 2.492064 0.377060
C 5.429689 3.233597 -0.786488
C -4.604412 0.800276 0.440703
C -4.800730 1.871041 1.480039
C -5.623527 0.188913 -0.203165
C -7.103961 0.471397 -0.076397
C -7.811079 -0.565016 0.818183
C -7.781877 -2.001449 0.310054
C -7.740962 0.567951 -1.466477
H -1.698723 -2.307013 -0.953404
H -2.273815 2.240517 0.739609
H 0.151760 2.638477 0.666589
H 2.539551 3.126414 0.428772
H 1.782894 -2.391218 -1.379602
H 5.251845 -0.673645 -1.602549
H 4.127612 -0.104644 2.067278
H 5.800389 -0.122864 1.517412
H 5.829038 -3.751972 0.962115
H 6.094660 -2.526356 -0.299280
H 6.833746 -2.335719 1.322013
H 5.702524 2.574072 -1.616124
H 4.621730 3.907566 -1.090603
H 6.302766 3.842346 -0.533825
H -4.592341 2.859921 1.058682
H -5.815331 1.886467 1.887245
H -4.140341 1.708418 2.339601
H -5.380683 -0.601406 -0.916403
H -7.254954 1.452249 0.388774
H -7.360886 -0.541906 1.818995
H -8.858895 -0.265471 0.946748
H -6.758060 -2.371730 0.203568
H -8.297993 -2.656287 1.020005
H -8.289269 -2.097088 -0.654128
H -7.300168 1.393960 -2.036383
H -8.816990 0.756335 -1.387326
H -7.598718 -0.346964 -2.051420
SCF Energy (B3LYP/6-31G**)= -1361.69947697
Number of imaginary frequencies = 0

1a_c166
MMFF Geometry
C -1.686329 -1.395252 -0.583118
C -2.107471 1.130797 0.320795
N -2.962304 -1.005398 -0.346066
C -0.559649 -0.590664 -0.398267
C -0.789666 0.716286 0.068368
C -3.169112 0.252723 0.106491
C 0.302855 1.581343 0.264650
C 1.615897 1.160044 0.016038
C 1.837729 -0.145881 -0.432050
C 0.760149 -1.013977 -0.655965
C 3.228503 -0.566769 -0.702884
C 4.390826 0.237606 -0.084560
C 4.001540 1.692264 0.111565
C 2.725437 2.096805 0.193050
O 0.943269 -2.289431 -1.133036
O 3.440969 -1.547594 -1.423145
O 5.470889 0.158066 -1.031342
C 4.870621 -0.342725 1.248299
C 4.848199 -1.852004 1.271933
C 6.073716 -2.580257 0.789489
O 3.839885 -2.456141 1.639306
O 5.004997 2.599347 0.423748
C 5.712611 3.042946 -0.733972
C -4.585349 0.638871 0.372492
C -4.817242 1.714395 1.399351
C -5.581108 -0.000272 -0.280751
C -7.071500 0.244260 -0.179904
C -7.731494 -0.932072 0.561479
C -9.216912 -0.725989 0.824589
C -7.644886 0.441521 -1.586902
H -1.589965 -2.418118 -0.940985
H -2.294452 2.141141 0.669331
H 0.121731 2.597036 0.615006
H 2.497358 3.134296 0.421040
H 1.896544 -2.422596 -1.331382
H 5.295507 -0.624584 -1.593363
H 4.231047 -0.013703 2.078357
H 5.889022 -0.011419 1.485831
H 5.963784 -3.650370 0.986620
H 6.199363 -2.428816 -0.284337
H 6.953356 -2.218460 1.327568
H 6.552561 2.375678 -0.944918
H 5.061660 3.132222 -1.610983
H 6.123007 4.033199 -0.514243
H -5.832957 1.704660 1.803315
H -4.156108 1.580482 2.263327
H -4.635437 2.704275 0.968153
H -5.311967 -0.792060 -0.983430
H -7.273174 1.165819 0.375506
H -7.596077 -1.863365 -0.003230
H -7.232086 -1.076703 1.528410
H -9.791494 -0.709452 -0.105980
H -9.605875 -1.545319 1.437849
H -9.394899 0.211565 1.360560
H -7.104173 1.229839 -2.123240
H -8.696805 0.741329 -1.546741
H -7.577617 -0.476772 -2.181130
SCF Energy (B3LYP/6-31G**)= -1361.69956450
Number of imaginary frequencies = 0

1a_c167
MMFF Geometry
C 1.756329 2.571369 0.205197
C 2.471406 0.015482 -0.380817
N 3.077505 2.273652 0.168617
C 0.724639 1.658710 -0.036298
C 1.107263 0.339017 -0.342051
C 3.421922 0.998747 -0.113615
C 0.117074 -0.623801 -0.609466
C -1.244120 -0.295910 -0.560948
C -1.619572 1.012305 -0.240724
C -0.644936 1.990418 0.004790
C -3.062867 1.338530 -0.176707
C -4.089591 0.192895 -0.066770
C -3.554919 -1.068817 -0.710277
C -2.247399 -1.305128 -0.893133
O -0.981786 3.291397 0.292861
O -3.414838 2.522684 -0.165269
O -5.252573 0.645166 -0.777851
C -4.433865 0.049410 1.419129
C -5.265561 -1.168062 1.757459
C -6.699943 -1.198245 1.296941
O -4.789316 -2.086402 2.427887
O -4.485440 -2.051790 -1.008700
C -4.805788 -2.061424 -2.400221
C 4.876884 0.717686 -0.145480
C 5.702978 1.788412 -0.806359
C 5.372260 -0.411269 0.405396
C 6.826600 -0.820875 0.497099
C 7.183615 -1.897320 -0.546183
C 6.444928 -3.222619 -0.403417
C 7.158390 -1.264729 1.925323
H 1.538720 3.610191 0.444323
H 2.789959 -0.991931 -0.630915
H 0.416420 -1.641670 -0.858445
H -1.913734 -2.257402 -1.295568
H -1.949991 3.405602 0.164300
H -5.302836 1.613110 -0.642352
H -4.984305 0.927488 1.780725
H -3.517416 -0.009165 2.021694

H -7.217439 -2.034354 1.776213
H -7.199365 -0.271441 1.590371
H -6.754164 -1.328472 0.215288
H -5.176568 -1.087309 -2.734454
H -3.938266 -2.360545 -2.997850
H -5.597538 -2.799788 -2.556917
H 5.849796 2.635473 -0.128071
H 6.686663 1.428750 -1.119958
H 5.211652 2.153518 -1.715904
H 4.682285 -1.110535 0.877717
H 7.469273 0.043691 0.295792
H 6.992094 -1.499972 -1.551329
H 8.261698 -2.095672 -0.495493
H 5.362230 -3.093429 -0.491495
H 6.761074 -3.910240 -1.194902
H 6.661980 -3.702512 0.555135
H 7.009849 -0.438731 2.630321
H 8.204451 -1.580633 1.999728
H 6.528197 -2.096726 2.257114
SCF Energy (B3LYP/6-31G**)= -1361.70058601
Number of imaginary frequencies = 0

1a_c168
MMFF Geometry
C -1.668729 -1.253317 -1.019015
C -2.119812 1.211232 0.028820
N -2.953080 -0.854335 -0.853100
C -0.547552 -0.487791 -0.690645
C -0.792926 0.786761 -0.148352
C -3.175041 0.373735 -0.330527
C 0.294306 1.609862 0.199353
C 1.616014 1.178711 0.024442
C 1.851722 -0.095103 -0.502090
C 0.781571 -0.920262 -0.874613
C 3.252652 -0.525830 -0.693251
C 4.374558 0.204220 0.073999
C 3.994651 1.648663 0.348693
C 2.722944 2.074531 0.359291
O 0.981547 -2.161411 -1.429105
O 3.506198 -1.455026 -1.466717
O 5.525617 0.170999 -0.788124
C 4.736140 -0.484217 1.392639
C 4.688557 -1.990228 1.299398
C 5.937815 -2.706640 0.862232
O 3.644554 -2.598304 1.538017
O 4.984058 2.507517 0.807692
C 5.794838 3.015188 -0.251857
C -4.601712 0.782270 -0.176406
C -4.893981 2.258310 -0.200647
C -5.553590 -0.169364 -0.053409
C -7.044096 0.015958 0.134975
C -7.502100 -0.771904 1.378124
C -8.965106 -0.545356 1.734459
C -7.769403 -0.430654 -1.136080
H -1.560657 -2.249993 -1.441586
H -2.317634 2.187673 0.458532
H 0.102263 2.600574 0.610202
H 2.494299 3.096994 0.646840
H 1.945319 -2.300686 -1.561585
H 5.383170 -0.563894 -1.419654
H 4.036828 -0.204375 2.191984
H 5.737066 -0.193585 1.734702
H 5.795805 -3.785860 0.968082
H 6.152450 -2.478344 -0.183538
H 6.776385 -2.404908 1.494649
H 6.639730 2.346594 -0.437405
H 5.223013 3.178394 -1.172314
H 6.199628 3.979099 0.071139
H -4.301928 2.762301 -0.973291
H -5.937031 2.482084 -0.438545
H -4.664817 2.712232 0.769052
H -5.241629 -1.216000 -0.077467
H -7.282963 1.069840 0.308479
H -7.335014 -1.846794 1.232326
H -6.891455 -0.473815 2.240441
H -9.632663 -0.946990 0.966740
H -9.203714 -1.050421 2.676088
H -9.179161 0.520758 1.858975
H -7.378683 0.094565 -2.015242
H -8.840130 -0.211306 -1.079093
H -7.653034 -1.506639 -1.308118
SCF Energy (B3LYP/6-31G**)= -1361.69960438
Number of imaginary frequencies = 0

1a_c169
MMFF Geometry
C -1.843707 -1.507302 -0.318501
C -2.276974 1.172147 -0.223580
N -3.119236 -1.061972 -0.422064
C -0.723515 -0.687450 -0.164393
C -0.959555 0.698281 -0.113823
C -3.331999 0.273516 -0.377055
C 0.126621 1.577523 0.054380
C 1.439998 1.098316 0.154028
C 1.667670 -0.277358 0.084536
C 0.595878 -1.170618 -0.053929
C 3.060889 -0.758767 0.192612
C 4.197742 0.223156 -0.181751
C 3.827969 1.642770 0.259024
C 2.544680 2.030211 0.392596
O 0.788298 -2.531261 -0.083167
O 3.242655 -1.929426 0.534494
O 4.327974 0.284147 -1.605569
C 5.514585 -0.243470 0.472419
C 5.981927 -1.582223 -0.064448
C 6.723865 -2.480638 0.887334
O 5.810736 -1.893266 -1.245404
O 4.922506 2.469102 0.452181
C 4.699255 3.856692 0.672955
C -4.747879 0.725617 -0.513474
C -4.966803 2.100680 -1.084586
C -5.744987 -0.110229 -0.146643
C -7.237927 0.130048 -0.184162
C -7.853498 0.142216 1.230657
C -7.787035 -1.173036 1.997430
C -7.907009 -0.896932 -1.100825
H -1.742094 -2.589414 -0.365684
H -2.468932 2.238844 -0.174456
H -0.059359 2.649553 0.107027
H 2.271524 3.040318 0.675059
H 1.731061 -2.718653 0.126085
H 4.740827 -0.559991 -1.891666
H 5.390100 -0.321277 1.559169
H 6.337543 0.451821 0.267895
H 6.991325 -3.412457 0.381532
H 7.636567 -1.982736 1.223350
H 6.085166 -2.716738 1.742032
H 4.155908 4.305435 -0.164903
H 4.171637 4.021144 1.617793
H 5.674803 4.346818 0.743347
H -4.293646 2.284419 -1.929993
H -5.975879 2.247969 -1.476030
H -4.789194 2.866405 -0.322241
H -5.467860 -1.095242 0.234147
H -7.452876 1.114884 -0.610094
H -7.361095 0.919397 1.829385
H -8.907319 0.438588 1.151675
H -6.754942 -1.495896 2.160273
H -8.252708 -1.051565 2.981184
H -8.324402 -1.970338 1.476143
H -7.547399 -0.786916 -2.130358
H -8.993283 -0.757659 -1.112193
H -7.699314 -1.926176 -0.789123
SCF Energy (B3LYP/6-31G**)= -1361.71201284
Number of imaginary frequencies = 0

1a_c170
MMFF Geometry
C -1.696840 -2.156386 -0.150876
C -2.188664 0.467927 -0.657816
N -2.989967 -1.758854 -0.231840
C -0.588947 -1.316458 -0.310957

C -0.856392 0.038705 -0.574939
C -3.219029 -0.452223 -0.479056
C 0.215657 0.931457 -0.752488
C 1.548812 0.500303 -0.666157
C 1.810607 -0.846137 -0.395597
C 0.749482 -1.752253 -0.226131
C 3.216793 -1.301752 -0.308354
C 4.355664 -0.271910 -0.392712
C 3.921757 1.137756 -0.744742
C 2.625500 1.473964 -0.869195
O 0.966430 -3.088247 0.021052
O 3.453993 -2.511423 -0.207681
O 5.176195 -0.748856 -1.486061
C 5.219902 -0.336697 0.873094
C 4.545722 0.280015 2.076809
C 5.443183 0.923870 3.099687
O 3.328703 0.215164 2.247238
O 4.985998 2.012469 -0.890251
C 4.722304 3.331776 -1.351660
C -4.639790 -0.043833 -0.552920
C -5.488370 -0.867837 -1.477446
C -5.075364 0.955667 0.244161
C -6.442027 1.592750 0.405828
C -7.099082 1.182597 1.740124
C -7.456874 -0.293632 1.857963
C -7.415736 1.481442 -0.769040
H -1.566923 -3.216666 0.055446
H -2.424370 1.506752 -0.867788
H 0.001761 1.979606 -0.958616
H 2.318700 2.485900 -1.107864
H 1.936178 -3.252187 0.018193
H 5.294924 -1.707565 -1.343229
H 6.171016 0.182006 0.697104
H 5.482855 -1.370335 1.128897
H 4.839782 1.329705 3.916371
H 6.132007 0.177788 3.503188
H 6.000090 1.741565 2.635426
H 4.234284 3.315410 -2.331549
H 4.123421 3.887026 -0.622812
H 5.680955 3.847436 -1.460951
H -4.949999 -1.725195 -1.896202
H -6.357642 -1.274437 -0.951867
H -5.823171 -0.266964 -2.328653
H -4.345281 1.404578 0.922349
H -6.234289 2.670099 0.491200
H -6.427855 1.442795 2.568947
H -8.010275 1.775812 1.888249
H -6.571986 -0.931025 1.772713
H -7.910906 -0.488237 2.835281
H -8.178352 -0.595819 1.093800
H -6.942483 1.805115 -1.702232
H -8.281908 2.132351 -0.603037
H -7.800916 0.468942 -0.909340
SCF Energy (B3LYP/6-31G**)= -1361.70080069
Number of imaginary frequencies = 0

1a_c171
MMFF Geometry
C -1.854366 -1.452193 -0.508996
C -2.035576 1.143314 0.264452
N -3.086030 -0.944096 -0.260939
C -0.659028 -0.737093 -0.400356
C -0.765584 0.605751 -0.002097
C -3.172963 0.348758 0.127862
C 0.401326 1.381342 0.118127
C 1.669298 0.841610 -0.148126
C 1.774208 -0.499379 -0.534895
C 0.614347 -1.283099 -0.666703
C 3.114570 -1.074311 -0.821879
C 4.374557 -0.201583 -0.612107
C 4.080579 1.234585 -0.240464
C 2.845299 1.699069 -0.009904
O 0.666426 -2.599213 -1.064718
O 3.174211 -2.224230 -1.268308
O 5.074998 -0.178047 -1.874726
C 5.319759 -0.896450 0.376108
C 4.800602 -0.915882 1.796264
C 5.830421 -1.052582 2.886422
O 3.599032 -0.877663 2.059186
O 5.187870 2.039603 -0.014112
C 5.403238 2.957127 -1.086417
C -4.542837 0.866546 0.411881
C -4.651816 2.014083 1.379638
C -5.610477 0.275754 -0.169500
C -7.068468 0.654356 -0.034067
C -7.814355 -0.275938 0.941972
C -7.881095 -1.742148 0.531895
C -7.730487 0.696261 -1.415145
H -1.854273 -2.497320 -0.811018
H -2.125579 2.182127 0.564120
H 0.315719 2.422146 0.428903
H 2.700053 2.723259 0.320974
H 1.607897 -2.841987 -1.219133
H 4.430437 0.068448 -2.559670
H 6.295999 -0.394555 0.368842
H 5.509575 -1.934521 0.076093
H 5.336778 -1.053108 3.862188
H 6.372331 -1.993182 2.761678
H 6.523037 -0.208332 2.844452
H 5.530745 2.433784 -2.039265
H 4.582147 3.678101 -1.157062
H 6.323307 3.509917 -0.875391
H -4.395991 2.958404 0.888049
H -5.653985 2.117283 1.804477
H -3.982057 1.869968 2.235245
H -5.430558 -0.573657 -0.831609
H -7.150986 1.671266 0.366366
H -7.340684 -0.213369 1.930179
H -8.839578 0.093851 1.069833
H -6.883464 -2.179486 0.431321
H -8.418254 -2.316141 1.294268
H -8.415250 -1.871083 -0.413776
H -7.255289 1.454606 -2.047981
H -8.791534 0.953685 -1.328527
H -7.655538 -0.262418 -1.939338
SCF Energy (B3LYP/6-31G**)= -1361.70014360
Number of imaginary frequencies = 0

1a_c172
MMFF Geometry
C 1.713841 2.127272 0.023766
C 2.217200 -0.446427 -0.689890
N 3.008468 1.748161 -0.106721
C 0.609703 1.292926 -0.184043
C 0.883326 -0.036039 -0.555184
C 3.243350 0.466085 -0.456294
C -0.184049 -0.921980 -0.785141
C -1.517515 -0.508407 -0.647747
C -1.787369 0.811349 -0.269468
C -0.730522 1.710224 -0.046091
C -3.197841 1.248827 -0.130007
C -4.336792 0.223548 -0.288144
C -3.879717 -1.136232 -0.768053
C -2.589611 -1.469119 -0.902385
O -0.953086 3.021495 0.305901
O -3.442916 2.445628 0.064126
O -5.176513 0.803048 -1.312770
C -5.172259 0.153595 0.994762
C -4.454060 -0.571310 2.109105
C -5.267810 -1.527720 2.938393
O -3.267440 -0.357779 2.356239
O -4.836923 -2.134751 -0.903465
C -5.634163 -1.962904 -2.075457
C 4.665837 0.076573 -0.581662
C 5.494764 0.976553 -1.451531
C 5.120351 -0.977916 0.129354
C 6.493734 -1.614314 0.221856
C 7.167336 -1.303320 1.574571
C 7.516269 0.162255 1.801495
C 7.449298 -1.404269 -0.954405
H 1.579488 3.167402 0.313071
H 2.457050 -1.464308 -0.981927

H 0.032213 -1.950218 -1.073278
H -2.318993 -2.483062 -1.182758
H -1.923745 3.175147 0.339814
H -5.268438 1.749991 -1.085510
H -6.123234 -0.354845 0.791023
H -5.439924 1.152355 1.360381
H -4.633221 -1.985145 3.702444
H -6.080487 -0.986409 3.428767
H -5.670419 -2.316567 2.298142
H -6.457125 -1.270396 -1.877631
H -5.039587 -1.626875 -2.932254
H -6.070875 -2.934315 -2.326138
H 4.944096 1.859086 -1.795269
H 6.368613 1.348701 -0.908311
H 5.821505 0.445984 -2.351215
H 4.403551 -1.484150 0.780868
H 6.294982 -2.696720 0.226310
H 6.510209 -1.632455 2.390077
H 8.084816 -1.898683 1.663414
H 6.625701 0.797013 1.778363
H 7.983161 0.284336 2.784487
H 8.224305 0.528650 1.053008
H 6.964783 -1.658689 -1.903138
H 8.322503 -2.058810 -0.851538
H 7.825026 -0.380758 -1.021193
SCF Energy (B3LYP/6-31G**)= -1361.69513863
Number of imaginary frequencies = 0

1a_c173
MMFF Geometry
C -1.734118 -1.209843 -1.028202
C -2.102166 1.298544 -0.054025
N -3.004379 -0.771088 -0.854689
C -0.587958 -0.464641 -0.742494
C -0.790019 0.832884 -0.237906
C -3.185117 0.478563 -0.368786
C 0.324744 1.637456 0.064851
C 1.631962 1.165011 -0.114834
C 1.821849 -0.130737 -0.602882
C 0.725517 -0.938306 -0.933610
C 3.206300 -0.603467 -0.793545
C 4.338797 0.098849 -0.019853
C 4.032638 1.576268 0.196180
C 2.771543 2.047821 0.157358
O 0.885324 -2.200224 -1.452394
O 3.431264 -1.545702 -1.558570
O 5.524174 -0.012749 -0.828817
C 4.621959 -0.560265 1.332202
C 4.540137 -2.066383 1.270721
C 5.773379 -2.822495 0.855271
O 3.479510 -2.643415 1.514571
O 5.154853 2.334936 0.477081
C 5.012269 3.742908 0.614409
C -4.598520 0.930046 -0.206122
C -4.849168 2.411841 -0.284399
C -5.568014 0.006331 -0.020924
C -7.051887 0.223365 0.176602
C -7.515245 -0.218973 1.580301
C -7.392617 -1.707246 1.884025
C -7.832562 -0.473816 -0.940173
H -1.659454 -2.222039 -1.420070
H -2.267162 2.293375 0.346154
H 0.164225 2.646439 0.443136
H 2.541946 3.092473 0.335992
H 1.842452 -2.365316 -1.602999
H 5.333083 -0.672130 -1.525584
H 3.894118 -0.241078 2.090148
H 5.613784 -0.287614 1.713313
H 5.619642 -3.891949 1.024854
H 5.973792 -2.656124 -0.204905
H 6.626202 -2.497902 1.456551
H 4.605520 4.187161 -0.299794
H 4.390420 3.989077 1.480958
H 6.006514 4.167251 0.782440
H -4.266436 2.864296 -1.095150
H -5.891000 2.661235 -0.497681
H -4.575654 2.898374 0.657694
H -5.271692 -1.044426 -0.002586
H -7.292000 1.288680 0.106334
H -6.948609 0.338555 2.337290
H -8.565949 0.070595 1.709846
H -6.355176 -2.048581 1.827572
H -7.750136 -1.908170 2.899499
H -7.996171 -2.308913 1.198523
H -7.580863 -0.040589 -1.915019
H -8.911232 -0.354521 -0.792667
H -7.612726 -1.545602 -0.990987
SCF Energy (B3LYP/6-31G**)= -1361.70429629
Number of imaginary frequencies = 0

1a_c174
MMFF Geometry
C -1.784752 -1.784284 -0.224681
C -2.086911 0.913678 -0.142308
N -3.040690 -1.288087 -0.111078
C -0.623414 -1.009939 -0.304601
C -0.792226 0.384631 -0.260354
C -3.183002 0.055923 -0.071662
C 0.338647 1.217661 -0.336238
C 1.633047 0.686665 -0.452761
C 1.796330 -0.701207 -0.490881
C 0.675342 -1.546323 -0.424297
C 3.160940 -1.261611 -0.618458
C 4.375539 -0.319150 -0.583343
C 4.041182 1.159872 -0.595540
C 2.773054 1.604093 -0.537669
O 0.793032 -2.916119 -0.476953
O 3.299817 -2.478414 -0.791048
O 5.074965 -0.606177 -1.818064
C 5.315928 -0.713711 0.562718
C 4.780882 -0.325527 1.921706
C 5.798138 0.016106 2.977524
O 3.577112 -0.338787 2.176900
O 5.164212 1.969410 -0.644134
C 4.984119 3.372274 -0.794132
C -4.574244 0.574195 0.040175
C -4.882871 1.812373 -0.751055
C -5.462279 -0.112728 0.791740
C -6.907873 0.155446 1.157727
C -7.880396 -0.733788 0.357917
C -7.920323 -0.468901 -1.141331
C -7.356247 1.618384 1.206158
H -1.733753 -2.870651 -0.250938
H -2.232991 1.987943 -0.091192
H 0.202460 2.297967 -0.301273
H 2.538781 2.662470 -0.533025
H 1.742591 -3.142941 -0.595966
H 5.121148 -1.579072 -1.888493
H 6.293437 -0.235815 0.418750
H 5.506776 -1.793735 0.573437
H 5.288976 0.277686 3.909242
H 6.444379 -0.846802 3.155547
H 6.392194 0.872983 2.650159
H 4.431415 3.601907 -1.710863
H 4.485686 3.797106 0.082947
H 5.973892 3.831595 -0.873104
H -5.902548 1.800831 -1.141761
H -4.741689 2.708946 -0.139464
H -4.241618 1.896105 -1.636225
H -5.098662 -1.030108 1.264550
H -6.987370 -0.183441 2.202224
H -7.616794 -1.787889 0.514759
H -8.893158 -0.609201 0.761523
H -6.944831 -0.637832 -1.606452
H -8.634972 -1.147518 -1.618793
H -8.239641 0.553353 -1.361944
H -6.628919 2.236399 1.743554
H -8.309780 1.698774 1.741076
H -7.514942 2.053447 0.217539
SCF Energy (B3LYP/6-31G**)= -1361.70107383
Number of imaginary frequencies = 0

1a_c175
MMFF Geometry
C -1.733766 -1.367545 -0.650603
C -2.126436 1.198797 0.148670
N -3.002808 -0.961518 -0.403396
C -0.600818 -0.560622 -0.526116
C -0.815779 0.767772 -0.112493
C -3.195418 0.316567 -0.003553
C 0.282801 1.638044 0.018426
C 1.586745 1.196626 -0.234708
C 1.793529 -0.130402 -0.627542
C 0.710438 -1.001736 -0.795035
C 3.184648 -0.568720 -0.883402
C 4.322123 0.212448 -0.174935
C 3.974841 1.697546 -0.162898
C 2.707458 2.136416 -0.184279
O 0.882272 -2.294043 -1.227149
O 3.376285 -1.545844 -1.608217
O 5.547577 0.026286 -0.888411
C 4.531826 -0.320042 1.247421
C 4.436309 -1.827638 1.318717
C 5.621998 -2.635203 0.862117
O 3.400398 -2.365223 1.712800
O 4.991556 2.632965 -0.295329
C 5.903125 2.649846 0.799832
C -4.604543 0.719830 0.275745
C -4.809088 1.839373 1.260300
C -5.614799 0.057394 -0.330416
C -7.102474 0.301827 -0.209776
C -7.820423 -0.891490 0.447308
C -7.405079 -1.119962 1.895292
C -7.683403 0.558888 -1.604439
H -1.648585 -2.405756 -0.964537
H -2.302744 2.224541 0.455056
H 0.113258 2.672317 0.315540
H 2.492746 3.200963 -0.217409
H 1.827816 -2.428093 -1.461758
H 5.422055 0.426615 -1.768479
H 3.774974 0.077818 1.936102
H 5.508691 -0.036493 1.654724
H 5.500046 -3.673549 1.183258
H 5.698500 -2.606255 -0.226487
H 6.534999 -2.241086 1.315323
H 5.376519 2.628733 1.760029
H 6.611798 1.820366 0.728760
H 6.473821 3.581709 0.746194
H -4.621636 2.808277 0.785957
H -5.818272 1.861557 1.678761
H -4.136573 1.732545 2.119259
H -5.360190 -0.759763 -1.009171
H -7.308826 1.196786 0.385292
H -8.903442 -0.716715 0.425473
H -7.639133 -1.809579 -0.125906
H -7.567894 -0.219760 2.496385
H -7.998258 -1.929860 2.331999
H -6.350759 -1.402062 1.972495
H -7.194657 1.416122 -2.081057
H -8.754575 0.779768 -1.542504
H -7.557597 -0.309738 -2.260868
SCF Energy (B3LYP/6-31G**)= -1361.69493293
Number of imaginary frequencies = 0

1a_c176
MMFF Geometry
C -1.705000 -1.370102 -0.990893
C -2.160689 1.195770 -0.224079
N -2.989690 -0.966089 -0.839928
C -0.585889 -0.561522 -0.781106
C -0.833375 0.765955 -0.382957
C -3.213761 0.312250 -0.457771
C 0.251081 1.635935 -0.162814
C 1.571084 1.195838 -0.314789
C 1.808733 -0.129680 -0.695068
C 0.742616 -1.001093 -0.948780
C 3.215841 -0.566574 -0.843171
C 4.293993 0.212149 -0.044959
C 3.945945 1.697096 -0.054425
C 2.683881 2.135723 -0.173279
O 0.948529 -2.292283 -1.369146
O 3.464089 -1.540776 -1.554609
O 5.571624 0.029183 -0.661083
C 4.391996 -0.325947 1.387336
C 4.293486 -1.833967 1.444705
C 5.512670 -2.638162 1.079504
O 3.230593 -2.374533 1.753671
O 4.969377 2.633295 -0.103295
C 5.792135 2.646399 1.060120
C -4.641049 0.722611 -0.316379
C -4.952587 2.182377 -0.506620
C -5.576724 -0.216746 -0.054430
C -7.062589 -0.025442 0.152182
C -7.530322 -0.644681 1.483945
C -6.921373 0.032464 2.705763
C -7.819764 -0.660193 -1.016791
H -1.594804 -2.407975 -1.298094
H -2.360627 2.213699 0.093696
H 0.058207 2.669009 0.124054
H 2.471832 3.200336 -0.218883
H 1.909477 -2.425075 -1.530388
H 5.515075 0.432846 -1.546738
H 3.582733 0.068150 2.015965
H 5.333385 -0.042955 1.871333
H 5.367855 -3.677774 1.386753
H 5.673911 -2.605377 0.000302
H 6.386783 -2.244224 1.603939
H 5.191785 2.621544 1.975927
H 6.504703 1.817411 1.041840
H 6.364715 3.578636 1.054884
H -4.386132 2.597462 -1.348236
H -6.003905 2.366543 -0.741743
H -4.705419 2.749577 0.396766
H -5.251836 -1.255294 0.040957
H -7.323404 1.037174 0.178221
H -8.622196 -0.562723 1.555167
H -7.293576 -1.715845 1.512691
H -7.136782 1.105716 2.706969
H -7.340847 -0.397760 3.620870
H -5.836140 -0.102544 2.742152
H -7.517707 -0.211375 -1.969760
H -8.899145 -0.509499 -0.906618
H -7.635859 -1.738899 -1.078281
SCF Energy (B3LYP/6-31G**)= -1361.69498836
Number of imaginary frequencies = 0

1a_c177
MMFF Geometry
C 1.799323 1.748248 -0.257038
C 2.118048 -0.944448 -0.097179
N 3.059740 1.262164 -0.149244
C 0.641137 0.966077 -0.294576
C 0.818800 -0.425955 -0.210693
C 3.210269 -0.079323 -0.070695
C -0.307743 -1.267174 -0.243068
C -1.605255 -0.745625 -0.356390
C -1.779392 0.640441 -0.432684
C -0.662435 1.492593 -0.409266
C -3.151152 1.190542 -0.558602
C -4.364986 0.244453 -0.488197
C -4.002095 -1.224079 -0.478509
C -2.740929 -1.665560 -0.390191
O -0.788327 2.859493 -0.501533
O -3.300093 2.400472 -0.768243
O -5.083970 0.521778 -1.711706
C -5.281792 0.640122 0.674438
C -4.701670 0.262666 2.017590
C -5.647912 -0.322685 3.030811
O -3.517985 0.464629 2.287427
O -5.034130 -2.149532 -0.377992
C -5.741089 -2.305845 -1.608617
C 4.606114 -0.586699 0.034481
C 4.908322 -1.848734 -0.720755
C 5.502823 0.128796 0.748159

C 6.955669 -0.120302 1.098561
C 7.910137 0.746576 0.253729
C 7.926682 0.432451 -1.236422
C 7.412494 -1.578699 1.187812
H 1.741927 2.833051 -0.315076
H 2.270641 -2.015940 -0.015261
H -0.167168 -2.345541 -0.176713
H -2.545021 -2.731372 -0.315593
H -1.741535 3.079123 -0.601894
H -5.105300 1.495745 -1.799685
H -6.259515 0.155622 0.557469
H -5.482110 1.718362 0.685162
H -5.105304 -0.553473 3.951695
H -6.436686 0.399609 3.254539
H -6.080394 -1.246000 2.637437
H -6.509671 -1.534057 -1.706590
H -5.068566 -2.298094 -2.473594
H -6.246525 -3.275882 -1.581045
H 4.253560 -1.964213 -1.592357
H 5.921580 -1.845461 -1.127951
H 4.781367 -2.725441 -0.078036
H 5.142116 1.059282 1.196958
H 7.050645 0.253337 2.129830
H 7.643610 1.804030 0.380093
H 8.930074 0.640189 0.644266
H 6.942762 0.581309 -1.690519
H 8.629761 1.098273 -1.747866
H 8.247690 -0.595020 -1.428496
H 6.697308 -2.182059 1.757205
H 8.375038 -1.636830 1.709310
H 7.557366 -2.045452 0.211604
SCF Energy (B3LYP/6-31G**)= -1361.69539371
Number of imaginary frequencies = 0

1a_c178
MMFF Geometry
C 1.928702 1.685304 0.005924
C 2.143340 -1.020102 -0.097103
N 3.165172 1.138355 0.090609
C 0.743624 0.958795 -0.131041
C 0.866932 -0.441331 -0.185684
C 3.269502 -0.209852 0.041108
C -0.288549 -1.230345 -0.334832
C -1.559959 -0.647058 -0.414726
C -1.677553 0.744234 -0.343938
C -0.534675 1.547036 -0.219046
C -3.029781 1.344513 -0.412238
C -4.262809 0.452801 -0.160802
C -3.974312 -0.981571 -0.549886
C -2.735392 -1.490420 -0.620953
O -0.614745 2.918373 -0.175610
O -3.143049 2.556575 -0.622622
O -5.302186 0.983238 -0.998042
C -4.654400 0.644455 1.307857
C -5.712993 -0.314135 1.806722
C -7.117796 -0.154036 1.285830
O -5.436137 -1.172089 2.647495
O -5.073862 -1.807388 -0.723657
C -5.365651 -2.002891 -2.107744
C 4.645529 -0.774588 0.154801
C 4.766273 -2.170358 0.704832
C 5.705655 -0.016133 -0.202494
C 7.171298 -0.394553 -0.188393
C 7.882340 0.364827 0.945958
C 9.339625 -0.038263 1.124840
C 7.781045 -0.095020 -1.561648
H 1.916522 2.772045 0.054174
H 2.248028 -2.098861 -0.150339
H -0.190751 -2.314359 -0.389495
H -2.587733 -2.544882 -0.836333
H -1.538994 3.189863 -0.373127
H -5.164024 1.951460 -1.033746
H -5.029538 1.660760 1.484340
H -3.777649 0.522611 1.958132
H -7.796166 -0.779577 1.873276
H -7.432213 0.887311 1.391780
H -7.177924 -0.460525 0.240685
H -5.533124 -1.051262 -2.621970
H -4.562997 -2.561965 -2.600101
H -6.283530 -2.593950 -2.176675
H 4.533301 -2.910329 -0.067834
H 5.764930 -2.390219 1.091221
H 4.084843 -2.318643 1.550498
H 5.517198 0.998867 -0.559235
H 7.289230 -1.469492 -0.018062
H 7.831476 1.447142 0.771183
H 7.358882 0.175459 1.892221
H 9.945430 0.250124 0.261005
H 9.760811 0.459882 2.004122
H 9.433555 -1.118897 1.270987
H 7.207843 -0.581557 -2.359265
H 8.807895 -0.468087 -1.628216
H 7.798357 0.981075 -1.768080
SCF Energy (B3LYP/6-31G**)= -1361.70265959
Number of imaginary frequencies = 0

1a_c179
MMFF Geometry
C -1.781082 -1.291453 -0.550699
C -2.135097 1.246211 0.349419
N -3.045458 -0.870807 -0.304457
C -0.634254 -0.513304 -0.377231
C -0.829529 0.799925 0.087558
C -3.218996 0.393090 0.146111
C 0.284725 1.639356 0.272651
C 1.585379 1.186437 0.014674
C 1.772616 -0.125533 -0.431535
C 0.673020 -0.968440 -0.644347
C 3.150802 -0.579943 -0.712367
C 4.336802 0.198287 -0.105657
C 3.983640 1.662259 0.089532
C 2.718168 2.097119 0.180149
O 0.822031 -2.249119 -1.119290
O 3.334157 -1.567390 -1.431576
O 5.406978 0.090746 -1.060861
C 4.813512 -0.389775 1.224918
C 4.755673 -1.898037 1.252908
C 5.959725 -2.656275 0.762675
O 3.736384 -2.477241 1.630022
O 5.010788 2.546120 0.391091
C 5.719061 2.970055 -0.773574
C -4.623610 0.812897 0.422524
C -4.822736 1.904415 1.439513
C -5.641075 0.187154 -0.210050
C -7.122044 0.470190 -0.091017
C -7.828977 -0.548624 0.823698
C -7.797192 -1.995130 0.345158
C -7.757335 0.537378 -1.483608
H -1.711900 -2.317264 -0.906407
H -2.294954 2.261548 0.696865
H 0.130540 2.659963 0.621619
H 2.516555 3.140309 0.407071
H 1.770247 -2.405343 -1.324928
H 5.208582 -0.688986 -1.619275
H 4.188605 -0.043597 2.059158
H 5.841333 -0.082007 1.453364
H 5.826180 -3.722978 0.963667
H 6.080217 -2.510636 -0.312544
H 6.851981 -2.313952 1.292662
H 6.541121 2.282506 -0.989800
H 5.063113 3.072749 -1.645377
H 6.154700 3.950773 -0.559761
H -4.614735 2.884731 0.998416
H -5.837998 1.927044 1.844726
H -4.163560 1.760059 2.303258
H -5.396241 -0.617179 -0.906741
H -7.274953 1.460166 0.353748
H -7.380147 -0.504397 1.824412
H -8.877360 -0.247926 0.944731
H -6.772744 -2.366125 0.247609
H -8.313376 -2.635979 1.067713
H -8.303180 -2.111198 -0.617518

H -7.316875 1.352123 -2.069755
H -8.833714 0.725902 -1.409726
H -7.613118 -0.389143 -2.049490
SCF Energy (B3LYP/6-31G**)= -1361.69875439
Number of imaginary frequencies = 0

1a_c180
MMFF Geometry
C -1.863493 -1.137502 -1.192617
C -2.104739 1.211405 0.145927
N -3.110613 -0.668170 -0.945418
C -0.680393 -0.499502 -0.811729
C -0.817829 0.715269 -0.120178
C -3.228190 0.501966 -0.276445
C 0.336234 1.405952 0.290340
C 1.621068 0.908883 0.021600
C 1.756103 -0.303880 -0.662278
C 0.609978 -1.003188 -1.080096
C 3.114805 -0.829215 -0.955802
C 4.356235 -0.022853 -0.500987
C 4.033314 1.264921 0.229441
C 2.781977 1.668435 0.479514
O 0.693016 -2.191765 -1.768332
O 3.208944 -1.871307 -1.612280
O 5.061559 0.326234 -1.711470
C 5.290794 -0.944987 0.293541
C 4.721349 -1.372427 1.628858
C 5.709276 -1.874197 2.648744
O 3.513980 -1.363196 1.866039
O 5.077612 1.977680 0.806932
C 5.923534 2.611065 -0.151441
C -4.617437 0.992173 -0.033822
C -4.792055 2.477927 0.130354
C -5.633240 0.101030 0.006995
C -7.104477 0.357595 0.246928
C -7.589641 -0.274439 1.568218
C -7.540060 -1.795999 1.636373
C -7.918625 -0.118171 -0.958751
H -1.839927 -2.083169 -1.729886
H -2.217232 2.141220 0.693482
H 0.227241 2.345273 0.831806
H 2.612117 2.566170 1.067575
H 1.643390 -2.427897 -1.869952
H 4.425555 0.769248 -2.299690
H 6.250527 -0.442963 0.468753
H 5.522808 -1.853369 -0.276306
H 5.183615 -2.149340 3.567315
H 6.224262 -2.754575 2.256719
H 6.430003 -1.085898 2.879732
H 6.653574 1.900096 -0.548272
H 5.346975 3.070906 -0.961533
H 6.476262 3.402747 0.363143
H -4.192038 3.025258 -0.605856
H -5.820866 2.810460 -0.024332
H -4.487882 2.792223 1.134246
H -5.390868 -0.952671 -0.145164
H -7.291524 1.431320 0.344421
H -6.996732 0.130605 2.398487
H -8.624967 0.042023 1.748653
H -6.520508 -2.174155 1.520153
H -7.907390 -2.134747 2.610895
H -8.172010 -2.253939 0.870135
H -7.645228 0.448290 -1.856381
H -8.990039 0.029931 -0.786527
H -7.752652 -1.178589 -1.176925
SCF Energy (B3LYP/6-31G**)= -1361.70036978
Number of imaginary frequencies = 0

1a_c181
MMFF Geometry
C 1.915848 1.745170 -0.355546
C 2.161269 -0.957190 -0.468073
N 3.162933 1.216101 -0.362130
C 0.734470 1.001456 -0.401649
C 0.873514 -0.397023 -0.459876
C 3.282953 -0.130574 -0.418242
C -0.278535 -1.203162 -0.513688
C -1.560661 -0.638307 -0.498183
C -1.692576 0.751640 -0.426384
C -0.555187 1.571142 -0.393922
C -3.054544 1.332565 -0.393557
C -4.251802 0.425437 -0.043717
C -3.973198 -1.007516 -0.444971
C -2.736147 -1.499467 -0.608024
O -0.651488 2.941439 -0.350497
O -3.200992 2.541601 -0.600909
O -5.359206 0.935538 -0.802756
C -4.533390 0.621943 1.449304
C -5.538910 -0.346157 2.032522
C -6.980834 -0.207727 1.617640
O -5.188549 -1.194254 2.855687
O -5.070710 -1.849940 -0.528433
C -5.465318 -2.059566 -1.884669
C 4.672476 -0.673696 -0.438262
C 4.869393 -2.017723 -1.086268
C 5.678281 0.059568 0.088596
C 7.144351 -0.302308 0.195653
C 7.600070 -0.152892 1.660559
C 9.028846 -0.620966 1.901788
C 7.950130 0.583279 -0.757008
H 1.891670 2.831815 -0.309731
H 2.276585 -2.035713 -0.499473
H -0.169565 -2.286056 -0.569419
H -2.590233 -2.553337 -0.827451
H -1.591941 3.198809 -0.478037
H -5.238566 1.905415 -0.853778
H -4.907048 1.634701 1.647953
H -3.608357 0.515230 2.031813
H -7.605134 -0.835704 2.259884
H -7.299528 0.830738 1.738120
H -7.115551 -0.524788 0.582650
H -5.685930 -1.114160 -2.390244
H -4.694812 -2.610690 -2.434047
H -6.376958 -2.664120 -1.879003
H 4.280687 -2.098527 -2.007359
H 5.905182 -2.199521 -1.384052
H 4.568709 -2.821070 -0.405749
H 5.436866 1.038048 0.509849
H 7.306449 -1.345431 -0.093164
H 7.509839 0.891942 1.984201
H 6.935478 -0.740098 2.307738
H 9.752952 0.015251 1.384842
H 9.260060 -0.582215 2.971240
H 9.167376 -1.652487 1.563119
H 7.563349 0.511581 -1.780092
H 9.000915 0.278764 -0.787643
H 7.911551 1.636303 -0.456119
SCF Energy (B3LYP/6-31G**)= -1361.70270807
Number of imaginary frequencies = 0

1a_c182
MMFF Geometry
C -1.733874 -1.906961 -0.386673
C -2.161359 0.686582 0.291490
N -3.004483 -1.518443 -0.119538
C -0.616577 -1.068737 -0.342801
C -0.850353 0.271150 0.006773
C -3.212764 -0.226092 0.220899
C 0.233592 1.165627 0.061113
C 1.544457 0.748677 -0.222818
C 1.774991 -0.587182 -0.563189
C 0.699008 -1.491008 -0.627049
C 3.158043 -1.033620 -0.860849
C 4.319542 -0.019706 -0.759206
C 3.907365 1.398780 -0.399475
C 2.627913 1.732049 -0.157764
O 0.876787 -2.811212 -0.972144
O 3.328582 -2.207912 -1.204742
O 4.918479 0.032998 -2.073108
C 5.400291 -0.591092 0.169355
C 4.969456 -0.704290 1.615096
C 6.051005 -1.044996 2.606856

O 3.802395 -0.559691 1.976675
O 4.975834 2.277991 -0.359855
C 4.734124 3.634451 -0.005140
C -4.620085 0.162355 0.527947
C -4.804181 1.267658 1.529691
C -5.630264 -0.505859 -0.072338
C -7.130092 -0.321480 0.020609
C -7.657015 1.105127 -0.229070
C -7.219932 1.685212 -1.568342
C -7.681805 -0.900265 1.326005
H -1.633150 -2.958023 -0.648780
H -2.352917 1.722133 0.552455
H 0.047072 2.203947 0.333486
H 2.341163 2.744588 0.103413
H 1.835417 -2.962287 -1.140089
H 4.346045 0.585929 -2.630969
H 6.301084 0.034342 0.126787
H 5.705706 -1.590500 -0.165773
H 5.626005 -1.096473 3.613112
H 6.485411 -2.015632 2.355525
H 6.821352 -0.270126 2.589794
H 4.075323 4.117486 -0.733797
H 4.322505 3.706693 1.006676
H 5.693022 4.160932 -0.017163
H -4.665565 2.244994 1.055923
H -5.790732 1.255660 1.996063
H -4.088590 1.173894 2.354743
H -5.359905 -1.313361 -0.757709
H -7.551899 -0.940235 -0.785504
H -8.754373 1.080563 -0.212783
H -7.361036 1.789160 0.571808
H -7.511292 1.028672 -2.394345
H -7.694871 2.658695 -1.727649
H -6.136957 1.834319 -1.609477
H -7.344434 -1.932553 1.472239
H -8.777211 -0.910711 1.308450
H -7.371321 -0.318680 2.199010
SCF Energy (B3LYP/6-31G**)= -1361.70223074
Number of imaginary frequencies = 0

1a_c183
MMFF Geometry
C -1.746112 -1.088491 -1.106797
C -2.135164 1.310670 0.105090
N -3.019983 -0.670497 -0.909929
C -0.606075 -0.373977 -0.732036
C -0.819294 0.867212 -0.104849
C -3.211249 0.524655 -0.305240
C 0.288160 1.639411 0.292675
C 1.598376 1.188906 0.084939
C 1.801608 -0.053348 -0.525954
C 0.711523 -0.825529 -0.949385
C 3.191838 -0.505800 -0.746591
C 4.323217 0.132642 0.083768
C 3.986803 1.570374 0.430964
C 2.728906 2.036608 0.466599
O 0.880687 -2.030953 -1.586455
O 3.425788 -1.379772 -1.586916
O 5.499405 0.119848 -0.743966
C 4.626177 -0.643964 1.367224
C 4.553109 -2.139100 1.172531
C 5.799789 -2.850877 0.720687
O 3.491219 -2.739687 1.341446
O 5.033337 2.355323 0.891188
C 5.517937 3.221856 -0.135275
C -4.628228 0.955972 -0.119505
C -4.881123 2.437808 -0.048806
C -5.598388 0.017449 -0.045384
C -7.085679 0.211837 0.150010
C -7.569319 -0.370637 1.494454
C -7.447063 -1.881816 1.648197
C -7.847300 -0.369771 -1.043477
H -1.663212 -2.057000 -1.595420
H -2.308187 2.260749 0.599608
H 0.120884 2.605317 0.768211
H 2.528646 3.050235 0.801414
H 1.840516 -2.184246 -1.731628
H 5.345226 -0.550633 -1.440341
H 3.905333 -0.400828 2.159335
H 5.620403 -0.397988 1.760013
H 5.639214 -3.931787 0.764887
H 6.038510 -2.569451 -0.306753
H 6.630296 -2.598139 1.384447
H 5.852816 2.660459 -1.012995
H 4.752606 3.950478 -0.423195
H 6.375231 3.770336 0.265841
H -4.288650 2.971457 -0.801003
H -5.920536 2.707048 -0.249273
H -4.621292 2.825389 0.941822
H -5.299949 -1.029437 -0.130054
H -7.327594 1.278505 0.183909
H -7.016003 0.108311 2.312621
H -8.622721 -0.097019 1.636177
H -6.407887 -2.214311 1.573738
H -7.819568 -2.184777 2.632490
H -8.038254 -2.411955 0.896108
H -7.581783 0.159982 -1.965554
H -8.928415 -0.267099 -0.901458
H -7.623938 -1.430715 -1.198856
SCF Energy (B3LYP/6-31G**)= -1361.69929214
Number of imaginary frequencies = 0

1a_c184
MMFF Geometry
C 1.792217 2.385440 -0.164066
C 2.399629 -0.184996 0.475666
N 3.079622 2.093519 0.140738
C 0.743947 1.459982 -0.174190
C 1.070378 0.132178 0.159882
C 3.372982 0.812083 0.448990
C 0.060840 -0.847554 0.166070
C -1.266127 -0.522545 -0.144282
C -1.587637 0.800268 -0.463174
C -0.589912 1.785407 -0.494135
C -2.997816 1.130864 -0.772531
C -4.110145 0.144617 -0.361748
C -3.581918 -1.273642 -0.325022
C -2.282309 -1.572055 -0.180633
O -0.869914 3.088666 -0.828967
O -3.260990 2.210907 -1.311515
O -5.122730 0.259400 -1.373791
C -4.673919 0.645413 0.971809
C -5.633519 -0.309087 1.647493
C -6.984630 -0.523193 1.015748
O -5.328399 -0.859487 2.707488
O -4.529072 -2.285587 -0.330515
C -4.647386 -2.882184 -1.622455
C 4.792088 0.534558 0.781477
C 5.442564 1.589925 1.635823
C 5.402709 -0.566508 0.292694
C 6.833250 -1.008305 0.514236
C 7.721453 -0.710937 -0.709957
C 7.343913 -1.441840 -1.992722
C 6.866891 -2.487787 0.910075
H 1.617070 3.430758 -0.409674
H 2.671023 -1.198932 0.753662
H 0.318131 -1.876758 0.415462
H -1.964632 -2.606896 -0.089538
H -1.798403 3.142887 -1.148295
H -5.121829 1.194112 -1.663820
H -5.204059 1.597522 0.840628
H -3.860323 0.841412 1.683175
H -7.626757 -1.073099 1.709911
H -7.450716 0.443527 0.809091
H -6.891717 -1.100431 0.094860
H -4.895273 -2.139527 -2.387370
H -3.727370 -3.410725 -1.893459
H -5.459952 -3.613362 -1.580993
H 4.841722 1.779155 2.532754
H 6.441006 1.314764 1.981128
H 5.543592 2.529590 1.081750
H 4.835781 -1.225309 -0.365595

H 7.268772 -0.462941 1.357578
H 7.708419 0.368543 -0.908591
H 8.760145 -0.964174 -0.462397
H 6.330183 -1.190698 -2.317912
H 8.029142 -1.155593 -2.797571
H 7.413026 -2.526738 -1.872539
H 6.331008 -2.647120 1.852854
H 7.898300 -2.827425 1.052979
H 6.399936 -3.128962 0.154855
SCF Energy (B3LYP/6-31G**)= -1361.70113952
Number of imaginary frequencies = 0

1a_c185
MMFF Geometry
C -1.805506 -1.723034 -0.237459
C -2.100921 0.971503 -0.093130
N -3.059437 -1.224187 -0.112912
C -0.641971 -0.951757 -0.300153
C -0.807188 0.440725 -0.224533
C -3.202665 0.118930 -0.040052
C 0.326830 1.270606 -0.282419
C 1.620108 0.738557 -0.413774
C 1.781659 -0.647976 -0.483903
C 0.655619 -1.489361 -0.431087
C 3.145571 -1.215358 -0.620204
C 4.362150 -0.263324 -0.652437
C 4.023837 1.217450 -0.586925
C 2.758574 1.657708 -0.478397
O 0.765812 -2.858786 -0.509596
O 3.257352 -2.441089 -0.726965
O 5.014752 -0.493505 -1.920880
C 5.368177 -0.703523 0.420367
C 4.874065 -0.516318 1.837889
C 5.892509 -0.717625 2.929512
O 3.704473 -0.244771 2.106190
O 5.141948 2.030490 -0.657714
C 4.970183 3.440406 -0.574841
C -4.598466 0.633089 0.081343
C -4.864070 1.990345 -0.507334
C -5.534451 -0.145168 0.669195
C -7.000835 0.109941 0.946500
C -7.899116 0.015049 -0.300707
C -7.812621 -1.332207 -1.007458
C -7.257398 1.404906 1.723817
H -1.758905 -2.808774 -0.288086
H -2.236206 2.045400 -0.018734
H 0.193677 2.350203 -0.220520
H 2.524395 2.715144 -0.426773
H 1.719323 -3.089472 -0.596780
H 4.499960 -0.027439 -2.600864
H 6.306613 -0.146019 0.306659
H 5.624793 -1.763150 0.294913
H 5.425421 -0.554019 3.904647
H 6.276715 -1.739701 2.887580
H 6.708645 -0.001507 2.806037
H 4.372530 3.811228 -1.413638
H 4.523108 3.724269 0.383267
H 5.959428 3.903587 -0.635430
H -5.920460 2.160835 -0.720748
H -4.526582 2.779030 0.173084
H -4.347223 2.110193 -1.466536
H -5.216230 -1.126991 1.029455
H -7.315571 -0.698533 1.623139
H -8.942263 0.176783 -0.000765
H -7.658396 0.806932 -1.017953
H -8.049692 -2.151358 -0.321179
H -8.527907 -1.367588 -1.835519
H -6.815226 -1.505328 -1.422879
H -6.582313 1.490624 2.582410
H -8.283302 1.416750 2.109183
H -7.137430 2.298109 1.105280
SCF Energy (B3LYP/6-31G**)= -1361.70241599
Number of imaginary frequencies = 0

1a_c186
MMFF Geometry
C 2.024966 1.558629 -0.122472
C 2.167906 -1.151580 -0.021625
N 3.246498 0.987007 0.004906
C 0.821025 0.855513 -0.206243
C 0.907205 -0.547514 -0.155332
C 3.315278 -0.363429 0.056910
C -0.268865 -1.314649 -0.246427
C -1.524426 -0.705592 -0.371797
C -1.605089 0.689704 -0.405956
C -0.441281 1.469100 -0.340081
C -2.940977 1.318713 -0.521156
C -4.197059 0.481357 -0.205218
C -3.946863 -0.985336 -0.484965
C -2.721894 -1.530682 -0.515768
O -0.484964 2.841459 -0.399937
O -3.022208 2.514103 -0.822206
O -5.222459 0.974662 -1.081485
C -4.582665 0.793165 1.244305
C -5.666022 -0.096867 1.812251
C -7.066363 0.060737 1.278736
O -5.411553 -0.896218 2.715531
O -5.067993 -1.792504 -0.597783
C -5.365599 -2.083704 -1.963686
C 4.676057 -0.953827 0.215005
C 4.760907 -2.312186 0.857901
C 5.757499 -0.247852 -0.183790
C 7.209792 -0.667534 -0.133502
C 7.950638 -0.028202 1.056808
C 8.037305 1.493144 1.037193
C 7.891030 -0.362732 -1.471577
H 2.041654 2.645861 -0.155993
H 2.243614 -2.233675 0.006775
H -0.199859 -2.401913 -0.219309
H -2.602333 -2.601888 -0.651064
H -1.401799 3.121696 -0.618656
H -5.058721 1.933467 -1.189654
H -4.930654 1.829430 1.343352
H -3.709110 0.697395 1.903186
H -7.760761 -0.500697 1.910545
H -7.353021 1.115057 1.305649
H -7.135141 -0.321760 0.259499
H -5.508090 -1.169324 -2.548220
H -4.578295 -2.699235 -2.411466
H -6.298844 -2.653792 -1.989338
H 4.502149 -3.095894 0.138389
H 5.756258 -2.533762 1.252409
H 4.081443 -2.382986 1.715060
H 5.595697 0.744656 -0.609115
H 7.276773 -1.754085 -0.006370
H 7.462370 -0.335797 1.990516
H 8.970331 -0.431656 1.097136
H 7.045458 1.954525 1.040816
H 8.569015 1.845589 1.927298
H 8.586121 1.853196 0.162319
H 7.417543 -0.927271 -2.283049
H 8.948171 -0.647621 -1.441879
H 7.832933 0.699169 -1.733211
SCF Energy (B3LYP/6-31G**)= -1361.70176949
Number of imaginary frequencies = 0

1a_c187
MMFF Geometry
C -1.834640 -1.146104 -1.216239
C -2.056412 1.202481 0.126215
N -3.077971 -0.670938 -0.961153
C -0.646198 -0.514380 -0.841628
C -0.773489 0.700456 -0.148049
C -3.185806 0.498897 -0.290112
C 0.386319 1.385980 0.255231
C 1.667108 0.882270 -0.020205
C 1.791670 -0.332318 -0.704377
C 0.639878 -1.024175 -1.118544
C 3.146011 -0.867482 -1.002449
C 4.391362 -0.111302 -0.482305
C 4.081038 1.205376 0.193860
C 2.835242 1.643090 0.420167

O 0.713113 -2.211047 -1.810824
O 3.233202 -1.880716 -1.703108
O 5.205481 0.186551 -1.637238
C 5.233329 -1.050851 0.390144
C 4.585188 -1.387640 1.714217
C 5.508516 -1.809474 2.826489
O 3.364903 -1.374577 1.872600
O 5.171878 1.896719 0.701318
C 5.497278 3.033059 -0.099238
C -4.571159 0.995541 -0.038576
C -4.737812 2.482014 0.127142
C -5.590820 0.109124 0.008331
C -7.059306 0.372447 0.257600
C -7.539186 -0.258121 1.581518
C -7.496412 -1.779941 1.648438
C -7.883194 -0.098719 -0.943256
H -1.818874 -2.091308 -1.754617
H -2.161303 2.132206 0.675381
H 0.284521 2.326259 0.796249
H 2.672035 2.564246 0.971877
H 1.661927 -2.441327 -1.936804
H 4.629338 0.607371 -2.297792
H 6.211881 -0.594165 0.587300
H 5.437881 -1.994493 -0.130745
H 4.926888 -2.024097 3.727294
H 6.050271 -2.710881 2.529972
H 6.210387 -1.001318 3.046710
H 5.707739 2.744625 -1.133918
H 4.694637 3.777275 -0.069179
H 6.399666 3.489841 0.317514
H -4.139513 3.026805 -0.612344
H -5.765936 2.819420 -0.021464
H -4.426316 2.794492 1.129353
H -5.354303 -0.945641 -0.145732
H -7.240660 1.446984 0.356920
H -6.939177 0.143577 2.408305
H -8.571848 0.063137 1.768622
H -6.479416 -2.162863 1.525612
H -7.859245 -2.117560 2.625034
H -8.135312 -2.234388 0.885893
H -7.612747 0.467022 -1.842232
H -8.952803 0.054305 -0.764252
H -7.723572 -1.159766 -1.163106
SCF Energy (B3LYP/6-31G**)= -1361.70004577
Number of imaginary frequencies = 0

1a_c188
MMFF Geometry
C -1.355507 -2.320222 -0.519530
C -2.303196 0.226797 -0.446928
N -2.697793 -2.142631 -0.460416
C -0.413740 -1.287194 -0.547299
C -0.916326 0.028504 -0.511494
C -3.157353 -0.873822 -0.414640
C -0.021807 1.114074 -0.554568
C 1.361154 0.906015 -0.608763
C 1.854067 -0.403675 -0.624764
C 0.978175 -1.496231 -0.616554
C 3.322086 -0.590840 -0.677645
C 4.209574 0.561095 -0.137839
C 3.591136 1.896563 -0.538136
C 2.273905 2.041737 -0.745814
O 1.435255 -2.789741 -0.682465
O 3.770671 -1.654459 -1.107017
O 5.518242 0.464578 -0.706628
C 4.359699 0.452841 1.384112
C 4.542035 -0.975851 1.845285
C 5.900891 -1.604013 1.685817
O 3.588982 -1.604411 2.307910
O 4.423101 2.961042 -0.855873
C 5.190109 3.439326 0.245751
C -4.631218 -0.725725 -0.353711
C -5.386929 -1.671836 -1.247749
C -5.197224 0.180272 0.472238
C -6.675672 0.427097 0.691463
C -7.048358 1.809072 0.125073
C -8.540444 2.106276 0.184484
C -6.989490 0.305804 2.185918
H -1.041939 -3.361810 -0.545389
H -2.714634 1.231346 -0.436989
H -0.413870 2.130453 -0.551194
H 1.868426 2.995834 -1.071030
H 2.408997 -2.776539 -0.819407
H 5.416357 0.601069 -1.666590
H 3.471274 0.840740 1.899504
H 5.215714 1.026518 1.756520
H 5.942325 -2.532143 2.262900
H 6.088227 -1.830252 0.634402
H 6.668803 -0.927430 2.069016
H 4.575035 3.547379 1.145614
H 6.044945 2.785268 0.437515
H 5.578687 4.426823 -0.020322
H -5.440325 -2.668179 -0.796492
H -6.406558 -1.336139 -1.453441
H -4.896856 -1.759740 -2.224568
H -4.550944 0.799625 1.094807
H -7.273872 -0.332065 0.177726
H -6.506669 2.599610 0.660029
H -6.731673 1.867509 -0.924501
H -8.891786 2.195371 1.216473
H -8.753852 3.054450 -0.319605
H -9.117904 1.321090 -0.313481
H -6.641507 -0.655011 2.582397
H -8.067211 0.359588 2.369311
H -6.508056 1.102589 2.764049
SCF Energy (B3LYP/6-31G**)= -1361.69278154
Number of imaginary frequencies = 0

1a_c189
MMFF Geometry
C -1.761309 -1.099295 -1.085861
C -2.155241 1.318391 0.086747
N -3.036054 -0.680313 -0.896881
C -0.622709 -0.376989 -0.721661
C -0.838509 0.873596 -0.114876
C -3.229737 0.524209 -0.311809
C 0.267530 1.653249 0.271517
C 1.578921 1.201803 0.071877
C 1.785069 -0.048659 -0.519082
C 0.696042 -0.829480 -0.930438
C 3.175644 -0.500658 -0.735109
C 4.314727 0.164023 0.065521
C 3.967986 1.600883 0.413959
C 2.706271 2.054267 0.449102
O 0.867158 -2.045099 -1.547601
O 3.407131 -1.394922 -1.555258
O 5.463696 0.148586 -0.799888
C 4.662204 -0.598399 1.346722
C 4.580254 -2.096280 1.177424
C 5.812362 -2.817734 0.701592
O 3.522970 -2.691852 1.387387
O 4.977139 2.412801 0.913596
C 5.798128 2.954949 -0.120711
C -4.647561 0.956296 -0.134385
C -4.902638 2.438709 -0.087293
C -5.616454 0.017649 -0.046498
C -7.104229 0.212934 0.144236
C -7.588434 -0.348753 1.497294
C -7.464086 -1.857101 1.675246
C -7.863739 -0.388756 -1.040612
H -1.676398 -2.075376 -1.558792
H -2.330003 2.276102 0.565700
H 0.098497 2.625928 0.732509
H 2.501205 3.065729 0.788638
H 1.827366 -2.199206 -1.689230
H 5.303885 -0.549956 -1.467487
H 3.970439 -0.343674 2.160895
H 5.669908 -0.348231 1.700665
H 5.645933 -3.897437 0.752894
H 6.030925 -2.541576 -0.331747
H 6.658320 -2.567593 1.346555
H 6.627484 2.277697 -0.341591

H 5.229217 3.177308 -1.030522
H 6.224956 3.891934 0.249671
H -4.310357 2.961262 -0.847390
H -5.942280 2.703260 -0.292744
H -4.644133 2.842265 0.897285
H -5.316408 -1.029997 -0.114319
H -7.347750 1.279643 0.160862
H -7.036704 0.144016 2.308293
H -8.642392 -0.074497 1.633539
H -6.424336 -2.189172 1.607176
H -7.837182 -2.144889 2.663858
H -8.053686 -2.400057 0.931093
H -7.598019 0.126616 -1.970745
H -8.945152 -0.285426 -0.901366
H -7.638669 -1.451713 -1.178819
SCF Energy (B3LYP/6-31G**)= -1361.69855976
Number of imaginary frequencies = 0

1a_c190
MMFF Geometry
C -1.726068 -1.969662 -0.505768
C -2.479261 0.620519 -0.174640
N -3.047756 -1.678841 -0.575239
C -0.712425 -1.033863 -0.275372
C -1.114609 0.303778 -0.106894
C -3.411227 -0.390090 -0.401564
C -0.142237 1.292011 0.128576
C 1.220796 0.969705 0.188669
C 1.618856 -0.357621 0.013562
C 0.658206 -1.358414 -0.204616
C 3.062195 -0.686265 0.095924
C 4.106480 0.448708 -0.065500
C 3.511594 1.793223 0.332474
C 2.197967 2.015180 0.471990
O 1.008855 -2.679592 -0.355166
O 3.372376 -1.871020 0.249807
O 4.455457 0.561956 -1.447461
C 5.335672 0.122717 0.808808
C 6.089350 -1.098702 0.320387
C 6.717643 -1.980713 1.364743
O 6.226880 -1.324333 -0.884006
O 4.388293 2.829684 0.634340
C 4.956839 3.394505 -0.549602
C -4.865772 -0.114187 -0.490146
C -5.551811 -0.823502 -1.624355
C -5.459576 0.691587 0.416811
C -6.916322 1.080078 0.566864
C -7.918833 -0.086052 0.665697
C -7.600015 -1.063412 1.790121
C -7.338456 2.091686 -0.501819
H -1.491621 -3.022548 -0.647878
H -2.811380 1.648300 -0.064846
H -0.455990 2.326447 0.265316
H 1.839928 2.993701 0.778505
H 1.974258 -2.768935 -0.187643
H 5.008824 -0.220327 -1.667585
H 5.025431 -0.043862 1.847579
H 6.070097 0.936486 0.801328
H 7.214677 -2.826583 0.881931
H 7.456916 -1.408051 1.929872
H 5.943964 -2.364495 2.034317
H 5.736774 2.743115 -0.954479
H 4.192071 3.599288 -1.306931
H 5.424498 4.344275 -0.273549
H -5.723224 -1.875734 -1.373814
H -6.512685 -0.376394 -1.883853
H -4.943989 -0.779607 -2.535773
H -4.838397 1.118297 1.205868
H -6.980546 1.613303 1.527118
H -8.919436 0.326571 0.848694
H -7.988684 -0.635965 -0.277621
H -7.532459 -0.546153 2.752456
H -8.390654 -1.816808 1.866864
H -6.657339 -1.589331 1.612255
H -6.645850 2.940197 -0.535489
H -8.335628 2.488485 -0.281453
H -7.374065 1.647374 -1.500735
SCF Energy (B3LYP/6-31G**)= -1361.70183467
Number of imaginary frequencies = 0

1a_c191
MMFF Geometry
C -1.398481 -2.172873 -0.470558
C -2.262755 0.275312 0.335971
N -2.712121 -2.029655 -0.169625
C -0.446646 -1.151319 -0.398028
C -0.905385 0.112489 0.023080
C -3.132213 -0.807802 0.222495
C 0.001249 1.185125 0.112283
C 1.355804 1.010522 -0.193468
C 1.807296 -0.251449 -0.596072
C 0.914471 -1.323077 -0.720052
C 3.246765 -0.405413 -0.907620
C 4.232528 0.591121 -0.243441
C 3.597134 1.977373 -0.215872
C 2.267888 2.154991 -0.186476
O 1.324428 -2.558610 -1.157989
O 3.600752 -1.329478 -1.640872
O 5.441946 0.647992 -1.004687
C 4.598923 0.120251 1.168890
C 4.809316 -1.375606 1.241501
C 6.114022 -1.932953 0.737889
O 3.917585 -2.106853 1.674554
O 4.401316 3.095448 -0.387421
C 5.331895 3.301596 0.671908
C -4.574777 -0.697346 0.546464
C -5.126185 -1.864511 1.320582
C -5.291528 0.369429 0.132135
C -6.772264 0.618968 0.332239
C -7.443998 0.824582 -1.039422
C -8.959042 0.954134 -0.959090
C -6.954961 1.825162 1.256072
H -1.117273 -3.176147 -0.784025
H -2.636380 1.235432 0.678131
H -0.359881 2.166519 0.417564
H 1.844197 3.155199 -0.209577
H 2.267320 -2.502437 -1.431660
H 5.205456 1.010972 -1.878101
H 3.804930 0.363311 1.887145
H 5.514394 0.595707 1.538228
H 6.214768 -2.972467 1.062586
H 6.140986 -1.896815 -0.352853
H 6.946580 -1.361153 1.155244
H 4.856689 3.183146 1.651649
H 6.188394 2.629275 0.573392
H 5.703074 4.327995 0.596609
H -4.425258 -2.178517 2.103011
H -6.061899 -1.629081 1.833678
H -5.307123 -2.716059 0.656228
H -4.790409 1.145165 -0.447576
H -7.249173 -0.243393 0.808090
H -7.037504 1.715890 -1.534276
H -7.209176 -0.029029 -1.688670
H -9.257205 1.866166 -0.434079
H -9.384973 0.999312 -1.966676
H -9.400505 0.095458 -0.443617
H -6.410307 1.684110 2.196772
H -8.008816 1.971705 1.512495
H -6.588586 2.747084 0.790537
SCF Energy (B3LYP/6-31G**)= -1361.69286592
Number of imaginary frequencies = 0

1a_c192
MMFF Geometry
C 1.638069 -2.282302 0.123542
C 2.543246 0.259323 -0.202500
N 2.978369 -2.084254 0.096315
C 0.677791 -1.273405 -0.004513
C 1.158633 0.037648 -0.175801
C 3.416795 -0.816366 -0.055887
C 0.242860 1.094595 -0.323243
C -1.140016 0.867297 -0.287462

C -1.615628 -0.433403 -0.106017
C -0.713615 -1.502149 0.021239
C -3.080520 -0.660859 -0.086464
C -4.028381 0.538771 0.172871
C -3.372266 1.848029 -0.245915
C -2.060170 1.982922 -0.479521
O -1.143416 -2.799823 0.171059
O -3.483036 -1.817786 -0.238196
O -4.265142 0.644924 1.578958
C -5.338784 0.316931 -0.611493
C -6.137342 -0.860859 -0.088466
C -6.901735 -1.674188 -1.097214
O -6.200145 -1.103605 1.118810
O -4.194542 2.948165 -0.463886
C -4.633521 3.524528 0.768626
C 4.889562 -0.642380 -0.084443
C 5.607063 -1.668600 -0.916428
C 5.466343 0.357716 0.616539
C 6.929420 0.717048 0.776217
C 7.567068 1.333607 -0.482750
C 6.852742 2.588812 -0.968963
C 7.775968 -0.434400 1.327325
H 1.341947 -3.320764 0.256380
H 2.937302 1.260196 -0.350025
H 0.617036 2.108015 -0.465253
H -1.658332 2.941171 -0.795645
H -2.122535 -2.818921 0.076174
H -4.853698 -0.102569 1.826949
H -5.118863 0.152784 -1.673441
H -6.012165 1.178496 -0.534579
H -7.419198 -2.494807 -0.592771
H -7.639313 -1.039818 -1.594596
H -6.208786 -2.094724 -1.830131
H -5.424556 2.918914 1.220321
H -3.802483 3.660609 1.469491
H -5.053467 4.509548 0.544232
H 5.087969 -1.827998 -1.868853
H 6.624400 -1.371171 -1.174394
H 5.660140 -2.626005 -0.387270
H 4.817844 1.011256 1.202343
H 6.957199 1.494993 1.553725
H 8.608574 1.598179 -0.260075
H 7.602672 0.607172 -1.301324
H 6.800118 3.342276 -0.176677
H 7.394920 3.024853 -1.814232
H 5.835307 2.367949 -1.305718
H 7.299881 -0.890359 2.202418
H 8.758429 -0.064648 1.642022
H 7.949825 -1.219760 0.587102
SCF Energy (B3LYP/6-31G**)= -1361.70185200
Number of imaginary frequencies = 0

1a_c193
MMFF Geometry
C -1.494846 -2.370185 -0.228047
C -2.216112 0.237531 0.016100
N -2.810456 -2.088349 -0.064642
C -0.472797 -1.416509 -0.283140
C -0.858339 -0.071249 -0.154562
C -3.157903 -0.788748 0.048691
C 0.124481 0.933359 -0.199084
C 1.483430 0.622685 -0.370146
C 1.865272 -0.716249 -0.493952
C 0.891557 -1.730919 -0.454125
C 3.299756 -1.049764 -0.672566
C 4.345530 0.087596 -0.688610
C 3.773959 1.490759 -0.562895
C 2.456819 1.715968 -0.418414
O 1.220455 -3.060861 -0.583851
O 3.605855 -2.236767 -0.826403
O 4.999257 0.009639 -1.974843
C 5.432349 -0.219740 0.351156
C 4.945268 -0.162536 1.782247
C 6.006505 -0.228249 2.849413
O 3.753055 -0.096940 2.078908
O 4.744256 2.476352 -0.620871
C 4.348624 3.836097 -0.483603
C -4.605236 -0.523806 0.233619
C -5.274049 -1.447802 1.213134
C -5.210212 0.463280 -0.462224
C -6.667123 0.883623 -0.485646
C -7.026499 1.716900 0.757839
C -8.414119 2.340302 0.687160
C -7.635185 -0.272741 -0.753342
H -1.272200 -3.431472 -0.316697
H -2.534732 1.268209 0.136907
H -0.180629 1.974157 -0.095386
H 2.055960 2.719013 -0.323954
H 2.196576 -3.130230 -0.695245
H 4.401527 0.409227 -2.628804
H 6.265923 0.485842 0.242898
H 5.853740 -1.219018 0.183039
H 5.540255 -0.175585 3.837170
H 6.552466 -1.171016 2.764997
H 6.690950 0.616340 2.738268
H 3.681188 4.133087 -1.298839
H 3.882027 4.011143 0.491112
H 5.248471 4.455470 -0.542478
H -4.628698 -1.632414 2.080088
H -6.200865 -1.034522 1.615224
H -5.502465 -2.409558 0.742016
H -4.603389 1.046280 -1.156675
H -6.760061 1.550497 -1.355658
H -6.964557 1.105773 1.665237
H -6.293329 2.525243 0.876616
H -9.197210 1.576393 0.695701
H -8.577309 2.991073 1.552313
H -8.527872 2.945610 -0.217607
H -7.252217 -0.937985 -1.535180
H -8.601424 0.108900 -1.101098
H -7.828739 -0.871013 0.140986
SCF Energy (B3LYP/6-31G**)= -1361.70094537
Number of imaginary frequencies = 0

1a_c194
MMFF Geometry
C -1.560009 -2.268283 -0.239036
C -2.180569 0.366928 -0.461258
N -2.870832 -1.923854 -0.251235
C -0.494598 -1.366791 -0.335366
C -0.828448 -0.006869 -0.453251
C -3.167375 -0.610674 -0.350984
C 0.198987 0.946751 -0.563491
C 1.552235 0.571102 -0.553932
C 1.882368 -0.781374 -0.428677
C 0.863869 -1.746276 -0.324270
C 3.310428 -1.183023 -0.415178
C 4.404037 -0.095549 -0.509429
C 3.887873 1.326277 -0.662154
C 2.574688 1.611990 -0.681381
O 1.142197 -3.089542 -0.215111
O 3.574799 -2.388149 -0.351002
O 5.166845 -0.399793 -1.698318
C 5.377592 -0.264303 0.665483
C 4.766720 0.037376 2.016181
C 5.723223 0.109783 3.177759
O 3.557058 0.188122 2.182521
O 4.903251 2.259415 -0.782500
C 4.559113 3.634806 -0.901426
C -4.612463 -0.274673 -0.362522
C -5.440997 -1.186981 -1.223897
C -5.073815 0.755120 0.380080
C -6.489260 1.266015 0.566585
C -7.445042 0.200135 1.135245
C -8.778857 0.775332 1.595115
C -7.015243 1.956738 -0.693962
H -1.379032 -3.337144 -0.146589
H -2.461931 1.410173 -0.565762
H -0.066569 1.999530 -0.654278
H 2.212964 2.628863 -0.785465
H 2.120231 -3.205620 -0.218725
H 4.649658 -0.092098 -2.461514

H 6.248702 0.388944 0.528202
H 5.766577 -1.289985 0.698111
H 5.172861 0.335195 4.095428
H 6.229662 -0.851327 3.295226
H 6.453828 0.903201 3.002111
H 3.981421 3.815644 -1.813486
H 4.015674 3.979933 -0.015997
H 5.487825 4.208716 -0.971678
H -6.430952 -0.784633 -1.442421
H -4.957314 -1.348036 -2.194464
H -5.575423 -2.159704 -0.738789
H -4.355263 1.308914 0.986654
H -6.411650 2.052155 1.332265
H -7.647612 -0.583166 0.398137
H -6.970181 -0.291695 1.993869
H -9.354937 1.172722 0.754386
H -9.380951 -0.007034 2.068482
H -8.631445 1.576603 2.325979
H -6.266065 2.639317 -1.110998
H -7.904419 2.554530 -0.468174
H -7.289769 1.241197 -1.473982
SCF Energy (B3LYP/6-31G**)= -1361.70127914
Number of imaginary frequencies = 0

1a_c195
MMFF Geometry
C -1.420579 -2.377015 -0.473946
C -2.382395 0.165600 -0.449404
N -2.763804 -2.205840 -0.417095
C -0.484616 -1.339460 -0.522302
C -0.994519 -0.026142 -0.511322
C -3.230358 -0.938988 -0.395166
C -0.106170 1.063405 -0.575981
C 1.277889 0.862086 -0.627340
C 1.778133 -0.444895 -0.618626
C 0.908397 -1.541985 -0.588714
C 3.247140 -0.624816 -0.669151
C 4.128546 0.542214 -0.152289
C 3.502326 1.866274 -0.577607
C 2.184149 2.000061 -0.786921
O 1.372683 -2.833941 -0.630189
O 3.701373 -1.693948 -1.078424
O 5.437315 0.442131 -0.720227
C 4.280395 0.464019 1.371331
C 4.471101 -0.954518 1.859664
C 5.833352 -1.577991 1.711136
O 3.521935 -1.579427 2.335050
O 4.328069 2.929101 -0.916404
C 5.093186 3.432722 0.175198
C -4.704766 -0.797821 -0.335985
C -5.457421 -1.765628 -1.209221
C -5.276506 0.124855 0.467251
C -6.756726 0.362065 0.676358
C -7.248719 1.607712 -0.085837
C -6.625812 2.930564 0.343714
C -7.070396 0.437038 2.173991
H -1.101193 -3.417137 -0.480122
H -2.799371 1.167896 -0.458561
H -0.503942 2.077447 -0.591772
H 1.773081 2.945459 -1.130034
H 2.346236 -2.817915 -0.768179
H 5.333959 0.559610 -1.682543
H 3.390184 0.856747 1.879938
H 5.133445 1.049525 1.731946
H 5.880428 -2.494629 2.305883
H 6.021189 -1.823307 0.664097
H 6.597725 -0.889882 2.080640
H 4.478175 3.554575 1.073338
H 5.951822 2.787259 0.378754
H 5.476019 4.417092 -0.110096
H -5.501639 -2.754031 -0.739916
H -6.480726 -1.440141 -1.414757
H -4.971505 -1.867214 -2.186740
H -4.633050 0.761127 1.074788
H -7.327371 -0.491742 0.293737
H -7.062181 1.468588 -1.158560
H -8.337350 1.685475 0.028221
H -5.540200 2.928737 0.209079
H -7.032637 3.746180 -0.263316
H -6.846962 3.157831 1.390458
H -6.821464 -0.509133 2.668109
H -8.136676 0.626464 2.337513
H -6.504149 1.228527 2.676344
SCF Energy (B3LYP/6-31G**)= -1361.69180308
Number of imaginary frequencies = 0

1a_c196
MMFF Geometry
C -2.011458 -1.529781 -0.729594
C -2.195338 1.144744 -0.295281
N -3.246211 -0.981007 -0.632762
C -0.813389 -0.820085 -0.621376
C -0.920566 0.564270 -0.396714
C -3.335801 0.351762 -0.415983
C 0.249694 1.337383 -0.283685
C 1.518658 0.751015 -0.378884
C 1.618768 -0.627581 -0.588516
C 0.463038 -1.409818 -0.725144
C 2.967052 -1.235062 -0.669770
C 4.181762 -0.445471 -0.140883
C 3.938419 1.045096 -0.245655
C 2.713983 1.588760 -0.309547
O 0.528175 -2.762618 -0.958724
O 3.087822 -2.380598 -1.116284
O 5.283032 -0.817995 -0.984161
C 4.447763 -0.945452 1.282592
C 5.470386 -0.138661 2.051852
C 6.911891 -0.224461 1.621577
O 5.133025 0.534105 3.028169
O 5.055174 1.861013 -0.154475
C 5.464473 2.330371 -1.439470
C -4.713661 0.919974 -0.330379
C -4.871644 2.371152 -0.696949
C -5.735397 0.118205 0.044953
C -7.197346 0.468971 0.212256
C -7.652190 0.348993 1.681752
C -7.614905 -1.052813 2.278236
C -8.048095 -0.384532 -0.731445
H -2.012100 -2.603593 -0.903968
H -2.285708 2.209906 -0.109070
H 0.165521 2.411543 -0.120164
H 2.593426 2.668362 -0.312318
H 1.463529 -3.011051 -1.132947
H 5.141024 -1.754579 -1.230054
H 4.797112 -1.985950 1.273925
H 3.521052 -0.936659 1.872101
H 7.545321 0.246392 2.378971
H 7.206301 -1.273091 1.531090
H 7.061390 0.291397 0.672145
H 5.667543 1.501356 -2.124719
H 4.710655 2.998877 -1.868528
H 6.389400 2.899772 -1.309479
H -4.286326 2.613848 -1.591434
H -5.901306 2.645109 -0.937180
H -4.538996 3.013728 0.124961
H -5.506722 -0.925748 0.268300
H -7.371989 1.511959 -0.068908
H -7.033187 1.008449 2.303765
H -8.679249 0.727422 1.762672
H -6.602447 -1.466236 2.279492
H -7.958758 -1.021787 3.317552
H -8.271665 -1.738649 1.735571
H -7.794090 -0.173567 -1.776594
H -9.113081 -0.166349 -0.598317
H -7.897559 -1.456939 -0.567388
SCF Energy (B3LYP/6-31G**)= -1361.70163421
Number of imaginary frequencies = 0

1a_c197
MMFF Geometry
C -1.697784 -2.084515 -0.117974
C -2.175527 0.547821 0.360004

N -2.970601 -1.711002 0.158883
C -0.603851 -1.214688 -0.178895
C -0.864074 0.144950 0.067538
C -3.194511 -0.400620 0.400934
C 0.193762 1.070147 0.011763
C 1.506206 0.664879 -0.278172
C 1.762038 -0.689183 -0.513887
C 0.713288 -1.624033 -0.472495
C 3.145659 -1.116325 -0.823189
C 4.289720 -0.090854 -0.758230
C 3.852831 1.345945 -0.548202
C 2.566932 1.675101 -0.331539
O 0.921183 -2.961566 -0.719052
O 3.353399 -2.285932 -1.167499
O 4.896411 -0.162316 -2.070853
C 5.352449 -0.549662 0.248493
C 4.913554 -0.379948 1.684605
C 5.993670 -0.093012 2.693224
O 3.740865 -0.522179 2.029503
O 4.907518 2.243504 -0.578475
C 4.616636 3.634887 -0.529250
C -4.594855 -0.019496 0.708338
C -4.797390 0.859716 1.906869
C -5.565366 -0.469382 -0.116054
C -7.069735 -0.285633 -0.122719
C -7.508304 0.608363 -1.302551
C -7.041250 2.056762 -1.221387
C -7.742697 0.138366 1.183372
H -1.573571 -3.150155 -0.298309
H -2.402310 1.594028 0.539911
H -0.014156 2.122647 0.201086
H 2.260360 2.702009 -0.168582
H 1.869961 -3.093613 -0.941893
H 5.003268 -1.111622 -2.273403
H 6.277837 0.019617 0.092855
H 5.618205 -1.603569 0.101373
H 5.551688 0.007352 3.688386
H 6.711722 -0.916546 2.704406
H 6.495290 0.843318 2.436375
H 3.969810 3.931023 -1.361436
H 4.169571 3.905680 0.432513
H 5.560721 4.179183 -0.625721
H -5.225851 1.822811 1.612093
H -5.449939 0.376449 2.639559
H -3.863160 1.071574 2.438168
H -5.239096 -1.093334 -0.954212
H -7.472883 -1.287171 -0.334699
H -7.137936 0.177100 -2.241741
H -8.603275 0.597648 -1.373708
H -5.949976 2.128162 -1.195257
H -7.387759 2.608004 -2.101839
H -7.443558 2.560737 -0.338159
H -7.485173 -0.548634 1.996384
H -8.832936 0.111236 1.073519
H -7.476053 1.153498 1.487590
SCF Energy (B3LYP/6-31G**)= -1361.69883488
Number of imaginary frequencies = 0

1a_c198
MMFF Geometry
C 1.876579 2.240351 -0.048897
C 2.343407 -0.392367 -0.538285
N 3.163980 1.820660 -0.091149
C 0.761558 1.417593 -0.237834
C 1.015615 0.057045 -0.493532
C 3.384292 0.509248 -0.325650
C -0.064011 -0.819813 -0.706375
C -1.389319 -0.366780 -0.651526
C -1.634733 0.981445 -0.382172
C -0.570705 1.875168 -0.191793
C -3.038271 1.438391 -0.309314
C -4.155152 0.394599 -0.119888
C -3.771462 -0.952686 -0.712915
C -2.487947 -1.293848 -0.931822
O -0.782868 3.211984 0.046527
O -3.282485 2.648674 -0.348072
O -5.297646 0.921968 -0.814619
C -4.467276 0.346869 1.380076
C -5.386262 -0.785375 1.782122
C -6.848193 -0.665584 1.437753
O -4.948478 -1.765239 2.389200
O -4.858398 -1.769177 -0.968342
C -4.630822 -3.097810 -1.420579
C 4.807083 0.092831 -0.366770
C 5.696072 1.036122 -1.128397
C 5.202866 -1.031592 0.268424
C 6.586784 -1.631010 0.414159
C 7.668884 -0.697785 0.991046
C 7.295873 -0.103344 2.343343
C 7.051449 -2.288795 -0.887776
H 1.759162 3.303633 0.149496
H 2.565097 -1.433881 -0.750365
H 0.137715 -1.869601 -0.917926
H -2.212109 -2.261667 -1.336078
H -1.738020 3.409599 -0.079983
H -5.235388 1.896505 -0.761116
H -4.932760 1.282248 1.716061
H -3.544235 0.235708 1.964727
H -7.408153 -1.451310 1.953078
H -7.227050 0.302933 1.773706
H -6.998226 -0.775834 0.362703
H -4.139760 -3.098615 -2.398841
H -4.049235 -3.666520 -0.687909
H -5.603307 -3.586449 -1.531456
H 5.927692 1.919962 -0.524637
H 6.638221 0.577403 -1.432454
H 5.210497 1.367159 -2.053970
H 4.448757 -1.606810 0.807968
H 6.477226 -2.449231 1.141521
H 8.595890 -1.271980 1.117186
H 7.910124 0.113436 0.297607
H 7.057364 -0.890376 3.065773
H 8.134454 0.477107 2.741419
H 6.435606 0.568019 2.265389
H 6.295470 -2.984878 -1.268263
H 7.971614 -2.859802 -0.721615
H 7.256623 -1.554275 -1.671929
SCF Energy (B3LYP/6-31G**)= -1361.70328245
Number of imaginary frequencies = 0

1a_c199
MMFF Geometry
C -1.719001 -2.027649 -0.354659
C -2.209175 0.521972 0.448919
N -2.997103 -1.691743 -0.054827
C -0.625425 -1.158125 -0.284485
C -0.892347 0.159146 0.129862
C -3.227225 -0.423145 0.349863
C 0.164153 1.083492 0.211906
C 1.480476 0.717092 -0.106297
C 1.745060 -0.596563 -0.508572
C 0.697392 -1.528045 -0.605117
C 3.136384 -0.981299 -0.849805
C 4.281728 0.028972 -0.647496
C 3.817870 1.420908 -0.279225
C 2.540898 1.718540 -0.007195
O 0.911273 -2.823025 -1.017556
O 3.352089 -2.096353 -1.339904
O 4.911503 0.094219 -1.947478
C 5.320187 -0.532027 0.330086
C 4.832129 -0.513711 1.759981
C 5.818684 -0.087864 2.813555
O 3.692714 -0.872488 2.056202
O 4.786601 2.388648 -0.041161
C 5.370595 2.876600 -1.249277
C -4.633324 -0.083792 0.679468
C -4.857536 0.636353 1.976294
C -5.589115 -0.425196 -0.211519
C -7.093465 -0.241392 -0.220320
C -7.513554 0.794624 -1.285125
C -7.050380 2.220966 -1.013814
C -7.789107 0.014736 1.117210

H -1.590100 -3.061557 -0.667601
H -2.440346 1.536606 0.757977
H -0.046390 2.103474 0.531825
H 2.277037 2.719838 0.320957
H 1.865556 -2.929633 -1.229440
H 4.993708 -0.830214 -2.256460
H 6.247699 0.050446 0.260645
H 5.596187 -1.563998 0.081689
H 5.342101 -0.115107 3.797345
H 6.671845 -0.770538 2.812882
H 6.150927 0.933676 2.612644
H 6.180632 2.217362 -1.573647
H 4.627473 3.001285 -2.044739
H 5.806241 3.857327 -1.035771
H -5.281154 1.629859 1.798752
H -5.522739 0.065095 2.630014
H -3.933031 0.777183 2.546733
H -5.247700 -0.937875 -1.116410
H -7.491323 -1.207957 -0.563832
H -7.126649 0.485233 -2.264831
H -8.607148 0.793483 -1.375642
H -5.959827 2.287985 -0.960305
H -7.382852 2.879126 -1.823394
H -7.468443 2.609578 -0.081001
H -7.544572 -0.769731 1.841150
H -8.877284 0.002635 0.986259
H -7.528981 0.983063 1.551750
SCF Energy (B3LYP/6-31G**)= -1361.69320974
Number of imaginary frequencies = 0

1a_c200
MMFF Geometry
C -1.555043 -1.858569 -0.953945
C -2.259931 0.690330 -0.324924
N -2.875739 -1.568104 -0.864471
C -0.519833 -0.941229 -0.747945
C -0.896696 0.376400 -0.425533
C -3.214198 -0.301829 -0.541306
C 0.098078 1.350840 -0.222108
C 1.458641 1.028709 -0.316537
C 1.825194 -0.286312 -0.618392
C 0.847924 -1.263707 -0.852978
C 3.265937 -0.603927 -0.713945
C 4.260238 0.331140 0.014191
C 3.775173 1.778900 -0.028261
C 2.470087 2.080135 -0.165582
O 1.180340 -2.551924 -1.195931
O 3.606405 -1.607208 -1.343044
O 5.532328 0.249226 -0.642223
C 4.472578 -0.096033 1.468292
C 4.580998 -1.595865 1.615983
C 5.890146 -2.250576 1.264525
O 3.606519 -2.255114 1.982856
O 4.794473 2.702577 0.110764
C 4.467716 4.087419 0.118007
C -4.668994 -0.026207 -0.455888
C -5.470884 -0.637035 -1.571253
C -5.163157 0.693293 0.574923
C -6.595291 1.058700 0.908133
C -7.586033 -0.117427 1.007129
C -7.155956 -1.187722 2.002473
C -7.122300 2.157667 -0.018240
H -1.340091 -2.894709 -1.207315
H -2.576273 1.702757 -0.093775
H -0.199709 2.372682 0.010313
H 2.109652 3.102998 -0.176187
H 2.155898 -2.608201 -1.312177
H 5.443829 0.713287 -1.492872
H 3.634650 0.222599 2.102501
H 5.378955 0.352136 1.893792
H 5.902220 -3.271797 1.655867
H 6.013444 -2.283057 0.180302
H 6.715239 -1.699025 1.722034
H 4.011736 4.386130 -0.831305
H 3.813809 4.330079 0.961742
H 5.397149 4.651754 0.238226
H -6.451865 -0.174311 -1.689938
H -4.960194 -0.509786 -2.533051
H -5.619177 -1.708162 -1.397923
H -4.462528 1.050545 1.331280
H -6.558148 1.505303 1.912976
H -8.561030 0.271716 1.327909
H -7.754726 -0.582925 0.031426
H -6.987666 -0.756433 2.994379
H -7.936888 -1.949550 2.093941
H -6.238414 -1.690506 1.682798
H -6.434046 3.009835 -0.048661
H -8.089988 2.527872 0.337981
H -7.262555 1.802471 -1.043263
SCF Energy (B3LYP/6-31G**)= -1361.69465062
Number of imaginary frequencies = 0

1a_c201
MMFF Geometry
C -1.658977 -1.422757 -0.531862
C -2.057341 1.176915 0.147890
N -2.927781 -1.011363 -0.292298
C -0.529043 -0.605537 -0.456642
C -0.746960 0.740070 -0.104801
C -3.123111 0.283348 0.048205
C 0.348301 1.620742 -0.025983
C 1.652092 1.173844 -0.270123
C 1.862033 -0.169446 -0.601149
C 0.781920 -1.052842 -0.716628
C 3.252957 -0.612967 -0.848983
C 4.392914 0.206451 -0.189399
C 4.038152 1.688789 -0.244930
C 2.768393 2.120203 -0.275209
O 0.956376 -2.363482 -1.088240
O 3.442894 -1.622676 -1.528147
O 5.612658 -0.007833 -0.904763
C 4.618480 -0.256526 1.254689
C 4.531357 -1.759429 1.398727
C 5.716892 -2.582320 0.969936
O 3.501910 -2.282424 1.827826
O 5.048791 2.621563 -0.431463
C 5.970348 2.694981 0.652954
C -4.531412 0.693605 0.320282
C -4.734349 1.858686 1.250990
C -5.545141 -0.001702 -0.241545
C -7.032285 0.252276 -0.118753
C -7.667555 -0.850939 0.746319
C -9.144805 -0.620426 1.033996
C -7.647940 0.320843 -1.520033
H -1.571373 -2.474331 -0.796843
H -2.236281 2.215346 0.406347
H 0.176206 2.667216 0.223047
H 2.547930 3.180938 -0.357048
H 1.900384 -2.504148 -1.325146
H 5.476952 0.349411 -1.801702
H 3.865995 0.170219 1.930747
H 5.597601 0.050716 1.638727
H 5.603245 -3.604704 1.341393
H 5.783159 -2.605034 -0.119487
H 6.632018 -2.162742 1.395133
H 5.452757 2.717277 1.618014
H 6.682575 1.866377 0.614817
H 6.535742 3.625879 0.549518
H -4.568024 2.804852 0.725679
H -5.737268 1.886456 1.684986
H -4.046882 1.804096 2.102985
H -5.295679 -0.854709 -0.876634
H -7.218944 1.220835 0.356098
H -7.547372 -1.829986 0.265109
H -7.139124 -0.907178 1.706986
H -9.747046 -0.688363 0.123463
H -9.513677 -1.380052 1.730835
H -9.308413 0.362270 1.487307
H -7.125029 1.056480 -2.142026
H -8.698748 0.623963 -1.475838
H -7.596772 -0.647890 -2.029771
SCF Energy (B3LYP/6-31G**)= -1361.69371767

Number of imaginary frequencies = 0

1a_c202
MMFF Geometry
C -1.454007 -2.230464 -0.510419
C -2.325573 0.290416 0.014083
N -2.783725 -2.042983 -0.327825
C -0.489259 -1.219864 -0.450181
C -0.951910 0.081351 -0.176384
C -3.206147 -0.785987 -0.075318
C -0.031006 1.143194 -0.106311
C 1.341086 0.926249 -0.290960
C 1.794382 -0.371278 -0.547306
C 0.889138 -1.437143 -0.646407
C 3.245656 -0.577473 -0.739539
C 4.208639 0.477844 -0.146178
C 3.600616 1.876532 -0.230403
C 2.268063 2.062866 -0.279873
O 1.303649 -2.713954 -0.938845
O 3.622017 -1.587405 -1.336728
O 5.430451 0.457744 -0.896209
C 4.564524 0.160190 1.307943
C 4.810565 -1.314034 1.529822
C 6.140687 -1.881997 1.112405
O 3.925661 -2.025424 2.009094
O 4.545364 2.885934 -0.224519
C 4.103674 4.237854 -0.267174
C -4.666773 -0.625053 0.126189
C -5.280311 -1.686582 0.996356
C -5.323716 0.394706 -0.467737
C -6.797107 0.746788 -0.446850
C -7.289952 1.312622 0.898021
C -6.530563 2.555839 1.345172
C -7.695753 -0.391530 -0.939669
H -1.170360 -3.261087 -0.713783
H -2.705299 1.281241 0.243590
H -0.396316 2.150227 0.091894
H 1.822326 3.050837 -0.318489
H 2.268555 -2.698556 -1.131470
H 5.239495 0.859223 -1.761735
H 3.752325 0.449258 1.988120
H 5.458851 0.705243 1.634318
H 6.268548 -2.872714 1.557735
H 6.185083 -1.971630 0.025302
H 6.947806 -1.238649 1.471519
H 3.554066 4.440209 -1.192009
H 3.496654 4.478404 0.611479
H 4.988224 4.881387 -0.252668
H -4.655140 -1.875934 1.876861
H -6.262704 -1.406997 1.379402
H -5.389592 -2.624683 0.441855
H -4.748903 1.074016 -1.099127
H -6.916783 1.551660 -1.187357
H -8.351230 1.575956 0.804963
H -7.228780 0.557228 1.688386
H -6.571944 3.337454 0.579954
H -6.974880 2.956822 2.261886
H -5.480401 2.331912 1.555826
H -7.320346 -0.811997 -1.879148
H -8.709281 -0.019224 -1.127100
H -7.780748 -1.204166 -0.213486
SCF Energy (B3LYP/6-31G**)= -1361.69469096
Number of imaginary frequencies = 0

1a_c203
MMFF Geometry
C -1.799092 -2.440244 0.144217
C -2.408762 0.202601 -0.035189
N -3.098856 -2.078530 0.267358
C -0.739415 -1.552473 -0.067811
C -1.066957 -0.186581 -0.157562
C -3.391579 -0.764172 0.167925
C -0.045339 0.756857 -0.372029
C 1.294919 0.362726 -0.486460
C 1.614295 -0.993071 -0.385013
C 0.607050 -1.949895 -0.192250
C 3.034106 -1.389527 -0.489795
C 4.125273 -0.319822 -0.295236
C 3.637140 1.061932 -0.703243
C 2.326902 1.363716 -0.765757
O 0.890415 -3.292430 -0.113513
O 3.317478 -2.577075 -0.677911
O 5.213022 -0.719839 -1.145455
C 4.579980 -0.411570 1.165735
C 5.493568 0.713759 1.598364
C 6.918037 0.696356 1.107677
O 5.083221 1.605147 2.344936
O 4.663808 1.950088 -0.968705
C 4.345375 3.306515 -1.252043
C -4.824427 -0.409926 0.313548
C -5.518069 -1.131461 1.435398
C -5.392765 0.473424 -0.535514
C -6.823838 0.964439 -0.615453
C -7.226887 1.917299 0.525313
C -6.339244 3.152087 0.623260
C -7.840878 -0.168118 -0.786171
H -1.623232 -3.510830 0.225727
H -2.684280 1.251730 -0.084060
H -0.304815 1.812224 -0.451886
H 1.978295 2.354131 -1.037288
H 1.835080 -3.432029 -0.349102
H 5.192381 -1.696620 -1.189417
H 5.108438 -1.354485 1.355861
H 3.714808 -0.402424 1.842178
H 7.498137 1.448411 1.650246
H 7.361393 -0.283888 1.299986
H 6.958179 0.924756 0.041554
H 3.762602 3.386192 -2.175170
H 3.816759 3.768284 -0.411915
H 5.284165 3.848739 -1.398518
H -4.893769 -1.139202 2.336603
H -6.459111 -0.662063 1.725372
H -5.731523 -2.168120 1.153933
H -4.769672 0.897795 -1.324400
H -6.881883 1.548596 -1.545982
H -8.259276 2.251224 0.361047
H -7.221002 1.399806 1.490303
H -6.322274 3.699462 -0.324520
H -6.720310 3.827131 1.396352
H -5.311266 2.888618 0.890102
H -7.530774 -0.861052 -1.576121
H -8.817328 0.240064 -1.070626
H -7.987636 -0.742962 0.131956
SCF Energy (B3LYP/6-31G**)= -1361.70329942
Number of imaginary frequencies = 0

1a_c204
MMFF Geometry
C 1.875130 2.464627 -0.040749
C 2.582312 -0.153380 -0.207002
N 3.196161 2.162979 -0.052241
C 0.839807 1.526272 -0.106595
C 1.218636 0.175288 -0.194341
C 3.536601 0.858644 -0.126941
C 0.223633 -0.814876 -0.270279
C -1.139512 -0.482302 -0.255090
C -1.516382 0.860638 -0.163812
C -0.530741 1.861080 -0.093743
C -2.958790 1.211964 -0.139662
C -4.023973 0.094790 -0.156083
C -3.443802 -1.292218 -0.326227
C -2.130530 -1.549983 -0.337526
O -0.853404 3.195582 -0.008299
O -3.266949 2.408405 -0.099201
O -4.810087 0.397344 -1.314007
C -4.820113 0.224243 1.154964
C -6.203760 -0.376122 1.079283
C -6.512615 -1.533398 1.990975
O -7.064580 0.109862 0.343693
O -4.323171 -2.367587 -0.337403
C -4.994587 -2.501075 -1.592683
C 4.991006 0.572701 -0.143440

C 5.796815 1.512716 -0.999235
C 5.498244 -0.443344 0.587137
C 6.950511 -0.849410 0.717526
C 7.192752 -2.262591 0.155791
C 6.950024 -2.361918 -1.345156
C 7.357653 -0.794983 2.193703
H 1.660203 3.529198 0.025007
H 2.897284 -1.188793 -0.293209
H 0.520995 -1.860852 -0.343036
H -1.782256 -2.576563 -0.412600
H -1.832435 3.287184 -0.058339
H -5.447366 1.095137 -1.065267
H -4.978457 1.276717 1.425501
H -4.268839 -0.215756 1.995294
H -7.459604 -1.991923 1.693273
H -5.727237 -2.289061 1.924175
H -6.593259 -1.171922 3.018960
H -5.867131 -1.843624 -1.632903
H -4.325181 -2.310275 -2.438826
H -5.352446 -3.532207 -1.669075
H 5.946180 2.468458 -0.485902
H 6.779021 1.112400 -1.261892
H 5.286582 1.706263 -1.950155
H 4.819481 -1.043944 1.193248
H 7.604780 -0.152910 0.184139
H 8.231027 -2.556257 0.354982
H 6.555653 -2.993123 0.670325
H 7.565886 -1.639657 -1.890383
H 7.209141 -3.364204 -1.701216
H 5.900486 -2.181585 -1.596487
H 7.207022 0.209386 2.605298
H 8.417144 -1.046859 2.312212
H 6.775813 -1.499102 2.799418
SCF Energy (B3LYP/6-31G**)= -1361.70339972
Number of imaginary frequencies = 0

1a_c205
MMFF Geometry
C 1.785985 -1.842446 0.083909
C 2.422425 0.732516 -0.498688
N 3.097012 -1.517374 -0.023954
C 0.725931 -0.947768 -0.082734
C 1.067453 0.382400 -0.383739
C 3.410343 -0.233665 -0.314132
C 0.045104 1.330917 -0.566772
C -1.305824 0.976538 -0.443867
C -1.641913 -0.343725 -0.136472
C -0.633450 -1.306272 0.030843
C -3.074737 -0.707958 -0.025632
C -4.125776 0.412918 0.183463
C -3.620766 1.739447 -0.369144
C -2.339395 1.979227 -0.677284
O -0.924934 -2.621491 0.306989
O -3.366008 -1.906799 -0.063766
O -4.314581 0.613046 1.586559
C -5.438363 0.001743 -0.516292
C -6.094105 -1.199096 0.136731
C -6.813401 -2.163956 -0.766059
O -6.084082 -1.344386 1.361136
O -4.556452 2.734055 -0.632287
C -5.000110 3.365616 0.571168
C 4.862746 0.084202 -0.440166
C 5.223962 1.197386 -1.383347
C 5.757313 -0.648213 0.260384
C 7.264734 -0.542236 0.354028
C 7.828424 0.848702 0.704369
C 7.260927 1.423038 1.996376
C 7.941810 -1.122357 -0.890351
H 1.602463 -2.888753 0.318658
H 2.695704 1.759632 -0.717237
H 0.309820 2.360200 -0.806950
H -2.048299 2.941912 -1.087313
H -1.900273 -2.742784 0.262106
H -4.815367 -0.163994 1.921325
H -5.246436 -0.228970 -1.571271
H -6.190344 0.798305 -0.475492
H -7.225964 -2.985128 -0.173509
H -7.629795 -1.647386 -1.276443
H -6.111872 -2.576157 -1.495541
H -5.708658 2.726764 1.106293
H -4.158991 3.637445 1.218454
H -5.523951 4.284354 0.291016
H 5.075311 2.171410 -0.905803
H 6.259101 1.143683 -1.724608
H 4.613035 1.156149 -2.292540
H 5.365264 -1.452294 0.888566
H 7.553359 -1.199336 1.188010
H 8.916928 0.766037 0.820056
H 7.667178 1.564216 -0.107385
H 7.415839 0.734711 2.833289
H 7.761299 2.366257 2.238410
H 6.189924 1.628778 1.910423
H 7.572989 -2.131805 -1.104372
H 9.024908 -1.191866 -0.740683
H 7.770301 -0.506918 -1.778365
SCF Energy (B3LYP/6-31G**)= -1361.70212774
Number of imaginary frequencies = 0

1a_c206
MMFF Geometry
C -1.703725 -1.788925 -0.412131
C -2.055407 0.886202 -0.113342
N -2.969328 -1.322783 -0.278964
C -0.555118 -0.992901 -0.409876
C -0.749460 0.389242 -0.254559
C -3.140070 0.010606 -0.129563
C 0.368296 1.242772 -0.244244
C 1.673899 0.744451 -0.385504
C 1.864443 -0.632074 -0.535129
C 0.754933 -1.496634 -0.550976
C 3.241466 -1.163981 -0.682267
C 4.438941 -0.188506 -0.637806
C 4.069858 1.278939 -0.490093
C 2.794452 1.687530 -0.376577
O 0.893969 -2.856567 -0.708739
O 3.379591 -2.378782 -0.859723
O 5.115454 -0.329978 -1.906718
C 5.437052 -0.672531 0.423451
C 4.917553 -0.579800 1.841226
C 5.922972 -0.826259 2.935659
O 3.738733 -0.347309 2.105802
O 5.172297 2.116096 -0.493960
C 4.970870 3.514986 -0.330347
C -4.546383 0.490802 0.000510
C -4.835186 1.867388 -0.529500
C -5.473435 -0.331286 0.540955
C -6.945317 -0.109000 0.824566
C -7.788611 -0.283023 -0.451408
C -9.288878 -0.276101 -0.190234
C -7.239395 1.188014 1.584634
H -1.634294 -2.868633 -0.526098
H -2.214513 1.950788 0.023084
H 0.212419 2.313986 -0.120582
H 2.538223 2.735302 -0.266138
H 1.853173 -3.063242 -0.793671
H 4.601874 0.165873 -2.566243
H 6.365686 -0.091296 0.358573
H 5.716892 -1.717651 0.239567
H 5.437756 -0.730160 3.910931
H 6.328337 -1.836428 2.839482
H 6.726280 -0.088477 2.868422
H 4.378828 3.923673 -1.155417
H 4.503476 3.732489 0.635397
H 5.951384 3.999887 -0.346935
H -4.282563 2.056772 -1.457054
H -5.887300 2.007535 -0.784720
H -4.557050 2.630308 0.204856
H -5.142441 -1.325341 0.852818
H -7.230869 -0.919796 1.511150
H -7.557130 0.503583 -1.178466
H -7.528666 -1.235803 -0.930686
H -9.627977 0.699268 0.171073

H -9.831638 -0.492969 -1.115977
H -9.563075 -1.035730 0.548497
H -6.502235 1.360092 2.376729
H -8.221965 1.134913 2.066379
H -7.250167 2.063374 0.929801
SCF Energy (B3LYP/6-31G**)= -1361.70129430
Number of imaginary frequencies = 0

1a_c207
MMFF Geometry
C -1.638748 -1.312304 -0.985095
C -2.071197 1.233514 -0.141814
N -2.919605 -0.903212 -0.816193
C -0.512503 -0.518603 -0.757383
C -0.747955 0.798549 -0.319609
C -3.132109 0.365112 -0.395945
C 0.344153 1.653585 -0.079616
C 1.660023 1.208193 -0.250801
C 1.885735 -0.107447 -0.670602
C 0.811800 -0.963075 -0.944289
C 3.288748 -0.550403 -0.838258
C 4.376563 0.196642 -0.023062
C 4.039845 1.683809 0.012344
C 2.780631 2.135176 -0.087508
O 1.005851 -2.242994 -1.403017
O 3.526191 -1.505325 -1.578925
O 5.649849 0.022093 -0.650517
C 4.477169 -0.383627 1.392489
C 4.367411 -1.891897 1.406484
C 5.578690 -2.694245 1.012085
O 3.301878 -2.433239 1.704813
O 5.070171 2.613319 -0.014328
C 5.898453 2.586386 1.144923
C -4.555633 0.782209 -0.236340
C -4.856431 2.249088 -0.385782
C -5.498129 -0.156969 0.001137
C -6.983363 0.040552 0.216598
C -7.399893 -0.632025 1.539489
C -8.852908 -0.378056 1.917550
C -7.743614 -0.522037 -0.986158
H -1.537931 -2.341598 -1.322863
H -2.261897 2.243263 0.206310
H 0.160538 2.679296 0.238092
H 2.576522 3.202228 -0.101108
H 1.964989 -2.378256 -1.572760
H 5.592233 0.451775 -1.523776
H 3.673899 -0.001934 2.036274
H 5.422953 -0.121910 1.879877
H 5.427384 -3.741241 1.289703
H 5.735112 -2.631285 -0.066489
H 6.458239 -2.322296 1.543444
H 5.302233 2.539405 2.062558
H 6.604564 1.752952 1.099060
H 6.478117 3.514053 1.163967
H -4.289219 2.682627 -1.217545
H -5.907027 2.447303 -0.612945
H -4.602332 2.789739 0.531825
H -5.181150 -1.200601 0.062572
H -7.222747 1.105209 0.300558
H -7.231258 -1.715255 1.487157
H -6.765580 -0.254881 2.352350
H -9.540726 -0.849858 1.209988
H -9.060659 -0.795874 2.907983
H -9.069071 0.694363 1.950903
H -7.382060 -0.078037 -1.920816
H -8.813368 -0.301380 -0.917205
H -7.626486 -1.608896 -1.062986
SCF Energy (B3LYP/6-31G**)= -1361.69378190
Number of imaginary frequencies = 0

1a_c208
MMFF Geometry
C -1.924059 2.279568 -0.054081
C -2.514010 -0.336235 -0.491141
N -3.220303 1.947862 -0.268437
C -0.858123 1.373875 -0.042381
C -1.176001 0.023883 -0.272110
C -3.503934 0.644066 -0.474827
C -0.147034 -0.933949 -0.278052
C 1.190297 -0.569770 -0.059132
C 1.506573 0.772543 0.168891
C 0.486165 1.739979 0.180187
C 2.922602 1.159006 0.392648
C 4.034080 0.090565 0.314898
C 3.509317 -1.316375 0.129222
C 2.218213 -1.605167 -0.073003
O 0.748881 3.071951 0.401860
O 3.172368 2.345900 0.631336
O 4.664972 0.168265 1.597952
C 4.968543 0.508161 -0.834839
C 6.362917 -0.058569 -0.711651
C 6.833447 -0.994172 -1.792904
O 7.107182 0.287349 0.207423
O 4.435021 -2.347534 0.031137
C 4.963589 -2.719675 1.306469
C -4.933101 0.325212 -0.705883
C -5.652802 1.308624 -1.589132
C -5.498583 -0.752204 -0.120521
C -6.943577 -1.194476 -0.205663
C -7.581311 -1.309130 1.192581
C -7.674339 0.026834 1.919651
C -7.014075 -2.542601 -0.927479
H -1.756287 3.341262 0.114684
H -2.779648 -1.370690 -0.686056
H -0.397338 -1.979883 -0.454564
H 1.916178 -2.633869 -0.250048
H 1.709469 3.176725 0.590784
H 5.290959 0.918292 1.575265
H 5.103416 1.597558 -0.866228
H 4.540917 0.236533 -1.807985
H 7.761922 -1.479245 -1.479379
H 6.085152 -1.767706 -1.977319
H 7.013577 -0.426266 -2.708851
H 5.790953 -2.061875 1.585681
H 4.192439 -2.725587 2.084775
H 5.362532 -3.734507 1.216518
H -5.027544 1.594486 -2.443170
H -6.573685 0.902330 -2.014553
H -5.907951 2.214133 -1.028549
H -4.886420 -1.379639 0.527910
H -7.541632 -0.481611 -0.781663
H -8.595199 -1.716771 1.093457
H -7.017961 -2.016783 1.813962
H -8.228136 0.759420 1.323914
H -8.198697 -0.100438 2.872187
H -6.683507 0.436528 2.137981
H -6.586808 -2.471352 -1.934062
H -8.053452 -2.872634 -1.030881
H -6.467278 -3.320847 -0.382899
SCF Energy (B3LYP/6-31G**)= -1361.70351763
Number of imaginary frequencies = 0

1a_c209
MMFF Geometry
C 1.852924 -1.611366 0.055340
C 2.365961 1.045927 -0.169450
N 3.146897 -1.209421 0.044182
C 0.751492 -0.757328 -0.041299
C 1.029283 0.615804 -0.159246
C 3.398800 0.115283 -0.066075
C -0.036796 1.526575 -0.270403
C -1.369500 1.091563 -0.251762
C -1.642704 -0.272084 -0.123807
C -0.589080 -1.195673 -0.032464
C -3.056150 -0.719421 -0.121697
C -4.175029 0.311875 0.177181
C -3.724535 1.720849 -0.186038
C -2.447916 2.061831 -0.405880
O -0.816836 -2.548294 0.065347
O -3.278594 -1.917281 -0.319583
O -4.426492 0.325603 1.584659
C -5.435851 -0.074806 -0.624330

C -6.047408 -1.379396 -0.152653
C -6.677922 -2.259347 -1.197405
O -6.074641 -1.675544 1.043984
O -4.703757 2.691740 -0.366240
C -5.226672 3.144959 0.884812
C 4.834523 0.522203 -0.054935
C 5.138527 1.882857 0.507013
C 5.765654 -0.347332 -0.506823
C 7.268328 -0.210361 -0.631877
C 8.019433 -0.312212 0.708610
C 7.756579 -1.616298 1.451807
C 7.703474 1.026198 -1.424696
H 1.719495 -2.687003 0.148838
H 2.589834 2.102674 -0.272073
H 0.178898 2.589862 -0.370812
H -2.195822 3.081582 -0.681900
H -1.781634 -2.712155 -0.036276
H -4.895362 -0.511469 1.799433
H -5.192421 -0.162111 -1.690241
H -6.232038 0.671288 -0.519078
H -7.065739 -3.167864 -0.728533
H -7.502380 -1.725220 -1.675750
H -5.928000 -2.541242 -1.940607
H -5.917812 2.409148 1.306046
H -4.426815 3.376479 1.596838
H -5.790301 4.063569 0.696523
H 4.532639 2.083957 1.398094
H 6.175926 1.986669 0.828903
H 4.937092 2.661273 -0.236209
H 5.414063 -1.319935 -0.860793
H 7.590771 -1.069222 -1.239263
H 9.097756 -0.239085 0.518476
H 7.764671 0.526647 1.365029
H 8.002337 -2.480657 0.826765
H 8.375232 -1.664865 2.353738
H 6.710505 -1.698767 1.762084
H 7.131528 1.120883 -2.354309
H 8.762307 0.946991 -1.696220
H 7.587077 1.952916 -0.856864
SCF Energy (B3LYP/6-31G**)= -1361.70223546
Number of imaginary frequencies = 0

1a_c210
MMFF Geometry
C 1.474581 -2.160045 0.462453
C 2.318351 0.256844 -0.451627
N 2.781824 -2.026635 0.130579
C 0.519590 -1.143066 0.370712
C 0.967648 0.104520 -0.106245
C 3.192460 -0.820113 -0.314677
C 0.057516 1.172114 -0.217611
C -1.290449 1.007624 0.121085
C -1.731652 -0.238892 0.579398
C -0.834616 -1.304164 0.725706
C -3.164432 -0.382649 0.924803
C -4.164870 0.587282 0.243405
C -3.532654 1.972217 0.151516
C -2.204567 2.150045 0.089300
O -1.233607 -2.522904 1.217499
O -3.502249 -1.279024 1.698917
O -5.359186 0.671287 1.025691
C -4.558110 0.063514 -1.142839
C -4.767168 -1.434239 -1.155316
C -6.060726 -1.973763 -0.605647
O -3.882771 -2.180213 -1.578174
O -4.335337 3.095057 0.296975
C -5.286923 3.260439 -0.750778
C 4.628714 -0.716746 -0.672688
C 5.167570 -1.914778 -1.408342
C 5.345983 0.366258 -0.302905
C 6.810394 0.642147 -0.567176
C 7.675860 0.392919 0.683765
C 7.380662 1.289421 1.880169
C 6.982564 2.060844 -1.118827
H 1.201490 -3.151279 0.818519
H 2.683351 1.203976 -0.836787
H 0.410825 2.141779 -0.566422
H -1.782319 3.150873 0.066634
H -2.171050 -2.457584 1.507381
H -5.106238 1.066981 1.880078
H -3.778838 0.280392 -1.885196
H -5.481530 0.523830 -1.511568
H -6.165929 -3.024810 -0.889193
H -6.066356 -1.896852 0.483299
H -6.902351 -1.418889 -1.027639
H -4.830845 3.105940 -1.734580
H -6.140100 2.591351 -0.610347
H -5.658410 4.288536 -0.706593
H 4.542962 -2.140467 -2.280259
H 6.184300 -1.774733 -1.780497
H 5.184008 -2.795162 -0.756559
H 4.850069 1.142132 0.280902
H 7.184782 -0.029431 -1.346374
H 7.560897 -0.652914 0.996684
H 8.732017 0.517100 0.412824
H 6.350200 1.173166 2.228165
H 8.041804 1.026032 2.712502
H 7.552570 2.343586 1.644785
H 6.456898 2.169548 -2.074467
H 8.040608 2.282520 -1.294848
H 6.585292 2.820812 -0.437552
SCF Energy (B3LYP/6-31G**)= -1361.69237238
Number of imaginary frequencies = 0

1a_c211
MMFF Geometry
C -1.658027 -1.908597 -0.312140
C -2.086601 0.728524 0.167529
N -2.930768 -1.498679 -0.090998
C -0.538875 -1.071876 -0.315470
C -0.773155 0.290511 -0.068565
C -3.140224 -0.184308 0.150441
C 0.312782 1.184258 -0.065849
C 1.625959 0.744815 -0.301025
C 1.856814 -0.612938 -0.539153
C 0.778986 -1.516730 -0.550524
C 3.242238 -1.082974 -0.784909
C 4.405414 -0.066762 -0.742844
C 3.992939 1.375207 -0.493857
C 2.711481 1.728037 -0.294184
O 0.957161 -2.859128 -0.795164
O 3.413631 -2.279747 -1.039201
O 5.021489 -0.112125 -2.049073
C 5.472284 -0.570540 0.239358
C 5.022393 -0.576172 1.683714
C 6.089934 -0.845311 2.711984
O 3.851196 -0.402874 2.018297
O 5.063489 2.252710 -0.504922
C 4.821348 3.632089 -0.254106
C -4.551099 0.227690 0.410577
C -4.743869 1.416202 1.310589
C -5.555910 -0.492606 -0.136529
C -7.058486 -0.307881 -0.068614
C -7.525534 1.087537 -0.525386
C -9.028815 1.167264 -0.761966
C -7.614698 -0.726020 1.294465
H -1.557226 -2.976336 -0.494741
H -2.277406 1.780941 0.349367
H 0.125942 2.240199 0.126940
H 2.424464 2.757645 -0.111969
H 1.917421 -3.024182 -0.938893
H 4.458023 0.399456 -2.653568
H 6.375457 0.048185 0.162770
H 5.778970 -1.592530 -0.017325
H 5.651766 -0.821727 3.713622
H 6.524557 -1.832729 2.538432
H 6.862831 -0.075439 2.648136
H 4.173543 4.061603 -1.024917
H 4.396851 3.779420 0.744152
H 5.781929 4.154247 -0.292170
H -5.741635 1.458641 1.750063
H -4.049757 1.383109 2.158333

H -4.579649 2.348186 0.759795
H -5.278732 -1.354650 -0.748950
H -7.467686 -1.022832 -0.797773
H -7.256552 1.855173 0.206643
H -7.017569 1.352429 -1.461494
H -9.588691 1.029211 0.167555
H -9.292935 2.150542 -1.164597
H -9.355376 0.409386 -1.480933
H -7.200273 -1.690335 1.609924
H -8.702692 -0.841410 1.253813
H -7.389604 0.007371 2.074059
SCF Energy (B3LYP/6-31G**)= -1361.70152384
Number of imaginary frequencies = 0

1a_c212
MMFF Geometry
C -1.754580 -1.309503 -0.523087
C -2.083752 1.291528 0.187733
N -3.011005 -0.869590 -0.269501
C -0.604322 -0.521250 -0.447580
C -0.786403 0.825336 -0.079549
C -3.172048 0.425750 0.086378
C 0.330841 1.677831 0.000595
C 1.621393 1.201687 -0.258181
C 1.795679 -0.142626 -0.605344
C 0.693261 -0.997822 -0.722266
C 3.173384 -0.617503 -0.868439
C 4.337994 0.166263 -0.208596
C 4.019567 1.657411 -0.245349
C 2.760667 2.120269 -0.261384
O 0.832573 -2.308185 -1.109365
O 3.333383 -1.624024 -1.559936
O 5.546847 -0.070073 -0.935412
C 4.562532 -0.318051 1.228636
C 4.439369 -1.819854 1.356988
C 5.601078 -2.666889 0.910368
O 3.400462 -2.322061 1.788115
O 5.051560 2.567023 -0.429336
C 5.982504 2.605732 0.648841
C -4.567879 0.867078 0.373647
C -4.736565 2.033924 1.309166
C -5.603778 0.195365 -0.176480
C -7.080701 0.488037 -0.033184
C -7.757085 -0.456787 0.978870
C -7.738162 -1.935831 0.612183
C -7.759415 0.448533 -1.406209
H -1.694957 -2.359944 -0.800051
H -2.234755 2.331100 0.459020
H 0.186478 2.725413 0.262264
H 2.565890 3.186950 -0.330033
H 1.771071 -2.469439 -1.354888
H 5.413492 0.300271 -1.827375
H 3.825740 0.119591 1.914936
H 5.551702 -0.039264 1.608610
H 5.464922 -3.690205 1.271547
H 5.658851 -2.679197 -0.179705
H 6.529349 -2.274681 1.333191
H 5.472646 2.630095 1.617959
H 6.673748 1.760315 0.596331
H 6.569955 3.523513 0.551276
H -4.544574 2.977147 0.787325
H -5.738454 2.088314 1.743604
H -4.049927 1.956321 2.159997
H -5.379681 -0.660572 -0.816396
H -7.221082 1.509414 0.338756
H -7.277168 -0.336167 1.958647
H -8.801606 -0.146707 1.109260
H -6.716723 -2.314179 0.511366
H -8.230496 -2.518753 1.397696
H -8.273897 -2.125100 -0.322379
H -7.338776 1.215409 -2.066774
H -8.833235 0.643142 -1.313322
H -7.631591 -0.518879 -1.903437
SCF Energy (B3LYP/6-31G**)= -1361.69288311
Number of imaginary frequencies = 0

1a_c213
MMFF Geometry
C 1.853975 2.254533 0.003189
C 2.310630 -0.363446 -0.569597
N 3.139491 1.829558 -0.045254
C 0.736254 1.444218 -0.219557
C 0.984865 0.091088 -0.518640
C 3.354617 0.525338 -0.320887
C -0.097497 -0.772761 -0.768740
C -1.420019 -0.314334 -0.706019
C -1.660355 1.025867 -0.391110
C -0.593465 1.907534 -0.166558
C -3.064983 1.486000 -0.306302
C -4.161043 0.420974 -0.072109
C -3.801416 -0.880922 -0.779844
C -2.524398 -1.217802 -1.038534
O -0.802595 3.236897 0.112056
O -3.284066 2.697113 -0.370210
O -5.394882 0.930259 -0.590944
C -4.317677 0.273766 1.445861
C -5.168247 -0.909847 1.847941
C -6.662817 -0.786233 1.706709
O -4.648002 -1.946536 2.266803
O -4.904202 -1.651215 -1.100335
C -4.694112 -2.993786 -1.524011
C 4.775536 0.102969 -0.366624
C 5.674271 1.066084 -1.091105
C 5.161479 -1.043651 0.234010
C 6.541407 -1.654629 0.369131
C 7.624161 -0.746362 0.983413
C 7.244960 -0.194622 2.351987
C 7.011616 -2.271813 -0.950571
H 1.740373 3.311279 0.235995
H 2.528676 -1.398579 -0.814096
H 0.100093 -1.815549 -1.015501
H -2.259070 -2.151250 -1.522919
H -1.756075 3.442097 -0.024842
H -5.346071 0.860409 -1.560430
H -4.769123 1.172715 1.884515
H -3.342577 0.151121 1.935458
H -7.147927 -1.591632 2.265624
H -6.996830 0.167518 2.122837
H -6.950483 -0.860750 0.656536
H -4.189177 -3.020712 -2.494687
H -4.133389 -3.559514 -0.772831
H -5.673918 -3.466067 -1.640978
H 5.906210 1.928488 -0.457219
H 6.616160 0.612701 -1.403846
H 5.196557 1.429722 -2.008498
H 4.400981 -1.632048 0.749865
H 6.422913 -2.495657 1.068542
H 8.547465 -1.329126 1.096758
H 7.874048 0.085888 0.318543
H 6.997723 -1.003658 3.046641
H 8.083676 0.368173 2.774387
H 6.388545 0.483336 2.290424
H 6.254801 -2.951157 -1.358653
H 7.927805 -2.852693 -0.797147
H 7.225717 -1.513080 -1.708856
SCF Energy (B3LYP/6-31G**)= -1361.69726207
Number of imaginary frequencies = 0

1a_c214
MMFF Geometry
C -1.775867 -2.447954 0.163845
C -2.376570 0.194050 -0.058332
N -3.073160 -2.078548 0.290802
C -0.714698 -1.568459 -0.072880
C -1.037415 -0.202632 -0.185031
C -3.361387 -0.764970 0.170533
C -0.014355 0.732892 -0.426783
C 1.322682 0.330583 -0.543795
C 1.637831 -1.025066 -0.417501
C 0.628661 -1.974151 -0.200927
C 3.058621 -1.428209 -0.521044
C 4.136277 -0.349655 -0.264501

C 3.664847 1.009029 -0.771232
C 2.357941 1.315949 -0.865869
O 0.909315 -3.315839 -0.102515
O 3.310762 -2.612570 -0.750447
O 5.328461 -0.738286 -0.956323
C 4.438496 -0.375271 1.238310
C 5.286450 0.788905 1.698485
C 6.762612 0.748746 1.401320
O 4.777599 1.745017 2.288172
O 4.704037 1.860991 -1.097521
C 4.408290 3.233709 -1.330595
C -4.791510 -0.402259 0.322135
C -5.477985 -1.100592 1.462949
C -5.363884 0.467872 -0.537799
C -6.793725 0.963044 -0.614030
C -7.182991 1.938033 0.512738
C -6.289793 3.170842 0.580427
C -7.816576 -0.168349 -0.755208
H -1.603256 -3.517575 0.263545
H -2.648562 1.243188 -0.123525
H -0.270303 1.787421 -0.525775
H 2.014969 2.287147 -1.205712
H 1.850488 -3.460818 -0.353829
H 5.180932 -0.557017 -1.901062
H 4.960049 -1.298914 1.519740
H 3.513514 -0.353974 1.829519
H 7.275099 1.502354 2.006149
H 7.166948 -0.232172 1.663392
H 6.941332 0.960383 0.345650
H 3.809171 3.353639 -2.238651
H 3.906046 3.679616 -0.465927
H 5.355376 3.760420 -1.480114
H -4.845736 -1.094617 2.358610
H -6.414553 -0.622176 1.752670
H -5.698018 -2.141279 1.202175
H -4.746145 0.875308 -1.339668
H -6.857784 1.530465 -1.554468
H -8.215509 2.273042 0.351519
H -7.170534 1.438105 1.486881
H -6.279128 3.700870 -0.377254
H -6.661402 3.861309 1.344449
H -5.260506 2.908155 0.842960
H -7.516133 -0.876734 -1.535122
H -8.793928 0.238508 -1.038459
H -7.957419 -0.725849 0.174468
SCF Energy (B3LYP/6-31G**)= -1361.69731650
Number of imaginary frequencies = 0

1a_c215
MMFF Geometry
C 1.556888 -2.252194 0.167334
C 2.459415 0.275954 -0.256725
N 2.896907 -2.055295 0.123577
C 0.595578 -1.248542 0.009607
C 1.075001 0.055431 -0.212280
C 3.334134 -0.793739 -0.077785
C 0.157948 1.106401 -0.391262
C -1.224608 0.880624 -0.337561
C -1.698673 -0.412651 -0.105980
C -0.795569 -1.476147 0.053396
C -3.163345 -0.639180 -0.067917
C -4.109606 0.569073 0.154617
C -3.456707 1.862344 -0.315699
C -2.146311 1.988651 -0.563237
O -1.224040 -2.767547 0.252979
O -3.566684 -1.800810 -0.174956
O -4.336541 0.725877 1.557584
C -5.425423 0.319159 -0.612042
C -6.220065 -0.838960 -0.041302
C -6.991314 -1.688117 -1.014689
O -6.274389 -1.037992 1.174354
O -4.280709 2.953954 -0.567407
C -4.711150 3.574392 0.646577
C 4.806580 -0.622687 -0.121437
C 5.520745 -1.679840 -0.916921
C 5.391467 0.403083 0.534496
C 6.860121 0.750970 0.682666
C 7.404103 1.424782 -0.590054
C 8.811285 1.982820 -0.424074
C 7.720099 -0.415734 1.177857
H 1.261926 -3.285149 0.339767
H 2.852034 1.270890 -0.442803
H 0.530885 2.114031 -0.572362
H -1.746897 2.934858 -0.916522
H -2.203798 -2.790008 0.165747
H -4.923193 -0.012158 1.836487
H -5.212906 0.116808 -1.668893
H -6.098421 1.182972 -0.561538
H -7.505073 -2.489980 -0.477368
H -7.732478 -1.072071 -1.529468
H -6.303411 -2.134851 -1.736780
H -5.498893 2.985506 1.125334
H -3.875250 3.735584 1.336245
H -5.132836 4.550715 0.389778
H 6.509743 -1.359267 -1.249448
H 5.636482 -2.594668 -0.326334
H 4.964281 -1.925609 -1.829123
H 4.749546 1.087576 1.091297
H 6.897988 1.503390 1.484359
H 7.404542 0.722104 -1.430842
H 6.739418 2.250582 -0.875211
H 9.542174 1.184772 -0.263501
H 9.107968 2.526424 -1.326901
H 8.862179 2.676881 0.420691
H 7.213623 -0.971901 1.974502
H 8.664058 -0.046693 1.593732
H 7.973294 -1.116887 0.378175
SCF Energy (B3LYP/6-31G**)= -1361.70071935
Number of imaginary frequencies = 0

1a_c216
MMFF Geometry
C -1.775182 -2.468977 -0.187673
C -2.540577 0.138066 -0.240144
N -3.101434 -2.194981 -0.144808
C -0.762458 -1.506706 -0.255442
C -1.170814 -0.161088 -0.284326
C -3.470720 -0.896543 -0.162897
C -0.199170 0.853073 -0.362773
C 1.170088 0.549849 -0.399973
C 1.570874 -0.787351 -0.362032
C 0.613058 -1.812522 -0.301674
C 3.015844 -1.099120 -0.400341
C 4.040143 0.027063 -0.167473
C 3.467174 1.415182 -0.405097
C 2.144417 1.637113 -0.507246
O 0.971508 -3.139633 -0.282593
O 3.365796 -2.268534 -0.588854
O 5.057566 -0.214391 -1.151673
C 4.591673 -0.105769 1.261200
C 6.101040 -0.101526 1.331798
C 6.817952 1.214622 1.197558
O 6.724560 -1.155877 1.470047
O 4.436687 2.400058 -0.470761
C 4.056323 3.710822 -0.871508
C -4.930315 -0.641567 -0.120968
C -5.745520 -1.568824 -0.981835
C -5.433032 0.337378 0.661540
C -6.888138 0.706787 0.854725
C -7.178595 2.133011 0.351908
C -6.989393 2.289746 -1.151840
C -7.243402 0.592168 2.340820
H -1.536616 -3.530359 -0.165745
H -2.880111 1.168489 -0.280432
H -0.521946 1.893386 -0.395307
H 1.741410 2.632040 -0.662117
H 1.942547 -3.209221 -0.429724
H 5.292323 -1.162925 -1.086211
H 4.262131 -1.046260 1.723789
H 4.207851 0.675103 1.929865
H 6.893816 1.491956 0.144503
H 6.286969 1.988524 1.757391

H 7.824103 1.123232 1.616739
H 3.581400 3.698246 -1.857856
H 3.401035 4.170931 -0.125261
H 4.964058 4.317438 -0.941177
H -5.858159 -2.544476 -0.497400
H -6.743885 -1.180742 -1.197781
H -5.263924 -1.718926 -1.955273
H -4.746608 0.930987 1.265847
H -7.545701 0.015116 0.319111
H -8.215309 2.396804 0.595981
H -6.539528 2.858963 0.870467
H -7.608469 1.573595 -1.701471
H -7.281176 3.297905 -1.463248
H -5.945553 2.141232 -1.444346
H -7.058044 -0.422369 2.711244
H -8.303212 0.816525 2.503526
H -6.655907 1.287363 2.951365
SCF Energy (B3LYP/6-31G**)= -1361.70394084
Number of imaginary frequencies = 0

1a_c217
MMFF Geometry
C 1.826780 -2.275157 -0.025171
C 2.469100 0.332807 -0.438813
N 3.123584 -1.971685 -0.274227
C 0.785412 -1.343393 0.033370
C 1.129612 0.002827 -0.184416
C 3.433753 -0.672188 -0.468924
C 0.125719 0.987240 -0.140373
C -1.213621 0.652397 0.109150
C -1.551146 -0.686239 0.319561
C -0.558902 -1.679609 0.293116
C -2.964985 -1.031713 0.580862
C -4.062627 0.014438 0.311725
C -3.533400 1.439088 0.267957
C -2.220040 1.713560 0.172475
O -0.853350 -3.004607 0.511642
O -3.233657 -2.168619 0.981858
O -4.944444 -0.103617 1.438912
C -4.771227 -0.352128 -1.002241
C -6.276835 -0.404310 -0.885619
C -7.036034 0.894625 -0.860739
O -6.860349 -1.485724 -0.787641
O -4.534969 2.392891 0.305044
C -4.174041 3.760584 0.455071
C 4.862733 -0.385317 -0.739315
C 5.529149 -1.370748 -1.661365
C 5.473719 0.666793 -0.153658
C 6.926478 1.073482 -0.276566
C 7.609962 1.146734 1.102717
C 7.690579 -0.203989 1.803526
C 7.009714 2.432093 -0.977047
H 1.637452 -3.335067 0.131881
H 2.755668 1.363220 -0.625183
H 0.399540 2.029465 -0.302495
H -1.850179 2.732081 0.126747
H -1.795399 -3.079897 0.787850
H -5.139385 -1.057547 1.544420
H -4.453096 -1.341946 -1.357431
H -4.507066 0.328416 -1.821555
H -7.000698 1.327813 0.140606
H -6.612928 1.589137 -1.590750
H -8.078926 0.709708 -1.133847
H -3.586686 3.914676 1.366037
H -3.633789 4.119283 -0.426661
H -5.095594 4.343301 0.544512
H 4.870290 -1.626056 -2.499679
H 6.446172 -0.980005 -2.109112
H 5.778750 -2.291957 -1.124364
H 4.898579 1.297693 0.524537
H 7.487627 0.356021 -0.883184
H 8.630581 1.530066 0.978792
H 7.084762 1.857286 1.753509
H 8.206374 -0.939679 1.178233
H 8.247314 -0.107243 2.741161
H 6.696634 -0.592099 2.045823
H 6.549783 2.389827 -1.970787
H 8.053641 2.737297 -1.107374
H 6.500506 3.214148 -0.402316
SCF Energy (B3LYP/6-31G**)= -1361.70400600
Number of imaginary frequencies = 0

1a_c218
MMFF Geometry
C 1.919718 1.987704 0.032677
C 2.264587 -0.704614 0.135026
N 3.173432 1.511097 0.222866
C 0.779449 1.194288 -0.115236
C 0.969972 -0.198264 -0.062708
C 3.341505 0.169652 0.274879
C -0.137871 -1.052431 -0.216655
C -1.429490 -0.541344 -0.405176
C -1.612589 0.842597 -0.441404
C -0.517512 1.709259 -0.313855
C -2.983200 1.364897 -0.621855
C -4.179483 0.431830 -0.355247
C -3.825910 -1.024100 -0.618764
C -2.551792 -1.458114 -0.617268
O -0.664745 3.074381 -0.373261
O -3.142400 2.550468 -0.930014
O -5.207947 0.853150 -1.266712
C -4.646033 0.708131 1.078492
C -5.672454 -0.276913 1.592564
C -7.080895 -0.168388 1.068458
O -5.362990 -1.128655 2.428688
O -4.931978 -1.828055 -0.827565
C -4.746682 -3.229907 -0.975939
C 4.732943 -0.317955 0.502643
C 4.872044 -1.623434 1.234633
C 5.770176 0.437089 0.076691
C 7.262995 0.192543 0.141544
C 7.756201 -1.157228 -0.415284
C 7.326035 -1.410823 -1.854672
C 7.808224 0.449215 1.548772
H 1.853954 3.073180 0.001886
H 2.424371 -1.777568 0.164027
H 0.014761 -2.131057 -0.189180
H -2.299898 -2.499475 -0.785403
H -1.586539 3.283014 -0.645499
H -5.087737 1.814083 -1.403205
H -5.082132 1.711543 1.164937
H -3.796878 0.681183 1.774471
H -7.739885 -0.807472 1.663254
H -7.428887 0.863576 1.159969
H -7.127303 -0.490043 0.026927
H -4.158902 -3.453456 -1.871863
H -4.281865 -3.660035 -0.082958
H -5.732545 -3.688447 -1.096785
H 4.714667 -2.466359 0.553773
H 5.851205 -1.744694 1.700921
H 4.146603 -1.694418 2.053331
H 5.533090 1.384048 -0.415075
H 7.714232 0.962859 -0.501580
H 8.853409 -1.169108 -0.382813
H 7.429219 -1.993729 0.209416
H 7.648072 -0.595372 -2.510071
H 7.777746 -2.337715 -2.222696
H 6.240331 -1.515190 -1.939028
H 7.495684 1.432292 1.918500
H 8.903668 0.431317 1.545058
H 7.468387 -0.303085 2.266626
SCF Energy (B3LYP/6-31G**)= -1361.70359415
Number of imaginary frequencies = 0

1a_c219
MMFF Geometry
C 1.675929 -2.053786 0.177444
C 2.431976 0.555806 0.264002
N 2.996734 -1.779144 0.304821
C 0.664991 -1.091835 0.086388
C 1.068130 0.256141 0.133123
C 3.361511 -0.479555 0.339641

C 0.098376 1.271731 0.041805
C -1.263612 0.964879 -0.080795
C -1.657796 -0.374225 -0.111398
C -0.703633 -1.399894 -0.047764
C -3.099431 -0.672665 -0.242533
C -4.108020 0.409808 0.213227
C -3.567115 1.802844 -0.123048
C -2.246062 2.039773 -0.238912
O -1.061905 -2.725221 -0.114933
O -3.422746 -1.783784 -0.668321
O -4.231517 0.378816 1.638612
C -5.471263 0.157922 -0.463473
C -6.099680 -1.150239 -0.024466
C -6.945007 -1.877048 -1.034758
O -5.969236 -1.567863 1.128481
O -4.552228 2.768890 -0.243482
C -4.160673 4.131730 -0.359134
C 4.814934 -0.223060 0.485938
C 5.488131 -1.100644 1.504213
C 5.418737 0.712467 -0.278680
C 6.877068 1.116983 -0.350578
C 7.880667 -0.021516 -0.617536
C 7.574568 -0.812539 -1.883156
C 7.287109 1.950278 0.866433
H 1.439880 -3.115529 0.150834
H 2.765581 1.587380 0.320211
H 0.414781 2.313781 0.067181
H -1.851328 3.027486 -0.447114
H -2.020182 -2.779808 -0.330506
H -4.745027 -0.427627 1.864007
H -5.355851 0.147355 -1.553954
H -6.203216 0.931168 -0.201070
H -7.325203 -2.804563 -0.597925
H -7.789440 -1.247440 -1.325213
H -6.339058 -2.124499 -1.909849
H -3.566917 4.444741 0.505786
H -3.616415 4.303005 -1.293259
H -5.068855 4.741247 -0.383009
H 5.662605 -2.101609 1.095588
H 6.445887 -0.700627 1.840692
H 4.869936 -1.197576 2.404489
H 4.806331 1.257086 -0.998974
H 6.952083 1.792283 -1.215958
H 8.883254 0.412745 -0.723316
H 7.939908 -0.710907 0.230028
H 7.517841 -0.152465 -2.754531
H 8.366046 -1.546332 -2.066768
H 6.629965 -1.358034 -1.799338
H 6.594152 2.784550 1.023308
H 8.286695 2.374679 0.721261
H 7.311459 1.356657 1.784876
SCF Energy (B3LYP/6-31G**)= -1361.70704221
Number of imaginary frequencies = 0

1a_c220
MMFF Geometry
C 1.586074 -2.307071 -0.181287
C 2.496354 0.254120 -0.168346
N 2.926672 -2.109687 -0.169620
C 0.628344 -1.288010 -0.188034
C 1.111370 0.034261 -0.184928
C 3.367384 -0.833582 -0.152237
C 0.198042 1.104498 -0.208359
C -1.184887 0.877408 -0.215175
C -1.657285 -0.436424 -0.199140
C -0.762269 -1.515951 -0.205529
C -3.119750 -0.650256 -0.207642
C -4.022057 0.492017 0.319447
C -3.431409 1.848859 -0.075991
C -2.113076 2.007161 -0.303371
O -1.201128 -2.818263 -0.232824
O -3.541553 -1.742439 -0.594435
O -4.026675 0.476737 1.750416
C -5.449752 0.316562 -0.237918
C -6.113500 -0.950075 0.266035
C -7.081956 -1.631641 -0.661907
O -5.910394 -1.368262 1.408216
O -4.365580 2.870153 -0.125436
C -3.906947 4.206766 -0.290200
C 4.840482 -0.660284 -0.142690
C 5.566079 -1.571006 -1.093571
C 5.410332 0.238797 0.688668
C 6.871840 0.571143 0.909387
C 7.523574 1.344976 -0.251718
C 6.815500 2.654329 -0.577958
C 7.711488 -0.644088 1.314846
H 1.287562 -3.353305 -0.185696
H 2.893181 1.264588 -0.181612
H 0.575405 2.126302 -0.220099
H -1.680432 2.968634 -0.554640
H -2.175821 -2.817376 -0.365906
H -4.564969 -0.297064 2.026686
H -5.427487 0.293065 -1.334061
H -6.110993 1.132811 0.076756
H -7.476934 -2.532854 -0.184972
H -7.910031 -0.955230 -0.886844
H -6.567574 -1.919007 -1.582244
H -3.225854 4.490977 0.518467
H -3.433712 4.338724 -1.268351
H -4.777186 4.868294 -0.246172
H 5.057069 -1.603990 -2.064034
H 6.586334 -1.244673 -1.299907
H 5.612800 -2.589250 -0.693014
H 4.755950 0.811389 1.348058
H 6.891758 1.241183 1.781818
H 8.562798 1.576068 0.014603
H 7.567520 0.731209 -1.157477
H 6.754842 3.298352 0.305076
H 7.367144 3.195530 -1.353431
H 5.801615 2.481374 -0.951432
H 7.225618 -1.209046 2.118026
H 8.690781 -0.320593 1.685407
H 7.892707 -1.326779 0.480555
SCF Energy (B3LYP/6-31G**)= -1361.70707261
Number of imaginary frequencies = 0

1a_c221
MMFF Geometry
C 1.610447 2.442056 -0.224701
C 2.366838 -0.166667 -0.293587
N 2.936332 2.164374 -0.202727
C 0.593284 1.483384 -0.279783
C 0.997678 0.137249 -0.317322
C 3.301357 0.864654 -0.228487
C 0.022380 -0.873307 -0.378569
C -1.345952 -0.565532 -0.401003
C -1.747392 0.772834 -0.361458
C -0.782873 1.793382 -0.301760
C -3.196071 1.095178 -0.371898
C -4.238000 -0.036470 -0.462502
C -3.634616 -1.420828 -0.512772
C -2.317834 -1.652811 -0.451791
O -1.131568 3.123073 -0.259321
O -3.539935 2.281826 -0.340850
O -4.866974 0.217386 -1.739553
C -5.305975 0.143657 0.626385
C -4.896707 -0.330875 2.005171
C -3.827459 0.438839 2.734486
O -5.429793 -1.309800 2.528267
O -4.487657 -2.515292 -0.478502
C -5.267674 -2.655510 -1.664559
C 4.760599 0.605089 -0.207796
C 5.566742 1.531908 -1.077493
C 5.271017 -0.377836 0.564669
C 6.727296 -0.753219 0.736136
C 7.005368 -2.178881 0.224794
C 6.794375 -2.330138 -1.276614
C 7.103913 -0.644631 2.217416
H 1.376175 3.504143 -0.196392
H 2.701569 -1.198553 -0.339552
H 0.339085 -1.915916 -0.408031
H -1.953126 -2.676714 -0.437487

H -2.110714 3.198436 -0.309143
H -5.179654 1.142436 -1.722539
H -6.217535 -0.401286 0.346143
H -5.625786 1.189798 0.711469
H -3.915090 0.250541 3.808503
H -2.840741 0.117682 2.397429
H -3.957324 1.511099 2.568561
H -6.129019 -1.982079 -1.641282
H -4.667756 -2.490965 -2.566309
H -5.648465 -3.680820 -1.693754
H 5.689019 2.506025 -0.592304
H 6.560871 1.141380 -1.308062
H 5.072281 1.685873 -2.043851
H 4.591129 -0.971433 1.176499
H 7.379780 -0.062242 0.193459
H 8.044446 -2.447219 0.453363
H 6.371019 -2.904153 0.750054
H 7.408250 -1.614486 -1.832692
H 7.078034 -3.338349 -1.595273
H 5.747060 -2.176916 -1.553871
H 6.927554 0.369392 2.593597
H 8.165075 -0.873377 2.364439
H 6.522522 -1.339614 2.834016
SCF Energy (B3LYP/6-31G**)= -1361.69840314
Number of imaginary frequencies = 0

1a_c222
MMFF Geometry
C -1.564638 -1.872770 -0.917394
C -2.277621 0.682404 -0.324172
N -2.886233 -1.584864 -0.834379
C -0.532235 -0.950094 -0.721222
C -0.913349 0.370731 -0.417194
C -3.228771 -0.315087 -0.529377
C 0.078396 1.350254 -0.223126
C 1.440153 1.031054 -0.310284
C 1.809455 -0.286560 -0.595175
C 0.836599 -1.270280 -0.818723
C 3.248528 -0.597125 -0.691545
C 4.255212 0.352972 -0.014501
C 3.757168 1.793405 -0.028496
C 2.448581 2.085434 -0.157106
O 1.173478 -2.562242 -1.142141
O 3.617654 -1.603823 -1.303099
O 5.473090 0.278747 -0.778205
C 4.571488 -0.057128 1.425784
C 4.682907 -1.553556 1.590508
C 6.015668 -2.195491 1.313286
O 3.695773 -2.221534 1.901536
O 4.763828 2.725081 0.151648
C 4.439310 4.107102 0.072086
C -4.684484 -0.042243 -0.451033
C -5.482146 -0.670134 -1.559925
C -5.182930 0.690024 0.568663
C -6.616748 1.056482 0.893372
C -7.604959 -0.120560 1.005890
C -7.174827 -1.176229 2.016709
C -7.144041 2.141511 -0.049128
H -1.346624 -2.911524 -1.156865
H -2.596951 1.697147 -0.107586
H -0.222396 2.374050 -0.003369
H 2.082309 3.106008 -0.141708
H 2.148634 -2.620977 -1.250260
H 5.390819 -0.494064 -1.372144
H 3.782840 0.272662 2.115267
H 5.506816 0.395537 1.777287
H 5.992511 -3.239078 1.639652
H 6.230541 -2.162529 0.243443
H 6.798967 -1.677526 1.872179
H 4.011468 4.354126 -0.904901
H 3.761648 4.395651 0.881849
H 5.365354 4.677707 0.189035
H -5.628266 -1.739166 -1.372398
H -6.463948 -0.211472 -1.687276
H -4.969408 -0.554729 -2.522129
H -4.484988 1.059742 1.321511
H -6.583160 1.516756 1.892151
H -8.581660 0.270568 1.318981
H -7.770145 -0.599646 0.036184
H -7.010009 -0.731129 3.003082
H -7.954188 -1.938609 2.116597
H -6.255316 -1.681110 1.706128
H -6.457738 2.994842 -0.089406
H -8.113496 2.514181 0.299637
H -7.280874 1.772103 -1.069581
SCF Energy (B3LYP/6-31G**)= -1361.69922360
Number of imaginary frequencies = 0

1a_c223
MMFF Geometry
C -1.616110 -1.749951 -0.549051
C -2.191790 0.744928 0.358212
N -2.908291 -1.454994 -0.266781
C -0.547315 -0.861279 -0.410056
C -0.856960 0.428613 0.058650
C -3.190690 -0.212593 0.187273
C 0.173907 1.375478 0.207302
C 1.505361 1.051184 -0.086379
C 1.806526 -0.238570 -0.533843
C 0.789644 -1.186154 -0.715204
C 3.217190 -0.559352 -0.838458
C 4.313229 0.307437 -0.174732
C 3.852893 1.757837 -0.042152
C 2.546607 2.079335 0.013789
O 1.051736 -2.446145 -1.196080
O 3.456193 -1.512671 -1.581625
O 5.489053 0.267533 -0.994263
C 4.702435 -0.234785 1.202403
C 4.801240 -1.742434 1.218869
C 6.042096 -2.378346 0.651483
O 3.869984 -2.420325 1.657088
O 4.898095 2.659514 0.035287
C 4.600079 4.042079 0.191072
C -4.620573 0.070146 0.505254
C -4.876774 1.065629 1.601777
C -5.585574 -0.592078 -0.171149
C -7.094271 -0.497573 -0.086742
C -7.695567 0.915964 -0.211447
C -7.275167 1.641663 -1.483380
C -7.628744 -1.224869 1.149710
H -1.455662 -2.765475 -0.905104
H -2.444100 1.740455 0.708226
H -0.072568 2.378239 0.554523
H 2.206377 3.102681 0.128903
H 2.003059 -2.501123 -1.441686
H 5.300049 0.796448 -1.788816
H 3.958790 0.039850 1.962298
H 5.664000 0.170897 1.540400
H 6.085233 -3.427410 0.957736
H 6.024742 -2.325949 -0.438814
H 6.929155 -1.871748 1.039793
H 4.031927 4.418153 -0.665731
H 4.063925 4.222929 1.128194
H 5.547314 4.587711 0.233540
H -4.787220 2.088937 1.222735
H -5.866264 0.956765 2.048988
H -4.166288 0.934261 2.426111
H -5.263560 -1.316820 -0.923487
H -7.472056 -1.059626 -0.953904
H -8.790046 0.831373 -0.216022
H -7.446979 1.536671 0.654483
H -7.520374 1.050886 -2.371677
H -7.800809 2.599021 -1.559403
H -6.201577 1.851629 -1.492212
H -7.237144 -2.246839 1.204917
H -8.721657 -1.292065 1.112900
H -7.360762 -0.711988 2.078108
SCF Energy (B3LYP/6-31G**)= -1361.69495504
Number of imaginary frequencies = 0

1a_c224
MMFF Geometry

C 1.619850 -2.078321 0.372612
C 2.424878 0.509009 0.165638
N 2.947554 -1.818231 0.449443
C 0.624718 -1.111901 0.193886
C 1.053671 0.223918 0.089476
C 3.337048 -0.530280 0.337398
C 0.100867 1.242193 -0.091814
C -1.268763 0.951092 -0.160585
C -1.693343 -0.374882 -0.049102
C -0.752599 -1.404588 0.114397
C -3.143481 -0.669589 -0.140004
C -4.163471 0.477052 0.082675
C -3.542694 1.827205 -0.251568
C -2.225374 2.028985 -0.386838
O -1.129739 -2.724178 0.201444
O -3.478650 -1.838728 -0.350506
O -4.504223 0.528918 1.470343
C -5.402785 0.219792 -0.800227
C -6.179408 -1.008674 -0.368745
C -6.830121 -1.824994 -1.452058
O -6.316496 -1.290722 0.823749
O -4.399118 2.894969 -0.497321
C -4.950917 3.412008 0.716011
C 4.797615 -0.287689 0.433060
C 5.472287 -1.069503 1.526027
C 5.402760 0.553085 -0.433939
C 6.865184 0.931938 -0.565410
C 7.780512 -0.278649 -0.829342
C 9.190975 0.116593 -1.248613
C 7.330145 1.815632 0.594600
H 1.364396 -3.131951 0.463947
H 2.777431 1.534081 0.105245
H 0.435289 2.275518 -0.178983
H -1.848548 3.013970 -0.646558
H -2.097479 -2.785457 0.034789
H -5.072660 -0.251857 1.654481
H -5.100413 0.098489 -1.847558
H -6.120249 1.046988 -0.748771
H -7.342444 -2.683399 -1.008986
H -7.559806 -1.210154 -1.984382
H -6.067333 -2.190885 -2.143777
H -5.742403 2.757419 1.092303
H -4.178912 3.563456 1.478523
H -5.400057 4.383602 0.489623
H 5.572453 -2.122633 1.242350
H 6.467422 -0.693837 1.767321
H 4.893257 -1.014846 2.455345
H 4.786756 1.026625 -1.200121
H 6.918151 1.561958 -1.465733
H 7.854707 -0.918820 0.055296
H 7.346217 -0.894518 -1.627287
H 9.719902 0.630395 -0.440619
H 9.769053 -0.776534 -1.507152
H 9.172457 0.772167 -2.124859
H 6.603256 2.609394 0.801178
H 8.280364 2.304276 0.355273
H 7.476807 1.245514 1.516149
SCF Energy (B3LYP/6-31G**)= -1361.70110427
Number of imaginary frequencies = 0

1a_c225
MMFF Geometry
C -1.463599 -2.236714 -0.474755
C -2.341948 0.287498 0.021319
N -2.794128 -2.050396 -0.297527
C -0.501064 -1.223551 -0.422044
C -0.967293 0.079409 -0.162776
C -3.220001 -0.791617 -0.059629
C -0.048733 1.143800 -0.099595
C 1.324406 0.928513 -0.278443
C 1.779665 -0.370436 -0.521606
C 0.878344 -1.439800 -0.612112
C 3.228187 -0.571681 -0.716223
C 4.200449 0.493458 -0.173441
C 3.584493 1.886715 -0.223795
C 2.250164 2.066414 -0.262533
O 1.296807 -2.718689 -0.888790
O 3.632640 -1.580816 -1.300817
O 5.359057 0.470384 -1.027290
C 4.657696 0.195801 1.256598
C 4.905469 -1.274754 1.491033
C 6.262314 -1.824855 1.142387
O 4.003735 -1.998902 1.915144
O 4.520762 2.903958 -0.174262
C 4.077091 4.248997 -0.299305
C -4.681603 -0.631659 0.135495
C -5.296007 -1.685466 1.014340
C -5.338551 0.380736 -0.470890
C -6.812643 0.730263 -0.458437
C -7.310977 1.308751 0.879007
C -6.555376 2.557863 1.316032
C -7.707543 -0.414686 -0.942637
H -1.177476 -3.268704 -0.667175
H -2.724265 1.279983 0.239216
H -0.416392 2.151872 0.088934
H 1.802362 3.054009 -0.271771
H 2.262079 -2.706392 -1.073618
H 5.296942 -0.340529 -1.570736
H 3.898682 0.502711 1.988594
H 5.575956 0.740905 1.507514
H 6.350839 -2.845827 1.524362
H 6.392320 -1.838531 0.058537
H 7.039707 -1.214490 1.608563
H 3.557385 4.403973 -1.250335
H 3.441020 4.529871 0.546111
H 4.958541 4.896853 -0.286889
H -6.280220 -1.403941 1.391244
H -5.401611 -2.629368 0.469048
H -4.673429 -1.864635 1.898804
H -4.762943 1.054849 -1.107171
H -6.931355 1.527364 -1.207454
H -8.372428 1.569125 0.779802
H -7.251012 0.561512 1.677179
H -6.595681 3.331612 0.542804
H -7.003461 2.967266 2.227173
H -5.505501 2.338064 1.532390
H -7.328264 -0.843931 -1.876574
H -8.721140 -0.046218 -1.137145
H -7.793412 -1.220089 -0.208541
SCF Energy (B3LYP/6-31G**)= -1361.69925832
Number of imaginary frequencies = 0

1a_c226
MMFF Geometry
C 2.087887 1.621702 -0.137857
C 2.339003 -1.075683 0.037529
N 3.335620 1.099721 -0.056399
C 0.908389 0.872957 -0.136872
C 1.051003 -0.521606 -0.046533
C 3.458426 -0.244789 0.030673
C -0.099193 -1.330447 -0.044333
C -1.384569 -0.773266 -0.126607
C -1.525501 0.614548 -0.213345
C -0.383366 1.434351 -0.222072
C -2.885529 1.204875 -0.292408
C -4.127286 0.290752 -0.218936
C -3.792189 -1.184611 -0.196650
C -2.543294 -1.659931 -0.121744
O -0.471657 2.804246 -0.310169
O -2.982914 2.431616 -0.409287
O -4.825307 0.573939 -1.436638
C -4.915822 0.716728 1.032523
C -6.380309 0.352909 0.972601
C -6.901877 -0.611949 2.003572
O -7.129790 0.880267 0.148930
O -4.843707 -2.088813 -0.115522
C -5.502734 -2.262635 -1.372437
C 4.847465 -0.779985 0.133527
C 5.015012 -2.111635 0.814288
C 5.875780 -0.049384 -0.352038
C 7.347564 -0.397590 -0.371188
C 8.167647 0.563123 0.509558

C 7.824034 0.462478 1.990493
C 7.856457 -0.346614 -1.815870
H 2.061050 2.707141 -0.206556
H 2.456567 -2.152832 0.093793
H 0.013588 -2.412438 0.021596
H -2.376339 -2.731784 -0.058280
H -1.418604 3.053357 -0.413095
H -5.335778 1.396084 -1.301031
H -4.896194 1.806463 1.166922
H -4.465662 0.299974 1.942101
H -7.907224 -0.938856 1.724161
H -6.256700 -1.491003 2.061199
H -6.940293 -0.115502 2.976190
H -6.247816 -1.477641 -1.526787
H -4.793582 -2.294582 -2.207032
H -6.031481 -3.219971 -1.339765
H 4.742253 -2.927402 0.136750
H 6.036504 -2.290546 1.158581
H 4.387210 -2.174215 1.710638
H 5.650659 0.918381 -0.805898
H 7.516799 -1.418562 -0.015438
H 9.235069 0.338951 0.389930
H 8.025826 1.599960 0.179131
H 7.949638 -0.562653 2.353145
H 8.485885 1.110812 2.573695
H 6.794470 0.775963 2.187615
H 7.294045 -1.037112 -2.454457
H 8.912190 -0.634890 -1.864403
H 7.763600 0.659029 -2.241658
SCF Energy (B3LYP/6-31G**)= -1361.70458188
Number of imaginary frequencies = 0

1a_c227
MMFF Geometry
C -1.674992 -2.226907 -0.296691
C -2.301264 0.309777 0.448395
N -2.970158 -1.950057 -0.011333
C -0.627348 -1.302043 -0.236164
C -0.963985 0.006550 0.152719
C -3.272388 -0.684274 0.348923
C 0.046054 0.980485 0.239282
C 1.382115 0.673712 -0.058072
C 1.715871 -0.627045 -0.446126
C 0.716412 -1.611228 -0.534694
C 3.132219 -0.952075 -0.747873
C 4.209683 0.146861 -0.670436
C 3.677292 1.496798 -0.249374
C 2.393766 1.718866 0.058357
O 0.999334 -2.904739 -0.906529
O 3.415797 -2.104557 -1.092372
O 4.647602 0.254628 -2.044311
C 5.403443 -0.344810 0.161481
C 5.210539 -0.267477 1.661671
C 4.203266 -1.188196 2.298300
O 5.867761 0.513175 2.350730
O 4.582892 2.526388 -0.033766
C 5.205430 2.985367 -1.232374
C -4.699750 -0.425485 0.654018
C -5.373797 -1.517711 1.440097
C -5.302879 0.698293 0.210631
C -6.752669 1.096193 0.385971
C -7.434374 1.354779 -0.971730
C -7.524345 0.108265 -1.843621
C -6.826489 2.351783 1.258593
H -1.492722 -3.259286 -0.587690
H -2.580388 1.309563 0.766831
H -0.217694 1.993476 0.543404
H 2.087504 2.701791 0.407154
H 1.958092 -2.975398 -1.113131
H 4.912957 -0.641306 -2.328298
H 6.296773 0.243681 -0.087691
H 5.675037 -1.377707 -0.090047
H 4.441926 -1.307364 3.359194
H 3.201222 -0.766710 2.205549
H 4.247611 -2.174494 1.829914
H 6.025508 2.321318 -1.519552
H 4.483576 3.090177 -2.049730
H 5.632562 3.972008 -1.029366
H -4.717706 -1.884034 2.238470
H -6.288841 -1.182335 1.934361
H -5.628732 -2.359766 0.787984
H -4.722534 1.408704 -0.378777
H -7.319643 0.309551 0.893277
H -8.452243 1.726131 -0.798510
H -6.903364 2.140615 -1.523763
H -8.046138 -0.699207 -1.320173
H -8.079496 0.331284 -2.760432
H -6.533078 -0.252117 -2.134789
H -6.367763 2.176135 2.238165
H -7.868249 2.645014 1.427917
H -6.311010 3.198650 0.791348
SCF Energy (B3LYP/6-31G**)= -1361.69856505
Number of imaginary frequencies = 0

1a_c228
MMFF Geometry
C 1.984758 1.779792 0.077498
C 2.207499 -0.906445 -0.257451
N 3.222135 1.230474 0.124592
C 0.802880 1.064669 -0.130249
C 0.929915 -0.325448 -0.302198
C 3.329953 -0.107830 -0.042819
C -0.223523 -1.101767 -0.520352
C -1.497285 -0.517536 -0.556489
C -1.616236 0.861630 -0.372506
C -0.476448 1.654585 -0.175780
C -2.967728 1.459025 -0.395157
C -4.192595 0.543964 -0.207805
C -3.921498 -0.867280 -0.706797
C -2.670799 -1.346179 -0.841472
O -0.561747 3.017125 -0.017704
O -3.085333 2.683291 -0.510313
O -5.242171 1.140310 -0.988076
C -4.576555 0.615611 1.274443
C -5.624490 -0.393587 1.688594
C -7.049001 -0.148628 1.263219
O -5.318035 -1.375181 2.368949
O -5.073225 -1.585788 -0.972511
C -4.962028 -2.955265 -1.338175
C 4.710220 -0.673736 -0.002501
C 4.949151 -1.911513 -0.821314
C 5.657844 -0.038802 0.722901
C 7.113320 -0.380188 0.963131
C 8.030743 -0.089763 -0.239129
C 7.988788 1.363705 -0.694919
C 7.325656 -1.799495 1.499290
H 1.968810 2.858464 0.217983
H 2.314700 -1.979683 -0.376582
H -0.120982 -2.176982 -0.664875
H -2.477062 -2.358718 -1.178188
H -1.484718 3.301416 -0.203845
H -5.084020 2.105418 -0.983052
H -4.958475 1.610893 1.535304
H -3.697632 0.447206 1.911084
H -7.709908 -0.842192 1.791093
H -7.341107 0.871249 1.525803
H -7.160170 -0.307119 0.189474
H -4.429938 -3.063703 -2.288574
H -4.475075 -3.533597 -0.546411
H -5.973234 -3.350923 -1.471235
H 6.003638 -2.070328 -1.052377
H 4.582400 -2.799051 -0.295329
H 4.442527 -1.844510 -1.791059
H 5.360771 0.871850 1.249497
H 7.439609 0.285760 1.775896
H 9.065155 -0.331348 0.036385
H 7.779489 -0.734211 -1.088256
H 8.237524 2.040602 0.128569
H 8.716029 1.526049 -1.497002
H 7.001986 1.635777 -1.081624
H 6.636767 -2.017914 2.322693
H 8.345481 -1.908279 1.885618

H 7.191332 -2.564618 0.730311
SCF Energy (B3LYP/6-31G**)= -1361.70376002
Number of imaginary frequencies = 0

1a_c229
MMFF Geometry
C -1.574463 -2.383261 -0.234473
C -2.286521 0.231783 -0.038852
N -2.890377 -2.094014 -0.087683
C -0.547967 -1.434262 -0.295306
C -0.928904 -0.085253 -0.192144
C -3.231333 -0.791086 0.002675
C 0.058280 0.914606 -0.244367
C 1.417386 0.595690 -0.397949
C 1.794782 -0.746640 -0.496254
C 0.816580 -1.756705 -0.448713
C 3.229452 -1.088738 -0.656276
C 4.280041 0.044014 -0.681190
C 3.713154 1.451325 -0.583927
C 2.395691 1.684138 -0.455325
O 1.141237 -3.089868 -0.553768
O 3.532030 -2.279300 -0.787975
O 4.945397 -0.057738 -1.959777
C 5.355848 -0.250491 0.373632
C 4.855727 -0.167762 1.798967
C 5.906721 -0.220058 2.876956
O 3.661087 -0.092574 2.083337
O 4.688007 2.431950 -0.649219
C 4.296769 3.795347 -0.538057
C -4.677898 -0.515811 0.168076
C -5.342601 -1.350778 1.224611
C -5.276048 0.414721 -0.606537
C -6.724123 0.851990 -0.715354
C -6.964490 2.210361 -0.026991
C -6.782960 2.212784 1.485334
C -7.796914 -0.180306 -0.359775
H -1.355118 -3.446644 -0.304345
H -2.604413 1.265065 0.060380
H -0.243483 1.958167 -0.160531
H 1.998141 2.690200 -0.381132
H 2.118034 -3.164896 -0.655121
H 4.355470 0.333437 -2.625797
H 6.193316 0.449870 0.261600
H 5.774626 -1.254084 0.225925
H 5.431533 -0.149263 3.859305
H 6.449565 -1.166242 2.813182
H 6.595635 0.619869 2.758336
H 3.638052 4.081473 -1.364197
H 3.822005 3.988348 0.429297
H 5.199673 4.410068 -0.598851
H -4.625597 -1.694086 1.979880
H -6.090913 -0.777729 1.777147
H -5.814136 -2.233351 0.781260
H -4.650576 0.938183 -1.333025
H -6.871319 1.033317 -1.791188
H -6.285147 2.957848 -0.457080
H -7.981026 2.553438 -0.257292
H -5.764003 1.934378 1.769450
H -6.972494 3.216597 1.879840
H -7.480827 1.529077 1.976441
H -7.589651 -1.144315 -0.836651
H -8.775005 0.157643 -0.721572
H -7.899025 -0.341687 0.715151
SCF Energy (B3LYP/6-31G**)= -1361.69874195
Number of imaginary frequencies = 0

1a_c230
MMFF Geometry
C 2.069264 1.734967 -0.336258
C 2.374555 -0.961827 -0.399889
N 3.327950 1.234948 -0.376920
C 0.904515 0.963518 -0.322043
C 1.075170 -0.430481 -0.353907
C 3.477453 -0.109198 -0.410584
C -0.059066 -1.261402 -0.338817
C -1.355832 -0.727025 -0.291657
C -1.524559 0.660017 -0.258948
C -0.398905 1.502165 -0.277364
C -2.896128 1.225587 -0.201197
C -4.115262 0.282260 -0.118553
C -3.755770 -1.182903 -0.234925
C -2.497189 -1.635881 -0.280289
O -0.514907 2.872539 -0.249242
O -3.020856 2.455351 -0.214052
O -4.898081 0.644268 -1.261530
C -4.824508 0.592833 1.211832
C -6.283601 0.203418 1.220041
C -6.718856 -0.849223 2.204107
O -7.095271 0.776661 0.491444
O -4.784747 -2.112881 -0.156175
C -5.524377 -2.202429 -1.376534
C 4.877338 -0.620990 -0.472054
C 5.082006 -1.971602 -1.103350
C 5.883456 0.143513 0.007346
C 7.358912 -0.182928 0.069308
C 7.913015 -0.016228 1.497956
C 7.300772 -0.992184 2.495377
C 8.117785 0.728676 -0.898060
H 2.020458 2.821594 -0.314277
H 2.513907 -2.037784 -0.413383
H 0.075289 -2.342596 -0.365965
H -2.309123 -2.705629 -0.312555
H -1.470490 3.108718 -0.270223
H -5.411794 1.442041 -1.027969
H -4.813565 1.669022 1.430158
H -4.307383 0.116495 2.054057
H -7.735390 -1.174835 1.966837
H -6.057226 -1.716169 2.149677
H -6.699522 -0.430484 3.213168
H -6.290628 -1.423804 -1.418812
H -4.872837 -2.154362 -2.256108
H -6.034352 -3.170413 -1.384667
H 4.461955 -2.082799 -2.000376
H 6.110172 -2.135295 -1.435792
H 4.825465 -2.768900 -0.398068
H 5.634340 1.122362 0.423075
H 7.546873 -1.217674 -0.233684
H 8.998462 -0.176196 1.483972
H 7.751560 1.008979 1.854533
H 7.441858 -2.027133 2.167894
H 7.779604 -0.878568 3.473276
H 6.229237 -0.814167 2.627306
H 7.753458 0.602124 -1.923768
H 9.187385 0.492345 -0.894535
H 8.006407 1.785053 -0.628113
SCF Energy (B3LYP/6-31G**)= -1361.70461952
Number of imaginary frequencies = 0

1a_c231
MMFF Geometry
C -1.692776 -1.535014 -0.552427
C -2.129169 1.071018 0.077688
N -2.970754 -1.131044 -0.352656
C -0.571759 -0.706870 -0.459384
C -0.809087 0.640663 -0.132045
C -3.184369 0.167103 -0.038085
C 0.278474 1.528424 -0.029641
C 1.594308 1.091084 -0.233441
C 1.822111 -0.253128 -0.542576
C 0.749838 -1.146148 -0.675042
C 3.217459 -0.693150 -0.754679
C 4.345725 0.161227 -0.130064
C 3.981760 1.644485 -0.155483
C 2.699551 2.054181 -0.186615
O 0.942105 -2.462009 -1.020051
O 3.417064 -1.728011 -1.392998
O 5.544964 -0.034666 -0.891138
C 4.646428 -0.268335 1.307660
C 4.641852 -1.770482 1.470853
C 5.856626 -2.536791 1.020145
O 3.651188 -2.341520 1.930536
O 5.083036 2.479544 -0.118800

C 4.875423 3.887129 -0.106178
C -4.605352 0.578021 0.158654
C -4.942946 2.001757 -0.184958
C -5.498323 -0.339843 0.591487
C -6.976317 -0.215166 0.895329
C -7.867989 -0.138051 -0.357826
C -7.709304 -1.334720 -1.287815
C -7.302312 0.908721 1.884061
H -1.589497 -2.589506 -0.799765
H -2.322506 2.105078 0.343227
H 0.089117 2.574233 0.209669
H 2.426662 3.103827 -0.183639
H 1.895509 -2.602376 -1.219232
H 5.421676 0.425299 -1.739671
H 3.895833 0.126049 2.005469
H 5.620332 0.105742 1.646780
H 5.817483 -3.551461 1.426470
H 5.882496 -2.590295 -0.069931
H 6.761252 -2.052346 1.395998
H 4.366154 4.215028 -1.018025
H 4.319153 4.191961 0.786015
H 5.855792 4.371529 -0.074465
H -4.430707 2.316459 -1.101550
H -6.006510 2.150593 -0.378106
H -4.650793 2.674742 0.627825
H -5.128140 -1.351789 0.776087
H -7.247113 -1.144918 1.417584
H -8.918365 -0.086380 -0.044270
H -7.669822 0.778499 -0.923650
H -7.902294 -2.272469 -0.757181
H -8.421499 -1.262602 -2.116156
H -6.703983 -1.379822 -1.717510
H -6.632673 0.879103 2.750647
H -8.327279 0.798470 2.256103
H -7.230963 1.901264 1.431771
SCF Energy (B3LYP/6-31G**)= -1361.69507201
Number of imaginary frequencies = 0

1a_c232
MMFF Geometry
C -1.716472 -2.074644 -0.580280
C -2.279873 0.576768 -0.398260
N -3.019577 -1.702434 -0.564539
C -0.631500 -1.194084 -0.513265
C -0.936076 0.175288 -0.422950
C -3.287680 -0.383169 -0.462494
C 0.111394 1.110926 -0.361346
C 1.455033 0.706383 -0.386685
C 1.757710 -0.657031 -0.468271
C 0.718716 -1.602497 -0.535312
C 3.179388 -1.088236 -0.503493
C 4.299376 -0.020015 -0.451289
C 3.799504 1.407532 -0.360397
C 2.500146 1.724392 -0.311091
O 0.968039 -2.952145 -0.633837
O 3.421466 -2.292311 -0.635465
O 5.023776 -0.151237 -1.693324
C 5.287183 -0.381936 0.665940
C 4.699817 -0.249093 2.054249
C 5.682765 -0.129697 3.188928
O 3.488145 -0.285094 2.265576
O 4.727891 2.421704 -0.156442
C 5.546522 2.672062 -1.297673
C -4.725020 -0.017565 -0.447846
C -5.553120 -0.761910 -1.457833
C -5.183965 0.891303 0.439718
C -6.594737 1.386638 0.684013
C -7.652048 0.300823 0.962840
C -7.290678 -0.608898 2.130329
C -7.051723 2.341556 -0.421847
H -1.558971 -3.148746 -0.652464
H -2.538047 1.629753 -0.342298
H -0.128796 2.171258 -0.287790
H 2.202317 2.760729 -0.177192
H 1.942648 -3.091178 -0.621675
H 4.374533 -0.087454 -2.415256
H 6.175680 0.258502 0.600827
H 5.646645 -1.412344 0.553012
H 5.143683 -0.037894 4.135902
H 6.311840 -1.022472 3.223245
H 6.298697 0.761934 3.048073
H 6.369841 1.953820 -1.344404
H 4.964839 2.659255 -2.225901
H 5.981524 3.669493 -1.183794
H -5.763263 -1.778908 -1.110120
H -6.505405 -0.272440 -1.667839
H -5.029837 -0.826304 -2.419123
H -4.468645 1.331846 1.136197
H -6.538475 1.993203 1.600228
H -8.605150 0.791942 1.198100
H -7.840821 -0.312155 0.076404
H -7.104371 -0.026844 3.038466
H -8.114669 -1.299905 2.335645
H -6.401468 -1.208478 1.914284
H -6.315469 3.136888 -0.583670
H -7.998839 2.819597 -0.148330
H -7.205488 1.827233 -1.374918
SCF Energy (B3LYP/6-31G**)= -1361.69521670
Number of imaginary frequencies = 0

1a_c233
MMFF Geometry
C -1.732813 -1.141274 -1.081738
C -2.106763 1.348646 -0.062027
N -3.003895 -0.715971 -0.881969
C -0.588685 -0.391188 -0.801588
C -0.793855 0.897215 -0.272575
C -3.187411 0.524340 -0.373500
C 0.317588 1.708446 0.024057
C 1.622881 1.246341 -0.180554
C 1.818338 -0.041614 -0.691482
C 0.725043 -0.851488 -1.021839
C 3.210804 -0.503524 -0.892576
C 4.315791 0.160002 -0.029616
C 4.013062 1.648341 0.110028
C 2.764404 2.134025 0.044543
O 0.889627 -2.100382 -1.569063
O 3.426028 -1.409634 -1.698482
O 5.584435 0.000758 -0.670402
C 4.403903 -0.519652 1.341853
C 4.259741 -2.022341 1.250972
C 5.452187 -2.822590 0.799108
O 3.182270 -2.558782 1.513082
O 5.064283 2.553829 0.145993
C 5.892356 2.427376 1.298792
C -4.601905 0.961253 -0.182652
C -4.865921 2.441880 -0.234569
C -5.560751 0.026589 0.003044
C -7.043175 0.227926 0.226179
C -7.481473 -0.239541 1.629782
C -7.341884 -1.731139 1.908877
C -7.834904 -0.458914 -0.889220
H -1.655686 -2.146487 -1.490756
H -2.273919 2.335815 0.355866
H 0.157570 2.713415 0.412866
H 2.584650 3.203786 0.105752
H 1.845343 -2.245214 -1.749857
H 5.536175 0.491483 -1.511465
H 3.609912 -0.165592 2.012385
H 5.355684 -0.314111 1.844264
H 5.277129 -3.882683 1.003375
H 5.609503 -2.688142 -0.272772
H 6.340251 -2.508717 1.353292
H 5.295709 2.330098 2.212186
H 6.579266 1.583356 1.193482
H 6.493018 3.338066 1.381188
H -4.299254 2.911479 -1.046957
H -5.912886 2.685589 -0.428411
H -4.582268 2.916421 0.710637
H -5.255337 -1.021728 0.000888
H -7.293161 1.292098 0.175798
H -6.908059 0.311286 2.386573

H -8.532458 0.039051 1.779537
H -6.302620 -2.062710 1.831596
H -7.682249 -1.950452 2.926438
H -7.950765 -2.327494 1.223445
H -7.601651 -0.008852 -1.861022
H -8.912163 -0.351079 -0.723682
H -7.606968 -1.527893 -0.959690
SCF Energy (B3LYP/6-31G**)= -1361.69271928
Number of imaginary frequencies = 0

1a_c234
MMFF Geometry
C -1.635060 -2.350095 -0.240240
C -2.349967 0.257324 0.025968
N -2.952613 -2.064491 -0.100997
C -0.607826 -1.400637 -0.260685
C -0.990493 -0.055176 -0.121207
C -3.296687 -0.765023 0.023718
C -0.003439 0.945836 -0.130447
C 1.355890 0.630333 -0.279966
C 1.736722 -0.709117 -0.414236
C 0.758555 -1.719579 -0.407757
C 3.174427 -1.045964 -0.581238
C 4.224136 0.092333 -0.605517
C 3.644021 1.482740 -0.443415
C 2.336458 1.713279 -0.275208
O 1.084304 -3.048854 -0.550332
O 3.480375 -2.229673 -0.757419
O 4.843499 0.026225 -1.908058
C 5.327420 -0.219814 0.414432
C 4.857031 -0.144562 1.850777
C 5.925897 0.026570 2.897751
O 3.673702 -0.265200 2.165878
O 4.520053 2.552452 -0.300795
C 5.217826 2.871637 -1.503491
C -4.746277 -0.494388 0.183597
C -5.436840 -1.428439 1.138025
C -5.329246 0.504672 -0.513727
C -6.776772 0.950056 -0.551011
C -7.246916 1.669676 0.726821
C -6.413588 2.898617 1.069570
C -7.741768 -0.170088 -0.951344
H -1.415388 -3.411134 -0.338538
H -2.666407 1.287719 0.155670
H -0.303510 1.987062 -0.015515
H 1.985988 2.725925 -0.095347
H 2.062932 -3.123807 -0.627818
H 4.130525 0.056084 -2.569529
H 6.164142 0.477978 0.284790
H 5.740520 -1.222949 0.250815
H 5.467306 0.069693 3.889533
H 6.613284 -0.822007 2.860676
H 6.466648 0.959514 2.720486
H 6.078063 2.209441 -1.635390
H 4.559018 2.833808 -2.377944
H 5.596548 3.893670 -1.408184
H -4.835419 -1.573001 2.043220
H -6.403602 -1.052760 1.475996
H -5.599815 -2.405849 0.671594
H -4.704242 1.084794 -1.194498
H -6.835705 1.687880 -1.365032
H -8.287786 1.989971 0.591477
H -7.243677 0.989378 1.584858
H -6.395654 3.606062 0.234469
H -6.841207 3.411205 1.937304
H -5.382572 2.629211 1.318137
H -7.383948 -0.699786 -1.841137
H -8.726874 0.246605 -1.189910
H -7.887361 -0.904008 -0.154408
SCF Energy (B3LYP/6-31G**)= -1361.69526365
Number of imaginary frequencies = 0

1a_c235
MMFF Geometry
C 1.898258 1.996783 0.043870
C 2.231671 -0.698667 0.101419
N 3.148423 1.511585 0.236125
C 0.756447 1.210660 -0.128001
C 0.940876 -0.183838 -0.098925
C 3.310611 0.168787 0.266258
C -0.168397 -1.031193 -0.279570
C -1.455585 -0.510892 -0.469113
C -1.633270 0.875121 -0.479407
C -0.536203 1.734849 -0.328580
C -3.003846 1.405976 -0.656932
C -4.189236 0.470018 -0.326348
C -3.850558 -0.971083 -0.692110
C -2.580287 -1.414473 -0.724216
O -0.679566 3.101010 -0.367271
O -3.130742 2.581571 -1.004368
O -5.325749 0.907411 -1.079932
C -4.509137 0.669251 1.159736
C -5.475233 -0.357077 1.706879
C -6.935808 -0.200684 1.373377
O -5.072388 -1.298134 2.394599
O -4.965715 -1.742241 -0.964011
C -4.807241 -3.153323 -1.063123
C 4.697968 -0.328459 0.497771
C 4.825313 -1.645502 1.210891
C 5.741847 0.428548 0.092061
C 7.233027 0.176659 0.165580
C 7.725237 -1.166816 -0.407132
C 7.306080 -1.397189 -1.853654
C 7.767504 0.410017 1.580967
H 1.836665 3.082923 0.032085
H 2.387336 -1.772533 0.113114
H -0.020143 -2.110679 -0.272175
H -2.332429 -2.442952 -0.963218
H -1.597262 3.314322 -0.654101
H -5.182924 0.624286 -2.000043
H -4.940179 1.662161 1.340454
H -3.599616 0.614509 1.772387
H -7.529072 -0.840984 2.032496
H -7.244133 0.835198 1.535405
H -7.120015 -0.491223 0.337605
H -4.209101 -3.416725 -1.941044
H -4.366607 -3.564238 -0.148946
H -5.800427 -3.594717 -1.187634
H 4.670890 -2.477281 0.515798
H 5.799716 -1.777730 1.684133
H 4.092139 -1.726120 2.021773
H 5.512761 1.384110 -0.386728
H 7.692829 0.954490 -0.462248
H 8.822076 -1.183888 -0.365732
H 7.389557 -2.011070 0.202340
H 7.637001 -0.573505 -2.494163
H 7.756980 -2.320458 -2.231644
H 6.220695 -1.495620 -1.948577
H 7.456053 1.388852 1.962676
H 8.862854 0.387375 1.586129
H 7.418427 -0.351374 2.284668
SCF Energy (B3LYP/6-31G**)= -1361.69752104
Number of imaginary frequencies = 0

1a_c236
MMFF Geometry
C -1.369312 -2.189979 -0.557528
C -2.241149 0.313020 0.046506
N -2.698730 -2.008897 -0.366577
C -0.404953 -1.181231 -0.467946
C -0.867714 0.110640 -0.153132
C -3.121323 -0.760529 -0.074086
C 0.052879 1.170288 -0.052223
C 1.424703 0.959888 -0.246269
C 1.878092 -0.328859 -0.543454
C 0.973146 -1.391627 -0.673580
C 3.329066 -0.528247 -0.744906
C 4.292762 0.508777 -0.121225
C 3.683902 1.909069 -0.161008
C 2.351162 2.096121 -0.201995
O 1.387653 -2.658579 -1.006155
O 3.704683 -1.519069 -1.373737

O 5.513041 0.512493 -0.874009
C 4.651785 0.146593 1.321671
C 4.899007 -1.333639 1.497446
C 6.228539 -1.887716 1.059966
O 4.015449 -2.059970 1.956360
O 4.628178 2.918319 -0.125895
C 4.185756 4.270673 -0.125917
C -4.581716 -0.608343 0.133678
C -5.195743 -1.698133 0.967981
C -5.243243 0.429895 -0.422178
C -6.722421 0.763819 -0.399194
C -7.130852 1.391831 0.945626
C -8.552728 1.937135 0.950873
C -7.620614 -0.397823 -0.834874
H -1.085620 -3.213654 -0.793319
H -2.620707 1.296152 0.307027
H -0.312474 2.170540 0.177704
H 1.904877 3.084573 -0.209199
H 2.352157 -2.636727 -1.200168
H 5.320109 0.940370 -1.726349
H 3.840838 0.414081 2.012096
H 5.546510 0.681810 1.662894
H 6.357816 -2.891624 1.474265
H 6.270719 -1.943755 -0.029473
H 7.036085 -1.255298 1.437085
H 3.634160 4.501171 -1.042950
H 3.580421 4.483668 0.760971
H 5.070027 4.913929 -0.093357
H -4.540983 -1.965270 1.805893
H -6.145251 -1.399803 1.416198
H -5.367397 -2.595607 0.364512
H -4.671644 1.138661 -1.023210
H -6.854005 1.539752 -1.167893
H -7.033501 0.663972 1.758906
H -6.446598 2.216774 1.182967
H -9.289495 1.135320 0.845372
H -8.754382 2.449320 1.897248
H -8.701459 2.655866 0.138935
H -7.198731 -0.923237 -1.698840
H -8.607441 -0.027985 -1.134199
H -7.779135 -1.126015 -0.034985
SCF Energy (B3LYP/6-31G**)= -1361.69359114
Number of imaginary frequencies = 0

1a_c237
MMFF Geometry
C -1.583670 -1.750418 -1.067696
C -2.309385 0.731211 -0.228837
N -2.906832 -1.472913 -0.974607
C -0.555521 -0.852105 -0.764925
C -0.943479 0.431112 -0.334231
C -3.255831 -0.240698 -0.547136
C 0.043125 1.386349 -0.027794
C 1.405828 1.077996 -0.129344
C 1.784264 -0.204769 -0.540974
C 0.815376 -1.160897 -0.874994
C 3.227459 -0.506456 -0.652529
C 4.234605 0.382618 0.104021
C 3.712890 1.802003 0.221610
C 2.407927 2.107167 0.151711
O 1.157474 -2.414456 -1.321342
O 3.603060 -1.456933 -1.345530
O 5.441099 0.396722 -0.678872
C 4.568320 -0.154671 1.497613
C 4.683441 -1.659491 1.527782
C 6.025796 -2.268025 1.223210
O 3.694178 -2.357024 1.755080
O 4.635689 2.770441 0.587302
C 5.058801 3.532552 -0.543856
C -4.713011 0.022086 -0.461435
C -5.494660 -0.491582 -1.638404
C -5.226417 0.647997 0.619815
C -6.665056 0.979436 0.960371
C -7.652623 -0.203348 0.940820
C -7.234184 -1.355018 1.846233
C -7.181615 2.153414 0.124563
H -1.360690 -2.759606 -1.407792
H -2.633157 1.718697 0.085343
H -0.261071 2.382401 0.292281
H 2.072407 3.125634 0.324421
H 2.134577 -2.465554 -1.413779
H 5.400393 -0.381537 -1.271236
H 3.787656 0.109214 2.223628
H 5.506762 0.268228 1.876763
H 5.993569 -3.342484 1.424711
H 6.276585 -2.111575 0.172279
H 6.788500 -1.819549 1.864573
H 5.497526 2.895980 -1.318466
H 4.225532 4.110282 -0.957868
H 5.825438 4.236199 -0.206476
H -6.475550 -0.022982 -1.732184
H -4.969399 -0.279761 -2.577172
H -5.641246 -1.574128 -1.561246
H -4.539241 0.940794 1.415255
H -6.645608 1.337095 2.000680
H -8.634101 0.153972 1.278421
H -7.804003 -0.582699 -0.074225
H -7.083334 -1.011157 2.874442
H -8.013475 -2.123886 1.858563
H -6.309726 -1.825790 1.498935
H -6.496332 3.006753 0.179411
H -8.156293 2.488788 0.495899
H -7.304159 1.888289 -0.929573
SCF Energy (B3LYP/6-31G**)= -1361.69415715
Number of imaginary frequencies = 0

1a_c238
MMFF Geometry
C -1.697812 -2.432928 0.098674
C -2.323095 0.210005 -0.014660
N -2.999380 -2.081824 0.232831
C -0.643666 -1.534113 -0.093221
C -0.979250 -0.168351 -0.148859
C -3.299892 -0.767012 0.166580
C 0.036592 0.785906 -0.341933
C 1.378881 0.402443 -0.468302
C 1.706212 -0.953558 -0.400653
C 0.704821 -1.920603 -0.229676
C 3.128072 -1.339127 -0.517563
C 4.213452 -0.268226 -0.298731
C 3.716651 1.120262 -0.671928
C 2.404591 1.415914 -0.724775
O 0.996010 -3.263010 -0.184377
O 3.417893 -2.520055 -0.735210
O 5.301895 -0.641018 -1.160360
C 4.671391 -0.393093 1.158771
C 5.579332 0.726553 1.617261
C 7.002959 0.729385 1.123836
O 5.165287 1.597010 2.386155
O 4.737716 2.020564 -0.917322
C 4.410987 3.381659 -1.166809
C -4.734767 -0.426735 0.322246
C -5.425167 -1.181780 1.423981
C -5.312406 0.476388 -0.499477
C -6.753864 0.942392 -0.571660
C -7.069555 1.951878 0.546834
C -8.435036 2.609773 0.399353
C -7.765099 -0.203714 -0.670763
H -1.515696 -3.504165 0.153479
H -2.604544 1.258391 -0.037577
H -0.229046 1.841403 -0.395468
H 2.049809 2.410651 -0.971365
H 1.941020 -3.391341 -0.424936
H 5.286756 -1.616531 -1.228216
H 5.205585 -1.337319 1.324837
H 3.807440 -0.405496 1.836720
H 7.579764 1.471242 1.683680
H 7.452267 -0.252709 1.291284
H 7.039803 0.984075 0.063566
H 3.826050 3.480543 -2.086704
H 3.881298 3.819664 -0.314716
H 5.346380 3.932719 -1.301576

H -4.777066 -1.269132 2.304040
H -6.331969 -0.683092 1.771082
H -5.693122 -2.190094 1.091417
H -4.693664 0.936020 -1.271722
H -6.833010 1.485757 -1.524957
H -7.018956 1.469997 1.529642
H -6.307035 2.741579 0.548007
H -9.244433 1.883439 0.518421
H -8.565395 3.379236 1.167178
H -8.537578 3.088161 -0.579714
H -7.414623 -0.984091 -1.355351
H -8.721069 0.161301 -1.062031
H -7.969701 -0.665276 0.298934
SCF Energy (B3LYP/6-31G**)= -1361.70224349
Number of imaginary frequencies = 0

1a_c239
MMFF Geometry
C 1.758761 -1.676277 0.186528
C 2.312270 0.960540 -0.154604
N 3.058554 -1.293644 0.170752
C 0.670800 -0.812097 0.039067
C 0.969562 0.550187 -0.138934
C 3.330025 0.020966 0.002904
C -0.081976 1.469730 -0.303371
C -1.420953 1.054750 -0.279006
C -1.715164 -0.297579 -0.091337
C -0.676021 -1.230674 0.053694
C -3.134903 -0.724471 -0.083784
C -4.241650 0.335113 0.155350
C -3.767162 1.719346 -0.267540
C -2.483591 2.031880 -0.489184
O -0.924374 -2.574010 0.211172
O -3.372703 -1.926948 -0.228734
O -4.506952 0.417371 1.557942
C -5.499867 -0.070464 -0.640897
C -6.134953 -1.343175 -0.116195
C -6.767692 -2.261512 -1.125981
O -6.178427 -1.583328 1.092449
O -4.730353 2.694500 -0.502728
C -5.259137 3.212352 0.720464
C 4.770286 0.408941 0.009015
C 5.090760 1.782242 0.529601
C 5.693404 -0.486386 -0.407595
C 7.198156 -0.364437 -0.539136
C 7.890755 -0.547704 0.823308
C 9.407378 -0.641615 0.722825
C 7.653161 0.886169 -1.297683
H 1.608682 -2.744491 0.327773
H 2.553548 2.007933 -0.303921
H 0.150140 2.524107 -0.450313
H -2.213983 3.034153 -0.809168
H -1.890378 -2.728821 0.106850
H -4.990033 -0.402230 1.806023
H -5.247085 -0.210315 -1.699003
H -6.286169 0.690781 -0.578597
H -7.173301 -3.141878 -0.619935
H -7.579502 -1.738540 -1.637158
H -6.014551 -2.587917 -1.847393
H -5.965031 2.506550 1.167775
H -4.463171 3.465327 1.429544
H -5.807500 4.129073 0.484042
H 6.117123 1.865511 0.892061
H 4.941530 2.535672 -0.250627
H 4.457258 2.036647 1.387257
H 5.333388 -1.468461 -0.724953
H 7.500730 -1.213516 -1.169884
H 7.635982 0.275237 1.500663
H 7.521246 -1.466352 1.297245
H 9.844596 0.298601 0.373957
H 9.835472 -0.860713 1.706365
H 9.707493 -1.440653 0.037688
H 7.015207 1.077084 -2.167737
H 8.675048 0.756719 -1.670958
H 7.652370 1.780357 -0.668712
SCF Energy (B3LYP/6-31G**)= -1361.70110172
Number of imaginary frequencies = 0

1a_c240
MMFF Geometry
C -1.487400 -2.172607 -0.604667
C -2.375399 0.306992 0.067613
N -2.820514 -2.000282 -0.432695
C -0.526627 -1.166333 -0.462781
C -0.998201 0.113479 -0.112932
C -3.251042 -0.762991 -0.106448
C -0.082397 1.170235 0.043059
C 1.292525 0.968836 -0.134041
C 1.755519 -0.308814 -0.467795
C 0.855863 -1.368149 -0.649180
C 3.209152 -0.497886 -0.661186
C 4.181281 0.534236 -0.055548
C 3.544108 1.910104 -0.014540
C 2.215384 2.098351 -0.011907
O 1.278233 -2.623708 -1.013616
O 3.620845 -1.470475 -1.300955
O 5.326711 0.580689 -0.924202
C 4.653858 0.149533 1.348324
C 4.904401 -1.332186 1.491228
C 6.272336 -1.851017 1.138279
O 3.998648 -2.087286 1.846630
O 4.399218 2.980061 0.202565
C 4.676511 3.673557 -1.014606
C -4.715397 -0.618377 0.080038
C -5.340746 -1.735979 0.867597
C -5.365003 0.437347 -0.456330
C -6.839417 0.785158 -0.437329
C -7.356360 1.260132 0.933405
C -6.607856 2.472191 1.474570
C -7.726670 -0.319412 -1.019488
H -1.197630 -3.187281 -0.869860
H -2.761450 1.280301 0.354653
H -0.452194 2.161446 0.303538
H 1.802687 3.096460 0.102674
H 2.247256 -2.600710 -1.175932
H 5.316113 -0.246756 -1.447139
H 3.903010 0.409554 2.106470
H 5.574088 0.680352 1.621604
H 6.349516 -2.902897 1.427581
H 6.437519 -1.765963 0.062497
H 7.033108 -1.286706 1.683232
H 5.117852 3.010860 -1.765452
H 3.769338 4.141644 -1.411405
H 5.398001 4.465309 -0.792666
H -4.730188 -1.981992 1.744340
H -6.330314 -1.483914 1.251271
H -5.437932 -2.635628 0.250641
H -4.781341 1.158209 -1.031069
H -6.948646 1.637034 -1.124981
H -8.416596 1.527476 0.839906
H -7.306574 0.454234 1.673061
H -6.638344 3.302616 0.762068
H -7.068622 2.811045 2.408079
H -5.560821 2.236378 1.687806
H -7.334375 -0.676310 -1.978171
H -8.737850 0.062982 -1.199128
H -7.821807 -1.178398 -0.350133
SCF Energy (B3LYP/6-31G**)= -1361.69423963
Number of imaginary frequencies = 0

1a_c241
MMFF Geometry
C 1.964203 1.790239 0.101281
C 2.173795 -0.888050 -0.301614
N 3.197999 1.232320 0.142575
C 0.779950 1.087728 -0.133312
C 0.899954 -0.298595 -0.340766
C 3.299214 -0.101881 -0.058164
C -0.255997 -1.062346 -0.589131
C -1.525256 -0.469276 -0.617899
C -1.637655 0.905786 -0.395992
C -0.495043 1.686742 -0.172371

C -2.988830 1.510758 -0.406923
C -4.199047 0.579913 -0.163387
C -3.947543 -0.794973 -0.772982
C -2.702863 -1.275778 -0.948469
O -0.575693 3.045284 0.018200
O -3.077334 2.731254 -0.553297
O -5.349655 1.177149 -0.771927
C -4.434855 0.548062 1.351153
C -5.421501 -0.513889 1.781134
C -6.887435 -0.247292 1.559937
O -5.031445 -1.569335 2.285820
O -5.110136 -1.468706 -1.099584
C -5.023788 -2.850912 -1.428461
C 4.675863 -0.676905 -0.022148
C 4.913839 -1.894967 -0.870218
C 5.621655 -0.066195 0.726086
C 7.073289 -0.422121 0.968389
C 8.001409 -0.106896 -0.219325
C 7.971228 1.357831 -0.638537
C 7.273423 -1.855741 1.470050
H 1.953179 2.864984 0.269871
H 2.275863 -1.958474 -0.447383
H -0.158686 -2.133626 -0.762958
H -2.516314 -2.260869 -1.362127
H -1.495479 3.337675 -0.177870
H -5.266599 1.040881 -1.731970
H -4.807629 1.514945 1.712411
H -3.499802 0.358340 1.894521
H -7.477833 -0.960704 2.142053
H -7.136486 0.760681 1.901008
H -7.136661 -0.360097 0.503369
H -4.483912 -2.993628 -2.369768
H -4.557439 -3.419313 -0.617183
H -6.041761 -3.228002 -1.563680
H 4.415014 -1.800427 -1.841711
H 5.969113 -2.054220 -1.097350
H 4.537945 -2.793308 -0.369641
H 5.325836 0.832585 1.273433
H 7.397245 0.221163 1.800119
H 9.032311 -0.361474 0.057643
H 7.752882 -0.728220 -1.086303
H 8.217619 2.012253 0.203612
H 8.705419 1.536049 -1.430858
H 6.988948 1.645409 -1.025534
H 6.577118 -2.090772 2.282569
H 8.289673 -1.980295 1.861010
H 7.140481 -2.600403 0.681006
SCF Energy (B3LYP/6-31G**)= -1361.69772974
Number of imaginary frequencies = 0

1a_c242
MMFF Geometry
C -2.013818 -1.607982 -0.282796
C -2.301897 1.072476 0.042109
N -3.266295 -1.112182 -0.137855
C -0.847199 -0.839709 -0.276057
C -1.008270 0.546349 -0.108355
C -3.407660 0.223502 0.023972
C 0.128801 1.375056 -0.100089
C 1.419870 0.844985 -0.244317
C 1.574005 -0.533776 -0.402485
C 0.448858 -1.373230 -0.429907
C 2.937590 -1.085655 -0.552706
C 4.153682 -0.199466 -0.224883
C 3.834426 1.286881 -0.248871
C 2.571190 1.749038 -0.255397
O 0.562133 -2.732432 -0.602201
O 3.066084 -2.260884 -0.910233
O 5.085521 -0.476669 -1.281720
C 4.708466 -0.623283 1.144622
C 6.195161 -0.893330 1.139935
C 7.134004 0.282591 1.144374
O 6.621661 -2.049428 1.107216
O 4.963605 2.086202 -0.232055
C 4.815924 3.485860 -0.438200
C -4.801197 0.728281 0.195249
C -4.969946 2.016673 0.954362
C -5.831441 0.008190 -0.301732
C -7.308481 0.331388 -0.260050
C -8.087577 -0.691053 0.587793
C -7.702901 -0.667379 2.061845
C -7.858051 0.352389 -1.690503
H -1.971974 -2.687759 -0.409469
H -2.435710 2.142897 0.158241
H -0.001196 2.450260 0.020383
H 2.350449 2.810882 -0.257737
H 1.500439 -2.950429 -0.805747
H 5.146965 -1.451442 -1.353617
H 4.226619 -1.546315 1.495255
H 4.488927 0.112590 1.928515
H 8.117580 -0.040746 1.497130
H 7.231646 0.685330 0.134493
H 6.765816 1.052525 1.826957
H 4.322000 3.692805 -1.393085
H 4.273198 3.946350 0.393294
H 5.816126 3.927733 -0.472296
H -4.731331 2.873699 0.316010
H -5.983354 2.157447 1.338029
H -4.316312 2.040704 1.833954
H -5.604433 -0.928899 -0.815168
H -7.483321 1.327830 0.157247
H -9.161397 -0.479030 0.510913
H -7.939126 -1.705205 0.195641
H -7.834052 0.333560 2.485055
H -8.337345 -1.358641 2.625890
H -6.663271 -0.973526 2.212093
H -7.325244 1.087104 -2.304667
H -8.919143 0.624616 -1.693168
H -7.761753 -0.626145 -2.174688
SCF Energy (B3LYP/6-31G**)= -1361.70512429
Number of imaginary frequencies = 0

1a_c243
MMFF Geometry
C 1.757341 2.331675 0.117146
C 2.290922 -0.260775 -0.501178
N 3.054760 1.945236 0.064401
C 0.663801 1.493496 -0.122542
C 0.952362 0.154171 -0.444800
C 3.308505 0.653065 -0.234199
C -0.104307 -0.736003 -0.710966
C -1.440495 -0.317853 -0.644312
C -1.720208 1.008705 -0.308921
C -0.679384 1.916491 -0.064505
C -3.134820 1.428004 -0.224739
C -4.227595 0.349776 -0.097943
C -3.807347 -0.956080 -0.755369
C -2.514455 -1.255174 -0.980531
O -0.925338 3.234039 0.239563
O -3.407797 2.632571 -0.204039
O -5.377426 0.884679 -0.774573
C -4.549248 0.218297 1.394989
C -5.443583 -0.954398 1.731296
C -6.905463 -0.851849 1.381191
O -4.986716 -1.953290 2.291491
O -4.872459 -1.783988 -1.061041
C -4.609738 -3.082090 -1.578229
C 4.741489 0.272412 -0.285951
C 5.611748 1.277096 -0.988960
C 5.158322 -0.875557 0.291288
C 6.553445 -1.458622 0.406969
C 7.537250 -0.527730 1.140841
C 8.844806 -1.213837 1.516288
C 7.071899 -1.964524 -0.941361
H 1.613000 3.380425 0.368162
H 2.538949 -1.284560 -0.764324
H 0.124139 -1.768576 -0.973851
H -2.212513 -2.194334 -1.431075
H -1.884038 3.415187 0.115255
H -5.339025 1.856423 -0.671131
H -5.039509 1.124059 1.774110
H -3.628023 0.099366 1.980973

H -7.045075 -0.910681 0.300707
H -7.450037 -1.675854 1.851229
H -7.309922 0.089047 1.762702
H -4.111786 -3.021419 -2.551113
H -4.019912 -3.673476 -0.870524
H -5.569363 -3.587320 -1.721935
H 5.773077 2.157350 -0.357522
H 6.590058 0.878378 -1.260192
H 5.146318 1.603964 -1.926205
H 4.414110 -1.491995 0.798237
H 6.439661 -2.350332 1.041176
H 7.775729 0.352253 0.535311
H 7.068210 -0.157789 2.061584
H 9.417934 -1.496916 0.628593
H 9.466840 -0.535672 2.109462
H 8.660959 -2.112537 2.113335
H 6.305574 -2.547791 -1.464472
H 7.937566 -2.620870 -0.804257
H 7.380161 -1.147240 -1.599248
SCF Energy (B3LYP/6-31G**)= -1361.70251172
Number of imaginary frequencies = 0

1a_c244
MMFF Geometry
C -1.994560 -1.695883 -0.553609
C -2.339992 0.990682 -0.362057
N -3.260574 -1.215189 -0.512471
C -0.841525 -0.908958 -0.501957
C -1.032215 0.479881 -0.401761
C -3.430304 0.123647 -0.418710
C 0.090406 1.326274 -0.346366
C 1.395300 0.811766 -0.380440
C 1.578341 -0.569345 -0.474174
C 0.469061 -1.427141 -0.545641
C 2.956252 -1.104743 -0.508690
C 4.134867 -0.187566 -0.132785
C 3.798251 1.291576 -0.235453
C 2.532480 1.732985 -0.346679
O 0.612100 -2.790031 -0.655282
O 3.124750 -2.290461 -0.810662
O 5.140870 -0.489051 -1.112151
C 4.599648 -0.551083 1.286660
C 6.086589 -0.797510 1.393732
C 7.007134 0.392541 1.418267
O 6.529707 -1.947125 1.433348
O 4.912794 2.108842 -0.171288
C 4.760974 3.497663 -0.438598
C -4.838553 0.614823 -0.392895
C -5.082493 2.012039 -0.895592
C -5.817964 -0.206255 0.046534
C -7.295410 0.087889 0.181037
C -7.802235 -0.213538 1.605155
C -7.173508 0.681214 2.666430
C -8.070674 -0.747941 -0.840266
H -1.929631 -2.779076 -0.632328
H -2.495984 2.060766 -0.273616
H -0.061932 2.403030 -0.276621
H 2.298317 2.790362 -0.404637
H 1.564970 -3.000614 -0.785928
H 5.220713 -1.464695 -1.141722
H 4.107114 -1.467891 1.638765
H 4.316953 0.209985 2.024937
H 7.968237 0.097977 1.849448
H 7.168844 0.759570 0.403016
H 6.582492 1.180991 2.044611
H 4.331700 3.662294 -1.432183
H 4.155774 3.978985 0.336030
H 5.755108 3.953923 -0.420105
H -4.491335 2.213073 -1.796481
H -6.122543 2.186113 -1.182738
H -4.816998 2.748325 -0.129882
H -5.541873 -1.213572 0.366210
H -7.507755 1.141909 -0.023415
H -8.889808 -0.071742 1.637692
H -7.614809 -1.263222 1.864578
H -7.339808 1.738391 2.436228
H -7.619929 0.473657 3.644234
H -6.095900 0.510811 2.750140
H -7.740355 -0.525267 -1.861114
H -9.142977 -0.531349 -0.784019
H -7.935554 -1.821906 -0.668366
SCF Energy (B3LYP/6-31G**)= -1361.70504552
Number of imaginary frequencies = 0

1a_c245
MMFF Geometry
C -1.598469 -2.366879 -0.270088
C -2.304311 0.241037 0.014742
N -2.914389 -2.078305 -0.121696
C -0.568636 -1.420277 -0.291033
C -0.946573 -0.074400 -0.141991
C -3.253935 -0.778631 0.012406
C 0.043053 0.924204 -0.152656
C 1.400752 0.605700 -0.310099
C 1.776892 -0.735116 -0.451617
C 0.795783 -1.742547 -0.447980
C 3.213374 -1.076554 -0.623574
C 4.272693 0.049299 -0.564103
C 3.693553 1.442034 -0.454831
C 2.383686 1.687865 -0.318401
O 1.116715 -3.071657 -0.602509
O 3.510921 -2.251908 -0.858528
O 5.006496 -0.008562 -1.806496
C 5.292584 -0.265171 0.537584
C 4.729111 -0.139182 1.935175
C 5.719442 0.127645 3.037732
O 3.533700 -0.298317 2.180239
O 4.615597 2.475270 -0.367410
C 4.694211 3.218799 -1.583573
C -4.701756 -0.504772 0.182350
C -5.389100 -1.441582 1.136402
C -5.286148 0.499245 -0.506609
C -6.732690 0.948601 -0.533073
C -7.193049 1.663726 0.750828
C -6.354479 2.889032 1.093804
C -7.703148 -0.167155 -0.932463
H -1.382397 -3.427974 -0.375535
H -2.617227 1.271508 0.151961
H -0.254182 1.965551 -0.032447
H 2.031037 2.705060 -0.175102
H 2.092695 -3.147334 -0.707033
H 4.355094 -0.014695 -2.528294
H 6.154108 0.409043 0.446966
H 5.689865 -1.281963 0.428421
H 5.195472 0.198916 3.994904
H 6.440810 -0.691565 3.087478
H 6.233212 1.073333 2.848125
H 4.958133 2.574591 -2.428216
H 3.753656 3.741867 -1.785417
H 5.481915 3.968985 -1.467920
H -4.782367 -1.592656 2.036977
H -6.352487 -1.064613 1.482493
H -5.558094 -2.416076 0.666030
H -4.663660 1.080883 -1.188366
H -6.794617 1.690205 -1.343429
H -8.233923 1.987236 0.623401
H -7.186183 0.979609 1.605805
H -6.339975 3.600144 0.261755
H -6.775362 3.398822 1.966467
H -5.322614 2.615938 1.334705
H -7.352129 -0.693959 -1.826670
H -8.688509 0.253269 -1.163308
H -7.846014 -0.904121 -0.137848
SCF Energy (B3LYP/6-31G**)= -1361.69495697
Number of imaginary frequencies = 0

1a_c246
MMFF Geometry
C -1.440918 -2.001397 -0.824205
C -2.205194 0.563982 -0.351627
N -2.768109 -1.736406 -0.753187
C -0.427346 -1.049772 -0.673826

C -0.834894 0.275968 -0.432309
C -3.136341 -0.461094 -0.507521
C 0.136929 1.283582 -0.288117
C 1.504689 0.987660 -0.362162
C 1.901827 -0.334511 -0.583292
C 0.947632 -1.346547 -0.758434
C 3.349639 -0.624164 -0.658698
C 4.320914 0.376050 0.011648
C 3.802892 1.807021 -0.119023
C 2.491503 2.069282 -0.275001
O 1.310085 -2.645254 -1.022264
O 3.714159 -1.655758 -1.225441
O 5.595757 0.283619 -0.637995
C 4.540299 0.043087 1.489139
C 4.682758 -1.442101 1.727855
C 6.007203 -2.086732 1.417476
O 3.722951 -2.100026 2.133713
O 4.800507 2.760619 -0.035994
C 4.442101 4.135464 -0.113165
C -4.597619 -0.213104 -0.440772
C -5.381756 -0.912438 -1.516423
C -5.108943 0.559776 0.542078
C -6.548914 0.925467 0.845809
C -7.440431 -0.302895 1.109096
C -8.800191 0.057798 1.694206
C -7.121596 1.891990 -0.193711
H -1.201866 -3.045805 -1.014096
H -2.544896 1.581176 -0.182944
H -0.184620 2.310553 -0.118432
H 2.107751 3.081054 -0.347977
H 2.286890 -2.686075 -1.134423
H 5.498223 0.692940 -1.515339
H 3.694128 0.380369 2.102414
H 5.435391 0.537000 1.886989
H 6.041927 -3.081723 1.870218
H 6.133224 -2.182237 0.337294
H 6.818601 -1.489582 1.840967
H 3.981151 4.365335 -1.079096
H 3.781252 4.413898 0.713950
H 5.358140 4.727272 -0.027087
H -5.459467 -1.983935 -1.303241
H -6.393710 -0.521456 -1.629216
H -4.897277 -0.787762 -2.491937
H -4.417463 0.973944 1.277665
H -6.508710 1.486927 1.791038
H -7.605445 -0.875930 0.191317
H -6.932735 -0.975413 1.812366
H -9.403624 0.629095 0.982799
H -9.354851 -0.853008 1.942146
H -8.691297 0.646739 2.610295
H -6.414171 2.699972 -0.412375
H -8.040447 2.360125 0.174436
H -7.362459 1.391525 -1.135683
SCF Energy (B3LYP/6-31G**)= -1361.69393717
Number of imaginary frequencies = 0

1a_c247
MMFF Geometry
C 1.448031 2.496404 0.323888
C 2.233815 0.028209 -0.496957
N 2.777017 2.233073 0.287356
C 0.442743 1.591318 -0.031207
C 0.861955 0.317483 -0.457604
C 3.155588 0.999828 -0.112166
C -0.100274 -0.633667 -0.842560
C -1.470063 -0.341346 -0.792752
C -1.882203 0.920233 -0.355257
C -0.934780 1.888670 0.010199
C -3.332998 1.208070 -0.293759
C -4.345452 0.043846 -0.387030
C -3.761805 -1.176647 -1.082498
C -2.440705 -1.331888 -1.254892
O -1.306105 3.150111 0.413235
O -3.712344 2.376550 -0.152030
O -5.407272 0.564060 -1.221965
C -4.959980 -0.173760 1.005722
C -4.078716 -0.932714 1.970391
C -4.770542 -1.833571 2.959263
O -2.860458 -0.773077 2.003735
O -4.572912 -2.106479 -1.724235
C -5.781557 -2.490487 -1.089190
C 4.617327 0.753507 -0.139306
C 5.429593 1.900545 -0.677467
C 5.125997 -0.411071 0.317158
C 6.585842 -0.805495 0.404744
C 6.867949 -1.932612 -0.604999
C 8.338001 -2.320827 -0.685106
C 6.909744 -1.217905 1.844040
H 1.200144 3.500554 0.661031
H 2.579762 -0.942558 -0.838069
H 0.226399 -1.615179 -1.183848
H -2.065751 -2.195347 -1.797994
H -2.278945 3.238530 0.303713
H -5.470385 1.520838 -1.027100
H -5.917945 -0.697792 0.920237
H -5.205152 0.785570 1.480068
H -4.026059 -2.337965 3.581522
H -5.425506 -1.237819 3.599609
H -5.350189 -2.591029 2.426620
H -5.595728 -2.829428 -0.066329
H -6.521829 -1.687381 -1.113233
H -6.197380 -3.335199 -1.646933
H 5.546095 2.680000 0.082838
H 6.425880 1.595380 -1.007502
H 4.944577 2.342920 -1.555608
H 4.442163 -1.165633 0.707236
H 7.230501 0.045870 0.164545
H 6.277707 -2.824011 -0.356851
H 6.546085 -1.612121 -1.604499
H 8.684946 -2.775903 0.247049
H 8.489120 -3.052393 -1.485516
H 8.963672 -1.449150 -0.901120
H 6.624970 -0.429876 2.550701
H 7.982144 -1.395054 1.972818
H 6.379267 -2.133396 2.129265
SCF Energy (B3LYP/6-31G**)= -1361.69711075
Number of imaginary frequencies = 0

1a_c248
MMFF Geometry
C 1.744933 -1.853472 -0.266071
C 2.376768 0.786561 -0.360566
N 3.055519 -1.511292 -0.301943
C 0.683530 -0.945367 -0.274157
C 1.022071 0.419129 -0.323522
C 3.366458 -0.195412 -0.348100
C -0.002327 1.384183 -0.341542
C -1.352494 1.010681 -0.295882
C -1.679897 -0.344956 -0.230928
C -0.674186 -1.321888 -0.239077
C -3.110251 -0.714347 -0.184797
C -4.114160 0.339552 0.342631
C -3.685296 1.740012 -0.105796
C -2.399244 2.031822 -0.380911
O -0.970193 -2.663938 -0.216075
O -3.423232 -1.856272 -0.528938
O -4.074360 0.364658 1.772902
C -5.530637 -0.003326 -0.163142
C -6.038835 -1.318673 0.394094
C -6.954962 -2.126413 -0.484419
O -5.758357 -1.679639 1.539407
O -4.725062 2.653891 -0.149169
C -4.418180 4.026911 -0.360429
C 4.818710 0.143054 -0.401015
C 5.183972 1.410150 -1.122217
C 5.709811 -0.703027 0.162467
C 7.215968 -0.613018 0.287655
C 7.771708 0.692035 0.890300
C 7.191076 1.020646 2.260000
C 7.905785 -0.955718 -1.035279
H 1.562973 -2.925396 -0.228525
H 2.648706 1.836704 -0.384276

H 0.261537 2.439876 -0.391497
H -2.080679 3.026604 -0.670108
H -1.942709 -2.771769 -0.317533
H -4.517735 -0.454006 2.086058
H -5.538637 -0.055672 -1.258484
H -6.266277 0.746269 0.152233
H -7.236245 -3.050610 0.027828
H -7.857414 -1.549269 -0.699648
H -6.439845 -2.383461 -1.413272
H -3.747433 4.403977 0.418370
H -3.991760 4.182234 -1.356512
H -5.352832 4.592627 -0.303296
H 6.221994 1.421241 -1.458755
H 4.580498 1.534467 -2.028820
H 5.028398 2.280489 -0.476327
H 5.315160 -1.608834 0.630101
H 7.499687 -1.410344 0.990779
H 8.859459 0.591769 0.998941
H 7.614946 1.542979 0.220804
H 7.341204 0.191842 2.959114
H 7.686010 1.905042 2.674124
H 6.120133 1.236548 2.203154
H 7.542425 -1.910089 -1.432729
H 8.987799 -1.049188 -0.890909
H 7.739703 -0.189301 -1.798022
SCF Energy (B3LYP/6-31G**)= -1361.70732305
Number of imaginary frequencies = 0

1a_c249
MMFF Geometry
C -1.863223 -1.522442 -0.547869
C -2.114648 1.087600 0.155599
N -3.109588 -1.032930 -0.342457
C -0.685035 -0.781929 -0.424106
C -0.827936 0.567705 -0.060737
C -3.232719 0.268029 0.008452
C 0.320583 1.366932 0.078044
C 1.604306 0.844171 -0.138138
C 1.744614 -0.499006 -0.498235
C 0.605486 -1.309338 -0.641280
C 3.104413 -1.053277 -0.713264
C 4.339910 -0.143189 -0.573186
C 4.009849 1.282332 -0.196185
C 2.763524 1.714110 0.031209
O 0.695214 -2.636367 -0.990960
O 3.216774 -2.240855 -1.036323
O 4.875792 -0.132079 -1.916127
C 5.381857 -0.807976 0.338355
C 5.101900 -0.683446 1.821529
C 3.932314 -1.442026 2.391023
O 5.814448 0.006980 2.550657
O 5.056113 2.156005 0.066128
C 5.816021 2.485845 -1.095125
C -4.620116 0.765055 0.241574
C -4.775518 1.936948 1.173023
C -5.657191 0.137903 -0.356882
C -7.129547 0.475292 -0.277936
C -7.929889 -0.643402 0.414305
C -7.555721 -0.832292 1.879120
C -7.668281 0.704910 -1.694173
H -1.836824 -2.573626 -0.826823
H -2.232498 2.132265 0.424395
H 0.208211 2.413843 0.360076
H 2.601027 2.739573 0.353054
H 1.639984 -2.864455 -1.140058
H 5.005367 -1.064182 -2.177264
H 6.372229 -0.371650 0.150735
H 5.502295 -1.873634 0.106357
H 4.083247 -1.586544 3.464788
H 3.010805 -0.879193 2.234724
H 3.861129 -2.427870 1.924992
H 6.534499 1.693606 -1.323410
H 5.171541 2.688811 -1.957435
H 6.383255 3.395198 -0.875029
H -4.518042 2.870964 0.662877
H -5.789923 2.039429 1.566045
H -4.129402 1.826427 2.051553
H -5.440301 -0.721198 -0.995639
H -7.292708 1.406138 0.273862
H -8.999860 -0.405891 0.362309
H -7.793596 -1.594892 -0.115239
H -7.675126 0.101504 2.437649
H -8.204454 -1.585644 2.337503
H -6.521870 -1.172733 1.990401
H -7.120164 1.509889 -2.196630
H -8.725172 0.991464 -1.664159
H -7.582974 -0.197620 -2.310126
SCF Energy (B3LYP/6-31G**)= -1361.69955889
Number of imaginary frequencies = 0

1a_c250
MMFF Geometry
C -1.692456 -2.111639 -0.399535
C -2.205249 0.550700 -0.583635
N -2.988683 -1.717389 -0.434177
C -0.591251 -1.249611 -0.454608
C -0.869610 0.124815 -0.552079
C -3.228365 -0.392362 -0.520110
C 0.195611 1.040155 -0.617535
C 1.532825 0.612208 -0.584477
C 1.807918 -0.754368 -0.480525
C 0.751126 -1.681208 -0.420103
C 3.218892 -1.211112 -0.442355
C 4.355178 -0.165039 -0.487628
C 3.897540 1.278508 -0.621965
C 2.597076 1.615137 -0.665763
O 0.975456 -3.036114 -0.332489
O 3.435471 -2.426619 -0.397927
O 5.134459 -0.474150 -1.664517
C 5.292771 -0.395331 0.705933
C 4.661283 -0.097900 2.048067
C 5.591465 -0.085913 3.232982
O 3.454533 0.095768 2.189231
O 4.950422 2.173935 -0.698586
C 4.662027 3.563749 -0.796170
C -4.652328 0.010674 -0.545581
C -5.494342 -0.702238 -1.563754
C -5.096104 0.902714 0.366237
C -6.468020 1.504262 0.602605
C -7.121579 0.930107 1.876772
C -7.467127 -0.552418 1.814591
C -7.440858 1.528128 -0.577998
H -1.554072 -3.188113 -0.323073
H -2.449291 1.605510 -0.664955
H -0.027404 2.104030 -0.692003
H 2.277068 2.647162 -0.756523
H 1.948164 -3.189763 -0.316258
H 4.648092 -0.131104 -2.432886
H 6.191277 0.226381 0.603101
H 5.641386 -1.435750 0.725774
H 5.028010 0.141790 4.142101
H 6.058095 -1.067960 3.341839
H 6.355674 0.682251 3.091358
H 4.113884 3.785374 -1.717423
H 4.110911 3.911495 0.083470
H 5.613402 4.102622 -0.832437
H -4.948974 -1.497930 -2.083085
H -6.359974 -1.176821 -1.091903
H -5.834482 -0.005409 -2.336042
H -4.369924 1.271914 1.094817
H -6.269187 2.565003 0.818060
H -6.452484 1.093504 2.731646
H -8.037645 1.493494 2.094801
H -6.576991 -1.167489 1.653627
H -7.919497 -0.867698 2.760702
H -8.186101 -0.765745 1.018753
H -6.970364 1.966360 -1.464659
H -8.312368 2.146994 -0.335162
H -7.817670 0.536955 -0.840288
SCF Energy (B3LYP/6-31G**)= -1361.69853529
Number of imaginary frequencies = 0

1a_c251
MMFF Geometry
C -1.625392 -1.759374 -0.510002
C -2.208891 0.747939 0.356873
N -2.918942 -1.462962 -0.236024
C -0.558705 -0.866626 -0.381161
C -0.872440 0.429665 0.066827
C -3.205385 -0.214071 0.197533
C 0.156269 1.380451 0.205720
C 1.489443 1.054926 -0.078739
C 1.793021 -0.240424 -0.506744
C 0.779882 -1.193419 -0.676903
C 3.201634 -0.557879 -0.809456
C 4.305103 0.326896 -0.197898
C 3.838352 1.769196 -0.039599
C 2.530175 2.083834 0.022878
O 1.046467 -2.460374 -1.136143
O 3.469760 -1.518617 -1.536623
O 5.414057 0.299240 -1.115205
C 4.794014 -0.196084 1.154920
C 4.894220 -1.701879 1.191807
C 6.166809 -2.335528 0.697653
O 3.941239 -2.380620 1.577160
O 4.878289 2.673718 0.079577
C 4.574298 4.061100 0.145605
C -4.636940 0.070619 0.506383
C -4.898874 1.084950 1.584159
C -5.598416 -0.605782 -0.160979
C -7.107581 -0.513232 -0.082775
C -7.711656 0.896411 -0.235074
C -7.288993 1.599742 -1.518769
C -7.644215 -1.219132 1.165091
H -1.462105 -2.779778 -0.850320
H -2.464032 1.748421 0.690418
H -0.092878 2.387508 0.538400
H 2.189539 3.103942 0.161743
H 1.998455 -2.521049 -1.372942
H 5.241822 -0.425270 -1.749396
H 4.106889 0.088587 1.962969
H 5.775804 0.217835 1.415804
H 6.163522 -3.400841 0.945084
H 6.243919 -2.222629 -0.385457
H 7.025822 -1.871036 1.188111
H 4.031046 4.386740 -0.747373
H 4.010801 4.293860 1.054723
H 5.519034 4.611303 0.185306
H -5.889766 0.982280 2.029710
H -4.191151 0.969498 2.413238
H -4.809789 2.101536 1.187333
H -5.272571 -1.342750 -0.899635
H -7.481471 -1.091870 -0.940669
H -8.805924 0.809243 -0.241399
H -7.467098 1.533393 0.620118
H -7.530153 0.992291 -2.396870
H -7.816545 2.554343 -1.613844
H -6.215854 1.811977 -1.528186
H -7.250438 -2.239001 1.240113
H -8.736851 -1.289567 1.126152
H -7.380323 -0.688803 2.084812
SCF Energy (B3LYP/6-31G**)= -1361.69955569
Number of imaginary frequencies = 0

1a_c252
MMFF Geometry
C -1.838182 -1.620500 -0.719734
C -2.147425 1.047590 -0.324642
N -3.097571 -1.124751 -0.661074
C -0.674227 -0.858681 -0.590406
C -0.846999 0.520478 -0.385308
C -3.249396 0.205425 -0.465502
C 0.285995 1.341024 -0.243392
C 1.583468 0.811160 -0.304741
C 1.753455 -0.560952 -0.509526
C 0.630117 -1.393172 -0.651854
C 3.126773 -1.121217 -0.561061
C 4.346226 -0.188782 -0.427011
C 3.985553 1.264779 -0.226349
C 2.724679 1.705443 -0.139840
O 0.748792 -2.748871 -0.850178
O 3.265595 -2.335183 -0.746304
O 4.979263 -0.311676 -1.721242
C 5.320205 -0.744594 0.622348
C 4.936127 -0.463860 2.060192
C 3.722179 -1.156452 2.620818
O 5.602126 0.297388 2.762370
O 5.007109 2.171214 0.021676
C 5.852205 2.386437 -1.107175
C -4.650689 0.715369 -0.422377
C -4.864580 2.154187 -0.808209
C -5.650296 -0.123639 -0.070985
C -7.125327 0.182612 0.063319
C -7.662384 -0.230251 1.447871
C -7.039497 0.560508 2.591940
C -7.894519 -0.550456 -1.038348
H -1.788546 -2.695254 -0.880809
H -2.287998 2.109968 -0.154057
H 0.150379 2.410550 -0.082430
H 2.536067 2.757073 0.060327
H 1.702627 -2.982037 -0.901999
H 5.131874 -1.264330 -1.872743
H 6.321547 -0.323687 0.459310
H 5.456288 -1.828106 0.515539
H 3.792699 -1.183108 3.712131
H 2.818855 -0.614457 2.336895
H 3.678107 -2.187196 2.260593
H 6.586738 1.581335 -1.197917
H 5.274395 2.493751 -2.031700
H 6.400179 3.318645 -0.940356
H -4.256253 2.420492 -1.680277
H -5.897031 2.369873 -1.094455
H -4.599614 2.818420 0.020956
H -5.395403 -1.158686 0.167016
H -7.318051 1.253541 -0.054047
H -8.748094 -0.073649 1.474141
H -7.495627 -1.301155 1.619981
H -7.185556 1.636042 2.449935
H -7.506653 0.278085 3.540874
H -5.966289 0.365697 2.678868
H -7.542453 -0.247370 -2.030826
H -8.964271 -0.321665 -0.981811
H -7.778772 -1.637236 -0.956371
SCF Energy (B3LYP/6-31G**)= -1361.69961180
Number of imaginary frequencies = 0

1a_c253
MMFF Geometry
C 1.813551 -1.627334 -0.202954
C 2.319493 1.034898 -0.016653
N 3.106170 -1.225574 -0.142185
C 0.710567 -0.770538 -0.177895
C 0.984123 0.606153 -0.083958
C 3.354279 0.101005 -0.046810
C -0.084017 1.522590 -0.068510
C -1.415010 1.087198 -0.126631
C -1.678709 -0.281774 -0.201245
C -0.627350 -1.208309 -0.246740
C -3.090007 -0.716562 -0.262098
C -4.167479 0.238664 0.306539
C -3.786901 1.692064 0.008037
C -2.505064 2.064576 -0.173381
O -0.858369 -2.558334 -0.362082
O -3.330697 -1.834291 -0.723741
O -4.195222 0.133621 1.733472
C -5.540621 -0.118558 -0.298956
C -6.009247 -1.500942 0.111287
C -6.843287 -2.264061 -0.881455
O -5.764715 -1.953191 1.232174
O -4.866837 2.559047 -0.006761
C -4.617830 3.957821 -0.080385
C 4.788342 0.504431 0.039110
C 5.083380 1.759988 0.811117
C 5.725553 -0.281062 -0.536957

C 7.228622 -0.122886 -0.627167
C 7.970114 -0.434773 0.685974
C 7.706254 -1.840956 1.210477
C 7.665606 1.224825 -1.210022
H 1.682568 -2.704937 -0.274721
H 2.541479 2.095198 0.044687
H 0.130178 2.589008 -0.009508
H -2.222042 3.094816 -0.355648
H -1.818755 -2.699041 -0.521984
H -4.612238 -0.729252 1.948913
H -5.495385 -0.069950 -1.393564
H -6.325737 0.565384 0.045045
H -7.102502 -3.243139 -0.469300
H -7.761918 -1.710149 -1.089148
H -6.273869 -2.411216 -1.802486
H -4.003569 4.292434 0.761827
H -4.153262 4.221305 -1.035842
H -5.580652 4.473937 -0.021462
H 4.468491 1.817752 1.716736
H 6.117425 1.812784 1.155494
H 4.886949 2.645453 0.197730
H 5.379669 -1.186658 -1.042092
H 7.558268 -0.873679 -1.360764
H 9.049561 -0.329954 0.518390
H 7.707534 0.288584 1.465269
H 7.959634 -2.594478 0.458019
H 8.318355 -2.030822 2.098088
H 6.658197 -1.974010 1.495459
H 7.100017 1.465046 -2.117032
H 8.726587 1.191796 -1.482709
H 7.542359 2.049228 -0.502941
SCF Energy (B3LYP/6-31G**)= -1361.70740126
Number of imaginary frequencies = 0

1a_c254
MMFF Geometry
C 1.497321 2.350656 -0.171142
C 2.196015 -0.239856 0.258478
N 2.794669 2.082384 0.114821
C 0.483378 1.391897 -0.263583
C 0.856953 0.054109 -0.037735
C 3.131938 0.790735 0.319108
C -0.116455 -0.958183 -0.118355
C -1.454262 -0.657152 -0.408067
C -1.822490 0.673892 -0.621535
C -0.860519 1.693876 -0.564311
C -3.242166 0.976932 -0.911630
C -4.319864 -0.100132 -0.651370
C -3.744760 -1.508370 -0.658840
C -2.428384 -1.738604 -0.543706
O -1.184589 3.009801 -0.798537
O -3.545937 2.095295 -1.343280
O -5.218123 0.017702 -1.780250
C -5.128791 0.316544 0.588288
C -4.443487 0.043570 1.907009
C -5.326238 -0.333723 3.067351
O -3.232298 0.191824 2.054713
O -4.532528 -2.615258 -0.956393
C -5.841212 -2.670517 -0.412484
C 4.559152 0.541214 0.634067
C 5.167189 1.539174 1.582491
C 5.216028 -0.493422 0.067120
C 6.674491 -0.864851 0.238789
C 7.356619 -0.901803 -1.142617
C 8.859020 -1.138871 -1.070640
C 6.765466 -2.206836 0.968552
H 1.283233 3.405295 -0.330311
H 2.502454 -1.262764 0.453910
H 0.176132 -1.994221 0.047701
H -2.052299 -2.756683 -0.600160
H -2.124027 3.053197 -1.084552
H -5.232826 0.964730 -2.025829
H -6.103968 -0.182066 0.590549
H -5.361197 1.389268 0.562158
H -4.711217 -0.524421 3.951239
H -6.015657 0.486086 3.283122
H -5.883225 -1.242486 2.827895
H -5.820343 -2.533328 0.672124
H -6.507185 -1.948960 -0.891429
H -6.243112 -3.668667 -0.611727
H 4.473367 1.773177 2.398545
H 6.077965 1.169825 2.060321
H 5.410988 2.469280 1.058366
H 4.677825 -1.138523 -0.628099
H 7.195361 -0.117320 0.844963
H 6.904516 -1.679466 -1.771419
H 7.186886 0.053986 -1.655427
H 9.090563 -2.137424 -0.689037
H 9.299297 -1.057275 -2.069733
H 9.343622 -0.398778 -0.426164
H 6.213848 -2.176085 1.915248
H 7.803444 -2.457874 1.208174
H 6.351025 -3.021761 0.364374
SCF Energy (B3LYP/6-31G**)= -1361.69715812
Number of imaginary frequencies = 0

1a_c255
MMFF Geometry
C 1.714862 -1.812872 0.053756
C 2.342261 0.787131 -0.415542
N 3.024880 -1.477118 -0.031533
C 0.651412 -0.917199 -0.082177
C 0.988223 0.425986 -0.324625
C 3.334325 -0.180852 -0.265035
C -0.037761 1.376413 -0.474627
C -1.387667 1.010718 -0.375931
C -1.719071 -0.322887 -0.126570
C -0.706845 -1.286739 0.006995
C -3.150720 -0.698343 -0.040890
C -4.208814 0.407693 0.207890
C -3.706899 1.758696 -0.285025
C -2.424750 2.017367 -0.574058
O -0.993414 -2.613804 0.225609
O -3.435624 -1.895912 -0.131294
O -4.407961 0.547740 1.616827
C -5.514606 0.020000 -0.517511
C -6.168565 -1.210365 0.079977
C -6.876897 -2.139876 -0.867464
O -6.165969 -1.406940 1.297240
O -4.645860 2.758990 -0.512530
C -5.100724 3.337220 0.713338
C 4.786879 0.147959 -0.368670
C 5.146638 1.314975 -1.245364
C 5.682169 -0.621892 0.289921
C 7.191102 -0.522527 0.389484
C 7.682547 0.847619 0.894578
C 9.152684 0.845029 1.295624
C 7.871185 -0.981198 -0.902542
H 1.535242 -2.869035 0.242948
H 2.610776 1.823952 -0.589314
H 0.223274 2.416150 -0.669551
H -2.135842 2.997828 -0.941279
H -1.967807 -2.737801 0.169072
H -4.907005 -0.245084 1.915209
H -5.314491 -0.165234 -1.579908
H -6.270884 0.810522 -0.448449
H -7.289216 -2.987202 -0.312775
H -7.692490 -1.606243 -1.361307
H -6.168427 -2.517673 -1.608771
H -5.809575 2.673034 1.216246
H -4.265325 3.585593 1.377245
H -5.627338 4.264416 0.468470
H 4.971196 2.260146 -0.721123
H 6.189157 1.299632 -1.566758
H 4.554044 1.310149 -2.167510
H 5.290332 -1.459954 0.872236
H 7.475495 -1.254168 1.160276
H 7.539252 1.624327 0.137164
H 7.090159 1.146440 1.768811
H 9.802943 0.670150 0.433583
H 9.425509 1.814605 1.724802
H 9.355054 0.074705 2.046344

H 7.439736 -1.922357 -1.262029
H 8.939321 -1.158445 -0.740101
H 7.777563 -0.241555 -1.702803
SCF Energy (B3LYP/6-31G**)= -1361.70141442
Number of imaginary frequencies = 0

1a_c256
MMFF Geometry
C 1.772599 2.502444 0.037193
C 2.511749 -0.089807 -0.302559
N 3.097147 2.220728 -0.012403
C 0.748873 1.555518 -0.071982
C 1.144139 0.218137 -0.249429
C 3.453857 0.928531 -0.173574
C 0.161185 -0.779334 -0.372397
C -1.205839 -0.467659 -0.316504
C -1.598937 0.861535 -0.136093
C -0.625609 1.869499 -0.018180
C -3.045304 1.190258 -0.068926
C -4.095779 0.061319 -0.139360
C -3.500068 -1.304079 -0.404231
C -2.183843 -1.542049 -0.450865
O -0.964474 3.191410 0.155108
O -3.368558 2.377394 0.050595
O -4.903740 0.424157 -1.264459
C -4.873064 0.097720 1.188774
C -6.249660 -0.516324 1.096151
C -6.528941 -1.732330 1.938287
O -7.128189 0.002226 0.405081
O -4.365235 -2.388998 -0.469592
C -5.054272 -2.453608 -1.720751
C 4.911781 0.665753 -0.228092
C 5.693897 1.673255 -1.027462
C 5.445045 -0.389426 0.424309
C 6.908072 -0.772982 0.510835
C 7.136319 -2.080610 -0.268617
C 8.600261 -2.491410 -0.347948
C 7.309532 -0.895032 1.984109
H 1.544733 3.557717 0.172473
H 2.838652 -1.113380 -0.457704
H 0.471139 -1.814460 -0.514629
H -1.823260 -2.556945 -0.594962
H -1.945285 3.272110 0.125217
H -5.546175 1.096102 -0.963330
H -5.041083 1.128990 1.526733
H -4.303010 -0.385816 1.991824
H -7.474289 -2.184842 1.626501
H -5.734757 -2.471155 1.812926
H -6.598368 -1.436672 2.987900
H -5.935950 -1.807352 -1.707013
H -4.400690 -2.201063 -2.563080
H -5.399616 -3.482952 -1.856105
H 5.852666 2.587084 -0.445319
H 6.670187 1.296854 -1.343411
H 5.161423 1.937493 -1.948725
H 4.784620 -1.044356 0.993223
H 7.538068 0.007551 0.072689
H 6.561129 -2.899281 0.182479
H 6.760717 -1.961476 -1.293331
H 8.997401 -2.756039 0.636243
H 8.708650 -3.368582 -0.994092
H 9.212572 -1.686041 -0.765563
H 7.062508 0.020179 2.534401
H 8.387491 -1.053579 2.088400
H 6.796112 -1.730603 2.473089
SCF Energy (B3LYP/6-31G**)= -1361.70234801
Number of imaginary frequencies = 0

1a_c257
MMFF Geometry
C 1.870157 -2.489640 0.018091
C 2.563268 0.135948 0.125656
N 3.187089 -2.183703 0.111290
C 0.833187 -1.552431 -0.027867
C 1.203927 -0.196327 0.029592
C 3.520607 -0.876156 0.156683
C 0.206477 0.794880 -0.015185
C -1.151405 0.456363 -0.104739
C -1.514735 -0.891434 -0.149156
C -0.531204 -1.892571 -0.126549
C -2.952703 -1.230580 -0.243674
C -3.983752 -0.163088 0.187332
C -3.472639 1.248039 -0.088315
C -2.158565 1.514620 -0.208235
O -0.855013 -3.226856 -0.200777
O -3.251245 -2.369326 -0.614369
O -4.085352 -0.321507 1.609763
C -5.314649 -0.500194 -0.516561
C -6.506376 0.245768 0.045344
C -7.593989 0.623119 -0.923726
O -6.608780 0.483822 1.250628
O -4.475181 2.196994 -0.172130
C -4.113988 3.573057 -0.211803
C 4.970066 -0.585200 0.265662
C 5.711227 -1.483661 1.218907
C 5.528970 0.398794 -0.471041
C 6.986853 0.803136 -0.513928
C 7.187870 2.240841 -0.000211
C 6.836864 2.406624 1.473199
C 7.500039 0.683838 -1.952755
H 1.659742 -3.556461 -0.018887
H 2.871703 1.174792 0.190203
H 0.498351 1.843795 0.020589
H -1.786376 2.517416 -0.384456
H -1.823938 -3.302471 -0.357054
H -4.945238 0.068294 1.878813
H -5.567211 -1.561959 -0.403331
H -5.231703 -0.295223 -1.590827
H -8.385683 1.163144 -0.397153
H -7.183380 1.273103 -1.700387
H -8.015356 -0.280646 -1.370392
H -3.526475 3.850679 0.669287
H -3.574609 3.807422 -1.134918
H -5.035819 4.162102 -0.202379
H 5.897342 -2.460889 0.761033
H 6.671817 -1.069017 1.534052
H 5.133119 -1.636168 2.137916
H 4.895695 0.969246 -1.150986
H 7.600587 0.133314 0.096288
H 8.237903 2.528530 -0.136596
H 6.589888 2.945561 -0.591907
H 7.411435 1.711472 2.093565
H 7.069592 3.424676 1.801688
H 5.771829 2.234461 1.655622
H 7.379566 -0.338452 -2.328611
H 8.565363 0.933486 -2.005346
H 6.963761 1.358239 -2.630104
SCF Energy (B3LYP/6-31G**)= -1361.71191318
Number of imaginary frequencies = 0

1a_c258
MMFF Geometry
C -1.505530 -2.174945 -0.569671
C -2.392591 0.315375 0.062617
N -2.838773 -1.999075 -0.402320
C -0.544178 -1.167268 -0.442059
C -1.015271 0.117968 -0.112901
C -3.268827 -0.756493 -0.096073
C -0.098592 1.175917 0.028431
C 1.276625 0.970961 -0.143887
C 1.739228 -0.311097 -0.457596
C 0.838568 -1.372589 -0.623274
C 3.192139 -0.503708 -0.649607
C 4.170189 0.544163 -0.079205
C 3.528589 1.919571 -0.037796
C 2.199588 2.100262 -0.029894
O 1.260089 -2.634343 -0.967193
O 3.601408 -1.492388 -1.266738
O 5.290358 0.570480 -0.981066
C 4.680489 0.180354 1.317444
C 4.919615 -1.300697 1.485644
C 6.274291 -1.840183 1.113069

O 4.015479 -2.038908 1.878427
O 4.349541 3.011353 0.209018
C 4.978648 3.490001 -0.979779
C -4.733358 -0.607880 0.085905
C -5.360858 -1.712725 0.889570
C -5.381265 0.440006 -0.467616
C -6.855404 0.789288 -0.456271
C -7.374035 1.285796 0.906168
C -6.625353 2.505473 1.429689
C -7.742715 -0.323404 -1.022662
H -1.216129 -3.193801 -0.818689
H -2.778146 1.293394 0.333896
H -0.467714 2.171194 0.274126
H 1.786704 3.098092 0.089308
H 2.229399 -2.614603 -1.128124
H 5.277310 -0.277439 -1.470891
H 3.955308 0.463814 2.092073
H 5.613620 0.706938 1.552604
H 6.343315 -2.890472 1.410079
H 6.421389 -1.766687 0.033817
H 7.051560 -1.280630 1.639325
H 5.910525 2.948850 -1.163754
H 4.318822 3.427488 -1.852453
H 5.232226 4.542592 -0.821444
H -4.751825 -1.945794 1.770899
H -6.350770 -1.453945 1.267857
H -5.457928 -2.621647 0.286340
H -4.796090 1.151527 -1.052389
H -6.962856 1.630530 -1.157166
H -8.433907 1.552515 0.806931
H -7.326032 0.491381 1.658261
H -6.654042 3.324803 0.704381
H -7.087277 2.859102 2.357127
H -5.578846 2.272132 1.648177
H -7.349240 -0.695430 -1.975088
H -8.753277 0.057026 -1.209787
H -7.839651 -1.171844 -0.340243
SCF Energy (B3LYP/6-31G**)= -1361.69356400
Number of imaginary frequencies = 0

1a_c259
MMFF Geometry
C 1.802930 2.395663 -0.109510
C 2.470260 -0.177163 0.453223
N 3.109587 2.111754 0.110289
C 0.763177 1.460932 -0.066776
C 1.121009 0.133704 0.227696
C 3.431763 0.828487 0.379361
C 0.119807 -0.851144 0.290415
C -1.228867 -0.536190 0.064817
C -1.584856 0.783503 -0.227599
C -0.592531 1.777432 -0.296054
C -3.012861 1.118251 -0.458480
C -4.092871 0.024274 -0.317812
C -3.526940 -1.356215 -0.066207
C -2.226741 -1.598050 0.139328
O -0.894646 3.088633 -0.582040
O -3.298016 2.284118 -0.754305
O -4.734727 0.019442 -1.597767
C -5.030745 0.472865 0.817379
C -6.409235 -0.138322 0.734590
C -6.845294 -1.031088 1.865377
O -7.169503 0.139775 -0.194357
O -4.422174 -2.406728 0.091329
C -4.948884 -2.857383 -1.159204
C 4.871074 0.562174 0.614839
C 5.566935 1.607907 1.444144
C 5.463381 -0.527947 0.081700
C 6.921758 -0.926004 0.180595
C 7.505060 -1.079565 -1.237552
C 9.003150 -1.351867 -1.250598
C 7.033211 -2.214788 0.998128
H 1.603789 3.442375 -0.329878
H 2.766533 -1.192585 0.697736
H 0.401042 -1.879405 0.517073
H -1.894280 -2.607625 0.365212
H -1.859133 3.156716 -0.767864
H -5.381647 0.751779 -1.607460
H -5.196383 1.558237 0.795193
H -4.588718 0.262763 1.799280
H -7.761749 -1.557257 1.584454
H -6.073979 -1.773003 2.082046
H -7.035090 -0.423023 2.753158
H -5.796598 -2.238004 -1.464065
H -4.183373 -2.880843 -1.942727
H -5.318106 -3.877202 -1.015065
H 4.937091 1.919646 2.285696
H 6.499600 1.247890 1.885471
H 5.795699 2.490613 0.837729
H 4.864532 -1.205750 -0.527424
H 7.500212 -0.151881 0.694068
H 6.993007 -1.887277 -1.776023
H 7.321447 -0.158320 -1.805803
H 9.238313 -2.326747 -0.813963
H 9.374516 -1.353489 -2.280536
H 9.547871 -0.581383 -0.696016
H 6.549683 -2.101821 1.975317
H 8.079464 -2.476509 1.184393
H 6.559531 -3.058890 0.484304
SCF Energy (B3LYP/6-31G**)= -1361.70240961
Number of imaginary frequencies = 0

1a_c260
MMFF Geometry
C 1.933408 -2.324832 -0.180909
C 2.510099 0.303483 -0.561258
N 3.236065 -1.971443 -0.302558
C 0.855451 -1.435737 -0.237529
C 1.165941 -0.078151 -0.438201
C 3.511907 -0.661735 -0.480601
C 0.124126 0.863878 -0.519550
C -1.217404 0.477432 -0.387794
C -1.519200 -0.869123 -0.172684
C -0.493531 -1.825445 -0.113389
C -2.940796 -1.258984 -0.035601
C -3.963644 -0.164508 0.344285
C -3.555905 1.195905 -0.213924
C -2.278835 1.475824 -0.534872
O -0.760438 -3.162896 0.061705
O -3.226540 -2.450649 -0.182362
O -3.889574 -0.090297 1.775268
C -5.350588 -0.649318 -0.126559
C -6.501946 0.145525 0.452240
C -7.712892 0.327960 -0.422243
O -6.472835 0.575665 1.607230
O -4.605443 2.089613 -0.325112
C -4.317400 3.449642 -0.629961
C 4.948406 -0.318670 -0.609274
C 5.743324 -1.282961 -1.447881
C 5.454214 0.762054 0.022722
C 6.894199 1.227555 0.042305
C 7.431322 1.338255 1.482500
C 7.495307 -0.003242 2.202485
C 6.992802 2.583706 -0.660951
H 1.770510 -3.389973 -0.030305
H 2.772484 1.342893 -0.732184
H 0.368156 1.912276 -0.686890
H -1.977686 2.445963 -0.913448
H -1.736382 -3.289582 0.040952
H -4.729271 0.314569 2.083054
H -5.537380 -1.684663 0.185093
H -5.404428 -0.621278 -1.221663
H -8.461829 0.925044 0.105269
H -7.428072 0.852481 -1.337687
H -8.140449 -0.648322 -0.662909
H -3.645211 3.885705 0.116079
H -3.900975 3.542840 -1.637882
H -5.258909 4.006096 -0.602513
H 5.183739 -1.570453 -2.345797
H 6.685157 -0.858910 -1.804554
H 5.972975 -2.189582 -0.878144
H 4.788058 1.373434 0.632163

H 7.542284 0.529850 -0.496974
H 8.442892 1.762612 1.457792
H 6.814752 2.030841 2.069372
H 8.100950 -0.721170 1.640299
H 7.950122 0.122731 3.190284
H 6.498486 -0.430465 2.347720
H 6.637686 2.515807 -1.695420
H 8.031367 2.930873 -0.688850
H 6.396967 3.347918 -0.148913
SCF Energy (B3LYP/6-31G**)= -1361.71199174
Number of imaginary frequencies = 0

1a_c261
MMFF Geometry
C -1.701958 -1.543864 -0.517506
C -2.147565 1.069689 0.073544
N -2.981558 -1.141025 -0.326555
C -0.583521 -0.711237 -0.433481
C -0.825682 0.640195 -0.126191
C -3.199823 0.161109 -0.031891
C 0.259098 1.532402 -0.032437
C 1.576905 1.096625 -0.226727
C 1.807984 -0.250781 -0.517173
C 0.739927 -1.149617 -0.639287
C 3.202131 -0.685069 -0.727628
C 4.339526 0.182609 -0.155037
C 3.965635 1.660231 -0.153004
C 2.680283 2.062123 -0.177104
O 0.937344 -2.469933 -0.963197
O 3.431365 -1.726730 -1.348977
O 5.476953 -0.003870 -1.017288
C 4.741997 -0.237524 1.260651
C 4.740406 -1.736125 1.442481
C 5.985757 -2.492749 1.065903
O 3.730599 -2.313752 1.847442
O 5.059151 2.503935 -0.074057
C 4.847238 3.907826 -0.149967
C -4.622541 0.570746 0.154776
C -4.962960 1.988527 -0.210203
C -5.514338 -0.343288 0.598023
C -6.993586 -0.218224 0.895594
C -7.881655 -0.161341 -0.361197
C -7.716967 -1.370677 -1.273590
C -7.325548 0.918714 1.867274
H -1.595223 -2.601404 -0.749698
H -2.344419 2.107071 0.323203
H 0.066377 2.580732 0.192896
H 2.404432 3.110482 -0.149241
H 1.891009 -2.613677 -1.152656
H 5.280046 -0.774411 -1.586741
H 4.045815 0.166042 2.008033
H 5.738798 0.137168 1.523886
H 5.902485 -3.526963 1.411725
H 6.110740 -2.489784 -0.018619
H 6.854725 -2.037786 1.547681
H 4.360570 4.180863 -1.091867
H 4.267520 4.261548 0.708527
H 5.824832 4.398008 -0.120686
H -6.026362 2.131705 -0.408435
H -4.674918 2.673892 0.593654
H -4.448979 2.291422 -1.129792
H -5.142040 -1.351459 0.798167
H -7.263525 -1.141178 1.430205
H -8.933110 -0.108060 -0.051552
H -7.684221 0.747602 -0.939409
H -7.909065 -2.301307 -0.730250
H -8.426838 -1.312271 -2.104999
H -6.710232 -1.419162 -1.699591
H -6.658469 0.903201 2.736199
H -8.351346 0.811008 2.237769
H -7.255447 1.904923 1.401148
SCF Energy (B3LYP/6-31G**)= -1361.69966855
Number of imaginary frequencies = 0

1a_c262
MMFF Geometry
C -1.673558 -2.441562 0.118579
C -2.290784 0.201289 -0.040028
N -2.972852 -2.083161 0.256052
C -0.618153 -1.550242 -0.098686
C -0.949387 -0.184146 -0.178011
C -3.269313 -0.768760 0.167711
C 0.067639 0.763021 -0.399095
C 1.406916 0.371762 -0.527517
C 1.730491 -0.984534 -0.433743
C 0.727440 -1.944602 -0.238217
C 3.153535 -1.376440 -0.548834
C 4.225031 -0.297686 -0.268441
C 3.744556 1.069681 -0.742251
C 2.435661 1.370763 -0.827720
O 1.016379 -3.286507 -0.171970
O 3.412495 -2.553492 -0.806517
O 5.418449 -0.662585 -0.970976
C 4.529789 -0.356936 1.232906
C 5.371405 0.801196 1.719224
C 6.847307 0.777069 1.419046
O 4.857711 1.739995 2.332075
O 4.778034 1.935442 -1.049825
C 4.473587 3.311447 -1.249985
C -4.701680 -0.420507 0.328884
C -5.385129 -1.152102 1.450627
C -5.283306 0.468830 -0.504974
C -6.723811 0.938283 -0.573758
C -7.026642 1.969928 0.527947
C -8.391190 2.629552 0.379562
C -7.739585 -0.206034 -0.642449
H -1.494329 -3.512178 0.192583
H -2.569078 1.249974 -0.080261
H -0.194833 1.818018 -0.472781
H 2.086260 2.347602 -1.144032
H 1.958016 -3.419760 -0.427989
H 5.268310 -0.459980 -1.910958
H 5.057377 -1.283770 1.491684
H 3.605637 -0.355244 1.825791
H 7.356183 1.519285 2.040775
H 7.257997 -0.207284 1.657268
H 7.023054 1.014679 0.368416
H 3.872310 3.449120 -2.154090
H 3.970019 3.733740 -0.374302
H 5.417218 3.847317 -1.388391
H -4.729813 -1.224989 2.326651
H -6.287259 -0.643747 1.795874
H -5.659281 -2.165605 1.139583
H -4.669657 0.911484 -1.291029
H -6.809338 1.463698 -1.536520
H -6.969202 1.506657 1.519290
H -6.261579 2.756904 0.507604
H -9.201894 1.908377 0.519311
H -8.512478 3.413918 1.133673
H -8.500551 3.089581 -0.607532
H -7.397509 -1.000483 -1.314993
H -8.697663 0.154745 -1.032489
H -7.937413 -0.648368 0.337560
SCF Energy (B3LYP/6-31G**)= -1361.69625157
Number of imaginary frequencies = 0

1a_c263
MMFF Geometry
C -1.779720 -1.749220 -0.370218
C -2.103604 0.927765 -0.046194
N -3.039886 -1.274656 -0.217477
C -0.624317 -0.961863 -0.376207
C -0.804500 0.421149 -0.207473
C -3.193158 0.058983 -0.056998
C 0.320233 1.265282 -0.204025
C 1.619618 0.757066 -0.365472
C 1.796547 -0.619799 -0.528623
C 0.679649 -1.475040 -0.537639
C 3.167117 -1.161986 -0.697182
C 4.373310 -0.197022 -0.659520
C 4.018591 1.272197 -0.495287
C 2.748205 1.690556 -0.362712

O 0.805417 -2.834760 -0.708020
O 3.292732 -2.376383 -0.886336
O 5.032980 -0.333689 -1.937793
C 5.380209 -0.698180 0.385357
C 4.878995 -0.612630 1.810145
C 5.895837 -0.875652 2.890071
O 3.705390 -0.373090 2.091112
O 5.127990 2.100048 -0.505921
C 4.940507 3.499178 -0.328353
C -4.588752 0.554249 0.096854
C -4.905108 1.857440 -0.578367
C -5.473492 -0.205491 0.779029
C -6.922211 0.016871 1.163022
C -7.885417 -0.802486 0.281480
C -7.923248 -0.401250 -1.187219
C -7.382289 1.465700 1.344114
H -1.719969 -2.828525 -0.493370
H -2.258331 1.991768 0.101945
H 0.174853 2.336688 -0.069722
H 2.502285 2.739502 -0.240562
H 1.761735 -3.048617 -0.806648
H 4.515726 0.172095 -2.586819
H 6.312924 -0.124327 0.313677
H 5.648798 -1.744096 0.189609
H 5.423458 -0.783493 3.872004
H 6.291950 -1.888173 2.780958
H 6.704089 -0.143682 2.818678
H 4.341885 3.919678 -1.142669
H 4.486875 3.712569 0.644840
H 5.924839 3.975931 -0.353064
H -4.262255 2.026964 -1.449999
H -5.923638 1.874081 -0.971863
H -4.772569 2.695122 0.113400
H -5.104088 -1.159653 1.167000
H -7.001942 -0.417180 2.171676
H -7.613914 -1.864498 0.341735
H -8.900259 -0.723251 0.691266
H -6.945152 -0.519276 -1.662465
H -8.631163 -1.038546 -1.727519
H -8.250032 0.634487 -1.314097
H -6.661348 2.037251 1.938518
H -8.337883 1.489301 1.880873
H -7.541741 1.988554 0.399112
SCF Energy (B3LYP/6-31G**)= -1361.69878220
Number of imaginary frequencies = 0

1a_c264
MMFF Geometry
C -1.768861 -1.808215 -0.624516
C -2.210320 0.654316 0.429546
N -3.044565 -1.460924 -0.326774
C -0.652794 -0.989305 -0.433288
C -0.894088 0.283412 0.110179
C -3.259854 -0.234383 0.201480
C 0.187604 1.156841 0.320604
C 1.502154 0.784015 -0.000255
C 1.741897 -0.486573 -0.534038
C 0.668244 -1.367836 -0.753097
C 3.131409 -0.881906 -0.882149
C 4.288057 0.121220 -0.649569
C 3.856266 1.449698 -0.062350
C 2.585491 1.732100 0.248407
O 0.853046 -2.620732 -1.291059
O 3.310655 -1.985810 -1.406604
O 4.848898 0.381593 -1.954275
C 5.391681 -0.565024 0.166562
C 4.984757 -0.869333 1.592010
C 6.103491 -1.106492 2.571883
O 3.807731 -0.969922 1.936380
O 4.839467 2.354294 0.321175
C 5.520678 2.946084 -0.783808
C -4.673045 0.106732 0.536810
C -4.876635 1.050537 1.688784
C -5.671436 -0.462189 -0.175160
C -7.172849 -0.290470 -0.083490
C -7.695913 1.158547 -0.127691
C -7.234288 1.930614 -1.357329
C -7.748462 -1.056022 1.110681
H -1.662956 -2.808136 -1.039818
H -2.406779 1.638967 0.840851
H -0.001675 2.142078 0.745893
H 2.345012 2.675734 0.730403
H 1.815766 -2.754084 -1.448189
H 4.118402 0.661655 -2.532826
H 6.288371 0.066894 0.187673
H 5.696388 -1.509074 -0.302096
H 5.688754 -1.313024 3.562406
H 6.696606 -1.965452 2.248831
H 6.731882 -0.214575 2.633719
H 6.305052 2.279376 -1.152818
H 4.829506 3.218121 -1.589041
H 6.002800 3.861181 -0.427127
H -5.872021 0.971817 2.129145
H -4.177179 0.832548 2.504146
H -4.728999 2.087290 1.369087
H -5.387871 -1.158862 -0.968284
H -7.579193 -0.781382 -0.980423
H -8.793367 1.134204 -0.135582
H -7.415343 1.715090 0.771445
H -7.509773 1.404995 -2.277138
H -7.706785 2.917987 -1.378693
H -6.150828 2.081848 -1.355713
H -7.413182 -2.099287 1.107787
H -8.843341 -1.061425 1.071408
H -7.454762 -0.611594 2.066188
SCF Energy (B3LYP/6-31G**)= -1361.69546420
Number of imaginary frequencies = 0

1a_c265
MMFF Geometry
C 1.733390 2.342447 0.168230
C 2.258092 -0.232263 -0.528322
N 3.029226 1.951227 0.110417
C 0.637458 1.517647 -0.103965
C 0.921250 0.187301 -0.466941
C 3.278424 0.667723 -0.226756
C -0.137803 -0.688787 -0.768927
C -1.471541 -0.265795 -0.695588
C -1.746860 1.051085 -0.317317
C -0.703514 1.945854 -0.040151
C -3.162885 1.472736 -0.222569
C -4.234491 0.371530 -0.050872
C -3.837798 -0.884854 -0.818523
C -2.550988 -1.177903 -1.082013
O -0.947072 3.254464 0.301586
O -3.410869 2.679941 -0.228791
O -5.476286 0.876152 -0.555074
C -4.399294 0.145839 1.456557
C -5.223904 -1.076196 1.792301
C -6.719898 -0.981551 1.643930
O -4.681855 -2.119560 2.164318
O -4.918987 -1.664544 -1.186377
C -4.673002 -2.979144 -1.673666
C 4.709845 0.281626 -0.281983
C 5.589259 1.303532 -0.947802
C 5.117590 -0.885901 0.261540
C 6.509171 -1.479183 0.367443
C 7.492719 -0.576621 1.136217
C 8.794638 -1.280583 1.497949
C 7.033724 -1.945107 -0.992882
H 1.592349 3.383427 0.451582
H 2.502983 -1.248507 -0.821794
H 0.087020 -1.713090 -1.064897
H -2.259266 -2.079679 -1.609319
H -1.904174 3.443307 0.166430
H -5.418257 0.855442 -1.526346
H -4.875881 1.010972 1.934824
H -3.425333 0.022532 1.948252
H -7.189592 -1.824945 2.158184
H -7.080248 -0.057780 2.103455
H -6.997493 -1.010972 0.588826
H -4.160033 -2.946018 -2.639928

H -4.104532 -3.567699 -0.946222
H -5.640086 -3.468378 -1.822494
H 5.750842 2.162754 -0.288091
H 6.567336 0.908663 -1.225432
H 5.131257 1.661910 -1.877153
H 4.367338 -1.513980 0.744756
H 6.387166 -2.389776 0.972629
H 7.739144 0.320748 0.560098
H 7.019646 -0.233456 2.065229
H 9.371998 -1.538475 0.605336
H 9.416118 -0.624427 2.115941
H 8.602805 -2.196663 2.065333
H 6.267951 -2.507864 -1.538771
H 7.895364 -2.609724 -0.871111
H 7.350044 -1.109132 -1.622866
SCF Energy (B3LYP/6-31G**)= -1361.69650588
Number of imaginary frequencies = 0

1a_c266
MMFF Geometry
C 1.883347 1.883672 -0.043068
C 2.163448 -0.807260 -0.285414
N 3.133534 1.364515 0.008343
C 0.715800 1.135085 -0.210969
C 0.872564 -0.257162 -0.335060
C 3.269385 0.023974 -0.113389
C -0.265184 -1.066555 -0.511249
C -1.552304 -0.512746 -0.552445
C -1.700671 0.869111 -0.416130
C -0.577157 1.694040 -0.261701
C -3.065661 1.434788 -0.443374
C -4.267025 0.499732 -0.208577
C -3.969445 -0.922027 -0.660396
C -2.709740 -1.377218 -0.793343
O -0.691853 3.059099 -0.151041
O -3.212585 2.651194 -0.600507
O -5.338868 1.044026 -0.996587
C -4.635477 0.615761 1.274797
C -5.655247 -0.401217 1.737393
C -7.089769 -0.203683 1.321092
O -5.318649 -1.350686 2.448402
O -5.107398 -1.675287 -0.886216
C -4.969086 -3.054141 -1.204291
C 4.661288 -0.510337 -0.068735
C 4.924525 -1.759444 -0.862554
C 5.599986 0.160662 0.635507
C 7.061568 -0.154404 0.882903
C 7.928187 0.250014 -0.323312
C 9.423684 0.149620 -0.054103
C 7.311940 -1.585421 1.368639
H 1.844156 2.966029 0.059424
H 2.294864 -1.881406 -0.366938
H -0.139687 -2.143674 -0.618720
H -2.496755 -2.396556 -1.096217
H -1.623167 3.315715 -0.335828
H -5.202806 2.012005 -1.027801
H -5.037028 1.610925 1.504805
H -3.745681 0.490016 1.906166
H -7.728555 -0.892517 1.881396
H -7.402076 0.818146 1.550774
H -7.209596 -0.403034 0.255116
H -4.445610 -3.184579 -2.156706
H -4.459954 -3.592646 -0.398513
H -5.972431 -3.476934 -1.310749
H 5.976637 -1.873591 -1.130393
H 4.616805 -2.646777 -0.299854
H 4.381550 -1.743306 -1.814676
H 5.289485 1.080355 1.138033
H 7.356646 0.493398 1.721722
H 7.688367 -0.367344 -1.196407
H 7.699227 1.286557 -0.602994
H 9.732984 -0.887435 0.106289
H 9.985645 0.533622 -0.911635
H 9.705367 0.737070 0.825387
H 6.558703 -1.893377 2.102277
H 8.288012 -1.657149 1.861064
H 7.311250 -2.310947 0.550847
SCF Energy (B3LYP/6-31G**)= -1361.70262975
Number of imaginary frequencies = 0

1a_c267
MMFF Geometry
C 1.505893 -2.281427 -0.151867
C 2.412620 0.280262 -0.218823
N 2.846230 -2.082062 -0.155981
C 0.546677 -1.264187 -0.180408
C 1.027810 0.058232 -0.218596
C 3.285307 -0.805352 -0.178655
C 0.112691 1.126188 -0.265446
C -1.269923 0.897248 -0.255273
C -1.740239 -0.416187 -0.198319
C -0.843692 -1.494310 -0.180831
C -3.202406 -0.632034 -0.189608
C -4.102400 0.523617 0.311640
C -3.516766 1.869420 -0.126654
C -2.200427 2.022863 -0.368458
O -1.280807 -2.797430 -0.167737
O -3.625500 -1.735313 -0.541971
O -4.096206 0.549008 1.742459
C -5.533997 0.330585 -0.229679
C -6.192054 -0.922053 0.315077
C -7.166452 -1.630956 -0.585767
O -5.979738 -1.307340 1.467113
O -4.452794 2.887717 -0.197979
C -3.997395 4.219689 -0.404105
C 4.758113 -0.631893 -0.183975
C 5.480127 -1.570458 -1.110330
C 5.336342 0.292856 0.612910
C 6.803428 0.614824 0.822416
C 7.360413 1.456732 -0.339785
C 8.765875 1.984493 -0.084321
C 7.658193 -0.610280 1.160749
H 1.208917 -3.327736 -0.124128
H 2.807646 1.290473 -0.263593
H 0.488409 2.147732 -0.309087
H -1.771122 2.977355 -0.650217
H -2.256479 -2.801570 -0.293368
H -4.631253 -0.217311 2.044674
H -5.519963 0.275968 -1.324849
H -6.194054 1.154611 0.066677
H -7.556491 -2.518746 -0.080438
H -7.997199 -0.962265 -0.823567
H -6.658584 -1.943718 -1.501431
H -3.310626 4.527612 0.390965
H -3.531768 4.324410 -1.389179
H -4.868253 4.881102 -0.372263
H 4.932794 -1.687465 -2.053144
H 6.472429 -1.208564 -1.385709
H 5.589887 -2.558067 -0.650243
H 4.688789 0.894576 1.252650
H 6.833195 1.249558 1.720472
H 7.369368 0.876581 -1.269314
H 6.698739 2.314930 -0.515143
H 9.495025 1.170850 -0.027866
H 9.071790 2.646852 -0.900606
H 8.808211 2.555482 0.848481
H 7.143599 -1.270110 1.868068
H 8.597942 -0.303490 1.632927
H 7.919369 -1.194977 0.274671
SCF Energy (B3LYP/6-31G**)= -1361.70597103
Number of imaginary frequencies = 0

1a_c268
MMFF Geometry
C -1.835265 -1.677355 -0.302551
C -2.149291 1.002225 -0.001394
N -3.094156 -1.192896 -0.171692
C -0.675306 -0.898289 -0.297727
C -0.850445 0.486699 -0.141572
C -3.246737 0.142729 -0.020079
C 0.278755 1.324573 -0.128457
C 1.575657 0.806989 -0.270624

C 1.748833 -0.572985 -0.420463
C 0.627601 -1.421420 -0.437356
C 3.119815 -1.123750 -0.579497
C 4.331381 -0.159215 -0.578558
C 3.968788 1.301466 -0.402236
C 2.710054 1.727126 -0.242227
O 0.747313 -2.782852 -0.596947
O 3.243107 -2.338015 -0.769610
O 4.947554 -0.303083 -1.876342
C 5.362801 -0.649196 0.446671
C 4.893649 -0.521738 1.879741
C 5.964287 -0.533958 2.938759
O 3.702523 -0.460700 2.182357
O 4.996308 2.222898 -0.237187
C 5.748425 2.445652 -1.428800
C -4.647653 0.641721 0.106872
C -4.909771 2.032964 -0.398215
C -5.590311 -0.176740 0.625430
C -7.063064 0.053712 0.890321
C -7.937077 0.036452 -0.377452
C -7.829802 -1.261514 -1.168563
C -7.341259 1.294205 1.745167
H -1.781584 -2.757888 -0.416942
H -2.291764 2.069062 0.135675
H 0.140805 2.397977 -0.001314
H 2.516592 2.779144 -0.051355
H 1.703693 -3.006153 -0.666034
H 4.254558 -0.157493 -2.543668
H 6.297530 -0.085667 0.334709
H 5.619810 -1.701434 0.272165
H 5.506881 -0.433782 3.926960
H 6.511534 -1.478697 2.894611
H 6.645673 0.305688 2.780882
H 6.498853 1.661293 -1.561274
H 5.101646 2.519265 -2.309924
H 6.277737 3.396563 -1.316279
H -5.963080 2.212781 -0.619006
H -4.588275 2.777659 0.337289
H -4.376849 2.216119 -1.338431
H -5.273516 -1.178148 0.928416
H -7.386234 -0.798410 1.506750
H -8.986582 0.173820 -0.087708
H -7.687273 0.874401 -1.036915
H -8.075261 -2.124646 -0.541711
H -8.529113 -1.245938 -2.010757
H -6.823796 -1.402923 -1.575130
H -6.683159 1.326468 2.620458
H -8.374384 1.276786 2.110506
H -7.214182 2.226314 1.188557
SCF Energy (B3LYP/6-31G**)= -1361.69567536
Number of imaginary frequencies = 0

1a_c269
MMFF Geometry
C -1.641600 -1.682812 -0.649018
C -2.242851 0.722599 0.460349
N -2.939692 -1.416548 -0.365640
C -0.578541 -0.807450 -0.414239
C -0.901774 0.436328 0.158836
C -3.234964 -0.218597 0.189046
C 0.122280 1.368533 0.409624
C 1.459246 1.074426 0.111746
C 1.774566 -0.171249 -0.441988
C 0.765207 -1.102319 -0.721764
C 3.189904 -0.458069 -0.759410
C 4.290594 0.374483 -0.072007
C 3.796417 1.778546 0.219782
C 2.495014 2.082936 0.340773
O 1.039746 -2.316906 -1.302252
O 3.468407 -1.351423 -1.565060
O 5.387111 0.453519 -0.999448
C 4.796775 -0.266716 1.222141
C 4.904715 -1.769074 1.122638
C 6.192549 -2.348358 0.602155
O 3.948295 -2.485002 1.421904
O 4.763913 2.719768 0.537946
C 5.044342 3.567708 -0.576382
C -4.671465 0.032237 0.504448
C -4.950929 0.932675 1.675053
C -5.621907 -0.574277 -0.241310
C -7.132222 -0.491261 -0.175699
C -7.737218 0.926070 -0.194313
C -7.298024 1.755090 -1.394741
C -7.684873 -1.319373 0.987164
H -1.471427 -2.663214 -1.088923
H -2.504457 1.683666 0.890824
H -0.132089 2.336007 0.841340
H 2.190338 3.083789 0.632524
H 1.996598 -2.357142 -1.522854
H 5.266743 -0.277436 -1.639340
H 4.117497 -0.061547 2.060392
H 5.778905 0.128944 1.508755
H 6.179215 -3.435182 0.723565
H 6.307087 -2.111169 -0.457302
H 7.034466 -1.947910 1.172254
H 5.377709 2.993950 -1.446656
H 4.168510 4.171870 -0.836049
H 5.851487 4.246751 -0.286392
H -5.948023 0.785323 2.093382
H -4.255188 0.734429 2.498590
H -4.857925 1.984120 1.384069
H -5.284011 -1.232764 -1.045723
H -7.492680 -0.981112 -1.092606
H -8.831091 0.839110 -0.224980
H -7.506179 1.474155 0.723908
H -7.525397 1.238696 -2.332747
H -7.826304 2.713993 -1.401022
H -6.225335 1.968052 -1.367469
H -7.289890 -2.341292 0.965021
H -8.776685 -1.386448 0.925703
H -7.435249 -0.883896 1.959182
SCF Energy (B3LYP/6-31G**)= -1361.69452178
Number of imaginary frequencies = 0

1a_c270
MMFF Geometry
C -1.379452 -2.196880 -0.521124
C -2.257544 0.310679 0.053968
N -2.709626 -2.016472 -0.335421
C -0.417108 -1.185506 -0.439444
C -0.883173 0.108744 -0.139423
C -3.135409 -0.765699 -0.057793
C 0.035288 1.170996 -0.045791
C 1.408099 0.961741 -0.234134
C 1.863176 -0.329012 -0.517860
C 0.961940 -1.395398 -0.639042
C 3.311322 -0.523733 -0.721697
C 4.284484 0.524653 -0.148753
C 3.668043 1.918590 -0.155177
C 2.333583 2.098938 -0.185494
O 1.380132 -2.665093 -0.955608
O 3.714763 -1.514400 -1.337733
O 5.441227 0.528064 -1.005430
C 4.744939 0.183602 1.270525
C 4.993652 -1.293340 1.459376
C 6.349880 -1.832054 1.091100
O 4.093067 -2.030427 1.863152
O 4.604143 2.934162 -0.076640
C 4.159834 4.282242 -0.159573
C -4.596720 -0.613952 0.143760
C -5.211666 -1.695937 0.987437
C -5.258176 0.417299 -0.425034
C -6.737934 0.749097 -0.410293
C -7.151660 1.390504 0.926563
C -8.574396 1.933562 0.921558
C -7.632897 -0.418493 -0.836619
H -1.093500 -3.222387 -0.745749
H -2.639481 1.295979 0.302692
H -0.332200 2.172725 0.174248
H 1.885496 3.086203 -0.163585
H 2.344993 -2.646832 -1.142080
H 5.378123 -0.265869 -1.573284

H 3.987437 0.467733 2.013206
H 5.663587 0.721112 1.535984
H 6.439553 -2.864177 1.441538
H 6.477479 -1.812595 0.007052
H 7.128129 -1.235922 1.573966
H 3.638002 4.466018 -1.104289
H 3.525542 4.536949 0.695413
H 5.041133 4.929715 -0.129299
H -4.559187 -1.953192 1.830206
H -6.163107 -1.394514 1.429443
H -5.379895 -2.599969 0.392860
H -4.685798 1.120807 -1.031544
H -6.868268 1.516715 -1.187504
H -7.055780 0.671355 1.747728
H -6.469460 2.218979 1.157431
H -9.309566 1.129512 0.822112
H -8.779886 2.455308 1.861866
H -8.721644 2.643513 0.101663
H -7.207428 -0.952252 -1.693684
H -8.619345 -0.053404 -1.142940
H -7.792820 -1.138517 -0.029646
SCF Energy (B3LYP/6-31G**)= -1361.69817746
Number of imaginary frequencies = 0

1a_c271
MMFF Geometry
C -1.594073 -1.575330 -0.690221
C -2.080581 0.998310 0.030315
N -2.881407 -1.197967 -0.498475
C -0.487465 -0.735097 -0.545579
C -0.750764 0.595443 -0.171620
C -3.119462 0.084108 -0.139302
C 0.321250 1.494233 -0.015725
C 1.646907 1.084022 -0.212089
C 1.900379 -0.243944 -0.567969
C 0.844219 -1.146635 -0.753749
C 3.305733 -0.655347 -0.771285
C 4.409801 0.190637 -0.094208
C 4.023992 1.668380 -0.069768
C 2.736492 2.060445 -0.108575
O 1.062419 -2.445412 -1.144979
O 3.532150 -1.661995 -1.444802
O 5.625076 0.041359 -0.840268
C 4.691591 -0.289329 1.331352
C 4.706846 -1.796538 1.437138
C 5.940810 -2.527400 0.979737
O 3.716985 -2.399012 1.856673
O 5.111720 2.517205 0.018672
C 4.882689 3.920132 0.081104
C -4.548535 0.467573 0.048794
C -4.905864 1.893352 -0.265243
C -5.433624 -0.473833 0.445795
C -6.915745 -0.372373 0.744764
C -7.745469 -0.390367 -0.551678
C -9.244819 -0.499312 -0.308497
C -7.278279 0.775899 1.691584
H -1.470388 -2.618021 -0.975362
H -2.295172 2.018234 0.332064
H 0.111910 2.527256 0.259735
H 2.447777 3.105164 -0.070611
H 2.021190 -2.564258 -1.332024
H 5.509820 0.531687 -1.672777
H 3.922947 0.067147 2.029908
H 5.653607 0.085488 1.702112
H 5.909868 -3.557351 1.346385
H 5.986663 -2.538724 -0.110936
H 6.831255 -2.044702 1.390116
H 4.384657 4.275374 -0.826698
H 4.306263 4.182485 0.974004
H 5.854923 4.417041 0.149041
H -4.359173 2.252327 -1.144951
H -5.962592 2.017866 -0.509355
H -4.671369 2.545983 0.581965
H -5.053087 -1.486486 0.603293
H -7.160991 -1.292447 1.295796
H -7.552711 0.509381 -1.146878
H -7.435553 -1.243763 -1.168717
H -9.634632 0.390539 0.194580
H -9.773153 -0.598297 -1.262271
H -9.481615 -1.376360 0.301960
H -6.553072 0.861226 2.508421
H -8.258083 0.599835 2.149107
H -7.332417 1.739805 1.178611
SCF Energy (B3LYP/6-31G**)= -1361.69389859
Number of imaginary frequencies = 0

1a_c272
MMFF Geometry
C 1.851446 2.536639 0.057427
C 2.592079 -0.051874 -0.306557
N 3.176159 2.256410 0.003759
C 0.828290 1.589947 -0.059067
C 1.224336 0.254525 -0.249046
C 3.533589 0.965998 -0.169365
C 0.242033 -0.742574 -0.379880
C -1.125155 -0.432477 -0.319568
C -1.519058 0.894731 -0.126695
C -0.546367 1.902354 -0.000824
C -2.965597 1.221708 -0.054864
C -4.015286 0.092632 -0.134259
C -3.418842 -1.269854 -0.412152
C -2.102491 -1.506370 -0.462479
O -0.886043 3.222382 0.184786
O -3.289621 2.407463 0.075752
O -4.824853 0.464988 -1.255086
C -4.791029 0.116431 1.195062
C -6.167265 -0.497816 1.098510
C -6.444626 -1.721593 1.929957
O -7.047002 0.026274 0.413181
O -4.283254 -2.354811 -0.486293
C -4.973719 -2.408656 -1.737174
C 4.991405 0.704703 -0.228103
C 5.773545 1.720077 -1.017523
C 5.526813 -0.359336 0.407962
C 6.990880 -0.736304 0.483442
C 7.330485 -1.900866 -0.466870
C 6.622119 -3.217821 -0.173092
C 7.381528 -1.038314 1.933651
H 1.622933 3.590478 0.202456
H 2.919485 -1.073782 -0.471488
H 0.552625 -1.776128 -0.531815
H -1.741299 -2.519643 -0.616159
H -1.866951 3.302581 0.156781
H -5.467457 1.133676 -0.947143
H -4.959437 1.144476 1.542522
H -4.219658 -0.373900 1.993040
H -7.389998 -2.171999 1.615209
H -5.650029 -2.458643 1.796993
H -6.513035 -1.435480 2.982278
H -5.855875 -1.763235 -1.716564
H -4.321326 -2.148007 -2.577955
H -5.318433 -3.437004 -1.881412
H 5.928822 2.629871 -0.428194
H 6.751885 1.347001 -1.332168
H 5.243475 1.989670 -1.938569
H 4.867080 -1.020931 0.969136
H 7.609775 0.115071 0.178224
H 7.095944 -1.603107 -1.497022
H 8.413191 -2.077179 -0.437940
H 5.534755 -3.113941 -0.232533
H 6.922593 -3.972193 -0.907843
H 6.882069 -3.601774 0.817482
H 7.242848 -0.151822 2.563143
H 8.435272 -1.329880 1.998372
H 6.779140 -1.844752 2.365200
SCF Energy (B3LYP/6-31G**)= -1361.70135656
Number of imaginary frequencies = 0

1a_c273
MMFF Geometry
C 1.625766 -2.267452 0.197515
C 2.528259 0.268496 -0.182155

N 2.965876 -2.068131 0.173258
C 0.664630 -1.263064 0.040652
C 1.144155 0.044896 -0.157887
C 3.401259 -0.803002 -0.007402
C 0.227530 1.096646 -0.333974
C -1.155122 0.867706 -0.300536
C -1.629555 -0.429432 -0.092344
C -0.726522 -1.493484 0.063996
C -3.094166 -0.658954 -0.075572
C -4.045355 0.544535 0.151711
C -3.389192 1.845148 -0.293119
C -2.076085 1.977059 -0.522718
O -1.155117 -2.788143 0.240941
O -3.493975 -1.819692 -0.203480
O -4.289618 0.681995 1.553797
C -5.351285 0.302737 -0.634213
C -6.150603 -0.864364 -0.088861
C -6.908468 -1.701545 -1.082913
O -6.219223 -1.079954 1.123244
O -4.212121 2.938597 -0.540303
C -4.658549 3.541976 0.676504
C 4.872427 -0.626961 -0.028544
C 5.594275 -1.609085 -0.905748
C 5.441021 0.350021 0.710320
C 6.893728 0.717519 0.944112
C 7.299935 1.975795 0.151319
C 7.289718 1.819747 -1.363814
C 7.928276 -0.404609 0.827978
H 1.330280 -3.302960 0.352948
H 2.923493 1.266085 -0.347820
H 0.600806 2.107260 -0.496824
H -1.674148 2.928626 -0.858305
H -2.133653 -2.811096 0.141039
H -4.878236 -0.060739 1.815558
H -5.125565 0.115054 -1.691033
H -6.026481 1.164624 -0.580248
H -7.427182 -2.511493 -0.562752
H -7.644511 -1.079830 -1.598226
H -6.211073 -2.137276 -1.802617
H -5.450880 2.945305 1.137730
H -3.831427 3.695476 1.378400
H -5.079029 4.520866 0.427619
H 4.945336 -1.994609 -1.701130
H 6.436645 -1.142628 -1.421959
H 5.952784 -2.461286 -0.320032
H 4.774718 0.984272 1.299402
H 6.931127 1.009187 2.005007
H 6.628082 2.802242 0.417435
H 8.304857 2.286722 0.463637
H 6.292751 1.567130 -1.736301
H 7.588894 2.761657 -1.835514
H 7.991042 1.048798 -1.694634
H 7.604882 -1.296917 1.374683
H 8.879965 -0.082091 1.266199
H 8.139363 -0.689855 -0.204500
SCF Energy (B3LYP/6-31G**)= -1361.69852834
Number of imaginary frequencies = 0

1a_c274
MMFF Geometry
C 1.876986 2.205002 -0.052593
C 2.367267 -0.427933 -0.517330
N 3.168438 1.799593 -0.108638
C 0.768809 1.367643 -0.215918
C 1.035226 0.006944 -0.459359
C 3.400564 0.487914 -0.330239
C -0.036434 -0.884908 -0.646726
C -1.364966 -0.445388 -0.579306
C -1.624723 0.903974 -0.321053
C -0.568018 1.811179 -0.155909
C -3.034711 1.349283 -0.235824
C -4.145460 0.298182 -0.035437
C -3.733556 -1.034727 -0.623303
C -2.455433 -1.385736 -0.828441
O -0.790951 3.148497 0.069658
O -3.290689 2.557083 -0.279689
O -5.290600 0.805578 -0.738220
C -4.452982 0.261112 1.464919
C -5.367608 -0.865620 1.891944
C -6.813728 -0.803523 1.473235
O -4.944790 -1.783212 2.598262
O -4.748375 -1.953106 -0.842131
C -5.112176 -2.009421 -2.221814
C 4.827378 0.087190 -0.386202
C 5.695177 1.031705 -1.170374
C 5.244590 -1.025431 0.256029
C 6.637054 -1.607637 0.389519
C 7.716192 -0.655881 0.941126
C 7.354797 -0.050424 2.291690
C 7.091494 -2.274831 -0.911236
H 1.750333 3.269119 0.135306
H 2.597695 -1.469520 -0.719713
H 0.172871 -1.935362 -0.847710
H -2.211552 -2.381714 -1.187139
H -1.750529 3.334120 -0.038729
H -5.259286 1.780170 -0.654609
H -4.920279 1.198617 1.792752
H -3.525527 0.160168 2.044601
H -7.380487 -1.569000 2.011206
H -7.228490 0.174639 1.729476
H -6.912353 -0.984933 0.402144
H -5.964777 -2.688202 -2.316322
H -5.414523 -1.027215 -2.598572
H -4.290511 -2.408866 -2.825585
H 5.924780 1.924921 -0.579786
H 6.638337 0.580227 -1.482038
H 5.193435 1.346676 -2.092927
H 4.504467 -1.603072 0.812167
H 6.546654 -2.418765 1.127382
H 8.651288 -1.218184 1.060972
H 7.938780 0.150111 0.235460
H 7.135055 -0.831871 3.026036
H 8.192041 0.543853 2.671773
H 6.485978 0.610327 2.218026
H 6.338391 -2.983596 -1.273581
H 8.020253 -2.833576 -0.751315
H 7.277675 -1.546957 -1.706251
SCF Energy (B3LYP/6-31G**)= -1361.69646260
Number of imaginary frequencies = 0

1a_c275
MMFF Geometry
C -1.714883 -1.474908 -0.615557
C -2.181677 1.075331 0.194686
N -2.999089 -1.094870 -0.409275
C -0.601655 -0.648945 -0.443037
C -0.855102 0.669755 -0.022788
C -3.227932 0.175742 -0.005032
C 0.223270 1.554447 0.164516
C 1.544968 1.141995 -0.049331
C 1.789563 -0.175267 -0.452732
C 0.726973 -1.063558 -0.666637
C 3.190434 -0.587748 -0.684170
C 4.325136 0.241389 -0.050054
C 3.921097 1.698516 0.069599
C 2.640929 2.099316 0.108062
O 0.933994 -2.350837 -1.099757
O 3.430242 -1.580844 -1.377682
O 5.451837 0.145387 -0.938903
C 4.746335 -0.285044 1.323853
C 4.754348 -1.793070 1.390702
C 6.015201 -2.507953 0.985498
O 3.742353 -2.408591 1.728118
O 4.941593 2.603425 0.320413
C 5.313981 3.302830 -0.867653
C -4.655245 0.562284 0.196032
C -4.996588 2.006288 -0.044057
C -5.549544 -0.390833 0.541113
C -7.033582 -0.297720 0.826279
C -7.903179 -0.129027 -0.433394
C -7.719674 -1.249523 -1.449707
C -7.384770 0.744796 1.892493

H -1.599864 -2.507840 -0.937236
H -2.386657 2.086236 0.531403
H 0.023831 2.578269 0.479318
H 2.397888 3.144071 0.278546
H 1.891807 -2.477848 -1.279515
H 5.300410 -0.642405 -1.499891
H 4.057285 0.054486 2.108773
H 5.743979 0.075403 1.603242
H 5.923907 -3.571675 1.222700
H 6.178599 -2.396693 -0.088163
H 6.864344 -2.102335 1.541040
H 5.633052 2.615822 -1.657537
H 4.490505 3.930809 -1.224094
H 6.157110 3.955280 -0.622460
H -4.471423 2.392551 -0.925287
H -6.057761 2.163784 -0.243310
H -4.722419 2.617008 0.822394
H -5.175816 -1.411791 0.655140
H -7.307493 -1.266397 1.270312
H -8.959328 -0.107433 -0.136136
H -7.701243 0.829493 -0.923228
H -7.915696 -2.226444 -0.996505
H -8.417408 -1.117622 -2.282978
H -6.706538 -1.255862 -1.863056
H -6.730695 0.651838 2.766456
H -8.415534 0.600854 2.235907
H -7.311795 1.769649 1.519472
SCF Energy (B3LYP/6-31G**)= -1361.69464176
Number of imaginary frequencies = 0

1a_c276
MMFF Geometry
C 1.802542 2.411710 0.089335
C 2.434152 -0.230354 -0.006630
N 3.107412 2.066070 0.204398
C 0.747945 1.507940 -0.075065
C 1.087181 0.142495 -0.122193
C 3.410849 0.751567 0.147095
C 0.072001 -0.817138 -0.287981
C -1.272391 -0.438186 -0.396453
C -1.605632 0.918384 -0.336430
C -0.604120 1.889534 -0.192152
C -3.032863 1.301469 -0.435827
C -4.118052 0.230041 -0.205374
C -3.602419 -1.143589 -0.578330
C -2.298374 -1.454317 -0.620641
O -0.897562 3.231764 -0.155853
O -3.328057 2.480891 -0.655095
O -5.207713 0.590391 -1.068806
C -4.567655 0.363516 1.253068
C -5.478792 -0.743943 1.734537
C -6.878477 -0.803383 1.179750
O -5.094466 -1.545583 2.588525
O -4.556746 -2.130609 -0.768636
C -4.784552 -2.375063 -2.156937
C 4.849003 0.415373 0.283103
C 5.552825 1.182937 1.367440
C 5.412590 -0.493240 -0.542135
C 6.846697 -0.974597 -0.626197
C 7.275426 -1.882070 0.541887
C 6.400604 -3.120036 0.697865
C 7.850749 0.159666 -0.853200
H 1.618108 3.482907 0.136471
H 2.718408 -1.278212 -0.022413
H 0.338502 -1.872845 -0.334000
H -1.984492 -2.473954 -0.825416
H -1.848156 3.355890 -0.374864
H -5.221067 1.568229 -1.107011
H -5.097461 1.311076 1.414941
H -3.697523 0.378827 1.923166
H -7.467742 -1.521212 1.757741
H -7.350401 0.178460 1.267861
H -6.865745 -1.122281 0.136671
H -5.086027 -1.463112 -2.681855
H -3.894540 -2.805259 -2.628147
H -5.598204 -3.101430 -2.240400
H 6.502403 0.732472 1.659607
H 5.752485 2.210549 1.045575
H 4.942150 1.217856 2.277297
H 4.781521 -0.951643 -1.305215
H 6.896095 -1.591533 -1.535837
H 8.308229 -2.212896 0.373999
H 7.279292 -1.330057 1.487566
H 6.374411 -3.701481 -0.229189
H 6.799323 -3.763379 1.488877
H 5.374387 -2.855868 0.970726
H 7.522556 0.820895 -1.662773
H 8.826481 -0.250209 -1.137684
H 8.006056 0.768586 0.041247
SCF Energy (B3LYP/6-31G**)= -1361.69648722
Number of imaginary frequencies = 0

1a_c277
MMFF Geometry
C -1.674657 -2.508773 -0.127936
C -2.470000 0.080958 -0.341977
N -3.004602 -2.249764 -0.123196
C -0.672393 -1.539036 -0.233333
C -1.096144 -0.202622 -0.345804
C -3.389170 -0.959314 -0.221746
C -0.135308 0.817821 -0.465413
C 1.237900 0.530584 -0.462039
C 1.653774 -0.797044 -0.341165
C 0.707252 -1.829328 -0.238730
C 3.102785 -1.091916 -0.337127
C 4.109394 0.058725 -0.151472
C 3.524302 1.423647 -0.477496
C 2.201019 1.622423 -0.614550
O 1.080996 -3.148508 -0.137954
O 3.469844 -2.265558 -0.452729
O 5.146835 -0.225134 -1.102914
C 4.637135 0.014304 1.291599
C 6.144864 0.041927 1.387433
C 6.848382 1.357430 1.190595
O 6.778299 -0.994782 1.596091
O 4.483084 2.415570 -0.582721
C 4.094352 3.696480 -1.063902
C -4.852446 -0.722171 -0.218103
C -5.643072 -1.712319 -1.030746
C -5.382109 0.296219 0.493048
C -6.848084 0.647856 0.642942
C -7.125730 1.980670 -0.075425
C -8.598621 2.366095 -0.090050
C -7.201947 0.703791 2.132323
H -1.423996 -3.564049 -0.041433
H -2.820626 1.103072 -0.446572
H -0.469745 1.850386 -0.562646
H 1.789048 2.601697 -0.832678
H 2.055237 -3.213877 -0.264394
H 5.391622 -1.165331 -0.979533
H 4.310646 -0.902564 1.801370
H 4.232331 0.826883 1.908074
H 6.939548 1.575487 0.124888
H 6.298453 2.154983 1.696331
H 7.847982 1.302911 1.631313
H 3.637110 3.621835 -2.055844
H 3.420577 4.189760 -0.356308
H 4.995923 4.309764 -1.152616
H -5.766491 -2.650876 -0.480239
H -6.635697 -1.342184 -1.299711
H -5.136979 -1.930581 -1.978538
H -4.714845 0.940503 1.066056
H -7.478384 -0.126686 0.194719
H -6.550441 2.791750 0.389058
H -6.782691 1.909401 -1.115883
H -8.967144 2.583903 0.916493
H -8.744321 3.265899 -0.696519
H -9.210117 1.566266 -0.519345
H -6.920238 -0.227556 2.636986
H -8.278458 0.837342 2.278255
H -6.687424 1.529078 2.637347
SCF Energy (B3LYP/6-31G**)= -1361.70294183

Number of imaginary frequencies = 0

1a_c278
MMFF Geometry
C 1.568609 -2.138595 0.050031
C 2.377463 0.450124 0.230487
N 2.895608 -1.895151 0.176806
C 0.576462 -1.154150 0.004465
C 1.006883 0.182756 0.099625
C 3.286891 -0.605427 0.258280
C 0.056989 1.220052 0.055656
C -1.311774 0.945101 -0.067202
C -1.732932 -0.383780 -0.146016
C -0.799047 -1.429756 -0.129927
C -3.181215 -0.648439 -0.276409
C -4.164406 0.435296 0.229132
C -3.598280 1.829350 -0.057791
C -2.273669 2.044476 -0.175547
O -1.184289 -2.744176 -0.245016
O -3.529966 -1.735684 -0.741901
O -4.277628 0.351667 1.653253
C -5.537542 0.236700 -0.445302
C -6.188683 -1.074799 -0.051719
C -7.056077 -1.745195 -1.082115
O -6.057853 -1.539167 1.083163
O -4.564731 2.818576 -0.132651
C -4.146842 4.176879 -0.199024
C 4.746508 -0.382590 0.403460
C 5.408348 -1.315176 1.379813
C 5.361761 0.572719 -0.327034
C 6.825880 0.962903 -0.386923
C 7.742986 -0.199429 -0.812261
C 9.158313 0.248508 -1.155206
C 7.278822 1.669524 0.893021
H 1.311170 -3.193670 -0.015324
H 2.731884 1.472028 0.323857
H 0.394396 2.253896 0.118470
H -1.860827 3.031521 -0.348874
H -2.145087 -2.771491 -0.454460
H -4.805463 -0.452596 1.851736
H -5.430681 0.266017 -1.536310
H -6.251832 1.013516 -0.147196
H -7.451444 -2.681220 -0.678135
H -7.889913 -1.088294 -1.341027
H -6.461894 -1.970545 -1.971133
H -3.540345 4.444437 0.672286
H -3.606413 4.373377 -1.130402
H -5.042785 4.804659 -0.191775
H 5.510895 -2.316734 0.948590
H 6.400999 -0.980456 1.683544
H 4.819010 -1.393520 2.300920
H 4.754507 1.152877 -1.023680
H 6.889320 1.715657 -1.186703
H 7.806904 -0.960257 -0.028064
H 7.316935 -0.693175 -1.695052
H 9.678788 0.639575 -0.276126
H 9.738410 -0.599608 -1.533195
H 9.149990 1.023237 -1.928286
H 6.550433 2.427173 1.203695
H 8.232081 2.185108 0.736700
H 7.414757 0.972500 1.724572
SCF Energy (B3LYP/6-31G**)= -1361.70626456
Number of imaginary frequencies = 0

1a_c279
MMFF Geometry
C -1.704513 -2.387778 -0.111998
C -2.424422 0.182720 0.397271
N -3.011343 -2.131163 0.137421
C -0.689848 -1.425314 -0.125171
C -1.074122 -0.098955 0.141865
C -3.360148 -0.849589 0.379805
C -0.098521 0.914464 0.144964
C 1.251720 0.627727 -0.105735
C 1.629039 -0.691536 -0.365183
C 0.665483 -1.712884 -0.386093
C 3.054139 -0.986322 -0.627411
C 4.119288 0.079149 -0.308174
C 3.549154 1.485428 -0.213559
C 2.227783 1.718637 -0.118904
O 0.999416 -3.019433 -0.653472
O 3.358305 -2.098575 -1.070031
O 5.012667 0.030378 -1.431377
C 4.828089 -0.318405 0.996641
C 6.335418 -0.323313 0.891201
C 7.057267 0.996582 0.923540
O 6.950395 -1.383097 0.756277
O 4.523245 2.467937 -0.204737
C 4.124440 3.829685 -0.304534
C -4.798793 -0.614304 0.649522
C -5.441927 -1.652703 1.529177
C -5.435641 0.443180 0.102187
C -6.901077 0.804947 0.232062
C -7.528582 0.900017 -1.172275
C -9.033374 1.131689 -1.149391
C -7.024647 2.114380 1.014276
H -1.483709 -3.434939 -0.308525
H -2.741692 1.196497 0.621318
H -0.403301 1.941362 0.345172
H 1.828564 2.723699 -0.036762
H 1.945303 -3.057130 -0.924196
H 5.235642 -0.912781 -1.572266
H 4.535749 -1.329994 1.310220
H 4.538325 0.321757 1.839509
H 8.102922 0.830721 1.198313
H 7.017147 1.467492 -0.060445
H 6.608907 1.649698 1.676331
H 3.539856 4.002645 -1.213894
H 3.567448 4.138123 0.585754
H 5.029578 4.441327 -0.363180
H -4.779759 -1.922780 2.360253
H -6.370704 -1.304060 1.987514
H -5.664369 -2.558706 0.955665
H -4.873393 1.117812 -0.544113
H -7.443103 0.031500 0.784800
H -7.054554 1.704504 -1.749029
H -7.335912 -0.032721 -1.718346
H -9.282783 2.112667 -0.734882
H -9.433854 1.092319 -2.167602
H -9.540611 0.364010 -0.556679
H -6.510531 2.043897 1.979769
H -8.071979 2.353585 1.223358
H -6.589241 2.954933 0.462200
SCF Energy (B3LYP/6-31G**)= -1361.70292045
Number of imaginary frequencies = 0

1a_c280
MMFF Geometry
C 1.840879 1.980617 0.064039
C 2.191076 -0.712433 0.020985
N 3.097595 1.497186 0.212817
C 0.699924 1.193784 -0.110401
C 0.893148 -0.199123 -0.133195
C 3.268947 0.155197 0.192267
C -0.215481 -1.046363 -0.317089
C -1.510188 -0.528835 -0.461835
C -1.695683 0.854716 -0.423354
C -0.600234 1.715781 -0.264959
C -3.069262 1.382884 -0.558429
C -4.260651 0.434688 -0.325409
C -3.908521 -1.004767 -0.669159
C -2.633888 -1.435619 -0.706891
O -0.750103 3.081836 -0.251024
O -3.234130 2.582600 -0.802126
O -5.301524 0.900872 -1.200206
C -4.708809 0.634804 1.126737
C -5.727067 -0.377845 1.601938
C -7.142383 -0.245011 1.102533
O -5.405531 -1.271437 2.388352
O -5.016103 -1.799027 -0.905257
C -4.830810 -3.190841 -1.128997
C 4.664831 -0.340050 0.377402

C 4.814296 -1.690725 1.020399
C 5.695909 0.447015 -0.003752
C 7.192155 0.209820 0.039299
C 7.629661 -1.076612 -0.687086
C 9.133777 -1.149548 -0.920505
C 7.738910 0.327196 1.463900
H 1.773309 3.066082 0.090982
H 2.351733 -1.785207 -0.007890
H -0.061023 -2.124638 -0.347967
H -2.382763 -2.466260 -0.932429
H -1.675669 3.302508 -0.499974
H -5.184470 1.867872 -1.287855
H -5.145126 1.631434 1.271120
H -3.850596 0.573309 1.809281
H -7.792668 -0.915612 1.671657
H -7.490566 0.780056 1.252299
H -7.201952 -0.511974 0.046321
H -4.254495 -3.366120 -2.042933
H -4.353746 -3.666008 -0.265803
H -5.817530 -3.644496 -1.260789
H 4.632715 -2.487432 0.291543
H 5.804670 -1.849573 1.449754
H 4.110226 -1.809312 1.852129
H 5.450716 1.422017 -0.432873
H 7.630449 1.045872 -0.525683
H 7.329705 -1.968850 -0.129040
H 7.129246 -1.131395 -1.662319
H 9.682859 -1.219091 0.023001
H 9.378324 -2.037762 -1.512096
H 9.490827 -0.271149 -1.467191
H 7.344527 1.218619 1.964889
H 8.829957 0.418202 1.457373
H 7.484100 -0.542518 2.076226
SCF Energy (B3LYP/6-31G**)= -1361.70292938
Number of imaginary frequencies = 0

1a_c281
MMFF Geometry
C -1.451037 -2.012115 -0.787058
C -2.222573 0.557038 -0.348245
N -2.778956 -1.749756 -0.721635
C -0.440045 -1.056234 -0.646412
C -0.851413 0.271421 -0.422221
C -3.150857 -0.472219 -0.493148
C 0.117627 1.283076 -0.287220
C 1.486391 0.990203 -0.354921
C 1.885843 -0.333413 -0.560170
C 0.935829 -1.350579 -0.724382
C 3.331716 -0.616479 -0.637578
C 4.315642 0.395125 -0.018683
C 3.785138 1.820455 -0.119167
C 2.470465 2.074479 -0.265207
O 1.302324 -2.651642 -0.969665
O 3.724428 -1.649429 -1.187564
O 5.536004 0.302852 -0.776431
C 4.639014 0.079475 1.443654
C 4.784021 -1.401459 1.697921
C 6.131370 -2.028491 1.459873
O 3.811798 -2.071777 2.048383
O 4.770185 2.783857 0.004829
C 4.414580 4.150895 -0.157583
C -4.612881 -0.227091 -0.432576
C -5.393004 -0.941858 -1.500967
C -5.128181 0.557096 0.539177
C -6.569650 0.923371 0.834999
C -7.459051 -0.303546 1.111836
C -8.820915 0.061494 1.689308
C -7.142085 1.875338 -0.218001
H -1.209259 -3.058145 -0.963846
H -2.564935 1.575560 -0.193296
H -0.206588 2.311126 -0.129348
H 2.081161 3.085566 -0.311134
H 2.278718 -2.694633 -1.073955
H 5.472120 -0.505878 -1.322915
H 3.842102 0.432082 2.112019
H 5.563348 0.573507 1.767442
H 6.131348 -3.050873 1.848282
H 6.347014 -2.054916 0.390004
H 6.901927 -1.460301 1.986757
H 3.982686 4.329075 -1.147679
H 3.729376 4.472071 0.633331
H 5.327299 4.748349 -0.075002
H -5.468756 -2.010727 -1.274315
H -6.405583 -0.554666 -1.620988
H -4.906617 -0.828516 -2.476915
H -4.439265 0.982674 1.270651
H -6.532852 1.496857 1.773120
H -7.620741 -0.888521 0.201026
H -6.951461 -0.965973 1.824695
H -9.424000 0.622395 0.969385
H -9.374127 -0.847335 1.947527
H -8.715380 0.662223 2.598107
H -6.435973 2.682096 -0.445259
H -8.062844 2.346003 0.142065
H -7.379617 1.362427 -1.154106
SCF Energy (B3LYP/6-31G**)= -1361.69850544
Number of imaginary frequencies = 0

1a_c282
MMFF Geometry
C -1.540341 -2.320499 -0.341386
C -2.265217 0.266324 0.063454
N -2.858641 -2.048080 -0.184083
C -0.517049 -1.367008 -0.314339
C -0.904831 -0.032361 -0.103352
C -3.207724 -0.758379 0.010051
C 0.078187 0.971846 -0.062481
C 1.438380 0.670326 -0.231777
C 1.824263 -0.658610 -0.437191
C 0.850207 -1.672083 -0.481379
C 3.262858 -0.980206 -0.625261
C 4.307882 0.162177 -0.592357
C 3.722591 1.539710 -0.355962
C 2.414570 1.755620 -0.172585
O 1.180934 -2.990657 -0.694446
O 3.573112 -2.151739 -0.864139
O 4.923927 0.167174 -1.898135
C 5.415216 -0.198494 0.406989
C 4.948472 -0.200767 1.846489
C 6.019510 -0.080418 2.898302
O 3.766509 -0.342724 2.157809
O 4.594674 2.604108 -0.159542
C 5.287846 2.988955 -1.345564
C -4.658149 -0.504347 0.186080
C -5.345160 -1.491766 1.088047
C -5.249909 0.529002 -0.451532
C -6.705885 0.952674 -0.472422
C -7.087150 1.699865 0.818353
C -8.472878 2.329207 0.765431
C -7.669687 -0.180926 -0.835768
H -1.316671 -3.373962 -0.495992
H -2.585297 1.287189 0.247672
H -0.225750 2.004343 0.107748
H 2.060500 2.755945 0.061115
H 2.159643 -3.057350 -0.778194
H 4.209024 0.228770 -2.555317
H 6.248748 0.508643 0.312074
H 5.831923 -1.189911 0.189848
H 5.563474 -0.091371 3.892137
H 6.710209 -0.923001 2.814950
H 6.556007 0.862797 2.768937
H 6.150389 2.338205 -1.514255
H 4.626798 2.994366 -2.219127
H 5.662694 4.006143 -1.197641
H -4.715829 -1.736135 1.951989
H -6.278950 -1.105247 1.500482
H -5.565421 -2.418789 0.548406
H -4.630188 1.157424 -1.092971
H -6.782662 1.677503 -1.296442
H -7.041991 1.028155 1.682906
H -6.355742 2.496864 1.005171
H -9.256471 1.567947 0.707745

H -8.651267 2.919754 1.669895
H -8.569887 2.995027 -0.097827
H -7.273055 -0.792033 -1.654129
H -8.629222 0.225358 -1.174044
H -7.879803 -0.838417 0.012035
SCF Energy (B3LYP/6-31G**)= -1361.69409038
Number of imaginary frequencies = 0

1a_c283
MMFF Geometry
C -1.546462 -1.732582 -0.537240
C -2.114851 0.800857 0.260649
N -2.839666 -1.417480 -0.282698
C -0.472970 -0.846364 -0.421757
C -0.778795 0.463542 -0.009694
C -3.119063 -0.155699 0.116806
C 0.257175 1.408618 0.112814
C 1.589616 1.063610 -0.151339
C 1.886639 -0.245298 -0.542361
C 0.865086 -1.192526 -0.697306
C 3.298416 -0.587806 -0.816646
C 4.392848 0.297826 -0.175529
C 3.941545 1.755513 -0.107519
C 2.637065 2.087888 -0.080447
O 1.123611 -2.472822 -1.123348
O 3.539200 -1.572205 -1.517514
O 5.577902 0.216624 -0.978548
C 4.761887 -0.190526 1.227078
C 4.849562 -1.696890 1.305949
C 6.092360 -2.363744 0.779838
O 3.908331 -2.350073 1.760094
O 4.992296 2.652474 -0.054150
C 4.702536 4.042225 0.041764
C -4.551325 0.148354 0.406711
C -4.812734 1.203228 1.445247
C -5.513070 -0.547937 -0.239815
C -7.023748 -0.449993 -0.170250
C -7.559785 0.966234 -0.454741
C -9.063317 0.998318 -0.700810
C -7.567349 -1.064340 1.121750
H -1.389329 -2.762881 -0.849639
H -2.363069 1.811588 0.567157
H 0.013957 2.426342 0.416010
H 2.302961 3.117358 -0.011097
H 2.077333 -2.544245 -1.355024
H 5.402094 0.714017 -1.796154
H 4.011364 0.119830 1.966178
H 5.722325 0.222061 1.559882
H 6.124299 -3.399738 1.128955
H 6.088196 -2.355660 -0.311818
H 6.978442 -1.847764 1.157891
H 4.147270 4.386979 -0.836395
H 4.156711 4.264692 0.964233
H 5.653152 4.582686 0.073470
H -4.694570 2.204073 1.017137
H -5.814171 1.138474 1.873524
H -4.123353 1.100321 2.291250
H -5.186861 -1.311516 -0.950879
H -7.389583 -1.087255 -0.988989
H -7.337093 1.648086 0.371678
H -7.059230 1.372712 -1.342835
H -9.622317 0.715630 0.195925
H -9.375542 2.009773 -0.980417
H -9.344137 0.321577 -1.513888
H -7.105573 -2.038450 1.319332
H -8.647532 -1.228738 1.051964
H -7.386394 -0.424618 1.990264
SCF Energy (B3LYP/6-31G**)= -1361.69423456
Number of imaginary frequencies = 0

1a_c284
MMFF Geometry
C 1.505017 2.488771 -0.075972
C 2.294834 -0.090837 -0.398388
N 2.834653 2.230068 -0.102579
C 0.499891 1.524161 -0.201745
C 0.921465 0.193438 -0.370314
C 3.216712 0.943871 -0.254670
C -0.041298 -0.821677 -0.507634
C -1.413798 -0.533683 -0.477918
C -1.832230 0.789199 -0.308150
C -0.880445 1.814243 -0.171239
C -3.285017 1.089404 -0.263764
C -4.313282 -0.044588 -0.440359
C -3.692547 -1.409260 -0.628525
C -2.371885 -1.626117 -0.611971
O -1.245960 3.129020 -0.000277
O -3.644019 2.262880 -0.117439
O -4.969194 0.316216 -1.677497
C -5.363153 0.018600 0.678780
C -4.922096 -0.574633 2.000626
C -3.849729 0.140519 2.779239
O -5.432413 -1.605334 2.440331
O -4.530147 -2.514890 -0.680610
C -5.330141 -2.557083 -1.860883
C 4.679680 0.706395 -0.282139
C 5.459011 1.726079 -1.068607
C 5.218976 -0.338576 0.381628
C 6.686529 -0.696953 0.495612
C 6.951368 -2.001496 -0.277408
C 8.423327 -2.387281 -0.329205
C 7.062891 -0.810182 1.976197
H 1.257225 3.540119 0.054318
H 2.642040 -1.109076 -0.545022
H 0.288648 -1.852300 -0.638615
H -1.993364 -2.641418 -0.698476
H -2.226779 3.193982 -0.026003
H -5.293803 1.230990 -1.569828
H -6.272439 -0.511798 0.365224
H -5.695195 1.047577 0.865547
H -3.914866 -0.147100 3.832653
H -2.865295 -0.133544 2.396958
H -3.996822 1.221447 2.715240
H -6.199796 -1.901620 -1.760709
H -4.749335 -2.301331 -2.753847
H -5.697723 -3.580948 -1.977935
H 5.591349 2.643261 -0.485130
H 6.447254 1.366084 -1.365962
H 4.939120 1.979948 -1.999918
H 4.559723 -1.004299 0.939379
H 7.311028 0.093681 0.067826
H 6.382064 -2.829304 0.164383
H 6.592670 -1.890141 -1.309030
H 8.806785 -2.643790 0.662532
H 8.558541 -3.263312 -0.971833
H 9.029353 -1.572095 -0.736821
H 6.790186 0.101383 2.520379
H 8.141306 -0.950109 2.100498
H 6.554939 -1.653784 2.457032
SCF Energy (B3LYP/6-31G**)= -1361.69738691
Number of imaginary frequencies = 0

1a_c285
MMFF Geometry
C 2.010796 1.647244 -0.009783
C 2.272454 -1.053693 0.068457
N 3.259976 1.127942 0.066860
C 0.834856 0.893962 -0.050287
C 0.982931 -0.502384 -0.010055
C 3.387941 -0.218303 0.105716
C -0.163406 -1.315639 -0.051373
C -1.450338 -0.761254 -0.127973
C -1.596757 0.628215 -0.164798
C -0.458449 1.452628 -0.129731
C -2.958532 1.215281 -0.237834
C -4.196760 0.293791 -0.212825
C -3.855038 -1.179943 -0.241416
C -2.604878 -1.652405 -0.169600
O -0.552138 2.824463 -0.167992
O -3.060309 2.445091 -0.310286
O -4.882064 0.619085 -1.427226
C -5.001608 0.669604 1.044304

C -6.463579 0.301988 0.953881
C -6.992373 -0.702779 1.942167
O -7.206053 0.856400 0.141711
O -4.903173 -2.091002 -0.206224
C -5.546873 -2.220740 -1.476336
C 4.777854 -0.751506 0.203779
C 4.945469 -2.107077 0.835744
C 5.809626 0.000101 -0.240503
C 7.281845 -0.351651 -0.258053
C 8.019259 0.490940 0.798005
C 9.489694 0.125040 0.946672
C 7.834758 -0.130521 -1.669700
H 1.979650 2.734360 -0.038608
H 2.394627 -2.131684 0.085749
H -0.046320 -2.398842 -0.024323
H -2.433648 -2.725157 -0.144028
H -1.498988 3.073192 -0.272709
H -5.397809 1.433506 -1.267177
H -4.988660 1.753669 1.219249
H -4.559958 0.221286 1.942979
H -7.992877 -1.023412 1.639107
H -6.343733 -1.580571 1.974640
H -7.044321 -0.243006 2.932032
H -6.293822 -1.433742 -1.610084
H -4.828026 -2.218565 -2.303211
H -6.071444 -3.180883 -1.485442
H 4.696119 -2.898561 0.121438
H 5.962212 -2.286225 1.194981
H 4.300348 -2.211593 1.715732
H 5.588818 0.986745 -0.653934
H 7.426849 -1.411263 -0.024429
H 7.940990 1.558789 0.556944
H 7.534745 0.353776 1.773563
H 10.057594 0.367695 0.043906
H 9.933186 0.685669 1.775944
H 9.609910 -0.942235 1.157441
H 7.242146 -0.676900 -2.412500
H 8.865502 -0.489589 -1.751361
H 7.823613 0.930250 -1.944533
SCF Energy (B3LYP/6-31G**)= -1361.70342994
Number of imaginary frequencies = 0

1a_c286
MMFF Geometry
C -1.806267 -1.689675 -0.348112
C -2.102295 0.986247 0.000053
N -3.061499 -1.199897 -0.202599
C -0.641537 -0.917867 -0.335947
C -0.807310 0.465430 -0.155562
C -3.205058 0.133793 -0.027404
C 0.326904 1.296548 -0.135424
C 1.620014 0.773576 -0.291420
C 1.783709 -0.605773 -0.463115
C 0.657203 -1.446660 -0.491403
C 3.150907 -1.163695 -0.633414
C 4.372392 -0.219049 -0.539225
C 4.016663 1.244256 -0.400213
C 2.759885 1.688672 -0.266715
O 0.767644 -2.805533 -0.677318
O 3.263680 -2.365366 -0.895023
O 5.101429 -0.362497 -1.777491
C 5.318852 -0.714345 0.561557
C 4.766976 -0.534799 1.957935
C 5.774826 -0.456547 3.074061
O 3.558842 -0.507303 2.190335
O 5.087618 2.118497 -0.281252
C 5.295610 2.866673 -1.479174
C -4.602201 0.639002 0.115362
C -4.858685 2.039757 -0.365689
C -5.546910 -0.182191 0.625792
C -7.016797 0.052588 0.902522
C -7.897770 0.060832 -0.360515
C -7.802552 -1.224872 -1.172905
C -7.282959 1.280807 1.778658
H -1.759774 -2.768422 -0.481054
H -2.237730 2.051396 0.155912
H 0.195388 2.368566 0.008346
H 2.569031 2.744944 -0.101142
H 1.720826 -3.031001 -0.776110
H 4.464414 -0.252672 -2.503789
H 6.275808 -0.180834 0.493750
H 5.553966 -1.777842 0.429953
H 5.258248 -0.326350 4.029048
H 6.356018 -1.381240 3.107443
H 6.434637 0.399555 2.912890
H 5.466583 2.207578 -2.336154
H 4.450235 3.533879 -1.677082
H 6.188791 3.482557 -1.339350
H -4.329489 2.235093 -1.305558
H -5.912035 2.229237 -0.578058
H -4.529046 2.770507 0.380124
H -5.234459 -1.190255 0.910723
H -7.341663 -0.807418 1.506995
H -8.944846 0.199711 -0.062816
H -7.646536 0.907778 -1.007832
H -8.049761 -2.096498 -0.558619
H -8.506319 -1.191654 -2.010867
H -6.799631 -1.365659 -1.587237
H -6.619903 1.295182 2.650679
H -8.314156 1.263556 2.149412
H -7.153412 2.220967 1.236342
SCF Energy (B3LYP/6-31G**)= -1361.69533641
Number of imaginary frequencies = 0

1a_c287
MMFF Geometry
C 1.999163 1.674542 -0.419630
C 2.290033 -1.020978 -0.280027
N 3.255289 1.166470 -0.416798
C 0.830167 0.912273 -0.353373
C 0.993359 -0.481060 -0.280118
C 3.397623 -0.177178 -0.349164
C -0.145452 -1.302722 -0.208424
C -1.439481 -0.759698 -0.207577
C -1.600784 0.626750 -0.279450
C -0.470483 1.459403 -0.355352
C -2.969489 1.202055 -0.270735
C -4.194102 0.273788 -0.123670
C -3.842096 -1.197791 -0.128472
C -2.585817 -1.659317 -0.133797
O -0.579133 2.828618 -0.430314
O -3.087451 2.428035 -0.376081
O -4.969886 0.553315 -1.294258
C -4.907504 0.686586 1.176354
C -6.368721 0.306334 1.206636
C -6.814046 -0.667541 2.264529
O -7.174030 0.827631 0.433336
O -4.876468 -2.114010 0.014623
C -5.611185 -2.290773 -1.199169
C 4.795009 -0.699412 -0.366187
C 4.994571 -2.093506 -0.896881
C 5.803970 0.093671 0.057878
C 7.278992 -0.234997 0.147018
C 7.779609 0.053971 1.575936
C 9.223696 -0.368797 1.809529
C 8.037159 0.572055 -0.908895
H 1.956178 2.760006 -0.479171
H 2.423623 -2.095635 -0.212463
H -0.016877 -2.383592 -0.154035
H -2.403447 -2.729430 -0.085106
H -1.533312 3.067445 -0.473477
H -5.480272 1.368930 -1.123451
H -4.891655 1.776017 1.313677
H -4.396704 0.271921 2.054304
H -7.831285 -1.004783 2.047381
H -6.156922 -1.539500 2.278232
H -6.796876 -0.174629 3.239550
H -6.372985 -1.513586 -1.303209
H -4.955519 -2.311935 -2.076734
H -6.126389 -3.254038 -1.137379
H 4.376435 -2.266722 -1.785373
H 6.022877 -2.285528 -1.213634

H 4.732100 -2.836243 -0.136402
H 5.558712 1.102966 0.396093
H 7.450181 -1.297578 -0.051590
H 7.681231 1.122468 1.806586
H 7.147206 -0.481729 2.295955
H 9.918950 0.228461 1.212671
H 9.489024 -0.229387 2.862551
H 9.369606 -1.424682 1.561056
H 7.618458 0.401748 -1.907456
H 9.091670 0.281687 -0.947776
H 7.989572 1.647411 -0.703404
SCF Energy (B3LYP/6-31G**)= -1361.70333929
Number of imaginary frequencies = 0

1a_c288
MMFF Geometry
C -1.548204 -2.315624 -0.465489
C -2.258476 0.163201 0.390672
N -2.854364 -2.090567 -0.184652
C -0.529301 -1.364613 -0.347145
C -0.909312 -0.086476 0.098855
C -3.198431 -0.852777 0.231125
C 0.070250 0.911176 0.245090
C 1.418307 0.657992 -0.048562
C 1.795187 -0.612445 -0.493419
C 0.826539 -1.619930 -0.641982
C 3.224083 -0.881315 -0.791246
C 4.267425 0.243540 -0.647280
C 3.690152 1.555725 -0.169795
C 2.396994 1.725374 0.130895
O 1.152202 -2.885395 -1.070956
O 3.545883 -2.007252 -1.186240
O 4.718433 0.429607 -2.008487
C 5.465172 -0.252428 0.176311
C 5.252008 -0.252653 1.675752
C 4.265026 -1.231797 2.254339
O 5.877322 0.512984 2.409879
O 4.562114 2.599739 0.106558
C 5.185091 3.133448 -1.060459
C -4.636430 -0.650814 0.529789
C -5.285340 -1.798007 1.256705
C -5.267763 0.475490 0.134466
C -6.731463 0.823145 0.313098
C -7.356945 1.116800 -1.064404
C -8.860568 1.350768 -1.009930
C -6.849282 2.011007 1.270618
H -1.331864 -3.327170 -0.802661
H -2.570875 1.138025 0.752617
H -0.227098 1.900352 0.593397
H 2.057506 2.681159 0.521814
H 2.115042 -2.918156 -1.267834
H 5.013660 -0.443619 -2.331100
H 6.343552 0.373076 -0.032596
H 5.770355 -1.263753 -0.120323
H 4.494365 -1.394618 3.311481
H 3.252061 -0.835769 2.168275
H 4.344351 -2.192821 1.740260
H 6.027928 2.508046 -1.367801
H 4.470311 3.256179 -1.881531
H 5.580251 4.121263 -0.805147
H -4.625615 -2.183402 2.043044
H -6.213354 -1.512074 1.757981
H -5.510749 -2.614960 0.563150
H -4.701467 1.231197 -0.410774
H -7.277993 -0.017184 0.752060
H -6.878220 1.991542 -1.522874
H -7.168400 0.268558 -1.735411
H -9.105463 2.265460 -0.462475
H -9.260128 1.456163 -2.023799
H -9.372334 0.510416 -0.530523
H -6.336587 1.803618 2.216975
H -7.895622 2.224029 1.510684
H -6.409036 2.918221 0.841618
SCF Energy (B3LYP/6-31G**)= -1361.69745331
Number of imaginary frequencies = 0

1a_c289
MMFF Geometry
C -1.658960 -1.685488 -0.618155
C -2.261832 0.738025 0.449882
N -2.957520 -1.414555 -0.341404
C -0.596179 -0.806389 -0.396196
C -0.920286 0.446557 0.155443
C -3.253624 -0.207436 0.192651
C 0.103725 1.382157 0.392853
C 1.441374 1.083274 0.101835
C 1.757707 -0.170315 -0.431026
C 0.748250 -1.106019 -0.696431
C 3.172464 -0.461159 -0.745100
C 4.278223 0.393196 -0.091108
C 3.778919 1.797110 0.201677
C 2.476494 2.092714 0.324329
O 1.023183 -2.330511 -1.255860
O 3.449151 -1.375028 -1.528823
O 5.344821 0.459223 -1.054049
C 4.825030 -0.228979 1.196292
C 4.921934 -1.733774 1.124320
C 6.192604 -2.330586 0.581914
O 3.970713 -2.438034 1.464676
O 4.713057 2.760072 0.558410
C 5.362254 3.330882 -0.577610
C -4.690657 0.048749 0.501391
C -4.971996 0.969701 1.655482
C -5.639938 -0.570859 -0.235033
C -7.150351 -0.486758 -0.173246
C -7.755328 0.929996 -0.218068
C -7.314265 1.737501 -1.432395
C -7.704822 -1.294053 1.003308
H -1.488123 -2.673046 -1.041454
H -2.523844 1.706227 0.863818
H -0.150908 2.356320 0.809216
H 2.171295 3.092483 0.620479
H 1.980440 -2.374521 -1.473888
H 5.222071 -0.298341 -1.662484
H 4.175871 -0.004338 2.053283
H 5.818103 0.166968 1.441972
H 7.050041 -1.934198 1.131341
H 6.170604 -3.416268 0.711901
H 6.287696 -2.102868 -0.481537
H 6.247873 2.747133 -0.842510
H 4.690211 3.423568 -1.438204
H 5.697761 4.334680 -0.299886
H -4.878200 2.015847 1.346223
H -5.969879 0.829986 2.074542
H -4.277801 0.785790 2.483637
H -5.300782 -1.243315 -1.027259
H -7.509374 -0.992883 -1.081841
H -8.849152 0.842465 -0.248886
H -7.525723 1.494372 0.690593
H -7.540164 1.204460 -2.361402
H -7.842542 2.696120 -1.456594
H -6.241620 1.950953 -1.407243
H -7.309772 -2.316181 1.000000
H -8.796533 -1.362279 0.941313
H -7.456766 -0.841318 1.967812
SCF Energy (B3LYP/6-31G**)= -1361.69378612
Number of imaginary frequencies = 0

1a_c290
MMFF Geometry
C 1.861779 1.897283 -0.017695
C 2.130107 -0.788107 -0.328822
N 3.108969 1.370303 0.028220
C 0.691911 1.159339 -0.213151
C 0.842359 -0.230369 -0.373162
C 3.238906 0.032698 -0.127260
C -0.297833 -1.029265 -0.580271
C -1.581051 -0.467355 -0.614407
C -1.723537 0.911736 -0.439746
C -0.597332 1.726617 -0.257727
C -3.088742 1.484240 -0.455489
C -4.273658 0.534651 -0.164519

C -3.996531 -0.854782 -0.728293
C -2.742863 -1.312347 -0.901923
O -0.708098 3.088813 -0.114129
O -3.207874 2.696365 -0.643710
O -5.444977 1.082883 -0.779634
C -4.491385 0.551140 1.352969
C -5.447607 -0.517565 1.832140
C -6.921873 -0.293237 1.619585
O -5.026910 -1.545029 2.368934
O -5.146439 -1.566638 -1.016704
C -5.031038 -2.957272 -1.297646
C 4.627795 -0.509800 -0.086633
C 4.890620 -1.739363 -0.910469
C 5.564684 0.137989 0.641352
C 7.022854 -0.190756 0.891000
C 7.900197 0.240140 -0.298173
C 9.393185 0.125250 -0.020959
C 7.262653 -1.635058 1.341467
H 1.826982 2.976762 0.113267
H 2.256951 -1.860394 -0.437294
H -0.177058 -2.103477 -0.717402
H -2.537694 -2.306893 -1.282844
H -1.636740 3.352496 -0.309163
H -5.369646 0.914558 -1.735218
H -4.882850 1.521298 1.684449
H -3.545969 0.402682 1.891222
H -7.488523 -0.999051 2.233686
H -7.190868 0.720155 1.927878
H -7.180357 -0.449336 0.570779
H -4.498680 -3.120731 -2.239861
H -4.542112 -3.485434 -0.472583
H -6.041242 -3.362580 -1.407026
H 4.354699 -1.695913 -1.865727
H 5.944079 -1.852001 -1.173611
H 4.574396 -2.639299 -0.373067
H 5.255055 1.046001 1.165264
H 7.314996 0.433736 1.748321
H 7.663711 -0.353330 -1.188570
H 7.678361 1.284685 -0.552694
H 9.696225 -0.917148 0.114873
H 9.963246 0.528319 -0.864277
H 9.671339 0.688442 0.875361
H 6.502610 -1.957943 2.061542
H 8.234765 -1.724419 1.838819
H 7.264320 -2.339284 0.505263
SCF Energy (B3LYP/6-31G**)= -1361.69656097
Number of imaginary frequencies = 0

1a_c291
MMFF Geometry
C -1.403792 -2.126522 -0.658676
C -2.291169 0.326012 0.107452
N -2.736478 -1.961375 -0.476730
C -0.443115 -1.125674 -0.482290
C -0.914335 0.140048 -0.084027
C -3.166743 -0.737265 -0.103335
C 0.001475 1.190818 0.108273
C 1.375991 0.996879 -0.080030
C 1.838623 -0.267303 -0.462053
C 0.938935 -1.319704 -0.679919
C 3.291786 -0.448285 -0.666401
C 4.265160 0.561311 -0.025803
C 3.627472 1.934365 0.067795
C 2.298675 2.121635 0.081132
O 1.360835 -2.560700 -1.091708
O 3.702105 -1.396346 -1.342820
O 5.408096 0.640463 -0.895377
C 4.741900 0.125285 1.361562
C 4.993558 -1.360555 1.448923
C 6.360725 -1.865198 1.073165
O 4.089187 -2.128795 1.778763
O 4.482704 2.996058 0.321851
C 4.756207 3.734193 -0.869654
C -4.630704 -0.602011 0.090800
C -5.256002 -1.749560 0.834270
C -5.285286 0.473043 -0.399422
C -6.765243 0.803414 -0.376458
C -7.196498 1.332541 1.003324
C -8.619295 1.875039 1.024787
C -7.654104 -0.324441 -0.909233
H -1.114337 -3.130505 -0.962126
H -2.676679 1.287919 0.431116
H -0.368007 2.171535 0.406230
H 1.885846 3.114599 0.233662
H 2.329382 -2.531178 -1.255797
H 5.396379 -0.167097 -1.448505
H 3.993080 0.356684 2.130907
H 5.662646 0.646183 1.651673
H 6.439243 -2.926994 1.323221
H 6.522778 -1.740397 0.000789
H 7.122786 -1.320940 1.636406
H 5.195709 3.099928 -1.645713
H 3.847692 4.216105 -1.246323
H 5.477965 4.517613 -0.620667
H -4.614461 -2.075770 1.661423
H -6.213313 -1.485140 1.287150
H -5.415928 -2.601396 0.165020
H -4.705378 1.224160 -0.937889
H -6.885863 1.632644 -1.089277
H -7.110914 0.548004 1.763494
H -6.517712 2.138709 1.310583
H -9.352757 1.082396 0.849823
H -8.837154 2.317514 2.002221
H -8.756254 2.650336 0.264460
H -7.217348 -0.785913 -1.801853
H -8.636665 0.065310 -1.197108
H -7.824147 -1.108509 -0.166577
SCF Energy (B3LYP/6-31G**)= -1361.69315109
Number of imaginary frequencies = 0

1a_c292
MMFF Geometry
C 2.089654 -1.658406 -0.096595
C 2.326421 1.046504 -0.066581
N 3.335261 -1.126140 -0.058506
C 0.906383 -0.916725 -0.122579
C 1.040664 0.482699 -0.106914
C 3.450464 0.221842 -0.042568
C -0.114407 1.285976 -0.137796
C -1.395983 0.717375 -0.171140
C -1.522825 -0.673326 -0.173104
C -0.381132 -1.489372 -0.164702
C -2.882991 -1.256683 -0.208331
C -4.068282 -0.370411 0.236616
C -3.817240 1.099194 -0.088732
C -2.573277 1.583879 -0.261708
O -0.471658 -2.860922 -0.199369
O -2.992425 -2.439428 -0.543559
O -4.094988 -0.503805 1.664962
C -5.343145 -0.950577 -0.410302
C -6.626888 -0.404660 0.178250
C -7.794371 -0.248144 -0.758171
O -6.729556 -0.152975 1.380737
O -4.970594 1.859210 -0.157671
C -4.853731 3.275167 -0.241049
C 4.838253 0.769486 -0.015021
C 5.038747 2.144588 -0.592448
C 5.838167 0.012746 0.489422
C 7.301970 0.365721 0.631683
C 8.188450 -0.530057 -0.253163
C 7.944672 -0.332164 -1.744063
C 7.712614 0.220580 2.101025
H 2.067570 -2.746048 -0.105831
H 2.437908 2.125348 -0.040549
H -0.007478 2.370059 -0.135285
H -2.385438 2.630294 -0.473545
H -1.417467 -3.106338 -0.318242
H -5.000062 -0.259955 1.956704
H -5.404967 -2.036314 -0.264327
H -5.331575 -0.764741 -1.491168
H -8.649905 0.162696 -0.215071
H -7.527850 0.440030 -1.564189

H -8.067496 -1.223438 -1.168116
H -4.295450 3.674291 0.611940
H -4.392256 3.572532 -1.187945
H -5.862618 3.697207 -0.211695
H 4.714354 2.911704 0.118347
H 6.079253 2.350686 -0.854913
H 4.471331 2.264621 -1.522570
H 5.591571 -0.984305 0.861428
H 7.485405 1.408993 0.356844
H 9.243251 -0.309390 -0.046636
H 8.034206 -1.587332 -0.002754
H 8.085021 0.715511 -2.028432
H 8.650324 -0.936918 -2.322714
H 6.933693 -0.636577 -2.031092
H 7.102056 0.864179 2.744365
H 8.759970 0.509880 2.240253
H 7.600464 -0.811719 2.451766
SCF Energy (B3LYP/6-31G**)= -1361.71294154
Number of imaginary frequencies = 0

1a_c293
MMFF Geometry
C -2.064462 -1.775488 -0.149469
C -2.356089 0.917898 -0.335322
N -3.316182 -1.276629 -0.293221
C -0.901304 -1.004555 -0.087552
C -1.064194 0.388746 -0.182275
C -3.458128 0.065440 -0.388450
C 0.068440 1.221078 -0.113728
C 1.356874 0.686642 0.031521
C 1.513360 -0.698872 0.109286
C 0.392645 -1.542453 0.066567
C 2.880528 -1.245891 0.261712
C 4.073092 -0.352341 -0.147862
C 3.769372 1.124095 0.089923
C 2.506451 1.585350 0.157567
O 0.510207 -2.908055 0.175422
O 2.993197 -2.408331 0.660779
O 4.196287 -0.551064 -1.563510
C 5.315031 -0.870264 0.606912
C 6.622453 -0.320512 0.076981
C 7.722392 -0.092140 1.078168
O 6.798213 -0.122128 -1.127022
O 4.898903 1.914670 0.198232
C 4.746123 3.329624 0.208398
C -4.848883 0.575607 -0.563853
C -5.006090 1.896620 -1.267099
C -5.887100 -0.165705 -0.117729
C -7.362058 0.164701 -0.167372
C -8.009130 0.063289 1.227918
C -7.459594 1.082677 2.218394
C -8.059656 -0.788842 -1.140418
H -2.019746 -2.860442 -0.082988
H -2.491651 1.992503 -0.397953
H -0.061542 2.300862 -0.175713
H 2.282248 2.635433 0.306591
H 1.451218 -3.123965 0.367237
H 5.113110 -0.298826 -1.807549
H 5.409803 -1.959727 0.517201
H 5.228567 -0.635006 1.674674
H 8.602567 0.314245 0.572372
H 7.388768 0.625679 1.831635
H 7.989147 -1.040432 1.550856
H 4.216278 3.657451 1.108284
H 5.745353 3.774676 0.225796
H 4.237386 3.676427 -0.696851
H -4.325945 1.967939 -2.123723
H -6.008790 2.044860 -1.675890
H -4.796014 2.724635 -0.582140
H -5.669180 -1.125256 0.356462
H -7.525542 1.184925 -0.528334
H -9.090689 0.223580 1.135618
H -7.875443 -0.944928 1.640098
H -7.574810 2.101920 1.836111
H -8.001856 1.013851 3.166913
H -6.399711 0.909696 2.428176
H -7.628461 -0.709147 -2.144681
H -9.126229 -0.551433 -1.217679
H -7.970459 -1.832068 -0.816485
SCF Energy (B3LYP/6-31G**)= -1361.71295450
Number of imaginary frequencies = 0

1a_c294
MMFF Geometry
C -1.888356 2.373156 0.129667
C -2.528080 -0.176054 -0.556946
N -3.189405 2.090482 -0.122801
C -0.841173 1.448351 0.061757
C -1.184691 0.133294 -0.296907
C -3.498574 0.818603 -0.453137
C -0.175458 -0.841112 -0.387770
C 1.167268 -0.527555 -0.127428
C 1.509047 0.780141 0.228864
C 0.508509 1.763390 0.325957
C 2.930859 1.113788 0.496602
C 4.020886 0.034345 0.324435
C 3.469026 -1.337284 0.002845
C 2.173938 -1.578366 -0.233528
O 0.796540 3.062539 0.674593
O 3.202883 2.267221 0.848261
O 4.642946 -0.023282 1.612849
C 4.972834 0.539272 -0.774817
C 6.354361 -0.065742 -0.697518
C 6.814492 -0.904233 -1.859873
O 7.098050 0.175725 0.254800
O 4.374270 -2.373735 -0.187266
C 4.884967 -2.876264 1.050070
C -4.932399 0.549761 -0.724129
C -5.624217 1.630828 -1.511209
C -5.518595 -0.566740 -0.240453
C -6.958636 -1.002748 -0.404265
C -7.785310 -0.742359 0.870398
C -7.345561 -1.510543 2.110863
C -7.012889 -2.469887 -0.841605
H -1.700400 3.410381 0.399242
H -2.812996 -1.181599 -0.851022
H -0.445666 -1.860094 -0.664264
H 1.852400 -2.579052 -0.509276
H 1.757564 3.128736 0.878195
H 5.284472 0.711991 1.665173
H 5.130085 1.623667 -0.701885
H 4.547585 0.370101 -1.771914
H 7.730421 -1.436156 -1.588227
H 6.052190 -1.640978 -2.121429
H 7.013405 -0.255925 -2.716642
H 5.723266 -2.265446 1.395535
H 4.107663 -2.939848 1.819629
H 5.263916 -3.886122 0.866449
H -5.068865 1.848817 -2.430646
H -6.638827 1.365615 -1.814648
H -5.696987 2.552482 -0.923472
H -4.920259 -1.244707 0.368815
H -7.434618 -0.432978 -1.208723
H -7.761782 0.330791 1.099958
H -8.835101 -0.988977 0.666893
H -6.316869 -1.268344 2.393003
H -7.990119 -1.248714 2.956588
H -7.421262 -2.591469 1.962236
H -6.524114 -2.600891 -1.813883
H -8.050351 -2.805806 -0.943641
H -6.509910 -3.132843 -0.129558
SCF Energy (B3LYP/6-31G**)= -1361.70192156
Number of imaginary frequencies = 0

1a_c295
MMFF Geometry
C -1.730793 -1.480978 -0.579633
C -2.201184 1.086535 0.171444
N -3.015673 -1.097762 -0.383656
C -0.618588 -0.650097 -0.424673
C -0.873948 0.677367 -0.035070
C -3.246305 0.181583 -0.008960

C 0.203515 1.566707 0.133769
C 1.526139 1.151061 -0.069375
C 1.772887 -0.173736 -0.442827
C 0.710965 -1.067899 -0.637087
C 3.173576 -0.588968 -0.667182
C 4.314253 0.263368 -0.073307
C 3.902616 1.720860 0.038287
C 2.620093 2.111267 0.076195
O 0.919342 -2.364854 -1.040076
O 3.411435 -1.603694 -1.330277
O 5.411604 0.148527 -0.996303
C 4.778065 -0.238530 1.296539
C 4.776255 -1.744634 1.399859
C 6.020562 -2.478984 0.978627
O 3.769839 -2.343547 1.780741
O 4.888132 2.657438 0.319015
C 5.615124 3.044641 -0.846876
C -4.674269 0.571206 0.181356
C -5.016523 2.009154 -0.091609
C -5.568231 -0.374718 0.546527
C -7.052750 -0.276537 0.827439
C -7.920704 -0.136834 -0.436908
C -7.734794 -1.279617 -1.427645
C -7.406325 0.789258 1.869576
H -1.614290 -2.520835 -0.877542
H -2.407409 2.104722 0.484659
H 0.002940 2.596992 0.426125
H 2.374513 3.156097 0.244761
H 1.877528 -2.494928 -1.215495
H 5.256404 -0.665535 -1.518224
H 4.119189 0.124381 2.096838
H 5.787344 0.119939 1.533567
H 5.921987 -3.538692 1.230399
H 6.163918 -2.382707 -0.099335
H 6.884949 -2.076118 1.512222
H 6.469372 2.379759 -1.000097
H 4.983048 3.072602 -1.741615
H 6.005845 4.052375 -0.676246
H -4.490625 2.375914 -0.980699
H -6.077584 2.161223 -0.295608
H -4.743913 2.639471 0.761192
H -5.193765 -1.392500 0.684030
H -7.326481 -1.235277 1.292645
H -8.977291 -0.109540 -0.141683
H -7.718905 0.810669 -0.947778
H -7.930616 -2.246332 -0.952980
H -8.431459 -1.167010 -2.264633
H -6.721068 -1.294295 -1.839333
H -6.753433 0.716417 2.746328
H -8.437465 0.652155 2.214658
H -7.333647 1.805578 1.473841
SCF Energy (B3LYP/6-31G**)= -1361.69390786
Number of imaginary frequencies = 0

1a_c296
MMFF Geometry
C 1.600998 2.225807 -0.250610
C 2.228133 -0.411277 -0.430116
N 2.912729 1.885868 -0.278216
C 0.537519 1.319005 -0.311309
C 0.875008 -0.042179 -0.407626
C 3.212768 0.571865 -0.355997
C -0.149616 -1.001957 -0.481152
C -1.502608 -0.629690 -0.458904
C -1.838068 0.724479 -0.354702
C -0.822148 1.694524 -0.285308
C -3.269691 1.122668 -0.339058
C -4.363559 0.031170 -0.440462
C -3.829431 -1.383117 -0.543055
C -2.522904 -1.672898 -0.529822
O -1.103872 3.038653 -0.199483
O -3.540560 2.327395 -0.306246
O -5.087567 0.314305 -1.657126
C -5.362878 0.212966 0.709868
C -4.776218 -0.095080 2.070378
C -5.759046 -0.392208 3.172061
O -3.566355 -0.059722 2.291923
O -4.733478 -2.437367 -0.485593
C -5.542697 -2.548151 -1.655266
C 4.658833 0.240932 -0.384290
C 5.470086 1.139478 -1.275999
C 5.135983 -0.772866 0.370247
C 6.556106 -1.275276 0.543706
C 7.516867 -0.195474 1.076584
C 8.859999 -0.757220 1.525845
C 7.064453 -1.988097 -0.711763
H 1.417713 3.295646 -0.175990
H 2.511452 -1.455614 -0.517747
H 0.116044 -2.056371 -0.552154
H -2.200371 -2.710463 -0.537417
H -2.081597 3.151604 -0.174243
H -4.435017 0.365525 -2.377073
H -6.235389 -0.433341 0.552268
H -5.746935 1.240326 0.737055
H -5.220459 -0.600228 4.100742
H -6.409701 0.472276 3.324622
H -6.352764 -1.270427 2.906645
H -6.383080 -1.849981 -1.608073
H -4.958985 -2.394392 -2.569468
H -5.953666 -3.561791 -1.681770
H 5.608500 2.121766 -0.811733
H 6.457971 0.736509 -1.502593
H 4.970440 1.280245 -2.241621
H 4.429324 -1.317771 0.998528
H 6.493678 -2.046932 1.325343
H 7.704708 0.574317 0.321560
H 7.053955 0.311012 1.933211
H 9.424052 -1.168594 0.683688
H 9.466654 0.036091 1.974529
H 8.727276 -1.544907 2.274083
H 6.311266 -2.681080 -1.103662
H 7.959360 -2.578410 -0.488959
H 7.323786 -1.286619 -1.509554
SCF Energy (B3LYP/6-31G**)= -1361.69448599
Number of imaginary frequencies = 0

1a_c297
MMFF Geometry
C -1.749768 -2.548024 -0.102456
C -2.550181 0.037991 -0.340874
N -3.080229 -2.291610 -0.099814
C -0.749420 -1.577359 -0.217298
C -1.175806 -0.242908 -0.342358
C -3.467289 -1.002920 -0.210403
C -0.217051 0.778263 -0.471919
C 1.156717 0.493785 -0.466164
C 1.575244 -0.831813 -0.332780
C 0.630794 -1.864954 -0.220294
C 3.024839 -1.123767 -0.326313
C 4.029212 0.030575 -0.151867
C 3.441341 1.391179 -0.490693
C 2.117634 1.586027 -0.629289
O 1.007175 -3.182374 -0.107086
O 3.394192 -2.297727 -0.430847
O 5.066979 -0.260261 -1.100841
C 4.557393 0.000914 1.291422
C 6.065087 0.032425 1.386590
C 6.765954 1.347388 1.177091
O 6.700624 -1.001004 1.604906
O 4.398133 2.383951 -0.605582
C 4.006749 3.659465 -1.098798
C -4.930796 -0.768484 -0.208782
C -5.721330 -1.768087 -1.009953
C -5.463150 0.259245 0.486767
C -6.930515 0.603409 0.627609
C -7.325208 1.797024 -0.263528
C -6.631749 3.115530 0.057592
C -7.275372 0.840785 2.101262
H -1.497001 -3.601938 -0.006034
H -2.902761 1.058408 -0.455258
H -0.553566 1.809191 -0.578874
H 1.703673 2.562369 -0.856604

H 1.981514 -3.247012 -0.233144
H 5.313665 -1.198753 -0.968588
H 4.232842 -0.911709 1.809972
H 4.151134 0.818515 1.900254
H 7.765767 1.299030 1.618044
H 6.856427 1.555498 0.109337
H 6.214571 2.148617 1.675378
H 3.549418 3.574503 -2.089869
H 3.332169 4.158106 -0.395744
H 4.907083 4.273661 -1.193563
H -5.839230 -2.702653 -0.451536
H -6.716713 -1.402496 -1.276207
H -5.218901 -1.991749 -1.958384
H -4.796653 0.911437 1.050881
H -7.543708 -0.247718 0.310433
H -7.121591 1.544392 -1.312147
H -8.409336 1.950247 -0.190301
H -5.545399 3.035945 -0.043374
H -6.972027 3.891741 -0.635897
H -6.863780 3.455309 1.070974
H -7.097955 -0.066575 2.689973
H -8.331466 1.108319 2.213805
H -6.673387 1.641779 2.543382
SCF Energy (B3LYP/6-31G**)= -1361.70191951
Number of imaginary frequencies = 0

1a_c298
MMFF Geometry
C 1.717827 -1.712056 -0.098399
C 2.265980 0.946889 -0.007438
N 3.016488 -1.329482 -0.042225
C 0.628917 -0.837038 -0.114156
C 0.924359 0.537676 -0.069493
C 3.285035 -0.004476 0.006039
C -0.128549 1.471267 -0.097052
C -1.466057 1.055611 -0.149658
C -1.751733 -0.310754 -0.175153
C -0.715499 -1.255288 -0.177445
C -3.169522 -0.724580 -0.231160
C -4.235312 0.268645 0.292620
C -3.828592 1.703455 -0.056962
C -2.539462 2.048077 -0.241790
O -0.967927 -2.604763 -0.244138
O -3.425164 -1.854784 -0.652646
O -4.275441 0.217702 1.722214
C -5.609599 -0.088761 -0.310161
C -6.104004 -1.447002 0.147703
C -6.943086 -2.233229 -0.822503
O -5.875330 -1.860768 1.286657
O -4.893941 2.586653 -0.112951
C -4.621353 3.977455 -0.236870
C 4.723878 0.379982 0.086975
C 5.036088 1.648664 0.830300
C 5.652640 -0.432458 -0.464918
C 7.157927 -0.287783 -0.562800
C 7.840961 -0.694091 0.755498
C 9.358557 -0.767399 0.652631
C 7.614859 1.072437 -1.098947
H 1.569495 -2.789310 -0.131108
H 2.506030 2.004861 0.015440
H 0.102793 2.535528 -0.076245
H -2.238124 3.066025 -0.460180
H -1.929278 -2.735758 -0.406467
H -4.708226 -0.629609 1.966406
H -5.555398 -0.082008 -1.405420
H -6.385877 0.620238 0.001664
H -7.221478 -3.191822 -0.376122
H -7.850884 -1.672694 -1.058176
H -6.369318 -2.424051 -1.732759
H -4.007985 4.333475 0.597176
H -4.145355 4.197300 -1.197724
H -5.575945 4.510951 -0.205119
H 6.058896 1.671706 1.211229
H 4.892128 2.520515 0.183905
H 4.394070 1.757333 1.712131
H 5.297810 -1.349304 -0.942747
H 7.467604 -1.019484 -1.323743
H 7.578701 0.004229 1.558328
H 7.470680 -1.679428 1.067061
H 9.795562 0.218506 0.468531
H 9.779993 -1.146422 1.589302
H 9.666114 -1.440744 -0.153569
H 6.982772 1.404552 -1.929966
H 8.639835 1.008642 -1.480651
H 7.606848 1.849360 -0.329850
SCF Energy (B3LYP/6-31G**)= -1361.70626496
Number of imaginary frequencies = 0

1a_c299
MMFF Geometry
C -1.472468 -1.905192 -0.940435
C -2.254868 0.609338 -0.267373
N -2.801685 -1.651399 -0.867696
C -0.465114 -0.966563 -0.695765
C -0.882365 0.333233 -0.350832
C -3.179114 -0.401968 -0.522330
C 0.082178 1.328078 -0.106421
C 1.451605 1.043883 -0.185540
C 1.859265 -0.254469 -0.511465
C 0.912538 -1.251601 -0.783351
C 3.309042 -0.530905 -0.600893
C 4.294267 0.427931 0.097054
C 3.741214 1.840113 0.120392
C 2.430079 2.111153 0.028728
O 1.283310 -2.523896 -1.145924
O 3.707387 -1.516305 -1.229415
O 5.502409 0.417233 -0.683348
C 4.635673 -0.009212 1.523409
C 4.783660 -1.505899 1.652545
C 6.139900 -2.103358 1.390534
O 3.809272 -2.208656 1.923764
O 4.641497 2.850600 0.422991
C 5.051050 3.545958 -0.755185
C -4.642332 -0.165084 -0.457161
C -5.407646 -0.775668 -1.598368
C -5.171346 0.522728 0.578142
C -6.617083 0.858119 0.889290
C -7.507442 -0.390248 1.037061
C -8.877112 -0.082812 1.629420
C -7.178237 1.905410 -0.075604
H -1.226477 -2.929343 -1.213299
H -2.600940 1.608079 -0.019499
H -0.244711 2.336037 0.147085
H 2.071809 3.131107 0.133674
H 2.261561 -2.559383 -1.233397
H 5.480488 -0.398946 -1.223356
H 3.847331 0.284484 2.229404
H 5.563528 0.458264 1.875294
H 6.130647 -3.162721 1.662136
H 6.390227 -2.010738 0.331957
H 6.890741 -1.597034 2.002146
H 5.505859 2.869745 -1.485670
H 4.206507 4.076729 -1.207453
H 5.801096 4.286986 -0.463665
H -5.483971 -1.861318 -1.475372
H -6.419471 -0.379737 -1.693710
H -4.909446 -0.569559 -2.552940
H -4.492442 0.877664 1.355290
H -6.593100 1.339870 1.878053
H -7.656617 -0.886133 0.072820
H -7.007466 -1.117036 1.690077
H -9.472289 0.543503 0.958521
H -9.431702 -1.012501 1.793122
H -8.784093 0.428904 2.592447
H -6.470948 2.730673 -0.216256
H -8.104343 2.338994 0.315933
H -7.403063 1.483606 -1.059132
SCF Energy (B3LYP/6-31G**)= -1361.69342366
Number of imaginary frequencies = 0

1a_c300
MMFF Geometry

C -1.580968 -2.526570 -0.052054
C -2.374680 0.048530 -0.399773
N -2.911015 -2.270336 -0.082149
C -0.577307 -1.561549 -0.186264
C -1.000906 -0.233197 -0.367911
C -3.294990 -0.986288 -0.246735
C -0.039741 0.782162 -0.514229
C 1.333201 0.496740 -0.480688
C 1.753679 -0.823763 -0.297911
C 0.803481 -1.849031 -0.151912
C 3.206924 -1.121119 -0.249525
C 4.233455 0.012830 -0.436215
C 3.610618 1.374606 -0.637927
C 2.289588 1.589425 -0.624468
O 1.171031 -3.161500 0.031928
O 3.567746 -2.292539 -0.091665
O 4.890905 -0.358734 -1.669346
C 5.282574 -0.037889 0.684264
C 4.839560 0.567314 2.000014
C 3.767690 -0.141999 2.784629
O 5.347947 1.602971 2.430206
O 4.446435 2.481071 -0.699963
C 5.247281 2.513278 -1.879973
C -4.758087 -0.751352 -0.277761
C -5.536813 -1.779915 -1.053285
C -5.300376 0.302441 0.369429
C -6.769255 0.653234 0.472981
C -7.147469 1.812217 -0.469537
C -6.457250 3.141391 -0.187840
C -7.137717 0.947206 1.930629
H -1.331547 -3.576216 0.088461
H -2.723356 1.064773 -0.556598
H -0.371296 1.810931 -0.655340
H 1.909460 2.603221 -0.720990
H 2.151977 -3.225074 0.007597
H 5.216952 -1.271890 -1.552662
H 6.191240 0.490942 0.366296
H 5.616134 -1.064483 0.881169
H 3.831530 0.155844 3.835278
H 2.783134 0.126825 2.398955
H 3.916526 -1.223260 2.731150
H 6.117931 1.860237 -1.772862
H 4.667592 2.248029 -2.770891
H 5.613274 3.536579 -2.006545
H -5.664624 -2.692769 -0.462095
H -6.527440 -1.424156 -1.348966
H -5.019553 -2.039339 -1.984478
H -4.642249 0.975600 0.918724
H -7.378543 -0.208829 0.178629
H -6.927189 1.519406 -1.504245
H -8.232403 1.969174 -0.419965
H -5.369528 3.056968 -0.267955
H -6.784982 3.890802 -0.915987
H -6.705196 3.519946 0.807875
H -6.971370 0.062887 2.556464
H -8.195079 1.219876 2.015561
H -6.541746 1.763961 2.351552
SCF Energy (B3LYP/6-31G**)= -1361.69636165
Number of imaginary frequencies = 0

1a_c301
MMFF Geometry
C 1.819052 1.990800 0.079913
C 2.158406 -0.702655 -0.015501
N 3.072559 1.498840 0.228371
C 0.676494 1.212522 -0.119638
C 0.863938 -0.180899 -0.169817
C 3.238328 0.156830 0.182195
C -0.246197 -1.019873 -0.381776
C -1.536813 -0.493275 -0.525030
C -1.717164 0.890736 -0.456693
C -0.619714 1.743666 -0.273621
C -3.090991 1.427025 -0.586242
C -4.270064 0.471261 -0.291696
C -3.933752 -0.946687 -0.740671
C -2.663225 -1.384370 -0.814231
O -0.766053 3.109516 -0.235145
O -3.224745 2.619617 -0.866727
O -5.417507 0.946481 -1.004721
C -4.570110 0.587697 1.207267
C -5.526822 -0.469619 1.710100
C -6.992074 -0.298864 1.405405
O -5.112966 -1.446025 2.339533
O -5.051090 -1.704609 -1.039498
C -4.891400 -3.107705 -1.218135
C 4.630497 -0.348159 0.368564
C 4.768996 -1.710614 0.988699
C 5.667836 0.440864 0.009118
C 7.162645 0.196167 0.059038
C 7.599875 -1.079750 -0.685828
C 9.105307 -1.155305 -0.909754
C 7.699237 0.286786 1.489443
H 1.755421 3.075781 0.128846
H 2.315204 -1.775239 -0.064711
H -0.095908 -2.097753 -0.435751
H -2.416761 -2.397520 -1.112737
H -1.687953 3.335935 -0.497394
H -5.286679 0.714714 -1.940847
H -5.000459 1.568068 1.448099
H -3.652253 0.501693 1.803679
H -7.575167 -0.975931 2.036251
H -7.300042 0.725757 1.628283
H -7.189813 -0.532513 0.357800
H -4.304777 -3.320949 -2.117189
H -4.437623 -3.567077 -0.333945
H -5.885380 -3.544147 -1.353315
H 4.590478 -2.493505 0.244285
H 5.755017 -1.881067 1.423617
H 4.057323 -1.841043 1.812145
H 5.430085 1.424592 -0.404032
H 7.608811 1.039684 -0.488409
H 7.292161 -1.979936 -0.145036
H 7.106248 -1.115926 -1.665377
H 9.647312 -1.243074 0.036321
H 9.350326 -2.034512 -1.514457
H 9.469985 -0.269411 -1.439033
H 7.305307 1.171507 2.002522
H 8.790718 0.372588 1.492590
H 7.435680 -0.591918 2.085010
SCF Energy (B3LYP/6-31G**)= -1361.69685292
Number of imaginary frequencies = 0

1a_c302
MMFF Geometry
C -1.440776 -2.206051 -0.586577
C -2.310996 0.304496 -0.009032
N -2.771392 -2.020657 -0.408876
C -0.474655 -1.198667 -0.495793
C -0.936721 0.097066 -0.195042
C -3.191273 -0.768816 -0.128407
C -0.014512 1.155265 -0.094551
C 1.358391 0.939897 -0.274874
C 1.811096 -0.352314 -0.557836
C 0.904666 -1.413909 -0.687602
C 3.263291 -0.556834 -0.744896
C 4.224058 0.482690 -0.120856
C 3.619165 1.884128 -0.177217
C 2.287320 2.074151 -0.231483
O 1.318973 -2.684404 -1.006656
O 3.641946 -1.553474 -1.362612
O 5.451016 0.477346 -0.862692
C 4.569262 0.131124 1.328026
C 4.810876 -1.348302 1.517797
C 6.142773 -1.909365 1.096725
O 3.921252 -2.068601 1.974495
O 4.565670 2.891276 -0.141814
C 4.126725 4.244682 -0.156522
C -4.651885 -0.609473 0.063504
C -5.263641 -1.631192 0.978550
C -5.306488 0.387615 -0.569750
C -6.778887 0.746946 -0.625142
C -7.102260 1.970721 0.254969

C -6.920607 1.759467 1.752452
C -7.785850 -0.390250 -0.436045
H -1.157331 -3.232042 -0.812422
H -2.692752 1.290200 0.238555
H -0.379365 2.158191 0.124254
H 1.843640 3.063620 -0.250525
H 2.285202 -2.666404 -1.192295
H 5.266813 0.898909 -1.720095
H 3.752948 0.406272 2.009050
H 5.462371 0.666687 1.672922
H 6.265539 -2.910330 1.520028
H 6.194635 -1.974077 0.008185
H 6.948616 -1.276168 1.476172
H 3.583828 4.469215 -1.080202
H 3.514138 4.466261 0.723255
H 5.012315 4.885978 -0.121274
H -6.045376 -1.193196 1.603613
H -5.680403 -2.464950 0.404968
H -4.526538 -2.038894 1.680579
H -4.715004 1.050081 -1.205538
H -6.937442 1.076802 -1.663420
H -6.470469 2.813867 -0.053517
H -8.138108 2.281527 0.069214
H -5.886308 1.504643 2.001040
H -7.171506 2.680056 2.289822
H -7.574794 0.969075 2.130576
H -7.519752 -1.258217 -1.048831
H -8.783063 -0.062610 -0.752251
H -7.877380 -0.715509 0.602110
SCF Energy (B3LYP/6-31G**)= -1361.69138115
Number of imaginary frequencies = 0

1a_c303
MMFF Geometry
C 2.104192 1.525569 -0.078404
C 2.299222 -1.175731 0.107399
N 3.340127 0.979011 0.019943
C 0.910052 0.800528 -0.089983
C 1.023661 -0.596321 0.005892
C 3.435057 -0.367402 0.112321
C -0.142415 -1.382044 -0.003997
C -1.415290 -0.799581 -0.103704
C -1.527383 0.590506 -0.195928
C -0.369075 1.387331 -0.192712
C -2.874264 1.207606 -0.293440
C -4.134851 0.318660 -0.232828
C -3.829603 -1.163023 -0.202450
C -2.591473 -1.662939 -0.110819
O -0.428877 2.758449 -0.285599
O -2.945670 2.435692 -0.414876
O -4.812081 0.612155 -1.459795
C -4.930013 0.763959 1.007662
C -6.400619 0.429180 0.930713
C -6.953892 -0.522102 1.957767
O -7.129309 0.968947 0.096492
O -4.899895 -2.045834 -0.131922
C -5.546778 -2.210102 -1.396408
C 4.811533 -0.929995 0.232960
C 4.946233 -2.268585 0.907827
C 5.863710 -0.217074 -0.227142
C 7.324272 -0.610170 -0.223549
C 8.099495 0.074012 0.918972
C 8.157454 1.595528 0.855569
C 7.946761 -0.330271 -1.595270
H 2.099931 2.611115 -0.150432
H 2.394431 -2.254856 0.167946
H -0.052104 -2.465871 0.066247
H -2.446764 -2.737718 -0.042424
H -1.369311 3.026095 -0.400959
H -5.307532 1.444785 -1.332733
H -4.890310 1.853483 1.139319
H -4.499430 0.341033 1.923845
H -7.962039 -0.829794 1.666866
H -6.327089 -1.413624 2.025706
H -6.994351 -0.022139 2.928500
H -6.274102 -1.410872 -1.561991
H -4.828212 -2.258560 -2.222109
H -6.094877 -3.156621 -1.367748
H 4.671374 -3.075812 0.221026
H 5.960651 -2.463476 1.266156
H 4.304171 -2.326618 1.794276
H 5.667431 0.760305 -0.672670
H 7.416070 -1.691532 -0.070411
H 7.654005 -0.216417 1.879191
H 9.127200 -0.310551 0.929402
H 7.158231 2.039660 0.886363
H 8.717164 1.981205 1.714172
H 8.664808 1.941030 -0.049672
H 7.452132 -0.924742 -2.372013
H 9.009269 -0.596064 -1.600071
H 7.859098 0.722957 -1.882472
SCF Energy (B3LYP/6-31G**)= -1361.70256879
Number of imaginary frequencies = 0

1a_c304
MMFF Geometry
C -1.781899 -2.440928 0.096071
C -2.394204 0.203210 -0.066036
N -3.083122 -2.082698 0.213214
C -0.722226 -1.549327 -0.101221
C -1.051256 -0.182995 -0.182399
C -3.375259 -0.767553 0.123878
C -0.029855 0.763691 -0.382818
C 1.311759 0.372528 -0.491264
C 1.632655 -0.983527 -0.398154
C 0.625719 -1.943484 -0.219681
C 3.053803 -1.376881 -0.496443
C 4.141863 -0.306995 -0.286120
C 3.654251 1.077094 -0.686761
C 2.343975 1.377274 -0.755841
O 0.910843 -3.286135 -0.149635
O 3.340405 -2.562457 -0.691945
O 5.236066 -0.698665 -1.131931
C 4.586715 -0.409357 1.177177
C 5.495311 0.714141 1.624811
C 6.923171 0.702948 1.143896
O 5.078239 1.599030 2.375376
O 4.681240 1.968901 -0.938379
C 4.362518 3.326977 -1.213372
C -4.808639 -0.417539 0.259210
C -5.491619 -1.072891 1.425121
C -5.381293 0.419381 -0.632601
C -6.813584 0.892333 -0.791274
C -6.995668 2.340768 -0.295621
C -6.796419 2.544672 1.200641
C -7.919408 -0.039496 -0.289488
H -1.604570 -3.511851 0.169646
H -2.673449 1.251571 -0.110536
H -0.290518 1.819234 -0.456231
H 1.995600 2.369207 -1.022081
H 1.857310 -3.422335 -0.379916
H 5.217305 -1.675105 -1.183648
H 5.115528 -1.352817 1.363577
H 3.716919 -0.406972 1.847723
H 7.498145 1.451892 1.696137
H 7.366949 -0.277947 1.331862
H 6.970279 0.939479 0.079832
H 3.785885 3.412880 -2.139790
H 3.827467 3.781388 -0.373306
H 5.301392 3.871803 -1.349272
H -6.211388 -0.400775 1.898244
H -6.000977 -1.989196 1.110833
H -4.779066 -1.336500 2.215793
H -4.745987 0.813967 -1.428701
H -6.966633 0.929142 -1.880856
H -6.294365 2.995426 -0.829300
H -8.001519 2.687300 -0.564446
H -5.785132 2.269482 1.513811
H -6.944226 3.599758 1.453914
H -7.513162 1.962245 1.786062
H -7.753406 -1.067203 -0.629993
H -8.888573 0.282394 -0.688078

H -8.014760 -0.047223 0.798071
SCF Energy (B3LYP/6-31G**)= -1361.69999061
Number of imaginary frequencies = 0

1a_c305
MMFF Geometry
C -1.740381 -1.905042 -0.531253
C -2.452871 0.704318 -0.257564
N -3.056169 -1.594478 -0.622891
C -0.713906 -0.981225 -0.305813
C -1.095323 0.366243 -0.166200
C -3.395772 -0.295920 -0.482626
C -0.109910 1.342163 0.065739
C 1.246558 0.998289 0.149327
C 1.624470 -0.338331 0.002076
C 0.650083 -1.326878 -0.211360
C 3.060940 -0.689271 0.109086
C 4.126019 0.425286 -0.058633
C 3.548843 1.786055 0.309011
C 2.237464 2.032101 0.427749
O 0.980570 -2.656123 -0.333982
O 3.349256 -1.876051 0.287846
O 4.493636 0.509042 -1.437858
C 5.338876 0.094096 0.836289
C 6.077799 -1.147884 0.378110
C 6.678774 -2.021899 1.445021
O 6.225887 -1.396601 -0.820472
O 4.438972 2.812799 0.604690
C 5.031373 3.348190 -0.581232
C -4.839928 0.012681 -0.581805
C -5.522839 -0.538212 -1.800123
C -5.438031 0.678651 0.429552
C -6.870970 1.134279 0.627522
C -7.589901 0.281122 1.693141
C -7.804693 -1.179142 1.315473
C -7.729136 1.328107 -0.624194
H -1.521482 -2.964201 -0.649704
H -2.774844 1.737047 -0.163413
H -0.408105 2.383836 0.180618
H 1.892025 3.021493 0.713104
H 1.942114 -2.758367 -0.152260
H 5.036442 -0.285958 -1.637793
H 5.013318 -0.049399 1.873817
H 6.086892 0.895331 0.824051
H 7.420731 -1.451803 2.009226
H 5.890866 -2.381185 2.111588
H 7.167315 -2.884107 0.982882
H 5.804968 2.677137 -0.965643
H 4.279437 3.553146 -1.351257
H 5.511725 4.294437 -0.315054
H -6.386624 -1.149218 -1.521319
H -5.845933 0.273753 -2.458922
H -4.868416 -1.181149 -2.398973
H -4.815161 0.944085 1.287338
H -6.781147 2.142478 1.059498
H -7.018474 0.315420 2.629886
H -8.565554 0.731813 1.914719
H -6.855780 -1.693246 1.137081
H -8.315033 -1.701898 2.131222
H -8.426941 -1.277031 0.421596
H -7.216356 1.960492 -1.356808
H -8.667320 1.831259 -0.363148
H -7.997273 0.385987 -1.107629
SCF Energy (B3LYP/6-31G**)= -1361.69837852
Number of imaginary frequencies = 0

1a_c306
MMFF Geometry
C -1.603694 -1.586261 -0.654385
C -2.098661 0.996232 0.027450
N -2.892416 -1.209768 -0.470976
C -0.499553 -0.741196 -0.519100
C -0.767298 0.593926 -0.164997
C -3.134738 0.076967 -0.131520
C 0.302046 1.497497 -0.018081
C 1.629422 1.088569 -0.205415
C 1.885803 -0.243246 -0.542793
C 0.833728 -1.152129 -0.717927
C 3.289772 -0.648940 -0.744865
C 4.403910 0.212509 -0.119569
C 4.007739 1.683407 -0.067930
C 2.717059 2.067304 -0.099515
O 1.056670 -2.456082 -1.088246
O 3.545377 -1.662595 -1.401256
O 5.558674 0.075672 -0.967973
C 4.788305 -0.255707 1.286019
C 4.806292 -1.760042 1.410605
C 6.069250 -2.483562 1.027663
O 3.798539 -2.367331 1.775287
O 5.086852 2.539187 0.062461
C 4.855064 3.941745 0.036324
C -4.565298 0.459481 0.047027
C -4.925114 1.879791 -0.288433
C -5.449306 -0.478289 0.454913
C -6.932535 -0.376022 0.748108
C -7.758442 -0.414403 -0.550328
C -9.258238 -0.523379 -0.309930
C -7.300469 0.784726 1.677473
H -1.476845 -2.632519 -0.924501
H -2.316464 2.020030 0.313492
H 0.089616 2.533517 0.243539
H 2.424946 3.109728 -0.036664
H 2.015514 -2.578631 -1.266107
H 5.383249 -0.675240 -1.569755
H 4.073346 0.108793 2.035746
H 5.774644 0.123015 1.580985
H 5.995730 -3.531365 1.332344
H 6.212785 -2.437236 -0.053572
H 6.922819 -2.034853 1.541749
H 4.380567 4.243629 -0.902979
H 4.255405 4.253873 0.897247
H 5.824485 4.444527 0.101586
H -4.376895 2.227388 -1.171752
H -5.981462 1.998282 -0.537134
H -4.694453 2.545074 0.549941
H -5.066885 -1.487651 0.627962
H -7.177278 -1.288742 1.311457
H -7.566025 0.477227 -1.157727
H -7.444773 -1.275769 -1.154257
H -9.651552 0.372628 0.179304
H -9.783569 -0.637161 -1.263712
H -9.494795 -1.392202 0.312268
H -6.577830 0.883358 2.495087
H -8.281182 0.612865 2.134646
H -7.355362 1.741120 1.150707
SCF Energy (B3LYP/6-31G**)= -1361.69849830
Number of imaginary frequencies = 0

1a_c307
MMFF Geometry
C 1.919866 1.956128 -0.122492
C 2.290968 -0.711381 0.230715
N 3.180780 1.509412 0.091257
C 0.784590 1.143751 -0.175427
C 0.988987 -0.236153 0.006424
C 3.361871 0.180398 0.268785
C -0.112544 -1.109949 -0.047339
C -1.410402 -0.628480 -0.263667
C -1.609436 0.745560 -0.427929
C -0.520134 1.628184 -0.400789
C -2.989402 1.240528 -0.638267
C -4.179481 0.326212 -0.282647
C -3.794101 -1.131456 -0.419465
C -2.526470 -1.566290 -0.365324
O -0.679539 2.980436 -0.587555
O -3.162124 2.392445 -1.049902
O -5.210990 0.646529 -1.229231
C -4.642870 0.730117 1.120624
C -5.665484 -0.198773 1.736904
C -7.053918 -0.214481 1.151723
O -5.376089 -0.888801 2.716570
O -4.836839 -2.041928 -0.489571

C -5.058405 -2.478776 -1.830889
C 4.761069 -0.272553 0.519569
C 4.923169 -1.501441 1.369929
C 5.784801 0.446529 0.007426
C 7.280596 0.220697 0.071542
C 7.777692 -1.172003 -0.362602
C 7.328239 -1.564359 -1.764472
C 7.844585 0.613980 1.439273
H 1.843853 3.033221 -0.255161
H 2.460688 -1.775429 0.359880
H 0.048739 -2.180033 0.081303
H -2.310406 -2.630391 -0.398353
H -1.608545 3.157425 -0.856973
H -5.126509 1.602675 -1.420184
H -5.082134 1.735978 1.114247
H -3.788812 0.771459 1.810023
H -7.719831 -0.777257 1.812347
H -7.431114 0.808331 1.073846
H -7.052477 -0.690500 0.170254
H -5.257562 -1.636312 -2.500715
H -4.205347 -3.059288 -2.197672
H -5.937660 -3.129556 -1.830654
H 4.762910 -2.406459 0.774850
H 5.910452 -1.570490 1.829692
H 4.211124 -1.499643 2.203330
H 5.531668 1.340611 -0.568133
H 7.715046 0.929931 -0.648773
H 8.875327 -1.172319 -0.346552
H 7.467819 -1.947928 0.343668
H 7.632891 -0.812342 -2.499232
H 7.782823 -2.518497 -2.050107
H 6.242388 -1.684602 -1.821326
H 7.528647 1.625278 1.719059
H 8.939967 0.604249 1.419887
H 7.522526 -0.069372 2.230516
SCF Energy (B3LYP/6-31G**)= -1361.69680684
Number of imaginary frequencies = 0

1a_c308
MMFF Geometry
C 1.829044 -1.650608 0.140890
C 2.365686 0.990987 -0.200158
N 3.127023 -1.262615 0.115894
C 0.735654 -0.790560 0.003198
C 1.025908 0.574066 -0.175010
C 3.386380 0.053993 -0.050849
C -0.031069 1.488757 -0.330214
C -1.367672 1.067014 -0.296296
C -1.653735 -0.287090 -0.108387
C -0.608993 -1.215443 0.027341
C -3.071294 -0.720921 -0.090698
C -4.181277 0.332834 0.158962
C -3.716985 1.719943 -0.265790
C -2.436738 2.039127 -0.496979
O -0.849791 -2.560199 0.184563
O -3.304354 -1.924278 -0.236041
O -4.435568 0.411870 1.563778
C -5.443936 -0.077820 -0.627591
C -6.068424 -1.354401 -0.099581
C -6.705138 -2.274263 -1.105472
O -6.100616 -1.596599 1.109010
O -4.686821 2.690579 -0.492160
C -5.208251 3.204504 0.735835
C 4.820511 0.453652 -0.058012
C 5.147406 1.751057 0.623614
C 5.722036 -0.379398 -0.622487
C 7.215953 -0.260957 -0.846208
C 8.019068 -1.116929 0.153066
C 7.921454 -0.680162 1.608810
C 7.789133 1.149919 -1.002640
H 1.684453 -2.719719 0.281259
H 2.606979 2.038606 -0.350249
H 0.194880 2.544438 -0.477387
H -2.174569 3.043132 -0.817724
H -1.815797 -2.719469 0.087171
H -4.912595 -0.410433 1.814614
H -5.199114 -0.214921 -1.687926
H -6.233447 0.679439 -0.557754
H -7.102226 -3.157359 -0.597426
H -7.523723 -1.754465 -1.609023
H -5.956490 -2.595909 -1.833666
H -5.906826 2.494642 1.188185
H -4.407825 3.460871 1.438648
H -5.763227 4.118624 0.504838
H 4.425342 1.984605 1.414702
H 6.115212 1.712131 1.127943
H 5.148410 2.577090 -0.094360
H 5.334832 -1.317953 -1.030410
H 7.377225 -0.725887 -1.831083
H 7.685428 -2.160491 0.083238
H 9.075927 -1.114063 -0.142144
H 6.891224 -0.722386 1.973918
H 8.521250 -1.347132 2.236991
H 8.300789 0.335405 1.751516
H 7.177932 1.750733 -1.684693
H 8.797448 1.097666 -1.429723
H 7.878217 1.686322 -0.055980
SCF Energy (B3LYP/6-31G**)= -1361.69861107
Number of imaginary frequencies = 0

1a_c309
MMFF Geometry
C 2.079370 1.477601 -0.099942
C 2.224176 -1.225465 0.099986
N 3.301988 0.909869 0.032828
C 0.874317 0.773126 -0.145206
C 0.959872 -0.625999 -0.043900
C 3.375406 -0.438114 0.132543
C -0.221521 -1.391353 -0.094066
C -1.479252 -0.786053 -0.236631
C -1.557885 0.606982 -0.321073
C -0.388687 1.383940 -0.287207
C -2.889577 1.240413 -0.458698
C -4.165207 0.404186 -0.230763
C -3.904820 -1.078973 -0.367472
C -2.677857 -1.618518 -0.326716
O -0.424098 2.754958 -0.389240
O -2.955613 2.445835 -0.728590
O -5.059457 0.840955 -1.265508
C -4.714074 0.803450 1.143639
C -6.050221 0.196257 1.518544
C -7.234388 0.436916 0.618689
O -6.178476 -0.429025 2.574447
O -5.013508 -1.911172 -0.377114
C -5.434557 -2.222807 -1.705581
C 4.744943 -1.008563 0.328139
C 4.826585 -2.367633 0.969849
C 5.822976 -0.278000 -0.040210
C 7.293596 -0.579816 0.165933
C 8.007396 0.564363 0.919050
C 8.157496 1.878478 0.159744
C 7.960220 -0.902793 -1.171213
H 2.096754 2.562052 -0.169947
H 2.294556 -2.305202 0.176482
H -0.153936 -2.476825 -0.023039
H -2.558051 -2.698116 -0.351694
H -1.345861 3.036677 -0.577817
H -4.989423 1.815935 -1.302687
H -4.844063 1.891355 1.208205
H -3.993877 0.529012 1.925939
H -8.157492 0.303193 1.189016
H -7.217218 1.464047 0.244682
H -7.228345 -0.270354 -0.212205
H -5.795940 -1.334276 -2.230375
H -4.631514 -2.698095 -2.277524
H -6.265696 -2.929620 -1.631440
H 4.439658 -3.138633 0.293542
H 5.843872 -2.666415 1.228027
H 4.247848 -2.392279 1.899565
H 5.638329 0.668113 -0.550817
H 7.411636 -1.459137 0.805363
H 7.473021 0.759342 1.855934

H 9.011060 0.226489 1.206433
H 7.207164 2.250344 -0.222437
H 8.555376 2.639190 0.833111
H 8.848180 1.772892 -0.682763
H 7.484753 -1.772113 -1.634127
H 9.019163 -1.140816 -1.029668
H 7.895518 -0.067992 -1.876157
SCF Energy (B3LYP/6-31G**)= -1361.70079230
Number of imaginary frequencies = 0

1a_c310
MMFF Geometry
C -1.936798 -1.639240 -0.168200
C -2.233963 1.049236 0.070814
N -3.190071 -1.144334 -0.026920
C -0.773716 -0.866152 -0.198771
C -0.939496 0.524005 -0.075389
C -3.335863 0.195310 0.092312
C 0.193735 1.357424 -0.106271
C 1.485723 0.828419 -0.246108
C 1.644645 -0.554155 -0.360093
C 0.523186 -1.398894 -0.348159
C 3.009188 -1.104838 -0.505937
C 4.224405 -0.203043 -0.220120
C 3.898033 1.280304 -0.290071
C 2.632665 1.736551 -0.298948
O 0.641022 -2.762560 -0.476519
O 3.139518 -2.290712 -0.825657
O 5.146813 -0.511148 -1.276673
C 4.794934 -0.578802 1.156925
C 6.282745 -0.842450 1.145977
C 7.216113 0.337003 1.101817
O 6.714259 -1.997149 1.147249
O 5.023604 2.084592 -0.311333
C 4.867354 3.475990 -0.562224
C -4.729635 0.700024 0.259956
C -4.898404 2.012568 0.976669
C -5.762829 -0.039917 -0.200252
C -7.239790 0.289013 -0.156210
C -7.935057 -0.626657 0.867092
C -9.405686 -0.293471 1.077800
C -7.829301 0.143933 -1.562897
H -1.891212 -2.722425 -0.259600
H -2.371783 2.122354 0.152467
H 0.059968 2.435462 -0.020153
H 2.406987 2.796774 -0.334207
H 1.578223 -2.983142 -0.682358
H 5.212025 -1.487497 -1.316878
H 4.320939 -1.491761 1.542853
H 4.579895 0.181718 1.918215
H 8.204689 0.029794 1.455012
H 7.301693 0.706410 0.078192
H 6.851259 1.127581 1.762232
H 4.362848 3.649006 -1.518326
H 4.330931 3.961481 0.259076
H 5.865100 3.920786 -0.621202
H -4.682042 2.849184 0.304276
H -5.906593 2.153097 1.375088
H -4.229025 2.075176 1.842404
H -5.540038 -0.996720 -0.677837
H -7.392966 1.330372 0.144640
H -7.848644 -1.676834 0.560174
H -7.425064 -0.540411 1.835467
H -9.995473 -0.490665 0.177998
H -9.817575 -0.909548 1.883703
H -9.534873 0.757283 1.355611
H -7.265718 0.742977 -2.287461
H -8.866897 0.490959 -1.594503
H -7.811013 -0.898218 -1.901248
SCF Energy (B3LYP/6-31G**)= -1361.70396717
Number of imaginary frequencies = 0

1a_c311
MMFF Geometry
C 1.982659 1.749700 0.072624
C 2.233621 -0.939383 -0.216341
N 3.227233 1.216233 0.111832
C 0.806711 1.017060 -0.105550
C 0.948652 -0.374552 -0.254235
C 3.349127 -0.123661 -0.032134
C -0.197441 -1.168824 -0.442565
C -1.477460 -0.599814 -0.472571
C -1.613317 0.782156 -0.310569
C -0.480344 1.591361 -0.143493
C -2.974180 1.366353 -0.327592
C -4.192626 0.442857 -0.124518
C -3.888283 -0.959293 -0.607548
C -2.643278 -1.444811 -0.722128
O -0.578840 2.955556 -0.008733
O -3.105854 2.587849 -0.457296
O -5.246260 1.011530 -0.917713
C -4.573892 0.533951 1.356480
C -5.616810 -0.467926 1.800323
C -7.027490 -0.292743 1.300594
O -5.323014 -1.374289 2.582507
O -4.978060 -1.787899 -0.824281
C -5.280724 -1.898384 -2.215403
C 4.736727 -0.672164 -0.000509
C 4.978566 -1.924711 -0.795721
C 5.687280 -0.010606 0.696759
C 7.150116 -0.329687 0.922529
C 8.046213 -0.055722 -0.299517
C 7.980161 1.386544 -0.786941
C 7.387154 -1.734037 1.486970
H 1.955674 2.830441 0.194155
H 2.351873 -2.013400 -0.316898
H -0.085383 -2.245540 -0.568144
H -2.483394 -2.481118 -1.006009
H -1.508506 3.226230 -0.180261
H -5.122112 1.981938 -0.891030
H -4.959587 1.531934 1.601485
H -3.690170 0.381128 1.990573
H -7.693867 -0.961569 1.853040
H -7.353104 0.736166 1.473254
H -7.092409 -0.534599 0.238911
H -5.466444 -0.918244 -2.665850
H -4.475283 -2.413029 -2.749855
H -6.190950 -2.497026 -2.313995
H 6.031524 -2.076926 -1.037879
H 4.629132 -2.804644 -0.245714
H 4.458108 -1.884317 -1.759594
H 5.387063 0.907552 1.208296
H 7.480470 0.358070 1.715247
H 9.087382 -0.278986 -0.034184
H 7.790274 -0.721976 -1.130209
H 8.232851 2.084636 0.017425
H 8.693537 1.539269 -1.603238
H 6.984594 1.638341 -1.164810
H 6.713251 -1.941731 2.325414
H 8.413841 -1.822414 1.860103
H 7.250379 -2.517775 0.737417
SCF Energy (B3LYP/6-31G**)= -1361.69696964
Number of imaginary frequencies = 0

1a_c312
MMFF Geometry
C -1.925075 -1.637431 -0.590000
C -2.253553 1.039303 -0.272494
N -3.188018 -1.151723 -0.521851
C -0.767110 -0.860568 -0.506152
C -0.949028 0.523210 -0.340792
C -3.349317 0.182283 -0.365319
C 0.178896 1.359479 -0.250354
C 1.480521 0.839429 -0.313214
C 1.654856 -0.536884 -0.471722
C 0.540153 -1.383875 -0.578806
C 3.029367 -1.078210 -0.536272
C 4.214825 -0.186467 -0.122591
C 3.886827 1.297808 -0.155130
C 2.623375 1.751364 -0.240953
O 0.674542 -2.741011 -0.752220
O 3.189618 -2.249578 -0.893657

O 5.215555 -0.448008 -1.118697
C 4.682349 -0.618328 1.276662
C 6.168134 -0.878225 1.366556
C 7.095953 0.303953 1.442925
O 6.604412 -2.031028 1.351004
O 5.006522 2.104622 -0.057243
C 4.862190 3.505244 -0.259122
C -4.754544 0.680196 -0.312109
C -4.991300 2.099931 -0.751548
C -5.738244 -0.154585 0.090679
C -7.215182 0.142137 0.240177
C -7.670102 -0.240839 1.662250
C -9.113457 0.141688 1.960711
C -7.990295 -0.613268 -0.841333
H -1.866980 -2.716157 -0.719221
H -2.402795 2.105028 -0.133907
H 0.033333 2.432707 -0.130072
H 2.395426 2.811654 -0.248820
H 1.625659 -2.950905 -0.896085
H 5.289388 -1.421675 -1.193875
H 4.185500 -1.547599 1.587629
H 4.406852 0.109238 2.050576
H 8.056751 -0.016004 1.856323
H 7.256344 0.716846 0.445233
H 6.678284 1.064906 2.106841
H 4.430460 3.718438 -1.242355
H 4.262625 3.953573 0.539320
H 5.859130 3.954284 -0.223181
H -4.400606 2.337569 -1.643806
H -6.030909 2.292157 -1.028847
H -4.720226 2.799989 0.045516
H -5.467545 -1.178018 0.360119
H -7.408801 1.211561 0.110995
H -7.548206 -1.319359 1.825035
H -7.025971 0.261711 2.395649
H -9.815907 -0.430796 1.348207
H -9.346338 -0.065324 3.010284
H -9.283167 1.207856 1.780689
H -7.603045 -0.375995 -1.838919
H -9.049962 -0.339662 -0.833500
H -7.919788 -1.698018 -0.702667
SCF Energy (B3LYP/6-31G**)= -1361.70386750
Number of imaginary frequencies = 0

1a_c313
MMFF Geometry
C -1.735683 -1.716423 -0.535308
C -2.103014 0.917934 0.008141
N -3.005190 -1.267618 -0.381612
C -0.590315 -0.921729 -0.438270
C -0.793048 0.439612 -0.157023
C -3.183800 0.045366 -0.109682
C 0.320275 1.291403 -0.046531
C 1.628454 0.811103 -0.213912
C 1.829107 -0.545780 -0.488509
C 0.723962 -1.407612 -0.603530
C 3.212234 -1.056273 -0.674502
C 4.405821 -0.075897 -0.562992
C 4.013916 1.355674 -0.257764
C 2.745283 1.743419 -0.080505
O 0.871029 -2.746021 -0.886970
O 3.360633 -2.245259 -0.974627
O 5.044622 -0.087662 -1.857753
C 5.429954 -0.642108 0.429762
C 4.936329 -0.656837 1.860200
C 5.990419 -0.749883 2.931728
O 3.739725 -0.644295 2.146669
O 5.021748 2.274737 0.009960
C 5.788109 2.620315 -1.142648
C -4.594140 0.507800 0.040664
C -4.878396 1.929221 -0.357090
C -5.527898 -0.364869 0.481049
C -7.005412 -0.173961 0.758179
C -7.825044 -0.222730 -0.543884
C -9.329776 -0.244027 -0.310662
C -7.317501 1.041287 1.636931
H -1.660342 -2.780555 -0.748566
H -2.268144 1.964558 0.242060
H 0.160749 2.345788 0.177551
H 2.529694 2.769641 0.204087
H 1.832292 -2.945836 -0.960087
H 4.359543 0.107979 -2.520441
H 6.355864 -0.055028 0.386799
H 5.708818 -1.669016 0.162541
H 5.515948 -0.750056 3.916956
H 6.555482 -1.677067 2.809282
H 6.658697 0.112142 2.864389
H 6.554701 1.864413 -1.334635
H 5.153872 2.765037 -2.024080
H 6.298158 3.565290 -0.933034
H -5.926500 2.092156 -0.615214
H -4.615059 2.617099 0.452951
H -4.310941 2.209190 -1.252202
H -5.199041 -1.383969 0.700600
H -7.300551 -1.048962 1.356176
H -7.583252 0.632071 -1.185589
H -7.553278 -1.123209 -1.109878
H -9.678634 0.690425 0.138752
H -9.855078 -0.369468 -1.262997
H -9.614605 -1.073220 0.344514
H -6.595310 1.135545 2.455458
H -8.308404 0.939378 2.093073
H -7.319416 1.976964 1.071528
SCF Energy (B3LYP/6-31G**)= -1361.69454049
Number of imaginary frequencies = 0

1a_c314
MMFF Geometry
C -1.785851 -1.598099 -0.337932
C -2.048949 1.065885 0.114072
N -3.033567 -1.099003 -0.166434
C -0.611874 -0.841686 -0.299279
C -0.760823 0.535740 -0.065820
C -3.162408 0.228821 0.059332
C 0.383176 1.351871 -0.017672
C 1.668334 0.818843 -0.197128
C 1.814693 -0.551857 -0.427883
C 0.680113 -1.379382 -0.479352
C 3.176168 -1.115653 -0.604025
C 4.406341 -0.188517 -0.566202
C 4.069818 1.264569 -0.324399
C 2.822843 1.708355 -0.125463
O 0.775893 -2.733319 -0.700745
O 3.293834 -2.328085 -0.812580
O 4.927741 -0.303401 -1.909976
C 5.462851 -0.755837 0.393191
C 5.197846 -0.490836 1.860582
C 4.040059 -1.198447 2.513662
O 5.913014 0.271098 2.511759
O 5.112349 2.166038 -0.159278
C 5.857333 2.387307 -1.355439
C -4.550550 0.737826 0.258232
C -4.706356 1.993341 1.073545
C -5.590631 0.050056 -0.263365
C -7.063266 0.395245 -0.201771
C -7.777200 -0.586567 0.744451
C -9.244482 -0.250090 0.972546
C -7.645922 0.366908 -1.618484
H -1.754661 -2.671052 -0.514866
H -2.171854 2.130865 0.282092
H 0.266094 2.420343 0.163574
H 2.656232 2.758999 0.098037
H 1.720672 -2.968724 -0.837937
H 5.061375 -1.255484 -2.081669
H 6.447912 -0.333352 0.153234
H 5.588671 -1.838100 0.263454
H 4.203530 -1.237945 3.594567
H 3.112794 -0.659125 2.314412
H 3.971187 -2.225014 2.145482
H 6.579162 1.581325 -1.514573
H 5.202184 2.502204 -2.225902
H 6.420156 3.317199 -1.230330

H -4.473094 2.876277 0.469500
H -5.715481 2.117404 1.475024
H -4.042902 1.978836 1.945957
H -5.377708 -0.869759 -0.812564
H -7.204280 1.412448 0.177434
H -7.703060 -1.611158 0.358116
H -7.272140 -0.582103 1.719221
H -9.831258 -0.369322 0.057200
H -9.669666 -0.920468 1.726531
H -9.361202 0.777909 1.329681
H -7.069790 1.011920 -2.291911
H -8.678513 0.729791 -1.628667
H -7.639627 -0.646322 -2.035880
SCF Energy (B3LYP/6-31G**)= -1361.69839646
Number of imaginary frequencies = 0

1a_c315
MMFF Geometry
C 1.673419 -1.822073 -0.271444
C 2.296256 0.821573 -0.282011
N 2.982913 -1.474196 -0.287887
C 0.608729 -0.918031 -0.259586
C 0.942582 0.448418 -0.265544
C 3.289978 -0.156492 -0.292000
C -0.085319 1.409941 -0.261846
C -1.434381 1.030503 -0.236929
C -1.757171 -0.327606 -0.214980
C -0.747778 -1.300273 -0.245375
C -3.186417 -0.703261 -0.190026
C -4.197894 0.330794 0.361665
C -3.771149 1.745586 -0.041513
C -2.484313 2.050049 -0.298669
O -1.038884 -2.643450 -0.264776
O -3.492731 -1.835444 -0.570438
O -4.168141 0.313095 1.792291
C -5.609529 -0.001730 -0.164163
C -6.116685 -1.334990 0.349769
C -7.023642 -2.119224 -0.559033
O -5.842854 -1.729173 1.485701
O -4.813989 2.656692 -0.064789
C -4.510773 4.036505 -0.232620
C 4.742281 0.187668 -0.325623
C 5.105619 1.487497 -0.987596
C 5.634433 -0.681650 0.199998
C 7.142155 -0.598271 0.328269
C 7.625219 0.666972 1.062946
C 9.091876 0.598476 1.470838
C 7.834363 -0.829710 -1.016909
H 1.495332 -2.895294 -0.267500
H 2.563461 1.873100 -0.272692
H 0.174917 2.467589 -0.278255
H -2.167460 3.054179 -0.555643
H -2.010265 -2.751633 -0.376238
H -4.610631 -0.516153 2.077612
H -5.609710 -0.021203 -1.260611
H -6.350136 0.735468 0.168341
H -7.305037 -3.059363 -0.076734
H -7.926720 -1.539064 -0.763245
H -6.501105 -2.346464 -1.491506
H -3.846862 4.392391 0.561844
H -4.078030 4.223147 -1.220567
H -5.447904 4.596956 -0.165145
H 4.923755 2.329214 -0.311290
H 6.150582 1.528431 -1.298679
H 4.520024 1.639264 -1.901702
H 5.240283 -1.607248 0.627718
H 7.422311 -1.449744 0.966077
H 7.486132 1.560755 0.447078
H 7.024506 0.812212 1.969891
H 9.749995 0.573367 0.597464
H 9.358175 1.481543 2.060739
H 9.290080 -0.287786 2.081730
H 7.408923 -1.696926 -1.534691
H 8.901686 -1.030069 -0.877660
H 7.744941 0.034910 -1.680759
SCF Energy (B3LYP/6-31G**)= -1361.70663541
Number of imaginary frequencies = 0

1a_c316
MMFF Geometry
C 1.786795 -2.368424 0.102712
C 2.480957 0.187668 -0.503374
N 3.087602 -2.112109 -0.176893
C 0.766015 -1.412444 0.101050
C 1.136925 -0.093471 -0.216230
C 3.424351 -0.837484 -0.467037
C 0.154670 0.913303 -0.236656
C -1.189295 0.626811 0.046001
C -1.553441 -0.685368 0.355389
C -0.582856 -1.699514 0.394042
C -2.972000 -0.980002 0.651102
C -4.049388 0.067714 0.314633
C -3.490310 1.473524 0.163787
C -2.172276 1.711547 0.039282
O -0.903536 -2.998464 0.710193
O -3.261347 -2.078764 1.135393
O -4.923626 0.050927 1.453719
C -4.777112 -0.377414 -0.964146
C -6.282410 -0.388052 -0.833222
C -7.013519 0.925572 -0.897289
O -6.887945 -1.446496 -0.652753
O -4.470960 2.449203 0.138664
C -4.079672 3.815942 0.186408
C 4.857063 -0.598710 -0.769870
C 5.493524 -1.666078 -1.619940
C 5.489891 0.479817 -0.259535
C 6.937086 0.880554 -0.448829
C 7.792264 0.543013 0.788376
C 7.412006 1.270625 2.072207
C 7.022765 2.362617 -0.826810
H 1.576375 -3.409855 0.337390
H 2.787577 1.195431 -0.766310
H 0.449254 1.934803 -0.475929
H -1.781201 2.715740 -0.082902
H -1.844555 -3.032959 0.997787
H -5.137998 -0.888354 1.629535
H -4.483265 -1.397072 -1.248856
H -4.505694 0.236090 -1.832559
H -6.960193 1.429297 0.069671
H -6.582123 1.555941 -1.678835
H -8.062465 0.744219 -1.148740
H -3.481269 4.022727 1.079573
H -3.539584 4.097913 -0.722869
H -4.987783 4.423639 0.239865
H 4.906017 -1.829944 -2.530588
H 6.506658 -1.418049 -1.942252
H 5.555184 -2.612836 -1.072195
H 4.930019 1.148902 0.394385
H 7.372041 0.331659 -1.290188
H 7.743187 -0.537727 0.974096
H 8.842633 0.767640 0.563514
H 6.385116 1.046519 2.375107
H 8.072676 0.954934 2.886524
H 7.515827 2.354220 1.966066
H 6.510040 2.548479 -1.777576
H 8.066467 2.672410 -0.946376
H 6.560954 3.009289 -0.073115
SCF Energy (B3LYP/6-31G**)= -1361.70246323
Number of imaginary frequencies = 0

1a_c317
MMFF Geometry
C -1.690330 -2.064564 -0.261027
C -2.190957 0.519637 0.414450
N -2.967292 -1.722684 0.036928
C -0.603100 -1.184181 -0.250446
C -0.875274 0.150071 0.097584
C -3.202657 -0.436674 0.377894
C 0.175739 1.083932 0.117468
C 1.492799 0.712558 -0.198720
C 1.762742 -0.616566 -0.536999
C 0.719176 -1.559615 -0.566972

C 3.152912 -1.014509 -0.868602
C 4.279054 0.041413 -0.803332
C 3.825065 1.446557 -0.441703
C 2.541080 1.734973 -0.168468
O 0.936281 -2.874735 -0.909105
O 3.357044 -2.184091 -1.210255
O 4.841652 0.106448 -2.132665
C 5.403409 -0.483954 0.100283
C 5.014493 -0.602089 1.557466
C 6.133206 -0.894741 2.522998
O 3.852513 -0.498445 1.948295
O 4.861485 2.364167 -0.435726
C 4.579818 3.713550 -0.083477
C -4.607152 -0.090869 0.707126
C -4.821557 0.691874 1.968827
C -5.570764 -0.483547 -0.153783
C -7.076571 -0.311614 -0.153008
C -7.517856 0.667020 -1.262562
C -7.062954 2.108500 -1.068179
C -7.758005 0.005368 1.178794
H -1.556784 -3.112541 -0.521029
H -2.426721 1.547062 0.673578
H -0.041598 2.116629 0.388363
H 2.224551 2.738317 0.093837
H 1.895069 -2.991780 -1.101550
H 4.235553 0.634740 -2.678720
H 6.279558 0.173214 0.030242
H 5.736059 -1.474116 -0.236358
H 5.736478 -0.954339 3.540289
H 6.597004 -1.850417 2.266903
H 6.873330 -0.091883 2.480761
H 3.885096 4.167000 -0.797640
H 4.192336 3.778261 0.938331
H 5.518255 4.274353 -0.124085
H -5.256978 1.671298 1.747105
H -5.472762 0.148405 2.659318
H -3.891132 0.869811 2.518911
H -5.236335 -1.039042 -1.035746
H -7.470720 -1.297006 -0.443166
H -7.140351 0.312195 -2.230461
H -8.612420 0.653228 -1.339240
H -5.972408 2.186230 -1.031735
H -7.410536 2.723101 -1.905188
H -7.472780 2.539876 -0.150631
H -7.498005 -0.740086 1.937725
H -8.847554 -0.021802 1.062306
H -7.500856 0.996157 1.561350
SCF Energy (B3LYP/6-31G**)= -1361.69654790
Number of imaginary frequencies = 0

1a_c318
MMFF Geometry
C -1.556037 -1.740704 -0.499348
C -2.131917 0.802973 0.259381
N -2.850521 -1.424790 -0.252861
C -0.484598 -0.850770 -0.393661
C -0.794344 0.464407 -0.001678
C -3.133799 -0.157631 0.126738
C 0.239511 1.413059 0.111481
C 1.573577 1.067512 -0.143717
C 1.872868 -0.245922 -0.515966
C 0.854983 -1.198141 -0.660043
C 3.282615 -0.584665 -0.788838
C 4.385158 0.316481 -0.199933
C 3.926922 1.767048 -0.104948
C 2.620390 2.092677 -0.070777
O 1.117813 -2.484236 -1.065035
O 3.552345 -1.575520 -1.473771
O 5.504534 0.244737 -1.102068
C 4.854462 -0.155208 1.178444
C 4.943481 -1.658956 1.276618
C 6.217211 -2.320333 0.823438
O 3.981236 -2.315373 1.677319
O 4.971843 2.668639 -0.009510
C 4.677044 4.059553 -0.002569
C -4.567639 0.147662 0.407739
C -4.834489 1.219932 1.426943
C -5.526034 -0.562079 -0.229095
C -7.037129 -0.466541 -0.165217
C -7.575696 0.943006 -0.476809
C -9.078696 0.967122 -0.726983
C -7.582811 -1.058730 1.136220
H -1.396191 -2.775034 -0.796516
H -2.382817 1.817782 0.549924
H -0.006270 2.434274 0.400662
H 2.285466 3.119779 0.023157
H 2.072049 -2.560735 -1.287789
H 5.334569 -0.503432 -1.708808
H 4.159979 0.166136 1.966195
H 5.836062 0.262264 1.434333
H 6.203429 -3.374846 1.113153
H 6.307794 -2.251378 -0.262315
H 7.073719 -1.842321 1.305210
H 4.146641 4.352839 -0.914286
H 4.104615 4.332257 0.889708
H 5.625167 4.604572 0.026467
H -4.716532 2.213419 0.981963
H -5.837306 1.160954 1.852792
H -4.147924 1.132856 2.277002
H -5.196207 -1.336836 -0.926228
H -7.399278 -1.119419 -0.973223
H -7.356520 1.640368 0.337518
H -7.073917 1.334336 -1.370997
H -9.639212 0.699620 0.173457
H -9.392528 1.972568 -1.025807
H -9.356031 0.274961 -1.528190
H -7.119086 -2.027868 1.352778
H -8.662362 -1.227223 1.066385
H -7.406036 -0.402864 1.993474
SCF Energy (B3LYP/6-31G**)= -1361.69883475
Number of imaginary frequencies = 0

1a_c319
MMFF Geometry
C -1.768693 -1.519377 -0.829964
C -2.062498 1.097096 -0.168031
N -3.025276 -1.026393 -0.714777
C -0.600267 -0.779522 -0.631725
C -0.765050 0.572929 -0.288618
C -3.169419 0.278182 -0.386170
C 0.372777 1.370095 -0.071825
C 1.667240 0.843267 -0.193375
C 1.829272 -0.502192 -0.535439
C 0.701046 -1.310974 -0.753874
C 3.199478 -1.060588 -0.650706
C 4.424274 -0.151687 -0.431056
C 4.071946 1.276068 -0.083829
C 2.813768 1.711462 0.053627
O 0.811884 -2.640519 -1.087585
O 3.331240 -2.250518 -0.957310
O 5.049800 -0.147197 -1.734733
C 5.401025 -0.814093 0.551743
C 5.026043 -0.676974 2.012670
C 3.811580 -1.416691 2.508203
O 5.699655 0.007108 2.783634
O 5.099324 2.148562 0.247784
C 5.939495 2.471966 -0.858714
C -4.567958 0.787748 -0.284822
C -4.776207 2.258559 -0.526780
C -5.570707 -0.077451 -0.015007
C -7.045155 0.221444 0.153793
C -7.528690 -0.346590 1.502675
C -8.970618 0.016051 1.831338
C -7.814261 -0.363540 -1.032534
H -1.725279 -2.572816 -1.098085
H -2.196901 2.137692 0.108765
H 0.243368 2.418728 0.196151
H 2.631482 2.738595 0.359137
H 1.764254 -2.871571 -1.167959
H 5.196853 -1.080563 -1.981701
H 6.403588 -0.383461 0.425826
H 5.531084 -1.882070 0.336258

H 3.887754 -1.552755 3.590945
H 2.909531 -0.844915 2.285172
H 3.760355 -2.405997 2.046914
H 6.669493 1.676732 -1.033832
H 5.357339 2.673886 -1.764500
H 6.493010 3.380335 -0.602582
H -4.169681 2.606429 -1.370952
H -5.808678 2.505569 -0.786749
H -4.505128 2.837216 0.362350
H -5.320109 -1.132680 0.114659
H -7.220364 1.301627 0.171428
H -7.425987 -1.439218 1.516100
H -6.888793 0.038385 2.307449
H -9.671835 -0.453876 1.135781
H -9.223479 -0.330077 2.838784
H -9.121040 1.099663 1.798482
H -7.407473 0.002688 -1.982186
H -8.869615 -0.075307 -1.001466
H -7.762567 -1.458103 -1.044559
SCF Energy (B3LYP/6-31G**)= -1361.69850551
Number of imaginary frequencies = 0

1a_c320
MMFF Geometry
C -1.618318 -1.501563 -0.760298
C -2.131848 1.005717 0.149952
N -2.910817 -1.146557 -0.561170
C -0.518980 -0.670198 -0.532517
C -0.796650 0.626063 -0.060698
C -3.162403 0.102749 -0.107529
C 0.266739 1.514848 0.183966
C 1.597174 1.128035 -0.023466
C 1.865747 -0.167528 -0.478604
C 0.818610 -1.058593 -0.749640
C 3.275664 -0.552630 -0.702172
C 4.388289 0.262019 -0.012584
C 3.963491 1.707620 0.162234
C 2.677791 2.090860 0.194714
O 1.049803 -2.323381 -1.233797
O 3.540380 -1.511862 -1.433271
O 5.531568 0.218139 -0.884200
C 4.792035 -0.318128 1.344833
C 4.818326 -1.827425 1.346943
C 6.095223 -2.508787 0.934295
O 3.808581 -2.469180 1.639204
O 4.967695 2.613137 0.470118
C 5.351838 3.367664 -0.679867
C -4.596714 0.463727 0.085887
C -4.956431 1.908904 -0.117713
C -5.483418 -0.510246 0.389912
C -6.970927 -0.439069 0.670330
C -7.778473 -0.354250 -0.637420
C -9.281140 -0.488371 -0.429907
C -7.355384 0.626570 1.701367
H -1.484221 -2.518238 -1.123700
H -2.356748 1.998216 0.526606
H 0.048607 2.521620 0.538809
H 2.418366 3.124258 0.405506
H 2.012180 -2.430876 -1.401500
H 5.400162 -0.546458 -1.481173
H 4.084977 -0.021205 2.131010
H 5.779883 0.041988 1.657477
H 6.013509 -3.582791 1.123883
H 6.276009 -2.349286 -0.130459
H 6.929169 -2.117267 1.522037
H 5.693531 2.719328 -1.492646
H 4.526729 4.000443 -1.023824
H 6.182070 4.019068 -0.391656
H -4.397884 2.339881 -0.956701
H -6.009803 2.048738 -0.367904
H -4.738686 2.491826 0.783103
H -5.100243 -1.530753 0.471801
H -7.220756 -1.402015 1.140059
H -7.580360 0.591814 -1.153818
H -7.453729 -1.153224 -1.316575
H -9.684020 0.355934 0.137290
H -9.792734 -0.511121 -1.397571
H -9.523686 -1.413241 0.102757
H -6.644576 0.647544 2.534879
H -8.341879 0.409997 2.125945
H -7.405798 1.628987 1.268012
SCF Energy (B3LYP/6-31G**)= -1361.69350521
Number of imaginary frequencies = 0

1a_c321
MMFF Geometry
C -1.765386 -2.523446 0.031896
C -2.490919 0.084589 -0.207397
N -3.084844 -2.239871 -0.091460
C -0.741239 -1.571312 0.046252
C -1.128673 -0.224531 -0.079363
C -3.434818 -0.940494 -0.202521
C -0.144661 0.780985 -0.069092
C 1.216221 0.465323 0.052714
C 1.596262 -0.873905 0.164784
C 0.626047 -1.888116 0.177319
C 3.037149 -1.188900 0.292546
C 4.059798 -0.128979 -0.175564
C 3.527102 1.286503 0.027269
C 2.208286 1.540711 0.118591
O 0.965997 -3.212994 0.318072
O 3.345561 -2.304942 0.719884
O 4.181581 -0.352420 -1.587645
C 5.385826 -0.414842 0.559506
C 6.574936 0.319959 -0.022295
C 7.645134 0.756864 0.941129
O 6.689659 0.502680 -1.236097
O 4.516204 2.251737 0.078565
C 4.136864 3.623167 0.049170
C -4.886768 -0.675532 -0.341590
C -5.606778 -1.632814 -1.253094
C -5.468612 0.337578 0.335487
C -6.935833 0.714445 0.343860
C -7.115798 2.067467 -0.367814
C -8.572668 2.481751 -0.522984
C -7.438690 0.745434 1.790500
H -1.541807 -3.584444 0.121565
H -2.811518 1.115110 -0.324267
H -0.449500 1.823064 -0.157621
H 1.821015 2.545562 0.242986
H 1.933748 -3.268190 0.489440
H 5.039750 0.035880 -1.864238
H 5.653449 -1.477251 0.499350
H 5.286540 -0.160948 1.621871
H 8.436476 1.282246 0.399431
H 7.216306 1.436914 1.681345
H 8.072355 -0.119247 1.434852
H 3.557103 3.851314 -0.851048
H 3.582795 3.893214 0.953648
H 5.051086 4.223468 0.023264
H -5.801076 -2.580560 -0.740058
H -6.560666 -1.238366 -1.612482
H -5.010541 -1.840044 -2.149550
H -4.851758 0.956465 0.987557
H -7.530380 -0.037901 -0.183962
H -6.576996 2.857071 0.171420
H -6.669733 2.011783 -1.369430
H -9.038101 2.685144 0.445717
H -8.640257 3.396977 -1.120038
H -9.150880 1.703348 -1.030635
H -7.226022 -0.201811 2.299373
H -8.522005 0.896839 1.829428
H -6.964174 1.549529 2.364213
SCF Energy (B3LYP/6-31G**)= -1361.71079995
Number of imaginary frequencies = 0

1a_c322
MMFF Geometry
C -1.758367 -2.449372 0.118338
C -2.361983 0.193894 -0.089817
N -3.057258 -2.083625 0.238776
C -0.697188 -1.565871 -0.104660

C -1.021539 -0.199619 -0.209923
C -3.345026 -0.769268 0.126955
C 0.001313 0.739321 -0.438688
C 1.339842 0.340168 -0.549130
C 1.656636 -1.015721 -0.429506
C 0.647796 -1.968115 -0.226150
C 3.078873 -1.415554 -0.525917
C 4.152689 -0.336751 -0.254745
C 3.682259 1.024338 -0.755922
C 2.375469 1.329485 -0.857638
O 0.930375 -3.309903 -0.134773
O 3.334829 -2.597883 -0.761482
O 5.350440 -0.718656 -0.940667
C 4.444389 -0.371639 1.249956
C 5.286748 0.791155 1.723696
C 6.765050 0.755805 1.436738
O 4.771832 1.742405 2.315976
O 4.722096 1.880292 -1.069450
C 4.425412 3.253951 -1.295684
C -4.775862 -0.411046 0.267759
C -5.451038 -1.041118 1.452038
C -5.353159 0.410686 -0.635115
C -6.785050 0.885939 -0.790462
C -6.957518 2.344352 -0.321336
C -6.744469 2.576327 1.168923
C -7.889848 -0.031812 -0.261219
H -1.584184 -3.519340 0.211418
H -2.637845 1.242235 -0.151897
H -0.255921 1.794008 -0.532537
H 2.033051 2.302247 -1.193547
H 1.873525 -3.451447 -0.380568
H 5.209200 -0.531542 -1.885228
H 4.965799 -1.296087 1.528997
H 3.515239 -0.356030 1.834772
H 7.271734 1.506529 2.049984
H 7.169494 -0.225976 1.695388
H 6.950800 0.974553 0.383735
H 3.832384 3.378676 -2.207086
H 3.916350 3.693326 -0.431659
H 5.372527 3.783345 -1.435206
H -4.732581 -1.292125 2.241468
H -6.164171 -0.357215 1.918268
H -5.966488 -1.961362 1.159977
H -4.723366 0.787258 -1.444168
H -6.947475 0.902312 -1.879186
H -6.258520 2.985913 -0.873622
H -7.964408 2.689465 -0.588088
H -5.731495 2.303382 1.478582
H -6.886217 3.636659 1.403027
H -7.458189 2.008040 1.771676
H -7.730564 -1.066524 -0.583213
H -8.861269 0.286016 -0.657572
H -7.975754 -0.018180 0.827069
SCF Energy (B3LYP/6-31G**)= -1361.69397851
Number of imaginary frequencies = 0

1a_c323
MMFF Geometry
C -1.809177 2.450219 -0.010133
C -2.463725 -0.134944 -0.533311
N -3.122090 2.141867 -0.143618
C -0.757784 1.535025 -0.122734
C -1.108497 0.200325 -0.397102
C -3.436732 0.852663 -0.392858
C -0.094869 -0.765264 -0.537199
C 1.257893 -0.425595 -0.392048
C 1.599649 0.897538 -0.103811
C 0.602490 1.878166 0.014951
C 3.032562 1.238013 0.046370
C 4.024788 0.094973 0.358388
C 3.575245 -1.219712 -0.272554
C 2.289194 -1.444320 -0.601028
O 0.908932 3.195466 0.262912
O 3.352201 2.427307 -0.035263
O 3.954793 -0.057159 1.783401
C 5.423190 0.565276 -0.092870
C 6.553445 -0.293578 0.433727
C 7.754778 -0.461491 -0.456777
O 6.516840 -0.786768 1.563016
O 4.597858 -2.135629 -0.439779
C 4.269068 -3.467580 -0.818440
C -4.883252 0.559050 -0.532753
C -5.651855 1.590558 -1.314149
C -5.418141 -0.541285 0.038707
C -6.871986 -0.966951 0.036988
C -7.357201 -1.128138 1.490787
C -8.845712 -1.428608 1.603232
C -7.012819 -2.259528 -0.769961
H -1.614720 3.500122 0.198760
H -2.756881 -1.155133 -0.760692
H -0.370003 -1.795174 -0.761423
H 1.958322 -2.382643 -1.031439
H 1.888049 3.294964 0.243465
H 4.783685 -0.502652 2.063069
H 5.641359 1.575718 0.275051
H 5.471446 0.597118 -1.188131
H 8.488314 -1.108518 0.032064
H 7.450868 -0.925455 -1.398402
H 8.209494 0.514033 -0.645026
H 3.587729 -3.925232 -0.093942
H 3.845736 -3.492120 -1.827493
H 5.194109 -4.051573 -0.827749
H -5.085182 1.912635 -2.195761
H -6.604990 1.212774 -1.692380
H -5.856112 2.469777 -0.694073
H -4.767236 -1.206303 0.607237
H -7.497690 -0.205096 -0.437992
H -6.795424 -1.924777 1.995090
H -7.153921 -0.202257 2.044625
H -9.090700 -2.408778 1.184199
H -9.148052 -1.434797 2.655487
H -9.440436 -0.669845 1.085053
H -6.597188 -2.139778 -1.777121
H -8.063953 -2.541337 -0.886316
H -6.490299 -3.093352 -0.287668
SCF Energy (B3LYP/6-31G**)= -1361.71088943
Number of imaginary frequencies = 0

1a_c324
MMFF Geometry
C -1.632632 -2.282155 -0.521166
C -2.314596 0.139866 0.502815
N -2.932174 -2.070400 -0.201743
C -0.607034 -1.344347 -0.362771
C -0.972354 -0.095638 0.170105
C -3.262923 -0.860295 0.297296
C 0.014587 0.887244 0.360207
C 1.355616 0.648231 0.025398
C 1.717881 -0.592935 -0.505845
C 0.741737 -1.585495 -0.699060
C 3.139574 -0.847650 -0.847367
C 4.190863 0.262094 -0.652818
C 3.629070 1.543580 -0.082510
C 2.342665 1.698587 0.253036
O 1.053079 -2.822524 -1.213149
O 3.448401 -1.947570 -1.318652
O 4.616867 0.532133 -2.007999
C 5.401610 -0.289221 0.114667
C 5.216956 -0.383299 1.614939
C 4.236300 -1.393308 2.149554
O 5.859902 0.332107 2.383688
O 4.511138 2.564881 0.242379
C 5.114488 3.168987 -0.900381
C -4.694347 -0.668918 0.637621
C -5.336000 -1.860746 1.296904
C -5.321685 0.483480 0.317371
C -6.766415 0.854564 0.573927
C -7.623827 0.737599 -0.701661
C -7.239644 1.671080 -1.843265
C -6.843209 2.251088 1.198851
H -1.427688 -3.271256 -0.925390
H -2.615263 1.091076 0.931547

H -0.271374 1.853510 0.775851
H 2.015251 2.628993 0.709918
H 2.011865 -2.846317 -1.430081
H 4.901778 -0.320021 -2.390647
H 6.278885 0.345019 -0.071273
H 5.696200 -1.280915 -0.250937
H 4.484819 -1.623340 3.189737
H 3.223849 -0.988871 2.108094
H 4.301137 -2.320277 1.574525
H 5.948312 2.561188 -1.262704
H 4.384893 3.345953 -1.698193
H 5.519196 4.137251 -0.590879
H -4.748700 -2.178760 2.165953
H -6.347488 -1.665201 1.658140
H -5.403284 -2.701390 0.597641
H -4.758334 1.250811 -0.214316
H -7.204531 0.172932 1.309977
H -7.581105 -0.295976 -1.068811
H -8.672813 0.926642 -0.440539
H -6.214170 1.495960 -2.181212
H -7.902371 1.502512 -2.698622
H -7.337079 2.721334 -1.553965
H -6.329239 2.269334 2.166782
H -7.885029 2.542088 1.370641
H -6.377672 3.013949 0.565742
SCF Energy (B3LYP/6-31G**)= -1361.69693473
Number of imaginary frequencies = 0

1a_c325
MMFF Geometry
C -1.692877 -1.834685 -0.491797
C -2.134970 0.726482 0.291791
N -2.970202 -1.453428 -0.247223
C -0.575261 -1.004173 -0.374818
C -0.816821 0.319442 0.028762
C -3.186447 -0.177285 0.146208
C 0.266556 1.206376 0.158883
C 1.582918 0.797299 -0.105425
C 1.822751 -0.523235 -0.499684
C 0.747528 -1.418905 -0.637424
C 3.214253 -0.957846 -0.787848
C 4.372878 0.060173 -0.648504
C 3.940679 1.444575 -0.209452
C 2.667795 1.762527 0.054863
O 0.932515 -2.722120 -1.038308
O 3.394258 -2.111447 -1.191070
O 4.949700 0.179811 -1.966667
C 5.463940 -0.540169 0.248244
C 5.039442 -0.691335 1.692905
C 6.145859 -0.827964 2.705395
O 3.858125 -0.750996 2.032061
O 4.923692 2.381102 0.088326
C 5.620189 2.850908 -1.064681
C -4.602646 0.201075 0.428017
C -4.813704 1.275636 1.457923
C -5.596271 -0.449935 -0.217537
C -7.100182 -0.274488 -0.154697
C -7.562108 1.164728 -0.453435
C -9.061272 1.271593 -0.704949
C -7.679184 -0.849158 1.140184
H -1.587061 -2.873178 -0.798167
H -2.330502 1.749670 0.595185
H 0.077108 2.231563 0.475886
H 2.426286 2.752484 0.432031
H 1.896317 -2.874635 -1.169176
H 4.227137 0.399940 -2.580039
H 6.363283 0.087352 0.213316
H 5.769523 -1.529355 -0.114865
H 5.718943 -0.927644 3.707196
H 6.738537 -1.718226 2.481506
H 6.777713 0.063302 2.680453
H 6.405565 2.146336 -1.352211
H 4.939497 3.039068 -1.902165
H 6.102521 3.796788 -0.800609
H -4.640757 2.265829 1.023700
H -5.819446 1.266891 1.880839
H -4.135302 1.142505 2.308571
H -5.306704 -1.233966 -0.922075
H -7.495174 -0.899334 -0.969454
H -7.307188 1.841660 0.367699
H -7.038718 1.537066 -1.343335
H -9.636695 1.026032 0.192335
H -9.320395 2.295153 -0.994580
H -9.373998 0.602816 -1.512955
H -7.268103 -1.843613 1.348113
H -8.766028 -0.959306 1.067107
H -7.469526 -0.211817 2.003970
SCF Energy (B3LYP/6-31G**)= -1361.69473507
Number of imaginary frequencies = 0

1a_c326
MMFF Geometry
C -2.031815 -1.516200 -0.208325
C -2.261658 1.171010 0.106862
N -3.272030 -0.994500 -0.049520
C -0.849993 -0.771631 -0.220650
C -0.980941 0.618168 -0.057820
C -3.384456 0.344496 0.107579
C 0.172591 1.423782 -0.068581
C 1.450728 0.867082 -0.226988
C 1.575000 -0.515245 -0.380222
C 0.432929 -1.331937 -0.388728
C 2.925111 -1.095243 -0.545461
C 4.162994 -0.232227 -0.238008
C 3.873416 1.260131 -0.265289
C 2.619757 1.747652 -0.257641
O 0.516553 -2.693954 -0.555651
O 3.025221 -2.274516 -0.898724
O 5.075078 -0.533204 -1.305522
C 4.727119 -0.660634 1.126243
C 6.207895 -0.960624 1.103498
C 7.170162 0.196134 1.089776
O 6.610604 -2.125229 1.071015
O 5.018571 2.036570 -0.267233
C 4.896380 3.437929 -0.478448
C -4.765006 0.878247 0.293628
C -4.900407 2.179922 1.037004
C -5.819063 0.171436 -0.171507
C -7.284683 0.539911 -0.107908
C -8.016617 -0.211952 1.020544
C -8.053623 -1.729284 0.883711
C -7.941824 0.317333 -1.473948
H -2.013410 -2.597184 -0.330170
H -2.372254 2.244477 0.219267
H 0.065854 2.501953 0.048172
H 2.420413 2.813711 -0.262432
H 1.447521 -2.931813 -0.770346
H 5.115946 -1.509350 -1.373321
H 4.231471 -1.572087 1.487760
H 4.532745 0.083231 1.909237
H 8.151592 -0.145288 1.431290
H 7.262595 0.592008 0.076696
H 6.826532 0.976578 1.773228
H 4.394202 3.650261 -1.427829
H 4.374012 3.913223 0.357742
H 5.904735 3.859357 -0.527814
H -4.658810 3.024144 0.382958
H -5.906616 2.339728 1.433801
H -4.233101 2.205801 1.906163
H -5.621079 -0.779933 -0.669543
H -7.388225 1.610838 0.100963
H -7.548255 0.038365 1.981174
H -9.049237 0.154802 1.078532
H -7.047404 -2.158018 0.864768
H -8.582767 -2.165610 1.737436
H -8.581393 -2.038303 -0.023045
H -7.478549 0.957132 -2.233825
H -9.007901 0.565601 -1.436090
H -7.846613 -0.718998 -1.815141
SCF Energy (B3LYP/6-31G**)= -1361.70311996
Number of imaginary frequencies = 0

1a_c327
MMFF Geometry
C 1.703141 2.402255 0.022124
C 2.348815 -0.238228 0.020549
N 3.009487 2.067710 0.152115
C 0.653675 1.487728 -0.112679
C 1.000151 0.123282 -0.111054
C 3.319955 0.753518 0.142097
C -0.009686 -0.846874 -0.245368
C -1.355768 -0.479046 -0.370084
C -1.696182 0.877014 -0.358441
C -0.700063 1.857873 -0.246104
C -3.125132 1.248862 -0.474413
C -4.205309 0.180479 -0.208940
C -3.681663 -1.202652 -0.532320
C -2.375925 -1.507802 -0.560783
O -1.000535 3.198990 -0.257612
O -3.425904 2.418297 -0.735588
O -5.294731 0.504585 -1.086917
C -4.659126 0.362651 1.242913
C -5.565689 -0.732013 1.760892
C -6.963696 -0.818230 1.205387
O -5.179299 -1.501177 2.643342
O -4.630397 -2.200749 -0.690020
C -4.853565 -2.494937 -2.069398
C 4.759761 0.431844 0.291871
C 5.459897 1.242768 1.346757
C 5.332796 -0.504471 -0.495197
C 6.776778 -0.962654 -0.566860
C 7.116752 -1.916275 0.592784
C 8.485241 -2.570218 0.456005
C 7.777179 0.185002 -0.734387
H 1.513069 3.473463 0.031085
H 2.638366 -1.284479 0.041938
H 0.262364 -1.902135 -0.253726
H -2.056268 -2.532326 -0.728960
H -1.951228 3.310352 -0.482962
H -5.313049 1.480397 -1.159444
H -5.194215 1.312508 1.370261
H -3.790710 0.406020 1.914008
H -7.550649 -1.518415 1.806896
H -7.440898 0.163609 1.257914
H -6.946780 -1.173467 0.174178
H -5.663238 -3.228055 -2.129151
H -5.158487 -1.603554 -2.626661
H -3.960196 -2.936703 -2.523229
H 6.375712 0.767698 1.703196
H 5.714708 2.236402 0.963534
H 4.823985 1.366328 2.231357
H 4.706604 -1.004786 -1.235488
H 6.846535 -1.549472 -1.494795
H 7.076451 -1.389274 1.552643
H 6.360746 -2.710683 0.642145
H 9.290367 -1.833126 0.529006
H 8.632902 -3.301883 1.256933
H 8.577501 -3.093169 -0.501048
H 7.410534 0.929856 -1.449401
H 8.730300 -0.190858 -1.122325
H 7.992071 0.692961 0.209547
SCF Energy (B3LYP/6-31G**)= -1361.69539860
Number of imaginary frequencies = 0

1a_c328
MMFF Geometry
C -2.091442 -1.511721 -0.636775
C -2.325259 1.157526 -0.200485
N -3.336313 -0.979439 -0.582615
C -0.906809 -0.787490 -0.481800
C -1.040500 0.592421 -0.255307
C -3.450390 0.351269 -0.366726
C 0.115247 1.375765 -0.088600
C 1.397278 0.807562 -0.142811
C 1.529195 -0.565641 -0.367144
C 0.381759 -1.359627 -0.539239
C 2.885132 -1.168448 -0.416812
C 4.128470 -0.289371 -0.163200
C 3.808204 1.181101 -0.006298
C 2.562193 1.667981 0.034656
O 0.461408 -2.714205 -0.764952
O 2.977357 -2.377572 -0.656712
O 4.905301 -0.454656 -1.354615
C 4.824770 -0.859102 1.085796
C 6.293638 -0.516965 1.163440
C 6.753385 0.323704 2.324385
O 7.092227 -0.967125 0.340106
O 4.861112 2.052805 0.241439
C 5.607012 2.346656 -0.942466
C -4.837054 0.902616 -0.331791
C -4.999543 2.350191 -0.710217
C -5.861465 0.089342 0.009842
C -7.332611 0.422633 0.123226
C -7.839853 0.296455 1.574960
C -7.808784 -1.105266 2.171999
C -8.137846 -0.440238 -0.851334
H -2.071457 -2.584593 -0.816137
H -2.435791 2.220827 -0.015019
H 0.009607 2.446556 0.084660
H 2.402562 2.729891 0.200896
H 1.410487 -2.967806 -0.830500
H 5.397190 -1.295080 -1.273181
H 4.784571 -1.956418 1.101617
H 4.317635 -0.532873 2.002338
H 7.779016 0.660229 2.149767
H 6.115226 1.203168 2.432131
H 6.719501 -0.273591 3.238722
H 6.352515 1.569124 -1.129157
H 4.957428 2.479396 -1.814755
H 6.142463 3.285518 -0.772284
H -4.382516 2.597688 -1.581785
H -6.022519 2.609082 -0.992264
H -4.708233 3.000059 0.121579
H -5.627918 -0.951158 0.244081
H -7.508912 1.463789 -0.163756
H -7.251677 0.962405 2.219557
H -8.873408 0.663218 1.618097
H -6.792480 -1.507295 2.210460
H -8.191034 -1.078840 3.197945
H -8.437346 -1.798056 1.605303
H -7.848137 -0.225507 -1.886381
H -9.209533 -0.234860 -0.757388
H -7.980630 -1.510891 -0.682159
SCF Energy (B3LYP/6-31G**)= -1361.70232810
Number of imaginary frequencies = 0

1a_c329
MMFF Geometry
C 1.887978 2.194933 0.047135
C 2.316967 -0.393071 -0.668511
N 3.170116 1.769102 -0.052871
C 0.760964 1.400542 -0.190146
C 0.995596 0.063036 -0.561958
C 3.368695 0.481428 -0.404873
C -0.096700 -0.783831 -0.824969
C -1.415858 -0.324719 -0.708236
C -1.642047 0.999139 -0.325014
C -0.565395 1.864608 -0.081876
C -3.039152 1.461282 -0.188647
C -4.163414 0.415665 -0.064041
C -3.804804 -0.881973 -0.772270
C -2.529147 -1.215696 -1.042534
O -0.759292 3.179059 0.269593
O -3.271221 2.672838 -0.123822
O -5.312648 1.008468 -0.691656
C -4.448829 0.248173 1.432650
C -5.372181 -0.904892 1.758236
C -6.838774 -0.744256 1.452488
O -4.933752 -1.934987 2.274973
O -4.904764 -1.664927 -1.072943
C -4.699477 -2.954082 -1.636429
C 4.779196 0.043063 -0.498762
C 5.660311 0.919165 -1.341518
C 5.179397 -1.031603 0.214704

C 6.527430 -1.716236 0.334778
C 7.179075 -1.437941 1.705252
C 7.573316 0.012990 1.951236
C 7.517447 -1.532200 -0.817090
H 1.784775 3.238670 0.336467
H 2.528807 -1.416848 -0.962196
H 0.090180 -1.814509 -1.125444
H -2.270929 -2.149642 -1.529626
H -1.714361 3.395117 0.177211
H -5.239105 1.974755 -0.559961
H -4.898335 1.157061 1.852605
H -3.516572 0.081292 1.988731
H -7.397476 -1.564590 1.912255
H -7.201378 0.196778 1.873673
H -7.009377 -0.764618 0.375048
H -4.226238 -2.879240 -2.620595
H -4.110776 -3.586118 -0.963792
H -5.678925 -3.423168 -1.768450
H 6.533386 1.256207 -0.774662
H 5.989659 0.383663 -2.237319
H 5.149681 1.823125 -1.691322
H 4.430485 -1.517560 0.845027
H 6.291030 -2.791031 0.326564
H 6.491670 -1.749277 2.502585
H 8.072897 -2.065608 1.811241
H 6.706174 0.678636 1.911945
H 8.020719 0.112143 2.945803
H 8.311232 0.359367 1.222336
H 7.046966 -1.763009 -1.778834
H 8.364408 -2.217855 -0.698644
H 7.930447 -0.522198 -0.867324
SCF Energy (B3LYP/6-31G**)= -1361.69982868
Number of imaginary frequencies = 0

1a_c330
MMFF Geometry
C -1.705244 -1.727055 -0.574892
C -2.056563 0.902500 0.001367
N -2.971704 -1.273785 -0.409483
C -0.555421 -0.939299 -0.474250
C -0.749815 0.419761 -0.176101
C -3.142285 0.036695 -0.121057
C 0.368173 1.265202 -0.062600
C 1.673179 0.780236 -0.240685
C 1.865353 -0.575607 -0.529941
C 0.755345 -1.429939 -0.652202
C 3.245330 -1.092825 -0.724021
C 4.447279 -0.139371 -0.525276
C 4.062097 1.299664 -0.264634
C 2.795233 1.708980 -0.115199
O 0.894182 -2.765047 -0.954717
O 3.384772 -2.264640 -1.088133
O 5.199044 -0.160561 -1.758069
C 5.384660 -0.713510 0.544541
C 4.806738 -0.667269 1.941159
C 5.794540 -0.670422 3.077759
O 3.594658 -0.681120 2.153346
O 5.114474 2.178229 -0.050108
C 5.328038 3.032604 -1.173707
C -4.549487 0.504290 0.042059
C -4.828926 1.931354 -0.338441
C -5.485092 -0.368705 0.477836
C -6.960153 -0.173818 0.765069
C -7.786986 -0.204446 -0.532988
C -9.290531 -0.221123 -0.291864
C -7.261657 1.033242 1.658676
H -1.636308 -2.788970 -0.801002
H -2.215449 1.947052 0.248394
H 0.214317 2.317950 0.172418
H 2.582027 2.743113 0.139095
H 1.852898 -2.964533 -1.055817
H 4.572072 0.001965 -2.483225
H 6.332482 -0.159780 0.541064
H 5.641690 -1.757075 0.324243
H 5.260052 -0.633813 4.031061
H 6.392284 -1.584340 3.040207
H 6.440789 0.207759 3.004292
H 5.525236 2.454694 -2.082105
H 4.473728 3.700100 -1.327314
H 6.207188 3.648951 -0.964308
H -5.877536 2.102098 -0.589372
H -4.558176 2.608932 0.477813
H -4.264671 2.218567 -1.233281
H -5.160001 -1.391755 0.684290
H -7.256281 -1.053883 1.355091
H -7.544515 0.656161 -1.166628
H -7.522608 -1.099967 -1.110235
H -9.632463 0.710012 0.169597
H -9.821532 -0.333643 -1.242651
H -9.575835 -1.056062 0.355764
H -6.534636 1.115125 2.474253
H -8.250577 0.931081 2.119048
H -7.262100 1.975028 1.103506
SCF Energy (B3LYP/6-31G**)= -1361.69421859
Number of imaginary frequencies = 0

1a_c331
MMFF Geometry
C -1.573191 -1.862324 -0.879330
C -2.284349 0.715712 -0.389466
N -2.894177 -1.573887 -0.787402
C -0.540578 -0.929614 -0.741808
C -0.920932 0.403545 -0.490389
C -3.235496 -0.292405 -0.534040
C 0.069597 1.394531 -0.359182
C 1.428228 1.071413 -0.451436
C 1.799325 -0.258072 -0.682279
C 0.827069 -1.251990 -0.849061
C 3.244564 -0.568704 -0.770065
C 4.227463 0.393405 -0.052962
C 3.738567 1.827424 -0.228296
C 2.442646 2.125245 -0.404924
O 1.164593 -2.554148 -1.126363
O 3.598843 -1.585194 -1.368486
O 5.528303 0.270292 -0.634429
C 4.349405 0.025983 1.430522
C 4.394481 -1.469291 1.652324
C 5.691730 -2.186091 1.388035
O 3.382435 -2.073806 2.009738
O 4.668109 2.848066 -0.371372
C 5.462934 3.068198 0.790708
C -4.690646 -0.019715 -0.444389
C -5.504173 -0.699107 -1.510561
C -5.174786 0.756921 0.549037
C -6.604230 1.134492 0.880091
C -7.586950 -0.038467 1.061969
C -7.137369 -1.046160 2.112511
C -7.149954 2.174399 -0.101954
H -1.355870 -2.910264 -1.075547
H -2.603259 1.738503 -0.213959
H -0.227800 2.428612 -0.188933
H 2.130009 3.152773 -0.569015
H 2.136972 -2.608688 -1.262777
H 5.450488 0.569711 -1.558999
H 3.494432 0.405464 2.005334
H 5.250093 0.449943 1.888316
H 5.641000 -3.195516 1.805952
H 5.869889 -2.254674 0.313183
H 6.513926 -1.654778 1.873827
H 4.849997 3.084872 1.698328
H 6.252013 2.315656 0.870373
H 5.943354 4.045699 0.687622
H -5.643898 -1.758892 -1.272483
H -6.489345 -0.249046 -1.642498
H -5.007457 -0.626178 -2.485245
H -4.465798 1.162109 1.272752
H -6.555885 1.639722 1.856281
H -8.559637 0.364256 1.372845
H -7.766336 -0.561474 0.117817
H -6.957977 -0.556351 3.074859
H -7.912716 -1.804987 2.259557
H -6.221480 -1.562534 1.810231

H -6.466965 3.026579 -0.192078
H -8.114763 2.560318 0.245286
H -7.302153 1.758719 -1.102232
SCF Energy (B3LYP/6-31G**)= -1361.68763949
Number of imaginary frequencies = 0

1a_c332
MMFF Geometry
C -1.571924 -1.776493 -1.010523
C -2.231473 0.787323 -0.388724
N -2.887600 -1.460082 -0.937168
C -0.520781 -0.879561 -0.791745
C -0.874360 0.445682 -0.472292
C -3.200447 -0.185805 -0.622684
C 0.137225 1.399152 -0.253727
C 1.491937 1.049235 -0.331304
C 1.835292 -0.272577 -0.630999
C 0.841358 -1.229553 -0.879757
C 3.270359 -0.619517 -0.708496
C 4.274074 0.293709 0.034302
C 3.819461 1.751200 -0.011765
C 2.522722 2.079410 -0.165349
O 1.151923 -2.523874 -1.220228
O 3.598340 -1.628534 -1.335050
O 5.552685 0.186662 -0.605616
C 4.458577 -0.140043 1.490255
C 4.533821 -1.642029 1.636630
C 5.833523 -2.323333 1.301017
O 3.541133 -2.281379 1.989646
O 4.855632 2.653502 0.142104
C 4.557301 4.044754 0.147733
C -4.644580 0.128065 -0.541234
C -5.451863 -0.289346 -1.736284
C -5.131768 0.677710 0.592177
C -6.533684 1.103787 0.983536
C -7.147038 0.136826 2.017499
C -7.410028 -1.274285 1.506871
C -7.513098 1.429909 -0.145712
H -1.374386 -2.816895 -1.260655
H -2.535048 1.802793 -0.153048
H -0.142661 2.426472 -0.023207
H 2.183522 3.109480 -0.178705
H 2.127521 -2.599815 -1.324292
H 5.484928 0.653927 -1.456416
H 3.619295 0.194991 2.114138
H 5.368476 0.288494 1.928277
H 5.819145 -3.345268 1.690413
H 5.970345 -2.356490 0.218438
H 6.663863 -1.789935 1.770403
H 4.119858 4.354301 -0.806815
H 3.897663 4.299440 0.983423
H 5.496491 4.589677 0.280981
H -4.866306 -0.860213 -2.465321
H -6.287026 -0.930742 -1.439130
H -5.834678 0.588343 -2.266195
H -4.423233 0.848509 1.406501
H -6.392835 2.059136 1.511361
H -6.483180 0.070790 2.889352
H -8.091731 0.557089 2.384996
H -6.488018 -1.762407 1.177937
H -7.838813 -1.884772 2.308504
H -8.120538 -1.276325 0.675629
H -7.072695 2.140381 -0.853421
H -8.416145 1.898072 0.262730
H -7.835937 0.545192 -0.699114
SCF Energy (B3LYP/6-31G**)= -1361.69117933
Number of imaginary frequencies = 0

1a_c333
MMFF Geometry
C -1.480570 -2.204594 -0.431022
C -2.350746 0.339593 -0.028520
N -2.808104 -2.009690 -0.240370
C -0.517234 -1.191097 -0.436976
C -0.979348 0.123010 -0.225302
C -3.229892 -0.741508 -0.049338
C -0.061369 1.189644 -0.224734
C 1.306712 0.962372 -0.413028
C 1.760526 -0.347174 -0.607171
C 0.858511 -1.417757 -0.641315
C 3.214098 -0.555520 -0.798392
C 4.172688 0.514690 -0.213841
C 3.561795 1.896942 -0.419676
C 2.236817 2.088658 -0.503250
O 1.273689 -2.705616 -0.876772
O 3.598006 -1.575356 -1.372334
O 5.429506 0.454795 -0.893725
C 4.438743 0.240911 1.271050
C 4.616959 -1.232259 1.562814
C 5.941183 -1.865693 1.228590
O 3.686052 -1.888398 2.032214
O 4.395277 2.973298 -0.689847
C 5.259198 3.316028 0.390352
C -4.688236 -0.572348 0.162129
C -5.288530 -1.587577 1.094486
C -5.354738 0.413971 -0.475976
C -6.828376 0.764864 -0.454804
C -7.305206 1.399803 0.864751
C -6.542606 2.665712 1.236398
C -7.731015 -0.398860 -0.875748
H -1.197604 -3.244046 -0.583943
H -2.729503 1.340813 0.152565
H -0.423811 2.207014 -0.082110
H 1.833497 3.079718 -0.691927
H 2.233118 -2.696227 -1.092422
H 5.256387 0.695248 -1.822566
H 3.604635 0.588314 1.894724
H 5.336947 0.754753 1.631266
H 6.001903 -2.851085 1.699207
H 6.039237 -1.981205 0.147482
H 6.755237 -1.249109 1.617738
H 4.719786 3.338722 1.343381
H 6.108066 2.628986 0.442380
H 5.652811 4.318759 0.199201
H -4.651905 -1.729596 1.975690
H -6.266585 -1.289648 1.474802
H -5.402883 -2.553637 0.591396
H -4.789241 1.060171 -1.149240
H -6.958970 1.529646 -1.234895
H -8.368087 1.656555 0.771589
H -7.232543 0.686957 1.692768
H -6.595211 3.406083 0.431862
H -6.976024 3.113639 2.136442
H -5.489423 2.454466 1.445059
H -7.366773 -0.867527 -1.796630
H -8.747563 -0.038176 -1.069443
H -7.805190 -1.172416 -0.106947
SCF Energy (B3LYP/6-31G**)= -1361.68770732
Number of imaginary frequencies = 0

1a_c334
MMFF Geometry
C 1.573671 -2.301435 -0.121197
C 2.481273 0.261442 -0.145045
N 2.914010 -2.102997 -0.105072
C 0.615161 -1.283120 -0.150301
C 1.096871 0.039814 -0.165798
C 3.351698 -0.825803 -0.107741
C 0.182780 1.108631 -0.211193
C -1.199859 0.880011 -0.222082
C -1.671118 -0.433901 -0.187791
C -0.775129 -1.512555 -0.171929
C -3.133326 -0.649283 -0.200989
C -4.039546 0.500280 0.303025
C -3.448027 1.851253 -0.110767
C -2.128609 2.007265 -0.333392
O -1.212762 -2.815541 -0.180751
O -3.552007 -1.747846 -0.572816
O -4.051847 0.507747 1.734013
C -5.464056 0.314521 -0.259126
C -6.129291 -0.944642 0.261325
C -7.092154 -1.641827 -0.660816

O -5.931873 -1.344454 1.411058
O -4.382879 2.870659 -0.181637
C -3.924615 4.204943 -0.365236
C 4.823086 -0.651227 -0.089395
C 5.553761 -1.505730 -1.084886
C 5.384121 0.214476 0.782098
C 6.834380 0.543718 1.079406
C 7.249633 1.897882 0.470646
C 7.255635 1.950672 -1.051612
C 7.869616 -0.553918 0.821677
H 1.275783 -3.347799 -0.109823
H 2.879237 1.271266 -0.169946
H 0.559268 2.130496 -0.237097
H -1.695492 2.965054 -0.597583
H -2.186676 -2.817714 -0.319394
H -4.590890 -0.262120 2.019658
H -5.435880 0.273641 -1.354622
H -6.127751 1.134980 0.038998
H -7.488821 -2.535765 -0.171748
H -7.919652 -0.969913 -0.900847
H -6.572611 -1.943226 -1.573735
H -3.248252 4.502751 0.442507
H -3.446130 4.321738 -1.342761
H -4.795710 4.866187 -0.336568
H 4.913086 -1.777892 -1.932144
H 6.401715 -0.974697 -1.523780
H 5.905735 -2.430563 -0.617299
H 4.711752 0.763137 1.445564
H 6.860506 0.687353 2.170575
H 6.575316 2.681409 0.840243
H 8.251275 2.161230 0.833204
H 6.262624 1.753285 -1.465705
H 7.560258 2.947707 -1.386763
H 7.960150 1.230928 -1.477389
H 7.540035 -1.512018 1.237735
H 8.816733 -0.296187 1.309850
H 8.091537 -0.695625 -0.237854
SCF Energy (B3LYP/6-31G**)= -1361.70375069
Number of imaginary frequencies = 0

1a_c335
MMFF Geometry
C -1.572332 -1.667111 -0.626414
C -2.165379 0.790565 0.366236
N -2.871002 -1.378104 -0.369073
C -0.504607 -0.790350 -0.420398
C -0.823548 0.480532 0.092247
C -3.162823 -0.153931 0.127390
C 0.205567 1.415157 0.311475
C 1.543073 1.097509 0.041458
C 1.853814 -0.174388 -0.451943
C 0.839752 -1.109316 -0.699978
C 3.269925 -0.486273 -0.740999
C 4.370311 0.368045 -0.080337
C 3.884719 1.787678 0.142358
C 2.584688 2.107311 0.235617
O 1.110206 -2.351089 -1.221906
O 3.549321 -1.417378 -1.502377
O 5.476887 0.396465 -0.998720
C 4.857919 -0.217723 1.246799
C 4.954564 -1.723836 1.216525
C 6.242882 -2.336040 0.736492
O 3.989292 -2.418059 1.537701
O 4.856600 2.734833 0.427874
C 5.155422 3.529185 -0.720639
C -4.601231 0.120875 0.416668
C -4.884724 1.090902 1.529572
C -5.549057 -0.526497 -0.297738
C -7.061125 -0.439802 -0.245445
C -7.599456 0.992085 -0.429468
C -9.099109 1.037502 -0.696212
C -7.622433 -1.153469 0.986551
H -1.405824 -2.667807 -1.019532
H -2.422520 1.772225 0.750305
H -0.045232 2.403159 0.696238
H 2.285271 3.122690 0.478531
H 2.068906 -2.408726 -1.430287
H 5.357095 -0.361806 -1.606107
H 4.171780 0.030470 2.067647
H 5.840265 0.182929 1.525631
H 6.219405 -3.416106 0.906812
H 6.370220 -2.148017 -0.331336
H 7.082158 -1.916665 1.296793
H 5.492993 2.913999 -1.560460
H 4.287298 4.127714 -1.016684
H 5.965095 4.214410 -0.453104
H -4.762874 2.122388 1.182975
H -5.893242 0.990720 1.933685
H -4.209897 0.923833 2.377068
H -5.207789 -1.230791 -1.060945
H -7.410664 -1.013783 -1.116449
H -7.392878 1.609623 0.449994
H -7.087082 1.467002 -1.275884
H -9.670634 0.685202 0.167370
H -9.411763 2.066230 -0.902785
H -9.364021 0.423799 -1.562887
H -7.158768 -2.137967 1.116715
H -8.700443 -1.316431 0.886360
H -7.459157 -0.581418 1.904367
SCF Energy (B3LYP/6-31G**)= -1361.69383960
Number of imaginary frequencies = 0

1a_c336
MMFF Geometry
C -1.450520 -2.213576 -0.550038
C -2.327435 0.301181 -0.001974
N -2.781911 -2.029216 -0.377738
C -0.486609 -1.203355 -0.467251
C -0.952207 0.094570 -0.181572
C -3.205200 -0.775128 -0.112395
C -0.032340 1.155568 -0.088465
C 1.341576 0.941719 -0.262936
C 1.796206 -0.352316 -0.532182
C 0.893682 -1.417719 -0.652863
C 3.245672 -0.551839 -0.721546
C 4.216079 0.498987 -0.148395
C 3.602909 1.894183 -0.171607
C 2.269188 2.077225 -0.215271
O 1.311879 -2.690779 -0.955629
O 3.652340 -1.548316 -1.325980
O 5.380421 0.492882 -0.994701
C 4.663086 0.168439 1.277652
C 4.906445 -1.307504 1.480736
C 6.264582 -1.852452 1.129045
O 4.000454 -2.039103 1.882394
O 4.540589 2.908286 -0.092956
C 4.100116 4.256630 -0.190726
C -4.666736 -0.616637 0.073097
C -5.279665 -1.629668 0.996926
C -5.321089 0.372449 -0.572838
C -6.793916 0.728559 -0.636877
C -7.122311 1.961066 0.229076
C -6.945192 1.766107 1.729310
C -7.799493 -0.408356 -0.438926
H -1.164630 -3.241280 -0.764510
H -2.711732 1.288877 0.233594
H -0.399517 2.159833 0.120243
H 1.823180 3.065626 -0.205347
H 2.278404 -2.676091 -1.133604
H 5.320620 -0.305544 -1.556583
H 3.899734 0.460443 2.011234
H 5.580669 0.705893 1.546878
H 6.348499 -2.881957 1.488494
H 6.402004 -1.841972 0.046074
H 7.039935 -1.254374 1.614158
H 3.587120 4.433904 -1.141504
H 3.458835 4.519654 0.656494
H 4.982588 4.902376 -0.157837
H -6.064233 -1.186490 1.614745
H -5.693044 -2.470233 0.430882
H -4.544144 -2.028546 1.705651
H -4.728714 1.029348 -1.213617

H -6.949642 1.047039 -1.679128
H -6.490990 2.802003 -0.086325
H -8.158088 2.268024 0.036646
H -5.911269 1.515790 1.983966
H -7.199457 2.691922 2.256014
H -7.599231 0.978624 2.113710
H -7.529882 -1.282324 -1.041563
H -8.796247 -0.085887 -0.761828
H -7.893823 -0.722702 0.602335
SCF Energy (B3LYP/6-31G**)= -1361.69596926
Number of imaginary frequencies = 0

1a_c337
MMFF Geometry
C 1.959906 1.831641 -0.009117
C 2.210343 -0.865664 -0.225894
N 3.203823 1.299847 0.060112
C 0.785513 1.093939 -0.183160
C 0.927131 -0.301597 -0.293645
C 3.321351 -0.042825 -0.050256
C -0.217654 -1.099727 -0.474452
C -1.497768 -0.531626 -0.533880
C -1.631664 0.853049 -0.411288
C -0.500471 1.666694 -0.252321
C -2.989769 1.433891 -0.457792
C -4.204137 0.514692 -0.227319
C -3.918214 -0.914356 -0.663347
C -2.662505 -1.384999 -0.779133
O -0.600943 3.033889 -0.154756
O -3.121139 2.650432 -0.627202
O -5.261411 1.064038 -1.031346
C -4.586626 0.648262 1.251016
C -5.622871 -0.352718 1.711894
C -7.050572 -0.142576 1.278662
O -5.304741 -1.299445 2.434961
O -5.062327 -1.656658 -0.894277
C -4.936536 -3.039844 -1.198450
C 4.701529 -0.597085 0.014871
C 4.995597 -1.746820 -0.905103
C 5.594702 -0.014309 0.844312
C 7.031460 -0.349600 1.189867
C 8.028463 0.602373 0.499970
C 8.078302 0.502444 -1.018914
C 7.451709 -1.817656 1.081354
H 1.931128 2.915212 0.083827
H 2.336650 -1.941612 -0.295238
H -0.103234 -2.179102 -0.571077
H -2.458100 -2.409386 -1.070688
H -1.527317 3.299273 -0.351618
H -5.114003 2.030098 -1.069634
H -4.978991 1.650001 1.467967
H -3.704952 0.517979 1.892775
H -7.703120 -0.818841 1.838419
H -7.353422 0.884842 1.495731
H -7.161533 -0.350351 0.213335
H -4.404800 -3.184909 -2.144150
H -4.441936 -3.576713 -0.382593
H -5.943505 -3.452171 -1.311533
H 4.362220 -1.720351 -1.799459
H 6.019312 -1.712342 -1.283686
H 4.831489 -2.702167 -0.396972
H 5.244251 0.852704 1.412357
H 7.106963 -0.128636 2.265797
H 7.783225 1.637990 0.769356
H 9.034636 0.414313 0.895392
H 7.110848 0.740607 -1.470325
H 8.810345 1.214927 -1.413496
H 8.380454 -0.495654 -1.347761
H 6.707411 -2.476215 1.541853
H 8.398015 -1.974953 1.612006
H 7.612389 -2.145027 0.052356
SCF Energy (B3LYP/6-31G**)= -1361.70010370
Number of imaginary frequencies = 0

1a_c338
MMFF Geometry
C 1.760443 2.292487 0.151558
C 2.315265 -0.290858 -0.485937
N 3.061369 1.921167 0.079834
C 0.673249 1.443304 -0.078060
C 0.973033 0.108719 -0.410696
C 3.325826 0.633190 -0.227726
C -0.076194 -0.792392 -0.667819
C -1.415050 -0.388702 -0.582818
C -1.708014 0.933748 -0.236064
C -0.673933 1.851341 -0.000250
C -3.128564 1.340166 -0.133641
C -4.216073 0.252972 -0.015051
C -3.768913 -1.027218 -0.688148
C -2.481477 -1.334414 -0.905001
O -0.929538 3.164848 0.313381
O -3.412186 2.542042 -0.097854
O -5.367375 0.780881 -0.692189
C -4.533921 0.107045 1.476489
C -5.425306 -1.066549 1.817843
C -6.869292 -1.008930 1.391203
O -4.986604 -2.020209 2.464145
O -4.760453 -1.951362 -0.977922
C -5.112471 -1.921997 -2.361493
C 4.762260 0.268989 -0.299271
C 5.612530 1.287071 -1.007400
C 5.199030 -0.877298 0.266459
C 6.601838 -1.445401 0.361717
C 7.584276 -0.507558 1.088535
C 8.903919 -1.181041 1.444022
C 7.109127 -1.938209 -0.995679
H 1.607745 3.338265 0.409878
H 2.571156 -1.310760 -0.756680
H 0.158961 -1.821738 -0.937639
H -2.211838 -2.297923 -1.328155
H -1.892329 3.335615 0.209144
H -5.359339 1.747994 -0.542427
H -5.025322 1.009265 1.862859
H -3.608754 -0.012017 2.056391
H -7.422139 -1.821860 1.870879
H -7.308581 -0.060105 1.709392
H -6.955626 -1.119295 0.309427
H -5.434711 -0.923559 -2.673537
H -4.277236 -2.260754 -2.983470
H -5.948341 -2.611933 -2.509525
H 5.771802 2.165717 -0.373207
H 6.591816 0.900807 -1.292768
H 5.131983 1.613664 -1.937081
H 4.468069 -1.504856 0.779088
H 6.505812 -2.341731 0.992340
H 7.805449 0.378288 0.484988
H 7.122611 -0.147851 2.017028
H 9.469106 -1.452907 0.547773
H 9.525693 -0.499185 2.033224
H 8.737471 -2.084948 2.038308
H 6.342896 -2.527159 -1.512521
H 7.983651 -2.585587 -0.872889
H 7.400134 -1.114000 -1.652785
SCF Energy (B3LYP/6-31G**)= -1361.69570225
Number of imaginary frequencies = 0

1a_c339
MMFF Geometry
C 1.843437 -2.557794 -0.054027
C 2.570310 0.045686 0.226937
N 3.162642 -2.277400 0.078998
C 0.820226 -1.604533 -0.058544
C 1.208361 -0.260147 0.088658
C 3.513297 -0.980170 0.210659
C 0.225373 0.746410 0.089230
C -1.135276 0.433951 -0.042922
C -1.516100 -0.903089 -0.176447
C -0.546796 -1.917981 -0.199992
C -2.956736 -1.214708 -0.314876
C -3.980337 -0.160825 0.164646
C -3.445467 1.256977 -0.014928
C -2.126043 1.511192 -0.096964

O -0.887401 -3.240282 -0.361806
O -3.264407 -2.323978 -0.760010
O -4.108241 -0.405075 1.572733
C -5.303544 -0.434418 -0.580114
C -6.494389 0.292858 0.007565
C -7.560129 0.745092 -0.953724
O -6.614024 0.457654 1.223457
O -4.433439 2.223857 -0.056014
C -4.052937 3.594319 -0.004694
C 4.964651 -0.718594 0.359803
C 5.681813 -1.690213 1.258374
C 5.550407 0.307645 -0.293735
C 7.018189 0.677251 -0.284186
C 7.295222 1.892480 0.621962
C 6.617442 3.192205 0.205079
C 7.513900 0.897130 -1.716957
H 1.619237 -3.617125 -0.160325
H 2.891274 1.074078 0.360582
H 0.530832 1.786752 0.194487
H -1.737309 2.517389 -0.204811
H -1.854479 -3.291957 -0.538008
H -4.967198 -0.020067 1.851476
H -5.572417 -1.497334 -0.536854
H -5.199573 -0.164884 -1.638169
H -8.353238 1.263169 -0.407592
H -7.127564 1.435626 -1.681963
H -7.986104 -0.123163 -1.462181
H -3.476732 3.808507 0.901219
H -3.494836 3.877196 -0.902749
H -4.966695 4.195085 0.026305
H 5.873879 -2.631486 0.732771
H 6.637081 -1.302097 1.621925
H 5.085116 -1.907502 2.152083
H 4.936199 0.937952 -0.936619
H 7.608608 -0.156807 0.111775
H 6.985431 1.653128 1.647450
H 8.378200 2.065018 0.661508
H 5.528039 3.093702 0.191557
H 6.868600 3.985911 0.916536
H 6.950223 3.519781 -0.783952
H 7.415820 -0.022901 -2.304565
H 8.571217 1.182849 -1.721572
H 6.948740 1.679220 -2.235010
SCF Energy (B3LYP/6-31G**)= -1361.70979999
Number of imaginary frequencies = 0

1a_c340
MMFF Geometry
C -1.666753 -1.553810 -0.634316
C -2.131329 1.022550 0.095658
N -2.950082 -1.172181 -0.425478
C -0.554605 -0.717553 -0.503044
C -0.806752 0.614226 -0.123890
C -3.173593 0.111007 -0.063113
C 0.270845 1.508191 0.019502
C 1.591946 1.092326 -0.194535
C 1.834898 -0.236287 -0.555531
C 0.772517 -1.134261 -0.728913
C 3.235724 -0.653526 -0.777460
C 4.352076 0.186485 -0.113213
C 3.973250 1.665896 -0.081878
C 2.687126 2.063745 -0.103686
O 0.980197 -2.433415 -1.124643
O 3.449062 -1.660081 -1.455373
O 5.557146 0.032808 -0.874769
C 4.649676 -0.296800 1.308007
C 4.659332 -1.804213 1.411497
C 5.884112 -2.540041 0.937671
O 3.672025 -2.402766 1.842511
O 5.065811 2.509735 -0.006236
C 4.843862 3.913571 0.061193
C -4.593822 0.504563 0.147289
C -4.976450 1.876245 -0.328391
C -5.439300 -0.394242 0.697359
C -6.899283 -0.308054 1.093320
C -7.813952 -1.035122 0.087838
C -7.867557 -0.420621 -1.304651
C -7.436810 1.071213 1.483507
H -1.550452 -2.596650 -0.922108
H -2.343778 2.041751 0.402510
H 0.069485 2.541676 0.299134
H 2.403584 3.109684 -0.060620
H 1.935958 -2.556022 -1.324259
H 5.433750 0.525158 -1.704914
H 3.891583 0.062181 2.016736
H 5.618018 0.073080 1.667071
H 5.853000 -3.570392 1.303179
H 5.916303 -2.549926 -0.153505
H 6.781822 -2.062055 1.337606
H 4.335826 4.272408 -0.839625
H 4.280103 4.177119 0.961796
H 5.819094 4.406050 0.117387
H -4.339013 2.208941 -1.155875
H -5.992336 1.898450 -0.728438
H -4.892899 2.605736 0.483199
H -5.021218 -1.375593 0.941640
H -6.959520 -0.892017 2.024711
H -7.486309 -2.078495 -0.008265
H -8.833137 -1.071411 0.492873
H -6.882736 -0.414708 -1.780630
H -8.538315 -1.005779 -1.942529
H -8.248755 0.603912 -1.278995
H -6.749945 1.583878 2.165513
H -8.394592 0.964174 2.006021
H -7.619841 1.720919 0.625476
SCF Energy (B3LYP/6-31G**)= -1361.69140668
Number of imaginary frequencies = 0

1a_c341
MMFF Geometry
C -1.634581 -1.509529 -0.726264
C -2.151206 1.015721 0.130668
N -2.927628 -1.151636 -0.536029
C -0.536158 -0.672614 -0.514713
C -0.815477 0.632769 -0.070519
C -3.180751 0.106744 -0.109019
C 0.247123 1.526840 0.157133
C 1.578356 1.137134 -0.041079
C 1.848820 -0.166325 -0.469225
C 0.802216 -1.063898 -0.722140
C 3.258475 -0.553675 -0.687221
C 4.377542 0.285010 -0.035603
C 3.944961 1.730789 0.134022
C 2.656964 2.103225 0.166756
O 1.034508 -2.338690 -1.179157
O 3.520944 -1.534423 -1.390914
O 5.491936 0.225489 -0.943278
C 4.824399 -0.272424 1.318312
C 4.840640 -1.781563 1.353835
C 6.101531 -2.480698 0.922131
O 3.835819 -2.409467 1.689564
O 4.913161 2.665836 0.473879
C 5.654747 3.114266 -0.660451
C -4.615584 0.470548 0.075111
C -4.976030 1.910988 -0.158623
C -5.502009 -0.497598 0.397994
C -6.989918 -0.421706 0.675011
C -7.795864 -0.364237 -0.635212
C -9.298703 -0.495114 -0.426875
C -7.376381 0.664549 1.683540
H -1.499201 -2.533430 -1.068230
H -2.377150 2.015759 0.486178
H 0.028036 2.540320 0.491868
H 2.394842 3.136274 0.377699
H 1.997179 -2.448847 -1.343297
H 5.356379 -0.566039 -1.503896
H 4.147201 0.045816 2.122318
H 5.824668 0.087443 1.589021
H 6.012622 -3.551826 1.124289
H 6.262031 -2.334115 -0.147718
H 6.951284 -2.091647 1.488479
H 6.520114 2.467563 -0.828382

H 5.037671 3.174854 -1.564002
H 6.029277 4.117994 -0.437822
H -4.416871 2.324951 -1.005733
H -6.029222 2.044931 -0.412755
H -4.759641 2.512503 0.730217
H -5.118225 -1.515915 0.501370
H -7.239713 -1.375005 1.164040
H -7.597727 0.571188 -1.170629
H -7.469733 -1.176728 -1.297454
H -9.702858 0.360352 0.122409
H -9.809058 -0.538025 -1.394510
H -9.541311 -1.409049 0.124313
H -6.666647 0.703058 2.517341
H -8.363273 0.456041 2.111220
H -7.426898 1.657854 1.229700
SCF Energy (B3LYP/6-31G**)= -1361.69276012
Number of imaginary frequencies = 0

1a_c342
MMFF Geometry
C -1.482003 -2.255415 -0.642646
C -2.411636 0.279887 -0.326720
N -2.820040 -2.078809 -0.525240
C -0.535324 -1.227010 -0.608469
C -1.028363 0.082070 -0.445749
C -3.271019 -0.816451 -0.361971
C -0.127316 1.162259 -0.414277
C 1.253216 0.954073 -0.520578
C 1.737526 -0.347061 -0.675245
C 0.853141 -1.432799 -0.733337
C 3.202228 -0.546866 -0.763410
C 4.127477 0.552942 -0.183399
C 3.491825 1.927108 -0.395829
C 2.168843 2.091109 -0.544261
O 1.303520 -2.720175 -0.900434
O 3.617166 -1.620106 -1.205141
O 5.376475 0.515513 -0.871326
C 4.327902 0.267856 1.317736
C 4.909686 -1.110333 1.562460
C 4.276158 -1.942154 2.644471
O 5.879496 -1.516019 0.922147
O 4.288413 3.048908 -0.595512
C 5.266704 3.299545 0.405233
C -4.741453 -0.668152 -0.247315
C -5.526425 -1.529211 -1.200177
C -5.277138 0.165864 0.669664
C -6.744984 0.408925 0.948096
C -7.142491 1.865029 0.643045
C -7.016652 2.225419 -0.832049
C -7.039725 0.081346 2.415629
H -1.176946 -3.291889 -0.771131
H -2.816697 1.281835 -0.222166
H -0.511430 2.176422 -0.307766
H 1.760919 3.079647 -0.737855
H 2.254404 -2.691142 -1.157557
H 5.736210 -0.390257 -0.751516
H 3.375585 0.343745 1.856506
H 5.027506 0.970385 1.781354
H 4.790010 -2.904528 2.718058
H 4.358476 -1.421497 3.601564
H 3.226222 -2.121658 2.399830
H 4.815397 3.300848 1.402667
H 6.091182 2.584300 0.351046
H 5.681417 4.295849 0.224511
H -5.559934 -2.564595 -0.845045
H -6.554351 -1.184585 -1.336442
H -5.071106 -1.519613 -2.197494
H -4.608838 0.722416 1.327176
H -7.376002 -0.251097 0.344883
H -8.184557 2.025126 0.947018
H -6.532698 2.559884 1.234353
H -7.608217 1.545586 -1.453417
H -7.382341 3.243249 -1.001723
H -5.976466 2.186087 -1.169217
H -6.777722 -0.957507 2.645838
H -8.105198 0.211306 2.634452
H -6.476170 0.730525 3.095471
SCF Energy (B3LYP/6-31G**)= -1361.70076725
Number of imaginary frequencies = 0

1a_c343
MMFF Geometry
C -1.661089 -1.847799 -0.523948
C -2.089104 0.710004 0.278233
N -2.935499 -1.463607 -0.268903
C -0.539821 -1.021962 -0.408924
C -0.774073 0.300131 0.004198
C -3.144618 -0.189270 0.133937
C 0.312978 1.183144 0.131451
C 1.626371 0.770851 -0.142286
C 1.858943 -0.549573 -0.543874
C 0.779592 -1.439864 -0.682836
C 3.247758 -0.989681 -0.838806
C 4.418405 -0.002108 -0.620847
C 3.988443 1.395019 -0.233187
C 2.714719 1.735970 0.003877
O 0.957219 -2.740465 -1.095371
O 3.416745 -2.123592 -1.298002
O 5.110643 0.102332 -1.883889
C 5.428159 -0.613445 0.358680
C 4.916319 -0.697974 1.779109
C 5.956829 -0.746311 2.866610
O 3.717221 -0.778635 2.043725
O 5.013669 2.300358 0.000589
C 5.137076 3.246104 -1.061675
C -4.557556 0.192282 0.427313
C -4.758152 1.259172 1.467204
C -5.557525 -0.449721 -0.217441
C -7.060342 -0.269083 -0.144082
C -7.518530 1.174415 -0.427533
C -9.018708 1.289071 -0.669392
C -7.633725 -0.852708 1.149291
H -1.560859 -2.884753 -0.837341
H -2.279352 1.731909 0.589136
H 0.128274 2.207460 0.453706
H 2.472331 2.737813 0.346081
H 1.917336 -2.889770 -1.253618
H 4.443944 0.293360 -2.565347
H 6.351601 -0.019944 0.355810
H 5.716267 -1.625165 0.047156
H 5.467607 -0.804836 3.842848
H 6.586653 -1.628877 2.731125
H 6.564712 0.161205 2.833013
H 5.311761 2.747973 -2.020539
H 4.250336 3.885575 -1.122936
H 6.000334 3.882388 -0.845886
H -4.584898 2.252268 1.039791
H -5.761053 1.250162 1.896831
H -4.074456 1.117055 2.312141
H -5.275182 -1.229219 -0.929912
H -7.462612 -0.885336 -0.961815
H -7.256330 1.843153 0.398011
H -6.998887 1.552594 -1.317166
H -9.589867 1.037764 0.229024
H -9.275633 2.316099 -0.948515
H -9.338614 0.628602 -1.481411
H -7.225389 -1.850565 1.346036
H -8.721430 -0.957885 1.081971
H -7.416176 -0.223751 2.017266
SCF Energy (B3LYP/6-31G**)= -1361.69444427
Number of imaginary frequencies = 0

1a_c344
MMFF Geometry
C -1.548773 -2.078729 -0.379834
C -2.343597 0.403382 0.394030
N -2.851942 -1.900667 -0.055238
C -0.573069 -1.077998 -0.341854
C -0.995545 0.202835 0.063285
C -3.238949 -0.661578 0.317090
C -0.061216 1.253134 0.123480
C 1.285080 1.041027 -0.197466

C 1.699566 -0.232162 -0.595696
C 0.778731 -1.285276 -0.681648
C 3.131938 -0.439973 -0.909013
C 4.163187 0.552159 -0.313775
C 3.552543 1.952108 -0.244983
C 2.227996 2.155737 -0.187534
O 1.159219 -2.543442 -1.082079
O 3.447539 -1.446120 -1.547146
O 5.307747 0.593724 -1.164003
C 4.551261 0.053839 1.091905
C 5.112757 -1.353838 1.061691
C 4.600005 -2.321179 2.093856
O 5.974699 -1.679204 0.245417
O 4.353628 3.078595 -0.393839
C 5.464023 3.169638 0.489393
C -4.673169 -0.511857 0.660881
C -5.242006 -1.653393 1.459958
C -5.371123 0.563607 0.237228
C -6.841911 0.850021 0.451182
C -7.576517 1.056849 -0.887665
C -7.594249 -0.192370 -1.760146
C -6.988100 2.095842 1.328671
H -1.297727 -3.092911 -0.683563
H -2.689743 1.378973 0.721495
H -0.392677 2.247604 0.421102
H 1.832222 3.167879 -0.184596
H 2.067263 -2.492725 -1.461596
H 5.648972 -0.325242 -1.220987
H 3.682083 0.067005 1.760708
H 5.329917 0.672675 1.548707
H 5.085789 -3.291967 1.962684
H 4.826172 -1.943088 3.093748
H 3.521385 -2.448079 1.971738
H 5.149310 3.036200 1.529443
H 6.248995 2.456825 0.224830
H 5.885040 4.175058 0.393760
H -4.539242 -1.970883 2.239297
H -6.166229 -1.388900 1.979515
H -5.450069 -2.510917 0.811374
H -4.862189 1.316438 -0.365416
H -7.334031 0.022271 0.971056
H -8.614789 1.349632 -0.687083
H -7.121357 1.881117 -1.451280
H -8.039296 -1.037502 -1.225393
H -8.188522 -0.011659 -2.661620
H -6.586410 -0.476148 -2.077944
H -6.491923 1.954988 2.295527
H -8.044349 2.308926 1.525780
H -6.550839 2.979727 0.850372
SCF Energy (B3LYP/6-31G**)= -1361.70085870
Number of imaginary frequencies = 0

1a_c345
MMFF Geometry
C -1.619423 -2.333770 -0.339765
C -2.335185 0.265275 0.002850
N -2.937899 -2.052469 -0.201048
C -0.591855 -1.384192 -0.322983
C -0.975070 -0.043241 -0.144505
C -3.280558 -0.757110 -0.036692
C 0.012154 0.957178 -0.116185
C 1.372402 0.645791 -0.265884
C 1.753863 -0.689074 -0.438683
C 0.775468 -1.698885 -0.470280
C 3.192647 -1.021221 -0.605907
C 4.242775 0.116799 -0.587503
C 3.661833 1.501817 -0.386859
C 2.353160 1.727647 -0.220925
O 1.102012 -3.023231 -0.651683
O 3.499574 -2.199038 -0.816504
O 4.871567 0.090544 -1.886936
C 5.338556 -0.227119 0.430313
C 4.857796 -0.195885 1.864855
C 5.919085 -0.057458 2.924298
O 3.672196 -0.325691 2.167532
O 4.537057 2.566254 -0.205167
C 5.243546 2.921930 -1.392437
C -4.730045 -0.491023 0.118066
C -5.414796 -1.389759 1.107340
C -5.313307 0.487221 -0.607573
C -6.758949 0.932565 -0.715866
C -7.011996 2.245177 0.052124
C -6.859092 2.152016 1.564744
C -7.838507 -0.118581 -0.446422
H -1.399014 -3.391215 -0.470002
H -2.654555 1.290957 0.159998
H -0.288489 1.994495 0.028700
H 2.001655 2.734442 -0.012500
H 2.081145 -3.096142 -0.724584
H 4.163413 0.140832 -2.552336
H 6.176372 0.473936 0.328265
H 5.752577 -1.224939 0.238891
H 5.453333 -0.044673 3.913596
H 6.606572 -0.904768 2.866114
H 6.461270 0.880280 2.779725
H 6.104600 2.263756 -1.538392
H 4.591061 2.911219 -2.272366
H 5.621746 3.940406 -1.263075
H -4.712353 -1.780578 1.853143
H -6.173521 -0.851592 1.680364
H -5.877786 -2.242242 0.600631
H -4.674047 1.054925 -1.287345
H -6.885722 1.181572 -1.780709
H -6.324485 3.017333 -0.317094
H -8.023924 2.603516 -0.175214
H -5.845743 1.854840 1.849905
H -7.055851 3.129229 2.018110
H -7.566255 1.439685 1.998522
H -7.622412 -1.050931 -0.979066
H -8.809479 0.242851 -0.804682
H -7.961067 -0.347202 0.614072
SCF Energy (B3LYP/6-31G**)= -1361.69187770
Number of imaginary frequencies = 0

1a_c346
MMFF Geometry
C 1.865452 2.206957 0.117411
C 2.283959 -0.352178 -0.701989
N 3.145723 1.778014 0.006505
C 0.735569 1.429529 -0.158481
C 0.964581 0.106758 -0.584222
C 3.339017 0.504666 -0.396708
C -0.130681 -0.722147 -0.889682
C -1.447050 -0.260177 -0.760021
C -1.667930 1.048097 -0.320893
C -0.588263 1.896961 -0.038136
C -3.066055 1.510348 -0.167584
C -4.168187 0.437686 -0.008915
C -3.835001 -0.797914 -0.838020
C -2.566511 -1.120664 -1.150976
O -0.779017 3.197152 0.363989
O -3.274128 2.724064 -0.117825
O -5.406362 1.001985 -0.455460
C -4.297788 0.155331 1.492391
C -5.152388 -1.052718 1.802785
C -6.648039 -0.904631 1.702450
O -4.634702 -2.127175 2.116419
O -4.951286 -1.526917 -1.204987
C -4.762675 -2.827405 -1.752067
C 4.747566 0.062221 -0.500657
C 5.638652 0.967234 -1.301332
C 5.137473 -1.043226 0.170217
C 6.480950 -1.739957 0.269950
C 7.125648 -1.522133 1.654584
C 7.526318 -0.084858 1.962552
C 7.479058 -1.514269 -0.867435
H 1.766185 3.238406 0.449239
H 2.491978 -1.364240 -1.036140
H 0.051897 -1.739937 -1.233356
H -2.319703 -2.008731 -1.722625
H -1.732698 3.421931 0.264300
H -5.376440 1.019610 -1.428064

H -4.731963 1.014689 2.019011
H -3.314945 -0.019015 1.949765
H -7.130576 -1.753231 2.195827
H -6.964748 0.010331 2.209311
H -6.956011 -0.881621 0.655649
H -4.276332 -2.770693 -2.730919
H -4.193673 -3.463344 -1.066101
H -5.749198 -3.279024 -1.891941
H 6.509891 1.275870 -0.715778
H 5.970831 0.467097 -2.216323
H 5.135062 1.887593 -1.616904
H 4.382119 -1.550193 0.775838
H 6.238707 -2.812124 0.215998
H 6.431642 -1.862123 2.434301
H 8.015343 -2.158751 1.739893
H 6.663099 0.586802 1.945615
H 7.968121 -0.029356 2.963000
H 8.270601 0.287033 1.252953
H 7.013287 -1.702596 -1.840660
H 8.321534 -2.209022 -0.772312
H 7.897836 -0.505414 -0.873531
SCF Energy (B3LYP/6-31G**)= -1361.69380332
Number of imaginary frequencies = 0

1a_c347
MMFF Geometry
C -1.589490 -1.670534 -0.592778
C -2.184314 0.805037 0.352914
N -2.888605 -1.377059 -0.342823
C -0.522144 -0.789924 -0.401487
C -0.842038 0.490012 0.086822
C -3.181337 -0.143819 0.130194
C 0.186920 1.428134 0.290675
C 1.525092 1.105910 0.028337
C 1.836943 -0.173780 -0.441345
C 0.822876 -1.113447 -0.672955
C 3.252494 -0.489660 -0.726293
C 4.358224 0.386449 -0.101695
C 3.867067 1.806302 0.119199
C 2.565832 2.117029 0.213923
O 1.093793 -2.365058 -1.170947
O 3.529949 -1.441616 -1.462996
O 5.435092 0.398955 -1.055345
C 4.886913 -0.178193 1.219353
C 4.972664 -1.685384 1.219525
C 6.244064 -2.316566 0.719378
O 4.012484 -2.365704 1.582657
O 4.805067 2.778109 0.440007
C 5.470689 3.289497 -0.714657
C -4.620249 0.136164 0.412067
C -4.905545 1.127908 1.505208
C -5.566952 -0.525404 -0.290742
C -7.079107 -0.438095 -0.242233
C -7.617505 0.989679 -0.455659
C -9.116833 1.029387 -0.725123
C -7.641967 -1.127111 1.003019
H -1.422253 -2.678313 -0.967004
H -2.441916 1.793723 0.718207
H -0.064222 2.422769 0.657853
H 2.265535 3.131917 0.459307
H 2.052853 -2.426465 -1.376482
H 5.312593 -0.385618 -1.628584
H 4.230820 0.091518 2.057867
H 5.880517 0.221564 1.456567
H 6.212166 -3.394695 0.900230
H 6.351818 -2.140098 -0.352570
H 7.098940 -1.900999 1.258527
H 6.354475 2.687355 -0.941823
H 4.808544 3.346057 -1.585990
H 5.811072 4.302889 -0.481245
H -4.782966 2.152374 1.138618
H -5.914773 1.035832 1.909462
H -4.232216 0.977440 2.356994
H -5.224473 -1.244420 -1.039533
H -7.427318 -1.029501 -1.102042
H -7.412137 1.624820 0.411464
H -7.104171 1.447605 -1.310813
H -9.689360 0.694403 0.144663
H -9.429433 2.053682 -0.952734
H -9.380544 0.398317 -1.579606
H -7.178170 -2.108635 1.153572
H -8.719777 -1.292437 0.904547
H -7.480233 -0.536715 1.909417
SCF Energy (B3LYP/6-31G**)= -1361.69309795
Number of imaginary frequencies = 0

1a_c348
MMFF Geometry
C 2.013340 -1.674616 -0.210086
C 2.261551 1.028171 -0.127840
N 3.261578 -1.148010 -0.179373
C 0.832838 -0.928066 -0.203416
C 0.973046 0.470225 -0.160869
C 3.382419 0.198886 -0.137604
C -0.179053 1.278345 -0.158098
C -1.463294 0.715413 -0.184282
C -1.595839 -0.674460 -0.213101
C -0.457494 -1.494829 -0.238371
C -2.958752 -1.251807 -0.240317
C -4.133761 -0.370456 0.240251
C -3.881501 1.104612 -0.058327
C -2.638233 1.588060 -0.239567
O -0.554136 -2.865015 -0.299994
O -3.077925 -2.426934 -0.598246
O -4.140094 -0.533311 1.665775
C -5.420324 -0.932261 -0.399671
C -6.693067 -0.393794 0.218872
C -7.873485 -0.213486 -0.696825
O -6.777087 -0.166693 1.427684
O -5.032620 1.870258 -0.094542
C -4.911196 3.287186 -0.150350
C 4.772289 0.740984 -0.118090
C 4.971530 2.127297 -0.668739
C 5.777865 -0.028901 0.354048
C 7.243501 0.326035 0.485134
C 8.053370 -0.460414 -0.561104
C 9.528013 -0.083003 -0.595098
C 7.704807 0.036295 1.917121
H 1.986644 -2.761747 -0.241320
H 2.378012 2.105802 -0.080830
H -0.067655 2.361729 -0.134771
H -2.449238 2.637920 -0.432483
H -1.502582 -3.104331 -0.409953
H -5.039794 -0.292132 1.975788
H -5.484446 -2.020547 -0.275264
H -5.423823 -0.724118 -1.476520
H -8.719286 0.189252 -0.132814
H -7.615979 0.490227 -1.492288
H -8.156574 -1.179034 -1.122769
H -4.462435 3.602362 -1.097596
H -5.917809 3.712347 -0.097414
H -4.338845 3.666426 0.702385
H 4.672115 2.880780 0.067106
H 6.008066 2.327377 -0.952447
H 4.383371 2.274472 -1.581917
H 5.536719 -1.035827 0.702141
H 7.396669 1.396444 0.314374
H 7.966360 -1.539219 -0.379036
H 7.632474 -0.276134 -1.558185
H 10.037519 -0.368748 0.329636
H 10.028048 -0.600090 -1.420487
H 9.654917 0.993842 -0.744265
H 7.061701 0.543038 2.645887
H 8.725771 0.393978 2.083636
H 7.682500 -1.036920 2.137589
SCF Energy (B3LYP/6-31G**)= -1361.71170752
Number of imaginary frequencies = 0

1a_c349
MMFF Geometry
C -1.474528 -2.140595 -0.689604
C -2.360784 0.321802 0.048094

N -2.808343 -1.970315 -0.521525
C -0.512396 -1.140867 -0.512335
C -0.983171 0.129939 -0.129154
C -3.236154 -0.741664 -0.161108
C -0.066055 1.179636 0.062330
C 1.309493 0.979864 -0.111644
C 1.771688 -0.289019 -0.478397
C 0.870810 -1.340609 -0.695499
C 3.226117 -0.476134 -0.667745
C 4.196668 0.536043 -0.026935
C 3.562425 1.911787 0.048796
C 2.234151 2.103186 0.048224
O 1.292734 -2.586329 -1.092765
O 3.639726 -1.431259 -1.332115
O 5.347831 0.604127 -0.886541
C 4.659222 0.110699 1.368525
C 4.905140 -1.375120 1.471217
C 6.274044 -1.887454 1.112587
O 3.995212 -2.137500 1.799366
O 4.418572 2.973061 0.301507
C 4.705386 3.699913 -0.893799
C -4.700227 -0.598266 0.016713
C -5.325006 -1.683115 0.846234
C -5.346393 0.440711 -0.554865
C -6.818394 0.801724 -0.609385
C -7.159137 1.959322 0.350121
C -7.002191 1.642013 1.831595
C -7.825934 -0.347023 -0.518875
H -1.185108 -3.147786 -0.982298
H -2.748662 1.287377 0.357969
H -0.435251 2.164001 0.348374
H 1.823026 3.098692 0.188287
H 2.262842 -2.561105 -1.248113
H 5.338751 -0.208228 -1.432652
H 3.904148 0.351266 2.128895
H 5.578985 0.631157 1.662521
H 6.346681 -2.947300 1.372498
H 6.446476 -1.772455 0.040734
H 7.032649 -1.340834 1.678202
H 5.150088 3.057559 -1.660180
H 3.801896 4.181251 -1.283078
H 5.427231 4.483300 -0.644960
H -4.598973 -2.138850 1.530024
H -6.118269 -1.291585 1.487394
H -5.730010 -2.474463 0.207813
H -4.745734 1.147788 -1.131311
H -6.960193 1.204621 -1.624045
H -6.524081 2.822922 0.113233
H -8.192390 2.281588 0.169513
H -5.971662 1.371112 2.078831
H -7.264069 2.521704 2.428893
H -7.660920 0.826043 2.141316
H -7.547684 -1.168854 -1.187345
H -8.818392 0.001365 -0.827779
H -7.934216 -0.745537 0.491767
SCF Energy (B3LYP/6-31G**)= -1361.69092789
Number of imaginary frequencies = 0

1a_c350
MMFF Geometry
C -2.019334 -1.479455 -0.749415
C -2.290532 1.168888 -0.214287
N -3.271584 -0.973084 -0.644521
C -0.845032 -0.737920 -0.599512
C -0.997329 0.631347 -0.321743
C -3.404490 0.346839 -0.379185
C 0.148254 1.432234 -0.160600
C 1.438440 0.890399 -0.262929
C 1.583272 -0.471842 -0.533244
C 0.450750 -1.282340 -0.711851
C 2.945861 -1.036360 -0.638836
C 4.148869 -0.207905 -0.150895
C 3.853538 1.280943 -0.062455
C 2.600663 1.767847 -0.113649
O 0.556109 -2.623369 -0.995556
O 3.081885 -2.177821 -1.090579
O 5.147613 -0.409105 -1.162883
C 4.601241 -0.763078 1.209301
C 6.080610 -1.062290 1.280846
C 7.033765 0.088836 1.455861
O 6.491614 -2.219324 1.171826
O 4.990205 2.052065 0.103700
C 4.877355 3.467362 0.016931
C -4.799624 0.869449 -0.288970
C -5.000051 2.324514 -0.616891
C -5.798483 0.025782 0.054524
C -7.271940 0.326281 0.218594
C -7.736367 0.143230 1.678580
C -7.662870 -1.276260 2.228025
C -8.087544 -0.520152 -0.761724
H -1.984356 -2.544979 -0.966572
H -2.416682 2.222775 0.010634
H 0.025726 2.494976 0.046872
H 2.395992 2.830006 -0.035132
H 1.502983 -2.841795 -1.153982
H 5.200472 -1.374750 -1.317379
H 4.082935 -1.703452 1.442025
H 4.338649 -0.095369 2.039605
H 7.207127 0.578385 0.495714
H 6.630204 0.801928 2.178992
H 7.985676 -0.285087 1.843741
H 4.454361 3.769829 -0.946489
H 4.284520 3.861981 0.848081
H 5.883724 3.889784 0.091765
H -4.410663 2.611734 -1.495376
H -6.034860 2.571100 -0.864795
H -4.700066 2.953790 0.227553
H -5.538943 -1.016769 0.249050
H -7.475482 1.372583 -0.028994
H -7.143001 0.799198 2.328633
H -8.774943 0.488277 1.761735
H -6.638631 -1.659544 2.225724
H -8.017023 -1.290554 3.264243
H -8.293944 -1.962356 1.656003
H -7.830692 -0.266304 -1.796591
H -9.159879 -0.339017 -0.632081
H -7.905655 -1.592435 -0.632259
SCF Energy (B3LYP/6-31G**)= -1361.70280505
Number of imaginary frequencies = 0

1a_c351
MMFF Geometry
C -1.997112 -1.732095 -0.186225
C -2.274162 0.968852 -0.230991
N -3.246876 -1.219590 -0.294796
C -0.829000 -0.971819 -0.093382
C -0.984336 0.425393 -0.115025
C -3.381593 0.126369 -0.319736
C 0.153666 1.246809 -0.012201
C 1.439931 0.698483 0.095706
C 1.588783 -0.690041 0.100873
C 0.462825 -1.524089 0.023163
C 2.953754 -1.251740 0.214706
C 4.148605 -0.344883 -0.157226
C 3.855328 1.118999 0.158508
C 2.595672 1.583133 0.259248
O 0.573097 -2.894116 0.060678
O 3.062416 -2.433804 0.552541
O 4.260460 -0.471088 -1.582107
C 5.392848 -0.907863 0.560586
C 6.699657 -0.338780 0.049903
C 7.808081 -0.168365 1.053273
O 6.867943 -0.079615 -1.143573
O 4.990259 1.896686 0.298920
C 4.845916 3.310060 0.383066
C -4.770550 0.652664 -0.458974
C -4.924268 2.007919 -1.094887
C -5.810624 -0.105113 -0.045765
C -7.285326 0.235471 -0.071612
C -7.876055 0.047882 1.339680
C -9.328092 0.492173 1.453143
C -7.983641 -0.638978 -1.115065

H -1.958322 -2.819265 -0.176022
H -2.403855 2.045992 -0.237290
H 0.029624 2.329056 -0.017549
H 2.378753 2.625368 0.463800
H 1.514168 -3.124822 0.234091
H 5.176984 -0.211694 -1.819682
H 5.480536 -1.991765 0.414209
H 5.315441 -0.727452 1.639659
H 8.686988 0.258662 0.562504
H 7.484116 0.511522 1.845158
H 8.072617 -1.141207 1.474522
H 4.332747 3.705855 -0.499298
H 4.324495 3.594007 1.302574
H 5.847857 3.748092 0.415880
H -4.248549 2.118069 -1.950917
H -5.928449 2.181200 -1.490188
H -4.705730 2.799908 -0.371104
H -5.595219 -1.090038 0.374699
H -7.434476 1.282620 -0.352868
H -7.801500 -1.002612 1.648660
H -7.284843 0.629094 2.059400
H -9.990209 -0.141691 0.856357
H -9.659388 0.426843 2.494615
H -9.449087 1.529065 1.124302
H -7.502353 -0.539769 -2.094841
H -9.031115 -0.347218 -1.239526
H -7.958351 -1.697826 -0.833845
SCF Energy (B3LYP/6-31G**)= -1361.71175022
Number of imaginary frequencies = 0

1a_c352
MMFF Geometry
C 1.938999 1.843492 0.013564
C 2.176806 -0.849180 -0.269238
N 3.179551 1.303161 0.078104
C 0.762266 1.116731 -0.187508
C 0.897121 -0.276752 -0.332472
C 3.290736 -0.036967 -0.064646
C -0.250135 -1.064165 -0.543558
C -1.525966 -0.487219 -0.596706
C -1.653569 0.895223 -0.437232
C -0.519608 1.698544 -0.251336
C -3.011562 1.483794 -0.473080
C -4.210823 0.551448 -0.185142
C -3.944652 -0.846827 -0.732049
C -2.694867 -1.321084 -0.887956
O -0.615701 3.063325 -0.122290
O -3.114105 2.695295 -0.674642
O -5.368951 1.107427 -0.818028
C -4.444137 0.585913 1.329724
C -5.418264 -0.466244 1.809452
C -6.887381 -0.226363 1.578984
O -5.015750 -1.493152 2.361047
O -5.100013 -1.547766 -1.025368
C -4.998497 -2.942493 -1.290977
C 4.667447 -0.600213 -0.002877
C 4.962748 -1.728141 -0.949019
C 5.557094 -0.043192 0.847773
C 6.989327 -0.394663 1.195970
C 7.996607 0.568978 0.537934
C 8.057908 0.506868 -0.982548
C 7.402885 -1.861752 1.053876
H 1.914936 2.924530 0.133853
H 2.298199 -1.923701 -0.364350
H -0.140749 -2.141054 -0.668709
H -2.497783 -2.321826 -1.256737
H -1.539020 3.335997 -0.329853
H -5.285850 0.928289 -1.770998
H -4.827181 1.564036 1.647350
H -3.506258 0.431496 1.879365
H -7.468985 -0.918908 2.194210
H -7.147248 0.793330 1.874049
H -7.136732 -0.390162 0.529142
H -4.458409 -3.121965 -2.225850
H -4.524565 -3.467975 -0.455519
H -6.012332 -3.336785 -1.406840
H 4.336477 -1.675977 -1.847241
H 5.989539 -1.689674 -1.318786
H 4.789790 -2.694993 -0.466216
H 5.206653 0.811238 1.434636
H 7.057467 -0.201142 2.277661
H 7.754563 1.598811 0.831313
H 8.998653 0.365719 0.936304
H 7.095275 0.761403 -1.435330
H 8.796678 1.225111 -1.353440
H 8.357529 -0.484271 -1.333985
H 6.651640 -2.527676 1.491959
H 8.344192 -2.037333 1.587676
H 7.569924 -2.164081 0.018249
SCF Energy (B3LYP/6-31G**)= -1361.69401044
Number of imaginary frequencies = 0

1a_c353
MMFF Geometry
C 1.882990 1.848173 -0.088219
C 2.188663 -0.847418 -0.224806
N 3.139303 1.344301 -0.033934
C 0.721205 1.081789 -0.209137
C 0.891501 -0.312898 -0.279036
C 3.287902 0.001269 -0.102434
C -0.239127 -1.140674 -0.406621
C -1.531427 -0.601337 -0.453276
C -1.695353 0.784562 -0.369691
C -0.578367 1.625726 -0.263942
C -3.068673 1.338093 -0.403250
C -4.264417 0.402576 -0.131778
C -3.935274 -1.018550 -0.536779
C -2.681423 -1.483667 -0.638835
O -0.704922 2.993070 -0.206938
O -3.228275 2.546969 -0.601649
O -5.339098 0.902019 -0.943142
C -4.630672 0.571457 1.346178
C -5.646542 -0.424596 1.860298
C -7.066274 -0.308324 1.369098
O -5.324320 -1.277660 2.689801
O -5.009231 -1.881023 -0.691477
C -5.325259 -2.078409 -2.069954
C 4.686029 -0.516566 -0.055398
C 4.951060 -1.796740 -0.797495
C 5.627710 0.193904 0.604805
C 7.095921 -0.095595 0.844064
C 7.940824 0.263238 -0.391621
C 9.441006 0.189499 -0.140456
C 7.368110 -1.501076 1.389015
H 1.833800 2.933326 -0.028766
H 2.330092 -1.922711 -0.263918
H -0.105155 -2.220269 -0.471177
H -2.502355 -2.531122 -0.864350
H -1.642132 3.233745 -0.382087
H -5.235722 1.874732 -0.974145
H -5.035062 1.573678 1.537961
H -3.736684 0.474236 1.976833
H -7.711697 -0.957730 1.967666
H -7.412091 0.721833 1.486043
H -7.138015 -0.612764 0.324086
H -5.537301 -1.130184 -2.573916
H -4.514994 -2.606203 -2.583896
H -6.223360 -2.700728 -2.122276
H 6.000545 -1.912707 -1.074654
H 4.659828 -2.661432 -0.192368
H 4.395241 -1.827378 -1.741815
H 5.314806 1.131465 1.071517
H 7.396349 0.591716 1.648855
H 7.694914 -0.394610 -1.232865
H 7.697090 1.284045 -0.713510
H 9.763351 -0.836336 0.061115
H 9.986540 0.540466 -1.022405
H 9.729197 0.818181 0.707865
H 6.628786 -1.783375 2.146733
H 8.351888 -1.541332 1.869541
H 7.363117 -2.262262 0.604324
SCF Energy (B3LYP/6-31G**)= -1361.69584095

Number of imaginary frequencies = 0

1a_c354
MMFF Geometry
C -1.863585 -1.247098 -1.080315
C -2.094466 1.225939 0.015631
N -3.107873 -0.750003 -0.880533
C -0.677776 -0.580416 -0.762251
C -0.810016 0.698064 -0.194853
C -3.220868 0.483155 -0.335162
C 0.346625 1.418904 0.151137
C 1.628009 0.888669 -0.060699
C 1.757639 -0.383065 -0.625852
C 0.610328 -1.115297 -0.975477
C 3.113946 -0.947331 -0.837009
C 4.360252 -0.119000 -0.469501
C 4.042393 1.237608 0.115110
C 2.795016 1.674173 0.327084
O 0.689136 -2.371506 -1.529609
O 3.217052 -2.071098 -1.340812
O 4.988199 0.090701 -1.755028
C 5.318568 -0.961894 0.384829
C 4.944235 -1.064843 1.848727
C 3.711069 -1.847816 2.214660
O 5.632900 -0.537769 2.722821
O 5.090229 2.015954 0.587270
C 5.940215 2.501250 -0.450433
C -4.607705 1.002189 -0.146499
C -4.775496 2.497890 -0.138710
C -5.627886 0.124999 -0.014628
C -7.098057 0.412876 0.195365
C -7.588814 -0.080232 1.572581
C -7.549094 -1.587525 1.793343
C -7.913282 -0.177031 -0.957976
H -1.845560 -2.240654 -1.523066
H -2.203414 2.207760 0.464628
H 0.242485 2.409920 0.592988
H 2.637400 2.638951 0.802404
H 1.635701 -2.608921 -1.650041
H 5.112812 -0.790733 -2.156694
H 6.331630 -0.540853 0.331351
H 5.422816 -1.980861 -0.008571
H 3.781793 -2.166959 3.258591
H 2.823722 -1.224655 2.093616
H 3.636527 -2.743243 1.592734
H 6.650811 1.729467 -0.759246
H 5.364935 2.867752 -1.307671
H 6.515362 3.339327 -0.045527
H -4.170090 2.962834 -0.925422
H -5.802084 2.817062 -0.331686
H -4.473349 2.913619 0.828226
H -5.391282 -0.939991 -0.056844
H -7.278804 1.492043 0.184265
H -6.994345 0.402472 2.359060
H -8.622318 0.259201 1.718394
H -6.531857 -1.981845 1.717662
H -7.919831 -1.824330 2.796265
H -8.183010 -2.116167 1.075793
H -7.635376 0.294825 -1.907440
H -8.984057 -0.006356 -0.803595
H -7.753131 -1.254918 -1.068180
SCF Energy (B3LYP/6-31G**)= -1361.69731769
Number of imaginary frequencies = 0

1a_c355
MMFF Geometry
C 1.899897 -2.429074 -0.001781
C 2.525975 0.141385 -0.621240
N 3.208432 -2.114737 -0.162003
C 0.839392 -1.526990 -0.132546
C 1.175349 -0.199817 -0.456857
C 3.509569 -0.832607 -0.458717
C 0.151928 0.752160 -0.617445
C -1.196032 0.406758 -0.443941
C -1.522981 -0.908393 -0.106237
C -0.516130 -1.876233 0.033715
C -2.951008 -1.255094 0.073330
C -3.949078 -0.110061 0.358224
C -3.518443 1.185720 -0.323010
C -2.238433 1.409297 -0.674945
O -0.808268 -3.186660 0.330524
O -3.261459 -2.449046 0.036557
O -3.863085 0.091603 1.776196
C -5.348796 -0.606932 -0.059215
C -6.479851 0.260358 0.451601
C -7.693294 0.387788 -0.429144
O -6.433741 0.792405 1.562758
O -4.550780 2.087021 -0.508867
C -4.237978 3.407754 -0.937119
C 4.952016 -0.528558 -0.627968
C 5.720553 -1.584058 -1.377558
C 5.477703 0.594529 -0.092760
C 6.915078 1.064492 -0.152017
C 7.657804 0.807275 1.173943
C 7.116148 1.550537 2.389019
C 6.965392 2.537635 -0.569150
H 1.717055 -3.472765 0.245261
H 2.807540 1.155557 -0.887204
H 0.415568 1.775927 -0.880288
H -1.920813 2.334895 -1.141388
H -1.786664 -3.294718 0.326661
H -4.692294 0.539719 2.050689
H -5.553864 -1.605845 0.345850
H -5.410072 -0.676847 -1.152053
H -8.426326 1.045297 0.046282
H -7.404802 0.821512 -1.389840
H -8.141935 -0.597287 -0.578139
H -3.551822 3.895603 -0.237292
H -3.827150 3.400941 -1.951601
H -5.168009 3.983565 -0.954992
H 5.234080 -1.803255 -2.334950
H 6.746926 -1.292288 -1.608401
H 5.774743 -2.510953 -0.796096
H 4.823257 1.250801 0.481648
H 7.458389 0.515181 -0.927534
H 7.643246 -0.268812 1.390108
H 8.713157 1.079615 1.046047
H 6.076339 1.282112 2.596903
H 7.706848 1.293005 3.274463
H 7.176960 2.634586 2.257261
H 6.541912 2.669392 -1.571494
H 7.999368 2.897877 -0.595623
H 6.399308 3.180542 0.113295
SCF Energy (B3LYP/6-31G**)= -1361.71038845
Number of imaginary frequencies = 0

1a_c356
MMFF Geometry
C 1.691049 -1.996258 0.186400
C 2.405543 0.621379 0.364987
N 3.005723 -1.707365 0.342070
C 0.667104 -1.045976 0.110373
C 1.049047 0.306188 0.203857
C 3.346142 -0.404237 0.427452
C 0.066132 1.310092 0.126826
C -1.289061 0.987348 -0.026056
C -1.662796 -0.355854 -0.102189
C -0.694631 -1.369266 -0.054199
C -3.097722 -0.670874 -0.264570
C -4.128946 0.382893 0.207512
C -3.603865 1.792912 -0.079435
C -2.284884 2.052179 -0.167843
O -1.032290 -2.696940 -0.166665
O -3.398112 -1.773206 -0.728289
O -4.272956 0.307915 1.629332
C -5.478181 0.131512 -0.496875
C -6.093383 -1.198070 -0.106161
C -6.913010 -1.906632 -1.150033
O -5.973527 -1.647880 1.035775
O -4.601319 2.747769 -0.186776
C -4.228370 4.118932 -0.256443
C 4.789059 -0.119018 0.592507

C 5.458271 -0.866945 1.709167
C 5.398224 0.705418 -0.286858
C 6.833259 1.185074 -0.390184
C 7.564150 0.520391 -1.575202
C 7.774783 -0.982806 -1.444163
C 7.677305 1.165804 0.885761
H 1.470888 -3.059827 0.122294
H 2.728739 1.654453 0.450217
H 0.366736 2.355337 0.187867
H -1.901770 3.051190 -0.340887
H -1.986307 -2.758710 -0.398566
H -4.777621 -0.512214 1.823102
H -5.346611 0.154989 -1.585322
H -6.225356 0.885954 -0.222895
H -7.285683 -2.852125 -0.746528
H -7.762401 -1.280922 -1.434391
H -6.290777 -2.119167 -2.022869
H -3.652420 4.414844 0.626350
H -3.672720 4.325492 -1.176621
H -5.145051 4.715729 -0.276538
H 4.797297 -1.600047 2.184504
H 6.325443 -1.423994 1.341899
H 5.773474 -0.177087 2.498113
H 4.784832 1.111250 -1.095065
H 6.748213 2.251375 -0.648844
H 7.003265 0.711554 -2.499356
H 8.542200 0.999969 -1.707646
H 6.823959 -1.517752 -1.364601
H 8.294287 -1.363029 -2.330003
H 8.386957 -1.229612 -0.572314
H 7.156226 1.667887 1.707934
H 8.618260 1.703889 0.722776
H 7.940192 0.155761 1.208094
SCF Energy (B3LYP/6-31G**)= -1361.70353218
Number of imaginary frequencies = 0

1a_c357
MMFF Geometry
C -1.632816 -1.710314 -0.531498
C -2.213734 0.804285 0.317408
N -2.923238 -1.416189 -0.240355
C -0.568180 -0.811998 -0.429112
C -0.880637 0.488593 0.010470
C -3.208248 -0.163765 0.184084
C 0.144447 1.446846 0.120923
C 1.472231 1.120068 -0.177990
C 1.777249 -0.179948 -0.596394
C 0.766421 -1.137499 -0.743958
C 3.191561 -0.496207 -0.900564
C 4.281073 0.369455 -0.215295
C 3.810932 1.819716 -0.170985
C 2.511142 2.150357 -0.147641
O 1.032575 -2.405322 -1.200148
O 3.441747 -1.444806 -1.645158
O 5.494912 0.295933 -0.968043
C 4.579105 -0.160614 1.192121
C 4.612525 -1.671647 1.244151
C 5.846896 -2.370921 0.740320
O 3.638090 -2.299271 1.661258
O 4.740917 2.839066 -0.320311
C 5.681619 2.919587 0.747032
C -4.636171 0.118339 0.511923
C -4.885839 1.144272 1.581598
C -5.604900 -0.570054 -0.132299
C -7.113042 -0.483076 -0.031652
C -7.725727 0.922031 -0.191297
C -7.326245 1.611730 -1.489727
C -7.627039 -1.176276 1.232754
H -1.470352 -2.733668 -0.863278
H -2.468084 1.807434 0.643496
H -0.101986 2.459438 0.438206
H 2.206903 3.193324 -0.158642
H 1.978240 -2.456226 -1.465093
H 5.308837 0.695043 -1.837885
H 3.813519 0.163751 1.909105
H 5.541048 0.199256 1.573855
H 5.822394 -3.419687 1.049603
H 5.886783 -2.322361 -0.349542
H 6.737340 -1.906912 1.171711
H 5.188895 2.842601 1.722242
H 6.454827 2.153731 0.642542
H 6.170153 3.896831 0.689849
H -5.869304 1.042812 2.043576
H -4.164963 1.041554 2.400962
H -4.807275 2.156451 1.171504
H -5.287230 -1.314277 -0.867236
H -7.497633 -1.073558 -0.876649
H -8.819560 0.830093 -0.179497
H -7.470748 1.570266 0.652316
H -7.578311 0.992765 -2.356653
H -7.859487 2.562848 -1.587927
H -6.254345 1.828447 -1.518422
H -7.227561 -2.193465 1.313775
H -8.719820 -1.251882 1.211697
H -7.351279 -0.633831 2.141866
SCF Energy (B3LYP/6-31G**)= -1361.68802547
Number of imaginary frequencies = 0

1a_c358
MMFF Geometry
C -1.581608 -1.793276 -0.971715
C -2.249573 0.777835 -0.390550
N -2.898259 -1.479683 -0.905615
C -0.533355 -0.890260 -0.764251
C -0.891354 0.438710 -0.465675
C -3.215327 -0.201447 -0.611707
C 0.117092 1.398035 -0.258051
C 1.473088 1.051318 -0.327509
C 1.819386 -0.273563 -0.607792
C 0.829934 -1.237705 -0.843883
C 3.253057 -0.613102 -0.685323
C 4.269763 0.316577 0.004966
C 3.801155 1.766768 -0.015404
C 2.500525 2.085135 -0.161049
O 1.145087 -2.536358 -1.162395
O 3.609784 -1.627163 -1.292054
O 5.495797 0.217618 -0.742721
C 4.558816 -0.099573 1.449235
C 4.637313 -1.597927 1.615391
C 5.960085 -2.267185 1.355481
O 3.632706 -2.245384 1.913606
O 4.823986 2.677951 0.177975
C 4.528490 4.066233 0.094402
C -4.660503 0.109575 -0.538320
C -5.463871 -0.328312 -1.728667
C -5.151778 0.675488 0.585277
C -6.555681 1.104084 0.966650
C -7.169111 0.151775 2.014076
C -7.427400 -1.267754 1.524826
C -7.533191 1.410164 -0.169835
H -1.380877 -2.836702 -1.206003
H -2.556269 1.796084 -0.171325
H -0.165955 2.427689 -0.042114
H 2.154760 3.112902 -0.150314
H 2.120169 -2.614630 -1.258053
H 5.405739 -0.553505 -1.337724
H 3.768216 0.246513 2.128419
H 5.498593 0.333883 1.812875
H 5.911189 -3.310049 1.681314
H 6.189550 -2.238736 0.288543
H 6.746507 -1.765404 1.924657
H 4.118501 4.321736 -0.888044
H 3.846286 4.368488 0.895303
H 5.464259 4.618044 0.223508
H -4.875209 -0.909133 -2.447266
H -6.298282 -0.966953 -1.423575
H -5.847401 0.540105 -2.273138
H -4.445560 0.861145 1.398356
H -6.418462 2.067873 1.479887
H -6.507173 0.100955 2.888405
H -8.115712 0.575330 2.372778
H -6.503409 -1.758613 1.205669

H -7.856578 -1.866788 2.334845
H -8.135927 -1.284487 0.692059
H -7.092842 2.110636 -0.887477
H -8.438355 1.882375 0.229151
H -7.852542 0.516148 -0.710161
SCF Energy (B3LYP/6-31G**)= -1361.69574874
Number of imaginary frequencies = 0

1a_c359
MMFF Geometry
C 1.788929 -1.678578 -0.140491
C 2.319336 0.985076 -0.045192
N 3.085695 -1.288872 -0.091891
C 0.694246 -0.809567 -0.146286
C 0.980719 0.567502 -0.099627
C 3.341716 0.037916 -0.040520
C -0.078016 1.494502 -0.117748
C -1.413058 1.070275 -0.162720
C -1.690080 -0.297894 -0.190136
C -0.647905 -1.235916 -0.201917
C -3.105562 -0.720606 -0.237949
C -4.174110 0.264238 0.295935
C -3.779056 1.702571 -0.052519
C -2.493444 2.056015 -0.245038
O -0.892370 -2.586760 -0.270822
O -3.356818 -1.851158 -0.661132
O -4.203988 0.209157 1.725629
C -5.550210 -0.100429 -0.298292
C -6.032597 -1.463109 0.159199
C -6.873503 -2.252002 -0.807257
O -5.793156 -1.878589 1.295315
O -4.850443 2.578983 -0.099031
C -4.587680 3.971833 -0.221205
C 4.774408 0.436380 0.029764
C 5.091583 1.603625 0.919482
C 5.682855 -0.289969 -0.657574
C 7.178049 -0.133290 -0.846427
C 7.976109 -1.141778 0.003454
C 7.865834 -0.952856 1.510650
C 7.747929 1.285028 -0.761713
H 1.646457 -2.756572 -0.175621
H 2.559009 2.043450 -0.021548
H 0.146778 2.560134 -0.095540
H -2.200138 3.076462 -0.462713
H -1.853929 -2.723313 -0.427258
H -4.629581 -0.641604 1.970464
H -5.503677 -0.090320 -1.393878
H -6.328870 0.602653 0.020901
H -7.142548 -3.213615 -0.361642
H -7.786536 -1.696667 -1.034876
H -6.305019 -2.436572 -1.722106
H -3.971037 4.329710 0.609625
H -4.119577 4.197180 -1.184659
H -5.545464 4.499054 -0.181688
H 4.361829 1.702298 1.731558
H 6.055011 1.483029 1.419283
H 5.096632 2.536998 0.347987
H 5.301764 -1.148988 -1.218015
H 7.348396 -0.427960 -1.893465
H 7.646324 -2.159887 -0.241311
H 9.035209 -1.088016 -0.278548
H 6.832942 -1.056962 1.855262
H 8.462840 -1.713741 2.024316
H 8.240849 0.025631 1.823004
H 7.140346 1.989555 -1.339836
H 8.759843 1.306230 -1.182909
H 7.827543 1.657075 0.261554
SCF Energy (B3LYP/6-31G**)= -1361.70381121
Number of imaginary frequencies = 0

1a_c360
MMFF Geometry
C 1.744198 -1.998051 -0.003602
C 2.453239 0.550986 -0.628750
N 3.063258 -1.721983 -0.143194
C 0.712042 -1.066731 -0.157296
C 1.091567 0.249263 -0.479935
C 3.403620 -0.452503 -0.457642
C 0.099371 1.231301 -0.649331
C -1.259674 0.925023 -0.492488
C -1.634033 -0.380010 -0.164396
C -0.656062 -1.375842 -0.009864
C -3.075766 -0.692963 -0.017761
C -4.082079 0.465742 0.204721
C -3.544548 1.768621 -0.372856
C -2.263137 1.960785 -0.712590
O -0.987080 -2.677316 0.286281
O -3.409339 -1.881166 -0.038142
O -4.230423 0.685302 1.609772
C -5.424433 0.094234 -0.459861
C -6.105623 -1.076996 0.220328
C -6.879720 -2.024063 -0.655577
O -6.071071 -1.211604 1.445509
O -4.451215 2.792594 -0.624651
C -4.843903 3.451067 0.582088
C 4.854103 -0.179468 -0.607495
C 5.265534 0.583276 -1.832038
C 5.680498 -0.613310 0.368639
C 7.180955 -0.515488 0.559002
C 7.525780 0.447155 1.715744
C 7.171054 1.907990 1.465296
C 8.040718 -0.240405 -0.675348
H 1.528257 -3.034436 0.247267
H 2.770275 1.562907 -0.861360
H 0.394303 2.248407 -0.905621
H -1.948486 2.908863 -1.138946
H -1.966807 -2.764789 0.263877
H -4.749769 -0.070650 1.963976
H -5.265826 -0.152662 -1.516710
H -6.147170 0.916880 -0.409296
H -7.306198 -2.824935 -0.045358
H -7.689720 -1.483760 -1.151368
H -6.210822 -2.467113 -1.397520
H -5.560829 2.842417 1.140742
H -3.978630 3.700147 1.206280
H -5.342547 4.384494 0.304526
H 5.714643 1.542877 -1.557275
H 5.973539 0.005091 -2.432534
H 4.422971 0.800203 -2.497591
H 5.210081 -1.144726 1.202094
H 7.485917 -1.517221 0.896744
H 7.011468 0.117270 2.627829
H 8.599741 0.380150 1.930821
H 6.099136 2.038899 1.290611
H 7.439582 2.509960 2.339741
H 7.714817 2.313060 0.607289
H 7.843072 -0.977191 -1.461068
H 9.104006 -0.319677 -0.421263
H 7.883004 0.759261 -1.087597
SCF Energy (B3LYP/6-31G**)= -1361.69640568
Number of imaginary frequencies = 0

1a_c361
MMFF Geometry
C -1.704170 -1.512682 -0.513859
C -2.151396 1.117190 0.001113
N -2.982268 -1.108554 -0.315043
C -0.588116 -0.673874 -0.474445
C -0.831142 0.686677 -0.205737
C -3.201182 0.201569 -0.058396
C 0.249859 1.587035 -0.160291
C 1.563672 1.146951 -0.358722
C 1.798219 -0.209656 -0.609191
C 0.733095 -1.114927 -0.689124
C 3.199154 -0.645172 -0.810385
C 4.316490 0.227632 -0.181313
C 3.941358 1.698838 -0.326629
C 2.666187 2.108419 -0.400827
O 0.932604 -2.442115 -0.980778
O 3.414396 -1.688703 -1.428088
O 5.550554 -0.008170 -0.864617
C 4.525316 -0.149314 1.290055

C 4.458797 -1.642937 1.517613
C 5.663740 -2.469377 1.154818
O 3.430439 -2.157608 1.959213
O 4.941372 2.634822 -0.550613
C 5.843824 2.784067 0.542067
C -4.622524 0.611650 0.137840
C -4.974766 2.015569 -0.267290
C -5.503149 -0.290943 0.624722
C -6.977958 -0.161968 0.941857
C -7.886206 -0.149238 -0.301666
C -7.730862 -1.386875 -1.176994
C -7.299302 1.004601 1.881454
H -1.596611 -2.576677 -0.714012
H -2.348718 2.161364 0.220266
H 0.058703 2.643193 0.026064
H 2.431868 3.159329 -0.546188
H 1.882035 -2.581506 -1.195751
H 5.424227 0.295633 -1.782371
H 3.755399 0.302699 1.929043
H 5.493197 0.194883 1.671218
H 5.560048 -3.470581 1.582608
H 5.747849 -2.553215 0.069622
H 6.565284 -2.011208 1.569055
H 5.310178 2.853381 1.496090
H 6.568455 1.965655 0.562507
H 6.397352 3.716120 0.394342
H -4.476791 2.290448 -1.204321
H -6.041797 2.148221 -0.452958
H -4.676928 2.727705 0.509263
H -5.123215 -1.290641 0.851311
H -7.235321 -1.068133 1.510319
H -8.932808 -0.089956 0.022968
H -7.701958 0.741233 -0.912077
H -7.910236 -2.300130 -0.600816
H -8.454154 -1.358153 -1.998331
H -6.730852 -1.445307 -1.617344
H -6.618396 1.019805 2.739592
H -8.318589 0.905062 2.271677
H -7.240901 1.975439 1.382594
SCF Energy (B3LYP/6-31G**)= -1361.68806629
Number of imaginary frequencies = 0

1a_c362
MMFF Geometry
C 1.841690 1.950752 -0.031453
C 2.216851 -0.736217 0.097188
N 3.105031 1.488898 0.129354
C 0.705712 1.144664 -0.136797
C 0.912150 -0.245248 -0.071222
C 3.288775 0.149934 0.194874
C -0.190209 -1.112565 -0.182672
C -1.490575 -0.616058 -0.342663
C -1.691355 0.766648 -0.391302
C -0.601606 1.644932 -0.305465
C -3.073723 1.276078 -0.543195
C -4.259477 0.334732 -0.248948
C -3.876133 -1.106515 -0.509507
C -2.608018 -1.543295 -0.506928
O -0.763003 3.007816 -0.378765
O -3.251307 2.457716 -0.856856
O -5.302606 0.730690 -1.153235
C -4.705302 0.621930 1.188339
C -5.720393 -0.355139 1.739068
C -7.115991 -0.323951 1.171825
O -5.419009 -1.122887 2.655236
O -4.919853 -2.009031 -0.641009
C -5.158202 -2.334693 -2.010751
C 4.691809 -0.320124 0.391487
C 4.863034 -1.624671 1.118860
C 5.709972 0.449194 -0.055036
C 7.208846 0.228091 -0.018265
C 7.648400 -1.099034 -0.665942
C 9.149778 -1.174196 -0.915702
C 7.774189 0.442513 1.387659
H 1.764367 3.035043 -0.074327
H 2.386954 -1.807227 0.136921
H -0.027589 -2.189465 -0.144152
H -2.392606 -2.600938 -0.629706
H -1.695251 3.205559 -0.621199
H -5.220319 1.699338 -1.266220
H -5.144382 1.624584 1.269854
H -3.842733 0.607321 1.868161
H -7.773781 -0.939463 1.792338
H -7.493884 0.701508 1.182670
H -7.126852 -0.717981 0.154721
H -5.365523 -1.440345 -2.606768
H -4.309898 -2.882501 -2.434446
H -6.037517 -2.984026 -2.052906
H 4.678444 -2.468756 0.446257
H 5.860898 -1.746910 1.542810
H 4.172118 -1.694550 1.966978
H 5.449603 1.391994 -0.543073
H 7.631334 1.029371 -0.642562
H 7.364556 -1.955669 -0.047002
H 7.135142 -1.221281 -1.628292
H 9.712426 -1.177670 0.022354
H 9.394500 -2.096836 -1.451940
H 9.490991 -0.330143 -1.523224
H 7.378285 1.361157 1.835447
H 8.864118 0.542346 1.359432
H 7.536270 -0.387747 2.058727
SCF Energy (B3LYP/6-31G**)= -1361.69613344
Number of imaginary frequencies = 0

1a_c363
MMFF Geometry
C 2.107478 -1.558457 -0.152135
C 2.287927 1.150862 -0.132443
N 3.342008 -1.000111 -0.130334
C 0.908715 -0.841784 -0.166146
C 1.013849 0.560174 -0.155958
C 3.429249 0.350024 -0.119642
C -0.158088 1.339019 -0.175329
C -1.427781 0.743646 -0.191875
C -1.525482 -0.649401 -0.188482
C -0.366928 -1.441363 -0.191383
C -2.873452 -1.261177 -0.205933
C -4.071706 -0.398543 0.250599
C -3.855361 1.075000 -0.081781
C -2.623952 1.585066 -0.270858
O -0.429104 -2.814604 -0.221174
O -2.962044 -2.446959 -0.536555
O -4.078738 -0.528148 1.679526
C -5.341691 -1.007192 -0.379524
C -6.629546 -0.486462 0.222711
C -7.811032 -0.357232 -0.700213
O -6.723250 -0.233340 1.425628
O -5.025114 1.810520 -0.139131
C -4.938928 3.228352 -0.227827
C 4.805229 0.926687 -0.109238
C 4.971374 2.309121 -0.680565
C 5.831265 0.189085 0.370541
C 7.284958 0.588674 0.491658
C 8.141077 -0.012206 -0.639906
C 8.209157 -1.534199 -0.675858
C 7.817235 0.218999 1.879988
H 2.108102 -2.646344 -0.158028
H 2.376971 2.231885 -0.110648
H -0.073871 2.425103 -0.177092
H -2.460587 2.634535 -0.487812
H -1.370904 -3.080106 -0.328109
H -4.985213 -0.302403 1.981316
H -5.379031 -2.093539 -0.229813
H -5.346785 -0.824424 -1.460962
H -8.668508 0.037268 -0.148135
H -7.568526 0.333922 -1.511248
H -8.068491 -1.339262 -1.104172
H -4.379093 3.641645 0.617359
H -4.495000 3.532434 -1.180959
H -5.956017 3.629290 -0.187671
H 4.644600 3.065834 0.040172
H 6.005270 2.532998 -0.956936

H 4.388281 2.425017 -1.601420
H 5.614838 -0.817516 0.734244
H 7.376501 1.678399 0.418518
H 7.757833 0.340778 -1.606012
H 9.163487 0.377265 -0.555263
H 7.218482 -1.980153 -0.803676
H 8.828507 -1.857680 -1.519057
H 8.658295 -1.938072 0.235955
H 7.266332 0.756725 2.660205
H 8.874500 0.489043 1.974102
H 7.720574 -0.851770 2.088724
SCF Energy (B3LYP/6-31G**)= -1361.71092528
Number of imaginary frequencies = 0

1a_c364
MMFF Geometry
C -1.676043 -1.564540 -0.598492
C -2.149599 1.020994 0.091975
N -2.960898 -1.183646 -0.398379
C -0.566458 -0.723437 -0.476566
C -0.823318 0.613094 -0.117688
C -3.188944 0.104353 -0.056118
C 0.251518 1.511867 0.016707
C 1.574490 1.097155 -0.187918
C 1.820600 -0.235466 -0.529973
C 0.762415 -1.139679 -0.692696
C 3.220109 -0.647109 -0.750233
C 4.345861 0.208589 -0.137980
C 3.957008 1.681187 -0.079841
C 2.667771 2.070891 -0.094754
O 0.975114 -2.444187 -1.067117
O 3.462913 -1.661067 -1.411003
O 5.489321 0.067480 -1.000869
C 4.745637 -0.262935 1.262203
C 4.758322 -1.767476 1.384932
C 6.013156 -2.496272 0.985639
O 3.752391 -2.370609 1.761357
O 5.041515 2.531976 0.037830
C 4.815696 3.935588 0.016306
C -4.610850 0.497163 0.144210
C -4.995400 1.860913 -0.352412
C -5.455850 -0.395743 0.704540
C -6.917287 -0.307389 1.094664
C -7.827087 -1.050942 0.096850
C -7.877785 -0.456538 -1.304441
C -7.459293 1.075999 1.463486
H -1.556382 -2.611064 -0.870949
H -2.365461 2.044201 0.382811
H 0.046884 2.548476 0.282168
H 2.381150 3.114543 -0.027129
H 1.931087 -2.570613 -1.257304
H 5.302944 -0.681866 -1.601308
H 4.041762 0.103958 2.021192
H 5.737296 0.110975 1.545229
H 5.938608 -3.544098 1.289988
H 6.143586 -2.449285 -0.097227
H 6.875024 -2.052074 1.489700
H 4.330732 4.240764 -0.916564
H 4.228362 4.249308 0.885111
H 5.788106 4.433956 0.069916
H -6.010190 1.874861 -0.755591
H -4.915901 2.602295 0.448743
H -4.356430 2.183105 -1.182865
H -5.036180 -1.372399 0.964265
H -6.979071 -0.878146 2.034106
H -7.496686 -2.094792 0.016690
H -8.847446 -1.083888 0.499206
H -6.891499 -0.455073 -1.777410
H -8.545170 -1.052359 -1.935956
H -8.261470 0.567340 -1.294618
H -6.775718 1.600038 2.140133
H -8.418382 0.974094 1.984627
H -7.641316 1.712927 0.595717
SCF Energy (B3LYP/6-31G**)= -1361.69600862
Number of imaginary frequencies = 0

1a_c365
MMFF Geometry
C -1.492180 -2.144186 -0.655801
C -2.377523 0.329743 0.043012
N -2.826103 -1.970472 -0.492138
C -0.529495 -1.142558 -0.492479
C -0.999810 0.134090 -0.129434
C -3.253467 -0.736109 -0.151136
C -0.081863 1.185472 0.047698
C 1.293956 0.982217 -0.121678
C 1.755775 -0.291512 -0.468837
C 0.853931 -1.345736 -0.670628
C 3.209513 -0.481990 -0.656831
C 4.185977 0.546693 -0.049978
C 3.547295 1.921943 0.025778
C 2.218664 2.105656 0.030151
O 1.275029 -2.598056 -1.047928
O 3.620714 -1.453490 -1.299420
O 5.312746 0.595949 -0.942578
C 4.685289 0.142107 1.339403
C 4.920104 -1.343677 1.466951
C 6.276252 -1.875443 1.088706
O 4.011612 -2.090689 1.832073
O 4.369645 3.003965 0.308196
C 4.999946 3.519287 -0.864522
C -4.717684 -0.588933 0.022478
C -5.344504 -1.660780 0.867212
C -5.362222 0.441918 -0.565417
C -6.833836 0.803291 -0.627348
C -7.174938 1.975309 0.314359
C -7.020311 1.679945 1.800604
C -7.842465 -0.343163 -0.521164
H -1.203109 -3.155906 -0.932774
H -2.764925 1.300286 0.337601
H -0.450399 2.174192 0.319343
H 1.807267 3.100743 0.174681
H 2.245404 -2.575980 -1.201961
H 5.301103 -0.237423 -1.456783
H 3.955044 0.405039 2.116515
H 5.617766 0.659643 1.596236
H 6.341055 -2.933840 1.356408
H 6.431047 -1.771834 0.013013
H 7.050954 -1.332711 1.635937
H 5.927686 2.978659 -1.069507
H 4.337922 3.491609 -1.737289
H 5.260961 4.563993 -0.670273
H -4.619825 -2.106779 1.558808
H -6.138395 -1.259116 1.501280
H -5.749193 -2.461280 0.240105
H -4.760173 1.139889 -1.151453
H -6.973899 1.191157 -1.648088
H -6.538830 2.834766 0.065534
H -8.207669 2.295696 0.127529
H -5.990356 1.411902 2.053281
H -7.282270 2.568639 2.384381
H -7.680158 0.869219 2.121509
H -7.563972 -1.175089 -1.176929
H -8.834200 0.001398 -0.836620
H -7.952497 -0.726495 0.495147
SCF Energy (B3LYP/6-31G**)= -1361.69028111
Number of imaginary frequencies = 0

1a_c366
MMFF Geometry
C -1.759892 -1.337535 -0.701948
C -2.165208 1.204655 0.165563
N -3.029308 -0.947043 -0.435295
C -0.631472 -0.527443 -0.558832
C -0.852772 0.787377 -0.108985
C -3.229499 0.320184 -0.004847
C 0.242673 1.656689 0.048920
C 1.549498 1.228249 -0.214867
C 1.763000 -0.079773 -0.655849
C 0.682245 -0.952289 -0.841386
C 3.154449 -0.519413 -0.907913
C 4.303931 0.253681 -0.212877

C 3.938085 1.734104 -0.102925
C 2.666629 2.161431 -0.101240
O 0.864001 -2.239023 -1.287766
O 3.329909 -1.537826 -1.579616
O 5.485510 0.132145 -1.002647
C 4.520260 -0.362767 1.182844
C 4.834004 -1.843886 1.108022
C 4.101961 -2.751085 2.059396
O 5.675448 -2.278386 0.321699
O 4.926769 2.710224 -0.153818
C 5.984447 2.570001 0.785869
C -4.640494 0.706970 0.288667
C -4.849616 1.812695 1.287944
C -5.648806 0.043868 -0.320126
C -7.137936 0.274400 -0.188566
C -7.842346 -0.933034 0.457287
C -7.417357 -1.175985 1.900106
C -7.728511 0.543979 -1.576815
H -1.671205 -2.365085 -1.048224
H -2.346078 2.221907 0.497067
H 0.069864 2.680590 0.379328
H 2.449962 3.225815 -0.066472
H 1.788006 -2.331246 -1.617619
H 5.668179 -0.828741 -1.089222
H 3.629378 -0.227291 1.808077
H 5.365762 0.092075 1.708365
H 4.421960 -3.784862 1.902788
H 4.328246 -2.461281 3.088327
H 3.026939 -2.683617 1.874430
H 5.593011 2.449086 1.801105
H 6.650015 1.743928 0.523400
H 6.575614 3.490469 0.762829
H -4.668849 2.788671 0.825637
H -5.857899 1.823932 1.708911
H -4.174679 1.698676 2.144072
H -5.391985 -0.761674 -1.011772
H -7.348664 1.160061 0.418694
H -8.926893 -0.767152 0.443315
H -7.656435 -1.842348 -0.128298
H -7.584450 -0.284722 2.513229
H -8.001408 -1.996209 2.329788
H -6.360321 -1.450142 1.968244
H -7.249444 1.411130 -2.045250
H -8.801120 0.755139 -1.506433
H -7.599097 -0.315317 -2.244721
SCF Energy (B3LYP/6-31G**)= -1361.70192284
Number of imaginary frequencies = 0

1a_c367
MMFF Geometry
C -1.731745 -1.321398 -1.052799
C -2.199282 1.211151 -0.188099
N -3.017331 -0.932900 -0.875681
C -0.616765 -0.514155 -0.817295
C -0.870278 0.794994 -0.367778
C -3.248057 0.330031 -0.448144
C 0.210835 1.659301 -0.113299
C 1.533478 1.232030 -0.283388
C 1.778552 -0.069788 -0.726332
C 0.714196 -0.937457 -1.005726
C 3.184688 -0.508637 -0.878343
C 4.278677 0.251549 -0.086272
C 3.907135 1.731016 0.017291
C 2.639688 2.161047 -0.071355
O 0.928197 -2.218329 -1.454656
O 3.409699 -1.517248 -1.550058
O 5.516953 0.138923 -0.785287
C 4.387151 -0.385666 1.312672
C 4.704065 -1.866230 1.240333
C 3.901903 -2.785258 2.121208
O 5.601652 -2.291333 0.513182
O 4.897909 2.705568 0.056105
C 5.880430 2.549519 1.071821
C -4.677074 0.725471 -0.284670
C -4.998552 2.189554 -0.418348
C -5.606088 -0.228840 -0.054960
C -7.092296 -0.054061 0.164554
C -7.551518 -0.728594 1.472214
C -6.941401 -0.097501 2.717871
C -7.850594 -0.645759 -1.026068
H -1.617889 -2.344721 -1.404205
H -2.403609 2.216189 0.166321
H 0.014422 2.678472 0.218534
H 2.422118 3.225264 -0.037303
H 1.874314 -2.307535 -1.715429
H 5.704478 -0.821027 -0.871936
H 3.451261 -0.257358 1.869976
H 5.190553 0.059626 1.907809
H 4.050810 -2.510514 3.168219
H 2.843820 -2.712924 1.857268
H 4.231656 -3.817387 1.974259
H 5.412337 2.414858 2.052282
H 6.563215 1.725838 0.849053
H 6.472637 3.468919 1.107427
H -4.438906 2.639554 -1.246467
H -6.052119 2.375887 -0.641348
H -4.750287 2.723863 0.504579
H -5.275527 -1.268473 -0.001613
H -7.358685 1.005203 0.234137
H -8.643526 -0.655524 1.550691
H -7.308967 -1.798739 1.457225
H -7.162554 0.973640 2.762816
H -7.354957 -0.566279 3.616588
H -5.855324 -0.227868 2.744790
H -7.554678 -0.157540 -1.961429
H -8.930333 -0.505571 -0.905982
H -7.661118 -1.720109 -1.131326
SCF Energy (B3LYP/6-31G**)= -1361.70185621
Number of imaginary frequencies = 0

1a_c368
MMFF Geometry
C 1.728504 2.062609 -0.436530
C 2.251626 -0.602103 -0.543667
N 3.025874 1.673282 -0.481373
C 0.630765 1.194526 -0.445049
C 0.914692 -0.181442 -0.503274
C 3.270842 0.347138 -0.528260
C -0.146085 -1.103699 -0.520414
C -1.483328 -0.679743 -0.480395
C -1.765650 0.688953 -0.415026
C -0.713067 1.621687 -0.401352
C -3.180749 1.141411 -0.380713
C -4.316504 0.089404 -0.419836
C -3.837889 -1.346746 -0.486109
C -2.543087 -1.685270 -0.492549
O -0.942754 2.977595 -0.354451
O -3.405874 2.355960 -0.383122
O -5.058515 0.359093 -1.628681
C -5.280473 0.347180 0.746039
C -4.673187 0.062496 2.102583
C -5.639658 -0.160767 3.235732
O -3.457886 0.059622 2.295313
O -4.778838 -2.363445 -0.372169
C -5.619326 -2.482400 -1.518761
C 4.696257 -0.049998 -0.564797
C 5.517933 0.634381 -1.618505
C 5.159440 -0.911159 0.366847
C 6.537944 -1.499087 0.599074
C 7.209434 -0.882736 1.843870
C 7.546677 0.598635 1.730032
C 7.491464 -1.555345 -0.596099
H 1.586309 3.140347 -0.391706
H 2.499271 -1.658044 -0.594624
H 0.078587 -2.169183 -0.561352
H -2.259590 -2.734003 -0.471974
H -1.914763 3.128221 -0.311498
H -4.421973 0.361688 -2.364600
H -6.179943 -0.270415 0.630462
H -5.625311 1.388714 0.746869
H -5.087040 -0.358037 4.158496
H -6.254170 0.732175 3.373531

H -6.271524 -1.024326 3.013854
H -6.431861 -1.751710 -1.476455
H -5.052326 -2.381404 -2.450661
H -6.068133 -3.480063 -1.501289
H 4.960175 1.410857 -2.153520
H 6.388722 1.127492 -1.176052
H 5.848978 -0.084806 -2.374066
H 4.447135 -1.260944 1.118395
H 6.347857 -2.553481 0.850559
H 6.555207 -1.022392 2.714318
H 8.131680 -1.435061 2.064137
H 6.651041 1.204503 1.564734
H 8.012886 0.945169 2.658326
H 8.251516 0.790244 0.916226
H 7.008734 -2.023087 -1.460835
H 8.369907 -2.162434 -0.348565
H 7.858975 -0.571138 -0.895232
SCF Energy (B3LYP/6-31G**)= -1361.69173449
Number of imaginary frequencies = 0

1a_c369
MMFF Geometry
C -1.560846 -2.355484 -0.553326
C -2.447797 0.198998 -0.275032
N -2.896236 -2.154238 -0.445730
C -0.596756 -1.342870 -0.530028
C -1.067693 -0.024002 -0.388296
C -3.325909 -0.882724 -0.298825
C -0.147935 1.040742 -0.372638
C 1.230462 0.809631 -0.475525
C 1.693129 -0.502198 -0.601029
C 0.788658 -1.573353 -0.646200
C 3.153998 -0.729878 -0.687214
C 4.099936 0.369363 -0.145276
C 3.486040 1.752611 -0.392774
C 2.159741 1.939556 -0.519344
O 1.214473 -2.870965 -0.799321
O 3.538283 -1.815985 -1.124953
O 5.356325 0.302481 -0.819238
C 4.306115 0.158208 1.363354
C 5.494680 -0.704173 1.743455
C 5.581571 -2.104990 1.199584
O 6.335385 -0.289819 2.546456
O 4.438834 2.753134 -0.467872
C 4.007753 4.101699 -0.615988
C -4.793782 -0.707590 -0.190282
C -5.591246 -1.566018 -1.135061
C -5.317134 0.145569 0.716137
C -6.781237 0.416400 0.988713
C -7.153192 1.875845 0.667821
C -7.018049 2.218812 -0.810602
C -7.084683 0.108989 2.458830
H -1.272980 -3.398922 -0.664147
H -2.836385 1.208650 -0.183488
H -0.518416 2.061297 -0.280071
H 1.724767 2.919943 -0.680878
H 2.171677 -2.863439 -1.031737
H 5.398194 1.050665 -1.441378
H 3.416538 -0.266596 1.844370
H 4.479980 1.127322 1.852100
H 6.327051 -2.668250 1.769003
H 4.618017 -2.607210 1.314949
H 5.887004 -2.088970 0.152275
H 3.480835 4.240300 -1.565367
H 3.382627 4.407342 0.229115
H 4.896501 4.739453 -0.627109
H -5.643593 -2.596437 -0.767968
H -6.612682 -1.205197 -1.278067
H -5.133367 -1.576002 -2.131200
H -4.640916 0.696972 1.369945
H -7.422110 -0.238925 0.390792
H -8.193027 2.056795 0.967710
H -6.532904 2.566264 1.253381
H -7.619798 1.542793 -1.426327
H -7.365940 3.240927 -0.991572
H -5.977965 2.158271 -1.144930
H -6.840909 -0.931749 2.700276
H -8.148258 0.259297 2.673821
H -6.511622 0.755443 3.133300
SCF Energy (B3LYP/6-31G**)= -1361.69994649
Number of imaginary frequencies = 0

1a_c370
MMFF Geometry
C -1.620391 -2.186395 -0.265309
C -2.375442 0.332191 0.424709
N -2.922020 -1.975352 0.045366
C -0.627864 -1.201469 -0.255599
C -1.029927 0.098409 0.104802
C -3.288727 -0.719201 0.378123
C -0.077960 1.134262 0.131250
C 1.266581 0.891491 -0.180670
C 1.662015 -0.402531 -0.526798
C 0.721868 -1.442057 -0.581650
C 3.090479 -0.645739 -0.832945
C 4.137201 0.357723 -0.290497
C 3.546275 1.772705 -0.281746
C 2.222277 1.999696 -0.207399
O 1.078502 -2.718610 -0.944673
O 3.375965 -1.675250 -1.447387
O 5.292447 0.352831 -1.128720
C 4.529469 -0.047019 1.139682
C 5.726083 -0.972763 1.249123
C 5.692182 -2.294758 0.530225
O 6.677937 -0.679357 1.977937
O 4.516574 2.756163 -0.358236
C 4.118579 4.120619 -0.279143
C -4.721017 -0.534419 0.712291
C -5.310133 -1.639054 1.547864
C -5.400184 0.537560 0.250965
C -6.866605 0.854658 0.451482
C -7.594857 1.029206 -0.895412
C -7.631071 -0.247597 -1.726404
C -6.994347 2.130889 1.287146
H -1.385088 -3.214504 -0.532754
H -2.706280 1.322884 0.720995
H -0.396690 2.141819 0.396956
H 1.805483 3.000860 -0.187458
H 1.996582 -2.700122 -1.301204
H 5.280803 1.173641 -1.652867
H 3.695244 -0.512704 1.678564
H 4.799229 0.848782 1.716788
H 6.484898 -2.939489 0.921588
H 4.734626 -2.789413 0.710150
H 5.859519 -2.151902 -0.538415
H 3.478701 4.389547 -1.125550
H 3.619266 4.327648 0.672816
H 5.020719 4.737387 -0.328826
H -4.613999 -1.942053 2.338836
H -6.230582 -1.341914 2.056365
H -5.531512 -2.514149 0.927786
H -4.877654 1.261388 -0.374912
H -7.373275 0.052535 0.996927
H -8.628648 1.345236 -0.707354
H -7.125106 1.826987 -1.484588
H -8.091004 -1.067337 -1.165366
H -8.220360 -0.087029 -2.634936
H -6.627307 -0.558002 -2.031975
H -6.502623 2.013894 2.259443
H -8.047402 2.367463 1.474297
H -6.541691 2.991337 0.781230
SCF Energy (B3LYP/6-31G**)= -1361.70004180
Number of imaginary frequencies = 0

1a_c371
MMFF Geometry
C -1.395273 -2.163722 -0.488478
C -2.266266 0.364555 0.003140
N -2.722611 -1.976206 -0.289324
C -0.432478 -1.150122 -0.460673
C -0.894986 0.155615 -0.203014
C -3.144832 -0.715597 -0.053535

C 0.022516 1.222071 -0.166965
C 1.390441 1.002190 -0.364981
C 1.844621 -0.299613 -0.604897
C 0.943081 -1.368854 -0.674710
C 3.298014 -0.500439 -0.805308
C 4.256931 0.549466 -0.185534
C 3.645049 1.937667 -0.342613
C 2.319854 2.131430 -0.417595
O 1.358541 -2.647582 -0.955144
O 3.681584 -1.499633 -1.414689
O 5.512777 0.513779 -0.868905
C 4.525304 0.224705 1.288627
C 4.704705 -1.257564 1.529069
C 6.028752 -1.878334 1.171218
O 3.774834 -1.930047 1.976897
O 4.477598 3.023170 -0.576653
C 5.342933 3.328858 0.513485
C -4.603067 -0.556381 0.164272
C -5.204300 -1.605299 1.058078
C -5.273824 0.452241 -0.433474
C -6.753369 0.784272 -0.408926
C -7.147116 1.476401 0.908524
C -8.570112 2.018784 0.904392
C -7.653955 -0.398863 -0.776508
H -1.112047 -3.197096 -0.677763
H -2.645087 1.358770 0.219017
H -0.340193 2.233720 0.011237
H 1.915767 3.128194 -0.571346
H 2.317648 -2.630228 -1.171728
H 5.338170 0.786071 -1.788635
H 3.691934 0.549887 1.925136
H 5.423772 0.726302 1.665077
H 6.090683 -2.879352 1.607445
H 6.125246 -1.956399 0.086623
H 6.843071 -1.275093 1.580216
H 4.804911 3.318324 1.467511
H 6.192219 2.640894 0.540520
H 5.735766 4.337808 0.356513
H -6.149150 -1.287301 1.502605
H -5.380870 -2.531508 0.501249
H -4.538912 -1.829918 1.900084
H -4.711138 1.132072 -1.075073
H -6.896018 1.521533 -1.212903
H -7.038287 0.789244 1.755078
H -6.462016 2.313154 1.097071
H -9.306194 1.211470 0.847032
H -8.761603 2.576160 1.827044
H -8.730279 2.696792 0.060240
H -7.241249 -0.965032 -1.618891
H -8.645201 -0.045856 -1.081499
H -7.801075 -1.087456 0.059800
SCF Energy (B3LYP/6-31G**)= -1361.68661429
Number of imaginary frequencies = 0

1a_c372
MMFF Geometry
C -1.810540 -1.679973 -0.498319
C -2.151600 0.951771 0.080147
N -3.075250 -1.225501 -0.323831
C -0.658454 -0.893206 -0.406204
C -0.847821 0.466657 -0.106507
C -3.237104 0.085655 -0.035934
C 0.272375 1.309598 0.000552
C 1.574966 0.822305 -0.188440
C 1.762788 -0.532605 -0.481535
C 0.650395 -1.385654 -0.593210
C 3.140013 -1.050212 -0.690448
C 4.341676 -0.079444 -0.581663
C 3.963488 1.351016 -0.254755
C 2.699822 1.745286 -0.057562
O 0.784958 -2.721576 -0.894137
O 3.276509 -2.236462 -1.006630
O 4.964865 -0.080102 -1.884064
C 5.373617 -0.664480 0.391902
C 4.897069 -0.692717 1.827913
C 5.963282 -0.804654 2.885547
O 3.704072 -0.676168 2.128863
O 4.980868 2.259876 0.011672
C 5.735675 2.614039 -1.145942
C -4.637446 0.559966 0.140015
C -4.946160 1.924090 -0.406139
C -5.532012 -0.266383 0.725061
C -6.988493 -0.086545 1.101915
C -7.932012 -0.814142 0.123857
C -7.943812 -0.266377 -1.297165
C -7.457201 1.335677 1.419997
H -1.744280 -2.742035 -0.724643
H -2.313020 1.995772 0.329418
H 0.122760 2.362265 0.239066
H 2.494848 2.769485 0.241794
H 1.743880 -2.926868 -0.981474
H 4.273308 0.128153 -2.536091
H 6.303073 -0.083362 0.344656
H 5.642001 -1.690057 0.109285
H 5.500629 -0.813310 3.876342
H 6.520991 -1.733820 2.745657
H 6.636122 0.053962 2.820099
H 6.494619 1.855290 -1.356210
H 5.091891 2.773649 -2.017831
H 6.254775 3.552932 -0.931278
H -4.288492 2.181553 -1.244545
H -5.957536 1.978720 -0.814344
H -4.828863 2.688021 0.368761
H -5.166311 -1.253971 1.022213
H -7.085529 -0.620636 2.059765
H -7.657793 -1.876321 0.081507
H -8.954633 -0.778571 0.520202
H -6.956566 -0.333991 -1.763251
H -8.639154 -0.846947 -1.912512
H -8.271950 0.776311 -1.324795
H -6.749468 1.845458 2.082551
H -8.422606 1.303074 1.938418
H -7.601186 1.951200 0.529926
SCF Energy (B3LYP/6-31G**)= -1361.69200905
Number of imaginary frequencies = 0

1a_c373
MMFF Geometry
C -1.599388 -1.648873 -1.139503
C -2.281131 0.836801 -0.272994
N -2.917558 -1.345889 -1.056155
C -0.555929 -0.775608 -0.813976
C -0.921316 0.509563 -0.368220
C -3.241545 -0.110688 -0.619920
C 0.081398 1.438886 -0.035977
C 1.438316 1.103299 -0.127690
C 1.794280 -0.180791 -0.555098
C 0.809363 -1.111284 -0.914011
C 3.231998 -0.511172 -0.655724
C 4.249120 0.344785 0.125278
C 3.756045 1.772924 0.259177
C 2.458625 2.106638 0.180002
O 1.130231 -2.365034 -1.375359
O 3.595263 -1.458756 -1.359186
O 5.464240 0.345275 -0.644295
C 4.556067 -0.220581 1.513910
C 4.638685 -1.727812 1.522207
C 5.970968 -2.360421 1.222504
O 3.632332 -2.407237 1.728351
O 4.694858 2.715938 0.649703
C 5.146329 3.486113 -0.464908
C -4.688335 0.188658 -0.530792
C -5.474767 -0.109597 -1.774407
C -5.195899 0.620978 0.643759
C -6.605673 1.001890 1.053011
C -7.230827 -0.065091 1.975526
C -7.479444 -1.419030 1.322699
C -7.568698 1.436055 -0.053736
H -1.393282 -2.657985 -1.490354
H -2.592789 1.822485 0.059174
H -0.205503 2.436120 0.296165
H 2.142648 3.129221 0.364844

H 2.106998 -2.435178 -1.458330
H 5.413818 -0.422893 -1.248954
H 3.773336 0.049019 2.235589
H 5.499123 0.176256 1.909432
H 5.913563 -3.436886 1.406976
H 6.236530 -2.193389 0.176844
H 6.736024 -1.938326 1.878797
H 5.580122 2.852303 -1.244545
H 4.329988 4.087594 -0.878821
H 5.923792 4.168153 -0.108470
H -4.875251 -0.603075 -2.547268
H -6.311618 -0.779894 -1.556297
H -5.853127 0.815525 -2.220146
H -4.500979 0.712458 1.482264
H -6.477436 1.900290 1.675557
H -6.580434 -0.215780 2.847007
H -8.183042 0.313705 2.367848
H -6.550213 -1.869239 0.961580
H -7.917975 -2.107638 2.052545
H -8.176839 -1.340261 0.484112
H -7.120477 2.214860 -0.679946
H -8.480183 1.858469 0.384733
H -7.878749 0.609963 -0.697665
SCF Energy (B3LYP/6-31G**)= -1361.69070462
Number of imaginary frequencies = 0

1a_c374
MMFF Geometry
C -1.689772 -1.485530 -0.704987
C -2.183342 1.023640 0.214815
N -2.978904 -1.128072 -0.489616
C -0.585170 -0.656641 -0.489738
C -0.852710 0.640402 -0.012689
C -3.216898 0.122074 -0.032677
C 0.215967 1.525780 0.220207
C 1.542500 1.135200 -0.004161
C 1.801533 -0.160564 -0.464411
C 0.748540 -1.048328 -0.723775
C 3.207468 -0.549497 -0.705811
C 4.330883 0.260736 -0.028632
C 3.912675 1.707364 0.153342
C 2.628642 2.094444 0.202071
O 0.970249 -2.313110 -1.212403
O 3.460350 -1.508382 -1.441543
O 5.463364 0.214608 -0.914116
C 4.749272 -0.322600 1.322972
C 4.770903 -1.831973 1.322626
C 6.040650 -2.516663 0.893745
O 3.762740 -2.471037 1.626038
O 4.923329 2.609388 0.450166
C 5.295622 3.364437 -0.703366
C -4.643126 0.490908 0.182508
C -5.025257 1.894872 -0.187991
C -5.493154 -0.452944 0.642847
C -6.960421 -0.405059 1.017851
C -7.853056 -1.052007 -0.059597
C -7.885373 -0.326943 -1.398528
C -7.512458 0.935785 1.508727
H -1.562293 -2.502128 -1.071049
H -2.406921 2.014927 0.596366
H 0.005077 2.532634 0.579163
H 2.374950 3.128321 0.417461
H 1.930182 -2.423047 -1.392119
H 5.322360 -0.548702 -1.510545
H 4.052731 -0.024640 2.118094
H 5.741953 0.034020 1.624103
H 6.882765 -2.128651 1.472090
H 5.957842 -3.590701 1.082662
H 6.209224 -2.356069 -0.172847
H 5.625294 2.716257 -1.521220
H 4.468298 4.000246 -1.036233
H 6.131339 4.012868 -0.424453
H -4.375979 2.295561 -0.975155
H -6.034406 1.945048 -0.602270
H -4.959278 2.557114 0.680859
H -5.073882 -1.448892 0.815186
H -7.033949 -1.062852 1.897646
H -7.517968 -2.082812 -0.233872
H -8.879046 -1.125172 0.322631
H -6.892319 -0.278468 -1.854549
H -8.541466 -0.861239 -2.093715
H -8.272640 0.690576 -1.296956
H -6.840478 1.394449 2.242083
H -8.478554 0.782697 2.003753
H -7.684275 1.652069 0.702968
SCF Energy (B3LYP/6-31G**)= -1361.69099374
Number of imaginary frequencies = 0

1a_c375
MMFF Geometry
C 1.785827 2.410635 0.028393
C 2.419517 -0.232684 -0.035657
N 3.091916 2.068470 0.139260
C 0.731213 1.503073 -0.116173
C 1.071640 0.137202 -0.147186
C 3.394517 0.753199 0.099364
C 0.056620 -0.825685 -0.293544
C -1.288897 -0.449654 -0.398041
C -1.623353 0.907243 -0.353830
C -0.622086 1.881413 -0.229286
C -3.051694 1.287352 -0.448751
C -4.134002 0.217892 -0.196636
C -3.619254 -1.159998 -0.554802
C -2.315151 -1.469809 -0.601802
O -0.916996 3.223667 -0.209086
O -3.349799 2.463388 -0.681826
O -5.229968 0.565480 -1.057314
C -4.573811 0.370287 1.262938
C -5.480123 -0.731797 1.765383
C -6.883532 -0.800328 1.221152
O -5.088829 -1.521541 2.627253
O -4.573655 -2.150522 -0.725516
C -4.810563 -2.413640 -2.108873
C 4.832895 0.421119 0.226833
C 5.527478 1.129979 1.353945
C 5.399507 -0.447509 -0.638115
C 6.833361 -0.914944 -0.798926
C 7.035654 -2.339871 -0.246250
C 6.860688 -2.482740 1.260087
C 7.938325 0.046071 -0.353454
H 1.600139 3.482140 0.061683
H 2.707240 -1.279722 -0.041361
H 0.324080 -1.881613 -0.327278
H -2.001438 -2.491730 -0.795134
H -1.869192 3.343736 -0.423342
H -5.244739 1.542706 -1.108485
H -5.103779 1.319256 1.415766
H -3.699146 0.395651 1.926801
H -7.467782 -1.511283 1.812574
H -7.356195 0.181974 1.299661
H -6.877581 -1.132859 0.182278
H -5.116690 -1.509076 -2.643786
H -3.923243 -2.849056 -2.580364
H -5.623883 -3.141961 -2.177166
H 4.824611 1.420215 2.143962
H 6.260319 0.484130 1.843301
H 6.023781 2.036668 0.994175
H 4.755767 -0.880482 -1.407019
H 6.970350 -0.995984 -1.888255
H 6.332235 -3.021979 -0.741374
H 8.040381 -2.689064 -0.515833
H 5.851818 -2.203030 1.577022
H 7.021609 -3.525078 1.554766
H 7.580953 -1.870505 1.809674
H 7.758117 1.057276 -0.733811
H 8.904186 -0.284195 -0.753212
H 8.049990 0.099956 0.731241
SCF Energy (B3LYP/6-31G**)= -1361.69319508
Number of imaginary frequencies = 0

1a_c376
MMFF Geometry

C -1.460534 -2.011567 -0.711013
C -2.229437 0.580314 -0.424448
N -2.787742 -1.746230 -0.641359
C -0.449044 -1.048276 -0.647506
C -0.859248 0.291657 -0.501231
C -3.158024 -0.456873 -0.488845
C 0.108860 1.311682 -0.448703
C 1.474453 1.012643 -0.516008
C 1.875290 -0.322265 -0.642018
C 0.925563 -1.347579 -0.729830
C 3.327180 -0.606593 -0.705895
C 4.287567 0.430441 -0.066987
C 3.767140 1.835050 -0.354043
C 2.465141 2.089187 -0.552835
O 1.292267 -2.659644 -0.904316
O 3.704701 -1.658761 -1.222899
O 5.591541 0.291066 -0.637663
C 4.415804 0.183335 1.440689
C 4.493805 -1.288556 1.779103
C 5.806984 -1.994850 1.571220
O 3.494991 -1.885527 2.183352
O 4.673948 2.861753 -0.577225
C 5.462246 3.190000 0.563597
C -4.619399 -0.209344 -0.421298
C -5.414791 -0.991702 -1.429355
C -5.120863 0.635286 0.506024
C -6.558121 1.018955 0.800122
C -7.441182 -0.188176 1.168958
C -8.794517 0.212390 1.742921
C -7.148315 1.901000 -0.302783
H -1.219783 -3.066574 -0.824638
H -2.571044 1.606468 -0.329880
H -0.211660 2.349057 -0.359965
H 2.129949 3.093463 -0.796878
H 2.265788 -2.703076 -1.036512
H 5.508238 0.515226 -1.582835
H 3.551904 0.587634 1.984392
H 5.306287 0.661829 1.863349
H 5.778162 -2.969262 2.067060
H 5.987968 -2.143552 0.504972
H 6.616567 -1.408916 2.013429
H 4.847966 3.264181 1.467423
H 6.267754 2.463772 0.701656
H 5.920952 4.166849 0.383893
H -5.485235 -2.043880 -1.133470
H -6.429761 -0.613696 -1.558151
H -4.943809 -0.940864 -2.418033
H -4.421289 1.107449 1.197722
H -6.507703 1.651517 1.698806
H -7.616029 -0.830528 0.300207
H -6.921563 -0.803160 1.914891
H -9.409571 0.725543 0.997942
H -9.342218 -0.678308 2.067531
H -8.675874 0.870303 2.609524
H -6.447096 2.691922 -0.592322
H -8.064136 2.393265 0.040607
H -7.399498 1.328894 -1.200221
SCF Energy (B3LYP/6-31G**)= -1361.68693675
Number of imaginary frequencies = 0

1a_c377
MMFF Geometry
C 1.881870 2.115222 0.184143
C 2.293937 -0.570352 0.293152
N 3.142219 1.674789 0.412622
C 0.768488 1.289149 -0.000079
C 0.994385 -0.099175 0.055872
C 3.334454 0.338300 0.470081
C -0.083558 -0.983164 -0.134829
C -1.381934 -0.506815 -0.363003
C -1.601620 0.871837 -0.402243
C -0.535377 1.768182 -0.238598
C -2.979758 1.356820 -0.624932
C -4.157775 0.390676 -0.397704
C -3.756354 -1.054269 -0.652893
C -2.471492 -1.453275 -0.612221
O -0.718586 3.128873 -0.300360
O -3.161715 2.538369 -0.935948
O -5.168445 0.786286 -1.340074
C -4.676274 0.650120 1.021258
C -5.690698 -0.364153 1.501161
C -7.084531 -0.293034 0.933408
O -5.384151 -1.209211 2.345106
O -4.832865 -1.887569 -0.897966
C -4.604584 -3.283382 -1.043404
C 4.723441 -0.115037 0.726412
C 4.897661 -1.160986 1.787761
C 5.710255 0.425223 -0.020767
C 7.210268 0.209169 -0.046724
C 7.637827 -0.517895 -1.339839
C 7.137774 -1.951819 -1.467840
C 7.863526 -0.411721 1.188695
H 1.785808 3.198441 0.151683
H 2.495805 -1.636663 0.325010
H 0.098009 -2.057224 -0.104695
H -2.185967 -2.486875 -0.774444
H -1.636789 3.312606 -0.601188
H -5.070301 1.750493 -1.471010
H -5.142337 1.640930 1.095910
H -3.848829 0.644613 1.743396
H -7.743846 -0.951797 1.505959
H -7.463643 0.728662 1.016078
H -7.089449 -0.612821 -0.109718
H -3.983093 -3.488093 -1.920890
H -4.156357 -3.703273 -0.137169
H -5.573213 -3.768377 -1.196131
H 5.306605 -2.081751 1.360004
H 5.555455 -0.802560 2.584647
H 3.955143 -1.424842 2.279663
H 5.405216 1.169703 -0.762995
H 7.637384 1.221019 -0.112180
H 7.284543 0.051094 -2.209713
H 8.733283 -0.522700 -1.403815
H 6.045001 -2.000733 -1.457144
H 7.478729 -2.380169 -2.416205
H 7.521722 -2.585533 -0.663523
H 7.615008 0.158376 2.090085
H 8.954917 -0.394347 1.088736
H 7.572152 -1.453406 1.344031
SCF Energy (B3LYP/6-31G**)= -1361.69792107
Number of imaginary frequencies = 0

1a_c378
MMFF Geometry
C -2.088226 -1.614125 -0.350637
C -2.307290 1.089642 -0.225636
N -3.325619 -1.068500 -0.437085
C -0.905206 -0.887037 -0.199462
C -1.030544 0.511787 -0.133447
C -3.431566 0.278983 -0.377086
C 0.123624 1.299910 0.031207
C 1.396781 0.717821 0.115044
C 1.515966 -0.671157 0.033111
C 0.373330 -1.473742 -0.107203
C 2.867460 -1.268691 0.122685
C 4.084998 -0.366439 -0.180524
C 3.819842 1.080527 0.225223
C 2.569417 1.564800 0.344481
O 0.454010 -2.845473 -0.156021
O 2.947546 -2.471898 0.385540
O 4.208286 -0.404400 -1.609540
C 5.309772 -1.000933 0.510623
C 6.633415 -0.429336 0.048400
C 7.735203 -0.347251 1.070026
O 6.819023 -0.098700 -1.124674
O 4.969627 1.822703 0.424595
C 4.854596 3.230708 0.597307
C -4.808375 0.843069 -0.494943
C -4.923034 2.236678 -1.051298
C -5.865042 0.085139 -0.125597
C -7.334546 0.443074 -0.145745
C -7.933911 0.489767 1.275299

C -7.964605 -0.834210 2.029215
C -8.091416 -0.518577 -1.064986
H -2.072714 -2.700332 -0.409449
H -2.413690 2.167594 -0.164592
H 0.022729 2.382772 0.093696
H 2.372722 2.596453 0.613091
H 1.388178 -3.107268 0.010436
H 5.132441 -0.150574 -1.822269
H 5.375798 -2.075022 0.296310
H 5.225511 -0.887699 1.598289
H 8.627838 0.090715 0.614973
H 7.417953 0.287941 1.900788
H 7.974756 -1.350423 1.430735
H 4.330208 3.467077 1.528515
H 5.865264 3.643734 0.666544
H 4.358790 3.692842 -0.262444
H -4.245331 2.374928 -1.901722
H -5.920884 2.467149 -1.431217
H -4.678261 2.978409 -0.283854
H -5.663154 -0.922445 0.243318
H -7.474825 1.446032 -0.560387
H -7.375916 1.219555 1.876130
H -8.961652 0.869328 1.209749
H -6.959833 -1.239296 2.178592
H -8.409937 -0.686116 3.018723
H -8.568289 -1.581271 1.506092
H -7.733909 -0.427072 -2.097060
H -9.163312 -0.293731 -1.064043
H -7.962944 -1.564081 -0.764898
SCF Energy (B3LYP/6-31G**)= -1361.71060260
Number of imaginary frequencies = 0

1a_c379
MMFF Geometry
C -1.568075 -1.917401 -0.448531
C -2.221886 0.558911 0.465448
N -2.865009 -1.676285 -0.139052
C -0.533303 -0.983548 -0.334874
C -0.883927 0.295962 0.137001
C -3.176833 -0.444497 0.320221
C 0.109753 1.285001 0.258994
C 1.446244 1.013768 -0.063659
C 1.789710 -0.264721 -0.513379
C 0.808734 -1.254114 -0.668810
C 3.205235 -0.528885 -0.848480
C 4.279384 0.384566 -0.212100
C 3.763388 1.815635 -0.074707
C 2.446641 2.083929 0.009297
O 1.111800 -2.504183 -1.151593
O 3.466468 -1.474682 -1.593775
O 5.437705 0.388682 -1.057120
C 4.720291 -0.135203 1.158093
C 4.880055 -1.637566 1.177955
C 6.132896 -2.225684 0.585895
O 3.986558 -2.350305 1.638912
O 4.772400 2.759452 -0.024131
C 4.421728 4.129439 0.133211
C -4.602369 -0.210671 0.657091
C -4.876046 0.429510 1.985889
C -5.531712 -0.568080 -0.255287
C -7.044786 -0.480721 -0.263693
C -7.527957 0.578761 -1.277369
C -7.158052 2.016297 -0.932073
C -7.758720 -0.338037 1.081113
H -1.372752 -2.926245 -0.806304
H -2.520188 1.541753 0.816820
H -0.170003 2.278283 0.608227
H 2.067536 3.093092 0.128175
H 2.058894 -2.521013 -1.418233
H 5.210330 0.906580 -1.848873
H 3.983075 0.112805 1.933258
H 5.671922 0.310361 1.473205
H 6.224703 -3.270865 0.894691
H 6.089926 -2.178670 -0.503939
H 7.007078 -1.682306 0.953111
H 3.819753 4.478115 -0.712005
H 3.899650 4.292605 1.081460
H 5.346610 4.713390 0.152395
H -5.366449 1.399173 1.854462
H -5.501088 -0.216598 2.608872
H -3.963577 0.605999 2.565715
H -5.155951 -1.013993 -1.181719
H -7.378778 -1.452178 -0.657739
H -7.119573 0.344702 -2.269174
H -8.618993 0.512157 -1.374027
H -6.074187 2.150268 -0.869235
H -7.530278 2.691892 -1.709344
H -7.602365 2.329661 0.016780
H -7.464961 -1.140196 1.766335
H -8.843467 -0.414535 0.943644
H -7.563609 0.622135 1.564973
SCF Energy (B3LYP/6-31G**)= -1361.68922004
Number of imaginary frequencies = 0

1a_c380
MMFF Geometry
C -1.780942 -1.689935 -0.537594
C -2.104798 0.936373 0.074461
N -3.042253 -1.230976 -0.350589
C -0.624202 -0.910431 -0.442339
C -0.804614 0.446825 -0.125324
C -3.195490 0.077354 -0.045842
C 0.320458 1.283094 -0.015676
C 1.619515 0.791099 -0.216208
C 1.798266 -0.562468 -0.524396
C 0.680752 -1.407644 -0.642881
C 3.171984 -1.086869 -0.742314
C 4.383154 -0.144462 -0.546906
C 4.011636 1.293938 -0.263948
C 2.749719 1.710304 -0.093620
O 0.806461 -2.739900 -0.963262
O 3.298450 -2.254981 -1.122704
O 5.119473 -0.155803 -1.789117
C 5.329508 -0.738311 0.504082
C 4.769286 -0.704898 1.908251
C 5.771057 -0.727621 3.032330
O 3.559861 -0.713693 2.135344
O 5.072936 2.162342 -0.052040
C 5.278287 3.029224 -1.167562
C -4.592273 0.556800 0.143804
C -4.897166 1.928551 -0.385058
C -5.487585 -0.271603 0.724776
C -6.940895 -0.088749 1.112258
C -7.893797 -0.800603 0.131714
C -7.911149 -0.236861 -1.282991
C -7.400527 1.332145 1.448971
H -1.721552 -2.749526 -0.777047
H -2.259497 1.978069 0.337167
H 0.176962 2.333900 0.234110
H 2.547147 2.742719 0.175907
H 1.762395 -2.944644 -1.079136
H 4.484788 0.020037 -2.504386
H 6.281251 -0.191403 0.495439
H 5.576142 -1.780937 0.268018
H 5.248670 -0.698903 3.992586
H 6.362429 -1.644754 2.976634
H 6.421951 0.147307 2.961095
H 5.459475 2.461376 -2.085591
H 4.427015 3.704746 -1.301705
H 6.164589 3.636527 -0.961923
H -5.910517 1.992826 -0.786920
H -4.771719 2.683013 0.397802
H -4.242872 2.192301 -1.224149
H -5.125177 -1.264348 1.008522
H -7.035010 -0.633057 2.064631
H -7.625206 -1.863595 0.075850
H -8.913885 -0.764380 0.534474
H -6.927004 -0.304159 -1.755633
H -8.613008 -0.807009 -1.900680
H -8.234170 0.807695 -1.297000
H -6.686404 1.830892 2.113065
H -8.363083 1.298573 1.972601

H -7.546494 1.958338 0.566699
SCF Energy (B3LYP/6-31G**)= -1361.69167999
Number of imaginary frequencies = 0

1a_c381
MMFF Geometry
C -1.766226 -2.208185 -0.014662
C -2.448974 0.396862 0.358692
N -3.056462 -1.925855 0.286727
C -0.750737 -1.256185 -0.146341
C -1.115510 0.089901 0.051312
C -3.387322 -0.628213 0.461029
C -0.140288 1.097051 -0.069740
C 1.190604 0.780174 -0.366207
C 1.549038 -0.558547 -0.552024
C 0.586188 -1.572320 -0.460328
C 2.968861 -0.871207 -0.843177
C 4.037982 0.163322 -0.408518
C 3.486688 1.571980 -0.596178
C 2.173718 1.842282 -0.567867
O 0.908580 -2.892308 -0.663651
O 3.235138 -1.967367 -1.339186
O 5.176088 -0.013972 -1.251359
C 4.375227 -0.162976 1.059374
C 5.852493 -0.274973 1.355267
C 6.539532 -1.574631 1.022321
O 6.454343 0.641266 1.919198
O 4.344608 2.590560 -0.987108
C 5.388116 2.867748 -0.055295
C -4.808678 -0.365563 0.790008
C -5.408946 -1.339417 1.768058
C -5.470195 0.656497 0.206039
C -6.924653 1.034847 0.386156
C -7.672538 1.056217 -0.961119
C -7.754487 -0.314269 -1.622282
C -7.006525 2.409657 1.054449
H -1.560425 -3.267669 -0.152278
H -2.750655 1.425678 0.530123
H -0.428032 2.139362 0.065253
H 1.816491 2.852074 -0.751517
H 1.820145 -2.941823 -1.032772
H 4.958848 0.382508 -2.114870
H 3.923582 -1.110972 1.382123
H 3.949859 0.585985 1.740336
H 6.221131 -1.944647 0.045869
H 7.621436 -1.418440 0.994756
H 6.298116 -2.314403 1.789528
H 5.021766 2.859591 0.976320
H 6.214162 2.166084 -0.190398
H 5.768104 3.871064 -0.270063
H -4.706376 -1.557118 2.581107
H -6.313062 -0.956524 2.247699
H -5.661804 -2.280303 1.267964
H -4.941634 1.281443 -0.514304
H -7.440668 0.321939 1.036631
H -8.694853 1.420557 -0.799585
H -7.194309 1.760238 -1.653869
H -8.223941 -1.043888 -0.954792
H -8.355937 -0.254811 -2.535078
H -6.764159 -0.687148 -1.900631
H -6.500392 2.404054 2.026375
H -8.050033 2.695589 1.225481
H -6.542386 3.186832 0.436554
SCF Energy (B3LYP/6-31G**)= -1361.69219914
Number of imaginary frequencies = 0

1a_c382
MMFF Geometry
C -1.651511 -2.384021 -0.264635
C -2.519560 0.191774 -0.313853
N -2.984376 -2.160347 -0.171325
C -0.680712 -1.384591 -0.382584
C -1.142185 -0.054408 -0.409207
C -3.404578 -0.877177 -0.188137
C -0.216533 0.996975 -0.540340
C 1.158021 0.742882 -0.622976
C 1.612513 -0.578904 -0.584364
C 0.701955 -1.639780 -0.481083
C 3.073712 -0.825905 -0.651345
C 4.027562 0.331621 -0.263564
C 3.414111 1.667074 -0.671418
C 2.095430 1.843602 -0.831445
O 1.118574 -2.949120 -0.467003
O 3.459864 -1.962042 -0.932549
O 5.258812 0.168709 -0.964100
C 4.236502 0.239895 1.259452
C 5.585619 -0.307200 1.670862
C 5.874794 -1.760649 1.397943
O 6.404421 0.412889 2.246594
O 4.259151 2.696954 -1.065946
C 5.138209 3.156724 -0.039700
C -4.870213 -0.678415 -0.090017
C -5.682470 -1.647486 -0.906443
C -5.379141 0.290567 0.700835
C -6.838671 0.606269 0.948418
C -7.204406 2.013427 0.441026
C -7.081755 2.157282 -1.070888
C -7.129462 0.497873 2.448924
H -1.371675 -3.435206 -0.241518
H -2.900539 1.207815 -0.351785
H -0.577349 2.024458 -0.580799
H 1.706642 2.800023 -1.170787
H 2.074531 -2.980020 -0.700873
H 5.118807 0.480313 -1.876673
H 3.468282 -0.371620 1.750157
H 4.141247 1.226291 1.730487
H 6.766033 -2.063877 1.954974
H 5.034862 -2.372733 1.736267
H 6.054299 -1.922298 0.333892
H 4.582003 3.411527 0.868233
H 5.917917 2.421643 0.175066
H 5.627126 4.065669 -0.402597
H -5.737399 -2.620384 -0.406730
H -6.703087 -1.301041 -1.086128
H -5.234804 -1.791536 -1.896842
H -4.692949 0.918480 1.269766
H -7.489658 -0.117284 0.448051
H -8.240027 2.240345 0.723747
H -6.573910 2.770287 0.924713
H -7.693914 1.410569 -1.586545
H -7.424861 3.149222 -1.382065
H -6.045452 2.045184 -1.403643
H -6.890008 -0.503750 2.823450
H -8.189857 0.683416 2.651758
H -6.545573 1.223242 3.026960
SCF Energy (B3LYP/6-31G**)= -1361.69405167
Number of imaginary frequencies = 0

1a_c383
MMFF Geometry
C -1.385617 -2.311232 -0.553830
C -2.339734 0.232580 -0.452372
N -2.727090 -2.137670 -0.478668
C -0.447128 -1.275139 -0.578244
C -0.952768 0.038195 -0.526510
C -3.190622 -0.870488 -0.422119
C -0.060130 1.125352 -0.558441
C 1.323823 0.921586 -0.619140
C 1.820459 -0.383321 -0.663668
C 0.945138 -1.477896 -0.657477
C 3.288093 -0.576638 -0.705130
C 4.192745 0.572094 -0.191229
C 3.552331 1.920553 -0.521443
C 2.231803 2.061362 -0.710083
O 1.408013 -2.770337 -0.716147
O 3.720061 -1.676798 -1.054690
O 5.456725 0.493126 -0.847550
C 4.362263 0.403941 1.331217
C 4.948125 -0.946542 1.692782
C 4.296839 -1.698961 2.821375
O 5.934535 -1.391451 1.106128
O 4.345260 3.030702 -0.789199

C 5.299651 3.365275 0.210082
C -4.664354 -0.727734 -0.349446
C -5.423527 -1.671652 -1.243114
C -5.227793 0.172710 0.484307
C -6.705794 0.413681 0.713871
C -7.087326 1.795453 0.152925
C -8.580065 2.087012 0.222904
C -7.009186 0.288079 2.210131
H -1.070646 -3.351828 -0.596200
H -2.753628 1.236147 -0.433165
H -0.453520 2.141341 -0.537871
H 1.821332 3.028725 -0.987229
H 2.364065 -2.752797 -0.954455
H 5.820056 -0.397749 -0.651299
H 3.397884 0.512463 1.842233
H 5.046656 1.145743 1.754621
H 4.815644 -2.648472 2.979151
H 4.354530 -1.106229 3.737470
H 3.253793 -1.905614 2.568816
H 4.826632 3.438672 1.194673
H 6.130084 2.655113 0.228223
H 5.711264 4.348453 -0.037315
H -5.471508 -2.670185 -0.796114
H -6.445267 -1.337539 -1.440755
H -4.939547 -1.753827 -2.223466
H -4.580035 0.792819 1.104402
H -7.304592 -0.346692 0.202622
H -6.544991 2.586854 0.685945
H -6.777864 1.857271 -0.898607
H -8.924847 2.172627 1.257396
H -8.800321 3.035452 -0.277727
H -9.157934 1.300748 -0.272882
H -6.654990 -0.672258 2.602240
H -8.085854 0.337453 2.400811
H -6.526877 1.085444 2.786732
SCF Energy (B3LYP/6-31G**)= -1361.69964686
Number of imaginary frequencies = 0

1a_c384
MMFF Geometry
C -1.705857 -1.492341 -0.671565
C -2.202712 1.034886 0.195174
N -2.995593 -1.131710 -0.465262
C -0.602200 -0.658158 -0.472227
C -0.871488 0.648058 -0.022671
C -3.235219 0.127524 -0.034819
C 0.196370 1.538479 0.193542
C 1.523756 1.144710 -0.021413
C 1.784771 -0.159005 -0.454811
C 0.732356 -1.053031 -0.696380
C 3.190464 -0.550490 -0.690406
C 4.319978 0.283463 -0.050863
C 3.894236 1.730383 0.126017
C 2.607955 2.106975 0.174803
O 0.955264 -2.327853 -1.158011
O 3.441293 -1.530885 -1.398821
O 5.423340 0.221668 -0.971760
C 4.780984 -0.277524 1.296826
C 4.792651 -1.786763 1.329844
C 6.046095 -2.489355 0.882356
O 3.789745 -2.411890 1.676315
O 4.869582 2.661697 0.455507
C 5.598037 3.110204 -0.687273
C -4.662040 0.499585 0.170769
C -5.044623 1.895422 -0.228871
C -5.512069 -0.435281 0.649101
C -6.979889 -0.380780 1.021007
C -7.870589 -1.050154 -0.044274
C -7.901520 -0.352535 -1.397739
C -7.533504 0.969393 1.483730
H -1.577021 -2.516177 -1.016320
H -2.427411 2.033761 0.555716
H -0.015535 2.552081 0.532493
H 2.351772 3.140555 0.390400
H 1.915534 -2.440658 -1.334001
H 5.278497 -0.568502 -1.531968
H 4.114407 0.041683 2.109279
H 5.785572 0.078631 1.556212
H 5.955990 -3.560512 1.083831
H 6.194598 -2.341557 -0.189058
H 6.903678 -2.104035 1.439375
H 6.458818 2.460834 -0.867979
H 4.969516 3.175175 -1.582593
H 5.979028 4.112049 -0.467145
H -6.053300 1.936439 -0.645299
H -4.980126 2.575364 0.626310
H -4.394660 2.280329 -1.023312
H -5.092336 -1.427177 0.842367
H -7.054212 -1.020576 1.913910
H -7.534574 -2.084063 -0.197054
H -8.897063 -1.116230 0.337947
H -6.907863 -0.312673 -1.853281
H -8.556290 -0.901332 -2.082803
H -8.289602 0.666574 -1.317448
H -6.862824 1.443384 2.208480
H -8.500158 0.825730 1.980489
H -7.704738 1.668990 0.663319
SCF Energy (B3LYP/6-31G**)= -1361.69025100
Number of imaginary frequencies = 0

1a_c385
MMFF Geometry
C -1.705825 -2.172852 -0.091407
C -2.444555 0.399892 0.383847
N -3.004204 -1.929131 0.208666
C -0.708570 -1.196164 -0.173094
C -1.102378 0.132471 0.077173
C -3.362939 -0.646875 0.433776
C -0.146335 1.162419 0.008829
C 1.193789 0.885353 -0.288007
C 1.581787 -0.436429 -0.527774
C 0.637623 -1.471857 -0.486674
C 3.010020 -0.711881 -0.819074
C 4.061092 0.333110 -0.368160
C 3.471965 1.736410 -0.467031
C 2.154320 1.977042 -0.427160
O 0.986559 -2.776546 -0.739986
O 3.301438 -1.794198 -1.331583
O 5.189697 0.256098 -1.236248
C 4.446045 -0.024519 1.079450
C 5.804804 -0.674757 1.219743
C 5.984252 -2.067816 0.674034
O 6.722493 -0.089408 1.799094
O 4.314998 2.793008 -0.788444
C 5.332642 3.043759 0.180493
C -4.792250 -0.426546 0.759251
C -5.381722 -1.452603 1.689343
C -5.468917 0.605963 0.212187
C -6.932369 0.947366 0.393412
C -7.668080 1.010780 -0.959233
C -7.716101 -0.331898 -1.678257
C -7.048274 2.290806 1.118087
H -1.477477 -3.221360 -0.271166
H -2.768535 1.414388 0.595239
H -0.456631 2.191921 0.185998
H 1.776927 2.988682 -0.550434
H 1.904048 -2.795501 -1.096873
H 4.957445 0.723561 -2.059062
H 3.711717 -0.687424 1.555080
H 4.458827 0.869020 1.716116
H 6.918727 -2.489218 1.055759
H 5.160266 -2.703092 1.008801
H 6.027148 -2.049621 -0.416097
H 4.902601 3.155854 1.181058
H 6.093993 2.259763 0.169806
H 5.820317 3.986379 -0.085127
H -4.682391 -1.690612 2.499488
H -6.297722 -1.108278 2.175628
H -5.610973 -2.376282 1.147535
H -4.946585 1.271134 -0.475944
H -7.439791 0.197488 1.008092
H -8.699024 1.347565 -0.792815

H -7.197871 1.752820 -1.616903
H -8.176785 -1.098267 -1.046810
H -8.310192 -0.245890 -2.593751
H -6.715886 -0.672912 -1.962061
H -6.551116 2.254198 2.093964
H -8.098901 2.548454 1.290481
H -6.594319 3.102473 0.538137
SCF Energy (B3LYP/6-31G**)= -1361.69413255
Number of imaginary frequencies = 0

1a_c386
MMFF Geometry
C -1.427870 -2.158873 -0.511930
C -2.298964 0.273477 0.334411
N -2.739327 -2.026616 -0.198964
C -0.480059 -1.134481 -0.427218
C -0.941955 0.120152 0.015157
C -3.164296 -0.811610 0.209632
C -0.037097 1.192200 0.123248
C 1.318333 1.026933 -0.186602
C 1.771940 -0.220510 -0.621941
C 0.880980 -1.294139 -0.755666
C 3.213196 -0.379464 -0.922869
C 4.209805 0.618827 -0.280768
C 3.560822 1.998930 -0.172996
C 2.230652 2.165276 -0.126461
O 1.300229 -2.527408 -1.193131
O 3.563756 -1.354574 -1.590265
O 5.363349 0.719676 -1.113714
C 4.593603 0.083385 1.112379
C 5.193183 -1.307057 1.042237
C 4.693809 -2.322277 2.034196
O 6.073737 -1.581436 0.227099
O 4.333035 3.150687 -0.273040
C 5.429467 3.241009 0.627540
C -4.606197 -0.712058 0.539818
C -5.152186 -1.894124 1.294898
C -5.327994 0.358944 0.145614
C -6.809236 0.599763 0.353426
C -7.485096 0.825868 -1.012976
C -9.000393 0.948530 -0.926829
C -6.993955 1.789650 1.297775
H -1.145789 -3.155118 -0.846248
H -2.675246 1.227798 0.690088
H -0.398939 2.166814 0.449762
H 1.807725 3.165876 -0.094699
H 2.211239 -2.439729 -1.558538
H 5.729921 -0.187438 -1.197071
H 3.716109 0.050704 1.769554
H 5.349530 0.706737 1.600169
H 5.207195 -3.274805 1.876883
H 4.897158 -1.972623 3.049223
H 3.620617 -2.473502 1.893388
H 5.105516 3.063915 1.658194
H 6.236614 2.558697 0.349618
H 5.824380 4.259917 0.571509
H -6.087196 -1.670512 1.814515
H -5.332318 -2.734526 0.616287
H -4.448266 -2.219577 2.069928
H -4.831833 1.146459 -0.422522
H -7.281889 -0.272151 0.815915
H -7.083046 1.726816 -1.493804
H -7.248798 -0.015888 -1.676997
H -9.300542 1.850542 -0.385879
H -9.428975 1.009035 -1.932485
H -9.437481 0.079732 -0.424763
H -6.446474 1.634831 2.234660
H -8.047689 1.928072 1.559157
H -6.632027 2.720555 0.846866
SCF Energy (B3LYP/6-31G**)= -1361.69970228
Number of imaginary frequencies = 0

1a_c387
MMFF Geometry
C -1.838832 -1.455584 -0.606926
C -2.208985 1.120716 0.169958
N -3.103109 -1.037602 -0.358155
C -0.699250 -0.656450 -0.491638
C -0.902435 0.676201 -0.090367
C -3.285415 0.245778 0.029419
C 0.205123 1.535818 0.033086
C 1.507399 1.082718 -0.218260
C 1.703367 -0.245495 -0.602629
C 0.608975 -1.108863 -0.756628
C 3.087605 -0.713576 -0.843576
C 4.251269 0.078942 -0.199678
C 3.903388 1.571864 -0.157433
C 2.633165 2.015056 -0.143203
O 0.769755 -2.412111 -1.161743
O 3.235307 -1.752477 -1.489904
O 5.438570 -0.089462 -0.973686
C 4.483298 -0.437948 1.229327
C 5.500414 -1.555631 1.360236
C 5.291471 -2.825549 0.579666
O 6.441138 -1.454080 2.152713
O 5.025868 2.380605 -0.137034
C 4.855299 3.788830 -0.018123
C -4.690498 0.662125 0.308975
C -4.884162 1.794819 1.280709
C -5.707536 -0.000231 -0.285832
C -7.193154 0.256336 -0.163116
C -7.917310 -0.923893 0.510582
C -7.498628 -1.138034 1.959793
C -7.777026 0.500908 -1.558801
H -1.763993 -2.496591 -0.914174
H -2.376576 2.150729 0.467092
H 0.042857 2.572205 0.327901
H 2.387448 3.070589 -0.099647
H 1.698259 -2.535387 -1.466635
H 5.590801 0.737431 -1.465221
H 3.552125 -0.775541 1.700634
H 4.862797 0.377960 1.860643
H 5.940704 -3.608626 0.982814
H 4.255887 -3.158103 0.684280
H 5.545214 -2.673575 -0.470533
H 4.320912 4.191146 -0.884578
H 4.340762 4.042945 0.914119
H 5.848445 4.246750 0.008255
H -4.691015 2.756595 0.794294
H -5.891894 1.829393 1.701791
H -4.209919 1.693303 2.138958
H -5.461685 -0.827033 -0.956056
H -7.391101 1.159782 0.421878
H -8.999128 -0.741651 0.490285
H -7.744519 -1.850026 -0.052220
H -7.652976 -0.229584 2.550624
H -8.096042 -1.938405 2.408137
H -6.446080 -1.426719 2.036831
H -7.283794 1.348850 -2.047270
H -8.846369 0.730217 -1.495915
H -7.659704 -0.376374 -2.205213
SCF Energy (B3LYP/6-31G**)= -1361.70092275
Number of imaginary frequencies = 0

1a_c388
MMFF Geometry
C -1.606413 -1.551397 -0.663058
C -2.102838 1.044822 -0.034380
N -2.893941 -1.175929 -0.468766
C -0.504254 -0.698986 -0.564555
C -0.772803 0.643864 -0.237157
C -3.136837 0.117500 -0.155917
C 0.293182 1.556797 -0.130151
C 1.616865 1.145714 -0.325098
C 1.876623 -0.194088 -0.634310
C 0.827150 -1.110095 -0.775631
C 3.287402 -0.599186 -0.830220
C 4.380028 0.259343 -0.140979
C 3.984841 1.730116 -0.224260
C 2.704990 2.124080 -0.302336
O 1.052194 -2.419090 -1.125182
O 3.529431 -1.609322 -1.491952

O 5.629288 0.073896 -0.812083
C 4.569000 -0.183149 1.314737
C 4.521604 -1.686619 1.471104
C 5.745296 -2.477428 1.092012
O 3.493789 -2.236420 1.869514
O 4.974050 2.690067 -0.386193
C 5.854926 2.801040 0.728431
C -4.566328 0.498124 0.035133
C -4.937632 1.908429 -0.328802
C -5.439722 -0.433338 0.478770
C -6.918793 -0.330408 0.791899
C -7.763981 -0.403511 -0.492564
C -9.259464 -0.513094 -0.226899
C -7.278060 0.850771 1.698638
H -1.478829 -2.603185 -0.910435
H -2.321033 2.074280 0.230284
H 0.082514 2.600136 0.101798
H 2.457056 3.177123 -0.402720
H 2.007256 -2.534345 -1.329223
H 5.514286 0.418564 -1.716796
H 3.781186 0.227079 1.960070
H 5.524667 0.156885 1.729017
H 5.649661 -3.499006 1.470692
H 5.849543 -2.509029 0.005786
H 6.632317 -2.026066 1.543409
H 5.303742 2.817730 1.674823
H 6.591626 1.993229 0.723799
H 6.396504 3.746974 0.634395
H -4.404381 2.236987 -1.228420
H -5.998142 2.016441 -0.564216
H -4.697433 2.594651 0.489781
H -5.049942 -1.436572 0.670120
H -7.150787 -1.230359 1.380614
H -7.584875 0.474083 -1.124002
H -7.455453 -1.277790 -1.080371
H -9.649519 0.392720 0.246633
H -9.798569 -0.652011 -1.169601
H -9.482586 -1.367718 0.419459
H -6.543667 0.972100 2.502612
H -8.250982 0.685782 2.174604
H -7.345278 1.793982 1.149986
SCF Energy (B3LYP/6-31G**)= -1361.68689608
Number of imaginary frequencies = 0

1a_c389
MMFF Geometry
C -1.815832 -1.456892 -0.954987
C -2.246561 1.115591 -0.196911
N -3.095684 -1.042247 -0.796669
C -0.689223 -0.656342 -0.753798
C -0.923611 0.673684 -0.360932
C -3.308176 0.239707 -0.420019
C 0.170541 1.532756 -0.147179
C 1.488581 1.082350 -0.303173
C 1.714464 -0.242722 -0.681913
C 0.635747 -1.105812 -0.923416
C 3.113410 -0.708196 -0.821799
C 4.224750 0.080246 -0.086919
C 3.872617 1.572473 -0.058520
C 2.604370 2.014717 -0.136102
O 0.828080 -2.406323 -1.323566
O 3.310216 -1.742433 -1.462522
O 5.466473 -0.080725 -0.772073
C 4.350784 -0.448177 1.351127
C 5.357992 -1.565127 1.548091
C 5.209521 -2.829469 0.744952
O 6.238127 -1.467842 2.407844
O 4.989404 2.381725 0.052828
C 4.808513 3.788685 0.171567
C -4.731290 0.661523 -0.271825
C -5.032648 2.123450 -0.462692
C -5.673207 -0.269846 -0.003725
C -7.156542 -0.066037 0.210234
C -7.622774 -0.680998 1.544513
C -7.002045 -0.008670 2.763058
C -7.924797 -0.694761 -0.954754
H -1.716780 -2.496268 -1.260906
H -2.436698 2.136651 0.117119
H -0.014660 2.566678 0.142732
H 2.354775 3.069692 -0.102298
H 1.776854 -2.526690 -1.559412
H 5.657096 0.752858 -1.238311
H 3.387869 -0.791343 1.748894
H 4.680178 0.363004 2.015781
H 5.828632 -3.614709 1.189180
H 4.169641 -3.164098 0.770379
H 5.539776 -2.669313 -0.282515
H 4.340060 4.198663 -0.728789
H 4.225189 4.033821 1.064898
H 5.796275 4.246978 0.276543
H -4.468025 2.532665 -1.308404
H -6.083847 2.315564 -0.691865
H -4.775585 2.690164 0.438237
H -5.357029 -1.311055 0.091164
H -7.408352 0.998714 0.237332
H -8.713552 -0.589862 1.621177
H -7.394884 -1.754102 1.572391
H -7.208459 1.066347 2.765036
H -7.420498 -0.435143 3.680386
H -5.917810 -0.152744 2.794029
H -7.623775 -0.248714 -1.909350
H -9.002323 -0.535063 -0.839242
H -7.750180 -1.774975 -1.016840
SCF Energy (B3LYP/6-31G**)= -1361.70102628
Number of imaginary frequencies = 0

1a_c390
MMFF Geometry
C 1.859882 2.121040 0.199317
C 2.260923 -0.568039 0.261614
N 3.116461 1.671279 0.430747
C 0.745402 1.302836 -0.010624
C 0.965447 -0.087635 0.020932
C 3.303020 0.333237 0.465302
C -0.113385 -0.964230 -0.198121
C -1.407146 -0.477911 -0.428094
C -1.621757 0.902976 -0.440560
C -0.553924 1.791858 -0.251945
C -2.999779 1.397162 -0.661372
C -4.169487 0.428527 -0.371569
C -3.780354 -1.001642 -0.729897
C -2.498187 -1.410525 -0.721982
O -0.733395 3.153736 -0.292165
O -3.147153 2.569975 -1.010135
O -5.292557 0.837717 -1.160332
C -4.542346 0.614046 1.103905
C -5.497313 -0.439868 1.617081
C -6.950066 -0.322119 1.236812
O -5.091425 -1.371784 2.315376
O -4.864880 -1.801605 -1.039812
C -4.665119 -3.207531 -1.137670
C 4.687799 -0.130269 0.726119
C 4.848118 -1.195262 1.770573
C 5.683508 0.419070 -0.002394
C 7.182868 0.197655 -0.018256
C 7.619737 -0.508384 -1.319878
C 7.115535 -1.937890 -1.477570
C 7.822224 -0.447255 1.212123
H 1.767809 3.205064 0.186630
H 2.458784 -1.635460 0.275658
H 0.064050 -2.039286 -0.188721
H -2.214988 -2.431118 -0.955500
H -1.646771 3.342844 -0.608287
H -5.112757 0.561318 -2.075996
H -5.005789 1.594316 1.273080
H -3.651863 0.581893 1.745444
H -7.546646 -0.980477 1.874809
H -7.291589 0.704463 1.391276
H -7.092723 -0.614132 0.194917
H -4.032021 -3.451483 -1.996490
H -4.243278 -3.609747 -0.210847
H -5.641446 -3.675104 -1.295510

H 5.258070 -2.109847 1.330696
H 5.499359 -0.853190 2.579937
H 3.899949 -1.464371 2.248575
H 5.388173 1.177890 -0.733961
H 7.614365 1.208816 -0.062038
H 7.276708 0.077083 -2.182895
H 8.715716 -0.516350 -1.373786
H 6.022535 -1.982717 -1.477839
H 7.463704 -2.350941 -2.430092
H 7.489599 -2.587047 -0.680950
H 7.567378 0.107950 2.121012
H 8.914560 -0.432349 1.122638
H 7.525561 -1.490362 1.346479
SCF Energy (B3LYP/6-31G**)= -1361.69186482
Number of imaginary frequencies = 0

1a_c391
MMFF Geometry
C 1.887616 2.156118 0.125041
C 2.340522 -0.405550 -0.666624
N 3.173719 1.750306 -0.003342
C 0.767739 1.353887 -0.119546
C 1.014976 0.030097 -0.531130
C 3.384264 0.475242 -0.392551
C -0.069023 -0.823912 -0.803659
C -1.391336 -0.384577 -0.658400
C -1.632089 0.925976 -0.234640
C -0.563088 1.797822 0.018792
C -3.035557 1.369489 -0.069557
C -4.153578 0.311197 0.024098
C -3.766452 -0.944520 -0.727690
C -2.496017 -1.278884 -0.998016
O -0.767999 3.099750 0.408684
O -3.279511 2.575861 0.037133
O -5.305762 0.909111 -0.590450
C -4.434406 0.096156 1.514706
C -5.352889 -1.066286 1.820522
C -6.805603 -0.942348 1.440128
O -4.926997 -2.065703 2.403118
O -4.794711 -1.821329 -1.035991
C -5.183807 -1.707967 -2.405224
C 4.798980 0.058173 -0.515895
C 5.658395 0.968893 -1.344199
C 5.221676 -1.030796 0.162034
C 6.579605 -1.701034 0.246427
C 7.244608 -1.453041 1.616305
C 7.623537 -0.004626 1.898528
C 7.552891 -1.471767 -0.911566
H 1.775009 3.190009 0.444598
H 2.561418 -1.418303 -0.990462
H 0.125644 -1.844130 -1.133690
H -2.269183 -2.226369 -1.478577
H -1.727292 3.305186 0.341279
H -5.262624 1.866235 -0.390812
H -4.885909 0.991134 1.961826
H -3.497765 -0.081486 2.060181
H -7.370476 -1.762559 1.892626
H -7.205503 0.001054 1.820339
H -6.925411 -0.992291 0.357066
H -5.482416 -0.685024 -2.655243
H -4.377407 -2.038642 -3.068250
H -6.045132 -2.363244 -2.564394
H 5.132016 1.875469 -1.662458
H 6.533989 1.301424 -0.778562
H 5.983536 0.462939 -2.258521
H 4.486872 -1.544102 0.787319
H 6.356705 -2.778144 0.210595
H 6.571108 -1.795591 2.412704
H 8.147517 -2.071813 1.693722
H 6.747653 0.650577 1.888512
H 8.081918 0.072264 2.890062
H 8.347959 0.371667 1.170924
H 7.073530 -1.681559 -1.873739
H 8.409899 -2.149367 -0.822733
H 7.952362 -0.455442 -0.938163
SCF Energy (B3LYP/6-31G**)= -1361.69298078
Number of imaginary frequencies = 0

1a_c392
MMFF Geometry
C -1.450315 -2.370012 -0.504969
C -2.419020 0.169775 -0.454094
N -2.792705 -2.202745 -0.431807
C -0.517869 -1.329208 -0.551460
C -1.031059 -0.018040 -0.525840
C -3.263461 -0.937395 -0.400359
C -0.144831 1.073417 -0.580869
C 1.240215 0.876488 -0.638980
C 1.744394 -0.426156 -0.657550
C 0.875472 -1.525446 -0.628137
C 3.213087 -0.611755 -0.696736
C 4.111552 0.552411 -0.207294
C 3.462965 1.890144 -0.564114
C 2.141449 2.019429 -0.754085
O 1.345806 -2.816104 -0.661073
O 3.651101 -1.716265 -1.024371
O 5.375309 0.467456 -0.863296
C 4.283577 0.416218 1.318062
C 4.877663 -0.923212 1.706333
C 4.231949 -1.656276 2.850756
O 5.866031 -1.374241 1.127703
O 4.249147 2.999202 -0.855264
C 5.202576 3.359547 0.135941
C -4.737706 -0.801551 -0.329065
C -5.493861 -1.768304 -1.200694
C -5.306823 0.116514 0.481301
C -6.786549 0.348157 0.700957
C -7.288264 1.593178 -0.055897
C -6.667083 2.917592 0.371327
C -7.090121 0.419732 2.200841
H -1.129337 -3.409410 -0.526594
H -2.838647 1.171169 -0.454971
H -0.544122 2.087311 -0.580492
H 1.725072 2.978562 -1.050362
H 2.301499 -2.797859 -0.900763
H 5.744015 -0.417127 -0.649398
H 3.319095 0.529500 1.827849
H 4.964059 1.170433 1.725540
H 4.756432 -2.599358 3.027205
H 4.287152 -1.044704 3.754537
H 3.189858 -1.874064 2.603670
H 4.730126 3.450196 1.119368
H 6.037147 2.654731 0.167578
H 5.608208 4.339865 -0.131844
H -5.531874 -2.758568 -0.734781
H -6.519523 -1.445029 -1.397785
H -5.014615 -1.864831 -2.182013
H -4.661848 0.754232 1.085520
H -7.356728 -0.507180 0.321055
H -7.108708 1.456336 -1.130102
H -8.376345 1.666886 0.065851
H -5.582436 2.919835 0.229128
H -7.081024 3.732665 -0.231607
H -6.881734 3.142495 1.419932
H -6.834345 -0.526269 2.691778
H -8.155926 0.605030 2.372018
H -6.523290 1.212520 2.700479
SCF Energy (B3LYP/6-31G**)= -1361.69867324
Number of imaginary frequencies = 0

1a_c393
MMFF Geometry
C 1.958468 1.799591 -0.057934
C 2.236159 -0.901732 -0.162521
N 3.209202 1.284538 0.015064
C 0.789930 1.042902 -0.182891
C 0.945956 -0.354723 -0.235858
C 3.340262 -0.060401 -0.038859
C -0.191578 -1.172368 -0.365832
C -1.477570 -0.620223 -0.431730
C -1.627884 0.768198 -0.365187
C -0.503389 1.599150 -0.257140

C -2.994995 1.335376 -0.419085
C -4.203124 0.415320 -0.149623
C -3.884674 -1.013577 -0.535320
C -2.634757 -1.492681 -0.619284
O -0.616573 2.968266 -0.216447
O -3.140001 2.543529 -0.632548
O -5.264151 0.916645 -0.977631
C -4.582748 0.604599 1.322475
C -5.614301 -0.374946 1.837088
C -7.027564 -0.249547 1.329742
O -5.309684 -1.221787 2.679518
O -4.965919 -1.866640 -0.691413
C -5.269858 -2.076425 -2.070775
C 4.727450 -0.596616 0.029833
C 5.020563 -1.783898 -0.841501
C 5.626428 0.032379 0.818247
C 7.071726 -0.272121 1.156624
C 8.047975 0.657487 0.408971
C 8.076394 0.488859 -1.104380
C 7.506284 -1.739240 1.108662
H 1.919032 2.885764 -0.010587
H 2.373204 -1.978362 -0.187003
H -0.068025 -2.253918 -0.417005
H -2.464309 -2.544404 -0.831224
H -1.549410 3.216452 -0.404036
H -5.150395 1.887839 -1.018325
H -4.978497 1.613076 1.498879
H -3.696355 0.505156 1.963420
H -7.685954 -0.885296 1.928849
H -7.363716 0.785425 1.431351
H -7.091675 -0.565185 0.287561
H -5.466797 -1.131853 -2.587562
H -4.459937 -2.618386 -2.570314
H -6.173837 -2.689977 -2.125380
H 6.038364 -1.756506 -1.236240
H 4.873789 -2.716828 -0.288405
H 4.374357 -1.804199 -1.726794
H 5.274899 0.920434 1.352071
H 7.160770 -0.001497 2.220104
H 7.795495 1.701764 0.634697
H 9.061883 0.497943 0.797101
H 7.099826 0.696301 -1.551307
H 8.794717 1.190017 -1.542105
H 8.384484 -0.520074 -1.392078
H 6.775995 -2.383662 1.609798
H 8.461888 -1.862490 1.631598
H 7.655508 -2.111576 0.093319
SCF Energy (B3LYP/6-31G**)= -1361.69332953
Number of imaginary frequencies = 0

1a_c394
MMFF Geometry
C -1.562804 -1.698616 -0.507351
C -2.137004 0.859331 0.205072
N -2.854661 -1.381773 -0.248070
C -0.493324 -0.803125 -0.437135
C -0.802266 0.520002 -0.068249
C -3.136962 -0.107595 0.108314
C 0.228077 1.476088 0.006673
C 1.557211 1.126148 -0.258109
C 1.858379 -0.195348 -0.606043
C 0.842815 -1.152712 -0.717831
C 3.274151 -0.535621 -0.875512
C 4.360995 0.356830 -0.221067
C 3.900467 1.810419 -0.256439
C 2.602826 2.150046 -0.266661
O 1.105781 -2.443762 -1.105589
O 3.527027 -1.522412 -1.567719
O 5.583637 0.237473 -0.953419
C 4.637645 -0.102920 1.215148
C 4.659903 -1.609539 1.344176
C 5.895607 -2.341279 0.892408
O 3.675971 -2.209006 1.779988
O 4.839345 2.814958 -0.445275
C 5.767125 2.943637 0.628638
C -4.567708 0.198762 0.403976
C -4.823471 1.292957 1.402512
C -5.532768 -0.528697 -0.202073
C -7.043075 -0.437195 -0.117594
C -7.591818 0.962892 -0.453162
C -9.098501 0.975879 -0.681007
C -7.566850 -1.001397 1.205106
H -1.403439 -2.738569 -0.784516
H -2.387450 1.879454 0.476829
H -0.015218 2.505045 0.268967
H 2.306040 3.193014 -0.334473
H 2.054317 -2.514086 -1.355396
H 5.411273 0.593031 -1.844714
H 3.865394 0.262275 1.904829
H 5.597200 0.269804 1.590505
H 5.859946 -3.372795 1.254164
H 5.949491 -2.348423 -0.197911
H 6.783775 -1.861564 1.311127
H 5.261683 2.919409 1.600070
H 6.536222 2.168583 0.573088
H 6.263141 3.913603 0.528267
H -4.715819 2.276498 0.933473
H -5.819668 1.240275 1.844417
H -4.124130 1.227495 2.244053
H -5.210323 -1.317839 -0.886410
H -7.414719 -1.109713 -0.904917
H -7.363127 1.679539 0.341571
H -7.105116 1.335397 -1.363577
H -9.644324 0.727114 0.233718
H -9.420747 1.973069 -0.997985
H -9.385163 0.264518 -1.461856
H -7.096056 -1.963609 1.436763
H -8.646640 -1.175407 1.155386
H -7.379916 -0.325413 2.044388
SCF Energy (B3LYP/6-31G**)= -1361.68727468
Number of imaginary frequencies = 0

1a_c395
MMFF Geometry
C 1.701963 -1.986017 -0.401421
C 2.408248 0.637577 -0.511712
N 3.021172 -1.686020 -0.473677
C 0.668735 -1.043866 -0.379251
C 1.046246 0.311316 -0.436527
C 3.360062 -0.379039 -0.531066
C 0.052240 1.307294 -0.423436
C -1.306634 0.974566 -0.339727
C -1.673063 -0.370822 -0.267746
C -0.698338 -1.378229 -0.306053
C -3.112159 -0.696589 -0.181323
C -4.067879 0.385817 0.376969
C -3.609799 1.773777 -0.081257
C -2.323929 2.027164 -0.392861
O -1.034859 -2.710600 -0.276403
O -3.469607 -1.827413 -0.518746
O -3.985861 0.405854 1.805525
C -5.508203 0.087410 -0.087997
C -6.039721 -1.213446 0.481369
C -7.005388 -1.990449 -0.371433
O -5.736952 -1.586026 1.617248
O -4.622140 2.718867 -0.093051
C -4.279829 4.082517 -0.310522
C 4.810879 -0.080067 -0.611807
C 5.230835 0.904653 -1.662679
C 5.629678 -0.692442 0.270519
C 7.128031 -0.631144 0.490285
C 7.459613 0.092119 1.813364
C 7.102747 1.573423 1.844522
C 7.997939 -0.123408 -0.660356
H 1.486858 -3.051644 -0.358726
H 2.724698 1.675639 -0.539808
H 0.346897 2.354519 -0.479093
H -1.983718 3.012579 -0.689404
H -2.012699 -2.788266 -0.350636
H -4.444653 -0.399822 2.129951
H -5.549589 0.038309 -1.182736
H -6.211257 0.858100 0.250044

H -7.299606 -2.907104 0.147062
H -7.895734 -1.385508 -0.558961
H -6.525769 -2.260424 -1.315502
H -3.575820 4.436927 0.449328
H -3.877688 4.227539 -1.318189
H -5.194814 4.676181 -0.225440
H 5.676058 1.793279 -1.204264
H 5.944684 0.453256 -2.357550
H 4.393231 1.245919 -2.280654
H 5.153326 -1.374919 0.981655
H 7.432927 -1.678699 0.632444
H 6.938170 -0.407267 2.640376
H 8.531809 -0.013575 2.021607
H 6.032033 1.734083 1.688252
H 7.361694 1.996775 2.820662
H 7.652886 2.136257 1.085246
H 7.809142 -0.695750 -1.574718
H 9.059142 -0.249078 -0.416470
H 7.841287 0.936676 -0.874431
SCF Energy (B3LYP/6-31G**)= -1361.70160296
Number of imaginary frequencies = 0

1a_c396
MMFF Geometry
C 1.964482 2.143642 -0.236855
C 2.490567 -0.518469 -0.375452
N 3.261498 1.754241 -0.284324
C 0.867623 1.275557 -0.252101
C 1.152902 -0.099114 -0.325953
C 3.511658 0.428933 -0.345131
C 0.092457 -1.021618 -0.351332
C -1.244390 -0.598346 -0.299408
C -1.528148 0.768164 -0.222015
C -0.476542 1.701460 -0.202304
C -2.942506 1.215592 -0.159000
C -4.079440 0.172098 -0.120701
C -3.598422 -1.253332 -0.282253
C -2.306219 -1.598566 -0.329953
O -0.707008 3.055817 -0.132203
O -3.169096 2.430486 -0.131506
O -4.880884 0.506765 -1.259115
C -4.822274 0.376861 1.211866
C -6.244532 -0.130466 1.190856
C -6.600101 -1.248564 2.133873
O -7.094408 0.399505 0.473132
O -4.547102 -2.267459 -0.245008
C -5.266380 -2.377335 -1.475829
C 4.943318 0.045892 -0.401311
C 5.764605 0.895068 -1.331228
C 5.404392 -0.968662 0.361943
C 6.811986 -1.507481 0.517131
C 7.890445 -0.476439 0.902534
C 7.564045 0.291734 2.177124
C 7.234286 -2.329079 -0.703636
H 1.822830 3.221155 -0.184571
H 2.733942 -1.573838 -0.451026
H 0.317218 -2.086238 -0.413415
H -2.029465 -2.647290 -0.396169
H -1.678922 3.211865 -0.152945
H -5.462440 1.249449 -1.003905
H -4.901381 1.442011 1.467134
H -4.274545 -0.084874 2.042800
H -7.584739 -1.647362 1.874820
H -5.869458 -2.056384 2.057095
H -6.623013 -0.864943 3.156728
H -6.094107 -1.663613 -1.501531
H -4.613633 -2.246578 -2.346054
H -5.694322 -3.383300 -1.520897
H 5.996728 1.860114 -0.868461
H 6.705197 0.421740 -1.617163
H 5.225146 1.081277 -2.267375
H 4.695431 -1.481416 1.013817
H 6.763367 -2.218507 1.355358
H 8.840110 -1.004374 1.058655
H 8.072445 0.235639 0.092009
H 7.385402 -0.392281 3.012840
H 8.401972 0.942579 2.447092
H 6.680249 0.924232 2.051763
H 6.483330 -3.089918 -0.944460
H 8.178952 -2.848415 -0.508036
H 7.378316 -1.706511 -1.591428
SCF Energy (B3LYP/6-31G**)= -1361.69720395
Number of imaginary frequencies = 0

1a_c397
MMFF Geometry
C -1.577488 -1.925596 -0.405427
C -2.238646 0.563758 0.466703
N -2.875751 -1.682116 -0.103825
C -0.544614 -0.988320 -0.303050
C -0.899076 0.297899 0.147349
C -3.191391 -0.443495 0.334278
C 0.092726 1.290202 0.258418
C 1.430891 1.016794 -0.055438
C 1.776466 -0.267644 -0.484902
C 0.799030 -1.261760 -0.627998
C 3.189532 -0.529677 -0.818518
C 4.270064 0.401671 -0.235822
C 3.749466 1.824924 -0.073331
C 2.431450 2.087153 0.017706
O 1.106384 -2.519058 -1.088400
O 3.479604 -1.482414 -1.547791
O 5.358485 0.413531 -1.177733
C 4.809675 -0.094223 1.107892
C 4.970490 -1.594636 1.149182
C 6.255920 -2.179924 0.629295
O 4.054082 -2.308571 1.558889
O 4.754621 2.771047 0.017965
C 4.396622 4.145443 0.084945
C -4.618540 -0.206898 0.662438
C -4.897963 0.456177 1.978774
C -5.544185 -0.582174 -0.246533
C -7.057383 -0.497726 -0.261620
C -7.538912 0.542601 -1.295722
C -7.172718 1.986745 -0.974906
C -7.776224 -0.332284 1.077950
H -1.379533 -2.939530 -0.746848
H -2.539635 1.551754 0.801018
H -0.189444 2.288030 0.592520
H 2.053265 3.093503 0.159801
H 2.054448 -2.542300 -1.346620
H 5.201024 -0.320255 -1.805060
H 4.130105 0.167266 1.930042
H 5.779829 0.359720 1.344609
H 6.300415 -3.243261 0.881205
H 6.304386 -2.069568 -0.455737
H 7.106510 -1.679299 1.098417
H 3.820538 4.444327 -0.796740
H 3.845041 4.360168 1.005733
H 5.319111 4.733317 0.100582
H -5.388735 1.422911 1.828490
H -5.524809 -0.179520 2.610582
H -3.987933 0.643580 2.559006
H -5.164509 -1.043297 -1.163868
H -7.388318 -1.476672 -0.639374
H -7.126704 0.291602 -2.281786
H -8.629491 0.472303 -1.394903
H -6.089313 2.123779 -0.910759
H -7.543435 2.647671 -1.765397
H -7.620857 2.316203 -0.033332
H -7.483602 -1.121640 1.778356
H -8.860364 -0.412991 0.938123
H -7.584300 0.636676 1.545290
SCF Energy (B3LYP/6-31G**)= -1361.69382041
Number of imaginary frequencies = 0

1a_c398
MMFF Geometry
C -1.894646 -2.383900 0.028359
C -2.556683 0.250669 0.084894
N -3.207923 -2.060890 0.113209
C -0.845774 -1.460084 -0.030532

C -1.201155 -0.100238 0.001927
C -3.526170 -0.749052 0.130387
C -0.191346 0.876446 -0.048476
C 1.163710 0.521458 -0.130842
C 1.517345 -0.830499 -0.162784
C 0.516531 -1.817169 -0.115814
C 2.951635 -1.206170 -0.241955
C 4.037070 -0.108582 -0.230600
C 3.477238 1.296734 -0.262239
C 2.170698 1.575997 -0.183454
O 0.816176 -3.159405 -0.146246
O 3.237025 -2.407078 -0.308597
O 4.754455 -0.333072 -1.449311
C 4.898597 -0.352269 1.021563
C 6.288415 0.229190 0.916948
C 6.669245 1.306429 1.896936
O 7.098617 -0.212022 0.100129
O 4.376633 2.355187 -0.237679
C 4.983985 2.575299 -1.513228
C -4.973057 -0.437490 0.227308
C -5.712237 -1.285619 1.224582
C -5.513096 0.520053 -0.557373
C -6.948630 0.990990 -0.670722
C -7.439337 1.810641 0.537218
C -6.588804 3.043903 0.816255
C -7.926557 -0.135308 -1.019013
H -1.697752 -3.453813 0.009307
H -2.853415 1.294221 0.127151
H -0.470700 1.929630 -0.024968
H 1.840035 2.610854 -0.160424
H 1.789030 -3.262922 -0.256889
H 5.390517 -1.057502 -1.289467
H 5.049286 -1.424984 1.201934
H 4.401713 0.030432 1.921732
H 7.608642 1.770724 1.584489
H 5.897501 2.078146 1.931204
H 6.796421 0.864554 2.888145
H 5.839221 1.908423 -1.651197
H 4.267264 2.462690 -2.334262
H 5.358905 3.603075 -1.528810
H -5.140722 -1.376851 2.155725
H -6.679516 -0.866546 1.505165
H -5.883954 -2.290580 0.824563
H -4.856413 1.035781 -1.259907
H -6.967296 1.669037 -1.536976
H -8.468345 2.141474 0.347460
H -7.476380 1.194318 1.441547
H -6.530674 3.688539 -0.066502
H -7.029768 3.626570 1.631496
H -5.571780 2.772211 1.114926
H -7.555064 -0.735172 -1.857165
H -8.894695 0.282995 -1.316861
H -8.111706 -0.806524 -0.176320
SCF Energy (B3LYP/6-31G**)= -1361.69734602
Number of imaginary frequencies = 0

1a_c399
MMFF Geometry
C -1.938838 -1.633569 -0.663216
C -2.325490 1.046227 -0.441034
N -3.210229 -1.176541 -0.564869
C -0.799799 -0.825654 -0.655866
C -1.011479 0.560961 -0.539331
C -3.400713 0.158694 -0.455746
C 0.094955 1.430849 -0.531264
C 1.402139 0.937242 -0.618162
C 1.605799 -0.441978 -0.723361
C 0.515560 -1.320987 -0.759461
C 2.999680 -0.942705 -0.791712
C 4.119235 -0.036413 -0.219480
C 3.790716 1.422641 -0.514157
C 2.534633 1.857946 -0.688997
O 0.688244 -2.677814 -0.888737
O 3.190273 -2.081378 -1.222469
O 5.336045 -0.388230 -0.877245
C 4.190665 -0.347299 1.288212
C 5.580939 -0.631718 1.806513
C 6.135771 -2.018789 1.606049
O 6.206222 0.222925 2.437656
O 4.822602 2.308779 -0.790411
C 5.744907 2.487059 0.282702
C -4.815657 0.622806 -0.366577
C -5.106501 2.019410 -0.845737
C -5.760388 -0.220125 0.106206
C -7.236015 0.045175 0.305462
C -7.676554 -0.277479 1.746876
C -7.020311 0.620309 2.788750
C -8.038226 -0.796621 -0.689802
H -1.857741 -2.714880 -0.751456
H -2.497439 2.112877 -0.340172
H -0.069927 2.505638 -0.460172
H 2.343629 2.897133 -0.943162
H 1.629755 -2.856611 -1.114997
H 5.301828 -0.001342 -1.771213
H 3.577843 -1.218904 1.555802
H 3.769353 0.473704 1.883218
H 5.919730 -2.383156 0.599908
H 7.220924 -2.000154 1.739368
H 5.690269 -2.692752 2.341888
H 5.232914 2.564645 1.247250
H 6.482927 1.681921 0.286847
H 6.280242 3.425338 0.108890
H -4.558770 2.238652 -1.769537
H -6.160985 2.176644 -1.086095
H -4.821450 2.754202 -0.085640
H -5.452648 -1.224572 0.405167
H -7.476235 1.096654 0.119692
H -8.764231 -0.156601 1.826425
H -7.458849 -1.325512 1.988738
H -7.215940 1.675986 2.575327
H -7.421000 0.396234 3.782587
H -5.937129 0.469796 2.825414
H -7.755511 -0.559364 -1.721600
H -9.111003 -0.600686 -0.586498
H -7.876199 -1.869240 -0.533455
SCF Energy (B3LYP/6-31G**)= -1361.69323171
Number of imaginary frequencies = 0

1a_c400
MMFF Geometry
C -1.685594 -1.396154 -0.582204
C -2.095332 1.184435 0.160904
N -2.954486 -0.998381 -0.323834
C -0.559818 -0.575020 -0.489978
C -0.783404 0.759687 -0.104393
C -3.156780 0.287817 0.044803
C 0.309201 1.640591 -0.000191
C 1.615667 1.205185 -0.254161
C 1.831520 -0.122325 -0.631124
C 0.753363 -1.007734 -0.762931
C 3.222767 -0.567957 -0.873599
C 4.374662 0.243951 -0.228617
C 4.002696 1.726412 -0.189485
C 2.729266 2.147743 -0.197551
O 0.937325 -2.314382 -1.146084
O 3.397136 -1.617930 -1.495120
O 5.549732 0.088194 -1.022085
C 4.606377 -0.300935 1.194145
C 4.926542 -1.782565 1.190603
C 4.207304 -2.644209 2.192739
O 5.763062 -2.252197 0.419369
O 4.986183 2.703061 -0.298150
C 6.052851 2.614644 0.637670
C -4.566703 0.683397 0.330490
C -4.773997 1.837049 1.274565
C -5.578483 -0.013282 -0.233279
C -7.066957 0.228260 -0.099985
C -7.689309 -0.888852 0.756598
C -9.166708 -0.672622 1.054421
C -7.690598 0.307051 -1.497209
H -1.595042 -2.439311 -0.877602
H -2.278380 2.216274 0.442450

H 0.134412 2.678978 0.280173
H 2.507800 3.211579 -0.214068
H 1.858786 -2.418959 -1.479347
H 5.736240 -0.875015 -1.062175
H 3.720466 -0.138213 1.819941
H 5.454339 0.183304 1.688515
H 4.530856 -3.683126 2.085098
H 4.441296 -2.302318 3.203801
H 3.130376 -2.590749 2.014399
H 5.671060 2.542975 1.661215
H 6.720020 1.779382 0.410758
H 6.639355 3.535375 0.563238
H -4.614027 2.789874 0.759402
H -5.775942 1.854811 1.711244
H -4.084328 1.776624 2.124381
H -5.326908 -0.856630 -0.880281
H -7.258447 1.190275 0.386047
H -7.564307 -1.861801 0.264374
H -7.155384 -0.951258 1.713840
H -9.773238 -0.735469 0.146373
H -9.526148 -1.442372 1.745052
H -9.335310 0.303942 1.519014
H -7.176484 1.053118 -2.114092
H -8.743372 0.601830 -1.444253
H -7.634987 -0.655822 -2.017474
SCF Energy (B3LYP/6-31G**)= -1361.70069762
Number of imaginary frequencies = 0

1a_c401
MMFF Geometry
C -1.905796 -1.482082 -0.478534
C -2.280751 1.155872 0.050044
N -3.169633 -1.046749 -0.259129
C -0.768721 -0.671738 -0.451153
C -0.974572 0.693058 -0.177455
C -3.354434 0.267586 0.004204
C 0.129407 1.565206 -0.146320
C 1.430091 1.093494 -0.362949
C 1.629726 -0.265501 -0.623738
C 0.538950 -1.143679 -0.684065
C 3.016821 -0.749567 -0.829004
C 4.179121 0.109947 -0.271938
C 3.817740 1.589132 -0.358820
C 2.552222 2.028018 -0.398186
O 0.702623 -2.479166 -0.962813
O 3.176855 -1.850460 -1.359481
O 5.341652 -0.117240 -1.065778
C 4.401665 -0.339696 1.184081
C 5.635049 -1.190884 1.391109
C 5.643047 -2.581111 0.809860
O 6.586406 -0.765273 2.050049
O 4.831604 2.511928 -0.584518
C 5.805137 2.576970 0.457265
C -4.759097 0.703846 0.254705
C -4.949449 1.928069 1.109099
C -5.778419 -0.019251 -0.260140
C -7.264095 0.242334 -0.149226
C -7.975852 -0.866830 0.647249
C -7.542452 -0.931842 2.106462
C -7.862358 0.342373 -1.556617
H -1.829326 -2.547458 -0.685292
H -2.449896 2.209336 0.247644
H -0.032612 2.625443 0.046511
H 2.342118 3.088419 -0.509698
H 1.630149 -2.630234 -1.256656
H 5.241160 0.400197 -1.885314
H 3.544771 -0.896668 1.584623
H 4.501078 0.524556 1.852749
H 6.476177 -3.147544 1.235988
H 4.712850 -3.093370 1.068549
H 5.763074 -2.542561 -0.273958
H 5.328065 2.726060 1.431324
H 6.438264 1.686213 0.466304
H 6.446925 3.440695 0.259768
H -4.764237 2.836589 0.526680
H -5.953808 2.001740 1.533173
H -4.267845 1.915631 1.967446
H -5.534806 -0.908183 -0.846342
H -7.461158 1.199384 0.343536
H -9.058727 -0.692028 0.619360
H -7.803651 -1.844329 0.179095
H -7.695842 0.030977 2.604095
H -8.131540 -1.685304 2.639172
H -6.487771 -1.206949 2.201831
H -7.378066 1.138683 -2.133016
H -8.932208 0.572484 -1.506597
H -7.746706 -0.595195 -2.112339
SCF Energy (B3LYP/6-31G**)= -1361.69517012
Number of imaginary frequencies = 0

1a_c402
MMFF Geometry
C -1.665472 -1.266841 -1.039842
C -2.109441 1.246687 -0.109711
N -2.947198 -0.873210 -0.846709
C -0.543214 -0.474195 -0.789743
C -0.784527 0.825151 -0.306339
C -3.166200 0.380310 -0.386542
C 0.304394 1.674627 -0.035703
C 1.622893 1.241784 -0.223013
C 1.855852 -0.050336 -0.699611
C 0.783548 -0.902672 -0.995332
C 3.257838 -0.495836 -0.869555
C 4.361402 0.235981 -0.064160
C 4.001718 1.715150 0.078238
C 2.737197 2.156822 0.006690
O 0.985567 -2.173464 -1.477250
O 3.471889 -1.488960 -1.567418
O 5.595421 0.131563 -0.771924
C 4.471673 -0.436892 1.317845
C 4.776879 -1.917527 1.206910
C 3.971956 -2.852255 2.068534
O 5.667667 -2.331040 0.464867
O 5.000113 2.680918 0.136491
C 5.986252 2.492057 1.143090
C -4.591446 0.782627 -0.206750
C -4.901896 2.251997 -0.305321
C -5.527550 -0.169709 0.001964
C -7.013189 0.012015 0.229314
C -7.421565 -0.710135 1.528413
C -8.874541 -0.477726 1.920241
C -7.774695 -0.510821 -0.990458
H -1.561142 -2.281864 -1.417312
H -2.304405 2.244063 0.270693
H 0.117393 2.686615 0.322502
H 2.527961 3.221462 0.068443
H 1.929716 -2.263247 -1.744868
H 5.775157 -0.827303 -0.883500
H 3.539469 -0.315533 1.882828
H 5.281301 -0.012726 1.919938
H 4.293076 -3.882837 1.894166
H 4.128011 -2.604953 3.121329
H 2.913197 -2.765357 1.811793
H 5.521831 2.336409 2.122190
H 6.661626 1.669085 0.896394
H 6.585657 3.405779 1.198742
H -4.341245 2.716819 -1.124544
H -5.954701 2.451294 -0.520984
H -4.646758 2.762943 0.628869
H -5.205126 -1.213083 0.025442
H -7.257816 1.071635 0.352619
H -7.247493 -1.789854 1.436393
H -6.786360 -0.359240 2.352259
H -9.562383 -0.927298 1.198374
H -9.076636 -0.932166 2.895604
H -9.096154 0.591623 1.993070
H -7.418768 -0.031398 -1.909647
H -8.845344 -0.298558 -0.910017
H -7.652123 -1.593547 -1.106918
SCF Energy (B3LYP/6-31G**)= -1361.70065803
Number of imaginary frequencies = 0

1a_c403
MMFF Geometry
C -1.884689 -1.534106 -0.792520
C -2.319799 1.107670 -0.333535
N -3.164701 -1.108682 -0.667858
C -0.759799 -0.711696 -0.699214
C -0.996689 0.654597 -0.460206
C -3.379377 0.207834 -0.440795
C 0.094587 1.537468 -0.360070
C 1.411523 1.075672 -0.476791
C 1.641177 -0.284369 -0.705449
C 0.565270 -1.173780 -0.831985
C 3.043578 -0.757566 -0.804534
C 4.155341 0.119585 -0.176344
C 3.788305 1.594105 -0.307597
C 2.525131 2.020392 -0.443157
O 0.760258 -2.511151 -1.079832
O 3.250830 -1.863106 -1.308328
O 5.373882 -0.105927 -0.881744
C 4.276559 -0.310446 1.297450
C 5.498808 -1.147948 1.602958
C 5.559804 -2.544876 1.041254
O 6.397005 -0.705939 2.322884
O 4.807749 2.523753 -0.471650
C 5.702959 2.610870 0.636653
C -4.802897 0.638854 -0.326317
C -5.113119 2.068048 -0.681059
C -5.737543 -0.258617 0.057807
C -7.219433 -0.036460 0.263333
C -7.668619 -0.495319 1.664596
C -7.038055 0.314853 2.790735
C -7.997144 -0.797656 -0.812982
H -1.784164 -2.601545 -0.977165
H -2.511155 2.157843 -0.138611
H -0.090083 2.598291 -0.192486
H 2.314405 3.077345 -0.582658
H 1.707579 -2.656830 -1.305290
H 5.328256 0.400985 -1.712663
H 3.397693 -0.870217 1.642652
H 4.320828 0.562660 1.960513
H 6.364520 -3.098784 1.533345
H 4.617497 -3.062254 1.238668
H 5.757382 -2.518152 -0.031502
H 5.155559 2.767393 1.571787
H 6.341407 1.726343 0.702837
H 6.349882 3.478160 0.474737
H -4.559981 2.378741 -1.574922
H -6.167700 2.229013 -0.918516
H -4.848553 2.735800 0.145399
H -5.415517 -1.280823 0.268512
H -7.475846 1.023481 0.170560
H -8.758927 -0.399603 1.742364
H -7.435525 -1.557319 1.813220
H -7.249371 1.382259 2.671312
H -7.444759 -0.004603 3.755625
H -5.952901 0.178922 2.826041
H -7.708355 -0.463438 -1.815840
H -9.074104 -0.629330 -0.704654
H -7.818231 -1.877248 -0.752309
SCF Energy (B3LYP/6-31G**)= -1361.69523398
Number of imaginary frequencies = 0

1a_c404
MMFF Geometry
C -1.728370 -1.975382 -0.548556
C -2.240737 0.465094 0.531926
N -3.010793 -1.681845 -0.223834
C -0.640938 -1.112943 -0.372983
C -0.919479 0.147625 0.183847
C -3.252090 -0.468337 0.319323
C 0.130816 1.061776 0.377705
C 1.451883 0.742022 0.028056
C 1.729876 -0.515466 -0.518383
C 0.687157 -1.437073 -0.721527
C 3.126422 -0.854275 -0.896840
C 4.247762 0.191711 -0.680772
C 3.776677 1.499166 -0.076895
C 2.502421 1.730217 0.260817
O 0.910059 -2.678637 -1.271286
O 3.337789 -1.947274 -1.432075
O 4.772034 0.480788 -1.994611
C 5.393148 -0.455600 0.109194
C 5.026827 -0.782890 1.540549
C 6.173181 -0.980017 2.497089
O 3.861741 -0.932145 1.907165
O 4.731585 2.439190 0.292651
C 5.366752 3.063279 -0.821945
C -4.662617 -0.176087 0.674029
C -4.900694 0.392741 2.041685
C -5.609970 -0.421126 -0.256798
C -7.115465 -0.246404 -0.256292
C -7.535204 0.899826 -1.201593
C -7.083525 2.289534 -0.768819
C -7.821987 -0.145777 1.096187
H -1.590555 -2.967323 -0.973648
H -2.480489 1.436976 0.951975
H -0.088289 2.036497 0.812788
H 2.235249 2.661173 0.753316
H 1.873884 -2.773145 -1.448538
H 4.019900 0.735633 -2.556860
H 6.265016 0.210449 0.116349
H 5.724664 -1.384672 -0.371051
H 5.786568 -1.207806 3.494305
H 6.793553 -1.813016 2.157661
H 6.766656 -0.064039 2.551763
H 6.168856 2.429763 -1.210786
H 4.649378 3.312604 -1.611461
H 5.820009 3.994499 -0.469164
H -5.329626 1.397421 1.973390
H -5.566648 -0.252091 2.622092
H -3.981173 0.474048 2.631440
H -5.259105 -0.827509 -1.210711
H -7.504451 -1.170761 -0.708835
H -7.139504 0.704392 -2.206813
H -8.628118 0.902349 -1.299956
H -5.993842 2.356596 -0.699863
H -7.414842 3.032232 -1.502140
H -7.510607 2.568760 0.198430
H -7.577217 -1.004950 1.729577
H -8.909132 -0.149515 0.956454
H -7.571330 0.769297 1.638366
SCF Energy (B3LYP/6-31G**)= -1361.68981907
Number of imaginary frequencies = 0

1a_c405
MMFF Geometry
C -1.464244 -2.402772 -0.482093
C -2.376651 0.156288 -0.392318
N -2.803120 -2.206909 -0.415783
C -0.508691 -1.382179 -0.506017
C -0.992698 -0.061191 -0.461853
C -3.245874 -0.932422 -0.362659
C -0.081632 1.010537 -0.497673
C 1.300388 0.785647 -0.556342
C 1.775678 -0.527353 -0.584986
C 0.880615 -1.607292 -0.577845
C 3.239755 -0.746983 -0.625508
C 4.165458 0.394208 -0.138083
C 3.546386 1.752449 -0.489407
C 2.221694 1.918586 -0.655621
O 1.319527 -2.908279 -0.635725
O 3.641609 -1.856126 -0.982209
O 5.436410 0.294320 -0.779891
C 4.341033 0.285109 1.385181
C 5.527811 -0.539440 1.846201
C 5.637026 -1.972301 1.398299
O 6.348006 -0.065297 2.637248
O 4.492860 2.754420 -0.610827
C 4.054703 4.086283 -0.856909
C -4.717197 -0.764976 -0.295531
C -5.489052 -1.697143 -1.190615
C -5.268240 0.145073 0.536067

C -6.742767 0.410222 0.761439
C -7.099679 1.798613 0.200625
C -8.587593 2.114784 0.266381
C -7.052731 0.288337 2.256656
H -1.166121 -3.448612 -0.517430
H -2.774510 1.166307 -0.375882
H -0.461802 2.031585 -0.480663
H 1.782757 2.882168 -0.890774
H 2.281386 -2.907465 -0.848187
H 5.485799 1.000103 -1.449234
H 3.444689 -0.115031 1.874710
H 4.496984 1.285944 1.812134
H 6.374496 -2.489902 2.019159
H 4.675113 -2.474514 1.526665
H 5.964586 -2.022781 0.358800
H 3.547111 4.157053 -1.824129
H 3.409362 4.441430 -0.047091
H 4.938567 4.729941 -0.891797
H -5.555094 -2.694235 -0.742706
H -6.504430 -1.346272 -1.391975
H -5.003287 -1.788471 -2.169271
H -4.612282 0.753879 1.158805
H -7.352515 -0.339644 0.247627
H -6.545936 2.580461 0.736008
H -6.786054 1.856217 -0.849912
H -8.934037 2.205187 1.299910
H -8.790603 3.067179 -0.234031
H -9.176896 1.338630 -0.231871
H -6.715661 -0.678076 2.648933
H -8.129008 0.355350 2.444131
H -6.559047 1.077102 2.835453
SCF Energy (B3LYP/6-31G**)= -1361.69880002
Number of imaginary frequencies = 0

1a_c406
MMFF Geometry
C -1.503358 -2.145973 -0.504267
C -2.354500 0.252569 0.450554
N -2.808292 -2.024844 -0.160838
C -0.552678 -1.126189 -0.399474
C -1.004164 0.110891 0.099185
C -3.224177 -0.826276 0.301406
C -0.095994 1.177577 0.230167
C 1.252886 1.023478 -0.112297
C 1.696257 -0.206765 -0.603539
C 0.801450 -1.274037 -0.760425
C 3.131124 -0.354963 -0.938544
C 4.141427 0.617671 -0.278710
C 3.496461 1.993034 -0.105857
C 2.167676 2.158215 -0.026894
O 1.210403 -2.490028 -1.252573
O 3.467255 -1.304209 -1.649247
O 5.278529 0.749477 -1.129745
C 4.551802 0.029473 1.085283
C 5.148162 -1.357599 0.950619
C 4.667266 -2.409458 1.913095
O 6.012090 -1.601296 0.108390
O 4.268045 3.147342 -0.177250
C 5.382049 3.202792 0.704388
C -4.659751 -0.735053 0.665295
C -5.193343 -1.948415 1.379341
C -5.382095 0.351848 0.317425
C -6.846938 0.617443 0.590390
C -7.715241 0.383938 -0.661595
C -7.427217 1.299870 -1.844959
C -7.023413 2.026678 1.164527
H -1.229263 -3.129037 -0.881502
H -2.722373 1.192965 0.849655
H -0.450133 2.139263 0.600521
H 1.746743 3.157114 0.051165
H 2.114205 -2.389059 -1.632172
H 5.642232 -0.154012 -1.254677
H 3.687316 -0.027726 1.757843
H 5.317920 0.633458 1.581462
H 5.176264 -3.355584 1.709651
H 4.891092 -2.098637 2.936414
H 3.591312 -2.554720 1.787957
H 5.078152 2.986852 1.733742
H 6.182707 2.531088 0.384950
H 5.777103 4.222877 0.679332
H -4.567174 -2.187365 2.246587
H -6.210420 -1.819448 1.754526
H -5.206698 -2.816867 0.711667
H -4.891299 1.140384 -0.253756
H -7.215927 -0.067622 1.360383
H -7.597066 -0.656497 -0.990883
H -8.771052 0.499689 -0.385627
H -6.397328 1.193158 -2.197684
H -8.089765 1.046588 -2.679307
H -7.602660 2.349594 -1.592923
H -6.495068 2.122866 2.120031
H -8.081819 2.240967 1.347382
H -6.631693 2.798841 0.493833
SCF Energy (B3LYP/6-31G**)= -1361.69921777
Number of imaginary frequencies = 0

1a_c407
MMFF Geometry
C -1.466917 -2.182818 -0.510469
C -2.336174 0.352847 -0.051090
N -2.795509 -1.991108 -0.324399
C -0.502248 -1.170529 -0.484357
C -0.964006 0.138985 -0.243729
C -3.214944 -0.727066 -0.103516
C -0.044773 1.203977 -0.211116
C 1.324257 0.979301 -0.395503
C 1.777707 -0.325833 -0.618238
C 0.874592 -1.394007 -0.684666
C 3.232311 -0.531563 -0.804453
C 4.188195 0.522742 -0.187468
C 3.580546 1.910463 -0.363978
C 2.256427 2.106082 -0.452012
O 1.289821 -2.676309 -0.948673
O 3.618890 -1.537688 -1.400378
O 5.449646 0.477478 -0.859862
C 4.443540 0.212693 1.292163
C 4.617578 -1.267387 1.549416
C 5.943193 -1.894684 1.209190
O 3.682453 -1.933180 1.996297
O 4.417276 2.991824 -0.602229
C 5.274134 3.307016 0.491893
C -4.673479 -0.560906 0.098579
C -5.271157 -1.535656 1.072222
C -5.338453 0.401391 -0.576327
C -6.812225 0.754965 -0.631350
C -7.127367 2.021530 0.189193
C -6.926405 1.887312 1.693083
C -7.814057 -0.372801 -0.371694
H -1.183990 -3.218459 -0.687460
H -2.717155 1.349484 0.149674
H -0.406883 2.218109 -0.046305
H 1.855709 3.102010 -0.619397
H 2.250734 -2.663018 -1.157395
H 5.283319 0.740558 -1.783798
H 3.605630 0.546256 1.918292
H 5.339965 0.716221 1.670900
H 5.999133 -2.891333 1.656099
H 6.048713 -1.984025 0.126307
H 6.755384 -1.289088 1.618938
H 4.728153 3.307428 1.441445
H 6.121716 2.617657 0.533126
H 5.670383 4.313496 0.327808
H -4.524327 -1.905520 1.784847
H -6.046165 -1.067796 1.683877
H -5.692908 -2.398440 0.547276
H -4.756528 1.031770 -1.252378
H -6.984588 1.031102 -1.683018
H -6.501478 2.848895 -0.169900
H -8.166192 2.320692 0.000967
H -5.888470 1.647258 1.941219
H -7.172673 2.833712 2.185913
H -7.573939 1.116153 2.119312

H -7.553656 -1.270476 -0.942704
H -8.815938 -0.063434 -0.691528
H -7.891743 -0.644780 0.682787
SCF Energy (B3LYP/6-31G**)= -1361.68438337
Number of imaginary frequencies = 0

1a_c408
MMFF Geometry
C -1.497855 -2.276299 -0.393736
C -2.329823 0.197565 0.366045
N -2.808290 -2.110688 -0.092615
C -0.532986 -1.265609 -0.340722
C -0.974786 0.010718 0.055532
C -3.213292 -0.876191 0.275010
C -0.052034 1.070649 0.126441
C 1.302172 0.874529 -0.176359
C 1.737010 -0.396584 -0.558261
C 0.826514 -1.458960 -0.657508
C 3.175047 -0.591050 -0.854263
C 4.187462 0.422629 -0.267506
C 3.557767 1.820056 -0.221393
C 2.227258 2.008549 -0.156280
O 1.222286 -2.713141 -1.056203
O 3.495790 -1.592307 -1.497335
O 5.351964 0.475824 -1.091119
C 4.574251 -0.016732 1.153922
C 5.794537 -0.912697 1.248869
C 5.805287 -2.211513 0.488214
O 6.729509 -0.616986 1.998314
O 4.501453 2.831422 -0.254803
C 4.065185 4.181359 -0.137610
C -4.653858 -0.741352 0.597925
C -5.220126 -1.886925 1.393406
C -5.357351 0.326713 0.164621
C -6.834846 0.598460 0.360949
C -7.504886 0.788001 -1.013857
C -9.018124 0.938027 -0.935146
C -7.001663 1.823169 1.263166
H -1.231290 -3.288600 -0.690546
H -2.691067 1.168936 0.689535
H -0.401392 2.060197 0.419871
H 1.782873 2.996888 -0.109894
H 2.143538 -2.658383 -1.400620
H 5.323710 1.312262 -1.589283
H 3.747012 -0.521824 1.667482
H 4.812621 0.867195 1.762308
H 6.610887 -2.846658 0.868794
H 4.859696 -2.737455 0.640573
H 5.980908 -2.030145 -0.573230
H 3.427917 4.459676 -0.982950
H 3.549459 4.344302 0.814166
H 4.950524 4.823651 -0.156651
H -4.522434 -2.196520 2.180472
H -6.151750 -1.629599 1.903422
H -5.413576 -2.747600 0.744523
H -4.847533 1.085510 -0.429673
H -7.322276 -0.249187 0.852316
H -7.087557 1.665149 -1.524733
H -7.281261 -0.079949 -1.647867
H -9.304430 1.862957 -0.426349
H -9.444148 0.970587 -1.943178
H -9.469997 0.094305 -0.404229
H -6.458191 1.692061 2.205985
H -8.053407 1.987581 1.517410
H -6.623986 2.731924 0.781067
SCF Energy (B3LYP/6-31G**)= -1361.69891067
Number of imaginary frequencies = 0

1a_c409
MMFF Geometry
C -1.595609 -1.854186 -0.574285
C -2.272897 0.524769 0.555357
N -2.898525 -1.644922 -0.267050
C -0.565461 -0.934089 -0.355063
C -0.928578 0.295256 0.228118
C -3.222228 -0.461688 0.299633
C 0.059560 1.269204 0.462109
C 1.401617 1.031489 0.138381
C 1.758086 -0.199043 -0.424503
C 0.783483 -1.171216 -0.688175
C 3.178028 -0.425822 -0.768974
C 4.255690 0.450472 -0.099693
C 3.709078 1.832214 0.204717
C 2.398617 2.081894 0.350608
O 1.098007 -2.371894 -1.277327
O 3.478496 -1.305104 -1.582183
O 5.330710 0.576696 -1.046878
C 4.811851 -0.171483 1.183285
C 4.979680 -1.667940 1.078098
C 6.280447 -2.192774 0.532628
O 4.059202 -2.423164 1.393084
O 4.642353 2.812347 0.507199
C 4.866165 3.673344 -0.609922
C -4.654316 -0.262345 0.631308
C -4.953214 0.255089 2.007366
C -5.566620 -0.537848 -0.325624
C -7.079590 -0.452381 -0.352878
C -7.548151 0.692657 -1.276404
C -7.188776 2.094170 -0.797666
C -7.817485 -0.431205 0.986371
H -1.391434 -2.824818 -1.021460
H -2.579666 1.470293 0.991770
H -0.227116 2.224310 0.901052
H 2.058006 3.068674 0.650473
H 2.051449 -2.371408 -1.515666
H 5.228943 -0.157370 -1.686433
H 4.140421 0.004076 2.034484
H 5.782011 0.263851 1.452631
H 6.313975 -3.279465 0.651282
H 6.365697 -1.948971 -0.528089
H 7.115522 -1.759184 1.088367
H 5.206517 3.115592 -1.487868
H 3.961237 4.241070 -0.851400
H 5.649767 4.384796 -0.333371
H -5.441864 1.233106 1.955166
H -5.589183 -0.444188 2.557602
H -4.051897 0.377265 2.617810
H -5.173401 -0.897364 -1.281855
H -7.403678 -1.385277 -0.837874
H -7.121710 0.548696 -2.277734
H -8.637113 0.633325 -1.397985
H -6.106600 2.223589 -0.703818
H -7.549318 2.835914 -1.518052
H -7.650689 2.320912 0.167300
H -7.533900 -1.291210 1.602202
H -8.899449 -0.496063 0.823359
H -7.633277 0.482019 1.557531
SCF Energy (B3LYP/6-31G**)= -1361.68882867
Number of imaginary frequencies = 0

1a_c410
MMFF Geometry
C -1.733273 -2.488308 -0.201573
C -2.466179 0.128204 -0.296661
N -3.053177 -2.194075 -0.119655
C -0.711790 -1.542360 -0.330756
C -1.102988 -0.190293 -0.381876
C -3.405991 -0.891409 -0.158405
C -0.123163 0.809855 -0.527564
C 1.237177 0.484470 -0.601249
C 1.617334 -0.858481 -0.531941
C 0.655332 -1.870408 -0.418550
C 3.058827 -1.176010 -0.603313
C 4.057098 -0.069744 -0.186902
C 3.550949 1.293882 -0.653963
C 2.235990 1.535634 -0.819566
O 1.006548 -3.197959 -0.388439
O 3.389777 -2.305905 -0.964409
O 5.304983 -0.368164 -0.815568
C 4.213560 -0.130064 1.341550
C 5.610916 0.105477 1.877682
C 6.304980 1.401756 1.557420

O 6.139477 -0.718236 2.629503
O 4.555511 2.218071 -0.863680
C 4.212363 3.515169 -1.336878
C -4.859701 -0.615273 -0.070875
C -5.717650 -1.553236 -0.876864
C -5.321010 0.390313 0.703314
C -6.763315 0.784350 0.939347
C -7.053775 2.201105 0.410445
C -6.917605 2.316802 -1.102729
C -7.065530 0.712549 2.439792
H -1.508008 -3.552000 -0.159079
H -2.794432 1.161591 -0.352330
H -0.432704 1.852897 -0.585652
H 1.858668 2.503799 -1.130731
H 1.960074 -3.281068 -0.620130
H 5.127166 -0.525603 -1.759434
H 3.923891 -1.127838 1.701024
H 3.535095 0.566761 1.849666
H 6.690262 1.386762 0.536877
H 5.614922 2.236866 1.699873
H 7.146595 1.539514 2.243038
H 3.720305 3.458069 -2.313075
H 3.585121 4.041675 -0.610537
H 5.138475 4.084718 -1.457997
H -5.825355 -2.514206 -0.362864
H -6.718030 -1.156964 -1.067086
H -5.273691 -1.735485 -1.862629
H -4.605616 0.989631 1.267040
H -7.448725 0.088277 0.446017
H -8.077450 2.485171 0.684903
H -6.387026 2.931265 0.886501
H -7.565393 1.595378 -1.610803
H -7.207709 3.320575 -1.429497
H -5.887112 2.146623 -1.428828
H -6.879671 -0.294637 2.829522
H -8.115761 0.955413 2.634824
H -6.447364 1.415026 3.010414
SCF Energy (B3LYP/6-31G**)= -1361.69683594
Number of imaginary frequencies = 0

1a_c411
MMFF Geometry
C -1.878129 -2.128846 -0.425150
C -2.453795 0.527016 -0.388174
N -3.182182 -1.761509 -0.409115
C -0.797828 -1.240433 -0.422279
C -1.108159 0.131381 -0.405325
C -3.456919 -0.439993 -0.382587
C -0.064739 1.074767 -0.411874
C 1.279933 0.674817 -0.422246
C 1.583036 -0.688358 -0.430074
C 0.553194 -1.643001 -0.441582
C 3.002616 -1.102258 -0.439726
C 4.096547 -0.063715 -0.129171
C 3.631803 1.370811 -0.323538
C 2.332167 1.692037 -0.454427
O 0.815439 -2.992280 -0.468970
O 3.274242 -2.285334 -0.667941
O 5.125151 -0.338037 -1.092923
C 4.591225 -0.294215 1.307875
C 6.094024 -0.402354 1.422299
C 6.906839 0.862688 1.365443
O 6.636072 -1.503908 1.533229
O 4.670604 2.284657 -0.318707
C 4.397619 3.634755 -0.673803
C -4.895965 -0.081657 -0.371331
C -5.731876 -0.889216 -1.325042
C -5.349834 0.876137 0.465983
C -6.760995 1.377190 0.697171
C -7.808269 0.304099 1.052622
C -7.427627 -0.532778 2.267510
C -7.236660 2.262295 -0.457802
H -1.716495 -3.204733 -0.441688
H -2.718367 1.579955 -0.393347
H -0.311379 2.136208 -0.409133
H 2.006225 2.719046 -0.578098
H 1.783269 -3.126049 -0.590703
H 5.289560 -1.302968 -1.061422
H 4.180864 -1.226356 1.719972
H 4.242842 0.484596 1.998002
H 7.890009 0.681562 1.809262
H 7.035886 1.176419 0.327892
H 6.414744 1.649642 1.942356
H 3.954872 3.696753 -1.673164
H 3.753185 4.110452 0.072121
H 5.348025 4.176283 -0.691014
H -5.932404 -1.884493 -0.914483
H -6.689142 -0.417676 -1.552644
H -5.219512 -1.008707 -2.286948
H -4.629540 1.361993 1.125932
H -6.697915 2.037391 1.575069
H -8.761520 0.803152 1.269900
H -8.003755 -0.361393 0.206434
H -7.234195 0.103132 3.137230
H -8.244908 -1.214820 2.523391
H -6.537380 -1.139316 2.076623
H -6.507270 3.050664 -0.675226
H -8.183489 2.750505 -0.201814
H -7.398259 1.691522 -1.376828
SCF Energy (B3LYP/6-31G**)= -1361.69776536
Number of imaginary frequencies = 0

1a_c412
MMFF Geometry
C -1.780622 -1.283982 -0.570482
C -2.122736 1.297936 0.201608
N -3.037546 -0.858555 -0.298171
C -0.634826 -0.491022 -0.478902
C -0.823406 0.844627 -0.078216
C -3.206331 0.428323 0.084828
C 0.290808 1.698004 0.026290
C 1.584486 1.234082 -0.242253
C 1.765543 -0.094283 -0.634212
C 0.665472 -0.952238 -0.766459
C 3.143812 -0.570579 -0.892024
C 4.319723 0.206888 -0.247949
C 3.983762 1.697349 -0.191010
C 2.720790 2.149093 -0.184882
O 0.815092 -2.258932 -1.164125
O 3.288204 -1.618013 -1.525404
O 5.484664 0.031188 -1.052113
C 4.549070 -0.357921 1.167408
C 4.833533 -1.846675 1.146466
C 4.101404 -2.701079 2.145461
O 5.652685 -2.328266 0.364046
O 4.989560 2.651246 -0.297540
C 6.060864 2.527749 0.628966
C -4.604237 0.854138 0.385652
C -4.778094 2.010346 1.333538
C -5.637831 0.179652 -0.165546
C -7.116434 0.458939 -0.011570
C -7.779499 -0.500776 0.995266
C -7.750315 -1.976154 0.614744
C -7.802373 0.426601 -1.381211
H -1.717432 -2.325950 -0.876977
H -2.278475 2.330886 0.495169
H 0.143143 2.737355 0.318435
H 2.524811 3.218038 -0.188971
H 1.731220 -2.382123 -1.505631
H 5.647690 -0.935747 -1.103337
H 3.672092 -0.180458 1.801722
H 5.412138 0.100800 1.659969
H 4.399096 -3.746285 2.024855
H 4.351167 -2.375261 3.158056
H 3.024754 -2.620038 1.976070
H 5.685236 2.454759 1.654696
H 6.706048 1.679128 0.388452
H 6.668724 3.434894 0.559209
H -4.593186 2.960047 0.820957
H -5.779160 2.054299 1.770971
H -4.089028 1.928150 2.181969
H -5.411072 -0.667101 -0.816601

H -7.263184 1.475644 0.370510
H -7.295287 -0.385232 1.973539
H -8.825825 -0.200616 1.134115
H -6.726339 -2.345000 0.504854
H -8.233454 -2.570415 1.397464
H -8.289603 -2.161234 -0.318612
H -7.391551 1.202911 -2.036916
H -8.877193 0.611658 -1.280681
H -7.669539 -0.535120 -1.888069
SCF Energy (B3LYP/6-31G**)= -1361.69985956
Number of imaginary frequencies = 0

1a_c413
MMFF Geometry
C -1.795477 -2.387501 -0.073038
C -2.513564 0.230971 0.045541
N -3.111843 -2.095112 0.059320
C -0.770334 -1.439122 -0.151270
C -1.153953 -0.087418 -0.086626
C -3.458458 -0.791302 0.107361
C -0.168278 0.913632 -0.157813
C 1.191089 0.590809 -0.284672
C 1.567492 -0.752719 -0.343237
C 0.594745 -1.764047 -0.287839
C 3.002195 -1.085317 -0.475873
C 4.058850 0.009005 -0.235827
C 3.501002 1.417583 -0.363612
C 2.179230 1.666459 -0.382177
O 0.928526 -3.095834 -0.358584
O 3.319152 -2.247756 -0.747555
O 5.017740 -0.192197 -1.285654
C 4.682771 -0.214216 1.151217
C 6.193545 -0.239034 1.140114
C 6.927991 1.070595 1.041651
O 6.802714 -1.309767 1.184702
O 4.484534 2.388735 -0.425112
C 4.108923 3.726679 -0.728731
C -4.907880 -0.514262 0.256331
C -5.594174 -1.392522 1.265330
C -5.495178 0.440976 -0.496563
C -6.944089 0.879918 -0.555894
C -7.412830 1.671914 0.678919
C -6.581742 2.920767 0.946991
C -7.907209 -0.264035 -0.887509
H -1.575658 -3.452329 -0.114983
H -2.832707 1.266521 0.113195
H -0.472252 1.959208 -0.114613
H 1.787912 2.675071 -0.458632
H 1.888541 -3.172890 -0.563158
H 5.237567 -1.146552 -1.286267
H 4.359862 -1.173937 1.577565
H 4.350415 0.533521 1.882397
H 7.953086 0.938607 1.399651
H 6.952992 1.406700 0.003383
H 6.442935 1.819731 1.672362
H 3.582511 3.779500 -1.687214
H 3.502878 4.152902 0.076895
H 5.023250 4.321555 -0.812072
H -6.560632 -0.999442 1.583799
H -5.756668 -2.395613 0.856856
H -4.989917 -1.483334 2.175610
H -4.874240 0.980797 -1.213278
H -7.006991 1.568763 -1.411482
H -8.454840 1.981279 0.527837
H -7.405731 1.042990 1.575265
H -6.567695 3.578201 0.071903
H -7.008463 3.482215 1.784399
H -5.549427 2.668802 1.208140
H -7.550229 -0.844020 -1.745720
H -8.893954 0.135629 -1.147475
H -8.048983 -0.950420 -0.048629
SCF Energy (B3LYP/6-31G**)= -1361.69786356
Number of imaginary frequencies = 0

1a_c414
MMFF Geometry
C -1.531044 -2.457358 -0.434935
C -2.456029 0.098383 -0.392340
N -2.870814 -2.266946 -0.370688
C -0.580601 -1.432661 -0.479248
C -1.071122 -0.113492 -0.459435
C -3.319786 -0.993913 -0.340968
C -0.165497 0.961904 -0.516588
C 1.217556 0.742826 -0.572675
C 1.699372 -0.568103 -0.577110
C 0.809729 -1.652164 -0.548503
C 3.164483 -0.781168 -0.615259
C 4.085017 0.373619 -0.150595
C 3.458764 1.721888 -0.526724
C 2.133072 1.878262 -0.694431
O 1.255076 -2.951810 -0.582360
O 3.571476 -1.894837 -0.951439
O 5.355722 0.267963 -0.791969
C 4.262865 0.294166 1.374239
C 5.454288 -0.515597 1.849272
C 5.570163 -1.956101 1.428372
O 6.273011 -0.022510 2.630196
O 4.400079 2.726089 -0.668178
C 3.954990 4.050875 -0.938799
C -4.791632 -0.832370 -0.275365
C -5.561416 -1.785574 -1.149927
C -5.347499 0.094668 0.533972
C -6.823738 0.351292 0.749464
C -7.301488 1.606293 -0.006414
C -6.658983 2.918948 0.425539
C -7.130842 0.425071 2.248520
H -1.227740 -3.502182 -0.450932
H -2.858827 1.106599 -0.394653
H -0.550761 1.981182 -0.518385
H 1.689058 2.835038 -0.947185
H 2.216683 -2.950216 -0.795956
H 5.400861 0.961290 -1.474500
H 3.369096 -0.101135 1.872333
H 4.414277 1.303647 1.782043
H 5.896785 -2.024574 0.389608
H 6.310923 -2.458201 2.057984
H 4.610924 -2.460587 1.567387
H 3.445963 4.100857 -1.906563
H 3.308792 4.418018 -0.135037
H 4.835584 4.698152 -0.986898
H -5.618413 -2.775121 -0.684428
H -6.580534 -1.444329 -1.350644
H -5.080370 -1.890269 -2.129526
H -4.693663 0.719816 1.141860
H -7.407361 -0.493294 0.365954
H -7.120834 1.468537 -1.080319
H -8.388527 1.698487 0.111999
H -5.574001 2.902789 0.286840
H -7.056861 3.742241 -0.177040
H -6.873141 3.145409 1.473910
H -6.892968 -0.526182 2.738307
H -8.193842 0.628356 2.416695
H -6.552052 1.206971 2.751592
SCF Energy (B3LYP/6-31G**)= -1361.69782325
Number of imaginary frequencies = 0

1a_c415
MMFF Geometry
C 1.716513 2.101584 -0.478946
C 2.271108 -0.558133 -0.437158
N 3.017626 1.724057 -0.484148
C 0.628682 1.222486 -0.452875
C 0.928829 -0.151107 -0.433681
C 3.282106 0.400305 -0.454676
C -0.121444 -1.085249 -0.412170
C -1.462609 -0.674367 -0.407608
C -1.760338 0.691289 -0.424565
C -0.720107 1.636156 -0.447731
C -3.180057 1.123444 -0.406259
C -4.306900 0.072414 -0.405604
C -3.811772 -1.355133 -0.403438
C -2.514906 -1.684665 -0.371877

O -0.965853 2.989244 -0.462930
O -3.432159 2.333157 -0.425730
O -4.959675 0.311171 -1.673584
C -5.319093 0.387412 0.705665
C -4.897897 -0.048580 2.093553
C -3.749435 0.672605 2.748216
O -5.483307 -0.957465 2.682893
O -4.743179 -2.377600 -0.286176
C -5.572805 -2.515128 -1.438400
C 4.718192 0.030255 -0.465415
C 5.545033 0.826281 -1.436460
C 5.177820 -0.927702 0.368564
C 6.588170 -1.440152 0.578653
C 7.650517 -0.374737 0.911762
C 7.297437 0.470821 2.128960
C 7.036835 -2.334473 -0.580017
H 1.564049 3.178669 -0.497928
H 2.526012 -1.613591 -0.439634
H 0.114490 -2.149361 -0.397766
H -2.228185 -2.731708 -0.316317
H -1.937669 3.137327 -0.486011
H -5.200844 1.257309 -1.694443
H -6.278341 -0.099117 0.483023
H -5.556293 1.457928 0.746441
H -3.813132 0.544753 3.832763
H -2.801777 0.261063 2.397584
H -3.804885 1.742140 2.531060
H -6.379609 -1.777030 -1.422766
H -4.994312 -2.441547 -2.365796
H -6.030556 -3.508264 -1.402878
H 5.760671 1.821673 -1.033916
H 6.494417 0.345603 -1.677565
H 5.017930 0.945768 -2.390370
H 4.464371 -1.405232 1.042035
H 6.533942 -2.095876 1.460488
H 8.602870 -0.881175 1.115470
H 7.837685 0.285262 0.059412
H 7.112973 -0.159509 3.004670
H 8.124768 1.146788 2.368120
H 6.409452 1.084300 1.950101
H 6.297110 -3.117376 -0.781701
H 7.983524 -2.829819 -0.337579
H 7.188047 -1.769183 -1.504188
SCF Energy (B3LYP/6-31G**)= -1361.69222265
Number of imaginary frequencies = 0

1a_c416
MMFF Geometry
C 2.005710 1.931219 -0.085865
C 2.418618 -0.717939 0.342267
N 3.279419 1.503449 0.089178
C 0.876951 1.107905 -0.061391
C 1.103287 -0.260858 0.160018
C 3.481626 0.182997 0.302193
C 0.007699 -1.141599 0.191920
C -1.305531 -0.681017 0.011215
C -1.530096 0.681309 -0.205833
C -0.442843 1.571828 -0.245576
C -2.920041 1.170080 -0.389141
C -4.103450 0.184813 -0.280650
C -3.676938 -1.257131 -0.112293
C -2.404930 -1.639380 0.052492
O -0.613810 2.919712 -0.460447
O -3.089693 2.372616 -0.619719
O -4.763824 0.312621 -1.544695
C -4.971578 0.665139 0.896236
C -6.406157 0.200607 0.813409
C -6.910890 -0.702607 1.906718
O -7.150272 0.602422 -0.082752
O -4.671687 -2.219033 0.010475
C -5.262582 -2.546534 -1.249667
C 4.895160 -0.249415 0.505139
C 5.107215 -1.451765 1.381884
C 5.888193 0.463948 -0.071454
C 7.387668 0.254158 -0.066579
C 7.880100 -1.145370 -0.483738
C 7.374342 -1.580742 -1.853358
C 8.006420 0.690701 1.263844
H 1.912920 3.002174 -0.253479
H 2.603771 -1.775532 0.499363
H 0.185821 -2.203648 0.359537
H -2.173405 -2.687934 0.218940
H -1.569286 3.094878 -0.620909
H -5.331374 1.107026 -1.503335
H -5.026625 1.761326 0.932470
H -4.536510 0.360542 1.856234
H -7.880470 -1.118873 1.619842
H -6.214925 -1.528370 2.068141
H -7.023119 -0.126402 2.828300
H -6.048200 -1.829699 -1.502925
H -4.516844 -2.604461 -2.050284
H -5.730798 -3.530430 -1.150398
H 4.928817 -2.374901 0.820757
H 6.114850 -1.499599 1.798089
H 4.433609 -1.431630 2.246414
H 5.600912 1.338328 -0.661196
H 7.782869 0.947117 -0.824366
H 8.977338 -1.135004 -0.515658
H 7.609346 -1.904219 0.256357
H 7.638845 -0.846637 -2.621045
H 7.826119 -2.538169 -2.132362
H 6.288373 -1.712651 -1.859368
H 7.692130 1.706340 1.529398
H 9.099960 0.690875 1.196729
H 7.726515 0.026613 2.086878
SCF Energy (B3LYP/6-31G**)= -1361.69758408
Number of imaginary frequencies = 0

1a_c417
MMFF Geometry
C -1.639310 -2.354102 -0.192719
C -2.345012 0.261912 0.011121
N -2.955325 -2.059816 -0.062206
C -0.608194 -1.409426 -0.229853
C -0.986164 -0.059408 -0.121259
C -3.295772 -0.756455 0.028618
C 0.004880 0.937294 -0.144822
C 1.362820 0.612097 -0.278247
C 1.737872 -0.730037 -0.387776
C 0.757563 -1.736928 -0.363408
C 3.176388 -1.072039 -0.516060
C 4.234275 0.047269 -0.567102
C 3.658163 1.439141 -0.449421
C 2.352559 1.684333 -0.285925
O 1.080657 -3.070092 -0.462193
O 3.496655 -2.261581 -0.614688
O 4.776900 -0.094054 -1.899955
C 5.364085 -0.251141 0.429574
C 5.053967 0.101173 1.869526
C 4.009227 -0.708253 2.591307
O 5.647606 1.012956 2.446066
O 4.534588 2.512177 -0.364994
C 5.244341 2.752946 -1.578729
C -4.745025 -0.476952 0.174694
C -5.445305 -1.387445 1.144694
C -5.320181 0.508993 -0.547355
C -6.765824 0.959040 -0.602724
C -7.240525 1.707849 0.656515
C -6.404307 2.940663 0.977627
C -7.732896 -0.165753 -0.984577
H -1.425171 -3.418077 -0.269085
H -2.658423 1.296496 0.113486
H -0.291194 1.982756 -0.057909
H 2.010935 2.707647 -0.152814
H 2.053209 -3.155367 -0.579531
H 5.071921 -1.020760 -1.989062
H 6.268548 0.301482 0.141476
H 5.664210 -1.306063 0.398395
H 4.165639 -0.620815 3.670448
H 3.013373 -0.336076 2.346143
H 4.099614 -1.763440 2.321963
H 6.090458 2.066565 -1.673218

H 4.587437 2.685382 -2.452824
H 5.645143 3.770049 -1.534296
H -4.849841 -1.515108 2.056344
H -6.412404 -1.000617 1.468818
H -5.609688 -2.373928 0.698287
H -4.689638 1.072033 -1.237398
H -6.817113 1.679384 -1.432765
H -8.279301 2.029173 0.508152
H -7.245008 1.046215 1.529015
H -6.378685 3.629862 0.127601
H -6.834919 3.473495 1.831577
H -5.375839 2.672695 1.238024
H -7.371954 -0.715899 -1.860593
H -8.714911 0.249494 -1.237949
H -7.886115 -0.881741 -0.172897
SCF Energy (B3LYP/6-31G**)= -1361.69226664
Number of imaginary frequencies = 0

1a_c418
MMFF Geometry
C 2.063538 1.714358 0.020403
C 2.361998 -0.983359 -0.051435
N 3.320374 1.209532 0.058110
C 0.897522 0.947676 -0.050852
C 1.064606 -0.446806 -0.086086
C 3.465725 -0.134896 0.020442
C -0.071139 -1.272859 -0.155143
C -1.366155 -0.733253 -0.188519
C -1.531515 0.654124 -0.152382
C -0.404001 1.491484 -0.087091
C -2.901787 1.225116 -0.179823
C -4.127217 0.286379 -0.188731
C -3.766229 -1.179084 -0.296268
C -2.509119 -1.637131 -0.262566
O -0.516572 2.861995 -0.052201
O -3.020794 2.455489 -0.187620
O -4.830517 0.663517 -1.377596
C -4.922638 0.586361 1.094527
C -6.380558 0.203784 1.000940
C -6.885034 -0.856710 1.942606
O -7.139229 0.788211 0.225612
O -4.801647 -2.105239 -0.295880
C -5.458467 -2.179290 -1.563734
C 4.864796 -0.653475 0.043444
C 5.104548 -1.959932 -0.660335
C 5.825233 0.078902 0.650483
C 7.301870 -0.193640 0.844993
C 8.146919 0.001181 -0.427553
C 8.031864 1.399578 -1.021964
C 7.589932 -1.544975 1.506734
H 2.017178 2.800908 0.050445
H 2.499512 -2.059582 -0.067062
H 0.060738 -2.354296 -0.184747
H -2.323310 -2.707315 -0.293446
H -1.467789 3.102552 -0.133963
H -5.356342 1.460667 -1.170319
H -4.922023 1.660137 1.324680
H -4.464940 0.098793 1.964268
H -7.884813 -1.174867 1.634584
H -6.224912 -1.726296 1.923327
H -6.931396 -0.448417 2.955063
H -6.217111 -1.396762 -1.649137
H -4.749447 -2.125515 -2.397319
H -5.970563 -3.144788 -1.615751
H 4.547380 -2.005953 -1.603208
H 6.150840 -2.110181 -0.931009
H 4.795844 -2.800295 -0.029953
H 5.523794 1.029316 1.098501
H 7.645452 0.559981 1.569245
H 9.201635 -0.181031 -0.185373
H 7.875546 -0.732496 -1.194065
H 8.298289 2.162869 -0.283997
H 8.711688 1.501683 -1.874011
H 7.018010 1.602320 -1.380206
H 6.951362 -1.699652 2.383416
H 8.631074 -1.584318 1.846738
H 7.443613 -2.387163 0.825488
SCF Energy (B3LYP/6-31G**)= -1361.69766458
Number of imaginary frequencies = 0

1a_c419
MMFF Geometry
C -1.554844 -2.426347 -0.175053
C -2.449019 0.128355 -0.428874
N -2.891582 -2.212390 -0.123811
C -0.592508 -1.426651 -0.347102
C -1.067415 -0.107599 -0.479607
C -3.325185 -0.939008 -0.242508
C -0.150428 0.942451 -0.669364
C 1.228116 0.699512 -0.706881
C 1.695669 -0.610413 -0.563193
C 0.794467 -1.671775 -0.400207
C 3.160437 -0.844115 -0.583955
C 4.094083 0.349904 -0.263498
C 3.474846 1.644692 -0.779415
C 2.157836 1.793297 -0.977135
O 1.224500 -2.971537 -0.282593
O 3.564094 -1.992998 -0.774025
O 5.340733 0.151207 -0.926559
C 4.273511 0.371761 1.265873
C 5.619834 -0.127754 1.741886
C 5.929712 -1.593643 1.581374
O 6.419334 0.642074 2.279098
O 4.316685 2.653183 -1.231800
C 5.170164 3.196910 -0.225198
C -4.794670 -0.752223 -0.186134
C -5.581502 -1.792730 -0.937177
C -5.331464 0.268467 0.516320
C -6.801863 0.574364 0.715379
C -7.154626 1.874358 -0.029671
C -8.639627 2.208708 0.004369
C -7.098190 0.663683 2.215653
H -1.264489 -3.469636 -0.069831
H -2.839531 1.134404 -0.548070
H -0.521165 1.959877 -0.791617
H 1.765836 2.717762 -1.392768
H 2.185211 -3.007932 -0.495229
H 5.215706 0.393876 -1.861957
H 3.502132 -0.211577 1.785099
H 4.158470 1.388608 1.661742
H 6.812789 -1.844841 2.176026
H 5.089687 -2.189496 1.947316
H 6.132137 -1.830134 0.535595
H 4.593271 3.510426 0.650891
H 5.953137 2.488800 0.057547
H 5.656617 4.082828 -0.644039
H -5.649760 -2.717972 -0.355415
H -6.596468 -1.464944 -1.176020
H -5.106041 -2.022007 -1.898138
H -4.665360 0.952559 1.042712
H -7.421689 -0.234566 0.315947
H -6.590373 2.718398 0.387183
H -6.850781 1.783065 -1.080695
H -8.975436 2.444481 1.018283
H -8.840913 3.084068 -0.621659
H -9.239038 1.375749 -0.376199
H -6.763999 -0.241657 2.735390
H -8.172118 0.764837 2.401277
H -6.593522 1.521085 2.675056
SCF Energy (B3LYP/6-31G**)= -1361.69308652
Number of imaginary frequencies = 0

1a_c420
MMFF Geometry
C -1.586606 -2.271799 -0.236418
C -2.400102 0.257535 0.339109
N -2.893941 -2.076224 0.060058
C -0.615814 -1.265931 -0.266410
C -1.048268 0.039752 0.035511
C -3.289892 -0.814832 0.335198
C -0.120241 1.097128 0.020726
C 1.229698 0.869231 -0.273446

C 1.656049 -0.430213 -0.564666
C 0.740314 -1.491587 -0.577186
C 3.093964 -0.654355 -0.852311
C 4.111702 0.398575 -0.346714
C 3.485234 1.788374 -0.392481
C 2.161166 1.991400 -0.356331
O 1.127278 -2.774444 -0.881605
O 3.419753 -1.705674 -1.406922
O 5.250054 0.389012 -1.205386
C 4.492784 -0.009653 1.088483
C 5.867517 -0.628279 1.215479
C 6.090242 -1.991556 0.613413
O 6.763331 -0.043450 1.828607
O 4.301918 2.880062 -0.660165
C 5.303211 3.116851 0.329105
C -4.727677 -0.647415 0.655462
C -5.295623 -1.726650 1.537709
C -5.427699 0.389618 0.147569
C -6.902265 0.684767 0.331585
C -7.579755 0.775357 -1.049637
C -9.091548 0.940555 -0.973592
C -7.056132 1.974501 1.140689
H -1.327891 -3.305185 -0.457799
H -2.753654 1.253007 0.589869
H -0.460312 2.109420 0.237896
H 1.757321 2.996794 -0.440777
H 2.048234 -2.753435 -1.229331
H 5.012753 0.884524 -2.010147
H 3.772525 -0.711770 1.527985
H 4.475069 0.856067 1.762377
H 7.032337 -2.403353 0.986802
H 5.280928 -2.662521 0.912428
H 6.142794 -1.925950 -0.474472
H 4.860937 3.174694 1.328946
H 6.085858 2.354879 0.293332
H 5.767289 4.082736 0.108624
H -4.595021 -1.980555 2.341939
H -6.222762 -1.426231 2.032090
H -5.497735 -2.632377 0.956294
H -4.917142 1.098850 -0.504592
H -7.391655 -0.120658 0.887753
H -7.160404 1.609187 -1.627062
H -7.365286 -0.138955 -1.618277
H -9.369138 1.902734 -0.533710
H -9.523671 0.900442 -1.978752
H -9.545117 0.141779 -0.378480
H -6.507559 1.910716 2.087503
H -8.105266 2.164184 1.388026
H -6.676075 2.842261 0.589831
SCF Energy (B3LYP/6-31G**)= -1361.69307007
Number of imaginary frequencies = 0

1a_c421
MMFF Geometry
C -1.763079 -1.506789 -0.490971
C -2.139297 1.098741 0.177965
N -3.027241 -1.084423 -0.248991
C -0.626533 -0.698155 -0.420299
C -0.832869 0.649557 -0.074787
C -3.212461 0.213479 0.085006
C 0.271379 1.518848 0.001601
C 1.573653 1.061428 -0.241802
C 1.772914 -0.281158 -0.570515
C 0.681572 -1.155438 -0.677413
C 3.157317 -0.752710 -0.803622
C 4.322598 0.072162 -0.204867
C 3.967590 1.563886 -0.223673
C 2.695325 2.001363 -0.217037
O 0.845345 -2.474130 -1.027636
O 3.304555 -1.817727 -1.406062
O 5.503880 -0.123852 -0.981578
C 4.569783 -0.381706 1.242879
C 5.593593 -1.488012 1.412528
C 5.384172 -2.791267 0.689129
O 6.540710 -1.348137 2.191438
O 5.086117 2.377972 -0.248170
C 4.909510 3.789198 -0.188345
C -4.616865 0.635624 0.358073
C -4.809433 1.808799 1.280895
C -5.637069 -0.056269 -0.196202
C -7.122148 0.209500 -0.071460
C -7.763141 -0.882654 0.803362
C -9.237920 -0.639279 1.093117
C -7.740965 0.272241 -1.471625
H -1.685696 -2.559653 -0.754018
H -2.309675 2.139850 0.431772
H 0.106491 2.566185 0.253077
H 2.444685 3.056637 -0.216658
H 1.771731 -2.606065 -1.335332
H 5.647510 0.681756 -1.509685
H 3.644496 -0.703015 1.736629
H 4.950737 0.462348 1.835105
H 6.040839 -3.553273 1.119683
H 4.351228 -3.123810 0.817234
H 5.627918 -2.683409 -0.368866
H 4.365486 4.151382 -1.066430
H 4.401936 4.080779 0.736721
H 5.900530 4.252448 -0.190630
H -4.637286 2.749942 0.748484
H -5.811156 1.847018 1.716830
H -4.120546 1.755234 2.131808
H -5.396048 -0.915418 -0.826221
H -7.300520 1.182874 0.396667
H -7.651330 -1.866068 0.329089
H -7.232616 -0.935658 1.763057
H -9.843019 -0.709529 0.184657
H -9.610474 -1.391046 1.796488
H -9.393198 0.347894 1.539579
H -7.214317 0.999415 -2.100380
H -8.789407 0.583378 -1.426968
H -7.698226 -0.700574 -1.974315
SCF Energy (B3LYP/6-31G**)= -1361.69974284
Number of imaginary frequencies = 0

1a_c422
MMFF Geometry
C -1.588546 -1.793723 -0.922068
C -2.255941 0.803389 -0.467111
N -2.905064 -1.480297 -0.850816
C -0.540203 -0.878486 -0.780243
C -0.898071 0.464470 -0.546103
C -3.221610 -0.188980 -0.619968
C 0.109123 1.437523 -0.408852
C 1.462393 1.087687 -0.479311
C 1.811069 -0.250643 -0.693723
C 0.822405 -1.227477 -0.865502
C 3.251188 -0.589502 -0.758422
C 4.242247 0.359923 -0.035663
C 3.783499 1.801406 -0.230117
C 2.496121 2.122339 -0.427490
O 1.138952 -2.538276 -1.126382
O 3.594242 -1.617768 -1.343107
O 5.548500 0.206864 -0.597554
C 4.336334 0.003347 1.452491
C 4.349129 -1.490487 1.687759
C 5.635698 -2.234622 1.447955
O 3.320601 -2.072048 2.036100
O 4.734390 2.802805 -0.368781
C 5.516940 3.018008 0.802518
C -4.666526 0.121764 -0.539653
C -5.487460 -0.380938 -1.691776
C -5.141503 0.745495 0.560090
C -6.540459 1.190268 0.940990
C -7.133568 0.293654 2.047607
C -7.394917 -1.150611 1.639025
C -7.537313 1.432739 -0.194043
H -1.387992 -2.847598 -1.103915
H -2.562593 1.831138 -0.297791
H -0.170860 2.478493 -0.251541
H 2.205577 3.154165 -0.604704
H 2.111880 -2.612396 -1.248921
H 5.489439 0.499630 -1.525636

H 3.481006 0.404404 2.011922
H 5.238656 0.413818 1.919276
H 5.559360 -3.239231 1.873512
H 5.827594 -2.315923 0.376359
H 6.461208 -1.715217 1.940952
H 4.891780 3.054439 1.701178
H 6.290204 2.251260 0.899834
H 6.017468 3.985197 0.697852
H -4.908456 -0.997568 -2.388037
H -6.314574 -1.004669 -1.339595
H -5.882812 0.456271 -2.275275
H -4.422754 0.976059 1.350381
H -6.398237 2.180424 1.399787
H -6.457365 0.291243 2.912443
H -8.075665 0.733340 2.398675
H -6.474558 -1.655448 1.331381
H -7.808838 -1.706662 2.486753
H -8.116810 -1.213557 0.820062
H -7.111090 2.095089 -0.955098
H -8.437536 1.923188 0.193968
H -7.862249 0.510342 -0.680667
SCF Energy (B3LYP/6-31G**)= -1361.68416057
Number of imaginary frequencies = 0

1a_c423
MMFF Geometry
C 1.489546 2.355519 0.054193
C 2.198138 -0.263349 -0.101963
N 2.801312 2.044037 0.192159
C 0.465407 1.428375 -0.164204
C 0.844361 0.075276 -0.241831
C 3.142282 0.740237 0.105388
C -0.138869 -0.906209 -0.462383
C -1.491023 -0.561984 -0.594524
C -1.863906 0.781920 -0.506536
C -0.893566 1.775651 -0.306528
C -3.297771 1.127568 -0.633163
C -4.362742 0.012313 -0.526978
C -3.798728 -1.360529 -0.860336
C -2.479149 -1.599927 -0.882846
O -1.223920 3.109296 -0.244880
O -3.622333 2.308290 -0.806042
O -5.333675 0.351734 -1.545408
C -5.084192 0.161185 0.822867
C -4.314065 -0.370422 2.009242
C -5.119742 -0.983000 3.124422
O -3.094747 -0.246813 2.102831
O -4.611919 -2.388957 -1.324418
C -5.881614 -2.563608 -0.717029
C 4.585873 0.441452 0.267384
C 5.242658 1.195654 1.389873
C 5.197959 -0.427249 -0.566414
C 6.653379 -0.847527 -0.634797
C 6.990592 -1.868538 0.466828
C 8.376785 -2.481475 0.318874
C 7.629700 0.330684 -0.702610
H 1.272129 3.418848 0.128361
H 2.511172 -1.302105 -0.140738
H 0.157321 -1.952189 -0.531338
H -2.114414 -2.582281 -1.171053
H -2.180066 3.203407 -0.452631
H -5.358551 1.328825 -1.590806
H -6.060308 -0.334393 0.788376
H -5.310732 1.214931 1.031981
H -4.448275 -1.345777 3.907621
H -5.787701 -0.229469 3.548644
H -5.697548 -1.827670 2.742194
H -5.787318 -2.650684 0.368862
H -6.573371 -1.764276 -0.992879
H -6.302511 -3.502904 -1.088638
H 4.584708 1.245144 2.265555
H 6.161781 0.718818 1.735302
H 5.481580 2.218278 1.079624
H 4.599371 -0.890413 -1.352415
H 6.757125 -1.369446 -1.597663
H 6.917074 -1.407657 1.458231
H 6.252673 -2.681009 0.446035
H 9.162511 -1.733258 0.459115
H 8.524291 -3.261445 1.072870
H 8.502086 -2.937309 -0.668271
H 7.261266 1.113154 -1.375277
H 8.599770 0.002781 -1.092026
H 7.811645 0.779332 0.277610
SCF Energy (B3LYP/6-31G**)= -1361.69104126
Number of imaginary frequencies = 0

1a_c424
MMFF Geometry
C -1.748881 -1.404880 -0.945931
C -2.157529 1.151351 -0.123415
N -3.024996 -0.984981 -0.771962
C -0.615582 -0.617657 -0.730206
C -0.838546 0.704031 -0.303965
C -3.226499 0.288888 -0.363198
C 0.262778 1.549689 -0.074134
C 1.576808 1.093708 -0.246871
C 1.791361 -0.223312 -0.658824
C 0.705329 -1.072419 -0.916572
C 3.186251 -0.695336 -0.816185
C 4.306445 0.066862 -0.067194
C 3.965388 1.560454 -0.000826
C 2.700103 2.013621 -0.062005
O 0.886422 -2.364117 -1.349193
O 3.372752 -1.715011 -1.482806
O 5.544039 -0.086285 -0.761552
C 4.434693 -0.497447 1.356955
C 5.434527 -1.626112 1.522146
C 5.273386 -2.869355 0.689067
O 6.319005 -1.556206 2.380101
O 5.088542 2.358648 0.125324
C 4.918479 3.763554 0.279179
C -4.645938 0.717543 -0.199149
C -4.936949 2.185704 -0.356239
C -5.594417 -0.212914 0.048690
C -7.077167 -0.002510 0.270190
C -7.492709 -0.665273 1.598344
C -8.941837 -0.397883 1.982026
C -7.847552 -0.564936 -0.926172
H -1.658742 -2.437161 -1.277557
H -2.338894 2.165803 0.216199
H 0.086394 2.577531 0.241730
H 2.458399 3.069232 -0.001360
H 1.833286 -2.485547 -1.592064
H 5.738854 0.757087 -1.208036
H 3.470985 -0.843280 1.750468
H 4.772823 0.294848 2.039755
H 5.888607 -3.669661 1.111265
H 4.231200 -3.196986 0.710923
H 5.600440 -2.686538 -0.335637
H 4.449245 4.198799 -0.608825
H 4.340750 3.990986 1.180790
H 5.910011 4.211991 0.390963
H -4.371371 2.609748 -1.193989
H -5.987316 2.390307 -0.578700
H -4.673574 2.730247 0.556438
H -5.285891 -1.258731 0.114646
H -7.307714 1.064399 0.350112
H -7.332947 -1.750057 1.550550
H -6.851539 -0.289228 2.406319
H -9.636755 -0.867628 1.280054
H -9.148176 -0.809195 2.975472
H -9.149304 0.676376 2.011131
H -7.486965 -0.128380 -1.864700
H -8.915192 -0.335492 -0.853250
H -7.739362 -1.653048 -0.998200
SCF Energy (B3LYP/6-31G**)= -1361.69981785
Number of imaginary frequencies = 0

1a_c425
MMFF Geometry
C -1.759259 -1.094466 -1.127272
C -2.145880 1.359129 -0.027049

N -3.031594 -0.686604 -0.904016
C -0.619300 -0.345725 -0.827133
C -0.830951 0.922736 -0.255834
C -3.222154 0.537127 -0.358698
C 0.277010 1.727567 0.068635
C 1.585279 1.279428 -0.151565
C 1.788733 0.017867 -0.715527
C 0.697292 -0.788468 -1.065292
C 3.180206 -0.445928 -0.919514
C 4.300971 0.204890 -0.069577
C 3.974489 1.678542 0.174049
C 2.720001 2.151886 0.136448
O 0.870262 -2.027706 -1.633041
O 3.371069 -1.393810 -1.683854
O 5.531308 0.121597 -0.786491
C 4.398300 -0.562642 1.263194
C 4.670397 -2.038683 1.051044
C 3.846360 -3.011828 1.849772
O 5.550576 -2.420354 0.279853
O 4.994204 2.615824 0.294785
C 5.977395 2.337237 1.283271
C -4.638753 0.957506 -0.148251
C -4.913195 2.437343 -0.151702
C -5.590780 0.010919 0.010280
C -7.073797 0.195475 0.244774
C -7.504789 -0.323373 1.632516
C -7.355907 -1.822999 1.858792
C -7.865020 -0.456738 -0.891559
H -1.678130 -2.083478 -1.573269
H -2.317748 2.332486 0.420666
H 0.113111 2.716746 0.495326
H 2.534595 3.214228 0.271007
H 1.811792 -2.119949 -1.908927
H 5.689507 -0.831204 -0.963481
H 3.469843 -0.459444 1.837875
H 5.218023 -0.198341 1.890304
H 4.144246 -4.035006 1.604841
H 4.009605 -2.840110 2.916427
H 2.789366 -2.884393 1.602866
H 5.511083 2.125700 2.250926
H 6.633923 1.518215 0.979225
H 6.597063 3.231595 1.399058
H -4.353048 2.936670 -0.950775
H -5.962544 2.679873 -0.333734
H -4.628999 2.883287 0.807167
H -5.279333 -1.034781 -0.028068
H -7.330252 1.259218 0.232549
H -6.931997 0.203922 2.406347
H -8.556831 -0.056243 1.795192
H -6.315058 -2.145712 1.766783
H -7.691720 -2.079726 2.869088
H -7.963660 -2.398488 1.154773
H -7.637632 0.028466 -1.847713
H -8.942362 -0.361341 -0.719070
H -7.630902 -1.521184 -1.000099
SCF Energy (B3LYP/6-31G**)= -1361.69960087
Number of imaginary frequencies = 0

1a_c426
MMFF Geometry
C -1.678749 -1.528136 -0.607263
C -2.153629 1.070610 0.032950
N -2.962099 -1.148283 -0.395210
C -0.571404 -0.679918 -0.521804
C -0.828978 0.664082 -0.188124
C -3.190680 0.146132 -0.078157
C 0.242238 1.572002 -0.093214
C 1.561231 1.155150 -0.306508
C 1.810650 -0.185235 -0.621922
C 0.755246 -1.096308 -0.751263
C 3.216875 -0.596356 -0.837192
C 4.322381 0.255588 -0.160440
C 3.933029 1.728343 -0.235574
C 2.654121 2.128408 -0.296067
O 0.969885 -2.405566 -1.106393
O 3.445550 -1.606114 -1.504232
O 5.561938 0.065733 -0.848098
C 4.528169 -0.190843 1.291790
C 4.475666 -1.694398 1.445678
C 5.690601 -2.490162 1.049193
O 3.450470 -2.240190 1.856173
O 4.924530 2.683995 -0.408515
C 5.820230 2.788704 0.694847
C -4.611172 0.537135 0.135636
C -5.009836 1.887616 -0.385506
C -5.443032 -0.346593 0.729195
C -6.898560 -0.255915 1.140195
C -7.820304 -1.025839 0.173838
C -7.895754 -0.463485 -1.239517
C -7.441322 1.133050 1.486242
H -1.558391 -2.579969 -0.858152
H -2.369787 2.099256 0.303719
H 0.039323 2.615750 0.143732
H 2.409801 3.182798 -0.391038
H 1.921646 -2.524595 -1.323304
H 5.436888 0.413051 -1.750461
H 3.750776 0.221737 1.948165
H 5.490749 0.143811 1.694283
H 5.595001 -3.512088 1.426942
H 5.780693 -2.519921 -0.038351
H 6.585498 -2.043964 1.490056
H 5.281394 2.806129 1.648311
H 6.553008 1.977470 0.679185
H 6.364981 3.732280 0.595596
H -6.030914 1.888068 -0.772733
H -4.921175 2.647299 0.397334
H -4.385555 2.193641 -1.233070
H -5.014585 -1.315232 1.004198
H -6.942915 -0.805560 2.093114
H -7.486222 -2.069765 0.112020
H -8.834073 -1.054189 0.592854
H -6.917006 -0.468352 -1.727873
H -8.570087 -1.076367 -1.846860
H -8.284118 0.558649 -1.246720
H -6.749749 1.675269 2.140051
H -8.391640 1.038719 2.024593
H -7.639941 1.749386 0.607314
SCF Energy (B3LYP/6-31G**)= -1361.68442270
Number of imaginary frequencies = 0

1a_c427
MMFF Geometry
C 1.798616 2.371363 -0.032393
C 2.471718 -0.256546 0.122137
N 3.112890 2.057341 0.068965
C 0.753935 1.441454 -0.061598
C 1.114989 0.085248 0.021433
C 3.436712 0.748312 0.135251
C 0.109472 -0.896990 0.002598
C -1.246752 -0.551136 -0.097638
C -1.605972 0.797217 -0.179806
C -0.609525 1.789285 -0.164847
C -3.041516 1.163516 -0.277888
C -4.122342 0.062337 -0.230799
C -3.556457 -1.340748 -0.209522
C -2.249062 -1.611274 -0.115834
O -0.914713 3.128222 -0.244989
O -3.331719 2.359966 -0.389065
O -4.836024 0.239483 -1.459444
C -4.989659 0.347398 1.008565
C -6.376574 -0.243580 0.920012
C -6.756495 -1.286369 1.936921
O -7.185570 0.164254 0.084854
O -4.451452 -2.401542 -0.150044
C -5.052980 -2.670231 -1.419028
C 4.884706 0.448659 0.247494
C 5.619133 1.337351 1.212573
C 5.434806 -0.536169 -0.495513
C 6.879426 -0.986882 -0.595690
C 7.277672 -1.853406 0.612734
C 8.646293 -2.504585 0.464551
C 7.859436 0.153954 -0.885601

H 1.597297 3.439009 -0.091038
H 2.772516 -1.296595 0.203004
H 0.393152 -1.947413 0.065245
H -1.914119 -2.643169 -0.054171
H -1.887555 3.223423 -0.362959
H -5.475645 0.966533 -1.328274
H -5.145606 1.425267 1.149413
H -4.494575 -0.000273 1.923811
H -7.692681 -1.765830 1.638035
H -5.981568 -2.052904 2.001904
H -6.889357 -0.809518 2.911027
H -5.910523 -2.012543 -1.584124
H -4.333615 -2.584179 -2.240963
H -5.423446 -3.699541 -1.398855
H 5.019258 1.517529 2.112491
H 6.554214 0.895988 1.562252
H 5.846775 2.303654 0.750472
H 4.784134 -1.091325 -1.172993
H 6.916928 -1.637293 -1.482169
H 7.271642 -1.260203 1.534010
H 6.533030 -2.648267 0.749739
H 9.445723 -1.757856 0.451451
H 8.835072 -3.176605 1.307982
H 8.704317 -3.093151 -0.456407
H 7.455346 0.843373 -1.635198
H 8.799701 -0.240822 -1.285923
H 8.107705 0.729173 0.010334
SCF Energy (B3LYP/6-31G**)= -1361.69615562
Number of imaginary frequencies = 0

1a_c428
MMFF Geometry
C -1.574327 -2.264490 -0.376583
C -2.384963 0.184853 0.478249
N -2.878345 -2.105881 -0.045183
C -0.605863 -1.258151 -0.309664
C -1.036573 0.005517 0.135952
C -3.273516 -0.883357 0.369350
C -0.109830 1.060730 0.223327
C 1.237807 0.871743 -0.111605
C 1.661869 -0.387245 -0.542633
C 0.746801 -1.443983 -0.658597
C 3.093448 -0.574998 -0.872344
C 4.119620 0.417154 -0.272830
C 3.494629 1.813630 -0.169454
C 2.166120 2.002628 -0.072688
O 1.131478 -2.685440 -1.105090
O 3.399143 -1.555661 -1.553363
O 5.268190 0.494615 -1.116646
C 4.532528 -0.068728 1.125930
C 5.752127 -0.969642 1.168298
C 5.744877 -2.243244 0.366101
O 6.702148 -0.700068 1.908618
O 4.440190 2.823621 -0.188320
C 4.009840 4.169943 -0.019157
C -4.707759 -0.752446 0.725826
C -5.262647 -1.925671 1.489186
C -5.410910 0.331117 0.331367
C -6.871375 0.632525 0.590454
C -7.742244 0.361087 -0.652060
C -7.437583 1.221325 -1.872474
C -7.024631 2.067480 1.104403
H -1.316279 -3.267293 -0.710816
H -2.737250 1.146078 0.839885
H -0.450899 2.040975 0.555216
H 1.725337 2.989852 0.014164
H 2.046138 -2.621506 -1.465144
H 5.232898 1.347069 -1.586407
H 3.714025 -0.588586 1.638754
H 4.784791 0.794577 1.757871
H 6.556025 -2.891912 0.710353
H 4.801047 -2.771986 0.519685
H 5.900475 -2.028045 -0.692143
H 3.357220 4.476785 -0.842628
H 3.512898 4.303030 0.947144
H 4.896356 4.810709 -0.034462
H -4.641979 -2.138100 2.367218
H -6.278097 -1.764218 1.856109
H -5.289283 -2.821196 0.858707
H -4.906019 1.086560 -0.271173
H -7.252608 -0.013153 1.388022
H -7.641333 -0.694150 -0.937003
H -8.796226 0.506133 -0.383031
H -6.409271 1.082543 -2.218578
H -8.103438 0.944250 -2.696564
H -7.595562 2.283544 -1.665215
H -6.495670 2.194975 2.055892
H -8.079453 2.307037 1.276284
H -6.619299 2.803991 0.402429
SCF Energy (B3LYP/6-31G**)= -1361.69844379
Number of imaginary frequencies = 0

1a_c429
MMFF Geometry
C 1.883055 2.096810 0.013863
C 2.320591 -0.562383 0.376226
N 3.150737 1.689843 0.262947
C 0.774155 1.248846 -0.071276
C 1.013528 -0.126014 0.115234
C 3.355641 0.366694 0.447141
C -0.058665 -1.032573 0.028675
C -1.363425 -0.589496 -0.224429
C -1.598671 0.778425 -0.392918
C -0.537638 1.693607 -0.333628
C -2.986308 1.232134 -0.642492
C -4.158163 0.282031 -0.321897
C -3.725586 -1.163188 -0.449089
C -2.447577 -1.560027 -0.359112
O -0.732599 3.040738 -0.523895
O -3.181640 2.379021 -1.058055
O -5.171380 0.573089 -1.297255
C -4.673279 0.669385 1.067939
C -5.684614 -0.290867 1.654016
C -7.054713 -0.347321 1.029458
O -5.402623 -0.973459 2.641025
O -4.738195 -2.104259 -0.549991
C -4.907830 -2.545326 -1.897485
C 4.752375 -0.049376 0.724040
C 4.951673 -0.987310 1.877954
C 5.723187 0.424121 -0.086711
C 7.224512 0.217703 -0.115326
C 7.639218 -0.626316 -1.339770
C 7.150078 -2.069586 -1.322584
C 7.901587 -0.277616 1.163236
H 1.777054 3.171086 -0.121477
H 2.532027 -1.619100 0.508565
H 0.131200 -2.097533 0.160751
H -2.198875 -2.617132 -0.387032
H -1.658416 3.189977 -0.819804
H -5.110077 1.531656 -1.484882
H -5.142240 1.661605 1.049922
H -3.840831 0.735172 1.781451
H -7.721835 -0.931295 1.670163
H -7.460295 0.663792 0.942048
H -7.010950 -0.821089 0.047876
H -5.112516 -1.708055 -2.572143
H -4.027650 -3.099651 -2.239884
H -5.766986 -3.221931 -1.923177
H 5.361169 -1.942275 1.533895
H 5.619044 -0.549728 2.625825
H 4.019206 -1.208627 2.408473
H 5.400547 1.091629 -0.891999
H 7.641629 1.221813 -0.283634
H 7.267980 -0.145629 -2.254297
H 8.733600 -0.629073 -1.420159
H 6.058065 -2.125370 -1.290101
H 7.480631 -2.583985 -2.231021
H 7.551613 -2.620738 -0.467636
H 7.661777 0.374245 2.009901
H 8.991162 -0.262107 1.044866
H 7.621527 -1.301739 1.421853
SCF Energy (B3LYP/6-31G**)= -1361.69108275

Number of imaginary frequencies = 0

1a_c430
MMFF Geometry
C -1.624120 -2.471293 -0.149367
C -2.528867 0.078028 -0.419109
N -2.961597 -2.262283 -0.096818
C -0.666094 -1.469017 -0.330357
C -1.146481 -0.152808 -0.471067
C -3.400416 -0.991486 -0.223365
C -0.234102 0.899532 -0.670058
C 1.145311 0.661802 -0.708744
C 1.618365 -0.645229 -0.556897
C 0.721733 -1.708999 -0.384640
C 3.084007 -0.873259 -0.579077
C 4.013549 0.326661 -0.268897
C 3.388108 1.615352 -0.792539
C 2.070114 1.757351 -0.988550
O 1.157176 -3.006193 -0.258869
O 3.491841 -2.021831 -0.761970
O 5.259615 0.128284 -0.933152
C 4.196031 0.359905 1.259908
C 5.545308 -0.130930 1.736586
C 5.860687 -1.596664 1.585659
O 6.342843 0.645797 2.266744
O 4.224998 2.623990 -1.253695
C 5.078369 3.178110 -0.252686
C -4.870293 -0.809973 -0.165401
C -5.656102 -1.859020 -0.905660
C -5.410932 0.216917 0.524978
C -6.882891 0.512190 0.719065
C -7.358201 1.665362 -0.185865
C -6.701171 3.016363 0.070196
C -7.174616 0.778424 2.199249
H -1.329405 -3.512669 -0.037522
H -2.923539 1.081702 -0.544677
H -0.609147 1.914599 -0.798625
H 1.673586 2.677331 -1.409799
H 2.117587 -3.040258 -0.473239
H 5.131704 0.363928 -1.869954
H 3.428050 -0.222851 1.784793
H 4.077756 1.379025 1.648917
H 6.745980 -1.840198 2.180205
H 5.023796 -2.193272 1.957502
H 6.061899 -1.839649 0.541135
H 4.502032 3.495443 0.622395
H 5.864733 2.475101 0.033360
H 5.560431 4.063006 -0.678703
H -5.715929 -2.781410 -0.318497
H -6.674578 -1.536866 -1.138593
H -5.185791 -2.090676 -1.868533
H -4.746256 0.908394 1.042609
H -7.476877 -0.368942 0.451084
H -7.189244 1.390777 -1.235122
H -8.443260 1.781672 -0.070568
H -5.617774 2.972919 -0.074880
H -7.098509 3.760076 -0.628540
H -6.903170 3.375989 1.083160
H -6.939409 -0.105077 2.803782
H -8.234276 1.011019 2.349569
H -6.584768 1.612587 2.593835
SCF Energy (B3LYP/6-31G**)= -1361.69206626
Number of imaginary frequencies = 0

1a_c431
MMFF Geometry
C -1.921550 -1.926312 -0.154096
C -2.379040 0.713050 0.293136
N -3.198312 -1.526203 0.058843
C -0.811101 -1.078448 -0.159680
C -1.059848 0.285477 0.071658
C -3.422823 -0.211024 0.281555
C 0.016841 1.191206 0.070925
C 1.334252 0.759204 -0.144453
C 1.576152 -0.598143 -0.365594
C 0.511189 -1.513000 -0.384949
C 2.967145 -1.046499 -0.590626
C 4.131875 -0.090374 -0.272969
C 3.710507 1.369646 -0.222921
C 2.419295 1.741726 -0.161935
O 0.710520 -2.853134 -0.617913
O 3.161340 -2.195021 -1.001344
O 5.036412 -0.260133 -1.375267
C 4.770196 -0.526955 1.055525
C 6.270481 -0.691443 0.982765
C 7.125713 0.546503 1.000201
O 6.773685 -1.812558 0.885698
O 4.781818 2.245139 -0.216059
C 4.529955 3.637891 -0.358047
C -4.838814 0.189987 0.526570
C -5.051643 1.382412 1.416518
C -5.832185 -0.541408 -0.026308
C -7.335107 -0.364104 0.022063
C -7.869041 1.027089 -0.371237
C -7.411617 1.482112 -1.751421
C -7.906481 -0.822381 1.366293
H -1.810507 -2.994694 -0.327231
H -2.583459 1.765863 0.458121
H -0.181759 2.249108 0.240863
H 2.126187 2.784708 -0.111691
H 1.652485 -2.996492 -0.865966
H 5.161747 -1.224642 -1.489895
H 4.367692 -1.494478 1.385670
H 4.532981 0.160700 1.877141
H 8.142829 0.279649 1.301316
H 7.153914 0.994029 0.004954
H 6.733837 1.261557 1.727808
H 3.984221 3.846489 -1.283926
H 3.991431 4.026455 0.512008
H 5.495180 4.149998 -0.411950
H -4.910467 2.312381 0.856079
H -6.047379 1.405205 1.862455
H -4.352485 1.372729 2.260705
H -5.543090 -1.406368 -0.628955
H -7.736588 -1.060579 -0.729168
H -8.966268 0.993164 -0.372408
H -7.593875 1.786837 0.366305
H -7.681880 0.747408 -2.516525
H -7.891537 2.431309 -2.010875
H -6.329333 1.637538 -1.787017
H -7.563201 -1.832699 1.615764
H -9.001231 -0.845654 1.330034
H -7.617572 -0.157692 2.185730
SCF Energy (B3LYP/6-31G**)= -1361.69806992
Number of imaginary frequencies = 0

1a_c432
MMFF Geometry
C 1.545255 2.248285 0.344842
C 2.157971 -0.212021 -0.632503
N 2.854594 1.911384 0.250091
C 0.477786 1.423538 -0.024488
C 0.807585 0.153142 -0.532114
C 3.146719 0.682349 -0.226999
C -0.220507 -0.718261 -0.935107
C -1.568406 -0.350706 -0.824130
C -1.891589 0.906392 -0.306327
C -0.877799 1.796965 0.078605
C -3.320239 1.273459 -0.181184
C -4.402317 0.176409 -0.302683
C -3.913360 -1.035321 -1.081499
C -2.609602 -1.256608 -1.305938
O -1.161337 3.053461 0.560654
O -3.625153 2.452218 0.035351
O -5.456153 0.802283 -1.072684
C -4.986296 -0.082166 1.096196
C -4.122873 -0.942839 1.988932
C -4.836746 -1.855675 2.950780
O -2.896835 -0.855745 1.990056
O -4.797199 -1.879976 -1.744590
C -6.006786 -2.228263 -1.091011
C 4.590519 0.352490 -0.317822

C 5.435403 1.469924 -0.863730
C 5.036306 -0.853667 0.095962
C 6.446327 -1.409314 0.141961
C 7.399061 -0.565232 1.009615
C 8.723319 -1.261702 1.297138
C 6.986919 -1.705068 -1.259117
H 1.367564 3.244866 0.743209
H 2.436169 -1.180718 -1.035970
H 0.036934 -1.696569 -1.339084
H -2.303098 -2.108856 -1.906639
H -2.130071 3.203711 0.488454
H -5.456498 1.748863 -0.824961
H -5.975812 -0.544614 1.014863
H -5.159667 0.861975 1.629081
H -4.105048 -2.435773 3.519971
H -5.435559 -1.259524 3.643578
H -5.476146 -2.547852 2.398057
H -5.810612 -2.633403 -0.094499
H -6.698492 -1.383752 -1.047379
H -6.488637 -3.015474 -1.678925
H 6.426599 1.140796 -1.178154
H 4.966510 1.912286 -1.750490
H 5.567178 2.256747 -0.113307
H 4.306052 -1.554950 0.503394
H 6.353939 -2.384525 0.642689
H 7.616004 0.397767 0.536864
H 6.914355 -0.342233 1.968760
H 9.309450 -1.400537 0.384019
H 9.322377 -0.657651 1.986298
H 8.561528 -2.240546 1.759322
H 6.240534 -2.229035 -1.866958
H 7.869971 -2.350456 -1.207559
H 7.275847 -0.794829 -1.791836
SCF Energy (B3LYP/6-31G**)= -1361.69123691
Number of imaginary frequencies = 0

1a_c433
MMFF Geometry
C -1.771610 -1.834921 -0.521174
C -2.201593 0.689904 0.380420
N -3.044763 -1.463781 -0.244394
C -0.651383 -1.012238 -0.376997
C -0.886679 0.292291 0.089114
C -3.255642 -0.205334 0.205187
C 0.199704 1.169704 0.253126
C 1.512107 0.768914 -0.038206
C 1.744599 -0.529499 -0.500385
C 0.668184 -1.416873 -0.669802
C 3.135351 -0.955511 -0.795097
C 4.299855 0.039006 -0.624211
C 3.872444 1.402728 -0.133551
C 2.604797 1.715238 0.161191
O 0.849463 -2.703999 -1.119017
O 3.329114 -2.104119 -1.208000
O 4.786036 0.189028 -1.977690
C 5.421868 -0.602802 0.205052
C 5.184946 -0.607296 1.700893
C 4.098901 -1.496329 2.246623
O 5.866295 0.084546 2.457963
O 4.855542 2.337022 0.162066
C 5.546259 2.809136 -0.993262
C -4.668014 0.162691 0.514816
C -4.870208 1.187213 1.596042
C -5.667971 -0.452064 -0.155736
C -7.168987 -0.268986 -0.078097
C -7.686916 1.174739 -0.228436
C -7.221506 1.853474 -1.510585
C -7.748329 -0.943675 1.167940
H -1.672335 -2.857924 -0.877740
H -2.393710 1.699754 0.728248
H 0.015466 2.181365 0.614777
H 2.373757 2.698691 0.562451
H 1.803465 -2.843895 -1.311784
H 4.979188 -0.708572 -2.310560
H 6.367846 -0.075719 0.021405
H 5.617278 -1.636013 -0.108388
H 4.298013 -1.704333 3.301956
H 3.131269 -0.999917 2.159138
H 4.088920 -2.448991 1.711390
H 6.316189 2.095768 -1.300315
H 4.858143 3.020461 -1.819075
H 6.047838 3.743257 -0.723158
H -4.716065 2.197801 1.203840
H -5.867260 1.145383 2.037591
H -4.174452 1.024125 2.427240
H -5.386931 -1.203591 -0.898001
H -7.576381 -0.822556 -0.937290
H -8.784441 1.153665 -0.235743
H -7.405149 1.794438 0.627996
H -7.498005 1.263112 -2.389924
H -7.690526 2.838271 -1.604447
H -6.137523 2.000710 -1.518748
H -7.416468 -1.985445 1.241442
H -8.843179 -0.948437 1.127732
H -7.454259 -0.431829 2.088958
SCF Energy (B3LYP/6-31G**)= -1361.69253878
Number of imaginary frequencies = 0

1a_c434
MMFF Geometry
C -1.987310 -1.712260 -0.152317
C -2.324718 0.980340 -0.044112
N -3.248883 -1.230064 -0.045480
C -0.835106 -0.924139 -0.208946
C -1.021599 0.467808 -0.151006
C -3.413825 0.111561 0.006807
C 0.100272 1.315270 -0.204281
C 1.401070 0.799220 -0.305322
C 1.580462 -0.584609 -0.356608
C 0.470784 -1.444117 -0.319811
C 2.954161 -1.121549 -0.462164
C 4.153270 -0.190767 -0.202487
C 3.807278 1.283319 -0.340544
C 2.535932 1.720551 -0.384067
O 0.609231 -2.810334 -0.385002
O 3.104683 -2.318342 -0.727755
O 5.092088 -0.531087 -1.234423
C 4.712862 -0.498207 1.195845
C 6.204162 -0.740944 1.212650
C 7.121711 0.448708 1.128625
O 6.651413 -1.888323 1.267647
O 4.921814 2.101990 -0.383359
C 4.749378 3.479290 -0.694232
C -4.818469 0.605476 0.104420
C -5.099035 1.947711 -0.511332
C -5.748536 -0.177410 0.695453
C -7.221976 0.059106 0.951759
C -8.104311 -0.073526 -0.303359
C -7.988358 -1.432180 -0.983571
C -7.508144 1.363974 1.701703
H -1.925165 -2.797733 -0.192632
H -2.478395 2.052981 0.012361
H -0.049469 2.394003 -0.166954
H 2.296086 2.774925 -0.467795
H 1.551701 -3.026286 -0.570733
H 5.172000 -1.507166 -1.230772
H 4.246996 -1.400161 1.616027
H 4.478613 0.291543 1.920838
H 7.214249 0.775402 0.091174
H 6.738280 1.261411 1.750537
H 8.110174 0.170886 1.505664
H 4.254206 3.604900 -1.662538
H 4.196319 3.990963 0.099684
H 5.741511 3.935499 -0.760283
H -6.155258 2.096415 -0.741271
H -4.783652 2.753622 0.159432
H -4.571696 2.059738 -1.465759
H -5.419597 -1.148144 1.075484
H -7.531371 -0.741622 1.639973
H -9.153259 0.076992 -0.018176
H -7.867954 0.708594 -1.032675
H -8.220226 -2.242079 -0.284649

H -8.693475 -1.494524 -1.818733
H -6.983655 -1.596961 -1.384521
H -6.844061 1.476864 2.565714
H -8.538294 1.366080 2.075754
H -7.395988 2.247314 1.067730
SCF Energy (B3LYP/6-31G**)= -1361.69816450
Number of imaginary frequencies = 0

1a_c435
MMFF Geometry
C -1.859363 -1.390883 -0.484872
C -2.167329 1.214599 0.218231
N -3.111063 -0.940865 -0.228514
C -0.703007 -0.611045 -0.412803
C -0.874022 0.736921 -0.049512
C -3.262463 0.357000 0.122413
C 0.251622 1.578105 0.029332
C 1.540619 1.092208 -0.229114
C 1.704773 -0.250679 -0.575520
C 0.591714 -1.096799 -0.685006
C 3.075593 -0.752832 -0.824443
C 4.264818 0.036065 -0.224288
C 3.946164 1.536093 -0.222410
C 2.685023 2.004237 -0.201162
O 0.720810 -2.414683 -1.052346
O 3.192582 -1.813632 -1.440795
O 5.435480 -0.179024 -1.011953
C 4.511033 -0.441442 1.216001
C 5.508745 -1.574284 1.364722
C 5.262537 -2.863081 0.627143
O 6.464451 -1.467031 2.138297
O 5.084009 2.323002 -0.245219
C 4.942291 3.737260 -0.166992
C -4.654312 0.809297 0.411024
C -4.813053 1.982808 1.340035
C -5.696227 0.141498 -0.132470
C -7.170865 0.445256 0.012910
C -7.850983 -0.488614 1.032594
C -7.843138 -1.969997 0.675058
C -7.853529 0.401932 -1.358046
H -1.809574 -2.442020 -0.761147
H -2.310136 2.256305 0.486110
H 0.114104 2.625969 0.294683
H 2.460199 3.065205 -0.186156
H 1.641517 -2.565305 -1.368394
H 5.595007 0.629298 -1.531278
H 3.581682 -0.746236 1.712580
H 4.916594 0.385765 1.815612
H 5.903447 -3.646044 1.043610
H 4.222720 -3.171987 0.758780
H 5.501390 -2.748182 -0.431226
H 4.401111 4.123330 -1.036616
H 4.448485 4.029684 0.765232
H 5.944273 4.176287 -0.170909
H -4.615597 2.921577 0.812232
H -5.813582 2.046731 1.776283
H -4.125148 1.905425 2.189861
H -5.480277 -0.719532 -0.768311
H -7.303235 1.469867 0.378828
H -7.367636 -0.365213 2.010335
H -8.893009 -0.170587 1.163800
H -6.824579 -2.355938 0.573889
H -8.337323 -2.544659 1.465478
H -8.382663 -2.161385 -0.256888
H -7.429365 1.161751 -2.024485
H -8.925722 0.604564 -1.263529
H -7.733750 -0.569414 -1.849574
SCF Energy (B3LYP/6-31G**)= -1361.69890354
Number of imaginary frequencies = 0

1a_c436
MMFF Geometry
C 1.852803 2.247332 -0.052962
C 2.438782 -0.388291 -0.356490
N 3.158429 1.890109 -0.115855
C 0.775604 1.358301 -0.131028
C 1.091810 -0.002287 -0.289980
C 3.438619 0.577192 -0.258636
C 0.052293 -0.944229 -0.381505
C -1.293915 -0.554409 -0.312340
C -1.608429 0.797878 -0.150317
C -0.577963 1.750749 -0.063866
C -3.032722 1.209412 -0.069100
C -4.146373 0.141032 -0.105297
C -3.632859 -1.260382 -0.353637
C -2.333047 -1.573593 -0.413944
O -0.838870 3.092546 0.090455
O -3.286420 2.414855 0.033909
O -4.948508 0.529931 -1.225851
C -4.901220 0.244272 1.232243
C -6.311704 -0.291599 1.168877
C -6.647747 -1.474959 2.036396
O -7.169010 0.264324 0.480209
O -4.558953 -2.295286 -0.387667
C -5.268530 -2.342249 -1.628090
C 4.879023 0.229008 -0.330217
C 5.685340 1.156679 -1.196488
C 5.356758 -0.824557 0.367334
C 6.773157 -1.352601 0.488423
C 7.759398 -0.310780 1.049623
C 9.103173 -0.909938 1.445464
C 7.246191 -2.014216 -0.808200
H 1.686969 3.316003 0.066536
H 2.705801 -1.431160 -0.497539
H 0.301027 -1.997574 -0.509496
H -2.032665 -2.609682 -0.544672
H -1.813879 3.228354 0.072748
H -5.547905 1.241975 -0.928134
H -5.005450 1.289025 1.554097
H -4.348145 -0.257376 2.036056
H -7.621776 -1.877837 1.745503
H -5.898903 -2.260121 1.913676
H -6.685053 -1.157832 3.081365
H -6.111759 -1.646571 -1.614317
H -4.613864 -2.142143 -2.483564
H -5.673736 -3.352403 -1.740023
H 5.847280 2.114508 -0.690593
H 6.662479 0.751149 -1.461767
H 5.168579 1.348658 -2.144117
H 4.655398 -1.389503 0.983445
H 6.715730 -2.158106 1.235539
H 7.943032 0.489925 0.326398
H 7.320779 0.162363 1.937526
H 9.644499 -1.290111 0.574232
H 9.729744 -0.145828 1.916882
H 8.974303 -1.728911 2.160029
H 6.475727 -2.678888 -1.215257
H 8.137132 -2.625564 -0.630870
H 7.499104 -1.280668 -1.578741
SCF Energy (B3LYP/6-31G**)= -1361.69639391
Number of imaginary frequencies = 0

1a_c437
MMFF Geometry
C 1.837678 1.690264 -0.210844
C 2.140877 -1.004282 -0.069911
N 3.093866 1.193245 -0.109939
C 0.674309 0.917205 -0.247225
C 0.843910 -0.475640 -0.172526
C 3.241914 -0.149819 -0.041014
C -0.288876 -1.308132 -0.200226
C -1.583035 -0.776636 -0.303143
C -1.749930 0.609040 -0.378050
C -0.626784 1.453181 -0.350267
C -3.120756 1.169617 -0.473798
C -4.338207 0.226533 -0.527591
C -3.980113 -1.239424 -0.450691
C -2.725118 -1.684810 -0.316191
O -0.742903 2.821926 -0.416631
O -3.256576 2.396016 -0.543056
O -4.874552 0.481061 -1.846086
C -5.392428 0.669934 0.497470

C -5.113926 0.242773 1.923547
C -3.950608 0.874417 2.641436
O -5.824175 -0.586875 2.492180
O -5.008912 -2.168050 -0.373648
C -5.766137 -2.267293 -1.578465
C 4.640988 -0.660944 0.050445
C 4.895856 -2.019722 -0.539981
C 5.588862 0.120585 0.614471
C 7.061696 -0.131815 0.858987
C 7.931225 -0.039881 -0.408653
C 7.826620 1.304832 -1.117793
C 7.337755 -1.423707 1.634739
H 1.790037 2.775813 -0.264134
H 2.279713 -2.078234 0.000675
H -0.155733 -2.388349 -0.140265
H -2.542234 -2.751180 -0.211411
H -1.692418 3.057763 -0.514923
H -5.024485 1.443985 -1.909995
H -6.375076 0.266909 0.217462
H -5.529826 1.758521 0.492769
H -4.101973 0.788109 3.721384
H -3.024278 0.365188 2.371323
H -3.888521 1.936894 2.393592
H -6.499162 -1.458130 -1.640847
H -5.120512 -2.278247 -2.463437
H -6.316528 -3.212456 -1.549283
H 4.358437 -2.143127 -1.487352
H 5.947566 -2.189370 -0.775899
H 4.574483 -2.806854 0.149973
H 5.278081 1.103196 0.978699
H 7.390668 0.679381 1.525531
H 8.981168 -0.199072 -0.131961
H 7.675507 -0.834539 -1.117578
H 8.077967 2.126663 -0.439847
H 8.522847 1.338438 -1.962014
H 6.819777 1.475077 -1.511015
H 6.682468 -1.507371 2.508734
H 8.372160 -1.432758 1.996748
H 7.205120 -2.319246 1.022205
SCF Energy (B3LYP/6-31G**)= -1361.69267830
Number of imaginary frequencies = 0

1a_c438
MMFF Geometry
C -1.959028 -2.166758 -0.151764
C -2.469673 0.499812 -0.276066
N -3.249217 -1.772637 -0.280469
C -0.862679 -1.301674 -0.076954
C -1.139098 0.076422 -0.143350
C -3.491742 -0.445525 -0.334114
C -0.078469 0.997915 -0.070527
C 1.250454 0.568373 0.055637
C 1.520412 -0.800879 0.108737
C 0.472386 -1.733213 0.058532
C 2.929041 -1.236430 0.242026
C 4.041248 -0.241597 -0.160048
C 3.619530 1.201132 0.102984
C 2.323739 1.556145 0.187438
O 0.703064 -3.086266 0.140449
O 3.139681 -2.392349 0.619861
O 4.170224 -0.407851 -1.579361
C 5.326805 -0.667360 0.579178
C 6.581004 -0.004273 0.050300
C 7.665199 0.299333 1.048668
O 6.731700 0.225325 -1.151549
O 4.681355 2.080127 0.214898
C 4.413187 3.477518 0.246014
C -4.915497 -0.057110 -0.481425
C -5.670770 -0.883382 -1.484960
C -5.427127 0.941788 0.270152
C -6.841299 1.481152 0.338828
C -7.945427 0.444993 0.625070
C -7.709837 -0.352056 1.901948
C -7.176294 2.330689 -0.889870
H -1.822277 -3.245320 -0.109840
H -2.706080 1.556952 -0.347407
H -0.297610 2.064142 -0.113320
H 2.015624 2.581988 0.354442
H 1.660042 -3.227101 0.322976
H 5.061436 -0.077318 -1.824775
H 5.509886 -1.743887 0.471826
H 5.228979 -0.456260 1.651002
H 8.505534 0.784071 0.544138
H 7.278798 0.976264 1.814658
H 8.012215 -0.630533 1.505593
H 3.869962 3.793460 -0.650453
H 3.866032 3.748701 1.154353
H 5.372689 4.002808 0.262177
H -5.936058 -1.857625 -1.060909
H -6.588385 -0.401151 -1.825493
H -5.067337 -1.050690 -2.384923
H -4.764316 1.437618 0.980990
H -6.850280 2.173355 1.193998
H -8.902960 0.972104 0.726249
H -8.071354 -0.248496 -0.211799
H -7.589052 0.312835 2.763076
H -8.565442 -1.006272 2.098278
H -6.820297 -0.984188 1.824534
H -6.409348 3.094485 -1.060801
H -8.131611 2.848306 -0.749138
H -7.258853 1.728253 -1.799139
SCF Energy (B3LYP/6-31G**)= -1361.70563066
Number of imaginary frequencies = 0

1a_c439
MMFF Geometry
C 1.895222 -2.419537 -0.107842
C 2.543761 0.219050 -0.167025
N 3.209404 -2.088590 -0.106710
C 0.839879 -1.502262 -0.138594
C 1.187783 -0.139264 -0.172559
C 3.519722 -0.774797 -0.125242
C 0.171241 0.832190 -0.220005
C -1.182923 0.468053 -0.217264
C -1.523013 -0.885620 -0.167165
C -0.521300 -1.868550 -0.144233
C -2.957500 -1.251632 -0.164613
C -3.983197 -0.177773 0.263375
C -3.514285 1.223102 -0.119054
C -2.213708 1.502894 -0.325190
O -0.823810 -3.209808 -0.130664
O -3.254588 -2.413527 -0.456134
O -4.003274 -0.256568 1.695890
C -5.344337 -0.575087 -0.344718
C -6.516937 0.183256 0.240377
C -7.663479 0.487147 -0.685630
O -6.556886 0.488647 1.434115
O -4.537544 2.149758 -0.201806
C -4.204981 3.526597 -0.340729
C 4.967961 -0.454254 -0.126106
C 5.780158 -1.312808 -1.055251
C 5.445643 0.519480 0.678813
C 6.866656 1.002595 0.884908
C 7.436030 1.806384 -0.298932
C 6.599689 3.028832 -0.657208
C 7.824543 -0.110917 1.319347
H 1.703000 -3.490139 -0.082844
H 2.837024 1.263504 -0.208629
H 0.445091 1.885865 -0.258302
H -1.870237 2.499567 -0.577882
H -1.798326 -3.308845 -0.227911
H -4.854035 0.134784 1.990497
H -5.570403 -1.632436 -0.157997
H -5.324585 -0.430539 -1.431757
H -8.434821 1.044130 -0.146661
H -7.308843 1.097700 -1.519740
H -8.091840 -0.447307 -1.055970
H -3.576326 3.864689 0.489361
H -3.720468 3.714720 -1.303983
H -5.135902 4.100667 -0.314955
H 5.275868 -1.422710 -2.022510
H 6.762265 -0.891451 -1.274144

H 5.929322 -2.310111 -0.627951
H 4.737857 1.040852 1.325391
H 6.820246 1.694487 1.739084
H 8.447115 2.147661 -0.042898
H 7.540307 1.175974 -1.188172
H 6.475722 3.687048 0.208574
H 7.093290 3.601584 -1.448966
H 5.607850 2.745037 -1.022004
H 7.398811 -0.699934 2.139198
H 8.766864 0.319042 1.677320
H 8.072364 -0.794201 0.502856
SCF Energy (B3LYP/6-31G**)= -1361.70577838
Number of imaginary frequencies = 0

1a_c440
MMFF Geometry
C -1.830212 -1.523776 -0.351391
C -2.210429 1.135631 0.050749
N -3.093779 -1.082441 -0.142540
C -0.695944 -0.709303 -0.373552
C -0.904526 0.666580 -0.165194
C -3.281091 0.242537 0.058000
C 0.196404 1.542869 -0.186480
C 1.496936 1.065420 -0.391109
C 1.699406 -0.304055 -0.586768
C 0.611443 -1.187746 -0.594494
C 3.086492 -0.792997 -0.780198
C 4.250300 0.096626 -0.276063
C 3.882663 1.568572 -0.432125
C 2.615223 2.000806 -0.481879
O 0.777716 -2.534735 -0.808951
O 3.246094 -1.918021 -1.257514
O 5.406837 -0.165336 -1.067941
C 4.486974 -0.280382 1.198231
C 5.725237 -1.116340 1.435919
C 5.733430 -2.533342 0.923392
O 6.680608 -0.655827 2.064903
O 4.891103 2.482552 -0.711595
C 5.873267 2.601797 0.317211
C -4.684941 0.686496 0.298008
C -4.874325 1.950865 1.092173
C -5.707209 -0.063815 -0.169779
C -7.192218 0.209019 -0.060318
C -7.820805 -0.785462 0.932090
C -9.293955 -0.517278 1.207894
C -7.823761 0.119791 -1.453353
H -1.751523 -2.597726 -0.506525
H -2.382047 2.196976 0.197672
H 0.032074 2.610738 -0.044396
H 2.400210 3.053767 -0.643351
H 1.703248 -2.696938 -1.103145
H 5.297391 0.310964 -1.910935
H 3.635616 -0.819869 1.633045
H 4.588903 0.615925 1.822871
H 6.572262 -3.075475 1.369471
H 4.807382 -3.035367 1.214946
H 5.844053 -2.547546 -0.162018
H 5.403996 2.796872 1.286926
H 6.509767 1.714652 0.364344
H 6.510114 3.456922 0.072046
H -4.709435 2.830370 0.461195
H -5.872757 2.032921 1.529582
H -4.178781 1.989903 1.938447
H -5.468295 -0.984264 -0.707163
H -7.370536 1.226257 0.302759
H -7.709090 -1.813559 0.564564
H -7.281540 -0.733898 1.886980
H -9.906819 -0.686157 0.317807
H -9.657069 -1.190776 1.990985
H -9.449399 0.511446 1.547772
H -7.305783 0.777289 -2.161073
H -8.873053 0.430566 -1.432423
H -7.781452 -0.901032 -1.849614
SCF Energy (B3LYP/6-31G**)= -1361.69402602
Number of imaginary frequencies = 0

1a_c441
MMFF Geometry
C -1.724962 -2.294267 -0.181849
C -2.459910 0.248398 0.437959
N -3.017989 -2.069992 0.154576
C -0.731483 -1.311842 -0.232209
C -1.123169 0.001418 0.092936
C -3.375632 -0.802219 0.451250
C -0.171091 1.037020 0.058818
C 1.163336 0.778949 -0.276265
C 1.548655 -0.528317 -0.588936
C 0.608784 -1.567470 -0.584705
C 2.971944 -0.780725 -0.919018
C 4.022779 0.232499 -0.398133
C 3.439468 1.639836 -0.449276
C 2.121327 1.876920 -0.385626
O 0.957455 -2.855278 -0.912747
O 3.256853 -1.819757 -1.517129
O 5.156070 0.159904 -1.262703
C 4.381427 -0.221376 1.030252
C 5.863606 -0.327475 1.302827
C 6.575050 -1.574876 0.844760
O 6.451200 0.545496 1.945002
O 4.271371 2.709302 -0.750171
C 5.317836 2.921775 0.195232
C -4.799354 -0.599587 0.816541
C -5.368118 -1.676938 1.701263
C -5.483226 0.457911 0.328536
C -6.930637 0.821749 0.580449
C -7.830966 0.454043 -0.615442
C -7.527898 1.183274 -1.918750
C -7.038584 2.303418 0.954237
H -1.497851 -3.331546 -0.419089
H -2.781724 1.249860 0.707220
H -0.479884 2.055537 0.292699
H 1.740657 2.891187 -0.471487
H 1.866218 -2.849874 -1.291925
H 4.921859 0.630118 -2.083730
H 3.953430 -1.205114 1.266733
H 3.946840 0.451310 1.781395
H 7.653032 -1.392743 0.823444
H 6.357119 -2.388037 1.541484
H 6.255060 -1.859397 -0.159433
H 4.961959 2.809439 1.224427
H 6.157406 2.254359 -0.011205
H 5.674019 3.948963 0.072026
H -4.735900 -1.818536 2.585316
H -6.371872 -1.453257 2.067709
H -5.429235 -2.629030 1.162782
H -4.971354 1.137210 -0.353620
H -7.312940 0.266948 1.443224
H -7.761570 -0.626186 -0.797622
H -8.875831 0.652404 -0.344879
H -6.510002 0.984284 -2.266261
H -8.215717 0.845224 -2.700982
H -7.655545 2.264420 -1.813615
H -6.489047 2.508681 1.880122
H -8.083748 2.586484 1.118814
H -6.628153 2.957190 0.177296
SCF Energy (B3LYP/6-31G**)= -1361.69064087
Number of imaginary frequencies = 0

1a_c442
MMFF Geometry
C -1.816477 -1.457553 -0.832597
C -2.230444 1.159318 -0.232965
N -3.092923 -1.029927 -0.681548
C -0.685174 -0.649541 -0.699440
C -0.911120 0.703875 -0.387649
C -3.297076 0.274252 -0.384388
C 0.187042 1.572149 -0.244389
C 1.500138 1.107508 -0.389603
C 1.718923 -0.240161 -0.690694
C 0.636010 -1.113678 -0.860722
C 3.117445 -0.717751 -0.819173
C 4.237522 0.116683 -0.148963

C 3.881214 1.598747 -0.200777
C 2.620906 2.040900 -0.309541
O 0.820246 -2.437437 -1.179678
O 3.314971 -1.796541 -1.381467
O 5.452356 -0.080102 -0.869200
C 4.359624 -0.391702 1.299597
C 5.576390 -1.253189 1.556421
C 5.625306 -2.618809 0.921342
O 6.479899 -0.856563 2.295869
O 4.907155 2.528253 -0.318622
C 5.806085 2.549948 0.789881
C -4.717096 0.709308 -0.243380
C -5.017176 2.157118 -0.524198
C -5.658294 -0.200147 0.094042
C -7.139803 0.022525 0.311817
C -7.538808 -0.524559 1.696530
C -8.985373 -0.230961 2.070605
C -7.919208 -0.644485 -0.823402
H -1.724486 -2.514421 -1.073834
H -2.413409 2.199083 0.017837
H 0.010819 2.623935 -0.020279
H 2.417741 3.105281 -0.392202
H 1.765813 -2.577934 -1.415544
H 5.408224 0.470449 -1.671954
H 3.477542 -0.962490 1.617475
H 4.412313 0.444700 2.007820
H 6.427206 -3.203919 1.380839
H 4.679689 -3.138967 1.094088
H 5.820077 -2.536747 -0.149120
H 5.262490 2.660737 1.733730
H 6.438049 1.658482 0.807101
H 6.459054 3.419814 0.672056
H -4.462660 2.509075 -1.401782
H -6.070850 2.337311 -0.752051
H -4.746958 2.779988 0.334807
H -5.343796 -1.234949 0.247217
H -7.374905 1.091379 0.301582
H -7.374101 -1.608742 1.741068
H -6.891532 -0.077378 2.462295
H -9.684871 -0.762541 1.419099
H -9.179769 -0.555632 3.097975
H -9.197916 0.840922 2.008854
H -7.570164 -0.289275 -1.799842
H -8.987208 -0.414018 -0.759563
H -7.806277 -1.734292 -0.802185
SCF Energy (B3LYP/6-31G**)= -1361.69404271
Number of imaginary frequencies = 0

1a_c443
MMFF Geometry
C -1.702283 -2.368131 -0.120073
C -2.428456 0.243666 0.074081
N -3.019220 -2.083781 0.023520
C -0.680354 -1.414645 -0.173845
C -1.068115 -0.066548 -0.070217
C -3.369903 -0.782891 0.109519
C -0.085718 0.939240 -0.115545
C 1.274353 0.624484 -0.254647
C 1.654833 -0.715642 -0.351895
C 0.685402 -1.731231 -0.322719
C 3.090246 -1.039770 -0.497215
C 4.144038 0.050743 -0.228918
C 3.581459 1.460562 -0.315711
C 2.258870 1.705618 -0.324166
O 1.023185 -3.059422 -0.431747
O 3.410191 -2.193075 -0.802272
O 5.101021 -0.117763 -1.286215
C 4.772000 -0.209418 1.149840
C 6.282813 -0.229053 1.134540
C 7.012890 1.085183 1.071251
O 6.895460 -1.298650 1.147587
O 4.561782 2.436211 -0.352168
C 4.181230 3.780952 -0.617146
C -4.820011 -0.516686 0.268334
C -5.503886 -1.427099 1.250244
C -5.415009 0.459305 -0.451367
C -6.871989 0.877261 -0.504087
C -7.252110 1.728077 0.721218
C -8.639150 2.348446 0.619348
C -7.834325 -0.284199 -0.770349
H -1.479247 -3.430639 -0.192643
H -2.750517 1.275890 0.171373
H -0.392840 1.982213 -0.042216
H 1.864200 2.714720 -0.371295
H 1.982935 -3.127609 -0.640665
H 5.323820 -1.071017 -1.314217
H 4.453145 -1.181793 1.549747
H 4.439052 0.516371 1.902550
H 8.039255 0.946481 1.423021
H 7.034335 1.450430 0.042791
H 6.526995 1.814724 1.723905
H 3.652323 3.859021 -1.572527
H 3.575812 4.182402 0.201570
H 5.093478 4.380869 -0.685862
H -4.872280 -1.598733 2.129894
H -6.437196 -1.008849 1.631628
H -5.724199 -2.395696 0.789395
H -4.798603 1.032528 -1.145556
H -6.951808 1.531293 -1.385086
H -7.203939 1.130355 1.638323
H -6.521863 2.539086 0.839830
H -9.421387 1.583647 0.626557
H -8.816878 3.011527 1.472202
H -8.739160 2.940344 -0.295854
H -7.438112 -0.960250 -1.536150
H -8.795288 0.091022 -1.139082
H -8.041545 -0.869625 0.129401
SCF Energy (B3LYP/6-31G**)= -1361.69666391
Number of imaginary frequencies = 0

1a_c444
MMFF Geometry
C 1.663440 -2.255521 0.234898
C 2.454975 0.251338 -0.456027
N 2.964184 -2.067085 -0.093639
C 0.688932 -1.252919 0.243137
C 1.109955 0.041124 -0.118661
C 3.350004 -0.816569 -0.425825
C 0.177778 1.094905 -0.128632
C -1.165434 0.874075 0.199733
C -1.580603 -0.414227 0.550519
C -0.660161 -1.470923 0.588124
C -3.011905 -0.631906 0.873875
C -4.043489 0.396306 0.345956
C -3.422343 1.789078 0.324446
C -2.100080 1.995285 0.255304
O -1.035734 -2.741993 0.950683
O -3.322690 -1.661416 1.475974
O -5.165542 0.416945 1.225631
C -4.449537 -0.070319 -1.064381
C -5.823725 -0.698412 -1.140689
C -6.029282 -2.037342 -0.480670
O -6.733353 -0.141723 -1.759646
O -4.238643 2.887633 0.563704
C -5.259259 3.081078 -0.415192
C 4.781280 -0.653164 -0.781018
C 5.338350 -1.768904 -1.624671
C 5.480510 0.405887 -0.318821
C 6.937479 0.733417 -0.565494
C 7.817449 0.385997 0.651428
C 7.514873 1.161539 1.927838
C 7.078750 2.200240 -0.984051
H 1.413501 -3.280235 0.501972
H 2.799322 1.237353 -0.752979
H 0.509345 2.098923 -0.392371
H -1.699120 3.004704 0.291955
H -1.950145 -2.710472 1.314517
H -4.915434 0.945129 2.005343
H -3.734593 -0.786880 -1.488999
H -4.448127 0.767825 -1.772498
H -6.976426 -2.467056 -0.819497
H -5.222833 -2.716839 -0.767934

H -6.061735 -1.928539 0.604534
H -4.835994 3.100496 -1.424697
H -6.037779 2.318332 -0.334303
H -5.723336 4.053327 -0.224724
H 4.713242 -1.926163 -2.511130
H 6.350323 -1.576525 -1.986247
H 5.374668 -2.704440 -1.055787
H 4.974833 1.116249 0.335744
H 7.317956 0.144485 -1.406173
H 7.724732 -0.686426 0.866398
H 8.868883 0.555411 0.386705
H 6.489549 0.993415 2.269770
H 8.187393 0.834880 2.728007
H 7.665049 2.236161 1.790513
H 6.543340 2.386990 -1.922053
H 8.131020 2.457605 -1.145521
H 6.673047 2.885947 -0.232573
SCF Energy (B3LYP/6-31G**)= -1361.69257785
Number of imaginary frequencies = 0

1a_c445
MMFF Geometry
C 1.966849 1.802964 -0.126634
C 2.315009 -0.889173 -0.065653
N 3.233394 1.323912 -0.078138
C 0.814719 1.012363 -0.145554
C 1.007548 -0.378908 -0.112207
C 3.402933 -0.017772 -0.050054
C -0.113078 -1.228026 -0.126015
C -1.418292 -0.714522 -0.171473
C -1.609329 0.669660 -0.203653
C -0.497142 1.529725 -0.194008
C -2.990329 1.213409 -0.243893
C -4.197896 0.253222 -0.190913
C -3.810573 -1.208978 -0.228384
C -2.544775 -1.641701 -0.186286
O -0.635148 2.897955 -0.227142
O -3.132559 2.439418 -0.312555
O -4.920958 0.556570 -1.389005
C -4.984931 0.603454 1.084764
C -6.436313 0.190060 1.027770
C -6.910536 -0.830253 2.027745
O -7.214179 0.720390 0.232821
O -4.828313 -2.152659 -0.168995
C -5.497256 -2.303008 -1.423690
C 4.810464 -0.510136 -0.015780
C 5.071412 -1.836631 -0.673057
C 5.762062 0.260661 0.556639
C 7.242706 0.011661 0.762276
C 8.036823 0.293527 -0.525926
C 9.546028 0.261005 -0.325174
C 7.559107 -1.346681 1.395704
H 1.900221 2.888612 -0.150835
H 2.473649 -1.962012 -0.027760
H 0.038836 -2.306976 -0.102396
H -2.339200 -2.708502 -0.165147
H -1.591553 3.116623 -0.309841
H -5.459415 1.353501 -1.216180
H -5.002051 1.687370 1.260258
H -4.508774 0.169150 1.972635
H -7.907410 -1.181859 1.747965
H -6.234399 -1.687494 2.044754
H -6.953656 -0.371958 3.018719
H -6.271372 -1.539808 -1.539678
H -4.798403 -2.278721 -2.267184
H -5.991617 -3.279087 -1.420780
H 4.482051 -1.944613 -1.590997
H 6.112034 -1.956055 -0.980563
H 4.818259 -2.659385 0.003458
H 5.449271 1.225372 0.964436
H 7.558996 0.759586 1.504503
H 7.773070 -0.426863 -1.308498
H 7.763647 1.284672 -0.910730
H 9.893594 -0.742616 -0.062840
H 10.053653 0.555901 -1.249280
H 9.852768 0.953433 0.465014
H 6.852185 -1.585111 2.198101
H 8.559806 -1.337203 1.841636
H 7.540045 -2.162265 0.667945
SCF Energy (B3LYP/6-31G**)= -1361.69655546
Number of imaginary frequencies = 0

1a_c446
MMFF Geometry
C -1.555052 -2.355908 -0.523400
C -2.452610 0.197846 -0.271758
N -2.891308 -2.158816 -0.421404
C -0.594970 -1.339420 -0.506701
C -1.071381 -0.020706 -0.378925
C -3.326404 -0.887521 -0.287762
C -0.155926 1.048215 -0.370549
C 1.223365 0.821759 -0.466845
C 1.688645 -0.489751 -0.576879
C 0.791300 -1.565539 -0.617455
C 3.146361 -0.706155 -0.667899
C 4.098103 0.392185 -0.151642
C 3.480744 1.774418 -0.376628
C 2.152285 1.952986 -0.506170
O 1.225071 -2.861111 -0.761547
O 3.562665 -1.770459 -1.132854
O 5.318062 0.313550 -0.893696
C 4.360303 0.194560 1.350587
C 5.535520 -0.695002 1.707432
C 5.550496 -2.113899 1.206250
O 6.429066 -0.289991 2.455004
O 4.416349 2.793467 -0.385087
C 3.986486 4.126361 -0.627366
C -4.795230 -0.717201 -0.184858
C -5.586843 -1.587611 -1.123553
C -5.324357 0.142647 0.711833
C -6.790231 0.410653 0.977685
C -7.166739 1.865463 0.641421
C -7.028735 2.194394 -0.839923
C -7.096635 0.116567 2.449913
H -1.263299 -3.399270 -0.623662
H -2.845300 1.206902 -0.191022
H -0.530206 2.068296 -0.287846
H 1.713936 2.935951 -0.640000
H 2.175085 -2.852366 -1.015483
H 5.274848 -0.485212 -1.453452
H 3.475969 -0.197280 1.868339
H 4.578635 1.164079 1.820306
H 6.265106 -2.697523 1.794458
H 4.563039 -2.563979 1.333886
H 5.857724 -2.142366 0.159827
H 3.508075 4.211505 -1.608345
H 3.316749 4.470703 0.167052
H 4.870255 4.771142 -0.625768
H -5.636274 -2.614678 -0.746793
H -6.609247 -1.232088 -1.272765
H -5.126192 -1.605341 -2.118306
H -4.652127 0.703218 1.361975
H -7.426971 -0.252934 0.384479
H -8.208080 2.045432 0.936638
H -6.550682 2.563934 1.221875
H -7.626220 1.510094 -1.450633
H -7.379942 3.213362 -1.031899
H -5.987498 2.134496 -1.170760
H -6.849650 -0.920822 2.702260
H -8.161362 0.264977 2.660479
H -6.527882 0.771772 3.119568
SCF Energy (B3LYP/6-31G**)= -1361.70281223
Number of imaginary frequencies = 0

1a_c447
MMFF Geometry
C -1.544037 -2.328425 -0.284701
C -2.260375 0.271405 0.048844
N -2.860860 -2.046623 -0.136158
C -0.517109 -1.378541 -0.278578
C -0.900532 -0.037122 -0.103071
C -3.206656 -0.750715 0.019622

C 0.086232 0.963871 -0.080912
C 1.445126 0.651559 -0.234044
C 1.825542 -0.681961 -0.410040
C 0.849603 -1.693135 -0.431971
C 3.265087 -1.010962 -0.559092
C 4.318038 0.114149 -0.558553
C 3.736381 1.496072 -0.371401
C 2.430266 1.727243 -0.192302
O 1.178047 -3.018455 -0.596679
O 3.590100 -2.192853 -0.716600
O 4.857067 0.040410 -1.898300
C 5.452236 -0.227553 0.419054
C 5.145160 0.052738 1.875386
C 4.106126 -0.795498 2.559875
O 5.736754 0.937897 2.493987
O 4.608492 2.567562 -0.237287
C 5.313390 2.870379 -1.439907
C -4.656806 -0.486854 0.181995
C -5.352934 -1.448275 1.104832
C -5.241316 0.532135 -0.484759
C -6.695609 0.960722 -0.524154
C -7.080678 1.741912 0.745181
C -8.463620 2.374974 0.668899
C -7.661957 -0.178039 -0.863815
H -1.325637 -3.386436 -0.413675
H -2.577693 1.298404 0.202253
H -0.213984 2.002542 0.057784
H 2.084712 2.741330 -0.008412
H 2.150578 -3.093623 -0.720892
H 5.155730 -0.879542 -2.033393
H 6.353432 0.342436 0.155607
H 5.756738 -1.278369 0.335561
H 4.265560 -0.760122 3.641533
H 3.107942 -0.416165 2.336087
H 4.200126 -1.835884 2.239101
H 6.162120 2.193160 -1.570260
H 4.654031 2.842642 -2.314325
H 5.709994 3.885859 -1.347073
H -4.729248 -1.673353 1.978058
H -6.287216 -1.047601 1.502358
H -5.574266 -2.387715 0.587567
H -4.616446 1.141840 -1.139240
H -6.765202 1.664695 -1.366690
H -7.042698 1.092236 1.626746
H -6.347122 2.540611 0.915596
H -9.249890 1.615479 0.626364
H -8.644426 2.988978 1.557118
H -8.553477 3.019017 -0.211485
H -7.263398 -0.811274 -1.664218
H -8.618048 0.223201 -1.217513
H -7.879221 -0.812948 -0.000740
SCF Energy (B3LYP/6-31G**)= -1361.69112826
Number of imaginary frequencies = 0

1a_c448
MMFF Geometry
C -1.615669 -2.185824 -0.227255
C -2.381027 0.339472 0.426710
N -2.918858 -1.974803 0.075979
C -0.626275 -1.197762 -0.226872
C -1.033678 0.105890 0.114797
C -3.290802 -0.715385 0.390704
C -0.085173 1.145523 0.131085
C 1.260997 0.903177 -0.172487
C 1.659156 -0.394530 -0.498094
C 0.724904 -1.438152 -0.545710
C 3.083978 -0.629389 -0.805937
C 4.134588 0.377144 -0.294358
C 3.545106 1.789262 -0.268714
C 2.218610 2.010578 -0.198970
O 1.088787 -2.716027 -0.895077
O 3.397121 -1.637984 -1.443865
O 5.243773 0.364677 -1.196987
C 4.582296 -0.009152 1.125106
C 5.760675 -0.959623 1.213376
C 5.657808 -2.306882 0.550820
O 6.758769 -0.667198 1.876396
O 4.509069 2.781529 -0.278979
C 4.101481 4.142957 -0.300407
C -4.724937 -0.530775 0.716875
C -5.314026 -1.626052 1.564662
C -5.405524 0.532996 0.238950
C -6.873626 0.848378 0.429740
C -7.597161 1.003405 -0.922076
C -7.626472 -0.284109 -1.736652
C -7.008275 2.134898 1.248369
H -1.376652 -3.216657 -0.480287
H -2.715999 1.333079 0.708465
H -0.407497 2.155511 0.383126
H 1.803619 3.011637 -0.155009
H 1.997104 -2.696561 -1.271484
H 5.100946 -0.361420 -1.833858
H 3.758966 -0.443582 1.705676
H 4.894937 0.890491 1.673894
H 6.420694 -2.972779 0.965377
H 4.679341 -2.748234 0.755136
H 5.825120 -2.214092 -0.523236
H 3.504096 4.359060 -1.191964
H 3.553041 4.400333 0.611289
H 5.001466 4.763733 -0.338561
H -4.620068 -1.916515 2.362221
H -6.237317 -1.325020 2.065667
H -5.530397 -2.509901 0.955328
H -4.882760 1.250363 -0.394179
H -7.380086 0.051866 0.983539
H -8.632579 1.318798 -0.742077
H -7.127437 1.794897 -1.519695
H -8.086211 -1.097891 -1.166850
H -8.212694 -0.137000 -2.649436
H -6.620640 -0.595485 -2.034334
H -6.519980 2.031891 2.223971
H -8.062724 2.370779 1.428415
H -6.556146 2.990074 0.733129
SCF Energy (B3LYP/6-31G**)= -1361.70290826
Number of imaginary frequencies = 0

1a_c449
MMFF Geometry
C -1.764729 -2.243366 -0.257521
C -2.399555 0.396220 -0.380576
N -3.076635 -1.904643 -0.258187
C -0.704534 -1.332828 -0.313818
C -1.045419 0.030200 -0.379494
C -3.381036 -0.590469 -0.311113
C -0.023370 0.994266 -0.448539
C 1.329873 0.624292 -0.440444
C 1.663357 -0.729753 -0.366114
C 0.655085 -1.705848 -0.314501
C 3.091799 -1.111956 -0.356781
C 4.163204 -0.032367 -0.115370
C 3.665889 1.376899 -0.395150
C 2.358990 1.660656 -0.539615
O 0.947330 -3.048137 -0.260395
O 3.389090 -2.300506 -0.513306
O 5.194647 -0.342467 -1.065139
C 4.667434 -0.163276 1.330851
C 6.172628 -0.230918 1.444900
C 6.956770 1.045927 1.306994
O 6.739516 -1.311332 1.620647
O 4.684005 2.312082 -0.450988
C 4.379785 3.631483 -0.886890
C -4.828031 -0.263130 -0.316690
C -5.647342 -1.149331 -1.213699
C -5.299315 0.736919 0.459582
C -6.719159 1.231756 0.657073
C -7.670784 0.140256 1.182470
C -9.010302 0.690361 1.656267
C -7.243511 1.963960 -0.580529
H -1.579175 -3.314416 -0.208968
H -2.687439 1.440786 -0.449373
H -0.293677 2.048157 -0.509721
H 2.009777 2.670680 -0.724496

H 1.917524 -3.167680 -0.378042
H 5.380696 -1.299771 -0.975323
H 4.279315 -1.077231 1.801126
H 4.303898 0.648506 1.973536
H 7.945137 0.914161 1.756632
H 7.075502 1.298203 0.251602
H 6.449023 1.855706 1.836836
H 3.932627 3.622247 -1.886168
H 3.727220 4.137667 -0.168613
H 5.317785 4.191832 -0.941523
H -5.778602 -2.139132 -0.763529
H -6.638434 -0.744964 -1.423104
H -5.158074 -1.273711 -2.186862
H -4.587595 1.274336 1.088256
H -6.650332 1.990412 1.450813
H -7.865068 -0.617270 0.416759
H -7.196777 -0.379187 2.025155
H -9.584980 1.114221 0.827592
H -9.609643 -0.112202 2.098277
H -8.871310 1.465697 2.416177
H -6.496765 2.665783 -0.969005
H -8.137626 2.547638 -0.337947
H -7.509615 1.275183 -1.387103
SCF Energy (B3LYP/6-31G**)= -1361.69699118
Number of imaginary frequencies = 0

1a_c450
MMFF Geometry
C -1.924445 -1.401844 -0.381466
C -2.239622 1.252285 0.103225
N -3.176117 -0.937342 -0.152363
C -0.771325 -0.614062 -0.385179
C -0.946234 0.759350 -0.134026
C -3.331207 0.384992 0.089220
C 0.175084 1.609667 -0.134717
C 1.462935 1.108152 -0.360870
C 1.631918 -0.259112 -0.599126
C 0.523266 -1.116255 -0.627708
C 3.006072 -0.774499 -0.814909
C 4.193051 0.071661 -0.290386
C 3.859709 1.555938 -0.399660
C 2.602661 2.019248 -0.429319
O 0.656493 -2.459630 -0.883849
O 3.136654 -1.887903 -1.327022
O 5.339255 -0.193232 -1.096191
C 4.427737 -0.355596 1.170458
C 5.646958 -1.227335 1.375859
C 5.619111 -2.627860 0.820520
O 6.615969 -0.808827 2.013271
O 4.888211 2.453988 -0.656767
C 5.877829 2.518608 0.369774
C -4.723068 0.853966 0.350496
C -4.880068 2.104251 1.173722
C -5.766200 0.135849 -0.121926
C -7.241124 0.444305 0.010106
C -7.906659 -0.398705 1.115015
C -7.893723 -1.905903 0.890124
C -7.936052 0.276330 -1.344999
H -1.872054 -2.472133 -0.569496
H -2.384955 2.312392 0.283482
H 0.036835 2.676287 0.040627
H 2.411930 3.081418 -0.557559
H 1.576501 -2.634574 -1.187904
H 5.237120 0.310986 -1.923725
H 3.565913 -0.887898 1.593343
H 4.553849 0.518598 1.821467
H 6.446991 -3.202931 1.245307
H 4.682835 -3.116596 1.101803
H 5.724162 -2.611581 -0.265414
H 5.417956 2.695015 1.347534
H 6.493320 1.615683 0.386428
H 6.533608 3.365566 0.147211
H -4.691292 2.993598 0.563574
H -5.877380 2.202451 1.610942
H -4.185150 2.104868 2.021362
H -5.551313 -0.776716 -0.681644
H -7.375909 1.496684 0.284989
H -7.415124 -0.186763 2.073298
H -8.949220 -0.075301 1.227490
H -6.873969 -2.294388 0.813769
H -8.377405 -2.410579 1.733080
H -8.440663 -2.181774 -0.015962
H -7.522237 0.976048 -2.080208
H -9.008447 0.481470 -1.258519
H -7.815376 -0.734204 -1.749619
SCF Energy (B3LYP/6-31G**)= -1361.69317474
Number of imaginary frequencies = 0

1a_c451
MMFF Geometry
C -1.629983 -2.535571 -0.098662
C -2.395857 0.055210 -0.404307
N -2.955042 -2.255499 -0.063797
C -0.618880 -1.587042 -0.279650
C -1.027041 -0.248599 -0.439755
C -3.324679 -0.964801 -0.207362
C -0.057905 0.752161 -0.641934
C 1.307858 0.442324 -0.665449
C 1.704460 -0.885871 -0.488174
C 0.753966 -1.900529 -0.316915
C 3.151191 -1.186551 -0.508491
C 4.126241 -0.037513 -0.158071
C 3.611202 1.279640 -0.735711
C 2.296657 1.488826 -0.943523
O 1.122161 -3.216751 -0.180352
O 3.504141 -2.335417 -0.777032
O 5.390058 -0.363728 -0.739300
C 4.253520 0.019203 1.373224
C 5.636839 0.314843 1.915949
C 6.319770 1.593525 1.511957
O 6.161443 -0.442264 2.737348
O 4.607284 2.200242 -0.995645
C 4.256207 3.453121 -1.571127
C -4.783629 -0.705412 -0.167030
C -5.614484 -1.718754 -0.907786
C -5.275359 0.351577 0.514422
C -6.730637 0.731597 0.696249
C -7.013945 2.035137 -0.071897
C -8.481131 2.441680 -0.055668
C -7.033835 0.859112 2.192383
H -1.391476 -3.589627 0.028093
H -2.736352 1.076626 -0.543697
H -0.380102 1.783224 -0.784148
H 1.912654 2.425255 -1.333628
H 2.081119 -3.302903 -0.387197
H 5.233227 -0.591229 -1.672530
H 3.970165 -0.952980 1.801304
H 3.556013 0.742077 1.814813
H 6.725110 1.507726 0.502744
H 5.616033 2.426727 1.578370
H 7.145910 1.794663 2.200724
H 3.784201 3.315926 -2.549251
H 3.607869 4.023136 -0.898109
H 5.176835 4.025639 -1.717431
H -5.732689 -2.629614 -0.311515
H -6.610196 -1.345774 -1.160952
H -5.143145 -1.987159 -1.860611
H -4.580793 1.010414 1.036161
H -7.386005 -0.052546 0.304192
H -6.412513 2.857330 0.336554
H -6.706797 1.912450 -1.118757
H -8.812886 2.709618 0.951569
H -8.634732 3.315682 -0.696924
H -9.117416 1.632836 -0.428307
H -6.748119 -0.052977 2.728957
H -8.102969 1.015222 2.367293
H -6.491600 1.698228 2.642657
SCF Energy (B3LYP/6-31G**)= -1361.69578891
Number of imaginary frequencies = 0

1a_c452
MMFF Geometry

C -1.844252 -1.239168 -1.042667
C -2.195025 1.266747 -0.055151
N -3.110489 -0.802999 -0.839157
C -0.693477 -0.493167 -0.778623
C -0.886502 0.802712 -0.267110
C -3.283318 0.445738 -0.347833
C 0.233665 1.606868 0.014746
C 1.537036 1.133822 -0.189926
C 1.721747 -0.158166 -0.686836
C 0.616782 -0.964287 -0.996829
C 3.105600 -0.650033 -0.877377
C 4.243093 0.036799 -0.082933
C 3.935634 1.530177 0.080866
C 2.680786 2.014493 0.051851
O 0.768576 -2.228924 -1.512667
O 3.268751 -1.628271 -1.609044
O 5.476473 -0.098555 -0.788399
C 4.359632 -0.621431 1.301352
C 5.334087 -1.780421 1.390894
C 5.144605 -2.963120 0.479324
O 6.220505 -1.786017 2.249676
O 5.076480 2.293230 0.256154
C 4.938050 3.688642 0.501143
C -4.693636 0.894230 -0.153878
C -4.948303 2.376031 -0.220117
C -5.657926 -0.031686 0.046954
C -7.137832 0.182446 0.275991
C -7.571742 -0.267646 1.686626
C -7.441187 -1.757292 1.980144
C -7.940404 -0.510073 -0.828091
H -1.777530 -2.249469 -1.440840
H -2.353060 2.260916 0.349727
H 0.080584 2.615742 0.396957
H 2.462846 3.069079 0.181326
H 1.712330 -2.355484 -1.764850
H 5.689988 0.767527 -1.179413
H 3.388692 -0.970516 1.673645
H 4.715843 0.117168 2.033380
H 5.474956 -2.721561 -0.532073
H 5.742063 -3.802627 0.847438
H 4.095366 -3.268183 0.482127
H 4.478118 4.190947 -0.355783
H 4.366143 3.869943 1.416813
H 5.939456 4.106632 0.639693
H -4.383948 2.832989 -1.041276
H -5.994844 2.624781 -0.409698
H -4.655187 2.858813 0.718005
H -5.360556 -1.082219 0.053967
H -7.380671 1.247765 0.216155
H -6.990326 0.286732 2.434667
H -8.619900 0.019928 1.839198
H -6.404726 -2.097023 1.900704
H -7.777585 -1.963841 3.001688
H -8.057965 -2.356221 1.304080
H -7.709284 -0.071552 -1.805661
H -9.015966 -0.392960 -0.657883
H -7.720351 -1.581294 -0.888890
SCF Energy (B3LYP/6-31G**)= -1361.69874387
Number of imaginary frequencies = 0

1a_c453
MMFF Geometry
C 1.600804 2.232501 -0.199027
C 2.220030 -0.401238 -0.449212
N 2.910780 1.888922 -0.237153
C 0.534638 1.330394 -0.278310
C 0.868160 -0.029228 -0.409827
C 3.207647 0.576850 -0.352892
C -0.159083 -0.984680 -0.499649
C -1.509892 -0.608890 -0.458770
C -1.840803 0.742690 -0.325850
C -0.823859 1.708734 -0.236592
C -3.270713 1.136226 -0.269493
C -4.371550 0.065148 -0.393544
C -3.841727 -1.341581 -0.546673
C -2.537349 -1.641633 -0.542922
O -1.102486 3.048877 -0.102994
O -3.552187 2.334246 -0.156095
O -5.024545 0.427982 -1.631625
C -5.395864 0.230307 0.738789
C -4.970065 -0.347325 2.072493
C -3.842381 0.323646 2.811264
O -5.535591 -1.329854 2.553030
O -4.748384 -2.392613 -0.550315
C -5.569478 -2.420710 -1.716432
C 4.652685 0.243708 -0.396097
C 5.462710 1.165646 -1.264964
C 5.130680 -0.792778 0.326413
C 6.550572 -1.303200 0.478592
C 7.516571 -0.241425 1.037592
C 8.860222 -0.819138 1.464510
C 7.051166 -1.981052 -0.799158
H 1.422213 3.301091 -0.099315
H 2.500448 -1.443521 -0.567574
H 0.102702 -2.037864 -0.602013
H -2.225387 -2.681234 -0.602133
H -2.077520 3.175456 -0.116219
H -5.288651 1.364555 -1.548833
H -6.341986 -0.251103 0.456769
H -5.659311 1.283681 0.896684
H -3.907567 0.074379 3.874403
H -2.883458 -0.023622 2.423660
H -3.923026 1.409082 2.713986
H -6.394124 -1.708346 -1.624439
H -4.989000 -2.230599 -2.625846
H -6.002990 -3.422235 -1.794746
H 6.448226 0.766522 -1.508039
H 4.959051 1.336231 -2.223675
H 5.606309 2.133445 -0.772690
H 4.425895 -1.355480 0.940767
H 6.489926 -2.096743 1.238150
H 7.703092 0.549086 0.303967
H 7.058869 0.241607 1.910411
H 9.419352 -1.207819 0.608407
H 9.471020 -0.040458 1.932776
H 8.728789 -1.627454 2.190649
H 6.294426 -2.660838 -1.207001
H 7.945698 -2.579528 -0.597647
H 7.308361 -1.257685 -1.577865
SCF Energy (B3LYP/6-31G**)= -1361.69145479
Number of imaginary frequencies = 0

1a_c454
MMFF Geometry
C 1.927964 1.918547 -0.031968
C 2.342866 -0.751454 0.232148
N 3.202901 1.480461 0.104525
C 0.798769 1.095769 -0.045877
C 1.026089 -0.283800 0.090711
C 3.406729 0.149515 0.235636
C -0.070011 -1.164467 0.080643
C -1.384551 -0.693445 -0.059038
C -1.609984 0.679514 -0.191739
C -0.522340 1.570414 -0.188781
C -3.001211 1.178883 -0.331568
C -4.184411 0.189287 -0.270603
C -3.757603 -1.260306 -0.193281
C -2.484383 -1.652185 -0.064379
O -0.694126 2.928824 -0.320662
O -3.172003 2.393144 -0.487905
O -4.857156 0.392912 -1.518076
C -5.040386 0.598455 0.941549
C -6.476145 0.140200 0.845293
C -6.970852 -0.826790 1.887441
O -7.228735 0.595282 -0.017711
O -4.751986 -2.227501 -0.118537
C -5.355657 -2.478613 -1.390110
C 4.822787 -0.293891 0.399343
C 5.040609 -1.558556 1.182392
C 5.812100 0.463738 -0.125275
C 7.314126 0.262332 -0.138083
C 7.745595 -1.089182 -0.739064

C 9.236974 -1.159273 -1.044061
C 7.931796 0.550839 1.232125
H 1.834642 2.997681 -0.133966
H 2.527557 -1.816720 0.324040
H 0.108739 -2.234724 0.182483
H -2.252202 -2.708913 0.036572
H -1.650972 3.113651 -0.460838
H -5.423518 1.183603 -1.423504
H -5.094055 1.690513 1.043998
H -4.596106 0.236686 1.877176
H -7.943614 -1.224780 1.585783
H -6.274088 -1.660978 1.992120
H -7.073404 -0.306854 2.842989
H -6.143077 -1.747609 -1.592076
H -4.617952 -2.488659 -2.200145
H -5.823788 -3.466533 -1.345382
H 4.838086 -2.437060 0.560962
H 6.056901 -1.649626 1.568680
H 4.387106 -1.592818 2.061769
H 5.519891 1.377616 -0.649128
H 7.700674 1.037540 -0.816268
H 7.497253 -1.918414 -0.069599
H 7.196936 -1.263053 -1.673539
H 9.835667 -1.111686 -0.129819
H 9.472735 -2.103624 -1.545383
H 9.543207 -0.341363 -1.703741
H 7.541024 1.484973 1.651471
H 9.018214 0.662596 1.155154
H 7.732650 -0.249134 1.950897
SCF Energy (B3LYP/6-31G**)= -1361.69691281
Number of imaginary frequencies = 0

1b_c001
MMFF Geometry
C -1.565233 -2.477100 -0.049091
C -2.305219 0.134864 0.049504
N -2.880280 -2.196172 0.120318
C -0.552657 -1.520807 -0.179065
C -0.947732 -0.172730 -0.124986
C -3.237529 -0.894785 0.160050
C 0.024882 0.835880 -0.246982
C 1.383086 0.525939 -0.420157
C 1.772205 -0.815975 -0.468549
C 0.810706 -1.834939 -0.355642
C 3.204115 -1.143185 -0.655821
C 4.246226 -0.012453 -0.669814
C 3.671274 1.390696 -0.664565
C 2.350698 1.619049 -0.551696
O 1.151625 -3.166545 -0.415779
O 3.535159 -2.320937 -0.837499
O 4.931266 -0.185619 -1.933465
C 5.286100 -0.240264 0.434792
C 4.751546 0.061822 1.815634
C 5.740007 0.579441 2.826202
O 3.579533 -0.153746 2.122726
O 4.641586 2.374799 -0.759182
C 4.225277 3.727624 -0.899001
C -4.683239 -0.629870 0.353146
C -5.345165 -1.521668 1.368787
C -5.306757 0.326863 -0.367425
C -6.772442 0.702040 -0.328084
C -6.970916 2.146532 0.167254
C -6.533164 2.351319 1.612269
C -7.370838 0.541349 -1.729477
H -1.334957 -3.539994 -0.077917
H -2.630153 1.168697 0.112911
H -0.287231 1.878744 -0.203750
H 1.944647 2.624003 -0.534792
H 2.119574 -3.233316 -0.576769
H 5.134515 -1.137843 -2.008145
H 6.164441 0.391515 0.250365
H 5.652961 -1.273895 0.435875
H 5.234047 0.757775 3.779077
H 6.530654 -0.159678 2.976229
H 6.165057 1.522300 2.473130
H 3.603931 3.857587 -1.790993
H 3.700832 4.068716 -0.000781
H 5.120998 4.344139 -1.019232
H -4.708592 -1.642349 2.253237
H -6.293207 -1.119435 1.734035
H -5.538261 -2.512262 0.943696
H -4.728302 0.894418 -1.096939
H -7.333450 0.034088 0.332833
H -6.425200 2.849486 -0.474905
H -8.033058 2.410893 0.090433
H -5.455586 2.203744 1.731703
H -6.766088 3.371658 1.933441
H -7.053936 1.658999 2.281381
H -8.442282 0.769252 -1.720964
H -7.252795 -0.486436 -2.090838
H -6.891334 1.210912 -2.452648
SCF Energy (B3LYP/6-31G**)= -1361.71052120
Number of imaginary frequencies = 0

1b_c002
MMFF Geometry
C -1.645736 -2.347879 -0.085431
C -2.254090 0.232961 -0.675783
N -2.954906 -2.010462 -0.181241
C -0.576144 -1.465480 -0.269696
C -0.903514 -0.133506 -0.577790
C -3.245785 -0.722890 -0.464977
C 0.128366 0.799591 -0.784525
C 1.479290 0.431541 -0.682999
C 1.800250 -0.892103 -0.367668
C 0.780129 -1.838432 -0.169340
C 3.225113 -1.281469 -0.264401
C 4.317182 -0.205274 -0.382380
C 3.821637 1.171225 -0.781218
C 2.511949 1.445002 -0.917533
O 1.055595 -3.154687 0.121551
O 3.515694 -2.475360 -0.123362
O 5.160241 -0.681163 -1.458926
C 5.181017 -0.189514 0.885262
C 4.477716 0.436032 2.067559
C 5.343658 1.152522 3.069082
O 3.264438 0.322838 2.239371
O 4.846163 2.087222 -0.955023
C 4.524914 3.377485 -1.460018
C -4.688482 -0.395597 -0.561499
C -5.512679 -1.432359 -1.276117
C -5.172921 0.736261 -0.007048
C -6.613231 1.199209 0.032809
C -7.089095 1.437012 1.479154
C -7.114973 0.164968 2.317799
C -6.749194 2.487066 -0.783513
H -1.469873 -3.394561 0.153577
H -2.529257 1.252336 -0.927791
H -0.131429 1.829790 -1.025460
H 2.160874 2.433792 -1.189811
H 2.031687 -3.275219 0.125270
H 5.321264 -1.628363 -1.284280
H 6.108413 0.364976 0.692593
H 5.489263 -1.201364 1.175314
H 4.721197 1.557890 3.871571
H 6.064094 0.451495 3.497408
H 5.864668 1.978376 2.578347
H 4.040129 3.306996 -2.439113
H 3.900391 3.929267 -0.750306
H 5.459867 3.931483 -1.585763
H -5.712063 -2.285348 -0.618881
H -6.471584 -1.043872 -1.627774
H -4.990396 -1.797145 -2.168413
H -4.484472 1.400710 0.515983
H -7.279844 0.455089 -0.413897
H -6.451888 2.180207 1.975019
H -8.103115 1.855783 1.460217
H -6.110538 -0.245934 2.457562
H -7.527965 0.377779 3.309072
H -7.740072 -0.601248 1.848288
H -7.789904 2.828595 -0.797598
H -6.438109 2.327780 -1.822117

H -6.136310 3.294911 -0.367603
SCF Energy (B3LYP/6-31G**)= -1361.71063116
Number of imaginary frequencies = 0

1b_c003
MMFF Geometry
C 1.593008 2.441653 -0.130500
C 2.338415 -0.163397 0.065699
N 2.911318 2.168993 0.026737
C 0.579517 1.480226 -0.201891
C 0.977748 0.135608 -0.097850
C 3.271046 0.870638 0.115690
C 0.005293 -0.878238 -0.159852
C -1.354777 -0.575542 -0.323415
C -1.749167 0.763326 -0.420396
C -0.787387 1.786407 -0.367129
C -3.186754 1.081890 -0.599442
C -4.232314 -0.048905 -0.560044
C -3.635996 -1.438624 -0.522732
C -2.323768 -1.668347 -0.386640
O -1.130847 3.114077 -0.477400
O -3.522913 2.250266 -0.827408
O -4.943827 0.101656 -1.809775
C -5.241120 0.197725 0.566994
C -4.654323 -0.074649 1.932450
C -5.524452 -0.808778 2.916589
O -3.531435 0.323262 2.241532
O -4.506766 -2.519274 -0.447964
C -5.133576 -2.795255 -1.700809
C 4.720365 0.615220 0.294216
C 5.399043 1.550601 1.258285
C 5.332247 -0.371677 -0.394970
C 6.798813 -0.744232 -0.365251
C 7.007698 -2.165596 0.189189
C 6.595836 -2.307143 1.649314
C 7.372117 -0.644782 -1.782727
H 1.360860 3.502262 -0.199749
H 2.665601 -1.193490 0.167504
H 0.317413 -1.918834 -0.077383
H -1.959806 -2.687629 -0.294348
H -2.102777 3.174703 -0.613842
H -5.119816 1.058749 -1.908801
H -6.122749 -0.439533 0.422085
H -5.613906 1.229147 0.560288
H -4.983918 -0.943673 3.857514
H -6.430592 -0.228705 3.107257
H -5.781781 -1.792233 2.515478
H -6.013484 -2.159694 -1.834208
H -4.440293 -2.682419 -2.541738
H -5.474985 -3.834588 -1.680206
H 4.778201 1.709053 2.147942
H 6.354042 1.165217 1.623569
H 5.583097 2.521981 0.787316
H 4.741601 -0.970996 -1.088435
H 7.370668 -0.047789 0.255755
H 6.451515 -2.896178 -0.411833
H 8.068619 -2.432657 0.105175
H 5.520369 -2.154907 1.781274
H 6.835598 -3.312364 2.010618
H 7.127564 -1.586012 2.278167
H 8.443806 -0.871693 -1.783263
H 7.246515 0.366167 -2.186542
H 6.880653 -1.345520 -2.467302
SCF Energy (B3LYP/6-31G**)= -1361.70481481
Number of imaginary frequencies = 0

1b_c004
MMFF Geometry
C 1.665711 2.314127 0.045765
C 2.282320 -0.227909 -0.687645
N 2.975481 1.992154 -0.087802
C 0.599233 1.434691 -0.168093
C 0.931079 0.122816 -0.550440
C 3.270648 0.723166 -0.442390
C -0.096864 -0.806329 -0.790120
C -1.447228 -0.452622 -0.650212
C -1.774540 0.850449 -0.259847
C -0.757916 1.792263 -0.027400
C -3.202775 1.224847 -0.117765
C -4.295939 0.152547 -0.286868
C -3.779770 -1.181510 -0.778931
C -2.476302 -1.456670 -0.914947
O -1.037335 3.089467 0.335960
O -3.499787 2.407973 0.087368
O -5.159239 0.704594 -1.307055
C -5.128630 0.034281 0.994297
C -4.380465 -0.669088 2.102700
C -5.152466 -1.667756 2.922029
O -3.204475 -0.406417 2.353259
O -4.692517 -2.219421 -0.924738
C -5.495689 -2.071397 -2.095930
C 4.713913 0.412243 -0.577442
C 5.520308 1.491399 -1.248215
C 5.214239 -0.743723 -0.091001
C 6.657967 -1.197562 -0.097065
C 7.157249 -1.508111 1.327430
C 7.187513 -0.282099 2.231901
C 6.789966 -2.439359 -0.982482
H 1.486702 3.345467 0.342189
H 2.560167 -1.230882 -0.996333
H 0.163996 -1.821468 -1.087618
H -2.161613 -2.455199 -1.204473
H -2.013771 3.200378 0.370781
H -5.292434 1.644466 -1.071056
H -6.056405 -0.513129 0.784659
H -5.439812 1.016977 1.368887
H -4.499180 -2.104267 3.682548
H -5.988342 -1.166964 3.416444
H -5.519822 -2.467342 2.273950
H -6.348123 -1.417217 -1.892522
H -4.915712 -1.701798 -2.948902
H -5.889572 -3.058480 -2.356162
H 5.723740 2.310028 -0.549813
H 6.476457 1.129250 -1.633841
H 4.982293 1.898776 -2.112289
H 4.538194 -1.440103 0.406094
H 7.312850 -0.425965 -0.513478
H 6.532511 -2.281174 1.792541
H 8.173584 -1.917840 1.271307
H 6.182645 0.113446 2.408107
H 7.616868 -0.544059 3.204272
H 7.800396 0.512467 1.794506
H 7.832557 -2.772015 -1.030057
H 6.462137 -2.227594 -2.006458
H 6.188759 -3.272570 -0.600995
SCF Energy (B3LYP/6-31G**)= -1361.70493633
Number of imaginary frequencies = 0

1b_c005
MMFF Geometry
C -1.838321 -1.631362 -0.338159
C -2.059222 1.061987 -0.084063
N -3.077960 -1.103168 -0.193300
C -0.653763 -0.891270 -0.368536
C -0.780249 0.501437 -0.235891
C -3.184901 0.239535 -0.066338
C 0.376322 1.302021 -0.259434
C 1.654055 0.739861 -0.409664
C 1.774562 -0.647125 -0.535424
C 0.627874 -1.459431 -0.522207
C 3.121388 -1.240317 -0.698450
C 4.364425 -0.339908 -0.604410
C 4.075853 1.146565 -0.523071
C 2.821931 1.625200 -0.436829
O 0.702946 -2.826123 -0.661542
O 3.222911 -2.447543 -0.947396
O 5.056551 -0.569874 -1.855094
C 5.290462 -0.834908 0.514030
C 4.765609 -0.517232 1.895134
C 5.791385 -0.276867 2.970447
O 3.561572 -0.507824 2.149220
O 5.223393 1.922490 -0.520876

C 5.086895 3.336904 -0.582106
C -4.566406 0.785309 0.075869
C -4.788926 2.201044 -0.383164
C -5.542826 -0.006648 0.572198
C -6.995288 0.336414 0.817216
C -7.931320 -0.487393 -0.086335
C -7.764124 -0.175781 -1.568333
C -7.329959 0.079285 2.290489
H -1.822183 -2.714882 -0.433638
H -2.162964 2.135566 0.032569
H 0.273713 2.381683 -0.156302
H 2.620406 2.687897 -0.365303
H 1.645213 -3.074443 -0.794709
H 5.072723 -1.537337 -1.986890
H 6.282410 -0.379150 0.400187
H 5.447987 -1.918809 0.456273
H 5.289188 -0.058990 3.917071
H 6.409053 -1.169860 3.092840
H 6.413453 0.579482 2.698411
H 4.543007 3.640795 -1.482354
H 4.600394 3.720826 0.320172
H 6.090488 3.769503 -0.632315
H -4.269591 2.391486 -1.329482
H -5.840472 2.428157 -0.574731
H -4.424188 2.910749 0.366643
H -5.282399 -1.029678 0.853005
H -7.187055 1.397998 0.633161
H -7.770045 -1.561014 0.073835
H -8.972777 -0.281752 0.191215
H -6.771062 -0.458667 -1.930182
H -8.502114 -0.733233 -2.154103
H -7.913337 0.890860 -1.763778
H -8.367104 0.358497 2.505905
H -6.683386 0.670640 2.948615
H -7.205358 -0.977087 2.554667
SCF Energy (B3LYP/6-31G**)= -1361.71156443
Number of imaginary frequencies = 0

1b_c006
MMFF Geometry
C -1.805002 -1.760248 -0.124546
C -2.086761 0.901868 0.324370
N -3.046123 -1.285600 0.141963
C -0.648294 -0.979281 -0.188718
C -0.806523 0.397126 0.043181
C -3.182807 0.041273 0.367685
C 0.319840 1.237991 -0.014811
C 1.599232 0.731329 -0.293379
C 1.752282 -0.640341 -0.515151
C 0.635008 -1.491613 -0.470935
C 3.100695 -1.174824 -0.812616
C 4.319264 -0.238854 -0.751413
C 3.992224 1.228982 -0.556566
C 2.734157 1.657222 -0.349850
O 0.740898 -2.843513 -0.703430
O 3.220225 -2.360265 -1.144454
O 4.924205 -0.368615 -2.060370
C 5.339764 -0.767786 0.264580
C 4.908972 -0.552253 1.697005
C 6.003707 -0.342396 2.708849
O 3.727225 -0.599363 2.037032
O 5.112437 2.043210 -0.589140
C 4.928354 3.453105 -0.553590
C -4.560285 0.524480 0.675158
C -4.673898 1.776020 1.502821
C -5.623912 -0.180390 0.229792
C -7.093069 0.134729 0.400864
C -7.826145 0.155501 -0.954862
C -7.340474 1.263628 -1.881341
C -7.727670 -0.907199 1.325192
H -1.763423 -2.834240 -0.292508
H -2.218217 1.965378 0.493650
H 0.192290 2.305098 0.163637
H 2.506064 2.705974 -0.197705
H 1.677841 -3.050045 -0.919631
H 4.959437 -1.325165 -2.253738
H 6.306355 -0.271794 0.108572
H 5.525325 -1.840118 0.127960
H 5.566475 -0.199400 3.700892
H 6.654943 -1.219529 2.730295
H 6.578329 0.549577 2.447126
H 4.309926 3.790133 -1.391786
H 4.498746 3.765940 0.403361
H 5.911663 3.922919 -0.649723
H -4.507041 2.663454 0.883520
H -5.649316 1.881086 1.984489
H -3.941908 1.774082 2.318708
H -5.435254 -1.091790 -0.341899
H -7.234435 1.117125 0.862145
H -7.717754 -0.810523 -1.464093
H -8.899925 0.299856 -0.781262
H -6.295675 1.117091 -2.171522
H -7.941009 1.277211 -2.796571
H -7.432300 2.243449 -1.401975
H -8.787494 -0.684577 1.489883
H -7.234517 -0.915700 2.303739
H -7.658140 -1.916339 0.903278
SCF Energy (B3LYP/6-31G**)= -1361.71162277
Number of imaginary frequencies = 0

1b_c007
MMFF Geometry
C -1.851220 -1.569249 -0.459802
C -2.090677 1.089228 0.033439
N -3.095905 -1.060947 -0.289157
C -0.670184 -0.825152 -0.403222
C -0.806541 0.549691 -0.147297
C -3.212100 0.264317 -0.042528
C 0.345302 1.354025 -0.078983
C 1.626715 0.811898 -0.259689
C 1.759078 -0.558634 -0.507084
C 0.616853 -1.372918 -0.585917
C 3.113661 -1.129984 -0.703976
C 4.355423 -0.236646 -0.521189
C 4.036958 1.229668 -0.329039
C 2.789610 1.694491 -0.183012
O 0.701139 -2.721067 -0.846878
O 3.226158 -2.308750 -1.061626
O 5.066311 -0.382101 -1.771920
C 5.259354 -0.800248 0.580667
C 4.690338 -0.574583 1.962115
C 5.653896 -0.150140 3.037309
O 3.500630 -0.771022 2.208505
O 5.096352 2.104252 -0.118181
C 5.808601 2.389983 -1.322226
C -4.598739 0.789615 0.125378
C -4.820466 2.241358 -0.203119
C -5.579634 -0.048545 0.528257
C -7.037793 0.265188 0.778679
C -7.953995 -0.473651 -0.214252
C -7.762907 -0.024181 -1.657456
C -7.396471 -0.129770 2.215350
H -1.827893 -2.639677 -0.652177
H -2.201667 2.147426 0.245396
H 0.237512 2.419702 0.120416
H 2.625874 2.748309 0.023246
H 1.647323 -2.955403 -0.976205
H 5.058262 -1.337925 -1.979624
H 6.251242 -0.334420 0.521262
H 5.427139 -1.877045 0.457382
H 5.118143 -0.018634 3.981444
H 6.419083 -0.919295 3.166983
H 6.115885 0.800769 2.760481
H 6.553599 1.613449 -1.517023
H 5.136774 2.509303 -2.179514
H 6.343078 3.333562 -1.176657
H -4.286791 2.520986 -1.118811
H -5.869804 2.482108 -0.390042
H -4.471296 2.879074 0.615616
H -5.318946 -1.092402 0.717058
H -7.231723 1.338667 0.691330
H -7.790099 -1.556994 -0.152340

H -9.001091 -0.298421 0.062944
H -6.762363 -0.268604 -2.026644
H -8.487909 -0.526878 -2.305666
H -7.914088 1.055557 -1.754919
H -8.438554 0.124560 2.437636
H -6.764287 0.399605 2.937148
H -7.271145 -1.205797 2.381776
SCF Energy (B3LYP/6-31G**)= -1361.70594775
Number of imaginary frequencies = 0

1b_c008
MMFF Geometry
C -1.821092 -1.705723 -0.326218
C -2.120138 0.874548 0.461978
N -3.068416 -1.271409 -0.022390
C -0.666132 -0.921805 -0.267399
C -0.833569 0.412604 0.139656
C -3.213681 0.014337 0.372840
C 0.289941 1.255686 0.212145
C 1.574110 0.790752 -0.109229
C 1.738400 -0.541004 -0.504881
C 0.623779 -1.391955 -0.591379
C 3.095532 -1.030313 -0.849649
C 4.313776 -0.106797 -0.658847
C 3.956514 1.317744 -0.296312
C 2.706542 1.711013 -0.020310
O 0.738162 -2.701694 -0.996616
O 3.225287 -2.161235 -1.333628
O 4.939640 -0.095466 -1.962309
C 5.313003 -0.738119 0.316755
C 4.835341 -0.676359 1.748950
C 5.857180 -0.322639 2.795560
O 3.673504 -0.945875 2.052609
O 4.995655 2.212032 -0.068068
C 5.608970 2.648161 -1.281461
C -4.598031 0.451396 0.716584
C -4.730254 1.584666 1.697718
C -5.651373 -0.191210 0.165182
C -7.124353 0.096880 0.350407
C -7.834549 0.292281 -1.003576
C -7.337436 1.511964 -1.770022
C -7.770584 -1.057109 1.120740
H -1.772878 -2.748601 -0.631921
H -2.257964 1.906591 0.766896
H 0.157803 2.290311 0.526999
H 2.519506 2.730875 0.303387
H 1.680791 -2.880647 -1.211857
H 4.951404 -1.025103 -2.266346
H 6.280691 -0.226269 0.239527
H 5.510502 -1.788963 0.072701
H 5.385188 -0.309155 3.781845
H 6.655678 -1.068511 2.793804
H 6.265411 0.669654 2.588198
H 6.366092 1.928476 -1.605176
H 4.873625 2.823450 -2.074604
H 6.117533 3.594835 -1.075623
H -4.555674 2.545241 1.202002
H -5.714244 1.625209 2.171634
H -4.012578 1.477499 2.519266
H -5.449705 -1.020360 -0.516630
H -7.277127 1.010914 0.932696
H -7.714017 -0.599348 -1.631856
H -8.911629 0.411301 -0.831205
H -6.287359 1.405865 -2.058748
H -7.922503 1.643281 -2.685951
H -7.440958 2.421173 -1.169278
H -8.833852 -0.859313 1.294661
H -7.293973 -1.191764 2.098255
H -7.690210 -2.002875 0.572765
SCF Energy (B3LYP/6-31G**)= -1361.70586962
Number of imaginary frequencies = 0

1b_c009
MMFF Geometry
C -1.456041 -2.501346 -0.133339
C -2.231288 0.089562 0.122927
N -2.772766 -2.249714 0.066392
C -0.458531 -1.524560 -0.219505
C -0.871741 -0.187721 -0.083944
C -3.147888 -0.958085 0.184781
C 0.085231 0.840285 -0.158820
C 1.445476 0.560611 -0.364516
C 1.852671 -0.770521 -0.493941
C 0.906882 -1.808185 -0.428883
C 3.286669 -1.065567 -0.715511
C 4.312669 0.079220 -0.676341
C 3.718306 1.471235 -0.584419
C 2.396111 1.673462 -0.444015
O 1.265581 -3.129071 -0.568930
O 3.631918 -2.226073 -0.967952
O 4.984949 -0.011532 -1.955357
C 5.368711 -0.196040 0.401805
C 4.846493 0.018925 1.803520
C 5.839613 0.492426 2.831014
O 3.681345 -0.230912 2.110770
O 4.673600 2.473202 -0.633328
C 4.236826 3.825574 -0.690875
C -4.595159 -0.726848 0.407779
C -5.235389 -1.690179 1.370842
C -5.241231 0.263577 -0.244144
C -6.715147 0.604959 -0.169030
C -6.885860 1.962204 0.536865
C -8.340716 2.343313 0.774203
C -7.303804 0.605477 -1.583266
H -1.211377 -3.557390 -0.225577
H -2.569519 1.113318 0.248848
H -0.240839 1.874321 -0.052520
H 1.976313 2.669776 -0.365122
H 2.232381 -3.172412 -0.744198
H 5.200546 -0.954874 -2.086664
H 6.235896 0.457992 0.244092
H 5.749936 -1.222582 0.339664
H 5.342610 0.608690 3.798091
H 6.642227 -0.242421 2.929712
H 6.247230 1.460002 2.527774
H 3.603143 3.997133 -1.567018
H 3.718414 4.107162 0.231156
H 5.122364 4.460963 -0.785565
H -4.582086 -1.871429 2.232477
H -6.176096 -1.315036 1.781920
H -5.435546 -2.648757 0.880691
H -4.679538 0.889041 -0.938525
H -7.258433 -0.154663 0.401866
H -6.379743 1.929982 1.510579
H -6.399796 2.757608 -0.042471
H -8.868266 1.557760 1.324243
H -8.395275 3.264164 1.363897
H -8.867853 2.523145 -0.167161
H -8.390926 0.730333 -1.559051
H -7.098449 -0.342784 -2.093250
H -6.884283 1.413369 -2.193345
SCF Energy (B3LYP/6-31G**)= -1361.70939963
Number of imaginary frequencies = 0

1b_c010
MMFF Geometry
C 1.510506 2.459152 0.148568
C 2.206268 -0.045750 -0.639253
N 2.830550 2.172596 0.037365
C 0.471386 1.560336 -0.114046
C 0.843876 0.268252 -0.523934
C 3.165027 0.921617 -0.344969
C -0.155770 -0.678067 -0.812708
C -1.518565 -0.362768 -0.693771
C -1.884412 0.920646 -0.277257
C -0.896909 1.880444 0.004067
C -3.321808 1.254677 -0.155525
C -4.377454 0.156800 -0.367674
C -3.834899 -1.167042 -0.870134
C -2.516192 -1.386975 -1.016620
O -1.216899 3.159905 0.395999
O -3.652030 2.423644 0.077226

O -5.229226 0.689471 -1.410227
C -5.247783 0.012639 0.887364
C -4.531429 -0.682011 2.022293
C -5.379428 -1.503181 2.956551
O -3.323519 -0.544015 2.212785
O -4.827938 -2.098710 -1.124656
C -4.461772 -3.333791 -1.727561
C 4.618321 0.649764 -0.455697
C 5.410981 1.765509 -1.081782
C 5.136887 -0.507116 0.009174
C 6.592480 -0.926337 0.020469
C 7.017838 -1.250787 1.465717
C 8.502483 -1.558575 1.606031
C 6.776096 -2.118330 -0.921444
H 1.299165 3.477357 0.468244
H 2.515968 -1.032473 -0.969112
H 0.138925 -1.676991 -1.132327
H -2.131430 -2.339227 -1.363420
H -2.196411 3.248392 0.401009
H -5.422112 1.614138 -1.162432
H -6.155428 -0.554244 0.643506
H -5.590605 0.987827 1.254062
H -4.748815 -1.950878 3.729638
H -6.124847 -0.861843 3.432961
H -5.870338 -2.303700 2.397547
H -3.973778 -3.170205 -2.693850
H -3.823769 -3.919918 -1.058537
H -5.377386 -3.905741 -1.904725
H 5.582139 2.569318 -0.358015
H 6.382113 1.435934 -1.459310
H 4.879359 2.183834 -1.944609
H 4.467344 -1.234079 0.470085
H 7.231942 -0.113875 -0.338050
H 6.785038 -0.394441 2.112085
H 6.441249 -2.100973 1.852081
H 9.112837 -0.744363 1.202975
H 8.760751 -1.683759 2.662589
H 8.771726 -2.484105 1.089077
H 7.833164 -2.382540 -1.024591
H 6.401938 -1.885726 -1.925118
H 6.239944 -3.002881 -0.559497
SCF Energy (B3LYP/6-31G**)= -1361.70950905
Number of imaginary frequencies = 0

1b_c011
MMFF Geometry
C -1.485647 -2.460545 -0.245930
C -2.265356 0.112402 0.144241
N -2.805644 -2.219413 -0.054937
C -0.486760 -1.481516 -0.259740
C -0.902637 -0.153860 -0.055469
C -3.182774 -0.936236 0.130791
C 0.054650 0.876209 -0.055822
C 1.416756 0.606273 -0.255799
C 1.828756 -0.716243 -0.452538
C 0.882200 -1.754742 -0.461705
C 3.268388 -0.999758 -0.669956
C 4.298318 0.140836 -0.561634
C 3.682981 1.515313 -0.418516
C 2.369410 1.715147 -0.251682
O 1.242937 -3.065912 -0.670351
O 3.618157 -2.143782 -0.984509
O 4.996538 0.090312 -1.826814
C 5.324214 -0.170361 0.533549
C 4.750389 -0.004720 1.921453
C 5.622060 0.670371 2.945681
O 3.637090 -0.440361 2.213898
O 4.539289 2.600767 -0.276538
C 5.146768 2.974614 -1.513424
C -4.633648 -0.716866 0.342265
C -5.290079 -1.733598 1.237106
C -5.268583 0.310380 -0.261874
C -6.743467 0.648563 -0.191713
C -6.925756 1.962524 0.589198
C -8.384322 2.330862 0.823914
C -7.308247 0.732270 -1.613192
H -1.239498 -3.509944 -0.393403
H -2.605438 1.127719 0.322851
H -0.271047 1.903520 0.104221
H 1.992280 2.719720 -0.082781
H 2.213884 -3.102163 -0.821636
H 5.184761 -0.854564 -1.995892
H 6.194940 0.488684 0.424641
H 5.711380 -1.193008 0.449046
H 5.091234 0.729822 3.899832
H 6.538555 0.091792 3.084289
H 5.860568 1.683629 2.612944
H 6.033844 2.363374 -1.701712
H 4.444903 2.911629 -2.352443
H 5.473727 4.014818 -1.422490
H -4.651487 -1.964909 2.097785
H -6.237650 -1.381960 1.653166
H -5.481852 -2.662085 0.689091
H -4.695156 0.974682 -0.909000
H -7.296439 -0.142429 0.324681
H -6.436100 1.872963 1.567730
H -6.429854 2.789790 0.065492
H -8.921210 1.515187 1.318255
H -8.448608 3.215812 1.465328
H -8.895511 2.565921 -0.114070
H -8.395596 0.856789 -1.599982
H -7.094535 -0.184873 -2.174112
H -6.878356 1.573873 -2.167970
SCF Energy (B3LYP/6-31G**)= -1361.70369152
Number of imaginary frequencies = 0

1b_c012
MMFF Geometry
C 1.534347 2.409076 0.298156
C 2.235733 -0.041271 -0.641973
N 2.854561 2.139551 0.151734
C 0.497526 1.519141 -0.001068
C 0.873142 0.255324 -0.489941
C 3.192008 0.914934 -0.306168
C -0.123414 -0.680724 -0.818765
C -1.485225 -0.381350 -0.663804
C -1.856094 0.873150 -0.167623
C -0.871196 1.822097 0.154933
C -3.296396 1.190588 -0.009574
C -4.354248 0.103971 -0.280948
C -3.792829 -1.166991 -0.879054
C -2.480366 -1.389898 -1.023961
O -1.193793 3.074519 0.624203
O -3.631744 2.342354 0.292186
O -5.225994 0.714592 -1.259736
C -5.193235 -0.148329 0.976505
C -4.432570 -0.920657 2.029090
C -5.179764 -2.008468 2.752271
O -3.267387 -0.644805 2.313661
O -4.671613 -2.215881 -1.122352
C -5.469484 -1.992917 -2.285269
C 4.645425 0.660908 -0.452428
C 5.421765 1.817008 -1.023252
C 5.178214 -0.517140 -0.062900
C 6.636520 -0.925585 -0.096020
C 7.084356 -1.330455 1.321888
C 8.572819 -1.634946 1.423651
C 6.814636 -2.059351 -1.108251
H 1.320923 3.405485 0.679199
H 2.547082 -1.005319 -1.031985
H 0.171318 -1.658412 -1.198662
H -2.132411 -2.350093 -1.393918
H -2.173458 3.152394 0.657855
H -5.390292 1.626437 -0.946142
H -6.101786 -0.703746 0.711138
H -5.537891 0.788949 1.429900
H -4.519408 -2.488245 3.479911
H -6.034786 -1.577202 3.278460
H -5.516817 -2.760645 2.034693
H -6.343489 -1.384623 -2.036143
H -4.894398 -1.534492 -3.097463
H -5.830330 -2.965693 -2.632681

H 5.597544 2.578757 -0.256386
H 6.389767 1.517237 -1.432339
H 4.875401 2.280648 -1.853080
H 4.520062 -1.274741 0.363890
H 7.265518 -0.088947 -0.415298
H 6.855098 -0.514916 2.020210
H 6.518862 -2.205909 1.665902
H 9.172074 -0.794185 1.060413
H 8.846792 -1.819503 2.467473
H 8.840796 -2.526815 0.849978
H 7.871853 -2.309311 -1.241110
H 6.424829 -1.771491 -2.091434
H 6.289461 -2.967397 -0.791171
SCF Energy (B3LYP/6-31G**)= -1361.70383718
Number of imaginary frequencies = 0

1b_c013
MMFF Geometry
C -1.529598 -2.536924 -0.166010
C -2.309055 0.045406 0.155532
N -2.845323 -2.293398 0.049760
C -0.535181 -1.555800 -0.237676
C -0.950552 -0.223498 -0.068316
C -3.222606 -1.005812 0.200491
C 0.003238 0.808488 -0.127157
C 1.362485 0.537177 -0.349898
C 1.771909 -0.789573 -0.512955
C 0.829221 -1.830953 -0.464223
C 3.204829 -1.075689 -0.752496
C 4.228288 0.070607 -0.695207
C 3.631253 1.458584 -0.566783
C 2.309720 1.654060 -0.411389
O 1.190048 -3.147340 -0.637275
O 3.550918 -2.229204 -1.034137
O 4.890636 0.010897 -1.981213
C 5.293513 -0.226443 0.368019
C 4.781890 -0.044958 1.778349
C 5.781949 0.407562 2.808563
O 3.619834 -0.304839 2.088957
O 4.583664 2.463924 -0.600267
C 4.143123 3.816102 -0.623429
C -4.668372 -0.783387 0.440199
C -5.300543 -1.770418 1.384482
C -5.321893 0.223773 -0.177794
C -6.795646 0.553573 -0.077072
C -7.048317 1.765041 0.841155
C -6.433800 3.080697 0.378684
C -7.386137 0.753324 -1.476473
H -1.283074 -3.589939 -0.284272
H -2.648675 1.065162 0.307655
H -0.324533 1.838955 0.005341
H 1.888112 2.647198 -0.306418
H 2.155532 -3.184106 -0.821094
H 5.107502 -0.928624 -2.135766
H 6.157811 0.433308 0.218497
H 5.676754 -1.250287 0.279407
H 5.292339 0.500373 3.781926
H 6.587132 -0.327219 2.884107
H 6.184760 1.382893 2.524342
H 3.502098 4.005965 -1.490407
H 3.631343 4.075176 0.308851
H 5.026318 4.455823 -0.710523
H -4.643269 -1.967214 2.239607
H -6.241639 -1.407242 1.806125
H -5.498868 -2.718948 0.874478
H -4.766477 0.867665 -0.859628
H -7.337054 -0.294300 0.357694
H -8.130983 1.908380 0.948226
H -6.668331 1.538911 1.845825
H -6.836831 3.394556 -0.588420
H -6.661391 3.870599 1.102202
H -5.345111 3.011670 0.296982
H -8.449056 1.010330 -1.415432
H -7.300085 -0.166433 -2.066389
H -6.876152 1.547996 -2.031326
SCF Energy (B3LYP/6-31G**)= -1361.70841638
Number of imaginary frequencies = 0

1b_c014
MMFF Geometry
C -1.559658 -2.495221 -0.275837
C -2.343448 0.067118 0.171618
N -2.878960 -2.261717 -0.071011
C -0.563489 -1.513307 -0.276677
C -0.981467 -0.191223 -0.042740
C -3.258169 -0.983622 0.143093
C -0.026994 0.841347 -0.028759
C 1.334412 0.579346 -0.243461
C 1.748585 -0.737760 -0.469748
C 0.804774 -1.778530 -0.493473
C 3.187440 -1.012762 -0.702688
C 4.215033 0.128305 -0.578333
C 3.597019 1.497868 -0.403218
C 2.284098 1.690588 -0.223404
O 1.167578 -3.084190 -0.731074
O 3.538098 -2.149200 -1.042719
O 4.904659 0.105349 -1.849015
C 5.249274 -0.202089 0.503294
C 4.684578 -0.066170 1.898153
C 5.561466 0.590526 2.929859
O 3.574491 -0.510775 2.189316
O 4.451376 2.582636 -0.245148
C 5.049309 2.983138 -1.478335
C -4.707881 -0.772500 0.368978
C -5.357346 -1.809117 1.245993
C -5.349684 0.268408 -0.203718
C -6.824708 0.594727 -0.110757
C -7.092626 1.753653 0.868912
C -6.468975 3.091917 0.491327
C -7.390195 0.873112 -1.507048
H -1.311718 -3.540732 -0.446161
H -2.684893 1.077688 0.373216
H -0.354344 1.864286 0.154268
H 1.905450 2.690473 -0.031628
H 2.137554 -3.114635 -0.889694
H 5.094243 -0.835380 -2.038470
H 6.117463 0.461473 0.401807
H 5.638586 -1.221723 0.395462
H 5.037064 0.629171 3.888621
H 6.480442 0.011850 3.050465
H 5.794958 1.610975 2.616080
H 5.936700 2.378334 -1.685024
H 4.341848 2.935162 -2.313639
H 5.374100 4.022207 -1.368596
H -4.715563 -2.053751 2.100537
H -6.305637 -1.468328 1.670195
H -5.547042 -2.727786 0.681007
H -4.781769 0.948357 -0.838652
H -7.374393 -0.275075 0.266505
H -8.176903 1.892735 0.964699
H -6.730661 1.471274 1.865973
H -6.854570 3.459834 -0.463756
H -6.708714 3.840725 1.253582
H -5.379065 3.025610 0.425121
H -8.453824 1.128210 -1.450525
H -7.294453 -0.012534 -2.145659
H -6.869846 1.696529 -2.007718
SCF Energy (B3LYP/6-31G**)= -1361.70271481
Number of imaginary frequencies = 0

1b_c015
MMFF Geometry
C -1.760305 -1.635634 -0.462943
C -1.995304 1.050672 -0.153181
N -3.004378 -1.114547 -0.330697
C -0.577896 -0.891506 -0.453577
C -0.711698 0.497504 -0.292356
C -3.118288 0.224649 -0.176165
C 0.442369 1.301855 -0.275216
C 1.724709 0.747008 -0.412840
C 1.852389 -0.636444 -0.567207
C 0.708442 -1.452254 -0.594695

C 3.204168 -1.221821 -0.716959
C 4.442043 -0.320053 -0.578401
C 4.146861 1.163356 -0.469504
C 2.889865 1.636203 -0.397229
O 0.790901 -2.815251 -0.763043
O 3.314668 -2.422852 -0.990800
O 5.159291 -0.519836 -1.820084
C 5.347704 -0.837268 0.546653
C 4.794844 -0.552232 1.923947
C 5.798557 -0.333055 3.024316
O 3.586041 -0.552078 2.154478
O 5.291483 1.942446 -0.427398
C 5.151381 3.357465 -0.459621
C -4.503716 0.763296 -0.047840
C -4.723560 2.188570 -0.478010
C -5.488541 -0.041467 0.409634
C -6.944983 0.301919 0.637538
C -7.806304 -0.400101 -0.427497
C -9.279526 -0.021336 -0.361776
C -7.339043 -0.099340 2.062684
H -1.738568 -2.716703 -0.582168
H -2.105146 2.121011 -0.014271
H 0.334068 2.378620 -0.149991
H 2.683350 2.696423 -0.305912
H 1.736437 -3.057715 -0.883181
H 5.181344 -1.484046 -1.973151
H 6.340126 -0.376116 0.462541
H 5.510032 -1.919126 0.467758
H 5.277193 -0.137970 3.965533
H 6.416730 -1.226749 3.138791
H 6.422929 0.531015 2.783798
H 4.624187 3.679869 -1.363353
H 4.646001 3.719588 0.441292
H 6.154282 3.794066 -0.480404
H -4.178504 2.407079 -1.403566
H -5.771583 2.410206 -0.695869
H -4.387119 2.881948 0.299735
H -5.234569 -1.072562 0.665601
H -7.102186 1.382413 0.558651
H -7.432405 -0.138116 -1.425920
H -7.714684 -1.489857 -0.334878
H -9.409445 1.063880 -0.419891
H -9.819934 -0.472119 -1.200487
H -9.745730 -0.378808 0.560854
H -8.349689 0.244045 2.304990
H -6.660352 0.348743 2.797581
H -7.310186 -1.186542 2.197048
SCF Energy (B3LYP/6-31G**)= -1361.71035280
Number of imaginary frequencies = 0

1b_c016
MMFF Geometry
C -1.740745 -1.728073 -0.031028
C -2.006555 0.965202 0.177202
N -2.980924 -1.223244 0.178345
C -0.577362 -0.963761 -0.150965
C -0.727337 0.428828 -0.043656
C -3.109695 0.119314 0.284130
C 0.406108 1.253550 -0.163058
C 1.684425 0.715491 -0.381118
C 1.829147 -0.671446 -0.478826
C 0.704862 -1.507818 -0.372113
C 3.176464 -1.239363 -0.712457
C 4.401565 -0.309978 -0.720509
C 4.083906 1.171545 -0.660115
C 2.827071 1.624982 -0.506440
O 0.802699 -2.875693 -0.482775
O 3.290299 -2.450333 -0.936659
O 5.019479 -0.559344 -2.005871
C 5.406957 -0.753735 0.349863
C 4.962525 -0.409172 1.752587
C 6.047951 -0.117901 2.754250
O 3.776871 -0.417931 2.081998
O 5.210715 1.972030 -0.751916
C 5.037256 3.380749 -0.843446
C -4.486552 0.637287 0.532684
C -4.598380 1.956736 1.247681
C -5.551420 -0.097312 0.141321
C -7.020973 0.241415 0.271480
C -7.693593 0.129980 -1.110931
C -9.148370 0.579744 -1.115384
C -7.660004 -0.687864 1.305495
H -1.705732 -2.812982 -0.102762
H -2.131557 2.040397 0.250241
H 0.284974 2.333117 -0.081243
H 2.605541 2.684620 -0.450350
H 1.740265 -3.106950 -0.669192
H 5.049319 -1.529485 -2.113381
H 6.379004 -0.280123 0.161553
H 5.585604 -1.835195 0.310845
H 5.601235 0.115424 3.724701
H 6.692082 -0.994085 2.860681
H 6.632260 0.743441 2.421118
H 4.430494 3.646366 -1.715169
H 4.599864 3.780087 0.077098
H 6.025170 3.833483 -0.969618
H -4.419273 2.786131 0.555659
H -5.577385 2.109204 1.709159
H -3.873908 2.020706 2.067783
H -5.363883 -1.058177 -0.343268
H -7.150191 1.271915 0.616783
H -7.143536 0.749041 -1.831720
H -7.640496 -0.902383 -1.479674
H -9.246713 1.597458 -0.724859
H -9.540045 0.570507 -2.137732
H -9.776549 -0.085282 -0.515829
H -8.697512 -0.403205 1.506398
H -7.122088 -0.641485 2.259401
H -7.654497 -1.730062 0.966852
SCF Energy (B3LYP/6-31G**)= -1361.71038580
Number of imaginary frequencies = 0

1b_c017
MMFF Geometry
C -1.773777 -1.565787 -0.570986
C -2.026061 1.084299 -0.040460
N -3.022183 -1.061616 -0.415781
C -0.595012 -0.821389 -0.480524
C -0.738020 0.549095 -0.205518
C -3.144713 0.259478 -0.150746
C 0.411232 1.353475 -0.102784
C 1.696553 0.815645 -0.267893
C 1.835414 -0.550654 -0.534415
C 0.695986 -1.364810 -0.647601
C 3.194239 -1.117366 -0.714793
C 4.431124 -0.225710 -0.494563
C 4.107102 1.237032 -0.285249
C 2.856596 1.698150 -0.155379
O 0.787009 -2.708568 -0.928209
O 3.315050 -2.290196 -1.088868
O 5.165585 -0.350559 -1.733848
C 5.314963 -0.805743 0.615039
C 4.719783 -0.602618 1.988940
C 5.662395 -0.194275 3.088657
O 3.525922 -0.804189 2.209765
O 5.161151 2.109293 -0.040675
C 5.895516 2.414836 -1.226430
C -4.534541 0.780788 0.000476
C -4.753606 2.237415 -0.307947
C -5.523220 -0.063496 0.370004
C -6.984649 0.254552 0.602680
C -7.825469 -0.354805 -0.533416
C -9.301203 0.012497 -0.459495
C -7.400098 -0.270155 1.980878
H -1.745343 -2.633033 -0.779672
H -2.142643 2.138815 0.186440
H 0.298251 2.415740 0.111395
H 2.687573 2.748390 0.064420
H 1.735777 -2.939813 -1.043390
H 5.162748 -1.302978 -1.956770
H 6.307154 -0.337970 0.581683
H 5.486507 -1.880270 0.477899

H 5.108819 -0.078325 4.024513
H 6.426045 -0.964589 3.220501
H 6.128224 0.761389 2.835667
H 6.645102 1.642281 -1.419444
H 5.239761 2.547018 -2.094211
H 6.425877 3.356556 -1.055909
H -4.195341 2.536833 -1.202470
H -5.799041 2.474293 -0.522138
H -4.432001 2.861667 0.532135
H -5.268948 -1.112112 0.540013
H -7.145278 1.337339 0.615319
H -7.436528 -0.005973 -1.498986
H -7.730617 -1.448214 -0.534253
H -9.434890 1.098274 -0.425318
H -9.825927 -0.365295 -1.343023
H -9.780788 -0.425147 0.420756
H -8.416034 0.047938 2.235288
H -6.735405 0.114361 2.763057
H -7.368715 -1.364831 2.020804
SCF Energy (B3LYP/6-31G**)= -1361.70475538
Number of imaginary frequencies = 0

1b_c018
MMFF Geometry
C 1.756304 1.696061 -0.101674
C 2.039215 -0.980370 0.252057
N 3.002322 1.208824 0.114730
C 0.594955 0.921584 -0.159903
C 0.754038 -0.462562 0.022338
C 3.139583 -0.125221 0.293463
C -0.376276 -1.297864 -0.032344
C -1.659004 -0.777265 -0.260638
C -1.814861 0.602378 -0.432102
C -0.693495 1.447796 -0.389861
C -3.170534 1.151380 -0.678764
C -4.395253 0.218115 -0.628888
C -4.047555 -1.249085 -0.507175
C -2.798778 -1.691327 -0.311613
O -0.799729 2.806916 -0.574861
O -3.294419 2.347046 -0.970472
O -5.032553 0.424253 -1.910373
C -5.380251 0.689125 0.446634
C -4.890670 0.390956 1.844692
C -5.906439 -0.121298 2.829819
O -3.723873 0.598750 2.176535
O -5.092597 -2.160944 -0.417400
C -5.720337 -2.388698 -1.679446
C 4.522911 -0.622713 0.548108
C 4.652733 -1.900435 1.332561
C 5.577730 0.093878 0.100196
C 7.050856 -0.230877 0.225433
C 7.701319 -0.192343 -1.171462
C 9.158159 -0.635389 -1.174328
C 7.701065 0.756345 1.196890
H 1.714715 2.775173 -0.232682
H 2.170524 -2.049316 0.383241
H -0.250625 -2.370945 0.108200
H -2.618024 -2.751691 -0.160497
H -1.742566 3.025537 -0.748545
H -5.038677 1.391103 -2.058578
H -6.353104 0.203891 0.296703
H -5.570491 1.767136 0.379253
H -5.425991 -0.299029 3.795893
H -6.698253 0.620713 2.957789
H -6.325278 -1.063419 2.467495
H -6.473809 -1.620274 -1.873812
H -4.993530 -2.437631 -2.497915
H -6.235505 -3.352496 -1.625659
H 4.466609 -2.767334 0.690130
H 5.639775 -2.023387 1.785642
H 3.941906 -1.922131 2.166689
H 5.377671 1.025869 -0.433265
H 7.190778 -1.240371 0.624429
H 7.143334 -0.852284 -1.848564
H 7.637152 0.818060 -1.595178
H 9.267829 -1.629777 -0.730382
H 9.533839 -0.680413 -2.201717
H 9.792133 0.064152 -0.622011
H 8.743035 0.487595 1.396615
H 7.178299 0.759903 2.160293
H 7.684889 1.778418 0.801934
SCF Energy (B3LYP/6-31G**)= -1361.70469937
Number of imaginary frequencies = 0

1b_c019
MMFF Geometry
C 1.604962 2.437942 0.210393
C 2.269798 -0.025564 -0.720302
N 2.921069 2.147705 0.066430
C 0.555098 1.562635 -0.086379
C 0.911590 0.291896 -0.570231
C 3.240766 0.916893 -0.386683
C -0.099440 -0.629876 -0.896365
C -1.457945 -0.311196 -0.742512
C -1.807799 0.950555 -0.252546
C -0.808867 1.886255 0.066994
C -3.240700 1.288359 -0.093871
C -4.308009 0.211535 -0.350837
C -3.782912 -1.087260 -0.931183
C -2.468061 -1.308893 -1.106863
O -1.113182 3.145048 0.531586
O -3.558234 2.445502 0.205952
O -5.168763 0.806180 -1.351670
C -5.163204 0.006297 0.906100
C -4.437994 -0.754077 1.992226
C -5.280575 -1.618230 2.891981
O -3.226640 -0.635624 2.173796
O -4.786801 -1.996285 -1.222370
C -4.438641 -3.199670 -1.895905
C 4.690580 0.636939 -0.533022
C 5.484737 1.781916 -1.102914
C 5.198450 -0.547714 -0.129592
C 6.639130 -1.007119 -0.189496
C 7.323366 -0.930738 1.189627
C 6.733885 -1.829600 2.269813
C 6.713996 -2.411138 -0.797594
H 1.406280 3.439028 0.587165
H 2.566983 -0.995395 -1.107066
H 0.182895 -1.612254 -1.273290
H -2.095666 -2.243875 -1.509637
H -2.091783 3.240555 0.554357
H -5.350798 1.717531 -1.051805
H -6.078570 -0.539715 0.644148
H -5.493205 0.962789 1.329432
H -4.643737 -2.111799 3.631302
H -6.014519 -0.998052 3.412208
H -5.785182 -2.383640 2.297147
H -3.961812 -2.987736 -2.858366
H -3.796925 -3.825925 -1.268062
H -5.361151 -3.754276 -2.091481
H 5.509989 2.623439 -0.401937
H 6.521281 1.522507 -1.326854
H 5.037831 2.126582 -2.042480
H 4.523036 -1.273740 0.323936
H 7.212558 -0.359433 -0.860342
H 8.384488 -1.184582 1.071813
H 7.294522 0.106678 1.547173
H 6.805357 -2.886268 1.996742
H 7.285072 -1.693713 3.206289
H 5.684908 -1.590365 2.466999
H 7.749798 -2.765814 -0.827586
H 6.334385 -2.406901 -1.825829
H 6.122190 -3.139014 -0.232373
SCF Energy (B3LYP/6-31G**)= -1361.70906499
Number of imaginary frequencies = 0

1b_c020
MMFF Geometry
C 1.626251 2.377814 0.404359
C 2.297867 0.001213 -0.726331
N 2.942457 2.112381 0.219168
C 0.579308 1.518833 0.054474

C 0.939512 0.293185 -0.532972
C 3.265725 0.923764 -0.333403
C -0.067804 -0.609926 -0.916574
C -1.425269 -0.315299 -0.719994
C -1.780705 0.900559 -0.125970
C -0.785020 1.817046 0.252471
C -3.216480 1.213421 0.076201
C -4.285316 0.156204 -0.260421
C -3.740894 -1.069498 -0.960086
C -2.432119 -1.288347 -1.140247
O -1.092542 3.032581 0.818804
O -3.539802 2.341214 0.468154
O -5.166580 0.843401 -1.178002
C -5.108258 -0.184908 0.986637
C -4.338113 -1.038266 1.967299
C -5.082377 -2.173014 2.617714
O -3.167231 -0.791103 2.254832
O -4.630043 -2.092198 -1.268509
C -5.442577 -1.778167 -2.399818
C 4.715576 0.669833 -0.521337
C 5.492129 1.863704 -1.009195
C 5.238882 -0.538340 -0.220395
C 6.682201 -0.978283 -0.336337
C 7.385338 -1.006018 1.035097
C 6.818574 -1.993675 2.047795
C 6.759717 -2.328505 -1.055742
H 1.425008 3.344143 0.861842
H 2.597135 -0.932696 -1.191972
H 0.215026 -1.558033 -1.372823
H -2.095848 -2.220057 -1.586068
H -2.071094 3.113301 0.872319
H -5.320328 1.730087 -0.794668
H -6.024157 -0.713720 0.693620
H -5.440207 0.717675 1.513891
H -4.415121 -2.709717 3.297740
H -5.927031 -1.777607 3.187048
H -5.434488 -2.867353 1.850769
H -6.308900 -1.185322 -2.093242
H -4.875832 -1.263407 -3.183629
H -5.814782 -2.720103 -2.813845
H 5.520887 2.646988 -0.243815
H 6.527326 1.632414 -1.267533
H 5.028814 2.277930 -1.912003
H 4.575968 -1.304421 0.182992
H 7.240820 -0.273857 -0.960896
H 8.446695 -1.239871 0.883006
H 7.353195 -0.000785 1.474843
H 6.894680 -3.024450 1.690119
H 7.381926 -1.927972 2.984586
H 5.770615 -1.780602 2.277686
H 7.797808 -2.670156 -1.128000
H 6.365521 -2.245635 -2.075120
H 6.181872 -3.104584 -0.542606
SCF Energy (B3LYP/6-31G**)= -1361.70331490
Number of imaginary frequencies = 0

1b_c021
MMFF Geometry
C -1.859918 -1.531429 -0.373390
C -2.017211 1.169577 -0.156194
N -3.087789 -0.970995 -0.251241
C -0.657097 -0.821211 -0.397839
C -0.750692 0.575778 -0.284429
C -3.163124 0.375471 -0.142762
C 0.425587 1.347255 -0.303124
C 1.690783 0.751900 -0.429739
C 1.778441 -0.639142 -0.536422
C 0.611891 -1.422716 -0.527763
C 3.112093 -1.267387 -0.674542
C 4.375787 -0.396724 -0.575846
C 4.123156 1.097334 -0.517000
C 2.880500 1.607782 -0.452995
O 0.654799 -2.792530 -0.648858
O 3.186782 -2.479868 -0.906890
O 5.077681 -0.659822 -1.814497
C 5.275123 -0.899877 0.560601
C 4.740971 -0.551557 1.930709
C 5.758780 -0.322808 3.016081
O 3.534436 -0.509104 2.169083
O 5.289462 1.844723 -0.509788
C 5.188854 3.261159 -0.590619
C -4.532052 0.956877 -0.024456
C -4.714280 2.375964 -0.491504
C -5.539341 0.193274 0.454358
C -6.981865 0.590915 0.674351
C -7.897623 0.072947 -0.451497
C -7.980142 -1.442685 -0.587299
C -7.448259 0.130009 2.059078
H -1.869475 -2.616166 -0.454903
H -2.095637 2.246808 -0.054397
H 0.348492 2.430360 -0.214959
H 2.704499 2.675956 -0.397491
H 1.592221 -3.065690 -0.766688
H 5.071518 -1.629013 -1.933857
H 6.279415 -0.470186 0.453827
H 5.406451 -1.987984 0.518549
H 5.250271 -0.080404 3.953326
H 6.352433 -1.229155 3.157651
H 6.405354 0.514319 2.741368
H 4.664044 3.566684 -1.501575
H 4.700700 3.668586 0.300388
H 6.203416 3.668224 -0.633327
H -4.175949 2.551643 -1.429982
H -5.758555 2.623372 -0.700623
H -4.346349 3.079972 0.262058
H -5.313465 -0.836891 0.737313
H -7.067777 1.683537 0.680009
H -8.911332 0.460849 -0.289938
H -7.559432 0.489112 -1.409238
H -8.387244 -1.905584 0.316157
H -8.642918 -1.704764 -1.418721
H -7.000729 -1.883834 -0.794144
H -8.497326 0.398246 2.223693
H -6.855178 0.610496 2.845697
H -7.350372 -0.952975 2.189556
SCF Energy (B3LYP/6-31G**)= -1361.70953709
Number of imaginary frequencies = 0

1b_c022
MMFF Geometry
C -1.871406 -1.488219 -0.415737
C -2.048791 1.200105 -0.084924
N -3.104520 -0.940159 -0.290086
C -0.672634 -0.771301 -0.390142
C -0.776884 0.619341 -0.218358
C -3.189872 0.399975 -0.124524
C 0.394123 1.397800 -0.184937
C 1.663220 0.814521 -0.318202
C 1.763487 -0.571217 -0.481784
C 0.601958 -1.360475 -0.524986
C 3.105150 -1.186427 -0.628614
C 4.367130 -0.314482 -0.485249
C 4.083795 1.168092 -0.384662
C 2.847153 1.671313 -0.280993
O 0.654857 -2.723511 -0.703995
O 3.191491 -2.386931 -0.913910
O 5.086587 -0.552091 -1.716822
C 5.245528 -0.833003 0.658595
C 4.668610 -0.511009 2.017487
C 5.631797 -0.046796 3.076457
O 3.471910 -0.662943 2.261466
O 5.162722 2.027481 -0.214240
C 5.893955 2.222907 -1.424929
C -4.564151 0.968223 -0.003388
C -4.746883 2.406368 -0.407855
C -5.574897 0.178345 0.422652
C -7.023208 0.558139 0.636046
C -7.916847 0.088029 -0.527931
C -7.988187 -1.420124 -0.734761
C -7.510162 0.031500 1.989919
H -1.873153 -2.568515 -0.543503
H -2.135085 2.271517 0.062255

H 0.311229 2.475842 -0.050779
H 2.707960 2.739305 -0.139919
H 1.596084 -2.988343 -0.808192
H 5.056540 -1.518166 -1.867164
H 6.249375 -0.396054 0.582873
H 5.387367 -1.919048 0.601748
H 5.090128 0.154213 4.004846
H 6.375944 -0.825434 3.260565
H 6.120443 0.874137 2.748733
H 6.621065 1.418031 -1.564433
H 5.233959 2.307049 -2.295479
H 6.450552 3.160112 -1.329828
H -4.194703 2.627509 -1.328504
H -5.789047 2.658079 -0.622277
H -4.394969 3.076854 0.383008
H -5.347763 -0.862496 0.662148
H -7.115593 1.648881 0.690553
H -8.935385 0.462915 -0.366019
H -7.565031 0.549647 -1.459579
H -8.407736 -1.926269 0.139331
H -8.635314 -1.646928 -1.588580
H -7.002859 -1.846286 -0.944915
H -8.563393 0.286578 2.148760
H -6.933210 0.478165 2.807843
H -7.408195 -1.055844 2.072052
SCF Energy (B3LYP/6-31G**)= -1361.70385878
Number of imaginary frequencies = 0

1b_c023
MMFF Geometry
C -1.557126 -2.462909 -0.177691
C -2.319472 0.132504 0.070112
N -2.875018 -2.202998 0.002961
C -0.552330 -1.492223 -0.249004
C -0.959020 -0.153489 -0.118288
C -3.243464 -0.909336 0.117709
C 0.005804 0.867622 -0.178411
C 1.367412 0.579748 -0.367256
C 1.770354 -0.752783 -0.493224
C 0.814743 -1.783795 -0.437822
C 3.207762 -1.062240 -0.690619
C 4.233619 0.092758 -0.723663
C 3.639932 1.486277 -0.593252
C 2.321231 1.689348 -0.431253
O 1.164499 -3.108265 -0.568945
O 3.531988 -2.244243 -0.845815
O 4.870831 0.022754 -2.018612
C 5.339728 -0.193390 0.301709
C 4.871458 -0.140918 1.739245
C 5.948232 -0.185829 2.791824
O 3.682488 -0.094938 2.052166
O 4.592368 2.488128 -0.666700
C 4.175459 3.841264 -0.527115
C -4.691773 -0.668196 0.321967
C -5.348578 -1.624127 1.281117
C -5.321751 0.324378 -0.342320
C -6.790736 0.684156 -0.284790
C -7.002738 2.094897 0.295014
C -6.570066 2.217047 1.750865
C -7.384538 0.602078 -1.694924
H -1.317693 -3.520494 -0.266793
H -2.653250 1.158077 0.193396
H -0.315623 1.903318 -0.072942
H 1.904616 2.685638 -0.333546
H 2.140061 -3.161262 -0.693451
H 4.257395 0.410410 -2.665172
H 6.159555 0.526034 0.180365
H 5.775786 -1.185752 0.130066
H 5.494773 -0.138654 3.785793
H 6.509025 -1.119349 2.702032
H 6.616540 0.670039 2.669333
H 3.491988 4.124786 -1.333815
H 3.719275 4.010758 0.453494
H 5.063700 4.475749 -0.599695
H -4.712973 -1.792000 2.158539
H -6.300674 -1.251973 1.667113
H -5.532590 -2.589234 0.797410
H -4.746643 0.939083 -1.035270
H -7.347502 -0.026645 0.333651
H -6.461611 2.839384 -0.302681
H -8.066932 2.354392 0.231082
H -5.491538 2.071715 1.864324
H -6.812468 3.214476 2.131489
H -7.086411 1.481789 2.376169
H -8.457919 0.820010 -1.675853
H -7.256839 -0.401354 -2.116440
H -6.909165 1.317508 -2.375655
SCF Energy (B3LYP/6-31G**)= -1361.70828689
Number of imaginary frequencies = 0

1b_c024
MMFF Geometry
C -1.640951 -2.320234 -0.279319
C -2.270301 0.297437 -0.640036
N -2.952788 -1.986220 -0.346699
C -0.578519 -1.416334 -0.384466
C -0.916785 -0.065710 -0.574206
C -3.254315 -0.681129 -0.515348
C 0.107844 0.889450 -0.696672
C 1.462398 0.524595 -0.629191
C 1.796641 -0.818344 -0.432594
C 0.781213 -1.784965 -0.315112
C 3.225999 -1.208467 -0.357803
C 4.315461 -0.118401 -0.468694
C 3.795694 1.291235 -0.701312
C 2.482015 1.566135 -0.772976
O 1.064085 -3.119821 -0.136534
O 3.495485 -2.407458 -0.230765
O 5.115623 -0.470589 -1.619175
C 5.254073 -0.226636 0.741265
C 4.600879 0.131428 2.057959
C 5.521391 0.263356 3.243140
O 3.385869 0.280711 2.180996
O 4.808667 2.225407 -0.833348
C 4.460082 3.591454 -1.024860
C -4.699610 -0.358289 -0.583458
C -5.515716 -1.335184 -1.386328
C -5.193141 0.716551 0.067972
C -6.637270 1.162203 0.147946
C -7.114486 1.267934 1.609452
C -7.129431 -0.073099 2.332982
C -6.784268 2.515681 -0.552006
H -1.456579 -3.382440 -0.132788
H -2.553812 1.332920 -0.800451
H -0.161101 1.935055 -0.843249
H 2.117548 2.574574 -0.933979
H 2.042450 -3.228740 -0.105303
H 4.619883 -0.201583 -2.410696
H 6.125027 0.426083 0.600525
H 5.647999 -1.246911 0.832139
H 4.942216 0.526065 4.132727
H 6.029753 -0.687721 3.419303
H 6.252243 1.053324 3.053523
H 3.909272 3.726649 -1.961136
H 3.887963 3.972233 -0.172739
H 5.387050 4.168411 -1.093143
H -5.708023 -2.243957 -0.806282
H -6.477814 -0.925134 -1.703042
H -4.990820 -1.616321 -2.306918
H -4.510162 1.338092 0.647819
H -7.297872 0.454743 -0.362674
H -6.483276 1.969925 2.168947
H -8.131962 1.678295 1.627095
H -6.121553 -0.486322 2.436393
H -7.543769 0.048275 3.338997
H -7.748326 -0.800238 1.797713
H -7.827786 2.848466 -0.536327
H -6.472309 2.450933 -1.600497
H -6.177940 3.288888 -0.066477
SCF Energy (B3LYP/6-31G**)= -1361.70835588
Number of imaginary frequencies = 0

1b_c025
MMFF Geometry
C -1.834944 -1.623365 0.178942
C -2.034042 1.083655 0.164424
N -3.059562 -1.072247 0.361351
C -0.655812 -0.900771 -0.019637
C -0.771275 0.499190 -0.027284
C -3.155238 0.277277 0.355746
C 0.379423 1.282756 -0.229881
C 1.641282 0.696904 -0.418675
C 1.751748 -0.696507 -0.401894
C 0.609755 -1.492967 -0.211062
C 3.081639 -1.315116 -0.604593
C 4.328082 -0.420269 -0.706179
C 4.046422 1.068548 -0.765828
C 2.803078 1.564207 -0.634394
O 0.673601 -2.867307 -0.208161
O 3.163486 -2.543002 -0.728170
O 4.920668 -0.791083 -1.974049
C 5.338592 -0.798075 0.384491
C 4.923408 -0.326957 1.759100
C 6.030238 0.019764 2.718800
O 3.742926 -0.278872 2.103362
O 5.190316 1.830443 -0.938821
C 5.048812 3.230502 -1.145628
C -4.516449 0.847813 0.578413
C -4.582208 2.222354 1.187581
C -5.601577 0.111562 0.249959
C -7.063316 0.480164 0.373535
C -7.748343 0.582865 -1.005215
C -7.838380 -0.712696 -1.802499
C -7.771990 -0.508054 1.302980
H -1.826632 -2.711010 0.197902
H -2.132231 2.163959 0.148988
H 0.285053 2.368057 -0.237109
H 2.607488 2.629996 -0.664283
H 1.602555 -3.136453 -0.386682
H 4.926009 -1.767249 -2.000399
H 6.318625 -0.366013 0.144695
H 5.491064 -1.883118 0.433763
H 5.603692 0.344114 3.672019
H 6.655022 -0.860194 2.889821
H 6.629682 0.835623 2.307281
H 4.435530 3.437515 -2.028602
H 4.634775 3.715860 -0.256142
H 6.045142 3.646537 -1.321985
H -4.376375 2.987952 0.432233
H -5.552691 2.448396 1.635025
H -3.852847 2.325277 1.999401
H -5.432907 -0.884040 -0.165261
H -7.167739 1.468511 0.831551
H -8.766303 0.967711 -0.862864
H -7.221091 1.329594 -1.612955
H -8.416181 -1.473524 -1.270071
H -8.342126 -0.525483 -2.756736
H -6.848732 -1.119830 -2.027922
H -8.839576 -0.274803 1.376278
H -7.350809 -0.456770 2.313593
H -7.672851 -1.543016 0.958504
SCF Energy (B3LYP/6-31G**)= -1361.70929948
Number of imaginary frequencies = 0

1b_c026
MMFF Geometry
C 1.849256 1.590791 0.222328
C 2.067833 -1.114242 0.163084
N 3.080746 1.046172 0.375865
C 0.672230 0.862685 0.031764
C 0.798030 -0.536441 0.000647
C 3.186098 -0.302449 0.348377
C -0.349421 -1.325934 -0.195289
C -1.616727 -0.745690 -0.355887
C -1.739542 0.647267 -0.314806
C -0.600634 1.448883 -0.129688
C -3.078852 1.260155 -0.490039
C -4.324428 0.360097 -0.598866
C -4.011002 -1.116337 -0.700313
C -2.775412 -1.613337 -0.560402
O -0.674078 2.822450 -0.104536
O -3.171897 2.489072 -0.595694
O -4.937253 0.775912 -1.840796
C -5.315250 0.683929 0.524622
C -4.853897 0.162878 1.865823
C -5.895725 -0.469905 2.748345
O -3.688040 0.289026 2.239427
O -5.077107 -2.005711 -0.764302
C -5.690222 -2.021727 -2.053691
C 4.554921 -0.866046 0.540289
C 4.640402 -2.249875 1.125618
C 5.629299 -0.116257 0.207106
C 7.095477 -0.475640 0.301816
C 7.759399 -0.550963 -1.088974
C 7.827190 0.757864 -1.866550
C 7.811419 0.502929 1.235895
H 1.833444 2.677901 0.258626
H 2.173256 -2.193506 0.129845
H -0.249593 -2.410584 -0.220111
H -2.620375 -2.688374 -0.572158
H -1.609092 3.087822 -0.253635
H -4.919846 1.753916 -1.838485
H -6.296117 0.251056 0.290322
H -5.480683 1.763766 0.621701
H -5.434211 -0.805424 3.681163
H -6.672866 0.262549 2.979622
H -6.329609 -1.334825 2.240452
H -6.423529 -1.214634 -2.136364
H -4.952166 -1.961874 -2.861295
H -6.227233 -2.969651 -2.154969
H 4.427518 -3.004515 0.361250
H 5.619701 -2.476173 1.553269
H 3.925314 -2.371343 1.947510
H 5.446771 0.884669 -0.188958
H 7.214442 -1.470363 0.742126
H 8.782315 -0.930197 -0.968821
H 7.228187 -1.291890 -1.700354
H 8.407651 1.514485 -1.331029
H 8.317180 0.589849 -2.831464
H 6.831101 1.160931 -2.069830
H 8.881719 0.276683 1.288695
H 7.406622 0.432236 2.252017
H 7.699230 1.542495 0.909775
SCF Energy (B3LYP/6-31G**)= -1361.70360825
Number of imaginary frequencies = 0

1b_c027
MMFF Geometry
C -1.833545 -1.578417 -0.499321
C -2.076259 1.069062 0.046998
N -3.077715 -1.078044 -0.303355
C -0.654639 -0.830661 -0.443882
C -0.792557 0.537673 -0.160609
C -3.195707 0.241605 -0.030060
C 0.358113 1.343523 -0.091778
C 1.641139 0.810940 -0.299473
C 1.775352 -0.552424 -0.576369
C 0.632530 -1.369034 -0.651962
C 3.128278 -1.120681 -0.794110
C 4.363715 -0.199538 -0.681589
C 4.054684 1.261298 -0.395394
C 2.797969 1.706014 -0.223946
O 0.715688 -2.713191 -0.934705
O 3.216095 -2.318628 -1.083105
O 5.017246 -0.250350 -1.969274
C 5.355978 -0.815879 0.314560
C 4.859344 -0.833233 1.743413
C 5.868537 -1.217119 2.793916
O 3.694320 -0.580736 2.047766
O 5.189080 2.052818 -0.341920
C 5.045073 3.437616 -0.048963
C -4.581883 0.758153 0.165919
C -4.813596 2.214874 -0.132017
C -5.554192 -0.091471 0.565562

C -7.010196 0.211370 0.840869
C -7.936419 -0.512291 -0.153941
C -7.765990 -0.034723 -1.590693
C -7.348485 -0.212274 2.274308
H -1.808650 -2.645038 -0.712024
H -2.188644 2.122434 0.280873
H 0.246270 2.404156 0.131013
H 2.584678 2.748332 -0.014779
H 1.664788 -2.947664 -1.053975
H 4.514782 0.322581 -2.572504
H 6.305813 -0.266825 0.287091
H 5.592035 -1.849733 0.032023
H 5.400457 -1.191993 3.781891
H 6.232094 -2.229091 2.599619
H 6.699598 -0.507679 2.780478
H 4.458697 3.941993 -0.823590
H 4.599447 3.583815 0.940212
H 6.043612 3.884397 -0.038026
H -4.292970 2.514029 -1.049050
H -5.866212 2.454760 -0.300731
H -4.456260 2.838406 0.694087
H -5.287189 -1.137737 0.730964
H -7.209438 1.285501 0.776496
H -7.767496 -1.595923 -0.114795
H -8.980475 -0.346706 0.140138
H -6.769415 -0.267935 -1.977450
H -8.497442 -0.528036 -2.238867
H -7.922651 1.046033 -1.665615
H -8.388555 0.033457 2.514941
H -6.708978 0.305929 2.997760
H -7.216786 -1.290735 2.418578
SCF Energy (B3LYP/6-31G**)= -1361.70938738
Number of imaginary frequencies = 0

1b_c028
MMFF Geometry
C -1.798684 -1.738683 -0.260030
C -2.103559 0.872542 0.414933
N -3.044721 -1.297832 0.039634
C -0.647973 -0.946186 -0.251443
C -0.818323 0.403396 0.096883
C -3.192957 0.003320 0.378589
C 0.301798 1.253720 0.116658
C 1.586624 0.783148 -0.200614
C 1.753990 -0.562385 -0.539728
C 0.641285 -1.422418 -0.569150
C 3.109311 -1.065426 -0.872558
C 4.312847 -0.098558 -0.808506
C 3.967924 1.336921 -0.445414
C 2.710005 1.722428 -0.170604
O 0.756372 -2.750243 -0.911576
O 3.223133 -2.247181 -1.214324
O 4.876790 -0.075752 -2.138652
C 5.395151 -0.708812 0.093124
C 5.000290 -0.798343 1.550749
C 6.094250 -1.178376 2.514204
O 3.850492 -0.605007 1.943511
O 5.071458 2.172620 -0.439866
C 4.894195 3.539216 -0.086006
C -4.575383 0.447747 0.720040
C -4.702374 1.620826 1.653859
C -5.631493 -0.223117 0.209123
C -7.103814 0.064890 0.399819
C -7.831178 0.198892 -0.952514
C -7.350199 1.387477 -1.775987
C -7.734222 -1.058663 1.226212
H -1.747724 -2.794112 -0.518916
H -2.244216 1.916271 0.675369
H 0.164669 2.299907 0.388268
H 2.471379 2.746810 0.092777
H 1.703184 -2.940557 -1.104767
H 4.311617 0.497120 -2.683646
H 6.318726 -0.120239 0.022427
H 5.650945 -1.721122 -0.244818
H 5.695545 -1.208333 3.532027
H 6.481984 -2.166870 2.256223
H 6.894486 -0.435428 2.471817
H 4.235443 4.045218 -0.798951
H 4.513864 3.632310 0.936297
H 5.872731 4.026646 -0.127129
H -4.540120 2.560473 1.115667
H -5.680549 1.675692 2.138270
H -3.973670 1.552831 2.469858
H -5.433288 -1.079629 -0.439112
H -7.254818 1.002045 0.944625
H -7.713040 -0.717987 -1.543820
H -8.906793 0.319524 -0.772290
H -6.303042 1.274672 -2.072671
H -7.946865 1.476714 -2.689496
H -7.451756 2.320839 -1.213143
H -8.796453 -0.859180 1.404490
H -7.245233 -1.149183 2.202696
H -7.654989 -2.026429 0.717920
SCF Energy (B3LYP/6-31G**)= -1361.70929660
Number of imaginary frequencies = 0

1b_c029
MMFF Geometry
C 1.608213 -2.357807 0.165304
C 2.561412 0.149692 -0.268859
N 2.951878 -2.188612 0.116956
C 0.667292 -1.335112 0.007537
C 1.172843 -0.042050 -0.219672
C 3.414802 -0.937045 -0.089126
C 0.277141 1.027091 -0.399183
C -1.109610 0.830077 -0.340730
C -1.609566 -0.452437 -0.103821
C -0.728114 -1.533859 0.056120
C -3.078476 -0.648581 -0.060777
C -3.998967 0.579606 0.160722
C -3.320899 1.857731 -0.315348
C -2.008915 1.956226 -0.567056
O -1.182506 -2.815556 0.260851
O -3.506002 -1.801967 -0.163008
O -4.218623 0.745227 1.563834
C -5.321841 0.354615 -0.601501
C -6.138576 -0.785156 -0.025041
C -6.929880 -1.621158 -0.993707
O -6.193586 -0.979373 1.191363
O -4.122933 2.965379 -0.567955
C -4.536930 3.598122 0.645397
C 4.889692 -0.796119 -0.138096
C 5.591312 -1.890239 -0.896824
C 5.500623 0.233881 0.485787
C 6.986448 0.508974 0.571862
C 7.356175 1.829563 -0.128606
C 7.106353 1.802583 -1.631433
C 7.403911 0.562093 2.045197
H 1.292620 -3.383988 0.341600
H 2.973257 1.135909 -0.459715
H 0.670260 2.026259 -0.584429
H -1.591100 2.892942 -0.924328
H -2.162782 -2.818039 0.176642
H -4.819559 0.020277 1.846611
H -5.116557 0.144781 -1.658322
H -5.976731 1.232279 -0.551740
H -7.659632 -0.991515 -1.508341
H -6.253352 -2.084146 -1.716307
H -7.458545 -2.410642 -0.452528
H -5.335303 3.027063 1.128122
H -3.695885 3.743984 1.332216
H -4.939067 4.582214 0.386896
H 5.053764 -2.127229 -1.822523
H 6.604337 -1.615085 -1.200211
H 5.654088 -2.800048 -0.290547
H 4.889543 0.954203 1.030526
H 7.564706 -0.296753 0.109142
H 6.798038 2.665491 0.311877
H 8.420070 2.040052 0.037990
H 6.041478 1.700259 -1.861190
H 7.455987 2.734970 -2.086303
H 7.643773 0.973843 -2.103230

H 8.483967 0.721163 2.135646
H 7.162728 -0.377329 2.555271
H 6.899313 1.375557 2.578929
SCF Energy (B3LYP/6-31G**)= -1361.70803464
Number of imaginary frequencies = 0

1b_c030
MMFF Geometry
C -1.687070 -2.105295 -0.523686
C -2.510796 0.479399 -0.363716
N -3.012579 -1.852174 -0.647330
C -0.703086 -1.132802 -0.318700
C -1.141587 0.201567 -0.238997
C -3.411937 -0.565318 -0.558552
C -0.200214 1.225802 -0.033169
C 1.167638 0.941961 0.084208
C 1.601936 -0.382690 -0.002816
C 0.672083 -1.418166 -0.190363
C 3.049506 -0.669608 0.139442
C 4.071138 0.480373 -0.056939
C 3.433052 1.829899 0.245741
C 2.110967 2.026192 0.334639
O 1.058077 -2.736503 -0.254627
O 3.382777 -1.835536 0.369838
O 4.458850 0.523653 -1.432525
C 5.280753 0.235732 0.869657
C 6.077392 -0.992046 0.474214
C 6.694394 -1.797217 1.585242
O 6.256552 -1.282151 -0.710744
O 4.275623 2.903663 0.512359
C 4.866048 3.414343 -0.685384
C -4.868844 -0.332687 -0.703407
C -5.523738 -1.156063 -1.779682
C -5.509371 0.533217 0.110942
C -6.988645 0.852395 0.135116
C -7.597528 0.597788 1.527729
C -7.556786 -0.868640 1.940063
C -7.193182 2.314849 -0.268390
H -1.423851 -3.158323 -0.598305
H -2.869218 1.503350 -0.322960
H -0.542210 2.258086 0.034848
H 1.720934 3.011211 0.573930
H 2.019885 -2.791796 -0.054839
H 5.036873 -0.255660 -1.591253
H 4.943347 0.120677 1.906944
H 5.995689 1.066047 0.836482
H 7.225568 -2.656329 1.166400
H 7.402559 -1.174932 2.137602
H 5.910065 -2.161756 2.253176
H 5.673001 2.760907 -1.029450
H 4.119807 3.556223 -1.474907
H 5.302604 4.389833 -0.451266
H -5.685496 -2.182511 -1.433938
H -6.486836 -0.749716 -2.098137
H -4.898422 -1.188098 -2.679682
H -4.936468 1.053973 0.878668
H -7.538003 0.233852 -0.581294
H -7.081097 1.198101 2.287450
H -8.644871 0.924954 1.527961
H -6.529150 -1.226209 2.055713
H -8.065325 -1.002372 2.900259
H -8.060918 -1.497977 1.199752
H -8.259367 2.565300 -0.289432
H -6.787531 2.506260 -1.268217
H -6.701287 2.999220 0.432250
SCF Energy (B3LYP/6-31G**)= -1361.70814659
Number of imaginary frequencies = 0

1b_c031
MMFF Geometry
C -1.449156 -2.483259 -0.247414
C -2.245391 0.084575 0.140930
N -2.768484 -2.252199 -0.040000
C -0.459039 -1.495616 -0.279378
C -0.883151 -0.171446 -0.076068
C -3.154115 -0.971650 0.144902
C 0.066466 0.865433 -0.093973
C 1.429815 0.607587 -0.311522
C 1.850103 -0.710721 -0.509696
C 0.909820 -1.757112 -0.497136
C 3.289438 -0.988585 -0.737675
C 4.298444 0.181527 -0.722336
C 3.686634 1.557718 -0.514604
C 2.367093 1.732887 -0.328643
O 1.276739 -3.067898 -0.699509
O 3.628566 -2.156253 -0.956498
O 4.922028 0.187013 -2.025772
C 5.419908 -0.140273 0.275417
C 4.967083 -0.168060 1.718579
C 6.056113 -0.250801 2.756149
O 3.781165 -0.155538 2.045876
O 4.623943 2.575882 -0.546663
C 4.189553 3.913873 -0.333687
C -4.603707 -0.763556 0.375311
C -5.239082 -1.781789 1.283651
C -5.255802 0.255290 -0.224770
C -6.732625 0.580641 -0.135962
C -6.916002 1.896562 0.641383
C -8.374469 2.253009 0.894357
C -7.317475 0.652680 -1.549946
H -1.195877 -3.531296 -0.393075
H -2.591908 1.097609 0.319854
H -0.268335 1.889581 0.067689
H 1.937568 2.716675 -0.175744
H 2.251500 -3.100165 -0.836737
H 4.295963 0.598078 -2.645199
H 6.228080 0.596359 0.182456
H 5.867988 -1.116083 0.048678
H 5.613201 -0.261037 3.755924
H 6.628981 -1.170201 2.612850
H 6.710862 0.619909 2.670612
H 3.493158 4.228105 -1.117634
H 3.742028 4.026424 0.659025
H 5.067877 4.564203 -0.383002
H -4.586619 -2.004010 2.136285
H -6.183623 -1.436149 1.711465
H -5.430737 -2.714231 0.742353
H -4.697368 0.921326 -0.883015
H -7.271400 -0.212819 0.391530
H -6.412259 1.815930 1.613520
H -6.434717 2.725748 0.107181
H -8.897237 1.434912 1.399707
H -8.437920 3.140341 1.532557
H -8.900492 2.479126 -0.037603
H -8.405617 0.767569 -1.522422
H -7.103216 -0.265130 -2.109563
H -6.902761 1.495482 -2.114373
SCF Energy (B3LYP/6-31G**)= -1361.70712954
Number of imaginary frequencies = 0

1b_c032
MMFF Geometry
C -1.506949 -2.449846 -0.008101
C -2.222065 0.097596 -0.622499
N -2.829155 -2.166322 -0.100022
C -0.474740 -1.527326 -0.208977
C -0.857258 -0.213728 -0.529507
C -3.173460 -0.894717 -0.395218
C 0.135456 0.756835 -0.752523
C 1.501342 0.444000 -0.657753
C 1.879604 -0.861403 -0.331118
C 0.896451 -1.843570 -0.112004
C 3.321095 -1.196254 -0.227044
C 4.374256 -0.088294 -0.452312
C 3.808293 1.273935 -0.820231
C 2.486167 1.498199 -0.910502
O 1.223049 -3.144373 0.196203
O 3.629745 -2.367474 0.016512
O 5.181852 -0.527181 -1.567242
C 5.319637 -0.045795 0.756471
C 4.659253 0.420130 2.035285
C 5.578668 0.698407 3.195732

O 3.440428 0.541971 2.150572
O 4.789998 2.222305 -1.050602
C 4.396668 3.550742 -1.374361
C -4.628814 -0.626872 -0.486679
C -5.413827 -1.703608 -1.186444
C -5.155667 0.491787 0.055937
C -6.614456 0.898011 0.096596
C -7.040718 1.119951 1.560831
C -8.527528 1.405981 1.722623
C -6.808217 2.149954 -0.761808
H -1.287746 -3.485825 0.241796
H -2.539455 1.102406 -0.883169
H -0.167733 1.773582 -1.000327
H 2.088665 2.473496 -1.168370
H 2.204524 -3.218301 0.231918
H 4.675189 -0.354079 -2.378386
H 6.168504 0.617255 0.546236
H 5.746752 -1.038883 0.945540
H 4.994034 1.029470 4.058534
H 6.118155 -0.213745 3.462082
H 6.282921 1.488612 2.924156
H 3.838855 3.574642 -2.315932
H 3.815143 3.995731 -0.560583
H 5.304207 4.147426 -1.505341
H -5.578334 -2.555781 -0.518625
H -6.387722 -1.356487 -1.540389
H -4.879962 -2.058638 -2.075843
H -4.491355 1.190787 0.565122
H -7.248070 0.106963 -0.316090
H -6.800652 0.223385 2.147220
H -6.470250 1.946272 2.003898
H -9.132054 0.616425 1.265375
H -8.785600 1.456900 2.785399
H -8.804426 2.362469 1.270075
H -7.867388 2.412417 -0.846118
H -6.433379 1.989161 -1.779208
H -6.278459 3.011899 -0.340735
SCF Energy (B3LYP/6-31G**)= -1361.70722056
Number of imaginary frequencies = 0

1b_c033
MMFF Geometry
C -1.417948 -2.298279 -0.529011
C -2.342913 0.183358 0.083049
N -2.749274 -2.148679 -0.324411
C -0.476988 -1.266913 -0.449799
C -0.967131 0.013663 -0.130638
C -3.198867 -0.910662 -0.028285
C -0.071051 1.094987 -0.039443
C 1.303325 0.917235 -0.247545
C 1.784208 -0.360492 -0.549050
C 0.903541 -1.444573 -0.669553
C 3.237367 -0.525374 -0.765389
C 4.182462 0.534658 -0.152009
C 3.540017 1.920021 -0.185835
C 2.202847 2.075241 -0.213108
O 1.344647 -2.701481 -1.005994
O 3.629987 -1.507323 -1.397745
O 5.394444 0.567090 -0.917403
C 4.564936 0.181862 1.287183
C 4.849329 -1.291944 1.460749
C 6.187102 -1.814278 1.009553
O 3.988253 -2.038976 1.929072
O 4.460282 2.951507 -0.160715
C 3.985770 4.292932 -0.156548
C -4.659588 -0.792752 0.194779
C -5.254575 -1.915798 1.001089
C -5.351348 0.243001 -0.326792
C -6.840418 0.497168 -0.232408
C -7.142404 1.794197 0.540784
C -6.718903 1.733952 2.003195
C -7.427305 0.580245 -1.645375
H -1.112474 -3.315085 -0.767284
H -2.742365 1.157455 0.347717
H -0.457762 2.086366 0.193955
H 1.733075 3.052772 -0.215946
H 2.306304 -2.656763 -1.209962
H 5.182536 0.989805 -1.767811
H 3.754993 0.430202 1.985888
H 5.450073 0.738502 1.619010
H 6.344724 -2.814551 1.422826
H 6.219205 -1.869946 -0.080249
H 6.983170 -1.162474 1.377965
H 3.419419 4.509740 -1.067908
H 3.384698 4.491900 0.736477
H 4.854743 4.957098 -0.133284
H -4.609515 -2.167885 1.851027
H -6.228206 -1.663185 1.427951
H -5.377297 -2.811004 0.382374
H -4.816674 0.985470 -0.919734
H -7.350775 -0.328571 0.272948
H -6.649804 2.649246 0.060625
H -8.220994 1.992330 0.504471
H -5.633326 1.642380 2.104956
H -7.024710 2.649330 2.519974
H -7.187381 0.885642 2.512234
H -8.512437 0.724844 -1.605553
H -7.235532 -0.342457 -2.204573
H -6.998467 1.414327 -2.212488
SCF Energy (B3LYP/6-31G**)= -1361.70090449
Number of imaginary frequencies = 0

1b_c034
MMFF Geometry
C -1.770346 -2.510648 0.166578
C -2.427584 0.124302 0.053794
N -3.073675 -2.173834 0.319247
C -0.729579 -1.600679 -0.044354
C -1.081622 -0.239122 -0.099461
C -3.390646 -0.862917 0.252875
C -0.079827 0.726015 -0.310997
C 1.264714 0.357489 -0.456392
C 1.608568 -0.994438 -0.389224
C 0.621034 -1.972249 -0.199907
C 3.032830 -1.364145 -0.526348
C 4.109027 -0.280153 -0.327527
C 3.590748 1.101262 -0.697749
C 2.274732 1.381717 -0.731707
O 0.928239 -3.311078 -0.154533
O 3.332870 -2.542440 -0.744423
O 5.188541 -0.643491 -1.204297
C 4.590328 -0.394693 1.123280
C 5.492287 0.736775 1.564224
C 6.908135 0.754081 1.049186
O 5.080059 1.605118 2.336477
O 4.597592 2.012313 -0.961548
C 4.251522 3.368690 -1.210647
C -4.826094 -0.538055 0.429330
C -5.509498 -1.323649 1.516073
C -5.424296 0.376524 -0.363794
C -6.877567 0.799366 -0.349228
C -7.028202 2.284511 0.028873
C -6.575254 2.591023 1.451024
C -7.489450 0.545314 -1.730848
H -1.575227 -3.579541 0.222329
H -2.720614 1.169534 0.032129
H -0.358357 1.778209 -0.364009
H 1.904866 2.371467 -0.976188
H 1.870915 -3.429446 -0.408957
H 5.183538 -1.619342 -1.268721
H 5.137693 -1.332211 1.284388
H 3.736914 -0.414512 1.814274
H 7.484898 1.504282 1.597844
H 7.371076 -0.222297 1.212880
H 6.925983 1.005639 -0.012322
H 3.651678 3.457622 -2.121918
H 3.729750 3.803623 -0.352119
H 5.178384 3.929931 -1.361399
H -4.871289 -1.393481 2.404812
H -6.441772 -0.862975 1.851660
H -5.736808 -2.338403 1.172758
H -4.833786 0.865424 -1.139131

H -7.454780 0.204588 0.365442
H -6.465078 2.916125 -0.669891
H -8.082131 2.574954 -0.064982
H -5.502023 2.419857 1.577889
H -6.774503 3.640676 1.689820
H -7.112961 1.971401 2.175704
H -8.553278 0.806546 -1.736654
H -7.405450 -0.511383 -2.008684
H -6.994082 1.139252 -2.507418
SCF Energy (B3LYP/6-31G**)= -1361.70949274
Number of imaginary frequencies = 0

1b_c035
MMFF Geometry
C 1.847193 2.382204 0.081183
C 2.383847 -0.176463 -0.662213
N 3.145590 2.009553 -0.022222
C 0.754053 1.545700 -0.165636
C 1.044105 0.224063 -0.552836
C 3.401382 0.733671 -0.383101
C -0.012531 -0.663035 -0.829251
C -1.349605 -0.259803 -0.709526
C -1.630327 1.048649 -0.310006
C -0.590264 1.954130 -0.054112
C -3.045326 1.451617 -0.170676
C -4.126162 0.359477 -0.061908
C -3.713674 -0.913277 -0.785800
C -2.425001 -1.191166 -1.057505
O -0.837981 3.255227 0.312862
O -3.326761 2.651764 -0.090840
O -5.297552 0.912920 -0.684495
C -4.407066 0.161267 1.431878
C -5.283528 -1.032326 1.740600
C -6.754872 -0.927306 1.433517
O -4.804661 -2.050393 2.245138
O -4.780239 -1.736565 -1.098796
C -4.521532 -3.008992 -1.678085
C 4.834582 0.369352 -0.486242
C 5.698055 1.423878 -1.124648
C 5.278734 -0.809943 -0.001254
C 6.704337 -1.317892 0.022521
C 7.155684 -1.663768 1.454857
C 7.209010 -0.450480 2.375278
C 6.811955 -2.553289 -0.875053
H 1.701735 3.417904 0.380972
H 2.631762 -1.185927 -0.975756
H 0.216640 -1.681355 -1.142310
H -2.128044 -2.107478 -1.555873
H -1.801000 3.433173 0.221755
H -5.263764 1.879623 -0.540257
H -4.893726 1.045717 1.862464
H -3.469747 0.025101 1.987817
H -7.280773 -1.775133 1.881956
H -7.155972 -0.006950 1.865360
H -6.922593 -0.941273 0.355523
H -4.050101 -2.902382 -2.660188
H -3.908632 -3.624979 -1.012208
H -5.480783 -3.515947 -1.818170
H 5.916188 2.225947 -0.411625
H 6.648375 1.029092 -1.492237
H 5.197063 1.861731 -1.996046
H 4.565435 -1.486121 0.470873
H 7.397912 -0.566691 -0.367710
H 6.490983 -2.418148 1.894556
H 8.157079 -2.110827 1.418641
H 6.215523 -0.019420 2.531599
H 7.603655 -0.739849 3.354560
H 7.861999 0.325492 1.963274
H 7.842220 -2.924494 -0.901096
H 6.518136 -2.317270 -1.904037
H 6.170557 -3.367627 -0.519128
SCF Energy (B3LYP/6-31G**)= -1361.70962261
Number of imaginary frequencies = 0

1b_c036
MMFF Geometry
C -1.520783 -2.520818 -0.265597
C -2.322869 0.037508 0.170395
N -2.839295 -2.297909 -0.044484
C -0.534359 -1.529225 -0.289047
C -0.961481 -0.210058 -0.061049
C -3.227897 -1.021924 0.163979
C -0.015675 0.830408 -0.069288
C 1.346854 0.581042 -0.301329
C 1.770205 -0.732359 -0.524076
C 0.833698 -1.782209 -0.521389
C 3.208716 -1.001292 -0.767443
C 4.213726 0.172051 -0.740748
C 3.598729 1.542477 -0.506057
C 2.280045 1.709845 -0.307275
O 1.203628 -3.088208 -0.747800
O 3.550230 -2.164027 -1.007769
O 4.827313 0.200924 -2.048612
C 5.343892 -0.161838 0.243116
C 4.902203 -0.214655 1.689028
C 5.999408 -0.310268 2.716833
O 3.718783 -0.211731 2.025466
O 4.532213 2.564401 -0.528681
C 4.094801 3.897189 -0.290726
C -4.676153 -0.822660 0.408822
C -5.302980 -1.857981 1.303768
C -5.336269 0.206945 -0.163499
C -6.813102 0.519336 -0.052277
C -7.078749 1.683988 0.921199
C -6.474010 3.025059 0.523353
C -7.401114 0.779973 -1.442679
H -1.264971 -3.565441 -0.430138
H -2.671418 1.046288 0.368524
H -0.352810 1.850581 0.111523
H 1.848275 2.689469 -0.135165
H 2.177419 -3.114724 -0.892922
H 4.195108 0.619718 -2.656517
H 6.148759 0.579115 0.156008
H 5.793624 -1.132220 -0.002831
H 5.564183 -0.338313 3.719639
H 6.574373 -1.225147 2.554284
H 6.650440 0.564076 2.640486
H 3.391348 4.221570 -1.064168
H 3.654482 3.992020 0.707046
H 4.970451 4.551398 -0.336133
H -4.646612 -2.089357 2.150896
H -6.248230 -1.522557 1.738893
H -5.492042 -2.783113 0.749213
H -4.784433 0.886707 -0.812540
H -7.348598 -0.352590 0.340176
H -8.162903 1.813112 1.031259
H -6.699855 1.413875 1.915363
H -6.876815 3.380843 -0.429220
H -6.710411 3.778024 1.282550
H -5.384539 2.969007 0.442140
H -8.466353 1.024940 -1.373159
H -7.305508 -0.110172 -2.075024
H -6.896251 1.604169 -1.957717
SCF Energy (B3LYP/6-31G**)= -1361.70615699
Number of imaginary frequencies = 0

1b_c037
MMFF Geometry
C -1.512548 -2.018890 -0.955074
C -2.295928 0.548510 -0.527311
N -2.841754 -1.755979 -0.927285
C -0.505883 -1.064068 -0.781130
C -0.923198 0.262660 -0.563137
C -3.220092 -0.479436 -0.703765
C 0.041862 1.273398 -0.396987
C 1.411806 0.979750 -0.426161
C 1.818042 -0.343310 -0.624207
C 0.871592 -1.358688 -0.820669
C 3.268023 -0.630544 -0.652376
C 4.216225 0.377028 0.039634
C 3.699844 1.805826 -0.118450
C 2.393443 2.064090 -0.317104

O 1.244413 -2.658720 -1.062624
O 3.651827 -1.665881 -1.199213
O 5.510727 0.282137 -0.569518
C 4.390397 0.056272 1.525820
C 4.528451 -1.426651 1.780684
C 5.863164 -2.070692 1.516498
O 3.557923 -2.083544 2.161974
O 4.692634 2.762148 -0.012147
C 4.334232 4.135583 -0.111312
C -4.682320 -0.236208 -0.682568
C -5.449509 -0.978553 -1.743480
C -5.230141 0.567114 0.254341
C -6.696911 0.882992 0.453180
C -7.162838 0.523882 1.877566
C -7.091176 -0.969453 2.172359
C -6.930658 2.371650 0.183556
H -1.265893 -3.064314 -1.128931
H -2.641805 1.566519 -0.377488
H -0.286671 2.301021 -0.245623
H 2.010266 3.074444 -0.410021
H 2.224301 -2.698406 -1.144106
H 5.439604 0.684243 -1.452717
H 3.524936 0.396456 2.109876
H 5.271718 0.555370 1.947246
H 6.659895 -1.468250 1.959874
H 5.885991 -3.061899 1.978208
H 6.022593 -2.174650 0.441522
H 3.903031 4.356858 -1.092862
H 3.647533 4.419136 0.692684
H 5.246056 4.729981 -0.001588
H -5.582883 -2.027582 -1.458787
H -6.436960 -0.549973 -1.931486
H -4.919313 -0.944518 -2.702530
H -4.578287 1.028525 0.996643
H -7.320836 0.320129 -0.247909
H -6.567401 1.065356 2.623563
H -8.202285 0.849759 2.009165
H -6.059766 -1.334382 2.155493
H -7.500427 -1.175389 3.166631
H -7.672577 -1.541333 1.442154
H -7.991582 2.622618 0.290203
H -6.627400 2.638062 -0.835220
H -6.365033 3.001392 0.879771
SCF Energy (B3LYP/6-31G**)= -1361.70102980
Number of imaginary frequencies = 0

1b_c038
MMFF Geometry
C 1.882493 -1.513461 0.177115
C 2.343414 1.131087 -0.231295
N 3.168088 -1.086844 0.140722
C 0.764562 -0.690973 0.018849
C 1.015360 0.675770 -0.193920
C 3.395018 0.231696 -0.061018
C -0.068427 1.554814 -0.370511
C -1.392241 1.095336 -0.324429
C -1.638557 -0.261315 -0.102335
C -0.566969 -1.154756 0.055396
C -3.042753 -0.735926 -0.072053
C -4.183150 0.290845 0.151610
C -3.759616 1.679953 -0.307826
C -2.489292 2.030541 -0.548073
O -0.767866 -2.501609 0.246804
O -3.240447 -1.948931 -0.186276
O -4.440123 0.397405 1.554117
C -5.432955 -0.176115 -0.623988
C -6.020066 -1.456799 -0.064002
C -6.628727 -2.420012 -1.046409
O -6.046021 -1.669340 1.150303
O -4.757398 2.615985 -0.557353
C -5.293940 3.144343 0.657941
C 4.822591 0.665387 -0.076246
C 5.104032 2.091966 0.311680
C 5.782543 -0.228965 -0.401128
C 7.275990 -0.009261 -0.495741
C 8.036428 -0.827417 0.564246
C 7.729082 -0.394514 1.992357
C 7.752556 -0.399257 -1.899111
H 1.770575 -2.582475 0.345047
H 2.544656 2.182743 -0.406566
H 0.126244 2.612681 -0.544300
H -2.256667 3.033506 -0.893865
H -1.728819 -2.691669 0.154851
H -4.892939 -0.432005 1.825579
H -5.183813 -0.332473 -1.680646
H -6.244301 0.559244 -0.572870
H -7.000068 -3.301330 -0.516285
H -7.461861 -1.937343 -1.562890
H -5.870411 -2.737739 -1.766272
H -5.971875 2.425789 1.127635
H -4.501542 3.440788 1.354057
H -5.875037 4.036134 0.404941
H 4.498171 2.389133 1.175464
H 6.142012 2.256937 0.610693
H 4.883798 2.767987 -0.521035
H 5.475296 -1.249175 -0.641834
H 7.529340 1.046831 -0.361214
H 7.809637 -1.895848 0.457722
H 9.115460 -0.717662 0.398117
H 6.682398 -0.578203 2.252821
H 8.350127 -0.957520 2.696514
H 7.939438 0.670468 2.132891
H 8.826167 -0.211367 -2.009065
H 7.232857 0.185521 -2.666467
H 7.575127 -1.460935 -2.105497
SCF Energy (B3LYP/6-31G**)= -1361.70912147
Number of imaginary frequencies = 0

1b_c039
MMFF Geometry
C 1.851051 -1.668360 -0.100081
C 2.373601 0.951846 -0.584102
N 3.145406 -1.286899 -0.223497
C 0.753517 -0.810436 -0.203466
C 1.036115 0.543657 -0.453908
C 3.403104 0.019310 -0.464543
C -0.026374 1.457768 -0.572345
C -1.359241 1.045218 -0.434464
C -1.636505 -0.299199 -0.177253
C -0.587812 -1.227191 -0.074834
C -3.051055 -0.724376 -0.049982
C -4.139295 0.344022 0.231144
C -3.699441 1.711875 -0.274425
C -2.436134 2.015178 -0.600639
O -0.821368 -2.563512 0.150342
O -3.296588 -1.931134 -0.133441
O -4.301844 0.476572 1.645493
C -5.451469 -0.088735 -0.456264
C -6.044389 -1.341338 0.158456
C -6.747187 -2.294774 -0.769124
O -5.999426 -1.538016 1.374875
O -4.678945 2.678927 -0.472883
C -5.117583 3.240399 0.766612
C 4.839107 0.397838 -0.607725
C 5.143473 1.615666 -1.437622
C 5.786833 -0.365796 -0.020115
C 7.284721 -0.157169 -0.012204
C 7.844665 -0.149549 1.423853
C 7.329064 1.014381 2.261471
C 7.952153 -1.265308 -0.830302
H 1.714168 -2.730194 0.093024
H 2.599637 1.997641 -0.764231
H 0.192399 2.506035 -0.773481
H -2.192477 3.005241 -0.974842
H -1.792088 -2.721403 0.122416
H -4.763995 -0.333274 1.957281
H -5.276218 -0.266543 -1.524307
H -6.232485 0.674916 -0.364059
H -7.113323 -3.156127 -0.203736
H -7.594968 -1.789822 -1.238302
H -6.047891 -2.647406 -1.531248
H -5.787900 2.551712 1.289024

H -4.272264 3.517380 1.406197
H -5.683054 4.148824 0.538617
H 4.962435 2.529045 -0.861523
H 6.176668 1.642078 -1.792914
H 4.521782 1.640133 -2.340054
H 5.464962 -1.245761 0.541264
H 7.551978 0.800086 -0.470519
H 7.605455 -1.091477 1.933575
H 8.939214 -0.084135 1.383061
H 6.248673 0.948001 2.421569
H 7.811608 1.011838 3.244129
H 7.549186 1.972026 1.779045
H 9.037403 -1.120707 -0.865443
H 7.584084 -1.266797 -1.862459
H 7.759009 -2.255287 -0.401528
SCF Energy (B3LYP/6-31G**)= -1361.70915884
Number of imaginary frequencies = 0

1b_c040
MMFF Geometry
C -1.756543 -1.576526 -0.600562
C -2.011874 1.066212 -0.037360
N -3.004725 -1.078575 -0.424825
C -0.579499 -0.828512 -0.516020
C -0.723957 0.537363 -0.224232
C -3.128955 0.238728 -0.143300
C 0.424529 1.343307 -0.126234
C 1.711773 0.813231 -0.313204
C 1.852409 -0.547713 -0.598688
C 0.711907 -1.364363 -0.703341
C 3.209765 -1.113334 -0.794635
C 4.442064 -0.192405 -0.650404
C 4.126387 1.265821 -0.358218
C 2.866224 1.708344 -0.207681
O 0.801606 -2.706088 -0.995481
O 3.304129 -2.308798 -1.091737
O 5.120636 -0.232147 -1.925465
C 5.415173 -0.816381 0.359788
C 4.890759 -0.845891 1.778480
C 5.879539 -1.237916 2.845264
O 3.719834 -0.596591 2.062116
O 5.258917 2.057553 -0.276042
C 5.108158 3.439785 0.025512
C -4.518637 0.753009 0.030961
C -4.747160 2.212082 -0.258216
C -5.499468 -0.099264 0.403030
C -6.959176 0.210243 0.657224
C -7.811550 -0.389595 -0.475323
C -9.287675 -0.029084 -0.379133
C -7.355579 -0.331681 2.034367
H -1.726648 -2.641363 -0.821415
H -2.129789 2.117469 0.203322
H 0.307539 2.401990 0.103092
H 2.648092 2.748763 0.005902
H 1.753035 -2.938993 -1.098105
H 4.629596 0.345457 -2.533626
H 6.364938 -0.266538 0.355417
H 5.657498 -1.847711 0.073360
H 5.392247 -1.221268 3.824087
H 6.247559 -2.248031 2.649712
H 6.710184 -0.527915 2.853938
H 4.536637 3.950221 -0.756217
H 4.643187 3.577501 1.006970
H 6.105952 3.887061 0.059655
H -4.201019 2.523833 -1.156006
H -5.796046 2.447158 -0.456959
H -4.417706 2.828094 0.584900
H -5.239272 -1.148812 0.557883
H -7.123822 1.292167 0.684128
H -7.435882 -0.028301 -1.441565
H -7.712475 -1.482534 -0.489746
H -9.425141 1.055690 -0.330985
H -9.821770 -0.398963 -1.260390
H -9.754682 -0.478582 0.501909
H -8.369529 -0.020567 2.304823
H -6.682795 0.046629 2.812645
H -7.319464 -1.426603 2.061468
SCF Energy (B3LYP/6-31G**)= -1361.70816099
Number of imaginary frequencies = 0

1b_c041
MMFF Geometry
C 1.746551 2.517124 0.190192
C 2.395378 -0.117951 0.032352
N 3.047327 2.172366 0.347369
C 0.704650 1.615230 -0.047250
C 1.052166 0.253225 -0.125925
C 3.360072 0.861763 0.259010
C 0.049332 -0.704253 -0.366586
C -1.291935 -0.327261 -0.515268
C -1.631912 1.025056 -0.422095
C -0.642781 1.995345 -0.207112
C -3.057149 1.401749 -0.558792
C -4.121072 0.310295 -0.299498
C -3.617969 -1.050013 -0.770128
C -2.304627 -1.336407 -0.835454
O -0.947501 3.333756 -0.140900
O -3.324887 2.576826 -0.816523
O -5.306924 0.664618 -1.020033
C -4.450695 0.360794 1.196907
C -5.287347 -0.808175 1.665544
C -6.758360 -0.799076 1.341486
O -4.773411 -1.743491 2.283497
O -4.636743 -1.925952 -1.097316
C -4.313939 -3.297794 -1.297293
C 4.792687 0.528208 0.442271
C 5.468682 1.290266 1.550237
C 5.395037 -0.373129 -0.362785
C 6.846660 -0.801379 -0.342911
C 6.988700 -2.294039 0.007943
C 6.521501 -2.626160 1.419695
C 7.472237 -0.523062 -1.713683
H 1.554445 3.585437 0.265120
H 2.685075 -1.163591 -0.006523
H 0.324591 -1.756030 -0.439647
H -1.939413 -2.308211 -1.149404
H -1.886305 3.457600 -0.411625
H -5.139367 0.467077 -1.958148
H -4.992508 1.280889 1.450285
H -3.536283 0.367020 1.804677
H -7.269103 -1.548945 1.952407
H -7.183612 0.179912 1.576246
H -6.914427 -1.034930 0.287359
H -3.696689 -3.425566 -2.192038
H -3.819916 -3.717713 -0.415056
H -5.249245 -3.843486 -1.452775
H 4.822425 1.345456 2.434178
H 6.396100 0.819719 1.885581
H 5.702851 2.310547 1.228597
H 4.810060 -0.844813 -1.152793
H 7.419230 -0.222501 0.388356
H 6.429947 -2.910099 -0.708004
H 8.042459 -2.586412 -0.081742
H 5.447727 -2.453603 1.539914
H 6.714932 -3.680913 1.640096
H 7.054560 -2.022500 2.161088
H 8.535176 -0.787954 -1.714681
H 7.394434 0.539066 -1.971921
H 6.982092 -1.100207 -2.506068
SCF Energy (B3LYP/6-31G**)= -1361.70349807
Number of imaginary frequencies = 0

1b_c042
MMFF Geometry
C -1.735441 -1.711332 -0.169373
C -2.023017 0.950400 0.277246
N -2.980273 -1.236234 0.078551
C -0.577668 -0.930542 -0.216315
C -0.739067 0.445211 0.013855
C -3.119930 0.090287 0.303746
C 0.388543 1.284816 -0.027165
C 1.672051 0.778262 -0.289638

C 1.830409 -0.592643 -0.511015
C 0.710226 -1.442809 -0.478691
C 3.184259 -1.133460 -0.785475
C 4.395903 -0.174649 -0.791448
C 4.060591 1.289388 -0.555719
C 2.803627 1.707344 -0.328018
O 0.816506 -2.795924 -0.705415
O 3.290601 -2.340975 -1.024019
O 4.972997 -0.270128 -2.112692
C 5.463723 -0.714657 0.170259
C 5.053843 -0.676172 1.625979
C 6.134823 -0.981608 2.629775
O 3.902076 -0.440521 1.988718
O 5.171615 2.113347 -0.609848
C 5.003407 3.506582 -0.375698
C -4.501477 0.573643 0.592252
C -4.625960 1.821672 1.423860
C -5.559256 -0.130099 0.131201
C -7.031835 0.184587 0.285476
C -7.698791 0.194134 -1.104117
C -9.156965 0.631730 -1.073771
C -7.667262 -0.839370 1.228263
H -1.691602 -2.785392 -0.336777
H -2.156691 2.013666 0.446335
H 0.258342 2.351467 0.152803
H 2.571817 2.752409 -0.155463
H 1.763378 -3.009863 -0.871723
H 4.418433 0.258791 -2.710460
H 6.393289 -0.141934 0.059345
H 5.713535 -1.754201 -0.077427
H 5.725940 -0.921278 3.642198
H 6.516016 -1.991686 2.461114
H 6.942187 -0.251630 2.532603
H 4.356304 3.955311 -1.136082
H 4.613974 3.689749 0.630887
H 5.986714 3.980607 -0.447948
H -4.451158 2.711910 0.810843
H -5.607608 1.924102 1.893478
H -3.904607 1.816239 2.249171
H -5.362694 -1.041730 -0.437645
H -7.170150 1.178527 0.722307
H -7.151029 0.880087 -1.763481
H -7.636468 -0.800084 -1.564783
H -9.264494 1.609048 -0.592929
H -9.545020 0.712438 -2.094354
H -9.782072 -0.089571 -0.539635
H -8.707617 -0.581750 1.449871
H -7.133014 -0.875803 2.184659
H -7.652567 -1.846464 0.796487
SCF Energy (B3LYP/6-31G**)= -1361.70808175
Number of imaginary frequencies = 0

1b_c043
MMFF Geometry
C 1.823888 2.393442 0.140577
C 2.351107 -0.142250 -0.684967
N 3.120846 2.017182 0.031497
C 0.727931 1.571619 -0.140007
C 1.012922 0.261692 -0.569947
C 3.371825 0.752536 -0.369919
C -0.046583 -0.609789 -0.883500
C -1.381331 -0.203004 -0.756463
C -1.657300 1.092921 -0.312090
C -0.614325 1.984022 -0.022043
C -3.073715 1.496667 -0.161612
C -4.131436 0.378924 -0.012107
C -3.744882 -0.837984 -0.845687
C -2.463121 -1.106886 -1.155461
O -0.859411 3.273631 0.384663
O -3.331594 2.700567 -0.106797
O -5.390113 0.894120 -0.460700
C -4.254709 0.084235 1.487358
C -5.060477 -1.159114 1.788859
C -6.560556 -1.071569 1.683652
O -4.500575 -2.213137 2.099288
O -4.828998 -1.610441 -1.220132
C -4.585220 -2.899544 -1.772328
C 4.803643 0.383880 -0.477088
C 5.676482 1.454327 -1.074985
C 5.238652 -0.813283 -0.028985
C 6.661389 -1.329514 -0.013762
C 7.102454 -1.724728 1.408981
C 7.156757 -0.542647 2.369097
C 6.767777 -2.535324 -0.950849
H 1.681978 3.419260 0.474289
H 2.595681 -1.142060 -1.030367
H 0.178686 -1.617627 -1.231141
H -2.178058 -1.981325 -1.730308
H -1.821262 3.459400 0.283150
H -5.357470 0.917560 -1.433094
H -4.725402 0.922624 2.016399
H -3.267193 -0.052146 1.947553
H -7.009867 -1.941340 2.171507
H -6.916046 -0.172574 2.193362
H -6.865519 -1.056352 0.635828
H -4.098177 -2.818387 -2.749108
H -3.992999 -3.514760 -1.087117
H -5.551867 -3.390625 -1.917878
H 5.894625 2.230994 -0.334377
H 6.626865 1.066778 -1.450038
H 5.183016 1.923699 -1.934185
H 4.519013 -1.500674 0.416593
H 7.361207 -0.569709 -0.375118
H 6.431201 -2.489501 1.819825
H 8.101671 -2.175824 1.363808
H 6.164653 -0.111537 2.533837
H 7.544101 -0.866188 3.340569
H 7.816240 0.242866 1.986572
H 7.796206 -2.911099 -0.983195
H 6.481277 -2.264023 -1.973178
H 6.120007 -3.357399 -0.625569
SCF Energy (B3LYP/6-31G**)= -1361.70360056
Number of imaginary frequencies = 0

1b_c044
MMFF Geometry
C -1.600398 -2.433479 0.053645
C -2.285731 0.082594 -0.705165
N -2.918879 -2.144546 -0.070025
C -0.557758 -1.531533 -0.183174
C -0.924872 -0.234097 -0.578425
C -3.248987 -0.888111 -0.436739
C 0.078872 0.715109 -0.840574
C 1.440726 0.396872 -0.711876
C 1.803601 -0.892102 -0.310974
C 0.809336 -1.853152 -0.052020
C 3.240740 -1.232442 -0.170825
C 4.306168 -0.146641 -0.441692
C 3.756617 1.198347 -0.889297
C 2.437697 1.428034 -1.008266
O 1.120780 -3.138112 0.329572
O 3.536185 -2.391346 0.139002
O 5.123603 -0.651225 -1.521088
C 5.236917 -0.046845 0.775047
C 4.564908 0.492396 2.018501
C 5.472358 0.825372 3.173950
O 3.345849 0.629908 2.111566
O 4.749279 2.125066 -1.157696
C 4.371510 3.437286 -1.557250
C -4.701119 -0.610256 -0.562456
C -5.487311 -1.720210 -1.207788
C -5.217742 0.540145 -0.078941
C -6.662175 0.990901 -0.106036
C -7.344221 0.813603 1.264857
C -6.760656 1.640102 2.404415
C -6.749053 2.433028 -0.615347
H -1.393403 -3.456342 0.361316
H -2.591062 1.074545 -1.023396
H -0.212442 1.719474 -1.146339
H 2.051875 2.391221 -1.323098
H 2.101065 -3.217923 0.381604
H 4.628498 -0.517883 -2.346750

H 6.094041 0.597126 0.540413
H 5.653024 -1.031731 1.022534
H 4.880039 1.206977 4.010229
H 6.000666 -0.075387 3.495716
H 6.186664 1.594221 2.869342
H 3.825524 3.415000 -2.505767
H 3.783929 3.930031 -0.775986
H 5.285739 4.018769 -1.708502
H -5.039617 -1.996432 -2.169335
H -5.504382 -2.607696 -0.565724
H -6.526381 -1.454617 -1.411897
H -4.547622 1.238942 0.422646
H -7.231072 0.386654 -0.819902
H -8.407462 1.066493 1.165552
H -7.306719 -0.245841 1.549595
H -6.840868 2.712554 2.205324
H -7.309640 1.435180 3.329554
H -5.709572 1.396204 2.583878
H -7.787725 2.780474 -0.620169
H -6.370516 2.503140 -1.641594
H -6.162562 3.124793 -0.001343
SCF Energy (B3LYP/6-31G**)= -1361.70677261
Number of imaginary frequencies = 0

1b_c045
MMFF Geometry
C -1.678181 -2.155099 0.021638
C -2.208150 0.503707 0.196751
N -2.962028 -1.776912 0.235111
C -0.597730 -1.277528 -0.117512
C -0.884290 0.095636 -0.023615
C -3.214542 -0.452919 0.312847
C 0.160653 1.028131 -0.152756
C 1.485131 0.617624 -0.371892
C 1.766415 -0.748998 -0.459744
C 0.731473 -1.692483 -0.340299
C 3.162609 -1.182106 -0.695476
C 4.289357 -0.135800 -0.716063
C 3.826500 1.307300 -0.664627
C 2.531470 1.634952 -0.508588
O 0.965226 -3.044802 -0.438337
O 3.395229 -2.377495 -0.911411
O 4.923844 -0.332337 -2.002484
C 5.338038 -0.469337 0.352775
C 4.866797 -0.158821 1.754646
C 5.920114 0.256466 2.746763
O 3.690303 -0.289509 2.091062
O 4.867997 2.214915 -0.766779
C 4.555334 3.598900 -0.865892
C -4.630070 -0.080807 0.552708
C -5.311411 -0.920847 1.596792
C -5.198591 0.916774 -0.158655
C -6.619655 1.441450 -0.134428
C -7.728949 0.395197 -0.356859
C -7.566500 -0.391946 -1.651165
C -6.884957 2.280296 1.118403
H -1.532719 -3.231706 -0.036356
H -2.450076 1.557790 0.290663
H -0.067456 2.090823 -0.078561
H 2.206052 2.667772 -0.458777
H 1.920196 -3.182871 -0.628789
H 5.049779 -1.295486 -2.102996
H 6.257787 0.096401 0.156197
H 5.622696 -1.528096 0.321425
H 5.455957 0.451405 3.717497
H 6.654054 -0.545324 2.858095
H 6.408117 1.171311 2.401480
H 3.921877 3.797501 -1.736466
H 4.084050 3.958746 0.054210
H 5.492991 4.146531 -0.999163
H -4.650471 -1.086459 2.455737
H -6.210735 -0.450655 1.997633
H -5.592629 -1.895602 1.184340
H -4.587066 1.423546 -0.906866
H -6.689838 2.138389 -0.982889
H -7.794427 -0.304396 0.481844
H -8.696360 0.912593 -0.393860
H -6.667360 -1.014944 -1.634250
H -8.426020 -1.054156 -1.797138
H -7.507385 0.279130 -2.513935
H -7.852547 2.788474 1.041666
H -6.116640 3.051249 1.245397
H -6.903598 1.671793 2.027191
SCF Energy (B3LYP/6-31G**)= -1361.70426631
Number of imaginary frequencies = 0

1b_c046
MMFF Geometry
C -1.599293 -2.417790 -0.159639
C -2.288934 0.208079 -0.257212
N -2.916674 -2.105329 -0.097713
C -0.560311 -1.487835 -0.272391
C -0.929914 -0.132439 -0.325118
C -3.246688 -0.796850 -0.136470
C 0.068846 0.850065 -0.448656
C 1.428658 0.507355 -0.513392
C 1.792429 -0.841116 -0.451603
C 0.804838 -1.834932 -0.339508
C 3.226410 -1.203061 -0.523772
C 4.290237 -0.092780 -0.540320
C 3.745076 1.316956 -0.662097
C 2.424934 1.573691 -0.650699
O 1.121271 -3.173250 -0.297086
O 3.544258 -2.395287 -0.609040
O 5.052170 -0.358414 -1.742429
C 5.251357 -0.265815 0.642762
C 4.634958 0.133364 1.963552
C 5.567186 0.695144 3.003562
O 3.440769 -0.039983 2.204886
O 4.739447 2.276850 -0.755570
C 4.360852 3.624141 -1.009479
C -4.697495 -0.495475 -0.069436
C -5.544446 -1.389234 -0.931878
C -5.146318 0.493428 0.733583
C -6.561264 0.963773 1.001202
C -7.202975 1.727763 -0.171944
C -6.403906 2.950631 -0.605869
C -7.479284 -0.149710 1.514611
H -1.389996 -3.484483 -0.115181
H -2.598761 1.247109 -0.311602
H -0.223915 1.898541 -0.491588
H 2.039333 2.584112 -0.724574
H 2.096209 -3.265891 -0.387711
H 5.240030 -1.316707 -1.743107
H 6.152565 0.338352 0.477083
H 5.596035 -1.303104 0.733395
H 5.004458 0.943204 3.907759
H 6.329009 -0.047900 3.251436
H 6.036115 1.605422 2.621714
H 3.801123 3.705264 -1.946888
H 3.786673 4.032031 -0.171549
H 5.274915 4.216776 -1.110145
H -5.656489 -2.376632 -0.471506
H -6.542716 -0.986931 -1.109654
H -5.089828 -1.518315 -1.921138
H -4.412933 1.041218 1.327700
H -6.480175 1.679130 1.833170
H -7.344529 1.072143 -1.037488
H -8.204072 2.062902 0.127726
H -5.428517 2.669749 -1.014623
H -6.946221 3.495303 -1.385481
H -6.244306 3.633652 0.234491
H -8.407717 0.277691 1.909981
H -7.003099 -0.710698 2.326330
H -7.759510 -0.858077 0.730620
SCF Energy (B3LYP/6-31G**)= -1361.70437463
Number of imaginary frequencies = 0

1b_c047
MMFF Geometry
C 1.555013 -2.385068 -0.179906
C 2.514450 0.157183 -0.243551

N 2.899178 -2.213448 -0.190060
C 0.616972 -1.348172 -0.200557
C 1.125300 -0.036002 -0.237161
C 3.364954 -0.946220 -0.210738
C 0.232370 1.050882 -0.276384
C -1.154638 0.850704 -0.260090
C -1.651884 -0.452839 -0.204707
C -0.777863 -1.549394 -0.194822
C -3.118162 -0.638298 -0.189490
C -3.991544 0.534284 0.319460
C -3.380127 1.868971 -0.117614
C -2.062058 1.995761 -0.365360
O -1.241875 -2.843197 -0.183475
O -3.565780 -1.731499 -0.542968
O -3.978117 0.555151 1.750300
C -5.429371 0.372720 -0.215621
C -6.110801 -0.867619 0.328541
C -7.103902 -1.553379 -0.569791
O -5.901204 -1.260734 1.478428
O -4.295088 2.906738 -0.181308
C -3.813043 4.229597 -0.385519
C 4.840376 -0.802889 -0.223210
C 5.549613 -1.778423 -1.123375
C 5.444933 0.125509 0.548763
C 6.929872 0.382003 0.689810
C 7.308237 1.788810 0.190770
C 7.075163 1.978917 -1.302939
C 7.330914 0.221617 2.159928
H 1.236587 -3.425093 -0.153948
H 2.929522 1.159341 -0.287860
H 0.629047 2.064522 -0.318837
H -1.614343 2.941992 -0.646326
H -2.218027 -2.826682 -0.304268
H -4.527558 -0.200806 2.052781
H -5.421609 0.321175 -1.311002
H -6.070743 1.209385 0.086301
H -7.509970 -2.434402 -0.065246
H -7.921658 -0.866848 -0.801714
H -6.606904 -1.873845 -1.488749
H -3.116220 4.520626 0.407163
H -3.350049 4.327750 -1.372508
H -4.669777 4.908882 -0.347416
H 5.022101 -1.879069 -2.079221
H 6.566066 -1.464772 -1.373226
H 5.605199 -2.765898 -0.653198
H 4.828203 0.761151 1.184894
H 7.512725 -0.350042 0.122285
H 6.745765 2.553904 0.740835
H 8.370345 1.970749 0.397549
H 6.012849 1.913103 -1.556662
H 7.430444 2.966268 -1.615044
H 7.617274 1.225487 -1.883182
H 8.409997 0.363590 2.284139
H 7.083450 -0.780884 2.526822
H 6.820922 0.950940 2.799622
SCF Energy (B3LYP/6-31G**)= -1361.71326244
Number of imaginary frequencies = 0

1b_c048
MMFF Geometry
C -1.699614 -2.124382 -0.072665
C -2.241136 0.519622 0.239637
N -2.988243 -1.761376 0.138430
C -0.619638 -1.238233 -0.144506
C -0.912512 0.127349 0.019854
C -3.246281 -0.444218 0.285074
C 0.130786 1.068286 -0.039632
C 1.458590 0.672368 -0.259546
C 1.748306 -0.687400 -0.416452
C 0.714671 -1.638014 -0.366452
C 3.151707 -1.104259 -0.654987
C 4.279806 -0.055955 -0.611496
C 3.790786 1.371613 -0.505594
C 2.504337 1.692293 -0.317317
O 0.954067 -2.982354 -0.534591
O 3.392087 -2.284929 -0.934823
O 4.938818 -0.211873 -1.889049
C 5.301844 -0.418230 0.471524
C 4.779844 -0.154521 1.864711
C 5.732853 0.475760 2.844059
O 3.640348 -0.479520 2.197380
O 4.742004 2.381530 -0.422616
C 5.348591 2.656991 -1.685489
C -4.666969 -0.089215 0.520515
C -5.363750 -0.988024 1.503693
C -5.226115 0.945704 -0.143357
C -6.648896 1.465426 -0.114334
C -7.751147 0.431211 -0.414500
C -7.564126 -0.281512 -1.748014
C -6.938184 2.231897 1.178905
H -1.549813 -3.195877 -0.186862
H -2.487723 1.566429 0.387697
H -0.099726 2.125273 0.089614
H 2.220855 2.731498 -0.177266
H 1.914009 -3.109394 -0.705805
H 5.039452 -1.174978 -2.027276
H 6.223802 0.157250 0.319268
H 5.595916 -1.473363 0.416195
H 5.233460 0.614943 3.806841
H 6.598273 -0.176550 2.983581
H 6.050853 1.451805 2.469219
H 6.173824 1.963649 -1.870523
H 4.623060 2.627026 -2.506002
H 5.767418 3.666808 -1.640198
H -4.717314 -1.200304 2.363411
H -6.271313 -0.542970 1.914537
H -5.634815 -1.938622 1.032278
H -4.603144 1.495370 -0.850738
H -6.706483 2.208882 -0.923340
H -7.829045 -0.314609 0.382258
H -8.719275 0.948002 -0.439143
H -6.663606 -0.902739 -1.750548
H -8.419076 -0.936112 -1.945917
H -7.492095 0.437177 -2.570515
H -7.905790 2.741702 1.114093
H -6.174424 2.995975 1.362382
H -6.970691 1.573164 2.051564
SCF Energy (B3LYP/6-31G**)= -1361.69855264
Number of imaginary frequencies = 0

1b_c049
MMFF Geometry
C -1.427738 -2.305492 -0.490876
C -2.359056 0.180991 0.090867
N -2.759850 -2.156294 -0.291676
C -0.488798 -1.271641 -0.420064
C -0.982295 0.011463 -0.116405
C -3.212687 -0.915742 -0.011099
C -0.088343 1.095263 -0.033014
C 1.287040 0.918380 -0.235235
C 1.769638 -0.361535 -0.522555
C 0.892719 -1.449079 -0.633606
C 3.219827 -0.522135 -0.741058
C 4.173691 0.549612 -0.178981
C 3.524371 1.928555 -0.180228
C 2.185773 2.077224 -0.197013
O 1.337587 -2.708871 -0.953352
O 3.640299 -1.503628 -1.360381
O 5.321224 0.579508 -1.047447
C 4.656559 0.221057 1.235788
C 4.942290 -1.249355 1.423231
C 6.307087 -1.756034 1.041393
O 4.063718 -2.007246 1.836873
O 4.436906 2.966092 -0.112129
C 3.959959 4.303181 -0.191654
C -4.674376 -0.797970 0.205623
C -5.270400 -1.913062 1.022081
C -5.365982 0.230861 -0.329669
C -6.855753 0.483737 -0.243290
C -7.162429 1.788688 0.514574
C -6.743967 1.745139 1.979027
C -7.437801 0.550402 -1.659125

H -1.119971 -3.324236 -0.717419
H -2.760945 1.157394 0.343195
H -0.477203 2.088240 0.189940
H 1.714666 3.053622 -0.171325
H 2.299780 -2.667968 -1.149753
H 5.271047 -0.216196 -1.614111
H 3.900104 0.487832 1.985936
H 5.564804 0.780547 1.491447
H 6.424969 -2.785308 1.391856
H 6.422947 -1.734831 -0.043940
H 7.075868 -1.140954 1.515586
H 3.424293 4.473514 -1.131133
H 3.328580 4.543731 0.669567
H 4.825934 4.971376 -0.170960
H -4.628012 -2.154654 1.877075
H -6.246011 -1.657462 1.442596
H -5.389350 -2.815244 0.412835
H -4.830410 0.967761 -0.928784
H -7.366618 -0.337209 0.269309
H -6.669455 2.639194 0.026790
H -8.241187 1.984722 0.472326
H -5.658615 1.656393 2.085573
H -7.052987 2.665644 2.484668
H -7.212928 0.901724 2.495698
H -8.523285 0.693734 -1.624684
H -7.242660 -0.378075 -2.207494
H -7.008245 1.378885 -2.233850
SCF Energy (B3LYP/6-31G**)= -1361.70547938
Number of imaginary frequencies = 0

1b_c050
MMFF Geometry
C -1.624037 -2.381943 -0.178952
C -2.320019 0.243350 -0.245761
N -2.943246 -2.074167 -0.134581
C -0.586012 -1.447617 -0.259336
C -0.959135 -0.092274 -0.296559
C -3.276562 -0.766063 -0.157419
C 0.037846 0.895192 -0.387527
C 1.398240 0.555967 -0.436344
C 1.767500 -0.792716 -0.388883
C 0.781273 -1.790496 -0.308806
C 3.205908 -1.150376 -0.446237
C 4.272261 -0.038496 -0.439953
C 3.706391 1.358832 -0.564569
C 2.393756 1.622077 -0.534036
O 1.100646 -3.128463 -0.282585
O 3.529177 -2.340024 -0.547812
O 5.057004 -0.312904 -1.623042
C 5.202793 -0.199869 0.767034
C 4.538425 0.204129 2.062585
C 5.363928 1.004115 3.033767
O 3.388337 -0.141796 2.331588
O 4.595067 2.426871 -0.530306
C 5.304206 2.578727 -1.760245
C -4.729240 -0.469896 -0.109628
C -5.559259 -1.352663 -0.999381
C -5.194215 0.505327 0.700900
C -6.614728 0.967679 0.952394
C -7.239415 1.747750 -0.219356
C -6.437000 2.979288 -0.621417
C -7.537710 -0.156026 1.433628
H -1.412417 -3.448634 -0.147672
H -2.631781 1.282400 -0.288261
H -0.255184 1.944138 -0.417663
H 2.047890 2.651541 -0.556339
H 2.077749 -3.217438 -0.348127
H 5.217197 -1.277855 -1.625228
H 6.105901 0.406793 0.623052
H 5.551795 -1.233904 0.875714
H 4.769009 1.232696 3.922327
H 6.240720 0.424530 3.332504
H 5.671732 1.942796 2.566403
H 6.176105 1.918920 -1.780696
H 4.661886 2.402715 -2.630292
H 5.667062 3.609775 -1.809954
H -5.675766 -2.347333 -0.556090
H -6.555733 -0.950502 -1.187271
H -5.088187 -1.465235 -1.982943
H -4.472319 1.046165 1.315172
H -6.549408 1.670454 1.796386
H -7.364813 1.105049 -1.096997
H -8.246283 2.075530 0.068902
H -5.454211 2.707355 -1.018325
H -6.968095 3.534325 -1.401443
H -6.293229 3.649821 0.231760
H -8.473772 0.262742 1.820150
H -7.073221 -0.728044 2.244425
H -7.802929 -0.853099 0.634440
SCF Energy (B3LYP/6-31G**)= -1361.69867360
Number of imaginary frequencies = 0

1b_c051
MMFF Geometry
C 2.010000 1.695866 -0.070506
C 2.183896 -1.009814 -0.227546
N 3.237813 1.128545 0.005125
C 0.814405 0.990056 -0.222855
C 0.916106 -0.410290 -0.302885
C 3.322297 -0.219664 -0.073608
C -0.252328 -1.178588 -0.461885
C -1.515990 -0.575415 -0.528794
C -1.609574 0.814796 -0.435989
C -0.454732 1.598515 -0.299449
C -2.950591 1.433411 -0.489774
C -4.189750 0.554766 -0.234421
C -3.946960 -0.891022 -0.640452
C -2.705813 -1.400034 -0.750884
O -0.515066 2.969838 -0.231646
O -3.047746 2.649260 -0.685190
O -5.234624 1.116791 -1.045855
C -4.561297 0.731180 1.242213
C -5.623436 -0.229599 1.728972
C -7.046642 0.012032 1.297372
O -5.328946 -1.169372 2.470880
O -5.112954 -1.604859 -0.850759
C -5.028312 -2.997277 -1.125612
C 4.693130 -0.804583 -0.002874
C 4.898631 -2.139213 -0.667279
C 5.676471 -0.113365 0.615370
C 7.120451 -0.515041 0.818916
C 8.079895 0.417974 0.057228
C 7.924456 0.334676 -1.456135
C 7.442558 -0.487263 2.316965
H 2.014143 2.781573 -0.001786
H 2.269749 -2.090371 -0.276363
H -0.169628 -2.262674 -0.535401
H -2.532216 -2.435927 -1.020945
H -1.434295 3.257644 -0.430449
H -5.059625 2.077123 -1.105922
H -4.923943 1.748181 1.438917
H -3.680687 0.589665 1.883053
H -7.715493 -0.633239 1.874213
H -7.319028 1.052076 1.493684
H -7.168615 -0.215091 0.237210
H -4.505249 -3.177787 -2.070047
H -4.545640 -3.530604 -0.300342
H -6.047207 -3.382754 -1.225860
H 4.387725 -2.176317 -1.636348
H 5.948603 -2.353524 -0.880889
H 4.513611 -2.946839 -0.036067
H 5.430067 0.860495 1.044355
H 7.297048 -1.540363 0.479630
H 7.934256 1.458223 0.375027
H 9.114669 0.154674 0.309954
H 6.940270 0.686084 -1.780206
H 8.677969 0.960348 -1.945280
H 8.058566 -0.693177 -1.807873
H 8.472517 -0.813966 2.497194
H 6.779035 -1.158852 2.873375
H 7.332180 0.519728 2.735322
SCF Energy (B3LYP/6-31G**)= -1361.71066562

Number of imaginary frequencies = 0

1b_c052
MMFF Geometry
C 1.978003 1.790799 0.152011
C 2.206871 -0.915015 0.198055
N 3.206719 1.256421 0.351567
C 0.807845 1.050780 -0.032586
C 0.938273 -0.349313 -0.008947
C 3.318065 -0.091857 0.375679
C -0.202153 -1.151517 -0.200372
C -1.467496 -0.581502 -0.398015
C -1.590994 0.809460 -0.405450
C -0.462428 1.625277 -0.240318
C -2.934864 1.393926 -0.596043
C -4.174112 0.507295 -0.370363
C -3.878239 -0.956240 -0.661429
C -2.623924 -1.444394 -0.649229
O -0.550417 2.996439 -0.270720
O -3.038235 2.591793 -0.879442
O -5.167920 0.992465 -1.288499
C -4.653089 0.771393 1.061577
C -5.729223 -0.179851 1.536123
C -7.122523 0.000698 0.991799
O -5.470760 -1.062509 2.357373
O -5.013716 -1.707208 -0.906512
C -4.885638 -3.112082 -1.083888
C 4.684227 -0.641609 0.613460
C 4.769689 -2.007225 1.239902
C 5.763686 0.103431 0.287568
C 7.225758 -0.261467 0.416545
C 7.969879 -0.087097 -0.922008
C 7.472840 -1.030359 -2.010765
C 7.870503 0.614012 1.493832
H 1.959259 2.878506 0.145097
H 2.318432 -1.994331 0.205069
H -0.096037 -2.236042 -0.195165
H -2.413652 -2.491600 -0.837089
H -1.457678 3.250444 -0.553023
H -5.004650 1.950157 -1.400947
H -5.047497 1.790437 1.163958
H -3.817959 0.692508 1.770459
H -7.818303 -0.622437 1.561072
H -7.427734 1.044397 1.101153
H -7.164652 -0.295577 -0.057415
H -4.292583 -3.340714 -1.975041
H -4.454929 -3.581426 -0.193560
H -5.887874 -3.525144 -1.231165
H 4.592366 -2.785976 0.490891
H 5.739247 -2.202810 1.704840
H 4.031408 -2.116960 2.042638
H 5.596383 1.095002 -0.138806
H 7.346094 -1.305558 0.721897
H 7.882710 0.947590 -1.277098
H 9.039459 -0.276335 -0.766545
H 6.433263 -0.821581 -2.280901
H 8.080807 -0.914169 -2.913732
H 7.543473 -2.073776 -1.687403
H 8.924855 0.349149 1.628367
H 7.369265 0.481057 2.459186
H 7.822126 1.677139 1.231860
SCF Energy (B3LYP/6-31G**)= -1361.71062539
Number of imaginary frequencies = 0

1b_c053
MMFF Geometry
C -1.632281 -2.188484 -0.196603
C -2.462097 0.389802 -0.423952
N -2.956701 -1.960260 -0.370023
C -0.652769 -1.193112 -0.124922
C -1.093926 0.138251 -0.244715
C -3.358967 -0.675487 -0.474722
C -0.157128 1.186094 -0.176360
C 1.209517 0.926953 -0.005082
C 1.641862 -0.396660 0.097970
C 0.720226 -1.452807 0.058097
C 3.087603 -0.644616 0.279414
C 4.075866 0.446143 -0.200489
C 3.485479 1.835970 0.056472
C 2.155413 2.037488 0.127549
O 1.115255 -2.762110 0.196082
O 3.431857 -1.724692 0.764535
O 4.238541 0.353548 -1.619254
C 5.427208 0.267374 0.521816
C 6.105367 -1.039720 0.160530
C 6.944067 -1.693275 1.224940
O 6.018417 -1.513553 -0.974650
O 4.438229 2.836190 0.156621
C 4.003874 4.190297 0.198512
C -4.814435 -0.471340 -0.668593
C -5.454520 -1.443436 -1.622820
C -5.466394 0.500422 0.005083
C -6.946477 0.814263 -0.034362
C -7.571788 0.760410 1.373124
C -7.533191 -0.631277 1.992645
C -7.148960 2.202681 -0.646505
H -1.366108 -3.240213 -0.114550
H -2.823227 1.407312 -0.536937
H -0.503175 2.215732 -0.258334
H 1.726422 3.021196 0.279287
H 2.068619 -2.777469 0.438169
H 4.781392 -0.446308 -1.793572
H 5.282841 0.303331 1.608298
H 6.142941 1.049786 0.242390
H 7.362971 -2.627791 0.841719
H 7.761513 -1.025504 1.507252
H 6.322233 -1.918749 2.094809
H 3.424563 4.444959 -0.694882
H 3.430000 4.387574 1.109496
H 4.892804 4.827743 0.216945
H -5.618844 -2.409918 -1.134755
H -6.414261 -1.090486 -2.008072
H -4.818017 -1.602648 -2.501205
H -4.903779 1.128171 0.696547
H -7.485833 0.097047 -0.660728
H -7.065836 1.465686 2.044578
H -8.619697 1.080329 1.313936
H -6.506340 -0.964683 2.170645
H -8.053133 -0.627353 2.956033
H -8.027023 -1.362438 1.344718
H -8.215300 2.443549 -0.715928
H -6.731529 2.249755 -1.658621
H -6.666991 2.982550 -0.045897
SCF Energy (B3LYP/6-31G**)= -1361.71340048
Number of imaginary frequencies = 0

1b_c054
MMFF Geometry
C -1.855613 -1.485834 -0.491066
C -2.034516 1.184280 -0.038435
N -3.088135 -0.947084 -0.325625
C -0.658200 -0.767225 -0.448778
C -0.763131 0.613165 -0.214056
C -3.174425 0.383834 -0.099127
C 0.407462 1.391010 -0.160632
C 1.678117 0.818716 -0.336291
C 1.779398 -0.556399 -0.564857
C 0.616445 -1.345435 -0.624575
C 3.119090 -1.166484 -0.748585
C 4.376990 -0.274115 -0.654579
C 4.103414 1.203205 -0.421918
C 2.857121 1.685784 -0.279184
O 0.667242 -2.700250 -0.860183
O 3.178429 -2.375564 -0.995389
O 5.041476 -0.386176 -1.932749
C 5.343087 -0.881028 0.372596
C 4.832275 -0.836260 1.795844
C 5.820798 -1.209610 2.869542
O 3.671352 -0.543409 2.078972
O 5.257504 1.966631 -0.383545
C 5.146838 3.363968 -0.139764
C -4.548186 0.942266 0.065114

C -4.739671 2.396464 -0.272156
C -5.551448 0.131186 0.468379
C -6.997807 0.496687 0.717227
C -7.905372 0.076485 -0.455032
C -7.975763 -1.421103 -0.728288
C -7.465829 -0.091486 2.052311
H -1.856517 -2.559461 -0.666744
H -2.121526 2.247643 0.158067
H 0.321176 2.461233 0.024476
H 2.669077 2.739850 -0.108141
H 1.611017 -2.963067 -0.961226
H 4.560018 0.178312 -2.560569
H 6.307144 -0.357876 0.336436
H 5.554835 -1.929752 0.128167
H 5.343936 -1.138344 3.851046
H 6.159624 -2.236730 2.713873
H 6.670231 -0.522749 2.840665
H 4.581396 3.856223 -0.937411
H 4.695566 3.555665 0.839026
H 6.156543 3.785004 -0.133529
H -4.199978 2.660237 -1.188976
H -5.785128 2.654397 -0.461703
H -4.379136 3.031944 0.543343
H -5.318916 -0.918747 0.657968
H -7.092100 1.583616 0.821728
H -8.922619 0.440699 -0.263376
H -7.566816 0.580398 -1.369519
H -8.382671 -1.967127 0.127588
H -8.633404 -1.611390 -1.582993
H -6.992231 -1.834478 -0.970021
H -8.517516 0.153074 2.235986
H -6.879359 0.319839 2.881917
H -7.360181 -1.181107 2.084325
SCF Energy (B3LYP/6-31G**)= -1361.70731586
Number of imaginary frequencies = 0

1b_c055
MMFF Geometry
C -1.723001 -1.389690 -0.683685
C -2.103875 1.157306 0.179151
N -2.992000 -0.977498 -0.446830
C -0.584457 -0.598004 -0.517776
C -0.793012 0.719194 -0.070133
C -3.179012 0.291250 -0.016270
C 0.313480 1.570415 0.110452
C 1.619741 1.124959 -0.133512
C 1.818708 -0.190536 -0.562701
C 0.727482 -1.044600 -0.773731
C 3.204371 -0.639670 -0.815309
C 4.350409 0.130529 -0.117827
C 4.018519 1.617539 -0.009071
C 2.745464 2.055892 -0.001534
O 0.891460 -2.327880 -1.236092
O 3.382027 -1.616952 -1.544410
O 5.545596 -0.021768 -0.895079
C 4.640947 -0.433130 1.274964
C 4.604012 -1.943425 1.302237
C 5.802249 -2.691912 0.782584
O 3.601097 -2.532161 1.710107
O 5.137517 2.421993 0.101277
C 4.960300 3.826871 0.240787
C -4.591233 0.711274 0.218995
C -4.895451 2.179158 0.088278
C -5.521390 -0.227050 0.503969
C -6.993296 -0.032023 0.792007
C -7.872832 -0.681147 -0.292551
C -7.715711 -0.032189 -1.662054
C -7.320755 -0.635597 2.162083
H -1.642624 -2.419625 -1.025074
H -2.273223 2.167540 0.536568
H 0.146621 2.594279 0.443166
H 2.495275 3.106635 0.096316
H 1.841747 -2.470333 -1.448249
H 5.432620 0.515067 -1.698708
H 3.898575 -0.087182 2.006452
H 5.622458 -0.112093 1.645094
H 5.741146 -3.737961 1.096010
H 5.827447 -2.647635 -0.307921
H 6.716904 -2.262757 1.199157
H 4.458842 4.246403 -0.637205
H 4.410126 4.062077 1.157495
H 5.950865 4.285223 0.314729
H -4.381208 2.609361 -0.778888
H -5.957316 2.383322 -0.069227
H -4.580091 2.719243 0.987041
H -5.202048 -1.270732 0.550672
H -7.247194 1.030940 0.849249
H -7.648471 -1.752110 -0.376379
H -8.926388 -0.603601 0.004029
H -6.705320 -0.168437 -2.059141
H -8.415423 -0.483239 -2.373016
H -7.927421 1.040681 -1.613931
H -8.374041 -0.472197 2.414907
H -6.714739 -0.173232 2.949389
H -7.134633 -1.715490 2.182721
SCF Energy (B3LYP/6-31G**)= -1361.70190991
Number of imaginary frequencies = 0

1b_c056
MMFF Geometry
C -1.522283 -2.031871 -0.918237
C -2.313563 0.538467 -0.524285
N -2.852212 -1.772533 -0.895880
C -0.518530 -1.072208 -0.754214
C -0.940000 0.255997 -0.553574
C -3.234523 -0.494181 -0.689567
C 0.021891 1.271341 -0.396806
C 1.392827 0.981719 -0.419800
C 1.801755 -0.342374 -0.601978
C 0.859824 -1.363414 -0.787274
C 3.250033 -0.622060 -0.632422
C 4.211818 0.397125 0.008447
C 3.681090 1.820147 -0.119760
C 2.370929 2.069380 -0.308387
O 1.236968 -2.665381 -1.010636
O 3.662149 -1.658240 -1.161748
O 5.455250 0.302241 -0.710475
C 4.490606 0.093933 1.482551
C 4.631778 -1.384519 1.752922
C 5.987565 -2.009351 1.561566
O 3.651054 -2.054996 2.078509
O 4.659407 2.787215 0.026921
C 4.305539 4.151944 -0.157246
C -4.697457 -0.254820 -0.674328
C -5.460452 -1.012582 -1.727323
C -5.249358 0.558980 0.251072
C -6.717347 0.873620 0.442735
C -7.185288 0.532089 1.870781
C -7.110353 -0.957038 2.185380
C -6.954403 2.357975 0.153031
H -1.272621 -3.078632 -1.078968
H -2.662347 1.557450 -0.388203
H -0.309620 2.299717 -0.257269
H 1.980687 3.078993 -0.374399
H 2.216223 -2.706512 -1.084359
H 5.410274 -0.510897 -1.252282
H 3.672400 0.449377 2.123107
H 5.403100 0.593267 1.830861
H 5.978427 -3.028569 1.958099
H 6.236136 -2.043729 0.499093
H 6.739943 -1.434610 2.107281
H 3.904009 4.321141 -1.161596
H 3.595405 4.477426 0.609548
H 5.213756 4.752555 -0.051257
H -5.591610 -2.058281 -1.429646
H -6.448655 -0.589088 -1.922758
H -4.928356 -0.989261 -2.685641
H -4.600252 1.031917 0.988520
H -7.338352 0.299913 -0.252132
H -6.592805 1.084909 2.610785
H -8.225849 0.856941 1.995956
H -6.077966 -1.319450 2.175413

H -7.521114 -1.150927 3.181451
H -7.688763 -1.540015 1.461603
H -8.016194 2.607541 0.254201
H -6.649732 2.611731 -0.868550
H -6.391857 2.998321 0.842031
SCF Energy (B3LYP/6-31G**)= -1361.70558604
Number of imaginary frequencies = 0

1b_c057
MMFF Geometry
C 1.514139 -2.379336 0.184272
C 2.488661 0.098793 -0.357627
N 2.858744 -2.227530 0.110797
C 0.582372 -1.352560 0.001144
C 1.098843 -0.074921 -0.281784
C 3.332681 -0.990088 -0.149077
C 0.212424 0.996978 -0.488927
C -1.175434 0.818334 -0.403654
C -1.686010 -0.448507 -0.111538
C -0.814293 -1.533243 0.076740
C -3.156215 -0.625930 -0.040548
C -4.059848 0.620256 0.147274
C -3.374678 1.871682 -0.386108
C -2.065454 1.945497 -0.659687
O -1.279749 -2.801012 0.336062
O -3.597836 -1.777335 -0.093148
O -4.257459 0.841003 1.546068
C -5.395945 0.382064 -0.587372
C -6.216802 -0.725737 0.043033
C -7.031091 -1.588375 -0.882258
O -6.256460 -0.873448 1.266549
O -4.168022 2.978249 -0.669074
C -4.557518 3.660846 0.525219
C 4.808389 -0.869072 -0.222828
C 5.488609 -2.003293 -0.941366
C 5.441181 0.178828 0.347139
C 6.933845 0.430548 0.405713
C 7.277285 1.648796 -0.470338
C 8.772434 1.914966 -0.579237
C 7.353896 0.625734 1.865866
H 1.189889 -3.394450 0.403651
H 2.908223 1.072441 -0.591213
H 0.613785 1.983882 -0.717232
H -1.642546 2.863246 -1.057852
H -2.261108 -2.795349 0.265759
H -4.862206 0.134172 1.864361
H -5.208207 0.130311 -1.638286
H -6.040351 1.268479 -0.561631
H -7.560579 -2.350809 -0.304344
H -7.761192 -0.970146 -1.410066
H -6.370151 -2.085969 -1.596229
H -5.355115 3.117582 1.040230
H -3.705123 3.822658 1.194243
H -4.952455 4.639121 0.235436
H 4.928436 -2.286509 -1.840292
H 6.493832 -1.744893 -1.284000
H 5.565077 -2.880673 -0.290567
H 4.847560 0.933527 0.863875
H 7.486247 -0.435835 0.028446
H 6.886001 1.487967 -1.483423
H 6.783060 2.548971 -0.083064
H 9.305061 1.027087 -0.934100
H 8.957530 2.727017 -1.289867
H 9.198370 2.215193 0.382440
H 8.442516 0.691117 1.958815
H 7.026673 -0.218480 2.483669
H 6.922954 1.539809 2.289702
SCF Energy (B3LYP/6-31G**)= -1361.70701170
Number of imaginary frequencies = 0

1b_c058
MMFF Geometry
C -1.685579 -1.595400 -0.407850
C -2.135133 0.926219 0.495569
N -2.955775 -1.247222 -0.088813
C -0.578964 -0.749037 -0.308816
C -0.823308 0.555193 0.158329
C -3.176443 0.008431 0.363441
C 0.248181 1.461495 0.267858
C 1.556270 1.082740 -0.063331
C 1.792647 -0.221015 -0.509071
C 0.734191 -1.128819 -0.651683
C 3.179601 -0.599513 -0.853672
C 4.328712 0.227917 -0.230733
C 3.930027 1.696275 -0.096098
C 2.639720 2.069571 -0.003197
O 0.931602 -2.401574 -1.129828
O 3.358043 -1.566768 -1.595839
O 5.476329 0.135855 -1.085303
C 4.738433 -0.319622 1.138323
C 4.778576 -1.829843 1.164101
C 5.975499 -2.518096 0.564333
O 3.835446 -2.467450 1.636088
O 5.011754 2.556429 -0.057743
C 4.773247 3.950749 0.095714
C -4.584375 0.348157 0.720450
C -4.784721 1.422981 1.754276
C -5.597296 -0.326951 0.133370
C -7.085020 -0.133996 0.324652
C -7.802263 0.084622 -1.022043
C -7.375135 1.366414 -1.726852
C -7.664817 -1.359106 1.035740
H -1.576482 -2.618989 -0.760186
H -2.335259 1.933652 0.844978
H 0.052141 2.475647 0.614128
H 2.343686 3.106351 0.113824
H 1.872039 -2.495923 -1.403742
H 5.283797 0.665998 -1.878188
H 4.029770 -0.010606 1.918182
H 5.725066 0.050199 1.443634
H 5.987005 -3.565848 0.877816
H 5.926553 -2.472786 -0.525321
H 6.893262 -2.044061 0.921126
H 4.194357 4.343028 -0.746511
H 4.273564 4.159184 1.047153
H 5.742062 4.458770 0.104987
H -4.666896 2.414952 1.305925
H -5.769891 1.382165 2.225736
H -4.062767 1.319166 2.572501
H -5.346434 -1.109646 -0.586098
H -7.291950 0.740698 0.949131
H -7.628659 -0.767366 -1.691553
H -8.884834 0.132616 -0.849655
H -6.320004 1.335322 -2.014973
H -7.964737 1.507371 -2.638430
H -7.532722 2.238240 -1.083924
H -8.738179 -1.231839 1.213525
H -7.183409 -1.512625 2.008110
H -7.528344 -2.271257 0.443656
SCF Energy (B3LYP/6-31G**)= -1361.70199053
Number of imaginary frequencies = 0

1b_c059
MMFF Geometry
C -1.568382 -2.242079 -0.368473
C -2.461190 0.324551 -0.370291
N -2.900241 -2.032856 -0.505418
C -0.610824 -1.232596 -0.227013
C -1.084981 0.091915 -0.230807
C -3.334048 -0.754125 -0.497166
C -0.171395 1.151937 -0.091754
C 1.203603 0.912801 0.040756
C 1.673288 -0.402594 0.036656
C 0.771534 -1.472366 -0.083485
C 3.128060 -0.641096 0.194040
C 4.118425 0.521147 -0.076368
C 3.444391 1.869511 0.141761
C 2.117528 2.035548 0.220547
O 1.192766 -2.781321 -0.065203
O 3.492546 -1.780823 0.496887
O 4.504630 0.487903 -1.452656
C 5.334309 0.367946 0.861586

C 6.163561 -0.860562 0.542905
C 6.802110 -1.577215 1.701369
O 6.350276 -1.219954 -0.621724
O 4.257876 2.980140 0.338556
C 4.834209 3.429835 -0.890102
C -4.796755 -0.570228 -0.654540
C -5.427913 -1.475677 -1.677933
C -5.460787 0.329169 0.102798
C -6.949124 0.611636 0.106022
C -7.501569 0.415745 1.531292
C -9.015940 0.548969 1.618047
C -7.189738 2.027789 -0.421577
H -1.277001 -3.290289 -0.377047
H -2.846870 1.339095 -0.393727
H -0.540962 2.176927 -0.088354
H 1.701232 3.022905 0.397949
H 2.155742 -2.798125 0.135898
H 5.103343 -0.284112 -1.562975
H 5.000266 0.309578 1.904684
H 6.026694 1.213361 0.774789
H 7.356093 -2.446509 1.336571
H 7.493394 -0.902578 2.212082
H 6.027943 -1.919757 2.392402
H 5.658365 2.777834 -1.193738
H 4.084315 3.501562 -1.685632
H 5.244451 4.429499 -0.718795
H -5.565502 -2.482096 -1.268749
H -6.399500 -1.113493 -2.023030
H -4.799267 -1.548804 -2.573227
H -4.901861 0.914392 0.833626
H -7.476755 -0.085711 -0.551896
H -7.226512 -0.584216 1.891481
H -7.042096 1.134839 2.221462
H -9.511481 -0.133319 0.920301
H -9.356072 0.304131 2.629508
H -9.342927 1.569345 1.398379
H -8.258919 2.231182 -0.536991
H -6.727199 2.162851 -1.406095
H -6.772208 2.784162 0.252562
SCF Energy (B3LYP/6-31G**)= -1361.70697519
Number of imaginary frequencies = 0

1b_c060
MMFF Geometry
C -1.604571 -2.414122 -0.312889
C -2.369205 0.158683 0.106316
N -2.925541 -2.164613 -0.140486
C -0.597503 -1.443399 -0.294702
C -1.005766 -0.116188 -0.076076
C -3.294936 -0.881644 0.060421
C -0.039712 0.904646 -0.043274
C 1.323508 0.626941 -0.228204
C 1.729832 -0.694671 -0.441297
C 0.772861 -1.724598 -0.477454
C 3.171571 -0.991072 -0.645942
C 4.197131 0.169031 -0.621692
C 3.590961 1.535721 -0.375198
C 2.281539 1.728687 -0.176852
O 1.123758 -3.036796 -0.697501
O 3.499228 -2.156551 -0.891116
O 4.798359 0.187786 -1.934234
C 5.321595 -0.174968 0.364317
C 4.871069 -0.188755 1.808910
C 5.951573 -0.052595 2.849050
O 3.695265 -0.351738 2.132995
O 4.446798 2.614447 -0.185603
C 5.119967 3.014117 -1.378228
C -4.746791 -0.652531 0.253230
C -5.423461 -1.674429 1.126728
C -5.362444 0.387781 -0.348505
C -6.831787 0.746412 -0.293225
C -7.053287 2.111162 0.384793
C -6.649134 2.124492 1.853844
C -7.397882 0.770136 -1.716879
H -1.364676 -3.463256 -0.472504
H -2.703973 1.173597 0.297182
H -0.359431 1.931355 0.132939
H 1.912968 2.722203 0.063380
H 2.102490 -3.086478 -0.792097
H 4.075201 0.238889 -2.583235
H 6.141751 0.546626 0.261980
H 5.752811 -1.158542 0.139958
H 5.506924 -0.073794 3.847867
H 6.655638 -0.882990 2.755804
H 6.470344 0.900005 2.716197
H 5.991578 2.378678 -1.558291
H 4.449189 3.010396 -2.244351
H 5.478980 4.037206 -1.231729
H -4.805576 -1.907569 2.001890
H -6.382674 -1.329208 1.520056
H -5.598792 -2.601125 0.570167
H -4.773033 1.050534 -0.982676
H -7.401533 -0.006504 0.260129
H -6.499553 2.896183 -0.145713
H -8.115679 2.377592 0.319442
H -5.573232 1.968235 1.977276
H -6.897667 3.091817 2.302186
H -7.178615 1.346567 2.413043
H -8.471142 0.989037 -1.702716
H -7.263253 -0.199847 -2.208678
H -6.908284 1.532477 -2.333600
SCF Energy (B3LYP/6-31G**)= -1361.70137607
Number of imaginary frequencies = 0

1b_c061
MMFF Geometry
C 1.681245 2.282484 -0.245403
C 2.316567 -0.331319 -0.624512
N 2.993215 1.954868 -0.336760
C 0.621236 1.374153 -0.335188
C 0.962813 0.025340 -0.534929
C 3.297963 0.651520 -0.513905
C -0.058521 -0.934775 -0.642248
C -1.412115 -0.575001 -0.552716
C -1.751343 0.766349 -0.345704
C -0.738916 1.736916 -0.241475
C -3.183867 1.151873 -0.258606
C -4.274557 0.061755 -0.400868
C -3.736390 -1.338227 -0.616399
C -2.428566 -1.618510 -0.663315
O -1.024933 3.069913 -0.055104
O -3.459973 2.349487 -0.134898
O -5.037582 0.421897 -1.572500
C -5.238528 0.157180 0.789343
C -4.608156 -0.238659 2.106912
C -5.554602 -0.617619 3.215319
O -3.392186 -0.209502 2.293105
O -4.632595 -2.400627 -0.603387
C -5.477285 -2.437392 -1.752469
C 4.743444 0.335714 -0.607195
C 5.541755 1.319359 -1.419606
C 5.252616 -0.739348 0.031718
C 6.699808 -1.178853 0.086005
C 7.201609 -1.288258 1.538982
C 7.222598 0.049939 2.267595
C 6.841184 -2.528861 -0.621763
H 1.494792 3.343330 -0.091966
H 2.601784 -1.365241 -0.792236
H 0.210378 -1.979875 -0.793457
H -2.100912 -2.650708 -0.751824
H -2.001969 3.173577 0.008090
H -4.408050 0.526816 -2.306973
H -6.112082 -0.483319 0.614729
H -5.627000 1.177407 0.898580
H -4.986255 -0.884543 4.110602
H -6.204788 0.229563 3.446620
H -6.151609 -1.480065 2.908747
H -6.319452 -1.750284 -1.631853
H -4.923170 -2.217370 -2.671529
H -5.883539 -3.449874 -1.835559
H 5.739295 2.226896 -0.839382
H 6.500451 0.915042 -1.753531

H 5.000574 1.601391 -2.330441
H 4.582150 -1.366443 0.620202
H 7.348740 -0.466411 -0.432591
H 6.582854 -1.995294 2.105986
H 8.221042 -1.694116 1.538138
H 6.214753 0.458223 2.389325
H 7.654058 -0.073577 3.266124
H 7.829333 0.781971 1.725122
H 7.886278 -2.857020 -0.624687
H 6.511631 -2.461341 -1.664683
H 6.246398 -3.306708 -0.129416
SCF Energy (B3LYP/6-31G**)= -1361.70155858
Number of imaginary frequencies = 0

1b_c062
MMFF Geometry
C -1.453351 -2.234005 -0.649342
C -2.393058 0.193968 0.131651
N -2.788190 -2.101126 -0.456874
C -0.515426 -1.211238 -0.476797
C -1.013625 0.041509 -0.070686
C -3.245048 -0.888909 -0.076402
C -0.121662 1.113153 0.118515
C 1.255623 0.952653 -0.080679
C 1.745324 -0.298869 -0.470559
C 0.869320 -1.371672 -0.685384
C 3.200801 -0.444504 -0.686353
C 4.154665 0.585876 -0.049512
C 3.485197 1.943025 0.053652
C 2.152458 2.098533 0.077547
O 1.317505 -2.600914 -1.104591
O 3.628462 -1.380276 -1.369112
O 5.288812 0.695139 -0.927294
C 4.652084 0.156722 1.332726
C 4.939876 -1.322951 1.412950
C 6.315827 -1.793447 1.025139
O 4.056683 -2.113735 1.746874
O 4.316811 3.023945 0.305027
C 4.563613 3.772369 -0.885891
C -4.709647 -0.788789 0.129470
C -5.316899 -1.970018 0.837419
C -5.393797 0.283659 -0.323463
C -6.884561 0.530031 -0.235667
C -7.201434 1.764152 0.629114
C -6.803132 1.592204 2.089736
C -7.447097 0.720987 -1.648078
H -1.142459 -3.229866 -0.958290
H -2.798197 1.145453 0.462063
H -0.512145 2.083792 0.422621
H 1.717263 3.080863 0.236619
H 2.283805 -2.547775 -1.275818
H 5.292091 -0.110572 -1.483224
H 3.903727 0.367531 2.108413
H 5.562254 0.698541 1.617849
H 6.421671 -2.853853 1.270987
H 6.466638 -1.661348 -0.048002
H 7.068918 -1.232886 1.584443
H 5.012163 3.151350 -1.667466
H 3.641056 4.233772 -1.254012
H 5.268406 4.571914 -0.639576
H -4.686511 -2.285781 1.677032
H -6.298336 -1.750929 1.264922
H -5.427206 -2.815645 0.150389
H -4.849933 1.069526 -0.848295
H -7.402540 -0.331895 0.196159
H -6.701691 2.653379 0.224332
H -8.279483 1.964572 0.589422
H -5.719358 1.493040 2.202944
H -7.118947 2.465399 2.669639
H -7.279256 0.707480 2.524239
H -8.532931 0.862224 -1.616080
H -7.244582 -0.156264 -2.272799
H -7.009564 1.595957 -2.142249
SCF Energy (B3LYP/6-31G**)= -1361.70044279
Number of imaginary frequencies = 0

1b_c063
MMFF Geometry
C 1.575059 -2.430624 0.184738
C 2.565760 0.040713 -0.358735
N 2.920587 -2.288050 0.109549
C 0.650094 -1.397508 0.002675
C 1.174919 -0.123469 -0.281095
C 3.402587 -1.053906 -0.150984
C 0.295638 0.954465 -0.487280
C -1.093295 0.785352 -0.400213
C -1.612172 -0.477922 -0.107260
C -0.747678 -1.568592 0.080069
C -3.083468 -0.645247 -0.034376
C -3.978293 0.607133 0.154417
C -3.285237 1.853759 -0.380019
C -1.975888 1.918557 -0.655276
O -1.221489 -2.833103 0.340160
O -3.533042 -1.793603 -0.086251
O -4.172603 0.829420 1.553429
C -5.316928 0.378011 -0.578493
C -6.144559 -0.724049 0.053117
C -6.965928 -1.581203 -0.871012
O -6.183666 -0.871323 1.276704
O -4.071335 2.965702 -0.662133
C -4.454613 3.651117 0.532557
C 4.878766 -0.942864 -0.226788
C 5.552011 -2.081955 -0.944294
C 5.520335 0.103679 0.335833
C 7.014769 0.338478 0.387070
C 7.461981 1.397521 -0.639119
C 6.888609 2.794698 -0.435153
C 7.441679 0.696926 1.814016
H 1.244136 -3.443471 0.404636
H 2.991595 1.011458 -0.593163
H 0.703480 1.938559 -0.716236
H -1.547203 2.833331 -1.054109
H -2.202875 -2.820716 0.271107
H -4.781776 0.126796 1.872595
H -5.132261 0.124835 -1.629608
H -5.955206 1.268828 -0.552058
H -7.499895 -2.339911 -0.292315
H -7.692445 -0.958050 -1.397975
H -6.309330 -2.083414 -1.585756
H -5.255259 3.113407 1.048665
H -3.600275 3.807172 1.200468
H -4.843202 4.632039 0.243136
H 4.992543 -2.359659 -1.845323
H 6.560625 -1.829900 -1.282676
H 5.619952 -2.960509 -0.294163
H 4.932183 0.863432 0.850553
H 7.544973 -0.588344 0.140272
H 8.556943 1.467603 -0.621215
H 7.193240 1.056028 -1.647111
H 7.190892 3.216599 0.527580
H 7.257714 3.464784 -1.218713
H 5.795974 2.794954 -0.488386
H 8.519383 0.886736 1.859875
H 7.219976 -0.127375 2.501573
H 6.924200 1.585975 2.189857
SCF Energy (B3LYP/6-31G**)= -1361.70600046
Number of imaginary frequencies = 0

1b_c064
MMFF Geometry
C 1.989571 1.708926 -0.044491
C 2.149978 -0.992419 -0.277744
N 3.213673 1.132563 0.023646
C 0.791569 1.014433 -0.225890
C 0.886055 -0.384007 -0.345691
C 3.291395 -0.213315 -0.092538
C -0.284949 -1.141219 -0.537308
C -1.543959 -0.528708 -0.595082
C -1.630788 0.859408 -0.460090
C -0.473091 1.632461 -0.294228
C -2.971322 1.486407 -0.500204
C -4.195500 0.594495 -0.189896

C -3.972663 -0.820450 -0.712826
C -2.737915 -1.333451 -0.865897
O -0.528623 3.001562 -0.189725
O -3.039849 2.696563 -0.723115
O -5.340401 1.172391 -0.827193
C -4.419796 0.662691 1.325192
C -5.421179 -0.352286 1.828331
C -6.884004 -0.074234 1.600750
O -5.045405 -1.380482 2.396157
O -5.149287 -1.492709 -0.988199
C -5.089477 -2.894242 -1.229509
C 4.658488 -0.807716 -0.027835
C 4.861977 -2.124495 -0.727468
C 5.640732 -0.139477 0.616861
C 7.080917 -0.554624 0.820184
C 8.051214 0.393887 0.092126
C 7.907133 0.353555 -1.424113
C 7.391481 -0.570294 2.320844
H 1.998774 2.792200 0.055670
H 2.230559 -2.071588 -0.356510
H -0.207624 -2.222915 -0.643504
H -2.571712 -2.345890 -1.217546
H -1.444718 3.297300 -0.397678
H -5.267207 0.974432 -1.777248
H -4.772949 1.656943 1.626978
H -3.483877 0.491172 1.873102
H -7.481983 -0.738859 2.230891
H -7.112887 0.957533 1.879024
H -7.143542 -0.249123 0.555174
H -4.559587 -3.105707 -2.163528
H -4.626661 -3.418464 -0.387057
H -6.114860 -3.260965 -1.333695
H 4.358626 -2.131747 -1.701162
H 5.912425 -2.338790 -0.938739
H 4.467515 -2.947142 -0.122010
H 5.396214 0.823443 1.070953
H 7.254705 -1.571075 0.453834
H 7.908634 1.425688 0.437644
H 9.082572 0.117944 0.345324
H 6.927386 0.719278 -1.745786
H 8.667753 0.988403 -1.889876
H 8.038508 -0.664850 -1.803297
H 8.418252 -0.907573 2.499772
H 6.720080 -1.253414 2.853248
H 7.283201 0.425270 2.766225
SCF Energy (B3LYP/6-31G**)= -1361.70457271
Number of imaginary frequencies = 0

1b_c065
MMFF Geometry
C 1.957225 1.798558 0.161609
C 2.173416 -0.909046 0.160669
N 3.181703 1.254720 0.362065
C 0.785655 1.067377 -0.046791
C 0.909348 -0.333962 -0.047755
C 3.286557 -0.094254 0.363222
C -0.232400 -1.127767 -0.265805
C -1.492593 -0.547707 -0.463249
C -1.610072 0.844584 -0.443597
C -0.479574 1.651960 -0.255249
C -2.953264 1.438588 -0.630076
C -4.183344 0.547821 -0.340442
C -3.901128 -0.897907 -0.734758
C -2.650828 -1.395361 -0.757302
O -0.563063 3.023596 -0.263864
O -3.023435 2.626254 -0.951576
O -5.287099 1.051468 -1.101318
C -4.519244 0.726670 1.144705
C -5.537900 -0.268801 1.652088
C -6.984531 -0.041188 1.299365
O -5.187767 -1.242098 2.323603
O -5.043830 -1.613326 -1.042325
C -4.944815 -3.027339 -1.172127
C 4.647901 -0.654623 0.603575
C 4.721295 -2.030174 1.209406
C 5.733661 0.090029 0.298444
C 7.192776 -0.284105 0.434082
C 7.949154 -0.092571 -0.895219
C 7.456876 -1.016230 -2.002783
C 7.832529 0.571207 1.530376
H 1.943107 2.886326 0.174535
H 2.280369 -1.988773 0.149483
H -0.131107 -2.212576 -0.281562
H -2.443646 -2.427914 -1.016587
H -1.465733 3.283255 -0.560003
H -5.141083 0.783760 -2.025533
H -4.909901 1.733037 1.342034
H -3.623363 0.618239 1.770170
H -7.615939 -0.669868 1.933770
H -7.250399 1.003194 1.480876
H -7.163728 -0.299653 0.254256
H -4.343972 -3.296430 -2.046470
H -4.537734 -3.477839 -0.261049
H -5.953912 -3.422080 -1.322638
H 4.547450 -2.796344 0.446732
H 5.685594 -2.237422 1.680207
H 3.975037 -2.148955 2.003431
H 5.574716 1.089060 -0.113519
H 7.305385 -1.333435 0.724061
H 7.870044 0.947956 -1.234794
H 9.016434 -0.289541 -0.733678
H 6.420672 -0.798083 -2.278420
H 8.073100 -0.888987 -2.898636
H 7.519680 -2.064908 -1.695210
H 8.884390 0.299005 1.669686
H 7.322406 0.425701 2.489244
H 7.791572 1.638524 1.284684
SCF Energy (B3LYP/6-31G**)= -1361.70456179
Number of imaginary frequencies = 0

1b_c066
MMFF Geometry
C -1.543453 -1.907403 -1.083538
C -2.344840 0.610307 -0.445946
N -2.874071 -1.652299 -1.053835
C -0.543597 -0.968545 -0.811019
C -0.970545 0.332883 -0.484805
C -3.261551 -0.401117 -0.725958
C -0.013395 1.328044 -0.213996
C 1.357625 1.042606 -0.248542
C 1.774353 -0.257333 -0.556135
C 0.835900 -1.254912 -0.853798
C 3.226008 -0.535110 -0.598521
C 4.189420 0.425880 0.126389
C 3.637038 1.838519 0.126530
C 2.329681 2.110073 -0.007698
O 1.216917 -2.528938 -1.199269
O 3.643254 -1.523305 -1.210165
O 5.421655 0.411222 -0.615317
C 4.485129 -0.005729 1.564584
C 4.628154 -1.501953 1.704272
C 5.991700 -2.101000 1.487744
O 3.645295 -2.203241 1.947250
O 4.528108 2.849613 0.453400
C 4.975257 3.539949 -0.714010
C -4.725050 -0.165350 -0.706491
C -5.472766 -0.815482 -1.839337
C -5.290584 0.552517 0.287527
C -6.761487 0.845996 0.489990
C -7.247481 0.364636 1.870943
C -7.174054 -1.148277 2.037646
C -6.997342 2.351624 0.345444
H -1.289862 -2.932862 -1.344236
H -2.697024 1.610493 -0.213194
H -0.347324 2.337246 0.024965
H 1.969082 3.130683 0.081746
H 2.197417 -2.565447 -1.255581
H 5.416138 -0.407070 -1.152539
H 3.674926 0.291098 2.244021
H 5.401598 0.462758 1.943895
H 5.973325 -3.159226 1.763288
H 6.275423 -2.012858 0.437241

H 6.723086 -1.592439 2.120702
H 5.452368 2.860492 -1.427052
H 4.145864 4.069485 -1.194858
H 5.716324 4.281623 -0.401892
H -5.605614 -1.885656 -1.648598
H -6.459235 -0.375475 -2.004733
H -4.928349 -0.696789 -2.783564
H -4.652051 0.950636 1.076634
H -7.372334 0.343573 -0.266358
H -6.665708 0.841795 2.669761
H -8.290161 0.675036 2.013894
H -6.140989 -1.507457 2.005501
H -7.597474 -1.439953 3.004142
H -7.741917 -1.657045 1.252177
H -8.060804 2.589470 0.456780
H -6.679770 2.705349 -0.641911
H -6.444993 2.920915 1.101777
SCF Energy (B3LYP/6-31G**)= -1361.70053407
Number of imaginary frequencies = 0

1b_c067
MMFF Geometry
C -1.746849 -1.991438 -0.122657
C -2.151715 0.661579 -0.531640
N -3.023866 -1.539035 -0.158095
C -0.611759 -1.192377 -0.282721
C -0.833488 0.178812 -0.493368
C -3.221277 -0.216376 -0.361778
C 0.269317 1.035759 -0.662066
C 1.586566 0.551627 -0.621899
C 1.801748 -0.812689 -0.406345
C 0.710018 -1.682680 -0.244365
C 3.190580 -1.324671 -0.368431
C 4.366650 -0.336774 -0.443516
C 3.980213 1.100761 -0.732725
C 2.695587 1.490602 -0.813979
O 0.879514 -3.033920 -0.048570
O 3.382443 -2.545388 -0.316314
O 5.141832 -0.803294 -1.573815
C 5.257243 -0.482208 0.797115
C 4.635733 0.112772 2.039513
C 5.581430 0.677772 3.065532
O 3.421093 0.090960 2.235805
O 5.074242 1.939169 -0.870869
C 4.851984 3.284629 -1.274961
C -4.639577 0.244600 -0.409910
C -4.924814 1.438983 -1.276583
C -5.574824 -0.438600 0.287194
C -7.060401 -0.195865 0.449069
C -7.473641 1.220136 0.895572
C -6.806664 1.664092 2.191427
C -7.835225 -0.637785 -0.795038
H -1.656271 -3.062401 0.045444
H -2.331964 1.721230 -0.679330
H 0.092672 2.098327 -0.825108
H 2.423319 2.521840 -1.007566
H 1.841677 -3.235125 -0.080647
H 5.226838 -1.770447 -1.468907
H 6.223163 0.006871 0.617008
H 5.486202 -1.534028 1.007459
H 5.013479 1.074927 3.911386
H 6.247022 -0.111495 3.423268
H 6.161864 1.489910 2.620745
H 4.341856 3.325292 -2.242807
H 4.291711 3.833331 -0.511281
H 5.827341 3.767242 -1.387327
H -4.353207 1.392459 -2.210751
H -5.971750 1.502679 -1.577840
H -4.666146 2.365719 -0.753977
H -5.239597 -1.310776 0.854504
H -7.380775 -0.868852 1.258421
H -7.273436 1.961943 0.116759
H -8.560113 1.234521 1.051873
H -5.724597 1.771993 2.072044
H -7.204208 2.635249 2.503559
H -6.997041 0.947277 2.996504
H -8.913816 -0.612674 -0.604396
H -7.573997 -1.663898 -1.077301
H -7.637167 0.007945 -1.655600
SCF Energy (B3LYP/6-31G**)= -1361.70459102
Number of imaginary frequencies = 0

1b_c068
MMFF Geometry
C -1.566396 -2.430162 -0.339780
C -2.323309 0.142525 0.093879
N -2.885778 -2.178741 -0.158135
C -0.557323 -1.461501 -0.324188
C -0.961555 -0.134195 -0.098138
C -3.251318 -0.895881 0.050071
C 0.006418 0.885073 -0.068913
C 1.368086 0.605372 -0.261845
C 1.770475 -0.717397 -0.480104
C 0.811141 -1.744917 -0.516891
C 3.211156 -1.017509 -0.689836
C 4.247061 0.126668 -0.584991
C 3.640163 1.498514 -0.393263
C 2.327558 1.707833 -0.225764
O 1.157858 -3.056550 -0.746715
O 3.530077 -2.171920 -0.991206
O 4.963026 0.151218 -1.838851
C 5.289970 -0.223440 0.483911
C 4.745120 -0.184853 1.893983
C 5.746252 0.043482 2.995439
O 3.557082 -0.382484 2.146743
O 4.541334 2.545645 -0.263768
C 4.584505 3.355246 -1.438814
C -4.701441 -0.664688 0.253087
C -5.374242 -1.688141 1.127763
C -5.319085 0.378626 -0.341360
C -6.787402 0.739658 -0.275385
C -7.002049 2.102684 0.408281
C -6.588303 2.110743 1.874697
C -7.362724 0.768795 -1.695234
H -1.329544 -3.479154 -0.504810
H -2.655065 1.157279 0.290620
H -0.311039 1.911757 0.110923
H 1.955520 2.707939 -0.023400
H 2.133565 -3.105068 -0.868509
H 4.301033 0.170113 -2.550722
H 6.135377 0.473623 0.417665
H 5.707250 -1.223877 0.314732
H 5.235403 0.051990 3.962271
H 6.485019 -0.761600 2.990786
H 6.237501 1.008769 2.850065
H 4.847420 2.763324 -2.321196
H 3.630232 3.868293 -1.597317
H 5.358534 4.114707 -1.294825
H -4.751006 -1.925280 1.998043
H -6.330160 -1.342376 1.528565
H -5.555026 -2.612697 0.569391
H -4.732614 1.042218 -0.977350
H -7.354863 -0.013970 0.279347
H -6.450389 2.888373 -0.123398
H -8.064369 2.371184 0.350687
H -5.511899 1.952209 1.990623
H -6.832185 3.077109 2.327640
H -7.115513 1.332021 2.434932
H -8.435478 0.989536 -1.673391
H -7.233028 -0.199892 -2.190898
H -6.875804 1.532184 -2.312776
SCF Energy (B3LYP/6-31G**)= -1361.70109292
Number of imaginary frequencies = 0

1b_c069
MMFF Geometry
C -1.829048 -1.626334 0.031787
C -2.051267 1.066939 0.284703
N -3.058917 -1.105865 0.262622
C -0.655561 -0.877842 -0.090209
C -0.783168 0.514435 0.040091
C -3.166134 0.236549 0.390929

C 0.361599 1.322686 -0.079778
C 1.629234 0.768935 -0.323390
C 1.754032 -0.617799 -0.445262
C 0.616326 -1.437393 -0.333377
C 3.091034 -1.208208 -0.699352
C 4.322980 -0.280453 -0.794291
C 4.023555 1.204252 -0.662360
C 2.779945 1.666510 -0.447305
O 0.689356 -2.805593 -0.461099
O 3.167091 -2.432107 -0.849658
O 4.875806 -0.486012 -2.113369
C 5.394649 -0.772622 0.188573
C 5.009979 -0.617819 1.643450
C 6.100627 -0.873246 2.650753
O 3.869789 -0.329858 2.005009
O 5.151174 1.996327 -0.793900
C 5.017226 3.406495 -0.660832
C -4.532653 0.770999 0.664176
C -4.611885 2.076283 1.408957
C -5.610488 0.063080 0.258539
C -7.075651 0.405724 0.412706
C -7.757359 0.640174 -0.951509
C -7.834347 -0.569965 -1.874484
C -7.778818 -0.676054 1.236082
H -1.811372 -2.710527 -0.056328
H -2.158682 2.142530 0.376883
H 0.257649 2.402386 0.022837
H 2.573822 2.726470 -0.348970
H 1.628443 -3.052823 -0.625385
H 4.323045 0.010139 -2.740196
H 6.334480 -0.231062 0.021728
H 5.617633 -1.833017 0.014652
H 5.710050 -0.729287 3.661966
H 6.456952 -1.901444 2.551498
H 6.921914 -0.171045 2.487812
H 4.367448 3.812806 -1.442487
H 4.648701 3.672059 0.335203
H 6.009267 3.851269 -0.782671
H -4.410828 2.916030 0.735567
H -5.585419 2.247660 1.873732
H -3.885450 2.102748 2.229448
H -5.432212 -0.884335 -0.254035
H -7.189684 1.342547 0.966683
H -8.778877 1.000829 -0.775320
H -7.234459 1.447999 -1.479657
H -8.407455 -1.384694 -1.422867
H -8.336768 -0.292378 -2.807166
H -6.840716 -0.944732 -2.135676
H -8.848543 -0.459896 1.328185
H -7.361152 -0.722581 2.248382
H -7.669941 -1.670600 0.790365
SCF Energy (B3LYP/6-31G**)= -1361.70704325
Number of imaginary frequencies = 0

1b_c070
MMFF Geometry
C 1.762460 1.960836 0.026965
C 2.180752 -0.654141 -0.570733
N 3.042114 1.522844 -0.059065
C 0.630890 1.165453 -0.171406
C 0.859829 -0.186239 -0.480068
C 3.246247 0.218979 -0.356383
C -0.238358 -1.039067 -0.691921
C -1.556954 -0.568501 -0.600287
C -1.781303 0.776078 -0.285778
C -0.693813 1.641384 -0.079271
C -3.175507 1.274157 -0.195025
C -4.351280 0.288314 -0.333252
C -3.939535 -1.108554 -0.742684
C -2.661359 -1.497211 -0.834723
O -0.869940 2.974293 0.212381
O -3.376155 2.486995 -0.057836
O -5.145794 0.853852 -1.400842
C -5.215011 0.308805 0.932596
C -4.549161 -0.392012 2.093885
C -5.418192 -1.273688 2.949493
O -3.359479 -0.216329 2.355609
O -4.932894 -2.074193 -0.854250
C -5.701516 -1.922863 -2.047977
C 4.667133 -0.225546 -0.457044
C 4.947485 -1.357532 -1.405270
C 5.608323 0.417812 0.269386
C 7.098031 0.177386 0.391930
C 7.528662 -1.261408 0.737798
C 6.885663 -1.795121 2.011729
C 7.849721 0.706639 -0.832123
H 1.666707 3.016689 0.270698
H 2.366215 -1.699660 -0.793773
H -0.058550 -2.086904 -0.930153
H -2.426110 -2.532575 -1.064003
H -1.834025 3.167708 0.218634
H -5.204477 1.812859 -1.217356
H -6.180932 -0.171319 0.730497
H -5.450402 1.332702 1.247346
H -4.818052 -1.720544 3.746907
H -6.216255 -0.676617 3.397298
H -5.841785 -2.074447 2.338224
H -6.499687 -1.190805 -1.896362
H -5.078715 -1.648144 -2.906570
H -6.171978 -2.886553 -2.265152
H 4.361585 -1.253577 -2.325856
H 5.990160 -1.392940 -1.725342
H 4.703129 -2.318912 -0.941691
H 5.275718 1.247391 0.898715
H 7.426337 0.798357 1.238817
H 7.321486 -1.952069 -0.084986
H 8.617562 -1.276982 0.876044
H 5.802650 -1.903960 1.902179
H 7.295212 -2.781345 2.253072
H 7.083590 -1.131208 2.859232
H 8.931357 0.678134 -0.660118
H 7.576470 1.746853 -1.042143
H 7.642834 0.117245 -1.730213
SCF Energy (B3LYP/6-31G**)= -1361.69897236
Number of imaginary frequencies = 0

1b_c071
MMFF Geometry
C 1.844195 -1.547277 -0.113138
C 2.296706 1.129331 -0.029022
N 3.128110 -1.117372 -0.060789
C 0.724418 -0.712652 -0.128175
C 0.970288 0.671677 -0.086971
C 3.350605 0.216524 -0.016260
C -0.115891 1.566461 -0.113684
C -1.437659 1.102669 -0.162209
C -1.673828 -0.273148 -0.184378
C -0.604108 -1.179524 -0.187356
C -3.075839 -0.738104 -0.236126
C -4.175654 0.217024 0.288177
C -3.821840 1.664905 -0.065056
C -2.546438 2.055518 -0.253613
O -0.807620 -2.537388 -0.250740
O -3.291409 -1.877709 -0.654688
O -4.210776 0.167513 1.717950
C -5.537451 -0.190998 -0.310808
C -5.981469 -1.565287 0.150788
C -6.793578 -2.383324 -0.816066
O -5.735625 -1.968183 1.290063
O -4.918531 2.508931 -0.120135
C -4.696663 3.908437 -0.247201
C 4.776206 0.649800 0.060591
C 5.048594 1.978883 0.711773
C 5.742127 -0.164857 -0.419173
C 7.235201 0.073157 -0.459421
C 7.991864 -0.927369 0.433676
C 7.672721 -0.771586 1.915312
C 7.723164 -0.044659 -1.907404
H 1.735244 -2.629284 -0.143202
H 2.495523 2.195696 -0.007895
H 0.076792 2.638444 -0.095464
H -2.282599 3.083262 -0.474621

H -1.763974 -2.703463 -0.410454
H -4.612121 -0.694393 1.964747
H -5.485926 -0.184487 -1.406198
H -6.338153 0.490122 0.001399
H -7.036193 -3.350443 -0.367204
H -7.721541 -1.856465 -1.050890
H -6.215175 -2.555131 -1.727175
H -4.094628 4.287954 0.584739
H -4.231148 4.143527 -1.209580
H -5.669833 4.407155 -0.214132
H 4.433727 2.107650 1.610025
H 6.083207 2.086809 1.046424
H 4.833359 2.798592 0.018474
H 5.440821 -1.122730 -0.849164
H 7.482922 1.085912 -0.126849
H 7.770523 -1.957407 0.126500
H 9.071527 -0.785081 0.299383
H 6.625068 -1.004102 2.128602
H 8.291324 -1.455068 2.505813
H 7.877433 0.248612 2.255127
H 8.796680 0.163751 -1.971903
H 7.206245 0.672386 -2.555114
H 7.551795 -1.049140 -2.310999
SCF Energy (B3LYP/6-31G**)= -1361.71429488
Number of imaginary frequencies = 0

1b_c072
MMFF Geometry
C -1.810147 -1.699564 -0.435609
C -2.084678 0.973152 -0.046953
N -3.060718 -1.203977 -0.270974
C -0.640258 -0.935561 -0.422705
C -0.794758 0.446439 -0.222946
C -3.193083 0.128034 -0.075370
C 0.345209 1.270590 -0.206987
C 1.634244 0.741951 -0.380310
C 1.783002 -0.634950 -0.570419
C 0.652919 -1.469854 -0.599274
C 3.141906 -1.192621 -0.756895
C 4.366271 -0.272918 -0.616282
C 4.047525 1.202136 -0.467717
C 2.783990 1.650971 -0.363409
O 0.755845 -2.826477 -0.803948
O 3.268409 -2.384509 -1.061977
O 5.066891 -0.431076 -1.873370
C 5.298535 -0.800452 0.481906
C 4.763457 -0.556998 1.874133
C 5.781172 -0.346407 2.963270
O 3.558736 -0.582812 2.123832
O 5.179108 2.000085 -0.426504
C 5.014101 3.412789 -0.425158
C -4.582165 0.635952 0.122946
C -4.729589 1.854586 0.990428
C -5.611198 -0.031779 -0.444906
C -7.098717 0.249905 -0.434052
C -7.790199 -0.092091 0.898735
C -7.614813 -1.547342 1.315340
C -7.454343 1.664650 -0.902051
H -1.771850 -2.776478 -0.584629
H -2.214182 2.040679 0.097031
H 0.220524 2.341962 -0.053843
H 2.560822 2.705004 -0.243952
H 1.703284 -3.049264 -0.945347
H 5.102294 -1.390865 -2.049928
H 6.281567 -0.320474 0.392958
H 5.477722 -1.877239 0.375073
H 5.272028 -0.182194 3.916988
H 6.415939 -1.231855 3.046835
H 6.387052 0.533545 2.733270
H 4.466202 3.745489 -1.312700
H 4.517875 3.746227 0.491772
H 6.008830 3.867332 -0.452870
H -4.494194 2.761982 0.424652
H -5.735398 1.965102 1.398802
H -4.063603 1.800910 1.859422
H -5.369160 -0.924498 -1.027483
H -7.528965 -0.420493 -1.192979
H -7.426486 0.553180 1.705476
H -8.864891 0.108541 0.803544
H -6.567240 -1.781649 1.527552
H -8.190834 -1.748957 2.224252
H -7.970398 -2.225178 0.532851
H -8.527824 1.734063 -1.111690
H -6.921640 1.921268 -1.824353
H -7.224676 2.426311 -0.152436
SCF Energy (B3LYP/6-31G**)= -1361.70464295
Number of imaginary frequencies = 0

1b_c073
MMFF Geometry
C 1.645785 2.298363 -0.298475
C 2.269805 -0.324820 -0.628256
N 2.956433 1.963124 -0.380950
C 0.581786 1.393417 -0.373887
C 0.917486 0.039526 -0.548313
C 3.255491 0.655387 -0.533347
C -0.107974 -0.917778 -0.641845
C -1.460054 -0.550585 -0.560439
C -1.793149 0.796386 -0.376130
C -0.776727 1.764368 -0.290097
C -3.224120 1.190319 -0.293902
C -4.315657 0.094839 -0.335094
C -3.787203 -1.299574 -0.586847
C -2.481988 -1.590479 -0.668115
O -1.057130 3.102128 -0.131374
O -3.497314 2.393662 -0.243552
O -5.188058 0.423132 -1.438075
C -5.183893 0.189708 0.925834
C -4.464982 -0.233887 2.186956
C -5.328825 -0.703067 3.327693
O -3.242974 -0.153060 2.307492
O -4.735648 -2.312305 -0.599785
C -4.986055 -2.786293 -1.922923
C 4.699676 0.331238 -0.617549
C 5.504237 1.295805 -1.446503
C 5.202496 -0.733914 0.042636
C 6.647541 -1.178873 0.108471
C 7.145746 -1.262913 1.564370
C 7.171328 0.088785 2.267467
C 6.784225 -2.542724 -0.573201
H 1.463891 3.362681 -0.164852
H 2.550788 -1.362856 -0.776418
H 0.156986 -1.966158 -0.775627
H -2.158195 -2.620905 -0.781916
H -2.034476 3.214107 -0.093242
H -4.627079 0.565250 -2.219386
H -6.077618 -0.436512 0.807087
H -5.548195 1.213406 1.077322
H -4.698926 -0.981316 4.177169
H -6.001238 0.102471 3.632529
H -5.903882 -1.578549 3.016122
H -5.316805 -1.976181 -2.580521
H -4.098763 -3.274208 -2.339601
H -5.787353 -3.528899 -1.867339
H 5.704749 2.213151 -0.882964
H 6.461754 0.880898 -1.770683
H 4.966285 1.563181 -2.363649
H 4.527882 -1.346610 0.641450
H 7.300833 -0.479382 -0.422142
H 6.522550 -1.956220 2.143337
H 8.163305 -1.673346 1.573494
H 6.165110 0.503886 2.379192
H 7.600098 -0.017684 3.269112
H 7.782561 0.807606 1.712521
H 7.827807 -2.875626 -0.567578
H 6.457201 -2.493548 -1.617941
H 6.184834 -3.308357 -0.067477
SCF Energy (B3LYP/6-31G**)= -1361.70123404
Number of imaginary frequencies = 0

1b_c074
MMFF Geometry

C -1.318361 -2.312030 -0.536954
C -2.268994 0.140344 0.149108
N -2.649428 -2.185095 -0.315992
C -0.389903 -1.271019 -0.439376
C -0.893235 -0.005786 -0.081943
C -3.112112 -0.961300 0.017153
C -0.010123 1.084321 0.029247
C 1.364319 0.929982 -0.196412
C 1.858489 -0.333030 -0.536002
C 0.990571 -1.424916 -0.676736
C 3.311449 -0.473277 -0.770300
C 4.248898 0.582506 -0.138147
C 3.588538 1.959807 -0.129388
C 2.249294 2.098287 -0.139950
O 1.444341 -2.666727 -1.050346
O 3.710360 -1.433120 -1.431978
O 5.452826 0.650764 -0.913826
C 4.649892 0.197087 1.287574
C 4.954764 -1.276937 1.419547
C 6.294616 -1.769795 0.942120
O 4.107923 -2.047143 1.876140
O 4.495775 3.002161 -0.085770
C 4.004258 4.336729 -0.041782
C -4.572299 -0.869208 0.255853
C -5.147577 -2.024970 1.029843
C -5.283245 0.173032 -0.225464
C -6.777490 0.395985 -0.113913
C -7.043177 1.596434 0.812300
C -8.521443 1.831771 1.090731
C -7.361265 0.600717 -1.515300
H -1.002343 -3.318174 -0.804885
H -2.677925 1.102015 0.442978
H -0.407126 2.064133 0.292321
H 1.767091 3.069364 -0.112595
H 2.403314 -2.604185 -1.262113
H 5.227236 1.092836 -1.750766
H 3.843712 0.416447 2.000191
H 5.531051 0.756308 1.625592
H 6.469040 -2.778421 1.327396
H 6.316752 -1.796454 -0.149025
H 7.085865 -1.117560 1.320029
H 3.426315 4.569983 -0.941733
H 3.409449 4.504374 0.861803
H 4.864877 5.011302 -0.009217
H -4.485149 -2.308518 1.856200
H -6.112321 -1.789832 1.486605
H -5.281281 -2.895583 0.379200
H -4.764586 0.945016 -0.794407
H -7.268507 -0.485665 0.309996
H -6.538468 1.430966 1.773131
H -6.611699 2.510830 0.385377
H -8.994913 0.929307 1.489897
H -8.640862 2.631272 1.829121
H -9.057386 2.135289 0.186840
H -8.454468 0.645797 -1.485575
H -7.089689 -0.229273 -2.177731
H -6.996986 1.528212 -1.971299
SCF Energy (B3LYP/6-31G**)= -1361.69985479
Number of imaginary frequencies = 0

1b_c075
MMFF Geometry
C -1.659156 -2.547993 0.094942
C -2.353438 0.079629 0.127236
N -2.965054 -2.237984 0.279194
C -0.633358 -1.613699 -0.079488
C -1.004481 -0.256269 -0.059534
C -3.300764 -0.929982 0.285115
C -0.018351 0.732617 -0.231377
C 1.329278 0.391214 -0.410543
C 1.692181 -0.957373 -0.417816
C 0.720257 -1.957561 -0.269009
C 3.119621 -1.299271 -0.589649
C 4.183282 -0.211987 -0.346319
C 3.642043 1.179358 -0.638221
C 2.322041 1.442563 -0.643068
O 1.046062 -3.292464 -0.296981
O 3.433029 -2.460259 -0.872207
O 5.257303 -0.513902 -1.252638
C 4.683005 -0.395061 1.091168
C 5.574653 0.724558 1.580657
C 6.984021 0.788653 1.051727
O 5.159762 1.545620 2.401661
O 4.633300 2.117049 -0.865121
C 4.265997 3.479522 -1.039274
C -4.738769 -0.636225 0.493137
C -5.401900 -1.492849 1.538162
C -5.359130 0.313705 -0.239084
C -6.821748 0.705084 -0.187544
C -6.946937 2.122219 0.400326
C -8.388087 2.566403 0.610001
C -7.419443 0.602780 -1.594275
H -1.448990 -3.615482 0.092597
H -2.660521 1.120383 0.163222
H -0.311692 1.782106 -0.226339
H 1.935967 2.438366 -0.831482
H 1.987223 -3.384095 -0.567539
H 5.264766 -1.485049 -1.367862
H 5.244858 -1.331865 1.197112
H 3.838068 -0.462869 1.789499
H 7.556952 1.517344 1.632454
H 7.462016 -0.188287 1.159125
H 6.986052 1.095362 0.004673
H 3.654399 3.607264 -1.938033
H 3.748443 3.861766 -0.153549
H 5.183344 4.060897 -1.170674
H -4.748497 -1.620153 2.409330
H -6.327540 -1.054344 1.919675
H -5.635133 -2.483097 1.132956
H -4.784149 0.860100 -0.987196
H -7.383844 0.013882 0.448372
H -6.435611 2.157832 1.371194
H -6.441016 2.850046 -0.246873
H -8.935367 1.847149 1.227338
H -8.411062 3.535481 1.118962
H -8.915775 2.681184 -0.341182
H -8.502145 0.762300 -1.576468
H -7.245996 -0.391375 -2.022088
H -6.979908 1.342479 -2.272902
SCF Energy (B3LYP/6-31G**)= -1361.70844696
Number of imaginary frequencies = 0

1b_c076
MMFF Geometry
C -1.811207 -1.507298 -0.265021
C -2.273497 1.132597 0.170925
N -3.096595 -1.079198 -0.239472
C -0.694187 -0.688604 -0.082321
C -0.945721 0.675740 0.144552
C -3.324094 0.237062 -0.024199
C 0.136974 1.550777 0.345995
C 1.460615 1.089843 0.310828
C 1.707813 -0.264271 0.074667
C 0.637051 -1.153966 -0.107912
C 3.111611 -0.740661 0.056123
C 4.256194 0.287072 -0.140051
C 3.828832 1.670911 0.331580
C 2.556062 2.020418 0.560174
O 0.838464 -2.498603 -0.313816
O 3.306137 -1.955325 0.157431
O 4.531064 0.410990 -1.537730
C 5.495414 -0.191636 0.645308
C 6.087769 -1.466042 0.076588
C 6.682563 -2.442586 1.054316
O 6.128818 -1.663214 -1.139891
O 4.824689 2.602141 0.605561
C 5.377326 3.145008 -0.596049
C -4.750826 0.673030 -0.020317
C -5.027023 2.104940 -0.392372
C -5.717546 -0.222841 0.279122
C -7.211223 0.008106 0.362200
C -7.899865 -0.679413 -0.830313

C -9.395267 -0.403909 -0.907989
C -7.725695 -0.509131 1.709464
H -1.698657 -2.574295 -0.444940
H -2.475666 2.182189 0.357295
H -0.058400 2.606660 0.530720
H 2.320487 3.019285 0.915697
H 1.797907 -2.691314 -0.212138
H 4.986116 -0.415623 -1.813944
H 5.232686 -0.360978 1.696660
H 6.308377 0.543038 0.613792
H 7.059349 -3.317698 0.517791
H 7.509763 -1.967814 1.587389
H 5.914733 -2.768218 1.760430
H 6.060147 2.431400 -1.066206
H 4.594227 3.451486 -1.298317
H 5.956424 4.032613 -0.324428
H -4.403015 2.417606 -1.237559
H -6.058929 2.269443 -0.713388
H -4.827969 2.768493 0.455522
H -5.418251 -1.249165 0.503091
H -7.437254 1.078680 0.327655
H -7.441031 -0.329637 -1.764342
H -7.739643 -1.764419 -0.789928
H -9.596832 0.671827 -0.918671
H -9.808287 -0.834593 -1.825925
H -9.930541 -0.849995 -0.064859
H -8.778805 -0.249623 1.856594
H -7.164213 -0.065327 2.539674
H -7.632373 -1.598429 1.784395
SCF Energy (B3LYP/6-31G**)= -1361.70792753
Number of imaginary frequencies = 0

1b_c077
MMFF Geometry
C -1.824173 -1.636788 -0.538989
C -2.116117 0.998004 0.046803
N -3.079379 -1.158469 -0.359081
C -0.657672 -0.872944 -0.448238
C -0.821416 0.489813 -0.146835
C -3.220426 0.154456 -0.064801
C 0.314087 1.313973 -0.048823
C 1.606435 0.802844 -0.241396
C 1.766389 -0.556271 -0.531857
C 0.640511 -1.389442 -0.642608
C 3.132626 -1.094730 -0.740584
C 4.356018 -0.183988 -0.523177
C 4.008342 1.269092 -0.286426
C 2.751610 1.704908 -0.131651
O 0.752019 -2.726283 -0.947817
O 3.269512 -2.259012 -1.135258
O 5.075349 -0.276036 -1.774148
C 5.265641 -0.764814 0.564960
C 4.686465 -0.593702 1.950035
C 5.637537 -0.187097 3.043102
O 3.499379 -0.818983 2.184114
O 5.049722 2.156796 -0.043192
C 5.760189 2.495097 -1.234612
C -4.614751 0.641942 0.147877
C -4.781368 1.791882 1.101466
C -5.631651 0.015551 -0.485217
C -7.120299 0.291297 -0.477845
C -7.831877 -0.151053 0.814214
C -7.657176 -1.632761 1.124211
C -7.474149 1.735915 -0.845084
H -1.779146 -2.699255 -0.768179
H -2.252033 2.051143 0.269590
H 0.184738 2.370450 0.183923
H 2.566561 2.748361 0.106776
H 1.703156 -2.938067 -1.079701
H 5.086446 -1.224717 -2.012226
H 6.248668 -0.278552 0.525182
H 5.454538 -1.833803 0.408686
H 5.095347 -0.095489 3.988276
H 6.415720 -0.946392 3.151873
H 6.083739 0.780231 2.799223
H 6.521011 1.740006 -1.451310
H 5.089014 2.628755 -2.090296
H 6.275449 3.443802 -1.056670
H -4.129916 1.674972 1.975257
H -4.539955 2.739674 0.609234
H -5.794306 1.869030 1.499743
H -5.376480 -0.830361 -1.128797
H -7.535435 -0.321976 -1.291528
H -7.484031 0.433421 1.672632
H -8.905714 0.052914 0.716495
H -6.612212 -1.879103 1.335656
H -8.247025 -1.903216 2.005972
H -7.997166 -2.251496 0.287692
H -8.544386 1.817627 -1.066542
H -6.927702 2.062013 -1.736893
H -7.259726 2.440335 -0.037242
SCF Energy (B3LYP/6-31G**)= -1361.69898214
Number of imaginary frequencies = 0

1b_c078
MMFF Geometry
C 1.714049 2.491655 0.257803
C 2.336297 -0.006354 -0.614042
N 3.024257 2.165522 0.146297
C 0.649567 1.635746 -0.042049
C 0.983844 0.346008 -0.495484
C 3.322711 0.918969 -0.278688
C -0.042354 -0.557688 -0.827288
C -1.392281 -0.203077 -0.698256
C -1.716897 1.073139 -0.233107
C -0.707705 1.995757 0.079004
C -3.144837 1.424035 -0.085195
C -4.191485 0.294618 -0.043321
C -3.733997 -0.924774 -0.829358
C -2.435318 -1.147773 -1.104000
O -0.998867 3.267382 0.511535
O -3.464487 2.608978 0.055034
O -5.375279 0.843441 -0.646227
C -4.476593 0.009309 1.435497
C -5.317202 -1.225555 1.673500
C -6.788910 -1.150260 1.359512
O -4.810103 -2.253434 2.128086
O -4.771859 -1.763167 -1.194422
C -4.469107 -2.994642 -1.837371
C 4.767522 0.605624 -0.389039
C 5.600429 1.718368 -0.966589
C 5.245855 -0.583807 0.035062
C 6.687749 -1.048634 0.040654
C 7.090326 -1.442461 1.475155
C 8.563447 -1.801835 1.614281
C 6.842410 -2.208087 -0.946003
H 1.533924 3.505106 0.610195
H 2.618025 -0.989642 -0.978122
H 0.220983 -1.550476 -1.191329
H -2.106125 -2.026870 -1.647140
H -1.966345 3.419865 0.421495
H -5.372979 1.801778 -0.451174
H -4.993903 0.854283 1.907625
H -3.539416 -0.126782 1.991695
H -7.291020 -2.036476 1.758246
H -7.221841 -0.266853 1.835524
H -6.948463 -1.112592 0.280845
H -3.994314 -2.821825 -2.808373
H -3.841850 -3.625520 -1.199389
H -5.410899 -3.523075 -2.012231
H 5.791685 2.487880 -0.211267
H 6.563315 1.372237 -1.350376
H 5.089150 2.186439 -1.816057
H 4.550767 -1.306994 0.462794
H 7.355196 -0.243206 -0.280811
H 6.878596 -0.605150 2.152942
H 6.484342 -2.288622 1.823496
H 9.202212 -0.992005 1.248107
H 8.808560 -1.976241 2.666987
H 8.808334 -2.714431 1.063337
H 7.891615 -2.501100 -1.051593
H 6.484363 -1.924823 -1.942509

H 6.275926 -3.088844 -0.623113
SCF Energy (B3LYP/6-31G**)= -1361.70851116
Number of imaginary frequencies = 0

1b_c079
MMFF Geometry
C 1.813548 -1.642761 -0.432996
C 2.327175 1.023823 -0.396836
N 3.106831 -1.240268 -0.470855
C 0.713243 -0.784391 -0.376537
C 0.990588 0.594453 -0.358629
C 3.360026 0.088753 -0.451890
C -0.075583 1.512096 -0.309789
C -1.407001 1.076492 -0.263331
C -1.673196 -0.294001 -0.265300
C -0.625718 -1.222954 -0.340650
C -3.084845 -0.729182 -0.216895
C -4.124753 0.249595 0.380861
C -3.765941 1.689414 0.000752
C -2.499034 2.052652 -0.278129
O -0.861792 -2.576318 -0.384327
O -3.353244 -1.864497 -0.616148
O -4.060954 0.201733 1.809680
C -5.533431 -0.129655 -0.120712
C -5.973336 -1.493798 0.373769
C -6.868179 -2.294945 -0.532285
O -5.657242 -1.901070 1.494001
O -4.845547 2.556699 0.021734
C -4.603213 3.951264 -0.121259
C 4.795279 0.492331 -0.507671
C 5.100252 1.848115 -1.084872
C 5.742051 -0.367244 -0.070244
C 7.238846 -0.159473 -0.009763
C 7.787740 -0.426516 1.405600
C 7.260381 0.552980 2.447071
C 7.917627 -1.087504 -1.020079
H 1.679979 -2.722296 -0.449568
H 2.550157 2.085257 -0.369272
H 0.140550 2.579752 -0.307776
H -2.229733 3.074480 -0.519066
H -1.830251 -2.722322 -0.476372
H -4.462260 -0.651256 2.085765
H -5.558378 -0.125028 -1.217040
H -6.295710 0.568658 0.245089
H -7.099444 -3.256325 -0.065463
H -7.798823 -1.748501 -0.702982
H -6.358549 -2.479753 -1.481132
H -3.936101 4.316930 0.666050
H -4.201676 4.177338 -1.114134
H -5.560724 4.470680 -0.019993
H 4.911160 2.632800 -0.344981
H 6.135830 1.945457 -1.420330
H 4.484889 2.044294 -1.970492
H 5.419952 -1.339804 0.308591
H 7.505171 0.868878 -0.273175
H 7.548981 -1.449627 1.722625
H 8.882256 -0.351136 1.387344
H 6.179115 0.453740 2.582342
H 7.735401 0.362943 3.414912
H 7.479764 1.586205 2.159691
H 9.002434 -0.935521 -1.017643
H 7.557473 -0.891551 -2.036306
H 7.725784 -2.142058 -0.791343
SCF Energy (B3LYP/6-31G**)= -1361.71436291
Number of imaginary frequencies = 0

1b_c080
MMFF Geometry
C -1.732245 -1.403086 -0.647616
C -2.122542 1.157109 0.170619
N -3.002979 -0.990720 -0.421025
C -0.596273 -0.605607 -0.491900
C -0.809770 0.718415 -0.067169
C -3.194730 0.284881 -0.013112
C 0.293938 1.575392 0.103438
C 1.602295 1.130101 -0.129431
C 1.804659 -0.191316 -0.537008
C 0.717630 -1.052752 -0.736481
C 3.189348 -0.635391 -0.785943
C 4.344579 0.154174 -0.140635
C 4.002507 1.633623 -0.008773
C 2.726109 2.063604 0.003613
O 0.886858 -2.343546 -1.175029
O 3.396746 -1.623332 -1.496322
O 5.478525 0.019600 -1.017021
C 4.736655 -0.396925 1.232402
C 4.702778 -1.905314 1.282236
C 5.931911 -2.652558 0.839460
O 3.680498 -2.494420 1.636145
O 5.113817 2.443479 0.142798
C 4.932193 3.852853 0.190275
C -4.608782 0.705041 0.210619
C -4.916319 2.169726 0.053875
C -5.537446 -0.230703 0.508662
C -7.010743 -0.034490 0.788727
C -7.885234 -0.704157 -0.287389
C -7.725372 -0.078167 -1.667226
C -7.341064 -0.615527 2.167830
H -1.648325 -2.438285 -0.971639
H -2.295501 2.172977 0.509985
H 0.123620 2.603864 0.419897
H 2.472820 3.111173 0.123482
H 1.837396 -2.490676 -1.377164
H 5.265661 -0.693716 -1.651570
H 4.048599 -0.045563 2.012941
H 5.741018 -0.068192 1.526494
H 5.826236 -3.711054 1.093518
H 6.057739 -2.557342 -0.240779
H 6.810069 -2.260231 1.358231
H 4.452429 4.217697 -0.723666
H 4.359367 4.142437 1.076962
H 5.920065 4.317467 0.261474
H -4.400505 2.586368 -0.818961
H -5.978206 2.368418 -0.110333
H -4.605115 2.725910 0.944229
H -5.215658 -1.272590 0.574118
H -7.267442 1.028647 0.827110
H -7.657972 -1.775821 -0.352299
H -8.939913 -0.624235 0.004533
H -6.713393 -0.218595 -2.058776
H -8.421710 -0.542993 -2.372605
H -7.939879 0.994829 -1.638010
H -8.395546 -0.450503 2.414542
H -6.738689 -0.138334 2.949059
H -7.152354 -1.694442 2.207400
SCF Energy (B3LYP/6-31G**)= -1361.70650250
Number of imaginary frequencies = 0

1b_c081
MMFF Geometry
C -1.383151 -2.200672 -0.780809
C -2.245570 0.368398 -0.570442
N -2.719786 -1.976769 -0.779762
C -0.406563 -1.204663 -0.683064
C -0.864694 0.122301 -0.577448
C -3.137542 -0.698158 -0.664263
C 0.068689 1.172111 -0.492321
C 1.446955 0.918711 -0.492090
C 1.893774 -0.403287 -0.578157
C 0.979325 -1.459666 -0.693057
C 3.351841 -0.647791 -0.577178
C 4.265254 0.441931 0.032204
C 3.707349 1.836407 -0.245793
C 2.395044 2.037610 -0.469895
O 1.392321 -2.763377 -0.824933
O 3.769759 -1.712772 -1.034803
O 5.565702 0.336166 -0.562317
C 4.439883 0.250857 1.540503
C 4.620674 -1.200978 1.918078
C 5.975639 -1.823940 1.713064
O 3.667921 -1.853134 2.349087
O 4.670430 2.827897 -0.215674

C 4.271727 4.176897 -0.429785
C -4.606630 -0.498425 -0.669423
C -5.343441 -1.347492 -1.670278
C -5.184728 0.364209 0.193760
C -6.662817 0.653202 0.357141
C -7.075934 0.384564 1.817319
C -8.573283 0.520269 2.058405
C -6.939469 2.096522 -0.069528
H -1.104290 -3.248949 -0.866429
H -2.622568 1.384408 -0.507088
H -0.291340 2.198315 -0.427974
H 1.982399 3.024710 -0.647737
H 2.373436 -2.779966 -0.899306
H 5.487965 0.661349 -1.476101
H 3.561092 0.611877 2.091193
H 5.303297 0.809645 1.922206
H 6.025289 -2.772327 2.255543
H 6.144655 -2.011738 0.651009
H 6.751277 -1.162954 2.107760
H 3.840092 4.302911 -1.427859
H 3.571968 4.505150 0.345427
H 5.164682 4.805704 -0.366503
H -5.450698 -2.372681 -1.300518
H -6.340289 -0.963247 -1.900271
H -4.805633 -1.378248 -2.625218
H -4.551540 0.907063 0.896179
H -7.258882 -0.005601 -0.281777
H -6.777123 -0.634424 2.096051
H -6.544367 1.063778 2.496033
H -9.141111 -0.121621 1.377615
H -8.815843 0.223709 3.084029
H -8.909906 1.552428 1.925463
H -8.012725 2.310904 -0.069173
H -6.573548 2.281449 -1.086073
H -6.450835 2.814008 0.599312
SCF Energy (B3LYP/6-31G**)= -1361.69993628
Number of imaginary frequencies = 0

1b_c082
MMFF Geometry
C -1.694749 -1.602974 -0.368006
C -2.152643 0.933148 0.489234
N -2.966639 -1.252201 -0.059054
C -0.590152 -0.752736 -0.280041
C -0.838852 0.558970 0.163384
C -3.191607 0.010979 0.369817
C 0.230586 1.469036 0.262084
C 1.540753 1.087867 -0.058009
C 1.779790 -0.222458 -0.481258
C 0.724978 -1.135641 -0.611613
C 3.165031 -0.598791 -0.821947
C 4.320504 0.246770 -0.252403
C 3.916288 1.707967 -0.095357
C 2.624076 2.075222 0.002313
O 0.927175 -2.416451 -1.065235
O 3.373004 -1.576165 -1.546632
O 5.399577 0.166418 -1.201689
C 4.828488 -0.282893 1.090752
C 4.870091 -1.791086 1.140230
C 6.101225 -2.479462 0.615227
O 3.902983 -2.427746 1.560616
O 4.994400 2.571240 -0.016175
C 4.747829 3.970213 0.044419
C -4.601565 0.353925 0.715808
C -4.807933 1.448817 1.727197
C -5.610990 -0.335365 0.139248
C -7.099796 -0.142334 0.321947
C -7.813253 0.047557 -1.031105
C -7.386994 1.316084 -1.760026
C -7.678873 -1.354460 1.055519
H -1.582623 -2.632219 -0.702309
H -2.355816 1.946336 0.819836
H 0.031700 2.488282 0.591465
H 2.328498 3.109364 0.140584
H 1.868504 -2.517378 -1.329325
H 5.179900 -0.556480 -1.822930
H 4.177587 0.036330 1.915703
H 5.833238 0.093926 1.318314
H 6.063267 -3.541458 0.873779
H 6.150177 -2.379777 -0.470814
H 6.992270 -2.045019 1.075122
H 4.191563 4.309125 -0.835512
H 4.221359 4.233635 0.967262
H 5.714425 4.482540 0.050627
H -4.690369 2.431867 1.259539
H -5.794776 1.415492 2.195721
H -4.088888 1.362812 2.550035
H -5.356007 -1.131462 -0.563848
H -7.310898 0.744121 0.928168
H -7.635432 -0.817193 -1.682912
H -8.896484 0.096276 -0.863125
H -6.330873 1.281882 -2.044140
H -7.974016 1.437339 -2.676091
H -7.548761 2.200176 -1.135139
H -8.753110 -1.226369 1.227329
H -7.200218 -1.487327 2.032279
H -7.538264 -2.277903 0.482203
SCF Energy (B3LYP/6-31G**)= -1361.70658174
Number of imaginary frequencies = 0

1b_c083
MMFF Geometry
C 1.790732 -1.609477 -0.144937
C 2.287076 1.037448 -0.491660
N 3.081424 -1.208604 -0.242649
C 0.684465 -0.758978 -0.209352
C 0.953517 0.609077 -0.388775
C 3.326065 0.110888 -0.415257
C -0.118249 1.516818 -0.465206
C -1.447160 1.083730 -0.354868
C -1.711008 -0.274950 -0.168157
C -0.652826 -1.195879 -0.108508
C -3.121417 -0.720964 -0.069447
C -4.222235 0.320098 0.260950
C -3.795036 1.716627 -0.171833
C -2.533728 2.049541 -0.476175
O -0.872925 -2.544374 0.046816
O -3.353611 -1.924373 -0.215763
O -4.392032 0.378244 1.679458
C -5.526833 -0.090640 -0.453841
C -6.108776 -1.379286 0.093135
C -6.797454 -2.291256 -0.885336
O -6.066713 -1.637589 1.298079
O -4.784057 2.682213 -0.325072
C -5.233813 3.174758 0.939492
C 4.758542 0.511360 -0.532882
C 5.052564 1.771756 -1.300871
C 5.712763 -0.271706 0.017687
C 7.209859 -0.048593 0.039163
C 7.714648 -0.120809 1.493777
C 9.190871 0.222372 1.641003
C 7.884116 -1.084891 -0.862267
H 1.664472 -2.681198 -0.007117
H 2.502644 2.093412 -0.617002
H 0.090066 2.576263 -0.611362
H -2.299192 3.059997 -0.798032
H -1.841768 -2.710836 0.006312
H -4.846726 -0.451345 1.947194
H -5.345278 -0.211601 -1.528760
H -6.316391 0.659013 -0.326288
H -7.156632 -3.184271 -0.366527
H -7.648675 -1.771867 -1.331981
H -6.091272 -2.596967 -1.661252
H -5.898830 2.453174 1.422813
H -4.394165 3.427474 1.596361
H -5.808079 4.087669 0.755685
H 4.861026 2.653861 -0.681125
H 6.086303 1.825473 -1.651727
H 4.432778 1.834557 -2.202777
H 5.398300 -1.183798 0.530005
H 7.457293 0.946049 -0.344602
H 7.138707 0.580480 2.111693

H 7.540030 -1.121357 1.909684
H 9.411577 1.203710 1.209509
H 9.465166 0.249411 2.700589
H 9.827187 -0.522647 1.154810
H 8.956658 -0.889020 -0.957695
H 7.460319 -1.060095 -1.872823
H 7.759117 -2.100540 -0.470202
SCF Energy (B3LYP/6-31G**)= -1361.70795560
Number of imaginary frequencies = 0

1b_c084
MMFF Geometry
C -1.647077 -2.228588 -0.397716
C -2.522139 0.342838 -0.464779
N -2.975564 -2.013414 -0.556786
C -0.684524 -1.222839 -0.264342
C -1.149490 0.104297 -0.302035
C -3.401201 -0.732364 -0.580551
C -0.230625 1.161000 -0.172817
C 1.140823 0.915676 -0.016904
C 1.601509 -0.402573 0.012666
C 0.694126 -1.468668 -0.097305
C 3.052390 -0.647287 0.194504
C 4.054193 0.502524 -0.086700
C 3.386564 1.859549 0.094271
C 2.059938 2.035907 0.151756
O 1.106072 -2.779700 -0.046150
O 3.404998 -1.782808 0.525867
O 4.458405 0.438006 -1.456692
C 5.256436 0.360966 0.870469
C 6.081417 -0.879351 0.588566
C 6.699639 -1.575802 1.770146
O 6.281090 -1.264229 -0.565724
O 4.204948 2.968677 0.278836
C 4.800606 3.388831 -0.951061
C -4.861024 -0.538293 -0.761952
C -5.485011 -1.465701 -1.770584
C -5.522482 0.379035 -0.023640
C -6.998769 0.710097 -0.062297
C -7.756524 0.092134 1.129310
C -7.340868 0.592734 2.507306
C -7.190945 2.227438 -0.148414
H -1.362887 -3.278672 -0.380743
H -2.900430 1.359278 -0.514547
H -0.593211 2.188231 -0.195708
H 1.648089 3.029467 0.302975
H 2.066159 -2.798642 0.168148
H 5.053231 -0.340061 -1.542888
H 4.908177 0.326594 1.909979
H 5.955684 1.199818 0.775407
H 7.252452 -2.456148 1.430977
H 7.388689 -0.895185 2.275920
H 5.914055 -1.898738 2.457728
H 5.624233 2.725231 -1.229999
H 4.061848 3.448865 -1.757897
H 5.215379 4.389134 -0.795079
H -5.461055 -2.500835 -1.412680
H -6.527588 -1.229001 -1.991606
H -4.943600 -1.415231 -2.722234
H -4.962782 0.949285 0.718203
H -7.452584 0.298982 -0.969688
H -8.829685 0.283492 1.002800
H -7.634942 -0.998516 1.104757
H -7.512251 1.667749 2.613099
H -7.931881 0.087799 3.278671
H -6.286426 0.385743 2.711694
H -8.255827 2.483478 -0.155511
H -6.748582 2.620528 -1.071009
H -6.721201 2.752331 0.690240
SCF Energy (B3LYP/6-31G**)= -1361.70649878
Number of imaginary frequencies = 0

1b_c085
MMFF Geometry
C -1.503340 -2.412505 -0.067382
C -2.204265 0.199924 -0.321621
N -2.822709 -2.103875 -0.036723
C -0.467611 -1.485079 -0.222506
C -0.842981 -0.136847 -0.356180
C -3.158515 -0.801423 -0.153768
C 0.152508 0.842202 -0.525320
C 1.514506 0.503468 -0.556595
C 1.883856 -0.837373 -0.414374
C 0.899759 -1.828320 -0.255855
C 3.320172 -1.195356 -0.451230
C 4.378962 -0.082285 -0.520239
C 3.828578 1.315369 -0.727661
C 2.507214 1.565444 -0.744307
O 1.221923 -3.160382 -0.133993
O 3.644409 -2.388829 -0.465176
O 5.154590 -0.411946 -1.697478
C 5.328505 -0.182672 0.680456
C 4.696547 0.287833 1.970042
C 5.615287 0.912758 2.985745
O 3.500725 0.122286 2.208748
O 4.819413 2.273561 -0.865575
C 4.437249 3.602223 -1.199652
C -4.611358 -0.506099 -0.117353
C -5.447381 -1.453581 -0.931937
C -5.076614 0.526224 0.619096
C -6.501929 0.986540 0.856212
C -7.041171 1.773995 -0.351632
C -8.386548 2.437290 -0.088731
C -7.436480 -0.130434 1.330508
H -1.289583 -3.473812 0.040060
H -2.518112 1.232569 -0.438073
H -0.144612 1.884998 -0.630903
H 2.117730 2.567996 -0.879538
H 2.198172 -3.252948 -0.209283
H 5.346884 -1.367731 -1.641647
H 6.228582 0.415775 0.489758
H 5.677019 -1.211308 0.833535
H 5.042034 1.208954 3.868591
H 6.377916 0.189009 3.283260
H 6.083952 1.802252 2.557421
H 3.886955 3.626938 -2.145792
H 3.852481 4.054143 -0.392188
H 5.349558 4.192930 -1.324636
H -4.954325 -1.692823 -1.881579
H -5.615145 -2.387530 -0.385409
H -6.418880 -1.033642 -1.198979
H -4.354129 1.121760 1.179489
H -6.439872 1.693117 1.697148
H -6.321082 2.556187 -0.624971
H -7.138828 1.122260 -1.227069
H -8.340556 3.084494 0.792718
H -8.674634 3.053742 -0.946412
H -9.175610 1.695255 0.064981
H -8.326238 0.291999 1.810175
H -6.945359 -0.770827 2.071575
H -7.783540 -0.761933 0.508328
SCF Energy (B3LYP/6-31G**)= -1361.70325915
Number of imaginary frequencies = 0

1b_c086
MMFF Geometry
C -1.830290 -1.648313 -0.519283
C -2.153372 0.802556 0.601784
N -3.082897 -1.265102 -0.171704
C -0.681337 -0.872034 -0.347667
C -0.861391 0.395159 0.230923
C -3.240228 -0.044475 0.390016
C 0.255883 1.226837 0.424199
C 1.545843 0.817701 0.051845
C 1.724582 -0.447674 -0.517035
C 0.614887 -1.287262 -0.719341
C 3.087724 -0.881224 -0.919615
C 4.284892 0.077167 -0.704046
C 3.918048 1.405586 -0.074074
C 2.668439 1.723125 0.284869
O 0.739132 -2.533066 -1.290150
O 3.212031 -1.978307 -1.473457

O 4.811456 0.348365 -2.020776
C 5.390483 -0.663977 0.059737
C 5.020089 -0.987055 1.491004
C 6.161406 -1.284162 2.427533
O 3.852178 -1.055827 1.872248
O 4.943516 2.267855 0.296033
C 5.608256 2.861549 -0.817924
C -4.630034 0.334664 0.777381
C -4.777525 1.325486 1.900307
C -5.674702 -0.234545 0.136155
C -7.151018 0.017420 0.346063
C -7.852573 0.387690 -0.975509
C -7.358387 1.701761 -1.568116
C -7.794796 -1.232956 0.950068
H -1.772464 -2.641494 -0.959454
H -2.300638 1.783716 1.040827
H 0.114350 2.207897 0.876831
H 2.476132 2.663018 0.794978
H 1.691111 -2.694977 -1.482025
H 4.072438 0.665653 -2.568465
H 6.308409 -0.062972 0.064689
H 5.647393 -1.606705 -0.439241
H 5.772379 -1.499296 3.426619
H 6.713756 -2.154959 2.066170
H 6.821792 -0.415557 2.487784
H 6.356972 2.177564 -1.227190
H 4.900959 3.174821 -1.593628
H 6.132713 3.751649 -0.457741
H -4.606105 2.344835 1.539116
H -5.765240 1.296220 2.367253
H -4.065156 1.113595 2.705987
H -5.462279 -0.964022 -0.648556
H -7.314465 0.844572 1.043895
H -7.721171 -0.411294 -1.716202
H -8.931702 0.476196 -0.797666
H -6.305491 1.641444 -1.859756
H -7.937591 1.950751 -2.463102
H -7.472709 2.521947 -0.852227
H -8.860676 -1.066509 1.140081
H -7.324462 -1.494127 1.904742
H -7.703759 -2.096556 0.281400
SCF Energy (B3LYP/6-31G**)= -1361.70246661
Number of imaginary frequencies = 0

1b_c087
MMFF Geometry
C -1.862228 -1.451297 -0.720044
C -2.124765 1.062360 0.269888
N -3.111631 -0.992367 -0.465021
C -0.687194 -0.724006 -0.514500
C -0.835657 0.575426 -0.002375
C -3.239570 0.260277 0.029922
C 0.309878 1.358224 0.226518
C 1.596817 0.869912 -0.048138
C 1.743263 -0.426453 -0.552831
C 0.605576 -1.218601 -0.788167
C 3.103633 -0.943920 -0.852544
C 4.333957 -0.037224 -0.602687
C 3.999644 1.333919 -0.050994
C 2.749024 1.728096 0.216919
O 0.698882 -2.493463 -1.297387
O 3.203602 -2.068917 -1.352713
O 4.951347 0.149937 -1.894357
C 5.352218 -0.797881 0.257286
C 4.881034 -1.039194 1.674959
C 5.947748 -1.348980 2.691863
O 3.690403 -1.035028 1.985537
O 5.044575 2.159880 0.346483
C 5.804840 2.670490 -0.747371
C -4.631145 0.734618 0.285891
C -4.861649 2.221167 0.244814
C -5.608480 -0.171736 0.510295
C -7.070526 0.078395 0.805818
C -7.974865 -0.458302 -0.319059
C -7.777923 0.264954 -1.645516
C -7.435425 -0.591113 2.135210
H -1.829764 -2.465511 -1.112232
H -2.244837 2.059493 0.680217
H 0.192741 2.363918 0.629314
H 2.575705 2.697630 0.675787
H 1.650889 -2.710109 -1.424041
H 4.263496 0.478144 -2.499260
H 6.298200 -0.243292 0.294266
H 5.589056 -1.773110 -0.186053
H 5.489229 -1.500804 3.672937
H 6.475999 -2.260393 2.401628
H 6.646417 -0.511228 2.757067
H 6.540080 1.932997 -1.081189
H 5.162527 2.984061 -1.577570
H 6.352434 3.548485 -0.391940
H -4.325306 2.676887 -0.595513
H -5.911679 2.487463 0.101600
H -4.521268 2.689400 1.174240
H -5.341504 -1.230862 0.494445
H -7.271884 1.147111 0.927804
H -7.803324 -1.531928 -0.468144
H -9.024981 -0.346785 -0.021106
H -6.773260 0.103208 -2.047540
H -8.495030 -0.106142 -2.384809
H -7.936482 1.342051 -1.531897
H -8.480759 -0.391250 2.394759
H -6.811791 -0.208751 2.951155
H -7.303206 -1.678169 2.089770
SCF Energy (B3LYP/6-31G**)= -1361.70247329
Number of imaginary frequencies = 0

1b_c088
MMFF Geometry
C -1.377626 -2.365858 -0.522777
C -2.344943 0.078007 0.170210
N -2.708297 -2.250537 -0.293188
C -0.457597 -1.316856 -0.430886
C -0.969529 -0.056129 -0.069781
C -3.179353 -1.030876 0.043221
C -0.095236 1.041579 0.036081
C 1.278996 0.899235 -0.198522
C 1.781895 -0.359349 -0.541733
C 0.922593 -1.458695 -0.677219
C 3.234471 -0.486914 -0.785474
C 4.166796 0.576788 -0.159045
C 3.494581 1.948304 -0.145548
C 2.154146 2.075159 -0.147395
O 1.384696 -2.696416 -1.054171
O 3.637405 -1.443075 -1.450041
O 5.365040 0.655710 -0.942476
C 4.580340 0.194463 1.263921
C 4.898837 -1.276896 1.393423
C 6.239795 -1.757976 0.907171
O 4.061683 -2.054549 1.855227
O 4.393004 2.998480 -0.107454
C 3.890221 4.328721 -0.059846
C -4.638470 -0.951357 0.291512
C -5.200608 -2.112457 1.067264
C -5.361697 0.087666 -0.178347
C -6.856299 0.290932 -0.051780
C -7.201585 1.328583 1.033895
C -6.683941 2.739907 0.783549
C -7.454056 0.656164 -1.414207
H -1.054627 -3.369157 -0.793045
H -2.760176 1.036040 0.467199
H -0.499027 2.017831 0.302048
H 1.663721 3.042007 -0.116593
H 2.341699 -2.625490 -1.272118
H 5.130211 1.096047 -1.777787
H 3.776917 0.406620 1.981814
H 5.458784 0.761215 1.596418
H 6.425452 -2.765159 1.290969
H 6.255098 -1.784133 -0.184103
H 7.027786 -1.099006 1.280173
H 3.304462 4.557196 -0.955962
H 3.299841 4.490950 0.847623
H 4.745147 5.010728 -0.032628

H -4.533965 -2.388681 1.892653
H -6.167220 -1.885310 1.524877
H -5.327343 -2.984762 0.417522
H -4.852760 0.865188 -0.747751
H -7.335443 -0.649743 0.242462
H -8.292319 1.375419 1.144716
H -6.810161 0.981633 1.998933
H -7.105008 3.166028 -0.131521
H -6.972233 3.393106 1.613874
H -5.592796 2.762936 0.709012
H -8.533304 0.823384 -1.331528
H -7.297813 -0.155436 -2.134104
H -7.001852 1.560240 -1.835642
SCF Energy (B3LYP/6-31G**)= -1361.69887581
Number of imaginary frequencies = 0

1b_c089
MMFF Geometry
C -1.738465 -2.581442 0.054237
C -2.431978 0.044280 0.163694
N -3.042873 -2.276664 0.256912
C -0.713871 -1.642675 -0.102479
C -1.084587 -0.286315 -0.042552
C -3.378266 -0.969352 0.301057
C -0.099683 0.706975 -0.194835
C 1.246470 0.370676 -0.393629
C 1.609097 -0.977190 -0.440586
C 0.638195 -1.981139 -0.311731
C 3.035123 -1.314175 -0.632675
C 4.100787 -0.233892 -0.367872
C 3.557534 1.164887 -0.617433
C 2.237574 1.428051 -0.604929
O 0.963575 -3.314757 -0.378711
O 3.346175 -2.466980 -0.949290
O 5.167768 -0.510837 -1.290337
C 4.611513 -0.456196 1.060186
C 5.507061 0.649667 1.573258
C 6.912333 0.728296 1.035468
O 5.098611 1.447933 2.419583
O 4.547162 2.108490 -0.826209
C 4.178738 3.475195 -0.960212
C -4.814325 -0.681310 0.528167
C -5.471502 -1.566538 1.552991
C -5.439852 0.292516 -0.167366
C -6.901745 0.676926 -0.087204
C -7.109143 1.966546 0.730411
C -6.456296 3.219690 0.159552
C -7.495532 0.781258 -1.495554
H -1.528483 -3.648457 0.021117
H -2.738496 1.083694 0.230641
H -0.392829 1.755914 -0.158832
H 1.850215 2.428613 -0.763057
H 1.902614 -3.398895 -0.658903
H 5.174195 -1.478467 -1.432145
H 5.174024 -1.395513 1.136139
H 3.771956 -0.543137 1.762872
H 7.489815 1.440866 1.631490
H 7.390997 -0.251186 1.112428
H 6.906366 1.063530 -0.002790
H 3.560272 3.627432 -1.850414
H 3.668063 3.833045 -0.060414
H 5.095136 4.060000 -1.082710
H -4.814715 -1.714610 2.418257
H -6.397068 -1.139171 1.947988
H -5.704405 -2.546410 1.123166
H -4.869712 0.860579 -0.902333
H -7.466780 -0.114882 0.417579
H -8.185901 2.153061 0.830307
H -6.729429 1.810642 1.748447
H -6.855979 3.466786 -0.828085
H -6.653439 4.072729 0.817336
H -5.370969 3.109063 0.078858
H -8.549280 1.076542 -1.450855
H -7.442969 -0.185690 -2.008980
H -6.964502 1.511343 -2.115579
SCF Energy (B3LYP/6-31G**)= -1361.70741538
Number of imaginary frequencies = 0

1b_c090
MMFF Geometry
C 1.528617 2.377607 -0.051588
C 2.235418 -0.232729 -0.312357
N 2.849484 2.073994 -0.041459
C 0.493960 1.446255 -0.189053
C 0.872611 0.098918 -0.326474
C 3.188351 0.772493 -0.161188
C -0.120982 -0.884463 -0.478512
C -1.483241 -0.549403 -0.490416
C -1.857843 0.790587 -0.343341
C -0.875202 1.784991 -0.201286
C -3.298260 1.144263 -0.361735
C -4.359705 0.029477 -0.425076
C -3.789032 -1.352556 -0.654379
C -2.475042 -1.610767 -0.654819
O -1.200069 3.116064 -0.076586
O -3.627606 2.336503 -0.375081
O -5.156863 0.383726 -1.578275
C -5.279378 0.099646 0.798863
C -4.600958 -0.392491 2.056081
C -5.413702 -1.263885 2.975181
O -3.449866 -0.060914 2.338521
O -4.672728 -2.424729 -0.688300
C -5.392910 -2.491936 -1.919361
C 4.642731 0.482712 -0.146669
C 5.462722 1.432517 -0.974690
C 5.123065 -0.547189 0.583471
C 6.553488 -1.002089 0.799225
C 7.077160 -1.788614 -0.416053
C 8.428811 -2.446754 -0.173137
C 7.491041 0.118720 1.258305
H 1.312714 3.438259 0.057902
H 2.551035 -1.264557 -0.431631
H 0.176235 -1.927117 -0.586199
H -2.124973 -2.634279 -0.753775
H -2.178127 3.204627 -0.126590
H -5.321225 1.345562 -1.510058
H -6.181196 -0.499677 0.620230
H -5.631780 1.121536 0.984329
H -4.809362 -1.552345 3.839634
H -6.290107 -0.711493 3.322607
H -5.721883 -2.168329 2.444804
H -6.267796 -1.836698 -1.884607
H -4.759684 -2.251081 -2.780493
H -5.751783 -3.518600 -2.039235
H 5.635198 2.367640 -0.431647
H 6.431659 1.016000 -1.256015
H 4.954456 1.668941 -1.916991
H 4.411424 -1.145055 1.155205
H 6.506848 -1.708151 1.641588
H 6.355868 -2.573673 -0.677755
H 7.159078 -1.137286 -1.293405
H 8.398625 -3.093355 0.709437
H 8.706063 -3.062892 -1.034605
H 9.217392 -1.701703 -0.032066
H 8.389557 -0.300026 1.724694
H 7.008918 0.757952 2.006253
H 7.823197 0.750774 0.430417
SCF Energy (B3LYP/6-31G**)= -1361.69756535
Number of imaginary frequencies = 0

1b_c091
MMFF Geometry
C -1.904199 -1.410708 -0.187634
C -2.305070 1.252535 0.157584
N -3.179439 -0.952878 -0.172406
C -0.768257 -0.611716 -0.037624
C -0.988023 0.765031 0.142380
C -3.376501 0.374893 -0.002282
C 0.115001 1.621782 0.309194
C 1.427761 1.130023 0.285358
C 1.643353 -0.236750 0.095753
C 0.552027 -1.107758 -0.051766

C 3.035967 -0.745194 0.088779
C 4.202539 0.248872 -0.147294
C 3.808533 1.657637 0.277238
C 2.544977 2.043655 0.498076
O 0.722179 -2.462970 -0.211324
O 3.203523 -1.959691 0.231733
O 4.473998 0.317749 -1.549429
C 5.434092 -0.230145 0.649848
C 5.995091 -1.536692 0.123870
C 6.572004 -2.491811 1.132971
O 6.026364 -1.777006 -1.085120
O 4.826309 2.575041 0.514916
C 5.385759 3.063022 -0.706914
C -4.793129 0.842597 -0.008779
C -5.039367 2.269895 -0.418203
C -5.780493 -0.023041 0.311771
C -7.266155 0.250325 0.387258
C -8.008171 -0.264268 -0.861360
C -7.951077 -1.770440 -1.086255
C -7.841514 -0.329530 1.683469
H -1.816561 -2.485596 -0.330642
H -2.482398 2.312364 0.307650
H -0.055710 2.687619 0.457623
H 2.333531 3.059388 0.819328
H 1.677475 -2.673728 -0.106452
H 4.909097 -0.528089 -1.798230
H 5.172227 -0.356753 1.707413
H 6.263252 0.484370 0.589765
H 6.926628 -3.393400 0.625993
H 7.412016 -2.017629 1.646165
H 5.800130 -2.775155 1.852778
H 6.050272 2.318168 -1.154288
H 4.606690 3.362491 -1.416649
H 5.985880 3.946209 -0.468569
H -4.417979 2.543318 -1.278738
H -6.071330 2.449734 -0.731515
H -4.815143 2.952066 0.408368
H -5.503451 -1.048876 0.563043
H -7.441268 1.330902 0.441377
H -9.061776 0.035341 -0.795592
H -7.602732 0.234875 -1.750914
H -8.415025 -2.317922 -0.260873
H -8.495542 -2.029585 -2.000379
H -6.922151 -2.122810 -1.203701
H -8.920687 -0.151863 1.741872
H -7.379086 0.144505 2.556873
H -7.670152 -1.408063 1.766308
SCF Energy (B3LYP/6-31G**)= -1361.70713985
Number of imaginary frequencies = 0

1b_c092
MMFF Geometry
C -1.744413 -1.314180 -0.753684
C -2.156857 1.146384 0.319401
N -3.020027 -0.930461 -0.505154
C -0.613828 -0.534616 -0.499240
C -0.839107 0.737911 0.056922
C -3.222827 0.295474 0.030093
C 0.257994 1.575354 0.332277
C 1.570529 1.159814 0.072750
C 1.786880 -0.113021 -0.466485
C 0.705534 -0.951580 -0.768736
C 3.178520 -0.528455 -0.743769
C 4.331011 0.206073 -0.030076
C 3.957993 1.650925 0.242049
C 2.686626 2.072291 0.325876
O 0.884838 -2.190721 -1.334337
O 3.396722 -1.448661 -1.537693
O 5.454720 0.179707 -0.927467
C 4.742080 -0.469484 1.280192
C 4.718288 -1.976094 1.189446
C 5.963376 -2.671045 0.707991
O 3.693826 -2.602139 1.464024
O 4.997601 2.503141 0.582824
C 5.383837 3.314651 -0.526958
C -4.641753 0.686723 0.274995
C -4.951949 2.159247 0.270945
C -5.571725 -0.277128 0.456819
C -7.049803 -0.114589 0.733237
C -7.905390 -0.666873 -0.421826
C -7.726563 0.103411 -1.724153
C -7.399234 -0.840386 2.036989
H -1.651826 -2.310140 -1.181612
H -2.338336 2.121139 0.759939
H 0.080569 2.564740 0.752658
H 2.465888 3.098435 0.605067
H 1.839609 -2.318443 -1.529131
H 5.286209 -0.541933 -1.567071
H 4.060979 -0.199315 2.098288
H 5.747338 -0.161103 1.592654
H 5.849879 -3.751552 0.833397
H 6.128043 -2.451952 -0.348752
H 6.821431 -2.343356 1.300069
H 5.687734 2.707214 -1.385078
H 4.573570 3.993495 -0.813592
H 6.240756 3.920162 -0.217563
H -4.425264 2.667541 -0.544981
H -6.012044 2.372482 0.113602
H -4.655250 2.617826 1.219943
H -5.247417 -1.319465 0.415855
H -7.310747 0.937868 0.881070
H -7.673464 -1.725037 -0.597400
H -8.964687 -0.620526 -0.139525
H -6.708229 0.007475 -2.112641
H -8.410413 -0.284532 -2.485966
H -7.945180 1.166742 -1.583717
H -8.457933 -0.704559 2.283443
H -6.810543 -0.448427 2.874066
H -7.207461 -1.917011 1.963916
SCF Energy (B3LYP/6-31G**)= -1361.70144477
Number of imaginary frequencies = 0

1b_c093
MMFF Geometry
C -1.634167 -2.555347 0.111930
C -2.321072 0.074757 0.104695
N -2.937633 -2.238255 0.301713
C -0.607609 -1.627097 -0.087423
C -0.974748 -0.267993 -0.088163
C -3.269620 -0.929390 0.288377
C 0.011998 0.715387 -0.287403
C 1.356502 0.366300 -0.470969
C 1.716097 -0.984051 -0.455098
C 0.742961 -1.978642 -0.282373
C 3.144681 -1.332296 -0.627590
C 4.196461 -0.241323 -0.321032
C 3.669250 1.135112 -0.711566
C 2.351422 1.405740 -0.746904
O 1.066776 -3.314167 -0.292303
O 3.425514 -2.487730 -0.951656
O 5.378571 -0.539049 -1.072654
C 4.544203 -0.368044 1.166733
C 5.370142 0.785714 1.689000
C 6.837254 0.815282 1.348877
O 4.850667 1.678725 2.362416
O 4.672000 2.042020 -1.001794
C 4.328060 3.417902 -1.123437
C -4.704905 -0.628014 0.504251
C -5.360646 -1.464650 1.569967
C -5.329475 0.310830 -0.238617
C -6.790569 0.706944 -0.180475
C -6.906795 2.134289 0.384070
C -8.344840 2.585865 0.599206
C -7.401377 0.582117 -1.579736
H -1.426594 -3.623292 0.126546
H -2.625231 1.116757 0.125674
H -0.278502 1.765523 -0.300229
H 1.969247 2.387775 -1.003606
H 2.003974 -3.409686 -0.579563
H 5.197364 -0.293431 -1.996771
H 5.101523 -1.292643 1.363837
H 3.637124 -0.420289 1.783216

H 7.344757 1.538210 1.994015
H 7.278622 -0.168783 1.525450
H 6.977740 1.110100 0.307477
H 3.698707 3.585063 -2.003134
H 3.838646 3.782295 -0.214359
H 5.253898 3.984585 -1.259251
H -4.699565 -1.578800 2.437157
H -6.281527 -1.016989 1.952364
H -5.600289 -2.461053 1.184053
H -4.759957 0.842597 -1.001255
H -7.348565 0.028261 0.472315
H -6.386512 2.185190 1.349483
H -6.404989 2.849543 -0.280125
H -8.888256 1.878780 1.233774
H -8.360708 3.563593 1.091625
H -8.880926 2.685719 -0.348961
H -8.483466 0.744820 -1.554808
H -7.234363 -0.419694 -1.991966
H -6.966210 1.308883 -2.274950
SCF Energy (B3LYP/6-31G**)= -1361.70242612
Number of imaginary frequencies = 0

1b_c094
MMFF Geometry
C 1.688948 2.499720 0.312901
C 2.303378 0.025918 -0.631522
N 2.998096 2.170349 0.197744
C 0.622032 1.658723 -0.018242
C 0.952093 0.381336 -0.509475
C 3.292538 0.935647 -0.263028
C -0.076548 -0.506432 -0.875707
C -1.424566 -0.148652 -0.741508
C -1.745270 1.114322 -0.236445
C -0.733597 2.022330 0.107100
C -3.174999 1.465340 -0.079765
C -4.197681 0.308829 0.004366
C -3.765870 -0.852491 -0.884465
C -2.473754 -1.065238 -1.194879
O -1.022931 3.281848 0.574507
O -3.471582 2.656331 0.031862
O -5.468537 0.805765 -0.430267
C -4.323434 -0.063565 1.486237
C -5.091506 -1.345071 1.718379
C -6.592729 -1.299660 1.603326
O -4.500758 -2.395050 1.981783
O -4.821772 -1.639168 -1.306856
C -4.532601 -2.891010 -1.919560
C 4.736357 0.618334 -0.375330
C 5.577922 1.743847 -0.914310
C 5.206470 -0.585808 0.015120
C 6.646006 -1.057901 0.014476
C 7.038799 -1.496469 1.438670
C 8.509354 -1.867315 1.574808
C 6.800350 -2.188117 -1.005591
H 1.511730 3.502776 0.695368
H 2.582399 -0.947290 -1.023625
H 0.183518 -1.488209 -1.270445
H -2.156368 -1.900701 -1.809388
H -1.989380 3.442057 0.473118
H -5.428911 0.878399 -1.399967
H -4.824832 0.732338 2.051453
H -3.335768 -0.191452 1.948546
H -7.017938 -2.206254 2.043405
H -6.980761 -0.438672 2.153324
H -6.889670 -1.242118 0.554668
H -4.040622 -2.746258 -2.886462
H -3.926538 -3.520489 -1.259883
H -5.481899 -3.404448 -2.098351
H 5.768820 2.489078 -0.134934
H 6.541184 1.404615 -1.303278
H 5.073652 2.240099 -1.751888
H 4.505492 -1.317908 0.417331
H 7.319155 -0.246635 -0.279215
H 6.827488 -0.678727 2.140070
H 6.426769 -2.349577 1.758316
H 9.154082 -1.050155 1.236426
H 8.747852 -2.074352 2.623112
H 8.752758 -2.764267 0.998117
H 7.848663 -2.483167 -1.114326
H 6.449147 -1.873380 -1.995064
H 6.227788 -3.075244 -0.712208
SCF Energy (B3LYP/6-31G**)= -1361.70247992
Number of imaginary frequencies = 0

1b_c095
MMFF Geometry
C 1.708430 -1.540328 0.499383
C 2.187700 0.889910 -0.614322
N 2.986098 -1.226345 0.175004
C 0.607933 -0.702488 0.305038
C 0.868014 0.554599 -0.270749
C 3.221683 -0.016106 -0.381934
C -0.196223 1.450925 -0.482216
C -1.511417 1.108019 -0.142461
C -1.764019 -0.150732 0.413813
C -0.713637 -1.046588 0.654714
C -3.157064 -0.489278 0.775754
C -4.308621 0.306936 0.129887
C -3.874333 1.729075 -0.169562
C -2.589219 2.080139 -0.331363
O -0.925634 -2.272998 1.236537
O -3.377032 -1.395881 1.584704
O -5.376003 0.342474 1.093308
C -4.834288 -0.345603 -1.150752
C -4.885840 -1.851304 -1.056715
C -6.134504 -2.478353 -0.497575
O -3.915215 -2.531450 -1.391584
O -4.884498 2.636818 -0.450185
C -5.157883 3.468831 0.677816
C 4.637843 0.285821 -0.740744
C 4.862879 1.268199 -1.858195
C 5.636279 -0.341125 -0.080009
C 7.128264 -0.171161 -0.259635
C 7.822439 0.158102 1.076607
C 7.389947 1.496851 1.662172
C 7.713822 -1.454605 -0.853439
H 1.587981 -2.528315 0.938714
H 2.398798 1.861998 -1.047560
H 0.009300 2.428920 -0.916235
H -2.329952 3.092585 -0.626968
H -1.872646 -2.348363 1.488200
H -5.208772 -0.386816 1.724510
H -4.190693 -0.112465 -2.009594
H -5.838684 0.016360 -1.402637
H -6.086950 -3.563413 -0.625979
H -6.222110 -2.250585 0.566507
H -7.008449 -2.105153 -1.037146
H -5.441905 2.879368 1.555070
H -4.295890 4.102475 0.912298
H -5.997652 4.120156 0.418507
H 4.741302 2.295089 -1.498152
H 5.856589 1.184442 -2.305410
H 4.156127 1.097326 -2.678431
H 5.368203 -1.058553 0.698781
H 7.351350 0.646473 -0.952144
H 7.632057 -0.633236 1.812681
H 8.908182 0.186862 0.920677
H 6.329655 1.494648 1.932466
H 7.963874 1.712305 2.569178
H 7.563953 2.310277 0.950602
H 8.790906 -1.347298 -1.021618
H 7.249104 -1.688178 -1.817963
H 7.561587 -2.312608 -0.188710
SCF Energy (B3LYP/6-31G**)= -1361.70153422
Number of imaginary frequencies = 0

1b_c096
MMFF Geometry
C 1.774940 2.484266 0.082377
C 2.453387 -0.147744 0.081326
N 3.083455 2.165383 0.229112
C 0.738830 1.557392 -0.069633

C 1.102200 0.197426 -0.067500
C 3.410766 0.855305 0.218963
C 0.106422 -0.784746 -0.218461
C -1.242313 -0.433245 -0.360363
C -1.599692 0.918446 -0.349224
C -0.617464 1.911197 -0.220333
C -3.031446 1.272930 -0.483502
C -4.101761 0.190385 -0.236062
C -3.556606 -1.185104 -0.556474
C -2.246907 -1.473952 -0.568067
O -0.934384 3.248523 -0.231791
O -3.343165 2.439391 -0.745147
O -5.183077 0.503794 -1.127838
C -4.577528 0.362238 1.210010
C -5.477492 -0.745164 1.712183
C -6.866588 -0.846839 1.137276
O -5.093747 -1.512312 2.597538
O -4.490626 -2.194369 -0.730134
C -4.691297 -2.486900 -2.113315
C 4.851580 0.550558 0.387584
C 5.544949 1.392025 1.425074
C 5.445269 -0.395925 -0.370739
C 6.902107 -0.806376 -0.359104
C 7.071141 -2.270597 0.086756
C 6.642852 -2.512448 1.528873
C 7.490376 -0.613778 -1.760770
H 1.571544 3.553014 0.092187
H 2.754874 -1.190590 0.103934
H 0.391682 -1.836516 -0.226349
H -1.912269 -2.493887 -0.734992
H -1.883237 3.348824 -0.469740
H -5.212516 1.479532 -1.197653
H -5.125986 1.305023 1.332961
H -3.718934 0.414104 1.893033
H -7.453054 -1.556022 1.728634
H -7.356531 0.128898 1.186091
H -6.831169 -1.198675 0.105371
H -4.999633 -1.597585 -2.672003
H -3.786410 -2.916136 -2.556220
H -5.490920 -3.229782 -2.186344
H 4.920072 1.498677 2.319612
H 6.486318 0.955181 1.767072
H 5.758044 2.391206 1.030751
H 4.846976 -0.925950 -1.112354
H 7.485255 -0.173755 0.317222
H 6.502649 -2.939139 -0.572171
H 8.125915 -2.557037 -0.009874
H 5.570299 -2.343729 1.664449
H 6.854658 -3.547951 1.814122
H 7.186430 -1.854844 2.214657
H 8.556159 -0.866775 -1.771011
H 7.393152 0.427790 -2.087177
H 6.988098 -1.247872 -2.500266
SCF Energy (B3LYP/6-31G**)= -1361.70266104
Number of imaginary frequencies = 0

1b_c097
MMFF Geometry
C -1.562002 -2.245068 -0.142932
C -2.161162 0.388146 0.152325
N -2.857466 -1.909406 0.071656
C -0.502516 -1.335449 -0.225847
C -0.824833 0.024053 -0.070042
C -3.144607 -0.597414 0.209184
C 0.197658 0.987217 -0.140649
C 1.534609 0.620603 -0.361873
C 1.851400 -0.732824 -0.511437
C 0.839311 -1.706237 -0.450870
C 3.260801 -1.119444 -0.749382
C 4.360883 -0.045853 -0.705085
C 3.861089 1.381214 -0.590590
C 2.556425 1.668873 -0.435962
O 1.108335 -3.046072 -0.610508
O 3.526130 -2.296919 -1.019289
O 5.015344 -0.164373 -1.991052
C 5.404922 -0.404560 0.360142
C 4.909386 -0.173772 1.769012
C 5.940025 0.219039 2.793569
O 3.732666 -0.349545 2.083380
O 4.880431 2.318145 -0.635504
C 4.534055 3.697155 -0.672239
C -4.571964 -0.271492 0.449458
C -5.243127 -1.180705 1.441255
C -5.155371 0.747716 -0.218084
C -6.586588 1.247003 -0.178473
C -7.612683 0.168831 -0.575565
C -9.007875 0.730272 -0.819759
C -6.908081 1.947279 1.144018
H -1.388697 -3.313722 -0.250426
H -2.430550 1.430206 0.293763
H -0.058129 2.039133 -0.018593
H 2.204414 2.689735 -0.340959
H 2.068684 -3.151175 -0.794862
H 5.166797 -1.118146 -2.135954
H 6.312327 0.192551 0.202936
H 5.716628 -1.453218 0.281799
H 5.459607 0.355396 3.766387
H 6.692633 -0.568825 2.875915
H 6.408799 1.161247 2.498943
H 3.906150 3.921822 -1.540497
H 4.042936 4.000442 0.257817
H 5.459074 4.273569 -0.766844
H -5.446182 -2.159038 0.992679
H -4.607856 -1.328982 2.322356
H -6.188043 -0.782719 1.813273
H -4.548111 1.301124 -0.936246
H -6.640066 2.025579 -0.953885
H -7.278358 -0.328911 -1.494860
H -7.687376 -0.607933 0.191882
H -8.986785 1.524698 -1.572335
H -9.671911 -0.061045 -1.182478
H -9.444196 1.132741 0.099073
H -7.827124 2.536471 1.059294
H -6.107270 2.639389 1.428247
H -7.046928 1.238071 1.964718
SCF Energy (B3LYP/6-31G**)= -1361.70348129
Number of imaginary frequencies = 0

1b_c098
MMFF Geometry
C 1.848008 2.343000 0.145748
C 2.406831 -0.193530 -0.655008
N 3.149492 1.989282 0.017535
C 0.762082 1.497769 -0.103186
C 1.063821 0.187691 -0.520503
C 3.416483 0.723908 -0.371367
C 0.015484 -0.707520 -0.800862
C -1.323869 -0.322793 -0.656470
C -1.618261 0.974480 -0.225959
C -0.585812 1.887215 0.034527
C -3.038807 1.360138 -0.061826
C -4.113493 0.257140 0.022888
C -3.674297 -0.977629 -0.734966
C -2.390783 -1.258968 -1.003905
O -0.843673 3.177850 0.430468
O -3.331407 2.555064 0.051174
O -5.287330 0.811733 -0.591314
C -4.389023 0.022334 1.511497
C -5.261121 -1.177592 1.808222
C -6.716658 -1.109581 1.424519
O -4.797140 -2.162562 2.386312
O -4.665646 -1.893384 -1.050848
C -5.055806 -1.787915 -2.420409
C 4.852682 0.379986 -0.500038
C 5.694768 1.458009 -1.127623
C 5.317649 -0.803165 -0.044860
C 6.749563 -1.293561 -0.049448
C 7.223548 -1.662638 1.369725
C 7.273802 -0.467638 2.313937
C 6.860795 -2.509103 -0.973305
H 1.693797 3.370561 0.468271
H 2.662959 -1.193653 -0.991200

H 0.251744 -1.717217 -1.136047
H -2.124809 -2.193765 -1.489214
H -1.810376 3.344715 0.362394
H -5.283285 1.768634 -0.386066
H -4.876953 0.895947 1.962588
H -3.447324 -0.120978 2.058359
H -7.249573 -1.953942 1.871328
H -7.154757 -0.184823 1.808422
H -6.831651 -1.158649 0.340895
H -5.394857 -0.776424 -2.665394
H -4.235181 -2.081975 -3.083231
H -5.889622 -2.476466 -2.585493
H 5.177347 1.906864 -1.983689
H 5.911903 2.248305 -0.401274
H 6.645212 1.082838 -1.514902
H 4.618902 -1.497802 0.422290
H 7.428762 -0.525974 -0.433019
H 6.573897 -2.434044 1.802318
H 8.229816 -2.096261 1.311757
H 6.277185 -0.052339 2.491550
H 7.684562 -0.771773 3.282055
H 7.911835 0.324648 1.909619
H 7.895128 -2.866736 -1.019922
H 6.550861 -2.256032 -1.993471
H 6.234103 -3.338466 -0.625978
SCF Energy (B3LYP/6-31G**)= -1361.70278861
Number of imaginary frequencies = 0

1b_c099
MMFF Geometry
C -1.585437 -2.201701 -0.272449
C -2.193771 0.403744 0.199067
N -2.884885 -1.882614 -0.056573
C -0.526105 -1.288186 -0.273195
C -0.853506 0.057019 -0.026758
C -3.176378 -0.583807 0.169365
C 0.167706 1.023616 -0.012064
C 1.507195 0.673199 -0.238241
C 1.831194 -0.666785 -0.476959
C 0.820000 -1.642204 -0.501559
C 3.246976 -1.035180 -0.722002
C 4.349113 0.033938 -0.596610
C 3.824893 1.440224 -0.407240
C 2.528934 1.718550 -0.216818
O 1.093455 -2.967249 -0.751169
O 3.518894 -2.189793 -1.072716
O 5.027345 -0.025111 -1.872251
C 5.366086 -0.372909 0.475294
C 4.820816 -0.210529 1.875048
C 5.746379 0.377891 2.905435
O 3.685385 -0.582200 2.170684
O 4.750643 2.464452 -0.247308
C 5.366007 2.833650 -1.481688
C -4.608075 -0.276548 0.408603
C -5.294197 -1.255027 1.321072
C -5.181892 0.788021 -0.193224
C -6.614232 1.282904 -0.141287
C -7.632221 0.235992 -0.631882
C -9.023970 0.813353 -0.857927
C -6.958313 1.885971 1.222768
H -1.408737 -3.260045 -0.452096
H -2.466857 1.433054 0.410018
H -0.089546 2.064639 0.180692
H 2.219026 2.739924 -0.015137
H 2.058192 -3.060996 -0.916808
H 5.152640 -0.974888 -2.069812
H 6.275850 0.232185 0.372554
H 5.685921 -1.415323 0.357292
H 5.231982 0.443955 3.868112
H 6.625328 -0.261882 3.015285
H 6.045600 1.382883 2.597624
H 6.209769 2.172684 -1.698856
H 4.651568 2.839280 -2.312399
H 5.760028 3.847912 -1.366726
H -4.673349 -1.466259 2.199607
H -6.245692 -0.885119 1.705104
H -5.488422 -2.198538 0.799870
H -4.563566 1.392251 -0.859087
H -6.656047 2.115337 -0.859348
H -7.282184 -0.194146 -1.579024
H -7.718461 -0.594076 0.076259
H -8.991598 1.659956 -1.550851
H -9.680899 0.050132 -1.287610
H -9.475855 1.148485 0.080208
H -7.876660 2.479553 1.165632
H -6.163223 2.555954 1.569259
H -7.109597 1.119422 1.987829
SCF Energy (B3LYP/6-31G**)= -1361.69777936
Number of imaginary frequencies = 0

1b_c100
MMFF Geometry
C 1.932027 1.721484 -0.204993
C 2.121534 -0.986311 -0.291472
N 3.164906 1.164286 -0.136441
C 0.738708 1.004184 -0.316192
C 0.848511 -0.397162 -0.359887
C 3.257093 -0.184966 -0.180216
C -0.317327 -1.176938 -0.476385
C -1.586093 -0.583855 -0.536526
C -1.687491 0.807755 -0.479973
C -0.535753 1.602240 -0.386057
C -3.033469 1.416140 -0.525997
C -4.261739 0.536972 -0.224081
C -4.016474 -0.917827 -0.594914
C -2.774082 -1.421785 -0.714174
O -0.604240 2.974495 -0.354562
O -3.142482 2.625537 -0.752688
O -5.325005 1.069915 -1.031235
C -4.607597 0.751408 1.253761
C -5.654131 -0.202384 1.785942
C -7.086558 0.018312 1.374086
O -5.339802 -1.119597 2.547678
O -5.181181 -1.644543 -0.764244
C -5.092016 -3.043404 -1.002515
C 4.632475 -0.759483 -0.117633
C 4.836571 -2.110885 -0.747879
C 5.623691 -0.046219 0.461665
C 7.071907 -0.443752 0.651912
C 7.956871 0.389524 -0.292272
C 9.423139 -0.019872 -0.267446
C 7.454807 -0.258739 2.123821
H 1.929949 2.808668 -0.165865
H 2.214056 -2.067249 -0.312001
H -0.228548 -2.262083 -0.521806
H -2.598332 -2.463565 -0.959019
H -1.528879 3.250905 -0.544245
H -5.157703 2.029333 -1.120672
H -4.973549 1.771099 1.429277
H -3.714544 0.633079 1.881958
H -7.740400 -0.615118 1.980500
H -7.362428 1.061603 1.546896
H -7.226214 -0.238561 0.322935
H -4.584972 -3.246401 -1.951111
H -4.590791 -3.550843 -0.171996
H -6.109901 -3.437942 -1.073498
H 4.300291 -2.183703 -1.701076
H 5.883847 -2.316087 -0.984577
H 4.479756 -2.903982 -0.082647
H 5.382992 0.941124 0.862004
H 7.213283 -1.504157 0.419651
H 7.591110 0.280500 -1.321648
H 7.881027 1.455981 -0.044367
H 9.536920 -1.087462 -0.480183
H 9.980615 0.537713 -1.027051
H 9.883584 0.192963 0.701610
H 8.456972 -0.650713 2.323955
H 6.760344 -0.796293 2.779739
H 7.441232 0.798110 2.413268
SCF Energy (B3LYP/6-31G**)= -1361.70947043
Number of imaginary frequencies = 0

1b_c101
MMFF Geometry
C 1.461517 -2.409062 -0.153321
C 2.441772 0.119138 -0.339716
N 2.806755 -2.250460 -0.192287
C 0.532244 -1.365328 -0.202683
C 1.051227 -0.060684 -0.302820
C 3.283309 -0.989643 -0.274148
C 0.167120 1.031830 -0.373467
C -1.221115 0.845428 -0.326418
C -1.728590 -0.449947 -0.208096
C -0.863991 -1.553179 -0.166016
C -3.195959 -0.620969 -0.161474
C -4.050724 0.580047 0.311511
C -3.435103 1.889438 -0.191240
C -2.120247 1.993438 -0.465279
O -1.338861 -2.840997 -0.092607
O -3.658726 -1.723866 -0.461245
O -4.013605 0.660923 1.739797
C -5.498488 0.409506 -0.193123
C -6.181547 -0.800465 0.413755
C -7.195131 -1.514135 -0.438794
O -5.956491 -1.146808 1.575695
O -4.342012 2.932078 -0.283922
C -3.852011 4.240646 -0.551455
C 4.759711 -0.861771 -0.315383
C 5.447216 -1.886247 -1.177461
C 5.386545 0.095694 0.401354
C 6.878525 0.334426 0.510033
C 7.231255 1.662559 -0.183648
C 8.727490 1.939007 -0.238760
C 7.283228 0.323101 1.987419
H 1.134660 -3.444076 -0.078309
H 2.864355 1.114750 -0.432825
H 0.571738 2.039065 -0.465022
H -1.669083 2.922820 -0.793067
H -2.316692 -2.820511 -0.198259
H -4.564493 -0.076506 2.082683
H -5.509170 0.311881 -1.285338
H -6.127580 1.264054 0.083487
H -7.600432 -2.369349 0.108927
H -8.010644 -0.830393 -0.686331
H -6.716067 -1.877567 -1.351265
H -3.139755 4.558232 0.216943
H -3.404494 4.292928 -1.549022
H -4.702117 4.928872 -0.528257
H 4.896352 -2.041004 -2.112700
H 6.455963 -1.584298 -1.470544
H 5.516841 -2.845631 -0.654090
H 4.787520 0.771765 1.012179
H 7.434780 -0.471612 0.021179
H 6.850620 1.645414 -1.213276
H 6.733068 2.500586 0.320411
H 9.263738 1.108570 -0.708611
H 8.920110 2.842065 -0.827033
H 9.143325 2.101309 0.759798
H 8.370818 0.373175 2.099896
H 6.949465 -0.598622 2.477808
H 6.847932 1.169682 2.530280
SCF Energy (B3LYP/6-31G**)= -1361.71223711
Number of imaginary frequencies = 0

1b_c102
MMFF Geometry
C 1.911620 1.753757 0.185389
C 2.128001 -0.951206 0.074703
N 3.139952 1.203791 0.339977
C 0.735894 1.030318 -0.027442
C 0.859855 -0.369378 -0.085046
C 3.245103 -0.144124 0.286013
C -0.286522 -1.154633 -0.308547
C -1.551275 -0.569037 -0.458719
C -1.668161 0.820590 -0.385547
C -0.533884 1.620984 -0.187373
C -3.011286 1.420540 -0.526944
C -4.252137 0.527645 -0.337702
C -3.966629 -0.918149 -0.715005
C -2.714617 -1.411409 -0.745181
O -0.615623 2.992004 -0.138656
O -3.112111 2.633023 -0.740493
O -5.253972 1.068448 -1.215127
C -4.713532 0.711737 1.112416
C -5.788757 -0.260523 1.544535
C -7.187272 -0.043488 1.027597
O -5.525217 -1.189586 2.311168
O -5.108414 -1.649217 -0.989220
C -4.989112 -3.042221 -1.247756
C 4.611186 -0.712328 0.476956
C 4.696316 -2.110812 1.026520
C 5.691044 0.045623 0.183371
C 7.153836 -0.332354 0.278734
C 7.838290 -0.064723 -1.076189
C 9.285262 -0.536166 -1.128100
C 7.800869 0.452253 1.421904
H 1.898018 2.840161 0.240606
H 2.234480 -2.029630 0.019068
H -0.185568 -2.238125 -0.366299
H -2.511529 -2.447078 -0.994764
H -1.524799 3.265442 -0.395500
H -5.087385 2.030303 -1.274789
H -5.101853 1.724922 1.277200
H -3.870787 0.589140 1.805906
H -7.879518 -0.695152 1.568528
H -7.486209 0.993543 1.199716
H -7.242745 -0.279334 -0.036218
H -4.407328 -3.222168 -2.157309
H -4.550573 -3.563313 -0.390652
H -5.994931 -3.442058 -1.406651
H 4.508016 -2.846588 0.237820
H 5.669485 -2.335313 1.470571
H 3.965411 -2.261470 1.829387
H 5.523804 1.061424 -0.181814
H 7.263192 -1.399434 0.496048
H 7.283120 -0.581274 -1.870100
H 7.805314 1.005735 -1.316181
H 9.363567 -1.595550 -0.864477
H 9.684475 -0.408981 -2.139605
H 9.920191 0.039424 -0.448471
H 8.831957 0.126990 1.591839
H 7.255329 0.299136 2.360190
H 7.815346 1.527951 1.213221
SCF Energy (B3LYP/6-31G**)= -1361.70946556
Number of imaginary frequencies = 0

1b_c103
MMFF Geometry
C -1.328721 -2.319032 -0.499796
C -2.285071 0.138782 0.158036
N -2.660527 -2.192060 -0.283836
C -0.402020 -1.275718 -0.410045
C -0.908376 -0.007654 -0.067032
C -3.126146 -0.965419 0.034804
C -0.027102 1.084734 0.036974
C 1.348289 0.930803 -0.183222
C 1.843831 -0.334694 -0.509678
C 0.979392 -1.429859 -0.641629
C 3.293643 -0.470978 -0.746431
C 4.239711 0.597254 -0.164826
C 3.573356 1.967396 -0.122529
C 2.232916 2.099491 -0.122366
O 1.436615 -2.674855 -0.999613
O 3.720045 -1.430047 -1.396087
O 5.378049 0.665044 -1.043204
C 4.740728 0.236893 1.235817
C 5.046560 -1.234288 1.380669
C 6.413656 -1.713156 0.972096
O 4.181653 -2.014120 1.782145
O 4.473586 3.014201 -0.035222
C 3.979320 4.346796 -0.073974
C -4.587269 -0.872982 0.267588
C -5.163751 -2.021493 1.051352
C -5.297897 0.163337 -0.226799

C -6.792777 0.385600 -0.122689
C -7.063092 1.595183 0.790206
C -8.542590 1.831550 1.061121
C -7.371961 0.575186 -1.528105
H -1.010675 -3.327326 -0.756831
H -2.696192 1.102999 0.440403
H -0.425981 2.066356 0.290427
H 1.750015 3.068798 -0.065750
H 2.396219 -2.616506 -1.204110
H 5.332075 -0.115684 -1.630673
H 3.988548 0.473764 2.000167
H 5.644462 0.800776 1.497734
H 6.547821 -2.749902 1.293472
H 6.518369 -1.661309 -0.113338
H 7.179445 -1.101342 1.455270
H 3.432224 4.535544 -1.003292
H 3.353642 4.556071 0.799487
H 4.837104 5.025110 -0.043587
H -4.503900 -2.295605 1.882937
H -6.130448 -1.783030 1.502222
H -5.293938 -2.898940 0.409232
H -4.778250 0.930228 -0.801771
H -7.284195 -0.492246 0.308586
H -6.561502 1.440226 1.754418
H -6.631238 2.505670 0.355385
H -9.016354 0.932658 1.467921
H -8.665511 2.638456 1.790830
H -9.075775 2.125097 0.152317
H -8.465313 0.619224 -1.502601
H -7.097110 -0.261239 -2.181023
H -7.007221 1.498389 -1.992371
SCF Energy (B3LYP/6-31G**)= -1361.70443494
Number of imaginary frequencies = 0

1b_c104
MMFF Geometry
C -1.649347 -1.374889 -0.767839
C -2.036176 1.173082 0.089404
N -2.920938 -0.957906 -0.554284
C -0.510975 -0.587730 -0.580404
C -0.722638 0.730018 -0.135832
C -3.110868 0.311372 -0.126694
C 0.383629 1.576781 0.065857
C 1.692437 1.126357 -0.154144
C 1.894188 -0.189643 -0.580478
C 0.803736 -1.039301 -0.812287
C 3.282532 -0.644048 -0.807661
C 4.418442 0.121226 -0.088525
C 4.090401 1.609455 0.015100
C 2.819147 2.052787 -0.000664
O 0.971252 -2.322938 -1.272387
O 3.469859 -1.621570 -1.534014
O 5.627226 -0.035261 -0.843602
C 4.680914 -0.444437 1.309017
C 4.637620 -1.954594 1.334542
C 5.842365 -2.707414 0.836651
O 3.625043 -2.539679 1.723350
O 5.210294 2.409450 0.146743
C 5.036007 3.814924 0.283930
C -4.525138 0.736879 0.083798
C -4.822656 2.205947 -0.049974
C -5.465738 -0.196711 0.349156
C -6.940880 0.013170 0.615835
C -7.753771 -0.501649 -0.585486
C -9.246542 -0.224858 -0.470655
C -7.321002 -0.692083 1.921759
H -1.566644 -2.404963 -1.108260
H -2.208340 2.183634 0.444640
H 0.214622 2.601084 0.396128
H 2.571293 3.104441 0.093265
H 1.924746 -2.468983 -1.466981
H 5.531251 0.502499 -1.648821
H 3.926455 -0.096061 2.026858
H 5.656634 -0.127459 1.697505
H 5.771416 -3.753409 1.148177
H 5.887924 -2.662568 -0.253170
H 6.750806 -2.282073 1.270380
H 4.552561 4.236969 -0.602915
H 4.469849 4.051705 1.190443
H 6.026813 4.269344 0.376550
H -4.283210 2.639195 -0.900138
H -5.879811 2.410223 -0.238798
H -4.532963 2.742043 0.859710
H -5.155600 -1.243226 0.393495
H -7.158661 1.076798 0.756144
H -7.388689 -0.020946 -1.502505
H -7.600995 -1.580677 -0.715474
H -9.437526 0.840594 -0.308520
H -9.754840 -0.523060 -1.393363
H -9.697837 -0.788373 0.351027
H -8.351127 -0.460931 2.210624
H -6.673797 -0.367755 2.744808
H -7.231295 -1.780565 1.832019
SCF Energy (B3LYP/6-31G**)= -1361.70073624
Number of imaginary frequencies = 0

1b_c105
MMFF Geometry
C -1.511532 -2.298796 -0.056605
C -2.411885 0.242491 -0.401574
N -2.842374 -2.114772 -0.233490
C -0.559067 -1.275178 -0.037560
C -1.036649 0.036629 -0.218020
C -3.279811 -0.847713 -0.396810
C -0.128307 1.111332 -0.204618
C 1.245563 0.897464 -0.028674
C 1.714048 -0.407642 0.134758
C 0.821215 -1.488874 0.150099
C 3.166721 -0.607736 0.319888
C 4.123050 0.485100 -0.216645
C 3.496287 1.869217 -0.022755
C 2.161556 2.038073 0.045917
O 1.252157 -2.779035 0.347927
O 3.542139 -1.653849 0.853861
O 4.282264 0.329463 -1.630279
C 5.481738 0.377305 0.505959
C 6.193563 -0.926810 0.203455
C 7.054088 -1.506174 1.293113
O 6.114765 -1.456316 -0.907458
O 4.421988 2.898313 0.024594
C 3.951271 4.240717 0.004523
C -4.741119 -0.692358 -0.593054
C -5.356996 -1.724532 -1.499075
C -5.416970 0.293557 0.035114
C -6.906162 0.566933 -0.014908
C -7.474941 0.552654 1.417310
C -8.990671 0.688614 1.468968
C -7.145136 1.902624 -0.722414
H -1.216609 -3.337895 0.073811
H -2.800832 1.243398 -0.560973
H -0.502466 2.126228 -0.333511
H 1.706697 3.016006 0.153110
H 2.206585 -2.757226 0.585283
H 4.845865 -0.462907 -1.769375
H 5.340976 0.461060 1.590279
H 6.174832 1.164477 0.185801
H 7.496566 -2.446280 0.952420
H 7.854304 -0.803993 1.538946
H 6.442217 -1.706606 2.176079
H 3.361552 4.436903 -0.896781
H 3.376063 4.465641 0.908230
H 4.822667 4.901976 -0.012171
H -5.496506 -2.670740 -0.965723
H -6.325395 -1.414035 -1.899138
H -4.717263 -1.909073 -2.370154
H -4.868608 0.970130 0.691207
H -7.423583 -0.211510 -0.583985
H -7.200901 -0.391569 1.905840
H -7.026121 1.356467 2.014800
H -9.475585 -0.079840 0.858834
H -9.342048 0.573782 2.499519
H -9.318382 1.670841 1.116582

H -8.213528 2.084406 -0.875155
H -6.671320 1.912630 -1.710775
H -6.738189 2.741151 -0.146079
SCF Energy (B3LYP/6-31G**)= -1361.71226734
Number of imaginary frequencies = 0

1b_c106
MMFF Geometry
C -1.393557 -2.209020 -0.744769
C -2.262899 0.359931 -0.564140
N -2.730709 -1.988739 -0.747927
C -0.419623 -1.209524 -0.656251
C -0.881405 0.117352 -0.565877
C -3.151955 -0.709893 -0.647641
C 0.049082 1.170483 -0.489325
C 1.428109 0.921209 -0.484242
C 1.877122 -0.400451 -0.556575
C 0.966924 -1.460976 -0.661052
C 3.333201 -0.638114 -0.559487
C 4.260731 0.458236 -0.000751
C 3.688789 1.849541 -0.246214
C 2.372901 2.043181 -0.458721
O 1.383741 -2.764900 -0.776358
O 3.778823 -1.701101 -1.001274
O 5.510456 0.342510 -0.705573
C 4.540083 0.284506 1.494044
C 4.723496 -1.162139 1.884468
C 6.098268 -1.759505 1.749023
O 3.761259 -1.833023 2.260474
O 4.637171 2.854251 -0.175711
C 4.244051 4.188236 -0.471624
C -4.621541 -0.514078 -0.657483
C -5.354441 -1.376034 -1.650145
C -5.203323 0.356786 0.194891
C -6.682395 0.644077 0.352263
C -7.097492 0.391471 1.814740
C -8.595596 0.526331 2.051572
C -6.961767 2.081645 -0.091700
H -1.112078 -3.257426 -0.818881
H -2.642418 1.375721 -0.512884
H -0.313541 2.196395 -0.435142
H 1.953284 3.031911 -0.608051
H 2.364186 -2.782500 -0.843334
H 5.492660 -0.513014 -1.179422
H 3.708107 0.666186 2.100880
H 5.435399 0.837680 1.803410
H 6.117112 -2.742891 2.227273
H 6.353738 -1.872748 0.693689
H 6.830187 -1.119948 2.248490
H 3.843347 4.263027 -1.487702
H 3.520274 4.553581 0.263816
H 5.133456 4.822456 -0.412032
H -5.459803 -2.397321 -1.269211
H -6.351833 -0.996876 -1.886145
H -4.814849 -1.416045 -2.603734
H -4.572728 0.909381 0.892035
H -7.275708 -0.023598 -0.279975
H -6.796724 -0.623467 2.105864
H -6.568791 1.079843 2.486429
H -9.160646 -0.124830 1.377306
H -8.839287 0.241155 3.080152
H -8.934470 1.556047 1.906008
H -8.035538 2.293398 -0.095748
H -6.594465 2.255600 -1.109682
H -6.476075 2.808069 0.569591
SCF Energy (B3LYP/6-31G**)= -1361.70449501
Number of imaginary frequencies = 0

1b_c107
MMFF Geometry
C -1.627801 -1.553813 -0.384283
C -2.051565 1.011816 0.399786
N -2.895877 -1.175354 -0.092404
C -0.511014 -0.717977 -0.314158
C -0.742014 0.609167 0.091148
C -3.103739 0.102291 0.300438
C 0.340357 1.505790 0.168754
C 1.646010 1.095726 -0.133498
C 1.868872 -0.229866 -0.517519
C 0.799789 -1.129664 -0.627984
C 3.253493 -0.641291 -0.832488
C 4.408265 0.198924 -0.237769
C 4.027801 1.676848 -0.173412
C 2.741791 2.070391 -0.108793
O 0.984361 -2.425272 -1.046110
O 3.425249 -1.643515 -1.528391
O 5.561478 0.053456 -1.077251
C 4.799633 -0.290919 1.158249
C 4.819738 -1.798791 1.252915
C 6.012351 -2.528825 0.695455
O 3.864521 -2.402185 1.745139
O 5.120373 2.524007 -0.164728
C 4.898942 3.926791 -0.076824
C -4.509925 0.475699 0.630117
C -4.703593 1.597164 1.614687
C -5.527423 -0.213229 0.067291
C -7.015147 0.005817 0.240832
C -7.671751 0.162945 -1.144833
C -9.150381 0.520542 -1.078064
C -7.604676 -1.161977 1.034438
H -1.529278 -2.593681 -0.688827
H -2.241317 2.036548 0.701432
H 0.154835 2.537062 0.466913
H 2.458444 3.115108 -0.041543
H 1.925670 -2.543957 -1.307268
H 5.382367 0.549381 -1.895031
H 4.088778 0.062274 1.917074
H 5.788524 0.079810 1.455035
H 6.933333 -2.050780 1.038336
H 6.007556 -3.561287 1.056268
H 5.972852 -2.532528 -0.395517
H 4.332107 4.287672 -0.941002
H 4.394327 4.184663 0.859782
H 5.874232 4.422326 -0.082191
H -4.570462 2.567166 1.124546
H -5.692278 1.588539 2.080627
H -3.988372 1.520413 2.441782
H -5.281088 -1.032528 -0.611995
H -7.206694 0.926572 0.800653
H -7.154690 0.954879 -1.702349
H -7.555893 -0.758717 -1.729368
H -9.310675 1.420622 -0.476329
H -9.534698 0.713937 -2.084835
H -9.741997 -0.293938 -0.650339
H -8.658297 -0.986883 1.273272
H -7.076135 -1.295985 1.985294
H -7.535937 -2.102490 0.476232
SCF Energy (B3LYP/6-31G**)= -1361.70075663
Number of imaginary frequencies = 0

1b_c108
MMFF Geometry
C -1.714032 -2.588469 0.065083
C -2.399618 0.040800 0.143430
N -3.015758 -2.277000 0.275088
C -0.688674 -1.654829 -0.114393
C -1.055122 -0.296243 -0.070794
C -3.347159 -0.968279 0.304150
C -0.069600 0.692535 -0.248106
C 1.273180 0.348884 -0.453233
C 1.632246 -1.001412 -0.481393
C 0.660085 -2.000582 -0.331017
C 3.059203 -1.344573 -0.676112
C 4.113980 -0.263668 -0.345159
C 3.584243 1.124130 -0.689310
C 2.266299 1.396008 -0.705583
O 0.983174 -3.335260 -0.384210
O 3.336821 -2.489681 -1.037459
O 5.289748 -0.538663 -1.115163
C 4.473844 -0.435661 1.135160
C 5.304581 0.701492 1.685482
C 6.768870 0.741064 1.334387

O 4.791065 1.573721 2.389989
O 4.585014 2.039216 -0.960044
C 4.240744 3.418238 -1.036976
C -4.780222 -0.673112 0.540934
C -5.429592 -1.541949 1.584629
C -5.410042 0.292375 -0.162309
C -6.870142 0.681648 -0.073396
C -7.066398 1.983250 0.727820
C -6.416079 3.226489 0.132925
C -7.477377 0.767567 -1.477249
H -1.506851 -3.656413 0.045552
H -2.702974 1.081803 0.198726
H -0.359707 1.742638 -0.226604
H 1.882501 2.385485 -0.929154
H 1.917940 -3.422208 -0.681926
H 5.101092 -0.265007 -2.029864
H 5.032322 -1.365953 1.299472
H 3.571823 -0.506430 1.757192
H 7.281985 1.443923 1.997024
H 7.211206 -0.248015 1.477323
H 6.900955 1.067387 0.301316
H 3.604284 3.612212 -1.905991
H 3.758969 3.754935 -0.113255
H 5.165708 3.988579 -1.163091
H -4.764736 -1.679516 2.445455
H -6.350129 -1.106497 1.982540
H -5.669233 -2.527179 1.171064
H -4.845668 0.848372 -0.910778
H -7.432167 -0.101470 0.448027
H -8.141671 2.173947 0.835515
H -6.677159 1.840796 1.744227
H -6.824773 3.460613 -0.854185
H -6.604699 4.089264 0.780430
H -5.331866 3.111916 0.043259
H -8.529912 1.066181 -1.426506
H -7.432193 -0.206686 -1.977395
H -6.950631 1.487424 -2.112696
SCF Energy (B3LYP/6-31G**)= -1361.70139586
Number of imaginary frequencies = 0

1b_c109
MMFF Geometry
C 1.812224 2.464587 0.290482
C 2.401550 0.000185 -0.691610
N 3.118036 2.132037 0.149725
C 0.736531 1.631184 -0.031660
C 1.053768 0.358728 -0.542262
C 3.400712 0.901903 -0.329709
C 0.015637 -0.521566 -0.898941
C -1.329422 -0.161111 -0.738991
C -1.637002 1.097348 -0.217317
C -0.615906 1.997480 0.120648
C -3.059872 1.453990 -0.037710
C -4.116145 0.332811 -0.030179
C -3.678949 -0.856647 -0.871679
C -2.385647 -1.079120 -1.171015
O -0.890418 3.252429 0.609136
O -3.367105 2.634754 0.155507
O -5.301898 0.916385 -0.595420
C -4.386493 -0.011404 1.438841
C -5.235338 -1.247914 1.635463
C -6.709888 -1.147083 1.342685
O -4.732220 -2.298112 2.040788
O -4.728563 -1.670188 -1.258709
C -4.444528 -2.876333 -1.955927
C 4.841503 0.577617 -0.472453
C 5.678089 1.709394 -1.007226
C 5.306824 -0.633054 -0.095794
C 6.732177 -1.138316 -0.157455
C 7.408334 -1.118001 1.227597
C 6.781490 -2.022626 2.281617
C 6.765342 -2.528917 -0.799188
H 1.645547 3.464059 0.686699
H 2.669974 -0.969508 -1.099727
H 0.265724 -1.500532 -1.307085
H -2.070809 -1.937611 -1.754155
H -1.857461 3.416828 0.537207
H -5.288645 1.865708 -0.360746
H -4.890617 0.817545 1.951870
H -3.444137 -0.178510 1.977561
H -7.215235 -2.044880 1.710197
H -7.129254 -0.280677 1.860272
H -6.881724 -1.063058 0.268529
H -3.979597 -2.667253 -2.924568
H -3.815577 -3.538607 -1.352359
H -5.393049 -3.388936 -2.141219
H 5.725016 2.531954 -0.285169
H 6.707434 1.421423 -1.229410
H 5.250953 2.091918 -1.941320
H 4.604876 -1.348169 0.334050
H 7.331503 -0.493949 -0.808562
H 8.461343 -1.403992 1.110647
H 7.411096 -0.089136 1.610129
H 6.820097 -3.074184 1.983360
H 7.329864 -1.927730 3.224772
H 5.739533 -1.753567 2.477586
H 7.789138 -2.916736 -0.831125
H 6.393569 -2.487260 -1.829448
H 6.145875 -3.250350 -0.255770
SCF Energy (B3LYP/6-31G**)= -1361.70799948
Number of imaginary frequencies = 0

1b_c110
MMFF Geometry
C -1.759961 -1.321907 -0.723860
C -2.176554 1.159415 0.298335
N -3.036363 -0.934762 -0.484845
C -0.630548 -0.536017 -0.483849
C -0.858028 0.747076 0.045923
C -3.241222 0.301648 0.024989
C 0.237992 1.590610 0.306047
C 1.551574 1.171616 0.056353
C 1.770385 -0.110536 -0.457061
C 0.689867 -0.956455 -0.743031
C 3.161981 -0.528514 -0.728033
C 4.320490 0.231637 -0.049540
C 3.939504 1.677104 0.218207
C 2.665452 2.087801 0.300882
O 0.870797 -2.206987 -1.282784
O 3.378297 -1.471377 -1.496251
O 5.414794 0.193293 -0.982459
C 4.774296 -0.423273 1.257617
C 4.740975 -1.931432 1.199521
C 5.969393 -2.642287 0.698841
O 3.722438 -2.546558 1.517070
O 4.944459 2.557670 0.594912
C 5.679819 3.051282 -0.524609
C -4.660909 0.696276 0.259934
C -4.972478 2.168139 0.226026
C -5.590248 -0.264657 0.459591
C -7.068885 -0.098050 0.730521
C -7.922225 -0.674027 -0.414589
C -7.742109 0.070317 -1.731739
C -7.419613 -0.798085 2.047942
H -1.665669 -2.326213 -1.131390
H -2.359494 2.142784 0.718675
H 0.059203 2.587738 0.707245
H 2.441872 3.113515 0.580729
H 1.825987 -2.337567 -1.473485
H 5.242358 -0.556969 -1.587901
H 4.123529 -0.134313 2.093860
H 5.790972 -0.113234 1.528864
H 5.848763 -3.720547 0.836290
H 6.114167 -2.434423 -0.363071
H 6.842319 -2.316773 1.270009
H 6.520266 2.388976 -0.748861
H 5.048901 3.186907 -1.410277
H 6.091140 4.027012 -0.248796
H -4.445237 2.660507 -0.599254
H -6.032569 2.377175 0.063127
H -4.677423 2.645900 1.166031
H -5.264886 -1.307277 0.439931

H -7.331025 0.956890 0.856991
H -7.689093 -1.735265 -0.568693
H -8.981989 -0.623036 -0.134855
H -6.723110 -0.032433 -2.116727
H -8.424478 -0.333339 -2.486685
H -7.961853 1.136039 -1.612845
H -8.478807 -0.658382 2.290068
H -6.832543 -0.388986 2.877926
H -7.226743 -1.875769 1.996598
SCF Energy (B3LYP/6-31G**)= -1361.70071236
Number of imaginary frequencies = 0

1b_c111
MMFF Geometry
C -1.470023 -2.184221 -0.809256
C -2.309924 0.395099 -0.643284
N -2.804453 -1.947748 -0.823725
C -0.484911 -1.196242 -0.716504
C -0.931391 0.136217 -0.633984
C -3.211623 -0.664194 -0.729823
C 0.011039 1.178412 -0.555114
C 1.386787 0.912206 -0.538473
C 1.821848 -0.414999 -0.601596
C 0.898530 -1.464322 -0.709993
C 3.277461 -0.673064 -0.583566
C 4.195511 0.416687 0.018756
C 3.653409 1.812191 -0.284231
C 2.345177 2.022411 -0.523368
O 1.300325 -2.773586 -0.819506
O 3.689457 -1.748338 -1.022093
O 5.500309 0.290285 -0.562044
C 4.354453 0.245593 1.531184
C 4.517897 -1.202325 1.931048
C 5.868692 -1.840872 1.747605
O 3.555030 -1.839253 2.362353
O 4.625520 2.794974 -0.259263
C 4.241590 4.144433 -0.496262
C -4.679272 -0.446903 -0.751765
C -5.415013 -1.308637 -1.743178
C -5.249839 0.430938 0.101452
C -6.717721 0.775620 0.231925
C -7.361255 0.091178 1.453954
C -6.802535 0.501886 2.810933
C -6.895940 2.296888 0.258031
H -1.200439 -3.236192 -0.877561
H -2.677759 1.415486 -0.597920
H -0.339869 2.208734 -0.508698
H 1.943540 3.010674 -0.719065
H 2.281880 -2.800410 -0.884509
H 5.434077 0.603134 -1.481026
H 3.474129 0.622715 2.068471
H 5.219620 0.801637 1.912925
H 5.904263 -2.781865 2.303891
H 6.045733 -2.045357 0.689947
H 6.646917 -1.181663 2.140175
H 3.820339 4.260161 -1.500001
H 3.537892 4.490278 0.267654
H 5.139843 4.765715 -0.433633
H -5.369073 -2.363178 -1.449912
H -6.471327 -1.051346 -1.842484
H -4.972308 -1.206486 -2.740613
H -4.610373 0.950545 0.815519
H -7.265692 0.425282 -0.648618
H -8.438815 0.298842 1.448150
H -7.258491 -0.996811 1.350905
H -6.946987 1.569631 2.998755
H -7.320522 -0.045092 3.605769
H -5.736007 0.274002 2.894944
H -7.952457 2.561433 0.373656
H -6.542401 2.742811 -0.678746
H -6.337361 2.765021 1.075559
SCF Energy (B3LYP/6-31G**)= -1361.69943453
Number of imaginary frequencies = 0

1b_c112
MMFF Geometry
C 1.724981 -1.541282 0.467809
C 2.206952 0.909129 -0.599277
N 3.003362 -1.221748 0.151746
C 0.625025 -0.699630 0.287063
C 0.886546 0.567699 -0.264544
C 3.240312 -0.001302 -0.381925
C -0.177353 1.467525 -0.461702
C -1.493485 1.118922 -0.130570
C -1.747677 -0.148783 0.402105
C -0.697504 -1.049429 0.627675
C -3.140326 -0.492310 0.758854
C -4.296305 0.326356 0.147432
C -3.856967 1.749205 -0.150094
C -2.570510 2.092094 -0.311922
O -0.910436 -2.286807 1.185771
O -3.358913 -1.420868 1.543670
O -5.332882 0.347608 1.144586
C -4.861971 -0.306256 -1.126665
C -4.903030 -1.814007 -1.063543
C -6.133470 -2.459497 -0.485308
O -3.938958 -2.481518 -1.439891
O -4.835709 2.680531 -0.469280
C -5.469110 3.219008 0.691175
C 4.657254 0.306929 -0.732306
C 4.884647 1.311018 -1.829818
C 5.654296 -0.333304 -0.082284
C 7.146660 -0.160474 -0.255907
C 7.838595 0.142186 1.087767
C 7.405591 1.469362 1.698748
C 7.732767 -1.432261 -0.873764
H 1.603439 -2.537225 0.888442
H 2.418884 1.889155 -1.013834
H 0.028838 2.453011 -0.878222
H -2.310789 3.104129 -0.609780
H -1.857987 -2.366635 1.433926
H -5.163666 -0.409211 1.742716
H -4.249211 -0.052765 -2.002207
H -5.875890 0.055601 -1.337268
H -6.077227 -3.542785 -0.624632
H -6.202009 -2.242622 0.582443
H -7.021802 -2.090206 -1.003654
H -6.325060 2.602175 0.977754
H -4.774473 3.328617 1.531604
H -5.848117 4.212462 0.432894
H 4.762423 2.330663 -1.449950
H 5.879254 1.235961 -2.276577
H 4.179561 1.156335 -2.654689
H 5.384574 -1.065724 0.681836
H 7.371308 0.670502 -0.931827
H 7.646600 -0.663374 1.807821
H 8.924625 0.173540 0.934348
H 6.344821 1.462302 1.967077
H 7.977995 1.666717 2.610822
H 7.581175 2.296531 1.003599
H 8.810191 -1.322134 -1.037899
H 7.269672 -1.646650 -1.843510
H 7.579014 -2.303087 -0.226284
SCF Energy (B3LYP/6-31G**)= -1361.70079454
Number of imaginary frequencies = 0

1b_c113
MMFF Geometry
C -1.707479 -1.822379 -0.261615
C -2.037901 0.866658 -0.087526
N -2.967835 -1.342004 -0.129504
C -0.554325 -1.035010 -0.317529
C -0.737773 0.355076 -0.228705
C -3.127454 -0.001762 -0.040468
C 0.384045 1.201915 -0.286481
C 1.683422 0.688528 -0.424851
C 1.861145 -0.695757 -0.504361
C 0.749331 -1.554134 -0.458705
C 3.230964 -1.237626 -0.653939
C 4.435714 -0.283755 -0.595613
C 4.085718 1.191365 -0.564369
C 2.813235 1.620185 -0.488553

O   0.880766 -2.920346 -0.554135
O   3.381906 -2.447276 -0.862838
O   5.133841 -0.527900 -1.840269
C   5.384223 -0.701018 0.535599
C   4.850381 -0.357421 1.907032
C   5.868266 -0.037912 2.969169
O   3.647669 -0.388686 2.165632
O   5.199894 2.013915 -0.594764
C   5.004272 3.418289 -0.706234
C -4.525269 0.492188 0.123635
C -4.697346 1.763133 0.907543
C -5.542450 -0.231313 -0.395156
C -7.034407 0.033271 -0.411355
C -7.680135 -0.349543 0.932529
C -9.202005 -0.301105 0.907657
C -7.408105 1.437576 -0.895830
H -1.646386 -2.906733 -0.323884
H -2.190987 1.939277 -0.029693
H   0.236812 2.279249 -0.219354
H   2.567794 2.675437 -0.453003
H   1.832159 -3.133595 -0.683147
H   5.189228 -1.497836 -1.939154
H   6.356199 -0.209011 0.402049
H   5.586226 -1.778865 0.514759
H   5.360058 0.191868 3.909752
H   6.522545 -0.899975 3.119822
H   6.453773 0.833323 2.665135
H   4.445199 3.666542 -1.614211
H   4.505152 3.814022 0.183967
H   5.988762 3.890160 -0.776797
H -4.516786 2.636326 0.272241
H -5.694640 1.857513 1.341444
H -4.005274 1.799484 1.756859
H -5.286018 -1.161108 -0.909528
H -7.439397 -0.653497 -1.169412
H -7.374165 -1.368043 1.204760
H -7.322928 0.306698 1.734227
H -9.602495 -0.928816 0.105453
H -9.604407 -0.668149 1.857379
H -9.567920 0.720154 0.766195
H -8.452912 1.463596 -1.224531
H -6.794458 1.738765 -1.752072
H -7.299292 2.192895 -0.112991
SCF Energy (B3LYP/6-31G**)= -1361.70350837
Number of imaginary frequencies = 0

1b_c114
MMFF Geometry
C -1.498385 -2.429431 -0.397006
C -2.295937 0.102517 0.179800
N -2.820868 -2.210449 -0.196933
C -0.505460 -1.446278 -0.332251
C -0.930653 -0.140484 -0.032223
C -3.206967 -0.947199 0.082666
C   0.020748 0.891262 0.050361
C   1.385758 0.645594 -0.164356
C   1.808948 -0.655002 -0.458481
C   0.866760 -1.695489 -0.545064
C   3.252507 -0.917002 -0.694770
C   4.261232 0.255185 -0.613200
C   3.637943 1.595795 -0.281052
C   2.328118 1.756921 -0.058431
O   1.234298 -2.987134 -0.845036
O   3.594408 -2.061088 -1.010971
O   4.847548 0.359587 -1.928466
C   5.401446 -0.128568 0.339486
C   4.967193 -0.233421 1.785323
C   6.057049 -0.141739 2.820565
O   3.797552 -0.432879 2.111304
O   4.479990 2.674527 -0.037442
C   5.134010 3.153259 -1.211461
C -4.660174 -0.752776 0.302169
C -5.314958 -1.838505 1.113356
C -5.298427 0.313919 -0.225254
C -6.775771 0.637106 -0.137366
C -6.970149 1.886070 0.741182
C -8.432138 2.226770 0.995523
C -7.333942 0.826537 -1.551281
H -1.245037 -3.463695 -0.620200
H -2.643036 1.099466 0.432943
H -0.311933 1.900974 0.289545
H   1.947766 2.729014 0.243223
H   2.212548 -3.016333 -0.952316
H   4.116579 0.437452 -2.565955
H   6.209797 0.610164 0.271154
H   5.844463 -1.090709 0.053615
H   5.623845 -0.227907 3.820918
H   6.772070 -0.954485 2.671743
H   6.560374 0.824762 2.738356
H   6.012753 2.542736 -1.437254
H   4.453810 3.189849 -2.069432
H   5.479657 4.171407 -1.009058
H -4.679212 -2.131327 1.957251
H -6.266582 -1.524881 1.550045
H -5.498580 -2.723478 0.495060
H -4.726079 1.029144 -0.816557
H -7.326392 -0.194458 0.313659
H -6.484850 1.724414 1.712605
H -6.476794 2.754017 0.285306
H -8.966401 1.372397 1.422790
H -8.505147 3.059411 1.702673
H -8.940080 2.530248 0.075591
H -8.422101 0.943317 -1.534490
H -7.111700 -0.043509 -2.179837
H -6.906545 1.710794 -2.037390
SCF Energy (B3LYP/6-31G**)= -1361.70032525
Number of imaginary frequencies = 0

1b_c115
MMFF Geometry
C   1.723437 1.779816 -0.321897
C   2.069361 -0.896860 -0.027220
N   2.988018 1.311134 -0.187959
C   0.573186 0.986297 -0.322624
C   0.764896 -0.397697 -0.171774
C   3.155364 -0.022848 -0.038382
C -0.352975 -1.251234 -0.171979
C -1.655234 -0.749138 -0.315153
C -1.843200 0.630051 -0.455432
C -0.735048 1.493733 -0.467026
C -3.219877 1.159516 -0.612104
C -4.424265 0.205008 -0.503614
C -4.046398 -1.257711 -0.425919
C -2.781203 -1.681542 -0.312283
O -0.874086 2.853406 -0.624173
O -3.380218 2.357030 -0.877265
O -5.140681 0.419095 -1.741255
C -5.350235 0.643428 0.636201
C -4.772762 0.333060 1.997580
C -5.718990 -0.211288 3.033437
O -3.591696 0.556059 2.262292
O -5.069412 -2.188364 -0.288146
C -5.767898 -2.408308 -1.513884
C   4.557954 -0.502927 0.126603
C   4.748021 -1.735929 0.965129
C   5.563692 0.200182 -0.440330
C   7.056470 -0.058808 -0.467274
C   7.720633 0.387458 0.847744
C   9.242167 0.344119 0.801762
C   7.429472 -1.482184 -0.893080
H   1.656371 2.859849 -0.433425
H   2.228099 -1.965016 0.078753
H -0.201841 -2.323963 -0.055987
H -2.574716 -2.740798 -0.188887
H -1.829044 3.058458 -0.737826
H -5.171207 1.387772 -1.873670
H -6.322457 0.144072 0.537191
H -5.561410 1.718942 0.597255
H -5.178536 -0.394999 3.966108
H -6.513751 0.514915 3.220288
H -6.143329 -1.155036 2.681908
H -6.543922 -1.650039 -1.650947

H -5.090747 -2.432936 -2.374927
H -6.263349 -3.381400 -1.444479
H 4.069563 -1.735843 1.826126
H 4.561449 -2.638035 0.373459
H 5.752411 -1.806581 1.386995
H 5.295050 1.104350 -0.992704
H 7.446694 0.594363 -1.261889
H 7.413979 1.416054 1.078010
H 7.378736 -0.233008 1.683860
H 9.627479 0.936248 -0.034216
H 9.657148 0.755684 1.727518
H 9.610749 -0.681031 0.701314
H 8.469301 -1.518896 -1.236230
H 6.804386 -1.824530 -1.725243
H 7.336103 -2.201523 -0.075147
SCF Energy (B3LYP/6-31G**)= -1361.69786467
Number of imaginary frequencies = 0

1b_c116
MMFF Geometry
C 1.880868 -1.515038 -0.299630
C 2.326268 1.156851 -0.487595
N 3.163087 -1.085053 -0.384052
C 0.758976 -0.682846 -0.301792
C 1.001674 0.698341 -0.399338
C 3.382577 0.246885 -0.477149
C -0.086927 1.589048 -0.410437
C -1.406691 1.125505 -0.315039
C -1.644252 -0.246697 -0.209648
C -0.569181 -1.149794 -0.216022
C -3.045374 -0.723944 -0.125913
C -4.161306 0.274408 0.277521
C -3.763583 1.702453 -0.072625
C -2.511564 2.076645 -0.367216
O -0.763484 -2.509108 -0.141748
O -3.257406 -1.920242 -0.343694
O -4.317840 0.242568 1.698387
C -5.465453 -0.115941 -0.449413
C -6.018663 -1.446171 0.022706
C -6.700729 -2.309184 -1.003630
O -5.959821 -1.776853 1.209115
O -4.771250 2.657123 -0.157553
C -5.217040 3.063041 1.138790
C 4.807377 0.679744 -0.583178
C 5.067108 1.989313 -1.277849
C 5.777297 -0.115961 -0.079480
C 7.271989 0.115573 -0.060231
C 7.809136 0.280542 1.376765
C 7.688542 -0.942731 2.277489
C 7.981840 -1.006830 -0.821226
H 1.775272 -2.595348 -0.226830
H 2.521180 2.222339 -0.550060
H 0.100869 2.659128 -0.493190
H -2.298427 3.109057 -0.628597
H -1.729543 -2.690696 -0.183758
H -4.754875 -0.610110 1.918812
H -5.292580 -0.167576 -1.531307
H -6.267003 0.609765 -0.269081
H -7.038596 -3.238746 -0.537360
H -7.565583 -1.779291 -1.409927
H -5.997049 -2.553757 -1.803140
H -5.864106 2.301069 1.582899
H -4.375502 3.290655 1.802384
H -5.809418 3.974682 1.016420
H 4.847048 2.828953 -0.610266
H 6.098688 2.098093 -1.619922
H 4.447419 2.083970 -2.177087
H 5.472909 -1.055672 0.385719
H 7.518338 1.047257 -0.578581
H 8.868371 0.563221 1.324612
H 7.291747 1.120077 1.858882
H 8.246340 -1.793967 1.876924
H 8.100570 -0.715868 3.266499
H 6.646112 -1.242557 2.416891
H 9.068202 -0.871855 -0.787750
H 7.679891 -1.009209 -1.874878
H 7.749178 -1.995438 -0.411158
SCF Energy (B3LYP/6-31G**)= -1361.70689630
Number of imaginary frequencies = 0

1b_c117
MMFF Geometry
C 1.521546 -2.461608 -0.133719
C 2.518879 0.057745 -0.347072
N 2.867793 -2.312746 -0.176346
C 0.599415 -1.412002 -0.192129
C 1.127222 -0.112056 -0.306209
C 3.352852 -1.056153 -0.271523
C 0.250617 0.985813 -0.386504
C -1.138789 0.809530 -0.335426
C -1.655028 -0.481039 -0.203270
C -0.798025 -1.589774 -0.151412
C -3.123469 -0.641394 -0.152660
C -3.969132 0.570225 0.309520
C -3.345272 1.870187 -0.207346
C -2.030173 1.962297 -0.484453
O -1.281683 -2.873459 -0.064303
O -3.594347 -1.744014 -0.440588
O -3.929150 0.665211 1.736859
C -5.418855 0.404667 -0.191135
C -6.109297 -0.794365 0.428955
C -7.129178 -1.509522 -0.414793
O -5.884767 -1.130554 1.593975
O -4.245088 2.918112 -0.309121
C -3.746468 4.220492 -0.590565
C 4.829808 -0.938775 -0.316471
C 5.510521 -1.976993 -1.167517
C 5.465373 0.025224 0.383640
C 6.959210 0.248731 0.481387
C 7.418196 1.431081 -0.393837
C 6.843051 2.791040 -0.016770
C 7.370269 0.418405 1.947384
H 1.187653 -3.493526 -0.047816
H 2.948100 1.049463 -0.451026
H 0.662065 1.989239 -0.488827
H -1.573114 2.885180 -0.822282
H -2.259519 -2.847248 -0.168639
H -4.484576 -0.064889 2.088008
H -5.431975 0.296133 -1.282293
H -6.041567 1.266301 0.077823
H -7.539507 -2.356347 0.142141
H -7.940333 -0.822660 -0.667943
H -6.654118 -1.885434 -1.324296
H -3.030791 4.540834 0.173498
H -3.300211 4.259636 -1.589297
H -4.591749 4.914801 -0.572980
H 4.960861 -2.135260 -2.102825
H 6.522850 -1.685106 -1.459633
H 5.571085 -2.932151 -0.635401
H 4.871686 0.712835 0.986008
H 7.491838 -0.639199 0.122348
H 8.512914 1.496304 -0.354932
H 7.160603 1.223627 -1.440496
H 7.134718 3.084032 0.995809
H 7.221097 3.556398 -0.702722
H 5.751078 2.800138 -0.081554
H 8.447454 0.598768 2.029354
H 7.140638 -0.487685 2.519773
H 6.848908 1.252137 2.429590
SCF Energy (B3LYP/6-31G**)= -1361.71121230
Number of imaginary frequencies = 0

1b_c118
MMFF Geometry
C 2.029779 1.597136 -0.168225
C 2.144050 -1.113889 -0.281169
N 3.246293 1.004976 -0.100889
C 0.817370 0.914638 -0.290839
C 0.888215 -0.488709 -0.348215
C 3.301157 -0.345864 -0.157745
C -0.298486 -1.234384 -0.476757
C -1.549967 -0.605506 -0.535476

C -1.612706 0.787759 -0.465276
C -0.439614 1.548762 -0.359248
C -2.940990 1.433951 -0.509994
C -4.194474 0.586487 -0.221341
C -3.988477 -0.870826 -0.605939
C -2.760184 -1.408095 -0.725819
O -0.469882 2.922013 -0.314206
O -3.015331 2.648121 -0.724888
O -5.239338 1.156959 -1.026873
C -4.539901 0.795754 1.257342
C -5.614666 -0.133703 1.776148
C -7.038789 0.131017 1.361449
O -5.328966 -1.066902 2.529702
O -5.172351 -1.562982 -0.786761
C -5.121344 -2.961338 -1.038818
C 4.659896 -0.958799 -0.096231
C 4.827574 -2.314413 -0.728461
C 5.672408 -0.274349 0.481145
C 7.106282 -0.718777 0.666499
C 8.042919 -0.085264 -0.380307
C 8.149564 1.434218 -0.333485
C 7.563088 -0.433782 2.100919
H 2.057956 2.683508 -0.118224
H 2.206148 -2.196719 -0.312545
H -0.239866 -2.321080 -0.532789
H -2.612633 -2.451885 -0.980522
H -1.385706 3.226038 -0.504400
H -5.044974 2.112169 -1.106051
H -4.877948 1.823465 1.441815
H -3.652921 0.646275 1.887500
H -7.712365 -0.489948 1.959092
H -7.286119 1.179823 1.543819
H -7.181515 -0.111320 0.307263
H -4.616501 -3.168942 -1.987591
H -4.637690 -3.490858 -0.211672
H -6.149562 -3.326546 -1.117428
H 4.298777 -2.367569 -1.687081
H 5.870700 -2.551595 -0.954437
H 4.439861 -3.097790 -0.069023
H 5.458962 0.718006 0.883481
H 7.175801 -1.805542 0.542668
H 9.048761 -0.506000 -0.256061
H 7.709947 -0.378836 -1.384188
H 8.552787 1.779541 0.622759
H 8.825952 1.782730 -1.121050
H 7.179332 1.912648 -0.495976
H 8.605995 -0.736771 2.242812
H 6.953593 -0.994842 2.818630
H 7.479899 0.627392 2.358695
SCF Energy (B3LYP/6-31G**)= -1361.70866000
Number of imaginary frequencies = 0

1b_c119
MMFF Geometry
C 1.551729 2.405820 0.096728
C 2.269685 -0.116593 -0.611248
N 2.873632 2.132795 -0.025809
C 0.520892 1.484887 -0.117758
C 0.905116 0.184180 -0.487067
C 3.219591 0.873089 -0.367075
C -0.085388 -0.784626 -0.725378
C -1.449876 -0.480743 -0.600871
C -1.831541 0.811699 -0.225275
C -0.850261 1.790999 0.011229
C -3.275710 1.139022 -0.100484
C -4.331339 0.043761 -0.390741
C -3.748973 -1.299648 -0.780782
C -2.432878 -1.531638 -0.853142
O -1.178398 3.079452 0.365677
O -3.589479 2.301680 0.174459
O -5.100653 0.528843 -1.512116
C -5.302438 -0.044680 0.794204
C -4.665167 -0.588019 2.054646
C -5.603650 -1.135529 3.097440
O -3.451429 -0.546217 2.252612
O -4.611680 -2.381578 -0.911966
C -5.450347 -2.295280 -2.062807
C 4.674686 0.616328 -0.489708
C 5.443630 1.719897 -1.165026
C 5.215310 -0.516975 0.007042
C 6.676429 -0.916737 0.011699
C 7.126355 -1.185341 1.460961
C 8.616819 -1.468872 1.590267
C 6.862759 -2.138214 -0.891096
H 1.331712 3.431815 0.384187
H 2.587555 -1.111008 -0.908852
H 0.216530 -1.792783 -1.007988
H -2.072877 -2.533319 -1.071450
H -2.158429 3.144338 0.434018
H -4.471843 0.746849 -2.221934
H -6.154922 -0.683521 0.531566
H -5.722938 0.940610 1.031040
H -5.030749 -1.498442 3.955271
H -6.280802 -0.345462 3.430935
H -6.172310 -1.968900 2.677515
H -6.313979 -1.655429 -1.861210
H -4.899793 -1.941650 -2.941420
H -5.824525 -3.300427 -2.278989
H 5.614129 2.550322 -0.471796
H 6.413710 1.390017 -1.544979
H 4.894517 2.101345 -2.033969
H 4.562331 -1.236272 0.502526
H 7.299854 -0.109023 -0.383870
H 6.891192 -0.310168 2.080709
H 6.566705 -2.028992 1.884744
H 9.210478 -0.661313 1.150557
H 8.891556 -1.553991 2.646658
H 8.891164 -2.408220 1.101696
H 7.921720 -2.392176 -1.000383
H 6.471459 -1.945411 -1.896667
H 6.343646 -3.016526 -0.491115
SCF Energy (B3LYP/6-31G**)= -1361.70044177
Number of imaginary frequencies = 0

1b_c120
MMFF Geometry
C -1.387640 -2.372650 -0.487065
C -2.360933 0.076209 0.179178
N -2.719107 -2.257414 -0.262554
C -0.469428 -1.321430 -0.402212
C -0.984517 -0.058105 -0.054762
C -3.193228 -1.035126 0.060094
C -0.112118 1.041790 0.044731
C 1.263119 0.899979 -0.184345
C 1.767510 -0.360881 -0.515177
C 0.911765 -1.463380 -0.642705
C 3.216914 -0.484381 -0.761259
C 4.157375 0.591472 -0.184630
C 3.479467 1.955751 -0.136771
C 2.137963 2.076230 -0.127969
O 1.377468 -2.704038 -1.004791
O 3.647466 -1.439123 -1.414543
O 5.289482 0.669927 -1.070145
C 4.670382 0.234177 1.212455
C 4.989851 -1.234432 1.353930
C 6.358417 -1.701064 0.936221
O 4.134296 -2.022095 1.760129
O 4.371139 3.010235 -0.054164
C 3.865120 4.338533 -0.088492
C -4.653349 -0.955394 0.302358
C -5.216796 -2.109754 1.087103
C -5.376245 0.078096 -0.180040
C -6.871546 0.280668 -0.060885
C -7.222096 1.328231 1.013526
C -6.705305 2.737755 0.751553
C -7.464770 0.632056 -1.428921
H -1.062514 -3.377925 -0.747055
H -2.778468 1.036600 0.465242
H -0.517874 2.019682 0.301666
H 1.647057 3.041265 -0.067346
H 2.335210 -2.637187 -1.215322
H 5.246529 -0.110623 -1.658080

H 3.921050 0.463821 1.981792
H 5.570846 0.805626 1.469182
H 6.503592 -2.736903 1.255731
H 6.455775 -1.647317 -0.149805
H 7.121937 -1.083083 1.415118
H 3.310516 4.523380 -1.014136
H 3.243217 4.541585 0.789125
H 4.717176 5.024223 -0.062894
H -4.552875 -2.377095 1.917595
H -6.185437 -1.879606 1.538888
H -5.339879 -2.988441 0.445307
H -4.866247 0.850913 -0.754945
H -7.350587 -0.657757 0.240620
H -8.313285 1.374724 1.119916
H -6.833771 0.991047 1.983266
H -7.123545 3.154543 -0.169096
H -6.997460 3.398511 1.574515
H -5.613924 2.761474 0.680790
H -8.544522 0.798675 -1.351786
H -7.304881 -0.186206 -2.140428
H -7.012156 1.532630 -1.857356
SCF Energy (B3LYP/6-31G**)= -1361.70345205
Number of imaginary frequencies = 0

1b_c121
MMFF Geometry
C -1.746493 -1.296402 -0.673093
C -2.060805 1.285494 0.108959
N -3.004834 -0.841558 -0.458492
C -0.587100 -0.532378 -0.522768
C -0.761227 0.803242 -0.116983
C -3.158803 0.444564 -0.068274
C 0.367738 1.628106 0.046003
C 1.662447 1.138809 -0.174424
C 1.826899 -0.194282 -0.561900
C 0.713328 -1.023354 -0.755101
C 3.200927 -0.689754 -0.790265
C 4.363658 0.068886 -0.107907
C 4.073342 1.567276 -0.046911
C 2.813207 2.041300 -0.062312
O 0.843932 -2.324293 -1.176972
O 3.355669 -1.693511 -1.487944
O 5.559207 -0.140688 -0.871144
C 4.628802 -0.459837 1.303526
C 4.548907 -1.966954 1.376420
C 5.728889 -2.764390 0.888791
O 3.527018 -2.514500 1.794445
O 5.213937 2.342927 0.047377
C 5.075697 3.755847 0.142760
C -4.559804 0.910988 0.144229
C -4.822342 2.383765 -0.019899
C -5.523249 0.008785 0.435279
C -6.987919 0.269835 0.707531
C -7.865293 -0.052479 -0.517526
C -7.857869 -1.508585 -0.967072
C -7.434179 -0.495429 1.957584
H -1.693101 -2.338178 -0.982322
H -2.203688 2.310560 0.434266
H 0.227763 2.665965 0.346158
H 2.592264 3.101169 0.001659
H 1.791195 -2.499867 -1.377503
H 5.466839 0.374232 -1.691571
H 3.891664 -0.070929 2.018541
H 5.616524 -0.155351 1.671113
H 5.636090 -3.798222 1.233416
H 5.762589 -2.754242 -0.202336
H 6.652543 -2.348570 1.298984
H 4.592220 4.162250 -0.751345
H 4.526295 4.034390 1.047711
H 6.078344 4.188194 0.210207
H -4.288472 2.782170 -0.890337
H -5.877902 2.610128 -0.193164
H -4.502183 2.933935 0.870978
H -5.239140 -1.043561 0.501166
H -7.137915 1.330622 0.938785
H -8.900935 0.235487 -0.296852
H -7.546734 0.572754 -1.361587
H -8.242125 -2.172002 -0.187030
H -8.499439 -1.628261 -1.846438
H -6.853090 -1.841008 -1.243768
H -8.498110 -0.326982 2.155742
H -6.874801 -0.157058 2.837348
H -7.273521 -1.574524 1.861647
SCF Energy (B3LYP/6-31G**)= -1361.69993547
Number of imaginary frequencies = 0

1b_c122
MMFF Geometry
C 1.910939 1.737874 -0.186033
C 2.087752 -0.968171 -0.339014
N 3.140536 1.172663 -0.123172
C 0.715144 1.029579 -0.323503
C 0.818141 -0.371198 -0.401933
C 3.226367 -0.175529 -0.199563
C -0.350352 -1.142309 -0.548046
C -1.614894 -0.540861 -0.600993
C -1.709884 0.850118 -0.507246
C -0.555294 1.636165 -0.387072
C -3.055720 1.466011 -0.541458
C -4.267020 0.575440 -0.180842
C -4.043048 -0.853396 -0.664005
C -2.807443 -1.362085 -0.824376
O -0.619422 3.007414 -0.323771
O -3.137691 2.668166 -0.800240
O -5.427974 1.125159 -0.814012
C -4.463657 0.688914 1.335373
C -5.447694 -0.317091 1.888293
C -6.916587 -0.056624 1.679788
O -5.053550 -1.324517 2.480376
O -5.219373 -1.542221 -0.896353
C -5.153268 -2.950093 -1.095418
C 4.598468 -0.758382 -0.141216
C 4.800624 -2.095121 -0.802514
C 5.588896 -0.064371 0.462315
C 7.033754 -0.473554 0.653312
C 7.929603 0.378069 -0.263851
C 9.393684 -0.039043 -0.238381
C 7.406682 -0.326305 2.132028
H 1.913623 2.823761 -0.119368
H 2.175335 -2.048686 -0.385615
H -0.266680 -2.226196 -0.622279
H -2.640010 -2.383718 -1.147712
H -1.541471 3.290039 -0.523484
H -5.370909 0.898415 -1.758723
H -4.818776 1.689498 1.612957
H -3.516434 0.541098 1.870617
H -7.497635 -0.705643 2.341257
H -7.148184 0.981615 1.930364
H -7.194147 -0.265659 0.645167
H -4.639203 -3.186605 -2.032270
H -4.670863 -3.444664 -0.246054
H -6.177555 -3.327159 -1.168951
H 4.271156 -2.142084 -1.761129
H 5.848614 -2.299907 -1.036387
H 4.434920 -2.902266 -0.159372
H 5.350028 0.914171 0.884820
H 7.171792 -1.528676 0.396305
H 7.570918 0.295927 -1.298201
H 7.857012 1.438540 0.009408
H 9.503948 -1.101689 -0.476237
H 9.959386 0.534100 -0.980116
H 9.847987 0.147878 0.738884
H 8.405469 -0.727978 2.329806
H 6.704857 -0.876224 2.769603
H 7.396008 0.723242 2.447037
SCF Energy (B3LYP/6-31G**)= -1361.70338119
Number of imaginary frequencies = 0

1b_c123
MMFF Geometry
C -1.355491 -2.249717 -0.651336
C -2.319522 0.145478 0.198108

N -2.690049 -2.139341 -0.443313
C -0.429445 -1.219149 -0.462247
C -0.940140 0.016446 -0.020727
C -3.159341 -0.943128 -0.028572
C -0.060479 1.094915 0.186546
C 1.316823 0.957749 -0.029319
C 1.819121 -0.277177 -0.454476
C 0.955222 -1.355956 -0.687569
C 3.274240 -0.398047 -0.687296
C 4.220591 0.629029 -0.034117
C 3.534410 1.974185 0.108700
C 2.200037 2.111265 0.148793
O 1.415415 -2.568450 -1.141132
O 3.707550 -1.310996 -1.396866
O 5.344721 0.774923 -0.919427
C 4.736849 0.172757 1.332437
C 5.044798 -1.304462 1.373457
C 6.423012 -1.746876 0.961388
O 4.175314 -2.114932 1.695967
O 4.354160 3.059610 0.378928
C 4.579671 3.840260 -0.795407
C -4.623392 -0.868472 0.192065
C -5.210705 -2.078412 0.867915
C -5.326942 0.207317 -0.221540
C -6.822883 0.422560 -0.118127
C -7.104243 1.550279 0.891143
C -8.586982 1.765288 1.162129
C -7.383602 0.732458 -1.509583
H -1.034607 -3.233450 -0.987703
H -2.733598 1.083215 0.555520
H -0.460693 2.052512 0.518109
H 1.753552 3.083502 0.336077
H 2.379248 -2.498250 -1.319951
H 5.353211 -0.016780 -1.495078
H 3.993273 0.354447 2.119998
H 5.642525 0.719524 1.622386
H 6.545133 -2.811483 1.180028
H 6.561738 -1.586496 -0.109527
H 7.174026 -1.190186 1.527311
H 5.028790 3.244648 -1.596194
H 3.647649 4.298209 -1.143453
H 5.276258 4.642851 -0.536002
H -4.562056 -2.423098 1.681869
H -6.183195 -1.877571 1.324615
H -5.332754 -2.898029 0.152014
H -4.799342 1.019631 -0.722096
H -7.320463 -0.488025 0.230230
H -6.615346 1.312686 1.844998
H -6.666156 2.493791 0.541257
H -9.066602 0.835794 1.484546
H -8.718847 2.507174 1.956421
H -9.108099 2.136291 0.274902
H -8.477164 0.776107 -1.494528
H -7.100842 -0.045666 -2.227930
H -7.012230 1.691272 -1.888584
SCF Energy (B3LYP/6-31G**)= -1361.69939952
Number of imaginary frequencies = 0

1b_c124
MMFF Geometry
C 1.890700 1.762326 0.198927
C 2.094829 -0.941131 0.033174
N 3.115067 1.202900 0.351541
C 0.713476 1.049321 -0.038595
C 0.830917 -0.349891 -0.125009
C 3.213946 -0.144100 0.270608
C -0.316887 -1.124940 -0.376250
C -1.576759 -0.529398 -0.523197
C -1.687798 0.859639 -0.418758
C -0.551546 1.650056 -0.196203
C -3.030469 1.468753 -0.552910
C -4.260947 0.566813 -0.302151
C -3.989904 -0.853720 -0.785765
C -2.742189 -1.353581 -0.853328
O -0.629030 3.020043 -0.121617
O -3.099196 2.673809 -0.801813
O -5.371474 1.119271 -1.017962
C -4.578255 0.657566 1.195031
C -5.595182 -0.362731 1.654284
C -7.044903 -0.109008 1.333512
O -5.241369 -1.375821 2.261983
O -5.139376 -1.545155 -1.121654
C -5.048211 -2.949200 -1.337135
C 4.575522 -0.723082 0.461047
C 4.649138 -2.131868 0.985307
C 5.661446 0.034495 0.189777
C 7.121487 -0.352858 0.289313
C 7.817777 -0.063822 -1.055137
C 9.262677 -0.541827 -1.104700
C 7.763657 0.407041 1.451753
H 1.881569 2.847409 0.277154
H 2.196865 -2.018646 -0.043881
H -0.220618 -2.207224 -0.458184
H -2.542720 -2.369500 -1.176501
H -1.534011 3.300348 -0.390639
H -5.237716 0.906906 -1.958267
H -4.962045 1.651742 1.457036
H -3.675445 0.508507 1.801894
H -7.671464 -0.772233 1.936783
H -7.303939 0.923598 1.580480
H -7.237736 -0.303693 0.277063
H -4.459076 -3.167618 -2.233300
H -4.632265 -3.455028 -0.459720
H -6.060782 -3.330448 -1.498691
H 4.463988 -2.851987 0.181556
H 5.617408 -2.369351 1.433314
H 3.910673 -2.293692 1.779031
H 5.502146 1.057847 -0.157497
H 7.223651 -1.424345 0.487647
H 7.266141 -0.562676 -1.862711
H 7.792137 1.011050 -1.275495
H 9.333516 -1.606301 -0.860160
H 9.670361 -0.398063 -2.110591
H 9.895257 0.017753 -0.409684
H 8.791711 0.073250 1.623521
H 7.210077 0.239479 2.382834
H 7.785278 1.486323 1.263164
SCF Energy (B3LYP/6-31G**)= -1361.70337279
Number of imaginary frequencies = 0

1b_c125
MMFF Geometry
C -1.418040 -2.108835 -0.903320
C -2.295028 0.433234 -0.507942
N -2.755530 -1.890512 -0.904275
C -0.447183 -1.120239 -0.714771
C -0.913176 0.193104 -0.513811
C -3.180717 -0.625889 -0.696694
C 0.013423 1.236357 -0.332504
C 1.392176 0.988560 -0.333373
C 1.847761 -0.321866 -0.515968
C 0.940135 -1.369490 -0.723711
C 3.306958 -0.560449 -0.524963
C 4.237295 0.491362 0.111688
C 3.645286 1.881344 -0.023030
C 2.331852 2.101407 -0.188574
O 1.359580 -2.658689 -0.946639
O 3.756590 -1.588986 -1.039431
O 5.475090 0.443045 -0.619254
C 4.534056 0.204574 1.585398
C 4.718183 -1.267336 1.865104
C 6.099713 -1.844433 1.712352
O 3.753677 -1.970916 2.167692
O 4.504942 2.943784 0.212287
C 4.941358 3.534734 -1.012324
C -4.650518 -0.431519 -0.707481
C -5.369521 -1.204055 -1.780626
C -5.244451 0.360919 0.210509
C -6.725864 0.632070 0.374207
C -7.158210 0.252314 1.803827
C -8.659342 0.364652 2.033124
C -7.002631 2.102676 0.054090

H -1.133626 -3.146412 -1.065747
H -2.676989 1.440267 -0.371694
H -0.350666 2.253419 -0.190639
H 1.941923 3.115095 -0.196763
H 2.341122 -2.672036 -0.994394
H 5.496780 -0.421640 -1.077609
H 3.710624 0.540050 2.230055
H 5.434009 0.732632 1.923793
H 6.109103 -2.872410 2.085551
H 6.388875 -1.846581 0.659638
H 6.811582 -1.258151 2.298620
H 5.442900 2.805718 -1.656308
H 4.101058 3.992957 -1.544718
H 5.658787 4.323346 -0.767245
H -5.477156 -2.254912 -1.491912
H -6.364867 -0.806888 -1.994610
H -4.818445 -1.159734 -2.727432
H -4.623490 0.851237 0.960906
H -7.310145 0.021650 -0.321162
H -6.859025 -0.783910 2.008870
H -6.639053 0.879846 2.539509
H -9.214887 -0.225616 1.297679
H -8.914958 -0.009498 3.029799
H -8.998427 1.402850 1.973900
H -8.076685 2.313020 0.055364
H -6.623323 2.365213 -0.940224
H -6.526435 2.768990 0.782209
SCF Energy (B3LYP/6-31G**)= -1361.69941436
Number of imaginary frequencies = 0

1b_c126
MMFF Geometry
C 2.007668 1.659161 -0.157094
C 2.210055 -1.048947 -0.129085
N 3.242870 1.111152 -0.061801
C 0.817936 0.932433 -0.241947
C 0.934679 -0.469198 -0.226729
C 3.341559 -0.238386 -0.047792
C -0.226476 -1.259107 -0.314708
C -1.496587 -0.674447 -0.404957
C -1.607188 0.719206 -0.405766
C -0.459115 1.521682 -0.340641
C -2.957741 1.321969 -0.484445
C -4.190601 0.450948 -0.168099
C -3.914202 -1.003483 -0.484664
C -2.678696 -1.521604 -0.547688
O -0.532781 2.893822 -0.366651
O -3.069040 2.521977 -0.756115
O -5.239864 0.941646 -1.017189
C -4.559548 0.722073 1.293881
C -5.616474 -0.201791 1.857632
C -7.027479 -0.060901 1.348074
O -5.332809 -1.015364 2.739252
O -5.019771 -1.831884 -0.597118
C -5.334260 -2.099221 -1.964108
C 4.719927 -0.802761 0.042561
C 4.930000 -2.180148 -0.526361
C 5.705061 -0.058681 0.592904
C 7.156394 -0.430087 0.801600
C 8.093493 0.454506 -0.041348
C 7.915587 0.259666 -1.541932
C 7.501339 -0.290094 2.288242
H 2.000675 2.746998 -0.163235
H 2.307074 -2.129404 -0.103814
H -0.134147 -2.344977 -0.313492
H -2.538922 -2.586786 -0.708719
H -1.458777 3.159357 -0.564086
H -5.098587 1.906055 -1.105364
H -4.926088 1.748720 1.421951
H -3.674073 0.628751 1.937012
H -7.701358 -0.648162 1.978624
H -7.333908 0.986881 1.400459
H -7.104274 -0.424359 0.322461
H -5.506117 -1.175458 -2.525532
H -4.541741 -2.687493 -2.438538
H -6.255447 -2.688702 -1.987146
H 4.405657 -2.292390 -1.482374
H 5.978973 -2.399493 -0.739723
H 4.562700 -2.943414 0.167503
H 5.454543 0.941195 0.954302
H 7.339144 -1.475572 0.534891
H 7.941220 1.513633 0.202352
H 9.134920 0.220647 0.212999
H 6.922658 0.576715 -1.874651
H 8.654437 0.855527 -2.087423
H 8.055656 -0.789688 -1.820185
H 8.537531 -0.592540 2.474962
H 6.854014 -0.925955 2.902684
H 7.386278 0.743538 2.634076
SCF Energy (B3LYP/6-31G**)= -1361.70382048
Number of imaginary frequencies = 0

1b_c127
MMFF Geometry
C 1.976809 1.771998 0.018177
C 2.234066 -0.915681 0.311848
N 3.213939 1.269267 0.246033
C 0.811441 1.008011 -0.077898
C 0.956865 -0.382854 0.073442
C 3.339360 -0.069894 0.394042
C -0.177402 -1.209780 -0.024239
C -1.450186 -0.671162 -0.254455
C -1.590705 0.713353 -0.388265
C -0.467832 1.550234 -0.317206
C -2.945060 1.268675 -0.613787
C -4.177921 0.394887 -0.305123
C -3.848252 -1.073438 -0.470603
C -2.600416 -1.560353 -0.401911
O -0.569073 2.911951 -0.473216
O -3.062946 2.436258 -0.999845
O -5.175645 0.779465 -1.263916
C -4.654276 0.783112 1.098231
C -5.726025 -0.118235 1.670341
C -7.101301 -0.063512 1.057109
O -5.485231 -0.842957 2.638034
O -4.924799 -1.939049 -0.584381
C -5.135390 -2.333921 -1.940400
C 4.714525 -0.583123 0.659989
C 4.823250 -1.883840 1.409035
C 5.781336 0.137843 0.249309
C 7.248798 -0.200733 0.388438
C 7.971074 -0.144755 -0.972090
C 7.467479 -1.189235 -1.960844
C 7.900595 0.776490 1.369714
H 1.946980 2.854173 -0.089005
H 2.356449 -1.988663 0.417856
H -0.061779 -2.288311 0.080832
H -2.426365 -2.631208 -0.457199
H -1.484372 3.132874 -0.756728
H -5.049025 1.735750 -1.429161
H -5.052707 1.805806 1.108238
H -3.813983 0.773089 1.805481
H -7.803040 -0.614517 1.689988
H -7.435479 0.975340 0.997118
H -7.098200 -0.515464 0.064331
H -5.286683 -1.468102 -2.592736
H -4.298646 -2.939341 -2.304486
H -6.039708 -2.948476 -1.974083
H 4.642388 -2.730341 0.738483
H 5.801696 -2.027025 1.874438
H 4.098440 -1.924816 2.230288
H 5.597574 1.084031 -0.264412
H 7.384292 -1.210914 0.787209
H 7.868105 0.851740 -1.420043
H 9.044721 -0.309400 -0.816674
H 6.421919 -1.015511 -2.232706
H 8.060702 -1.151975 -2.880176
H 7.553535 -2.197509 -1.543412
H 8.959476 0.534478 1.511533
H 7.415186 0.729259 2.351026
H 7.837506 1.810275 1.011222
SCF Energy (B3LYP/6-31G**)= -1361.70382657

Number of imaginary frequencies = 0

1b_c128
MMFF Geometry
C 1.788354 2.471013 0.349689
C 2.368875 0.035271 -0.707211
N 3.092984 2.135486 0.204491
C 0.709876 1.654394 -0.003998
C 1.022381 0.396610 -0.553559
C 3.371160 0.919377 -0.311847
C -0.018581 -0.465701 -0.944949
C -1.361588 -0.102434 -0.779016
C -1.664723 1.140357 -0.216322
C -0.640782 2.023979 0.153448
C -3.089219 1.496623 -0.027210
C -4.121361 0.346424 0.019717
C -3.710755 -0.779262 -0.923355
C -2.424412 -0.989589 -1.258417
O -0.912985 3.264671 0.677989
O -3.373549 2.684220 0.139081
O -5.392688 0.872535 -0.377598
C -4.232928 -0.088277 1.485672
C -5.009816 -1.371947 1.671992
C -6.511822 -1.308756 1.577374
O -4.425555 -2.437309 1.882858
O -4.778740 -1.537907 -1.366155
C -4.508315 -2.764652 -2.035557
C 4.810842 0.591365 -0.457471
C 5.656677 1.734658 -0.951678
C 5.267332 -0.633262 -0.116834
C 6.690169 -1.144468 -0.186866
C 7.359078 -1.171090 1.201592
C 6.721630 -2.104444 2.223722
C 6.719048 -2.514605 -0.871399
H 1.624988 3.458258 0.776860
H 2.634207 -0.922582 -1.144224
H 0.227830 -1.431781 -1.384619
H -2.121987 -1.800546 -1.912055
H -1.879064 3.437438 0.595428
H -5.363920 0.986441 -1.343705
H -4.720333 0.686849 2.090608
H -3.241057 -0.244399 1.929953
H -6.940009 -2.229788 1.983258
H -6.885461 -0.468964 2.168568
H -6.820641 -1.203550 0.535870
H -4.026548 -2.582648 -3.001307
H -3.900238 -3.427151 -1.411001
H -5.464320 -3.261716 -2.224587
H 5.704351 2.533629 -0.203650
H 6.685566 1.447889 -1.177507
H 5.236764 2.149070 -1.875386
H 4.559115 -1.357403 0.286829
H 7.296478 -0.483588 -0.814540
H 8.411086 -1.459326 1.081200
H 7.365530 -0.154648 1.615966
H 6.755974 -3.146425 1.893090
H 7.265508 -2.042052 3.172175
H 5.680158 -1.835651 2.422616
H 7.740840 -2.907058 -0.910153
H 6.352968 -2.438815 -1.901754
H 6.092737 -3.249055 -0.353880
SCF Energy (B3LYP/6-31G**)= -1361.70196115
Number of imaginary frequencies = 0

1b_c129
MMFF Geometry
C -1.663492 -1.964482 -0.239137
C -2.072026 0.712107 -0.433183
N -2.940880 -1.511730 -0.232254
C -0.529655 -1.154289 -0.340400
C -0.753235 0.229033 -0.439957
C -3.140710 -0.177199 -0.328547
C 0.348316 1.097691 -0.545264
C 1.666022 0.613138 -0.550262
C 1.883004 -0.763803 -0.445158
C 0.792548 -1.644919 -0.347696
C 3.272275 -1.275896 -0.454954
C 4.447431 -0.284068 -0.456853
C 4.058793 1.171581 -0.628714
C 2.773571 1.565464 -0.672354
O 0.963694 -3.007311 -0.261066
O 3.465040 -2.496707 -0.501196
O 5.217248 -0.658334 -1.624512
C 5.344242 -0.527054 0.763869
C 4.728583 -0.033537 2.052758
C 5.679064 0.448685 3.115929
O 3.514936 -0.071968 2.252609
O 5.151675 2.019345 -0.704984
C 4.926697 3.392511 -0.999521
C -4.560576 0.283984 -0.333855
C -4.846955 1.554712 -1.084163
C -5.495390 -0.464365 0.293838
C -6.984170 -0.245241 0.472525
C -7.330450 1.109183 1.120270
C -8.778081 1.195598 1.588297
C -7.752238 -0.521374 -0.822094
H -1.571641 -3.045368 -0.157488
H -2.252619 1.780071 -0.495529
H 0.170323 2.169712 -0.622262
H 2.499795 2.608593 -0.781830
H 1.925790 -3.204450 -0.313760
H 5.303286 -1.630692 -1.597416
H 6.308991 -0.024277 0.618573
H 5.574808 -1.592076 0.888608
H 5.115104 0.776664 3.993510
H 6.346777 -0.366018 3.406358
H 6.256922 1.294190 2.734474
H 4.411755 3.509665 -1.958541
H 4.369924 3.878119 -0.191824
H 5.901227 3.883445 -1.077862
H -4.291980 1.586004 -2.028922
H -5.897970 1.658794 -1.357936
H -4.568269 2.427899 -0.485140
H -5.158029 -1.384025 0.779056
H -7.303137 -1.018225 1.187397
H -6.679917 1.273538 1.988771
H -7.147709 1.937688 0.429130
H -9.019308 0.379129 2.276136
H -8.945757 2.140964 2.114486
H -9.474614 1.157994 0.745706
H -8.824954 -0.619981 -0.626472
H -7.421725 -1.458793 -1.283796
H -7.623080 0.278583 -1.556708
SCF Energy (B3LYP/6-31G**)= -1361.70389393
Number of imaginary frequencies = 0

1b_c130
MMFF Geometry
C 1.772200 -1.558578 -0.010085
C 2.226926 1.118822 0.017038
N 3.055935 -1.128823 0.048161
C 0.653710 -0.723457 -0.058515
C 0.900761 0.661229 -0.046670
C 3.279420 0.205512 0.064478
C -0.183879 1.556184 -0.107899
C -1.505518 1.092643 -0.162007
C -1.743107 -0.283092 -0.154866
C -0.674563 -1.190312 -0.123287
C -3.144946 -0.747792 -0.213168
C -4.250003 0.220418 0.274863
C -3.890020 1.659279 -0.107773
C -2.611886 2.044144 -0.289528
O -0.878968 -2.549094 -0.157386
O -3.356718 -1.896693 -0.607562
O -4.302960 0.204485 1.704856
C -5.604755 -0.200238 -0.331314
C -6.056189 -1.562901 0.156797
C -6.857221 -2.402616 -0.800666
O -5.825040 -1.939199 1.308180
O -4.984888 2.502827 -0.196280
C -4.759713 3.898755 -0.353346
C 4.704206 0.639575 0.147772

C 4.971824 1.983789 0.769399
C 5.676486 -0.186496 -0.298323
C 7.169704 0.059782 -0.328169
C 7.853141 -0.826196 0.728690
C 9.346829 -0.564495 0.864967
C 7.695355 -0.209029 -1.741940
H 1.662265 -2.640887 -0.016274
H 2.426988 2.185186 0.015318
H 0.009905 2.628113 -0.112410
H -2.344038 3.066170 -0.531260
H -1.833469 -2.717949 -0.325072
H -4.708416 -0.651013 1.966765
H -5.539607 -0.219465 -1.425829
H -6.408427 0.488780 -0.045156
H -7.106602 -3.358711 -0.332319
H -7.781536 -1.880529 -1.059327
H -6.267740 -2.596295 -1.700216
H -4.167596 4.297107 0.476905
H -4.281976 4.110761 -1.315097
H -5.732596 4.399041 -0.344105
H 4.339269 2.141021 1.650671
H 6.000309 2.090980 1.123950
H 4.777748 2.786260 0.050222
H 5.382780 -1.157964 -0.702175
H 7.391020 1.107949 -0.102773
H 7.386575 -0.648813 1.706511
H 7.697700 -1.887330 0.495509
H 9.543849 0.492898 1.067580
H 9.755440 -1.149843 1.695080
H 9.889618 -0.852550 -0.039899
H 8.748356 0.075447 -1.833008
H 7.137657 0.373280 -2.484478
H 7.607012 -1.268066 -2.009391
SCF Energy (B3LYP/6-31G**)= -1361.71309350
Number of imaginary frequencies = 0

1b_c131
MMFF Geometry
C -1.570785 -2.468434 -0.401863
C -2.373764 0.055425 0.202122
N -2.892764 -2.256103 -0.191501
C -0.581033 -1.482217 -0.334837
C -1.009024 -0.180670 -0.020688
C -3.281606 -0.996770 0.101468
C -0.060912 0.853843 0.064905
C 1.303577 0.615113 -0.160629
C 1.729618 -0.681292 -0.468827
C 0.790699 -1.724471 -0.558536
C 3.172585 -0.935868 -0.716572
C 4.177526 0.239291 -0.631151
C 3.551488 1.574629 -0.283311
C 2.242533 1.728953 -0.050963
O 1.161025 -3.012075 -0.872138
O 3.516631 -2.075874 -1.044947
O 4.755038 0.357354 -1.949155
C 5.325200 -0.148547 0.310864
C 4.900584 -0.267640 1.758459
C 5.996694 -0.180972 2.787512
O 3.733786 -0.474270 2.090112
O 4.391135 2.654302 -0.035616
C 5.035882 3.145699 -1.209532
C -4.733830 -0.809497 0.332016
C -5.381382 -1.904762 1.136251
C -5.379350 0.262438 -0.175588
C -6.857012 0.572805 -0.069989
C -7.137781 1.663518 0.981748
C -6.520910 3.027443 0.696020
C -7.416850 0.938361 -1.448344
H -1.315095 -3.499773 -0.635674
H -2.722758 1.048858 0.466346
H -0.395752 1.860183 0.315034
H 1.860573 2.696958 0.261610
H 2.138669 -3.036709 -0.985886
H 4.019729 0.438094 -2.581273
H 6.130398 0.593743 0.243867
H 5.769889 -1.106505 0.013761
H 5.570211 -0.277483 3.789807
H 6.713709 -0.989732 2.627029
H 6.495953 0.788068 2.710576
H 5.915384 2.540431 -1.446257
H 4.350079 3.187270 -2.062803
H 5.379101 4.163311 -1.000415
H -4.742619 -2.200491 1.976790
H -6.334049 -1.597914 1.576260
H -5.562232 -2.785938 0.511776
H -4.812681 0.985834 -0.761647
H -7.402975 -0.323208 0.246428
H -8.223445 1.789315 1.080154
H -6.779317 1.318811 1.960301
H -6.903799 3.454620 -0.235187
H -6.769629 3.723314 1.504181
H -5.430250 2.972360 0.631905
H -8.482425 1.182584 -1.381405
H -7.311854 0.096979 -2.142902
H -6.899274 1.795980 -1.891126
SCF Energy (B3LYP/6-31G**)= -1361.69934432
Number of imaginary frequencies = 0

1b_c132
MMFF Geometry
C -1.457939 -2.442704 -0.428352
C -2.250653 0.086901 0.165115
N -2.779089 -2.224003 -0.219280
C -0.464094 -1.460420 -0.364810
C -0.886738 -0.155693 -0.056223
C -3.162786 -0.961962 0.068787
C 0.065493 0.875593 0.024375
C 1.429291 0.629996 -0.197917
C 1.850017 -0.670687 -0.498608
C 0.906530 -1.709647 -0.587169
C 3.292944 -0.934499 -0.739495
C 4.311527 0.218938 -0.580395
C 3.685482 1.566450 -0.298704
C 2.371748 1.743766 -0.104968
O 1.271242 -2.999567 -0.897764
O 3.626999 -2.064711 -1.108622
O 5.008171 0.330602 -1.840439
C 5.375724 -0.176994 0.450946
C 4.850259 -0.242473 1.867478
C 5.866962 -0.093779 2.968390
O 3.666099 -0.459595 2.122263
O 4.571897 2.618480 -0.117451
C 4.582220 3.501775 -1.238928
C -4.614476 -0.768084 0.298526
C -5.264271 -1.857314 1.109065
C -5.255940 0.301060 -0.219954
C -6.732723 0.623746 -0.121118
C -6.921669 1.868213 0.764958
C -8.382064 2.207464 1.030189
C -7.299852 0.820264 -1.530502
H -1.206470 -3.476056 -0.657898
H -2.595871 1.082746 0.425016
H -0.266036 1.884475 0.268167
H 1.986320 2.723578 0.161486
H 2.246414 -3.025154 -1.030455
H 4.335248 0.390840 -2.539617
H 6.205687 0.540763 0.420191
H 5.810743 -1.155245 0.211227
H 5.369773 -0.155253 3.940413
H 6.605042 -0.896190 2.895269
H 6.356826 0.879612 2.886183
H 4.835685 2.970871 -2.161992
H 3.618624 4.010486 -1.346778
H 5.349088 4.261269 -1.060895
H -4.622902 -2.154504 1.947154
H -6.212649 -1.545197 1.553820
H -5.452679 -2.739227 0.487850
H -4.687355 1.018847 -0.811748
H -7.280465 -0.210150 0.329120
H -6.430283 1.701663 1.732483
H -6.431237 2.738508 0.310413
H -8.913594 1.350856 1.456389

H -8.450704 3.036451 1.742051
H -8.895770 2.515615 0.115023
H -8.387905 0.936780 -1.506234
H -7.081487 -0.046548 -2.164862
H -6.875656 1.707051 -2.014807
SCF Energy (B3LYP/6-31G**)= -1361.70014608
Number of imaginary frequencies = 0

1b_c133
MMFF Geometry
C 1.680536 1.933481 -0.199106
C 2.101820 -0.738538 -0.427421
N 2.960516 1.488331 -0.216427
C 0.549946 1.117870 -0.292262
C 0.780388 -0.263281 -0.409974
C 3.166743 0.155984 -0.329351
C -0.316834 -1.137738 -0.508567
C -1.635826 -0.659741 -0.490527
C -1.861642 0.715250 -0.365940
C -0.775130 1.601322 -0.274480
C -3.256206 1.220360 -0.352999
C -4.431431 0.224256 -0.360762
C -4.017679 -1.215538 -0.571043
C -2.739094 -1.612505 -0.601031
O -0.952546 2.961642 -0.170680
O -3.457301 2.440421 -0.385692
O -5.219454 0.636611 -1.500857
C -5.302624 0.418811 0.885027
C -4.643860 -0.114655 2.135842
C -5.518233 -0.870116 3.099889
O -3.455669 0.096002 2.377776
O -5.010642 -2.187858 -0.554110
C -5.772161 -2.203138 -1.761802
C 4.589160 -0.296560 -0.360348
C 4.871266 -1.560202 -1.124183
C 5.529354 0.452668 0.258235
C 7.021975 0.241014 0.412127
C 7.385966 -1.115501 1.045622
C 8.841333 -1.196803 1.489969
C 7.768186 0.529933 -0.892451
H 1.583787 3.012839 -0.104148
H 2.287318 -1.804822 -0.503364
H -0.135954 -2.208337 -0.598836
H -2.502795 -2.669480 -0.683826
H -1.916592 3.153582 -0.196827
H -5.278939 1.611734 -1.451843
H -6.267470 -0.085127 0.745440
H -5.539577 1.476228 1.054044
H -4.922952 -1.202324 3.954871
H -6.318680 -0.217275 3.456272
H -5.938539 -1.747792 2.602572
H -6.571000 -1.457580 -1.717376
H -5.144224 -2.049222 -2.646413
H -6.241614 -3.187826 -1.846655
H 4.302957 -1.587363 -2.061109
H 5.918740 -1.656851 -1.413748
H 4.605746 -2.439258 -0.527764
H 5.194289 1.366575 0.755730
H 7.347720 1.011229 1.126936
H 6.750285 -1.288922 1.923310
H 7.196716 -1.940660 0.352234
H 9.089063 -0.383313 2.179025
H 9.022537 -2.144488 2.007429
H 9.524104 -1.150083 0.636638
H 8.843200 0.633817 -0.712814
H 7.424968 1.468220 -1.342984
H 7.632469 -0.266147 -1.630079
SCF Energy (B3LYP/6-31G**)= -1361.69821035
Number of imaginary frequencies = 0

1b_c134
MMFF Geometry
C -1.658976 -1.388281 -0.733686
C -2.054793 1.171613 0.082786
N -2.932170 -0.971720 -0.529554
C -0.523117 -0.595323 -0.555482
C -0.739499 0.728586 -0.131842
C -3.126613 0.303774 -0.122704
C 0.364029 1.581086 0.060924
C 1.674795 1.131437 -0.148873
C 1.879750 -0.189845 -0.555598
C 0.793409 -1.046814 -0.776889
C 3.267136 -0.638525 -0.780061
C 4.413219 0.145355 -0.111913
C 4.074221 1.625776 0.016940
C 2.799399 2.060468 0.006852
O 0.965924 -2.337290 -1.215093
O 3.483911 -1.625700 -1.488703
O 5.562558 0.008464 -0.967645
C 4.777999 -0.410155 1.266857
C 4.737709 -1.918513 1.312698
C 5.972027 -2.669329 0.890885
O 3.706962 -2.504608 1.646475
O 5.185528 2.431170 0.190685
C 5.008242 3.841103 0.237958
C -4.542564 0.728817 0.077135
C -4.843310 2.194910 -0.080428
C -5.481637 -0.202881 0.354305
C -6.958055 0.007577 0.613426
C -7.766198 -0.527586 -0.582193
C -9.259942 -0.252614 -0.475979
C -7.340377 -0.678419 1.928932
H -1.572893 -2.423127 -1.058279
H -2.230386 2.187272 0.421499
H 0.191702 2.609499 0.376485
H 2.547802 3.108709 0.124383
H 1.919467 -2.487516 -1.400068
H 5.358761 -0.702694 -1.607572
H 4.077012 -0.057944 2.035415
H 5.777985 -0.085768 1.580068
H 5.857852 -3.727971 1.140619
H 6.118000 -2.572259 -0.186652
H 6.841943 -2.281355 1.426541
H 4.546685 4.209698 -0.683819
H 4.420299 4.130897 1.114625
H 5.996340 4.301889 0.328312
H -4.302527 2.616169 -0.935752
H -5.900421 2.393563 -0.275388
H -4.557531 2.745872 0.821579
H -5.169122 -1.247786 0.415785
H -7.178733 1.072713 0.736740
H -7.399540 -0.060174 -1.505433
H -7.610521 -1.608119 -0.695121
H -9.453890 0.814747 -0.330811
H -9.764822 -0.566178 -1.395466
H -9.712341 -0.804500 0.352958
H -8.371884 -0.445303 2.211209
H -6.696357 -0.339940 2.748775
H -7.247868 -1.767936 1.856204
SCF Energy (B3LYP/6-31G**)= -1361.70533233
Number of imaginary frequencies = 0

1b_c135
MMFF Geometry
C 1.753137 -1.583576 -0.442297
C 2.240233 1.085004 -0.311220
N 3.042593 -1.167545 -0.458665
C 0.644114 -0.738341 -0.362779
C 0.907740 0.641681 -0.295713
C 3.282568 0.162416 -0.392403
C -0.167693 1.546742 -0.221589
C -1.494988 1.096943 -0.198047
C -1.747634 -0.275174 -0.248822
C -0.690638 -1.190802 -0.349949
C -3.155169 -0.725393 -0.223800
C -4.208003 0.222066 0.401171
C -3.861297 1.677550 0.073044
C -2.596498 2.062430 -0.185651
O -0.913103 -2.544070 -0.441651
O -3.410123 -1.848789 -0.663529
O -4.151713 0.125525 1.827844
C -5.610050 -0.153240 -0.121505

C -6.039230 -1.537830 0.323051
C -6.921059 -2.315829 -0.615357
O -5.725389 -1.980461 1.430429
O -4.949505 2.533118 0.117521
C -4.720144 3.934067 0.024134
C 4.714113 0.581666 -0.426477
C 5.008283 1.958486 -0.958013
C 5.667650 -0.283351 -0.014951
C 7.163578 -0.063195 0.057359
C 7.657988 -0.388690 1.480573
C 9.131626 -0.072508 1.697607
C 7.848812 -0.922828 -1.006878
H 1.630309 -2.663136 -0.496854
H 2.452628 2.146962 -0.245851
H 0.037882 2.615732 -0.181507
H -2.335950 3.094523 -0.389626
H -1.879547 -2.696164 -0.544390
H -4.546116 -0.740327 2.071984
H -5.628921 -0.111009 -1.217150
H -6.381211 0.524626 0.263629
H -7.145431 -3.294943 -0.183326
H -7.856081 -1.772837 -0.772620
H -6.404346 -2.462833 -1.566979
H -4.061075 4.278754 0.827518
H -4.315321 4.198167 -0.957961
H -5.683281 4.440397 0.137582
H 4.809020 2.716971 -0.193896
H 6.044159 2.076026 -1.286273
H 4.394369 2.177647 -1.839257
H 5.353310 -1.272314 0.326315
H 7.409594 0.984151 -0.143536
H 7.074569 0.191319 2.207585
H 7.484561 -1.447291 1.712631
H 9.351333 0.970058 1.447193
H 9.398027 -0.231475 2.747584
H 9.774637 -0.718560 1.093051
H 8.921183 -0.710129 -1.057832
H 7.432327 -0.721891 -2.000662
H 7.725253 -1.991961 -0.800551
SCF Energy (B3LYP/6-31G**)= -1361.71311414
Number of imaginary frequencies = 0

1b_c136
MMFF Geometry
C -1.414822 -2.308620 -0.627789
C -2.395601 0.083428 0.211224
N -2.749242 -2.208680 -0.413649
C -0.496964 -1.268722 -0.450080
C -1.016297 -0.034853 -0.013858
C -3.226930 -1.013977 -0.004295
C -0.145209 1.052656 0.182016
C 1.232132 0.926256 -0.040039
C 1.743179 -0.306906 -0.459870
C 0.887696 -1.394750 -0.681624
C 3.198110 -0.416429 -0.699380
C 4.138633 0.623142 -0.057649
C 3.441302 1.963084 0.080088
C 2.105989 2.088642 0.126156
O 1.356303 -2.606033 -1.129776
O 3.635880 -1.330069 -1.405314
O 5.256918 0.773245 -0.949632
C 4.665844 0.180250 1.309126
C 4.987064 -1.293900 1.358008
C 6.367030 -1.726783 0.941702
O 4.126441 -2.109915 1.690156
O 4.252770 3.057416 0.339156
C 4.465390 3.832449 -0.841290
C -4.690208 -0.950659 0.223511
C -5.265244 -2.161406 0.908523
C -5.405631 0.118588 -0.186702
C -6.902197 0.314249 -0.072165
C -7.266275 1.275423 1.075975
C -6.744644 2.700060 0.931854
C -7.476511 0.772565 -1.416420
H -1.086948 -3.291642 -0.959461
H -2.816034 1.019776 0.564910
H -0.552212 2.008796 0.509499
H 1.651867 3.058087 0.309511
H 2.318555 -2.528481 -1.313990
H 5.269493 -0.022039 -1.520251
H 3.924697 0.360440 2.099317
H 5.568104 0.736841 1.590919
H 6.499683 -2.788841 1.166516
H 6.498889 -1.572085 -0.130915
H 7.115952 -1.159857 1.500188
H 4.915688 3.235679 -1.640550
H 3.527593 4.279909 -1.187461
H 5.156152 4.642807 -0.590603
H -4.613040 -2.493548 1.724789
H -6.239870 -1.965304 1.363483
H -5.380084 -2.987435 0.198854
H -4.887155 0.932992 -0.692481
H -7.386164 -0.643896 0.148661
H -8.358761 1.315655 1.171003
H -6.891446 0.862773 2.021548
H -7.149968 3.188296 0.041028
H -7.047304 3.295128 1.799905
H -5.652381 2.727045 0.877838
H -8.557049 0.934816 -1.341009
H -7.307779 0.012037 -2.187448
H -7.017284 1.702934 -1.767097
SCF Energy (B3LYP/6-31G**)= -1361.69837430
Number of imaginary frequencies = 0

1b_c137
MMFF Geometry
C 2.009589 1.611700 -0.155928
C 2.109716 -1.097133 -0.327593
N 3.222268 1.011100 -0.093101
C 0.794580 0.938864 -0.302319
C 0.857862 -0.463680 -0.390410
C 3.270057 -0.338465 -0.178695
C -0.331657 -1.200034 -0.545905
C -1.578331 -0.562356 -0.598704
C -1.633916 0.830069 -0.495304
C -0.457790 1.581940 -0.365717
C -2.961499 1.484377 -0.529523
C -4.198968 0.626236 -0.179277
C -4.014179 -0.804957 -0.671944
C -2.793042 -1.347583 -0.831896
O -0.482966 2.953981 -0.292827
O -3.008228 2.690181 -0.779956
O -5.341554 1.213357 -0.812284
C -4.397562 0.734544 1.337062
C -5.411804 -0.246855 1.879524
C -6.871937 0.056908 1.668099
O -5.048609 -1.269295 2.465636
O -5.208865 -1.458239 -0.913098
C -5.182270 -2.865977 -1.122066
C 4.624938 -0.960275 -0.120217
C 4.789996 -2.303158 -0.779640
C 5.636809 -0.293888 0.479007
C 7.066806 -0.750082 0.665633
C 8.014772 -0.099797 -0.360486
C 8.129256 1.417740 -0.280697
C 7.514237 -0.498018 2.109145
H 2.043091 2.696568 -0.081540
H 2.166294 -2.179331 -0.381943
H -0.278705 -2.285317 -0.627633
H -2.653704 -2.371266 -1.162004
H -1.395871 3.264235 -0.493530
H -5.287683 0.991788 -1.758410
H -4.724967 1.742866 1.620652
H -3.456808 0.555969 1.874332
H -7.473553 -0.579947 2.322971
H -7.074700 1.099527 1.925391
H -7.151750 -0.136760 0.631096
H -4.671895 -3.110421 -2.058900
H -4.717146 -3.380139 -0.274700
H -6.216638 -3.213101 -1.201678
H 4.268348 -2.333063 -1.743171
H 5.833503 -2.541485 -1.002629

H 4.392860 -3.098050 -0.139884
H 5.425718 0.690932 0.900707
H 7.131403 -1.834355 0.519332
H 9.017356 -0.528646 -0.237658
H 7.687854 -0.370230 -1.372821
H 8.527059 1.740528 0.685637
H 8.813478 1.779058 -1.055622
H 7.162882 1.904881 -0.440273
H 8.554385 -0.809732 2.252384
H 6.896288 -1.070728 2.810233
H 7.434825 0.557915 2.388710
SCF Energy (B3LYP/6-31G**)= -1361.70258010
Number of imaginary frequencies = 0

1b_c138
MMFF Geometry
C -1.637195 -1.559889 -0.345990
C -2.069055 1.017640 0.393094
N -2.906909 -1.179619 -0.064116
C -0.522379 -0.720586 -0.286427
C -0.757610 0.612695 0.095778
C -3.118941 0.104239 0.305923
C 0.322747 1.512678 0.163110
C 1.630419 1.101035 -0.128151
C 1.855836 -0.229839 -0.490352
C 0.790329 -1.134539 -0.589033
C 3.238804 -0.638524 -0.801741
C 4.400483 0.217015 -0.260421
C 4.014050 1.688770 -0.172337
C 2.725971 2.076331 -0.102493
O 0.979546 -2.436827 -0.983207
O 3.439965 -1.649911 -1.480203
O 5.486161 0.080613 -1.195673
C 4.890507 -0.258363 1.109450
C 4.912058 -1.763234 1.226600
C 6.138376 -2.489938 0.743664
O 3.933335 -2.368154 1.666520
O 5.102624 2.541028 -0.122345
C 4.873795 3.944315 -0.126462
C -4.527081 0.480017 0.624622
C -4.726512 1.619874 1.586715
C -5.541247 -0.222285 0.072378
C -7.030025 -0.003722 0.237413
C -7.683000 0.124941 -1.152899
C -9.162707 0.479980 -1.097266
C -7.618920 -1.157494 1.051730
H -1.535734 -2.604403 -0.633003
H -2.261756 2.047076 0.676413
H 0.134455 2.548078 0.444896
H 2.442758 3.119279 -0.013140
H 1.921610 -2.561360 -1.234279
H 5.262180 -0.666525 -1.785925
H 4.237083 0.105678 1.913585
H 5.898182 0.115531 1.328754
H 6.084477 -3.538728 1.049049
H 6.197502 -2.439605 -0.345275
H 7.031212 -2.046641 1.191477
H 4.329213 4.250491 -1.025495
H 4.343260 4.255427 0.779044
H 5.846896 4.444150 -0.134675
H -4.593708 2.580526 1.078406
H -5.716770 1.618173 2.049365
H -4.014025 1.560592 2.417597
H -5.290972 -1.053714 -0.590496
H -7.225538 0.927166 0.778789
H -7.166305 0.907282 -1.724131
H -7.563144 -0.807536 -1.719189
H -9.326995 1.391101 -0.513493
H -9.544585 0.652914 -2.108677
H -9.753522 -0.327597 -0.655574
H -8.673670 -0.980535 1.284120
H -7.092815 -1.271759 2.006506
H -7.546188 -2.108427 0.511989
SCF Energy (B3LYP/6-31G**)= -1361.70533962
Number of imaginary frequencies = 0

1b_c139
MMFF Geometry
C -1.589109 -2.290102 -0.083254
C -2.472307 0.246160 -0.499054
N -2.916368 -2.104239 -0.283838
C -0.632155 -1.270625 -0.073359
C -1.100851 0.038703 -0.290636
C -3.345923 -0.839682 -0.481959
C -0.187790 1.109485 -0.288328
C 1.182336 0.893714 -0.087312
C 1.642243 -0.409350 0.112693
C 0.744207 -1.486036 0.139465
C 3.091087 -0.611504 0.323752
C 4.060304 0.464615 -0.223342
C 3.437270 1.855521 -0.070310
C 2.102483 2.031723 -0.025213
O 1.166101 -2.773219 0.372950
O 3.453711 -1.646785 0.886882
O 4.239504 0.276033 -1.630558
C 5.407724 0.367432 0.521523
C 6.117784 -0.946340 0.259345
C 6.959529 -1.504392 1.374497
O 6.052787 -1.500746 -0.840256
O 4.367000 2.881384 -0.032761
C 3.902939 4.225017 -0.090403
C -4.804085 -0.678669 -0.704318
C -5.411732 -1.732666 -1.591257
C -5.478126 0.323236 -0.099375
C -6.955086 0.638375 -0.198244
C -7.725200 0.176519 1.054599
C -7.328680 0.854377 2.360555
C -7.152046 2.130554 -0.483327
H -1.301107 -3.327284 0.074962
H -2.854093 1.244941 -0.686972
H -0.555280 2.122784 -0.445808
H 1.650700 3.013825 0.052941
H 2.117050 -2.750186 0.623774
H 4.801361 -0.521755 -1.743231
H 5.251463 0.476541 1.601457
H 6.109126 1.144060 0.193797
H 7.402541 -2.453953 1.061876
H 7.759338 -0.800264 1.616047
H 6.333855 -1.681932 2.252677
H 3.327433 4.403100 -1.004561
H 3.315600 4.473066 0.799342
H 4.777584 4.881914 -0.109304
H -5.388363 -2.712365 -1.101551
H -6.452319 -1.532314 -1.853714
H -4.858522 -1.803371 -2.534798
H -4.929800 0.987909 0.568644
H -7.395989 0.110252 -1.049722
H -8.797440 0.343915 0.891572
H -7.599081 -0.907377 1.173622
H -7.505550 1.933065 2.323520
H -7.927279 0.450836 3.184020
H -6.276066 0.681509 2.602607
H -8.217740 2.377690 -0.536251
H -6.699788 2.402715 -1.443941
H -6.694780 2.762617 0.285384
SCF Energy (B3LYP/6-31G**)= -1361.71178160
Number of imaginary frequencies = 0

1b_c140
MMFF Geometry
C -1.479896 -2.193776 -0.772268
C -2.327444 0.384832 -0.637833
N -2.814925 -1.961501 -0.791197
C -0.497707 -1.201990 -0.689335
C -0.948212 0.130070 -0.622989
C -3.225939 -0.677998 -0.713407
C -0.008975 1.175890 -0.553324
C 1.367626 0.914411 -0.531559
C 1.805268 -0.412145 -0.580040
C 0.886478 -1.465916 -0.677350
C 3.259089 -0.662845 -0.565695
C 4.191307 0.433385 -0.014576

C 3.634336 1.825931 -0.286057
C 2.322290 2.028143 -0.513801
O 1.292449 -2.775097 -0.769377
O 3.699115 -1.736260 -0.987419
O 5.446428 0.295964 -0.705780
C 4.455163 0.279512 1.485203
C 4.621651 -1.162730 1.898809
C 5.992084 -1.774452 1.785250
O 3.649838 -1.819191 2.275645
O 4.591112 2.823056 -0.221554
C 4.212899 4.155933 -0.540959
C -4.694169 -0.465271 -0.740331
C -5.425709 -1.340628 -1.722861
C -5.268680 0.421055 0.101385
C -6.737709 0.763331 0.225132
C -7.381356 0.092628 1.454701
C -6.826030 0.522051 2.807273
C -6.920118 2.284310 0.231706
H -1.207394 -3.245671 -0.828336
H -2.698085 1.404728 -0.605280
H -0.362783 2.205689 -0.517788
H 1.913076 3.018245 -0.681745
H 2.273273 -2.802483 -0.826852
H 5.425269 -0.566349 -1.167016
H 3.621127 0.677729 2.078433
H 5.352606 0.829137 1.794730
H 5.997433 -2.750741 2.278156
H 6.256348 -1.905695 0.734174
H 6.725147 -1.134174 2.282108
H 3.822347 4.219106 -1.561768
H 3.485671 4.538696 0.182114
H 5.107440 4.782988 -0.482423
H -5.377527 -2.391494 -1.417031
H -6.482503 -1.087350 -1.827233
H -4.981353 -1.249124 -2.720596
H -4.631883 0.951207 0.810072
H -7.283316 0.400351 -0.651746
H -8.459477 0.297191 1.444523
H -7.275404 -0.996288 1.365611
H -6.973741 1.591684 2.981310
H -7.343828 -0.016250 3.608133
H -5.759019 0.298215 2.895905
H -7.977536 2.547394 0.342319
H -6.566296 2.719315 -0.710085
H -6.364126 2.764281 1.044118
SCF Energy (B3LYP/6-31G**)= -1361.70399801
Number of imaginary frequencies = 0

1b_c141
MMFF Geometry
C 2.004185 1.627982 0.348821
C 2.155556 -1.069765 0.074647
N 3.216757 1.038821 0.481787
C 0.813677 0.948732 0.080113
C 0.903977 -0.447502 -0.061908
C 3.289708 -0.305524 0.346019
C -0.258645 -1.188541 -0.344640
C -1.506839 -0.563222 -0.471406
C -1.590470 0.821853 -0.314420
C -0.439292 1.579847 -0.056135
C -2.916749 1.462939 -0.432341
C -4.181232 0.591692 -0.310704
C -3.926884 -0.835128 -0.772970
C -2.687075 -1.356996 -0.820664
O -0.487948 2.946964 0.075676
O -2.985327 2.688346 -0.572198
O -5.159241 1.210056 -1.163428
C -4.654801 0.698555 1.143232
C -5.758450 -0.270903 1.504108
C -7.145140 0.012366 0.987365
O -5.526648 -1.251333 2.214996
O -5.082985 -1.519129 -1.102973
C -4.994872 -2.896333 -1.445147
C 4.640010 -0.918295 0.516139
C 4.680252 -2.347889 0.984941
C 5.738984 -0.171460 0.267170
C 7.193866 -0.575390 0.358974
C 7.884410 -0.552722 -1.020662
C 8.000042 0.813669 -1.685380
C 7.913860 0.303771 1.384337
H 2.016586 2.708975 0.470501
H 2.235881 -2.145131 -0.045767
H -0.183628 -2.268691 -0.467657
H -2.506551 -2.380299 -1.131165
H -1.387105 3.258372 -0.173081
H -4.968466 2.169263 -1.162340
H -5.020090 1.709249 1.365835
H -3.823424 0.512745 1.836389
H -7.859357 -0.653507 1.480300
H -7.420593 1.044138 1.219731
H -7.194011 -0.156619 -0.089416
H -4.407146 -3.035058 -2.358095
H -4.579226 -3.479609 -0.617194
H -6.008276 -3.260281 -1.638388
H 4.465125 -3.030479 0.156175
H 5.644734 -2.634131 1.410305
H 3.945593 -2.519013 1.780305
H 5.589726 0.863314 -0.047428
H 7.279532 -1.605818 0.717237
H 8.895111 -0.966963 -0.913419
H 7.348135 -1.226595 -1.701287
H 8.587449 1.508185 -1.078096
H 8.505724 0.713308 -2.651605
H 7.018455 1.257803 -1.873020
H 8.977021 0.046253 1.438096
H 7.486483 0.159895 2.383342
H 7.833755 1.369284 1.143707
SCF Energy (B3LYP/6-31G**)= -1361.70832134
Number of imaginary frequencies = 0

1b_c142
MMFF Geometry
C -1.786075 -1.456163 -0.772504
C -2.059799 1.079424 0.156573
N -3.038713 -0.988903 -0.550493
C -0.613012 -0.727117 -0.561812
C -0.767266 0.583690 -0.081343
C -3.172177 0.274757 -0.086062
C 0.376005 1.368760 0.151058
C 1.666409 0.871567 -0.089252
C 1.818533 -0.435984 -0.562431
C 0.683197 -1.230574 -0.800883
C 3.182736 -0.963043 -0.825808
C 4.410734 -0.053821 -0.573697
C 4.070122 1.329949 -0.058600
C 2.815901 1.732791 0.177727
O 0.782337 -2.516614 -1.280004
O 3.288770 -2.099067 -1.299081
O 5.051948 0.103310 -1.857742
C 5.411201 -0.797559 0.321249
C 4.913736 -1.006382 1.735096
C 5.960995 -1.296050 2.777824
O 3.717684 -0.992617 2.023799
O 5.109866 2.162135 0.339494
C 5.891214 2.646690 -0.751350
C -4.566461 0.757844 0.135151
C -4.793831 2.243521 0.057952
C -5.551610 -0.139676 0.360599
C -7.016583 0.129281 0.630218
C -7.846408 -0.298720 -0.593389
C -9.325048 0.043735 -0.472915
C -7.436632 -0.608071 1.905847
H -1.749169 -2.478930 -1.141404
H -2.184832 2.085539 0.542852
H 0.254251 2.383386 0.529338
H 2.636867 2.712633 0.611839
H 1.735906 -2.738168 -1.384470
H 4.376059 0.419615 -2.482155
H 6.357827 -0.244424 0.363182
H 5.653449 -1.782891 -0.096065
H 5.484352 -1.425090 3.753507
H 6.491965 -2.214868 2.517430

H 6.660621 -0.458666 2.837222
H 6.630418 1.900325 -1.055366
H 5.264911 2.943271 -1.599824
H 6.434613 3.531090 -0.405487
H -4.233270 2.682912 -0.775228
H -5.839748 2.505067 -0.122477
H -4.480473 2.729995 0.987473
H -5.291374 -1.200542 0.365777
H -7.184740 1.195633 0.811853
H -7.454129 0.199909 -1.489364
H -7.744032 -1.377814 -0.765482
H -9.466404 1.109820 -0.269369
H -9.841887 -0.193461 -1.408489
H -9.806847 -0.529711 0.324210
H -8.456220 -0.339874 2.200030
H -6.779257 -0.347494 2.743362
H -7.397994 -1.695201 1.773370
SCF Energy (B3LYP/6-31G**)= -1361.70129045
Number of imaginary frequencies = 0

1b_c143
MMFF Geometry
C -1.583646 -2.414613 -0.075699
C -2.273481 0.208893 -0.236327
N -2.900654 -2.100876 -0.013548
C -0.545424 -1.487588 -0.218909
C -0.915187 -0.133610 -0.303775
C -3.229162 -0.793490 -0.085522
C 0.082789 0.845299 -0.457873
C 1.442147 0.500748 -0.522145
C 1.806212 -0.845834 -0.428642
C 0.819299 -1.836379 -0.285526
C 3.239732 -1.209742 -0.500294
C 4.303488 -0.100436 -0.551113
C 3.757675 1.305891 -0.705071
C 2.437638 1.563089 -0.692228
O 1.136050 -3.173234 -0.211162
O 3.557028 -2.403792 -0.557463
O 5.058221 -0.396381 -1.750679
C 5.271636 -0.243838 0.630200
C 4.663148 0.188536 1.944194
C 5.601631 0.775916 2.964244
O 3.470381 0.021686 2.196879
O 4.751511 2.262932 -0.828635
C 4.371466 3.603480 -1.114108
C -4.677918 -0.490485 -0.014034
C -5.526286 -1.339570 -0.916398
C -5.119916 0.474634 0.820749
C -6.520563 0.947700 1.158660
C -6.849694 2.291630 0.478721
C -6.926726 2.248010 -1.041892
C -7.656273 -0.069274 1.021291
H -1.373723 -3.479764 -0.004907
H -2.585511 1.245907 -0.312616
H -0.210185 1.892417 -0.525421
H 2.051655 2.571410 -0.789203
H 2.110413 -3.268280 -0.305406
H 5.246044 -1.354423 -1.728361
H 6.171858 0.355810 0.444012
H 5.616815 -1.278584 0.744829
H 5.044323 1.046757 3.865255
H 6.364778 0.039072 3.226155
H 6.068450 1.676178 2.556914
H 3.806131 3.661092 -2.049892
H 3.802341 4.032449 -0.283286
H 5.284939 4.193211 -1.235137
H -4.953501 -1.720168 -1.770474
H -5.931449 -2.198402 -0.372258
H -6.347113 -0.765523 -1.352715
H -4.371205 1.003459 1.414975
H -6.480305 1.162009 2.237737
H -7.806304 2.664089 0.866353
H -6.094134 3.035022 0.764580
H -7.709748 1.567110 -1.386727
H -7.162324 3.244586 -1.429874
H -5.975698 1.938593 -1.484965
H -8.552931 0.300845 1.531960
H -7.388596 -1.024162 1.486288
H -7.941488 -0.258059 -0.015508
SCF Energy (B3LYP/6-31G**)= -1361.70101397
Number of imaginary frequencies = 0

1b_c144
MMFF Geometry
C -1.719404 -1.495058 -0.243348
C -2.082534 1.104568 0.454497
N -2.974882 -1.082732 0.056927
C -0.586685 -0.678218 -0.223327
C -0.786304 0.666693 0.137632
C -3.152665 0.211970 0.406496
C 0.313294 1.545154 0.163634
C 1.605604 1.100211 -0.146532
C 1.797485 -0.242235 -0.486164
C 0.710523 -1.125303 -0.545098
C 3.168476 -0.690473 -0.810379
C 4.348880 0.148837 -0.266910
C 3.996351 1.634963 -0.249440
C 2.719038 2.054510 -0.176464
O 0.864038 -2.438304 -0.919206
O 3.309388 -1.720055 -1.472450
O 5.483752 -0.048178 -1.120749
C 4.756800 -0.297806 1.138827
C 4.751392 -1.801419 1.287691
C 5.920265 -2.573174 0.736178
O 3.794561 -2.368778 1.818151
O 5.103988 2.461343 -0.290711
C 4.909517 3.870268 -0.249790
C -4.546401 0.622727 0.747566
C -4.697447 1.777974 1.700058
C -5.583866 -0.060644 0.214591
C -7.065666 0.176162 0.404781
C -7.752975 0.600414 -0.909909
C -7.762726 -0.440698 -2.022670
C -7.714574 -1.062526 1.027018
H -1.645217 -2.546876 -0.511590
H -2.248007 2.142814 0.722220
H 0.151875 2.589751 0.427461
H 2.455840 3.106076 -0.142121
H 1.798126 -2.583844 -1.192435
H 5.298730 0.421390 -1.952650
H 4.066435 0.095638 1.896766
H 5.757486 0.064775 1.404513
H 5.903378 -3.591761 1.134149
H 5.860845 -2.615259 -0.353085
H 6.855827 -2.100464 1.045320
H 4.333568 4.210416 -1.116348
H 4.426844 4.170969 0.685608
H 5.893317 4.346997 -0.290294
H -4.539223 2.728439 1.179842
H -5.680207 1.817658 2.175229
H -3.976077 1.703612 2.522074
H -5.354097 -0.898109 -0.447245
H -7.230991 1.000147 1.105682
H -8.792686 0.874100 -0.689202
H -7.270835 1.510709 -1.289185
H -8.294311 -1.347553 -1.720636
H -8.275101 -0.036998 -2.902374
H -6.749781 -0.715477 -2.329933
H -8.794552 -0.919701 1.140008
H -7.299349 -1.256302 2.022709
H -7.551742 -1.961656 0.423124
SCF Energy (B3LYP/6-31G**)= -1361.69966149
Number of imaginary frequencies = 0

1b_c145
MMFF Geometry
C -1.447378 -2.271168 -0.446835
C -2.368912 0.238212 0.048168
N -2.776107 -2.112702 -0.232161
C -0.507265 -1.236295 -0.433565
C -0.995821 0.059444 -0.173931
C -3.224054 -0.861269 0.004932

C -0.102106 1.146288 -0.151793
C 1.267931 0.956527 -0.365706
C 1.748286 -0.335480 -0.607579
C 0.870182 -1.424840 -0.663597
C 3.203304 -0.504304 -0.824982
C 4.145808 0.568664 -0.219697
C 3.501086 1.942339 -0.373091
C 2.171072 2.106143 -0.432576
O 1.310804 -2.694787 -0.945519
O 3.601843 -1.496483 -1.436217
O 5.393821 0.559113 -0.918118
C 4.439122 0.254355 1.251975
C 4.654776 -1.222789 1.494188
C 5.988042 -1.814670 1.121903
O 3.745799 -1.914698 1.955125
O 4.306197 3.045522 -0.620098
C 5.177475 3.373765 0.458689
C -4.682098 -0.733642 0.240193
C -5.264958 -1.814192 1.110782
C -5.382126 0.273141 -0.325229
C -6.870236 0.530557 -0.225209
C -7.164422 1.865466 0.483861
C -6.722400 1.881085 1.941943
C -7.475038 0.540145 -1.633021
H -1.143414 -3.298548 -0.636568
H -2.766598 1.224510 0.266403
H -0.485268 2.150080 0.028024
H 1.742899 3.093142 -0.584114
H 2.266603 -2.656601 -1.173744
H 5.202087 0.824741 -1.836385
H 3.606338 0.562565 1.897629
H 5.330512 0.777149 1.616210
H 6.077839 -2.812699 1.560126
H 6.073076 -1.893859 0.036431
H 6.793460 -1.191996 1.519234
H 4.651317 3.354004 1.419164
H 6.042254 2.705094 0.477326
H 5.545645 4.390797 0.294267
H -4.608953 -2.021224 1.964503
H -6.233704 -1.541049 1.536113
H -5.393686 -2.740336 0.540740
H -4.856102 0.984697 -0.962272
H -7.372907 -0.268467 0.328484
H -6.679235 2.695051 -0.045949
H -8.243688 2.060514 0.451069
H -5.635485 1.795845 2.034507
H -7.023068 2.821621 2.414686
H -7.183108 1.059737 2.499937
H -8.559799 0.685663 -1.586946
H -7.288935 -0.410001 -2.146292
H -7.054662 1.344243 -2.247776
SCF Energy (B3LYP/6-31G**)= -1361.69390512
Number of imaginary frequencies = 0

1b_c146
MMFF Geometry
C -1.766428 -1.652591 -0.344666
C -2.072174 0.928727 0.437135
N -3.017457 -1.214904 -0.061690
C -0.610778 -0.871035 -0.268408
C -0.781858 0.463593 0.134664
C -3.166193 0.071118 0.331055
C 0.342537 1.303221 0.224770
C 1.630813 0.836166 -0.078943
C 1.800523 -0.495350 -0.472656
C 0.683696 -1.344095 -0.570584
C 3.161937 -0.991309 -0.802457
C 4.367359 -0.023869 -0.706482
C 4.009777 1.380012 -0.262196
C 2.761001 1.754130 0.040770
O 0.799133 -2.655972 -0.969257
O 3.278847 -2.153535 -1.204136
O 4.905969 0.064498 -2.043151
C 5.459840 -0.666797 0.158457
C 5.076046 -0.792754 1.616858
C 6.207604 -0.972922 2.594139
O 3.904826 -0.799274 1.994078
O 5.041433 2.274381 -0.001692
C 5.720093 2.708107 -1.179212
C -4.554709 0.511256 0.653535
C -4.699072 1.642436 1.635586
C -5.601279 -0.129355 0.086908
C -7.077601 0.160754 0.253168
C -7.719588 0.346568 -1.135799
C -9.179763 0.774417 -1.076501
C -7.725825 -0.976191 1.045802
H -1.715717 -2.696078 -0.648095
H -2.212460 1.960956 0.740380
H 0.207922 2.337130 0.541467
H 2.574786 2.755374 0.419486
H 1.750725 -2.850827 -1.129883
H 4.174145 0.312791 -2.634392
H 6.383849 -0.079027 0.090976
H 5.710543 -1.669966 -0.208309
H 5.809340 -1.049342 3.609689
H 6.753499 -1.889195 2.356647
H 6.876453 -0.110189 2.543654
H 6.464595 1.968765 -1.487434
H 5.021550 2.921728 -1.995624
H 6.251134 3.633377 -0.936443
H -4.515957 2.604028 1.144993
H -5.689668 1.682895 2.095733
H -3.993292 1.532099 2.466980
H -5.390995 -0.960394 -0.590123
H -7.227619 1.090578 0.810646
H -7.162773 1.111944 -1.692120
H -7.645154 -0.580607 -1.718354
H -9.299692 1.682185 -0.476921
H -9.549781 0.984141 -2.085358
H -9.811529 -0.010151 -0.650285
H -8.770984 -0.750619 1.279418
H -7.208673 -1.133575 1.999334
H -7.699459 -1.919897 0.489378
SCF Energy (B3LYP/6-31G**)= -1361.70125636
Number of imaginary frequencies = 0

1b_c147
MMFF Geometry
C -1.531312 -2.484116 -0.423161
C -2.327537 0.040104 0.188188
N -2.851638 -2.270566 -0.203753
C -0.540085 -1.499028 -0.361919
C -0.964547 -0.197136 -0.043990
C -3.237107 -1.011125 0.092971
C -0.015095 0.836835 0.034745
C 1.347810 0.596703 -0.198540
C 1.770476 -0.701233 -0.508346
C 0.829686 -1.742777 -0.595316
C 3.212438 -0.959118 -0.761033
C 4.228848 0.195947 -0.599884
C 3.600360 1.540574 -0.310008
C 2.287340 1.713079 -0.107468
O 1.196272 -3.029947 -0.915030
O 3.547072 -2.085357 -1.141564
O 4.921731 0.314837 -1.861291
C 5.296941 -0.201615 0.426716
C 4.776765 -0.263949 1.845228
C 5.792959 -0.086553 2.942262
O 3.597348 -0.500874 2.104503
O 4.484287 2.594614 -0.128261
C 4.491751 3.478484 -1.249320
C -4.687508 -0.822536 0.333639
C -5.330422 -1.917411 1.142127
C -5.335555 0.250076 -0.169287
C -6.812191 0.561674 -0.053412
C -7.084733 1.652512 1.000357
C -6.468703 3.015945 0.710494
C -7.381261 0.927854 -1.427815
H -1.278260 -3.515542 -0.659470
H -2.673887 1.033610 0.455496
H -0.348095 1.843482 0.285692
H 1.900665 2.690698 0.165150

H 2.170365 -3.051517 -1.055985
H 4.246898 0.366246 -2.559367
H 6.128146 0.514518 0.391373
H 5.729350 -1.180887 0.186578
H 5.299403 -0.149961 3.916010
H 6.546514 -0.874844 2.873651
H 6.263314 0.895625 2.850996
H 4.749980 2.949543 -2.172159
H 3.525502 3.981826 -1.358805
H 5.254013 4.242188 -1.069470
H -4.686036 -2.213979 1.978066
H -6.279671 -1.609764 1.588914
H -5.516505 -2.798244 0.518707
H -4.772316 0.972995 -0.759201
H -7.356706 -0.333909 0.266694
H -8.169581 1.779218 1.106305
H -6.719781 1.307397 1.976365
H -6.857684 3.443547 -0.217990
H -6.711215 3.711939 1.520433
H -5.378562 2.959944 0.638803
H -8.446138 1.172975 -1.353464
H -7.281799 0.086457 -2.123170
H -6.866038 1.785079 -1.874088
SCF Energy (B3LYP/6-31G**)= -1361.69904582
Number of imaginary frequencies = 0

1b_c148
MMFF Geometry
C 1.866856 -1.449782 -0.048073
C 2.258012 1.236557 0.032509
N 3.139999 -0.990852 0.018869
C 0.728874 -0.640676 -0.079962
C 0.943027 0.748951 -0.040445
C 3.331983 0.347835 0.061734
C -0.162589 1.618941 -0.083844
C -1.472880 1.125336 -0.147312
C -1.677750 -0.255516 -0.167691
C -0.587962 -1.137558 -0.154157
C -3.068186 -0.752106 -0.235382
C -4.195722 0.179656 0.271804
C -3.870136 1.634017 -0.081959
C -2.601568 2.052668 -0.255875
O -0.760063 -2.499858 -0.215403
O -3.252782 -1.897602 -0.652668
O -4.247740 0.133894 1.701187
C -5.540341 -0.260838 -0.342787
C -5.959123 -1.643314 0.117948
C -6.740359 -2.482507 -0.856187
O -5.718677 -2.036951 1.261603
O -4.984750 2.452941 -0.153675
C -4.792829 3.856682 -0.282768
C 4.746213 0.813436 0.154063
C 4.982879 2.156041 0.791801
C 5.739602 0.014740 -0.295558
C 7.224439 0.301885 -0.314602
C 7.960495 -0.406737 0.838879
C 7.908943 -1.929615 0.814894
C 7.811066 -0.056962 -1.683727
H 1.782690 -2.534058 -0.075838
H 2.432379 2.307217 0.052377
H 0.005686 2.695046 -0.066908
H -2.358146 3.085391 -0.477103
H -1.710351 -2.687947 -0.386510
H -4.632679 -0.736049 1.945907
H -5.475172 -0.256636 -1.437461
H -6.360029 0.403054 -0.042989
H -6.966802 -3.453420 -0.407066
H -7.676901 -1.977443 -1.104453
H -6.146784 -2.644124 -1.759368
H -4.210009 4.252293 0.555335
H -4.320633 4.099160 -1.240045
H -5.777308 4.333450 -0.263605
H 4.352882 2.284619 1.679484
H 6.011365 2.285334 1.139625
H 4.762964 2.962648 0.084786
H 5.468854 -0.957712 -0.711839
H 7.394886 1.377444 -0.190580
H 9.013106 -0.096709 0.830555
H 7.546805 -0.060690 1.794883
H 8.380906 -2.333608 -0.085261
H 8.448535 -2.332368 1.678520
H 6.880917 -2.300042 0.865831
H 8.889780 0.131544 -1.704496
H 7.352306 0.551506 -2.471565
H 7.645189 -1.108150 -1.942473
SCF Energy (B3LYP/6-31G**)= -1361.71229101
Number of imaginary frequencies = 0

1b_c149
MMFF Geometry
C 1.608518 2.380110 -0.080722
C 2.304779 -0.242700 -0.226516
N 2.927490 2.071372 -0.037765
C 0.571153 1.448476 -0.197474
C 0.944487 0.094642 -0.275065
C 3.259306 0.764318 -0.101735
C -0.051791 -0.889384 -0.402834
C -1.411858 -0.548567 -0.449322
C -1.781474 0.798041 -0.361961
C -0.795844 1.792759 -0.244583
C -3.219542 1.157487 -0.416862
C -4.285843 0.046093 -0.451830
C -3.719147 -1.346724 -0.616654
C -2.406706 -1.610964 -0.586384
O -1.115579 3.129260 -0.178285
O -3.542278 2.349766 -0.483146
O -5.063400 0.357475 -1.630503
C -5.223697 0.169760 0.753943
C -4.567199 -0.274631 2.040251
C -5.398675 -1.104178 2.981064
O -3.418698 0.062346 2.326823
O -4.607947 -2.415197 -0.621220
C -5.309612 -2.528401 -1.859582
C 4.710126 0.466883 -0.050989
C 5.540841 1.310303 -0.974797
C 5.168976 -0.488963 0.785373
C 6.576489 -0.954321 1.104887
C 6.899187 -2.303391 0.432083
C 6.951473 -2.273578 -1.089915
C 7.706337 0.065060 0.939808
H 1.396246 3.445208 -0.016608
H 2.618830 -1.279541 -0.297258
H 0.241488 -1.936910 -0.463986
H -2.060657 -2.639275 -0.638772
H -2.092264 3.220328 -0.247083
H -5.223651 1.322041 -1.603470
H -6.125869 -0.431998 0.585575
H -5.573423 1.199935 0.892776
H -4.809161 -1.360532 3.865637
H -6.277153 -0.533863 3.292425
H -5.703818 -2.027738 2.482849
H -6.181414 -1.868169 -1.864656
H -4.662044 -2.325330 -2.719787
H -5.672094 -3.557344 -1.943729
H 5.951583 2.175638 -0.445338
H 6.356636 0.735012 -1.418839
H 4.953075 1.680812 -1.823110
H 4.431809 -1.014882 1.396430
H 6.554413 -1.158779 2.186398
H 7.863209 -2.669114 0.807573
H 6.150899 -3.046581 0.736962
H 7.726480 -1.593332 -1.453633
H 7.184181 -3.272920 -1.472488
H 5.992351 -1.971389 -1.520349
H 8.612381 -0.297361 1.439310
H 7.442919 1.023338 1.400240
H 7.974144 0.245154 -0.103162
SCF Energy (B3LYP/6-31G**)= -1361.69532957
Number of imaginary frequencies = 0

1b_c150
MMFF Geometry

C -1.532992 -2.048855 -0.816385
C -2.321492 0.543977 -0.601326
N -2.862795 -1.788678 -0.790883
C -0.528061 -1.079677 -0.741112
C -0.948319 0.260645 -0.632155
C -3.243401 -0.498508 -0.673814
C 0.013362 1.286209 -0.570070
C 1.381649 0.992712 -0.591333
C 1.791886 -0.342305 -0.680477
C 0.849672 -1.373723 -0.777146
C 3.246260 -0.620616 -0.696084
C 4.182735 0.432794 -0.048531
C 3.664895 1.829269 -0.376975
C 2.368268 2.073260 -0.618904
O 1.227104 -2.686987 -0.916137
O 3.643545 -1.680309 -1.181973
O 5.503652 0.289340 -0.577724
C 4.267295 0.214236 1.466617
C 4.342006 -1.250739 1.834518
C 5.663986 -1.954000 1.678632
O 3.334428 -1.845278 2.220173
O 4.573421 2.856125 -0.592344
C 5.326028 3.209353 0.565000
C -4.706299 -0.259037 -0.652165
C -5.485479 -1.093693 -1.632656
C -5.243994 0.621683 0.218726
C -6.709016 0.949576 0.410641
C -7.152756 0.715990 1.867850
C -7.070541 -0.745351 2.291889
C -6.952922 2.408001 0.014477
H -1.284451 -3.104611 -0.903006
H -2.669480 1.570220 -0.536153
H -0.314175 2.323460 -0.510230
H 2.036065 3.071183 -0.891434
H 2.204297 -2.728071 -1.018643
H 5.447414 0.495598 -1.528950
H 3.385782 0.624049 1.976894
H 5.142628 0.705029 1.906469
H 5.625058 -2.919117 2.191662
H 5.876998 -2.121649 0.621148
H 6.457478 -1.355743 2.133332
H 4.684853 3.297220 1.448695
H 6.130269 2.489806 0.740381
H 5.785565 4.184965 0.380759
H -5.609942 -2.114268 -1.255512
H -6.477507 -0.686405 -1.842611
H -4.969477 -1.141852 -2.598817
H -4.583352 1.148712 0.907669
H -7.340646 0.325299 -0.228849
H -6.548866 1.322818 2.554384
H -8.191553 1.049155 1.985608
H -6.037956 -1.107337 2.291949
H -7.464469 -0.864085 3.306382
H -7.660051 -1.381033 1.623562
H -8.013241 2.664280 0.114297
H -6.665575 2.584509 -1.028173
H -6.379939 3.098285 0.644096
SCF Energy (B3LYP/6-31G**)= -1361.69402154
Number of imaginary frequencies = 0

1b_c151
MMFF Geometry
C -1.755819 -1.307876 -0.641295
C -2.079956 1.284373 0.101312
N -3.016036 -0.853972 -0.436421
C -0.599057 -0.538253 -0.499239
C -0.778335 0.802720 -0.113705
C -3.174932 0.437573 -0.066289
C 0.347761 1.633121 0.041351
C 1.644725 1.145140 -0.168585
C 1.812811 -0.192440 -0.537146
C 0.703477 -1.028587 -0.720785
C 3.186219 -0.681791 -0.762584
C 4.358565 0.093315 -0.130753
C 4.057207 1.584861 -0.043330
C 2.793318 2.050391 -0.053086
O 0.839509 -2.335528 -1.121424
O 3.370827 -1.694903 -1.443174
O 5.494437 -0.097603 -0.994087
C 4.725346 -0.429276 1.260301
C 4.648839 -1.934114 1.352364
C 5.859622 -2.727546 0.940464
O 3.607977 -2.484257 1.714722
O 5.189700 2.367440 0.094072
C 5.047356 3.782104 0.100361
C -4.577901 0.902959 0.135200
C -4.844113 2.372395 -0.051772
C -5.539654 0.002449 0.436912
C -7.005845 0.263478 0.700930
C -7.878666 -0.079368 -0.521803
C -7.865930 -1.541946 -0.949697
C -7.453772 -0.484416 1.960862
H -1.698717 -2.353815 -0.935267
H -2.226602 2.313893 0.410599
H 0.204140 2.674511 0.327340
H 2.568677 3.107587 0.035217
H 1.786949 -2.514759 -1.311848
H 5.266132 -0.822461 -1.609912
H 4.041928 -0.036882 2.025209
H 5.736433 -0.120404 1.552877
H 5.722528 -3.775048 1.223430
H 5.995666 -2.666588 -0.141013
H 6.744764 -2.345231 1.454823
H 4.584480 4.134055 -0.827241
H 4.476634 4.112595 0.974006
H 6.047346 4.220867 0.166185
H -4.308879 2.759218 -0.926585
H -5.899804 2.593112 -0.231405
H -4.528076 2.936778 0.831654
H -5.252835 -1.047962 0.519316
H -7.159412 1.327154 0.916006
H -8.915744 0.208968 -0.308480
H -7.559285 0.534173 -1.374092
H -8.250719 -2.194798 -0.161051
H -8.504529 -1.676408 -1.829090
H -6.859423 -1.875652 -1.218458
H -8.518745 -0.315985 2.153356
H -6.897952 -0.131512 2.837165
H -7.289900 -1.564366 1.881401
SCF Energy (B3LYP/6-31G**)= -1361.70454373
Number of imaginary frequencies = 0

1b_c152
MMFF Geometry
C -1.671883 -1.296929 -0.824667
C -2.088420 1.172589 0.226001
N -2.949364 -0.904547 -0.600303
C -0.541334 -0.522106 -0.555852
C -0.768753 0.755126 -0.011453
C -3.154143 0.325912 -0.076410
C 0.328271 1.588086 0.277473
C 1.642632 1.163571 0.042931
C 1.860846 -0.113791 -0.484729
C 0.780023 -0.948137 -0.800232
C 3.254606 -0.538752 -0.735681
C 4.398964 0.193719 -0.006946
C 4.029639 1.642306 0.249928
C 2.759462 2.071320 0.309472
O 0.961844 -2.191771 -1.355065
O 3.481025 -1.465090 -1.520117
O 5.537616 0.155465 -0.884861
C 4.783799 -0.475975 1.314254
C 4.752975 -1.982963 1.232329
C 6.002103 -2.687841 0.776447
O 3.720439 -2.601555 1.493210
O 5.068155 2.490773 0.603166
C 5.477863 3.293186 -0.504812
C -4.574365 0.726644 0.143032
C -4.877588 2.200612 0.125983
C -5.514406 -0.229429 0.313439
C -6.995081 -0.048087 0.570270
C -7.784121 -0.464346 -0.683982

C -9.280121 -0.203812 -0.573209
C -7.394212 -0.860959 1.806095
H -1.577692 -2.296090 -1.244717
H -2.271968 2.150924 0.657714
H 0.149371 2.581048 0.688698
H 2.539877 3.100404 0.578569
H 1.919047 -2.326064 -1.532751
H 5.375890 -0.569180 -1.522817
H 4.090411 -0.196925 2.118937
H 5.785329 -0.171286 1.641937
H 5.880342 -3.766896 0.906520
H 6.185976 -2.476253 -0.278657
H 6.851808 -2.361270 1.381053
H 5.792844 2.678730 -1.353889
H 4.676490 3.974775 -0.809374
H 6.332841 3.895787 -0.184556
H -4.326766 2.704732 -0.676478
H -5.932595 2.415606 -0.062904
H -4.605648 2.660910 1.081506
H -5.199464 -1.274751 0.276771
H -7.220957 0.999322 0.794588
H -7.405646 0.092354 -1.551233
H -7.623360 -1.528231 -0.900419
H -9.479573 0.843722 -0.326551
H -9.770634 -0.426201 -1.526426
H -9.742694 -0.835441 0.190629
H -8.430434 -0.658573 2.094740
H -6.763205 -0.603730 2.664662
H -7.297192 -1.937856 1.627843
SCF Energy (B3LYP/6-31G**)= -1361.70027967
Number of imaginary frequencies = 0

1b_c153
MMFF Geometry
C 1.642754 2.382259 0.220794
C 2.332091 -0.077817 -0.701081
N 2.960830 2.111472 0.058197
C 0.602141 1.488888 -0.054152
C 0.971550 0.220485 -0.534029
C 3.293198 0.881972 -0.389337
C -0.029320 -0.719055 -0.837355
C -1.389702 -0.417562 -0.669632
C -1.756551 0.842152 -0.184151
C -0.764820 1.792332 0.118317
C -3.196416 1.167513 -0.013111
C -4.264073 0.106625 -0.377563
C -3.697126 -1.203991 -0.884409
C -2.383901 -1.437799 -0.993661
O -1.078419 3.049464 0.581653
O -3.497681 2.305716 0.360453
O -5.044455 0.687068 -1.444669
C -5.220050 -0.072469 0.809442
C -4.570302 -0.721553 2.012174
C -5.499031 -1.346804 3.019379
O -3.353775 -0.703937 2.196164
O -4.569685 -2.265884 -1.091945
C -5.422788 -2.079989 -2.220175
C 4.744952 0.623183 -0.554412
C 5.513848 1.776702 -1.141491
C 5.276498 -0.551207 -0.151444
C 6.723087 -0.989011 -0.228783
C 7.425407 -0.893037 1.139982
C 6.864953 -1.793501 2.234193
C 6.810934 -2.395740 -0.828841
H 1.434465 3.382695 0.594231
H 2.638340 -1.046565 -1.083626
H 0.261207 -1.702553 -1.205772
H -2.034432 -2.420497 -1.298291
H -2.056944 3.114926 0.668510
H -4.423456 0.958527 -2.142840
H -6.080765 -0.682053 0.507112
H -5.629879 0.892764 1.132043
H -4.917602 -1.782602 3.836580
H -6.165753 -0.582459 3.425848
H -6.079443 -2.139212 2.540392
H -6.278808 -1.453267 -1.954919
H -4.881213 -1.659007 -3.074339
H -5.807397 -3.061556 -2.512861
H 5.535749 2.622604 -0.445688
H 6.551119 1.531756 -1.377995
H 5.049115 2.109037 -2.076840
H 4.618827 -1.284866 0.315748
H 7.276993 -0.337157 -0.911886
H 8.488520 -1.131529 1.008799
H 7.385755 0.146187 1.491184
H 6.948714 -2.850764 1.966982
H 7.427096 -1.643002 3.161881
H 5.815316 -1.568905 2.444640
H 7.851539 -2.734740 -0.871270
H 6.416827 -2.404141 -1.851581
H 6.238426 -3.128800 -0.250593
SCF Energy (B3LYP/6-31G**)= -1361.69999096
Number of imaginary frequencies = 0

1b_c154
MMFF Geometry
C -1.650625 -1.501602 -0.460456
C -2.103593 0.989622 0.521730
N -2.925882 -1.154187 -0.161623
C -0.540026 -0.669449 -0.302514
C -0.786495 0.619337 0.205154
C -3.148397 0.086510 0.329832
C 0.288544 1.511255 0.376922
C 1.601012 1.133626 0.064545
C 1.839856 -0.155700 -0.423610
C 0.778864 -1.048872 -0.624692
C 3.230362 -0.531171 -0.757831
C 4.388661 0.282305 -0.146453
C 3.971962 1.723787 0.075467
C 2.690781 2.099871 0.209916
O 0.977709 -2.306704 -1.140314
O 3.442935 -1.481624 -1.516887
O 5.462350 0.253298 -1.103060
C 4.897479 -0.309402 1.170042
C 4.929063 -1.818512 1.154986
C 6.172473 -2.490459 0.637869
O 3.947209 -2.467272 1.518197
O 4.992658 2.631437 0.315321
C 5.284307 3.399711 -0.852612
C -4.562486 0.425384 0.663398
C -4.780083 1.465706 1.728842
C -5.565913 -0.222750 0.031087
C -7.057737 -0.025470 0.195384
C -7.691895 0.235761 -1.185017
C -9.173712 0.579515 -1.116227
C -7.653015 -1.254549 0.885391
H -1.540941 -2.512694 -0.846851
H -2.304123 1.985694 0.902326
H 0.093700 2.513222 0.757953
H 2.443522 3.129728 0.450553
H 1.925137 -2.407683 -1.381205
H 5.289085 -0.505664 -1.696527
H 4.251844 -0.023193 2.011124
H 5.905148 0.051828 1.409603
H 6.109334 -3.566603 0.822338
H 6.269722 -2.319754 -0.436015
H 7.048092 -2.101248 1.163228
H 5.565666 2.761525 -1.695976
H 4.432494 4.031650 -1.125716
H 6.131264 4.052466 -0.621847
H -4.643717 2.471601 1.318416
H -5.776858 1.416596 2.174579
H -4.079384 1.327533 2.560373
H -5.302933 -0.985019 -0.705834
H -7.264677 0.847876 0.821722
H -7.170620 1.071346 -1.670202
H -7.560273 -0.636965 -1.837389
H -9.349917 1.429161 -0.449162
H -9.542244 0.848776 -2.111402
H -9.767198 -0.268816 -0.763460
H -8.711626 -1.104000 1.118833
H -7.139740 -1.459510 1.831954

H -7.568791 -2.148353 0.256977
SCF Energy (B3LYP/6-31G**)= -1361.70035424
Number of imaginary frequencies = 0

1b_c155
MMFF Geometry
C 1.665904 2.518591 -0.016786
C 2.379766 -0.098133 0.158574
N 2.976753 2.228264 0.164377
C 0.644232 1.569348 -0.120286
C 1.025844 0.217631 -0.026901
C 3.322001 0.924990 0.242152
C 0.045176 -0.786183 -0.125480
C -1.306395 -0.463641 -0.304420
C -1.682021 0.880784 -0.383922
C -0.714815 1.893553 -0.308224
C -3.116717 1.205626 -0.556349
C -4.175241 0.126068 -0.251744
C -3.607886 -1.259238 -0.477275
C -2.294376 -1.529642 -0.455952
O -1.049506 3.222782 -0.408932
O -3.440964 2.348423 -0.895667
O -5.250024 0.366422 -1.173593
C -4.670386 0.383325 1.175022
C -5.561288 -0.702317 1.737276
C -6.941974 -0.860234 1.154858
O -5.177804 -1.405734 2.674172
O -4.526129 -2.290683 -0.596081
C -4.706437 -2.673924 -1.959771
C 4.765105 0.653211 0.444810
C 5.437799 1.574647 1.426681
C 5.381118 -0.334877 -0.238988
C 6.847044 -0.713542 -0.186699
C 6.991029 -2.091993 0.483133
C 8.438260 -2.513974 0.696747
C 7.422715 -0.692328 -1.606119
H 1.448113 3.582791 -0.077856
H 2.694638 -1.133088 0.251181
H 0.344601 -1.832159 -0.062767
H -1.944102 -2.553319 -0.553438
H -1.996737 3.294202 -0.663200
H -5.291743 1.335178 -1.306167
H -5.232877 1.324182 1.231232
H -3.820725 0.490937 1.862693
H -7.525842 -1.538348 1.784020
H -7.445519 0.109593 1.135590
H -6.889592 -1.276852 0.148103
H -5.020054 -1.826637 -2.577695
H -3.790674 -3.117749 -2.364280
H -5.495080 -3.431217 -1.993770
H 4.796782 1.749424 2.298820
H 6.372265 1.165479 1.819111
H 5.657705 2.540487 0.959803
H 4.798764 -0.928781 -0.943944
H 7.413811 0.018056 0.397804
H 6.494671 -2.072310 1.462188
H 6.480522 -2.860473 -0.111320
H 8.989730 -1.755593 1.261269
H 8.475711 -3.450553 1.262520
H 8.952331 -2.682496 -0.253890
H 8.506656 -0.843885 -1.595537
H 7.235863 0.273241 -2.090077
H 6.978192 -1.474144 -2.232253
SCF Energy (B3LYP/6-31G**)= -1361.70158283
Number of imaginary frequencies = 0

1b_c156
MMFF Geometry
C -1.502687 -2.087327 -0.940307
C -2.358678 0.466712 -0.580516
N -2.837970 -1.856183 -0.955920
C -0.524045 -1.106205 -0.753409
C -0.979167 0.213538 -0.570989
C -3.253338 -0.585831 -0.765570
C -0.044290 1.249860 -0.392415
C 1.331964 0.988954 -0.377825
C 1.776564 -0.327668 -0.541997
C 0.860827 -1.368787 -0.746803
C 3.233413 -0.580238 -0.534873
C 4.167961 0.469548 0.098948
C 3.590627 1.863557 -0.056298
C 2.280945 2.094309 -0.236380
O 1.269825 -2.664285 -0.951881
O 3.677808 -1.618580 -1.033964
O 5.411881 0.401451 -0.619921
C 4.448432 0.196057 1.578367
C 4.615650 -1.274408 1.875727
C 5.992838 -1.866424 1.742053
O 3.641608 -1.965307 2.176985
O 4.458285 2.920259 0.175458
C 4.911577 3.493619 -1.051382
C -4.721687 -0.373651 -0.792075
C -5.438356 -1.156713 -1.859732
C -5.309233 0.431451 0.119368
C -6.780547 0.759130 0.254769
C -7.438005 -0.021092 1.410179
C -6.900961 0.284804 2.803109
C -6.966837 2.273008 0.396744
H -1.226917 -3.129285 -1.089030
H -2.732058 1.478874 -0.458767
H -0.399883 2.271822 -0.264949
H 1.900888 3.111518 -0.259162
H 2.251590 -2.687525 -0.990398
H 5.429453 -0.468363 -1.068644
H 3.622444 0.546484 2.211703
H 5.350349 0.719094 1.919321
H 5.988788 -2.890323 2.126396
H 6.291622 -1.882816 0.692156
H 6.704968 -1.280684 2.328548
H 5.411953 2.752868 -1.682758
H 4.080644 3.953993 -1.596471
H 5.634293 4.277984 -0.808217
H -5.391690 -2.231006 -1.650233
H -6.494289 -0.896177 -1.954509
H -4.981314 -0.974226 -2.839075
H -4.682885 0.895866 0.881530
H -7.314131 0.476327 -0.658282
H -8.516428 0.182059 1.404719
H -7.328165 -1.097313 1.224206
H -7.053566 1.334072 3.071382
H -7.427513 -0.324535 3.545206
H -5.834599 0.055314 2.884821
H -8.026223 2.523557 0.517189
H -6.602247 2.792028 -0.497169
H -6.422350 2.678152 1.256339
SCF Energy (B3LYP/6-31G**)= -1361.69887088
Number of imaginary frequencies = 0

1b_c157
MMFF Geometry
C 1.718267 2.442455 0.345112
C 2.359983 -0.022436 -0.603538
N 3.030860 2.136044 0.208468
C 0.660428 1.582261 0.034209
C 1.004999 0.309801 -0.459361
C 3.339167 0.905376 -0.254290
C -0.013509 -0.597192 -0.804372
C -1.365005 -0.261944 -0.650099
C -1.701947 0.997145 -0.144050
C -0.699695 1.922378 0.182020
C -3.134783 1.329704 0.029174
C -4.176313 0.192204 0.035578
C -3.693862 -0.980133 -0.791924
C -2.400289 -1.206244 -1.064740
O -0.999638 3.178061 0.653504
O -3.464257 2.506234 0.212083
O -5.361329 0.748413 -0.555250
C -4.456731 -0.141632 1.504226
C -5.294573 -1.382842 1.718326
C -6.748329 -1.334583 1.325002
O -4.805772 -2.387263 2.239714
O -4.654491 -1.903758 -1.173070

C -5.036505 -1.726003 -2.537442
C 4.786240 0.613079 -0.390165
C 5.598993 1.751863 -0.945175
C 5.283707 -0.581984 -0.005759
C 6.730941 -1.029249 -0.030017
C 7.155098 -1.458754 1.387931
C 8.633904 -1.804023 1.499791
C 6.887595 -2.158238 -1.051090
H 1.530465 3.443263 0.728253
H 2.648861 -0.991938 -0.997849
H 0.255861 -1.576952 -1.198281
H -2.102256 -2.101180 -1.603609
H -1.970356 3.320046 0.586134
H -5.387865 1.690602 -0.291855
H -4.974480 0.687782 2.002991
H -3.515615 -0.290269 2.050670
H -7.259119 -2.220211 1.714026
H -7.217267 -0.448536 1.760326
H -6.852966 -1.320414 0.239311
H -5.403959 -0.711878 -2.723498
H -4.202006 -1.954336 -3.208787
H -5.847766 -2.427543 -2.752620
H 5.789790 2.501983 -0.170478
H 6.561436 1.428623 -1.349481
H 5.072308 2.237463 -1.775168
H 4.602365 -1.325564 0.409022
H 7.384976 -0.206970 -0.336012
H 6.941535 -0.643669 2.091737
H 6.563316 -2.321765 1.718919
H 9.258681 -0.976423 1.149565
H 8.893507 -2.005277 2.544200
H 8.883009 -2.697559 0.920238
H 7.938865 -2.435365 -1.177062
H 6.514452 -1.851146 -2.034918
H 6.335421 -3.054629 -0.746985
SCF Energy (B3LYP/6-31G**)= -1361.70168232
Number of imaginary frequencies = 0

1b_c158
MMFF Geometry
C -1.756867 -1.463730 -0.806218
C -2.012717 1.065269 0.145602
N -3.005905 -0.992212 -0.573117
C -0.579031 -0.742285 -0.596099
C -0.723975 0.565333 -0.103900
C -3.130420 0.268038 -0.097212
C 0.424353 1.343563 0.127232
C 1.710995 0.841683 -0.123107
C 1.853623 -0.464200 -0.605981
C 0.713076 -1.250221 -0.847153
C 3.213975 -0.998500 -0.877017
C 4.449869 -0.128738 -0.546352
C 4.117510 1.270677 -0.079110
C 2.866295 1.700495 0.131918
O 0.803071 -2.531713 -1.340066
O 3.310857 -2.109747 -1.407029
O 5.202973 0.000918 -1.771669
C 5.363121 -0.885715 0.426163
C 4.784273 -1.020872 1.816613
C 5.768788 -1.225720 2.937503
O 3.572071 -1.019982 2.028541
O 5.201117 2.069583 0.256960
C 5.448773 3.068864 -0.732053
C -4.521000 0.756009 0.136206
C -4.741747 2.243278 0.071964
C -5.509030 -0.138598 0.360600
C -6.971069 0.135102 0.641138
C -7.810245 -0.279545 -0.580689
C -9.286502 0.068874 -0.448640
C -7.386914 -0.610116 1.913567
H -1.727108 -2.483547 -1.183839
H -2.130757 2.068666 0.540972
H 0.309034 2.356214 0.512489
H 2.690862 2.694118 0.533769
H 1.753979 -2.749832 -1.471767
H 4.583972 0.289572 -2.463678
H 6.330705 -0.372540 0.499843
H 5.582000 -1.895151 0.056355
H 5.233976 -1.307743 3.887793
H 6.331937 -2.146248 2.765906
H 6.447659 -0.370959 2.989992
H 5.626798 2.621382 -1.714966
H 4.620008 3.782531 -0.785170
H 6.349507 3.615723 -0.438352
H -4.183907 2.686563 -0.760980
H -5.787435 2.511141 -0.100347
H -4.420723 2.720956 1.003429
H -5.253795 -1.200691 0.355926
H -7.133091 1.200796 0.831984
H -7.421011 0.224155 -1.475153
H -7.714001 -1.357744 -0.761702
H -9.421598 1.134003 -0.236039
H -9.810070 -0.158665 -1.382870
H -9.766201 -0.508458 0.346945
H -8.403438 -0.339443 2.215968
H -6.723294 -0.359091 2.749073
H -7.354199 -1.696358 1.772492
SCF Energy (B3LYP/6-31G**)= -1361.70101013
Number of imaginary frequencies = 0

1b_c159
MMFF Geometry
C -1.882726 -1.405769 -0.599982
C -2.083457 1.202813 0.122685
N -3.120747 -0.890718 -0.403301
C -0.689863 -0.691729 -0.461840
C -0.806363 0.657148 -0.087969
C -3.217981 0.409366 -0.041663
C 0.358386 1.429561 0.067480
C 1.633266 0.882333 -0.145414
C 1.747781 -0.462677 -0.512233
C 0.590671 -1.245263 -0.673051
C 3.095413 -1.043389 -0.746471
C 4.348089 -0.147790 -0.582372
C 4.047528 1.281293 -0.177963
C 2.806495 1.733284 0.038245
O 0.652651 -2.567989 -1.046998
O 3.167988 -2.216348 -1.126842
O 4.976509 -0.111419 -1.881750
C 5.341641 -0.841358 0.359343
C 4.857319 -0.922330 1.790747
C 5.910468 -1.152735 2.842285
O 3.665723 -0.855168 2.090192
O 5.112019 2.116695 0.139908
C 5.890880 2.491452 -0.995069
C -4.597432 0.943297 0.154130
C -4.789349 2.424776 -0.029302
C -5.604455 0.091155 0.448777
C -7.056972 0.424212 0.707163
C -7.940042 0.129843 -0.520831
C -7.998245 -1.329974 -0.954352
C -7.547698 -0.304935 1.962149
H -1.875253 -2.455661 -0.884874
H -2.178965 2.239837 0.426865
H 0.265973 2.474252 0.363227
H 2.656683 2.749279 0.393076
H 1.599191 -2.821128 -1.143306
H 4.300548 0.170046 -2.522547
H 6.301784 -0.310545 0.345976
H 5.554732 -1.863042 0.021011
H 5.443310 -1.190250 3.830291
H 6.415722 -2.102682 2.651817
H 6.630823 -0.331130 2.825856
H 6.607943 1.704596 -1.245243
H 5.261164 2.733855 -1.858100
H 6.459803 3.387086 -0.728177
H -4.234555 2.786371 -0.902717
H -5.832360 2.698933 -0.209272
H -4.446328 2.970355 0.855878
H -5.370705 -0.972383 0.529882
H -7.158274 1.493408 0.925732
H -8.962441 0.468784 -0.311086

H -7.586438 0.729803 -1.369268
H -8.418903 -1.965685 -0.170031
H -8.638536 -1.429179 -1.837191
H -7.008362 -1.712515 -1.219766
H -8.603808 -0.084122 2.150466
H -6.979062 0.016510 2.842324
H -7.437694 -1.391436 1.879242
SCF Energy (B3LYP/6-31G**)= -1361.70055724
Number of imaginary frequencies = 0

1b_c160
MMFF Geometry
C 1.983885 1.632710 0.366884
C 2.121708 -1.058952 0.029460
N 3.191855 1.033059 0.495388
C 0.791927 0.967179 0.072149
C 0.875034 -0.426185 -0.102939
C 3.257867 -0.308105 0.328589
C -0.288978 -1.153512 -0.414949
C -1.531627 -0.517309 -0.535854
C -1.608778 0.864596 -0.343397
C -0.455603 1.609392 -0.059115
C -2.933994 1.515672 -0.451489
C -4.189681 0.631743 -0.271570
C -3.948972 -0.761451 -0.842828
C -2.713570 -1.288152 -0.929938
O -0.499336 2.973334 0.102679
O -2.969374 2.735573 -0.623024
O -5.277508 1.257528 -0.961418
C -4.521991 0.634621 1.225106
C -5.569588 -0.386307 1.607606
C -7.008626 -0.075081 1.289378
O -5.248514 -1.445067 2.152372
O -5.111545 -1.399905 -1.233965
C -5.053351 -2.789157 -1.538396
C 4.602918 -0.933015 0.495535
C 4.630622 -2.372233 0.934771
C 5.708331 -0.188142 0.270431
C 7.159887 -0.603297 0.364512
C 7.861523 -0.554205 -1.008833
C 7.991230 0.825971 -1.641711
C 7.877154 0.248049 1.414935
H 2.001238 2.710406 0.514794
H 2.197117 -2.131605 -0.115630
H -0.219120 -2.230458 -0.565382
H -2.536064 -2.286117 -1.315784
H -1.393765 3.293679 -0.156766
H -5.138263 1.102369 -1.912049
H -4.883617 1.619586 1.546663
H -3.630346 0.423667 1.830112
H -7.658676 -0.759157 1.842423
H -7.244403 0.945934 1.599705
H -7.194012 -0.196595 0.220717
H -4.459531 -2.964934 -2.440825
H -4.660542 -3.360763 -0.691226
H -6.073275 -3.132882 -1.734249
H 4.419182 -3.036155 0.090049
H 5.589542 -2.672811 1.362855
H 3.887725 -2.555552 1.719699
H 5.567813 0.854035 -0.023106
H 7.236216 -1.642062 0.700136
H 8.868659 -0.977403 -0.903035
H 7.326378 -1.209078 -1.708624
H 8.578231 1.502782 -1.014376
H 8.503945 0.743986 -2.605962
H 7.014049 1.280684 -1.826992
H 8.938243 -0.017368 1.471172
H 7.440992 0.084536 2.407098
H 7.805543 1.319175 1.197687
SCF Energy (B3LYP/6-31G**)= -1361.70224668
Number of imaginary frequencies = 0

1b_c161
MMFF Geometry
C -1.736736 -1.664126 -0.387305
C -2.025670 0.907709 0.431270
N -2.983852 -1.224924 -0.089716
C -0.577160 -0.888669 -0.308599
C -0.739511 0.441290 0.113428
C -3.124108 0.056216 0.321492
C 0.388886 1.275493 0.205514
C 1.673183 0.806768 -0.112009
C 1.834232 -0.521563 -0.522445
C 0.712867 -1.363345 -0.626496
C 3.191895 -1.021346 -0.863689
C 4.411274 -0.086481 -0.683779
C 4.056043 1.327150 -0.280573
C 2.807040 1.722936 -0.000991
O 0.819514 -2.669453 -1.046017
O 3.295736 -2.160462 -1.329311
O 5.065115 -0.009277 -1.969046
C 5.425236 -0.743859 0.260970
C 4.957603 -0.809530 1.697546
C 6.030508 -0.906429 2.749755
O 3.765565 -0.838071 2.001838
O 5.127133 2.186161 -0.079946
C 5.256864 3.128043 -1.144886
C -4.508314 0.497902 0.659816
C -4.640771 1.614689 1.659820
C -5.561598 -0.129264 0.090620
C -7.035398 0.165056 0.271197
C -7.685741 0.374929 -1.110433
C -9.143464 0.808530 -1.034817
C -7.683664 -0.980817 1.050831
H -1.692687 -2.703654 -0.705043
H -2.159561 1.936301 0.749321
H 0.260068 2.306107 0.534790
H 2.620278 2.733561 0.350149
H 1.766397 -2.860306 -1.236123
H 4.385126 0.212191 -2.627783
H 6.373174 -0.191296 0.228031
H 5.658271 -1.766416 -0.061058
H 5.571866 -0.945947 3.741667
H 6.616010 -1.815477 2.592357
H 6.676314 -0.026436 2.696996
H 5.378067 2.624959 -2.109398
H 4.397396 3.805765 -1.176429
H 6.153726 3.725528 -0.957042
H -4.457098 2.582814 1.182466
H -5.627860 1.652250 2.127697
H -3.929481 1.488904 2.484298
H -5.359685 -0.950978 -0.600215
H -7.177304 1.086975 0.843733
H -7.129057 1.146114 -1.658805
H -7.619543 -0.543608 -1.707487
H -9.255132 1.707612 -0.420728
H -9.519204 1.035278 -2.037860
H -9.776054 0.020478 -0.616321
H -8.726168 -0.754016 1.294885
H -7.160910 -1.155067 1.998358
H -7.665450 -1.916061 0.479981
SCF Energy (B3LYP/6-31G**)= -1361.70095889
Number of imaginary frequencies = 0

1b_c162
MMFF Geometry
C -1.670742 -2.144865 -0.115175
C -2.223782 0.490389 0.244617
N -2.958495 -1.792961 0.118865
C -0.597306 -1.250731 -0.188380
C -0.895907 0.109433 0.000499
C -3.222538 -0.480072 0.289253
C 0.141910 1.056309 -0.058723
C 1.470834 0.673396 -0.303384
C 1.766344 -0.680410 -0.487121
C 0.736786 -1.637688 -0.433345
C 3.168891 -1.090233 -0.743611
C 4.278686 -0.015537 -0.773169
C 3.797885 1.413287 -0.576560
C 2.504364 1.709414 -0.363100
O 0.979764 -2.979248 -0.620226
O 3.396548 -2.287033 -0.948993

O 4.867769 -0.087262 -2.090617
C 5.391210 -0.420725 0.203999
C 4.973625 -0.384312 1.657560
C 6.076258 -0.547839 2.670894
O 3.802065 -0.259908 2.012033
O 4.821485 2.342370 -0.649206
C 4.514073 3.717278 -0.450711
C -4.641811 -0.137543 0.549996
C -5.319195 -1.055735 1.528903
C -5.216682 0.903716 -0.090111
C -6.642282 1.413477 -0.033261
C -7.741996 0.376707 -0.333913
C -7.569101 -0.314357 -1.680668
C -6.918310 2.158240 1.275468
H -1.515883 -3.213625 -0.247570
H -2.474837 1.533083 0.412523
H -0.095737 2.108892 0.091938
H 2.168578 2.730198 -0.218331
H 1.943989 -3.101248 -0.779665
H 4.265920 0.368449 -2.702763
H 6.259538 0.238624 0.078794
H 5.744359 -1.436426 -0.015152
H 5.659122 -0.502018 3.680708
H 6.560804 -1.517229 2.531218
H 6.803993 0.258966 2.554206
H 3.829294 4.080160 -1.223806
H 4.103292 3.885659 0.549954
H 5.445591 4.285086 -0.532448
H -4.659421 -1.277046 2.376120
H -6.223580 -0.622782 1.959216
H -5.591045 -2.000702 1.046738
H -4.607456 1.467827 -0.797990
H -6.715969 2.168793 -0.829883
H -7.803988 -0.381667 0.452320
H -8.713652 0.887418 -0.337182
H -6.664761 -0.929524 -1.705232
H -8.422541 -0.971485 -1.876694
H -7.513166 0.417247 -2.492972
H -7.889956 2.662593 1.231928
H -6.156939 2.924448 1.459990
H -6.934404 1.486105 2.138306
SCF Energy (B3LYP/6-31G**)= -1361.70202406
Number of imaginary frequencies = 0

1b_c163
MMFF Geometry
C -1.592530 -2.395440 -0.287815
C -2.303794 0.225664 -0.230592
N -2.912579 -2.097825 -0.210839
C -0.561093 -1.451873 -0.343260
C -0.941914 -0.099433 -0.316345
C -3.253513 -0.792285 -0.171885
C 0.049269 0.895920 -0.379063
C 1.412492 0.568891 -0.463502
C 1.789772 -0.776918 -0.482203
C 0.807545 -1.782633 -0.426760
C 3.228696 -1.127534 -0.568417
C 4.282594 0.001804 -0.605273
C 3.716153 1.412637 -0.599297
C 2.395258 1.652491 -0.534786
O 1.132099 -3.119573 -0.458384
O 3.532947 -2.323299 -0.630986
O 4.998899 -0.162326 -1.849407
C 5.313983 -0.242242 0.505304
C 4.756912 -0.090529 1.903594
C 5.763190 -0.094003 3.024663
O 3.552106 0.001260 2.135314
O 4.695520 2.388114 -0.673495
C 4.303836 3.755624 -0.645706
C -4.706841 -0.507330 -0.089789
C -5.545346 -1.355994 -1.004508
C -5.164989 0.428807 0.769265
C -6.584251 0.870517 1.061865
C -7.231304 1.696642 -0.065336
C -6.442324 2.949657 -0.425436
C -7.493176 -0.279001 1.507573
H -1.374408 -3.461212 -0.305703
H -2.622141 1.263506 -0.222920
H -0.252687 1.942564 -0.357459
H 1.997801 2.661280 -0.525715
H 2.112054 -3.200286 -0.515035
H 4.437832 0.197331 -2.556850
H 6.157193 0.451275 0.393755
H 5.735644 -1.252119 0.423155
H 5.248955 0.024656 3.982425
H 6.304015 -1.043481 3.028005
H 6.460016 0.737625 2.894373
H 3.679420 4.000931 -1.510699
H 3.791333 3.996668 0.291130
H 5.210105 4.365929 -0.700650
H -5.649540 -2.369614 -0.603049
H -6.546784 -0.952312 -1.159920
H -5.088615 -1.422969 -1.998936
H -4.437131 0.946622 1.396141
H -6.510185 1.536419 1.934547
H -7.366349 1.091852 -0.968127
H -8.235546 2.005055 0.251989
H -5.464143 2.701597 -0.848503
H -6.988502 3.534571 -1.172519
H -6.289452 3.583440 0.453850
H -8.425649 0.116496 1.925968
H -7.013003 -0.882641 2.285646
H -7.766511 -0.942434 0.682893
SCF Energy (B3LYP/6-31G**)= -1361.70212056
Number of imaginary frequencies = 0

1b_c164
MMFF Geometry
C -1.766926 -1.232831 -0.715732
C -2.114740 1.285203 0.240501
N -3.032287 -0.802676 -0.492075
C -0.616009 -0.474079 -0.491411
C -0.807854 0.828561 0.004261
C -3.202989 0.451896 -0.015155
C 0.311146 1.647006 0.246670
C 1.612584 1.183587 0.013689
C 1.795412 -0.118336 -0.465122
C 0.692251 -0.939583 -0.734248
C 3.175998 -0.584670 -0.715424
C 4.345665 0.149752 -0.030129
C 4.012152 1.615447 0.173090
C 2.752755 2.075413 0.230526
O 0.839090 -2.208053 -1.241146
O 3.371604 -1.546307 -1.464890
O 5.471877 0.050711 -0.919266
C 4.732256 -0.475678 1.312087
C 4.666660 -1.983629 1.290751
C 5.893768 -2.734414 0.848566
O 3.623951 -2.567610 1.588360
O 5.073814 2.453280 0.479843
C 5.487208 3.201568 -0.664060
C -4.611111 0.893105 0.204566
C -4.881155 2.372505 0.144287
C -5.573324 -0.033568 0.410453
C -7.044506 0.198479 0.673772
C -7.897088 -0.037140 -0.587982
C -7.871285 -1.455895 -1.143734
C -7.508227 -0.659716 1.855251
H -1.700545 -2.249724 -1.096721
H -2.270525 2.283944 0.634716
H 0.159785 2.659319 0.620028
H 2.559704 3.119104 0.460923
H 1.790703 -2.371145 -1.424660
H 5.285852 -0.694765 -1.525826
H 4.055623 -0.149164 2.113165
H 5.744459 -0.181231 1.615434
H 5.749552 -3.804397 1.023038
H 6.068839 -2.569072 -0.216251
H 6.758226 -2.403663 1.429524
H 5.777509 2.546898 -1.491608
H 4.697465 3.888691 -0.986067
H 6.359468 3.796685 -0.378270

H -4.335356 2.836796 -0.685135
H -5.935147 2.604979 -0.030386
H -4.580151 2.857595 1.078599
H -5.283076 -1.086229 0.404286
H -7.206080 1.238361 0.980000
H -8.938572 0.227882 -0.366062
H -7.567534 0.650656 -1.377389
H -8.265118 -2.177394 -0.422225
H -8.495803 -1.513716 -2.041428
H -6.859315 -1.761286 -1.425340
H -8.576740 -0.512306 2.045356
H -6.967369 -0.384321 2.767947
H -7.338386 -1.727837 1.682824
SCF Energy (B3LYP/6-31G**)= -1361.69945442
Number of imaginary frequencies = 0

1b_c165
MMFF Geometry
C -1.687446 -1.304977 -0.796812
C -2.108066 1.183545 0.206018
N -2.965675 -0.910013 -0.581389
C -0.558080 -0.523790 -0.541560
C -0.787666 0.763095 -0.021972
C -3.172476 0.330042 -0.081391
C 0.308256 1.602141 0.252674
C 1.623633 1.175041 0.027358
C 1.844289 -0.110757 -0.476061
C 0.764289 -0.952408 -0.776236
C 3.238036 -0.537353 -0.721334
C 4.388898 0.220045 -0.026938
C 4.011130 1.668789 0.227296
C 2.738067 2.086567 0.286336
O 0.947693 -2.206525 -1.306759
O 3.462327 -1.485103 -1.481204
O 5.498661 0.171381 -0.940916
C 4.816953 -0.430785 1.290896
C 4.776674 -1.939018 1.239506
C 6.009637 -2.658686 0.763183
O 3.749669 -2.547286 1.542670
O 5.014255 2.545900 0.616723
C 5.770730 3.030495 -0.492626
C -4.593415 0.733277 0.128698
C -4.898123 2.206358 0.083576
C -5.532686 -0.220419 0.315864
C -7.013865 -0.035829 0.567395
C -7.800888 -0.476350 -0.679818
C -9.297291 -0.215340 -0.575822
C -7.413742 -0.825805 1.817743
H -1.591571 -2.311809 -1.197692
H -2.293051 2.169777 0.618750
H 0.127993 2.602154 0.645952
H 2.515240 3.114806 0.557379
H 1.905270 -2.343047 -1.480573
H 5.332537 -0.580927 -1.545584
H 4.153595 -0.134348 2.114535
H 5.830491 -0.124705 1.577919
H 5.881046 -3.735612 0.903840
H 6.173434 -2.456858 -0.297127
H 6.874475 -2.334847 1.347455
H 6.611148 2.362681 -0.700008
H 5.155294 3.165520 -1.389212
H 6.182740 4.005232 -0.214342
H -4.346942 2.695854 -0.727643
H -5.953140 2.416665 -0.110458
H -4.627709 2.684844 1.030559
H -5.216606 -1.265901 0.299245
H -7.241094 1.015364 0.771758
H -7.421876 0.064387 -1.556880
H -7.638771 -1.543937 -0.876068
H -9.498120 0.836427 -0.349096
H -9.786366 -0.456070 -1.525315
H -9.760192 -0.833019 0.199145
H -8.450535 -0.619119 2.101245
H -6.784089 -0.551871 2.672126
H -7.315403 -1.905753 1.659834
SCF Energy (B3LYP/6-31G**)= -1361.69955332
Number of imaginary frequencies = 0

1b_c166
MMFF Geometry
C -1.666865 -1.502812 -0.426111
C -2.122803 1.008334 0.502175
N -2.942841 -1.149931 -0.136891
C -0.556929 -0.666879 -0.283903
C -0.804979 0.632013 0.195829
C -3.166853 0.100852 0.327662
C 0.269580 1.527382 0.351020
C 1.583001 1.144133 0.048652
C 1.823595 -0.154000 -0.412214
C 0.762933 -1.051923 -0.595626
C 3.213751 -0.534578 -0.739898
C 4.376683 0.301221 -0.165995
C 3.954514 1.743798 0.050302
C 2.671757 2.111701 0.184142
O 0.962838 -2.320636 -1.083808
O 3.424858 -1.507430 -1.471285
O 5.419712 0.254445 -1.155522
C 4.925820 -0.268052 1.144683
C 4.947136 -1.777404 1.164279
C 6.172457 -2.469483 0.630789
O 3.971891 -2.410752 1.569754
O 4.943579 2.678395 0.325006
C 5.591317 3.144398 -0.858670
C -4.581721 0.445998 0.651471
C -4.801683 1.509541 1.693253
C -5.583744 -0.216644 0.032092
C -7.075951 -0.016766 0.189609
C -7.708061 0.213406 -1.197247
C -9.190204 0.557610 -1.138442
C -7.671556 -1.230665 0.905715
H -1.555975 -2.521764 -0.790873
H -2.324275 2.012205 0.861185
H 0.073946 2.536766 0.711657
H 2.423698 3.141798 0.424463
H 1.910780 -2.426209 -1.320595
H 5.244175 -0.531687 -1.712613
H 4.311000 0.040886 2.000756
H 5.943135 0.091581 1.342320
H 6.100880 -3.542707 0.828666
H 6.250595 -2.312245 -0.446705
H 7.062366 -2.083963 1.134404
H 6.440699 2.501742 -1.105257
H 4.903435 3.216980 -1.708615
H 5.981972 4.145455 -0.652325
H -4.664906 2.506172 1.260945
H -5.799268 1.469935 2.138115
H -4.102502 1.390045 2.528946
H -5.319110 -0.994809 -0.687408
H -7.284463 0.870094 0.796118
H -7.186537 1.038390 -1.699989
H -7.574841 -0.673465 -1.829921
H -9.368015 1.421703 -0.490639
H -9.557309 0.804520 -2.139921
H -9.783717 -0.283096 -0.767917
H -8.730637 -1.075694 1.134078
H -7.159674 -1.414269 1.857402
H -7.585753 -2.138108 0.297384
SCF Energy (B3LYP/6-31G**)= -1361.69961537
Number of imaginary frequencies = 0

1b_c167
MMFF Geometry
C 1.744090 2.551736 -0.058816
C 2.458284 -0.058836 0.190825
N 3.053831 2.267399 0.139134
C 0.723754 1.599244 -0.144288
C 1.105533 0.250789 -0.012427
C 3.399297 0.966942 0.253721
C 0.126253 -0.756113 -0.091195
C -1.224227 -0.439547 -0.288087
C -1.600146 0.901991 -0.405847
C -0.634148 1.917220 -0.350264

C -3.033805 1.220956 -0.596870
C -4.093747 0.148977 -0.271161
C -3.523933 -1.241352 -0.455895
C -2.210433 -1.510008 -0.418210
O -0.968981 3.243033 -0.488523
O -3.356396 2.354085 -0.968638
O -5.162187 0.363912 -1.206561
C -4.599063 0.443565 1.144779
C -5.493187 -0.627526 1.729379
C -6.869650 -0.801980 1.141705
O -5.115834 -1.305534 2.687262
O -4.440657 -2.276344 -0.553733
C -4.611136 -2.695760 -1.908009
C 4.840860 0.701661 0.473710
C 5.507990 1.649580 1.434006
C 5.462116 -0.307606 -0.173448
C 6.927723 -0.677831 -0.094495
C 7.157060 -1.915479 0.794473
C 6.503940 -3.204135 0.309438
C 7.499757 -0.861854 -1.503690
H 1.526045 3.613766 -0.149567
H 2.773044 -1.090755 0.313195
H 0.425916 -1.799808 0.001329
H -1.858824 -2.535620 -0.486058
H -1.914448 3.306897 -0.751226
H -5.203594 1.328776 -1.365073
H -5.162542 1.385113 1.172076
H -3.754320 0.570081 1.835285
H -7.457478 -1.463664 1.784524
H -7.373675 0.166574 1.093205
H -6.809933 -1.245096 0.146741
H -4.920946 -1.865415 -2.550369
H -3.692270 -3.149398 -2.294179
H -5.399035 -3.454339 -1.927436
H 4.864104 1.844075 2.299778
H 6.442818 1.251929 1.838115
H 5.726932 2.603971 0.943756
H 4.884315 -0.921701 -0.864075
H 7.495191 0.145876 0.353298
H 8.236541 -2.089286 0.887669
H 6.792515 -1.701815 1.807557
H 6.889563 -3.506906 -0.668240
H 6.717454 -4.015508 1.013218
H 5.416723 -3.104996 0.240044
H 8.556110 -1.147643 -1.458768
H 7.432317 0.072645 -2.072481
H 6.964027 -1.630563 -2.070723
SCF Energy (B3LYP/6-31G**)= -1361.70054484
Number of imaginary frequencies = 0

1b_c168
MMFF Geometry
C -1.688920 -2.095763 -0.076829
C -2.177875 0.547583 0.322881
N -2.964099 -1.720860 0.187946
C -0.598403 -1.222777 -0.161534
C -0.864093 0.142282 0.047587
C -3.192314 -0.405124 0.381958
C 0.190953 1.069325 -0.025126
C 1.505876 0.661301 -0.299290
C 1.766951 -0.697311 -0.500912
C 0.721258 -1.634604 -0.439730
C 3.153115 -1.127270 -0.794593
C 4.293605 -0.096693 -0.750679
C 3.851426 1.343255 -0.576275
C 2.563686 1.673316 -0.372354
O 0.934955 -2.977377 -0.650293
O 3.365862 -2.304230 -1.109558
O 4.904714 -0.197405 -2.059303
C 5.354559 -0.527711 0.270098
C 4.910104 -0.324561 1.700105
C 5.984698 -0.002711 2.704045
O 3.737508 -0.467447 2.045064
O 4.903450 2.243212 -0.623522
C 4.608180 3.634443 -0.606422
C -4.594158 -0.023303 0.664367
C -5.249321 -0.795502 1.772713
C -5.189194 0.905485 -0.114953
C -6.584388 1.499241 -0.087978
C -7.439765 0.978213 -1.261493
C -7.762358 -0.510074 -1.215839
C -7.345135 1.453427 1.238666
H -1.558998 -3.165608 -0.225800
H -2.410058 1.593502 0.498790
H -0.021478 2.125352 0.137777
H 2.253260 2.702774 -0.234677
H 1.884795 -3.111419 -0.867398
H 5.015793 -1.150898 -2.238561
H 6.278734 0.040478 0.103660
H 5.624105 -1.584017 0.149493
H 5.538845 0.119614 3.695014
H 6.709485 -0.819671 2.738550
H 6.479350 0.930828 2.424520
H 3.963530 3.909910 -1.447347
H 4.156772 3.925252 0.347436
H 5.550964 4.179375 -0.711631
H -4.620650 -1.608400 2.152504
H -5.457633 -0.141337 2.624987
H -6.179915 -1.257784 1.429968
H -4.597151 1.319580 -0.934886
H -6.430042 2.571669 -0.280863
H -8.382313 1.539117 -1.294767
H -6.924640 1.191377 -2.207222
H -8.336328 -0.770937 -0.322362
H -8.365610 -0.785609 -2.087237
H -6.854694 -1.120209 -1.236949
H -8.248126 2.071661 1.176311
H -6.734543 1.854707 2.054589
H -7.667595 0.445897 1.510886
SCF Energy (B3LYP/6-31G**)= -1361.70078144
Number of imaginary frequencies = 0

1b_c169
MMFF Geometry
C 1.844955 -1.466036 -0.567041
C 2.278991 1.201172 -0.296284
N 3.125569 -1.023275 -0.568412
C 0.719674 -0.649233 -0.436173
C 0.955836 0.730353 -0.297004
C 3.339336 0.305902 -0.432172
C -0.137160 1.607899 -0.168655
C -1.454913 1.130330 -0.161686
C -1.680119 -0.242032 -0.284193
C -0.605540 -1.129067 -0.440000
C -3.078068 -0.721755 -0.275310
C -4.145603 0.169260 0.405267
C -3.830594 1.646962 0.153328
C -2.575598 2.070963 -0.091581
O -0.801213 -2.479814 -0.602561
O -3.313216 -1.825139 -0.772934
O -4.077759 -0.001925 1.824415
C -5.543268 -0.206406 -0.128762
C -5.941372 -1.621223 0.243689
C -6.813636 -2.366092 -0.729883
O -5.611168 -2.115655 1.324063
O -4.935547 2.476417 0.249526
C -4.735179 3.884767 0.229735
C 4.762833 0.755269 -0.452373
C 5.021668 2.163863 -0.914653
C 5.732624 -0.110630 -0.081828
C 7.226026 0.119301 -0.011623
C 7.751613 0.041628 1.436971
C 7.630036 -1.316308 2.117760
C 7.946781 -0.856486 -0.944968
H 1.743639 -2.543441 -0.678604
H 2.469918 2.262336 -0.175714
H 0.047024 2.677237 -0.072724
H -2.337337 3.117585 -0.241654
H -1.765054 -2.646045 -0.707829
H -4.452938 -0.887462 2.024225
H -5.570369 -0.106300 -1.220468
H -6.325357 0.434012 0.296351

H -7.015256 -3.371221 -0.349459
H -7.760477 -1.834809 -0.852628
H -6.300508 -2.451577 -1.690914
H -4.077827 4.199663 1.046641
H -4.342386 4.209031 -0.739134
H -5.707569 4.364450 0.375463
H 4.793577 2.878407 -0.116853
H 6.055063 2.331766 -1.225982
H 4.407633 2.406759 -1.789619
H 5.429055 -1.116201 0.216235
H 7.471885 1.125824 -0.363600
H 8.809868 0.332625 1.441836
H 7.226687 0.786189 2.049367
H 8.194814 -2.086004 1.584107
H 8.033580 -1.257918 3.134185
H 6.588014 -1.638851 2.196149
H 9.032222 -0.725474 -0.880348
H 7.652763 -0.682324 -1.986401
H 7.715646 -1.900815 -0.709197
SCF Energy (B3LYP/6-31G**)= -1361.71211958
Number of imaginary frequencies = 0

1b_c170
MMFF Geometry
C 1.728536 -1.498701 0.204941
C 2.100423 1.112122 -0.444510
N 2.985912 -1.084410 -0.084129
C 0.597825 -0.678872 0.195859
C 0.802013 0.671823 -0.140232
C 3.168217 0.216143 -0.409186
C -0.295544 1.553193 -0.155507
C -1.590158 1.106536 0.142428
C -1.784913 -0.240832 0.458485
C -0.701571 -1.128363 0.505136
C -3.154492 -0.686700 0.777925
C -4.340931 0.166287 0.288797
C -3.982911 1.647301 0.249147
C -2.703524 2.061338 0.171876
O -0.860081 -2.447679 0.853574
O -3.325186 -1.726112 1.421482
O -5.407197 -0.025034 1.236674
C -4.846456 -0.267195 -1.089316
C -4.842685 -1.766835 -1.262204
C -6.046842 -2.533899 -0.785968
O -3.861047 -2.336500 -1.740979
O -5.087498 2.480202 0.249569
C -4.884156 3.886430 0.302176
C 4.564229 0.629430 -0.738005
C 4.721514 1.804044 -1.665531
C 5.598172 -0.068104 -0.216618
C 7.081235 0.168003 -0.397720
C 7.765755 0.564400 0.927075
C 7.768832 -0.498229 2.019346
C 7.728433 -1.060218 -1.042118
H 1.651168 -2.554877 0.454312
H 2.269074 2.154769 -0.692546
H -0.131120 2.601676 -0.401599
H -2.440927 3.112020 0.116707
H -1.795131 -2.599124 1.115829
H -5.159275 -0.789190 1.794688
H -4.214140 0.138620 -1.890323
H -5.864502 0.095505 -1.277282
H -5.979244 -3.569478 -1.131124
H -6.087640 -2.524985 0.304937
H -6.955373 -2.091266 -1.201725
H -4.329368 4.169307 1.202601
H -4.375497 4.240769 -0.599994
H -5.865874 4.367227 0.345629
H 4.563217 2.743766 -1.126162
H 5.706219 1.851678 -2.135884
H 4.003279 1.748409 -2.491761
H 5.364111 -0.918266 0.427254
H 7.251154 1.004965 -1.081929
H 8.806981 0.839086 0.714924
H 7.285228 1.468632 1.322555
H 8.298562 -1.400689 1.701311
H 8.279694 -0.113363 2.908320
H 6.754088 -0.775766 2.318083
H 8.809154 -0.918397 -1.149104
H 7.315648 -1.233291 -2.042626
H 7.561194 -1.970485 -0.456386
SCF Energy (B3LYP/6-31G**)= -1361.70422663
Number of imaginary frequencies = 0

1b_c171
MMFF Geometry
C -1.854787 -1.411536 -0.639780
C -2.035606 1.187450 0.121635
N -3.088549 -0.891984 -0.428611
C -0.656862 -0.706765 -0.498075
C -0.762995 0.637369 -0.104206
C -3.175818 0.403191 -0.047516
C 0.407152 1.401119 0.054430
C 1.677590 0.849123 -0.172064
C 1.781553 -0.492537 -0.556737
C 0.618865 -1.265012 -0.724888
C 3.124591 -1.080329 -0.802197
C 4.385831 -0.219935 -0.552669
C 4.094319 1.218576 -0.188192
C 2.856997 1.694834 0.004277
O 0.670660 -2.581780 -1.120764
O 3.186985 -2.230562 -1.247450
O 5.124852 -0.201626 -1.793201
C 5.293640 -0.924985 0.463031
C 4.730220 -0.942937 1.866238
C 5.723958 -1.094581 2.987470
O 3.521551 -0.892044 2.091752
O 5.201780 2.012500 0.073733
C 5.459098 2.929474 -0.989746
C -4.550870 0.942415 0.164023
C -4.735129 2.427377 0.002368
C -5.561123 0.092114 0.452860
C -7.010098 0.429932 0.724597
C -7.902183 0.157693 -0.501985
C -7.971524 -1.295623 -0.955261
C -7.497585 -0.313676 1.972339
H -1.855265 -2.457232 -0.939815
H -2.123302 2.220476 0.441289
H 0.321988 2.442453 0.363552
H 2.711443 2.719955 0.332118
H 1.614074 -2.833474 -1.246349
H 4.504005 0.051917 -2.497196
H 6.273954 -0.431652 0.487475
H 5.483676 -1.964053 0.166608
H 5.200179 -1.092291 3.947395
H 6.258766 -2.040966 2.876585
H 6.427101 -0.258213 2.969840
H 5.610668 2.406362 -1.939194
H 4.647742 3.658686 -1.084271
H 6.377655 3.472768 -0.749518
H -4.183218 2.797919 -0.869123
H -5.777529 2.710048 -0.167712
H -4.383843 2.958545 0.893054
H -5.333144 -0.973797 0.517751
H -7.103831 1.496569 0.958511
H -8.921310 0.499599 -0.281405
H -7.550134 0.767287 -1.344178
H -8.391209 -1.939690 -0.177262
H -8.617643 -1.378897 -1.835496
H -6.985501 -1.680163 -1.231915
H -8.551244 -0.089405 2.170068
H -6.921838 -0.007729 2.853416
H -7.394433 -1.399542 1.873774
SCF Energy (B3LYP/6-31G**)= -1361.70025512
Number of imaginary frequencies = 0

1b_c172
MMFF Geometry
C -1.709796 -2.050536 -0.272953
C -2.211305 0.534568 0.399807
N -2.990266 -1.706410 0.007668
C -0.619720 -1.173421 -0.244441

C -0.892225 0.161574 0.105325
C -3.224569 -0.419197 0.338260
C 0.161250 1.091719 0.150996
C 1.479999 0.714924 -0.143964
C 1.749998 -0.615010 -0.484617
C 0.705577 -1.553447 -0.541969
C 3.143865 -1.010725 -0.802111
C 4.285545 0.011060 -0.641004
C 3.816245 1.416929 -0.338410
C 2.537266 1.722882 -0.085756
O 0.925395 -2.865834 -0.891510
O 3.364744 -2.146229 -1.240195
O 4.921060 0.018956 -1.939778
C 5.320972 -0.501792 0.365773
C 4.825755 -0.419464 1.790897
C 5.802760 0.066259 2.827302
O 3.688101 -0.774233 2.098514
O 4.781262 2.397277 -0.140198
C 5.369750 2.831386 -1.366535
C -4.631947 -0.070374 0.635587
C -5.304817 -0.958069 1.642165
C -5.215100 0.937453 -0.048722
C -6.611603 1.524921 0.018144
C -7.445511 1.134365 -1.219735
C -7.766188 -0.350058 -1.341416
C -7.395223 1.335521 1.318484
H -1.575123 -3.098288 -0.533139
H -2.448432 1.555492 0.683320
H -0.053947 2.124513 0.423194
H 2.269177 2.737229 0.195594
H 1.880684 -2.978721 -1.095515
H 5.007302 -0.918347 -2.205924
H 6.247399 0.079319 0.274070
H 5.600811 -1.543271 0.166131
H 5.321371 0.081858 3.809003
H 6.662330 -0.607350 2.863619
H 6.126373 1.080088 2.578876
H 6.183052 2.160365 -1.656778
H 4.630114 2.917628 -2.170299
H 5.801800 3.822045 -1.195875
H -5.529120 -0.400070 2.556507
H -6.228458 -1.380465 1.235156
H -4.681354 -1.807355 1.942622
H -4.609541 1.438265 -0.808172
H -6.455861 2.611932 -0.054542
H -8.388338 1.695673 -1.208449
H -6.914398 1.448804 -2.127614
H -8.355158 -0.706233 -0.491683
H -8.353686 -0.529379 -2.247980
H -6.857205 -0.954405 -1.412685
H -8.298129 1.956922 1.307764
H -6.799654 1.645900 2.183667
H -7.720513 0.304426 1.474145
SCF Energy (B3LYP/6-31G**)= -1361.69506926
Number of imaginary frequencies = 0

1b_c173
MMFF Geometry
C -1.785337 -1.775181 -0.301277
C -2.086765 0.917652 -0.114325
N -3.041508 -1.283216 -0.172797
C -0.623475 -0.998822 -0.347223
C -0.792176 0.393219 -0.251991
C -3.182983 0.057994 -0.076607
C 0.338492 1.228581 -0.300125
C 1.632934 0.702077 -0.434937
C 1.796376 -0.683653 -0.520704
C 0.675383 -1.530621 -0.484852
C 3.161152 -1.239168 -0.666467
C 4.375606 -0.298434 -0.597121
C 4.041076 1.180127 -0.560202
C 2.772837 1.622013 -0.488474
O 0.793093 -2.897629 -0.586824
O 3.300375 -2.449234 -0.881030
O 5.077560 -0.543746 -1.839392
C 5.313765 -0.731375 0.536821
C 4.776532 -0.388795 1.907180
C 5.792207 -0.084244 2.975805
O 3.572287 -0.409326 2.159668
O 5.164015 1.990974 -0.580999
C 4.983786 3.397867 -0.686550
C -4.571816 0.570541 0.081121
C -4.743288 1.734348 1.014210
C -5.572355 -0.059056 -0.572960
C -7.056973 0.225134 -0.676700
C -7.891124 -0.737980 0.190967
C -7.687115 -0.601347 1.694035
C -7.501203 1.682510 -0.527416
H -1.734569 -2.859745 -0.369313
H -2.235177 1.991002 -0.051939
H 0.202156 2.307036 -0.228188
H 2.538379 2.679618 -0.448849
H 1.742833 -3.120117 -0.712390
H 5.123127 -1.513708 -1.942929
H 6.291618 -0.249157 0.410668
H 5.504363 -1.811205 0.511683
H 5.281604 0.146234 3.914919
H 6.437188 -0.953417 3.125694
H 6.387790 0.782544 2.678710
H 4.431892 3.656421 -1.596042
H 4.484449 3.794538 0.203111
H 5.973543 3.859629 -0.749949
H -4.699793 2.680724 0.466213
H -5.686145 1.679563 1.562444
H -3.966601 1.749542 1.787690
H -5.293763 -0.930093 -1.173756
H -7.305630 -0.024868 -1.719677
H -8.955278 -0.589481 -0.031818
H -7.660714 -1.772422 -0.095485
H -7.962334 0.395427 2.049467
H -8.318008 -1.324826 2.221071
H -6.649982 -0.799597 1.979543
H -8.528382 1.798645 -0.892440
H -6.867852 2.350795 -1.120716
H -7.496016 2.030791 0.507236
SCF Energy (B3LYP/6-31G**)= -1361.70101887
Number of imaginary frequencies = 0

1b_c174
MMFF Geometry
C -1.732904 -1.357058 -0.677483
C -2.126329 1.206832 0.129355
N -3.002497 -0.950738 -0.433645
C -0.599672 -0.551801 -0.545197
C -0.814804 0.774674 -0.125531
C -3.195650 0.326567 -0.031527
C 0.284769 1.641424 0.019629
C 1.589444 1.199004 -0.227923
C 1.796032 -0.125582 -0.629013
C 0.712356 -0.993668 -0.809020
C 3.187869 -0.565008 -0.878925
C 4.322242 0.208309 -0.157025
C 3.978070 1.694045 -0.136207
C 2.711825 2.135895 -0.162936
O 0.884493 -2.283696 -1.247742
O 3.382218 -1.537049 -1.609820
O 5.552104 0.024867 -0.863576
C 4.521057 -0.335423 1.262639
C 4.423180 -1.843429 1.321441
C 5.611155 -2.648917 0.867113
O 3.383757 -2.382821 1.703631
O 4.997697 2.628166 -0.254734
C 5.901546 2.635055 0.846924
C -4.608683 0.739672 0.211221
C -4.927796 2.201190 0.048220
C -5.526617 -0.199517 0.530883
C -6.996540 -0.010328 0.832515
C -7.883488 -0.693711 -0.224615
C -7.749194 -0.077413 -1.611523
C -7.301564 -0.582743 2.221018
H -1.647550 -2.393966 -0.995647
H -2.300371 2.224140 0.463855

H 0.115452 2.673831 0.323282
H 2.499684 3.201137 -0.189407
H 1.831129 -2.417998 -1.477740
H 5.433385 0.432013 -1.741451
H 3.759779 0.058069 1.948942
H 5.495333 -0.056341 1.679114
H 5.486107 -3.689365 1.180157
H 5.694930 -2.612396 -0.220729
H 6.521486 -2.258896 1.329146
H 5.368138 2.608056 1.803211
H 6.608937 1.804576 0.774747
H 6.474583 3.566042 0.804138
H -4.428259 2.614308 -0.835688
H -5.993344 2.392047 -0.100888
H -4.606293 2.766089 0.929379
H -5.197130 -1.238822 0.599147
H -7.259344 1.051458 0.866807
H -7.650479 -1.764411 -0.284910
H -8.933959 -0.618099 0.083195
H -6.742592 -0.214567 -2.017817
H -8.453518 -0.551993 -2.302328
H -7.970013 0.994422 -1.587127
H -8.353086 -0.422391 2.482971
H -6.690091 -0.095790 2.989044
H -7.105411 -1.660125 2.265847
SCF Energy (B3LYP/6-31G**)= -1361.69490456
Number of imaginary frequencies = 0

1b_c175
MMFF Geometry
C 1.799935 1.731148 -0.364301
C 2.118146 -0.948295 -0.052592
N 3.060570 1.251276 -0.233611
C 0.641333 0.948716 -0.353501
C 0.818947 -0.436715 -0.194093
C 3.210425 -0.083138 -0.074782
C -0.307417 -1.278709 -0.183037
C -1.605008 -0.763944 -0.323124
C -1.779315 0.616221 -0.471785
C -0.662362 1.468399 -0.494817
C -3.151239 1.158886 -0.625127
C -4.364904 0.217766 -0.503837
C -4.001879 -1.248258 -0.418335
C -2.740592 -1.684503 -0.308338
O -0.788319 2.828338 -0.660596
O -3.300528 2.356157 -0.897743
O -5.085713 0.431097 -1.739052
C -5.280093 0.673405 0.637926
C -4.698570 0.365937 1.998236
C -5.644614 -0.162639 3.042395
O -3.513997 0.578977 2.255386
O -5.033799 -2.167234 -0.269101
C -5.740688 -2.388213 -1.489825
C 4.604294 -0.581463 0.084582
C 4.796533 -1.699127 1.068704
C 5.591135 0.020871 -0.614463
C 7.075337 -0.261512 -0.728505
C 7.918265 0.744919 0.079459
C 7.738783 0.678290 1.590367
C 7.529228 -1.708341 -0.518160
H 1.742683 2.810979 -0.483248
H 2.272705 -2.016780 0.059413
H -0.166679 -2.352131 -0.060580
H -2.544491 -2.745020 -0.178948
H -1.741696 3.042401 -0.770885
H -5.106829 1.399137 -1.877814
H -6.258025 0.183682 0.547396
H -5.480184 1.750802 0.592968
H -5.101091 -0.345794 3.973391
H -6.431263 0.572460 3.228784
H -6.079976 -1.104369 2.699026
H -6.509520 -1.622888 -1.628190
H -5.068183 -2.425673 -2.354041
H -6.245806 -3.355628 -1.411331
H 4.032732 -1.680715 1.854843
H 4.748559 -2.670553 0.566857
H 5.747962 -1.614747 1.598035
H 5.298619 0.861290 -1.251135
H 7.306193 -0.059766 -1.785927
H 8.979490 0.590661 -0.153164
H 7.678143 1.763741 -0.251533
H 8.024602 -0.299471 1.987768
H 8.374305 1.428454 2.072633
H 6.705321 0.885324 1.882866
H 8.550993 -1.837127 -0.893974
H 6.889819 -2.406508 -1.069033
H 7.542407 -2.007575 0.531657
SCF Energy (B3LYP/6-31G**)= -1361.69535395
Number of imaginary frequencies = 0

1b_c176
MMFF Geometry
C -1.699073 -1.553449 -0.393429
C -2.156390 0.988614 0.447977
N -2.967760 -1.205914 -0.067673
C -0.597573 -0.697292 -0.330524
C -0.845986 0.618022 0.105589
C -3.192303 0.060122 0.353158
C 0.218759 1.535926 0.177309
C 1.523427 1.154607 -0.156799
C 1.764902 -0.159814 -0.571616
C 0.713431 -1.077885 -0.680820
C 3.156204 -0.534113 -0.913638
C 4.298422 0.293550 -0.268414
C 3.886941 1.761536 -0.224067
C 2.602342 2.143345 -0.166185
O 0.916154 -2.358966 -1.132528
O 3.347099 -1.497956 -1.656213
O 5.485830 0.166069 -1.055339
C 4.616649 -0.236281 1.134663
C 4.592579 -1.746986 1.199805
C 5.783345 -2.498328 0.666827
O 3.607100 -2.332406 1.650945
O 4.851351 2.742186 -0.409790
C 5.825446 2.794253 0.628983
C -4.598553 0.399396 0.717710
C -4.794828 1.503548 1.721009
C -5.613256 -0.300847 0.164050
C -7.100004 -0.113506 0.368001
C -7.835412 0.058930 -0.975602
C -7.427184 1.322087 -1.723914
C -7.661256 -1.320993 1.122773
H -1.586927 -2.585152 -0.720093
H -2.359577 2.003954 0.771937
H 0.021649 2.560042 0.492068
H 2.339005 3.197371 -0.178124
H 1.850906 -2.449357 -1.424300
H 5.290000 0.564858 -1.923184
H 3.885647 0.123780 1.870457
H 5.602681 0.088621 1.485004
H 5.727110 -3.542718 0.986598
H 5.792918 -2.460428 -0.424145
H 6.703545 -2.066060 1.067662
H 5.358957 2.744688 1.618766
H 6.564669 1.997695 0.509196
H 6.349978 3.750986 0.548561
H -4.689357 2.482334 1.241669
H -5.774195 1.470276 2.204969
H -4.062852 1.429397 2.533534
H -5.365046 -1.102766 -0.534844
H -7.306289 0.778011 0.968427
H -7.663238 -0.811523 -1.621306
H -8.916138 0.103873 -0.791233
H -6.375447 1.290336 -2.024123
H -8.029016 1.430998 -2.631884
H -7.583824 2.211687 -1.105572
H -8.733345 -1.196598 1.310022
H -7.166777 -1.441446 2.093241
H -7.524759 -2.249521 0.556723
SCF Energy (B3LYP/6-31G**)= -1361.69505382
Number of imaginary frequencies = 0

1b_c177
MMFF Geometry
C -1.781969 -1.239474 -0.688474
C -2.134699 1.296440 0.217055
N -3.048277 -0.806994 -0.474852
C -0.632428 -0.474629 -0.477946
C -0.826877 0.837081 -0.008566
C -3.221413 0.456614 -0.023261
C 0.290800 1.661322 0.219279
C 1.593462 1.195575 -0.003339
C 1.779181 -0.114216 -0.456485
C 0.677063 -0.942449 -0.709936
C 3.159949 -0.582058 -0.699952
C 4.335846 0.176352 -0.050027
C 3.993656 1.642426 0.149312
C 2.731055 2.091172 0.205709
O 0.825900 -2.220836 -1.191054
O 3.353708 -1.564624 -1.423112
O 5.432973 0.065486 -0.973781
C 4.765369 -0.430267 1.288264
C 4.690777 -1.938032 1.298756
C 5.901425 -2.704318 0.838166
O 3.654231 -2.509950 1.638050
O 5.020798 2.511461 0.491355
C 5.774340 2.933378 -0.645264
C -4.630454 0.900032 0.185939
C -4.902429 2.377602 0.096319
C -5.591671 -0.023769 0.408707
C -7.063507 0.211365 0.665524
C -7.914097 -0.050054 -0.592487
C -7.885638 -1.479367 -1.120356
C -7.527640 -0.624201 1.862956
H -1.713572 -2.263592 -1.049174
H -2.292252 2.302632 0.591123
H 0.137749 2.680269 0.573552
H 2.534575 3.134341 0.437193
H 1.777993 -2.386088 -1.369984
H 5.242707 -0.706671 -1.545410
H 4.118940 -0.085713 2.106624
H 5.788932 -0.136040 1.551086
H 5.750536 -3.771462 1.023975
H 6.056689 -2.549307 -0.231266
H 6.780375 -2.376947 1.398938
H 6.597068 2.238739 -0.834694
H 5.151208 3.045972 -1.539628
H 6.211347 3.908944 -0.411593
H -4.356302 2.826270 -0.741444
H -5.956530 2.605156 -0.084077
H -4.603173 2.881354 1.021270
H -5.299963 -1.075932 0.423556
H -7.226892 1.256810 0.951153
H -8.956232 0.217821 -0.377145
H -7.584427 0.622629 -1.394764
H -8.279455 -2.187162 -0.385390
H -8.508883 -1.555589 -2.017561
H -6.872883 -1.788812 -1.394617
H -8.596602 -0.474570 2.048752
H -6.988365 -0.330271 2.770794
H -7.356131 -1.695255 1.711669
SCF Energy (B3LYP/6-31G**)= -1361.69874036
Number of imaginary frequencies = 0

1b_c178
MMFF Geometry
C -1.858380 -1.595794 -0.084587
C -2.101702 1.060259 0.419528
N -3.095639 -1.104663 0.169697
C -0.687189 -0.834435 -0.111565
C -0.825991 0.538952 0.148204
C -3.213402 0.218963 0.423732
C 0.315346 1.360144 0.129361
C 1.588949 0.836875 -0.142976
C 1.726587 -0.532391 -0.393785
C 0.592515 -1.363647 -0.381840
C 3.072284 -1.088696 -0.690121
C 4.297113 -0.141488 -0.718136
C 3.973933 1.308997 -0.421849
C 2.738067 1.739003 -0.140914
O 0.676166 -2.712816 -0.638809
O 3.160144 -2.289452 -0.966251
O 4.812962 -0.208113 -2.065004
C 5.392889 -0.710194 0.193368
C 5.033308 -0.671043 1.662811
C 6.178836 -0.768448 2.635561
O 3.869198 -0.613057 2.057247
O 5.026669 2.204796 -0.275691
C 5.691844 2.495659 -1.503777
C -4.588212 0.719151 0.719682
C -4.689449 1.948682 1.581620
C -5.653913 0.047453 0.229207
C -7.123805 0.367492 0.386941
C -7.781507 0.726226 -0.961952
C -7.833612 -0.391973 -1.995955
C -7.835347 -0.790152 1.091373
H -1.832690 -2.666655 -0.274589
H -2.216864 2.121705 0.612248
H 0.205845 2.424609 0.335061
H 2.577490 2.778710 0.130781
H 1.620875 -2.943263 -0.792813
H 4.075311 -0.009717 -2.667739
H 6.326344 -0.152147 0.048228
H 5.618113 -1.751857 -0.067267
H 5.797686 -0.727280 3.659684
H 6.703158 -1.715904 2.489364
H 6.862630 0.070003 2.481803
H 6.416701 1.712399 -1.742516
H 4.982721 2.634727 -2.327141
H 6.244382 3.430548 -1.370550
H -4.480001 2.848567 0.993916
H -5.672852 2.072183 2.040748
H -3.979436 1.900910 2.415413
H -5.460280 -0.846960 -0.366252
H -7.254039 1.247451 1.024294
H -8.808398 1.064007 -0.772182
H -7.254022 1.582578 -1.401689
H -8.409826 -1.248224 -1.634008
H -8.320350 -0.030108 -2.907831
H -6.832927 -0.735924 -2.272190
H -8.907931 -0.588431 1.182976
H -7.436263 -0.929896 2.102593
H -7.711987 -1.737820 0.556261
SCF Energy (B3LYP/6-31G**)= -1361.70021188
Number of imaginary frequencies = 0

1b_c179
MMFF Geometry
C 1.930686 1.671998 -0.302801
C 2.146911 -1.032556 -0.183763
N 3.170132 1.133049 -0.210680
C 0.743222 0.937590 -0.340777
C 0.867142 -0.462136 -0.278631
C 3.275643 -0.214708 -0.151514
C -0.291473 -1.259661 -0.317891
C -1.565872 -0.684019 -0.405613
C -1.683365 0.708258 -0.453230
C -0.538297 1.517482 -0.436973
C -3.038077 1.302047 -0.528571
C -4.260753 0.437350 -0.159709
C -3.982868 -1.026089 -0.430085
C -2.746138 -1.540762 -0.497222
O -0.619216 2.887534 -0.509660
O -3.160160 2.491287 -0.840044
O -5.327456 0.893406 -1.006378
C -4.604819 0.758028 1.298305
C -5.646920 -0.150020 1.913044
C -7.067491 -0.033182 1.424410
O -5.343522 -0.930917 2.817351
O -5.086158 -1.862694 -0.493482
C -5.423729 -2.179204 -1.844371
C 4.657716 -0.769991 -0.065229
C 4.866014 -2.165790 -0.588299
C 5.650346 -0.003849 0.439350

C 7.105193 -0.372011 0.637966
C 7.967414 0.390530 -0.383847
C 9.437947 -0.002549 -0.349429
C 7.508300 -0.065982 2.084072
H 1.918164 2.758916 -0.346771
H 2.249900 -2.111017 -0.122060
H -0.193756 -2.344410 -0.280311
H -2.603997 -2.610327 -0.623350
H -1.549899 3.141970 -0.699523
H -5.192552 1.854726 -1.130807
H -4.974070 1.786951 1.396944
H -3.707531 0.691148 1.928219
H -7.727082 -0.600823 2.087212
H -7.378095 1.014466 1.445565
H -7.160824 -0.432771 0.413718
H -5.610174 -1.276469 -2.434604
H -4.636924 -2.780338 -2.312141
H -6.342252 -2.773104 -1.830116
H 4.317193 -2.319267 -1.524634
H 5.911776 -2.380474 -0.823237
H 4.526238 -2.906047 0.143432
H 5.405756 1.010155 0.763386
H 7.253778 -1.446346 0.489297
H 7.587345 0.195934 -1.395262
H 7.884544 1.472741 -0.221086
H 9.559289 -1.082728 -0.477647
H 9.978288 0.497130 -1.160006
H 9.910732 0.291513 0.592049
H 8.517258 -0.431748 2.299135
H 6.829227 -0.555222 2.791868
H 7.488439 1.010541 2.288024
SCF Energy (B3LYP/6-31G**)= -1361.70259505
Number of imaginary frequencies = 0

1b_c180
MMFF Geometry
C 1.910735 1.731437 0.124527
C 2.154487 -0.973313 0.154848
N 3.147026 1.202743 0.289601
C 0.739780 0.986267 -0.032288
C 0.878204 -0.413439 -0.017365
C 3.265749 -0.145079 0.306407
C -0.262059 -1.221254 -0.182152
C -1.533795 -0.656419 -0.345678
C -1.667169 0.735250 -0.343829
C -0.538516 1.555601 -0.204446
C -3.020319 1.316677 -0.500197
C -4.255310 0.423512 -0.264020
C -3.936362 -1.023663 -0.573858
C -2.690902 -1.521323 -0.565842
O -0.632985 2.926520 -0.227300
O -3.134963 2.516653 -0.770568
O -5.260374 0.903924 -1.170490
C -4.714947 0.676945 1.175265
C -5.786316 -0.269901 1.669262
C -7.167410 -0.149219 1.078967
O -5.540119 -1.085838 2.559917
O -5.019304 -1.868726 -0.759020
C -5.246118 -2.129823 -2.144460
C 4.640354 -0.688712 0.507568
C 4.747874 -2.055101 1.129310
C 5.707958 0.063197 0.158795
C 7.175838 -0.294759 0.253209
C 7.838834 -0.092550 -1.123553
C 9.289878 -0.551788 -1.171038
C 7.830083 0.555661 1.344011
H 1.886450 2.819044 0.122536
H 2.271391 -2.052133 0.155647
H -0.151993 -2.305469 -0.182825
H -2.523999 -2.582725 -0.726008
H -1.549748 3.178458 -0.478358
H -5.129575 1.871028 -1.244099
H -5.106976 1.695928 1.288125
H -3.867567 0.594422 1.869190
H -7.866026 -0.755129 1.663252
H -7.495809 0.892270 1.123038
H -7.177199 -0.503265 0.047253
H -5.398729 -1.204334 -2.708600
H -4.416859 -2.701570 -2.574190
H -6.154489 -2.733615 -2.227544
H 4.556300 -2.833614 0.383596
H 5.729475 -2.246154 1.570437
H 4.029928 -2.169931 1.949605
H 5.524930 1.056455 -0.257226
H 7.299468 -1.347696 0.525014
H 7.278371 -0.655888 -1.881092
H 7.791236 0.963312 -1.419277
H 9.383004 -1.594893 -0.852947
H 9.673856 -0.474441 -2.193421
H 9.927899 0.065235 -0.531863
H 8.866789 0.250048 1.516222
H 7.299077 0.447096 2.296737
H 7.830296 1.618881 1.078596
SCF Energy (B3LYP/6-31G**)= -1361.70265813
Number of imaginary frequencies = 0

1b_c181
MMFF Geometry
C 1.813582 2.410545 0.410268
C 2.424122 -0.000536 -0.684759
N 3.121942 2.103012 0.239438
C 0.745200 1.577022 0.064938
C 1.073625 0.332375 -0.504457
C 3.415361 0.898628 -0.295839
C 0.043865 -0.546351 -0.887179
C -1.302942 -0.210915 -0.696592
C -1.623703 1.019703 -0.115546
C -0.610265 1.917800 0.249478
C -3.051617 1.351970 0.094866
C -4.101800 0.223319 0.050198
C -3.638907 -0.903443 -0.848710
C -2.350659 -1.122788 -1.150693
O -0.894452 3.146726 0.795341
O -3.369600 2.518488 0.348546
O -5.289781 0.820559 -0.492675
C -4.366344 -0.191495 1.501045
C -5.210986 -1.436981 1.655202
C -6.669157 -1.356265 1.284175
O -4.723436 -2.472816 2.112339
O -4.611400 -1.797105 -1.269171
C -5.009115 -1.539379 -2.616185
C 4.858593 0.600994 -0.469039
C 5.673647 1.766643 -0.962658
C 5.344497 -0.618521 -0.151538
C 6.775580 -1.100911 -0.251523
C 7.467659 -1.132712 1.125426
C 6.865326 -2.091730 2.145092
C 6.819611 -2.461108 -0.954812
H 1.638516 3.389332 0.851878
H 2.700366 -0.948103 -1.137313
H 0.300672 -1.503990 -1.339542
H -2.066384 -1.987713 -1.743320
H -1.864803 3.299174 0.748749
H -5.305690 1.746409 -0.175893
H -4.871377 0.611825 2.052748
H -3.419623 -0.377536 2.025928
H -7.181819 -2.259032 1.628807
H -7.125748 -0.493204 1.775234
H -6.787324 -1.279549 0.202498
H -5.370969 -0.513748 -2.739598
H -4.184911 -1.735061 -3.310177
H -5.828450 -2.221807 -2.860288
H 5.718359 2.557093 -0.205451
H 6.703964 1.502937 -1.209133
H 5.230311 2.184124 -1.873957
H 4.657290 -1.361612 0.254077
H 7.358629 -0.420126 -0.879954
H 8.522921 -1.398702 0.984114
H 7.461273 -0.121878 1.553300
H 6.914354 -3.128397 1.799926
H 7.423469 -2.031207 3.085344
H 5.822276 -1.845996 2.364480

H 7.848006 -2.833014 -1.015390
H 6.435230 -2.378937 -1.977987
H 6.216183 -3.214373 -0.437147
SCF Energy (B3LYP/6-31G**)= -1361.70111895
Number of imaginary frequencies = 0

1b_c182
MMFF Geometry
C 1.740666 -1.454500 0.315645
C 2.136288 1.079339 -0.578373
N 3.004538 -1.075085 0.007571
C 0.614443 -0.635265 0.209608
C 0.831231 0.675717 -0.252715
C 3.198638 0.187001 -0.439640
C -0.260691 1.555742 -0.371028
C -1.561070 1.145059 -0.049912
C -1.770565 -0.165599 0.392790
C -0.692137 -1.048229 0.540573
C -3.148583 -0.576239 0.737211
C -4.330624 0.240731 0.178174
C -3.940797 1.696237 0.004112
C -2.668386 2.098117 -0.138738
O -0.861621 -2.327814 1.011190
O -3.332125 -1.558166 1.463053
O -5.385857 0.157843 1.152010
C -4.854329 -0.308873 -1.150676
C -4.861857 -1.817782 -1.192780
C -6.084779 -2.529220 -0.679644
O -3.876773 -2.436058 -1.597986
O -4.979886 2.595466 -0.182593
C -5.261940 3.313990 1.018881
C 4.601641 0.560935 -0.785592
C 4.778882 1.640572 -1.818964
C 5.624034 -0.087660 -0.184340
C 7.110853 0.123792 -0.365074
C 7.778220 0.643191 0.925516
C 7.759404 -0.309191 2.114995
C 7.760530 -1.163484 -0.878392
H 1.654047 -2.481233 0.664868
H 2.313787 2.092269 -0.924244
H -0.088698 2.574511 -0.716886
H -2.441829 3.140279 -0.344210
H -1.802733 -2.453398 1.264931
H -5.189768 -0.620163 1.712814
H -4.228953 0.019623 -1.991611
H -5.871821 0.044573 -1.358184
H -6.008086 -3.596415 -0.906175
H -6.164908 -2.400964 0.401538
H -6.975955 -2.134840 -1.174078
H -5.517609 2.639738 1.842122
H -4.415259 3.948999 1.300684
H -6.123166 3.961050 0.828406
H 4.617099 2.628275 -1.374751
H 5.770849 1.639474 -2.276269
H 4.073048 1.508375 -2.647176
H 5.375933 -0.871104 0.534511
H 7.295382 0.890170 -1.124099
H 8.823953 0.891783 0.703579
H 7.297068 1.583342 1.224775
H 8.288635 -1.240317 1.893384
H 8.259407 0.157442 2.970348
H 6.738869 -0.552383 2.423769
H 8.843462 -1.037086 -0.982320
H 7.361375 -1.430493 -1.863655
H 7.579758 -2.012357 -0.210279
SCF Energy (B3LYP/6-31G**)= -1361.69927498
Number of imaginary frequencies = 0

1b_c183
MMFF Geometry
C -1.742342 -1.951548 -0.349588
C -2.167907 0.728042 -0.435641
N -3.022853 -1.507938 -0.333535
C -0.613420 -1.130163 -0.409684
C -0.845945 0.254055 -0.453774
C -3.230784 -0.172063 -0.375555
C 0.250537 1.132681 -0.515146
C 1.572146 0.657378 -0.533118
C 1.800461 -0.720660 -0.484293
C 0.712731 -1.610723 -0.426566
C 3.194416 -1.227992 -0.500534
C 4.366201 -0.221659 -0.541576
C 3.957587 1.241044 -0.612105
C 2.669420 1.624587 -0.608047
O 0.889555 -2.974923 -0.390193
O 3.367920 -2.451184 -0.503617
O 5.101485 -0.515312 -1.750271
C 5.326928 -0.527729 0.616050
C 4.742958 -0.257555 1.985191
C 5.704097 -0.323663 3.143309
O 3.548558 -0.025029 2.166874
O 5.039751 2.101099 -0.685883
C 4.799441 3.502883 -0.724426
C -4.652689 0.280259 -0.369977
C -4.946503 1.570348 -1.082951
C -5.583459 -0.491102 0.235080
C -7.071202 -0.281917 0.422182
C -7.496432 1.064551 1.039752
C -6.834864 1.349637 2.382232
C -7.840702 -0.572293 -0.868942
H -1.643431 -3.034288 -0.311306
H -2.356397 1.796358 -0.453563
H 0.064977 2.205847 -0.546298
H 2.384735 2.669858 -0.653190
H 1.856041 -3.163696 -0.406645
H 4.606556 -0.128907 -2.492122
H 6.244046 0.065603 0.509967
H 5.638397 -1.579709 0.589105
H 5.173957 -0.109479 4.075458
H 6.136230 -1.325487 3.203493
H 6.492594 0.420772 3.008261
H 4.235482 3.778157 -1.621341
H 4.284771 3.836519 0.182341
H 5.768423 4.008887 -0.767385
H -4.695382 2.427353 -0.449430
H -4.373844 1.643881 -2.014734
H -5.993651 1.662925 -1.375836
H -5.242173 -1.424068 0.691213
H -7.387304 -1.052511 1.141119
H -7.301085 1.898717 0.359375
H -8.583204 1.050982 1.194028
H -5.753533 1.479940 2.279724
H -7.240636 2.271482 2.811375
H -7.020604 0.537091 3.091799
H -8.919722 -0.579486 -0.679212
H -7.570788 -1.553422 -1.275577
H -7.646705 0.176656 -1.642374
SCF Energy (B3LYP/6-31G**)= -1361.70230362
Number of imaginary frequencies = 0

1b_c184
MMFF Geometry
C -1.805188 -1.656002 -0.546892
C -2.101516 0.977580 0.040894
N -3.060104 -1.183078 -0.351377
C -0.641171 -0.886465 -0.471785
C -0.807087 0.474856 -0.169492
C -3.203410 0.129122 -0.055967
C 0.326770 1.302833 -0.086849
C 1.620889 0.798651 -0.296372
C 1.783356 -0.558190 -0.589791
C 0.657325 -1.396277 -0.681261
C 3.148236 -1.096600 -0.808777
C 4.364627 -0.152765 -0.678387
C 4.025313 1.298149 -0.376534
C 2.759266 1.715689 -0.205430
O 0.768375 -2.734439 -0.982298
O 3.261101 -2.288700 -1.112856
O 5.025838 -0.175267 -1.962963
C 5.363599 -0.761385 0.315777
C 4.860116 -0.805606 1.741663
C 5.871310 -1.182280 2.792861

O 3.688838 -0.579618 2.042856
O 5.143443 2.111431 -0.308955
C 4.970455 3.489930 -0.002285
C -4.596928 0.610430 0.174356
C -4.756964 1.757550 1.132385
C -5.618621 -0.019107 -0.447761
C -7.108295 0.250043 -0.421890
C -7.802318 -0.198845 0.877439
C -7.617512 -1.680603 1.181276
C -7.472875 1.694062 -0.780897
H -1.758084 -2.718524 -0.775796
H -2.239755 2.030032 0.265262
H 0.192835 2.358143 0.148672
H 2.524301 2.751050 0.014785
H 1.722488 -2.948499 -1.099977
H 4.515348 0.394872 -2.562099
H 6.302568 -0.193499 0.299950
H 5.621413 -1.786954 0.022271
H 5.397823 -1.178187 3.778569
H 6.255630 -2.184541 2.588452
H 6.688351 -0.456613 2.792288
H 4.377422 3.990579 -0.774260
H 4.517740 3.616805 0.986337
H 5.959785 3.956445 0.017807
H -4.526605 2.707522 0.639065
H -5.765063 1.828736 1.543857
H -4.093673 1.642177 1.997431
H -5.367643 -0.862890 -1.095833
H -7.530448 -0.362887 -1.232211
H -7.446620 0.384847 1.733174
H -8.878124 0.000729 0.793245
H -6.569022 -1.922978 1.379428
H -8.195501 -1.955963 2.069350
H -7.964914 -2.298571 0.347239
H -8.546018 1.771591 -0.989426
H -6.938502 2.025033 -1.678209
H -7.252024 2.397219 0.026315
SCF Energy (B3LYP/6-31G**)= -1361.70237487
Number of imaginary frequencies = 0

1b_c185
MMFF Geometry
C -1.829852 -1.610429 -0.145165
C -2.054361 1.032386 0.431979
N -3.062479 -1.119491 0.131110
C -0.654531 -0.855138 -0.160003
C -0.783569 0.511645 0.137453
C -3.170748 0.197354 0.421689
C 0.362007 1.327289 0.131043
C 1.630894 0.804362 -0.162932
C 1.758860 -0.559688 -0.449012
C 0.619953 -1.384152 -0.453902
C 3.100040 -1.117279 -0.764933
C 4.340148 -0.195077 -0.694927
C 4.019548 1.257223 -0.420773
C 2.783834 1.703265 -0.158324
O 0.693779 -2.725816 -0.750171
O 3.173345 -2.296984 -1.122834
O 4.970757 -0.252485 -1.992777
C 5.359247 -0.781640 0.290057
C 4.917882 -0.702382 1.734238
C 6.008591 -0.722125 2.772324
O 3.731525 -0.677592 2.060281
O 5.110371 2.108945 -0.320488
C 5.237609 2.943758 -1.471560
C -4.540440 0.697002 0.741076
C -4.628466 1.901544 1.638957
C -5.613384 0.046107 0.238527
C -7.080202 0.370265 0.415286
C -7.744754 0.770658 -0.918445
C -7.810817 -0.317757 -1.982981
C -7.794049 -0.802471 1.091854
H -1.811600 -2.675921 -0.364049
H -2.162359 2.088828 0.653988
H 0.259073 2.386721 0.363837
H 2.623005 2.746052 0.099111
H 1.633068 -2.953220 -0.937725
H 4.282682 -0.079852 -2.657697
H 6.316706 -0.254349 0.188894
H 5.566656 -1.834528 0.061937
H 5.568244 -0.658798 3.771268
H 6.573670 -1.653835 2.691336
H 6.669827 0.135496 2.625730
H 5.330813 2.349911 -2.386287
H 4.390715 3.633160 -1.552061
H 6.149072 3.537557 -1.356349
H -4.418562 2.816967 1.075936
H -5.607711 2.016699 2.109035
H -3.912444 1.825978 2.465526
H -5.429085 -0.831926 -0.383683
H -7.200726 1.232775 1.077916
H -8.768216 1.109169 -0.712174
H -7.214935 1.635813 -1.337667
H -8.389894 -1.180329 -1.641174
H -8.301451 0.072482 -2.880962
H -6.814189 -0.659859 -2.275682
H -8.864747 -0.597075 1.196436
H -7.389064 -0.972902 2.095997
H -7.680045 -1.735453 0.529552
SCF Energy (B3LYP/6-31G**)= -1361.69985875
Number of imaginary frequencies = 0

1b_c186
MMFF Geometry
C 2.026806 1.557840 -0.227023
C 2.170375 -1.153876 -0.188915
N 3.250883 0.983289 -0.145536
C 0.820461 0.857005 -0.292691
C 0.906759 -0.546757 -0.272402
C 3.320354 -0.368009 -0.126598
C -0.272460 -1.311411 -0.341230
C -1.530587 -0.699468 -0.417392
C -1.610711 0.696256 -0.423323
C -0.444611 1.473660 -0.377225
C -2.948702 1.328111 -0.486924
C -4.195958 0.485724 -0.150127
C -3.955663 -0.975802 -0.463469
C -2.732726 -1.521145 -0.540430
O -0.488613 2.846956 -0.408741
O -3.037379 2.529051 -0.762767
O -5.245517 0.995530 -0.987512
C -4.539299 0.771334 1.315329
C -5.608513 -0.126657 1.897417
C -7.022799 0.042860 1.405996
O -5.330911 -0.942354 2.779004
O -5.080473 -1.780269 -0.557340
C -5.418979 -2.046682 -1.918762
C 4.686829 -0.962075 -0.050990
C 4.859917 -2.351280 -0.603951
C 5.700512 -0.234104 0.468209
C 7.141949 -0.652018 0.656591
C 8.055105 -0.072864 -0.441204
C 8.145496 1.447703 -0.487013
C 7.617787 -0.276937 2.063845
H 2.043528 2.645530 -0.238144
H 2.243976 -2.236085 -0.159751
H -0.203878 -2.399028 -0.336106
H -2.618426 -2.589836 -0.698277
H -1.411142 3.131842 -0.595121
H -5.084386 1.956210 -1.082115
H -4.881582 1.806320 1.443410
H -3.647548 0.661489 1.947077
H -7.700845 -0.526709 2.048201
H -7.305535 1.097325 1.457516
H -7.121238 -0.323352 0.383215
H -5.578106 -1.121863 -2.482196
H -4.645916 -2.654263 -2.400896
H -6.353051 -2.615937 -1.926799
H 4.317922 -2.466682 -1.549639
H 5.902124 -2.591275 -0.831162
H 4.490264 -3.097742 0.106691
H 5.482269 0.778157 0.814434

H 7.221650 -1.743482 0.596849
H 9.067387 -0.475104 -0.308301
H 7.709893 -0.429465 -1.420232
H 8.559653 1.853818 0.440233
H 8.805628 1.755275 -1.304842
H 7.167574 1.905599 -0.662081
H 8.666074 -0.560268 2.206710
H 7.025783 -0.800136 2.823566
H 7.526834 0.796879 2.259052
SCF Energy (B3LYP/6-31G**)= -1361.70179793
Number of imaginary frequencies = 0

1b_c187
MMFF Geometry
C -1.348505 -2.280373 -0.473703
C -2.295406 0.198625 0.117064
N -2.676905 -2.145797 -0.241398
C -0.420812 -1.235127 -0.433004
C -0.922404 0.044639 -0.123840
C -3.137789 -0.909043 0.043564
C -0.041594 1.140993 -0.072143
C 1.328497 0.975709 -0.305211
C 1.821977 -0.301085 -0.596387
C 0.956512 -1.398581 -0.682204
C 3.276743 -0.444290 -0.833001
C 4.212107 0.618566 -0.199460
C 3.549470 1.988676 -0.298920
C 2.217071 2.138015 -0.340344
O 1.409591 -2.652439 -1.012286
O 3.681234 -1.409655 -1.482093
O 5.453320 0.648683 -0.909323
C 4.523420 0.256984 1.257586
C 4.759167 -1.224901 1.446173
C 6.095782 -1.786975 1.041141
O 3.863093 -1.943582 1.891136
O 4.338838 3.109627 -0.514785
C 5.216517 3.410939 0.566668
C -4.595258 -0.808640 0.295515
C -5.159274 -1.930526 1.125520
C -5.313678 0.210951 -0.221864
C -6.806807 0.436948 -0.102259
C -7.063381 1.676133 0.774173
C -8.538607 1.921731 1.059772
C -7.407516 0.580112 -1.504130
H -1.034127 -3.296714 -0.701995
H -2.702478 1.171890 0.373061
H -0.435010 2.133139 0.146071
H 1.775622 3.124320 -0.453432
H 2.362609 -2.594565 -1.247874
H 5.249518 0.943650 -1.815962
H 3.693280 0.532269 1.921272
H 5.411942 0.777790 1.631547
H 6.201830 -2.798428 1.443487
H 6.171224 -1.827380 -0.047173
H 6.897468 -1.168559 1.452425
H 4.699964 3.351339 1.530704
H 6.089410 2.752752 0.553982
H 5.570829 4.437536 0.434380
H -4.486745 -2.177034 1.955588
H -6.119106 -1.677188 1.582898
H -5.298867 -2.828729 0.514843
H -4.803025 0.958335 -0.829677
H -7.291349 -0.426019 0.365300
H -6.547031 1.553011 1.735180
H -6.638459 2.571626 0.302910
H -9.005868 1.036911 1.503291
H -8.650517 2.752359 1.764198
H -9.085732 2.185210 0.150040
H -8.500359 0.625272 -1.463337
H -7.142517 -0.277526 -2.133167
H -7.050149 1.487354 -2.004212
SCF Energy (B3LYP/6-31G**)= -1361.69283882
Number of imaginary frequencies = 0

1b_c188
MMFF Geometry
C 1.756575 -1.453501 0.277814
C 2.155727 1.101225 -0.552576
N 3.021332 -1.067658 -0.018437
C 0.631110 -0.631025 0.189872
C 0.849772 0.690564 -0.239485
C 3.217216 0.204994 -0.433893
C -0.241565 1.573517 -0.338647
C -1.543066 1.156215 -0.029878
C -1.754603 -0.163714 0.380568
C -0.676625 -1.050628 0.508032
C -3.132400 -0.580682 0.716066
C -4.318639 0.258628 0.197670
C -3.923496 1.715768 0.034429
C -2.649382 2.110254 -0.106097
O -0.847453 -2.341625 0.946274
O -3.314785 -1.586366 1.409802
O -5.342852 0.153526 1.202260
C -4.882066 -0.264990 -1.125914
C -4.879576 -1.772381 -1.208057
C -6.083911 -2.506361 -0.682846
O -3.901742 -2.371988 -1.656337
O -4.932119 2.644297 -0.183964
C -5.565914 3.049986 1.029103
C 4.621189 0.586312 -0.767802
C 4.801171 1.692181 -1.772581
C 5.641987 -0.078586 -0.181825
C 7.129308 0.135964 -0.354570
C 7.794858 0.621932 0.949902
C 7.772963 -0.360161 2.114917
C 7.778730 -1.138548 -0.899125
H 1.668534 -2.488431 0.601510
H 2.334373 2.122305 -0.872971
H -0.068579 2.600095 -0.660211
H -2.422150 3.153413 -0.307523
H -1.789187 -2.472728 1.194783
H -5.144839 -0.651974 1.722960
H -4.287753 0.089374 -1.978865
H -5.908392 0.085446 -1.290762
H -5.998701 -3.569132 -0.926452
H -6.145267 -2.395154 0.401447
H -6.988749 -2.115299 -1.154577
H -6.400394 2.383593 1.263247
H -4.864175 3.098742 1.869366
H -5.976106 4.052083 0.871466
H 4.638829 2.668170 -1.303379
H 5.794138 1.702486 -2.227581
H 4.097114 1.581716 -2.605475
H 5.391956 -0.880014 0.516225
H 7.315896 0.921073 -1.093679
H 8.841232 0.874983 0.736173
H 7.314083 1.554717 1.271950
H 8.301684 -1.285935 1.870808
H 8.271872 0.084214 2.982672
H 6.751633 -0.610029 2.415628
H 8.861954 -1.010581 -0.997957
H 7.381060 -1.380204 -1.891508
H 7.596032 -2.003859 -0.252986
SCF Energy (B3LYP/6-31G**)= -1361.69854815
Number of imaginary frequencies = 0

1b_c189
MMFF Geometry
C 1.721020 -2.058506 -0.029846
C 2.480683 0.512293 -0.476686
N 3.046825 -1.796073 -0.128123
C 0.706170 -1.102554 -0.141789
C 1.111746 0.224117 -0.376013
C 3.413240 -0.513939 -0.340283
C 0.137875 1.229588 -0.510737
C -1.228573 0.936213 -0.400892
C -1.628927 -0.379553 -0.157153
C -0.669174 -1.398141 -0.041498
C -3.077939 -0.677641 -0.059517
C -4.071850 0.482026 0.209030
C -3.503014 1.807408 -0.280674
C -2.211830 1.998608 -0.581758

O -1.025455 -2.709683 0.168401
O -3.428284 -1.857225 -0.156293
O -4.247302 0.622424 1.621089
C -5.404820 0.170796 -0.503658
C -6.117496 -1.026902 0.093046
C -6.886461 -1.909236 -0.852233
O -6.111167 -1.232783 1.308759
O -4.389060 2.858283 -0.491705
C -4.798107 3.452491 0.742628
C 4.872580 -0.270246 -0.443667
C 5.603086 -1.289833 -1.272438
C 5.431754 0.778577 0.198009
C 6.883472 1.202791 0.287908
C 7.870369 0.129271 0.786144
C 7.492542 -0.456846 2.140737
C 7.362304 1.839254 -1.019480
H 1.484246 -3.105283 0.148834
H 2.816295 1.525937 -0.673110
H 0.453351 2.254916 -0.701456
H -1.874141 2.964511 -0.945967
H -2.005741 -2.779760 0.121837
H -4.785103 -0.144450 1.919947
H -5.227064 -0.017062 -1.569564
H -6.116432 1.000442 -0.420307
H -7.337598 -2.737237 -0.298575
H -7.677654 -1.328332 -1.332232
H -6.208397 -2.319131 -1.604836
H -5.535492 2.824013 1.250380
H -3.942957 3.651905 1.397842
H -5.277266 4.407926 0.509128
H 5.037590 -1.531476 -2.180115
H 6.578615 -0.938718 -1.612415
H 5.753769 -2.213225 -0.703236
H 4.779330 1.423113 0.789296
H 6.908706 2.005773 1.039656
H 7.978077 -0.683626 0.061758
H 8.865193 0.583864 0.879001
H 6.554370 -1.017335 2.090224
H 8.272353 -1.145640 2.481415
H 7.385969 0.330837 2.893338
H 8.351830 2.290336 -0.886332
H 6.679253 2.632304 -1.344012
H 7.439361 1.109566 -1.830732
SCF Energy (B3LYP/6-31G**)= -1361.70175836
Number of imaginary frequencies = 0

1b_c190
MMFF Geometry
C -1.498471 -2.392488 -0.203101
C -2.219308 0.225096 -0.294825
N -2.820237 -2.096326 -0.156004
C -0.469741 -1.449230 -0.298697
C -0.855441 -0.098825 -0.348722
C -3.166116 -0.791866 -0.191456
C 0.133050 0.895569 -0.454635
C 1.498197 0.569836 -0.507115
C 1.880186 -0.773524 -0.449329
C 0.900814 -1.778635 -0.349983
C 3.321114 -1.122616 -0.501533
C 4.371535 0.007231 -0.588276
C 3.800317 1.414101 -0.664206
C 2.478012 1.651883 -0.626447
O 1.230180 -3.114053 -0.306092
O 3.630036 -2.318810 -0.496363
O 5.101483 -0.220842 -1.814269
C 5.391958 -0.172601 0.544466
C 4.819642 0.051990 1.926701
C 5.814026 0.112798 3.056703
O 3.612156 0.151422 2.140633
O 4.777125 2.388013 -0.780665
C 4.380566 3.753486 -0.830652
C -4.621206 -0.510508 -0.138686
C -5.449350 -1.412398 -1.011077
C -5.095099 0.470601 0.659674
C -6.524223 0.904034 0.923284
C -7.069129 1.760247 -0.234184
C -8.419914 2.395304 0.067808
C -7.450033 -0.247496 1.326798
H -1.276587 -3.456802 -0.161104
H -2.541008 1.260601 -0.346206
H -0.172611 1.940669 -0.492617
H 2.077089 2.658127 -0.675868
H 2.210948 -3.193848 -0.348094
H 4.546705 0.097985 -2.545840
H 6.233956 0.517215 0.404455
H 5.817863 -1.183769 0.521225
H 5.289308 0.280836 4.001299
H 6.357952 -0.833006 3.116797
H 6.509409 0.938919 2.889034
H 3.764523 3.949418 -1.714046
H 3.857362 4.042591 0.086462
H 5.285306 4.363507 -0.908969
H -4.953941 -1.588625 -1.973198
H -5.610018 -2.379617 -0.523456
H -6.423995 -0.984403 -1.252638
H -4.377870 1.035772 1.257029
H -6.468274 1.557829 1.806289
H -6.355188 2.563536 -0.457973
H -7.161109 1.163067 -1.148294
H -8.379567 2.987191 0.987581
H -8.712488 3.061225 -0.750467
H -9.203083 1.638930 0.174614
H -8.343388 0.137421 1.830738
H -6.954170 -0.928482 2.027415
H -7.791610 -0.829783 0.466864
SCF Energy (B3LYP/6-31G**)= -1361.70100675
Number of imaginary frequencies = 0

1b_c191
MMFF Geometry
C -1.405612 -2.231124 -0.583876
C -2.271234 0.345297 -0.642597
N -2.742630 -2.009460 -0.587316
C -0.430051 -1.229882 -0.609416
C -0.890157 0.101351 -0.641990
C -3.161705 -0.726044 -0.606453
C 0.040568 1.155977 -0.686667
C 1.417002 0.902778 -0.675515
C 1.866845 -0.421410 -0.623788
C 0.955905 -1.484897 -0.612889
C 3.328854 -0.656358 -0.608514
C 4.231072 0.486512 -0.073881
C 3.673629 1.824856 -0.547452
C 2.371401 2.003418 -0.815074
O 1.372524 -2.793661 -0.613072
O 3.759303 -1.748794 -0.980227
O 5.557878 0.327857 -0.583648
C 4.315630 0.430445 1.455922
C 4.432004 -0.985067 1.975411
C 5.774823 -1.661064 1.895572
O 3.440795 -1.565721 2.420117
O 4.552348 2.850130 -0.868235
C 5.289295 3.344884 0.246557
C -4.631263 -0.529406 -0.612491
C -5.379986 -1.483727 -1.503639
C -5.199236 0.422450 0.158741
C -6.675495 0.726773 0.310098
C -7.067568 0.620678 1.796697
C -8.561694 0.781210 2.042481
C -6.961266 2.114001 -0.269289
H -1.125649 -3.282270 -0.559128
H -2.649567 1.361909 -0.685932
H -0.317693 2.183568 -0.736239
H 2.011041 2.956967 -1.191060
H 2.350931 -2.816116 -0.709600
H 5.499603 0.431585 -1.551316
H 3.420247 0.864982 1.919452
H 5.174219 0.990474 1.842894
H 5.762235 -2.567875 2.506883
H 5.997172 -1.932112 0.861722
H 6.548369 -0.995091 2.285956
H 4.642071 3.505660 1.115462

H 6.113678 2.671948 0.497278
H 5.720606 4.309098 -0.038400
H -5.479460 -2.462314 -1.022272
H -6.380948 -1.127963 -1.760064
H -4.855637 -1.618458 -2.457053
H -4.557428 1.039993 0.787834
H -7.278979 0.001076 -0.243711
H -6.762655 -0.361128 2.181925
H -6.528056 1.370994 2.388729
H -9.137577 0.067742 1.444777
H -8.789241 0.599507 3.097892
H -8.902406 1.792176 1.801224
H -8.034883 2.326415 -0.277423
H -6.610001 2.185834 -1.305105
H -6.464913 2.901243 0.309311
SCF Energy (B3LYP/6-31G**)= -1361.69287040
Number of imaginary frequencies = 0

1b_c192
MMFF Geometry
C 1.640335 -2.257622 0.301910
C 2.543359 0.288690 0.007672
N 2.979246 -2.051133 0.333816
C 0.680473 -1.255186 0.128207
C 1.160109 0.058936 -0.021626
C 3.416950 -0.783359 0.178576
C 0.244627 1.111304 -0.200341
C -1.137158 0.875678 -0.222155
C -1.611869 -0.428578 -0.065650
C -0.709387 -1.492284 0.095935
C -3.074885 -0.664969 -0.107159
C -4.040172 0.527887 0.116597
C -3.375213 1.842911 -0.268805
C -2.055417 1.986676 -0.447169
O -1.137220 -2.792684 0.226499
O -3.463574 -1.823831 -0.278898
O -4.336735 0.626455 1.511861
C -5.314874 0.301529 -0.723342
C -6.127602 -0.883200 -0.239256
C -6.843252 -1.697094 -1.282711
O -6.240216 -1.131203 0.963324
O -4.194412 2.939180 -0.515955
C -4.688044 3.507050 0.699710
C 4.887908 -0.598811 0.226252
C 5.562308 -1.373953 1.323804
C 5.500434 0.192802 -0.680610
C 6.971058 0.508632 -0.861597
C 7.555135 1.430482 0.225043
C 6.826440 2.763719 0.342032
C 7.835331 -0.740004 -1.061957
H 1.344959 -3.297298 0.426668
H 2.935694 1.296498 -0.088157
H 0.618124 2.127536 -0.322695
H -1.646734 2.948812 -0.741993
H -2.111210 -2.817688 0.089509
H -4.930729 -0.125538 1.731668
H -5.049260 0.143198 -1.775685
H -5.996175 1.158736 -0.671563
H -7.376593 -2.522867 -0.803865
H -7.562929 -1.065222 -1.808676
H -6.117108 -2.110455 -1.987015
H -5.493888 2.894860 1.114886
H -3.887982 3.644272 1.435525
H -5.103827 4.490827 0.462476
H 5.634069 -2.434360 1.059539
H 6.568608 -1.013912 1.542246
H 5.000413 -1.288278 2.261309
H 4.881976 0.671585 -1.441503
H 7.037550 1.062210 -1.810080
H 7.550353 0.937599 1.202839
H 8.606916 1.635941 -0.011302
H 5.793847 2.629822 0.678457
H 7.332654 3.404716 1.071031
H 6.813177 3.289340 -0.618042
H 8.832718 -0.456695 -1.416832
H 7.396313 -1.407518 -1.811645
H 7.972033 -1.308950 -0.138618
SCF Energy (B3LYP/6-31G**)= -1361.70187174
Number of imaginary frequencies = 0

1b_c193
MMFF Geometry
C -1.555713 -2.223910 -0.270586
C -2.176177 0.380214 0.190538
N -2.854502 -1.912136 -0.040483
C -0.502999 -1.302829 -0.291273
C -0.836424 0.040866 -0.050249
C -3.152303 -0.614102 0.180364
C 0.179248 1.013264 -0.055131
C 1.520058 0.671962 -0.296508
C 1.850159 -0.665702 -0.532217
C 0.843075 -1.648064 -0.533050
C 3.265359 -1.031686 -0.785481
C 4.349528 0.068815 -0.754412
C 3.832248 1.476565 -0.505357
C 2.529314 1.733321 -0.298252
O 1.120401 -2.974595 -0.772107
O 3.524174 -2.213198 -1.037237
O 4.957614 0.066158 -2.065158
C 5.458301 -0.351050 0.220822
C 5.020678 -0.385393 1.668526
C 6.113323 -0.565630 2.689813
O 3.841877 -0.303241 2.011106
O 4.834298 2.431423 -0.524618
C 4.491603 3.789270 -0.273444
C -4.582809 -0.315113 0.436272
C -5.250013 -1.292176 1.364257
C -5.172099 0.741120 -0.165298
C -6.607334 1.225831 -0.098048
C -7.623827 0.168166 -0.568254
C -9.022616 0.733784 -0.780090
C -6.938167 1.835797 1.266218
H -1.373711 -3.282310 -0.445021
H -2.453991 1.408838 0.398402
H -0.085397 2.052786 0.135822
H 2.167407 2.739047 -0.115909
H 2.089293 -3.067355 -0.922700
H 4.353273 0.533050 -2.666325
H 6.312259 0.332984 0.135596
H 5.838433 -1.348173 -0.035372
H 5.681825 -0.571940 3.694600
H 6.622638 -1.516778 2.516821
H 6.823119 0.262059 2.617231
H 3.808717 4.168264 -1.040459
H 4.063654 3.905907 0.727395
H 5.410334 4.381510 -0.318147
H -4.616261 -1.493014 2.235990
H -6.199022 -0.926358 1.758221
H -5.444286 -2.240571 0.852013
H -4.567166 1.344866 -0.843678
H -6.664610 2.052971 -0.821145
H -7.282917 -0.265907 -1.516930
H -7.694756 -0.657621 0.146558
H -9.005481 1.575811 -1.479107
H -9.679402 -0.037151 -1.196001
H -9.464768 1.072059 0.161548
H -7.861494 2.422317 1.216948
H -6.143593 2.513887 1.597797
H -7.073955 1.073486 2.038385
SCF Energy (B3LYP/6-31G**)= -1361.70124118
Number of imaginary frequencies = 0

1b_c194
MMFF Geometry
C -1.409452 -2.331260 -0.465714
C -2.371698 0.137311 0.143539
N -2.737174 -2.208417 -0.223217
C -0.489834 -1.278814 -0.426802
C -0.999332 -0.004557 -0.107986
C -3.205772 -0.976724 0.070768
C -0.126975 1.098578 -0.057445
C 1.242656 0.945366 -0.301177

C 1.744182 -0.326063 -0.601944
C 0.886906 -1.430054 -0.686700
C 3.198290 -0.456497 -0.849728
C 4.129714 0.610853 -0.217938
C 3.455461 1.976058 -0.306132
C 2.121646 2.114908 -0.337228
O 1.347572 -2.678685 -1.025962
O 3.605752 -1.415549 -1.506276
O 5.365463 0.654231 -0.936593
C 4.454469 0.244978 1.235097
C 4.703394 -1.235836 1.414970
C 6.041477 -1.785283 0.997647
O 3.816336 -1.963744 1.862988
O 4.234271 3.104295 -0.522388
C 5.117343 3.407577 0.554112
C -4.661888 -0.889009 0.333812
C -5.212843 -2.019452 1.161066
C -5.392253 0.130284 -0.167191
C -6.885394 0.337258 -0.030235
C -7.218570 1.416250 1.018290
C -6.705782 2.817111 0.706684
C -7.500308 0.648572 -1.398400
H -1.088633 -3.343981 -0.701029
H -2.784540 1.106111 0.407215
H -0.526716 2.086505 0.168290
H 1.671531 3.098160 -0.442461
H 2.298363 -2.612073 -1.268149
H 5.152740 0.951791 -1.840330
H 3.626997 0.510500 1.906053
H 5.341497 0.771135 1.605091
H 6.158509 -2.797723 1.394429
H 6.109340 -1.819998 -0.091362
H 6.841167 -1.162391 1.406054
H 4.608292 3.339339 1.521559
H 5.995345 2.756473 0.532013
H 5.462483 4.437587 0.424124
H -4.535838 -2.262543 1.988442
H -6.174185 -1.775602 1.621175
H -5.346146 -2.916607 0.547482
H -4.891288 0.885558 -0.772578
H -7.359734 -0.591562 0.306401
H -8.307914 1.466561 1.140651
H -6.814878 1.107535 1.991231
H -7.138585 3.206859 -0.219064
H -6.984656 3.502017 1.514368
H -5.615664 2.838034 0.617863
H -8.578660 0.818103 -1.309001
H -7.351949 -0.190406 -2.087939
H -7.054419 1.535832 -1.860354
SCF Energy (B3LYP/6-31G**)= -1361.69185293
Number of imaginary frequencies = 0

1b_c195
MMFF Geometry
C -2.002127 1.602985 -0.350390
C -2.183206 -1.096792 -0.117068
N -3.223754 1.030939 -0.475023
C -0.816379 0.905274 -0.109335
C -0.922356 -0.492071 0.011992
C -3.311435 -0.314528 -0.360106
C 0.234115 -1.252086 0.266709
C 1.490398 -0.643424 0.386624
C 1.591693 0.744174 0.249377
C 0.446241 1.519432 0.018798
C 2.929062 1.370613 0.361353
C 4.187114 0.488670 0.226423
C 3.897799 -0.928453 0.673751
C 2.664327 -1.454415 0.702239
O 0.508805 2.887868 -0.092236
O 3.012690 2.593725 0.513631
O 5.169611 1.079707 1.091300
C 4.658472 0.610871 -1.226135
C 5.757330 -0.353727 -1.614003
C 7.127926 -0.142522 -1.024691
O 5.540929 -1.258714 -2.422583
O 4.997561 -1.724925 0.951847
C 5.213269 -1.843463 2.358431
C -4.671451 -0.908012 -0.521143
C -4.735667 -2.329465 -1.011654
C -5.757650 -0.151906 -0.245495
C -7.218505 -0.536685 -0.323688
C -7.890055 -0.527365 1.065448
C -7.979839 0.829693 1.752890
C -7.941356 0.367180 -1.325288
H -2.002938 2.685806 -0.455180
H -2.274878 -2.173011 -0.013143
H 0.149064 -2.333357 0.372827
H 2.519963 -2.498732 0.964242
H 1.416365 3.185256 0.141976
H 5.015692 2.045933 1.068203
H 5.028316 1.623044 -1.434634
H 3.821815 0.440360 -1.917022
H 7.847413 -0.785803 -1.539578
H 7.432813 0.897232 -1.167665
H 7.133172 -0.393361 0.036880
H 5.337567 -0.863738 2.830408
H 4.392177 -2.390196 2.833957
H 6.134217 -2.414110 2.509498
H -4.517423 -3.027608 -0.196774
H -5.709388 -2.597370 -1.427808
H -4.014285 -2.496788 -1.819875
H -5.591380 0.875889 0.083066
H -7.321715 -1.560226 -0.696753
H -8.907146 -0.927574 0.965594
H -7.353012 -1.218327 1.728096
H -8.566765 1.540758 1.164589
H -8.473645 0.720272 2.724260
H -6.990403 1.258878 1.933983
H -9.008243 0.123451 -1.368508
H -7.529297 0.233879 -2.332164
H -7.844873 1.427721 -1.069206
SCF Energy (B3LYP/6-31G**)= -1361.70150602
Number of imaginary frequencies = 0

1b_c196
MMFF Geometry
C -1.706073 -2.149365 -0.109068
C -2.187982 0.460890 -0.692377
N -2.997027 -1.748321 -0.202964
C -0.594980 -1.319192 -0.291373
C -0.857066 0.029189 -0.591762
C -3.223960 -0.449100 -0.496906
C 0.219116 0.913868 -0.784899
C 1.550490 0.480543 -0.682267
C 1.806535 -0.859818 -0.378367
C 0.741529 -1.757577 -0.190416
C 3.210726 -1.318134 -0.274672
C 4.353644 -0.294753 -0.380258
C 3.925956 1.107868 -0.766454
C 2.631361 1.445549 -0.903591
O 0.952672 -3.087447 0.092200
O 3.443144 -2.525809 -0.143562
O 5.175158 -0.800211 -1.459951
C 5.214344 -0.332959 0.889066
C 4.538852 0.313363 2.076403
C 5.435735 0.975859 3.087819
O 3.320993 0.257669 2.244004
O 4.993804 1.975326 -0.928088
C 4.736388 3.285150 -1.419077
C -4.645636 -0.037100 -0.596555
C -5.022104 0.769733 -1.803881
C -5.476736 -0.396244 0.405548
C -6.953663 -0.156623 0.646713
C -7.166154 0.827469 1.817181
C -6.681680 2.249208 1.559245
C -7.823584 0.206839 -0.557302
H -1.581409 -3.203778 0.128018
H -2.414137 1.500770 -0.906506
H 0.010125 1.957573 -1.016922
H 2.329089 2.452780 -1.166644
H 1.921757 -3.255187 0.096075
H 5.290560 -1.755592 -1.293972

H 6.167537 0.178639 0.703791
H 5.473363 -1.361237 1.169320
H 4.831406 1.402464 3.893153
H 6.119812 0.235903 3.510232
H 5.997902 1.780528 2.607417
H 4.251704 3.249082 -2.400092
H 4.136954 3.858251 -0.704640
H 5.697231 3.794975 -1.536213
H -4.184432 0.911317 -2.495401
H -5.800360 0.265067 -2.383385
H -5.369704 1.765359 -1.510747
H -5.031583 -0.975807 1.220620
H -7.341729 -1.126672 0.991605
H -8.233869 0.862129 2.068028
H -6.655817 0.444336 2.710493
H -7.212286 2.709657 0.721165
H -6.862216 2.868722 2.443972
H -5.608530 2.278113 1.349068
H -8.880604 0.227562 -0.267990
H -7.723140 -0.540426 -1.351522
H -7.584979 1.189519 -0.971493
SCF Energy (B3LYP/6-31G**)= -1361.69884503
Number of imaginary frequencies = 0

1b_c197
MMFF Geometry
C -1.545094 -1.994622 -0.408154
C -2.253025 0.531208 0.305169
N -2.851661 -1.751824 -0.142645
C -0.526137 -1.039034 -0.343779
C -0.904109 0.265189 0.028125
C -3.193011 -0.492464 0.203694
C 0.074427 1.273505 0.107406
C 1.421612 0.999394 -0.164208
C 1.791315 -0.301738 -0.517937
C 0.826707 -1.313129 -0.627302
C 3.217631 -0.568656 -0.800853
C 4.264888 0.400048 -0.202546
C 3.728606 1.829980 -0.174120
C 2.407087 2.085467 -0.139796
O 1.156944 -2.589523 -1.013757
O 3.508230 -1.559010 -1.473904
O 5.443347 0.363227 -1.018423
C 4.678313 -0.020666 1.209581
C 4.856752 -1.515922 1.333966
C 6.131329 -2.124077 0.812768
O 3.961309 -2.209237 1.820104
O 4.724761 2.788640 -0.163054
C 4.354020 4.161146 -0.104944
C -4.631005 -0.271716 0.491780
C -5.256927 -1.353518 1.327433
C -5.263916 0.812398 -0.006775
C -6.717546 1.225596 0.101947
C -7.752548 0.177681 -0.351155
C -7.533407 -0.310658 -1.777587
C -7.042637 1.766252 1.496809
H -1.327666 -3.024267 -0.684819
H -2.567026 1.524560 0.610388
H -0.225981 2.284072 0.381928
H 2.013154 3.095087 -0.098015
H 2.110015 -2.611121 -1.257840
H 5.229388 0.824371 -1.848094
H 3.919016 0.267910 1.948541
H 5.616093 0.458589 1.516675
H 6.229237 -3.144602 1.193729
H 6.114436 -2.151245 -0.278458
H 6.989071 -1.544386 1.162821
H 3.768727 4.445253 -0.985315
H 3.807055 4.379310 0.817879
H 5.271121 4.757546 -0.102651
H -4.588557 -1.650693 2.144149
H -6.186931 -1.036915 1.802176
H -5.470689 -2.237317 0.717062
H -4.686629 1.502582 -0.623852
H -6.832689 2.077573 -0.584653
H -7.772667 -0.683778 0.323099
H -8.752776 0.626564 -0.296382
H -6.594064 -0.862926 -1.875045
H -8.345537 -0.983200 -2.072099
H -7.517641 0.527561 -2.481399
H -8.042311 2.214381 1.510839
H -6.328834 2.542876 1.793709
H -7.022360 0.982226 2.259306
SCF Energy (B3LYP/6-31G**)= -1361.69468128
Number of imaginary frequencies = 0

1b_c198
MMFF Geometry
C -1.865868 -2.178576 0.243396
C -2.327834 0.498890 0.162634
N -3.137336 -1.747621 0.424930
C -0.764845 -1.346257 0.017808
C -1.015897 0.038122 -0.020549
C -3.357001 -0.416256 0.374494
C 0.050345 0.928185 -0.245768
C 1.360689 0.462352 -0.421120
C 1.604550 -0.911847 -0.370423
C 0.551134 -1.815438 -0.167859
C 2.994459 -1.384817 -0.539157
C 4.151850 -0.385215 -0.354746
C 3.730260 1.033874 -0.704654
C 2.438268 1.411280 -0.709808
O 0.759008 -3.173674 -0.138177
O 3.201857 -2.580197 -0.771555
O 5.183835 -0.820025 -1.255874
C 4.651987 -0.547394 1.085106
C 5.643881 0.510473 1.515504
C 7.046667 0.427330 0.971934
O 5.312491 1.400381 2.301996
O 4.796501 1.870080 -0.982549
C 4.547175 3.250445 -1.214938
C -4.761475 0.012590 0.581654
C -5.458592 -0.706453 1.702917
C -5.308822 0.951842 -0.219999
C -6.716436 1.512125 -0.241769
C -7.852569 0.477786 -0.360074
C -7.716511 -0.433175 -1.573682
C -6.954130 2.473151 0.925947
H -1.749459 -3.259418 0.287517
H -2.543755 1.562925 0.153658
H -0.150329 1.998529 -0.285725
H 2.138097 2.427741 -0.939766
H 1.685087 -3.359347 -0.412557
H 5.105215 -1.792253 -1.326954
H 5.131442 -1.524167 1.228430
H 3.813313 -0.509921 1.793203
H 7.688159 1.128232 1.513882
H 7.439130 -0.581938 1.119498
H 7.062129 0.685716 -0.087970
H 3.937634 3.391352 -2.113126
H 4.076244 3.715457 -0.342889
H 5.509959 3.742776 -1.380605
H -4.797596 -0.806604 2.571889
H -6.343539 -0.177220 2.059708
H -5.766597 -1.708449 1.385930
H -4.689390 1.369892 -1.015038
H -6.773806 2.126482 -1.152712
H -7.931106 -0.136659 0.541825
H -8.806974 1.013583 -0.443643
H -6.833094 -1.074255 -1.499823
H -8.593007 -1.084343 -1.653301
H -7.645184 0.150761 -2.496797
H -7.909174 2.995970 0.803819
H -6.166210 3.233044 0.976450
H -6.983076 1.954852 1.888776
SCF Energy (B3LYP/6-31G**)= -1361.70324884
Number of imaginary frequencies = 0

1b_c199
MMFF Geometry
C -1.801847 -2.459756 0.034494
C -2.412931 0.173328 -0.251557

N -3.107466 -2.101670 0.084856
C -0.736879 -1.572897 -0.153481
C -1.065632 -0.212590 -0.303493
C -3.399086 -0.790092 -0.048689
C -0.039802 0.728022 -0.510050
C 1.305758 0.337681 -0.552073
C 1.626107 -1.011644 -0.387139
C 0.615374 -1.966829 -0.204935
C 3.050851 -1.403229 -0.415383
C 4.127600 -0.320686 -0.211616
C 3.652213 1.042056 -0.692257
C 2.344730 1.333641 -0.823367
O 0.900396 -3.304380 -0.068546
O 3.347424 -2.595017 -0.548086
O 5.254493 -0.745135 -0.996484
C 4.516216 -0.355729 1.270728
C 5.402778 0.791435 1.702207
C 6.848359 0.765966 1.278149
O 4.953656 1.707036 2.395242
O 4.685867 1.925613 -0.945669
C 4.374516 3.268212 -1.295044
C -4.839440 -0.440751 0.007417
C -5.721991 -1.371587 -0.776739
C -5.250484 0.619765 0.735830
C -6.648301 1.151578 0.978188
C -7.279307 1.843170 -0.244623
C -6.449057 3.004813 -0.777047
C -7.593404 0.108672 1.582610
H -1.625879 -3.526726 0.154226
H -2.692778 1.214342 -0.381736
H -0.299973 1.778254 -0.639759
H 2.004173 2.311135 -1.146956
H 1.855507 -3.446394 -0.255543
H 5.239513 -1.722962 -1.006021
H 5.040877 -1.287164 1.519035
H 3.621329 -0.328206 1.906808
H 7.398517 1.542836 1.816926
H 7.288107 -0.202723 1.528522
H 6.936102 0.953306 0.206886
H 3.832810 3.308852 -2.245330
H 3.807301 3.758799 -0.497504
H 5.316553 3.809984 -1.420900
H -5.858927 -2.316757 -0.240689
H -6.709440 -0.953495 -0.977046
H -5.281794 -1.590272 -1.756730
H -4.495655 1.189862 1.279903
H -6.537528 1.925379 1.752433
H -7.449081 1.128209 -1.056440
H -8.266931 2.230197 0.036667
H -5.486661 2.664336 -1.171038
H -6.983114 3.504752 -1.591548
H -6.260688 3.744627 0.007418
H -8.504497 0.592868 1.951993
H -7.125455 -0.403259 2.430722
H -7.902872 -0.648331 0.857270
SCF Energy (B3LYP/6-31G**)= -1361.70332432
Number of imaginary frequencies = 0

1b_c200
MMFF Geometry
C -1.659080 -1.341471 -0.767279
C -2.058623 1.221247 0.040226
N -2.931673 -0.931334 -0.546426
C -0.525684 -0.540756 -0.610793
C -0.744025 0.785151 -0.190969
C -3.127819 0.345426 -0.144159
C 0.355676 1.647398 -0.021917
C 1.663247 1.201009 -0.245936
C 1.872706 -0.123020 -0.647384
C 0.789559 -0.986556 -0.850935
C 3.267482 -0.566722 -0.872092
C 4.390611 0.200100 -0.126085
C 4.051446 1.687009 -0.106928
C 2.787541 2.133572 -0.156398
O 0.965426 -2.275786 -1.290524
O 3.472282 -1.537095 -1.602350
O 5.633095 0.014457 -0.809603
C 4.560281 -0.348959 1.295310
C 4.455913 -1.856782 1.347227
C 5.649475 -2.665102 0.913015
O 3.407453 -2.393633 1.707692
O 5.076504 2.617775 -0.202844
C 5.959134 2.617785 0.915907
C -4.543311 0.763034 0.074054
C -4.855483 2.226177 -0.088598
C -5.472258 -0.172615 0.371304
C -6.945926 0.030381 0.651027
C -7.768381 -0.517879 -0.528797
C -9.262089 -0.251507 -0.402125
C -7.304266 -0.649078 1.976637
H -1.571349 -2.377688 -1.087050
H -2.235533 2.237986 0.375026
H 0.184286 2.679426 0.281857
H 2.579776 3.199663 -0.183392
H 1.915800 -2.412804 -1.502821
H 5.532648 0.424871 -1.688237
H 3.787419 0.045067 1.968230
H 5.527399 -0.074774 1.731263
H 5.514760 -3.706095 1.220182
H 5.754152 -2.625373 -0.172902
H 6.552195 -2.279879 1.393633
H 5.407437 2.589632 1.861728
H 6.664788 1.784965 0.854513
H 6.536225 3.546806 0.887170
H -4.330081 2.645078 -0.954600
H -5.916594 2.417018 -0.269112
H -4.559826 2.784851 0.805446
H -5.152161 -1.215101 0.434931
H -7.171486 1.094931 0.770509
H -7.418477 -0.054444 -1.460505
H -7.607566 -1.598160 -0.636830
H -9.460606 0.815582 -0.261195
H -9.778609 -0.574419 -1.311860
H -9.698591 -0.800584 0.437136
H -8.332915 -0.420500 2.272722
H -6.650273 -0.301051 2.784501
H -7.205944 -1.738463 1.909802
SCF Energy (B3LYP/6-31G**)= -1361.69373127
Number of imaginary frequencies = 0

1b_c201
MMFF Geometry
C 1.723120 2.113213 0.123627
C 2.215772 -0.417232 -0.737040
N 3.015468 1.736033 -0.030755
C 0.615523 1.296939 -0.128886
C 0.883426 -0.010373 -0.573064
C 3.247725 0.476638 -0.462167
C -0.188223 -0.879889 -0.843005
C -1.519751 -0.470059 -0.677704
C -1.783532 0.828763 -0.229387
C -0.722627 1.710826 0.037096
C -3.191868 1.262860 -0.060842
C -4.335053 0.251156 -0.268234
C -3.884575 -1.083259 -0.820255
C -2.596173 -1.412795 -0.977291
O -0.939044 3.001728 0.460838
O -3.431944 2.448518 0.197314
O -5.176165 0.886332 -1.258128
C -5.166303 0.117075 1.012327
C -4.446457 -0.666791 2.084909
C -5.260536 -1.661709 2.867235
O -3.258100 -0.470435 2.337837
O -4.845617 -2.070426 -1.003431
C -5.648775 -1.834206 -2.160065
C 4.670879 0.090248 -0.624664
C 5.034535 -0.585529 -1.913649
C 5.514895 0.352617 0.396494
C 6.997096 0.102770 0.590237
C 7.234829 -0.993279 1.651271
C 6.756771 -2.385651 1.257120
C 7.850888 -0.128912 -0.657105

H 1.594353 3.135658 0.472250
H 2.446145 -1.427111 -1.061870
H 0.022885 -1.892371 -1.185576
H -2.330248 -2.411685 -1.311077
H -1.908969 3.157017 0.506079
H -5.264139 1.820332 -0.981157
H -6.119545 -0.377328 0.785919
H -5.429595 1.096277 1.430280
H -4.624672 -2.160118 3.604108
H -6.069228 -1.143462 3.388162
H -5.668635 -2.415114 2.188906
H -6.468366 -1.150742 -1.921068
H -5.057818 -1.454948 -3.001175
H -6.090042 -2.789630 -2.459472
H 4.187804 -0.661383 -2.604469
H 5.801245 -0.018320 -2.448999
H 5.392257 -1.603397 -1.729229
H 5.078138 0.841513 1.273205
H 7.383428 1.036119 1.026290
H 8.306521 -1.043850 1.881618
H 6.735609 -0.707433 2.586317
H 7.277717 -2.753859 0.368877
H 6.955429 -3.090258 2.071488
H 5.680745 -2.402484 1.060786
H 8.912351 -0.170110 -0.386878
H 7.733142 0.694476 -1.369456
H 7.612664 -1.066289 -1.165761
SCF Energy (B3LYP/6-31G**)= -1361.69319591
Number of imaginary frequencies = 0

1b_c202
MMFF Geometry
C -1.457536 -2.198095 -0.691497
C -2.328077 0.328433 -0.193514
N -2.792669 -1.997767 -0.573267
C -0.486679 -1.197950 -0.578064
C -0.949251 0.107070 -0.322981
C -3.213043 -0.741189 -0.315345
C -0.023087 1.161049 -0.214327
C 1.353912 0.931704 -0.337073
C 1.806704 -0.370160 -0.571392
C 0.897201 -1.427775 -0.711613
C 3.263272 -0.589339 -0.698364
C 4.208086 0.458465 -0.063856
C 3.616780 1.862277 -0.176866
C 2.289461 2.060174 -0.286000
O 1.312732 -2.707970 -0.987393
O 3.657048 -1.603363 -1.277115
O 5.461912 0.426485 -0.758623
C 4.496000 0.140101 1.405063
C 4.718914 -1.335841 1.639519
C 6.061205 -1.916148 1.282398
O 3.807319 -2.038616 2.079879
O 4.569187 2.863388 -0.130222
C 4.141844 4.219018 -0.195432
C -4.680894 -0.563747 -0.196976
C -5.468299 -1.294686 -1.248628
C -5.193441 0.182976 0.804992
C -6.636551 0.480249 1.157559
C -7.336925 1.444550 0.182189
C -6.628157 2.786690 0.046121
C -7.471178 -0.782029 1.394715
H -1.174867 -3.230095 -0.889153
H -2.710875 1.328272 -0.013530
H -0.387936 2.171593 -0.033929
H 1.854617 3.051940 -0.345875
H 2.285739 -2.701564 -1.134399
H 5.313336 0.828461 -1.632196
H 3.656867 0.437373 2.047991
H 5.379657 0.677846 1.770310
H 6.160390 -2.907189 1.734267
H 6.153088 -2.008013 0.198463
H 6.857215 -1.279290 1.676203
H 3.636069 4.423915 -1.144404
H 3.498195 4.467020 0.654613
H 5.030519 4.854814 -0.142578
H -5.508834 -2.366135 -1.025465
H -6.493085 -0.932392 -1.341051
H -5.011166 -1.164073 -2.236529
H -4.497379 0.630275 1.516425
H -6.601266 0.991410 2.131191
H -7.436980 0.994888 -0.811203
H -8.357378 1.632130 0.539688
H -5.637774 2.674838 -0.405530
H -7.211767 3.455349 -0.594864
H -6.512175 3.269967 1.021409
H -8.425027 -0.521556 1.867265
H -6.952131 -1.478466 2.062481
H -7.705522 -1.311128 0.467307
SCF Energy (B3LYP/6-31G**)= -1361.69474903
Number of imaginary frequencies = 0

1b_c203
MMFF Geometry
C -1.703022 -1.785236 -0.400315
C -2.054077 0.876568 0.001161
N -2.967337 -1.326690 -0.232945
C -0.555599 -0.987562 -0.385111
C -0.749851 0.387982 -0.179129
C -3.137315 -0.000336 -0.030004
C 0.366054 1.243768 -0.160660
C 1.670084 0.753705 -0.340006
C 1.860900 -0.616759 -0.537365
C 0.752960 -1.483074 -0.564026
C 3.236405 -1.140096 -0.724051
C 4.432523 -0.163664 -0.666480
C 4.062892 1.297431 -0.466563
C 2.788663 1.698953 -0.319556
O 0.891983 -2.836528 -0.770124
O 3.374051 -2.348072 -0.943496
O 5.090048 -0.263081 -1.949298
C 5.447512 -0.678821 0.363632
C 4.949569 -0.632550 1.791397
C 5.971962 -0.912038 2.861918
O 3.774623 -0.410848 2.081276
O 5.163396 2.137121 -0.461968
C 4.961624 3.529894 -0.252919
C -4.539152 0.467727 0.170808
C -4.723142 1.664601 1.061341
C -5.549269 -0.215366 -0.412527
C -7.043135 0.038854 -0.411248
C -7.690087 -0.463999 0.891873
C -9.212194 -0.424563 0.865666
C -7.425892 1.477231 -0.773196
H -1.633574 -2.860030 -0.553867
H -2.215417 1.939078 0.150574
H 0.210058 2.310016 0.000163
H 2.532075 2.742075 -0.172128
H 1.850128 -3.037970 -0.876800
H 4.565484 0.252118 -2.584928
H 6.373943 -0.093595 0.303256
H 5.726498 -1.716890 0.142302
H 5.501585 -0.848164 3.847053
H 6.377237 -1.917833 2.727232
H 6.773105 -0.170953 2.806138
H 4.355493 3.961767 -1.055647
H 4.509379 3.716881 0.726332
H 5.940701 4.017657 -0.270538
H -4.547488 2.590893 0.504740
H -5.722335 1.713606 1.498367
H -4.033712 1.632306 1.912967
H -5.284402 -1.095491 -1.004229
H -7.440604 -0.582305 -1.227641
H -7.377313 -1.500076 1.075684
H -7.340268 0.122622 1.748887
H -9.605499 -0.983026 0.010462
H -9.614736 -0.875679 1.778395
H -9.585333 0.602461 0.812148
H -8.469839 1.524178 -1.102308
H -6.811874 1.856176 -1.597702
H -7.325210 2.162372 0.072753
SCF Energy (B3LYP/6-31G**)= -1361.70126621

Number of imaginary frequencies = 0

1b_c204
MMFF Geometry
C 1.869784 2.459234 0.006969
C 2.576456 -0.159397 0.165023
N 3.187127 2.162523 0.120264
C 0.837895 1.515643 -0.032998
C 1.216238 0.164359 0.052042
C 3.528167 0.857964 0.187943
C 0.224143 -0.831193 0.022954
C -1.135586 -0.503366 -0.089549
C -1.511994 0.840162 -0.173759
C -0.529061 1.845408 -0.148583
C -2.951324 1.187384 -0.284810
C -4.017754 0.071854 -0.248548
C -3.433417 -1.323588 -0.223541
C -2.123443 -1.576759 -0.118099
O -0.851318 3.180246 -0.230240
O -3.256431 2.379961 -0.397685
O -4.722672 0.240850 -1.483383
C -4.899912 0.343988 0.983216
C -6.277904 -0.265494 0.881694
C -6.652752 -1.314533 1.894050
O -7.084892 0.132434 0.039844
O -4.314672 -2.396302 -0.173406
C -4.901011 -2.671428 -1.448105
C 4.978228 0.577105 0.313698
C 5.707877 1.497735 1.254567
C 5.548027 -0.417604 -0.399924
C 7.008410 -0.814597 -0.424734
C 7.213441 -2.241269 0.117369
C 6.852585 -2.381297 1.591072
C 7.531452 -0.719463 -1.861813
H 1.654878 3.524186 -0.052366
H 2.889633 -1.195598 0.248211
H 0.521243 -1.877801 0.087211
H -1.775329 -2.604163 -0.054436
H -1.824194 3.262587 -0.357251
H -5.372770 0.959466 -1.357367
H -5.071595 1.419526 1.123593
H -4.408434 0.002096 1.902574
H -7.579591 -1.806338 1.586219
H -5.868086 -2.070535 1.965249
H -6.800870 -0.840658 2.867408
H -5.765810 -2.025132 -1.620193
H -4.175465 -2.574640 -2.263385
H -5.257745 -3.705656 -1.432493
H 5.122446 1.664760 2.166388
H 6.668365 1.094474 1.584437
H 5.892073 2.466962 0.779242
H 4.923301 -1.004383 -1.073990
H 7.614040 -0.130064 0.177188
H 6.623614 -2.960276 -0.465254
H 8.265981 -2.525644 -0.006016
H 5.785333 -2.211630 1.762550
H 7.088397 -3.391698 1.940327
H 7.418851 -1.671449 2.202367
H 8.598474 -0.964111 -1.902013
H 7.408172 0.294906 -2.257685
H 7.003823 -1.409437 -2.530210
SCF Energy (B3LYP/6-31G**)= -1361.70346215
Number of imaginary frequencies = 0

1b_c205
MMFF Geometry
C -1.788809 -1.788768 -0.358196
C -2.421088 0.841277 -0.117159
N -3.096670 -1.438737 -0.413737
C -0.730122 -0.893800 -0.184350
C -1.069627 0.464718 -0.058699
C -3.407664 -0.127438 -0.295396
C -0.049403 1.414638 0.128785
C 1.298808 1.033168 0.178548
C 1.633954 -0.315831 0.042762
C 0.625533 -1.278452 -0.124404
C 3.062294 -0.706729 0.113413
C 4.153368 0.373709 -0.103384
C 3.626297 1.756354 0.257959
C 2.326541 2.042158 0.410624
O 0.913211 -2.618823 -0.232448
O 3.321756 -1.898485 0.302903
O 4.482739 0.424962 -1.493817
C 5.381841 0.021613 0.761773
C 6.071430 -1.248253 0.303139
C 6.677503 -2.122849 1.366684
O 6.177844 -1.519592 -0.894957
O 4.553587 2.761527 0.510428
C 5.127117 3.259543 -0.700780
C -4.855513 0.222970 -0.379413
C -5.183226 1.578808 -0.939457
C -5.774317 -0.687393 0.013581
C -7.285252 -0.606941 0.067544
C -7.875821 0.606775 0.811397
C -7.371749 0.742404 2.242728
C -7.900839 -0.762250 -1.325430
H -1.606696 -2.856251 -0.462412
H -2.693512 1.886005 -0.008751
H -0.313442 2.466376 0.235153
H 2.018105 3.045522 0.689473
H 1.876633 -2.746308 -0.078896
H 4.996558 -0.388522 -1.696026
H 5.082665 -0.096129 1.810427
H 6.151709 0.800558 0.714948
H 7.127638 -3.005942 0.904978
H 7.451406 -1.565974 1.900350
H 5.899097 -2.448716 2.061011
H 5.870147 2.560066 -1.094694
H 4.359780 3.473154 -1.453055
H 5.641497 4.195866 -0.464804
H -4.530853 1.822802 -1.785888
H -6.201089 1.641272 -1.327957
H -5.065567 2.351760 -0.172929
H -5.404039 -1.651150 0.372559
H -7.605477 -1.491112 0.638777
H -7.684394 1.540186 0.273808
H -8.967682 0.498555 0.845868
H -6.299745 0.958203 2.274363
H -7.890431 1.564761 2.746030
H -7.558244 -0.173149 2.812990
H -8.988959 -0.868495 -1.254438
H -7.514393 -1.655537 -1.828937
H -7.694649 0.100060 -1.966262
SCF Energy (B3LYP/6-31G**)= -1361.70214695
Number of imaginary frequencies = 0

1b_c206
MMFF Geometry
C -1.640702 -1.516347 -0.358452
C -2.073036 1.074777 0.333153
N -2.907742 -1.135365 -0.065264
C -0.528937 -0.671579 -0.333382
C -0.764426 0.669313 0.025100
C -3.119874 0.155173 0.280985
C 0.311305 1.576666 0.055140
C 1.613956 1.161016 -0.244071
C 1.842321 -0.177690 -0.581417
C 0.780207 -1.087500 -0.648733
C 3.231757 -0.587847 -0.888075
C 4.378588 0.261009 -0.279617
C 3.985564 1.734087 -0.322490
C 2.705490 2.134308 -0.298881
O 0.970590 -2.394544 -1.025163
O 3.417012 -1.594548 -1.572975
O 5.571351 0.074512 -1.046414
C 4.677270 -0.192219 1.154255
C 4.633249 -1.696383 1.304603
C 5.819066 -2.491329 0.826686
O 3.636303 -2.243109 1.778524
O 4.964102 2.690704 -0.554008
C 5.929328 2.789669 0.489623
C -4.524876 0.531736 0.612716

C -4.715088 1.691678 1.552410
C -5.543987 -0.186943 0.091496
C -7.031093 0.028745 0.274202
C -7.705499 0.123893 -1.108558
C -9.185844 0.473649 -1.038348
C -7.602285 -1.109339 1.122499
H -1.538818 -2.566222 -0.625063
H -2.266159 2.109259 0.597186
H 0.124486 2.619333 0.309468
H 2.455796 3.189111 -0.373059
H 1.906710 -2.512811 -1.302207
H 5.388522 0.425793 -1.937320
H 3.944306 0.217968 1.861298
H 5.664171 0.139905 1.495257
H 5.746552 -3.515122 1.204515
H 5.839009 -2.515513 -0.264536
H 6.741062 -2.048351 1.211376
H 5.453290 2.802079 1.476017
H 6.659341 1.978582 0.422366
H 6.466782 3.733785 0.360286
H -4.594108 2.641446 1.021209
H -5.698370 1.696118 2.029689
H -3.990139 1.653627 2.373699
H -5.299785 -1.031666 -0.556678
H -7.222800 0.970481 0.797886
H -7.201114 0.895684 -1.704603
H -7.589820 -0.820314 -1.655967
H -9.345589 1.396696 -0.472320
H -9.583693 0.622585 -2.047419
H -9.766172 -0.326550 -0.570122
H -8.654236 -0.931876 1.366881
H -7.061357 -1.200246 2.071485
H -7.533237 -2.071615 0.602749
SCF Energy (B3LYP/6-31G**)= -1361.69377610
Number of imaginary frequencies = 0

1b_c207
MMFF Geometry
C 1.937603 2.293489 -0.110529
C 2.529039 -0.322930 -0.541989
N 3.242769 1.947755 -0.225479
C 0.863459 1.401528 -0.195602
C 1.182397 0.051043 -0.420974
C 3.526171 0.643915 -0.431919
C 0.145787 -0.893065 -0.522720
C -1.199982 -0.515464 -0.398863
C -1.517055 0.826662 -0.170836
C -0.489724 1.781845 -0.073443
C -2.940640 1.224936 -0.031791
C -4.049019 0.151612 -0.081127
C -3.535277 -1.234601 -0.403339
C -2.236056 -1.536325 -0.513978
O -0.753364 3.114181 0.144917
O -3.197856 2.423277 0.129106
O -4.885539 0.582115 -1.160573
C -4.765199 0.193280 1.280780
C -6.173870 -0.349162 1.235532
C -6.477705 -1.570490 2.061306
O -7.054083 0.229487 0.596253
O -4.456311 -2.273293 -0.454816
C -5.201286 -2.272358 -1.675209
C 4.965137 0.310189 -0.556544
C 5.762606 1.298017 -1.365000
C 5.470771 -0.783814 0.052337
C 6.912736 -1.243455 0.071334
C 7.438635 -1.390132 1.512465
C 7.490614 -0.068017 2.268372
C 7.023222 -2.579954 -0.666895
H 1.769556 3.355042 0.058854
H 2.796798 -1.357270 -0.734611
H 0.396456 -1.938405 -0.702201
H -1.933931 -2.563936 -0.697104
H -1.729201 3.244151 0.160612
H -5.479509 1.277311 -0.815879
H -4.865930 1.222831 1.649348
H -4.186139 -0.337916 2.046403
H -7.457400 -1.967562 1.781676
H -5.728182 -2.344469 1.883886
H -6.486517 -1.298105 3.119441
H -6.047655 -1.583686 -1.607524
H -4.572845 -2.031600 -2.539827
H -5.603889 -3.279593 -1.818460
H 5.984329 2.191082 -0.771220
H 6.708757 0.886477 -1.724886
H 5.208728 1.605579 -2.259803
H 4.802762 -1.413895 0.640128
H 7.562018 -0.528786 -0.443745
H 6.820493 -2.100787 2.075601
H 8.452306 -1.809263 1.484825
H 6.490701 0.350860 2.416636
H 7.937904 -0.218272 3.256213
H 8.097556 0.667245 1.730520
H 8.063544 -2.921766 -0.695429
H 6.676245 -2.486064 -1.702093
H 6.426705 -3.360095 -0.180306
SCF Energy (B3LYP/6-31G**)= -1361.70348799
Number of imaginary frequencies = 0

1b_c208
MMFF Geometry
C 1.853030 -1.592246 0.240128
C 2.365569 1.030774 -0.241425
N 3.146712 -1.191368 0.198552
C 0.751625 -0.753419 0.052851
C 1.029089 0.602273 -0.195436
C 3.398512 0.116164 -0.041176
C -0.037143 1.497420 -0.396116
C -1.369632 1.064673 -0.342168
C -1.642475 -0.281038 -0.086925
C -0.588659 -1.190496 0.097007
C -3.055580 -0.727825 -0.048868
C -4.176661 0.325788 0.145669
C -3.725406 1.694660 -0.346925
C -2.448009 2.014817 -0.592107
O -0.815968 -2.527747 0.323081
O -3.276161 -1.939323 -0.133323
O -4.435189 0.472167 1.544280
C -5.433193 -0.136404 -0.621867
C -6.046177 -1.391310 -0.032026
C -6.670825 -2.366785 -0.992044
O -6.079193 -1.573110 1.187087
O -4.704435 2.643104 -0.622513
C -5.233418 3.211920 0.577749
C 4.834962 0.517248 -0.096424
C 5.169911 1.681079 -0.986818
C 5.741050 -0.191977 0.613049
C 7.236915 -0.013842 0.765017
C 8.048513 -0.462414 -0.464426
C 7.814990 -1.919437 -0.844723
C 7.639801 1.392819 1.218855
H 1.719857 -2.653865 0.437025
H 2.588400 2.077688 -0.419382
H 0.178264 2.546715 -0.595526
H -2.195359 3.004301 -0.961856
H -1.780068 -2.701820 0.232400
H -4.904436 -0.341445 1.834950
H -5.184390 -0.323754 -1.673552
H -6.230421 0.615365 -0.591488
H -7.060355 -3.227385 -0.441334
H -7.493246 -1.881215 -1.522782
H -5.916986 -2.716770 -1.701575
H -5.926182 2.518517 1.063340
H -4.437027 3.510353 1.268439
H -5.796595 4.108136 0.300979
H 4.936146 2.627847 -0.488947
H 6.221728 1.704999 -1.276072
H 4.607634 1.631029 -1.926446
H 5.369739 -1.037126 1.198523
H 7.527840 -0.677267 1.593126
H 7.827600 0.169293 -1.331377
H 9.117220 -0.332222 -0.251835
H 6.783989 -2.090342 -1.169025

H 8.474307 -2.201073 -1.672101
H 8.028296 -2.584161 -0.001614
H 8.684800 1.397929 1.549124
H 7.026242 1.726451 2.062954
H 7.552849 2.134011 0.420191
SCF Energy (B3LYP/6-31G**)= -1361.70222985
Number of imaginary frequencies = 0

1b_c209
MMFF Geometry
C -1.492486 -2.228191 -0.577604
C -2.336297 0.351687 -0.721290
N -2.827569 -1.996020 -0.600306
C -0.508468 -1.236080 -0.623649
C -0.957292 0.097093 -0.700308
C -3.236388 -0.710829 -0.661231
C -0.017599 1.142529 -0.767622
C 1.356551 0.878896 -0.735824
C 1.795018 -0.446672 -0.640292
C 0.875317 -1.502096 -0.606318
C 3.254841 -0.692638 -0.604082
C 4.161403 0.458550 -0.095264
C 3.619380 1.786483 -0.613901
C 2.321199 1.967266 -0.899115
O 1.281130 -2.813546 -0.563460
O 3.679737 -1.799101 -0.938860
O 5.491537 0.274180 -0.587399
C 4.231295 0.447678 1.436268
C 4.331093 -0.952515 1.998926
C 5.668953 -1.641320 1.952175
O 3.331003 -1.511629 2.451289
O 4.509424 2.794689 -0.956797
C 5.240031 3.316760 0.149679
C -4.704807 -0.498957 -0.687888
C -5.453052 -1.478385 -1.552518
C -5.264738 0.478622 0.057060
C -6.731265 0.836388 0.165353
C -7.354496 0.312016 1.474013
C -6.776326 0.891721 2.759277
C -6.912709 2.348617 0.001254
H -1.221517 -3.280353 -0.518983
H -2.705765 1.369606 -0.798627
H -0.366974 2.170971 -0.851267
H 1.972176 2.911949 -1.306837
H 2.260174 -2.846650 -0.649960
H 5.443112 0.349367 -1.558243
H 3.335285 0.903072 1.878056
H 5.090871 1.012157 1.814485
H 5.643129 -2.529330 2.590091
H 5.898722 -1.944910 0.929047
H 6.444299 -0.970188 2.329951
H 4.586112 3.508623 1.007189
H 6.056498 2.645123 0.428237
H 5.681870 4.268554 -0.159845
H -5.400829 -2.487761 -1.129905
H -6.511170 -1.235692 -1.668025
H -5.024973 -1.502203 -2.561222
H -4.615821 1.084312 0.690201
H -7.291769 0.376998 -0.655101
H -8.432534 0.516681 1.458470
H -7.250599 -0.780204 1.508111
H -6.920544 1.974586 2.812446
H -7.280887 0.449644 3.624866
H -5.708094 0.676878 2.855156
H -7.967968 2.625215 0.098735
H -6.574581 2.672389 -0.989821
H -6.342836 2.916821 0.744154
SCF Energy (B3LYP/6-31G**)= -1361.69238208
Number of imaginary frequencies = 0

1b_c210
MMFF Geometry
C -1.754596 -1.266841 -0.668288
C -2.083651 1.331775 0.051480
N -3.014341 -0.818394 -0.448393
C -0.600743 -0.488772 -0.552819
C -0.782574 0.856061 -0.178291
C -3.175536 0.476178 -0.089751
C 0.339244 1.696909 -0.052186
C 1.633113 1.211368 -0.274340
C 1.806301 -0.130746 -0.630451
C 0.700607 -0.974504 -0.791191
C 3.187392 -0.615739 -0.854346
C 4.337132 0.149691 -0.148527
C 4.033661 1.644109 -0.178844
C 2.780235 2.119064 -0.230463
O 0.840577 -2.282389 -1.186318
O 3.360462 -1.616081 -1.551629
O 5.566724 -0.090074 -0.838485
C 4.510409 -0.352531 1.289655
C 4.370828 -1.854596 1.396588
C 5.539636 -2.706447 0.978559
O 3.314210 -2.352800 1.787436
O 5.079358 2.545832 -0.319146
C 5.974847 2.564181 0.789186
C -4.578187 0.935061 0.128452
C -4.856702 2.400534 -0.071237
C -5.529230 0.032068 0.455612
C -6.992746 0.286884 0.739613
C -7.882363 -0.075805 -0.465160
C -7.866809 -1.543194 -0.876157
C -7.415999 -0.448998 2.015052
H -1.695355 -2.315554 -0.951803
H -2.232093 2.363832 0.351348
H 0.196037 2.742912 0.216340
H 2.597573 3.188237 -0.293289
H 1.784863 -2.449804 -1.403918
H 5.465727 0.291197 -1.730089
H 3.755110 0.083702 1.956450
H 5.488838 -0.086522 1.704946
H 5.383818 -3.732325 1.324192
H 5.632453 -2.707809 -0.109161
H 6.456853 -2.326367 1.435227
H 5.433810 2.582939 1.741376
H 6.659731 1.712889 0.749896
H 6.573484 3.477373 0.720966
H -4.337648 2.780519 -0.958705
H -5.916464 2.612526 -0.236868
H -4.530610 2.977103 0.800589
H -5.234226 -1.015459 0.545570
H -7.149855 1.352018 0.944682
H -8.917828 0.208447 -0.238910
H -7.580373 0.529702 -1.329450
H -8.234937 -2.189168 -0.074010
H -8.518218 -1.692003 -1.743764
H -6.862486 -1.873655 -1.156829
H -8.478904 -0.285079 2.222306
H -6.848842 -0.082317 2.878336
H -7.246347 -1.528746 1.945592
SCF Energy (B3LYP/6-31G**)= -1361.69292175
Number of imaginary frequencies = 0

1b_c211
MMFF Geometry
C -1.659956 -1.915967 -0.437309
C -2.088037 0.762642 -0.339859
N -2.940648 -1.475612 -0.385110
C -0.531936 -1.091392 -0.447181
C -0.765718 0.292309 -0.396473
C -3.150432 -0.140180 -0.335401
C 0.329888 1.174177 -0.403794
C 1.651737 0.702554 -0.460340
C 1.881255 -0.675389 -0.505816
C 0.794438 -1.568407 -0.503004
C 3.275458 -1.179037 -0.563140
C 4.446325 -0.171024 -0.541891
C 4.036345 1.292682 -0.511484
C 2.747947 1.673748 -0.475194
O 0.972349 -2.931767 -0.559761
O 3.449787 -2.399050 -0.649673
O 5.175796 -0.381639 -1.771253
C 5.413006 -0.553570 0.587692

C 4.835660 -0.377068 1.974805
C 5.802579 -0.520295 3.121081
O 3.642025 -0.158523 2.177639
O 5.117530 2.156869 -0.532351
C 4.876056 3.557810 -0.474989
C -4.573709 0.308520 -0.293389
C -4.868240 1.653125 -0.897517
C -5.504261 -0.511998 0.243969
C -6.995039 -0.325047 0.441755
C -7.352697 0.945388 1.236749
C -8.802002 0.968459 1.706995
C -7.758363 -0.459332 -0.877914
H -1.560237 -2.998722 -0.472867
H -2.276390 1.829674 -0.285099
H 0.143455 2.246812 -0.361489
H 2.462314 2.719388 -0.448254
H 1.938867 -3.118028 -0.593661
H 4.676908 0.053448 -2.482882
H 6.329170 0.046518 0.517417
H 5.725071 -1.601008 0.488239
H 5.276931 -0.370086 4.068160
H 6.235663 -1.523467 3.111337
H 6.589906 0.232342 3.032758
H 4.307445 3.892421 -1.348487
H 4.365677 3.828943 0.454755
H 5.844460 4.066558 -0.488460
H -4.312473 1.793583 -1.831829
H -5.919643 1.780039 -1.159888
H -4.596343 2.455788 -0.204149
H -5.161184 -1.477720 0.624434
H -7.309753 -1.175806 1.064213
H -6.705396 1.015449 2.120262
H -7.174370 1.847698 0.643853
H -9.038884 0.078061 2.297766
H -8.977775 1.847362 2.335755
H -9.496277 1.021135 0.863348
H -8.830682 -0.587253 -0.697685
H -7.419765 -1.336352 -1.441250
H -7.633893 0.419167 -1.517452
SCF Energy (B3LYP/6-31G**)= -1361.70157519
Number of imaginary frequencies = 0

1b_c212
MMFF Geometry
C -1.778325 -2.470133 0.061810
C -2.380675 0.159427 -0.274000
N -3.082076 -2.105325 0.114019
C -0.711348 -1.591845 -0.152221
C -1.035430 -0.233088 -0.328200
C -3.369189 -0.795499 -0.043931
C -0.007702 0.698932 -0.563512
C 1.335170 0.301433 -0.606175
C 1.651357 -1.046070 -0.412925
C 0.638394 -1.993086 -0.205326
C 3.077438 -1.443014 -0.437978
C 4.136760 -0.347790 -0.175692
C 3.681565 0.986430 -0.756906
C 2.378604 1.280371 -0.921446
O 0.920977 -3.328471 -0.045773
O 3.345816 -2.633451 -0.610348
O 5.361301 -0.754861 -0.796749
C 4.370362 -0.312002 1.339098
C 5.188841 0.876368 1.790699
C 6.677324 0.836500 1.563111
O 4.647336 1.850890 2.317861
O 4.729945 1.831740 -1.070837
C 4.437815 3.191540 -1.373720
C -4.807635 -0.438871 0.015813
C -5.700007 -1.381916 -0.742210
C -5.208719 0.638051 0.725470
C -6.602454 1.180520 0.967685
C -7.240274 1.849483 -0.264148
C -6.409594 2.996491 -0.826775
C -7.546932 0.154164 1.600731
H -1.605571 -3.535112 0.202523
H -2.657436 1.198683 -0.423231
H -0.264406 1.747014 -0.715057
H 2.046045 2.234837 -1.314834
H 1.873558 -3.476058 -0.247718
H 5.255983 -0.612072 -1.753663
H 4.884534 -1.219552 1.680143
H 3.419338 -0.275170 1.886604
H 7.156590 1.618370 2.159239
H 7.075524 -0.129533 1.883399
H 6.902987 1.006476 0.508903
H 3.879329 3.269655 -2.311744
H 3.895065 3.669217 -0.551542
H 5.387898 3.718283 -1.502690
H -5.836576 -2.315303 -0.185806
H -6.687296 -0.963893 -0.943451
H -5.268341 -1.622504 -1.720863
H -4.447405 1.215739 1.252236
H -6.482527 1.969587 1.724972
H -7.419278 1.118724 -1.059752
H -8.224103 2.246306 0.016792
H -5.451690 2.643999 -1.221147
H -6.948028 3.481821 -1.647208
H -6.212116 3.751462 -0.059171
H -8.453178 0.649603 1.967118
H -7.074398 -0.342169 2.455546
H -7.865065 -0.616294 0.893531
SCF Energy (B3LYP/6-31G**)= -1361.69730219
Number of imaginary frequencies = 0

1b_c213
MMFF Geometry
C 1.843829 2.183712 0.262455
C 2.295015 -0.493989 0.132149
N 3.111812 1.743337 0.446193
C 0.741505 1.360926 0.010620
C 0.986786 -0.023964 -0.053641
C 3.326077 0.412261 0.371546
C -0.080709 -0.904972 -0.307835
C -1.386786 -0.429208 -0.484040
C -1.625568 0.945484 -0.405041
C -0.570282 1.840138 -0.176920
C -3.015609 1.427333 -0.570865
C -4.163340 0.420889 -0.325468
C -3.755571 -0.976721 -0.778740
C -2.466560 -1.361561 -0.817577
O -0.774483 3.198115 -0.124197
O -3.189246 2.617339 -0.840397
O -5.305671 0.858267 -1.070275
C -4.515698 0.506695 1.164133
C -5.446435 -0.592729 1.623576
C -6.906396 -0.475377 1.271843
O -5.015787 -1.559636 2.256383
O -4.831244 -1.775842 -1.120142
C -4.609109 -3.169485 -1.306048
C 4.726728 -0.026898 0.583042
C 5.416243 0.666543 1.724974
C 5.277643 -0.952320 -0.232139
C 6.683071 -1.518132 -0.251944
C 7.824554 -0.486572 -0.338866
C 7.703731 0.449071 -1.535185
C 6.905746 -2.503355 0.898439
H 1.731339 3.263960 0.327464
H 2.506665 -1.558530 0.104153
H 0.115810 -1.974972 -0.369653
H -2.169889 -2.360296 -1.119014
H -1.696137 3.390291 -0.413345
H -5.136276 0.641956 -2.003908
H -4.991291 1.466689 1.401959
H -3.614750 0.448524 1.789019
H -7.483254 -1.180452 1.877227
H -7.260989 0.534419 1.493075
H -7.060405 -0.706205 0.216301
H -3.986844 -3.349760 -2.188193
H -4.164494 -3.619038 -0.412207
H -5.579852 -3.644360 -1.476060
H 4.747490 0.752239 2.589546
H 6.295528 0.126381 2.079380

H 5.731473 1.673297 1.430997
H 4.663980 -1.351598 -1.041131
H 6.746494 -2.114386 -1.174442
H 7.897137 0.109355 0.575865
H 8.777471 -1.024728 -0.424239
H 6.822328 1.092375 -1.456746
H 8.583636 1.097890 -1.593539
H 7.638687 -0.115962 -2.470448
H 7.859723 -3.027762 0.774790
H 6.114240 -3.260685 0.926322
H 6.927753 -2.004561 1.871697
SCF Energy (B3LYP/6-31G**)= -1361.69725805
Number of imaginary frequencies = 0

1b_c214
MMFF Geometry
C 1.559394 -2.231130 0.273939
C 2.459563 0.322807 0.043206
N 2.897749 -2.023207 0.317290
C 0.598691 -1.226521 0.118689
C 1.076816 0.091482 0.001646
C 3.334112 -0.751348 0.193944
C 0.160337 1.146157 -0.157403
C -1.220916 0.908830 -0.191607
C -1.694111 -0.399457 -0.067591
C -0.790573 -1.465109 0.073966
C -3.156483 -0.637235 -0.121907
C -4.124978 0.548560 0.124386
C -3.460408 1.873209 -0.227245
C -2.139990 2.023212 -0.395588
O -1.216759 -2.768881 0.172344
O -3.542270 -1.792472 -0.322242
O -4.428716 0.614453 1.520032
C -5.395045 0.339528 -0.726986
C -6.208109 -0.857377 -0.274447
C -6.917056 -1.648192 -1.339989
O -6.326341 -1.133207 0.921515
O -4.280283 2.973539 -0.453198
C -4.781004 3.512414 0.772723
C 4.804493 -0.567592 0.252248
C 5.477271 -1.369348 1.331647
C 5.423351 0.248031 -0.628840
C 6.899252 0.550962 -0.801769
C 7.392866 1.544052 0.265878
C 8.809480 2.044361 0.016863
C 7.771941 -0.701332 -0.928871
H 1.265244 -3.273905 0.373071
H 2.850418 1.333196 -0.027629
H 0.532625 2.165560 -0.254429
H -1.731529 2.992558 -0.666097
H -2.190008 -2.792321 0.029924
H -5.022482 -0.143382 1.719444
H -5.123871 0.205933 -1.781340
H -6.078104 1.194194 -0.658919
H -7.451344 -2.485657 -0.883003
H -7.635190 -1.005557 -1.854908
H -6.186646 -2.044018 -2.049932
H -5.587850 2.889497 1.169592
H -3.984887 3.633937 1.515544
H -5.197311 4.500720 0.556131
H 5.612299 -2.408279 1.013073
H 6.453172 -0.966169 1.608277
H 4.880802 -1.364854 2.251633
H 4.810094 0.755693 -1.374941
H 6.976176 1.061135 -1.773412
H 6.722060 2.412659 0.290511
H 7.352823 1.092477 1.263455
H 8.900917 2.486829 -0.980034
H 9.070950 2.811588 0.752811
H 9.541297 1.236224 0.107532
H 8.735108 -0.452412 -1.387732
H 7.296228 -1.453327 -1.568154
H 7.986354 -1.161104 0.039555
SCF Energy (B3LYP/6-31G**)= -1361.70076484
Number of imaginary frequencies = 0

1b_c215
MMFF Geometry
C -1.766757 -2.461374 -0.070274
C -2.531389 0.139145 0.124381
N -3.086260 -2.196997 0.087462
C -0.760482 -1.492391 -0.138550
C -1.168048 -0.150261 -0.034456
C -3.456624 -0.901584 0.173343
C -0.202049 0.870575 -0.093798
C 1.161106 0.576632 -0.247426
C 1.561338 -0.757693 -0.345122
C 0.608272 -1.788158 -0.302004
C 2.999900 -1.059865 -0.505764
C 4.039494 0.047742 -0.252510
C 3.454040 1.448354 -0.337066
C 2.127774 1.672753 -0.331256
O 0.965475 -3.111260 -0.410987
O 3.334354 -2.208966 -0.811124
O 4.987098 -0.109109 -1.319999
C 4.686836 -0.198363 1.119850
C 6.197507 -0.194475 1.087646
C 6.906196 1.130782 1.012226
O 6.826911 -1.254337 1.097045
O 4.418540 2.439036 -0.387491
C 4.014074 3.776844 -0.652143
C -4.908124 -0.657079 0.348150
C -5.583193 -1.598602 1.308961
C -5.525730 0.325362 -0.342314
C -6.995647 0.685645 -0.317215
C -7.218133 2.105066 0.236902
C -6.812174 2.249611 1.698384
C -7.563560 0.581793 -1.736549
H -1.528083 -3.520523 -0.142151
H -2.867677 1.166705 0.222852
H -0.524585 1.908852 -0.020011
H 1.716886 2.675430 -0.376874
H 1.923742 -3.165128 -0.630623
H 5.224330 -1.058880 -1.347796
H 4.387713 -1.174363 1.526121
H 4.351024 0.524449 1.874152
H 6.910428 1.493059 -0.017498
H 6.416286 1.854718 1.668111
H 7.938474 1.009221 1.352860
H 3.473280 3.843653 -1.601705
H 3.411727 4.171389 0.172175
H 4.916011 4.390681 -0.732962
H -4.963574 -1.754496 2.199924
H -6.541549 -1.220036 1.672580
H -5.759639 -2.570355 0.835861
H -4.938645 0.929449 -1.034593
H -7.563677 -0.015702 0.301766
H -6.666101 2.840412 -0.362135
H -8.280960 2.363325 0.149528
H -5.735918 2.106232 1.833783
H -7.061407 3.252715 2.059163
H -7.339950 1.523933 2.325334
H -8.637109 0.799703 -1.740452
H -7.428181 -0.427950 -2.140216
H -7.075813 1.286829 -2.419366
SCF Energy (B3LYP/6-31G**)= -1361.70398097
Number of imaginary frequencies = 0

1b_c216
MMFF Geometry
C -1.847941 -2.287593 -0.341112
C -2.492493 0.343693 -0.555689
N -3.160981 -1.956463 -0.386905
C -0.791193 -1.372984 -0.394217
C -1.137020 -0.014380 -0.507494
C -3.470713 -0.646049 -0.485325
C -0.118286 0.953254 -0.575782
C 1.236069 0.591491 -0.520440
C 1.574179 -0.757988 -0.398997
C 0.569778 -1.738054 -0.347407
C 3.003725 -1.131514 -0.340016
C 4.062672 -0.039416 -0.099903

C 3.566161 1.358567 -0.432702
C 2.262306 1.630607 -0.620664
O 0.867141 -3.076595 -0.247616
O 3.311159 -2.322233 -0.454933
O 5.121358 -0.370009 -1.012038
C 4.527638 -0.126885 1.362661
C 6.029453 -0.182536 1.519647
C 6.810620 1.094496 1.367342
O 6.596760 -1.254284 1.741150
O 4.580726 2.297688 -0.487074
C 4.281936 3.602608 -0.967998
C -4.917867 -0.329111 -0.537083
C -5.727599 -1.267316 -1.391237
C -5.418849 0.706854 0.169341
C -6.866234 1.137937 0.270755
C -7.344734 1.165900 1.735405
C -7.350610 -0.210267 2.389786
C -7.022307 2.524212 -0.359563
H -1.658507 -3.355777 -0.257335
H -2.782859 1.384399 -0.661682
H -0.392091 2.003443 -0.673898
H 1.913223 2.633018 -0.843353
H 1.840778 -3.193770 -0.335406
H 5.309633 -1.323342 -0.890301
H 4.131379 -1.029580 1.847666
H 4.142478 0.700420 1.972097
H 7.786901 0.981140 1.847375
H 6.957055 1.317802 0.308908
H 6.284402 1.915823 1.860088
H 3.862369 3.562860 -1.978431
H 3.607408 4.124884 -0.282267
H 5.218254 4.166630 -1.012783
H -5.915913 -2.205987 -0.859511
H -6.691320 -0.846334 -1.688058
H -5.199826 -1.497484 -2.324258
H -4.740940 1.302351 0.781293
H -7.521746 0.452768 -0.275571
H -6.718641 1.842890 2.330336
H -8.365049 1.567767 1.773143
H -6.339922 -0.621236 2.472738
H -7.766119 -0.143088 3.400386
H -7.964262 -0.913505 1.817804
H -8.068098 2.848598 -0.327722
H -6.709530 2.515010 -1.409767
H -6.421494 3.275868 0.165087
SCF Energy (B3LYP/6-31G**)= -1361.70403735
Number of imaginary frequencies = 0

1b_c217
MMFF Geometry
C 1.676847 -2.050893 -0.381751
C 2.435167 0.559405 -0.387241
N 3.002849 -1.774855 -0.423560
C 0.661555 -1.090034 -0.341736
C 1.065920 0.258360 -0.348636
C 3.368390 -0.475047 -0.415157
C 0.091135 1.272984 -0.326539
C -1.275189 0.964820 -0.278289
C -1.668606 -0.374629 -0.252695
C -0.712813 -1.399468 -0.303850
C -3.115096 -0.674249 -0.202767
C -4.060289 0.410186 0.369405
C -3.567155 1.801460 -0.039778
C -2.270929 2.038333 -0.319872
O -1.075236 -2.725232 -0.322791
O -3.487726 -1.787441 -0.580050
O -4.004781 0.387441 1.799205
C -5.497069 0.153703 -0.130232
C -6.063925 -1.152375 0.391047
C -7.028277 -1.885154 -0.501465
O -5.789548 -1.563614 1.520786
O -4.560889 2.766158 -0.043210
C -4.188693 4.128294 -0.216657
C 4.828090 -0.216698 -0.462990
C 5.567939 -1.082163 -1.444621
C 5.379355 0.709468 0.351113
C 6.829369 1.113018 0.524608
C 7.813703 -0.028690 0.844351
C 7.424673 -0.834390 2.077522
C 7.318901 1.960444 -0.652617
H 1.440344 -3.112868 -0.381570
H 2.771028 1.591552 -0.408840
H 0.407051 2.315307 -0.346118
H -1.906226 3.024890 -0.581486
H -2.052973 -2.781135 -0.415764
H -4.484849 -0.418124 2.091531
H -5.518843 0.137086 -1.226511
H -6.191479 0.927965 0.217383
H -7.349618 -2.810471 -0.015475
H -7.903214 -1.257600 -0.687203
H -6.536436 -2.137062 -1.444240
H -3.492554 4.448065 0.565482
H -3.764922 4.292851 -1.212389
H -5.093679 4.737152 -0.132012
H 5.011043 -1.168002 -2.385127
H 6.545795 -0.678494 -1.711785
H 5.715302 -2.088043 -1.037392
H 4.720332 1.244648 1.036689
H 6.846220 1.778141 1.400887
H 7.929500 -0.708148 -0.005396
H 8.806791 0.404121 1.021554
H 6.488046 -1.378691 1.924775
H 8.202638 -1.570427 2.304731
H 7.309796 -0.184532 2.950848
H 8.306313 2.383040 -0.436302
H 6.637336 2.796547 -0.845513
H 7.404738 1.377602 -1.574268
SCF Energy (B3LYP/6-31G**)= -1361.70709535
Number of imaginary frequencies = 0

1b_c218
MMFF Geometry
C 1.930942 2.025974 0.066588
C 2.277482 -0.593292 -0.563581
N 3.196146 1.545827 0.004626
C 0.779564 1.270766 -0.169045
C 0.970722 -0.084188 -0.494218
C 3.365394 0.240888 -0.309979
C -0.149525 -0.897479 -0.747714
C -1.452288 -0.385826 -0.669563
C -1.634293 0.957446 -0.333997
C -0.529495 1.788709 -0.099401
C -3.014670 1.476340 -0.237906
C -4.180274 0.478717 -0.100599
C -3.859346 -0.854716 -0.758670
C -2.592945 -1.245166 -0.994273
O -0.678278 3.120084 0.207333
O -3.201063 2.697284 -0.216185
O -5.294345 1.093198 -0.769418
C -4.498686 0.372528 1.395046
C -5.472429 -0.732328 1.740423
C -6.925860 -0.525224 1.401565
O -5.084225 -1.760944 2.298611
O -4.983115 -1.605496 -1.053297
C -4.817725 -2.920515 -1.567992
C 4.773048 -0.248002 -0.384798
C 5.039773 -1.373342 -1.344932
C 5.716941 0.352808 0.373844
C 7.194960 0.061923 0.526796
C 7.570773 -1.395480 0.858360
C 6.880778 -1.928383 2.107806
C 7.992130 0.586068 -0.670383
H 1.865270 3.081262 0.322683
H 2.435169 -1.640369 -0.800956
H 0.002118 -1.944036 -1.010791
H -2.362470 -2.204156 -1.445233
H -1.622399 3.369562 0.090112
H -5.186609 2.059853 -0.667240
H -4.919484 1.311680 1.776387
H -3.583764 0.188330 1.973982
H -7.524707 -1.306695 1.878183
H -7.258264 0.443259 1.783628

H -7.078016 -0.576204 0.322373
H -4.325104 -2.898273 -2.545215
H -4.265321 -3.550210 -0.863141
H -5.812011 -3.356706 -1.701862
H 4.479367 -1.236098 -2.276959
H 6.088051 -1.437125 -1.641405
H 4.754102 -2.333565 -0.903083
H 5.397107 1.183084 1.008764
H 7.522894 0.658175 1.391412
H 7.361227 -2.065759 0.019482
H 8.655005 -1.448652 1.021676
H 5.797749 -2.000201 1.971225
H 7.252578 -2.931140 2.342023
H 7.079657 -1.284936 2.970730
H 9.067901 0.519602 -0.473827
H 7.757541 1.637874 -0.869111
H 7.787778 0.018271 -1.582849
SCF Energy (B3LYP/6-31G**)= -1361.70367186
Number of imaginary frequencies = 0

1b_c219
MMFF Geometry
C 1.586061 -2.306508 0.004366
C 2.495637 0.254742 0.042969
N 2.924760 -2.109104 0.076279
C 0.629971 -1.287456 -0.051971
C 1.112493 0.034835 -0.030476
C 3.365632 -0.832979 0.087387
C 0.200681 1.105040 -0.088904
C -1.180737 0.877860 -0.154082
C -1.653197 -0.436020 -0.159938
C -0.758620 -1.515479 -0.128418
C -3.113930 -0.650172 -0.230462
C -4.038229 0.491305 0.258842
C -3.431296 1.848925 -0.108185
C -2.104534 2.007710 -0.279152
O -1.195965 -2.817847 -0.172309
O -3.518595 -1.742226 -0.635526
O -4.104869 0.473465 1.688239
C -5.440389 0.316788 -0.360265
C -6.125462 -0.950560 0.112414
C -7.052348 -1.630848 -0.857952
O -5.972485 -1.370339 1.261803
O -4.362551 2.870423 -0.194789
C -3.897318 4.207542 -0.335132
C 4.836157 -0.659902 0.174641
C 5.496230 -1.567116 1.175427
C 5.460458 0.236323 -0.619870
C 6.933586 0.567864 -0.743136
C 7.505951 1.346266 0.456125
C 6.777824 2.656845 0.728924
C 7.798413 -0.648821 -1.086585
H 1.287630 -3.352726 -0.005578
H 2.890889 1.265263 0.079261
H 0.578062 2.126889 -0.083173
H -1.661772 2.969780 -0.509638
H -2.163809 -2.817184 -0.348443
H -4.654840 -0.300628 1.939552
H -5.370526 0.294993 -1.454441
H -6.114688 1.132593 -0.073379
H -7.467740 -2.532732 -0.400007
H -7.869843 -0.954137 -1.117952
H -6.498179 -1.916921 -1.755308
H -3.251429 4.488866 0.502896
H -3.382699 4.342831 -1.291691
H -4.768659 4.869024 -0.326067
H 4.923138 -1.596885 2.109590
H 5.569821 -2.586731 0.782469
H 6.500272 -1.239729 1.448755
H 4.851674 0.807361 -1.322651
H 7.012191 1.234503 -1.614860
H 7.488767 0.736036 1.365172
H 8.560777 1.576369 0.259375
H 5.741091 2.485294 1.034072
H 7.276206 3.201098 1.537609
H 6.776794 3.297413 -0.158694
H 8.800365 -0.326611 -1.391958
H 7.367424 -1.217042 -1.918281
H 7.923278 -1.328168 -0.239331
SCF Energy (B3LYP/6-31G**)= -1361.70710947
Number of imaginary frequencies = 0

1b_c220
MMFF Geometry
C -1.554363 -2.001547 -0.367841
C -2.269865 0.532271 0.308405
N -2.862179 -1.758133 -0.109537
C -0.537527 -1.043057 -0.313245
C -0.919477 0.265327 0.039659
C -3.207382 -0.494676 0.217902
C 0.056847 1.276567 0.109587
C 1.405559 1.002260 -0.154238
C 1.777564 -0.302489 -0.490220
C 0.816799 -1.318035 -0.588648
C 3.201387 -0.565740 -0.773091
C 4.255771 0.416756 -0.227750
C 3.713569 1.840088 -0.170767
C 2.390548 2.089093 -0.127890
O 1.151455 -2.599043 -0.955046
O 3.520803 -1.559862 -1.431409
O 5.366346 0.382678 -1.142858
C 4.768869 0.016911 1.157725
C 4.949188 -1.475106 1.300034
C 6.255045 -2.072942 0.850007
O 4.032813 -2.175174 1.733100
O 4.704293 2.804195 -0.117864
C 4.327504 4.174881 -0.147195
C -4.646850 -0.272952 0.497775
C -5.273827 -1.344418 1.345805
C -5.279983 0.803038 -0.017808
C -6.734737 1.215002 0.079965
C -7.766173 0.159072 -0.362534
C -7.540941 -0.348277 -1.781362
C -7.065901 1.774026 1.466131
H -1.334181 -3.034330 -0.630073
H -2.586786 1.529228 0.598598
H -0.246146 2.289891 0.370918
H 1.996409 3.097096 -0.060518
H 2.105299 -2.625409 -1.190737
H 5.233191 -0.392422 -1.724460
H 4.065761 0.320540 1.944848
H 5.726618 0.499908 1.387352
H 6.308172 -3.116820 1.171909
H 6.328590 -2.032654 -0.238473
H 7.086805 -1.529740 1.305322
H 3.768938 4.408876 -1.059237
H 3.751416 4.439993 0.745017
H 5.241875 4.775507 -0.147954
H -4.607923 -1.629130 2.168951
H -6.206172 -1.023159 1.812765
H -5.483669 -2.236881 0.746787
H -4.701713 1.485968 -0.642058
H -6.848907 2.057314 -0.618618
H -7.787184 -0.693142 0.323342
H -8.767409 0.606791 -0.317519
H -6.600246 -0.900068 -1.867866
H -8.350766 -1.026292 -2.069605
H -7.524131 0.480287 -2.496491
H -8.066432 2.220437 1.470421
H -6.354599 2.555957 1.754992
H -7.046980 1.000509 2.239323
SCF Energy (B3LYP/6-31G**)= -1361.69926000
Number of imaginary frequencies = 0

1b_c221
MMFF Geometry
C -1.609138 -2.420644 -0.254410
C -2.364847 0.165995 0.092639
N -2.928747 -2.160947 -0.090830
C -0.598082 -1.453963 -0.257496
C -1.001774 -0.119547 -0.074800
C -3.294684 -0.871385 0.071308

C -0.031580 0.897668 -0.061077
C 1.330552 0.608323 -0.229738
C 1.731099 -0.718313 -0.412935
C 0.771895 -1.745512 -0.426515
C 3.174185 -1.023029 -0.578435
C 4.208256 0.119430 -0.586819
C 3.605831 1.491093 -0.390130
C 2.298098 1.700144 -0.196261
O 1.120446 -3.064850 -0.597784
O 3.517024 -2.199021 -0.742184
O 4.733698 0.057513 -1.932550
C 5.358641 -0.205449 0.377522
C 5.063080 0.066789 1.837759
C 4.045518 -0.799622 2.531675
O 5.647060 0.960105 2.451854
O 4.461464 2.576634 -0.263052
C 5.147973 2.893723 -1.472612
C -4.746446 -0.631572 0.250450
C -5.432840 -1.627792 1.145774
C -5.354488 0.395343 -0.381293
C -6.822726 0.761268 -0.343701
C -7.042267 2.144015 0.297487
C -6.646271 2.193754 1.767975
C -7.380758 0.750372 -1.770693
H -1.374721 -3.474642 -0.388017
H -2.696748 1.187404 0.252360
H -0.347475 1.930906 0.083252
H 1.937787 2.707952 -0.006273
H 2.092668 -3.123579 -0.732805
H 5.046064 -0.857065 -2.073036
H 6.247342 0.379894 0.105434
H 5.679508 -1.250918 0.288384
H 4.216172 -0.763830 3.611607
H 3.038911 -0.436141 2.319620
H 4.152908 -1.837692 2.207619
H 6.006335 2.230982 -1.613848
H 4.479589 2.856928 -2.339815
H 5.528673 3.915450 -1.381748
H -4.820847 -1.841524 2.029984
H -6.392488 -1.268253 1.524949
H -5.609529 -2.567487 0.611903
H -4.759413 1.039347 -1.029447
H -7.398715 0.025266 0.225701
H -6.482280 2.912771 -0.250002
H -8.103148 2.412975 0.219214
H -5.571744 2.036363 1.901532
H -6.893218 3.173374 2.189707
H -7.182146 1.432742 2.344101
H -8.453160 0.973877 -1.768307
H -7.247422 -0.232561 -2.236440
H -6.884558 1.494506 -2.404146
SCF Energy (B3LYP/6-31G**)= -1361.69840415
Number of imaginary frequencies = 0

1b_c222
MMFF Geometry
C 1.614497 -2.130166 0.105930
C 2.424657 0.406238 -0.437439
N 2.945653 -1.895736 0.008757
C 0.618211 -1.161515 -0.053288
C 1.049867 0.147051 -0.337241
C 3.337428 -0.630083 -0.251431
C 0.095654 1.163556 -0.521012
C -1.276775 0.899452 -0.411433
C -1.703078 -0.397959 -0.118320
C -0.763075 -1.428082 0.046762
C -3.158038 -0.665795 -0.021475
C -4.132707 0.521451 0.191295
C -3.535796 1.815485 -0.346477
C -2.238946 1.971125 -0.643666
O -1.144946 -2.723448 0.306249
O -3.529103 -1.841854 -0.073636
O -4.316943 0.722064 1.594939
C -5.465355 0.205578 -0.519951
C -6.204605 -0.953978 0.118312
C -6.981930 -1.859940 -0.797316
O -6.211848 -1.110520 1.341352
O -4.400744 2.872682 -0.607530
C -4.808797 3.523647 0.598173
C 4.802109 -0.416328 -0.353204
C 5.519034 -1.484578 -1.131476
C 5.374008 0.650034 0.247294
C 6.831471 1.061939 0.322162
C 7.727433 -0.006921 0.976220
C 9.120664 0.506362 1.318055
C 7.351525 1.560673 -1.028222
H 1.357224 -3.164464 0.324503
H 2.780131 1.404911 -0.671974
H 0.431359 2.174486 -0.750179
H -1.880722 2.915264 -1.043576
H -2.125944 -2.777753 0.253954
H -4.871074 -0.022297 1.919761
H -5.282473 -0.028468 -1.575801
H -6.162323 1.050558 -0.476416
H -7.452586 -2.656601 -0.214683
H -7.758470 -1.284851 -1.307311
H -6.305428 -2.312092 -1.526768
H -5.561626 2.929589 1.124364
H -3.955456 3.734056 1.252306
H -5.268501 4.477287 0.322100
H 4.981301 -1.715028 -2.058638
H 6.525946 -1.191127 -1.430901
H 5.600525 -2.403636 -0.541322
H 4.728203 1.326304 0.809858
H 6.848884 1.933292 0.993543
H 7.255362 -0.358384 1.902637
H 7.836172 -0.882341 0.328356
H 9.067983 1.394382 1.955686
H 9.681770 -0.263791 1.857234
H 9.687119 0.758136 0.416751
H 8.293190 2.105828 -0.905581
H 6.638857 2.250407 -1.494560
H 7.534936 0.741610 -1.729144
SCF Energy (B3LYP/6-31G**)= -1361.70101301
Number of imaginary frequencies = 0

1b_c223
MMFF Geometry
C -1.624602 -1.672205 -0.856988
C -2.194907 0.908214 -0.228385
N -2.926660 -1.316649 -0.736122
C -0.542693 -0.805987 -0.682552
C -0.849353 0.527865 -0.356721
C -3.206805 -0.031115 -0.422323
C 0.196371 1.452282 -0.174291
C 1.537607 1.064686 -0.297026
C 1.833509 -0.266393 -0.605323
C 0.805135 -1.195133 -0.816763
C 3.254680 -0.653729 -0.731207
C 4.308578 0.231873 -0.025447
C 3.893403 1.701472 -0.058309
C 2.601841 2.065736 -0.168118
O 1.066900 -2.498675 -1.162808
O 3.532452 -1.672333 -1.366438
O 5.560700 0.088763 -0.709182
C 4.531379 -0.205667 1.424006
C 4.570827 -1.709051 1.570224
C 5.839140 -2.425713 1.190987
O 3.573977 -2.320934 1.958425
O 4.958982 2.574569 0.058829
C 4.700025 3.973600 0.073250
C -4.650981 0.326696 -0.311903
C -5.018504 1.740205 -0.666505
C -5.534846 -0.625194 0.062282
C -7.030847 -0.553148 0.283648
C -7.526278 0.561377 1.225755
C -6.874466 0.523417 2.602306
C -7.789827 -0.539307 -1.045712
H -1.467325 -2.719035 -1.108413
H -2.442703 1.930995 0.035705
H -0.045873 2.487449 0.063590
H 2.291402 3.104894 -0.171447

H 2.035623 -2.602433 -1.301238
H 5.475746 0.555214 -1.558877
H 3.723546 0.152564 2.075884
H 5.467177 0.198261 1.829652
H 5.810747 -3.446405 1.582865
H 5.937142 -2.463878 0.104370
H 6.699476 -1.914442 1.629990
H 4.239020 4.295203 -0.866103
H 4.076707 4.246681 0.930728
H 5.658078 4.492061 0.173772
H -4.453591 2.086054 -1.539907
H -6.070090 1.848553 -0.937328
H -4.813669 2.414172 0.171748
H -5.140661 -1.625112 0.261930
H -7.300363 -1.500126 0.774761
H -7.380655 1.553031 0.787203
H -8.609846 0.449024 1.361100
H -5.802478 0.734535 2.547247
H -7.328093 1.278586 3.252349
H -7.011590 -0.454456 3.074722
H -8.865860 -0.654036 -0.874476
H -7.468632 -1.364917 -1.690710
H -7.641017 0.394300 -1.596171
SCF Energy (B3LYP/6-31G**)= -1361.69493172
Number of imaginary frequencies = 0

1b_c224
MMFF Geometry
C -1.467180 -2.206081 -0.657252
C -2.344688 0.323309 -0.187060
N -2.802967 -2.007842 -0.543919
C -0.498804 -1.202672 -0.551426
C -0.965040 0.103802 -0.310579
C -3.226893 -0.749524 -0.300204
C -0.041496 1.160860 -0.208856
C 1.336420 0.934015 -0.326036
C 1.791304 -0.368945 -0.547501
C 0.885924 -1.430600 -0.679114
C 3.245387 -0.582273 -0.677129
C 4.201207 0.475523 -0.092353
C 3.599654 1.873719 -0.171573
C 2.269914 2.064304 -0.270000
O 1.305416 -2.712604 -0.939385
O 3.667234 -1.595277 -1.242428
O 5.396616 0.441801 -0.893501
C 4.591614 0.176042 1.356967
C 4.816541 -1.296175 1.603605
C 6.183018 -1.857846 1.316463
O 3.890799 -2.012327 1.987657
O 4.541150 2.883327 -0.081494
C 4.114758 4.231685 -0.228952
C -4.695441 -0.574319 -0.187346
C -5.478488 -1.317731 -1.233484
C -5.212249 0.181645 0.805460
C -6.656914 0.479543 1.151066
C -7.356604 1.432292 0.163926
C -6.650226 2.774432 0.015957
C -7.489606 -0.781949 1.398971
H -1.181924 -3.239312 -0.844225
H -2.730039 1.324186 -0.018446
H -0.408818 2.172182 -0.037887
H 1.831142 3.055526 -0.300393
H 2.278377 -2.708673 -1.078668
H 5.352053 -0.369175 -1.438571
H 3.803195 0.490114 2.054011
H 5.502199 0.713941 1.648166
H 6.245983 -2.878979 1.703049
H 6.361170 -1.874083 0.239514
H 6.943826 -1.253223 1.816271
H 3.639562 4.388739 -1.202654
H 3.443752 4.519741 0.586509
H 5.000129 4.872286 -0.178229
H -5.517350 -2.386906 -0.999383
H -6.503785 -0.958600 -1.332358
H -5.018998 -1.196343 -2.221468
H -4.519034 0.638219 1.513780
H -6.625313 1.000769 2.119474
H -7.453020 0.972216 -0.825048
H -8.378412 1.621417 0.516708
H -5.658387 2.659999 -0.431836
H -7.233459 3.435241 -0.633458
H -6.537896 3.267966 0.986525
H -8.445249 -0.518595 1.866273
H -6.970941 -1.470411 2.075249
H -7.720387 -1.321039 0.476437
SCF Energy (B3LYP/6-31G**)= -1361.69930141
Number of imaginary frequencies = 0

1b_c225
MMFF Geometry
C -1.984128 -1.775509 -0.200819
C -2.208167 0.931159 -0.159828
N -3.222709 -1.238612 -0.088002
C -0.801719 -1.037933 -0.297564
C -0.929665 0.362579 -0.277415
C -3.330897 0.109902 -0.066585
C 0.223645 1.162219 -0.382753
C 1.498395 0.589248 -0.491192
C 1.618401 -0.802045 -0.495665
C 0.478716 -1.615235 -0.415727
C 2.970963 -1.389691 -0.591478
C 4.193084 -0.507661 -0.273313
C 3.922841 0.957913 -0.578677
C 2.672209 1.449723 -0.654774
O 0.565087 -2.986517 -0.445577
O 3.091295 -2.586692 -0.871827
O 5.249172 -0.992022 -1.119532
C 4.566933 -0.779024 1.188267
C 5.608498 0.167165 1.743234
C 7.036946 -0.015158 1.299989
O 5.293770 1.047168 2.547521
O 5.075015 1.706511 -0.738126
C 4.963776 3.112458 -0.918345
C -4.708840 0.664070 0.077622
C -4.825663 1.990390 0.775404
C -5.756043 -0.055261 -0.384114
C -7.238679 0.251854 -0.394515
C -7.917656 0.104192 0.979754
C -7.760030 -1.284810 1.586303
C -7.577723 1.596989 -1.044633
H -1.967560 -2.863236 -0.210882
H -2.318646 2.010660 -0.156024
H 0.120272 2.247021 -0.380574
H 2.478800 2.498304 -0.852818
H 1.489667 -3.242011 -0.662875
H 5.091903 -1.948763 -1.247537
H 4.950316 -1.799610 1.314411
H 3.683089 -0.700658 1.835459
H 7.691458 0.603763 1.920496
H 7.330840 -1.060139 1.426730
H 7.155341 0.285137 0.257776
H 4.437075 3.346413 -1.848931
H 4.471288 3.579493 -0.059384
H 5.975111 3.523149 -0.991300
H -4.582623 2.809524 0.090690
H -5.824302 2.172544 1.175569
H -4.149817 2.041641 1.636899
H -5.536869 -1.021961 -0.844873
H -7.689869 -0.506631 -1.051522
H -7.532949 0.845410 1.688325
H -8.990176 0.309164 0.870365
H -6.713599 -1.506952 1.816469
H -8.327011 -1.352360 2.520402
H -8.136837 -2.055192 0.906067
H -8.652661 1.656650 -1.249758
H -7.053370 1.717865 -1.998943
H -7.325808 2.448270 -0.406943
SCF Energy (B3LYP/6-31G**)= -1361.70380045
Number of imaginary frequencies = 0

1b_c226
MMFF Geometry

C 2.087886 1.620215 -0.159952
C 2.339317 -1.076770 0.021542
N 3.335798 1.098469 -0.079740
C 0.908367 0.871480 -0.154188
C 1.051081 -0.522733 -0.058570
C 3.458701 -0.245916 0.008848
C -0.099329 -1.331153 -0.045218
C -1.384937 -0.774061 -0.124290
C -1.525907 0.613276 -0.218316
C -0.383631 1.432738 -0.236503
C -2.886141 1.203543 -0.294155
C -4.127770 0.290210 -0.209521
C -3.792978 -1.185098 -0.180204
C -2.543864 -1.660336 -0.108540
O -0.472047 2.802238 -0.330437
O -2.983750 2.429626 -0.417581
O -4.831404 0.566477 -1.425593
C -4.910273 0.723730 1.043140
C -6.375069 0.359785 0.992371
C -6.891905 -0.598548 2.031777
O -7.128331 0.882096 0.168930
O -4.844329 -2.088498 -0.088628
C -5.509711 -2.269359 -1.341186
C 4.849009 -0.783118 0.076451
C 5.061483 -2.185650 -0.426154
C 5.840862 0.005159 0.547387
C 7.305496 -0.331417 0.718579
C 8.193701 0.520875 -0.206574
C 7.961955 0.242321 -1.686461
C 7.704544 -0.105693 2.180932
H 2.060907 2.705425 -0.232138
H 2.456201 -2.152124 0.106369
H 0.013475 -2.412766 0.026604
H -2.376897 -2.731835 -0.039387
H -1.419341 3.051025 -0.430931
H -5.341436 1.389262 -1.292219
H -4.889871 1.814239 1.170979
H -4.455823 0.312291 1.953005
H -7.898715 -0.926800 1.759299
H -6.246733 -1.477459 2.091618
H -6.925405 -0.096139 3.001511
H -6.255261 -1.484979 -1.496388
H -4.804751 -2.306453 -2.179112
H -6.038646 -3.226275 -1.300298
H 4.501638 -2.358010 -1.352633
H 6.105043 -2.399070 -0.669970
H 4.736106 -2.916077 0.321864
H 5.586411 1.019353 0.863370
H 7.496462 -1.386825 0.501209
H 8.032025 1.589114 -0.014086
H 9.248109 0.317541 0.018888
H 6.951515 0.525223 -1.996474
H 8.668625 0.819345 -2.291582
H 8.109957 -0.818218 -1.913480
H 8.752368 -0.381176 2.342813
H 7.092695 -0.717490 2.853414
H 7.584334 0.943205 2.475211
SCF Energy (B3LYP/6-31G**)= -1361.70450939
Number of imaginary frequencies = 0

1b_c227
MMFF Geometry
C 1.680400 2.288518 -0.209142
C 2.307673 -0.319647 -0.640655
N 2.990561 1.958390 -0.309214
C 0.617833 1.384513 -0.310672
C 0.955412 0.038352 -0.536215
C 3.291972 0.658364 -0.515195
C -0.068292 -0.918034 -0.652685
C -1.419417 -0.556419 -0.546525
C -1.754257 0.781707 -0.320180
C -0.740952 1.748611 -0.203273
C -3.184055 1.159460 -0.195024
C -4.281666 0.089190 -0.350670
C -3.748315 -1.301156 -0.606638
C -2.442650 -1.590321 -0.662079
O -1.023419 3.075710 0.021425
O -3.469146 2.345917 0.000890
O -4.975477 0.522133 -1.543187
C -5.270876 0.176234 0.820901
C -4.800243 -0.479433 2.102439
C -3.653016 0.152474 2.845551
O -5.345622 -1.493382 2.538987
O -4.648162 -2.357267 -0.646745
C -5.505493 -2.320365 -1.786236
C 4.736219 0.341748 -0.621472
C 5.533213 1.341044 -1.416114
C 5.246181 -0.748269 -0.009068
C 6.692919 -1.191610 0.028061
C 7.200961 -1.336880 1.475725
C 7.227651 -0.016668 2.236259
C 6.828575 -2.524414 -0.712649
H 1.498579 3.347473 -0.038076
H 2.589847 -1.350529 -0.831823
H 0.196586 -1.960785 -0.827945
H -2.126322 -2.621765 -0.794948
H -1.999142 3.194960 0.046557
H -5.242656 1.449666 -1.394618
H -6.222599 -0.294057 0.538963
H -5.535252 1.215789 1.051895
H -3.683066 -0.162482 3.892713
H -2.705169 -0.163443 2.407210
H -3.742209 1.241254 2.818341
H -6.331131 -1.621603 -1.624935
H -4.955190 -2.070201 -2.699863
H -5.935172 -3.318639 -1.912502
H 5.736665 2.234735 -0.816761
H 6.488780 0.941428 -1.764372
H 4.987990 1.645063 -2.317419
H 4.577733 -1.388055 0.567748
H 7.340902 -0.467978 -0.476025
H 6.583422 -2.056299 2.028295
H 8.219628 -1.744376 1.460534
H 6.221116 0.390347 2.372244
H 7.663253 -0.164968 3.229602
H 7.833372 0.727149 1.708893
H 7.873014 -2.854301 -0.728144
H 6.494546 -2.431176 -1.752155
H 6.234476 -3.312819 -0.236535
SCF Energy (B3LYP/6-31G**)= -1361.69852454
Number of imaginary frequencies = 0

1b_c228
MMFF Geometry
C -1.577093 -2.395983 -0.210692
C -2.288255 0.226560 -0.209243
N -2.896753 -2.097249 -0.132385
C -0.546294 -1.453382 -0.294295
C -0.927124 -0.100419 -0.295757
C -3.236026 -0.790795 -0.123012
C 0.063408 0.893346 -0.386597
C 1.426143 0.564535 -0.471790
C 1.803559 -0.781353 -0.462512
C 0.821905 -1.785693 -0.378537
C 3.242030 -1.133705 -0.549378
C 4.295475 -0.005411 -0.617697
C 3.728804 1.405157 -0.640133
C 2.408266 1.646315 -0.573250
O 1.146652 -3.122963 -0.381872
O 3.546156 -2.330548 -0.586968
O 5.004602 -0.197404 -1.861953
C 5.333324 -0.224379 0.492091
C 4.784339 -0.041338 1.889830
C 5.797103 -0.019575 3.004837
O 3.580880 0.055558 2.126419
O 4.707524 2.378780 -0.742034
C 4.315745 3.746546 -0.742737
C -4.687287 -0.504591 -0.035325
C -5.527765 -1.303636 -0.989336
C -5.138218 0.403566 0.856642
C -6.543184 0.843076 1.221060
C -6.883201 2.223432 0.624575

C -6.958589 2.272737 -0.895945
C -7.670057 -0.173114 1.019922
H -1.358465 -3.461793 -0.205158
H -2.608682 1.263751 -0.220656
H -0.238627 1.940190 -0.386722
H 2.010678 2.655030 -0.584539
H 2.126263 -3.204820 -0.442667
H 4.439394 0.146247 -2.574040
H 6.175735 0.466517 0.360108
H 5.754703 -1.235817 0.430201
H 5.288408 0.120530 3.962661
H 6.338120 -0.968705 3.026361
H 6.493002 0.808960 2.851864
H 3.686199 3.972274 -1.609340
H 3.808719 4.008590 0.191436
H 5.221555 4.355515 -0.816746
H -4.951135 -1.626029 -1.864517
H -5.926004 -2.197748 -0.499633
H -6.353119 -0.710761 -1.390516
H -4.394713 0.900830 1.483732
H -6.505672 0.990981 2.311325
H -7.843278 2.563243 1.033070
H -6.134251 2.954211 0.956619
H -7.735485 1.607723 -1.283049
H -7.202357 3.289252 -1.222250
H -6.004586 1.999209 -1.355909
H -8.570236 0.157274 1.551231
H -7.394594 -1.152497 1.425647
H -7.952863 -0.300162 -0.026891
SCF Energy (B3LYP/6-31G**)= -1361.69876538
Number of imaginary frequencies = 0

1b_c229
MMFF Geometry
C -2.060318 1.748217 -0.068517
C -2.364521 -0.925879 -0.424103
N -3.312882 1.266660 -0.255997
C -0.901155 0.968656 -0.045398
C -1.071223 -0.413541 -0.229642
C -3.462024 -0.066270 -0.432955
C 0.057292 -1.252196 -0.213008
C 1.348173 -0.736568 -0.020245
C 1.516647 0.639151 0.159850
C 0.396021 1.488120 0.150922
C 2.883013 1.185981 0.355899
C 4.104752 0.244038 0.300436
C 3.736500 -1.217549 0.167637
C 2.483521 -1.652939 -0.010562
O 0.511461 2.847320 0.328181
O 3.002347 2.400365 0.553564
O 4.733658 0.434457 1.572617
C 4.978203 0.722318 -0.873329
C 6.427181 0.315325 -0.748840
C 6.987516 -0.600080 -1.804277
O 7.137361 0.771551 0.148804
O 4.769112 -2.143899 0.093865
C 5.345787 -2.410748 1.374725
C -4.854376 -0.556456 -0.649269
C -5.013517 -1.839638 -1.419099
C -5.892477 0.168836 -0.177134
C -7.369137 -0.147917 -0.257978
C -8.029777 -0.112287 1.134195
C -7.497339 -1.184313 2.077303
C -8.050201 0.858620 -1.188438
H -2.011795 2.826425 0.068358
H -2.504375 -1.994113 -0.552927
H -0.077068 -2.324775 -0.351895
H 2.295202 -2.714061 -0.149588
H 1.456029 3.063628 0.502426
H 5.270900 1.248905 1.518417
H 4.992873 1.818273 -0.941426
H 4.574601 0.373371 -1.831975
H 7.965811 -0.970743 -1.486145
H 6.326220 -1.455971 -1.954102
H 7.097130 -0.047525 -2.740540
H 6.098600 -1.657240 1.621065
H 4.586417 -2.473034 2.162092
H 5.852506 -3.378624 1.314851
H -4.811979 -2.702142 -0.775363
H -6.014104 -1.962287 -1.841231
H -4.328155 -1.871160 -2.273948
H -5.673623 1.101317 0.347915
H -7.536011 -1.147358 -0.671598
H -7.893497 0.872857 1.598075
H -9.111394 -0.259832 1.023219
H -6.438516 -1.029852 2.306035
H -8.048818 -1.159474 3.022674
H -7.615527 -2.182109 1.642901
H -9.117530 0.633269 -1.288117
H -7.609311 0.826522 -2.191140
H -7.957178 1.883489 -0.811343
SCF Energy (B3LYP/6-31G**)= -1361.70463909
Number of imaginary frequencies = 0

1b_c230
MMFF Geometry
C -1.692522 -1.478370 -0.701359
C -2.130045 1.065337 0.145417
N -2.971389 -1.090602 -0.476671
C -0.571218 -0.662865 -0.531415
C -0.809355 0.653072 -0.094447
C -3.185344 0.175980 -0.052771
C 0.277860 1.529353 0.082924
C 1.594357 1.109479 -0.151040
C 1.823157 -0.205377 -0.567191
C 0.751173 -1.083917 -0.776608
C 3.219283 -0.627379 -0.808873
C 4.345255 0.173414 -0.113121
C 3.981532 1.654041 -0.020395
C 2.699459 2.065325 -0.022861
O 0.944413 -2.366997 -1.228131
O 3.421259 -1.607460 -1.527818
O 5.547367 0.038888 -0.882938
C 4.640514 -0.370552 1.286489
C 4.633947 -1.880923 1.328618
C 5.849513 -2.610096 0.821923
O 3.641187 -2.485718 1.737838
O 5.082727 2.483070 0.087052
C 4.875178 3.885291 0.210608
C -4.604156 0.560407 0.203505
C -4.833573 1.593863 1.270538
C -5.585325 -0.053059 -0.495167
C -7.087191 0.136291 -0.465316
C -7.770370 -0.478385 0.770346
C -7.512076 -1.972289 0.924007
C -7.522588 1.589256 -0.680537
H -1.588699 -2.508509 -1.035771
H -2.326245 2.081599 0.470848
H 0.087730 2.552470 0.405301
H 2.426581 3.111427 0.063479
H 1.898713 -2.491333 -1.433585
H 5.427416 0.565828 -1.692094
H 3.887835 -0.032563 2.011135
H 5.613552 -0.026060 1.657905
H 5.807610 -3.654243 1.144704
H 5.879499 -2.575331 -0.268805
H 6.753261 -2.158963 1.239081
H 4.369215 4.284724 -0.674149
H 4.315729 4.118538 1.122188
H 5.855464 4.365289 0.284187
H -4.173435 1.424990 2.129184
H -4.648795 2.599840 0.879718
H -5.847919 1.570651 1.672055
H -5.284383 -0.812656 -1.221440
H -7.468480 -0.410617 -1.340544
H -7.454268 0.031425 1.686686
H -8.854155 -0.325850 0.690231
H -6.454523 -2.180444 1.112678
H -8.084137 -2.366403 1.770170
H -7.818316 -2.517956 0.025868
H -8.596049 1.633355 -0.897024
H -6.996714 2.037475 -1.530726

H -7.347179 2.215876 0.197743
SCF Energy (B3LYP/6-31G**)= -1361.69505396
Number of imaginary frequencies = 0

1b_c231
MMFF Geometry
C -1.709515 -2.097282 -0.285824
C -2.272961 0.489351 0.324673
N -3.002100 -1.769484 -0.043745
C -0.635910 -1.201196 -0.248552
C -0.940266 0.133917 0.069252
C -3.271122 -0.480001 0.252290
C 0.096569 1.081655 0.125671
C 1.428804 0.723567 -0.132438
C 1.731848 -0.606145 -0.444211
C 0.703281 -1.563339 -0.505015
C 3.140298 -0.986944 -0.726365
C 4.246103 0.095844 -0.671944
C 3.748280 1.482010 -0.315910
C 2.464108 1.751185 -0.052074
O 0.951506 -2.879393 -0.820926
O 3.374846 -2.153685 -1.057292
O 4.798645 0.158673 -2.004364
C 5.378576 -0.388154 0.243598
C 4.981462 -0.464994 1.701819
C 6.106309 -0.471405 2.703116
O 3.809708 -0.567577 2.063175
O 4.685152 2.484731 -0.094832
C 5.340911 2.913919 -1.286899
C -4.695717 -0.164459 0.518177
C -5.391270 -1.176405 1.385560
C -5.259012 0.936908 -0.024164
C -6.685901 1.439820 0.056252
C -7.778616 0.440057 -0.369317
C -7.578848 -0.111160 -1.775540
C -6.988074 2.048411 1.428091
H -1.550916 -3.147737 -0.520565
H -2.528193 1.510234 0.591559
H -0.142496 2.114237 0.378705
H 2.176324 2.749929 0.264662
H 1.920059 -2.989937 -0.958835
H 4.057655 0.301326 -2.618655
H 6.243758 0.280465 0.152849
H 5.730102 -1.382345 -0.059247
H 5.698184 -0.527000 3.716102
H 6.745584 -1.340369 2.529373
H 6.686357 0.449929 2.608735
H 6.157893 2.233158 -1.541878
H 4.640286 3.012631 -2.123263
H 5.776971 3.898226 -1.091819
H -4.748067 -1.483023 2.218827
H -6.304383 -0.788392 1.839649
H -5.652618 -2.067415 0.804917
H -4.636465 1.569701 -0.658595
H -6.744719 2.272057 -0.661008
H -7.855279 -0.394020 0.334652
H -8.750475 0.949536 -0.339773
H -6.673657 -0.721664 -1.844668
H -8.427717 -0.744103 -2.053766
H -7.507713 0.698932 -2.508274
H -7.959148 2.555613 1.416964
H -6.231147 2.791162 1.704117
H -7.020367 1.292308 2.217889
SCF Energy (B3LYP/6-31G**)= -1361.69517730
Number of imaginary frequencies = 0

1b_c232
MMFF Geometry
C -1.730846 -1.456575 -0.205404
C -2.103678 1.171430 0.372361
N -2.985270 -1.038493 0.091391
C -0.603707 -0.632775 -0.238665
C -0.808369 0.727427 0.061155
C -3.167924 0.270527 0.380600
C 0.284031 1.614629 0.030680
C 1.573346 1.161011 -0.271141
C 1.771284 -0.195902 -0.549859
C 0.691795 -1.087541 -0.556711
C 3.147317 -0.646028 -0.860856
C 4.320104 0.208063 -0.312129
C 3.953175 1.684788 -0.416592
C 2.681206 2.109872 -0.389703
O 0.851627 -2.413919 -0.875272
O 3.301940 -1.686551 -1.501367
O 5.495329 -0.036157 -1.089716
C 4.636198 -0.184191 1.135917
C 4.567528 -1.678719 1.356646
C 5.729944 -2.517226 0.895995
O 3.569446 -2.183984 1.872350
O 4.944603 2.610992 -0.708942
C 5.930042 2.739624 0.312324
C -4.560480 0.688105 0.718912
C -4.708295 1.890222 1.612127
C -5.599443 -0.029360 0.235942
C -7.080478 0.206195 0.433410
C -7.787665 0.559166 -0.891759
C -7.803150 -0.536317 -1.950968
C -7.711848 -1.004368 1.125190
H -1.652837 -2.519391 -0.424575
H -2.272941 2.220449 0.591469
H 0.120771 2.671267 0.239495
H 2.449409 3.164683 -0.508472
H 1.780327 -2.562566 -1.162476
H 5.302963 0.277135 -1.992700
H 3.923633 0.271997 1.835591
H 5.634941 0.144486 1.444210
H 5.645575 -3.520971 1.322076
H 5.729928 -2.592011 -0.193112
H 6.666581 -2.074607 1.243979
H 5.472088 2.806502 1.305070
H 6.643886 1.912601 0.270315
H 6.482138 3.666414 0.130145
H -4.562315 2.813420 1.041645
H -5.685793 1.948765 2.096080
H -3.977147 1.862777 2.428387
H -5.371743 -0.898020 -0.385122
H -7.243339 1.062955 1.094432
H -8.826651 0.835947 -0.671502
H -7.317585 1.452858 -1.322209
H -8.323659 -1.430803 -1.597189
H -8.329751 -0.180860 -2.842999
H -6.791996 -0.818697 -2.257244
H -8.791368 -0.863691 1.245050
H -7.282493 -1.145142 2.123789
H -7.549811 -1.931420 0.564874
SCF Energy (B3LYP/6-31G**)= -1361.69265463
Number of imaginary frequencies = 0

1b_c233
MMFF Geometry
C -1.637231 -2.346995 -0.356185
C -2.352039 0.269043 -0.195930
N -2.958740 -2.053213 -0.290944
C -0.605724 -1.401947 -0.351022
C -0.988643 -0.051712 -0.271402
C -3.301650 -0.750650 -0.199958
C 0.001798 0.945986 -0.271173
C 1.365236 0.622128 -0.347258
C 1.746320 -0.722096 -0.418122
C 0.764658 -1.729188 -0.424032
C 3.188800 -1.068084 -0.506966
C 4.243176 0.066058 -0.521481
C 3.661930 1.462669 -0.433974
C 2.348961 1.702142 -0.334354
O 1.090997 -3.063190 -0.509812
O 3.497685 -2.257904 -0.628346
O 4.921583 -0.046292 -1.791035
C 5.296995 -0.213804 0.558376
C 4.761275 -0.088728 1.968131
C 5.781804 0.111825 3.057276
O 3.563923 -0.192942 2.231927
O 4.534855 2.534428 -0.287751

C 5.288321 2.810528 -1.467285
C -4.756556 -0.469976 -0.131575
C -5.577841 -1.278367 -1.096946
C -5.230943 0.427992 0.758844
C -6.655575 0.856468 1.044900
C -7.283160 1.730504 -0.056890
C -6.489017 2.998033 -0.348293
C -7.571274 -0.311373 1.424154
H -1.418058 -3.410967 -0.416588
H -2.671123 1.305627 -0.147571
H -0.298960 1.991296 -0.205761
H 1.994725 2.721266 -0.205780
H 2.071896 -3.143392 -0.537153
H 4.240024 -0.035888 -2.485396
H 6.141530 0.477219 0.444039
H 5.713216 -1.222905 0.447999
H 5.278255 0.190046 4.024807
H 6.464724 -0.740854 3.080012
H 6.336118 1.035728 2.874634
H 6.150992 2.142047 -1.536873
H 4.670148 2.744446 -2.369294
H 5.666438 3.834270 -1.389547
H -5.688142 -2.308456 -0.741700
H -6.576766 -0.868562 -1.252509
H -5.103473 -1.302079 -2.085070
H -4.514900 0.918617 1.420313
H -6.597687 1.483994 1.946756
H -7.401523 1.165323 -0.987266
H -8.293185 2.024764 0.255486
H -5.503216 2.768642 -0.764147
H -7.022305 3.614650 -1.079015
H -6.352437 3.593199 0.560177
H -8.511420 0.065531 1.842539
H -7.104518 -0.948052 2.183842
H -7.829252 -0.938540 0.566797
SCF Energy (B3LYP/6-31G**)= -1361.69531040
Number of imaginary frequencies = 0

1b_c234
MMFF Geometry
C 1.702323 2.453764 0.105765
C 2.328409 -0.157837 -0.314172
N 3.010727 2.103009 0.126244
C 0.641591 1.569484 -0.115176
C 0.978040 0.220498 -0.334424
C 3.309974 0.801591 -0.074226
C -0.043488 -0.716275 -0.576757
C -1.391999 -0.334037 -0.586914
C -1.719764 1.003325 -0.353417
C -0.713719 1.955740 -0.134516
C -3.147312 1.385574 -0.348997
C -4.214711 0.286694 -0.187974
C -3.734940 -1.047607 -0.738913
C -2.426883 -1.323066 -0.896496
O -1.006356 3.282989 0.069595
O -3.453172 2.580246 -0.420661
O -5.352097 0.740711 -0.940494
C -4.588946 0.246802 1.297943
C -5.463535 -0.926306 1.681432
C -6.913351 -0.890592 1.272850
O -5.001515 -1.871293 2.324760
O -4.765083 -1.925200 -1.025214
C -4.448179 -3.246948 -1.442500
C 4.753192 0.461980 -0.047339
C 5.624010 1.437387 -0.789478
C 5.181960 -0.631136 0.620339
C 6.591494 -1.147925 0.835547
C 7.118563 -1.867460 -0.419131
C 8.441979 -2.586025 -0.192525
C 7.553540 -0.093456 1.391007
H 1.520382 3.512328 0.279134
H 2.613742 -1.189286 -0.497344
H 0.222410 -1.757066 -0.759935
H -2.082974 -2.281192 -1.270533
H -1.964192 3.427070 -0.101175
H -5.343774 1.717927 -0.902533
H -5.117351 1.161277 1.596305
H -3.687678 0.194770 1.923393
H -7.452990 -1.696634 1.778386
H -7.357075 0.061620 1.574220
H -7.010361 -1.026281 0.194622
H -3.915596 -3.237434 -2.398745
H -3.869855 -3.771649 -0.675225
H -5.387749 -3.788714 -1.585625
H 5.814202 2.326479 -0.179200
H 6.585076 1.007757 -1.077971
H 5.146695 1.755434 -1.723921
H 4.438237 -1.242770 1.133528
H 6.501682 -1.907587 1.626200
H 6.378764 -2.608029 -0.749767
H 7.242607 -1.161247 -1.247719
H 8.369643 -3.289656 0.642707
H 8.719836 -3.151303 -1.088002
H 9.250611 -1.879438 0.016545
H 8.426308 -0.573291 1.847339
H 7.074382 0.509682 2.170183
H 7.926221 0.581544 0.615960
SCF Energy (B3LYP/6-31G**)= -1361.70222573
Number of imaginary frequencies = 0

1b_c235
MMFF Geometry
C -1.573353 -1.939113 -0.520394
C -2.303927 0.517389 0.385710
N -2.885337 -1.722597 -0.259234
C -0.559261 -0.989695 -0.359412
C -0.949370 0.278550 0.111827
C -3.237871 -0.496724 0.183319
C 0.023120 1.279162 0.293029
C 1.375170 1.032333 0.022109
C 1.757657 -0.234656 -0.432392
C 0.799799 -1.236572 -0.640061
C 3.187904 -0.471384 -0.723167
C 4.239809 0.467497 -0.099385
C 3.672381 1.862537 0.081099
C 2.356301 2.107740 0.174673
O 1.141142 -2.477409 -1.121152
O 3.517176 -1.408630 -1.456566
O 5.337214 0.530982 -1.026962
C 4.769077 -0.044778 1.242118
C 4.955857 -1.542613 1.259173
C 6.276191 -2.091700 0.789892
O 4.035688 -2.282753 1.608966
O 4.588256 2.874233 0.327681
C 4.831805 3.646977 -0.848279
C -4.681700 -0.304073 0.462406
C -5.318680 -1.453082 1.193368
C -5.309486 0.816575 0.044705
C -6.766215 1.216467 0.161192
C -7.789347 0.208038 -0.396417
C -7.543078 -0.159078 -1.854424
C -7.118031 1.638129 1.590199
H -1.347365 -2.941873 -0.877113
H -2.626722 1.481618 0.766138
H -0.284082 2.262459 0.647788
H 1.998683 3.111043 0.387189
H 2.099895 -2.484192 -1.337086
H 5.258602 -0.252318 -1.608770
H 4.073861 0.188326 2.059741
H 5.727141 0.422743 1.500595
H 6.318588 -3.165152 0.994850
H 6.386509 -1.931039 -0.284279
H 7.091590 -1.604863 1.330548
H 5.199431 3.025688 -1.670818
H 3.927835 4.183419 -1.155829
H 5.601362 4.387012 -0.609938
H -4.664101 -1.815949 1.994561
H -6.258243 -1.179150 1.675767
H -5.518190 -2.283334 0.507639
H -4.723570 1.557425 -0.501504
H -6.871999 2.122568 -0.453895
H -7.818547 -0.706746 0.203127

H -8.791932 0.648843 -0.322484
H -6.600249 -0.699461 -1.980614
H -8.347497 -0.806254 -2.218671
H -7.517622 0.735005 -2.485438
H -8.119340 2.081565 1.623499
H -6.412334 2.388643 1.963733
H -7.108657 0.793210 2.284847
SCF Energy (B3LYP/6-31G**)= -1361.69420777
Number of imaginary frequencies = 0

1b_c236
MMFF Geometry
C -1.374374 -2.180761 -0.673255
C -2.243957 0.359717 -0.249737
N -2.710151 -1.974456 -0.574292
C -0.402316 -1.180183 -0.575414
C -0.864337 0.131986 -0.358882
C -3.130227 -0.710584 -0.353599
C 0.063326 1.186307 -0.267521
C 1.440972 0.950810 -0.369756
C 1.892943 -0.357646 -0.565765
C 0.982314 -1.416471 -0.688376
C 3.350210 -0.583275 -0.671660
C 4.290930 0.478380 -0.054326
C 3.704355 1.880140 -0.209569
C 2.378756 2.078129 -0.337774
O 1.397509 -2.704266 -0.926746
O 3.747503 -1.612723 -1.219978
O 5.551892 0.425766 -0.734774
C 4.562616 0.197222 1.425238
C 4.779424 -1.272694 1.699836
C 6.123948 -1.864931 1.371888
O 3.861541 -1.961922 2.148466
O 4.658720 2.879993 -0.178563
C 4.235477 4.234451 -0.283053
C -4.598697 -0.528981 -0.254434
C -5.378965 -1.287716 -1.291748
C -5.123492 0.246840 0.718825
C -6.573736 0.536598 1.054680
C -7.174022 1.571055 0.085499
C -8.557386 2.055113 0.499154
C -7.429155 -0.723322 1.218483
H -1.092229 -3.218152 -0.841196
H -2.625920 1.364693 -0.099338
H -0.300838 2.201977 -0.117000
H 1.947050 3.068998 -0.427676
H 2.372020 -2.703820 -1.063578
H 5.413490 0.805479 -1.619902
H 3.717511 0.512751 2.051425
H 5.443710 0.742234 1.785877
H 6.215967 -2.844233 1.850100
H 6.226937 -1.984853 0.291699
H 6.917357 -1.219895 1.757545
H 3.740220 4.416032 -1.242265
H 3.583550 4.505639 0.553496
H 5.125135 4.869411 -0.237138
H -5.481086 -2.339524 -1.004745
H -6.377920 -0.877151 -1.449815
H -4.879937 -1.242601 -2.266992
H -4.436710 0.723464 1.420011
H -6.550341 1.005865 2.049428
H -6.508972 2.442547 0.028257
H -7.236823 1.161299 -0.928814
H -8.545793 2.455609 1.517687
H -8.892771 2.851099 -0.173562
H -9.294945 1.248571 0.450573
H -8.340142 -0.497386 1.783512
H -6.890620 -1.499163 1.773941
H -7.741817 -1.143587 0.258881
SCF Energy (B3LYP/6-31G**)= -1361.69362174
Number of imaginary frequencies = 0

1b_c237
MMFF Geometry
C 1.758218 -1.683811 0.141538
C 2.312511 0.945460 -0.252357
N 3.058426 -1.304213 0.103127
C 0.670164 -0.819988 -0.007087
C 0.969250 0.538712 -0.210098
C 3.330460 0.006333 -0.093330
C -0.082664 1.458612 -0.370154
C -1.422037 1.047197 -0.320103
C -1.716342 -0.301789 -0.109918
C -0.677182 -1.235175 0.033076
C -3.136705 -0.725263 -0.075563
C -4.237616 0.339968 0.165361
C -3.766401 1.717376 -0.282734
C -2.485577 2.023956 -0.527386
O -0.926093 -2.575440 0.214076
O -3.379104 -1.929056 -0.200620
O -4.482478 0.441418 1.570398
C -5.508072 -0.073221 -0.607182
C -6.138219 -1.337400 -0.056531
C -6.787414 -2.267560 -1.044823
O -6.164757 -1.561443 1.155690
O -4.730845 2.691631 -0.516548
C -5.240280 3.226856 0.707386
C 4.772118 0.385912 -0.145437
C 5.123601 1.561685 -1.013531
C 5.670723 -0.351919 0.544111
C 7.169022 -0.188783 0.702050
C 7.919378 -0.739361 -0.524069
C 9.429553 -0.792113 -0.335254
C 7.598030 1.226278 1.102037
H 1.607907 -2.749208 0.302433
H 2.553286 1.993933 -0.394706
H 0.149512 2.510412 -0.534446
H -2.218589 3.021358 -0.864331
H -1.893773 -2.729587 0.125497
H -4.963564 -0.373739 1.836334
H -5.270912 -0.227659 -1.666875
H -6.291767 0.690609 -0.543652
H -7.187562 -3.140200 -0.521347
H -7.605393 -1.749519 -1.551163
H -6.045452 -2.605243 -1.772600
H -5.941190 2.528801 1.174263
H -4.433349 3.487059 1.401295
H -5.789882 4.141807 0.467018
H 4.943932 2.501785 -0.481914
H 6.165631 1.547785 -1.338627
H 4.530684 1.562321 -1.935520
H 5.291099 -1.208271 1.107503
H 7.438591 -0.827055 1.556610
H 7.565309 -1.755871 -0.739499
H 7.699537 -0.138890 -1.414037
H 9.694519 -1.367198 0.557502
H 9.898886 -1.273399 -1.199377
H 9.856860 0.210757 -0.242862
H 8.601863 1.212214 1.540745
H 6.923888 1.647511 1.856042
H 7.630044 1.910318 0.249841
SCF Energy (B3LYP/6-31G**)= -1361.70110310
Number of imaginary frequencies = 0

1b_c238
MMFF Geometry
C 1.909372 2.037050 0.125353
C 2.244213 -0.557880 -0.605254
N 3.172053 1.551783 0.051612
C 0.755205 1.298710 -0.146492
C 0.940083 -0.043935 -0.523992
C 3.335438 0.259057 -0.312495
C -0.183129 -0.839723 -0.817778
C -1.482458 -0.323128 -0.725915
C -1.658524 1.007092 -0.335789
C -0.550643 1.822052 -0.063909
C -3.039407 1.528552 -0.221642
C -4.185491 0.505867 -0.045536
C -3.888242 -0.770202 -0.825575
C -2.628723 -1.152833 -1.105580
O -0.695590 3.141447 0.291963
O -3.200011 2.750455 -0.216384

O -5.392967 1.102044 -0.532862
C -4.350366 0.281546 1.462093
C -5.257932 -0.879958 1.798731
C -6.744604 -0.675831 1.667956
O -4.789173 -1.962826 2.157172
O -5.026495 -1.467886 -1.185887
C -4.880876 -2.794082 -1.681979
C 4.740637 -0.235272 -0.397673
C 5.006868 -1.324229 -1.398962
C 5.683209 0.329897 0.389466
C 7.158474 0.024305 0.540208
C 7.523576 -1.447100 0.817442
C 6.822465 -2.023671 2.041045
C 7.966375 0.589441 -0.630844
H 1.848111 3.081799 0.422636
H 2.397373 -1.595823 -0.882290
H -0.036265 -1.875379 -1.123021
H -2.408366 -2.069479 -1.641790
H -1.637715 3.400315 0.168138
H -5.346289 1.085242 -1.504813
H -4.757878 1.175374 1.951313
H -3.382796 0.084331 1.942179
H -7.268768 -1.486492 2.182219
H -7.031830 0.268396 2.137320
H -7.035019 -0.677540 0.615895
H -4.377315 -2.792607 -2.653739
H -4.348326 -3.425886 -0.963685
H -5.882160 -3.211809 -1.822285
H 4.453321 -1.147561 -2.328445
H 6.056641 -1.382990 -1.691155
H 4.712646 -2.299043 -0.996425
H 5.364192 1.136912 1.054140
H 7.484346 0.584673 1.429259
H 7.315466 -2.083151 -0.048023
H 8.606408 -1.513189 0.985270
H 5.739930 -2.083516 1.895102
H 7.186850 -3.036992 2.238575
H 7.019594 -1.415295 2.929434
H 9.040453 0.508847 -0.430356
H 7.739249 1.649557 -0.790179
H 7.764550 0.058594 -1.565839
SCF Energy (B3LYP/6-31G**)= -1361.69762635
Number of imaginary frequencies = 0

1b_c239
MMFF Geometry
C -1.491205 -2.127020 -0.787134
C -2.377521 0.359263 -0.137967
N -2.828632 -1.937974 -0.677312
C -0.525133 -1.134723 -0.592476
C -0.996203 0.149752 -0.259505
C -3.257057 -0.701806 -0.344130
C -0.075773 1.196015 -0.064888
C 1.303100 0.978387 -0.181839
C 1.765163 -0.304679 -0.495515
C 0.861745 -1.352915 -0.718118
C 3.223557 -0.510775 -0.625454
C 4.180392 0.509947 0.022432
C 3.558205 1.893192 0.036137
C 2.232937 2.097016 -0.019178
O 1.284579 -2.612529 -1.067760
O 3.651364 -1.488067 -1.247238
O 5.363068 0.542891 -0.795455
C 4.586836 0.119943 1.445402
C 4.813561 -1.364577 1.598506
C 6.189386 -1.899459 1.305245
O 3.884399 -2.108937 1.913853
O 4.415540 2.953082 0.290637
C 4.753420 3.643258 -0.913044
C -4.727179 -0.535791 -0.237660
C -5.495764 -1.198466 -1.346848
C -5.257724 0.143379 0.802288
C -6.707036 0.414511 1.150398
C -7.394713 1.438744 0.228558
C -6.687766 2.788199 0.191091
C -7.541674 -0.862026 1.291913
H -1.202590 -3.143153 -1.047958
H -2.765764 1.344512 0.101438
H -0.444886 2.191587 0.179436
H 1.827361 3.099936 0.077448
H 2.260239 -2.601327 -1.185439
H 5.365562 -0.284407 -1.318724
H 3.806766 0.388836 2.170219
H 5.500434 0.639990 1.758710
H 6.241580 -2.952118 1.597286
H 6.402211 -1.816540 0.237698
H 6.932281 -1.344001 1.882972
H 5.218966 2.975377 -1.644431
H 3.869865 4.122054 -1.348478
H 5.473946 4.426410 -0.659686
H -5.536493 -2.282228 -1.194371
H -6.520087 -0.833042 -1.431772
H -5.023634 -1.003035 -2.316837
H -4.574270 0.545690 1.551843
H -6.688566 0.861500 2.155560
H -7.477773 1.054273 -0.793362
H -8.421219 1.600843 0.581155
H -5.690058 2.707719 -0.251019
H -7.263116 3.495939 -0.414419
H -6.588587 3.207414 1.197380
H -8.503591 -0.634509 1.765126
H -7.031217 -1.599358 1.921294
H -7.759867 -1.330282 0.328546
SCF Energy (B3LYP/6-31G**)= -1361.69427151
Number of imaginary frequencies = 0

1b_c240
MMFF Geometry
C 1.963604 1.788584 -0.191983
C 2.174590 -0.919796 -0.197410
N 3.198732 1.243597 -0.079359
C 0.778764 1.058455 -0.311127
C 0.899758 -0.343250 -0.315281
C 3.300274 -0.105561 -0.080607
C -0.256042 -1.135863 -0.445861
C -1.526378 -0.554255 -0.553025
C -1.639937 0.838334 -0.530692
C -0.497447 1.644426 -0.428932
C -2.992261 1.434048 -0.620918
C -4.199230 0.546759 -0.238296
C -3.948865 -0.901088 -0.646113
C -2.704340 -1.400946 -0.757985
O -0.579283 3.016169 -0.437183
O -3.083921 2.620519 -0.941424
O -5.354925 1.049334 -0.918481
C -4.425264 0.732320 1.266679
C -5.405508 -0.259240 1.851476
C -6.873740 -0.030523 1.604612
O -5.008583 -1.230363 2.499707
O -5.112117 -1.615632 -0.866247
C -5.025051 -3.030337 -0.997093
C 4.674318 -0.668879 0.064886
C 4.779417 -2.006179 0.743254
C 5.728334 0.052249 -0.378124
C 7.209498 -0.261959 -0.382360
C 7.879030 -0.137939 0.998861
C 7.723742 1.242754 1.624677
C 7.546841 -1.599041 -1.049747
H 1.951888 2.876425 -0.182491
H 2.280341 -1.999663 -0.211618
H -0.157812 -2.220911 -0.464435
H -2.518285 -2.434933 -1.027392
H -1.500814 3.276248 -0.667578
H -5.277476 0.777369 -1.849717
H -4.798949 1.740185 1.487805
H -3.486167 0.624875 1.825354
H -7.457941 -0.655535 2.286119
H -7.124096 1.015047 1.800756
H -7.129315 -0.292884 0.576498
H -4.490215 -3.304043 -1.911934
H -4.553091 -3.477924 -0.116384
H -6.043065 -3.423817 -1.071733

H 4.538362 -2.813655 0.044145
H 5.773914 -2.198924 1.148765
H 4.096240 -2.067472 1.598287
H 5.517181 1.027115 -0.825267
H 7.669128 0.503889 -1.024812
H 7.485442 -0.887575 1.693570
H 8.951310 -0.346657 0.894312
H 6.676741 1.466728 1.850437
H 8.284130 1.293699 2.563794
H 8.109338 2.021169 0.958661
H 8.622942 -1.660906 -1.248011
H 7.028846 -1.703357 -2.009468
H 7.286283 -2.458348 -0.426456
SCF Energy (B3LYP/6-31G**)= -1361.69772509
Number of imaginary frequencies = 0

1b_c241
MMFF Geometry
C -2.013687 -1.605005 -0.304966
C -2.302433 1.075289 0.020971
N -3.266519 -1.109262 -0.162942
C -0.847002 -0.836849 -0.293987
C -1.008277 0.548862 -0.123574
C -3.408225 0.226496 -0.002123
C 0.129287 1.376707 -0.104591
C 1.420837 0.846398 -0.243536
C 1.574997 -0.531782 -0.406647
C 0.449553 -1.370510 -0.443036
C 2.939034 -1.083941 -0.551578
C 4.153895 -0.199893 -0.213609
C 3.835662 1.286744 -0.232390
C 2.572769 1.749733 -0.243881
O 0.563075 -2.729257 -0.618730
O 3.068697 -2.257795 -0.913187
O 5.090854 -0.472749 -1.267054
C 4.701558 -0.630298 1.156722
C 6.188070 -0.901342 1.158105
C 7.127639 0.273899 1.173695
O 6.613967 -2.057531 1.121019
O 4.965233 2.085233 -0.205335
C 4.819510 3.486190 -0.403864
C -4.804305 0.735094 0.132398
C -5.055016 2.155981 -0.295301
C -5.768268 -0.092457 0.593717
C -7.232410 0.209240 0.824135
C -8.134119 -0.613722 -0.114409
C -7.952011 -0.260793 -1.585342
C -7.583093 -0.093102 2.285048
H -1.971606 -2.684591 -0.433182
H -2.434842 2.142683 0.163566
H -0.000717 2.451457 0.019861
H 2.352711 2.811717 -0.242331
H 1.502208 -2.947014 -0.818695
H 5.152732 -1.447223 -1.342576
H 4.217380 -1.554574 1.500802
H 4.478614 0.102165 1.942843
H 8.109158 -0.051980 1.529826
H 7.230763 0.681938 0.166489
H 6.756414 1.040446 1.858442
H 4.330983 3.699159 -1.360198
H 4.272472 3.942018 0.427375
H 5.820142 3.927643 -0.429801
H -4.525343 2.382891 -1.227748
H -6.108711 2.361658 -0.498897
H -4.720361 2.855457 0.477784
H -5.487782 -1.115961 0.852600
H -7.447223 1.270235 0.663627
H -7.949098 -1.686859 0.021493
H -9.184406 -0.440293 0.151626
H -6.946916 -0.510429 -1.938325
H -8.667107 -0.820873 -2.196453
H -8.124302 0.806543 -1.756499
H -8.629918 0.155519 2.490905
H -6.961276 0.496787 2.967881
H -7.436725 -1.152520 2.524726
SCF Energy (B3LYP/6-31G**)= -1361.70507841
Number of imaginary frequencies = 0

1b_c242
MMFF Geometry
C -1.600463 -2.363211 -0.388037
C -2.306151 0.253912 -0.206708
N -2.920737 -2.065694 -0.314871
C -0.565924 -1.421516 -0.380999
C -0.944111 -0.070526 -0.290593
C -3.259012 -0.762753 -0.213280
C 0.049315 0.924344 -0.289519
C 1.411436 0.596793 -0.372443
C 1.787744 -0.749093 -0.451666
C 0.802907 -1.752850 -0.462708
C 3.229184 -1.100270 -0.544148
C 4.289566 0.023828 -0.471929
C 3.712231 1.421133 -0.436587
C 2.398502 1.675229 -0.370507
O 1.124550 -3.087081 -0.561657
O 3.532173 -2.283689 -0.725581
O 5.080842 -0.077629 -1.675679
C 5.254944 -0.256817 0.686543
C 4.626865 -0.083388 2.051144
C 5.565997 0.211933 3.190641
O 3.420018 -0.227188 2.244046
O 4.634012 2.453854 -0.340952
C 4.770241 3.157926 -1.575370
C -4.712671 -0.477892 -0.136322
C -5.540887 -1.276603 -1.103829
C -5.180111 0.415016 0.762814
C -6.602058 0.845855 1.058521
C -7.231889 1.729896 -0.033966
C -6.435064 2.997020 -0.319749
C -7.519712 -0.321856 1.433424
H -1.384945 -3.427437 -0.456643
H -2.621888 1.291065 -0.149860
H -0.248541 1.969984 -0.217958
H 2.043996 2.697674 -0.278306
H 2.104141 -3.169201 -0.615120
H 4.464226 -0.102743 -2.427008
H 6.122610 0.411762 0.614741
H 5.652575 -1.277734 0.629615
H 4.997915 0.316610 4.119150
H 6.277871 -0.609773 3.300144
H 6.095430 1.147956 2.996225
H 5.066913 2.485893 -2.386831
H 3.842594 3.678678 -1.834705
H 5.556658 3.907738 -1.449024
H -5.652866 -2.308912 -0.755620
H -6.539193 -0.862517 -1.251848
H -5.071064 -1.294579 -2.094242
H -4.459496 0.898307 1.424713
H -6.538066 1.466581 1.964659
H -7.356295 1.171912 -0.967892
H -8.239537 2.025034 0.285183
H -5.451906 2.767581 -0.741788
H -6.969722 3.620640 -1.043494
H -6.292447 3.585092 0.592406
H -8.456750 0.054919 1.858840
H -7.051522 -0.965529 2.186305
H -7.783565 -0.941927 0.572700
SCF Energy (B3LYP/6-31G**)= -1361.69498979
Number of imaginary frequencies = 0

1b_c243
MMFF Geometry
C -1.742947 -1.853567 -0.035471
C -2.374950 0.779525 -0.249055
N -3.049867 -1.518707 -0.161367
C -0.685561 -0.941159 -0.005815
C -1.024422 0.419855 -0.114432
C -3.360419 -0.206486 -0.270362
C -0.005414 1.390007 -0.076796
C 1.341593 1.023464 0.048480
C 1.671236 -0.330292 0.137247
C 0.667945 -1.309715 0.130520

C 3.098180 -0.692099 0.269290
C 4.152321 0.318280 -0.245172
C 3.683019 1.749646 0.032785
C 2.376234 2.055468 0.149270
O 0.961985 -2.646294 0.258354
O 3.372521 -1.796832 0.743279
O 4.257056 0.217465 -1.668833
C 5.509867 0.030948 0.428814
C 6.068943 -1.324768 0.043636
C 6.890207 -2.045874 1.077581
O 5.904574 -1.786898 -1.087793
O 4.714850 2.670939 0.101232
C 4.390882 4.054931 0.161250
C -4.806896 0.123687 -0.426840
C -5.127633 1.360672 -1.218181
C -5.730172 -0.704407 0.110887
C -7.241410 -0.616304 0.136406
C -7.837182 0.708965 0.650133
C -7.345808 1.094115 2.039882
C -7.844642 -1.013978 -1.213189
H -1.560469 -2.923135 0.043714
H -2.648164 1.827089 -0.321825
H -0.270929 2.444115 -0.147257
H 2.031824 3.069463 0.316344
H 1.919089 -2.737755 0.466428
H 4.728167 -0.622443 -1.862426
H 5.407278 0.075129 1.519731
H 6.275072 0.754962 0.124205
H 7.219778 -3.009573 0.679680
H 7.767560 -1.445884 1.330764
H 6.283579 -2.223803 1.968946
H 3.803319 4.359120 -0.711014
H 3.865969 4.292408 1.091957
H 5.327641 4.619942 0.150433
H -4.465982 1.454225 -2.087110
H -6.141231 1.353011 -1.622512
H -5.017716 2.255584 -0.596967
H -5.364117 -1.590742 0.635525
H -7.567772 -1.386534 0.851133
H -7.639859 1.533495 -0.041328
H -8.929435 0.608031 0.693224
H -6.273874 1.312541 2.042824
H -7.868001 1.991889 2.386193
H -7.538511 0.292897 2.760390
H -8.933502 -1.106353 -1.134577
H -7.454896 -1.981711 -1.548470
H -7.631458 -0.277534 -1.993560
SCF Energy (B3LYP/6-31G**)= -1361.70730829
Number of imaginary frequencies = 0

1b_c244
MMFF Geometry
C -1.434826 -2.052150 -0.526316
C -2.202798 0.441683 0.235283
N -2.749792 -1.840864 -0.275579
C -0.435827 -1.079048 -0.423513
C -0.844842 0.208361 -0.026839
C -3.121219 -0.596988 0.094818
C 0.112493 1.233066 0.091633
C 1.468754 0.991444 -0.165264
C 1.869154 -0.293500 -0.543873
C 0.926271 -1.320287 -0.692232
C 3.304547 -0.526157 -0.810662
C 4.323458 0.447832 -0.173268
C 3.758955 1.866122 -0.119378
C 2.432326 2.095320 -0.099706
O 1.287217 -2.580492 -1.103554
O 3.624638 -1.494339 -1.502335
O 5.514666 0.453200 -0.971247
C 4.723535 0.001381 1.234797
C 4.929264 -1.492730 1.326511
C 6.223257 -2.063648 0.811071
O 4.040265 -2.214554 1.782017
O 4.735897 2.843171 -0.070014
C 4.337558 4.206525 0.014780
C -4.567649 -0.410223 0.367022
C -5.183658 -1.526307 1.164557
C -5.212494 0.675599 -0.112502
C -6.673357 1.068102 -0.005936
C -7.625516 0.018819 -0.610424
C -9.052961 0.526781 -0.770995
C -7.045874 1.492261 1.416756
H -1.193158 -3.070547 -0.823730
H -2.540536 1.421218 0.559277
H -0.211752 2.230810 0.385276
H 2.018143 3.095905 -0.040194
H 2.244126 -2.577959 -1.333129
H 5.304360 0.929927 -1.793007
H 3.947606 0.257534 1.968411
H 5.646994 0.491034 1.567793
H 6.335299 -3.090950 1.169238
H 6.223478 -2.064954 -0.280623
H 7.064085 -1.476172 1.188140
H 3.760267 4.500389 -0.867669
H 3.772475 4.391960 0.933836
H 5.242685 4.820178 0.045557
H -5.321361 -2.416266 0.541180
H -4.544248 -1.794079 2.013880
H -6.153878 -1.263097 1.587686
H -4.639325 1.388152 -0.707754
H -6.773505 1.970824 -0.626722
H -7.255225 -0.279778 -1.599539
H -7.653431 -0.888713 0.000735
H -9.079770 1.445534 -1.365219
H -9.662590 -0.224664 -1.283220
H -9.518842 0.725616 0.198556
H -8.000705 2.028153 1.425897
H -6.292920 2.169331 1.835965
H -7.142501 0.637236 2.091581
SCF Energy (B3LYP/6-31G**)= -1361.69390646
Number of imaginary frequencies = 0

1b_c245
MMFF Geometry
C 1.980799 -1.745301 0.022380
C 2.325276 0.916876 -0.387025
N 3.235897 -1.291060 -0.209882
C 0.838399 -0.942572 0.066103
C 1.028640 0.433682 -0.146001
C 3.405217 0.035524 -0.413886
C -0.082585 1.295883 -0.106943
C -1.377178 0.808511 0.127855
C -1.560893 -0.560879 0.329800
C -0.460691 -1.433000 0.310891
C -2.928103 -1.067708 0.575486
C -4.135946 -0.153213 0.298910
C -3.773335 1.322987 0.266113
C -2.499303 1.746875 0.185142
O -0.602223 -2.783737 0.524029
O -3.068299 -2.229795 0.969696
O -5.009467 -0.377279 1.416418
C -4.784776 -0.592189 -1.023690
C -6.275576 -0.817100 -0.922994
C -7.178845 0.386166 -0.900834
O -6.732283 -1.958723 -0.835526
O -4.878067 2.155370 0.296879
C -4.678068 3.554400 0.458329
C 4.799558 0.494517 -0.679407
C 4.960550 1.758897 -1.479302
C 5.836979 -0.241417 -0.222559
C 7.316407 0.044967 -0.351988
C 8.016373 0.023644 1.021126
C 7.531803 1.123956 1.957441
C 7.951106 -0.992508 -1.281244
H 1.915768 -2.820032 0.178726
H 2.482693 1.979790 -0.537588
H 0.070642 2.363549 -0.262142
H -2.248471 2.801342 0.147387
H -1.532307 -2.968494 0.788948
H -5.094352 -1.347797 1.516498
H -4.351774 -1.537319 -1.379143
H -4.592218 0.118096 -1.837699

H -8.190890 0.084431 -1.185665
H -7.203411 0.815291 0.102581
H -6.830845 1.128392 -1.623521
H -4.121342 3.768753 1.376316
H -4.173854 3.978640 -0.415612
H -5.661272 4.026905 0.541195
H 4.795693 2.637710 -0.847296
H 5.950105 1.852785 -1.933617
H 4.250269 1.787723 -2.313660
H 5.615958 -1.159201 0.326952
H 7.489943 1.032760 -0.790145
H 7.875083 -0.949452 1.508350
H 9.096901 0.147991 0.876288
H 6.477341 0.994549 2.219445
H 8.109912 1.106834 2.886941
H 7.656107 2.110625 1.499943
H 9.019144 -0.789878 -1.415954
H 7.481977 -0.971391 -2.271343
H 7.849794 -2.007844 -0.881198
SCF Energy (B3LYP/6-31G**)= -1361.70513231
Number of imaginary frequencies = 0

1b_c246
MMFF Geometry
C 1.751069 2.289688 0.101719
C 2.281759 -0.375985 0.124454
N 3.034946 1.899118 0.287486
C 0.670008 1.421042 -0.078798
C 0.956607 0.043171 -0.063744
C 3.288976 0.572950 0.288428
C -0.088191 -0.882322 -0.241777
C -1.411768 -0.457708 -0.422342
C -1.690783 0.910722 -0.424764
C -0.659379 1.848463 -0.269744
C -3.094012 1.340709 -0.598186
C -4.223283 0.320145 -0.361618
C -3.768200 -1.100755 -0.658149
C -2.466939 -1.444868 -0.661312
O -0.902187 3.201018 -0.292852
O -3.334541 2.519945 -0.876582
O -5.274485 0.691365 -1.268951
C -4.714166 0.527939 1.075556
C -5.674224 -0.536570 1.558554
C -7.083650 -0.511544 1.026499
O -5.312583 -1.384958 2.376954
O -4.814869 -1.974595 -0.891017
C -4.531909 -3.356441 -1.069926
C 4.706065 0.187951 0.499606
C 5.393633 0.973125 1.582085
C 5.268428 -0.771914 -0.266671
C 6.686661 -1.308843 -0.272621
C 7.741899 -0.222077 -0.552656
C 9.124260 -0.790835 -0.847619
C 6.981765 -2.149140 0.972184
H 1.607046 3.368101 0.103407
H 2.524938 -1.433685 0.156589
H 0.139859 -1.947918 -0.240466
H -2.142588 -2.461832 -0.853037
H -1.835273 3.351621 -0.564979
H -5.221829 1.661590 -1.380089
H -5.217499 1.497147 1.184171
H -3.868306 0.540790 1.775939
H -7.701687 -1.205993 1.602913
H -7.500829 0.492521 1.137140
H -7.102166 -0.813165 -0.021870
H -3.927229 -3.518146 -1.967896
H -4.041022 -3.773365 -0.184666
H -5.483061 -3.879586 -1.205636
H 4.756511 1.046849 2.471206
H 6.325631 0.514781 1.915155
H 5.624382 1.986647 1.236955
H 4.652948 -1.233619 -1.040455
H 6.725967 -2.004842 -1.123711
H 7.426176 0.375735 -1.417272
H 7.830851 0.469663 0.290843
H 9.088382 -1.503006 -1.677952
H 9.810204 0.016366 -1.124380
H 9.544298 -1.296489 0.026733
H 7.886367 -2.749765 0.831073
H 6.162061 -2.846122 1.180508
H 7.132807 -1.531715 1.861998
SCF Energy (B3LYP/6-31G**)= -1361.70250175
Number of imaginary frequencies = 0

1b_c247
MMFF Geometry
C -1.864192 -1.514462 -0.573264
C -2.115826 1.090447 0.148879
N -3.110774 -1.025745 -0.367271
C -0.685857 -0.775696 -0.440747
C -0.828744 0.570737 -0.065697
C -3.234102 0.273063 -0.008570
C 0.320262 1.366504 0.088145
C 1.604296 0.844179 -0.127253
C 1.744485 -0.495309 -0.500933
C 0.604965 -1.302637 -0.657275
C 3.104532 -1.049157 -0.715583
C 4.340405 -0.141549 -0.562812
C 4.010609 1.279780 -0.170070
C 2.763915 1.710501 0.057319
O 0.694600 -2.626439 -1.019007
O 3.216728 -2.233764 -1.049379
O 4.878291 -0.114967 -1.904768
C 5.380611 -0.817747 0.342420
C 5.102985 -0.704801 1.826970
C 3.924634 -1.454749 2.389749
O 5.825510 -0.031838 2.562624
O 5.056935 2.148817 0.106870
C 5.821764 2.491522 -1.047428
C -4.623682 0.778088 0.192125
C -4.847266 2.252791 -0.008761
C -5.605768 -0.094605 0.509789
C -7.067276 0.192845 0.773001
C -7.971956 -0.456396 -0.290605
C -7.768468 0.120192 -1.686135
C -7.438447 -0.329219 2.165254
H -1.837764 -2.563349 -0.860738
H -2.232260 2.126323 0.450321
H 0.207985 2.410459 0.380962
H 2.601370 2.732255 0.390735
H 1.639759 -2.854352 -1.165919
H 5.009472 -1.043972 -2.175936
H 6.372274 -0.383219 0.157435
H 5.497050 -1.881878 0.101604
H 4.072407 -1.608016 3.462744
H 3.009294 -0.881237 2.236128
H 3.843881 -2.436626 1.916955
H 6.539254 1.700599 -1.283247
H 5.180771 2.706812 -1.909347
H 6.390238 3.396732 -0.813869
H -4.306267 2.613602 -0.891162
H -5.895621 2.506634 -0.183411
H -4.507510 2.816393 0.866366
H -5.344568 -1.150687 0.607498
H -7.263375 1.269448 0.779782
H -7.805622 -1.540627 -0.323818
H -9.022118 -0.308591 -0.009061
H -6.763785 -0.088412 -2.065908
H -8.485873 -0.324205 -2.383525
H -7.921770 1.204018 -1.688538
H -8.483287 -0.097693 2.399215
H -6.814587 0.134958 2.937410
H -7.311729 -1.415552 2.236390
SCF Energy (B3LYP/6-31G**)= -1361.69959386
Number of imaginary frequencies = 0

1b_c248
MMFF Geometry
C -1.681653 -2.078933 -0.216976
C -2.193662 0.523350 0.375628
N -2.960951 -1.734403 0.068503
C -0.597926 -1.193458 -0.231935

C -0.875660 0.149634 0.076363
C -3.200669 -0.438811 0.359222
C 0.172485 1.086768 0.077417
C 1.492049 0.710938 -0.222443
C 1.767329 -0.625926 -0.524158
C 0.726873 -1.572950 -0.532490
C 3.160079 -1.028258 -0.839305
C 4.282683 0.032672 -0.798353
C 3.823264 1.445238 -0.474437
C 2.537355 1.736780 -0.213890
O 0.949845 -2.896531 -0.836115
O 3.369151 -2.205968 -1.148542
O 4.848832 0.065137 -2.127365
C 5.406118 -0.465451 0.121703
C 5.013329 -0.546362 1.580369
C 6.130602 -0.806283 2.556857
O 3.849474 -0.439292 1.964637
O 4.857080 2.365717 -0.487457
C 4.570350 3.722489 -0.169279
C -4.606517 -0.090132 0.663559
C -5.259335 -0.948393 1.708229
C -5.206397 0.889753 -0.046395
C -6.606571 1.468667 0.019407
C -7.453685 1.030145 -1.193183
C -7.764165 -0.459902 -1.260296
C -7.371376 1.317714 1.335882
H -1.542340 -3.133908 -0.443717
H -2.434949 1.551255 0.628583
H -0.049434 2.125525 0.320004
H 2.216905 2.745512 0.021669
H 1.909650 -3.015339 -1.022371
H 4.242747 0.577397 -2.688494
H 6.280496 0.192318 0.036755
H 5.742708 -1.463091 -0.187831
H 5.730973 -0.840651 3.574178
H 6.600693 -1.765815 2.328115
H 6.866238 -0.000563 2.495172
H 3.877231 4.156448 -0.896979
H 4.178665 3.810986 0.849139
H 5.507413 4.284773 -0.219907
H -5.475893 -0.361627 2.606285
H -6.184958 -1.390895 1.327973
H -4.625476 -1.782575 2.028593
H -4.615073 1.368769 -0.830634
H -6.460407 2.553717 -0.092115
H -8.400702 1.584447 -1.188131
H -6.937161 1.317595 -2.118262
H -8.338936 -0.791481 -0.391110
H -8.362225 -0.674223 -2.152236
H -6.851443 -1.059499 -1.323437
H -8.279134 1.931790 1.316382
H -6.766807 1.661566 2.181956
H -7.686584 0.290145 1.530669
SCF Energy (B3LYP/6-31G**)= -1361.69850442
Number of imaginary frequencies = 0

1b_c249
MMFF Geometry
C -1.718310 -1.764364 -0.343474
C -2.342265 0.859547 -0.027571
N -3.025801 -1.410108 -0.377079
C -0.655903 -0.876619 -0.156613
C -0.991054 0.478760 0.007791
C -3.333277 -0.101791 -0.221244
C 0.033234 1.421310 0.209932
C 1.380984 1.035875 0.236493
C 1.711630 -0.309806 0.062291
C 0.699362 -1.265594 -0.120036
C 3.139664 -0.705412 0.108700
C 4.231116 0.378034 -0.090468
C 3.710823 1.751883 0.311921
C 2.413287 2.036322 0.484700
O 0.982788 -2.603287 -0.265865
O 3.398154 -1.902258 0.264430
O 4.547124 0.464859 -1.482235
C 5.467079 0.000910 0.753207
C 6.149219 -1.257991 0.254921
C 6.763482 -2.161326 1.289346
O 6.243414 -1.498180 -0.950824
O 4.642874 2.748194 0.581411
C 5.205802 3.276399 -0.621956
C -4.781965 0.253034 -0.283148
C -5.110724 1.631306 -0.785451
C -5.699854 -0.672456 0.075808
C -7.212261 -0.596884 0.135592
C -7.733710 0.563904 1.004278
C -9.219553 0.451494 1.323332
C -7.833036 -0.645018 -1.262455
H -1.539842 -2.829141 -0.477418
H -2.610202 1.901894 0.110391
H -0.227261 2.470472 0.346275
H 2.109935 3.032716 0.792606
H 1.947360 -2.736778 -0.125173
H 5.057049 -0.344147 -1.710582
H 5.177794 -0.143535 1.801298
H 6.238281 0.779202 0.719217
H 7.207060 -3.033005 0.800407
H 7.543812 -1.620198 1.829812
H 5.991057 -2.503578 1.982469
H 5.943359 2.585884 -1.041155
H 4.431715 3.511114 -1.360909
H 5.724613 4.205194 -0.366717
H -4.477499 1.899105 -1.639184
H -6.137711 1.719370 -1.143158
H -4.965805 2.374999 0.005035
H -5.328163 -1.649324 0.395950
H -7.524991 -1.522970 0.640422
H -7.181081 0.585092 1.952250
H -7.563414 1.529938 0.519242
H -9.448656 -0.506046 1.801415
H -9.516775 1.251115 2.009607
H -9.831342 0.545290 0.421418
H -8.906153 -0.854750 -1.206408
H -7.380903 -1.439559 -1.866973
H -7.709078 0.298679 -1.801287
SCF Energy (B3LYP/6-31G**)= -1361.70143784
Number of imaginary frequencies = 0

1b_c250
MMFF Geometry
C -1.833558 -1.674014 -0.398062
C -2.143908 0.859529 0.526767
N -3.083192 -1.257862 -0.081045
C -0.680190 -0.893801 -0.284531
C -0.853565 0.415745 0.194042
C -3.235524 0.004944 0.380255
C 0.268768 1.251990 0.328825
C 1.556269 0.805204 -0.003852
C 1.727032 -0.498286 -0.478449
C 0.614220 -1.344823 -0.618967
C 3.091728 -0.973161 -0.816989
C 4.296509 -0.022760 -0.677078
C 3.934296 1.352995 -0.167885
C 2.688388 1.709953 0.166423
O 0.734552 -2.635270 -1.078967
O 3.230760 -2.125797 -1.240582
O 4.747021 0.116701 -2.043964
C 5.418621 -0.709573 0.115169
C 5.226681 -0.713490 1.617444
C 4.125734 -1.565329 2.191888
O 5.955251 -0.050972 2.356561
O 4.959399 2.249205 0.101842
C 5.631988 2.701909 -1.071804
C -4.624207 0.421126 0.732204
C -4.769481 1.508645 1.762251
C -5.670742 -0.199018 0.143302
C -7.146413 0.075960 0.328533
C -7.845731 0.329749 -1.021436
C -7.346319 1.584121 -1.728062
C -7.795116 -1.113487 1.040653
H -1.782906 -2.698021 -0.761858
H -2.286390 1.873941 0.884784

H 0.132741 2.267726 0.700267
H 2.505706 2.699001 0.578704
H 1.676525 -2.808852 -1.301231
H 4.896942 -0.785555 -2.386392
H 6.377348 -0.216395 -0.094678
H 5.566534 -1.747520 -0.208469
H 4.348728 -1.786189 3.239806
H 3.174715 -1.033528 2.135845
H 4.064835 -2.514067 1.653069
H 6.365716 1.962559 -1.404929
H 4.927619 2.942574 -1.875590
H 6.175325 3.615662 -0.813036
H -4.591290 2.490940 1.312505
H -5.758563 1.526495 2.226804
H -4.060642 1.365240 2.585941
H -5.461278 -0.995480 -0.574149
H -7.307451 0.962439 0.949844
H -7.716785 -0.532409 -1.688010
H -8.924644 0.437608 -0.853230
H -6.293459 1.494308 -2.012142
H -7.924008 1.754621 -2.642217
H -7.458095 2.465129 -1.088061
H -8.860525 -0.927054 1.214021
H -7.326400 -1.290393 2.015247
H -7.706715 -2.033434 0.451508
SCF Energy (B3LYP/6-31G**)= -1361.69955221
Number of imaginary frequencies = 0

1b_c251
MMFF Geometry
C -1.634152 -1.686662 -0.818617
C -2.213114 0.901570 -0.231688
N -2.937426 -1.333169 -0.705957
C -0.555000 -0.814853 -0.655084
C -0.866215 0.523037 -0.350644
C -3.221921 -0.043498 -0.413368
C 0.176485 1.452976 -0.178792
C 1.519252 1.067910 -0.292606
C 1.818228 -0.266533 -0.580955
C 0.794233 -1.202147 -0.780052
C 3.238108 -0.647125 -0.705994
C 4.303755 0.254356 -0.053213
C 3.875868 1.717237 -0.060532
C 2.580613 2.072292 -0.162909
O 1.060778 -2.510342 -1.103634
O 3.544943 -1.672067 -1.321645
O 5.499875 0.120002 -0.842667
C 4.630671 -0.168110 1.381121
C 4.673177 -1.667884 1.547671
C 5.967203 -2.373972 1.244144
O 3.661805 -2.286827 1.881428
O 4.930051 2.599199 0.096593
C 4.671397 3.995259 0.021139
C -4.667358 0.312009 -0.312301
C -5.037591 1.718963 -0.689613
C -5.549779 -0.636351 0.074075
C -7.046574 -0.564740 0.290222
C -7.547424 0.562768 1.213837
C -6.899298 0.547535 2.592565
C -7.801931 -0.573203 -1.041245
H -1.473595 -2.736718 -1.053793
H -2.464184 1.927802 0.015520
H -0.069003 2.490748 0.044089
H 2.264799 3.109484 -0.142412
H 2.029243 -2.616903 -1.233073
H 5.368352 -0.650181 -1.431143
H 3.873844 0.200656 2.086344
H 5.594100 0.239407 1.711326
H 5.900569 -3.414683 1.573698
H 6.160728 -2.353198 0.169931
H 6.786263 -1.893573 1.785036
H 4.235832 4.261877 -0.947246
H 4.025500 4.317099 0.844205
H 5.626264 4.520341 0.117790
H -4.471333 2.052764 -1.566823
H -6.088748 1.820395 -0.964733
H -4.836648 2.406301 0.138666
H -5.153608 -1.632031 0.290114
H -7.315051 -1.504787 0.795031
H -7.403125 1.547979 0.760575
H -8.631070 0.449692 1.347951
H -5.827705 0.760565 2.537185
H -7.356631 1.311371 3.229759
H -7.035224 -0.423358 3.079503
H -8.878133 -0.688095 -0.871187
H -7.476856 -1.407746 -1.672661
H -7.654002 0.352256 -1.605526
SCF Energy (B3LYP/6-31G**)= -1361.69953391
Number of imaginary frequencies = 0

1b_c252
MMFF Geometry
C 1.871902 -2.498265 -0.075603
C 2.565390 0.124249 -0.239547
N 3.191505 -2.190667 -0.101478
C 0.832291 -1.564328 -0.128341
C 1.203343 -0.209966 -0.216570
C 3.524759 -0.884178 -0.172102
C 0.203206 0.776919 -0.288463
C -1.156936 0.437195 -0.257043
C -1.519769 -0.907473 -0.153135
C -0.534983 -1.906459 -0.105137
C -2.960231 -1.247699 -0.120992
C -3.962134 -0.140823 0.278289
C -3.473562 1.236554 -0.160689
C -2.170864 1.485489 -0.391515
O -0.860409 -3.240754 -0.038612
O -3.280759 -2.414119 -0.365897
O -3.966952 -0.166618 1.712867
C -5.336910 -0.536006 -0.299783
C -6.489354 0.263945 0.269746
C -7.641060 0.554046 -0.654272
O -6.510225 0.613491 1.451799
O -4.481518 2.177577 -0.266074
C -4.126806 3.542298 -0.459195
C 4.977637 -0.591042 -0.200188
C 5.780268 -1.521717 -1.068876
C 5.486256 0.422455 0.532946
C 6.937509 0.834638 0.654103
C 7.168019 2.253270 0.101241
C 6.911882 2.362904 -1.396760
C 7.357608 0.770861 2.126262
H 1.661625 -3.563667 -0.008893
H 2.875286 1.161278 -0.323387
H 0.494746 1.823462 -0.368734
H -1.813200 2.465986 -0.684575
H -1.837583 -3.326030 -0.121110
H -4.807350 0.250265 2.002144
H -5.579059 -1.581621 -0.071723
H -5.327270 -0.431818 -1.391566
H -8.396355 1.143994 -0.127797
H -7.285536 1.127262 -1.514102
H -8.089810 -0.385586 -0.985282
H -3.650506 3.686465 -1.434056
H -5.047269 4.133361 -0.444148
H -3.482634 3.899286 0.350846
H 5.262791 -1.711357 -2.016660
H 6.758393 -1.115201 -1.337196
H 5.938330 -2.480115 -0.563156
H 4.809612 1.014734 1.149708
H 7.590526 0.145523 0.109694
H 6.531827 2.976678 0.626844
H 8.206531 2.550517 0.293776
H 5.861106 2.179325 -1.640457
H 7.163040 3.369155 -1.747294
H 7.526527 1.647886 -1.952802
H 8.416839 1.027050 2.237629
H 7.215426 -0.237379 2.531347
H 6.777579 1.467411 2.742376
SCF Energy (B3LYP/6-31G**)= -1361.71190142
Number of imaginary frequencies = 0

1b_c253
MMFF Geometry
C 1.765041 2.492723 -0.058686
C 2.503644 -0.103939 0.246665
N 3.084616 2.220253 0.085378
C 0.746014 1.534494 -0.060249
C 1.140765 0.194912 0.100919
C 3.441900 0.925876 0.226531
C 0.162007 -0.814120 0.113332
C -1.200374 -0.511188 -0.032140
C -1.593055 0.820754 -0.192006
C -0.623570 1.839135 -0.208980
C -3.035280 1.142062 -0.337972
C -4.087573 0.015678 -0.254706
C -3.485602 -1.368430 -0.148834
C -2.173853 -1.597878 -0.014991
O -0.962009 3.163277 -0.365235
O -3.354365 2.322728 -0.517647
O -4.779516 0.109419 -1.504792
C -4.988123 0.340752 0.950630
C -6.356769 -0.291910 0.865863
C -6.730282 -1.290824 1.928180
O -7.158548 0.049855 -0.005134
O -4.353464 -2.448828 -0.051915
C -4.920605 -2.799017 -1.316852
C 4.894193 0.672799 0.382756
C 5.602955 1.656884 1.274288
C 5.486480 -0.353636 -0.264563
C 6.955853 -0.722207 -0.253791
C 7.138439 -2.056193 0.492321
C 8.596486 -2.458194 0.667458
C 7.468791 -0.786020 -1.695810
H 1.537183 3.550021 -0.177117
H 2.828824 -1.130012 0.388307
H 0.471778 -1.851734 0.236569
H -1.813295 -2.615652 0.107197
H -1.934238 3.225582 -0.507676
H -5.440264 0.824895 -1.424660
H -5.175413 1.419791 1.031617
H -4.503497 0.054804 1.892453
H -7.646837 -1.810819 1.636303
H -5.936796 -2.031241 2.048474
H -6.896392 -0.768085 2.873206
H -5.791540 -2.174547 -1.532952
H -4.186487 -2.735701 -2.127734
H -5.264077 -3.835717 -1.250424
H 4.999672 1.883375 2.161321
H 6.556576 1.277087 1.649792
H 5.795446 2.592414 0.738646
H 4.878013 -0.992236 -0.905548
H 7.542590 0.046309 0.259256
H 6.685223 -1.978336 1.489237
H 6.607726 -2.861936 -0.030821
H 9.166886 -1.664206 1.159570
H 8.665056 -3.357999 1.287402
H 9.069659 -2.682783 -0.292774
H 8.553174 -0.932115 -1.723696
H 7.254340 0.147134 -2.229369
H 7.002716 -1.606750 -2.252639
SCF Energy (B3LYP/6-31G**)= -1361.70233747
Number of imaginary frequencies = 0

1b_c254
MMFF Geometry
C 1.813477 -1.637464 -0.032511
C 2.319075 1.030838 -0.081276
N 3.105756 -1.231438 0.000859
C 0.710613 -0.782212 -0.090767
C 0.983882 0.597475 -0.115915
C 3.353972 0.098383 -0.022449
C -0.084410 1.511501 -0.182096
C -1.415183 1.072266 -0.207963
C -1.678542 -0.298165 -0.166572
C -0.626987 -1.224780 -0.127600
C -3.089516 -0.737218 -0.196086
C -4.169681 0.262539 0.283962
C -3.787651 1.685520 -0.134936
C -2.505059 2.041735 -0.341918
O -0.857525 -2.579807 -0.126467
O -3.327997 -1.890480 -0.561458
O -4.204573 0.279004 1.714487
C -5.539810 -0.145281 -0.295872
C -6.010734 -1.487926 0.228008
C -6.839838 -2.332886 -0.700510
O -5.771986 -1.843222 1.384498
O -4.867432 2.547891 -0.227452
C -4.617893 3.935767 -0.415927
C 4.789204 0.506948 -0.000433
C 5.126885 1.798800 -0.690664
C 5.692133 -0.304117 0.594725
C 7.185951 -0.148743 0.786470
C 8.009519 -0.393721 -0.491480
C 7.784908 -1.772177 -1.100977
C 7.579577 1.168699 1.462033
H 1.682734 -2.717242 -0.009753
H 2.539645 2.093183 -0.086290
H 0.129507 2.579153 -0.213250
H -2.221302 3.052977 -0.609461
H -1.817093 -2.734199 -0.278459
H -4.622700 -0.562600 2.000372
H -5.489041 -0.189872 -1.390409
H -6.326568 0.565192 -0.015231
H -7.101313 -3.273458 -0.207952
H -7.757306 -1.798876 -0.959203
H -6.265761 -2.557643 -1.602790
H -4.007295 4.339345 0.398260
H -4.149087 4.119049 -1.387919
H -5.580890 4.454783 -0.404340
H 4.885998 2.653331 -0.049853
H 6.180806 1.870912 -0.963842
H 4.571987 1.898312 -1.630743
H 5.319205 -1.232793 1.034578
H 7.472494 -0.934857 1.500927
H 7.793324 0.367429 -1.248697
H 9.075900 -0.296306 -0.251433
H 6.757323 -1.891896 -1.457455
H 8.452190 -1.916760 -1.956781
H 7.993809 -2.562139 -0.372615
H 8.621724 1.123724 1.798235
H 6.957639 1.362178 2.342957
H 7.496524 2.027373 0.790786
SCF Energy (B3LYP/6-31G**)= -1361.70748511
Number of imaginary frequencies = 0

1b_c255
MMFF Geometry
C -1.780342 -1.735008 -0.440034
C -2.103416 0.928723 -0.019937
N -3.040728 -1.265310 -0.274578
C -0.624410 -0.948776 -0.413946
C -0.804441 0.427778 -0.198573
C -3.193083 0.061046 -0.061690
C 0.320085 1.271670 -0.169453
C 1.619482 0.768990 -0.347128
C 1.796543 -0.602023 -0.553689
C 0.679635 -1.456483 -0.591191
C 3.167228 -1.138529 -0.738615
C 4.373389 -0.175344 -0.668761
C 4.018440 1.288126 -0.459897
C 2.747934 1.702113 -0.315306
O 0.805399 -2.809782 -0.806715
O 3.293003 -2.346236 -0.966511
O 5.035494 -0.272559 -1.949393
C 5.378281 -0.708670 0.362000
C 4.874626 -0.667180 1.787906
C 5.889456 -0.964438 2.860833
O 3.700720 -0.435613 2.074274
O 5.127756 2.116012 -0.444719
C 4.939861 3.509354 -0.226702
C -4.586280 0.547400 0.135054
C -4.769896 1.623005 1.166497
C -5.579759 -0.029640 -0.575796

C -7.066277 0.251268 -0.659678
C -7.895451 -0.790965 0.116508
C -7.697173 -0.785925 1.626529
C -7.522494 1.686364 -0.384114
H -1.720829 -2.809251 -0.601577
H -2.260449 1.991240 0.136221
H   0.174569 2.338304 -0.001536
H   2.501795 2.746832 -0.161480
H   1.761821 -3.020456 -0.911023
H   4.519368 0.252920 -2.583515
H   6.311170 -0.132995 0.309775
H   5.647123 -1.748075 0.134478
H   5.415463 -0.902578 3.844363
H   6.285089 -1.973351 2.720902
H   6.698311 -0.231174 2.813638
H   4.341749 3.953252 -1.028887
H   4.485470 3.694269 0.751948
H   5.924114 3.986801 -0.236800
H -3.995648 1.575956 1.941140
H -4.732370 2.614331 0.704236
H -5.713913 1.512768 1.704293
H -5.292564 -0.842473 -1.249677
H -7.309712 0.092368 -1.721574
H -8.960054 -0.631499 -0.096327
H -7.655972 -1.794316 -0.259191
H -7.981370 0.173481 2.067448
H -8.323945 -1.557861 2.085316
H -6.659390 -1.000691 1.897351
H -8.549441 1.826415 -0.741312
H -6.892648 2.409189 -0.913793
H -7.523221 1.942042 0.677235
SCF Energy (B3LYP/6-31G**)= -1361.69876410
Number of imaginary frequencies = 0

1b_c256
MMFF Geometry
C   1.677802 2.464255 0.139170
C   2.295992 -0.139800 -0.337616
N   2.984613 2.107376 0.159783
C   0.615173 1.589867 -0.109445
C   0.947381 0.244533 -0.358338
C   3.279779 0.809666 -0.068620
C -0.075938 -0.682110 -0.631200
C -1.422042 -0.293419 -0.640173
C -1.746038 1.039860 -0.374730
C -0.737923 1.982798 -0.128589
C -3.175147 1.426454 -0.365330
C -4.223752 0.310981 -0.149407
C -3.764809 -0.988018 -0.802954
C -2.461488 -1.262957 -0.995168
O -1.028568 3.305973 0.102285
O -3.453916 2.622052 -0.473544
O -5.457477 0.739892 -0.736669
C -4.441655 0.195565 1.363747
C -5.246868 -1.020761 1.761569
C -6.737838 -0.980800 1.550908
O -4.692983 -2.016860 2.232546
O -4.810157 -1.824217 -1.149686
C -4.511244 -3.164340 -1.524991
C   4.721378 0.463300 -0.039814
C   5.601371 1.452084 -0.752866
C   5.140977 -0.646832 0.605236
C   6.546852 -1.174356 0.818286
C   7.080045 -1.866609 -0.449089
C   8.398917 -2.595768 -0.229950
C   7.509075 -0.137131 1.405026
H   1.498749 3.519187 0.336344
H   2.578564 -1.167867 -0.542639
H   0.186795 -1.718994 -0.839005
H -2.126023 -2.193324 -1.440402
H -1.984203 3.456329 -0.082450
H -5.360865 0.647306 -1.700650
H -4.958890 1.080368 1.756078
H -3.484865 0.138017 1.899291
H -7.205341 -1.795952 2.110690
H -7.139745 -0.035612 1.924347
H -6.972974 -1.098146 0.491612
H -3.961789 -3.189802 -2.471219
H -3.956686 -3.679392 -0.733841
H -5.458735 -3.691144 -1.671579
H   5.790750 2.325943 -0.120724
H   6.562756 1.025290 -1.044490
H   5.132022 1.793751 -1.682996
H   4.391182 -1.266856 1.099168
H   6.448342 -1.951881 1.590319
H   6.339689 -2.596178 -0.802170
H   7.212864 -1.141784 -1.260056
H   8.317770 -3.318385 0.588085
H   8.680941 -3.141154 -1.136393
H   9.208843 -1.897622 0.001295
H   8.376647 -0.631090 1.856164
H   7.026751 0.449623 2.194687
H   7.889959 0.554240 0.648621
SCF Energy (B3LYP/6-31G**)= -1361.69616292
Number of imaginary frequencies = 0

1b_c257
MMFF Geometry
C   1.814273 2.412456 0.093014
C   2.482783 -0.149781 -0.514914
N   3.129390 2.112727 -0.036912
C   0.766643 1.498458 -0.060907
C   1.125365 0.176509 -0.377269
C   3.451212 0.834945 -0.331325
C   0.116823 -0.787254 -0.551249
C -1.239823 -0.457510 -0.410537
C -1.596445 0.855972 -0.092075
C -0.597487 1.830824 0.078247
C -3.031539 1.203070 0.064697
C -4.108339 0.104766 -0.066421
C -3.553418 -1.240549 -0.480581
C -2.245547 -1.496871 -0.603163
O -0.900370 3.136738 0.386723
O -3.323877 2.379543 0.306605
O -4.951793 0.585393 -1.118981
C -4.831840 0.031288 1.290262
C -6.224088 -0.546455 1.198046
C -6.496541 -1.830382 1.935040
O -7.117564 0.050035 0.594567
O -4.443938 -2.298855 -0.610186
C -5.182870 -2.234172 -1.832537
C   4.899868 0.551877 -0.469925
C   5.670884 1.615118 -1.205276
C   5.434508 -0.567863 0.062781
C   6.890064 -0.987901 0.055649
C   7.364213 -1.206215 1.505648
C   8.852947 -1.505615 1.618003
C   7.042454 -2.246145 -0.801872
H   1.614961 3.454473 0.334471
H   2.781010 -1.160181 -0.777223
H   0.398300 -1.810050 -0.801141
H -1.913126 -2.500272 -0.855114
H -1.879605 3.237568 0.404850
H -5.567125 1.237815 -0.730877
H -4.963895 1.029443 1.728682
H -4.241353 -0.535213 2.021055
H -7.463072 -2.234692 1.621973
H -5.724224 -2.568563 1.709381
H -6.518179 -1.632600 3.009441
H -6.049014 -1.576170 -1.722899
H -4.557547 -1.916121 -2.674132
H -5.555646 -3.240020 -2.048036
H   5.867536 2.470413 -0.550266
H   6.628058 1.255080 -1.590555
H   5.109777 1.969179 -2.078143
H   4.781969 -1.257460 0.599039
H   7.516580 -0.205100 -0.382798
H   7.152831 -0.304321 2.094901
H   6.801552 -2.024698 1.972601
H   9.448818 -0.724294 1.135921
H   9.146840 -1.553424 2.671582
H   9.105163 -2.467072 1.161573

H 8.095586 -2.519235 -0.920921
H 6.634494 -2.087103 -1.806727
H 6.519324 -3.100719 -0.358102
SCF Energy (B3LYP/6-31G**)= -1361.70246035
Number of imaginary frequencies = 0

1b_c258
MMFF Geometry
C -1.701592 -1.489495 -0.669726
C -2.148367 1.064457 0.140354
N -2.982070 -1.102332 -0.453804
C -0.582881 -0.668821 -0.507659
C -0.825856 0.652375 -0.089510
C -3.200748 0.169650 -0.048572
C 0.258607 1.533628 0.080459
C 1.577090 1.114638 -0.143823
C 1.809145 -0.204632 -0.542499
C 0.741361 -1.089637 -0.742979
C 3.204115 -0.620946 -0.781961
C 4.339239 0.196681 -0.135855
C 3.965558 1.669423 -0.015286
C 2.680347 2.072468 -0.011584
O 0.939737 -2.378586 -1.174721
O 3.435824 -1.608609 -1.485178
O 5.480354 0.080276 -1.005511
C 4.735683 -0.337293 1.242682
C 4.731682 -1.845676 1.302560
C 5.977397 -2.571168 0.870081
O 3.719789 -2.452648 1.656136
O 5.058845 2.503489 0.136014
C 4.847454 3.909058 0.171897
C -4.621431 0.553764 0.197971
C -4.856737 1.603956 1.247278
C -5.599048 -0.073883 -0.493071
C -7.101529 0.111613 -0.470161
C -7.786204 -0.484150 0.773909
C -7.523987 -1.974484 0.953411
C -7.540466 1.559484 -0.710996
H -1.594322 -2.523724 -0.989976
H -2.347943 2.084777 0.450791
H 0.065128 2.560171 0.389795
H 2.404470 3.115229 0.100128
H 1.894284 -2.506938 -1.370682
H 5.285354 -0.640607 -1.637240
H 4.037009 0.005393 2.017628
H 5.731842 0.013442 1.539248
H 5.891566 -3.629920 1.130466
H 6.106261 -2.480328 -0.210179
H 6.845213 -2.158183 1.390220
H 4.364613 4.256871 -0.747050
H 4.264350 4.193179 1.053637
H 5.824999 4.394854 0.244241
H -4.673030 2.603866 0.840697
H -5.872319 1.584964 1.645857
H -4.199038 1.450627 2.110697
H -5.294098 -0.844074 -1.206366
H -7.479013 -0.451068 -1.337004
H -7.474048 0.041952 1.682355
H -8.870210 -0.336181 0.688393
H -6.466341 -2.176313 1.148328
H -8.097154 -2.355966 1.804599
H -7.826249 -2.536110 0.063802
H -8.613502 1.596861 -0.930826
H -7.013730 1.994752 -1.567358
H -7.368997 2.201373 0.156974
SCF Energy (B3LYP/6-31G**)= -1361.69964573
Number of imaginary frequencies = 0

1b_c259
MMFF Geometry
C -1.928774 -2.303214 -0.113287
C -2.504422 0.332959 -0.437015
N -3.225035 -1.954199 -0.298897
C -0.857105 -1.405613 -0.074600
C -1.167030 -0.043685 -0.244358
C -3.500637 -0.641240 -0.451604
C -0.132419 0.909205 -0.212499
C 1.203544 0.525957 -0.025958
C 1.507070 -0.828348 0.129784
C 0.484800 -1.790115 0.121498
C 2.922645 -1.214955 0.325703
C 4.018683 -0.220656 -0.119762
C 3.553737 1.224893 0.030923
C 2.247584 1.549446 0.060335
O 0.747889 -3.127348 0.303901
O 3.153362 -2.335524 0.788716
O 4.187327 -0.482217 -1.520430
C 5.295838 -0.558379 0.677239
C 6.545477 0.100072 0.132031
C 7.596150 0.502465 1.131310
O 6.720318 0.248902 -1.079227
O 4.589592 2.138230 0.104782
C 4.285018 3.526527 0.030956
C -4.929448 -0.303901 -0.657422
C -5.661317 -1.243619 -1.577499
C -5.483627 0.750622 -0.021749
C -6.926239 1.205009 -0.077277
C -7.553279 1.263594 1.329251
C -7.649810 -0.101692 1.999151
C -6.993139 2.583224 -0.740204
H -1.765669 -3.372093 0.007694
H -2.764978 1.375851 -0.588416
H -0.377651 1.963459 -0.335399
H 1.909247 2.575670 0.147339
H 1.703356 -3.228946 0.517723
H 5.075665 -0.145517 -1.767866
H 5.509066 -1.634376 0.650447
H 5.165861 -0.275547 1.728930
H 8.436154 0.973462 0.613392
H 7.173420 1.220592 1.838329
H 7.955350 -0.383242 1.660791
H 3.756274 3.763989 -0.897810
H 3.708740 3.845592 0.905084
H 5.230050 4.077660 0.031975
H -5.918483 -2.171054 -1.054977
H -6.582433 -0.813563 -1.978362
H -5.043677 -1.495927 -2.447502
H -4.862394 1.345588 0.648170
H -7.532954 0.521140 -0.678821
H -6.980952 1.940482 1.976178
H -8.565179 1.681361 1.255137
H -6.660132 -0.526485 2.192426
H -8.166542 -0.012102 2.960106
H -8.212576 -0.804643 1.376715
H -8.031067 2.923779 -0.821725
H -6.573500 2.552532 -1.752037
H -6.437465 3.334045 -0.166866
SCF Energy (B3LYP/6-31G**)= -1361.71195512
Number of imaginary frequencies = 0

1b_c260
MMFF Geometry
C 1.775642 1.897825 -0.412533
C 2.214136 -0.780919 -0.372597
N 3.058562 1.461273 -0.397641
C 0.650162 1.069630 -0.412084
C 0.889656 -0.314397 -0.391642
C 3.273040 0.125803 -0.376276
C -0.202232 -1.200426 -0.389809
C -1.525021 -0.731559 -0.410721
C -1.762010 0.646991 -0.425503
C -0.678697 1.543495 -0.429633
C -3.161044 1.146996 -0.455981
C -4.330781 0.132281 -0.473016
C -3.900040 -1.320330 -0.449947
C -2.617904 -1.701005 -0.400835
O -0.862231 2.906860 -0.458777
O -3.344106 2.366423 -0.528574
O -5.029854 0.361256 -1.715385
C -5.316833 0.483425 0.649215
C -4.756837 0.252280 2.035942
C -5.760849 0.123246 3.150952

O -3.548052 0.218913 2.263558
O -4.877981 -2.298202 -0.310389
C -5.691534 -2.449515 -1.472488
C 4.697380 -0.319158 -0.372992
C 4.985180 -1.644497 -1.020892
C 5.634961 0.487547 0.172586
C 7.126931 0.295031 0.343241
C 7.570224 -1.014067 1.025111
C 6.934599 -1.228438 2.393038
C 7.871398 0.518037 -0.975657
H 1.671896 2.980688 -0.425705
H 2.407576 -1.848070 -0.340088
H -0.013992 -2.273495 -0.367958
H -2.371302 -2.755868 -0.316850
H -1.829205 3.091861 -0.452942
H -4.373378 0.306164 -2.431463
H -6.233353 -0.109826 0.540301
H -5.625829 1.534200 0.585141
H -5.240661 -0.042313 4.098471
H -6.347110 1.042495 3.223324
H -6.416435 -0.729303 2.957086
H -6.479378 -1.691311 -1.490837
H -5.097007 -2.416345 -2.392032
H -6.174596 -3.429589 -1.416166
H 4.397184 -1.769982 -1.937448
H 6.027614 -1.748726 -1.326364
H 4.748944 -2.467290 -0.338194
H 5.297129 1.441980 0.584701
H 7.452074 1.104889 1.013333
H 7.366840 -1.884824 0.394775
H 8.659533 -0.987619 1.158391
H 5.852272 -1.368622 2.317661
H 7.352834 -2.123874 2.864088
H 7.128990 -0.377724 3.053881
H 8.953621 0.540163 -0.806421
H 7.589005 1.474437 -1.429941
H 7.667425 -0.272678 -1.703605
SCF Energy (B3LYP/6-31G**)= -1361.69555651
Number of imaginary frequencies = 0

1b_c261
MMFF Geometry
C -1.835379 -1.561577 -0.692213
C -2.150035 0.986836 0.188988
N -3.095046 -1.114739 -0.468150
C -0.675000 -0.805323 -0.507138
C -0.850648 0.512520 -0.053509
C -3.247555 0.154628 -0.026026
C 0.278460 1.326463 0.146132
C 1.576057 0.850265 -0.097757
C 1.749907 -0.464736 -0.541977
C 0.628635 -1.287574 -0.749371
C 3.121731 -0.969913 -0.808629
C 4.333379 -0.030160 -0.591916
C 3.969951 1.358243 -0.105723
C 2.710358 1.740934 0.135453
O 0.749018 -2.581722 -1.201025
O 3.246008 -2.114449 -1.256264
O 4.957214 0.109370 -1.886502
C 5.358589 -0.731920 0.308897
C 4.880926 -0.917323 1.732821
C 5.945151 -1.162325 2.769817
O 3.688111 -0.919885 2.034954
O 4.996622 2.220688 0.260618
C 5.755306 2.695376 -0.850382
C -4.646564 0.605334 0.231728
C -4.827360 1.636528 1.310363
C -5.655027 0.049369 -0.476195
C -7.145713 0.312544 -0.447119
C -7.861665 -0.280631 0.780475
C -7.677895 -1.787017 0.918694
C -7.508027 1.787500 -0.647697
H -1.781312 -2.590884 -1.040248
H -2.294936 2.006329 0.530873
H 0.139891 2.347090 0.501812
H 2.515897 2.727054 0.548185
H 1.705789 -2.786360 -1.310637
H 4.268423 0.397969 -2.510246
H 6.293986 -0.158754 0.327143
H 5.616585 -1.721715 -0.087917
H 5.481966 -1.277786 3.753650
H 6.491098 -2.076529 2.524596
H 6.628858 -0.310422 2.801801
H 6.506429 1.957415 -1.145575
H 5.113410 2.958899 -1.698114
H 6.284015 3.598685 -0.531690
H -4.590049 2.636334 0.932273
H -5.843181 1.660468 1.708047
H -4.180235 1.424015 2.169220
H -5.389962 -0.715339 -1.210932
H -7.551469 -0.205502 -1.328886
H -7.523228 0.203197 1.702895
H -8.936377 -0.073879 0.699694
H -6.632418 -2.049172 1.107361
H -8.270958 -2.161304 1.759382
H -8.008346 -2.307352 0.014125
H -8.577495 1.887092 -0.865524
H -6.958680 2.217942 -1.492211
H -7.303664 2.395424 0.237434
SCF Energy (B3LYP/6-31G**)= -1361.69557169
Number of imaginary frequencies = 0

1b_c262
MMFF Geometry
C 1.881942 1.877403 -0.034904
C 2.160886 -0.820967 -0.174559
N 3.130615 1.359186 0.051149
C 0.715472 1.124395 -0.190243
C 0.871877 -0.271513 -0.263539
C 3.265551 0.014786 -0.017054
C -0.264042 -1.085160 -0.431649
C -1.549525 -0.531785 -0.511189
C -1.697989 0.853958 -0.422757
C -0.575756 1.682564 -0.279246
C -3.061671 1.419537 -0.489324
C -4.267480 0.493735 -0.240923
C -3.965290 -0.942141 -0.642326
C -2.704375 -1.402551 -0.741632
O -0.690060 3.050726 -0.217278
O -3.204711 2.630094 -0.689269
O -5.327809 1.012950 -1.060760
C -4.656211 0.658147 1.232669
C -5.681703 -0.344058 1.714409
C -7.110707 -0.160840 1.273330
O -5.354177 -1.269925 2.459951
O -5.100825 -1.701157 -0.860920
C -4.959626 -3.089192 -1.134716
C 4.652556 -0.521305 0.098584
C 4.794305 -1.880811 0.724213
C 5.687969 0.240340 -0.319876
C 7.175245 -0.048530 -0.354925
C 7.812880 0.156300 1.031076
C 9.333838 0.082505 1.013775
C 7.529403 -1.390458 -1.003184
H 1.843026 2.962789 0.028042
H 2.294570 -1.895739 -0.243206
H -0.138436 -2.165336 -0.501828
H -2.488236 -2.431265 -1.008437
H -1.618265 3.301736 -0.424098
H -5.189340 1.979107 -1.122744
H -5.061247 1.659990 1.424365
H -3.775129 0.553931 1.879981
H -7.756368 -0.833273 1.845548
H -7.426783 0.867087 1.467968
H -7.216206 -0.392310 0.212343
H -4.422243 -3.249098 -2.074817
H -4.463224 -3.603287 -0.305397
H -5.961837 -3.514171 -1.243128
H 4.604860 -2.667219 -0.013588
H 5.785254 -2.045695 1.151497
H 4.093072 -2.006589 1.557323
H 5.454226 1.230093 -0.720537

H 7.600615 0.717351 -1.020370
H 7.522005 1.139891 1.422238
H 7.435390 -0.585393 1.744059
H 9.754124 0.795162 0.297240
H 9.732250 0.324042 2.004506
H 9.683155 -0.920799 0.752561
H 8.577090 -1.396011 -1.323599
H 6.919862 -1.573970 -1.894929
H 7.398874 -2.233003 -0.318892
SCF Energy (B3LYP/6-31G**)= -1361.70263957
Number of imaginary frequencies = 0

1b_c263
MMFF Geometry
C -1.522583 -2.499871 -0.090472
C -2.307646 0.095106 0.091626
N -2.836140 -2.248231 0.128906
C -0.533068 -1.521758 -0.232006
C -0.951387 -0.181719 -0.134396
C -3.216131 -0.954872 0.210065
C -0.004320 0.849494 -0.270026
C 1.350681 0.566164 -0.489018
C 1.763067 -0.766271 -0.575069
C 0.828437 -1.806775 -0.461680
C 3.199932 -1.049115 -0.791598
C 4.237444 0.078786 -0.589988
C 3.623994 1.462950 -0.737322
C 2.298492 1.661583 -0.685379
O 1.197022 -3.127270 -0.571127
O 3.549670 -2.190270 -1.115300
O 5.177700 -0.106964 -1.674671
C 5.013645 -0.205906 0.706621
C 4.275154 0.157044 1.973933
C 5.105803 0.658587 3.125658
O 3.064122 -0.015248 2.094300
O 4.390615 2.564207 -1.103168
C 5.677365 2.705698 -0.523687
C -4.659557 -0.723496 0.455812
C -5.275373 -1.655991 1.464067
C -5.324984 0.244251 -0.210553
C -6.798389 0.575224 -0.109295
C -7.043221 1.837024 0.740741
C -6.439592 3.125476 0.194861
C -7.408049 0.692104 -1.509848
H -1.273780 -3.557150 -0.151207
H -2.648901 1.121094 0.188430
H -0.331282 1.886417 -0.203346
H 1.896436 2.659135 -0.841437
H 2.146760 -3.165943 -0.821594
H 5.227352 -1.070544 -1.837261
H 5.973893 0.320813 0.697750
H 5.276580 -1.269318 0.780468
H 4.455075 0.904778 3.969351
H 5.809764 -0.118336 3.433511
H 5.645355 1.560374 2.827182
H 5.622278 2.660662 0.567446
H 6.379551 1.966830 -0.916829
H 6.058081 3.695164 -0.794863
H -4.606196 -1.800681 2.320362
H -6.212324 -1.271979 1.876378
H -5.476590 -2.633178 1.012665
H -4.780831 0.849510 -0.935574
H -7.330961 -0.247617 0.380947
H -8.124882 1.983269 0.853869
H -6.649170 1.670405 1.751607
H -6.856484 3.381771 -0.783272
H -6.660440 3.955232 0.874531
H -5.351836 3.054913 0.102711
H -8.470976 0.949245 -1.449502
H -7.326540 -0.259971 -2.046756
H -6.908280 1.454793 -2.116467
SCF Energy (B3LYP/6-31G**)= -1361.69617816
Number of imaginary frequencies = 0

1b_c264
MMFF Geometry
C 1.505496 -2.275860 -0.027884
C 2.411737 0.284677 0.079810
N 2.843578 -2.078730 0.055176
C 0.548400 -1.256839 -0.062782
C 1.029166 0.065037 -0.005615
C 3.282861 -0.802707 0.100993
C 0.116198 1.135278 -0.041274
C -1.264619 0.908199 -0.118603
C -1.735351 -0.405661 -0.159825
C -0.839529 -1.484505 -0.151421
C -3.195464 -0.619683 -0.242533
C -4.123494 0.507991 0.271109
C -3.516619 1.875156 -0.058693
C -2.189285 2.039762 -0.219413
O -1.274976 -2.785865 -0.230245
O -3.596839 -1.701638 -0.676909
O -4.196742 0.453988 1.699273
C -5.522541 0.347518 -0.358747
C -6.208164 -0.932163 0.078584
C -7.129644 -1.588830 -0.912970
O -6.059992 -1.380641 1.217718
O -4.448779 2.897424 -0.123820
C -3.984626 4.238204 -0.228196
C 4.752758 -0.632080 0.198168
C 5.411921 -1.565760 1.175060
C 5.382729 0.286464 -0.565990
C 6.860975 0.604334 -0.682973
C 7.344004 1.453290 0.506863
C 8.764150 1.976755 0.338678
C 7.733072 -0.624530 -0.957421
H 1.208485 -3.321840 -0.065772
H 2.805330 1.294449 0.143217
H 0.492200 2.157110 -0.007994
H -1.746705 3.007864 -0.423474
H -2.241997 -2.781893 -0.410800
H -4.746871 -0.326848 1.928394
H -5.447575 0.353439 -1.452787
H -6.199216 1.155028 -0.054512
H -7.545993 -2.502475 -0.479882
H -7.946797 -0.906734 -1.159635
H -6.570937 -1.851507 -1.814655
H -3.342985 4.499036 0.619666
H -3.465760 4.398205 -1.178627
H -4.856851 4.898225 -0.206496
H 5.549178 -2.556462 0.729227
H 6.385119 -1.203517 1.511412
H 4.804877 -1.675924 2.081443
H 4.778644 0.885943 -1.248705
H 6.949733 1.232787 -1.581555
H 6.674342 2.313862 0.633492
H 7.291910 0.879574 1.438969
H 8.867610 2.541190 -0.593336
H 9.018457 2.644204 1.168414
H 9.493605 1.161437 0.334734
H 8.701779 -0.322599 -1.370516
H 7.263462 -1.288433 -1.691712
H 7.935746 -1.203446 -0.052438
SCF Energy (B3LYP/6-31G**)= -1361.70598995
Number of imaginary frequencies = 0

1b_c265
MMFF Geometry
C 1.727937 2.297413 0.117656
C 2.249357 -0.370443 0.095269
N 3.008893 1.898675 0.306641
C 0.645712 1.436066 -0.088045
C 0.927327 0.056674 -0.096593
C 3.258270 0.571823 0.285558
C -0.118560 -0.862083 -0.302428
C -1.438461 -0.428734 -0.484946
C -1.713154 0.941308 -0.461347
C -0.680089 1.872256 -0.281971
C -3.117045 1.378977 -0.632877
C -4.235159 0.353967 -0.333527
C -3.794388 -1.050357 -0.731824
C -2.495967 -1.402450 -0.767784

O -0.919998 3.225407 -0.284244
O -3.324897 2.551541 -0.950373
O -5.395589 0.729238 -1.084212
C -4.575932 0.493416 1.154714
C -5.472686 -0.609689 1.669577
C -6.938443 -0.545767 1.328339
O -5.010540 -1.537297 2.337991
O -4.851427 -1.891231 -1.028170
C -4.593857 -3.285165 -1.157591
C 4.672124 0.177957 0.502093
C 5.352451 0.940494 1.605169
C 5.238208 -0.769419 -0.276884
C 6.654700 -1.310945 -0.280090
C 7.715947 -0.222751 -0.530279
C 9.099064 -0.790622 -0.823403
C 6.935799 -2.175320 0.951398
H 1.587250 3.376113 0.138474
H 2.488877 -1.429338 0.110148
H 0.105906 -1.928199 -0.321229
H -2.175509 -2.404893 -1.030012
H -1.849088 3.380877 -0.571654
H -5.229098 0.478142 -2.009624
H -5.074791 1.449769 1.356871
H -3.668027 0.485453 1.772142
H -7.490650 -1.239528 1.968767
H -7.317833 0.462808 1.510572
H -7.095959 -0.825015 0.285085
H -3.975165 -3.486173 -2.037767
H -4.129156 -3.684640 -0.250284
H -5.553091 -3.792183 -1.297426
H 4.707526 0.999942 2.489722
H 6.279836 0.472758 1.938059
H 5.589731 1.959428 1.281039
H 4.628221 -1.214445 -1.064616
H 6.699460 -1.991079 -1.143646
H 7.409994 0.392168 -1.386369
H 7.799521 0.452818 0.326767
H 9.068381 -1.487044 -1.667194
H 9.790100 0.019257 -1.078855
H 9.509537 -1.313941 0.045078
H 7.839693 -2.776284 0.807229
H 6.111991 -2.873274 1.139281
H 7.080774 -1.575123 1.853917
SCF Energy (B3LYP/6-31G**)= -1361.69648938
Number of imaginary frequencies = 0

1b_c266
MMFF Geometry
C -1.593474 -1.588492 -0.638311
C -2.080071 0.971582 0.128963
N -2.879550 -1.219510 -0.423177
C -0.488331 -0.745802 -0.496494
C -0.751900 0.578358 -0.100696
C -3.117343 0.055168 -0.038507
C 0.317838 1.481312 0.046434
C 1.642060 1.080517 -0.177116
C 1.896384 -0.242040 -0.552035
C 0.841818 -1.147944 -0.731221
C 3.300270 -0.643599 -0.783391
C 4.411145 0.201043 -0.115790
C 4.018099 1.676332 -0.069101
C 2.728071 2.061741 -0.081693
O 1.059939 -2.440542 -1.142474
O 3.520623 -1.641706 -1.471479
O 5.614480 0.066161 -0.883632
C 4.719419 -0.292382 1.299655
C 4.743028 -1.800495 1.389519
C 5.972077 -2.521036 0.903419
O 3.763177 -2.411647 1.819862
O 5.102702 2.530092 0.009616
C 4.867406 3.931115 0.089238
C -4.542179 0.420897 0.207536
C -4.792200 1.469126 1.255402
C -5.512802 -0.221624 -0.479679
C -7.017637 -0.044003 -0.462506
C -7.646429 -0.758048 0.747571
C -9.168387 -0.790280 0.706868
C -7.470498 1.410580 -0.622325
H -1.469531 -2.626347 -0.940482
H -2.297084 1.993524 0.422057
H 0.107812 2.510120 0.336752
H 2.434506 3.104414 -0.027855
H 2.016202 -2.552270 -1.346033
H 5.482757 0.564745 -1.708768
H 3.961306 0.053344 2.014927
H 5.685986 0.082962 1.657833
H 5.951508 -3.555074 1.259127
H 5.999628 -2.520208 -0.187926
H 6.867317 -2.039062 1.304104
H 4.352148 4.292191 -0.806571
H 4.304881 4.181925 0.994238
H 5.838034 4.432612 0.145407
H -4.661149 2.472407 0.837160
H -5.794986 1.405813 1.682257
H -4.106711 1.351276 2.102656
H -5.200242 -0.995370 -1.185780
H -7.377990 -0.561733 -1.363847
H -7.282751 -1.793058 0.787085
H -7.332110 -0.282553 1.683565
H -9.527559 -1.240324 -0.223937
H -9.553264 -1.385699 1.541113
H -9.592708 0.214306 0.794238
H -8.513372 1.452302 -0.955518
H -6.871255 1.932597 -1.376534
H -7.410060 1.972714 0.313377
SCF Energy (B3LYP/6-31G**)= -1361.69389612
Number of imaginary frequencies = 0

1b_c267
MMFF Geometry
C -1.649987 -1.565925 -0.965913
C -2.244965 0.940296 -0.103917
N -2.955812 -1.228922 -0.833739
C -0.575667 -0.716272 -0.691473
C -0.895474 0.579285 -0.245242
C -3.248328 0.019888 -0.403717
C 0.141298 1.486045 0.043738
C 1.485935 1.117860 -0.094502
C 1.795791 -0.177061 -0.524523
C 0.776603 -1.086053 -0.839038
C 3.220566 -0.542473 -0.674978
C 4.285606 0.294482 0.061443
C 3.831361 1.734810 0.202490
C 2.540100 2.098712 0.168240
O 1.050076 -2.351008 -1.300167
O 3.535220 -1.503394 -1.383730
O 5.471061 0.261019 -0.752377
C 4.630357 -0.268589 1.442117
C 4.678780 -1.777249 1.458717
C 5.984406 -2.442445 1.115420
O 3.665417 -2.431885 1.706505
O 4.805631 2.657920 0.551558
C 5.234915 3.408977 -0.584650
C -4.696207 0.359832 -0.284331
C -5.064931 1.800280 -0.503906
C -5.581588 -0.627349 -0.021719
C -7.081543 -0.582437 0.179171
C -7.598660 0.431193 1.218562
C -6.970463 0.258542 2.595789
C -7.817594 -0.438948 -1.155402
H -1.483179 -2.583294 -1.313474
H -2.502127 1.932409 0.252401
H -0.108939 2.492193 0.378604
H 2.255328 3.129699 0.356962
H 2.021045 -2.445218 -1.419309
H 5.380625 -0.510239 -1.348393
H 3.881040 0.023856 2.189893
H 5.596142 0.108962 1.799872
H 5.909285 -3.516014 1.310126
H 6.215169 -2.288738 0.059510
H 6.782450 -2.033636 1.740078
H 5.625896 2.759431 -1.373908

H 4.418538 4.026368 -0.974364
H 6.040170 4.075212 -0.261582
H -4.487840 2.232398 -1.329622
H -6.112546 1.931436 -0.779931
H -4.876889 2.388957 0.399953
H -5.185882 -1.640707 0.085447
H -7.354801 -1.574440 0.568871
H -7.450429 1.462036 0.883453
H -8.683826 0.302429 1.322937
H -5.898752 0.477575 2.580895
H -7.438971 0.943793 3.309613
H -7.110822 -0.761932 2.966125
H -8.895814 -0.573764 -1.015405
H -7.481150 -1.195216 -1.873515
H -7.664127 0.545275 -1.607635
SCF Energy (B3LYP/6-31G**)= -1361.69448725
Number of imaginary frequencies = 0

1b_c268
MMFF Geometry
C -1.841990 -2.528083 -0.079011
C -2.582530 0.062835 0.267489
N -3.161247 -2.259593 0.075140
C -0.824239 -1.568505 -0.071423
C -1.219989 -0.231958 0.111052
C -3.519529 -0.967912 0.236642
C -0.242565 0.778179 0.133588
C 1.119516 0.479310 -0.022637
C 1.513225 -0.849617 -0.203698
C 0.545038 -1.869015 -0.230958
C 2.955180 -1.166736 -0.361053
C 4.006341 -0.040199 -0.266330
C 3.403019 1.341375 -0.137522
C 2.091606 1.567005 0.005771
O 0.884518 -3.190267 -0.408031
O 3.275002 -2.344215 -0.559367
O 4.692533 -0.114778 -1.520867
C 4.912973 -0.381495 0.929929
C 6.280360 0.254252 0.847972
C 6.657525 1.238141 1.922945
O 7.078495 -0.073691 -0.031642
O 4.269878 2.421470 -0.028953
C 4.830601 2.790816 -1.291300
C -4.971159 -0.719022 0.403431
C -5.676281 -1.717110 1.282276
C -5.567978 0.319492 -0.220036
C -7.037896 0.679721 -0.190981
C -7.315602 1.870546 0.746807
C -6.648657 3.184289 0.357288
C -7.545319 0.931733 -1.614333
H -1.613272 -3.583232 -0.213831
H -2.908306 1.086318 0.425714
H -0.553158 1.813450 0.273349
H 1.730261 2.582387 0.144434
H 1.856150 -3.249122 -0.555903
H 5.354614 -0.830408 -1.454238
H 5.102081 -1.461333 0.994341
H 4.432393 -0.109945 1.878066
H 7.572005 1.763609 1.634378
H 5.863626 1.975619 2.057703
H 6.828768 0.701955 2.859482
H 5.701347 2.170781 -1.520534
H 4.092768 2.738268 -2.099574
H 5.172994 3.826922 -1.211401
H -5.071966 -1.953150 2.166057
H -6.631149 -1.343974 1.662186
H -5.866624 -2.646046 0.734561
H -4.962715 0.969342 -0.851989
H -7.620431 -0.167440 0.188480
H -8.399277 2.035385 0.798301
H -6.996941 1.607933 1.763849
H -6.990571 3.534056 -0.620970
H -6.899381 3.958679 1.089872
H -5.558812 3.092861 0.333586
H -8.604345 1.210913 -1.604375
H -7.445966 0.027073 -2.225135
H -6.988666 1.729833 -2.116948
SCF Energy (B3LYP/6-31G**)= -1361.70132919
Number of imaginary frequencies = 0

1b_c269
MMFF Geometry
C -1.384575 -2.187908 -0.638690
C -2.260672 0.354787 -0.243116
N -2.720939 -1.983760 -0.544382
C -0.414860 -1.184288 -0.548772
C -0.880311 0.129005 -0.346553
C -3.144334 -0.718482 -0.337984
C 0.044863 1.186204 -0.262433
C 1.423351 0.953292 -0.359356
C 1.877198 -0.355963 -0.542458
C 0.970536 -1.418588 -0.656113
C 3.331999 -0.575865 -0.651390
C 4.284394 0.494472 -0.084109
C 3.687242 1.891524 -0.205541
C 2.359081 2.082527 -0.322694
O 1.389444 -2.707829 -0.878879
O 3.757105 -1.604052 -1.186003
O 5.487943 0.437417 -0.871633
C 4.659014 0.231534 1.376424
C 4.877708 -1.234341 1.663177
C 6.245677 -1.806261 1.404807
O 3.946263 -1.938306 2.055874
O 4.630311 2.900979 -0.131661
C 4.208889 4.246056 -0.318195
C -4.613424 -0.539187 -0.244040
C -5.389618 -1.310257 -1.275286
C -5.142180 0.245678 0.719777
C -6.593817 0.536015 1.049047
C -7.193715 1.559186 0.067721
C -8.579054 2.044748 0.472916
C -7.447181 -0.723831 1.223785
H -1.100022 -3.226277 -0.795875
H -2.645006 1.360538 -0.104137
H -0.321614 2.202437 -0.121389
H 1.923164 3.073631 -0.383153
H 2.363802 -2.709702 -1.008076
H 5.446964 -0.387223 -1.396095
H 3.864200 0.565201 2.056903
H 5.567872 0.774724 1.663167
H 6.302108 -2.817223 1.818204
H 6.434898 -1.850659 0.330542
H 7.002785 -1.190642 1.896747
H 3.744206 4.379061 -1.300515
H 3.530205 4.556538 0.482549
H 5.095310 4.885742 -0.274745
H -5.490340 -2.359220 -0.977575
H -6.388996 -0.903377 -1.440068
H -4.888287 -1.274312 -2.249730
H -4.458080 0.731447 1.417306
H -6.573808 1.015595 2.038938
H -6.530236 2.431359 0.003098
H -7.253179 1.138819 -0.942443
H -8.570786 2.455793 1.487270
H -8.914323 2.833056 -0.208838
H -9.314904 1.236281 0.430871
H -8.359988 -0.493863 1.784231
H -6.908502 -1.492828 1.788543
H -7.756675 -1.154604 0.267823
SCF Energy (B3LYP/6-31G**)= -1361.69817743
Number of imaginary frequencies = 0

1b_c270
MMFF Geometry
C 1.627157 -2.256231 0.251771
C 2.528378 0.294068 -0.019624
N 2.966264 -2.050591 0.278049
C 0.666447 -1.251424 0.095063
C 1.145228 0.064532 -0.043355
C 3.401305 -0.780449 0.135789
C 0.228895 1.118752 -0.205678
C -1.152993 0.883350 -0.221937

C -1.626886 -0.422507 -0.076530
C -0.723606 -1.487973 0.068353
C -3.090156 -0.658353 -0.112001
C -4.053939 0.532066 0.130440
C -3.391205 1.851067 -0.245074
C -2.072483 1.996679 -0.429783
O -1.150904 -2.789665 0.187347
O -3.479998 -1.815247 -0.294075
O -4.341822 0.615832 1.528485
C -5.333860 0.314699 -0.703916
C -6.143656 -0.875104 -0.227418
C -6.865988 -1.677735 -1.274983
O -6.248696 -1.135962 0.973126
O -4.211791 2.949884 -0.475739
C -4.697905 3.505020 0.748806
C 4.871239 -0.597226 0.173616
C 5.547272 -1.305751 1.312149
C 5.478156 0.150505 -0.773095
C 6.942164 0.450376 -1.031659
C 7.321116 1.876692 -0.585781
C 7.242884 2.130192 0.914117
C 7.963234 -0.594460 -0.574841
H 1.332100 -3.297174 0.366351
H 2.922359 1.301851 -0.108999
H 0.601766 2.136219 -0.319371
H -1.665483 2.961860 -0.716884
H -2.125672 -2.813113 0.055732
H -4.934514 -0.138432 1.743982
H -5.074815 0.167575 -1.759518
H -6.014751 1.171319 -0.638767
H -7.588900 -1.040227 -1.789588
H -6.144362 -2.083567 -1.988253
H -7.396390 -2.508562 -0.801646
H -5.501136 2.888462 1.162581
H -3.893300 3.634653 1.481035
H -5.115183 4.491172 0.524407
H 5.926029 -2.281187 0.991358
H 6.368839 -0.713685 1.722186
H 4.861288 -1.469010 2.151886
H 4.842597 0.601473 -1.538461
H 7.028343 0.448862 -2.129174
H 8.340876 2.098624 -0.924784
H 6.667100 2.598565 -1.092122
H 7.923831 1.479211 1.469268
H 7.527063 3.165397 1.130435
H 6.228787 1.980562 1.295854
H 8.935449 -0.395197 -1.040525
H 7.658524 -1.601991 -0.877690
H 8.126605 -0.592966 0.504623
SCF Energy (B3LYP/6-31G**)= -1361.69853199
Number of imaginary frequencies = 0

1b_c271
MMFF Geometry
C -1.714716 -1.408995 -0.763302
C -2.182311 1.063056 0.260637
N -2.999643 -1.044968 -0.533886
C -0.601188 -0.603465 -0.513268
C -0.855229 0.675595 0.015217
C -3.228692 0.186078 -0.021800
C 0.222953 1.540931 0.278535
C 1.545219 1.146061 0.036852
C 1.790536 -0.133790 -0.472604
C 0.728103 -0.999865 -0.764336
C 3.192106 -0.527416 -0.731177
C 4.324698 0.245656 -0.026352
C 3.921214 1.688459 0.210859
C 2.641180 2.086076 0.276856
O 0.935860 -2.246660 -1.302756
O 3.434202 -1.460554 -1.502772
O 5.455112 0.221722 -0.915360
C 4.739815 -0.392186 1.301431
C 4.744753 -1.900634 1.244929
C 6.005507 -2.582767 0.786473
O 3.730506 -2.539414 1.527976
O 4.941314 2.568667 0.539193
C 5.318847 3.362987 -0.585927
C -4.654215 0.543956 0.236312
C -4.908267 1.499091 1.368840
C -5.619640 -0.023980 -0.520423
C -7.122949 0.155295 -0.503291
C -7.823084 -0.546894 0.674914
C -7.558019 -2.046509 0.729426
C -7.563620 1.617473 -0.625119
H -1.599304 -2.411038 -1.171037
H -2.389586 2.051749 0.656697
H 0.022944 2.535659 0.675527
H 2.398144 3.113610 0.531337
H 1.894460 -2.359713 -1.487573
H 5.305373 -0.516974 -1.539981
H 4.048465 -0.116456 2.109011
H 5.737057 -0.057900 1.612934
H 5.911335 -3.662129 0.935507
H 6.172641 -2.384354 -0.273963
H 6.853620 -2.225875 1.375987
H 5.640424 2.742891 -1.428410
H 4.497253 4.019059 -0.892638
H 6.161540 3.992131 -0.284720
H -4.721744 2.530748 1.052971
H -5.929547 1.443885 1.749048
H -4.262916 1.272884 2.225532
H -5.301794 -0.728896 -1.293077
H -7.486083 -0.331643 -1.420650
H -7.525833 -0.100127 1.629655
H -8.906295 -0.394732 0.586721
H -6.502527 -2.261805 0.921589
H -8.141853 -2.501161 1.536271
H -7.845533 -2.530272 -0.209456
H -8.633574 1.671089 -0.856315
H -7.026413 2.125974 -1.433265
H -7.406703 2.182788 0.297155
SCF Energy (B3LYP/6-31G**)= -1361.69457978
Number of imaginary frequencies = 0

1b_c272
MMFF Geometry
C 1.805134 2.426142 -0.006042
C 2.437633 -0.208117 -0.227565
N 3.114824 2.081881 0.034171
C 0.746026 1.524666 -0.153159
C 1.086110 0.163651 -0.270304
C 3.417226 0.769755 -0.066586
C 0.067262 -0.792204 -0.435276
C -1.281506 -0.414852 -0.468849
C -1.615263 0.936379 -0.335650
C -0.610824 1.904992 -0.194842
C -3.046500 1.316889 -0.355924
C -4.117474 0.231628 -0.123645
C -3.615421 -1.122092 -0.578929
C -2.313729 -1.424838 -0.692193
O -0.905596 3.243431 -0.091449
O -3.354264 2.503173 -0.511611
O -5.246055 0.623939 -0.920574
C -4.501125 0.299660 1.357975
C -5.384870 -0.833074 1.831602
C -6.808420 -0.876217 1.339582
O -4.958471 -1.669162 2.630835
O -4.574760 -2.104043 -0.770246
C -4.862909 -2.288532 -2.156601
C 4.861606 0.435588 -0.021983
C 5.723827 1.352747 -0.843919
C 5.293334 -0.600703 0.728986
C 6.699516 -1.112746 0.965401
C 7.319654 -1.831637 -0.247183
C 6.493144 -3.015043 -0.735688
C 7.643036 -0.044994 1.527425
H 1.620748 3.494344 0.086950
H 2.725401 -1.249879 -0.332331
H 0.334181 -1.843747 -0.538565
H -2.006549 -2.433072 -0.955290
H -1.865607 3.372144 -0.261169
H -5.263329 1.602451 -0.916194

H -5.026931 1.236167 1.584336
H -3.601670 0.291401 1.988298
H -7.367300 -1.623240 1.910714
H -7.279909 0.097626 1.493900
H -6.842528 -1.147432 0.283574
H -5.189289 -1.355780 -2.627278
H -3.993516 -2.693955 -2.684822
H -5.677777 -3.013985 -2.236056
H 5.859166 2.313504 -0.335905
H 6.712356 0.938564 -1.047003
H 5.267656 1.540347 -1.823048
H 4.551921 -1.162814 1.299255
H 6.607276 -1.866079 1.761939
H 7.470913 -1.137596 -1.080530
H 8.314883 -2.201665 0.030152
H 5.521980 -2.694394 -1.124768
H 7.020324 -3.532094 -1.543962
H 6.323145 -3.734846 0.071267
H 8.563949 -0.510446 1.896551
H 7.182327 0.485568 2.368017
H 7.934779 0.694738 0.777341
SCF Energy (B3LYP/6-31G**)= -1361.69649159
Number of imaginary frequencies = 0

1b_c273
MMFF Geometry
C 1.867232 2.159480 0.155282
C 2.354232 -0.514110 0.215606
N 3.145561 1.750688 0.339897
C 0.770987 1.306315 -0.006731
C 1.035346 -0.075964 0.027379
C 3.377453 0.420536 0.360254
C -0.024688 -0.986991 -0.131871
C -1.340885 -0.542562 -0.313237
C -1.600199 0.831211 -0.333733
C -0.552381 1.753367 -0.197129
C -2.999148 1.284679 -0.509600
C -4.150164 0.286283 -0.270712
C -3.698016 -1.130397 -0.554281
C -2.412601 -1.512388 -0.528387
O -0.771869 3.109494 -0.239030
O -3.219811 2.465389 -0.798539
O -5.184741 0.659651 -1.194278
C -4.646358 0.518556 1.159971
C -5.635212 -0.512176 1.658178
C -7.014097 -0.524909 1.050760
O -5.328583 -1.288267 2.565531
O -4.697895 -2.073292 -0.734154
C -4.886398 -2.376302 -2.116791
C 4.789054 0.016527 0.569048
C 5.496074 0.803728 1.637068
C 5.333258 -0.961704 -0.186823
C 6.745648 -1.510119 -0.198848
C 7.869994 -0.474326 -0.392841
C 7.707067 0.365471 -1.653556
C 7.009972 -2.401603 1.017301
H 1.741050 3.240031 0.141874
H 2.580117 -1.575111 0.262646
H 0.184213 -2.056445 -0.114948
H -2.148408 -2.556578 -0.669748
H -1.705156 3.272995 -0.502172
H -5.142244 1.633535 -1.281169
H -5.128361 1.500249 1.253333
H -3.802628 0.521616 1.863182
H -7.663782 -1.179370 1.639127
H -7.433653 0.484075 1.071125
H -6.979764 -0.897315 0.026062
H -5.116669 -1.477718 -2.697828
H -4.004544 -2.877299 -2.529785
H -5.735459 -3.061309 -2.197199
H 5.789606 1.788909 1.259439
H 4.847357 0.946937 2.509258
H 6.391359 0.303230 2.009204
H 4.705830 -1.429747 -0.946963
H 6.795182 -2.174398 -1.074534
H 7.956237 0.190702 0.471699
H 8.828084 -1.005721 -0.460929
H 6.818809 1.002043 -1.602192
H 8.576042 1.018654 -1.783322
H 7.627429 -0.270234 -2.541108
H 7.968010 -2.922192 0.910006
H 6.230168 -3.164229 1.122730
H 7.048419 -1.829547 1.948851
SCF Energy (B3LYP/6-31G**)= -1361.69642415
Number of imaginary frequencies = 0

1b_c274
MMFF Geometry
C -1.662791 -2.488089 -0.109067
C -2.457382 0.092515 0.197840
N -2.983644 -2.247016 0.072871
C -0.669359 -1.504446 -0.148851
C -1.092323 -0.172900 0.013273
C -3.369249 -0.960924 0.214778
C -0.139649 0.861716 -0.015195
C 1.225272 0.591571 -0.194727
C 1.640832 -0.732561 -0.350030
C 0.701067 -1.775984 -0.338326
C 3.081164 -1.009697 -0.537681
C 4.109733 0.100073 -0.251081
C 3.506164 1.495364 -0.273293
C 2.177376 1.702412 -0.244637
O 1.073323 -3.089011 -0.504029
O 3.426394 -2.141315 -0.892355
O 5.047466 -0.001891 -1.333831
C 4.775032 -0.192402 1.103477
C 6.285098 -0.167951 1.055696
C 6.976520 1.168198 1.026074
O 6.927593 -1.219223 1.016034
O 4.457767 2.499494 -0.294030
C 4.033963 3.841537 -0.500600
C -4.822030 -0.743197 0.413407
C -5.477627 -1.736116 1.335394
C -5.460111 0.261246 -0.224821
C -6.937610 0.591991 -0.172146
C -7.131910 1.928294 0.566824
C -8.593876 2.294424 0.783113
C -7.495912 0.628058 -1.598188
H -1.411931 -3.540388 -0.225929
H -2.804903 1.111031 0.340805
H -0.474114 1.892021 0.103532
H 1.753680 2.700787 -0.245723
H 2.029719 -3.121835 -0.735642
H 5.296061 -0.946691 -1.402171
H 4.492432 -1.187598 1.473381
H 4.438600 0.495340 1.889620
H 8.013881 1.046308 1.350774
H 6.965023 1.571352 0.011714
H 6.484918 1.859039 1.715529
H 3.482024 3.939327 -1.441021
H 3.435856 4.195116 0.345118
H 4.927324 4.469571 -0.566163
H -4.841555 -1.938850 2.205100
H -6.428433 -1.376673 1.737160
H -5.662863 -2.681300 0.814344
H -4.888426 0.908544 -0.890382
H -7.488189 -0.186150 0.365863
H -6.646532 1.872165 1.549965
H -6.638584 2.742099 0.020101
H -9.128111 1.491036 1.299939
H -8.666823 3.198372 1.396495
H -9.101889 2.497082 -0.164110
H -8.584075 0.745927 -1.593968
H -7.273680 -0.304602 -2.129423
H -7.068601 1.454849 -2.176722
SCF Energy (B3LYP/6-31G**)= -1361.70287432
Number of imaginary frequencies = 0

1b_c275
MMFF Geometry
C 1.568336 -2.145890 -0.249826
C 2.378641 0.445870 -0.368632

N 2.899788 -1.898192 -0.296626
C 0.572218 -1.164431 -0.258771
C 1.003447 0.173994 -0.324163
C 3.291422 -0.606895 -0.344495
C 0.048999 1.207610 -0.353348
C -1.323418 0.928956 -0.298929
C -1.743605 -0.399944 -0.215163
C -0.808217 -1.444690 -0.214691
C -3.196004 -0.668418 -0.159904
C -4.122063 0.459203 0.357448
C -3.599332 1.820563 -0.111283
C -2.297328 2.018922 -0.394481
O -1.196915 -2.762554 -0.175891
O -3.588984 -1.789963 -0.488683
O -4.073783 0.500021 1.787109
C -5.561295 0.208784 -0.138189
C -6.156564 -1.060961 0.438267
C -7.131114 -1.814174 -0.425700
O -5.895796 -1.426042 1.586930
O -4.573590 2.803531 -0.163806
C -4.173469 4.148828 -0.396412
C 4.756551 -0.378895 -0.397062
C 5.482128 -1.305537 -1.332831
C 5.321185 0.575149 0.374802
C 6.777202 0.969566 0.529336
C 7.669007 -0.191639 1.008172
C 9.058255 0.259126 1.441996
C 7.308582 1.682284 -0.716616
H 1.310729 -3.201918 -0.203203
H 2.734942 1.469217 -0.435186
H 0.385745 2.241547 -0.418145
H -1.911777 2.985261 -0.698267
H -2.175124 -2.803315 -0.271726
H -4.571211 -0.281879 2.112763
H -5.578210 0.143060 -1.232708
H -6.241752 1.011512 0.170239
H -7.473153 -2.710059 0.099712
H -7.992464 -1.178524 -0.644473
H -6.639944 -2.118093 -1.353371
H -3.474791 4.489836 0.374411
H -3.741797 4.259817 -1.396155
H -5.066515 4.778620 -0.344403
H 4.953007 -1.381575 -2.289984
H 6.491161 -0.966899 -1.571203
H 5.559933 -2.308626 -0.899996
H 4.669181 1.149808 1.034788
H 6.787095 1.719539 1.334166
H 7.189200 -0.689803 1.860370
H 7.785012 -0.949438 0.227018
H 8.998369 1.031042 2.215616
H 9.615757 -0.588674 1.853235
H 9.632384 0.654847 0.599149
H 8.248187 2.200251 -0.497940
H 6.598996 2.438804 -1.070546
H 7.499681 0.988753 -1.540201
SCF Energy (B3LYP/6-31G**)= -1361.70627653
Number of imaginary frequencies = 0

1b_c276
MMFF Geometry
C -1.721773 -2.423237 -0.140050
C -2.443351 0.165126 -0.546004
N -3.044017 -2.134727 -0.205578
C -0.692306 -1.484713 -0.264211
C -1.077898 -0.148831 -0.476252
C -3.392149 -0.844718 -0.399304
C -0.087995 0.840317 -0.619396
C 1.276441 0.523170 -0.542265
C 1.654055 -0.803238 -0.322711
C 0.678829 -1.805600 -0.195371
C 3.093997 -1.129660 -0.241351
C 4.120865 0.007524 -0.086260
C 3.583210 1.362062 -0.519608
C 2.271697 1.581438 -0.722547
O 1.015362 -3.123895 0.001905
O 3.435980 -2.316172 -0.269126
O 5.187519 -0.358647 -0.975230
C 4.590196 0.041935 1.377121
C 6.093211 0.041731 1.532484
C 6.836467 1.326194 1.283574
O 6.691939 -0.993771 1.830486
O 4.569787 2.323700 -0.646738
C 4.232288 3.580275 -1.221623
C -4.848163 -0.574863 -0.470025
C -5.629841 -1.595932 -1.252457
C -5.378994 0.496448 0.157622
C -6.839453 0.893112 0.228281
C -7.266854 1.001520 1.704949
C -8.754751 1.268936 1.887277
C -7.037612 2.205981 -0.532539
H -1.501052 -3.476354 0.021659
H -2.764109 1.186255 -0.727720
H -0.392538 1.871978 -0.793821
H 1.893132 2.554078 -1.017401
H 1.991891 -3.218877 -0.080410
H 5.403780 -1.294496 -0.784099
H 4.221186 -0.833590 1.928842
H 4.181831 0.900463 1.925171
H 7.816302 1.277056 1.767221
H 6.974857 1.474846 0.211034
H 6.287115 2.166033 1.716199
H 3.812683 3.453788 -2.224880
H 3.543705 4.131957 -0.573946
H 5.151641 4.166433 -1.311223
H -5.793413 -2.497574 -0.652855
H -6.603924 -1.224413 -1.580160
H -5.093912 -1.879931 -2.165815
H -4.717891 1.156612 0.719738
H -7.470075 0.133366 -0.243562
H -7.023578 0.063860 2.221597
H -6.699560 1.793868 2.209835
H -9.356242 0.514195 1.371038
H -9.013238 1.237972 2.950721
H -9.035106 2.256027 1.508603
H -8.097715 2.470263 -0.597142
H -6.662000 2.124362 -1.559044
H -6.511072 3.035324 -0.046905
SCF Energy (B3LYP/6-31G**)= -1361.70291963
Number of imaginary frequencies = 0

1b_c277
MMFF Geometry
C -1.444585 -2.060244 -0.486919
C -2.219336 0.441590 0.240595
N -2.760581 -1.848425 -0.242531
C -0.447558 -1.084155 -0.393356
C -0.860144 0.207415 -0.014134
C -3.135466 -0.600430 0.110452
C 0.095129 1.235105 0.095594
C 1.452678 0.993366 -0.154405
C 1.854989 -0.295213 -0.516822
C 0.915785 -1.326194 -0.654867
C 3.287685 -0.524000 -0.784449
C 4.314553 0.464581 -0.199065
C 3.743882 1.875397 -0.115178
C 2.415764 2.097868 -0.086259
O 1.280729 -2.591013 -1.047634
O 3.636094 -1.495068 -1.462184
O 5.438910 0.474662 -1.097774
C 4.814941 0.040140 1.183739
C 5.022248 -1.451239 1.291874
C 6.346006 -2.012519 0.847099
O 4.113448 -2.179335 1.693550
O 4.714708 2.856681 -0.023434
C 4.311700 4.220203 -0.024613
C -4.583140 -0.412753 0.375335
C -5.200198 -1.519673 1.184685
C -5.228036 0.665909 -0.120017
C -6.689839 1.057490 -0.023453
C -7.638312 -0.000780 -0.617985
C -9.065956 0.502908 -0.789850
C -7.067926 1.498899 1.392505

H -1.200471 -3.081786 -0.771086
H -2.559675 1.424728 0.550769
H -0.231405 2.235591 0.377246
H 2.001070 3.096137 0.000002
H 2.238268 -2.593220 -1.269391
H 5.329490 -0.288083 -1.700284
H 4.094546 0.310482 1.967360
H 5.759599 0.535505 1.439737
H 6.414764 -3.062908 1.143882
H 6.434751 -1.943635 -0.238810
H 7.160215 -1.465037 1.328277
H 3.762144 4.466178 -0.938974
H 3.717486 4.451851 0.865024
H 5.214081 4.838063 0.003352
H -4.563366 -1.775693 2.039551
H -6.172321 -1.252738 1.601057
H -5.334291 -2.417636 0.572092
H -4.653900 1.371961 -0.722114
H -6.789183 1.952186 -0.655877
H -7.264143 -0.311206 -1.601986
H -7.666975 -0.900605 0.004433
H -9.092090 1.414081 -1.395662
H -9.672670 -0.255858 -1.294691
H -9.535497 0.713183 0.175513
H -8.023596 2.033372 1.391624
H -6.317457 2.182353 1.805767
H -7.165592 0.652263 2.077680
SCF Energy (B3LYP/6-31G**)= -1361.69848803
Number of imaginary frequencies = 0

1b_c278
MMFF Geometry
C -1.543665 -2.348768 -0.243743
C -2.267708 0.269652 -0.261757
N -2.866566 -2.055963 -0.210359
C -0.515036 -1.401786 -0.290679
C -0.902660 -0.050598 -0.303142
C -3.214241 -0.751369 -0.208380
C 0.084907 0.948425 -0.358674
C 1.449958 0.625079 -0.400479
C 1.835586 -0.719550 -0.379737
C 0.856930 -1.728302 -0.329131
C 3.279849 -1.065543 -0.432091
C 4.331043 0.068920 -0.510724
C 3.744955 1.466216 -0.520153
C 2.430398 1.707076 -0.448833
O 1.187924 -3.063907 -0.324424
O 3.593323 -2.259660 -0.472495
O 5.021991 -0.123723 -1.763763
C 5.375194 -0.136122 0.595014
C 4.825531 0.078868 1.988462
C 5.834919 0.353660 3.071774
O 3.625990 -0.012065 2.247178
O 4.613332 2.548292 -0.436006
C 5.377365 2.749423 -1.623879
C -4.670585 -0.473857 -0.171063
C -5.482447 -1.348450 -1.085490
C -5.159607 0.480659 0.650091
C -6.593667 0.903772 0.903394
C -7.120627 1.795802 -0.235180
C -8.477366 2.419295 0.064206
C -7.524099 -0.261149 1.254428
H -1.320855 -3.413634 -0.232262
H -2.590033 1.306066 -0.284052
H -0.219452 1.994721 -0.364564
H 2.071999 2.731171 -0.390474
H 2.169267 -3.142284 -0.337148
H 4.347138 -0.161048 -2.463726
H 6.218794 0.548921 0.443754
H 5.795372 -1.148809 0.554737
H 5.321830 0.493133 4.027328
H 6.520022 -0.493333 3.156612
H 6.388329 1.265590 2.834414
H 6.242584 2.080844 -1.641493
H 4.768129 2.622427 -2.525450
H 5.751772 3.777372 -1.609608
H -6.453741 -0.914247 -1.329459
H -4.970811 -1.493151 -2.044372
H -5.649402 -2.330985 -0.631781
H -4.453501 1.027661 1.276957
H -6.553620 1.529141 1.807599
H -6.404433 2.606669 -0.421266
H -7.196282 1.227957 -1.169263
H -8.453421 2.981605 1.002915
H -8.757371 3.110616 -0.737228
H -9.260903 1.658966 0.133720
H -8.426420 0.106432 1.755328
H -7.038841 -0.963536 1.941205
H -7.850241 -0.816126 0.370771
SCF Energy (B3LYP/6-31G**)= -1361.69420744
Number of imaginary frequencies = 0

1b_c279
MMFF Geometry
C 1.844543 2.015277 -0.016779
C 2.197286 -0.636524 -0.486098
N 3.110574 1.534063 -0.044622
C 0.695142 1.245588 -0.210913
C 0.889465 -0.126143 -0.452893
C 3.283510 0.212697 -0.278855
C -0.228729 -0.954923 -0.661449
C -1.532476 -0.441498 -0.619617
C -1.717602 0.919277 -0.366438
C -0.614847 1.764841 -0.177953
C -3.099025 1.440915 -0.307435
C -4.263908 0.451606 -0.114947
C -3.938537 -0.918596 -0.690083
C -2.670668 -1.320606 -0.896547
O -0.766565 3.112073 0.047258
O -3.287067 2.660661 -0.360153
O -5.375983 1.022923 -0.824133
C -4.588389 0.435324 1.383074
C -5.562112 -0.648205 1.790502
C -7.014397 -0.464161 1.433885
O -5.174881 -1.640688 2.411270
O -5.060108 -1.687495 -0.943468
C -4.890896 -3.030898 -1.377260
C 4.693226 -0.276733 -0.319209
C 4.961111 -1.470738 -1.192522
C 5.637057 0.382941 0.389282
C 7.119004 0.113555 0.557103
C 7.429376 -1.306805 1.067191
C 8.869877 -1.472486 1.535870
C 7.906489 0.502102 -0.696339
H 1.776592 3.084019 0.174787
H 2.356338 -1.695783 -0.659705
H -0.074680 -2.015213 -0.860305
H -2.437090 -2.304692 -1.287905
H -1.710510 3.352598 -0.088609
H -5.269910 1.994145 -0.780015
H -5.011993 1.395111 1.705357
H -3.675640 0.287801 1.975783
H -7.614190 -1.216417 1.954308
H -7.349648 0.525070 1.755526
H -7.162015 -0.580328 0.359120
H -4.394246 -3.066859 -2.352032
H -4.340620 -3.616107 -0.633481
H -5.884057 -3.475886 -1.488634
H 4.416602 -1.393206 -2.140702
H 6.012719 -1.570138 -1.465674
H 4.656824 -2.393831 -0.688276
H 5.314910 1.255906 0.962905
H 7.446882 0.802939 1.349282
H 6.766468 -1.543440 1.909246
H 7.236082 -2.056953 0.294278
H 9.121421 -0.735302 2.304778
H 9.011742 -2.469697 1.964998
H 9.575769 -1.365562 0.707125
H 8.978895 0.556740 -0.482619
H 7.600777 1.488453 -1.063505
H 7.768390 -0.216447 -1.509277
SCF Energy (B3LYP/6-31G**)= -1361.70294290

Number of imaginary frequencies = 0

1b_c280
MMFF Geometry
C 2.012521 1.629926 -0.276056
C 2.275050 -1.061877 -0.041326
N 3.263173 1.113503 -0.204107
C 0.835538 0.878196 -0.236222
C 0.984020 -0.513274 -0.113248
C 3.391501 -0.228340 -0.089184
C -0.163529 -1.324417 -0.064868
C -1.451924 -0.772699 -0.135835
C -1.598627 0.611926 -0.257137
C -0.459315 1.433982 -0.310422
C -2.961694 1.196669 -0.324421
C -4.199031 0.281913 -0.201117
C -3.859256 -1.191504 -0.145660
C -2.607717 -1.661645 -0.083294
O -0.553423 2.800922 -0.432042
O -3.065002 2.419565 -0.472386
O -4.922253 0.530277 -1.411703
C -4.963393 0.739849 1.054053
C -6.427655 0.370810 1.033823
C -6.925322 -0.566623 2.101265
O -7.195218 0.873366 0.211295
O -4.906260 -2.095661 -0.018618
C -5.590371 -2.304968 -1.256571
C 4.783934 -0.760467 -0.030572
C 4.994596 -2.173051 -0.505266
C 5.782002 0.039406 0.406311
C 7.248634 -0.299816 0.565582
C 8.061207 0.432912 -0.516929
C 9.537956 0.062294 -0.521183
C 7.699148 0.070027 1.982479
H 1.981038 2.713287 -0.370731
H 2.396752 -2.134819 0.064867
H -0.046270 -2.403936 0.028175
H -2.436381 -2.730962 0.006011
H -1.502921 3.044881 -0.523128
H -5.432647 1.354340 -1.287954
H -4.944422 1.832889 1.158331
H -4.493623 0.349173 1.965288
H -7.935190 -0.903439 1.851503
H -6.276538 -1.442239 2.169737
H -6.945390 -0.043772 3.060507
H -6.340675 -1.526148 -1.416781
H -4.898346 -2.357904 -2.104371
H -6.115625 -3.262269 -1.187180
H 4.413369 -2.372190 -1.413013
H 6.034113 -2.381785 -0.771285
H 4.694460 -2.887973 0.267817
H 5.533436 1.062396 0.697509
H 7.408379 -1.377095 0.454418
H 7.647729 0.192301 -1.505071
H 7.967479 1.519537 -0.394462
H 9.671347 -1.020366 -0.610661
H 10.040666 0.536395 -1.370414
H 10.039990 0.400760 0.389740
H 8.720837 -0.272444 2.174564
H 7.053944 -0.399787 2.733784
H 7.669891 1.153520 2.143976
SCF Energy (B3LYP/6-31G**)= -1361.70332289
Number of imaginary frequencies = 0

1b_c281
MMFF Geometry
C -1.548129 -1.649775 -0.841495
C -2.113479 0.919150 -0.164021
N -2.849259 -1.295420 -0.707601
C -0.464702 -0.787670 -0.657205
C -0.768757 0.540271 -0.306096
C -3.127517 -0.015615 -0.369207
C 0.278633 1.460614 -0.112749
C 1.618942 1.074421 -0.249336
C 1.912269 -0.251148 -0.582767
C 0.882119 -1.175289 -0.805332
C 3.332477 -0.637103 -0.722920
C 4.390686 0.235156 -0.007064
C 3.976410 1.705370 -0.011640
C 2.684558 2.072347 -0.108216
O 1.141085 -2.472638 -1.175881
O 3.606189 -1.644448 -1.377571
O 5.639116 0.103427 -0.699776
C 4.620734 -0.228207 1.433202
C 4.659851 -1.733976 1.552488
C 5.925641 -2.444594 1.153943
O 3.664599 -2.352024 1.934981
O 5.043219 2.575572 0.115428
C 4.785360 3.974291 0.156032
C -4.571868 0.340589 -0.246194
C -4.937044 1.765735 -0.554591
C -5.457255 -0.622046 0.096210
C -6.955423 -0.558224 0.315262
C -7.377979 0.496504 1.355909
C -8.825302 0.344358 1.807876
C -7.712862 -0.451139 -1.010264
H -1.393056 -2.692054 -1.112428
H -2.358231 1.937504 0.119114
H 0.038411 2.491554 0.144737
H 2.374862 3.111597 -0.091462
H 2.108999 -2.574568 -1.321162
H 5.550059 0.584939 -1.540604
H 3.816577 0.118900 2.095559
H 5.558932 0.167857 1.841056
H 5.898551 -3.472071 1.527768
H 6.017937 -2.463507 0.066321
H 6.788633 -1.941753 1.597448
H 4.319706 4.312816 -0.775045
H 4.166713 4.232501 1.021463
H 5.744301 4.490276 0.260743
H -4.388278 2.130551 -1.430557
H -5.993401 1.890505 -0.797486
H -4.709330 2.415500 0.296815
H -5.063593 -1.627728 0.266006
H -7.223884 -1.535942 0.742194
H -6.733133 0.413382 2.240081
H -7.249325 1.511233 0.966985
H -9.013095 -0.661337 2.196780
H -9.045949 1.061330 2.605355
H -9.523489 0.536360 0.987997
H -8.776839 -0.668500 -0.870949
H -7.330168 -1.171881 -1.741866
H -7.634937 0.547260 -1.450053
SCF Energy (B3LYP/6-31G**)= -1361.69422538
Number of imaginary frequencies = 0

1b_c282
MMFF Geometry
C -1.502647 -2.438335 -0.339984
C -2.292008 0.111157 0.169002
N -2.823890 -2.208918 -0.146952
C -0.505914 -1.457929 -0.296303
C -0.926899 -0.143097 -0.029956
C -3.206928 -0.937056 0.096032
C 0.028493 0.886170 0.033853
C 1.392635 0.628193 -0.166534
C 1.810312 -0.678929 -0.432848
C 0.866091 -1.718002 -0.497260
C 3.255644 -0.951215 -0.631872
C 4.272775 0.205363 -0.583329
C 3.652758 1.553553 -0.298676
C 2.344480 1.730728 -0.078512
O 1.231795 -3.019350 -0.750691
O 3.613649 -2.110022 -0.869168
O 4.783237 0.231598 -1.935976
C 5.439013 -0.158693 0.347390
C 5.156675 0.021707 1.824381
C 4.160052 -0.899831 2.476401
O 5.734756 0.885721 2.484276
O 4.493900 2.642545 -0.116384
C 5.161467 3.041432 -1.312259
C -4.660207 -0.731471 0.303798

C -5.324184 -1.794112 1.137788
C -5.291444 0.324467 -0.252909
C -6.767969 0.655780 -0.180635
C -6.961499 1.926879 0.665762
C -8.423300 2.279743 0.904090
C -7.318231 0.812428 -1.601647
H -1.254519 -3.478820 -0.538818
H -2.636559 1.116176 0.392802
H -0.300702 1.904065 0.242954
H 1.971763 2.719841 0.175128
H 2.203118 -3.055115 -0.899508
H 5.107228 -0.668138 -2.134132
H 6.315831 0.455276 0.101060
H 5.774016 -1.191999 0.192602
H 4.342828 -0.925714 3.554630
H 3.145839 -0.539763 2.297326
H 4.278738 -1.915041 2.089881
H 6.027689 2.401436 -1.502063
H 4.483560 3.046069 -2.172812
H 5.528264 4.061638 -1.164768
H -4.693929 -2.069349 1.991661
H -6.276365 -1.465556 1.562102
H -5.509009 -2.692952 0.540203
H -4.713787 1.022794 -0.859175
H -7.324343 -0.162115 0.287972
H -6.481779 1.787319 1.643360
H -6.462201 2.781256 0.191109
H -8.963302 1.438395 1.349600
H -8.496358 3.129919 1.590052
H -8.925322 2.562436 -0.025662
H -8.405965 0.933984 -1.593326
H -7.096456 -0.073794 -2.207352
H -6.884718 1.682655 -2.107266
SCF Energy (B3LYP/6-31G**)= -1361.69735030
Number of imaginary frequencies = 0

1b_c283
MMFF Geometry
C 1.860580 1.890015 -0.014985
C 2.127678 -0.806093 -0.215250
N 3.106125 1.363621 0.067687
C 0.691923 1.146470 -0.196207
C 0.842010 -0.248549 -0.301167
C 3.235013 0.020506 -0.030244
C -0.296105 -1.052868 -0.498333
C -1.577530 -0.490880 -0.573182
C -1.720197 0.893806 -0.450641
C -0.595431 1.713394 -0.281408
C -3.084017 1.466939 -0.508236
C -4.274724 0.529844 -0.200788
C -3.991612 -0.879087 -0.710707
C -2.736245 -1.344121 -0.848595
O -0.705790 3.080158 -0.190687
O -3.198270 2.671483 -0.742454
O -5.436170 1.057056 -0.851907
C -4.514389 0.600713 1.311830
C -5.477561 -0.450485 1.815156
C -6.948596 -0.234261 1.573775
O -5.064735 -1.457958 2.394296
O -5.138271 -1.599157 -0.991524
C -5.020913 -2.998514 -1.224156
C 4.618474 -0.525005 0.083295
C 4.749134 -1.897929 0.681374
C 5.660422 0.240050 -0.312046
C 7.146481 -0.055406 -0.342945
C 7.775474 0.117829 1.051289
C 9.296140 0.036843 1.042878
C 7.498644 -1.385549 -1.016098
H 1.826039 2.973838 0.073184
H 2.257099 -1.879597 -0.307514
H -0.175153 -2.131462 -0.594902
H -2.526943 -2.351757 -1.190919
H -1.631011 3.337540 -0.408998
H -5.346989 0.855260 -1.799793
H -4.910419 1.582010 1.602595
H -3.576922 0.471962 1.868738
H -7.524089 -0.918961 2.203360
H -7.221958 0.788956 1.843345
H -7.191927 -0.426215 0.527299
H -4.474522 -3.193948 -2.152144
H -4.545505 -3.499395 -0.374547
H -6.030003 -3.406088 -1.335380
H 4.561744 -2.667831 -0.074147
H 5.736018 -2.076359 1.112628
H 4.040926 -2.037787 1.506300
H 5.434213 1.239269 -0.693109
H 7.580112 0.721805 -0.989655
H 7.486648 1.094635 1.460555
H 7.389455 -0.636434 1.746296
H 9.724824 0.761924 0.343999
H 9.688831 0.256059 2.041055
H 9.642413 -0.962626 0.763537
H 8.548480 -1.389746 -1.329422
H 6.894399 -1.547791 -1.915538
H 7.359339 -2.241233 -0.350080
SCF Energy (B3LYP/6-31G**)= -1361.69655227
Number of imaginary frequencies = 0

1b_c284
MMFF Geometry
C 1.550441 2.409046 0.080746
C 2.261357 -0.118155 -0.619251
N 2.870803 2.131770 -0.043060
C 0.517266 1.488721 -0.124516
C 0.898019 0.185070 -0.488509
C 3.213913 0.870762 -0.382946
C -0.094603 -0.784526 -0.713918
C -1.456911 -0.477844 -0.581097
C -1.834670 0.817819 -0.217114
C -0.852783 1.797508 0.009737
C -3.276208 1.136113 -0.064339
C -4.338926 0.055211 -0.341347
C -3.760974 -1.283121 -0.738698
C -2.446625 -1.524570 -0.813788
O -1.177840 3.084001 0.371320
O -3.599336 2.285791 0.253732
O -5.040184 0.590693 -1.486950
C -5.336055 -0.012889 0.824753
C -4.851306 -0.786028 2.033475
C -3.728097 -0.201592 2.848636
O -5.366710 -1.856818 2.355768
O -4.627032 -2.356135 -0.897714
C -5.479567 -2.224909 -2.033839
C 4.668003 0.611936 -0.511143
C 5.435614 1.713288 -1.191858
C 5.209616 -0.521430 -0.015600
C 6.670501 -0.922712 -0.017724
C 7.126476 -1.193068 1.429330
C 8.617214 -1.478156 1.551884
C 6.851660 -2.143554 -0.922415
H 1.334651 3.437816 0.361419
H 2.576587 -1.113767 -0.916349
H 0.203709 -1.794186 -0.996410
H -2.097445 -2.525909 -1.052512
H -2.156935 3.169912 0.400992
H -5.337055 1.488599 -1.243212
H -6.271174 -0.479606 0.486867
H -5.634035 0.987698 1.162332
H -3.753334 -0.626609 3.856293
H -2.768686 -0.439998 2.387059
H -3.851280 0.880679 2.936110
H -6.327498 -1.572866 -1.806201
H -4.932964 -1.862482 -2.911337
H -5.877092 -3.216873 -2.268572
H 5.611545 2.544048 -0.500393
H 6.402892 1.380976 -1.576812
H 4.882602 2.095004 -2.058210
H 4.558672 -1.240367 0.482852
H 7.293006 -0.115205 -0.415164
H 6.894857 -0.318232 2.050885
H 6.567863 -2.036565 1.854782
H 9.209735 -0.670767 1.110332

H 8.896466 -1.564511 2.606990
H 8.888518 -2.417318 1.061262
H 7.909875 -2.398503 -1.036520
H 6.456204 -1.949436 -1.926104
H 6.333378 -3.021692 -0.520979
SCF Energy (B3LYP/6-31G**)= -1361.69746943
Number of imaginary frequencies = 0

1b_c285
MMFF Geometry
C 2.089972 -1.659289 -0.076907
C 2.326495 1.045652 -0.042041
N 3.335487 -1.127130 -0.034488
C 0.906758 -0.917525 -0.105677
C 1.041021 0.481934 -0.089767
C 3.450270 0.220795 -0.014517
C -0.113664 1.285330 -0.129864
C -1.395070 0.716824 -0.169721
C -1.522094 -0.673874 -0.169546
C -0.380589 -1.490067 -0.153866
C -2.882185 -1.256996 -0.211127
C -4.069516 -0.369563 0.226023
C -3.816684 1.099113 -0.102178
C -2.571709 1.583238 -0.269396
O -0.471111 -2.861628 -0.187995
O -2.990165 -2.440279 -0.544891
O -4.102824 -0.499035 1.654607
C -5.341426 -0.951410 -0.425161
C -6.627783 -0.403632 0.155899
C -7.790947 -0.249720 -0.786309
O -6.735909 -0.148435 1.357171
O -4.969620 1.858883 -0.180077
C -4.852278 3.274454 -0.269131
C 4.836502 0.768394 0.052176
C 5.001543 2.126336 0.679370
C 5.864505 0.025467 -0.414838
C 7.333659 0.381661 -0.461864
C 8.168406 -0.539026 0.447393
C 7.838266 -0.383156 1.926609
C 7.829037 0.277759 -1.908392
H 2.067854 -2.746925 -0.086961
H 2.439098 2.124703 -0.040295
H -0.006586 2.369400 -0.129109
H -2.382515 2.628994 -0.483289
H -1.416373 -3.107029 -0.311160
H -5.009268 -0.254440 1.941446
H -5.404055 -2.036719 -0.276366
H -5.324829 -0.768648 -1.506484
H -8.648899 0.162817 -0.248335
H -7.520648 0.436055 -1.593114
H -8.062326 -1.226178 -1.194637
H -4.298835 3.677382 0.585218
H -4.385446 3.567603 -1.214717
H -5.861312 3.696604 -0.247363
H 4.378548 2.221252 1.576236
H 6.024252 2.322804 1.010328
H 4.721058 2.913742 -0.027901
H 5.640814 -0.960976 -0.827297
H 7.499465 1.416740 -0.147594
H 8.030287 -1.588765 0.158780
H 9.233138 -0.313027 0.308746
H 6.812704 -0.695117 2.145775
H 8.509878 -1.004287 2.527976
H 7.960607 0.656021 2.248058
H 8.882363 0.570390 -1.978413
H 7.256076 0.939488 -2.567724
H 7.738639 -0.744207 -2.293947
SCF Energy (B3LYP/6-31G**)= -1361.71291869
Number of imaginary frequencies = 0

1b_c286
MMFF Geometry
C -1.666671 -1.583244 -0.922554
C -2.264234 0.944258 -0.127425
N -2.972888 -1.243975 -0.800386
C -0.593186 -0.725811 -0.669674
C -0.914372 0.580569 -0.258065
C -3.266687 0.015568 -0.403750
C 0.121688 1.494879 0.008294
C 1.466977 1.124436 -0.119456
C 1.778474 -0.180009 -0.515490
C 0.759694 -1.097883 -0.806271
C 3.203001 -0.547092 -0.659222
C 4.275606 0.318445 0.034085
C 3.812588 1.759076 0.162048
C 2.518833 2.110760 0.126885
O 1.033999 -2.374684 -1.233413
O 3.514886 -1.532723 -1.335386
O 5.433770 0.265084 -0.817442
C 4.664559 -0.213469 1.415853
C 4.702715 -1.721531 1.473463
C 5.993978 -2.405658 1.113439
O 3.692683 -2.361241 1.768841
O 4.748221 2.714319 0.535316
C 5.537212 3.159619 -0.567836
C -4.714922 0.357510 -0.294675
C -5.084310 1.791552 -0.551923
C -5.599963 -0.623091 -0.007408
C -7.100143 -0.573922 0.190756
C -7.618902 0.465926 1.203078
C -6.991983 0.329334 2.584928
C -7.834946 -0.465463 -1.147805
H -1.498774 -2.609243 -1.243134
H -2.522234 1.945282 0.202396
H -0.129362 2.509013 0.317638
H 2.230523 3.142098 0.310224
H 2.005145 -2.471250 -1.349005
H 5.338203 -0.535979 -1.372736
H 3.944030 0.104366 2.181438
H 5.644660 0.166294 1.729713
H 5.912115 -3.475303 1.326150
H 6.204175 -2.270762 0.050686
H 6.808877 -1.995242 1.714845
H 6.419818 2.525006 -0.684279
H 4.964156 3.197968 -1.501107
H 5.884579 4.172248 -0.341504
H -4.506805 2.202403 -1.388137
H -6.131775 1.914861 -0.832096
H -4.897359 2.403640 0.336480
H -5.203730 -1.633055 0.126443
H -7.373204 -1.555683 0.605711
H -7.470944 1.487851 0.841573
H -8.704096 0.339233 1.309677
H -5.920387 0.548573 2.565430
H -7.461612 1.032524 3.280332
H -7.132106 -0.681309 2.981390
H -8.913223 -0.597301 -1.005428
H -7.497322 -1.239836 -1.845787
H -7.681633 0.506831 -1.625193
SCF Energy (B3LYP/6-31G**)= -1361.69374023
Number of imaginary frequencies = 0

1b_c287
MMFF Geometry
C -1.993901 1.703122 -0.135485
C -2.283275 -0.991114 -0.300118
N -3.244684 1.202094 -0.278702
C -0.829508 0.934077 -0.067016
C -0.991911 -0.458643 -0.152449
C -3.386439 -0.140844 -0.360493
C 0.142176 -1.287268 -0.086082
C 1.431076 -0.751637 0.059658
C 1.591893 0.634318 0.141428
C 0.465691 1.473822 0.081950
C 2.956094 1.201759 0.287648
C 4.183523 0.265599 0.288608
C 3.823861 -1.203890 0.261644
C 2.572445 -1.658233 0.124495
O 0.573566 2.842783 0.162462
O 3.068964 2.427719 0.398651
O 4.820798 0.548642 1.539206
C 5.044895 0.665521 -0.922793

C 6.497413 0.276987 -0.781684
C 7.055719 -0.706884 -1.774750
O 7.211372 0.799374 0.076008
O 4.861932 -2.126916 0.244762
C 5.450036 -2.299714 1.536521
C -4.777150 -0.653421 -0.531423
C -4.932978 -1.987077 -1.211113
C -5.817053 0.097122 -0.104631
C -7.293522 -0.233234 -0.155420
C -7.896884 -0.086959 1.255429
C -9.352584 -0.525523 1.340762
C -7.976340 0.678423 -1.177061
H -1.951398 2.788552 -0.074990
H -2.417148 -2.066573 -0.352626
H 0.013797 -2.367739 -0.148293
H 2.390035 -2.727608 0.061780
H 1.517989 3.076457 0.313521
H 5.352264 1.360486 1.423687
H 5.051863 1.754058 -1.067736
H 4.636334 0.247707 -1.851323
H 8.038805 -1.048413 -1.439251
H 6.398930 -1.575139 -1.858842
H 7.154610 -0.220784 -2.748325
H 6.199740 -1.526262 1.723343
H 4.697081 -2.311128 2.332382
H 5.962620 -3.266344 1.540635
H -4.722405 -2.802699 -0.511612
H -5.935321 -2.143436 -1.617873
H -4.252494 -2.072872 -2.066153
H -5.600538 1.066459 0.350104
H -7.446332 -1.269892 -0.471325
H -7.316202 -0.694483 1.961869
H -7.819097 0.952679 1.598454
H -9.476540 -1.550649 0.977893
H -9.693645 -0.491288 2.380567
H -10.005048 0.131146 0.758171
H -9.024297 0.397437 -1.320760
H -7.486136 0.607405 -2.154863
H -7.947392 1.727596 -0.861973
SCF Energy (B3LYP/6-31G**)= -1361.70348484
Number of imaginary frequencies = 0

1b_c288
MMFF Geometry
C -1.409016 -2.107286 -0.771914
C -2.293565 0.396011 -0.188768
N -2.746785 -1.911700 -0.679202
C -0.441675 -1.113666 -0.591040
C -0.911775 0.179548 -0.292255
C -3.174496 -0.666728 -0.379048
C 0.010210 1.227198 -0.112948
C 1.389428 1.002771 -0.211679
C 1.850284 -0.288560 -0.491341
C 0.945619 -1.338898 -0.698382
C 3.309167 -0.502003 -0.602575
C 4.263125 0.530467 0.030775
C 3.645320 1.915533 0.006740
C 2.321303 2.122032 -0.065901
O 1.367661 -2.607511 -1.014881
O 3.739675 -1.494640 -1.197612
O 5.453595 0.541005 -0.776330
C 4.654804 0.172093 1.466148
C 4.875330 -1.309181 1.655477
C 6.252155 -1.854757 1.387720
O 3.940854 -2.043323 1.978967
O 4.503634 2.978405 0.244982
C 4.855134 3.639673 -0.970964
C -4.644926 -0.496021 -0.289686
C -5.407286 -1.184699 -1.387389
C -5.186662 0.211174 0.725667
C -6.642603 0.474527 1.058526
C -7.231673 1.570793 0.152463
C -8.622531 2.022882 0.577019
C -7.496423 -0.795384 1.124051
H -1.121240 -3.130054 -1.006390
H -2.680666 1.387653 0.024413
H -0.357945 2.229253 0.104885
H 1.918083 3.128132 0.003768
H 2.344421 -2.601975 -1.123472
H 5.458358 -0.298117 -1.280404
H 3.868757 0.459907 2.177107
H 5.567046 0.696490 1.776142
H 6.298231 -2.900558 1.704392
H 6.475327 -1.797090 0.320624
H 6.991321 -1.288344 1.959563
H 5.325420 2.953733 -1.682331
H 3.977300 4.110990 -1.425699
H 5.575763 4.426268 -0.728808
H -5.510136 -2.253731 -1.173632
H -6.405160 -0.766708 -1.532274
H -4.894102 -1.072635 -2.349807
H -4.511879 0.641642 1.467253
H -6.635431 0.875582 2.082921
H -6.568576 2.445568 0.164157
H -7.278129 1.230324 -0.888018
H -8.627306 2.353713 1.620316
H -8.950383 2.861764 -0.045407
H -9.356761 1.219901 0.463049
H -8.416381 -0.610054 1.689322
H -6.963762 -1.605838 1.633875
H -7.793525 -1.150526 0.133687
SCF Energy (B3LYP/6-31G**)= -1361.69315077
Number of imaginary frequencies = 0

1b_c289
MMFF Geometry
C 2.067874 -1.777274 -0.307063
C 2.358599 0.918573 -0.453515
N 3.323640 -1.272594 -0.375409
C 0.899872 -1.011285 -0.306783
C 1.062066 0.383192 -0.383098
C 3.465664 0.070949 -0.447466
C -0.076858 1.209714 -0.392909
C -1.368906 0.669843 -0.315442
C -1.522690 -0.715287 -0.226489
C -0.398724 -1.555558 -0.237462
C -2.893573 -1.268179 -0.145310
C -4.032003 -0.330242 0.315801
C -3.772719 1.111578 -0.111443
C -2.532433 1.556468 -0.387214
O -0.517900 -2.924325 -0.184368
O -3.046284 -2.465706 -0.401917
O -3.974052 -0.379010 1.748611
C -5.355039 -0.917781 -0.217814
C -6.589581 -0.308833 0.412666
C -7.808783 -0.180602 -0.459975
O -6.613726 0.015218 1.601896
O -4.913109 1.892643 -0.156715
C -4.774000 3.298202 -0.331633
C 4.862635 0.587571 -0.534004
C 5.055973 1.921351 -1.203257
C 5.875973 -0.158560 -0.041037
C 7.350158 0.176313 0.001615
C 7.914531 0.053141 1.430655
C 7.304258 1.054430 2.403882
C 8.106964 -0.759110 -0.944405
H 2.024065 -2.862781 -0.249649
H 2.492676 1.994239 -0.498439
H 0.051127 2.289323 -0.461750
H -2.337534 2.584052 -0.672219
H -1.473801 -3.154407 -0.231578
H -4.854709 -0.097670 2.078805
H -5.428968 -1.991376 -0.003612
H -5.405859 -0.795953 -1.306627
H -8.621401 0.280995 0.107709
H -7.578612 0.452802 -1.320267
H -8.125369 -1.171694 -0.793684
H -4.157017 3.733674 0.460997
H -4.365493 3.528809 -1.320542
H -5.770803 3.744393 -0.267355
H 4.800061 2.736351 -0.518270
H 6.080995 2.081118 -1.547117

H 4.429317 2.004848 -2.098697
H 5.634329 -1.126356 0.404098
H 7.531165 1.202786 -0.332170
H 7.760368 -0.962167 1.817499
H 8.999082 0.217638 1.404745
H 6.234496 0.875313 2.548135
H 7.790138 0.971087 3.381352
H 7.438173 2.080214 2.045966
H 9.175415 -0.517837 -0.954893
H 7.735226 -0.663667 -1.970820
H 8.002469 -1.807827 -0.643601
SCF Energy (B3LYP/6-31G**)= -1361.71302486
Number of imaginary frequencies = 0

1b_c290
MMFF Geometry
C -1.730365 -1.414314 -0.737019
C -2.201506 1.074389 0.243744
N -3.015949 -1.047657 -0.515585
C -0.617847 -0.603616 -0.499277
C -0.873767 0.683845 0.006812
C -3.246778 0.191837 -0.025054
C 0.203533 1.553929 0.257246
C 1.526713 1.156370 0.023832
C 1.774139 -0.130662 -0.463825
C 0.712353 -1.002669 -0.741639
C 3.175550 -0.526128 -0.717638
C 4.314102 0.269381 -0.045927
C 3.903063 1.711885 0.191200
C 2.620685 2.098932 0.257818
O 0.921433 -2.258652 -1.258088
O 3.415720 -1.478898 -1.466290
O 5.415811 0.234871 -0.970207
C 4.771136 -0.351305 1.276710
C 4.766283 -1.860652 1.247955
C 6.010903 -2.557981 0.768742
O 3.757324 -2.488406 1.571334
O 4.888434 2.618840 0.556778
C 5.615007 3.111150 -0.569075
C -4.672993 0.552845 0.224926
C -4.929320 1.528121 1.339647
C -5.636998 -0.029635 -0.522518
C -7.140492 0.148460 -0.510376
C -7.841355 -0.532689 0.679690
C -7.574899 -2.030788 0.762015
C -7.582393 1.607732 -0.659523
H -1.613498 -2.423038 -1.127464
H -2.409950 2.069591 0.622525
H 0.002412 2.554751 0.638161
H 2.375118 3.125272 0.516103
H 1.880411 -2.374032 -1.439370
H 5.262406 -0.529763 -1.562630
H 4.109408 -0.058412 2.102927
H 5.779971 -0.017017 1.548421
H 5.909389 -3.635454 0.926332
H 6.158720 -2.367958 -0.296089
H 6.873896 -2.205271 1.338857
H 6.466924 2.461051 -0.785558
H 4.981608 3.224562 -1.456091
H 6.009118 4.097349 -0.305649
H -4.743004 2.554029 1.005460
H -5.951118 1.479147 1.719308
H -4.285092 1.317844 2.201227
H -5.317568 -0.748027 -1.281986
H -7.502102 -0.355575 -1.419062
H -7.545693 -0.068194 1.626428
H -8.924607 -0.383247 0.587438
H -6.519429 -2.241474 0.959342
H -8.159259 -2.471144 1.576375
H -7.860817 -2.531978 -0.168173
H -8.652139 1.656074 -0.892835
H -7.044763 2.101812 -1.476287
H -7.427020 2.190046 0.252377
SCF Energy (B3LYP/6-31G**)= -1361.69386490
Number of imaginary frequencies = 0

1b_c291
MMFF Geometry
C 1.905061 2.389696 0.127721
C 2.545650 -0.146446 -0.605017
N 3.216076 2.086398 -0.032288
C 0.848322 1.491629 -0.054568
C 1.192602 0.183062 -0.435578
C 3.524632 0.821341 -0.388395
C 0.174391 -0.764248 -0.640836
C -1.177769 -0.431302 -0.468131
C -1.520009 0.868782 -0.085394
C -0.511308 1.827370 0.116815
C -2.950267 1.218928 0.105140
C -4.037315 0.135830 -0.062762
C -3.498207 -1.193156 -0.544944
C -2.194009 -1.453269 -0.695487
O -0.799954 3.119959 0.488556
O -3.230226 2.385165 0.404441
O -4.889516 0.670494 -1.081679
C -4.744976 0.005543 1.298006
C -6.142869 -0.556838 1.196683
C -6.416855 -1.871105 1.877484
O -7.038654 0.073419 0.632208
O -4.398655 -2.237774 -0.711679
C -5.151745 -2.111699 -1.920526
C 4.969503 0.530593 -0.559685
C 5.740474 1.624509 -1.249548
C 5.494891 -0.616228 -0.077073
C 6.934425 -1.078114 -0.148471
C 7.665667 -0.886080 1.194802
C 7.116965 -1.691605 2.366253
C 6.993241 -2.528082 -0.639216
H 1.717129 3.421048 0.418973
H 2.832489 -1.145987 -0.917151
H 0.444674 -1.776542 -0.940824
H -1.872683 -2.446497 -0.997225
H -1.778059 3.227098 0.523630
H -5.495068 1.308794 -0.656480
H -4.863651 0.983460 1.783349
H -4.150326 -0.598377 1.994663
H -7.390368 -2.253279 1.558305
H -5.653371 -2.604004 1.608675
H -6.423887 -1.722647 2.959996
H -6.011168 -1.452920 -1.770211
H -4.534082 -1.760181 -2.754429
H -5.535192 -3.103811 -2.177068
H 5.786455 2.521560 -0.622364
H 6.769792 1.347938 -1.485878
H 5.261168 1.889387 -2.198975
H 4.838360 -1.303218 0.457455
H 7.481980 -0.488580 -0.890737
H 8.722954 -1.148316 1.061869
H 7.645605 0.177628 1.465034
H 7.182659 -2.767397 2.180299
H 7.699667 -1.477394 3.268379
H 6.074586 -1.437307 2.578974
H 8.028660 -2.883285 -0.675529
H 6.578176 -2.610225 -1.650335
H 6.424049 -3.206498 0.005209
SCF Energy (B3LYP/6-31G**)= -1361.70197874
Number of imaginary frequencies = 0

1b_c292
MMFF Geometry
C -1.734559 -2.527765 -0.121562
C -2.535065 0.046026 0.224497
N -3.055019 -2.293290 0.071556
C -0.744381 -1.540566 -0.154279
C -1.170389 -0.212626 0.028141
C -3.443624 -1.010422 0.232799
C -0.221109 0.825282 0.007713
C 1.143504 0.561843 -0.183680
C 1.562161 -0.758795 -0.359143
C 0.625688 -1.805239 -0.355657
C 3.002163 -1.028875 -0.559199
C 4.029057 0.080211 -0.264066

C 3.421092 1.473769 -0.264017
C 2.091875 1.676238 -0.224547
O 1.000950 -3.114765 -0.541100
O 3.348690 -2.154579 -0.931016
O 4.960476 -0.004380 -1.353741
C 4.703499 -0.228177 1.082416
C 6.213163 -0.198345 1.025818
C 6.900296 1.140246 1.009814
O 6.858621 -1.246972 0.968263
O 4.369469 2.481074 -0.277137
C 3.940303 3.824408 -0.463238
C -4.895576 -0.799764 0.443225
C -5.544402 -1.807162 1.354313
C -5.540643 0.214766 -0.171613
C -7.018549 0.534150 -0.099453
C -7.300000 1.727299 0.834254
C -6.683034 3.054634 0.409995
C -7.577580 0.755803 -1.508452
H -1.481191 -3.577627 -0.253921
H -2.884710 1.061493 0.383286
H -0.558012 1.852855 0.142191
H 1.665123 2.673202 -0.209756
H 1.956010 -3.141491 -0.778926
H 5.211538 -0.947400 -1.436180
H 4.426209 -1.229093 1.440725
H 4.369764 0.447978 1.879682
H 6.881376 1.556824 0.001004
H 6.410797 1.820303 1.711380
H 7.939988 1.017308 1.326567
H 3.382339 3.932963 -1.398910
H 3.346287 4.164821 0.390725
H 4.831319 4.456064 -0.525841
H -4.905860 -2.016967 2.220470
H -6.496588 -1.456108 1.761058
H -5.726357 -2.746857 0.822329
H -4.974297 0.873857 -0.829452
H -7.564663 -0.324582 0.307190
H -8.385728 1.862433 0.918559
H -6.942089 1.485192 1.843298
H -7.065423 3.383653 -0.560446
H -6.932235 3.829967 1.142100
H -5.592336 2.993355 0.352487
H -8.643224 1.005430 -1.467641
H -7.472057 -0.152582 -2.112654
H -7.059835 1.563386 -2.036843
SCF Energy (B3LYP/6-31G**)= -1361.70186953
Number of imaginary frequencies = 0

1b_c293
MMFF Geometry
C -1.596960 -2.160410 -0.452501
C -2.225011 0.383314 0.265183
N -2.899712 -1.871060 -0.215803
C -0.543618 -1.244698 -0.358396
C -0.881343 0.067894 0.014559
C -3.201103 -0.602305 0.133658
C 0.133851 1.033589 0.129705
C 1.477113 0.715583 -0.123714
C 1.813308 -0.591946 -0.490468
C 0.806658 -1.566709 -0.610253
C 3.233592 -0.929956 -0.767756
C 4.314830 0.172158 -0.647477
C 3.782063 1.529982 -0.236963
C 2.488690 1.759939 0.018912
O 1.087804 -2.861570 -0.981337
O 3.498064 -2.075286 -1.147339
O 4.885088 0.307039 -1.967025
C 5.444146 -0.329131 0.262643
C 5.027746 -0.480471 1.709612
C 6.137875 -0.508838 2.726827
O 3.853380 -0.623881 2.047856
O 4.693745 2.541015 0.043570
C 5.357245 3.037577 -1.117653
C -4.636042 -0.328437 0.393015
C -5.320926 -1.395994 1.200617
C -5.213499 0.786784 -0.104592
C -6.649377 1.265511 -0.012817
C -7.657823 0.265900 -0.610166
C -9.052064 0.854394 -0.786739
C -7.004775 1.729387 1.401835
H -1.412340 -3.195598 -0.731817
H -2.505865 1.385427 0.574196
H -0.131117 2.048369 0.425370
H 2.174834 2.737009 0.375926
H 2.060413 -2.945433 -1.109145
H 4.150147 0.461926 -2.585603
H 6.295893 0.360892 0.215518
H 5.821424 -1.300950 -0.079308
H 5.716444 -0.618952 3.729857
H 6.798226 -1.355433 2.523919
H 6.699179 0.427775 2.683084
H 6.192352 2.386362 -1.390466
H 4.666818 3.159511 -1.959418
H 5.769061 4.020913 -0.871644
H -4.703557 -1.689091 2.057850
H -6.276827 -1.071221 1.613439
H -5.506293 -2.284711 0.587908
H -4.595644 1.457470 -0.703850
H -6.692776 2.164298 -0.645752
H -7.299839 -0.066852 -1.592891
H -7.742502 -0.630323 0.012348
H -9.021456 1.765245 -1.392826
H -9.701654 0.133252 -1.293373
H -9.511214 1.092893 0.177060
H -7.926649 2.320231 1.397808
H -6.215961 2.366754 1.817332
H -7.155210 0.890406 2.086875
SCF Energy (B3LYP/6-31G**)= -1361.69440849
Number of imaginary frequencies = 0

1b_c294
MMFF Geometry
C 1.717821 -1.711567 -0.147874
C 2.266284 0.948493 -0.106202
N 3.016947 -1.327916 -0.114580
C 0.628507 -0.837111 -0.162794
C 0.923978 0.538124 -0.141234
C 3.285959 -0.002258 -0.093255
C -0.129802 1.470934 -0.162069
C -1.467780 1.054231 -0.188593
C -1.753084 -0.312428 -0.193782
C -0.716498 -1.256561 -0.200326
C -3.171303 -0.727510 -0.223036
C -4.229677 0.271265 0.305176
C -3.828957 1.702247 -0.066323
C -2.543022 2.045213 -0.274934
O -0.969220 -2.606874 -0.245500
O -3.432595 -1.862767 -0.627140
O -4.248780 0.236278 1.735665
C -5.612511 -0.093268 -0.273327
C -6.099653 -1.446374 0.206997
C -6.952512 -2.243668 -0.741934
O -5.854224 -1.847244 1.347073
O -4.895407 2.584551 -0.115239
C -4.625223 3.974199 -0.256206
C 4.726729 0.383590 -0.071420
C 5.081449 1.684198 -0.736249
C 5.622012 -0.453778 0.498332
C 7.118416 -0.315406 0.693396
C 7.880573 -0.660833 -0.598631
C 9.389302 -0.740468 -0.407703
C 7.539642 1.017973 1.318777
H 1.569509 -2.789221 -0.162440
H 2.505233 2.006483 -0.074683
H 0.101259 2.535449 -0.157032
H -2.245656 3.060850 -0.508953
H -1.932777 -2.740267 -0.392032
H -4.677506 -0.608376 1.995686
H -5.574353 -0.098849 -1.369270
H -6.384436 0.619013 0.041805
H -7.224002 -3.197225 -0.280762
H -7.863863 -1.686017 -0.970633

H -6.392018 -2.444620 -1.658259
H -3.999304 4.338092 0.565021
H -4.164041 4.185195 -1.226223
H -5.579405 4.507771 -0.214842
H 4.895058 2.526266 -0.061814
H 6.125968 1.724370 -1.051038
H 4.495551 1.832213 -1.650816
H 5.240457 -1.390199 0.913604
H 7.382939 -1.081980 1.436848
H 7.531504 -1.630260 -0.977353
H 7.666151 0.074230 -1.382628
H 9.648733 -1.450826 0.383518
H 9.867245 -1.076073 -1.333748
H 9.812665 0.235364 -0.151987
H 8.539872 0.935554 1.758196
H 6.857992 1.311632 2.124723
H 7.576497 1.829866 0.587580
SCF Energy (B3LYP/6-31G**)= -1361.70631235
Number of imaginary frequencies = 0

1b_c295
MMFF Geometry
C -1.422784 -2.379264 -0.295129
C -2.230891 0.203680 -0.044641
N -2.741441 -2.143432 -0.092830
C -0.438194 -1.390145 -0.383844
C -0.868438 -0.056348 -0.251280
C -3.133360 -0.855950 0.021514
C 0.073337 0.985524 -0.331608
C 1.433927 0.717738 -0.527770
C 1.856837 -0.607890 -0.651113
C 0.929664 -1.658111 -0.593088
C 3.302561 -0.873519 -0.827250
C 4.319376 0.232639 -0.470737
C 3.708950 1.615215 -0.631286
C 2.383845 1.819022 -0.663510
O 1.315739 -2.970634 -0.726383
O 3.670987 -1.996170 -1.187898
O 5.389538 0.099689 -1.429284
C 4.916606 -0.110914 0.903849
C 3.895254 -0.191433 2.015685
C 3.757631 -1.511745 2.727377
O 3.209947 0.778049 2.334647
O 4.519115 2.700283 -0.946229
C 5.537751 2.981227 0.003785
C -4.583010 -0.642225 0.245830
C -5.207969 -1.606770 1.217396
C -5.241229 0.335880 -0.412496
C -6.715690 0.668545 -0.335009
C -6.945651 2.076978 0.243563
C -6.494567 2.211898 1.692682
C -7.327926 0.570847 -1.736235
H -1.166265 -3.432461 -0.386934
H -2.582326 1.223646 0.078200
H -0.261033 2.018208 -0.236488
H 1.993032 2.818929 -0.830247
H 2.253231 -2.995860 -1.021908
H 5.448974 -0.848295 -1.659196
H 5.694716 0.593335 1.212119
H 5.433412 -1.078725 0.843899
H 2.974906 -1.440246 3.487654
H 3.481103 -2.289290 2.011155
H 4.702495 -1.764698 3.214418
H 5.150165 2.955720 1.027611
H 6.378556 2.292714 -0.116208
H 5.907929 3.992347 -0.190617
H -4.556990 -1.759297 2.086305
H -6.161459 -1.251365 1.615540
H -5.380344 -2.576997 0.739681
H -4.687882 0.959641 -1.115203
H -7.250220 -0.050478 0.293467
H -6.427101 2.829454 -0.364059
H -8.015304 2.316420 0.194135
H -5.412002 2.087044 1.791081
H -6.750075 3.205875 2.073765
H -6.988107 1.469193 2.327527
H -8.404800 0.768848 -1.702435
H -7.187579 -0.431405 -2.156534
H -6.875732 1.292768 -2.425846
SCF Energy (B3LYP/6-31G**)= -1361.69672767
Number of imaginary frequencies = 0

1b_c296
MMFF Geometry
C -1.465510 -1.988166 -0.637066
C -2.252911 0.434467 0.311203
N -2.785005 -1.799988 -0.392131
C -0.470756 -1.025421 -0.439064
C -0.890245 0.225070 0.053776
C -3.166028 -0.590556 0.071459
C 0.061711 1.237846 0.272613
C 1.421925 1.020301 0.017943
C 1.833499 -0.229465 -0.458309
C 0.896618 -1.242702 -0.703358
C 3.272016 -0.435142 -0.731358
C 4.297528 0.507379 -0.070233
C 3.703103 1.888121 0.131989
C 2.381646 2.108634 0.209927
O 1.267004 -2.466452 -1.206238
O 3.628982 -1.349967 -1.479953
O 5.407984 0.610377 -0.978533
C 4.814680 -0.025694 1.267889
C 5.027275 -1.520122 1.254779
C 6.364145 -2.035908 0.794620
O 4.114817 -2.283535 1.573363
O 4.597232 2.909548 0.415502
C 4.845572 3.712493 -0.739028
C -4.617386 -0.428432 0.333197
C -5.244234 -1.606814 1.025679
C -5.256606 0.692553 -0.066267
C -6.719884 1.073832 0.047444
C -7.659457 0.077058 -0.657292
C -9.085105 0.595375 -0.799714
C -7.117179 1.379240 1.493684
H -1.216554 -2.978728 -1.012113
H -2.598155 1.384243 0.707960
H -0.268097 2.207552 0.644091
H 2.003309 3.100665 0.438853
H 2.228979 -2.452018 -1.406924
H 5.352135 -0.160931 -1.578704
H 4.102843 0.177119 2.079261
H 5.760295 0.452231 1.551916
H 6.422061 -3.112801 0.976422
H 6.488382 -1.849359 -0.273832
H 7.162396 -1.547438 1.358879
H 5.236765 3.116161 -1.569134
H 3.937286 4.240157 -1.049038
H 5.598290 4.460050 -0.472102
H -4.618732 -1.942877 1.860967
H -6.222120 -1.380190 1.452323
H -5.369239 -2.442668 0.329038
H -4.675009 1.452530 -0.590540
H -6.811555 2.024466 -0.498634
H -7.271940 -0.138898 -1.661193
H -7.695665 -0.877680 -0.123370
H -9.103922 1.559860 -1.316765
H -9.684395 -0.111884 -1.382231
H -9.567654 0.713438 0.174872
H -8.073221 1.911761 1.530704
H -6.372878 2.020172 1.979815
H -7.223209 0.471528 2.094196
SCF Energy (B3LYP/6-31G**)= -1361.69347058
Number of imaginary frequencies = 0

1b_c297
MMFF Geometry
C 1.784995 2.451238 0.110341
C 2.396929 -0.175956 -0.230132
N 3.090299 2.092188 0.160468
C 0.721036 1.568600 -0.103487
C 1.050285 0.211492 -0.281191
C 3.380776 0.783688 -0.001986

C 0.025518 -0.724184 -0.513509
C -1.319691 -0.332572 -0.554624
C -1.640699 1.013015 -0.362520
C -0.630858 1.963806 -0.154057
C -3.065206 1.405527 -0.390297
C -4.143258 0.319184 -0.215746
C -3.665467 -1.033052 -0.722935
C -2.357332 -1.322147 -0.853038
O -0.916535 3.298155 0.009661
O -3.360836 2.599970 -0.499078
O -5.265747 0.760714 -0.997492
C -4.540015 0.322506 1.264851
C -5.428974 -0.833508 1.666759
C -6.872231 -0.798670 1.235511
O -4.983672 -1.763867 2.342385
O -4.697862 -1.910645 -1.001006
C -4.384828 -3.245513 -1.377452
C 4.819110 0.432848 0.057888
C 5.701076 1.319437 -0.773586
C 5.224767 -0.603528 0.822791
C 6.607943 -1.141732 1.135018
C 6.904097 -2.441328 0.360340
C 6.996731 -2.288533 -1.152324
C 7.774131 -0.151748 1.081371
H 1.608131 3.515271 0.252782
H 2.679236 -1.213858 -0.378734
H 0.286225 -1.771460 -0.664330
H -2.015170 -2.292564 -1.195676
H -1.870531 3.444432 -0.179726
H -5.250526 1.738517 -0.985980
H -5.065991 1.248488 1.530184
H -3.648642 0.281207 1.905106
H -7.425312 -1.586972 1.754318
H -7.313320 0.164420 1.504438
H -6.954159 -0.962725 0.159978
H -3.837872 -3.265867 -2.325378
H -3.822132 -3.753313 -0.587521
H -5.326253 -3.784166 -1.520146
H 6.126250 2.123621 -0.165020
H 6.508652 0.754151 -1.244666
H 5.148114 1.778493 -1.601766
H 4.455922 -1.152483 1.371179
H 6.551233 -1.433439 2.195047
H 7.845717 -2.869595 0.726435
H 6.124485 -3.180839 0.585168
H 7.802502 -1.607783 -1.440370
H 7.206819 -3.260518 -1.610919
H 6.059356 -1.919255 -1.578313
H 8.654604 -0.584893 1.570168
H 7.529955 0.774041 1.613325
H 8.074811 0.103926 0.063453
SCF Energy (B3LYP/6-31G**)= -1361.70001029
Number of imaginary frequencies = 0

1b_c298
MMFF Geometry
C 2.105761 1.518940 -0.199518
C 2.301117 -1.184545 -0.048640
N 3.342942 0.969744 -0.138693
C 0.910544 0.795627 -0.188259
C 1.024275 -0.602242 -0.108604
C 3.437964 -0.377825 -0.065472
C -0.143061 -1.385938 -0.090646
C -1.417175 -0.800650 -0.149547
C -1.529278 0.590298 -0.227714
C -0.369899 1.385260 -0.250293
C -2.877243 1.210316 -0.282205
C -4.137236 0.322939 -0.192970
C -3.833976 -1.159427 -0.182618
C -2.594667 -1.662027 -0.129903
O -0.429840 2.757251 -0.329141
O -2.949794 2.439434 -0.391957
O -4.848171 0.627008 -1.398112
C -4.896318 0.759905 1.072834
C -6.369069 0.427898 1.034895
C -6.894813 -0.530080 2.070153
O -7.120026 0.974839 0.225460
O -4.903453 -2.041031 -0.088535
C -5.586379 -2.194356 -1.335311
C 4.816776 -0.945344 -0.018142
C 4.994772 -2.351319 -0.525113
C 5.835164 -0.180705 0.434838
C 7.290134 -0.566163 0.583974
C 8.150928 -0.013335 -0.568350
C 8.211219 1.505736 -0.674065
C 7.810772 -0.129873 1.957300
H 2.101384 2.605181 -0.260159
H 2.395635 -2.262995 0.023874
H -0.052787 -2.470414 -0.031224
H -2.450052 -2.737524 -0.073674
H -1.372605 3.027309 -0.416442
H -5.338775 1.459132 -1.250557
H -4.851073 1.848327 1.211613
H -4.440761 0.329318 1.973236
H -7.911388 -0.833884 1.805694
H -6.268026 -1.423141 2.113659
H -6.906790 -0.037310 3.045322
H -6.316564 -1.392616 -1.473968
H -4.891688 -2.237644 -2.181477
H -6.135280 -3.140123 -1.298360
H 4.418962 -2.512252 -1.443780
H 6.031873 -2.581347 -0.783862
H 4.667034 -3.076508 0.226915
H 5.610983 0.840418 0.749938
H 7.387901 -1.657580 0.561858
H 9.174766 -0.392761 -0.458954
H 7.776295 -0.412640 -1.519684
H 8.651843 1.953747 0.221132
H 8.834724 1.793440 -1.527112
H 7.219121 1.939767 -0.829001
H 8.868768 -0.389368 2.070874
H 7.257298 -0.634114 2.757793
H 7.707004 0.948826 2.115731
SCF Energy (B3LYP/6-31G**)= -1361.70248104
Number of imaginary frequencies = 0

1b_c299
MMFF Geometry
C -1.575936 -2.475605 -0.351808
C -2.370125 0.065254 0.191565
N -2.896692 -2.252729 -0.148030
C -0.582043 -1.492545 -0.302894
C -1.005510 -0.182256 -0.018712
C -3.282189 -0.985012 0.111928
C -0.053041 0.849309 0.051201
C 1.310640 0.598100 -0.160548
C 1.730807 -0.704486 -0.444598
C 0.789511 -1.745808 -0.515278
C 3.175739 -0.969751 -0.655646
C 4.189474 0.189482 -0.599914
C 3.566952 1.532260 -0.295811
C 2.259505 1.702624 -0.065570
O 1.157774 -3.042935 -0.786049
O 3.535949 -2.124559 -0.908586
O 4.691484 0.233064 -1.955274
C 5.362590 -0.181559 0.319332
C 5.088807 -0.019232 1.800027
C 4.099173 -0.951518 2.447396
O 5.668194 0.838946 2.466360
O 4.405745 2.621788 -0.106048
C 5.064633 3.036688 -1.301289
C -4.734549 -0.786411 0.331119
C -5.391883 -1.860885 1.155243
C -5.372616 0.277025 -0.203092
C -6.849514 0.596221 -0.112260
C -7.130851 1.710824 0.913965
C -6.506251 3.065438 0.601950
C -7.400398 0.934128 -1.501241
H -1.325738 -3.512912 -0.564196
H -2.716360 1.066515 0.429294
H -0.384181 1.863633 0.274131
H 1.885224 2.687513 0.201830

H 2.128267 -3.073815 -0.941240
H 5.017098 -0.663257 -2.165858
H 6.235914 0.438075 0.074779
H 5.699914 -1.211904 0.150487
H 4.288690 -0.989319 3.524109
H 3.082731 -0.592701 2.278752
H 4.218702 -1.961781 2.048377
H 5.931693 2.401754 -1.503839
H 4.381405 3.049108 -2.157546
H 5.429093 4.056300 -1.144209
H -4.758940 -2.141692 2.005249
H -6.345181 -1.539916 1.583652
H -5.573994 -2.754348 0.548857
H -4.800234 0.985167 -0.802200
H -7.401222 -0.290251 0.220623
H -8.216434 1.843569 1.003763
H -6.779143 1.385921 1.901702
H -6.882250 3.473927 -0.340382
H -6.756057 3.779852 1.393424
H -5.415530 3.004113 0.544927
H -8.465202 1.184419 -1.445370
H -7.295513 0.077366 -2.176753
H -6.876637 1.779625 -1.959779
SCF Energy (B3LYP/6-31G**)= -1361.69634764
Number of imaginary frequencies = 0

1b_c300
MMFF Geometry
C 1.734748 -2.009338 -0.013402
C 2.454995 0.546149 -0.598064
N 3.055644 -1.737982 -0.146709
C 0.706670 -1.070765 -0.155050
C 1.092080 0.248066 -0.459625
C 3.399090 -0.464520 -0.432338
C 0.104466 1.235414 -0.623968
C -1.256264 0.932425 -0.476245
C -1.636737 -0.374719 -0.163702
C -0.663187 -1.376123 -0.016738
C -3.080252 -0.683756 -0.026511
C -4.083003 0.476310 0.204863
C -3.538483 1.783172 -0.356928
C -2.255037 1.974125 -0.689592
O -1.000451 -2.679909 0.261736
O -3.418266 -1.870365 -0.061527
O -4.235472 0.681504 1.611656
C -5.424416 0.117031 -0.468285
C -6.112338 -1.058838 0.196949
C -6.887246 -1.993306 -0.691678
O -6.082303 -1.206871 1.420703
O -4.440460 2.812995 -0.601552
C -4.834978 3.460664 0.610414
C 4.848683 -0.195861 -0.563693
C 5.582298 -1.118221 -1.493782
C 5.405274 0.775373 0.191959
C 6.831247 1.282114 0.291403
C 7.493347 0.841623 1.613480
C 7.713296 -0.658997 1.758072
C 7.747684 1.051181 -0.911766
H 1.513011 -3.048378 0.221110
H 2.780618 1.553115 -0.841077
H 0.404585 2.253929 -0.868419
H -1.935147 2.925342 -1.104940
H -1.980476 -2.762940 0.235385
H -4.758905 -0.076204 1.955955
H -5.263060 -0.119125 -1.527170
H -6.144242 0.941810 -0.411362
H -7.318809 -2.799081 -0.091573
H -7.693529 -1.444488 -1.184165
H -6.217661 -2.430888 -1.436241
H -5.555797 2.848882 1.160576
H -3.970963 3.700534 1.239932
H -5.329553 4.398513 0.340585
H 4.950640 -1.929348 -1.872372
H 5.942608 -0.571709 -2.370818
H 6.426887 -1.594175 -0.986506
H 4.746007 1.308065 0.881885
H 6.729914 2.376086 0.355347
H 8.461437 1.348053 1.715702
H 6.879718 1.183883 2.456993
H 8.375206 -1.046376 0.978582
H 8.181232 -0.873728 2.724556
H 6.769409 -1.210463 1.718983
H 8.677063 1.619796 -0.791667
H 7.274474 1.397221 -1.836918
H 8.029825 0.003506 -1.038171
SCF Energy (B3LYP/6-31G**)= -1361.69829372
Number of imaginary frequencies = 0

1b_c301
MMFF Geometry
C -1.444446 -2.201132 -0.647444
C -2.312686 0.330884 -0.169548
N -2.778951 -2.000493 -0.523432
C -0.473395 -1.199117 -0.549842
C -0.934825 0.108500 -0.304783
C -3.196573 -0.740714 -0.277262
C -0.008442 1.163685 -0.211385
C 1.367878 0.933470 -0.339849
C 1.819705 -0.370484 -0.564314
C 0.909774 -1.429701 -0.689117
C 3.275603 -0.590483 -0.697504
C 4.223697 0.463561 -0.078410
C 3.631356 1.866081 -0.201531
C 2.303377 2.062603 -0.304965
O 1.324241 -2.712371 -0.954843
O 3.666391 -1.609879 -1.268794
O 5.473585 0.425199 -0.779914
C 4.520008 0.159374 1.391843
C 4.744478 -1.314218 1.639216
C 6.084827 -1.897789 1.280125
O 3.835488 -2.012816 2.091447
O 4.583723 2.867818 -0.169955
C 4.155623 4.222659 -0.245747
C -4.662126 -0.563433 -0.149025
C -5.456472 -1.223733 -1.239086
C -5.163851 0.137031 0.890843
C -6.591617 0.416220 1.319389
C -7.017700 1.859169 0.983032
C -7.102183 2.181236 -0.503315
C -7.655184 -0.612575 0.927792
H -1.162190 -3.234939 -0.836075
H -2.696642 1.331221 0.005264
H -0.372567 2.175815 -0.038573
H 1.867911 3.053646 -0.371876
H 2.296373 -2.707070 -1.107559
H 5.319970 0.818746 -1.656447
H 3.684498 0.462728 2.036645
H 5.405639 0.700702 1.746872
H 6.186668 -2.884483 1.740830
H 6.170660 -1.999945 0.196615
H 6.882939 -1.257151 1.663401
H 3.644371 4.418305 -1.193737
H 3.516772 4.478670 0.605542
H 5.044402 4.859141 -0.204090
H -4.864943 -1.344937 -2.154393
H -5.797574 -2.214824 -0.924084
H -6.317985 -0.618480 -1.530828
H -4.449707 0.554873 1.603970
H -6.558744 0.364132 2.418558
H -7.995047 2.059511 1.439913
H -6.313400 2.560973 1.448491
H -7.838607 1.552334 -1.011075
H -7.408895 3.223574 -0.639914
H -6.135151 2.055000 -0.998711
H -8.571612 -0.440350 1.504218
H -7.318747 -1.631087 1.149435
H -7.934117 -0.562823 -0.126559
SCF Energy (B3LYP/6-31G**)= -1361.69137343
Number of imaginary frequencies = 0

1b_c302
MMFF Geometry

C -1.602891 -1.598309 -0.603387
C -2.097891 0.971353 0.125238
N -2.890380 -1.229678 -0.396762
C -0.500132 -0.751008 -0.470640
C -0.768100 0.578077 -0.094666
C -3.132475 0.050064 -0.031737
C 0.299122 1.485467 0.043845
C 1.625116 1.085319 -0.170280
C 1.882245 -0.241375 -0.526739
C 0.831663 -1.153157 -0.695732
C 3.284649 -0.637821 -0.756308
C 4.404775 0.222340 -0.139933
C 4.002476 1.690617 -0.066053
C 2.709604 2.068338 -0.071988
O 1.054498 -2.451247 -1.086188
O 3.534321 -1.642982 -1.427870
O 5.546235 0.100197 -1.008348
C 4.814357 -0.258877 1.254101
C 4.840084 -1.764321 1.362712
C 6.098929 -2.478935 0.950625
O 3.841009 -2.379200 1.738391
O 5.079466 2.550383 0.055319
C 4.840570 3.951970 0.046298
C -4.558976 0.415753 0.204857
C -4.814504 1.480857 1.234260
C -5.526217 -0.240803 -0.473831
C -7.031555 -0.066859 -0.463351
C -7.661556 -0.761602 0.757288
C -9.183319 -0.798510 0.713369
C -7.487748 1.383559 -0.649393
H -1.475841 -2.639985 -0.890579
H -2.317955 1.997072 0.402556
H 0.086096 2.517489 0.320363
H 2.413515 3.108569 0.006684
H 2.010992 -2.567132 -1.280419
H 5.364154 -0.644389 -1.615980
H 4.110629 0.094984 2.019392
H 5.804025 0.120686 1.536567
H 6.034421 -3.530179 1.245328
H 6.223817 -2.420613 -0.132344
H 6.959513 -2.032437 1.454847
H 4.349147 4.260331 -0.882136
H 4.253765 4.253020 0.919928
H 5.808524 4.458894 0.100318
H -4.131404 1.378685 2.085469
H -4.684333 2.477279 0.799666
H -5.818453 1.422553 1.659067
H -5.209918 -1.025180 -1.166375
H -7.388335 -0.601003 -1.356502
H -7.295316 -1.794823 0.815520
H -7.350839 -0.269255 1.685738
H -9.538981 -1.265457 -0.210432
H -9.568770 -1.380479 1.556791
H -9.610440 0.206319 0.782365
H -8.529891 1.416799 -0.985812
H -6.887965 1.894029 -1.411040
H -7.431087 1.961939 0.276593
SCF Energy (B3LYP/6-31G**)= -1361.69851065
Number of imaginary frequencies = 0

1b_c303
MMFF Geometry
C 1.822063 2.029586 0.031438
C 2.163852 -0.606227 -0.529106
N 3.085708 1.542631 -0.006201
C 0.670089 1.273398 -0.196556
C 0.858519 -0.090357 -0.486084
C 3.253149 0.229337 -0.285353
C -0.262400 -0.905307 -0.732386
C -1.562861 -0.386139 -0.678947
C -1.742439 0.966107 -0.375898
C -0.636838 1.798761 -0.152806
C -3.124526 1.491443 -0.300827
C -4.269780 0.480221 -0.063696
C -3.967565 -0.842867 -0.758878
C -2.706385 -1.240538 -1.008845
O -0.785094 3.138078 0.116833
O -3.286955 2.710874 -0.374797
O -5.476209 1.041719 -0.592997
C -4.440265 0.353061 1.454598
C -5.347406 -0.785972 1.861775
C -6.833856 -0.593278 1.712450
O -4.878436 -1.842722 2.290919
O -5.103354 -1.564253 -1.077931
C -4.953815 -2.919352 -1.487141
C 4.660556 -0.266059 -0.334015
C 4.927879 -1.430540 -1.246432
C 5.603247 0.363577 0.402765
C 7.082673 0.080641 0.570431
C 7.382442 -1.358131 1.033047
C 8.819045 -1.547647 1.504650
C 7.880182 0.508001 -0.663896
H 1.758222 3.091223 0.260512
H 2.318676 -1.659568 -0.738672
H -0.112828 -1.958203 -0.969804
H -2.482557 -2.189394 -1.484123
H -1.727108 3.386825 -0.026966
H -5.425667 0.962524 -1.561668
H -4.851046 1.275793 1.883731
H -3.474302 0.188834 1.950090
H -7.358823 -1.370114 2.275762
H -7.124343 0.378669 2.119025
H -7.120125 -0.663132 0.661571
H -4.446429 -2.979499 -2.455043
H -4.423168 -3.502734 -0.727660
H -5.953918 -3.346970 -1.604135
H 4.389984 -1.317259 -2.194789
H 5.980711 -1.526184 -1.516192
H 4.615476 -2.368860 -0.776416
H 5.281914 1.217863 1.004339
H 7.408998 0.740494 1.387992
H 6.712956 -1.620124 1.862292
H 7.190211 -2.080097 0.233489
H 9.069479 -0.838808 2.300114
H 8.953011 -2.559825 1.899940
H 9.530758 -1.415985 0.684489
H 8.951458 0.549492 -0.441720
H 7.581901 1.508059 -0.998668
H 7.743627 -0.181313 -1.502020
SCF Energy (B3LYP/6-31G**)= -1361.69691807
Number of imaginary frequencies = 0

1b_c304
MMFF Geometry
C 1.828784 -1.645286 0.184951
C 2.365550 0.988387 -0.212510
N 3.126673 -1.257308 0.155730
C 0.735521 -0.788924 0.025228
C 1.025756 0.572005 -0.179297
C 3.386259 0.054735 -0.043603
C -0.031324 1.483889 -0.349647
C -1.367903 1.062664 -0.308627
C -1.653862 -0.287979 -0.097170
C -0.609071 -1.213524 0.056186
C -3.071342 -0.721713 -0.072469
C -4.181718 0.336009 0.157868
C -3.717344 1.715652 -0.290467
C -2.437024 2.031009 -0.526455
O -0.849807 -2.555069 0.238867
O -3.304040 -1.927511 -0.196627
O -4.437324 0.439285 1.560855
C -5.443601 -0.088442 -0.622578
C -6.068357 -1.355821 -0.073137
C -6.703973 -2.293062 -1.063563
O -6.101633 -1.577106 1.139425
O -4.687180 2.682135 -0.534004
C -5.209211 3.217109 0.684723
C 4.821104 0.449961 -0.084417
C 5.186909 1.505839 -1.087357
C 5.690762 -0.184581 0.731927
C 7.173559 0.002294 0.981444
C 8.016302 -1.094338 0.300705

C 7.988217 -1.084797 -1.221992
C 7.747697 1.404243 0.761257
H 1.684173 -2.711191 0.347867
H 2.606065 2.037289 -0.354659
H 0.194535 2.536917 -0.514856
H -2.174857 3.029461 -0.864081
H -1.815631 -2.716413 0.143103
H -4.914325 -0.378685 1.825506
H -5.197807 -0.243815 -1.680164
H -6.233298 0.669773 -0.566549
H -7.101400 -3.167306 -0.540690
H -7.522167 -1.782198 -1.576801
H -5.954586 -2.627123 -1.785378
H -5.908142 2.515185 1.148759
H -4.409150 3.485388 1.383494
H -5.763937 4.127202 0.437776
H 5.159625 2.500349 -0.631263
H 6.176744 1.333836 -1.515310
H 4.503627 1.502326 -1.944592
H 5.279407 -0.972858 1.369663
H 7.286645 -0.166108 2.063572
H 9.058518 -1.001333 0.631224
H 7.673344 -2.078546 0.645566
H 8.379991 -0.147615 -1.626762
H 8.612019 -1.897208 -1.609385
H 6.975559 -1.235018 -1.607244
H 8.734984 1.481094 1.231564
H 7.109555 2.168219 1.218263
H 7.883517 1.653680 -0.292859
SCF Energy (B3LYP/6-31G**)= -1361.69858087
Number of imaginary frequencies = 0

1b_c305
MMFF Geometry
C 1.929938 1.987796 0.124949
C 2.301437 -0.614587 -0.558665
N 3.200231 1.525448 0.037019
C 0.785076 1.222517 -0.109870
C 0.989431 -0.123684 -0.463246
C 3.381954 0.228698 -0.303703
C -0.122981 -0.946773 -0.717998
C -1.429928 -0.452554 -0.614332
C -1.627129 0.882569 -0.249485
C -0.529547 1.722669 -0.013018
C -3.014876 1.385378 -0.127555
C -4.174309 0.375012 -0.011059
C -3.823346 -0.925002 -0.702978
C -2.562422 -1.318182 -0.936163
O -0.689906 3.046182 0.320882
O -3.213782 2.603503 -0.074433
O -5.291857 0.989913 -0.671116
C -4.488497 0.233478 1.481680
C -5.457391 -0.878560 1.817902
C -6.897344 -0.714338 1.405589
O -5.081423 -1.868596 2.448853
O -4.879236 -1.774788 -0.993039
C -5.242066 -1.703839 -2.372298
C 4.794704 -0.240467 -0.405630
C 5.062468 -1.347562 -1.386506
C 5.741412 0.360791 0.349174
C 7.224988 0.086682 0.477329
C 7.623731 -1.370504 0.781967
C 6.958003 -1.930515 2.032723
C 7.998734 0.638622 -0.722725
H 1.854481 3.037073 0.402133
H 2.468903 -1.655155 -0.817198
H 0.036905 -1.986724 -1.001865
H -2.364432 -2.293050 -1.372646
H -1.639120 3.285438 0.227540
H -5.215472 1.952185 -0.509820
H -4.911684 1.163216 1.883252
H -3.568939 0.042502 2.051184
H -7.502143 -1.491337 1.882112
H -7.265605 0.259620 1.737674
H -7.000665 -0.806492 0.323595
H -5.498082 -0.681817 -2.668939
H -4.438241 -2.091127 -3.007204
H -6.124746 -2.332954 -2.519593
H 4.488392 -1.202821 -2.309029
H 6.107470 -1.394007 -1.697381
H 4.794195 -2.317907 -0.956006
H 5.419932 1.176796 1.001503
H 7.557420 0.674295 1.346134
H 7.410950 -2.031014 -0.063817
H 8.710721 -1.412184 0.929528
H 5.874166 -2.014188 1.909996
H 7.345646 -2.931761 2.246859
H 7.160767 -1.297327 2.902306
H 9.077886 0.583159 -0.541921
H 7.748072 1.690125 -0.902533
H 7.788989 0.081735 -1.640670
SCF Energy (B3LYP/6-31G**)= -1361.69687484
Number of imaginary frequencies = 0

1b_c306
MMFF Geometry
C -1.939292 -1.607880 -0.413115
C -2.237867 1.067198 -0.054894
N -3.195203 -1.115941 -0.285575
C -0.774194 -0.838315 -0.371621
C -0.940586 0.544664 -0.184689
C -3.341713 0.217251 -0.108870
C 0.195142 1.373740 -0.134715
C 1.489728 0.847211 -0.258991
C 1.648880 -0.528339 -0.438677
C 0.525581 -1.368038 -0.505961
C 3.016082 -1.076529 -0.568006
C 4.223570 -0.195619 -0.197034
C 3.903296 1.290694 -0.200483
C 2.640034 1.752053 -0.227061
O 0.644291 -2.724039 -0.698623
O 3.153795 -2.245027 -0.943713
O 5.178797 -0.452416 -1.238038
C 4.748485 -0.644397 1.176302
C 6.235196 -0.913370 1.199279
C 7.172450 0.262841 1.247325
O 6.663545 -2.068334 1.153324
O 5.030946 2.090287 -0.143006
C 4.886358 3.493682 -0.324407
C -4.740273 0.722285 0.010051
C -4.987523 2.148902 -0.400540
C -5.712225 -0.111969 0.441426
C -7.179142 0.194717 0.655035
C -8.010036 -0.508200 -0.433318
C -9.492206 -0.163747 -0.381497
C -7.582968 -0.239502 2.067731
H -1.893250 -2.685526 -0.555522
H -2.374591 2.132259 0.100657
H 0.061287 2.446461 0.002498
H 2.418261 2.813606 -0.214438
H 1.587047 -2.937676 -0.885560
H 5.243529 -1.425651 -1.326094
H 4.260014 -1.574069 1.499120
H 4.510976 0.076682 1.968657
H 8.148279 -0.066625 1.615583
H 7.292077 0.685073 0.247824
H 6.788352 1.019218 1.936269
H 4.413860 3.719337 -1.285872
H 4.324493 3.937078 0.503659
H 5.886570 3.936837 -0.327094
H -4.434674 2.395069 -1.314459
H -6.037290 2.349532 -0.630070
H -4.678429 2.837157 0.392942
H -5.438823 -1.141422 0.683582
H -7.359672 1.272617 0.592125
H -7.628874 -0.220928 -1.421990
H -7.894989 -1.597045 -0.357690
H -9.645891 0.919115 -0.423236
H -10.011023 -0.612191 -1.234966
H -9.962460 -0.547241 0.528543
H -8.604272 0.076957 2.301873
H -6.924451 0.211099 2.819254

H -7.531284 -1.327914 2.184264
SCF Energy (B3LYP/6-31G**)= -1361.70389621
Number of imaginary frequencies = 0

1b_c307
MMFF Geometry
C 1.982303 1.728642 -0.306985
C 2.234174 -0.964508 -0.058959
N 3.227866 1.213081 -0.173334
C 0.805858 0.975610 -0.326886
C 0.948474 -0.418129 -0.200058
C 3.349892 -0.128663 -0.048245
C -0.197564 -1.234104 -0.224321
C -1.478393 -0.682004 -0.357443
C -1.615035 0.705023 -0.467232
C -0.482109 1.531343 -0.468576
C -2.976760 1.274451 -0.589677
C -4.192571 0.408267 -0.202049
C -3.889476 -1.061062 -0.405124
C -2.644580 -1.559532 -0.430821
O -0.581454 2.895452 -0.603535
O -3.110931 2.446995 -0.954817
O -5.252678 0.811532 -1.083064
C -4.563583 0.785946 1.235569
C -5.600003 -0.112027 1.874207
C -7.015094 -0.039014 1.361746
O -5.297318 -0.848848 2.815009
O -4.979539 -1.916008 -0.449952
C -5.290298 -2.294903 -1.791193
C 4.735397 -0.657715 0.116625
C 4.875962 -1.924900 0.913040
C 5.768308 0.032882 -0.415785
C 7.253616 -0.261051 -0.425616
C 7.951543 -0.001568 0.922345
C 7.789072 1.428727 1.422279
C 7.596348 -1.649379 -0.975227
H 1.954842 2.812116 -0.400958
H 2.355247 -2.039438 0.027632
H -0.084870 -2.314643 -0.138423
H -2.485055 -2.631229 -0.508951
H -1.512581 3.127264 -0.818544
H -5.129208 1.768357 -1.247551
H -4.950685 1.811883 1.284108
H -3.674943 0.761300 1.880632
H -7.674809 -0.590665 2.037791
H -7.343017 1.003244 1.336225
H -7.087313 -0.480420 0.366847
H -5.479628 -1.421055 -2.422555
H -4.487516 -2.903820 -2.220137
H -6.200502 -2.901121 -1.766023
H 4.630514 -2.796733 0.297779
H 5.882369 -2.066888 1.309987
H 4.213984 -1.914992 1.786684
H 5.532595 0.958997 -0.946393
H 7.687350 0.448309 -1.146183
H 7.584940 -0.689150 1.691958
H 9.024311 -0.204882 0.812294
H 6.744022 1.658730 1.650997
H 8.369249 1.573319 2.339372
H 8.147915 2.147509 0.678791
H 8.668654 -1.715256 -1.191905
H 7.059072 -1.848268 -1.909059
H 7.362465 -2.451008 -0.269907
SCF Energy (B3LYP/6-31G**)= -1361.69688252
Number of imaginary frequencies = 0

1b_c308
MMFF Geometry
C -1.561660 -2.174784 -0.478325
C -2.179401 0.369310 0.246955
N -2.862236 -1.882884 -0.232889
C -0.505667 -1.261644 -0.389390
C -0.838008 0.051338 -0.012609
C -3.158455 -0.614057 0.120563
C 0.179848 1.015107 0.096378
C 1.521035 0.694075 -0.163996
C 1.851928 -0.615124 -0.532453
C 0.842103 -1.586644 -0.650264
C 3.270606 -0.958390 -0.813777
C 4.364320 0.118038 -0.618028
C 3.831071 1.488407 -0.264783
C 2.534766 1.740020 -0.038746
O 1.117695 -2.880929 -1.027450
O 3.524575 -2.087585 -1.244613
O 5.050996 0.243173 -1.882226
C 5.413325 -0.393241 0.377457
C 4.904268 -0.480880 1.798674
C 5.941198 -0.420577 2.888982
O 3.714029 -0.647718 2.062961
O 4.785977 2.472070 -0.050457
C 4.842976 3.399576 -1.134219
C -4.590941 -0.337644 0.390468
C -5.271841 -1.404488 1.202393
C -5.169981 0.779018 -0.102025
C -6.604355 1.260088 0.000602
C -7.619309 0.261742 -0.587809
C -9.013814 0.852656 -0.754071
C -6.947595 1.725582 1.417715
H -1.381127 -3.209988 -0.760270
H -2.456228 1.371521 0.559192
H -0.081847 2.030375 0.392926
H 2.217818 2.729032 0.279025
H 2.086668 -2.961895 -1.181522
H 4.374238 0.369288 -2.568845
H 6.290476 0.266566 0.361436
H 5.776387 -1.388154 0.091177
H 5.454214 -0.491773 3.865496
H 6.637951 -1.254884 2.777522
H 6.477148 0.530142 2.833067
H 5.056668 2.893602 -2.081017
H 3.911928 3.970687 -1.211535
H 5.656602 4.102284 -0.932076
H -4.647721 -1.701122 2.053508
H -6.222830 -1.077257 1.624553
H -5.465551 -2.291505 0.589801
H -4.555286 1.449046 -0.705217
H -6.651324 2.158413 -0.632725
H -7.269667 -0.072705 -1.572959
H -7.700892 -0.633660 0.036285
H -8.986285 1.762816 -1.361348
H -9.668706 0.132185 -1.254820
H -9.464956 1.093014 0.213050
H -7.868850 2.317382 1.420830
H -6.154704 2.362473 1.826108
H -7.093152 0.887315 2.104689
SCF Energy (B3LYP/6-31G**)= -1361.69412937
Number of imaginary frequencies = 0

1b_c309
MMFF Geometry
C -1.735342 -1.709555 -0.540719
C -2.102444 0.895505 0.129663
N -3.003957 -1.272622 -0.349923
C -0.590939 -0.916313 -0.421999
C -0.793752 0.430405 -0.077407
C -3.181997 0.025093 -0.012455
C 0.318063 1.281632 0.050936
C 1.625084 0.814429 -0.157388
C 1.826135 -0.528672 -0.492752
C 0.722282 -1.389215 -0.628185
C 3.207966 -1.025035 -0.721627
C 4.399705 -0.045354 -0.586822
C 4.007622 1.370329 -0.215025
C 2.740567 1.744928 -0.001871
O 0.869463 -2.713249 -0.972543
O 3.355839 -2.199595 -1.074230
O 5.018369 0.000477 -1.890566
C 5.441036 -0.649012 0.365129
C 4.969698 -0.726836 1.801054
C 6.040548 -0.863321 2.851108
O 3.777691 -0.729722 2.106355
O 5.016312 2.281374 0.075855

C 5.762938 2.679091 -1.072893
C -4.588809 0.467490 0.210939
C -4.792211 1.566446 1.216185
C -5.586481 -0.153901 -0.456741
C -7.081022 0.096334 -0.455159
C -7.748284 -0.538122 0.778438
C -9.269854 -0.498776 0.730137
C -7.462529 1.563504 -0.674945
H -1.659836 -2.762541 -0.803506
H -2.269539 1.936397 0.386657
H 0.158363 2.324874 0.322098
H 2.525844 2.757304 0.329168
H 1.830196 -2.905984 -1.068177
H 4.322605 0.222309 -2.533593
H 6.364068 -0.056720 0.333139
H 5.719378 -1.662405 0.050048
H 5.581494 -0.907307 3.842638
H 6.605443 -1.782864 2.679953
H 6.706013 0.002557 2.810760
H 6.529109 1.935365 -1.309038
H 5.114218 2.858523 -1.937237
H 6.272830 3.616457 -0.831188
H -4.117966 1.447663 2.072285
H -4.609503 2.545191 0.760823
H -5.799143 1.569218 1.637893
H -5.308563 -0.968194 -1.130905
H -7.462460 -0.438860 -1.337426
H -7.435118 -1.587030 0.860832
H -7.415111 -0.041566 1.696800
H -9.646650 -0.967599 -0.184289
H -9.686295 -1.041464 1.584939
H -9.645555 0.527713 0.775356
H -8.500892 1.642045 -1.015499
H -6.835906 2.025699 -1.445754
H -7.378589 2.158860 0.238119
SCF Energy (B3LYP/6-31G**)= -1361.69447688
Number of imaginary frequencies = 0

1b_c310
MMFF Geometry
C 1.673589 -1.826582 0.033831
C 2.296338 0.814191 0.159392
N 2.980169 -1.482518 0.136502
C 0.612007 -0.919979 -0.013785
C 0.946025 0.445023 0.049261
C 3.286731 -0.166339 0.201482
C -0.077593 1.409365 -0.008320
C -1.424151 1.033655 -0.108915
C -1.748724 -0.323521 -0.152489
C -0.741131 -1.298145 -0.125312
C -3.175281 -0.695065 -0.259212
C -4.228798 0.326482 0.234039
C -3.768465 1.750447 -0.092660
C -2.464220 2.057826 -0.231395
O -1.030513 -2.639238 -0.208772
O -3.449365 -1.815083 -0.696020
O -4.319322 0.269403 1.661094
C -5.591528 0.012889 -0.417403
C -6.140937 -1.332497 0.015008
C -6.968984 -2.088638 -0.988037
O -5.963643 -1.758662 1.158539
O -4.804924 2.664978 -0.179419
C -4.487602 4.047755 -0.285371
C 4.734080 0.173549 0.334565
C 5.055985 1.445975 1.067506
C 5.656371 -0.676906 -0.169334
C 7.169054 -0.591652 -0.202921
C 7.695592 0.701562 -0.854322
C 9.183955 0.645750 -1.176566
C 7.778456 -0.879441 1.171126
H 1.495140 -2.898845 -0.010276
H 2.564729 1.864786 0.197345
H 0.183958 2.466230 0.026869
H -2.125882 3.067552 -0.433131
H -1.989183 -2.741029 -0.404473
H -4.784871 -0.566009 1.885193
H -5.499680 0.023656 -1.510117
H -6.356904 0.742882 -0.127869
H -7.290489 -3.040872 -0.557290
H -7.851350 -1.500356 -1.251079
H -6.370214 -2.291644 -1.879357
H -3.892940 4.381185 0.571254
H -3.972785 4.258442 -1.228090
H -5.426671 4.609017 -0.282817
H 4.918756 2.313429 0.413573
H 4.413351 1.565238 1.947480
H 6.079004 1.470811 1.446033
H 5.288835 -1.585232 -0.653911
H 7.487436 -1.416880 -0.857069
H 7.150149 0.886031 -1.788643
H 7.520475 1.569427 -0.211185
H 9.417845 -0.214919 -1.810979
H 9.485396 1.551794 -1.712219
H 9.788778 0.582387 -0.267333
H 8.852493 -1.075368 1.088946
H 7.323246 -1.766687 1.625818
H 7.647731 -0.042817 1.863415
SCF Energy (B3LYP/6-31G**)= -1361.70656657
Number of imaginary frequencies = 0

1b_c311
MMFF Geometry
C -1.917761 -1.702715 0.002683
C -2.244407 0.978574 0.280948
N -3.171186 -1.228319 0.201326
C -0.768579 -0.911736 -0.068839
C -0.949602 0.474518 0.075350
C -3.331631 0.107776 0.339996
C 0.168848 1.325354 0.005375
C 1.461509 0.817426 -0.193800
C 1.635968 -0.561433 -0.328237
C 0.528646 -1.423229 -0.277801
C 3.001181 -1.089872 -0.536676
C 4.213847 -0.172406 -0.293002
C 3.862872 1.306391 -0.334293
C 2.591628 1.743378 -0.285536
O 0.661178 -2.783673 -0.424690
O 3.135508 -2.270560 -0.873477
O 5.095454 -0.456457 -1.390340
C 4.847308 -0.552390 1.055067
C 6.337084 -0.793236 0.979104
C 7.250188 0.400658 0.907724
O 6.785332 -1.941190 0.950663
O 4.974509 2.127893 -0.394628
C 4.787241 3.519053 -0.624743
C -4.724517 0.589624 0.571021
C -4.881573 1.890681 1.310864
C -5.764366 -0.159691 0.142017
C -7.243756 0.143464 0.248130
C -7.885035 0.046337 -1.150172
C -9.349227 0.463659 -1.176007
C -7.882698 -0.821473 1.249005
H -1.859998 -2.784171 -0.101419
H -2.394626 2.048648 0.379064
H 0.022858 2.400370 0.107854
H 2.348979 2.800325 -0.300643
H 1.592061 -2.988007 -0.671829
H 5.173390 -1.431278 -1.443051
H 4.403549 -1.476057 1.451331
H 4.653240 0.197561 1.832309
H 8.257190 0.105221 1.216059
H 7.287146 0.780962 -0.114819
H 6.901763 1.179325 1.590776
H 4.240456 3.693369 -1.557070
H 4.278701 3.988555 0.223160
H 5.774921 3.979529 -0.721015
H -4.705434 2.738434 0.640665
H -5.873673 2.012132 1.753136
H -4.177144 1.952602 2.148396
H -5.546002 -1.105525 -0.359022
H -7.403471 1.163294 0.611812
H -7.334395 0.692555 -1.846255

H -7.800978 -0.976571 -1.538960
H -9.478630 1.470466 -0.766719
H -9.719436 0.467306 -2.206357
H -9.974403 -0.227668 -0.603681
H -8.930331 -0.564320 1.433811
H -7.365765 -0.783283 2.214801
H -7.846522 -1.855898 0.889088
SCF Energy (B3LYP/6-31G**)= -1361.70397755
Number of imaginary frequencies = 0

1b_c312
MMFF Geometry
C -1.787259 -1.514504 -0.652217
C -2.050940 1.093288 0.055030
N -3.037229 -1.024641 -0.470798
C -0.611224 -0.775557 -0.501280
C -0.760373 0.572382 -0.134129
C -3.166460 0.275635 -0.119724
C 0.385948 1.368380 0.037514
C 1.673481 0.844820 -0.152495
C 1.819922 -0.496149 -0.518386
C 0.683122 -1.303728 -0.692276
C 3.183434 -1.051250 -0.706142
C 4.416675 -0.143446 -0.534439
C 4.080343 1.279497 -0.153254
C 2.829888 1.711486 0.049773
O 0.778814 -2.628955 -1.047176
O 3.301224 -2.237175 -1.033267
O 4.978873 -0.122230 -1.866490
C 5.439995 -0.816463 0.392157
C 5.135493 -0.697663 1.870970
C 3.946797 -1.445010 2.415146
O 5.844821 -0.022109 2.617018
O 5.121797 2.149270 0.139297
C 5.907615 2.487258 -1.002223
C -4.558824 0.782004 0.054930
C -4.779798 2.255975 -0.154649
C -5.548364 -0.087822 0.356727
C -7.012734 0.210747 0.597755
C -7.841879 -0.318900 -0.585876
C -9.319060 0.039854 -0.499894
C -7.439355 -0.409948 1.931959
H -1.755982 -2.564541 -0.934977
H -2.172625 2.130264 0.350647
H 0.268762 2.413493 0.324209
H 2.661703 2.734574 0.376227
H 1.726400 -2.857728 -1.176008
H 5.114597 -1.052320 -2.131637
H 6.435027 -0.382979 0.223535
H 5.560368 -1.881560 0.157624
H 4.074985 -1.594161 3.491236
H 3.034639 -0.871779 2.242697
H 3.874236 -2.428687 1.944772
H 6.628967 1.695201 -1.221899
H 5.282478 2.699408 -1.876479
H 6.472098 3.393187 -0.761873
H -4.214901 2.617474 -1.021651
H -5.824075 2.504479 -0.361142
H -4.467154 2.821796 0.729069
H -5.293595 -1.145289 0.455962
H -7.175946 1.289710 0.684849
H -7.445069 0.097389 -1.521038
H -7.744496 -1.409401 -0.662445
H -9.455552 1.120342 -0.390961
H -9.834941 -0.275975 -1.412473
H -9.805535 -0.459195 0.343119
H -8.458253 -0.112125 2.198756
H -6.782582 -0.080117 2.745183
H -7.405854 -1.504677 1.895363
SCF Energy (B3LYP/6-31G**)= -1361.69846467
Number of imaginary frequencies = 0

1b_c313
MMFF Geometry
C -1.813309 -2.406203 -0.132177
C -2.508513 0.173552 -0.626518
N -3.132310 -2.108921 -0.220634
C -0.774574 -1.480680 -0.274722
C -1.146528 -0.149101 -0.532594
C -3.467924 -0.823098 -0.457867
C -0.146822 0.826922 -0.696268
C 1.213994 0.501184 -0.594836
C 1.577996 -0.820714 -0.329796
C 0.592984 -1.810524 -0.181373
C 3.014181 -1.156231 -0.223046
C 4.049083 -0.023201 -0.092953
C 3.527483 1.321403 -0.574214
C 2.220044 1.545050 -0.797730
O 0.916164 -3.124683 0.060708
O 3.346274 -2.345800 -0.209921
O 5.121608 -0.425919 -0.958751
C 4.503695 0.053391 1.373472
C 6.004984 0.045688 1.544531
C 6.761725 1.315513 1.263249
O 6.591737 -0.984824 1.881148
O 4.523488 2.270434 -0.721053
C 4.202653 3.511020 -1.338622
C -4.921189 -0.539876 -0.553733
C -5.703804 -1.582835 -1.306616
C -5.442175 0.554246 0.042601
C -6.888733 0.999179 0.060851
C -7.569557 0.681577 1.406793
C -6.989069 1.391349 2.623960
C -6.982041 2.484938 -0.300438
H -1.603381 -3.455544 0.064656
H -2.818553 1.191134 -0.843650
H -0.440769 1.855039 -0.906184
H 1.852842 2.511009 -1.126920
H 1.892642 -3.230240 -0.008265
H 5.327918 -1.357027 -0.736072
H 4.121568 -0.801257 1.948506
H 4.097117 0.932086 1.889983
H 7.736113 1.273536 1.758437
H 6.912330 1.429205 0.188081
H 6.215202 2.173047 1.663535
H 3.792310 3.356467 -2.341779
H 3.512190 4.088451 -0.715870
H 5.127849 4.086498 -1.436886
H -5.716333 -2.530353 -0.756862
H -6.744252 -1.302609 -1.482057
H -5.256247 -1.759213 -2.291443
H -4.775135 1.202849 0.610938
H -7.455216 0.467624 -0.710439
H -8.633892 0.938682 1.333430
H -7.527471 -0.401020 1.583185
H -7.073935 2.478063 2.534110
H -7.536801 1.091762 3.523680
H -5.936879 1.135095 2.777920
H -8.022228 2.826538 -0.270427
H -6.604036 2.659847 -1.314295
H -6.398461 3.113819 0.380335
SCF Energy (B3LYP/6-31G**)= -1361.70241906
Number of imaginary frequencies = 0

1b_c314
MMFF Geometry
C -1.558116 -1.662979 -0.804596
C -2.131601 0.914064 -0.166152
N -2.860403 -1.310321 -0.678475
C -0.477257 -0.795527 -0.630397
C -0.785597 0.536608 -0.299311
C -3.142753 -0.026218 -0.359971
C 0.258977 1.462221 -0.115674
C 1.600754 1.078170 -0.243798
C 1.896880 -0.250903 -0.558624
C 0.870905 -1.181679 -0.769753
C 3.315694 -0.630367 -0.698300
C 4.385682 0.257805 -0.034471
C 3.959206 1.720948 -0.012162
C 2.663778 2.079008 -0.100991
O 1.134391 -2.483827 -1.119190
O 3.618238 -1.643901 -1.334614

O 5.577457 0.137175 -0.832664
C 4.719798 -0.191694 1.389953
C 4.761693 -1.694357 1.528217
C 6.053379 -2.395809 1.204641
O 3.751495 -2.318521 1.855748
O 5.015079 2.598880 0.155812
C 4.757413 3.996336 0.107819
C -4.588308 0.328092 -0.245855
C -4.956052 1.747796 -0.575765
C -5.472327 -0.631641 0.108057
C -6.971235 -0.568162 0.322164
C -7.399015 0.500332 1.346507
C -8.847217 0.351316 1.796700
C -7.725365 -0.481846 -1.006760
H -1.399978 -2.708581 -1.060344
H -2.379420 1.935984 0.101153
H 0.015728 2.495872 0.127863
H 2.349103 3.115916 -0.059430
H 2.102050 -2.588842 -1.255727
H 5.442056 -0.621761 -1.434715
H 3.967098 0.164504 2.105971
H 5.685374 0.208714 1.722572
H 5.987461 -3.442440 1.515036
H 6.241206 -2.355130 0.129986
H 6.875786 -1.926343 1.750036
H 4.316986 4.281387 -0.853087
H 4.116205 4.303321 0.940175
H 5.713299 4.518670 0.209182
H -4.405994 2.101182 -1.455597
H -6.012093 1.866544 -0.823000
H -4.731963 2.410355 0.266697
H -5.076795 -1.633808 0.293358
H -7.238597 -1.540301 0.762326
H -6.756393 0.431318 2.233507
H -7.271601 1.509703 0.943490
H -9.033792 -0.649159 2.199409
H -9.071658 1.079081 2.583269
H -9.543599 0.529997 0.972288
H -8.789193 -0.699761 -0.867168
H -7.339002 -1.212040 -1.726971
H -7.648641 0.510353 -1.460567
SCF Energy (B3LYP/6-31G**)= -1361.69882708
Number of imaginary frequencies = 0

1b_c315
MMFF Geometry
C -1.768836 -1.666473 -0.230398
C -2.062655 0.968319 0.354394
N -3.016968 -1.202313 0.018643
C -0.609122 -0.887690 -0.208923
C -0.773954 0.474374 0.093764
C -3.161072 0.110989 0.310469
C 0.355089 1.311722 0.128773
C 1.640909 0.814814 -0.131423
C 1.803151 -0.540702 -0.430990
C 0.683583 -1.389082 -0.470251
C 3.165941 -1.066861 -0.692654
C 4.378450 -0.116345 -0.666665
C 4.025180 1.316905 -0.343910
C 2.780096 1.724619 -0.070045
O 0.795407 -2.729354 -0.757480
O 3.297470 -2.265915 -0.961545
O 4.840939 -0.159596 -2.035979
C 5.487835 -0.703542 0.218189
C 5.284104 -0.510445 1.706442
C 4.170762 -1.271203 2.376827
O 6.013032 0.236535 2.359683
O 5.056501 2.232066 -0.183779
C 5.742465 2.522689 -1.400230
C -4.548487 0.580847 0.593568
C -4.690797 1.792520 1.474919
C -5.596792 -0.102366 0.082542
C -7.072458 0.205765 0.221386
C -7.712378 0.274001 -1.179254
C -9.171164 0.710127 -1.158757
C -7.725269 -0.857051 1.107595
H -1.724933 -2.729486 -0.457517
H -2.198462 2.021811 0.576283
H 0.225690 2.368233 0.363786
H 2.603475 2.760370 0.208506
H 1.737420 -2.938162 -0.946838
H 4.985060 -1.099938 -2.256853
H 6.452757 -0.249797 -0.045361
H 5.628544 -1.775954 0.033562
H 4.383464 -1.355729 3.446524
H 3.225234 -0.743364 2.243591
H 4.105203 -2.281427 1.965380
H 6.471813 1.740242 -1.627820
H 5.046693 2.662587 -2.234771
H 6.292304 3.457807 -1.257572
H -4.501334 2.707835 0.904670
H -5.682619 1.876893 1.926317
H -3.988396 1.750270 2.315372
H -5.389349 -0.988385 -0.521679
H -7.220084 1.180379 0.696926
H -7.152404 0.987080 -1.798352
H -7.640472 -0.699910 -1.680144
H -9.288655 1.666320 -0.639321
H -9.539431 0.833843 -2.182361
H -9.806009 -0.032965 -0.667717
H -8.769936 -0.608986 1.319608
H -7.209670 -0.933823 2.071806
H -7.701391 -1.845025 0.634076
SCF Energy (B3LYP/6-31G**)= -1361.69838658
Number of imaginary frequencies = 0

1b_c316
MMFF Geometry
C -1.483328 -1.990755 -0.604602
C -2.270696 0.446368 0.305500
N -2.803105 -1.798192 -0.364623
C -0.488265 -1.025680 -0.419648
C -0.907759 0.232186 0.053402
C -3.184111 -0.581584 0.079887
C 0.044768 1.247111 0.258752
C 1.405433 1.025144 0.009204
C 1.817117 -0.230911 -0.447804
C 0.879546 -1.247368 -0.678387
C 3.254929 -0.440620 -0.719084
C 4.285509 0.520503 -0.091233
C 3.686922 1.900924 0.112442
C 2.365192 2.113603 0.194242
O 1.249554 -2.479235 -1.161711
O 3.609948 -1.373634 -1.446623
O 5.368650 0.607329 -1.033669
C 4.840727 0.007203 1.239811
C 5.042128 -1.488891 1.251992
C 6.364003 -2.023552 0.770548
O 4.132932 -2.238824 1.609285
O 4.548091 2.942083 0.430755
C 5.155261 3.512013 -0.728782
C -4.635802 -0.414622 0.336819
C -5.264645 -1.582209 1.045572
C -5.273561 0.700810 -0.080147
C -6.736714 1.084816 0.025592
C -7.675927 0.078501 -0.665934
C -9.100953 0.595726 -0.818226
C -7.136088 1.411734 1.466550
H -1.234375 -2.987004 -0.964243
H -2.615792 1.402330 0.687245
H -0.284674 2.222272 0.616118
H 1.987018 3.104858 0.428271
H 2.211899 -2.468466 -1.360754
H 5.310968 -0.187583 -1.602775
H 4.156214 0.231186 2.069010
H 5.797892 0.483759 1.484876
H 6.413489 -3.099529 0.960040
H 6.469733 -1.847295 -0.301636
H 7.178166 -1.539114 1.315201
H 6.069782 2.970972 -0.985757
H 4.471352 3.540083 -1.584527
H 5.436186 4.541206 -0.485597

H -5.389067 -2.428176 0.361145
H -4.640794 -1.906317 1.886794
H -6.243088 -1.348645 1.467154
H -4.690539 1.452604 -0.614561
H -6.826781 2.027395 -0.534533
H -7.286985 -0.152454 -1.665936
H -7.713712 -0.868271 -0.118116
H -9.118215 1.552534 -1.349403
H -9.699858 -0.119563 -1.391262
H -9.584962 0.728434 0.153750
H -8.091775 1.945466 1.494204
H -6.392068 2.059177 1.944418
H -7.243790 0.513022 2.080160
SCF Energy (B3LYP/6-31G**)= -1361.69276453
Number of imaginary frequencies = 0

1b_c317
MMFF Geometry
C -1.701946 -2.104413 -0.383434
C -2.204231 0.556417 -0.612939
N -2.995949 -1.704330 -0.425490
C -0.597306 -1.248661 -0.451567
C -0.869996 0.125078 -0.569488
C -3.233203 -0.379365 -0.542639
C 0.199612 1.035042 -0.640612
C 1.534984 0.602062 -0.596895
C 1.804007 -0.764217 -0.475368
C 0.743078 -1.685610 -0.406641
C 3.212880 -1.226390 -0.426438
C 4.353755 -0.185762 -0.480960
C 3.902615 1.258048 -0.633426
C 2.603733 1.599564 -0.685720
O 0.961256 -3.040085 -0.299286
O 3.424149 -2.442139 -0.366076
O 5.134889 -0.511887 -1.651987
C 5.287111 -0.406003 0.717833
C 4.652993 -0.090536 2.054592
C 5.579682 -0.069189 3.242103
O 3.446712 0.110130 2.189845
O 4.959496 2.148187 -0.716345
C 4.677336 3.538133 -0.828992
C -4.658045 0.031207 -0.587714
C -5.040831 0.990698 -1.675428
C -5.486525 -0.466500 0.355598
C -6.965403 -0.273361 0.625007
C -7.186039 0.541967 1.917348
C -6.712954 1.989312 1.854352
C -7.837969 0.242414 -0.520036
H -1.569071 -3.180078 -0.288681
H -2.438559 1.613860 -0.685363
H -0.018252 2.098940 -0.728596
H 2.288463 2.631838 -0.789212
H 1.933214 -3.197826 -0.278711
H 4.651729 -0.176511 -2.425733
H 6.188427 0.210842 0.610414
H 5.631431 -1.447522 0.750680
H 5.014544 0.171338 4.146863
H 6.041596 -1.051992 3.363528
H 6.347732 0.693918 3.094069
H 4.133210 3.752680 -1.754290
H 4.124770 3.897127 0.045204
H 5.631105 4.072599 -0.867527
H -4.204203 1.231120 -2.340401
H -5.814846 0.562731 -2.318809
H -5.396546 1.934782 -1.250932
H -5.037072 -1.147375 1.085564
H -7.345730 -1.284144 0.835270
H -8.254050 0.533966 2.169198
H -6.672825 0.045790 2.751409
H -7.247067 2.554476 1.085425
H -6.898649 2.482247 2.814385
H -5.640027 2.054844 1.651390
H -8.895183 0.215472 -0.231946
H -7.731340 -0.389946 -1.407746
H -7.607206 1.273888 -0.797444
SCF Energy (B3LYP/6-31G**)= -1361.69655705
Number of imaginary frequencies = 0

1b_c318
MMFF Geometry
C -1.617556 -1.518959 -0.710178
C -2.131462 0.972168 0.243190
N -2.909110 -1.173483 -0.489290
C -0.519463 -0.685178 -0.485062
C -0.797510 0.603510 0.006863
C -3.160620 0.066969 -0.011994
C 0.263985 1.496753 0.243320
C 1.593353 1.120638 0.010909
C 1.862790 -0.168415 -0.461868
C 0.816976 -1.062889 -0.726795
C 3.271425 -0.542224 -0.711312
C 4.389636 0.272546 -0.030917
C 3.958616 1.713543 0.165272
C 2.671212 2.088144 0.221898
O 1.048232 -2.320429 -1.229466
O 3.530669 -1.492376 -1.456108
O 5.519341 0.244770 -0.920716
C 4.818435 -0.318606 1.314016
C 4.852422 -1.827681 1.301011
C 6.125597 -2.498350 0.860312
O 3.851006 -2.477444 1.604041
O 4.961881 2.622363 0.466403
C 5.322745 3.391031 -0.681769
C -4.591536 0.406370 0.237076
C -4.864626 1.373682 1.354780
C -5.547291 -0.188499 -0.511212
C -7.053231 -0.019659 -0.505579
C -7.697059 -0.822805 0.638961
C -9.217961 -0.859091 0.571157
C -7.511973 1.440565 -0.566730
H -1.483127 -2.529836 -1.089290
H -2.358466 1.967729 0.610400
H 0.045196 2.498467 0.611813
H 2.408646 3.117716 0.447054
H 2.008637 -2.420229 -1.412532
H 5.383207 -0.514368 -1.523561
H 4.122773 -0.033057 2.114449
H 5.809379 0.043673 1.614110
H 6.052440 -3.574576 1.040557
H 6.287695 -2.327437 -0.205680
H 6.967318 -2.108327 1.437879
H 5.655299 2.753206 -1.506570
H 4.488290 4.022025 -1.005883
H 6.153464 4.044725 -0.400226
H -4.193103 1.196263 2.202838
H -4.731555 2.405529 1.013810
H -5.874178 1.275437 1.758406
H -5.218980 -0.906378 -1.267333
H -7.396189 -0.472147 -1.447894
H -7.328014 -1.856237 0.609053
H -7.400523 -0.415262 1.612068
H -9.559600 -1.241872 -0.395645
H -9.612618 -1.515435 1.353511
H -9.649481 0.134483 0.724375
H -8.549640 1.501531 -0.912960
H -6.903862 2.018806 -1.271191
H -7.469708 1.933497 0.408161
SCF Energy (B3LYP/6-31G**)= -1361.69344554
Number of imaginary frequencies = 0

1b_c319
MMFF Geometry
C 1.767911 -2.538070 -0.023096
C 2.493815 0.062671 -0.330377
N 3.090942 -2.250661 -0.083333
C 0.740155 -1.593572 -0.108619
C 1.127864 -0.250812 -0.271019
C 3.440724 -0.954261 -0.225395
C 0.139926 0.745072 -0.378827
C -1.224001 0.426506 -0.310787
C -1.603084 -0.905927 -0.133372
C -0.631047 -1.914893 -0.048562

C -3.047298 -1.223848 -0.063451
C -4.028349 -0.084192 0.293104
C -3.528103 1.262156 -0.222330
C -2.225798 1.480756 -0.484029
O -0.973129 -3.239400 0.090487
O -3.386905 -2.396588 -0.244010
O -4.012174 -0.037109 1.727059
C -5.416700 -0.488865 -0.244547
C -6.549816 0.355031 0.299787
C -7.711167 0.614036 -0.621395
O -6.548470 0.764328 1.462689
O -4.524918 2.210680 -0.361146
C -4.155010 3.558741 -0.628506
C 4.897107 -0.684354 -0.287989
C 5.676431 -1.673967 -1.111838
C 5.431360 0.360792 0.379557
C 6.893322 0.748984 0.461782
C 7.109416 2.073200 -0.292741
C 8.570865 2.492057 -0.375829
C 7.306596 0.842836 1.933848
H 1.544557 -3.595658 0.100859
H 2.815854 1.089739 -0.471219
H 0.444097 1.782007 -0.516437
H -1.859542 2.440014 -0.831684
H -1.952473 -3.315043 0.026361
H -4.842569 0.405646 2.006616
H -5.669305 -1.518066 0.039872
H -5.421911 -0.440337 -1.340246
H -8.450644 1.240464 -0.114990
H -7.360868 1.137867 -1.514241
H -8.177239 -0.334782 -0.897750
H -3.494142 3.947264 0.153074
H -3.691424 3.646594 -1.616114
H -5.067206 4.162668 -0.630481
H 5.137497 -1.923339 -2.033486
H 6.648749 -1.288082 -1.428592
H 5.843304 -2.597695 -0.547868
H 4.772676 1.001341 0.966770
H 7.522955 -0.019609 0.002521
H 6.725747 1.973252 -1.316518
H 6.534524 2.879959 0.179693
H 9.183106 1.698086 -0.814827
H 8.670066 3.382660 -1.004850
H 8.975103 2.738390 0.610219
H 8.384698 1.004062 2.032399
H 7.068151 -0.084479 2.467405
H 6.793694 1.665974 2.444001
SCF Energy (B3LYP/6-31G**)= -1361.71081110
Number of imaginary frequencies = 0

1b_c320
MMFF Geometry
C 1.761382 2.460501 0.141087
C 2.364666 -0.161552 -0.252899
N 3.064774 2.094502 0.192448
C 0.695504 1.587449 -0.099939
C 1.020125 0.232725 -0.305553
C 3.350797 0.788583 0.003714
C -0.006445 -0.693283 -0.567832
C -1.348925 -0.294332 -0.608889
C -1.665825 1.048530 -0.386559
C -0.653834 1.990185 -0.151467
C -3.091653 1.446479 -0.410509
C -4.152691 0.345946 -0.179215
C -3.694631 -0.974759 -0.788372
C -2.390852 -1.265357 -0.952370
O -0.937105 3.321616 0.037291
O -3.358772 2.640639 -0.557148
O -5.373702 0.767605 -0.797477
C -4.394626 0.275532 1.333051
C -5.215722 -0.922667 1.752807
C -6.702932 -0.877203 1.518061
O -4.677233 -1.909154 2.260442
O -4.741472 -1.812226 -1.127446
C -4.448022 -3.164814 -1.459900
C 4.787143 0.430108 0.066605
C 5.679205 1.331000 -0.738301
C 5.182541 -0.624576 0.811601
C 6.560999 -1.175451 1.122565
C 6.857806 -2.458977 0.321784
C 6.962870 -2.273381 -1.186392
C 7.731655 -0.189559 1.099701
H 1.587688 3.521853 0.306003
H 2.643896 -1.197122 -0.422001
H 0.250839 -1.737709 -0.741958
H -2.056364 -2.210674 -1.365709
H -1.888459 3.474163 -0.166735
H -5.263149 0.646813 -1.756827
H -4.910549 1.175162 1.691965
H -3.446635 0.225924 1.884794
H -7.185544 -1.672495 2.093372
H -7.102763 0.081286 1.858357
H -6.922844 -1.022772 0.458998
H -3.884353 -3.221555 -2.396359
H -3.909975 -3.661485 -0.645926
H -5.397513 -3.688081 -1.606220
H 5.134623 1.810459 -1.560452
H 6.102867 2.119816 -0.108922
H 6.488144 0.772777 -1.215428
H 4.407199 -1.181885 1.342138
H 6.494823 -1.490131 2.175451
H 7.794760 -2.899239 0.685613
H 6.073408 -3.199881 0.524267
H 7.773678 -1.589949 -1.453168
H 7.172499 -3.235951 -1.664634
H 6.030386 -1.890789 -1.611365
H 8.606486 -0.637122 1.585630
H 7.487165 0.725367 1.649988
H 8.041328 0.087109 0.090001
SCF Energy (B3LYP/6-31G**)= -1361.69397920
Number of imaginary frequencies = 0

1b_c321
MMFF Geometry
C 1.641425 2.388433 0.177994
C 2.323139 -0.087004 -0.709333
N 2.957816 2.111555 0.016367
C 0.598385 1.492209 -0.077483
C 0.964033 0.215271 -0.538386
C 3.287030 0.875795 -0.416432
C -0.039023 -0.729092 -0.818192
C -1.396993 -0.423394 -0.644874
C -1.759728 0.845640 -0.184544
C -0.767280 1.800011 0.097535
C -3.196553 1.162061 0.010614
C -4.271281 0.110843 -0.327547
C -3.709160 -1.199737 -0.826998
C -2.397860 -1.444167 -0.937439
O -1.077384 3.059716 0.554080
O -3.506406 2.288377 0.414026
O -4.983753 0.731195 -1.422286
C -5.253080 -0.033249 0.844630
C -4.758014 -0.893560 1.988530
C -3.619567 -0.375989 2.827405
O -5.277112 -1.981148 2.241108
O -4.585603 -2.252810 -1.049686
C -5.452307 -2.035325 -2.161594
C 4.737589 0.613349 -0.584930
C 5.504942 1.758539 -1.190374
C 5.270087 -0.556608 -0.170461
C 6.715966 -0.996851 -0.250523
C 7.425868 -0.885089 1.113119
C 6.870484 -1.771642 2.221186
C 6.799161 -2.410819 -0.834024
H 1.437559 3.396160 0.533863
H 2.626476 -1.061145 -1.080884
H 0.247626 -1.718100 -1.175825
H -2.059726 -2.428341 -1.251545
H -2.055291 3.149980 0.603466
H -5.270322 1.611488 -1.111261
H -6.196239 -0.468485 0.487614
H -5.538771 0.942801 1.256498

H -3.634522 -0.871364 3.802612
H -2.668271 -0.587344 2.336766
H -3.733336 0.698122 2.993170
H -6.292004 -1.395377 -1.876390
H -4.914767 -1.615129 -3.018691
H -5.860653 -3.005358 -2.460603
H 5.530951 2.613196 -0.505494
H 6.540852 1.509628 -1.428639
H 5.036069 2.079423 -2.127654
H 4.614643 -1.284690 0.308207
H 7.266760 -0.353918 -0.944491
H 8.488005 -1.126300 0.979033
H 7.389190 0.158350 1.451927
H 6.951734 -2.832151 1.966349
H 7.437820 -1.610521 3.143916
H 5.822231 -1.543400 2.434593
H 7.839199 -2.751342 -0.878130
H 6.399362 -2.431167 -1.854384
H 6.229202 -3.136276 -0.243797
SCF Energy (B3LYP/6-31G**)= -1361.69695752
Number of imaginary frequencies = 0

1b_c322
MMFF Geometry
C -1.806064 -2.425374 0.042198
C -2.459898 0.174120 -0.405739
N -3.112933 -2.123313 -0.151317
C -0.760747 -1.496496 0.029710
C -1.111069 -0.153998 -0.204132
C -3.427488 -0.827865 -0.365914
C -0.104428 0.828675 -0.225942
C 1.243116 0.493616 -0.030139
C 1.586734 -0.842144 0.189389
C 0.592826 -1.832174 0.235073
C 3.014003 -1.177812 0.394167
C 4.078213 -0.175394 -0.106818
C 3.572271 1.261824 -0.023625
C 2.257415 1.549715 -0.001077
O 0.895677 -3.150752 0.480657
O 3.279733 -2.267266 0.909686
O 4.246709 -0.500257 -1.494190
C 5.368996 -0.436809 0.696883
C 6.595957 0.229837 0.111598
C 7.639951 0.710729 1.082790
O 6.759754 0.324154 -1.106654
O 4.581525 2.207103 -0.001548
C 4.236320 3.580785 -0.140888
C -4.866666 -0.542423 -0.578914
C -5.574235 -1.545880 -1.449542
C -5.448270 0.526600 0.006175
C -6.904583 0.937631 -0.065358
C -7.477683 1.032428 1.362063
C -8.973855 1.313199 1.397152
C -7.012964 2.261717 -0.824827
H -1.611289 -3.481919 0.214016
H -2.751392 1.200407 -0.606229
H -0.380871 1.868155 -0.398436
H 1.889869 2.568809 0.038096
H 1.854848 -3.214231 0.692597
H 5.123516 -0.150663 -1.763873
H 5.613265 -1.506247 0.721141
H 5.236618 -0.106681 1.734395
H 8.463056 1.179775 0.536732
H 7.200406 1.450151 1.756835
H 8.027645 -0.137293 1.652354
H 3.695812 3.757191 -1.076462
H 3.655809 3.925542 0.720592
H 5.164921 4.158267 -0.173266
H -5.804921 -2.453332 -0.881604
H -6.507280 -1.160793 -1.868339
H -4.951585 -1.823640 -2.308175
H -4.840671 1.172917 0.640200
H -7.491350 0.190359 -0.608345
H -7.296394 0.086009 1.888126
H -6.956911 1.812532 1.931960
H -9.527154 0.571200 0.813007
H -9.338528 1.271919 2.428654
H -9.205494 2.307747 1.005286
H -8.058680 2.537839 -0.992369
H -6.536518 2.188751 -1.809274
H -6.530499 3.079503 -0.277700
SCF Energy (B3LYP/6-31G**)= -1361.71084572
Number of imaginary frequencies = 0

1b_c323
MMFF Geometry
C 1.707276 2.418083 0.093274
C 2.353273 -0.194060 -0.291765
N 3.019258 2.080398 0.101010
C 0.652189 1.520292 -0.098261
C 0.999191 0.170970 -0.299947
C 3.328534 0.778612 -0.081446
C -0.015594 -0.779762 -0.512770
C -1.366815 -0.409733 -0.511499
C -1.707222 0.928911 -0.294472
C -0.707197 1.893744 -0.104836
C -3.140788 1.300775 -0.278996
C -4.203168 0.196703 -0.102631
C -3.697478 -1.124049 -0.642808
C -2.395152 -1.411112 -0.785736
O -1.008819 3.221660 0.081355
O -3.456939 2.492263 -0.360443
O -5.341432 0.628841 -0.864414
C -4.573333 0.173340 1.383788
C -5.446002 -0.991358 1.796513
C -6.873806 -1.014014 1.315577
O -5.007298 -1.871110 2.540293
O -4.652797 -2.098896 -0.884054
C -4.952827 -2.201803 -2.276332
C 4.775272 0.453346 -0.068434
C 5.626710 1.424911 -0.837564
C 5.223490 -0.624734 0.610822
C 6.640662 -1.124627 0.815447
C 7.158121 -1.859172 -0.434524
C 8.491306 -2.561431 -0.214147
C 7.599664 -0.052294 1.341177
H 1.517517 3.477562 0.251986
H 2.645945 -1.225840 -0.461212
H 0.256472 -1.821497 -0.681361
H -2.084549 -2.399774 -1.111600
H -1.971113 3.354167 -0.071695
H -5.364392 1.605200 -0.801175
H -5.102441 1.091222 1.670655
H -3.667988 0.133040 2.004370
H -7.422981 -1.797439 1.845891
H -7.349490 -0.054179 1.532215
H -6.916190 -1.221711 0.245558
H -5.289058 -1.244628 -2.687249
H -4.086057 -2.569257 -2.835723
H -5.764138 -2.926398 -2.391834
H 5.816153 2.325535 -0.244196
H 6.588087 0.999914 -1.131824
H 5.134131 1.723219 -1.770593
H 4.492595 -1.235210 1.143522
H 6.568639 -1.872316 1.619225
H 6.421247 -2.611931 -0.743450
H 7.264400 -1.165200 -1.275826
H 8.436791 -3.252223 0.633068
H 8.762820 -3.138345 -1.104132
H 9.295735 -1.843946 -0.027137
H 8.482986 -0.516435 1.793413
H 7.124983 0.558692 2.116971
H 7.955526 0.613693 0.550584
SCF Energy (B3LYP/6-31G**)= -1361.69540494
Number of imaginary frequencies = 0

1b_c324
MMFF Geometry
C -2.034087 -1.501230 -0.343368
C -2.264397 1.187660 -0.042918
N -3.276559 -0.975589 -0.218941
C -0.850182 -0.760039 -0.326307

C -0.981252 0.630413 -0.169193
C -3.389227 0.364412 -0.070928
C 0.174855 1.432025 -0.144888
C 1.455222 0.871005 -0.265539
C 1.579259 -0.511700 -0.415502
C 0.435212 -1.324621 -0.456981
C 2.931645 -1.096282 -0.541180
C 4.162947 -0.237521 -0.197558
C 3.879311 1.255821 -0.232135
C 2.627627 1.747594 -0.261040
O 0.519197 -2.687075 -0.620122
O 3.037999 -2.275861 -0.891593
O 5.104419 -0.540857 -1.238572
C 4.686056 -0.668636 1.182111
C 6.165824 -0.973735 1.201717
C 7.132031 0.179702 1.217616
O 6.565312 -2.139678 1.178834
O 5.026655 2.028345 -0.199608
C 4.915393 3.430524 -0.411371
C -4.774258 0.906043 0.045206
C -4.988559 2.332596 -0.384143
C -5.766978 0.102687 0.487988
C -7.222569 0.455826 0.698667
C -8.108885 -0.052836 -0.454649
C -8.149237 -1.565494 -0.635274
C -7.695250 -0.057375 2.062714
H -2.015602 -2.582564 -0.462057
H -2.373511 2.259005 0.089414
H 0.068381 2.510506 -0.030796
H 2.432124 2.814329 -0.270700
H 1.455111 -2.928067 -0.808169
H 5.144588 -1.517107 -1.305309
H 4.177089 -1.578563 1.528651
H 4.471784 0.075464 1.959669
H 7.255161 0.576614 0.208212
H 6.771377 0.960418 1.891925
H 8.101960 -0.165572 1.586976
H 4.442019 3.646444 -1.374639
H 4.370284 3.905925 0.410114
H 5.926161 3.848626 -0.430244
H -4.441893 2.549571 -1.309067
H -6.036125 2.557724 -0.601803
H -4.650635 3.024260 0.394484
H -5.518168 -0.929223 0.744044
H -7.337524 1.545202 0.734617
H -9.134644 0.303511 -0.296502
H -7.768928 0.399831 -1.395049
H -8.556060 -2.065088 0.248548
H -8.793514 -1.820438 -1.483290
H -7.155736 -1.974618 -0.840753
H -8.753207 0.178394 2.219974
H -7.125847 0.415289 2.871262
H -7.570391 -1.140774 2.163289
SCF Energy (B3LYP/6-31G**)= -1361.70304369
Number of imaginary frequencies = 0

1b_c325
MMFF Geometry
C -1.694565 -1.842460 -0.554416
C -2.134991 0.817780 -0.241925
N -2.977657 -1.411154 -0.488169
C -0.569723 -1.017214 -0.476922
C -0.810174 0.357287 -0.315637
C -3.193709 -0.084973 -0.330685
C 0.281119 1.240039 -0.230930
C 1.604116 0.777434 -0.306730
C 1.842018 -0.592281 -0.461758
C 0.759342 -1.485201 -0.549910
C 3.241201 -1.084829 -0.550652
C 4.410080 -0.072018 -0.472184
C 3.978428 1.370305 -0.300321
C 2.696330 1.742397 -0.205591
O 0.943646 -2.838442 -0.717431
O 3.424703 -2.290492 -0.746831
O 5.101907 -0.173707 -1.735373
C 5.403051 -0.533162 0.603157
C 4.851154 -0.443693 2.009372
C 5.861691 -0.426444 3.125819
O 3.643722 -0.434967 2.246259
O 4.956472 2.330429 -0.068653
C 5.762985 2.599146 -1.214327
C -4.619426 0.354842 -0.275424
C -4.908614 1.746538 -0.764696
C -5.556203 -0.511594 0.171494
C -7.050941 -0.346009 0.358160
C -7.427186 0.848248 1.256002
C -8.884807 0.826974 1.699877
C -7.790434 -0.364614 -0.981631
H -1.590249 -2.918337 -0.677410
H -2.328012 1.876147 -0.101320
H 0.092262 2.305200 -0.099945
H 2.449467 2.783100 -0.014288
H 1.910767 -3.021840 -0.735905
H 4.441138 -0.047603 -2.438400
H 6.318472 0.069261 0.549231
H 5.712427 -1.571719 0.431697
H 5.347036 -0.357901 4.088227
H 6.448988 -1.347563 3.101722
H 6.515542 0.442135 3.015055
H 6.551257 1.847693 -1.313593
H 5.163021 2.658017 -2.129040
H 6.245662 3.569195 -1.062354
H -4.338525 1.968901 -1.674143
H -5.956083 1.894119 -1.031876
H -4.650389 2.486143 0.000331
H -5.216215 -1.505928 0.472711
H -7.373492 -1.249206 0.897136
H -6.796168 0.840768 2.153925
H -7.241188 1.799922 0.748842
H -9.129337 -0.112769 2.204874
H -9.074840 1.646277 2.400830
H -9.563910 0.953149 0.851681
H -8.865213 -0.510743 -0.832573
H -7.438428 -1.187349 -1.614369
H -7.658440 0.567459 -1.538465
SCF Energy (B3LYP/6-31G**)= -1361.69484243
Number of imaginary frequencies = 0

1b_c326
MMFF Geometry
C -1.566485 -1.960000 -0.350985
C -2.276094 0.593395 0.256455
N -2.870504 -1.710220 -0.079412
C -0.550787 -0.999057 -0.343072
C -0.929729 0.320198 -0.024947
C -3.212771 -0.437445 0.213503
C 0.044010 1.336005 -0.005012
C 1.386852 1.051798 -0.278465
C 1.758579 -0.263659 -0.578338
C 0.798938 -1.282012 -0.632880
C 3.187561 -0.533201 -0.857982
C 4.230460 0.445666 -0.257879
C 3.686522 1.867960 -0.343841
C 2.371469 2.132423 -0.345841
O 1.131280 -2.571158 -0.970587
O 3.486824 -1.531380 -1.514442
O 5.447463 0.366311 -1.004890
C 4.553339 0.061465 1.190838
C 4.665017 -1.434909 1.379284
C 5.934855 -2.111991 0.937032
O 3.723748 -2.071960 1.854281
O 4.563270 2.915624 -0.588666
C 5.496267 3.141786 0.464434
C -4.648106 -0.209099 0.509122
C -5.259430 -1.254310 1.400231
C -5.291427 0.849256 -0.029823
C -6.745196 1.262057 0.078092
C -7.781507 0.191295 -0.314570
C -7.577856 -0.360049 -1.720185
C -7.055397 1.863869 1.451129
H -1.348459 -3.000241 -0.583942
H -2.590367 1.598688 0.519534

H -0.255008 2.359028 0.220289
H 2.014059 3.153119 -0.450246
H 2.078129 -2.596790 -1.235005
H 5.242591 0.675948 -1.906381
H 3.770836 0.409344 1.877906
H 5.494721 0.503777 1.535390
H 5.964677 -3.128033 1.340684
H 5.973579 -2.160975 -0.152855
H 6.799327 -1.564876 1.321282
H 5.006040 3.129324 1.443856
H 6.307941 2.410204 0.428589
H 5.934167 4.133427 0.316685
H -5.476981 -2.165466 0.832946
H -4.580013 -1.512025 2.221226
H -6.184897 -0.919912 1.871631
H -4.724357 1.513101 -0.684239
H -6.872035 2.081847 -0.644678
H -7.789996 -0.638986 0.397960
H -8.782757 0.638793 -0.267689
H -6.637598 -0.912955 -1.804300
H -8.390856 -1.047927 -1.974075
H -7.573984 0.445668 -2.461141
H -8.056587 2.308778 1.457334
H -6.341100 2.655492 1.703955
H -7.022732 1.115040 2.247810
SCF Energy (B3LYP/6-31G**)= -1361.68764157
Number of imaginary frequencies = 0

1b_c327
MMFF Geometry
C -2.083724 1.586872 -0.323890
C -2.315494 -1.117620 -0.283225
N -3.322163 1.049487 -0.439861
C -0.904520 0.850607 -0.185376
C -1.037116 -0.547704 -0.164871
C -3.435475 -0.298546 -0.418960
C 0.113114 -1.344198 -0.024297
C 1.388961 -0.771375 0.091905
C 1.520119 0.619901 0.068293
C 0.377489 1.427939 -0.066215
C 2.870572 1.226102 0.183239
C 4.117038 0.319391 0.267952
C 3.788218 -1.155233 0.350660
C 2.548082 -1.645570 0.237034
O 0.456066 2.801043 -0.090302
O 2.956777 2.459109 0.200520
O 4.734262 0.711146 1.499128
C 4.983454 0.643641 -0.962327
C 6.442062 0.298563 -0.777777
C 7.031887 -0.746226 -1.686847
O 7.135271 0.900465 0.044073
O 4.845322 -2.054284 0.414623
C 5.422434 -2.114911 1.721388
C -4.814299 -0.852131 -0.563547
C -4.930889 -2.236467 -1.142397
C -5.871096 -0.093970 -0.194788
C -7.342605 -0.441814 -0.238263
C -7.956910 -0.517210 1.175116
C -7.985604 0.788998 1.959489
C -8.083834 0.545805 -1.142689
H -2.064419 2.674402 -0.346871
H -2.426023 -2.196580 -0.254247
H 0.007856 -2.428770 -0.004372
H 2.388577 -2.720282 0.255014
H 1.393727 3.065887 0.051154
H 5.250302 1.522821 1.326554
H 4.969408 1.717730 -1.190591
H 4.594125 0.147036 -1.859805
H 8.018081 -1.039605 -1.316700
H 6.394368 -1.632417 -1.710172
H 7.131488 -0.334196 -2.693938
H 6.153752 -1.313413 1.855405
H 4.661039 -2.081605 2.508557
H 5.954938 -3.067091 1.804614
H -4.691652 -2.990995 -0.385730
H -5.927601 -2.458686 -1.529917
H -4.249922 -2.363043 -1.992022
H -5.668191 0.905843 0.194227
H -7.485228 -1.433707 -0.677814
H -8.986655 -0.887405 1.090330
H -7.410427 -1.265002 1.764276
H -8.578380 1.552629 1.447946
H -8.442113 0.621234 2.940728
H -6.979445 1.182832 2.128390
H -9.157039 0.327830 -1.158135
H -7.716020 0.476037 -2.172838
H -7.952170 1.583216 -0.816993
SCF Energy (B3LYP/6-31G**)= -1361.70232490
Number of imaginary frequencies = 0

1b_c328
MMFF Geometry
C -1.559086 -1.929515 -0.429662
C -2.223879 0.563556 0.429270
N -2.857911 -1.688160 -0.127584
C -0.528032 -0.988836 -0.333398
C -0.884027 0.298574 0.112954
C -3.173863 -0.446502 0.295542
C 0.106629 1.291725 0.224565
C 1.445306 1.018231 -0.086649
C 1.793848 -0.266725 -0.513613
C 0.816195 -1.261369 -0.656477
C 3.211621 -0.533090 -0.837167
C 4.280503 0.392805 -0.210012
C 3.760812 1.824772 -0.098271
C 2.443084 2.091564 -0.025630
O 1.125063 -2.519201 -1.114813
O 3.478661 -1.490251 -1.565712
O 5.443244 0.385922 -1.048917
C 4.715170 -0.104112 1.170625
C 4.878906 -1.605549 1.214955
C 6.136685 -2.199332 0.639232
O 3.984758 -2.313573 1.681897
O 4.767606 2.771429 -0.057294
C 4.413297 4.142830 0.078086
C -4.600070 -0.211557 0.613793
C -5.212143 -1.211781 1.551470
C -5.248826 0.803089 0.002501
C -6.680681 1.291770 0.107858
C -7.489998 0.937719 -1.157027
C -7.713326 -0.551845 -1.386064
C -7.447472 0.958719 1.389236
H -1.358149 -2.944878 -0.765348
H -2.527267 1.543572 0.784793
H -0.177475 2.289768 0.556306
H 2.061238 3.101655 0.075256
H 2.073180 -2.537771 -1.377706
H 5.219248 0.891376 -1.849621
H 3.972990 0.154000 1.937707
H 5.663839 0.348994 1.483910
H 6.229949 -3.239076 0.965453
H 6.099441 -2.170124 -0.451436
H 7.007255 -1.647397 1.002242
H 3.814906 4.477724 -0.775209
H 3.886074 4.318829 1.021181
H 5.336858 4.728984 0.093319
H -4.534299 -2.038375 1.791353
H -5.470480 -0.737287 2.503212
H -6.107157 -1.662564 1.112242
H -4.679224 1.395228 -0.717926
H -6.596352 2.389034 0.113903
H -8.467439 1.434006 -1.107757
H -6.982531 1.350704 -2.038569
H -8.275846 -1.005987 -0.565627
H -8.289915 -0.703394 -2.304620
H -6.766979 -1.088835 -1.498454
H -8.388866 1.519309 1.421024
H -6.871431 1.244029 2.276002
H -7.704763 -0.099847 1.469448
SCF Energy (B3LYP/6-31G**)= -1361.69118360
Number of imaginary frequencies = 0

1b_c329
MMFF Geometry
C -1.884172 -1.443774 -0.500858
C -2.073911 1.213336 0.022601
N -3.119212 -0.909726 -0.343666
C -0.688186 -0.727107 -0.412711
C -0.799080 0.646769 -0.140477
C -3.211756 0.414986 -0.084134
C 0.368918 1.422596 -0.035541
C 1.640619 0.853434 -0.199246
C 1.748969 -0.513352 -0.470667
C 0.590184 -1.301134 -0.577140
C 3.095833 -1.115802 -0.630551
C 4.353558 -0.230756 -0.534149
C 4.057640 1.223829 -0.251650
C 2.820808 1.701634 -0.068832
O 0.648308 -2.650117 -0.838295
O 3.179902 -2.324498 -0.874042
O 4.902133 -0.318192 -1.869176
C 5.369027 -0.863361 0.428839
C 5.083106 -0.632766 1.898123
C 3.881592 -1.308711 2.504024
O 5.817079 0.075002 2.588381
O 5.123946 2.084503 -0.030398
C 5.906118 2.320488 -1.199727
C -4.589003 0.968337 0.066078
C -4.774010 2.431804 -0.233496
C -5.600585 0.146239 0.423704
C -7.052237 0.505330 0.651050
C -7.933501 0.120821 -0.553091
C -7.997837 -1.367927 -0.872276
C -7.549931 -0.121924 1.957311
H -1.882711 -2.511565 -0.708990
H -2.165700 2.271455 0.244923
H 0.281450 2.488022 0.177545
H 2.682204 2.749236 0.185896
H 1.588343 -2.912127 -0.958869
H 5.011278 -1.267891 -2.069057
H 6.372964 -0.469003 0.221428
H 5.459769 -1.945114 0.269148
H 4.017220 -1.385006 3.586812
H 2.982521 -0.725474 2.299401
H 3.779163 -2.320844 2.104982
H 6.604705 1.496310 -1.369043
H 5.277466 2.486757 -2.081360
H 6.495999 3.226026 -1.028968
H -4.214899 2.721640 -1.130599
H -5.815264 2.695203 -0.438030
H -4.431388 3.043599 0.607442
H -5.372640 -0.908874 0.587761
H -7.148730 1.588731 0.785778
H -8.954748 0.479884 -0.373363
H -7.574658 0.651470 -1.444424
H -8.423736 -1.938879 -0.042297
H -8.636275 -1.532253 -1.746698
H -7.009176 -1.774616 -1.104230
H -8.605414 0.117897 2.124744
H -6.982015 0.264085 2.811623
H -7.445163 -1.212097 1.959238
SCF Energy (B3LYP/6-31G**)= -1361.69759626
Number of imaginary frequencies = 0

1b_c330
MMFF Geometry
C 1.873478 2.125280 0.198084
C 2.297423 -0.559825 0.265627
N 3.136472 1.688354 0.419746
C 0.763590 1.296240 0.002183
C 0.995307 -0.091965 0.039728
C 3.333339 0.353476 0.447606
C -0.079718 -0.978431 -0.155192
C -1.380802 -0.504925 -0.373417
C -1.606111 0.873214 -0.397255
C -0.543080 1.772230 -0.226617
C -2.986849 1.355077 -0.610427
C -4.160496 0.381791 -0.391260
C -3.753994 -1.058962 -0.661370
C -2.467447 -1.453180 -0.628373
O -0.732463 3.132782 -0.270835
O -3.174355 2.539379 -0.907457
O -5.173932 0.782928 -1.328344
C -4.677922 0.624976 1.030969
C -5.690119 -0.396059 1.501201
C -7.084100 -0.322806 0.934068
O -5.381798 -1.248266 2.337267
O -4.827879 -1.894262 -0.911131
C -4.594664 -3.287991 -1.068327
C 4.724092 -0.093773 0.684542
C 5.391098 0.518832 1.882033
C 5.301950 -0.932673 -0.202246
C 6.682810 -1.558560 -0.244721
C 7.557543 -0.916382 -1.341407
C 7.914650 0.546484 -1.109824
C 7.436057 -1.696562 1.079813
H 1.771394 3.208365 0.182070
H 2.504924 -1.624878 0.308643
H 0.106650 -2.051947 -0.136844
H -2.178349 -2.484068 -0.801221
H -1.652071 3.316051 -0.567671
H -5.080730 1.749025 -1.448471
H -5.145728 1.614069 1.116740
H -3.849581 0.613288 1.752006
H -7.742504 -0.986328 1.502156
H -7.464585 0.697781 1.023840
H -7.088701 -0.635397 -0.111237
H -3.974853 -3.483292 -1.949139
H -4.142306 -3.713381 -0.166720
H -5.561854 -3.775470 -1.222260
H 4.779894 1.293635 2.357819
H 5.578508 -0.240798 2.647236
H 6.334536 0.997036 1.602000
H 4.705870 -1.227955 -1.069261
H 6.504546 -2.594638 -0.570149
H 8.486870 -1.491302 -1.441086
H 7.043527 -0.997854 -2.308022
H 8.488886 0.680367 -0.188890
H 8.529724 0.913617 -1.938113
H 7.021693 1.176167 -1.057936
H 8.324474 -2.324065 0.943975
H 6.810982 -2.181172 1.837448
H 7.780684 -0.738863 1.476343
SCF Energy (B3LYP/6-31G**)= -1361.69977340
Number of imaginary frequencies = 0

1b_c331
MMFF Geometry
C -1.482704 -2.185158 -0.604293
C -2.353454 0.364417 -0.239344
N -2.816590 -1.981048 -0.478538
C -0.513082 -1.178543 -0.561641
C -0.976023 0.138956 -0.374365
C -3.236842 -0.712368 -0.287464
C -0.052728 1.200429 -0.339987
C 1.321033 0.964251 -0.465569
C 1.775258 -0.348947 -0.632127
C 0.869110 -1.414274 -0.702572
C 3.234665 -0.566590 -0.757335
C 4.172648 0.501458 -0.136492
C 3.579691 1.885970 -0.376695
C 2.260905 2.084704 -0.520271
O 1.286367 -2.705341 -0.915827
O 3.637714 -1.591706 -1.308300
O 5.458025 0.430626 -0.759578
C 4.370970 0.234570 1.360182
C 4.527272 -1.237912 1.667493
C 5.861204 -1.880813 1.395817
O 3.572537 -1.886005 2.098627
O 4.430695 2.956064 -0.615281
C 5.248022 3.299810 0.500268
C -4.703509 -0.531442 -0.159422
C -5.502972 -1.323429 -1.156325
C -5.204574 0.271107 0.804471

C -6.643559 0.585789 1.158656
C -7.359313 1.490777 0.138638
C -6.655818 2.824027 -0.084399
C -7.471776 -0.662088 1.479680
H -1.200118 -3.226158 -0.747047
H -2.736424 1.372344 -0.112374
H -0.415103 2.220670 -0.219382
H 1.872259 3.076934 -0.732202
H 2.254894 -2.702688 -1.086278
H 5.327725 0.666960 -1.696437
H 3.512114 0.590262 1.944343
H 5.255368 0.745285 1.757128
H 5.895062 -2.863889 1.873917
H 6.006464 -2.002936 0.320764
H 6.660859 -1.266753 1.817451
H 4.667138 3.330812 1.428361
H 6.089680 2.608257 0.593412
H 5.655630 4.299222 0.321469
H -5.537614 -2.380267 -0.871119
H -6.529885 -0.969059 -1.255610
H -5.059515 -1.249248 -2.156236
H -4.500138 0.760636 1.478908
H -6.596566 1.152516 2.100517
H -7.471467 0.984177 -0.825632
H -8.375380 1.696953 0.498383
H -5.671266 2.687948 -0.542111
H -7.249613 3.453431 -0.755089
H -6.528059 3.363161 0.859638
H -8.419882 -0.376347 1.949174
H -6.942115 -1.317796 2.179574
H -7.717152 -1.244398 0.587686
SCF Energy (B3LYP/6-31G**)= -1361.68771583
Number of imaginary frequencies = 0

1b_c332
MMFF Geometry
C 1.573323 -2.298309 -0.056197
C 2.480839 0.263723 0.017124
N 2.912288 -2.101329 0.010670
C 0.616183 -1.278884 -0.090519
C 1.097719 0.043636 -0.051490
C 3.350357 -0.824610 0.040683
C 0.184837 1.113830 -0.088312
C -1.196748 0.886426 -0.148713
C -1.668214 -0.427643 -0.171770
C -0.772671 -1.506751 -0.162158
C -3.129193 -0.641902 -0.236805
C -4.051340 0.491136 0.275619
C -3.447752 1.854707 -0.074470
C -2.122176 2.017237 -0.251045
O -1.209388 -2.808630 -0.223501
O -3.535499 -1.727899 -0.656249
O -4.109014 0.451470 1.704967
C -5.457211 0.324827 -0.337292
C -6.138264 -0.950215 0.120261
C -7.070761 -1.616393 -0.854509
O -5.977652 -1.387399 1.262095
O -4.380332 2.876566 -0.139941
C -3.917030 4.216069 -0.262857
C 4.819986 -0.651965 0.118512
C 5.481511 -1.503055 1.164143
C 5.438928 0.209587 -0.717150
C 6.906296 0.535946 -0.918146
C 7.281908 1.892059 -0.288331
C 7.186318 1.950961 1.230707
C 7.920469 -0.561842 -0.587602
H 1.275329 -3.344408 -0.081126
H 2.877662 1.273197 0.064387
H 0.561464 2.135795 -0.069232
H -1.681581 2.983080 -0.469596
H -2.178265 -2.806035 -0.393836
H -4.656765 -0.326866 1.947885
H -5.394199 0.319799 -1.432090
H -6.130353 1.135547 -0.033758
H -7.482523 -2.525546 -0.407800
H -7.890425 -0.936505 -1.098930
H -6.522079 -1.888207 -1.759636
H -3.266285 4.485261 0.575399
H -3.408341 4.366268 -1.220362
H -4.788828 4.876561 -0.238501
H 5.863095 -2.429989 0.724797
H 6.298691 -0.970858 1.656725
H 4.785281 -1.771434 1.967650
H 4.812928 0.756908 -1.425373
H 7.005428 0.675199 -2.005688
H 8.305902 2.152779 -0.584217
H 6.634904 2.674883 -0.705213
H 7.859777 1.232095 1.705410
H 7.469333 2.948971 1.581532
H 6.167582 1.756430 1.578328
H 8.898429 -0.307135 -1.012441
H 7.618058 -1.521208 -1.021015
H 8.070953 -0.699594 0.484926
SCF Energy (B3LYP/6-31G**)= -1361.70370915
Number of imaginary frequencies = 0

1b_c333
MMFF Geometry
C 1.960332 1.832354 -0.073288
C 2.209906 -0.870381 -0.208184
N 3.204515 1.302797 0.007429
C 0.785320 1.089826 -0.220686
C 0.926776 -0.308309 -0.291723
C 3.321130 -0.042900 -0.057620
C -0.217849 -1.110964 -0.452280
C -1.497990 -0.544712 -0.526392
C -1.631982 0.842703 -0.440196
C -0.500760 1.660440 -0.304291
C -2.990265 1.421813 -0.500985
C -4.204009 0.508759 -0.244185
C -3.918431 -0.930922 -0.644090
C -2.662760 -1.404236 -0.748990
O -0.601202 3.029816 -0.244832
O -3.122181 2.633300 -0.702949
O -5.263413 1.036909 -1.059499
C -4.583213 0.680206 1.231086
C -5.616019 -0.310705 1.720496
C -7.045473 -0.114230 1.286641
O -5.293721 -1.238148 2.466358
O -5.062688 -1.678893 -0.855138
C -4.936909 -3.068823 -1.126842
C 4.698735 -0.596989 0.049034
C 4.839333 -1.870194 0.832618
C 5.717923 0.089367 -0.512660
C 7.198515 -0.208238 -0.636602
C 8.039389 0.624012 0.351591
C 7.815296 0.306431 1.824142
C 7.615004 -1.681092 -0.665021
H 1.931854 2.918238 -0.012953
H 2.338804 -1.946161 -0.275387
H -0.103338 -2.192477 -0.520788
H -2.458395 -2.435633 -1.014727
H -1.527929 3.289573 -0.447451
H -5.115500 2.001472 -1.124395
H -4.977239 1.686441 1.422722
H -3.699767 0.568621 1.873932
H -7.694686 -0.779133 1.863620
H -7.350335 0.917151 1.480895
H -7.158910 -0.346714 0.226695
H -4.406103 -3.236001 -2.069412
H -4.441365 -3.586248 -0.299083
H -5.943903 -3.483894 -1.229149
H 4.785355 -2.740689 0.171451
H 5.776745 -1.901262 1.391913
H 4.053881 -1.966093 1.591307
H 5.462490 1.032940 -1.004207
H 7.463875 0.164125 -1.638139
H 9.103282 0.483897 0.122226
H 7.830814 1.690157 0.193340
H 8.068509 -0.731738 2.055929
H 8.452732 0.946949 2.442709
H 6.778572 0.487662 2.122169

H 8.644066 -1.770718 -1.032209
H 6.976708 -2.258628 -1.342346
H 7.591829 -2.154572 0.318395
SCF Energy (B3LYP/6-31G**)= -1361.70008733
Number of imaginary frequencies = 0

1b_c334
MMFF Geometry
C -1.574908 -1.552259 -0.934065
C -2.163939 0.944437 -0.041465
N -2.879760 -1.216164 -0.790275
C -0.498644 -0.706085 -0.657041
C -0.815333 0.584587 -0.194956
C -3.169909 0.027878 -0.344765
C 0.223559 1.487902 0.097307
C 1.567211 1.120878 -0.053149
C 1.873983 -0.169430 -0.498925
C 0.852563 -1.074685 -0.816899
C 3.297677 -0.533599 -0.662066
C 4.367518 0.295135 0.076691
C 3.914623 1.733989 0.235974
C 2.623296 2.098578 0.213592
O 1.122725 -2.334686 -1.293283
O 3.607632 -1.486960 -1.382994
O 5.547914 0.270090 -0.744748
C 4.720603 -0.282795 1.449094
C 4.768615 -1.791559 1.449231
C 6.071865 -2.453396 1.090747
O 3.756584 -2.448535 1.696264
O 4.891330 2.653046 0.588904
C 5.313856 3.416126 -0.541814
C -4.617934 0.366496 -0.214143
C -4.984675 1.812948 -0.395872
C -5.504491 -0.626292 0.023082
C -7.006373 -0.587377 0.221637
C -7.450699 0.364432 1.348837
C -8.904800 0.167150 1.759726
C -7.742311 -0.359419 -1.100726
H -1.410714 -2.565857 -1.293669
H -2.417627 1.933053 0.326704
H -0.024248 2.490478 0.444470
H 2.340033 3.127558 0.415108
H 2.092908 -2.427904 -1.419397
H 5.453551 -0.494714 -1.348423
H 3.976020 0.001825 2.204579
H 5.688708 0.090637 1.804896
H 5.997586 -3.528969 1.274402
H 6.296139 -2.288446 0.035135
H 6.873899 -2.051522 1.714794
H 5.699758 2.774974 -1.340380
H 4.495297 4.037871 -0.919853
H 6.121303 4.078649 -0.216592
H -4.772501 2.382380 0.514994
H -4.424569 2.258291 -1.226233
H -6.037821 1.956520 -0.642225
H -5.109151 -1.642082 0.106476
H -7.277574 -1.601415 0.550978
H -6.820502 0.200624 2.232304
H -7.319598 1.411386 1.058685
H -9.095055 -0.870962 2.049657
H -9.141739 0.805865 2.616819
H -9.589844 0.433279 0.949527
H -8.807376 -0.592354 -1.000284
H -7.344107 -1.007418 -1.889835
H -7.662042 0.675936 -1.444044
SCF Energy (B3LYP/6-31G**)= -1361.69378787
Number of imaginary frequencies = 0

1b_c335
MMFF Geometry
C -1.454174 -2.208724 -0.612035
C -2.329276 0.326185 -0.163131
N -2.789341 -2.010015 -0.493028
C -0.485541 -1.203591 -0.522501
C -0.950579 0.105499 -0.292294
C -3.210463 -0.748496 -0.261667
C -0.026741 1.163632 -0.206303
C 1.350508 0.935761 -0.329089
C 1.804362 -0.369312 -0.540090
C 0.898488 -1.432437 -0.655795
C 3.257737 -0.583716 -0.675853
C 4.216671 0.480023 -0.107178
C 3.614415 1.877270 -0.197004
C 2.284099 2.066677 -0.289737
O 1.316866 -2.716929 -0.905408
O 3.676564 -1.602289 -1.233338
O 5.407493 0.438407 -0.914764
C 4.615414 0.195176 1.342823
C 4.841768 -1.274481 1.603027
C 6.206592 -1.839080 1.313771
O 3.918243 -1.986671 1.999580
O 4.556217 2.887856 -0.122447
C 4.128737 4.234609 -0.281006
C -4.676736 -0.573247 -0.139046
C -5.466786 -1.246917 -1.224031
C -5.182721 0.137381 0.891822
C -6.612208 0.418248 1.313476
C -7.040349 1.856566 0.960316
C -7.121467 2.162299 -0.529662
C -7.672593 -0.616958 0.930234
H -1.169381 -3.243764 -0.789496
H -2.715747 1.327588 -0.000156
H -0.393284 2.176566 -0.043392
H 1.844977 3.057489 -0.327573
H 2.289003 -2.714207 -1.050348
H 5.360026 -0.378067 -1.451316
H 3.831013 0.516303 2.041184
H 5.527648 0.735953 1.623336
H 6.271733 -2.856313 1.710153
H 6.378646 -1.866065 0.236047
H 6.970220 -1.229552 1.803212
H 3.647959 4.381810 -1.253506
H 3.462346 4.530757 0.535340
H 5.014264 4.875802 -0.241779
H -4.872546 -1.376794 -2.136388
H -5.806716 -2.235249 -0.899245
H -6.328740 -0.646636 -1.524607
H -4.471373 0.564834 1.602044
H -6.582196 0.378171 2.413230
H -8.019342 2.059816 1.412366
H -6.338762 2.564853 1.420015
H -7.855210 1.526380 -1.032540
H -7.429961 3.202448 -0.678400
H -6.152836 2.032719 -1.021055
H -8.590928 -0.440396 1.502296
H -7.334661 -1.632298 1.163833
H -7.948777 -0.579244 -0.125340
SCF Energy (B3LYP/6-31G**)= -1361.69594709
Number of imaginary frequencies = 0

1b_c336
MMFF Geometry
C 1.847239 -2.572090 -0.005054
C 2.574038 0.025393 -0.336633
N 3.170342 -2.286015 -0.070018
C 0.819862 -1.627783 -0.097245
C 1.208046 -0.286746 -0.272223
C 3.520562 -0.991140 -0.224062
C 0.220537 0.708743 -0.387261
C -1.143452 0.391606 -0.314128
C -1.523011 -0.938975 -0.124239
C -0.551423 -1.947734 -0.032066
C -2.967293 -1.255401 -0.049107
C -3.947089 -0.112034 0.298917
C -3.446916 1.229382 -0.229291
C -2.144921 1.444871 -0.495078
O -0.894046 -3.270747 0.119318
O -3.307881 -2.429498 -0.218666
O -3.928516 -0.052215 1.732368
C -5.336563 -0.520643 -0.232825
C -6.468287 0.328733 0.305852
C -7.631008 0.580233 -0.615681

O -6.464781 0.748348 1.465066
O -4.443407 2.177229 -0.374881
C -4.073158 3.522643 -0.654809
C 4.976750 -0.722523 -0.291611
C 5.755887 -1.720003 -1.106187
C 5.513233 0.331763 0.359589
C 6.976197 0.713629 0.429985
C 7.302456 1.898553 -0.499609
C 6.591106 3.206187 -0.173212
C 7.378242 0.988176 1.882531
H 1.623478 -3.628407 0.128633
H 2.896339 1.051009 -0.487303
H 0.525095 1.744229 -0.534589
H -1.778684 2.400782 -0.851850
H -1.873539 -3.346370 0.057493
H -4.758189 0.393504 2.009347
H -5.589295 -1.547124 0.061148
H -5.343558 -0.481853 -1.328903
H -8.369283 1.211577 -0.113646
H -7.281881 1.095899 -1.513724
H -8.098088 -0.370726 -0.882824
H -3.410769 3.917693 0.122198
H -3.611159 3.601449 -1.643923
H -4.985005 4.127074 -0.660638
H 5.218788 -1.974924 -2.027336
H 6.729922 -1.337161 -1.422457
H 5.919994 -2.639768 -0.535008
H 4.855400 0.979040 0.939576
H 7.596047 -0.128631 0.102258
H 8.384614 2.079050 -0.476574
H 7.060124 1.620514 -1.533463
H 6.858130 3.571302 0.822589
H 6.881889 3.976497 -0.895215
H 5.503713 3.098707 -0.225253
H 8.431245 1.283111 1.943960
H 7.248917 0.088595 2.495220
H 6.776187 1.783062 2.335449
SCF Energy (B3LYP/6-31G**)= -1361.70982877
Number of imaginary frequencies = 0

1b_c337
MMFF Geometry
C -1.667512 -1.542119 -0.667304
C -2.131489 1.019897 0.111804
N -2.951070 -1.163285 -0.454738
C -0.554929 -0.709547 -0.517338
C -0.807033 0.615622 -0.115668
C -3.173859 0.111527 -0.063593
C 0.270233 1.508069 0.039117
C 1.591296 1.096011 -0.182330
C 1.834521 -0.227093 -0.562863
C 0.772244 -1.122488 -0.749809
C 3.235401 -0.640465 -0.791734
C 4.351851 0.190561 -0.116385
C 3.972408 1.669209 -0.064031
C 2.686059 2.066645 -0.078878
O 0.979955 -2.414987 -1.166777
O 3.448762 -1.637181 -1.484029
O 5.556479 0.048035 -0.880808
C 4.650701 -0.312387 1.297729
C 4.659605 -1.821058 1.380558
C 5.883414 -2.551021 0.895297
O 3.672458 -2.424819 1.804615
O 5.064639 2.512461 0.022083
C 4.842097 3.915228 0.107520
C -4.591850 0.494623 0.178569
C -4.839062 1.405387 1.346395
C -5.548627 -0.027604 -0.619622
C -7.047230 0.186149 -0.685062
C -7.826085 -0.995055 -0.073183
C -7.639110 -1.190570 1.425589
C -7.580063 1.543623 -0.219326
H -1.551394 -2.579412 -0.974560
H -2.346288 2.041545 0.408739
H 0.068655 2.537341 0.333734
H 2.401999 3.111740 -0.021082
H 1.935818 -2.534416 -1.367846
H 5.432379 0.552034 -1.703836
H 3.893552 0.037211 2.012140
H 5.619606 0.052029 1.660837
H 5.851645 -3.586527 1.245875
H 5.914881 -2.545166 -0.195927
H 6.781795 -2.079622 1.301494
H 4.333067 4.285237 -0.788204
H 4.279045 4.167043 1.011913
H 5.817152 4.407417 0.169084
H -4.068368 1.291301 2.117602
H -4.851312 2.451955 1.026455
H -5.779328 1.172672 1.850684
H -5.214616 -0.723348 -1.395337
H -7.274403 0.164737 -1.762125
H -8.896045 -0.861935 -0.277076
H -7.531614 -1.922675 -0.581142
H -7.976399 -0.317784 1.991378
H -8.227760 -2.049886 1.763643
H -6.593442 -1.387824 1.678832
H -8.610462 1.679524 -0.568086
H -6.985404 2.364433 -0.634529
H -7.601501 1.648436 0.867132
SCF Energy (B3LYP/6-31G**)= -1361.69143203
Number of imaginary frequencies = 0

1b_c338
MMFF Geometry
C -1.633665 -1.524728 -0.678460
C -2.150490 0.983973 0.225651
N -2.925766 -1.176034 -0.465959
C -0.536396 -0.685952 -0.468124
C -0.815994 0.611610 -0.001731
C -3.178744 0.073335 -0.013247
C 0.244831 1.509444 0.219242
C 1.574993 1.130025 -0.004433
C 1.846193 -0.166650 -0.452309
C 0.800826 -1.066941 -0.700687
C 3.254522 -0.543104 -0.696582
C 4.378674 0.294440 -0.052278
C 3.940562 1.735561 0.140642
C 2.651138 2.100052 0.197823
O 1.033096 -2.334178 -1.178282
O 3.511727 -1.513986 -1.415725
O 5.479521 0.252085 -0.977256
C 4.849344 -0.275918 1.288117
C 4.873205 -1.785247 1.306383
C 6.130189 -2.473406 0.846575
O 3.876858 -2.421669 1.651210
O 4.909023 2.672022 0.475740
C 5.624101 3.142988 -0.666486
C -4.610238 0.416537 0.227306
C -4.885353 1.406168 1.324805
C -5.564705 -0.194124 -0.509830
C -7.070769 -0.026337 -0.509337
C -7.715340 -0.806336 0.650691
C -9.236136 -0.845138 0.581907
C -7.530489 1.431985 -0.600949
H -1.498034 -2.542632 -1.037792
H -2.378419 1.986377 0.573158
H 0.025190 2.517636 0.569234
H 2.386428 3.129094 0.424517
H 1.993834 -2.436865 -1.357860
H 5.339851 -0.533397 -1.545300
H 4.183341 0.029963 2.106153
H 5.852016 0.085782 1.547244
H 6.049453 -3.547175 1.037855
H 6.272809 -2.313902 -0.223959
H 6.987132 -2.086759 1.403659
H 6.487794 2.502732 -0.864883
H 4.987056 3.216987 -1.555054
H 5.999922 4.144116 -0.434526
H -4.752292 2.430962 0.963182
H -5.895423 1.315650 1.728934
H -4.214951 1.246286 2.177224
H -5.234991 -0.926872 -1.250920

H -7.412327 -0.498309 -1.442559
H -7.345514 -1.839888 0.642390
H -7.420230 -0.378720 1.615581
H -9.576379 -1.247909 -0.377236
H -9.631224 -1.485603 1.377098
H -9.668550 0.151046 0.714234
H -8.567805 1.485067 -0.949519
H -6.921992 1.996099 -1.316445
H -7.489681 1.944851 0.363667
SCF Energy (B3LYP/6-31G**)= -1361.69272382
Number of imaginary frequencies = 0

1b_c339
MMFF Geometry
C 1.754491 2.261870 -0.017544
C 2.307566 -0.392111 0.178881
N 3.044256 1.894214 0.174128
C 0.677939 1.375319 -0.121845
C 0.976471 0.003596 -0.017486
C 3.309211 0.573051 0.262127
C -0.062443 -0.939770 -0.116225
C -1.390894 -0.537320 -0.306078
C -1.684023 0.826796 -0.396487
C -0.657830 1.779762 -0.320774
C -3.095356 1.236592 -0.580395
C -4.219097 0.225045 -0.275963
C -3.735178 -1.193491 -0.489060
C -2.440585 -1.542642 -0.457407
O -0.910914 3.126081 -0.432036
O -3.347782 2.394602 -0.929034
O -5.271663 0.523757 -1.206342
C -4.706575 0.521278 1.145875
C -5.664843 -0.504836 1.709135
C -7.048978 -0.582964 1.118788
O -5.330256 -1.223894 2.652870
O -4.713367 -2.168327 -0.607403
C -4.908146 -2.549030 -1.969812
C 4.732426 0.213760 0.477513
C 5.429536 1.076867 1.492452
C 5.291179 -0.792980 -0.228919
C 6.713417 -1.319139 -0.219230
C 7.755721 -0.247089 -0.589793
C 9.138096 -0.825307 -0.865682
C 7.033447 -2.069062 1.076116
H 1.601739 3.336850 -0.086698
H 2.559641 -1.443416 0.279877
H 0.172792 -2.001486 -0.044951
H -2.152277 -2.586250 -0.546145
H -1.850506 3.252923 -0.692956
H -5.253945 1.492365 -1.345226
H -5.211559 1.494719 1.192453
H -3.856216 0.582029 1.838403
H -7.676528 -1.220515 1.748307
H -7.492931 0.415327 1.090322
H -7.015720 -1.008526 0.114964
H -5.166169 -1.688470 -2.595066
H -4.018424 -3.050047 -2.365549
H -5.740904 -3.257504 -2.004213
H 4.805040 1.207557 2.383981
H 6.370011 0.649267 1.842164
H 5.646989 2.065628 1.074537
H 4.667994 -1.311918 -0.959034
H 6.745802 -2.072162 -1.020618
H 7.422574 0.287162 -1.488799
H 7.851533 0.502044 0.202372
H 9.095713 -1.593538 -1.644111
H 9.813439 -0.034482 -1.208022
H 9.574952 -1.266227 0.035073
H 7.940671 -2.671691 0.963203
H 6.222495 -2.755637 1.344574
H 7.192544 -1.390411 1.918681
SCF Energy (B3LYP/6-31G**)= -1361.69567206
Number of imaginary frequencies = 0

1b_c340
MMFF Geometry
C -1.476999 -2.259766 -0.486280
C -2.404984 0.239857 0.045062
N -2.803894 -2.109303 -0.258091
C -0.540670 -1.221590 -0.463886
C -1.032221 0.068218 -0.184650
C -3.256365 -0.862100 -0.006430
C -0.140329 1.155762 -0.146321
C 1.230489 0.973246 -0.365498
C 1.713387 -0.308711 -0.639001
C 0.836708 -1.400441 -0.701845
C 3.169411 -0.483013 -0.845546
C 4.125101 0.589816 -0.264340
C 3.463712 1.966127 -0.340558
C 2.132045 2.121594 -0.381585
O 1.285353 -2.668916 -0.980751
O 3.560193 -1.520417 -1.384279
O 5.320548 0.613181 -1.042153
C 4.437562 0.212076 1.196666
C 5.047685 -1.171009 1.308790
C 4.502111 -2.078873 2.377509
O 5.971661 -1.521799 0.575084
O 4.233766 3.108965 -0.524413
C 5.280472 3.308171 0.416910
C -4.713804 -0.742928 0.236607
C -5.290725 -1.838197 1.092603
C -5.419340 0.269725 -0.311235
C -6.908060 0.520675 -0.202427
C -7.204208 1.844042 0.527161
C -6.757234 1.839730 1.983808
C -7.517732 0.548868 -1.607889
H -1.172069 -3.283033 -0.695536
H -2.805059 1.222586 0.275435
H -0.524344 2.155779 0.053464
H 1.702511 3.114978 -0.480406
H 2.213925 -2.610936 -1.305620
H 5.696023 -0.293463 -1.007609
H 3.526701 0.240616 1.806963
H 5.163151 0.892835 1.652757
H 5.028359 -3.036991 2.351190
H 4.649501 -1.619539 3.357905
H 3.438730 -2.256429 2.198252
H 4.903399 3.239635 1.442421
H 6.104955 2.608960 0.256878
H 5.672165 4.319458 0.271502
H -4.631097 -2.056455 1.940718
H -6.258623 -1.574250 1.525593
H -5.418996 -2.755871 0.508924
H -4.898605 0.992587 -0.939986
H -7.406189 -0.288058 0.341173
H -6.723577 2.682930 0.007954
H -8.284216 2.035941 0.500890
H -5.669735 1.756755 2.071429
H -7.059355 2.772222 2.471331
H -7.213329 1.008753 2.531235
H -8.602809 0.690011 -1.556000
H -7.330217 -0.393006 -2.135683
H -7.102170 1.363324 -2.212191
SCF Energy (B3LYP/6-31G**)= -1361.70078941
Number of imaginary frequencies = 0

1b_c341
MMFF Geometry
C -1.561013 -2.028279 -0.870118
C -2.359475 0.556472 -0.599563
N -2.891081 -1.774920 -0.825342
C -0.560140 -1.055981 -0.782080
C -0.985363 0.279377 -0.643461
C -3.277277 -0.488568 -0.684271
C -0.026793 1.305996 -0.560311
C 1.343251 1.018726 -0.591640
C 1.759752 -0.308210 -0.722730
C 0.818955 -1.341355 -0.831516
C 3.213183 -0.591949 -0.731332
C 4.167028 0.453313 -0.099127
C 3.623898 1.861577 -0.342671
C 2.322599 2.101234 -0.562658

O 1.203087 -2.652628 -0.976788
O 3.589285 -1.687617 -1.152285
O 5.447503 0.343614 -0.717988
C 4.268501 0.158354 1.410030
C 4.755921 -1.250812 1.682870
C 4.017485 -2.044016 2.726696
O 5.734360 -1.712064 1.095491
O 4.493314 2.935578 -0.495959
C 5.428293 3.130351 0.557246
C -4.741015 -0.256937 -0.647908
C -5.523380 -1.079794 -1.636025
C -5.276981 0.607957 0.239759
C -6.742440 0.925806 0.445904
C -7.174788 0.671385 1.903059
C -7.082042 -0.794777 2.307849
C -6.996579 2.387847 0.070062
H -1.309465 -3.080692 -0.984097
H -2.711233 1.580131 -0.514521
H -0.357249 2.340648 -0.472514
H 1.983304 3.111433 -0.775883
H 2.166680 -2.678510 -1.181613
H 5.747544 -0.581241 -0.580868
H 3.294130 0.289291 1.896235
H 4.980958 0.819862 1.912770
H 4.470421 -3.034280 2.825934
H 4.077153 -1.528223 3.688093
H 2.973910 -2.163055 2.424672
H 4.924030 3.158369 1.528579
H 6.212057 2.368858 0.546422
H 5.908790 4.101031 0.401767
H -5.641498 -2.106400 -1.273499
H -6.518243 -0.673198 -1.833556
H -5.013448 -1.111405 -2.606088
H -4.614702 1.129317 0.931274
H -7.375306 0.306313 -0.197009
H -6.569274 1.272611 2.593081
H -8.214450 0.997549 2.032230
H -6.047627 -1.151306 2.296076
H -7.468192 -0.928467 3.323471
H -7.672959 -1.424997 1.635603
H -8.057469 2.637315 0.180503
H -6.717457 2.579078 -0.972226
H -6.422731 3.073046 0.704427
SCF Energy (B3LYP/6-31G**)= -1361.70086692
Number of imaginary frequencies = 0

1b_c342
MMFF Geometry
C -1.662532 -1.853272 -0.597436
C -2.088487 0.804519 -0.246474
N -2.943211 -1.416623 -0.520142
C -0.533314 -1.034743 -0.512937
C -0.766224 0.338691 -0.331852
C -3.152004 -0.091765 -0.343321
C 0.329665 1.215258 -0.240690
C 1.650094 0.747202 -0.326544
C 1.880238 -0.622522 -0.499036
C 0.792929 -1.508442 -0.597967
C 3.276817 -1.122344 -0.595193
C 4.450831 -0.129500 -0.424289
C 4.027506 1.316581 -0.294754
C 2.747858 1.707871 -0.229874
O 0.969880 -2.859753 -0.787541
O 3.453903 -2.315752 -0.858988
O 5.254250 -0.230736 -1.619966
C 5.353015 -0.596544 0.724954
C 4.715850 -0.453587 2.088867
C 5.654654 -0.345347 3.261281
O 3.496374 -0.477765 2.252111
O 5.052869 2.232679 -0.108236
C 5.297346 3.000551 -1.286646
C -4.575272 0.354221 -0.276140
C -4.859750 1.753516 -0.746047
C -5.514319 -0.513449 0.163543
C -7.007419 -0.343158 0.358861
C -7.374051 0.841307 1.273494
C -8.829802 0.821215 1.723522
C -7.752728 -0.341080 -0.977830
H -1.564025 -2.927851 -0.735661
H -2.275899 1.861718 -0.090518
H 0.145943 2.279523 -0.096560
H 2.504348 2.753760 -0.067488
H 1.935645 -3.045071 -0.833437
H 4.655464 -0.139854 -2.380594
H 6.289358 -0.023677 0.715933
H 5.639060 -1.648173 0.598938
H 5.079786 -0.245862 4.186177
H 6.270744 -1.245820 3.320759
H 6.286591 0.538548 3.144563
H 5.543826 2.357512 -2.137412
H 4.437660 3.634534 -1.527471
H 6.154879 3.651051 -1.091397
H -4.292162 1.985075 -1.654763
H -5.907548 1.909469 -1.007130
H -4.595016 2.481839 0.027531
H -5.177857 -1.513259 0.450287
H -7.331992 -1.251628 0.887664
H -6.739156 0.819421 2.168442
H -7.185764 1.798485 0.777662
H -9.076576 -0.123727 2.217608
H -9.012877 1.632406 2.435673
H -9.512004 0.961366 0.880021
H -8.827564 -0.483876 -0.825984
H -7.407476 -1.157374 -1.622520
H -7.618531 0.597371 -1.523311
SCF Energy (B3LYP/6-31G**)= -1361.69449670
Number of imaginary frequencies = 0

1b_c343
MMFF Geometry
C -1.621776 -2.350903 -0.272986
C -2.336632 0.268999 -0.175427
N -2.943054 -2.056491 -0.207090
C -0.590817 -1.405178 -0.298608
C -0.973830 -0.053204 -0.250953
C -3.284339 -0.751684 -0.149096
C 0.016031 0.944530 -0.281646
C 1.379127 0.619276 -0.357721
C 1.760437 -0.726196 -0.397016
C 0.779284 -1.733523 -0.371596
C 3.202549 -1.073792 -0.485735
C 4.256267 0.059985 -0.534942
C 3.674862 1.458153 -0.479223
C 2.362388 1.699606 -0.377892
O 1.105888 -3.069134 -0.425538
O 3.511312 -2.266172 -0.579062
O 4.927336 -0.084059 -1.805194
C 5.316489 -0.192244 0.545456
C 4.788932 -0.031900 1.954721
C 5.815695 0.196347 3.032496
O 3.593187 -0.129839 2.228044
O 4.548099 2.533562 -0.365204
C 5.294517 2.780091 -1.555720
C -4.737397 -0.470448 -0.075696
C -5.559723 -1.230522 -1.076194
C -5.205446 0.400344 0.844279
C -6.617226 0.823806 1.201243
C -6.948513 2.227034 0.655363
C -6.997012 2.338031 -0.862999
C -7.738962 -0.184111 0.939028
H -1.402044 -3.415932 -0.307113
H -2.657915 1.305718 -0.148983
H -0.284856 1.991049 -0.240780
H 2.008424 2.721527 -0.272940
H 2.086644 -3.149605 -0.456853
H 4.241740 -0.091532 -2.495608
H 6.160008 0.495969 0.408835
H 5.732550 -1.203663 0.458090
H 5.317756 0.298730 4.000676
H 6.499146 -0.655262 3.072699
H 6.368492 1.115544 2.823424
H 6.157118 2.110392 -1.613462

H 4.671120 2.691034 -2.452141
H 5.672544 3.805611 -1.506053
H -5.965120 -2.144232 -0.630518
H -6.378822 -0.622459 -1.467328
H -4.967302 -1.516386 -1.953520
H -4.473903 0.872465 1.504087
H -6.599236 0.927317 2.297109
H -7.916142 2.549342 1.060205
H -6.206567 2.944206 1.030062
H -7.766012 1.688789 -1.290530
H -7.236356 3.366806 -1.152027
H -6.034633 2.084042 -1.316675
H -8.648848 0.123802 1.467294
H -7.469360 -1.179001 1.309422
H -8.003045 -0.268672 -0.116930
SCF Energy (B3LYP/6-31G**)= -1361.69198250
Number of imaginary frequencies = 0

1b_c344
MMFF Geometry
C 2.012956 -1.681170 0.032892
C 2.260942 1.022811 0.005317
N 3.260243 -1.153308 0.077573
C 0.833434 -0.935442 -0.028096
C 0.973658 0.463433 -0.044377
C 3.380628 0.194194 0.066621
C -0.176895 1.270380 -0.117969
C -1.460223 0.706359 -0.159653
C -1.593378 -0.683392 -0.127379
C -0.455759 -1.503666 -0.078084
C -2.955430 -1.261739 -0.171390
C -4.144020 -0.359081 0.229682
C -3.880764 1.100151 -0.130855
C -2.631736 1.574945 -0.294574
O -0.551921 -2.875267 -0.080146
O -3.064602 -2.452309 -0.477722
O -4.195134 -0.453739 1.660469
C -5.410537 -0.951298 -0.422664
C -6.701380 -0.384294 0.129274
C -7.852397 -0.248476 -0.830469
O -6.822867 -0.099603 1.322615
O -5.029310 1.862555 -0.241139
C -4.904653 3.275054 -0.362988
C 4.768033 0.737949 0.135767
C 4.933131 2.110595 0.730296
C 5.799752 -0.019621 -0.298233
C 7.269564 0.339834 -0.338818
C 8.021689 -0.470395 0.732085
C 9.491220 -0.092289 0.857294
C 7.810697 0.084701 -1.749277
H 1.986141 -2.768632 0.048748
H 2.378472 2.101110 -0.017996
H -0.065042 2.353697 -0.142139
H -2.435359 2.614417 -0.531388
H -1.496704 -3.119679 -0.208835
H -5.103885 -0.198528 1.930254
H -5.479749 -2.032412 -0.248427
H -5.380086 -0.794879 -1.507811
H -8.714949 0.180516 -0.313115
H -7.569356 0.416415 -1.650280
H -8.123140 -1.233420 -1.218327
H -4.359785 3.696298 0.488033
H -4.425156 3.543259 -1.309640
H -5.912003 3.701763 -0.363722
H 4.292535 2.235990 1.610864
H 5.951218 2.303310 1.078632
H 4.676206 2.881610 -0.003440
H 5.580443 -1.018486 -0.682045
H 7.410328 1.406072 -0.134460
H 7.545373 -0.310348 1.708195
H 7.947579 -1.544658 0.519937
H 9.606997 0.980841 1.038785
H 9.945675 -0.628319 1.696833
H 10.052216 -0.355396 -0.044058
H 8.838519 0.447280 -1.850030
H 7.208057 0.607974 -2.500598
H 7.803230 -0.982991 -1.996002
SCF Energy (B3LYP/6-31G**)= -1361.71172022
Number of imaginary frequencies = 0

1b_c345
MMFF Geometry
C 1.851522 2.130338 0.213753
C 2.264319 -0.557631 0.233483
N 3.110731 1.683854 0.438256
C 0.740466 1.309692 -0.008187
C 0.966231 -0.080382 0.004379
C 3.301962 0.347906 0.442715
C -0.109875 -0.959037 -0.219190
C -1.406385 -0.475381 -0.438887
C -1.626474 0.904736 -0.435390
C -0.561689 1.795874 -0.239519
C -3.007103 1.395979 -0.646300
C -4.172345 0.419561 -0.364782
C -3.778554 -1.005338 -0.738456
C -2.494782 -1.409178 -0.738673
O -0.747210 3.157379 -0.261629
O -3.160015 2.572317 -0.980596
O -5.298201 0.832675 -1.147558
C -4.543409 0.588326 1.113140
C -5.495742 -0.472777 1.616341
C -6.949035 -0.354432 1.238312
O -5.087393 -1.410767 2.304990
O -4.860839 -1.806576 -1.052932
C -4.656183 -3.210946 -1.162522
C 4.688607 -0.109467 0.684388
C 5.345983 0.477062 1.900146
C 5.272044 -0.933146 -0.212953
C 6.650942 -1.563423 -0.254064
C 7.538905 -0.903300 -1.329251
C 7.899090 0.553352 -1.065507
C 7.390463 -1.730285 1.074877
H 1.753493 3.213955 0.217928
H 2.467476 -1.624078 0.258267
H 0.072236 -2.033361 -0.221995
H -2.208379 -2.426305 -0.983173
H -1.662054 3.346425 -0.573563
H -5.119409 0.565310 -2.066092
H -5.008469 1.565821 1.293585
H -3.651852 0.550960 1.752908
H -7.543909 -1.018929 1.871521
H -7.292220 0.670265 1.401374
H -7.092106 -0.638267 0.194216
H -4.024655 -3.445708 -2.025049
H -4.230216 -3.618970 -0.240133
H -5.631231 -3.680852 -1.321331
H 4.732997 1.244867 2.384906
H 5.522917 -0.298070 2.652183
H 6.293950 0.956893 1.638819
H 4.683476 -1.208952 -1.091397
H 6.472098 -2.592227 -0.601508
H 8.467049 -1.479766 -1.431008
H 7.034233 -0.963836 -2.302307
H 8.464617 0.666950 -0.136494
H 8.523733 0.934232 -1.880319
H 7.007993 1.185366 -1.010110
H 8.277833 -2.358500 0.935529
H 6.756081 -2.227166 1.816662
H 7.734717 -0.781895 1.493490
SCF Energy (B3LYP/6-31G**)= -1361.69374566
Number of imaginary frequencies = 0

1b_c346
MMFF Geometry
C -1.591671 -1.565813 -0.895808
C -2.183225 0.950312 -0.061690
N -2.896912 -1.227546 -0.760882
C -0.516214 -0.712645 -0.637521
C -0.834251 0.587893 -0.205726
C -3.188315 0.026310 -0.344578
C 0.203961 1.497940 0.066917
C 1.548259 1.128760 -0.074298

C 1.856639 -0.170156 -0.490400
C 0.835601 -1.083412 -0.787664
C 3.280057 -0.535815 -0.648047
C 4.357376 0.319521 0.050594
C 3.895886 1.758423 0.201509
C 2.602117 2.111107 0.179265
O 1.106564 -2.354239 -1.234303
O 3.587245 -1.512063 -1.339794
O 5.510209 0.277558 -0.808768
C 4.754622 -0.231776 1.422369
C 4.792351 -1.740511 1.458716
C 6.080994 -2.420112 1.081162
O 3.783842 -2.383838 1.751431
O 4.834289 2.707975 0.582270
C 5.616738 3.168231 -0.519402
C -4.636696 0.366868 -0.223101
C -5.004103 1.808617 -0.437950
C -5.522910 -0.620822 0.035721
C -7.025008 -0.578308 0.231855
C -7.470978 0.398403 1.336886
C -8.925401 0.209657 1.750630
C -7.759771 -0.380658 -1.096028
H -1.426414 -2.587286 -1.231832
H -2.437725 1.947055 0.283297
H -0.044627 2.507778 0.391915
H 2.315470 3.139909 0.378752
H 2.076926 -2.449627 -1.357221
H 5.410801 -0.515647 -1.374568
H 4.039025 0.075666 2.196773
H 5.736850 0.143150 1.735396
H 5.999898 -3.492584 1.279445
H 6.284653 -2.270501 0.019106
H 6.899829 -2.018477 1.683150
H 6.498306 2.534940 -0.650038
H 5.038007 3.219763 -1.448528
H 5.965975 4.177480 -0.281166
H -4.443631 2.235274 -1.277820
H -6.057144 1.945962 -0.688263
H -4.792931 2.398735 0.459889
H -5.127046 -1.634201 0.142562
H -7.295972 -1.584821 0.583703
H -6.841608 0.254856 2.224459
H -7.340099 1.438628 1.023375
H -9.115436 -0.821767 2.063656
H -9.163543 0.867374 2.592891
H -9.609744 0.457129 0.933950
H -8.824790 -0.611954 -0.991405
H -7.360384 -1.046010 -1.869952
H -7.679808 0.646753 -1.462501
SCF Energy (B3LYP/6-31G**)= -1361.69304249
Number of imaginary frequencies = 0

1b_c347
MMFF Geometry
C -1.478119 -2.130282 -0.749271
C -2.362195 0.360820 -0.112433
N -2.815034 -1.941847 -0.633051
C -0.511755 -1.135454 -0.567408
C -0.981719 0.151250 -0.240465
C -3.240713 -0.702717 -0.307709
C -0.060971 1.199350 -0.057942
C 1.317339 0.981783 -0.181353
C 1.778465 -0.303065 -0.489140
C 0.874538 -1.353426 -0.699510
C 3.236245 -0.509072 -0.626027
C 4.196072 0.516558 0.009569
C 3.573042 1.899461 0.017610
C 2.247342 2.102061 -0.031473
O 1.296350 -2.615083 -1.042992
O 3.661173 -1.490218 -1.243703
O 5.374027 0.544793 -0.815280
C 4.610927 0.136322 1.432757
C 4.839253 -1.347030 1.594539
C 6.213627 -1.883257 1.296964
O 3.912279 -2.089659 1.920239
O 4.431095 2.961572 0.260145
C 4.761655 3.643914 -0.950018
C -4.708714 -0.538068 -0.190836
C -5.483814 -1.124540 -1.335565
C -5.228943 0.089287 0.885963
C -6.664074 0.335976 1.309763
C -7.089315 1.797507 1.065058
C -7.151503 2.219254 -0.397251
C -7.718179 -0.666131 0.832700
H -1.189909 -3.148031 -1.004200
H -2.751662 1.346156 0.124719
H -0.429366 2.196288 0.181855
H 1.841653 3.105347 0.060794
H 2.271302 -2.603977 -1.166391
H 5.374131 -0.286013 -1.332967
H 3.834928 0.409731 2.160248
H 5.526063 0.658882 1.737274
H 6.267978 -2.933935 1.595667
H 6.420306 -1.807322 0.227692
H 6.959551 -1.323662 1.866741
H 5.223499 2.971468 -1.679565
H 3.875310 4.119215 -1.383607
H 5.483068 4.429211 -0.705993
H -4.877728 -1.181979 -2.247565
H -5.826525 -2.135491 -1.093976
H -6.342670 -0.502797 -1.599098
H -4.527379 0.460007 1.636692
H -6.648246 0.209692 2.403275
H -8.074317 1.964477 1.518906
H -6.394577 2.467636 1.588015
H -7.877920 1.624653 -0.957989
H -7.459281 3.267818 -0.467879
H -6.176439 2.128814 -0.884669
H -8.644036 -0.535206 1.404904
H -7.382072 -1.696619 0.990209
H -7.980757 -0.545689 -0.220145
SCF Energy (B3LYP/6-31G**)= -1361.69086691
Number of imaginary frequencies = 0

1b_c348
MMFF Geometry
C 1.939611 1.845052 -0.053287
C 2.176500 -0.855438 -0.249244
N 3.180465 1.307076 0.023969
C 0.762213 1.112215 -0.226672
C 0.896912 -0.285041 -0.329589
C 3.290633 -0.037341 -0.071048
C -0.250133 -1.078152 -0.519269
C -1.525993 -0.503058 -0.588567
C -1.653739 0.883323 -0.468082
C -0.519775 1.691822 -0.306376
C -3.011924 1.470282 -0.519769
C -4.210533 0.546105 -0.204029
C -3.944640 -0.866652 -0.712611
C -2.694870 -1.344842 -0.856197
O -0.615863 3.059830 -0.217971
O -3.115068 2.675438 -0.755993
O -5.369990 1.083942 -0.849981
C -4.441320 0.622443 1.309702
C -5.412383 -0.417899 1.820365
C -6.882423 -0.187271 1.586372
O -5.006680 -1.428291 2.399454
O -5.100115 -1.575422 -0.985941
C -4.998359 -2.976331 -1.216590
C 4.664509 -0.601009 0.033218
C 4.792784 -1.891550 0.790069
C 5.691233 0.092017 -0.506100
C 7.171172 -0.210495 -0.625706
C 8.008909 0.595977 0.386242
C 7.772378 0.248001 1.849953
C 7.580447 -1.684540 -0.682724
H 1.915847 2.929387 0.032427
H 2.300780 -1.929996 -0.340159
H -0.140614 -2.158100 -0.614310
H -2.497742 -2.355244 -1.197608
H -1.539556 3.326121 -0.432048
H -5.288092 0.879181 -1.797890

H -4.825797 1.608296 1.600562
H -3.502226 0.485291 1.861841
H -7.461442 -0.864894 2.220366
H -7.143983 0.839180 1.855353
H -7.133265 -0.378779 0.541589
H -4.458936 -3.179041 -2.147090
H -4.523617 -3.480533 -0.368576
H -6.012156 -3.373715 -1.321718
H 4.739699 -2.747246 0.109788
H 5.725671 -1.939423 1.355718
H 4.000975 -1.999908 1.540429
H 5.444173 1.047497 -0.978591
H 7.445756 0.181899 -1.617055
H 9.073747 0.455316 0.161645
H 7.806860 1.666341 0.249458
H 8.018666 -0.796203 2.061181
H 8.408441 0.871780 2.486773
H 6.734407 0.428147 2.144275
H 8.611777 -1.771608 -1.044119
H 6.944361 -2.244068 -1.377031
H 7.547475 -2.178904 0.290080
SCF Energy (B3LYP/6-31G**)= -1361.69400184
Number of imaginary frequencies = 0

1b_c349
MMFF Geometry
C 2.007918 -1.606194 -0.154142
C 2.276349 1.091753 -0.303933
N 3.248697 -1.096409 -0.344239
C 0.843945 -0.843963 -0.029894
C 0.994841 0.551157 -0.107866
C 3.380367 0.248004 -0.418548
C -0.139711 1.373891 0.017281
C -1.419063 0.829829 0.206749
C -1.563647 -0.557307 0.275301
C -0.439757 -1.392178 0.169231
C -2.914982 -1.123836 0.473891
C -4.149154 -0.221182 0.290279
C -3.827847 1.260862 0.399984
C -2.566508 1.726279 0.357476
O -0.542623 -2.760660 0.250600
O -3.020829 -2.322153 0.753774
O -5.010355 -0.576983 1.382894
C -4.792342 -0.547983 -1.067094
C -6.275777 -0.823484 -0.985065
C -7.212011 0.346056 -0.844087
O -6.700121 -1.980618 -1.007636
O -4.955098 2.054933 0.513995
C -4.793208 3.436803 0.809933
C 4.760939 0.769297 -0.642832
C 4.877667 2.106489 -1.323423
C 5.818590 0.023733 -0.251628
C 7.292411 0.345760 -0.363247
C 7.951093 0.509438 1.022422
C 7.987931 -0.739885 1.894245
C 7.992600 -0.712830 -1.218732
H 1.973566 -2.692429 -0.102820
H 2.403082 2.168264 -0.351239
H -0.016999 2.455462 -0.033938
H -2.345255 2.786081 0.421600
H -1.465856 -2.996334 0.498538
H -5.067780 -1.554632 1.388522
H -4.335081 -1.441636 -1.513639
H -4.623797 0.243002 -1.808801
H -8.216737 0.044959 -1.154366
H -7.243310 0.675079 0.196277
H -6.888509 1.164315 -1.492222
H -4.237971 3.576762 1.743054
H -4.305438 3.957759 -0.019928
H -5.788720 3.871202 0.940595
H 4.674395 2.917081 -0.615824
H 5.863986 2.283659 -1.757972
H 4.170615 2.183192 -2.157570
H 5.614883 -0.942758 0.213731
H 7.433913 1.302832 -0.874551
H 8.982227 0.857926 0.881252
H 7.432453 1.303906 1.574490
H 8.555052 -1.545468 1.419269
H 8.476713 -0.511528 2.847304
H 6.982707 -1.106520 2.120266
H 9.067527 -0.512512 -1.281373
H 7.594071 -0.708778 -2.239770
H 7.857727 -1.723223 -0.818045
SCF Energy (B3LYP/6-31G**)= -1361.70294002
Number of imaginary frequencies = 0

1b_c350
MMFF Geometry
C 1.881761 1.842260 -0.102129
C 2.186563 -0.856943 -0.103045
N 3.136860 1.341299 -0.007646
C 0.720887 1.071206 -0.200408
C 0.890948 -0.325289 -0.202381
C 3.284652 -0.003538 -0.006201
C -0.238098 -1.157902 -0.310861
C -1.529083 -0.621115 -0.401704
C -1.693210 0.767165 -0.383285
C -0.577293 1.612195 -0.299502
C -3.065507 1.318593 -0.462494
C -4.265639 0.396374 -0.165977
C -3.931679 -1.041464 -0.502311
C -2.676805 -1.510795 -0.564908
O -0.703569 2.980721 -0.308050
O -3.221404 2.517125 -0.718238
O -5.328792 0.858848 -1.013597
C -4.652225 0.631411 1.297699
C -5.673741 -0.342268 1.842520
C -7.087152 -0.249815 1.328563
O -5.361400 -1.157242 2.713092
O -5.003912 -1.909821 -0.634421
C -5.299422 -2.168445 -2.007318
C 4.678600 -0.519490 0.117587
C 4.842826 -1.842882 0.811522
C 5.700343 0.228960 -0.354829
C 7.189875 -0.047229 -0.395717
C 7.844205 0.235923 0.968545
C 9.365461 0.175766 0.933615
C 7.548875 -1.417812 -0.978006
H 1.832729 2.929030 -0.095570
H 2.330052 -1.932603 -0.116235
H -0.103957 -2.239313 -0.324799
H -2.495141 -2.567241 -0.741000
H -1.637996 3.213118 -0.507553
H -5.223824 1.829060 -1.087661
H -5.060331 1.640573 1.438383
H -3.766867 0.564414 1.944266
H -7.739281 -0.874812 1.945561
H -7.436017 0.783640 1.397479
H -7.144865 -0.598155 0.296475
H -5.502894 -1.243587 -2.556238
H -4.482074 -2.718950 -2.484937
H -6.197275 -2.792159 -2.045309
H 4.154666 -1.931234 1.660197
H 4.651017 -2.668719 0.118786
H 5.841278 -1.975834 1.232409
H 5.451027 1.194042 -0.803362
H 7.598304 0.686781 -1.106173
H 7.548649 1.235837 1.311888
H 7.484071 -0.470972 1.724555
H 9.768641 0.853893 0.174976
H 9.774846 0.472607 1.904651
H 9.721422 -0.836476 0.720229
H 8.592103 -1.430166 -1.312475
H 6.929152 -1.653513 -1.850252
H 7.436305 -2.224606 -0.248906
SCF Energy (B3LYP/6-31G**)= -1361.69579444
Number of imaginary frequencies = 0

1b_c351
MMFF Geometry
C 1.998107 -1.726215 -0.357037
C 2.274733 0.975083 -0.357066

N 3.251407 -1.212051 -0.392064
C 0.825928 -0.967459 -0.321336
C 0.980828 0.429935 -0.321844
C 3.386409 0.134150 -0.391156
C -0.162557 1.249826 -0.292851
C -1.451971 0.699828 -0.250834
C -1.598569 -0.688865 -0.237156
C -0.469975 -1.521447 -0.287725
C -2.966762 -1.252477 -0.192526
C -4.112437 -0.346635 0.312694
C -3.859087 1.117396 -0.035212
C -2.619997 1.582936 -0.280595
O -0.581890 -2.891694 -0.308818
O -3.111716 -2.435267 -0.513829
O -4.060853 -0.472023 1.741083
C -5.429749 -0.911555 -0.258205
C -6.670516 -0.343827 0.397994
C -7.886350 -0.175261 -0.472483
O -6.701941 -0.084278 1.602779
O -5.003520 1.893796 -0.044067
C -4.871308 3.307433 -0.142534
C 4.780960 0.662069 -0.443694
C 4.969541 2.029704 -1.042560
C 5.796878 -0.104231 0.011854
C 7.270617 0.235618 0.075519
C 7.781098 0.020686 1.513967
C 9.224335 0.461575 1.717358
C 8.026723 -0.619388 -0.943491
H 1.959974 -2.813454 -0.358220
H 2.403170 2.052296 -0.343575
H -0.040175 2.332229 -0.302984
H -2.429418 2.625355 -0.509035
H -1.536288 -3.123899 -0.373000
H -4.944555 -0.213434 2.081580
H -5.498782 -1.995482 -0.102342
H -5.476197 -0.731662 -1.339140
H -8.704105 0.250912 0.115172
H -7.655676 0.504652 -1.296371
H -8.195950 -1.148629 -0.860496
H -4.260393 3.702888 0.675368
H -4.459502 3.592991 -1.115606
H -5.870827 3.744311 -0.059275
H 4.707552 2.807109 -0.317383
H 5.994822 2.211582 -1.374721
H 4.345109 2.156318 -1.934506
H 5.558821 -1.096705 0.401312
H 7.435196 1.287770 -0.176890
H 7.150186 0.588547 2.210257
H 7.689580 -1.035446 1.798172
H 9.363344 1.504454 1.415692
H 9.496557 0.376131 2.774375
H 9.919140 -0.161319 1.146759
H 9.079474 -0.326199 -1.003251
H 7.601288 -0.501120 -1.946715
H 7.985895 -1.683380 -0.684382
SCF Energy (B3LYP/6-31G**)= -1361.71176778
Number of imaginary frequencies = 0

1b_c352
MMFF Geometry
C 1.861489 1.589769 0.030924
C 2.091779 -1.099931 0.318722
N 3.095557 1.072904 0.243510
C 0.685932 0.839462 -0.051967
C 0.817887 -0.551298 0.097431
C 3.208263 -0.267848 0.386945
C -0.328499 -1.361996 0.021954
C -1.599433 -0.810577 -0.198560
C -1.728951 0.573288 -0.345597
C -0.591759 1.395638 -0.273202
C -3.076196 1.155646 -0.565356
C -4.308169 0.235378 -0.663761
C -3.989662 -1.232559 -0.499768
C -2.757857 -1.696009 -0.256174
O -0.671375 2.761859 -0.408971
O -3.178710 2.379955 -0.699114
O -4.747079 0.442583 -2.025821
C -5.419712 0.743819 0.266003
C -5.244450 0.380167 1.725705
C -4.127015 1.035950 2.493160
O -5.999071 -0.419248 2.280251
O -5.041991 -2.136811 -0.458365
C -5.713654 -2.273511 -1.709342
C 4.581910 -0.796783 0.635213
C 4.680738 -2.101816 1.378482
C 5.649466 -0.085595 0.208416
C 7.118779 -0.422550 0.334649
C 7.772902 -0.664063 -1.041762
C 7.825499 0.539721 -1.974703
C 7.835146 0.667240 1.135877
H 1.843119 2.672305 -0.075292
H 2.201883 -2.174437 0.422432
H -0.224648 -2.440846 0.138099
H -2.607080 -2.760176 -0.093752
H -1.605675 3.011500 -0.586769
H -4.868301 1.404759 -2.141235
H -6.389247 0.344079 -0.060533
H -5.535415 1.833042 0.201506
H -4.355514 1.004364 3.562472
H -3.190374 0.505671 2.314556
H -4.034127 2.084327 2.199195
H -6.422992 -1.454445 -1.857459
H -5.007256 -2.334085 -2.544482
H -6.284790 -3.206307 -1.680785
H 4.462427 -2.941937 0.710930
H 5.665819 -2.273616 1.817899
H 3.975783 -2.127657 2.217514
H 5.458831 0.860142 -0.302695
H 7.247729 -1.354853 0.892838
H 8.799364 -1.019340 -0.884402
H 7.242459 -1.477751 -1.553121
H 8.404441 1.360009 -1.540946
H 8.309508 0.257505 -2.915694
H 6.825038 0.908529 -2.217701
H 8.907233 0.455068 1.207517
H 7.438155 0.719585 2.156181
H 7.713948 1.658281 0.685563
SCF Energy (B3LYP/6-31G**)= -1361.69739093
Number of imaginary frequencies = 0

1b_c353
MMFF Geometry
C -1.893516 -2.401515 0.043290
C -2.519102 0.185262 -0.504905
N -3.194919 -2.096093 -0.179798
C -0.840354 -1.482017 0.013673
C -1.176044 -0.145965 -0.272202
C -3.496078 -0.806841 -0.443847
C -0.161049 0.827439 -0.313731
C 1.180698 0.488945 -0.086555
C 1.509964 -0.840910 0.184725
C 0.507268 -1.820891 0.250525
C 2.931282 -1.180341 0.422075
C 4.010925 -0.204549 -0.098091
C 3.515848 1.238628 -0.072835
C 2.203253 1.537430 -0.079703
O 0.795500 -3.132344 0.546630
O 3.180455 -2.253028 0.979336
O 4.196670 -0.579379 -1.470581
C 5.287770 -0.447600 0.733165
C 6.528557 0.188382 0.142926
C 7.562434 0.694981 1.111868
O 6.710664 0.238451 -1.075350
O 4.532535 2.176161 -0.069152
C 4.200851 3.546740 -0.261725
C -4.929857 -0.514010 -0.689097
C -5.633898 -1.545758 -1.529717
C -5.504756 0.578300 -0.140959
C -6.942695 1.032183 -0.270568
C -7.761804 0.711027 0.995100
C -7.307523 1.410264 2.270615
C -6.989416 2.520570 -0.629995

H -1.710158 -3.452903 0.254747
H -2.799020 1.206186 -0.745584
H -0.426328 1.862328 -0.526631
H 1.843700 2.560121 -0.081731
H 1.750966 -3.195865 0.774687
H 5.080157 -0.246376 -1.739462
H 5.522728 -1.517402 0.798536
H 5.143221 -0.080107 1.756393
H 8.397206 1.138038 0.561831
H 7.119416 1.461105 1.753016
H 7.934790 -0.135469 1.716479
H 3.675360 3.694285 -1.210754
H 3.610898 3.926192 0.578494
H 5.134612 4.115426 -0.300737
H -5.710282 -2.497450 -0.992486
H -6.648098 -1.257123 -1.812448
H -5.085205 -1.717417 -2.462872
H -4.897059 1.217933 0.499552
H -7.428863 0.508617 -1.099933
H -8.811275 0.975031 0.812861
H -7.744250 -0.372801 1.168050
H -7.376563 2.497974 2.179276
H -7.947757 1.108917 3.106409
H -6.278567 1.146672 2.531835
H -8.025107 2.868610 -0.706092
H -6.507017 2.698976 -1.597906
H -6.476206 3.141742 0.111810
SCF Energy (B3LYP/6-31G**)= -1361.71035773
Number of imaginary frequencies = 0

1b_c354
MMFF Geometry
C 1.692534 -1.996921 -0.398532
C 2.409565 0.624539 -0.487410
N 3.013532 -1.703198 -0.467801
C 0.663215 -1.049825 -0.370192
C 1.046418 0.304308 -0.419345
C 3.355245 -0.398172 -0.505887
C 0.056950 1.304644 -0.407856
C -1.303536 0.977444 -0.330102
C -1.675959 -0.366640 -0.263558
C -0.705551 -1.378278 -0.302721
C -3.116781 -0.686303 -0.183065
C -4.069479 0.398528 0.375743
C -3.604102 1.785789 -0.077208
C -2.316131 2.034531 -0.383829
O -1.048109 -2.709274 -0.280770
O -3.478148 -1.814377 -0.525485
O -3.991422 0.414055 1.804591
C -5.509749 0.107661 -0.094122
C -6.048358 -1.192628 0.469834
C -7.015155 -1.962804 -0.387865
O -5.750159 -1.570019 1.605336
O -4.612432 2.735141 -0.089985
C -4.263855 4.097655 -0.304650
C 4.805050 -0.107426 -0.571663
C 5.549188 -0.836878 -1.652458
C 5.352303 0.704504 0.358593
C 6.775874 1.185146 0.564668
C 7.427888 0.503938 1.785788
C 7.650931 -0.996742 1.646653
C 7.703256 1.186467 -0.652198
H 1.471833 -3.061763 -0.364435
H 2.734597 1.659489 -0.534292
H 0.356681 2.350644 -0.459529
H -1.970648 3.019219 -0.676716
H -2.026156 -2.782092 -0.357292
H -4.454539 -0.390625 2.125323
H -5.548295 0.062022 -1.189114
H -6.210451 0.880321 0.244308
H -7.314685 -2.879769 0.127027
H -7.902384 -1.353433 -0.575826
H -6.534252 -2.231944 -1.331520
H -3.560714 4.448174 0.457803
H -3.858064 4.242333 -1.310903
H -5.176646 4.694967 -0.221556
H 4.923027 -1.562651 -2.182957
H 5.915524 -0.134283 -2.407340
H 6.390694 -1.399088 -1.236549
H 4.685631 1.096208 1.130967
H 6.670816 2.247279 0.832931
H 8.393595 0.983172 1.990389
H 6.806061 0.680151 2.673187
H 8.320616 -1.229286 0.814044
H 8.111187 -1.389390 2.559428
H 6.709014 -1.532085 1.495772
H 8.629945 1.723308 -0.418655
H 7.237012 1.700289 -1.499654
H 7.989451 0.181793 -0.971460
SCF Energy (B3LYP/6-31G**)= -1361.70357103
Number of imaginary frequencies = 0

1b_c355
MMFF Geometry
C -1.638895 -1.673701 -0.788588
C -2.218344 0.936895 -0.308453
N -2.941563 -1.319422 -0.670339
C -0.560643 -0.792297 -0.682006
C -0.872207 0.557831 -0.432013
C -3.226135 -0.018745 -0.431073
C 0.168285 1.499329 -0.321546
C 1.507891 1.110414 -0.437051
C 1.809747 -0.236072 -0.669425
C 0.787398 -1.181866 -0.813264
C 3.236245 -0.617047 -0.782282
C 4.277811 0.298223 -0.087028
C 3.855907 1.753841 -0.258664
C 2.572947 2.113809 -0.412799
O 1.055751 -2.500306 -1.088938
O 3.529574 -1.651380 -1.383079
O 5.560121 0.110428 -0.691871
C 4.409230 -0.070585 1.395312
C 4.386421 -1.565698 1.621667
C 5.642580 -2.344865 1.336231
O 3.353259 -2.119820 1.999741
O 4.831018 2.727835 -0.422247
C 5.657412 2.911709 0.724011
C -4.671159 0.336406 -0.322556
C -5.052872 1.723639 -0.757101
C -5.543466 -0.596275 0.120757
C -7.036806 -0.519732 0.357879
C -7.527070 0.646907 1.237529
C -6.856677 0.696287 2.604762
C -7.813572 -0.590974 -0.959315
H -1.478050 -2.732729 -0.979145
H -2.469394 1.972483 -0.103876
H -0.074730 2.547270 -0.149785
H 2.307708 3.154892 -0.574791
H 2.021372 -2.602830 -1.244263
H 5.479414 0.409258 -1.616378
H 3.584230 0.351218 1.984252
H 5.337552 0.310730 1.835048
H 5.551248 -3.349394 1.759014
H 5.797108 -2.425225 0.258541
H 6.498290 -1.852234 1.804599
H 5.063256 2.960191 1.642906
H 6.410432 2.121931 0.791242
H 6.182649 3.864495 0.608832
H -4.502154 2.019108 -1.657644
H -6.108724 1.809173 -1.019351
H -4.841266 2.448453 0.035821
H -5.139820 -1.579832 0.375130
H -7.293379 -1.436770 0.909069
H -7.393979 1.610999 0.737804
H -8.607954 0.536584 1.394309
H -5.786962 0.909992 2.522294
H -7.306634 1.486781 3.214084
H -6.980907 -0.251947 3.137337
H -8.886423 -0.701460 -0.766663
H -7.495488 -1.452253 -1.557514
H -7.678420 0.308411 -1.567273
SCF Energy (B3LYP/6-31G**)= -1361.68795025

Number of imaginary frequencies = 0

1b_c356
MMFF Geometry
C -1.568340 -1.937895 -0.387552
C -2.240874 0.566455 0.431419
N -2.868543 -1.694827 -0.093218
C -0.539351 -0.993902 -0.301901
C -0.899404 0.299356 0.123955
C -3.188456 -0.447349 0.309618
C 0.089131 1.295812 0.225285
C 1.429473 1.020965 -0.077548
C 1.780375 -0.269174 -0.485214
C 0.806497 -1.268509 -0.616296
C 3.195694 -0.532650 -0.807857
C 4.271003 0.410228 -0.234166
C 3.746150 1.834497 -0.096091
C 2.427055 2.094805 -0.015786
O 1.119880 -2.532705 -1.053159
O 3.491676 -1.495720 -1.521006
O 5.364171 0.410392 -1.170624
C 4.804829 -0.063797 1.119721
C 4.970453 -1.562893 1.184144
C 6.260834 -2.151343 0.680324
O 4.054128 -2.273935 1.599055
O 4.748557 2.784379 -0.014012
C 4.386863 4.158661 0.032249
C -4.616300 -0.210330 0.618901
C -5.230404 -1.197578 1.568878
C -5.264366 0.794081 -0.009780
C -6.697378 1.281954 0.082814
C -7.501171 0.908056 -1.179881
C -7.721270 -0.585072 -1.387872
C -7.468663 0.966481 1.365940
H -1.364597 -2.957676 -0.707642
H -2.547225 1.551307 0.770772
H -0.197590 2.297813 0.542641
H 2.045677 3.102225 0.109186
H 2.068922 -2.557063 -1.307690
H 5.211568 -0.333186 -1.787533
H 4.119871 0.207563 1.934161
H 5.772158 0.396909 1.354929
H 6.307709 -3.210551 0.948664
H 6.314936 -2.057341 -0.405980
H 7.107028 -1.640559 1.146438
H 3.814268 4.443858 -0.856215
H 3.830366 4.384719 0.947352
H 5.307819 4.749021 0.044106
H -4.552151 -2.019370 1.823635
H -5.493266 -0.709491 2.512471
H -6.122940 -1.656344 1.132881
H -4.692915 1.376659 -0.736562
H -6.614794 2.379324 0.073081
H -8.479572 1.403428 -1.141721
H -6.990894 1.308870 -2.065410
H -8.286308 -1.028021 -0.563058
H -8.293998 -0.751034 -2.306352
H -6.773650 -1.122124 -1.488650
H -8.411085 1.525892 1.385803
H -6.896580 1.265761 2.250660
H -7.724542 -0.091226 1.460699
SCF Energy (B3LYP/6-31G**)= -1361.69576674
Number of imaginary frequencies = 0

1b_c357
MMFF Geometry
C -1.317151 -2.386787 -0.382844
C -2.158348 0.164575 0.029643
N -2.636595 -2.182539 -0.153172
C -0.347480 -1.379981 -0.422639
C -0.794750 -0.062865 -0.206327
C -3.045320 -0.910003 0.042042
C 0.131523 0.995518 -0.234274
C 1.493416 0.759699 -0.460223
C 1.933303 -0.549955 -0.666727
C 1.021529 -1.615132 -0.661724
C 3.380502 -0.783584 -0.873438
C 4.385787 0.314109 -0.462262
C 3.754425 1.694753 -0.533803
C 2.426335 1.880811 -0.540015
O 1.424273 -2.911659 -0.877017
O 3.760412 -1.877338 -1.304157
O 5.446843 0.253929 -1.438104
C 5.003157 -0.101784 0.883131
C 3.995613 -0.262878 1.998804
C 3.884031 -1.625019 2.631882
O 3.300802 0.676032 2.382164
O 4.545828 2.808289 -0.791964
C 5.571073 3.047085 0.162461
C -4.495570 -0.732142 0.292760
C -5.098972 -1.766823 1.204142
C -5.175463 0.276665 -0.293250
C -6.656934 0.573136 -0.183102
C -6.855115 1.881129 0.603930
C -8.316642 2.208093 0.878170
C -7.261874 0.641416 -1.588795
H -1.047117 -3.428824 -0.539979
H -2.522216 1.170315 0.216542
H -0.216062 2.015757 -0.074322
H 2.019802 2.883065 -0.642809
H 2.358634 -2.905642 -1.183279
H 5.516861 -0.677753 -1.724688
H 5.774913 0.594082 1.224623
H 5.532552 -1.056768 0.760308
H 3.108996 -1.610070 3.403172
H 3.610114 -2.362460 1.873484
H 4.837674 -1.892961 3.093114
H 5.195417 2.955139 1.186923
H 6.419976 2.379237 -0.007075
H 5.924933 4.073254 0.024808
H -4.431118 -1.980301 2.047037
H -6.044368 -1.441820 1.646006
H -5.279481 -2.700259 0.660730
H -4.639012 0.957442 -0.954900
H -7.173300 -0.233352 0.347042
H -6.336993 1.805032 1.568849
H -6.396983 2.722253 0.068037
H -8.816843 1.377411 1.385825
H -8.388573 3.090632 1.522090
H -8.859289 2.429002 -0.045471
H -8.351555 0.735189 -1.546228
H -7.037539 -0.269112 -2.156303
H -6.870969 1.495033 -2.153961
SCF Energy (B3LYP/6-31G**)= -1361.69565987
Number of imaginary frequencies = 0

1b_c358
MMFF Geometry
C -1.749845 -1.890660 -0.541484
C -2.450895 0.722711 -0.277110
N -3.063747 -1.573252 -0.633950
C -0.719285 -0.971626 -0.318565
C -1.094872 0.377432 -0.180867
C -3.399725 -0.270475 -0.506864
C -0.105397 1.348043 0.056573
C 1.249226 0.997690 0.143894
C 1.621318 -0.340430 -0.004093
C 0.642774 -1.324071 -0.220639
C 3.055819 -0.698200 0.106625
C 4.126470 0.411632 -0.056999
C 3.554668 1.774473 0.311420
C 2.244118 2.026420 0.426941
O 0.966989 -2.654982 -0.341763
O 3.338154 -1.886465 0.284999
O 4.497585 0.495925 -1.435254
C 5.335715 0.073400 0.840181
C 6.069912 -1.171240 0.381635
C 6.664404 -2.049792 1.448450
O 6.219565 -1.418643 -0.817025
O 4.448731 2.796561 0.611305
C 5.047367 3.330843 -0.571980
C -4.844073 0.049253 -0.618470

C -5.205545 1.190201 -1.523075
C -5.710380 -0.687428 0.110084
C -7.218707 -0.648951 0.253830
C -7.623749 -0.140158 1.654075
C -7.269889 1.314785 1.937818
C -8.023520 0.040724 -0.848495
H -1.536810 -2.951363 -0.656312
H -2.765513 1.755044 -0.159172
H -0.398650 2.391006 0.172350
H 1.902520 3.016864 0.713252
H 1.927738 -2.761855 -0.158573
H 5.037165 -0.301266 -1.635241
H 5.007108 -0.070280 1.876722
H 6.087464 0.871169 0.830990
H 7.149960 -2.913512 0.985988
H 7.407736 -1.484086 2.015256
H 5.873333 -2.406491 2.112656
H 5.818783 2.656578 -0.955146
H 4.298764 3.540716 -1.343925
H 5.531587 4.274287 -0.302875
H -4.335228 1.616970 -2.033319
H -5.879921 0.859456 -2.318060
H -5.675477 1.999711 -0.955728
H -5.274106 -1.477407 0.729614
H -7.530575 -1.703250 0.212160
H -8.705820 -0.268412 1.783751
H -7.148889 -0.767752 2.419498
H -7.777223 1.994612 1.247613
H -7.582931 1.581406 2.952731
H -6.192220 1.489160 1.867938
H -9.096590 -0.112808 -0.686096
H -7.784040 -0.382283 -1.829815
H -7.855585 1.119929 -0.884005
SCF Energy (B3LYP/6-31G**)= -1361.69642920
Number of imaginary frequencies = 0

1b_c359
MMFF Geometry
C 1.788587 -1.680446 -0.099395
C 2.319241 0.984529 -0.058293
N 3.085249 -1.289924 -0.054996
C 0.694084 -0.811651 -0.126088
C 0.980558 0.566022 -0.104489
C 3.341664 0.037664 -0.035195
C -0.078349 1.492484 -0.136294
C -1.413392 1.067466 -0.174010
C -1.690277 -0.300981 -0.179380
C -0.648014 -1.238979 -0.174935
C -3.105660 -0.724695 -0.220591
C -4.174707 0.268424 0.296669
C -3.779486 1.701204 -0.073757
C -2.493877 2.051686 -0.271597
O -0.892384 -2.590851 -0.219886
O -3.356475 -1.862112 -0.625261
O -4.206095 0.235659 1.726994
C -5.550181 -0.105523 -0.293254
C -6.033203 -1.460814 0.185068
C -6.873048 -2.264757 -0.769841
O -5.795126 -1.858407 1.327848
O -4.850833 2.576876 -0.133612
C -4.587886 3.967929 -0.274435
C 4.775415 0.437234 0.001012
C 5.146109 1.641716 -0.815208
C 5.640153 -0.318968 0.712152
C 7.120121 -0.172022 1.001853
C 7.972190 -1.143236 0.161049
C 7.957096 -0.888713 -1.340445
C 7.691583 1.248127 1.015256
H 1.646072 -2.758921 -0.112954
H 2.557822 2.042906 -0.025585
H 0.146333 2.558359 -0.131006
H -2.200656 3.068736 -0.504722
H -1.853760 -2.730204 -0.374966
H -4.631902 -0.611181 1.984687
H -5.502429 -0.112648 -1.388811
H -6.329168 0.602520 0.013951
H -7.142700 -3.219222 -0.309469
H -7.785766 -1.713009 -1.007240
H -6.303509 -2.463737 -1.681006
H -3.971503 4.336929 0.551711
H -4.119420 4.180217 -1.240672
H -5.545634 4.495722 -0.242387
H 5.112331 2.549500 -0.204794
H 6.139814 1.542575 -1.257069
H 4.469632 1.775623 -1.667374
H 5.225899 -1.200517 1.210774
H 7.224477 -0.512200 2.043741
H 9.011236 -1.102777 0.511555
H 7.629468 -2.170743 0.339893
H 8.349307 0.102111 -1.585544
H 8.586792 -1.627038 -1.847964
H 6.948257 -0.976757 -1.753910
H 8.674680 1.250054 1.500380
H 7.047222 1.927379 1.583723
H 7.835323 1.664284 0.016266
SCF Energy (B3LYP/6-31G**)= -1361.70382227
Number of imaginary frequencies = 0

1b_c360
MMFF Geometry
C 2.107176 -1.560549 -0.025293
C 2.287652 1.148399 0.026964
N 3.340887 -1.003173 0.034562
C 0.909376 -0.843074 -0.063850
C 1.014653 0.558720 -0.038811
C 3.427809 0.346682 0.063298
C -0.155780 1.338480 -0.088078
C -1.424678 0.744056 -0.145979
C -1.523069 -0.648932 -0.154789
C -0.365277 -1.441620 -0.130172
C -2.870306 -1.259575 -0.215413
C -4.080623 -0.399146 0.212591
C -3.854262 1.076231 -0.104699
C -2.617653 1.586714 -0.254730
O -0.427162 -2.814534 -0.172756
O -2.950053 -2.442927 -0.556783
O -4.127821 -0.537319 1.639968
C -5.332664 -1.003268 -0.456567
C -6.636664 -0.485261 0.112341
C -7.791746 -0.349926 -0.842575
O -6.763936 -0.239105 1.313624
O -5.021599 1.812633 -0.191974
C -4.932371 3.230792 -0.272000
C 4.801429 0.922251 0.148281
C 4.933227 2.285460 0.772402
C 5.853618 0.199138 -0.295446
C 7.311478 0.601067 -0.320187
C 8.101980 -0.036582 0.838671
C 8.169432 -1.559000 0.829565
C 7.922680 0.275657 -1.686913
H 2.107585 -2.648333 -0.041541
H 2.377875 2.229518 0.035777
H -0.071042 2.424496 -0.080221
H -2.447542 2.637317 -0.460748
H -1.365686 -3.078571 -0.308291
H -5.042369 -0.312984 1.917491
H -5.374680 -2.090454 -0.314375
H -5.307293 -0.814114 -1.536621
H -8.664182 0.041896 -0.312464
H -7.526275 0.345780 -1.642450
H -8.038233 -1.329428 -1.259313
H -4.460746 3.538780 -1.210463
H -5.950058 3.632036 -0.259753
H -4.397265 3.640155 0.590929
H 4.296089 2.373240 1.659959
H 5.948864 2.498093 1.117006
H 4.650207 3.065398 0.057948
H 5.659077 -0.795584 -0.701611
H 7.397599 1.687814 -0.206934
H 9.127167 0.354554 0.825278
H 7.663682 0.285501 1.792081
H 8.670410 -1.933752 -0.067554

H 8.739855 -1.909906 1.695996
H 7.173521 -2.008011 0.886113
H 8.983367 0.547547 -1.711626
H 7.416889 0.838702 -2.479717
H 7.839039 -0.787761 -1.935124
SCF Energy (B3LYP/6-31G**)= -1361.71091903
Number of imaginary frequencies = 0

1b_c361
MMFF Geometry
C -1.703727 -1.444803 -0.696230
C -2.152625 1.114707 0.096646
N -2.982925 -1.062601 -0.463893
C -0.587415 -0.616602 -0.559472
C -0.831547 0.707993 -0.149352
C -3.202520 0.212047 -0.067383
C 0.249026 1.598670 -0.006767
C 1.563700 1.181380 -0.244792
C 1.799527 -0.141846 -0.634133
C 0.734793 -1.033323 -0.813147
C 3.201370 -0.554384 -0.873847
C 4.316019 0.248233 -0.153178
C 3.941116 1.726629 -0.146407
C 2.666127 2.141865 -0.182525
O 0.935565 -2.322467 -1.242238
O 3.419336 -1.528240 -1.595615
O 5.552755 0.084365 -0.852513
C 4.519374 -0.279301 1.271944
C 4.450871 -1.788350 1.343514
C 5.656411 -2.574210 0.901247
O 3.420594 -2.344683 1.726185
O 4.941735 2.680666 -0.268158
C 5.840278 2.715288 0.837303
C -4.621508 0.590606 0.197325
C -4.846588 1.650507 1.239086
C -5.606101 -0.049849 -0.471727
C -7.109078 0.127330 -0.427312
C -7.770830 -0.458942 0.833560
C -7.497125 -1.945748 1.024788
C -7.560164 1.570019 -0.676648
H -1.595433 -2.481433 -1.008303
H -2.353273 2.136987 0.399902
H 0.056892 2.629798 0.287516
H 2.432069 3.202218 -0.219036
H 1.886053 -2.439026 -1.465970
H 5.429917 0.481872 -1.734237
H 3.747555 0.104999 1.951656
H 5.486162 0.022518 1.690021
H 5.549903 -3.614436 1.221797
H 5.744910 -2.544503 -0.186426
H 6.556714 -2.163072 1.364618
H 5.303232 2.685122 1.791457
H 6.564980 1.899050 0.775110
H 6.394163 3.657543 0.789275
H -4.174861 1.509903 2.093827
H -4.674828 2.647069 0.819277
H -5.855803 1.630164 1.653455
H -5.307727 -0.825752 -1.181608
H -7.496712 -0.446750 -1.282117
H -7.447687 0.078640 1.731378
H -8.856882 -0.318076 0.763398
H -6.435412 -2.139449 1.205313
H -8.054747 -2.321336 1.888826
H -7.809881 -2.518596 0.146028
H -8.636699 1.598951 -0.880029
H -7.049402 1.999042 -1.545743
H -7.378918 2.222157 0.181641
SCF Energy (B3LYP/6-31G**)= -1361.68808474
Number of imaginary frequencies = 0

1b_c362
MMFF Geometry
C -1.676651 -1.553004 -0.633118
C -2.149641 1.018853 0.106941
N -2.961733 -1.174789 -0.429345
C -0.566632 -0.715433 -0.492113
C -0.823450 0.614785 -0.110480
C -3.189113 0.105234 -0.058018
C 0.251105 1.512032 0.035775
C 1.574065 1.100879 -0.175943
C 1.820416 -0.226459 -0.537806
C 0.762306 -1.128120 -0.714648
C 3.220002 -0.634374 -0.764571
C 4.345691 0.212725 -0.140285
C 3.956426 1.684222 -0.061036
C 2.667036 2.073614 -0.069544
O 0.975030 -2.426252 -1.110622
O 3.462945 -1.638533 -1.440091
O 5.489087 0.084199 -1.005225
C 4.745948 -0.278582 1.252937
C 4.757395 -1.784677 1.354721
C 6.011067 -2.509003 0.943826
O 3.751336 -2.391996 1.724007
O 5.040704 2.533665 0.067858
C 4.814468 3.937379 0.064477
C -4.608876 0.487992 0.174343
C -4.861768 1.417202 1.326342
C -5.562141 -0.049934 -0.617614
C -7.061140 0.158667 -0.690482
C -7.838489 -1.014294 -0.061088
C -7.654998 -1.184370 1.441211
C -7.598825 1.522272 -0.248847
H -1.557176 -2.594228 -0.925254
H -2.367751 2.044339 0.387920
H 0.046278 2.544594 0.316427
H 2.380055 3.116106 0.012901
H 1.931122 -2.549662 -1.302188
H 5.302321 -0.655728 -1.617109
H 4.042848 0.078271 2.017416
H 5.738081 0.090535 1.540554
H 5.935740 -3.560976 1.233314
H 6.140460 -2.446755 -0.138395
H 6.873893 -2.072881 1.453271
H 4.328972 4.254390 -0.864162
H 4.227458 4.239721 0.937523
H 5.786759 4.435300 0.124045
H -4.875505 2.458355 0.989252
H -5.802997 1.190557 1.831586
H -4.093175 1.317573 2.101637
H -5.224214 -0.757117 -1.381170
H -7.285395 0.118723 -1.767630
H -8.908250 -0.887465 -0.269973
H -7.540207 -1.949432 -0.552778
H -7.996103 -0.303212 1.991525
H -8.242273 -2.039529 1.791961
H -6.609492 -1.374577 1.700442
H -8.628582 1.649621 -0.602692
H -7.005137 2.337652 -0.675966
H -7.623635 1.645056 0.835654
SCF Energy (B3LYP/6-31G**)= -1361.69602448
Number of imaginary frequencies = 0

1b_c363
MMFF Geometry
C 1.845375 1.978261 -0.018013
C 2.221771 -0.675775 -0.455174
N 3.116281 1.510813 -0.056584
C 0.702087 1.193535 -0.186216
C 0.908932 -0.179365 -0.412106
C 3.301055 0.188147 -0.274271
C -0.201855 -1.023561 -0.594857
C -1.509558 -0.523117 -0.543871
C -1.709227 0.839894 -0.305413
C -0.613235 1.699322 -0.142576
C -3.097803 1.350531 -0.237417
C -4.257298 0.354223 -0.033364
C -3.902271 -1.004029 -0.599999
C -2.640014 -1.415943 -0.789548
O -0.775852 3.047958 0.066280
O -3.297565 2.568144 -0.298481
O -5.371875 0.904187 -0.753050
C -4.578791 0.351453 1.464519

C -5.548756 -0.725553 1.897711
C -6.986744 -0.601673 1.464951
O -5.175391 -1.652446 2.619576
O -4.956291 -1.878059 -0.815144
C -5.312331 -1.935675 -2.196792
C 4.715656 -0.286170 -0.326259
C 4.984847 -1.492550 -1.182040
C 5.661893 0.396207 0.357116
C 7.148974 0.146109 0.508592
C 7.482188 -1.261029 1.040443
C 8.931035 -1.402548 1.490952
C 7.914454 0.519793 -0.762876
H 1.768167 3.048683 0.160132
H 2.390040 -1.735709 -0.615719
H -0.040080 -2.085211 -0.780258
H -2.439383 -2.426902 -1.132759
H -1.724712 3.276639 -0.053451
H -5.296764 1.877343 -0.681339
H -5.004443 1.314016 1.776050
H -3.661963 0.215022 2.053760
H -7.593480 -1.331738 2.008513
H -7.357147 0.398518 1.703511
H -7.084679 -0.793844 0.395671
H -5.567386 -0.945813 -2.588186
H -4.505182 -2.379406 -2.789115
H -6.193957 -2.576581 -2.289411
H 4.427620 -1.437137 -2.124361
H 6.033861 -1.586182 -1.466898
H 4.696595 -2.409705 -0.657923
H 5.338034 1.275346 0.920224
H 7.480316 0.853666 1.283115
H 6.834144 -1.488830 1.896391
H 7.285897 -2.027592 0.284580
H 9.185637 -0.648377 2.242176
H 9.089947 -2.389934 1.936511
H 9.623651 -1.303773 0.650082
H 8.989031 0.590414 -0.565342
H 7.592424 1.495597 -1.144007
H 7.773348 -0.215398 -1.560261
SCF Energy (B3LYP/6-31G**)= -1361.69616690
Number of imaginary frequencies = 0

1b_c364
MMFF Geometry
C -1.758997 -1.327506 -0.725870
C -2.165072 1.209894 0.155460
N -3.028915 -0.937936 -0.460259
C -0.630446 -0.518997 -0.574565
C -0.851969 0.792680 -0.115645
C -3.229366 0.327345 -0.024354
C 0.244140 1.658427 0.056505
C 1.551557 1.230185 -0.204554
C 1.765110 -0.074151 -0.656316
C 0.683959 -0.943388 -0.854537
C 3.157093 -0.513870 -0.905205
C 4.304219 0.250842 -0.197118
C 3.940363 1.730880 -0.075918
C 2.669647 2.160400 -0.076866
O 0.866068 -2.226801 -1.310231
O 3.334279 -1.526314 -1.585416
O 5.489821 0.134206 -0.981578
C 4.511937 -0.378338 1.194227
C 4.823538 -1.859289 1.108062
C 4.086350 -2.773242 2.048944
O 5.667401 -2.288560 0.321458
O 4.931000 2.705637 -0.113084
C 5.983176 2.555417 0.831236
C -4.643883 0.723900 0.236035
C -4.972942 2.187624 0.116586
C -5.554957 -0.229881 0.531266
C -7.025060 -0.058275 0.843141
C -7.911681 -0.714773 -0.231165
C -7.785650 -0.056376 -1.599372
C -7.322085 -0.673901 2.214800
H -1.669991 -2.352788 -1.078731
H -2.343977 2.217114 0.517293
H 0.071363 2.679488 0.395597
H 2.454612 3.224795 -0.033551
H 1.791342 -2.317513 -1.636938
H 5.671111 -0.826227 -1.075822
H 3.617890 -0.246844 1.815782
H 5.355352 0.070370 1.728307
H 4.405269 -3.806196 1.884875
H 4.309123 -2.492473 3.081141
H 3.012163 -2.702443 1.860406
H 5.585883 2.426389 1.843194
H 6.648766 1.730468 0.565303
H 6.576046 3.475003 0.819466
H -4.480108 2.629152 -0.757294
H -6.040303 2.376016 -0.022215
H -4.650854 2.728924 1.012229
H -5.219809 -1.268870 0.567025
H -7.293824 1.000436 0.910247
H -7.672759 -1.781807 -0.324333
H -8.961511 -0.654549 0.082176
H -6.779671 -0.175405 -2.012856
H -8.489591 -0.514123 -2.301828
H -8.012536 1.012936 -1.542102
H -8.373606 -0.527589 2.484854
H -6.710782 -0.206697 2.995130
H -7.119645 -1.750988 2.226594
SCF Energy (B3LYP/6-31G**)= -1361.70178744
Number of imaginary frequencies = 0

1b_c365
MMFF Geometry
C -1.726719 -1.529451 -0.438047
C -2.193327 0.992100 0.458456
N -2.994380 -1.196256 -0.095927
C -0.629169 -0.669577 -0.359684
C -0.882084 0.634054 0.105826
C -3.225077 0.060681 0.349048
C 0.180747 1.551646 0.200897
C 1.487545 1.182197 -0.140740
C 1.732930 -0.114993 -0.596878
C 0.683237 -1.035113 -0.720451
C 3.125319 -0.491826 -0.931844
C 4.278133 0.332110 -0.303986
C 3.853527 1.794654 -0.168862
C 2.566545 2.164543 -0.090968
O 0.895693 -2.312442 -1.180091
O 3.306158 -1.501419 -1.615332
O 5.415190 0.263578 -1.162498
C 4.604006 -0.273852 1.074949
C 4.978758 -1.739424 0.977968
C 4.345610 -2.678416 1.968639
O 5.790826 -2.135857 0.142200
O 4.792782 2.814115 -0.275619
C 5.909397 2.721570 0.599575
C -4.632102 0.384779 0.724778
C -4.832778 1.479797 1.737348
C -5.644246 -0.319066 0.170863
C -7.131573 -0.145133 0.383460
C -7.874818 0.032492 -0.955163
C -7.480165 1.304935 -1.694972
C -7.679572 -1.363248 1.130864
H -1.612750 -2.551991 -0.791682
H -2.399914 2.000359 0.802242
H -0.017890 2.566808 0.543525
H 2.304882 3.218147 -0.040764
H 1.801776 -2.363175 -1.564189
H 5.635246 -0.688163 -1.262151
H 3.746492 -0.178378 1.752070
H 5.457900 0.218503 1.550741
H 4.701739 -3.696825 1.790778
H 4.619157 -2.378634 2.983130
H 3.259590 -2.659235 1.847691
H 5.584493 2.583351 1.635856
H 6.594588 1.926138 0.296150
H 6.456368 3.667616 0.543974
H -4.733418 2.462976 1.265739
H -5.811006 1.437659 2.222829

H -4.099042 1.402892 2.548025
H -5.394115 -1.112620 -0.536805
H -7.342162 0.739688 0.992175
H -7.698939 -0.831246 -1.608836
H -8.954959 0.067371 -0.765264
H -6.429680 1.283957 -2.000462
H -8.087231 1.416542 -2.599122
H -7.640791 2.188163 -1.068570
H -8.751705 -1.248977 1.324214
H -7.179491 -1.487728 2.097947
H -7.538416 -2.285962 0.556510
SCF Energy (B3LYP/6-31G**)= -1361.70199140
Number of imaginary frequencies = 0

1b_c366
MMFF Geometry
C -1.719417 -2.005457 -0.473415
C -2.243241 0.483265 0.484740
N -3.004167 -1.702674 -0.166091
C -0.635574 -1.130573 -0.337407
C -0.919773 0.153969 0.159119
C -3.249642 -0.463159 0.307433
C 0.127388 1.078159 0.318602
C 1.450869 0.746352 -0.009883
C 1.734368 -0.533459 -0.498782
C 0.694968 -1.466276 -0.664838
C 3.133490 -0.885116 -0.855442
C 4.251361 0.172246 -0.680676
C 3.774463 1.503776 -0.136981
C 2.498095 1.746321 0.184284
O 0.923853 -2.731228 -1.155704
O 3.349882 -2.000109 -1.341010
O 4.780912 0.404645 -2.003617
C 5.394711 -0.436652 0.142094
C 5.022706 -0.700543 1.585002
C 6.164965 -0.846625 2.555475
O 3.856546 -0.840466 1.951886
O 4.725536 2.461377 0.195453
C 5.364365 3.037746 -0.942499
C -4.661268 -0.159126 0.632333
C -5.334774 -1.160447 1.525567
C -5.246813 0.917030 0.063912
C -6.647784 1.484399 0.187682
C -7.471045 1.233229 -1.092723
C -7.780723 -0.229386 -1.385982
C -7.438404 1.142504 1.452137
H -1.575785 -3.016391 -0.849088
H -2.489134 1.463538 0.881651
H -0.096439 2.070489 0.709233
H 2.226447 2.697254 0.634365
H 1.888590 -2.830399 -1.325346
H 4.030690 0.632240 -2.579925
H 6.265136 0.231054 0.123182
H 5.730340 -1.385449 -0.294758
H 5.774280 -1.031119 3.560027
H 6.792216 -1.690313 2.257639
H 6.752573 0.074615 2.571376
H 6.169597 2.389779 -1.299783
H 4.650022 3.250663 -1.745309
H 5.813984 3.984551 -0.628704
H -4.707641 -2.034917 1.731162
H -5.568766 -0.712374 2.496223
H -6.252882 -1.538794 1.066095
H -4.639896 1.505410 -0.628628
H -6.499057 2.573508 0.241114
H -8.417763 1.783785 -1.023934
H -6.936305 1.652895 -1.954773
H -8.372635 -0.684208 -0.586877
H -8.361181 -0.307184 -2.311283
H -6.867168 -0.816020 -1.519559
H -8.345399 1.755632 1.506252
H -6.850495 1.355220 2.351410
H -7.757727 0.098443 1.486300
SCF Energy (B3LYP/6-31G**)= -1361.69168675
Number of imaginary frequencies = 0

1b_c367
MMFF Geometry
C -1.551014 -2.364921 -0.382956
C -2.437306 0.160965 0.090327
N -2.876252 -2.186342 -0.165725
C -0.596919 -1.342621 -0.379683
C -1.066970 -0.039200 -0.131863
C -3.307604 -0.926877 0.059299
C -0.156232 1.033302 -0.117580
C 1.213003 0.824638 -0.331254
C 1.675368 -0.472255 -0.565706
C 0.778309 -1.549785 -0.607266
C 3.127972 -0.677628 -0.768559
C 4.102127 0.399635 -0.232518
C 3.461558 1.787575 -0.353925
C 2.128229 1.965985 -0.374583
O 1.202429 -2.831892 -0.861995
O 3.487996 -1.732451 -1.294546
O 5.306993 0.386656 -0.997608
C 4.418369 0.103993 1.242504
C 5.638130 -0.764393 1.485307
C 5.696601 -2.130320 0.855657
O 6.531390 -0.386918 2.248776
O 4.398924 2.801904 -0.437593
C 3.948190 4.151685 -0.473989
C -4.763254 -0.777490 0.295603
C -5.360089 -1.842822 1.175545
C -5.450451 0.234016 -0.277092
C -6.935012 0.512123 -0.177275
C -7.210344 1.856524 0.521351
C -6.766189 1.877969 1.978708
C -7.541536 0.518452 -1.584369
H -1.262747 -3.397969 -0.566321
H -2.821400 1.154652 0.299683
H -0.526825 2.042407 0.060541
H 1.675443 2.948954 -0.446709
H 2.139748 -2.799898 -1.163218
H 5.298730 1.170716 -1.575341
H 3.569251 -0.358769 1.760227
H 4.619283 1.045206 1.773571
H 6.485830 -2.716380 1.336171
H 4.747973 -2.650065 1.010382
H 5.925268 -2.050169 -0.208152
H 3.353303 4.339710 -1.373392
H 3.383215 4.399693 0.430276
H 4.829021 4.799395 -0.510535
H -4.705543 -2.052734 2.029684
H -6.323962 -1.552465 1.600537
H -5.503152 -2.771358 0.612862
H -4.916408 0.933344 -0.920907
H -7.447587 -0.275615 0.383452
H -6.714806 2.675234 -0.015744
H -8.286944 2.065747 0.488320
H -5.680392 1.778897 2.070613
H -7.053579 2.826239 2.444206
H -7.237120 1.067407 2.543894
H -8.624196 0.678804 -1.538117
H -7.368814 -0.438195 -2.090186
H -7.111305 1.311892 -2.206114
SCF Energy (B3LYP/6-31G**)= -1361.69988806
Number of imaginary frequencies = 0

1b_c368
MMFF Geometry
C -1.811271 -1.653879 -0.583982
C -2.151832 0.948534 0.115074
N -3.076200 -1.206630 -0.393158
C -0.658786 -0.873379 -0.451663
C -0.848109 0.472017 -0.092488
C -3.237351 0.088732 -0.040769
C 0.271897 1.310026 0.049393
C 1.574568 0.831175 -0.159424
C 1.762570 -0.510217 -0.509134
C 0.650189 -1.357546 -0.658328
C 3.139947 -1.018645 -0.738517
C 4.341521 -0.053360 -0.588095

C 3.963104 1.362182 -0.201936
C 2.699296 1.747902 0.010637
O 0.784840 -2.679094 -1.017124
O 3.276697 -2.190638 -1.103928
O 4.965834 0.000416 -1.888837
C 5.372545 -0.678748 0.361041
C 4.894715 -0.767421 1.794188
C 5.959908 -0.926370 2.846831
O 3.701526 -0.761439 2.094801
O 4.980309 2.259062 0.102965
C 5.735502 2.661711 -1.038442
C -4.635782 0.548610 0.181337
C -4.841832 1.508049 1.317874
C -5.614344 0.048647 -0.604651
C -7.100165 0.334273 -0.684963
C -7.939181 -0.785855 -0.038741
C -7.767745 -0.940711 1.466623
C -7.566441 1.731269 -0.267155
H -1.745125 -2.704468 -0.858715
H -2.315182 1.987709 0.382580
H 0.122100 2.351795 0.331632
H 2.494083 2.758762 0.352183
H 1.743862 -2.880822 -1.111424
H 4.275078 0.237017 -2.531998
H 6.301996 -0.096110 0.339183
H 5.641251 -1.691519 0.035762
H 5.496469 -0.976483 3.836028
H 6.516052 -1.849736 2.667786
H 6.634345 -0.066979 2.818578
H 6.494647 1.912576 -1.280116
H 5.092039 2.857740 -1.903111
H 6.254366 3.590822 -0.784282
H -4.798955 2.543017 0.964572
H -5.795239 1.340055 1.823164
H -4.081909 1.379631 2.097471
H -5.312238 -0.687287 -1.355881
H -7.324163 0.288462 -1.761934
H -9.000413 -0.606551 -0.252620
H -7.689433 -1.743268 -0.514346
H -8.063189 -0.033990 2.001605
H -8.399762 -1.758040 1.829587
H -6.734161 -1.181224 1.731803
H -8.587283 1.906700 -0.626227
H -6.929768 2.507272 -0.705570
H -7.587250 1.872902 0.815128
SCF Energy (B3LYP/6-31G**)= -1361.69200365
Number of imaginary frequencies = 0

1b_c369
MMFF Geometry
C -1.645145 -2.126037 -0.789224
C -2.401361 0.473925 -0.548931
N -2.970984 -1.850145 -0.752659
C -0.628504 -1.169230 -0.710211
C -1.031776 0.173849 -0.588759
C -3.336234 -0.556612 -0.624295
C -0.055939 1.185201 -0.519370
C 1.310884 0.877122 -0.548928
C 1.705392 -0.458498 -0.654646
C 0.746056 -1.477090 -0.752660
C 3.153720 -0.767813 -0.663076
C 4.129193 0.276010 -0.066659
C 3.608024 1.691808 -0.341245
C 2.303041 1.953097 -0.538182
O 1.106096 -2.796431 -0.887832
O 3.499560 -1.873364 -1.083872
O 5.414357 0.139314 -0.672365
C 4.241597 0.052111 1.450014
C 5.357736 -0.876064 1.890000
C 5.395173 -2.279006 1.345955
O 6.175822 -0.510403 2.738712
O 4.618056 2.637296 -0.360991
C 4.271847 4.008095 -0.526662
C -4.795902 -0.300399 -0.592386
C -5.590679 -1.119196 -1.573902
C -5.318493 0.580614 0.287362
C -6.778802 0.923752 0.489643
C -7.216729 0.686779 1.948069
C -7.148350 -0.777788 2.363345
C -7.008651 2.387042 0.103160
H -1.410692 -3.183905 -0.888479
H -2.736931 1.503728 -0.473981
H -0.373074 2.224871 -0.442750
H 1.933039 2.956533 -0.718928
H 2.073347 -2.842474 -1.068442
H 5.531536 0.884537 -1.288401
H 3.304975 -0.322514 1.880800
H 4.442912 1.009442 1.951286
H 5.757033 -2.279125 0.316654
H 6.076136 -2.883825 1.952358
H 4.400172 -2.726418 1.407330
H 3.805468 4.177014 -1.502433
H 3.620190 4.347544 0.284828
H 5.194248 4.594920 -0.487428
H -5.726025 -2.140404 -1.202269
H -6.578509 -0.698090 -1.776266
H -5.080313 -1.167958 -2.543025
H -4.648570 1.096020 0.976032
H -7.421040 0.310170 -0.149631
H -6.602182 1.282929 2.634500
H -8.251057 1.030778 2.073745
H -6.119879 -1.151215 2.355321
H -7.537708 -0.897943 3.379433
H -7.748757 -1.403047 1.694886
H -8.065440 2.654565 0.210611
H -6.725366 2.566285 -0.940135
H -6.424350 3.067275 0.733315
SCF Energy (B3LYP/6-31G**)= -1361.70004199
Number of imaginary frequencies = 0

1b_c370
MMFF Geometry
C -1.398734 -2.174262 -0.566737
C -2.269241 0.388164 -0.305339
N -2.733564 -1.963876 -0.463486
C -0.427949 -1.168046 -0.550201
C -0.890716 0.156034 -0.416530
C -3.153876 -0.688374 -0.324130
C 0.034046 1.216791 -0.410636
C 1.408721 0.974341 -0.512038
C 1.862487 -0.344673 -0.625308
C 0.955320 -1.410642 -0.666874
C 3.322819 -0.568709 -0.726065
C 4.255672 0.520099 -0.134233
C 3.667852 1.895680 -0.431579
C 2.351102 2.090783 -0.597139
O 1.372676 -2.709214 -0.827997
O 3.730206 -1.613852 -1.234578
O 5.547802 0.424791 -0.739841
C 4.436796 0.307912 1.373336
C 4.587150 -1.152536 1.736047
C 5.922929 -1.806681 1.503039
O 3.626563 -1.783177 2.179879
O 4.523335 2.955186 -0.699587
C 5.328739 3.338451 0.411748
C -4.621451 -0.502954 -0.218212
C -5.413190 -1.332352 -1.190689
C -5.135518 0.337190 0.706145
C -6.581944 0.647321 1.040123
C -7.197030 1.611847 0.010019
C -8.576062 2.121257 0.407372
C -7.432300 -0.599551 1.301239
H -1.116387 -3.220179 -0.667961
H -2.651641 1.400576 -0.219448
H -0.327843 2.241258 -0.331532
H 1.966585 3.075109 -0.849588
H 2.343049 -2.714106 -0.987547
H 5.428386 0.626842 -1.686124
H 3.572070 0.685856 1.934408
H 5.317570 0.831669 1.761243
H 5.949792 -2.771620 2.017176
H 6.079939 -1.968304 0.434873

H 6.718884 -1.178615 1.910817
H 4.737575 3.404181 1.331502
H 6.168099 2.649652 0.539582
H 5.740061 4.330105 0.201089
H -5.508976 -2.362129 -0.830849
H -6.415127 -0.935202 -1.363471
H -4.926967 -1.353524 -2.173188
H -4.440797 0.862436 1.363413
H -6.546816 1.183895 1.999881
H -6.534775 2.478273 -0.115633
H -7.271941 1.133223 -0.972827
H -8.552277 2.590855 1.395731
H -8.921904 2.868633 -0.314048
H -9.312333 1.312250 0.423936
H -8.336442 -0.336577 1.861215
H -6.884881 -1.334622 1.901644
H -7.756346 -1.085224 0.376939
SCF Energy (B3LYP/6-31G**)= -1361.68658787
Number of imaginary frequencies = 0

1b_c371
MMFF Geometry
C -1.586448 -1.870574 -0.552097
C -2.275295 0.532334 0.518416
N -2.891303 -1.660413 -0.253003
C -0.560304 -0.940937 -0.351752
C -0.929146 0.299783 0.203659
C -3.219214 -0.462920 0.275666
C 0.055483 1.280067 0.425095
C 1.399652 1.039197 0.112920
C 1.761629 -0.201082 -0.424621
C 0.790791 -1.180945 -0.673611
C 3.183791 -0.431149 -0.757456
C 4.256094 0.459826 -0.099095
C 3.705109 1.845918 0.176258
C 2.393386 2.095547 0.310472
O 1.111482 -2.392794 -1.235970
O 3.490276 -1.324858 -1.552491
O 5.336009 0.570414 -1.042650
C 4.806324 -0.136792 1.198392
C 4.979286 -1.634410 1.121589
C 6.285107 -2.165035 0.594078
O 4.059090 -2.386695 1.444358
O 4.634829 2.833501 0.465136
C 4.862706 3.673918 -0.666721
C -4.651824 -0.260517 0.587598
C -5.278018 -1.343743 1.417487
C -5.292328 0.805474 0.060820
C -6.727213 1.280758 0.184479
C -7.512223 1.044437 -1.122500
C -7.727159 -0.417777 -1.492464
C -7.516415 0.829634 1.415114
H -1.376395 -2.850651 -0.975427
H -2.587919 1.475454 0.956390
H -0.235890 2.242482 0.844573
H 2.049157 3.087015 0.590068
H 2.065842 -2.394113 -1.470645
H 5.239034 -0.175666 -1.668908
H 4.129248 0.052260 2.042192
H 5.773552 0.306387 1.465475
H 6.321280 -3.249229 0.733068
H 6.376281 -1.940641 -0.470423
H 7.115309 -1.718540 1.146891
H 5.208620 3.100537 -1.532341
H 3.957939 4.235206 -0.923375
H 5.643513 4.391994 -0.399544
H -5.555137 -0.959451 2.404005
H -6.163515 -1.753156 0.922050
H -4.602287 -2.188129 1.592602
H -4.711254 1.462767 -0.590711
H -6.646096 2.372840 0.293616
H -8.491784 1.532974 -1.045620
H -6.989839 1.537793 -1.952507
H -8.303381 -0.946505 -0.728131
H -8.286361 -0.484282 -2.431632
H -6.777416 -0.941046 -1.636485
H -8.459859 1.383751 1.481172
H -6.957572 1.032343 2.334995
H -7.772054 -0.232121 1.392271
SCF Energy (B3LYP/6-31G**)= -1361.69072284
Number of imaginary frequencies = 0

1b_c372
MMFF Geometry
C -1.690292 -1.471204 -0.738594
C -2.183408 1.020528 0.227630
N -2.979633 -1.116244 -0.520298
C -0.585313 -0.647795 -0.504879
C -0.852832 0.641228 -0.006563
C -3.217050 0.123653 -0.036012
C 0.215621 1.523717 0.237972
C 1.542182 1.136759 0.007603
C 1.801433 -0.152189 -0.471290
C 0.748485 -1.035903 -0.744390
C 3.207458 -0.537325 -0.718322
C 4.330777 0.263580 -0.029925
C 3.912219 1.707366 0.172548
C 2.628086 2.093321 0.227103
O 0.970229 -2.292692 -1.253231
O 3.460532 -1.485730 -1.467458
O 5.463126 0.230235 -0.916165
C 4.749740 -0.338593 1.313219
C 4.769754 -1.847826 1.292329
C 6.038110 -2.527975 0.852266
O 3.761335 -2.489825 1.588611
O 4.922716 2.605429 0.481656
C 5.294103 3.376934 -0.661245
C -4.641453 0.480071 0.209776
C -4.913974 1.300313 1.437599
C -5.581544 0.013866 -0.641216
C -7.080134 0.224604 -0.716225
C -7.861899 -1.002361 -0.206675
C -7.698817 -1.307284 1.276538
C -7.628961 1.541376 -0.160825
H -1.562952 -2.480975 -1.123137
H -2.408921 2.015304 0.598838
H 0.004554 2.525353 0.611143
H 2.374121 3.124031 0.456888
H 1.930302 -2.400028 -1.433777
H 5.322063 -0.524372 -1.523547
H 4.054182 -0.050815 2.112945
H 5.743027 0.012882 1.618372
H 5.954298 -3.604431 1.026393
H 6.205430 -2.352810 -0.212229
H 6.881456 -2.148970 1.434776
H 5.623381 2.740538 -1.488463
H 4.466429 4.017241 -0.984480
H 6.129864 4.021505 -0.373654
H -4.926226 2.367757 1.196288
H -5.861654 1.026926 1.906318
H -4.156374 1.132082 2.211862
H -5.230322 -0.620207 -1.460887
H -7.289166 0.281753 -1.795659
H -8.929093 -0.859698 -0.418462
H -7.553343 -1.888440 -0.776648
H -8.050807 -0.480369 1.899395
H -8.287810 -2.192075 1.540069
H -6.656351 -1.517693 1.532377
H -8.654051 1.697838 -0.516545
H -7.032237 2.393458 -0.503780
H -7.669618 1.565420 0.929863
SCF Energy (B3LYP/6-31G**)= -1361.69099060
Number of imaginary frequencies = 0

1b_c373
MMFF Geometry
C 1.788549 2.418462 0.081768
C 2.421763 -0.209877 -0.207172
N 3.098005 2.073362 0.120467
C 0.730299 1.521101 -0.096089
C 1.070816 0.163333 -0.247640
C 3.399163 0.764212 -0.015661

C 0.052890 -0.787587 -0.443537
C -1.295602 -0.409086 -0.474739
C -1.629963 0.938248 -0.307686
C -0.626275 1.902589 -0.135431
C -3.061037 1.319501 -0.325995
C -4.133404 0.228742 -0.128506
C -3.628939 -1.112562 -0.616679
C -2.326662 -1.412529 -0.730598
O -0.921632 3.237848 0.001921
O -3.367815 2.509556 -0.452019
O -5.257490 0.642281 -0.921041
C -4.525309 0.257624 1.352234
C -5.411602 -0.887161 1.790821
C -6.832429 -0.917082 1.290028
O -4.989564 -1.744181 2.569944
O -4.587285 -2.088858 -0.839341
C -4.867669 -2.236456 -2.231694
C 4.841654 0.428735 0.031573
C 5.703107 1.304825 -0.831880
C 5.268271 -0.586245 0.813577
C 6.660897 -1.104062 1.118129
C 6.959073 -2.417807 0.368496
C 7.029111 -2.298092 -1.148356
C 7.816372 -0.104401 1.025940
H 1.603333 3.483714 0.202782
H 2.711980 -1.248338 -0.335538
H 0.320292 -1.836087 -0.573156
H -2.018115 -2.413517 -1.018589
H -1.880622 3.371239 -0.169939
H -5.274679 1.620339 -0.890886
H -5.052418 1.187869 1.600340
H -3.629382 0.232623 1.987124
H -7.394359 -1.679066 1.837931
H -7.304848 0.052319 1.467711
H -6.860726 -1.159994 0.226983
H -5.191314 -1.291474 -2.679298
H -3.995364 -2.627943 -2.765596
H -5.682146 -2.959349 -2.334923
H 6.128554 2.126652 -0.247558
H 6.509740 0.736933 -1.301438
H 5.134188 1.739638 -1.662332
H 4.512643 -1.130240 1.384870
H 6.621844 -1.372454 2.185092
H 7.909892 -2.828717 0.730687
H 6.190022 -3.159523 0.620757
H 7.824018 -1.616287 -1.462875
H 7.242396 -3.278060 -1.588100
H 6.082287 -1.947455 -1.569167
H 8.707798 -0.518005 1.511800
H 7.570464 0.830717 1.540494
H 8.100328 0.131222 -0.001574
SCF Energy (B3LYP/6-31G**)= -1361.69316790
Number of imaginary frequencies = 0

1b_c374
MMFF Geometry
C -1.457875 -2.018757 -0.478969
C -2.226775 0.501060 0.192498
N -2.770479 -1.802389 -0.220228
C -0.461580 -1.039088 -0.427128
C -0.871204 0.262487 -0.076078
C -3.142439 -0.545820 0.104589
C 0.081885 1.295856 -0.011095
C 1.433994 1.045926 -0.272087
C 1.836047 -0.252569 -0.604939
C 0.897625 -1.287173 -0.704327
C 3.274066 -0.486009 -0.870360
C 4.288404 0.494464 -0.225586
C 3.717806 1.907923 -0.277845
C 2.397960 2.146889 -0.292300
O 1.260334 -2.559019 -1.074789
O 3.602694 -1.458190 -1.551334
O 5.517684 0.460958 -0.955770
C 4.597287 0.073492 1.215947
C 4.735703 -1.425405 1.361782
C 6.025094 -2.064335 0.919342
O 3.800261 -2.094409 1.803170
O 4.577220 2.979162 -0.478050
C 5.489843 3.191755 0.595531
C -4.586402 -0.353488 0.386163
C -5.188858 -1.439284 1.234181
C -5.240927 0.710397 -0.128159
C -6.701872 1.102056 -0.020075
C -7.657043 0.026615 -0.571510
C -9.088138 0.523165 -0.735258
C -7.059464 1.580784 1.389065
H -1.215930 -3.047216 -0.739059
H -2.564492 1.491802 0.480585
H -0.240599 2.305732 0.239710
H 2.022041 3.163156 -0.371987
H 2.211323 -2.558475 -1.325220
H 5.320129 0.793360 -1.850764
H 3.797958 0.385531 1.900854
H 5.524532 0.523417 1.587891
H 6.068994 -3.091210 1.293196
H 6.080948 -2.080002 -0.170790
H 6.872790 -1.512346 1.332880
H 4.985484 3.140594 1.566484
H 6.316256 2.477378 0.550591
H 5.910208 4.195635 0.483927
H -4.538672 -1.671181 2.085872
H -6.155136 -1.162866 1.657914
H -5.330338 -2.353606 0.648018
H -4.677295 1.400993 -0.757519
H -6.812566 1.979241 -0.674753
H -7.297118 -0.309590 -1.552349
H -7.674489 -0.856166 0.075256
H -9.125254 1.417649 -1.364898
H -9.700845 -0.249908 -1.210272
H -9.543505 0.758595 0.231088
H -8.016145 2.113445 1.388288
H -6.304264 2.276365 1.772393
H -7.145016 0.752731 2.098153
SCF Energy (B3LYP/6-31G**)= -1361.68688670
Number of imaginary frequencies = 0

1b_c375
MMFF Geometry
C 1.897501 2.182709 0.080259
C 2.316568 -0.387931 -0.700891
N 3.177318 1.753666 -0.032163
C 0.767412 1.399238 -0.174724
C 0.996628 0.070034 -0.577520
C 3.373873 0.475673 -0.425318
C -0.099849 -0.767992 -0.851836
C -1.417119 -0.307390 -0.719678
C -1.637445 1.008913 -0.308263
C -0.556925 1.865324 -0.050983
C -3.032391 1.472755 -0.156512
C -4.159890 0.428676 -0.049833
C -3.808145 -0.855643 -0.785231
C -2.534650 -1.187953 -1.067166
O -0.744969 3.172154 0.330740
O -3.259979 2.683453 -0.065137
O -5.308737 1.038195 -0.661971
C -4.441522 0.231862 1.444007
C -5.369339 -0.923298 1.748851
C -6.836014 -0.749777 1.450627
O -4.934289 -1.965719 2.243231
O -4.911786 -1.628975 -1.097106
C -4.712858 -2.908159 -1.685048
C 4.785296 0.034887 -0.544098
C 5.153901 -0.687350 -1.806316
C 5.615001 0.294241 0.489480
C 7.082763 -0.002662 0.723835
C 7.257353 -1.078969 1.816846
C 6.738797 -2.463271 1.446189
C 7.955604 -0.295699 -0.497141
H 1.799545 3.219234 0.395959
H 2.518349 -1.413415 -0.994911
H 0.082011 -1.792949 -1.174126
H -2.281510 -2.112849 -1.573773

H -1.699438 3.393818 0.245837
H -5.231937 2.001226 -0.509857
H -4.885487 1.134024 1.883893
H -3.508337 0.049339 1.993581
H -7.397395 -1.575893 1.896569
H -7.192778 0.184889 1.890562
H -7.009838 -0.748993 0.373507
H -4.243007 -2.816672 -2.669429
H -4.123767 -3.554426 -1.026420
H -5.694335 -3.371541 -1.822044
H 4.320479 -0.753230 -2.514127
H 5.951259 -0.160932 -2.338563
H 5.472233 -1.711390 -1.587164
H 5.177280 0.821645 1.342984
H 7.492266 0.927352 1.145654
H 8.321126 -1.161293 2.073263
H 6.747845 -0.751989 2.732664
H 7.265932 -2.872067 0.579616
H 6.893965 -3.153445 2.282080
H 5.667500 -2.447285 1.225450
H 9.008642 -0.366549 -0.201308
H 7.882818 0.512655 -1.232356
H 7.696143 -1.237081 -0.987671
SCF Energy (B3LYP/6-31G**)= -1361.69786879
Number of imaginary frequencies = 0

1b_c376
MMFF Geometry
C 2.090635 -1.590839 -0.530483
C 2.309646 1.108110 -0.327548
N 3.332340 -1.048186 -0.533902
C 0.902875 -0.862776 -0.430408
C 1.027947 0.533689 -0.326056
C 3.438857 0.296745 -0.431481
C -0.132435 1.323862 -0.228352
C -1.409471 0.744228 -0.220438
C -1.526301 -0.644398 -0.311013
C -0.380613 -1.445916 -0.431138
C -2.881777 -1.239528 -0.301048
C -4.042657 -0.399681 0.278075
C -3.823247 1.091627 0.040437
C -2.596266 1.601332 -0.176366
O -0.463410 -2.812771 -0.555215
O -3.003887 -2.397553 -0.710079
O -3.977564 -0.631424 1.692549
C -5.351909 -0.948526 -0.326075
C -6.599489 -0.459224 0.378583
C -7.825225 -0.252029 -0.469380
O -6.627290 -0.292137 1.599717
O -4.984022 1.841285 0.097140
C -4.882759 3.260872 0.105125
C 4.822393 0.857122 -0.452655
C 4.973429 2.269093 -0.950942
C 5.853959 0.081067 -0.051185
C 7.323683 0.430628 0.024467
C 7.839386 0.432910 1.478683
C 7.819017 -0.912102 2.194765
C 8.128661 -0.508934 -0.876648
H 2.075737 -2.675444 -0.613867
H 2.414772 2.183794 -0.233530
H -0.033269 2.406355 -0.157262
H -2.429709 2.661941 -0.326406
H -1.413093 -3.060269 -0.631130
H -4.863971 -0.418654 2.056895
H -5.396609 -2.042377 -0.252267
H -5.410391 -0.688572 -1.390004
H -8.647401 0.110576 0.153700
H -7.615384 0.493031 -1.240829
H -8.116917 -1.199855 -0.928067
H -4.274251 3.606693 0.946964
H -4.484516 3.628024 -0.845979
H -5.890716 3.668269 0.227127
H 4.681785 2.984101 -0.174547
H 5.993279 2.510165 -1.258871
H 4.350612 2.439320 -1.836789
H 5.627952 -0.937240 0.271568
H 7.492525 1.443945 -0.352510
H 8.871166 0.807089 1.483979
H 7.251088 1.149190 2.066726
H 8.448253 -1.648200 1.686421
H 8.206573 -0.795245 3.212370
H 6.805095 -1.314252 2.273663
H 9.199625 -0.290485 -0.807100
H 7.832119 -0.385999 -1.924652
H 7.978618 -1.561789 -0.614698
SCF Energy (B3LYP/6-31G**)= -1361.71069557
Number of imaginary frequencies = 0

1b_c377
MMFF Geometry
C -1.707335 -2.405147 -0.043735
C -2.514761 0.189559 0.042763
N -3.022746 -2.155133 0.162379
C -0.725509 -1.424227 -0.213448
C -1.154959 -0.083590 -0.164626
C -3.414959 -0.863342 0.194063
C -0.216170 0.951325 -0.330934
C 1.140911 0.669206 -0.526621
C 1.563110 -0.663268 -0.564770
C 0.638546 -1.706940 -0.426324
C 3.008457 -0.936618 -0.750971
C 4.008863 0.181649 -0.362559
C 3.412258 1.540011 -0.712385
C 2.089875 1.751592 -0.777933
O 1.025033 -3.024047 -0.485483
O 3.346179 -2.060972 -1.125494
O 5.198481 -0.021444 -1.124568
C 4.276607 0.009500 1.145284
C 5.738369 -0.006525 1.526600
C 6.495339 -1.298700 1.355527
O 6.269927 0.984973 2.030692
O 4.248652 2.555699 -1.154172
C 5.226426 2.966081 -0.200460
C -4.861417 -0.635351 0.424392
C -5.471420 -1.533063 1.467121
C -5.530584 0.295810 -0.288714
C -7.004605 0.631366 -0.212734
C -7.229111 2.075511 0.272399
C -6.757590 2.308743 1.702397
C -7.636573 0.438351 -1.595188
H -1.451355 -3.462346 -0.068441
H -2.867102 1.215009 0.099589
H -0.553019 1.987461 -0.311299
H 1.702808 2.722469 -1.075583
H 1.956987 -3.066152 -0.800670
H 5.013862 0.282314 -2.032140
H 3.846682 -0.923989 1.533366
H 3.783665 0.798394 1.728587
H 6.247205 -1.772535 0.403866
H 7.569824 -1.096287 1.369008
H 6.241919 -1.973864 2.176551
H 4.803708 3.037595 0.806867
H 6.086508 2.293323 -0.224874
H 5.576792 3.961253 -0.490351
H -4.807652 -1.627623 2.334597
H -6.419164 -1.151654 1.854780
H -5.650076 -2.532861 1.057552
H -4.989018 0.870864 -1.040327
H -7.528679 -0.043982 0.470590
H -6.720839 2.785644 -0.392196
H -8.299844 2.309931 0.222304
H -5.673478 2.191961 1.793396
H -7.009610 3.325984 2.018947
H -7.240490 1.610232 2.393129
H -8.713255 0.637043 -1.559392
H -7.500197 -0.589915 -1.948640
H -7.195737 1.112393 -2.338520
SCF Energy (B3LYP/6-31G**)= -1361.69210146
Number of imaginary frequencies = 0

1b_c378
MMFF Geometry

C -1.581001 -1.753964 -1.034290
C -2.230215 0.809868 -0.401518
N -2.894812 -1.431385 -0.958393
C -0.526176 -0.863231 -0.811545
C -0.874148 0.461200 -0.483591
C -3.205343 -0.153995 -0.646334
C 0.142120 1.406863 -0.252445
C 1.495279 1.050509 -0.329121
C 1.832561 -0.270217 -0.640026
C 0.834313 -1.219754 -0.899273
C 3.265947 -0.624132 -0.716554
C 4.272265 0.276608 0.037821
C 3.825509 1.736885 0.004372
C 2.531029 2.073509 -0.150136
O 1.138957 -2.513135 -1.248462
O 3.590409 -1.628889 -1.351707
O 5.552187 0.168903 -0.599339
C 4.450082 -0.172057 1.490090
C 4.517485 -1.675753 1.622167
C 5.814905 -2.360185 1.284098
O 3.520540 -2.313612 1.965761
O 4.865946 2.632102 0.170343
C 4.574951 4.024770 0.189655
C -4.650818 0.170831 -0.572154
C -5.095116 1.403564 -1.302324
C -5.442500 -0.645721 0.156094
C -6.927613 -0.630189 0.458186
C -7.182801 -0.280451 1.940107
C -6.791473 1.135945 2.344165
C -7.836729 0.172985 -0.472923
H -1.388879 -2.794233 -1.288943
H -2.524204 1.820246 -0.134833
H -0.132623 2.433505 -0.012952
H 2.197440 3.105473 -0.154480
H 2.114278 -2.593232 -1.351854
H 5.489068 0.643487 -1.446430
H 3.610521 0.161109 2.114594
H 5.360719 0.247718 1.935010
H 5.794396 -3.385645 1.663833
H 5.954876 -2.383842 0.201671
H 6.646375 -1.835264 1.760980
H 4.142083 4.346598 -0.762915
H 3.914088 4.274201 1.025964
H 5.516585 4.563282 0.331437
H -4.278021 1.889903 -1.846175
H -5.847869 1.159841 -2.057428
H -5.501164 2.141812 -0.603658
H -4.950209 -1.497783 0.635851
H -7.249376 -1.674887 0.333205
H -8.246644 -0.426988 2.165726
H -6.636055 -0.986402 2.578810
H -7.362355 1.885397 1.788873
H -6.996575 1.287809 3.409060
H -5.725517 1.321697 2.183249
H -8.888458 -0.002802 -0.219173
H -7.701833 -0.137898 -1.514285
H -7.666362 1.250327 -0.406269
SCF Energy (B3LYP/6-31G**)= -1361.68919511
Number of imaginary frequencies = 0

1b_c379
MMFF Geometry
C -1.642872 -2.364904 -0.162758
C -2.508045 0.202713 0.059784
N -2.964989 -2.155235 0.045369
C -0.681618 -1.355052 -0.269613
C -1.140989 -0.029221 -0.150504
C -3.385963 -0.876067 0.144821
C -0.223738 1.032902 -0.250484
C 1.141127 0.792041 -0.450288
C 1.593856 -0.526359 -0.559886
C 0.690224 -1.595580 -0.485867
C 3.046240 -0.761311 -0.749518
C 4.027711 0.361106 -0.328832
C 3.390724 1.725038 -0.573842
C 2.064558 1.910219 -0.625560
O 1.104121 -2.899731 -0.612416
O 3.409005 -1.867073 -1.155343
O 5.206756 0.264941 -1.124987
C 4.342069 0.136550 1.161945
C 5.714906 -0.440439 1.428629
C 5.980105 -1.863189 1.009028
O 6.573899 0.228593 2.007386
O 4.208853 2.788685 -0.934024
C 5.158189 3.159715 0.065181
C -4.838786 -0.692553 0.374689
C -5.436767 -1.659878 1.360601
C -5.522954 0.262090 -0.291598
C -7.004631 0.560720 -0.210238
C -7.264928 1.970291 0.352486
C -6.809873 2.133479 1.797448
C -7.621468 0.431487 -1.606890
H -1.363531 -3.413369 -0.243514
H -2.883353 1.215574 0.170096
H -0.583892 2.058647 -0.175091
H 1.655553 2.891426 -0.851357
H 2.040733 -2.906778 -0.916098
H 5.004301 0.655073 -1.994637
H 3.608045 -0.518060 1.649388
H 4.282959 1.077448 1.723313
H 6.906772 -2.211700 1.474183
H 5.163710 -2.505369 1.349172
H 6.085182 -1.929626 -0.074993
H 4.666981 3.331857 1.028361
H 5.949036 2.411388 0.159972
H 5.623066 4.098737 -0.249526
H -4.777652 -1.788281 2.227276
H -6.395560 -1.321131 1.760523
H -5.590296 -2.638931 0.894613
H -4.988614 0.889636 -1.005425
H -7.518818 -0.162990 0.429714
H -6.767382 2.727315 -0.266932
H -8.340180 2.183815 0.305859
H -5.724181 2.035362 1.891593
H -7.086862 3.125740 2.167843
H -7.282539 1.387163 2.443838
H -8.702551 0.604355 -1.569716
H -7.459515 -0.572217 -2.015800
H -7.190028 1.155440 -2.307577
SCF Energy (B3LYP/6-31G**)= -1361.69403513
Number of imaginary frequencies = 0

1b_c380
MMFF Geometry
C -1.706027 -1.477631 -0.708148
C -2.202350 1.031762 0.209180
N -2.995958 -1.119668 -0.498385
C -0.601966 -0.648968 -0.488875
C -0.871186 0.648969 -0.015908
C -3.234982 0.129203 -0.038479
C 0.196493 1.536291 0.213488
C 1.523895 1.146234 -0.007974
C 1.785067 -0.150381 -0.462144
C 0.732665 -1.040147 -0.719053
C 3.190864 -0.537920 -0.703789
C 4.320130 0.285936 -0.050852
C 3.894248 1.729844 0.148242
C 2.607940 2.105546 0.203087
O 0.955566 -2.306665 -1.203020
O 3.441958 -1.506981 -1.427529
O 5.424010 0.238753 -0.971958
C 4.780683 -0.295855 1.288141
C 4.790804 -1.805412 1.298281
C 6.043233 -2.502453 0.839427
O 3.787423 -2.434612 1.635897
O 4.870071 2.655653 0.491431
C 5.590321 3.128855 -0.646625
C -4.660002 0.489173 0.198528
C -4.934659 1.333544 1.409409
C -5.598720 0.005301 -0.644084
C -7.097380 0.213292 -0.725051
C -7.878804 -1.003837 -0.191934

C -7.717260 -1.278848 1.297282
C -7.647894 1.540501 -0.196825
H -1.577368 -2.494473 -1.073081
H -2.428912 2.033386 0.560834
H -0.015549 2.544423 0.568296
H 2.351626 3.135691 0.434381
H 1.915987 -2.416721 -1.379927
H 5.279380 -0.542250 -1.544906
H 4.114468 0.011631 2.105401
H 5.785626 0.055335 1.552868
H 5.952019 -3.576476 1.024475
H 6.191380 -2.338386 -0.229666
H 6.901517 -2.126678 1.401861
H 6.447609 2.481672 -0.850221
H 4.954333 3.216638 -1.534694
H 5.976076 4.124202 -0.406245
H -4.947202 2.395964 1.146872
H -5.882812 1.068988 1.882216
H -4.178024 1.181203 2.187893
H -5.246003 -0.644628 -1.450581
H -7.305171 0.248622 -1.805659
H -8.945854 -0.866297 -0.407796
H -7.568882 -1.900914 -0.743671
H -8.070631 -0.439898 1.903028
H -8.305885 -2.158647 1.577796
H -6.674938 -1.483260 1.558516
H -8.672650 1.689010 -0.556890
H -7.051375 2.386005 -0.556031
H -7.689958 1.586356 0.893111
SCF Energy (B3LYP/6-31G**)= -1361.69026449
Number of imaginary frequencies = 0

1b_c381
MMFF Geometry
C -1.378770 -2.268168 -0.512563
C -2.331352 0.200163 0.113020
N -2.705173 -2.141666 -0.267583
C -0.454604 -1.220166 -0.462619
C -0.958777 0.053178 -0.134680
C -3.170231 -0.909499 0.031233
C -0.079432 1.149483 -0.066820
C 1.291357 0.991252 -0.304334
C 1.786801 -0.274590 -0.626160
C 0.922521 -1.374045 -0.718834
C 3.242797 -0.424029 -0.851514
C 4.191111 0.639695 -0.242171
C 3.512832 2.009693 -0.265007
C 2.179099 2.150322 -0.288820
O 1.383484 -2.626651 -1.045314
O 3.640781 -1.437490 -1.429145
O 5.378918 0.704273 -1.029302
C 4.521576 0.215773 1.202108
C 5.148993 -1.162857 1.261045
C 4.624083 -2.113487 2.302582
O 6.070216 -1.476982 0.507531
O 4.267593 3.167428 -0.416174
C 5.320577 3.346832 0.522125
C -4.626991 -0.817388 0.290076
C -5.185575 -1.952839 1.105141
C -5.350403 0.207246 -0.210121
C -6.843961 0.426880 -0.082716
C -7.101584 1.652659 0.812066
C -8.576655 1.889585 1.105679
C -7.449509 0.588089 -1.480543
H -1.063790 -3.279861 -0.759759
H -2.740545 1.169593 0.380550
H -0.473338 2.137320 0.170732
H 1.736961 3.141235 -0.349510
H 2.308251 -2.546293 -1.376217
H 5.765381 -0.198403 -1.029377
H 3.616157 0.212283 1.821096
H 5.243291 0.889192 1.674936
H 5.161339 -3.063687 2.238568
H 4.775105 -1.686396 3.296903
H 3.561259 -2.297642 2.126712
H 4.953888 3.238515 1.547971
H 6.151738 2.663584 0.330758
H 5.698929 4.367195 0.408206
H -4.509702 -2.209923 1.929265
H -6.144480 -1.708765 1.569438
H -5.324920 -2.842238 0.481658
H -4.844707 0.965083 -0.809223
H -7.324385 -0.444152 0.373998
H -6.581855 1.517470 1.769622
H -6.680881 2.556083 0.352298
H -9.039816 0.997096 1.537991
H -8.688905 2.709753 1.822203
H -9.127424 2.164280 0.201481
H -8.542354 0.629210 -1.435727
H -7.183811 -0.259666 -2.122546
H -7.096545 1.503483 -1.968753
SCF Energy (B3LYP/6-31G**)= -1361.69971131
Number of imaginary frequencies = 0

1b_c382
MMFF Geometry
C -1.727912 -2.211019 -0.426619
C -2.473990 0.394548 -0.638606
N -3.052397 -1.926495 -0.423279
C -0.707582 -1.260451 -0.528832
C -1.105936 0.085619 -0.640266
C -3.412487 -0.628657 -0.520714
C -0.127440 1.089400 -0.760397
C 1.236655 0.773290 -0.749174
C 1.627691 -0.563582 -0.626617
C 0.665468 -1.578750 -0.532896
C 3.077596 -0.875616 -0.594867
C 4.058778 0.256524 -0.200394
C 3.531340 1.597035 -0.701336
C 2.233585 1.822306 -0.947865
O 1.020716 -2.902704 -0.437567
O 3.427520 -2.038844 -0.803313
O 5.320073 0.009419 -0.817922
C 4.174296 0.225358 1.334966
C 5.470940 -0.359645 1.849918
C 5.709258 -1.835040 1.657477
O 6.286655 0.350218 2.442485
O 4.443775 2.570827 -1.088104
C 5.281525 3.038832 -0.031478
C -4.871008 -0.363850 -0.517712
C -5.679935 -1.339371 -1.329760
C -5.380434 0.661585 0.197978
C -6.837146 1.042575 0.352740
C -7.258029 1.070586 1.834974
C -7.188907 -0.297277 2.503001
C -7.067380 2.415106 -0.284885
H -1.497911 -3.271058 -0.341508
H -2.805429 1.423235 -0.742584
H -0.438285 2.128466 -0.866533
H 1.909012 2.778037 -1.350706
H 1.986277 -2.985065 -0.611957
H 5.247731 0.284875 -1.749791
H 3.351840 -0.329774 1.804327
H 4.096646 1.235340 1.756666
H 6.551850 -2.150448 2.279645
H 4.824130 -2.394620 1.970550
H 5.942925 -2.052458 0.614075
H 4.685518 3.358243 0.829559
H 6.013244 2.281630 0.261172
H 5.831484 3.908584 -0.402950
H -5.813459 -2.278166 -0.781917
H -6.669048 -0.955943 -1.591916
H -5.181620 -1.561086 -2.280850
H -4.700701 1.287845 0.776342
H -7.488894 0.328447 -0.159969
H -6.633449 1.776058 2.397521
H -8.289742 1.436646 1.909353
H -6.161710 -0.671381 2.549672
H -7.566236 -0.233105 3.528661
H -7.799230 -1.028270 1.963160
H -8.121998 2.702784 -0.214594
H -6.796261 2.404787 -1.346596

H -6.473479 3.193484 0.207690
SCF Energy (B3LYP/6-31G**)= -1361.69418583
Number of imaginary frequencies = 0

1b_c383
MMFF Geometry
C -1.434533 -2.221896 -0.621651
C -2.308521 0.352411 -0.648261
N -2.771573 -2.004042 -0.608083
C -0.462546 -1.217090 -0.643278
C -0.926774 0.112372 -0.658198
C -3.195437 -0.721822 -0.614858
C 0.001389 1.169335 -0.690992
C 1.379284 0.920498 -0.687312
C 1.834507 -0.400004 -0.665866
C 0.924430 -1.465844 -0.657794
C 3.295540 -0.640317 -0.639885
C 4.215770 0.497470 -0.129305
C 3.633443 1.852432 -0.532400
C 2.326857 2.027412 -0.780236
O 1.346913 -2.773353 -0.652291
O 3.705117 -1.765299 -0.932813
O 5.501861 0.355536 -0.729690
C 4.318387 0.379158 1.403772
C 4.844917 -0.974992 1.835990
C 4.124628 -1.665544 2.962201
O 5.839079 -1.471290 1.306423
O 4.472260 2.926958 -0.805607
C 5.396115 3.267471 0.220017
C -4.665560 -0.530476 -0.608153
C -5.417802 -1.481581 -1.500019
C -5.231382 0.414943 0.172534
C -6.707814 0.713179 0.335435
C -7.088754 0.600874 1.824482
C -8.581676 0.754848 2.081519
C -7.003042 2.101118 -0.237449
H -1.152194 -3.272648 -0.614745
H -2.689990 1.368406 -0.681032
H -0.359184 2.197166 -0.722049
H 1.959610 2.996462 -1.107585
H 2.311835 -2.794213 -0.851349
H 5.827779 -0.538564 -0.487725
H 3.338297 0.536030 1.870415
H 5.009020 1.114076 1.829041
H 4.605448 -2.624443 3.174209
H 4.164748 -1.042226 3.858724
H 3.086392 -1.848586 2.674106
H 4.886549 3.391046 1.181072
H 6.201555 2.532901 0.297147
H 5.849170 4.227783 -0.044204
H -5.511564 -2.463188 -1.023687
H -6.421308 -1.126864 -1.747822
H -4.899454 -1.609430 -2.457656
H -4.587849 1.032523 0.799645
H -7.312490 -0.013179 -0.216209
H -6.777293 -0.380956 2.204373
H -6.547882 1.351400 2.415001
H -9.159105 0.041044 1.485711
H -8.800909 0.568943 3.137958
H -8.928003 1.765243 1.845932
H -8.077487 2.309455 -0.237139
H -6.659568 2.177559 -1.275543
H -6.505498 2.888418 0.340048
SCF Energy (B3LYP/6-31G**)= -1361.69976684
Number of imaginary frequencies = 0

1b_c384
MMFF Geometry
C -1.838646 -1.447089 -0.631062
C -2.209582 1.127430 0.151286
N -3.103427 -1.028905 -0.385212
C -0.698849 -0.649219 -0.509409
C -0.902221 0.681893 -0.103212
C -3.286313 0.254019 0.003625
C 0.206226 1.538443 0.032976
C 1.509197 1.084461 -0.213200
C 1.705060 -0.241682 -0.604680
C 0.610088 -1.102315 -0.769662
C 3.089816 -0.710923 -0.840370
C 4.251304 0.075653 -0.185379
C 3.905648 1.568809 -0.134893
C 2.636164 2.014191 -0.125157
O 0.771044 -2.403831 -1.180257
O 3.239246 -1.746518 -1.491613
O 5.442205 -0.089389 -0.954627
C 4.475311 -0.451196 1.241275
C 5.491016 -1.570433 1.369692
C 5.284194 -2.835463 0.580665
O 6.428821 -1.474234 2.166274
O 5.029451 2.375350 -0.102326
C 4.860674 3.782922 0.026561
C -4.695246 0.679796 0.247527
C -5.003819 2.142614 0.075186
C -5.619737 -0.249774 0.576347
C -7.087391 -0.046634 0.880800
C -7.982810 -0.729630 -0.169416
C -7.847446 -0.123431 -1.560662
C -7.393132 -0.607568 2.273832
H -1.763607 -2.487085 -0.941666
H -2.374847 2.149536 0.475748
H 0.044091 2.573222 0.333454
H 2.392104 3.069845 -0.075727
H 1.700925 -2.526948 -1.481002
H 5.602211 0.743141 -1.434074
H 3.541414 -0.791398 1.705263
H 4.851907 0.360021 1.880324
H 5.930661 -3.621681 0.982135
H 4.247757 -3.167412 0.678544
H 5.542893 -2.677514 -0.467445
H 4.332025 4.192916 -0.839814
H 4.341197 4.030719 0.957765
H 5.854457 4.238826 0.062229
H -4.504558 2.545437 -0.813617
H -6.068391 2.340777 -0.071218
H -4.674473 2.711307 0.950995
H -5.299119 -1.291362 0.650713
H -7.341510 1.017431 0.908987
H -7.758636 -1.802550 -0.223400
H -9.031763 -0.643519 0.140801
H -6.843133 -0.271354 -1.968836
H -8.557521 -0.596758 -2.246421
H -8.059537 0.950290 -1.542621
H -8.442572 -0.436964 2.537656
H -6.775549 -0.120660 3.036982
H -7.205621 -1.686200 2.325101
SCF Energy (B3LYP/6-31G**)= -1361.70092927
Number of imaginary frequencies = 0

1b_c385
MMFF Geometry
C -1.606127 -1.559316 -0.616945
C -2.102123 1.022650 0.068095
N -2.892089 -1.192054 -0.398091
C -0.505477 -0.705009 -0.517770
C -0.774029 0.630997 -0.163500
C -3.134529 0.093734 -0.054928
C 0.289584 1.547245 -0.061335
C 1.611573 1.145174 -0.284537
C 1.871904 -0.188687 -0.618001
C 0.824038 -1.106963 -0.756629
C 3.280973 -0.584413 -0.843277
C 4.381258 0.268994 -0.159852
C 3.978347 1.738988 -0.214888
C 2.695645 2.128121 -0.265609
O 1.048652 -2.409097 -1.131229
O 3.516318 -1.583699 -1.523641
O 5.619829 0.099027 -0.854468
C 4.596747 -0.193660 1.285877
C 4.557003 -1.699338 1.421518
C 5.776565 -2.480336 1.010161
O 3.538054 -2.258300 1.829830
O 4.960474 2.705756 -0.379298

C 5.859862 2.804251 0.721622
C -4.559115 0.457927 0.195592
C -4.804168 1.543145 1.206363
C -5.533354 -0.216882 -0.454601
C -7.039144 -0.049394 -0.425012
C -7.647479 -0.721139 0.819262
C -9.169539 -0.765661 0.799102
C -7.504909 1.394781 -0.634440
H -1.478511 -2.605944 -0.885303
H -2.322812 2.052867 0.327718
H 0.078306 2.586098 0.189433
H 2.441424 3.181351 -0.346494
H 2.000846 -2.517003 -1.352032
H 5.488133 0.456375 -1.751963
H 3.818804 0.204480 1.950475
H 5.558264 0.143842 1.688540
H 5.690298 -3.507855 1.374756
H 5.862836 -2.495127 -0.077996
H 6.669606 -2.032977 1.453602
H 5.324925 2.804452 1.677436
H 6.599844 1.999962 0.692420
H 6.395676 3.753926 0.632403
H -4.108083 1.461593 2.049238
H -4.684993 2.530848 0.749292
H -5.801434 1.489475 1.647218
H -5.223727 -1.014080 -1.135423
H -7.406773 -0.603726 -1.301303
H -7.275536 -1.751289 0.893552
H -7.325200 -0.208061 1.732418
H -9.536811 -1.253447 -0.109258
H -9.539544 -1.331497 1.660232
H -9.600313 0.238505 0.853510
H -8.552134 1.416373 -0.955683
H -6.919050 1.891781 -1.415585
H -7.437061 1.992718 0.278277
SCF Energy (B3LYP/6-31G**)= -1361.68694549
Number of imaginary frequencies = 0

1b_c386
MMFF Geometry
C -1.800068 -1.643547 -0.333288
C -2.233183 0.916257 0.465306
N -3.064219 -1.279219 -0.010481
C -0.690698 -0.796454 -0.284722
C -0.926472 0.527175 0.128450
C -3.277698 -0.004047 0.387973
C 0.148805 1.433382 0.186922
C 1.452162 1.035555 -0.140775
C 1.681569 -0.283911 -0.536602
C 0.617766 -1.193355 -0.626388
C 3.067925 -0.693191 -0.858748
C 4.232621 0.149409 -0.284023
C 3.824584 1.626272 -0.220727
C 2.539679 2.015075 -0.131630
O 0.809798 -2.489402 -1.040889
O 3.221485 -1.726185 -1.513131
O 5.378481 0.030055 -1.126573
C 4.570010 -0.354210 1.128635
C 5.639707 -1.427371 1.199013
C 5.439496 -2.706623 0.431507
O 6.620198 -1.284290 1.934853
O 4.910874 2.482054 -0.266005
C 4.687793 3.881992 -0.136631
C -4.680319 0.353625 0.748186
C -4.866384 1.478934 1.729794
C -5.701647 -0.349003 0.209936
C -7.186616 -0.145374 0.412369
C -7.922802 0.006464 -0.933298
C -7.505418 1.251121 -1.707120
C -7.756492 -1.333016 1.191761
H -1.699156 -2.681022 -0.645000
H -2.426997 1.938907 0.771846
H -0.039838 2.462533 0.491232
H 2.252233 3.059292 -0.073228
H 1.723188 -2.573699 -1.399899
H 5.466211 0.861651 -1.625856
H 3.683355 -0.730115 1.653727
H 4.950882 0.478390 1.736910
H 6.144032 -3.460563 0.795435
H 4.426941 -3.082685 0.596674
H 5.624395 -2.546156 -0.631725
H 4.086767 4.259442 -0.969981
H 4.218582 4.115908 0.824369
H 5.660377 4.381785 -0.168491
H -4.753294 2.447174 1.231160
H -5.845393 1.463382 2.215344
H -4.134093 1.414670 2.542877
H -5.461795 -1.166391 -0.473764
H -7.384613 0.759533 0.995282
H -7.758873 -0.878011 -1.561864
H -9.002814 0.063985 -0.748244
H -6.454475 1.204732 -2.008201
H -8.107828 1.346987 -2.616177
H -7.653706 2.154068 -1.106312
H -8.827216 -1.196130 1.378099
H -7.261414 -1.438277 2.163688
H -7.628533 -2.273666 0.644027
SCF Energy (B3LYP/6-31G**)= -1361.70101506
Number of imaginary frequencies = 0

1b_c387
MMFF Geometry
C 1.874946 2.193449 0.157964
C 2.283472 -0.342465 -0.735412
N 3.152858 1.762062 0.032819
C 0.741991 1.428915 -0.137788
C 0.965525 0.117379 -0.598977
C 3.344142 0.501634 -0.415639
C -0.134008 -0.700809 -0.918241
C -1.448463 -0.238343 -0.771173
C -1.663372 1.059425 -0.299319
C -0.579791 1.897422 0.000066
C -3.059293 1.522171 -0.128493
C -4.164365 0.449544 0.008215
C -3.838439 -0.766830 -0.851609
C -2.572302 -1.085840 -1.177659
O -0.764644 3.187210 0.436909
O -3.263124 2.734943 -0.047760
O -5.402205 1.028570 -0.420043
C -4.289490 0.131403 1.502727
C -5.148189 -1.080166 1.787033
C -6.643552 -0.923268 1.696278
O -4.634043 -2.164213 2.072180
O -4.958498 -1.483567 -1.231036
C -4.776263 -2.772259 -1.807397
C 4.753644 0.057966 -0.546024
C 5.125704 -0.609002 -1.837262
C 5.578518 0.265535 0.502996
C 7.043072 -0.050427 0.732364
C 7.204780 -1.175788 1.776873
C 6.680529 -2.538887 1.341325
C 7.921626 -0.293574 -0.495450
H 1.780949 3.215289 0.519563
H 2.481233 -1.354996 -1.073651
H 0.043466 -1.710601 -1.287216
H -2.330839 -1.960789 -1.771370
H -1.717756 3.417995 0.345978
H -5.375567 1.070026 -1.392011
H -4.717950 0.979563 2.051708
H -3.305671 -0.058136 1.951885
H -7.127885 -1.781068 2.171656
H -6.954213 -0.019053 2.225668
H -6.955532 -0.874439 0.651549
H -4.293660 -2.695483 -2.786729
H -4.206559 -3.424961 -1.137956
H -5.764785 -3.217593 -1.953187
H 4.296133 -0.638172 -2.552029
H 5.929156 -0.063901 -2.340766
H 5.436987 -1.643699 -1.662544
H 5.138638 0.756751 1.376780
H 7.455343 0.857211 1.197954
H 8.266475 -1.275963 2.035539

H 6.691581 -0.887218 2.703476
H 7.210588 -2.911511 0.460346
H 6.826605 -3.266841 2.146233
H 5.610712 -2.506537 1.115266
H 8.972419 -0.383992 -0.196978
H 7.857971 0.547414 -1.193993
H 7.659733 -1.210384 -1.029314
SCF Energy (B3LYP/6-31G**)= -1361.69179892
Number of imaginary frequencies = 0

1b_c388
MMFF Geometry
C 1.874654 2.108183 0.047734
C 2.324170 -0.557442 0.342017
N 3.144962 1.702039 0.285563
C 0.769340 1.256844 -0.057315
C 1.014629 -0.121193 0.096982
C 3.354376 0.376230 0.426859
C -0.054434 -1.029948 -0.002830
C -1.361832 -0.586684 -0.241400
C -1.602837 0.783576 -0.380557
C -0.545192 1.701648 -0.305221
C -2.993043 1.237119 -0.615718
C -4.160355 0.275889 -0.312215
C -3.722666 -1.164774 -0.469474
C -2.442865 -1.558392 -0.391690
O -0.746429 3.051916 -0.464331
O -3.194048 2.391942 -1.005892
O -5.176472 0.582838 -1.279696
C -4.674174 0.632917 1.086188
C -5.683361 -0.340882 1.653372
C -7.053388 -0.388286 1.027895
O -5.399918 -1.041837 2.627000
O -4.732049 -2.107542 -0.585652
C -4.904234 -2.522952 -1.940944
C 4.752643 -0.037764 0.679569
C 5.432433 0.682740 1.807813
C 5.324293 -0.947095 -0.139214
C 6.709803 -1.563998 -0.148592
C 7.561948 -1.014375 -1.311362
C 7.909649 0.465813 -1.215020
C 7.484426 -1.579244 1.170704
H 1.762579 3.184975 -0.060365
H 2.541739 -1.613081 0.474760
H 0.140338 -2.096595 0.106661
H -2.190287 -2.613791 -0.441266
H -1.673616 3.203662 -0.754669
H -5.119957 1.545451 -1.446990
H -5.144753 1.624536 1.089716
H -3.840750 0.685009 1.799692
H -7.719854 -0.983752 1.658632
H -7.460184 0.623678 0.957641
H -7.009067 -0.845323 0.038439
H -5.114160 -1.673482 -2.598527
H -4.023070 -3.067166 -2.296768
H -5.760936 -3.202180 -1.977188
H 4.821797 1.491854 2.223408
H 5.638171 -0.005219 2.633693
H 6.367247 1.141436 1.471814
H 4.717520 -1.322309 -0.967006
H 6.535735 -2.626004 -0.378775
H 8.494672 -1.588913 -1.374998
H 7.033930 -1.184444 -2.258711
H 8.496726 0.684509 -0.318748
H 8.508698 0.763170 -2.082133
H 7.012075 1.090962 -1.204364
H 8.376166 -2.209634 1.076333
H 6.875328 -1.999921 1.977925
H 7.826615 -0.587816 1.475937
SCF Energy (B3LYP/6-31G**)= -1361.69294087
Number of imaginary frequencies = 0

1b_c389
MMFF Geometry
C 1.958833 1.796776 -0.142717
C 2.235775 -0.906663 -0.135156
N 3.209799 1.284910 -0.053075
C 0.789757 1.035820 -0.231827
C 0.945639 -0.362843 -0.229622
C 3.340037 -0.061154 -0.046616
C -0.191773 -1.184872 -0.329270
C -1.477808 -0.635664 -0.415270
C -1.628193 0.754279 -0.400979
C -0.503662 1.588799 -0.326099
C -2.995464 1.318809 -0.475013
C -4.202963 0.409489 -0.168726
C -3.884923 -1.032734 -0.501657
C -2.635063 -1.514581 -0.569235
O -0.616830 2.958454 -0.339018
O -3.141008 2.517867 -0.734377
O -5.266103 0.879344 -1.012294
C -4.579319 0.653544 1.296138
C -5.607204 -0.308235 1.850039
C -7.022441 -0.203950 1.343425
O -5.298001 -1.123028 2.721900
O -4.966316 -1.890965 -0.624962
C -5.271560 -2.151671 -1.995334
C 4.725002 -0.594790 0.069078
C 4.890476 -1.822766 0.917394
C 5.728667 0.070700 -0.543404
C 7.210466 -0.218337 -0.673064
C 8.056279 0.673780 0.256966
C 7.856497 0.433011 1.747468
C 7.642305 -1.686388 -0.629275
H 1.919643 2.883987 -0.140228
H 2.375049 -1.983238 -0.145596
H -0.068141 -2.267568 -0.340059
H -2.464699 -2.573377 -0.742504
H -1.550012 3.199134 -0.534506
H -5.152013 1.848201 -1.090483
H -4.976894 1.667109 1.435318
H -3.691156 0.580326 1.938167
H -7.677156 -0.820596 1.966062
H -7.361008 0.833032 1.410712
H -7.089113 -0.555174 0.312856
H -5.468742 -1.226852 -2.546612
H -4.462214 -2.711997 -2.475152
H -6.175729 -2.766586 -2.026337
H 4.836026 -2.727931 0.304592
H 5.836238 -1.814689 1.463265
H 4.117290 -1.885493 1.691984
H 5.456140 0.983999 -1.080762
H 7.457609 0.102707 -1.696783
H 9.118257 0.532225 0.019745
H 7.834060 1.727834 0.045301
H 8.124081 -0.588740 2.030413
H 8.495735 1.111931 2.321625
H 6.822219 0.619568 2.050662
H 8.666904 -1.785211 -1.006451
H 7.000613 -2.305577 -1.265337
H 7.638333 -2.106933 0.378173
SCF Energy (B3LYP/6-31G**)= -1361.69326990
Number of imaginary frequencies = 0

1b_c390
MMFF Geometry
C -1.439323 -2.320565 -0.501337
C -2.407565 0.137825 0.139083
N -2.764931 -2.205805 -0.246431
C -0.523459 -1.265130 -0.454657
C -1.035687 0.002960 -0.118956
C -3.237890 -0.978467 0.059601
C -0.164962 1.106278 -0.053829
C 1.205325 0.960132 -0.301715
C 1.708839 -0.300542 -0.631226
C 0.853000 -1.406803 -0.721267
C 3.164353 -0.437227 -0.867534
C 4.108247 0.632339 -0.261564
C 3.418561 1.996754 -0.275052
C 2.083577 2.126439 -0.288808
O 1.321898 -2.654508 -1.055131
O 3.566490 -1.445513 -1.451311

O 5.289768 0.709245 -1.057006
C 4.452614 0.206540 1.178910
C 5.091774 -1.167041 1.228838
C 4.582247 -2.125303 2.271021
O 6.010087 -1.471135 0.467695
O 4.162650 3.161162 -0.427870
C 5.220870 3.346252 0.503407
C -4.693209 -0.899051 0.329337
C -5.238455 -2.041865 1.143235
C -5.428724 0.124163 -0.155915
C -6.922209 0.324459 -0.011631
C -7.255539 1.388716 1.051796
C -6.748301 2.795162 0.756707
C -7.542512 0.651367 -1.373717
H -1.117809 -3.328836 -0.754048
H -2.822670 1.103016 0.412861
H -0.565279 2.090058 0.189768
H 1.632866 3.113855 -0.343085
H 2.243558 -2.565452 -1.392414
H 5.683641 -0.190203 -1.062805
H 3.551747 0.193591 1.804382
H 5.172153 0.884381 1.648726
H 5.126834 -3.070824 2.200046
H 4.736922 -1.700163 3.265616
H 3.519733 -2.317667 2.102202
H 4.862504 3.231627 1.531507
H 6.056226 2.670508 0.303838
H 5.589973 4.370065 0.390096
H -4.558030 -2.294112 1.965046
H -6.198860 -1.806802 1.609824
H -5.371389 -2.931125 0.518186
H -4.932916 0.889022 -0.753613
H -7.392452 -0.610185 0.314452
H -8.344644 1.433858 1.178230
H -6.847739 1.068789 2.019387
H -7.185321 3.195407 -0.162558
H -7.026818 3.468660 1.574050
H -5.658546 2.820815 0.664730
H -8.621130 0.816102 -1.278739
H -7.393565 -0.178126 -2.074512
H -7.101052 1.546019 -1.825556
SCF Energy (B3LYP/6-31G**)= -1361.69870204
Number of imaginary frequencies = 0

1b_c391
MMFF Geometry
C 1.664572 2.103481 0.237885
C 2.204744 -0.553890 0.060684
N 2.947041 1.703582 0.417038
C 0.590442 1.249779 -0.033360
C 0.882228 -0.124122 -0.121395
C 3.204554 0.381564 0.319357
C -0.154592 -1.034727 -0.394325
C -1.475410 -0.599581 -0.568555
C -1.762135 0.764590 -0.470104
C -0.736598 1.688580 -0.217957
C -3.164543 1.206492 -0.641722
C -4.303704 0.162838 -0.593463
C -3.819259 -1.239112 -0.930775
C -2.518338 -1.565143 -0.910372
O -0.980615 3.040098 -0.144503
O -3.404204 2.408658 -0.805102
O -5.212070 0.581511 -1.639743
C -5.063018 0.338495 0.732177
C -4.373693 -0.261162 1.935684
C -5.258393 -0.836028 3.010313
O -3.153163 -0.220121 2.074957
O -4.680651 -2.204086 -1.441799
C -5.980578 -2.303441 -0.883348
C 4.618564 -0.015248 0.526877
C 5.281563 0.678309 1.684132
C 5.201424 -0.906338 -0.304176
C 6.624333 -1.425744 -0.330751
C 7.731821 -0.356125 -0.394341
C 7.583602 0.597903 -1.572930
C 6.876217 -2.425108 0.801263
H 1.514947 3.177627 0.323096
H 2.450151 -1.610381 0.014534
H 0.074547 -2.096868 -0.471212
H -2.208599 -2.564986 -1.202757
H -1.920212 3.200603 -0.384561
H -5.170986 1.558683 -1.668642
H -6.067661 -0.090664 0.654051
H -5.227359 1.401613 0.951732
H -4.641507 -1.254512 3.810438
H -5.890568 -0.046472 3.423712
H -5.875979 -1.634559 2.592748
H -5.932250 -2.413248 0.203500
H -6.607674 -1.455843 -1.169495
H -6.448337 -3.206932 -1.286375
H 4.608149 0.724856 2.548077
H 6.177486 0.160883 2.030739
H 5.563874 1.700523 1.410772
H 4.602277 -1.309827 -1.122057
H 6.709307 -2.001732 -1.264318
H 7.782592 0.224171 0.531845
H 8.701987 -0.861249 -0.486970
H 6.681494 1.210550 -1.485001
H 8.442031 1.276020 -1.616079
H 7.539396 0.049123 -2.519026
H 7.847083 -2.915712 0.670540
H 6.109757 -3.208196 0.812259
H 6.879570 -1.944606 1.783928
SCF Energy (B3LYP/6-31G**)= -1361.69207153
Number of imaginary frequencies = 0

1b_c392
MMFF Geometry
C -1.699893 -1.987775 -0.181867
C -2.403901 0.630259 -0.392722
N -3.012811 -1.694183 -0.341908
C -0.672220 -1.041590 -0.115596
C -1.048878 0.310815 -0.222687
C -3.349942 -0.390172 -0.452230
C -0.062443 1.311537 -0.147571
C 1.290978 0.984971 0.013008
C 1.659528 -0.358983 0.099718
C 0.687693 -1.368956 0.056261
C 3.092734 -0.677968 0.269371
C 4.129129 0.368771 -0.207030
C 3.608458 1.782447 0.069896
C 2.290125 2.047093 0.151998
O 1.019681 -2.696891 0.181580
O 3.387693 -1.777845 0.742268
O 4.276091 0.284434 -1.628032
C 5.475799 0.116733 0.501975
C 6.086595 -1.217726 0.121135
C 6.901261 -1.922757 1.171259
O 5.967339 -1.674380 -1.018146
O 4.609121 2.734106 0.175479
C 4.241107 4.106918 0.238443
C -4.792991 -0.098115 -0.633733
C -5.145108 0.859425 -1.733646
C -5.666495 -0.692072 0.207769
C -7.176196 -0.630978 0.326974
C -7.595673 0.123225 1.607120
C -7.244796 1.606175 1.625095
C -7.969592 -0.154973 -0.890594
H -1.485180 -3.051555 -0.104893
H -2.720481 1.666254 -0.463418
H -0.358596 2.357539 -0.216651
H 1.910066 3.048459 0.317998
H 1.972888 -2.760532 0.416231
H 4.777935 -0.538894 -1.815518
H 5.341940 0.147705 1.589954
H 6.226491 0.866499 0.224785
H 7.271045 -2.872284 0.774628
H 7.752516 -1.298563 1.453367
H 6.276389 -2.127222 2.044138
H 3.669102 4.401398 -0.647387
H 3.683281 4.319275 1.155983
H 5.159950 4.700362 0.259070

H -4.269268 1.190176 -2.302370
H -5.809551 0.388137 -2.463447
H -5.622847 1.756477 -1.327341
H -5.236449 -1.356211 0.964208
H -7.487546 -1.675874 0.474631
H -8.679021 0.018638 1.746971
H -7.128717 -0.353793 2.478696
H -7.744948 2.148171 0.817616
H -7.568356 2.052339 2.571339
H -6.166462 1.766978 1.535668
H -9.044249 -0.279006 -0.714091
H -7.719501 -0.748629 -1.776203
H -7.801825 0.900212 -1.119881
SCF Energy (B3LYP/6-31G**)= -1361.70157253
Number of imaginary frequencies = 0

1b_c393
MMFF Geometry
C -1.561789 -1.639246 -0.796300
C -2.136243 0.955984 -0.234785
N -2.863485 -1.287173 -0.661679
C -0.482045 -0.762769 -0.667795
C -0.790981 0.579411 -0.375708
C -3.146148 0.005862 -0.381609
C 0.251151 1.516035 -0.241499
C 1.589790 1.129407 -0.374643
C 1.889038 -0.209747 -0.648922
C 0.864940 -1.149659 -0.816745
C 3.314570 -0.588635 -0.779576
C 4.360275 0.304131 -0.061642
C 3.939324 1.764709 -0.187343
C 2.656118 2.130503 -0.324822
O 1.130478 -2.459445 -1.133351
O 3.604020 -1.604658 -1.412631
O 5.639674 0.133364 -0.677596
C 4.497822 -0.109433 1.408279
C 4.474251 -1.610677 1.589468
C 5.728213 -2.382178 1.275023
O 3.442120 -2.174886 1.955239
O 4.914843 2.742189 -0.325761
C 5.746527 2.890469 0.821827
C -4.591303 0.358504 -0.257222
C -4.969948 1.764866 -0.629246
C -5.465725 -0.591974 0.142534
C -6.961410 -0.526864 0.377460
C -7.378015 0.574163 1.371509
C -8.818517 0.435108 1.848777
C -7.736116 -0.488455 -0.941842
H -1.403163 -2.692190 -1.019604
H -2.384259 1.985438 0.001694
H 0.010156 2.558570 -0.037092
H 2.391389 3.176281 -0.454108
H 2.095284 -2.558265 -1.295972
H 5.555223 0.460064 -1.592289
H 3.675936 0.295247 2.013366
H 5.428532 0.257451 1.855187
H 5.637564 -3.398929 1.667663
H 5.877879 -2.430095 0.194719
H 6.586568 -1.904824 1.754245
H 5.156509 2.911767 1.744412
H 6.498912 2.098240 0.861790
H 6.272364 3.845756 0.733154
H -4.735879 2.456388 0.186812
H -4.434816 2.090288 -1.528848
H -6.030118 1.871273 -0.864132
H -5.063046 -1.585779 0.355459
H -7.217888 -1.484504 0.854347
H -6.721619 0.537679 2.250321
H -7.261066 1.569748 0.932622
H -8.994660 -0.551818 2.287964
H -9.033994 1.188296 2.613620
H -9.528177 0.583122 1.029628
H -8.796761 -0.705431 -0.778572
H -7.357734 -1.241228 -1.642787
H -7.670505 0.488058 -1.430109
SCF Energy (B3LYP/6-31G**)= -1361.68723068
Number of imaginary frequencies = 0

1b_c394
MMFF Geometry
C -1.955738 2.141056 -0.114312
C -2.480725 -0.516142 -0.329674
N -3.246539 1.759334 -0.270118
C -0.864550 1.267549 -0.057569
C -1.149013 -0.104457 -0.172526
C -3.497034 0.436184 -0.366247
C -0.093722 -1.032144 -0.128318
C 1.237293 -0.616411 0.028429
C 1.520387 0.747675 0.142342
C 0.473462 1.685594 0.102495
C 2.929537 1.188064 0.300253
C 4.068833 0.146630 0.279926
C 3.580166 -1.284005 0.218174
C 2.293595 -1.622035 0.070823
O 0.703216 3.037441 0.212200
O 3.150811 2.396373 0.438443
O 4.725293 0.342422 1.537312
C 4.965607 0.496366 -0.921326
C 6.377906 -0.021455 -0.787520
C 6.851193 -1.026791 -1.802976
O 7.131868 0.415810 0.083474
O 4.532339 -2.294776 0.178839
C 5.099279 -2.550738 1.466245
C -4.921733 0.061331 -0.537369
C -5.665748 0.926337 -1.516347
C -5.444368 -0.960042 0.175451
C -6.862027 -1.493252 0.215865
C -7.962219 -0.461294 0.530966
C -7.730799 0.289190 1.836502
C -7.193443 -2.297325 -1.044000
H -1.814288 3.216848 -0.033710
H -2.722195 -1.569317 -0.435767
H -0.318144 -2.095047 -0.215842
H 2.016997 -2.669172 -0.017457
H 1.664444 3.182294 0.368598
H 5.329579 1.104255 1.440502
H 5.068715 1.583032 -1.041675
H 4.524648 0.137032 -1.859459
H 7.799859 -1.460405 -1.474840
H 6.121504 -1.832307 -1.907896
H 6.994805 -0.528594 -2.764795
H 5.913506 -1.851093 1.672934
H 4.345945 -2.515701 2.261057
H 5.524905 -3.558500 1.446482
H -5.055446 1.121236 -2.406068
H -6.583799 0.461652 -1.879342
H -5.928335 1.886808 -1.060396
H -4.789814 -1.484037 0.873750
H -6.880701 -2.214899 1.046179
H -8.078568 0.261786 -0.281900
H -8.923367 -0.986001 0.607320
H -6.837214 0.918422 1.787121
H -8.584019 0.941137 2.050006
H -7.619586 -0.406122 2.674585
H -8.152549 -2.814009 -0.927731
H -6.429620 -3.059104 -1.236547
H -7.266531 -1.662999 -1.932142
SCF Energy (B3LYP/6-31G**)= -1361.69719058
Number of imaginary frequencies = 0

1b_c395
MMFF Geometry
C -1.590919 -1.771692 -0.994040
C -2.248374 0.800068 -0.403637
N -2.905707 -1.451723 -0.925788
C -0.538903 -0.874746 -0.783119
C -0.891217 0.453779 -0.476910
C -3.220379 -0.170173 -0.635266
C 0.121955 1.405583 -0.257273
C 1.476397 1.052346 -0.325629
C 1.816581 -0.271824 -0.616281
C 0.822735 -1.228842 -0.862202

C 3.248617 -0.618344 -0.692715
C 4.268303 0.299932 0.008286
C 3.807259 1.752675 -0.000303
C 2.508752 2.079192 -0.146884
O 1.131876 -2.526969 -1.188566
O 3.601644 -1.629082 -1.307099
O 5.495596 0.201346 -0.737382
C 4.551627 -0.130569 1.449490
C 4.622279 -1.630724 1.602478
C 5.942502 -2.304143 1.340362
O 3.613668 -2.275878 1.892045
O 4.834252 2.656671 0.204423
C 4.546166 4.047214 0.133800
C -4.666901 0.151995 -0.569957
C -5.112215 1.372403 -1.320036
C -5.458631 -0.655335 0.168466
C -6.944600 -0.638724 0.466324
C -7.204558 -0.267275 1.942128
C -6.817612 1.155981 2.325786
C -7.853119 0.148168 -0.479155
H -1.395675 -2.815245 -1.232145
H -2.545432 1.813938 -0.154023
H -0.155914 2.434860 -0.033037
H 2.168419 3.108633 -0.127710
H 2.106659 -2.609537 -1.283533
H 5.403605 -0.564934 -1.338325
H 3.761032 0.213384 2.129764
H 5.492618 0.294939 1.819323
H 5.887509 -3.349662 1.656575
H 6.175261 -2.267172 0.274400
H 6.729698 -1.811436 1.916356
H 4.140274 4.314540 -0.847199
H 3.863264 4.345067 0.935756
H 5.484398 4.592864 0.270954
H -4.295014 1.851971 -1.869710
H -5.862856 1.115481 -2.072858
H -5.521262 2.120489 -0.633693
H -4.965639 -1.498721 0.662540
H -7.263606 -1.685944 0.356279
H -8.268655 -0.412914 2.167132
H -6.657877 -0.962212 2.592852
H -7.388761 1.895612 1.757747
H -7.025902 1.323408 3.387725
H -5.751668 1.341812 2.164884
H -8.905101 -0.026167 -0.225452
H -7.714843 -0.178152 -1.515338
H -7.685350 1.226784 -0.428390
SCF Energy (B3LYP/6-31G**)= -1361.69379722
Number of imaginary frequencies = 0

1b_c396
MMFF Geometry
C -1.897435 -2.386042 -0.096311
C -2.559807 0.248837 -0.131465
N -3.212999 -2.061738 -0.078107
C -0.846454 -1.463386 -0.130382
C -1.202206 -0.103386 -0.150293
C -3.530609 -0.749665 -0.087600
C -0.190782 0.872059 -0.191501
C 1.166342 0.515787 -0.208020
C 1.520378 -0.836235 -0.184205
C 0.518025 -1.821833 -0.149405
C 2.956530 -1.213001 -0.193600
C 4.041072 -0.114726 -0.170669
C 3.483669 1.288503 -0.268547
C 2.175123 1.569008 -0.252043
O 0.817978 -3.164209 -0.130340
O 3.243886 -2.415138 -0.212621
O 4.808432 -0.375866 -1.351037
C 4.849687 -0.319340 1.123062
C 6.242904 0.259819 1.058559
C 6.582947 1.367257 2.019707
O 7.086318 -0.205786 0.290346
O 4.381770 2.347946 -0.239751
C 5.041199 2.529056 -1.495420
C -4.980403 -0.436880 -0.074461
C -5.795936 -1.296151 -1.000199
C -5.456620 0.531587 0.737664
C -6.878561 1.006552 0.956550
C -7.461216 1.811887 -0.219753
C -6.633944 3.039965 -0.579822
C -7.827373 -0.113426 1.394281
H -1.700547 -3.456042 -0.083011
H -2.858201 1.292234 -0.165022
H -0.470391 1.925244 -0.211690
H 1.844264 2.603812 -0.274682
H 1.794632 -3.270132 -0.196110
H 5.436435 -1.095056 -1.142956
H 4.992254 -1.385930 1.342202
H 4.316004 0.090251 1.989708
H 7.534628 1.822644 1.732327
H 5.810639 2.138864 1.997998
H 6.668715 0.956386 3.028543
H 5.901332 1.859226 -1.577207
H 4.358936 2.390153 -2.341405
H 5.416549 3.556294 -1.527475
H -5.936809 -2.295653 -0.575228
H -6.781780 -0.878815 -1.209763
H -5.298936 -1.400211 -1.971870
H -4.746544 1.055530 1.379448
H -6.829036 1.695459 1.812968
H -7.569173 1.184327 -1.110568
H -8.472012 2.146987 0.045416
H -5.643537 2.762654 -0.953388
H -7.136609 3.613171 -1.365527
H -6.506610 3.695523 0.287486
H -8.768918 0.310433 1.761457
H -7.392161 -0.703460 2.208402
H -8.078370 -0.794782 0.577156
SCF Energy (B3LYP/6-31G**)= -1361.69730427
Number of imaginary frequencies = 0

1b_c397
MMFF Geometry
C -1.685440 -1.309625 -0.813971
C -2.097063 1.227994 0.064112
N -2.958501 -0.914961 -0.571843
C -0.556427 -0.506337 -0.639804
C -0.780882 0.805499 -0.182749
C -3.161586 0.350483 -0.137754
C 0.315534 1.666080 0.011886
C 1.625791 1.232528 -0.225044
C 1.842183 -0.071925 -0.675116
C 0.761201 -0.936025 -0.895504
C 3.236710 -0.517349 -0.898285
C 4.373482 0.240999 -0.167036
C 4.013888 1.722413 -0.050001
C 2.745320 2.157555 -0.074339
O 0.946332 -2.219417 -1.350039
O 3.422378 -1.529337 -1.576911
O 5.573295 0.120526 -0.928989
C 4.551884 -0.391599 1.226825
C 4.858540 -1.873762 1.143814
C 4.099490 -2.786135 2.068712
O 5.715370 -2.305352 0.372648
O 5.009326 2.692839 -0.066456
C 6.042645 2.536262 0.897451
C -4.578601 0.752910 0.097605
C -4.900462 2.218151 -0.023900
C -5.501005 -0.196094 0.372105
C -6.974950 -0.009132 0.662198
C -7.798362 -0.526710 -0.530736
C -9.293006 -0.272030 -0.391677
C -7.325496 -0.729237 1.968303
H -1.594194 -2.334697 -1.166875
H -2.278570 2.235219 0.424705
H 0.140805 2.687281 0.349552
H 2.534175 3.222811 -0.033121
H 1.877249 -2.313628 -1.659253
H 5.752126 -0.840529 -1.021572
H 3.646749 -0.257254 1.831491
H 5.386942 0.052396 1.777711

H 4.416928 -3.820196 1.908801
H 4.303741 -2.508232 3.105505
H 3.029424 -2.710220 1.859832
H 5.625576 2.407169 1.901412
H 6.709553 1.708855 0.642690
H 6.639664 3.453234 0.898712
H -4.381524 2.663645 -0.880478
H -5.963487 2.407321 -0.194594
H -4.604173 2.754119 0.883734
H -5.175508 -1.238204 0.404761
H -7.205594 1.050221 0.813830
H -7.453864 -0.034202 -1.449452
H -7.632321 -1.602479 -0.671053
H -9.496565 0.789390 -0.218753
H -9.810789 -0.570714 -1.308939
H -9.723959 -0.847870 0.432378
H -8.354337 -0.514861 2.274179
H -6.670675 -0.401763 2.784050
H -7.221806 -1.815650 1.869062
SCF Energy (B3LYP/6-31G**)= -1361.70062880
Number of imaginary frequencies = 0

1b_c398
MMFF Geometry
C -1.904996 -1.474732 -0.506133
C -2.280982 1.159910 0.038107
N -3.169346 -1.040630 -0.287255
C -0.767840 -0.664826 -0.470131
C -0.974007 0.697849 -0.186269
C -3.354779 0.272334 -0.017604
C 0.130712 1.568394 -0.140066
C 1.432068 1.097473 -0.354332
C 1.631755 -0.259138 -0.627204
C 0.540554 -1.135764 -0.700959
C 3.019431 -0.742625 -0.829828
C 4.179626 0.110241 -0.258336
C 3.820140 1.590570 -0.332844
C 2.555283 2.031137 -0.374587
O 0.704599 -2.468967 -0.990214
O 3.181137 -1.838472 -1.370151
O 5.346142 -0.110444 -1.048162
C 4.394049 -0.353641 1.194429
C 5.625070 -1.208720 1.399519
C 5.633958 -2.593262 0.804876
O 6.573725 -0.790983 2.067333
O 4.836104 2.514424 -0.544347
C 5.803878 2.568724 0.503407
C -4.763500 0.713452 0.199064
C -5.079948 2.153292 -0.103715
C -5.681047 -0.185375 0.619962
C -7.146864 0.039687 0.917770
C -8.048408 -0.739629 -0.057473
C -7.927709 -0.262768 -1.499485
C -7.438128 -0.392530 2.358959
H -1.828198 -2.538624 -0.720275
H -2.447783 2.207260 0.267604
H -0.031279 2.626838 0.062394
H 2.346832 3.092795 -0.476794
H 1.633265 -2.618115 -1.281429
H 5.250693 0.415283 -1.863004
H 3.534265 -0.913042 1.585271
H 4.491437 0.504018 1.871822
H 6.463772 -3.165230 1.230078
H 4.701499 -3.106412 1.053457
H 5.760037 -2.544394 -0.277840
H 5.321527 2.709266 1.476139
H 6.436016 1.677226 0.507684
H 6.447652 3.433550 0.317532
H -4.590739 2.474577 -1.030510
H -6.146669 2.333574 -0.257376
H -4.744940 2.801049 0.713049
H -5.355267 -1.214594 0.786468
H -7.405309 1.100971 0.850846
H -7.820105 -1.812202 -0.015143
H -9.094994 -0.629158 0.253021
H -6.926347 -0.443912 -1.901469
H -8.641663 -0.799308 -2.132655
H -8.144242 0.807357 -1.577788
H -8.485966 -0.202155 2.615525
H -6.816054 0.164289 3.068792
H -7.245562 -1.461292 2.506951
SCF Energy (B3LYP/6-31G**)= -1361.69519036
Number of imaginary frequencies = 0

1b_c399
MMFF Geometry
C -1.870466 -1.638976 -0.193079
C -2.302391 0.983507 0.368063
N -3.132798 -1.247748 0.104276
C -0.762125 -0.790129 -0.231309
C -0.997425 0.566263 0.059935
C -3.345823 0.058886 0.383903
C 0.075959 1.475749 0.027550
C 1.376422 1.049393 -0.269482
C 1.606133 -0.301435 -0.547395
C 0.544333 -1.216426 -0.545019
C 2.993915 -0.737187 -0.838380
C 4.157882 0.160625 -0.349358
C 3.741590 1.626700 -0.408182
C 2.461770 2.021957 -0.368942
O 0.736277 -2.544948 -0.838625
O 3.158925 -1.830884 -1.382051
O 5.277119 -0.024959 -1.213153
C 4.483694 -0.283969 1.088654
C 5.755713 -1.092732 1.217052
C 5.775837 -2.480721 0.630770
O 6.730261 -0.636018 1.818794
O 4.707765 2.584199 -0.691226
C 5.739999 2.680584 0.289850
C -4.746706 0.446890 0.719164
C -4.928262 1.661922 1.588300
C -5.770393 -0.306488 0.259743
C -7.254295 -0.086844 0.453099
C -8.001106 -0.067628 -0.895146
C -7.592823 1.096826 -1.789311
C -7.815159 -1.194311 1.348453
H -1.770356 -2.700665 -0.408530
H -2.495376 2.030202 0.579411
H -0.110165 2.529416 0.234135
H 2.209191 3.074737 -0.463217
H 1.648858 -2.663499 -1.188784
H 5.109410 0.490324 -2.022990
H 3.672231 -0.870803 1.538177
H 4.593989 0.581874 1.753547
H 5.828418 -2.435946 -0.458168
H 6.652161 -3.019067 1.003295
H 4.881100 -3.025106 0.943319
H 5.318171 2.811304 1.291644
H 6.402552 1.812070 0.256879
H 6.338736 3.566216 0.057282
H -4.819894 2.577380 0.997440
H -5.904093 1.692727 2.079460
H -4.190724 1.677391 2.399029
H -5.533790 -1.185701 -0.343614
H -7.450232 0.869969 0.947001
H -7.839711 -1.008621 -1.436208
H -9.079820 0.005621 -0.708088
H -6.544091 1.023344 -2.092698
H -8.202390 1.102945 -2.698632
H -7.738839 2.053538 -1.277791
H -8.884786 -1.041956 1.529000
H -7.312435 -1.203869 2.322107
H -7.688941 -2.183429 0.893564
SCF Energy (B3LYP/6-31G**)= -1361.69519924
Number of imaginary frequencies = 0

1b_c400
MMFF Geometry
C -1.668609 -1.493513 -0.403532
C -2.110139 1.081188 0.341852
N -2.934859 -1.125201 -0.093660
C -0.560581 -0.644478 -0.363887

C -0.800545 0.686902 0.023371
C -3.153044 0.158102 0.276005
C 0.273380 1.594945 0.076056
C 1.578269 1.189947 -0.230819
C 1.810701 -0.134364 -0.609174
C 0.750145 -1.046742 -0.690028
C 3.201011 -0.547431 -0.908116
C 4.358788 0.296683 -0.317287
C 3.952278 1.769829 -0.270679
C 2.669624 2.159820 -0.226924
O 0.949949 -2.351016 -1.073332
O 3.374778 -1.596934 -1.530598
O 5.502655 0.164444 -1.159142
C 4.664009 -0.232893 1.097363
C 5.020279 -1.706237 1.088514
C 4.365740 -2.578523 2.125193
O 5.834684 -2.160388 0.285012
O 4.905827 2.769500 -0.426565
C 6.013067 2.713433 0.463510
C -4.559012 0.520970 0.619250
C -4.753486 1.673021 1.567919
C -5.575801 -0.201248 0.098245
C -7.063650 0.002332 0.289903
C -7.745646 0.102784 -1.088772
C -9.228104 0.441280 -1.008640
C -7.622297 -1.146168 1.132499
H -1.564886 -2.536087 -0.696651
H -2.306604 2.110086 0.625118
H 0.085014 2.630588 0.357774
H 2.421423 3.217787 -0.239969
H 1.858761 -2.435236 -1.444896
H 5.711051 -0.794161 -1.201729
H 3.801667 -0.087739 1.759356
H 5.519929 0.275228 1.552490
H 4.710028 -3.609906 2.009712
H 4.633894 -2.224278 3.123438
H 3.281223 -2.552622 1.992575
H 5.676898 2.639261 1.502757
H 6.690463 1.893249 0.213111
H 6.572947 3.647719 0.359030
H -4.638377 2.627299 1.043527
H -5.735741 1.669218 2.047230
H -4.026800 1.632446 2.387548
H -5.329704 -1.038682 -0.558572
H -7.259714 0.938699 0.821472
H -7.249821 0.882585 -1.681564
H -7.625916 -0.836497 -1.643731
H -9.391660 1.358958 -0.435021
H -9.632068 0.594748 -2.014598
H -9.800295 -0.366524 -0.543468
H -8.674293 -0.978256 1.383349
H -7.076018 -1.240115 2.078117
H -7.548795 -2.104037 0.605272
SCF Energy (B3LYP/6-31G**)= -1361.70074198
Number of imaginary frequencies = 0

1b_c401
MMFF Geometry
C 1.737924 2.055724 -0.412772
C 2.249574 -0.608401 -0.575638
N 3.032935 1.660429 -0.466483
C 0.636436 1.193669 -0.437901
C 0.914245 -0.181985 -0.522012
C 3.274899 0.333607 -0.549718
C -0.151114 -1.098883 -0.548898
C -1.486330 -0.669614 -0.496915
C -1.762167 0.699034 -0.408255
C -0.705215 1.626414 -0.382271
C -3.175019 1.157346 -0.361457
C -4.315763 0.111297 -0.413374
C -3.843967 -1.325749 -0.504829
C -2.550754 -1.669980 -0.521480
O -0.928318 2.982299 -0.310170
O -3.394562 2.372779 -0.343588
O -5.061168 0.403967 -1.614760
C -5.274129 0.354382 0.760268
C -4.662981 0.044933 2.109623
C -5.626098 -0.193187 3.242597
O -3.446992 0.034120 2.297633
O -4.789071 -2.339988 -0.404003
C -5.635228 -2.435694 -1.548597
C 4.700928 -0.071548 -0.607594
C 5.069216 -1.063486 -1.670983
C 5.543633 0.459512 0.304441
C 7.027911 0.281264 0.553700
C 7.274918 -0.490749 1.867711
C 6.807557 -1.941303 1.859979
C 7.882712 -0.268006 -0.589149
H 1.601646 3.133006 -0.345443
H 2.487515 -1.666588 -0.621549
H 0.068094 -2.164658 -0.607687
H -2.272072 -2.720195 -0.518812
H -1.899483 3.136838 -0.262615
H -4.427139 0.418112 -2.352706
H -6.176840 -0.257193 0.638154
H -5.614214 1.397303 0.779379
H -5.070832 -0.407855 4.159872
H -6.236498 0.699782 3.397445
H -6.262253 -1.050458 3.008906
H -6.444247 -1.702216 -1.490214
H -5.071944 -2.321456 -2.481219
H -6.088482 -3.431495 -1.545992
H 4.222851 -1.326557 -2.314715
H 5.831416 -0.653816 -2.339852
H 5.434656 -1.992928 -1.223075
H 5.103799 1.160966 1.020644
H 7.407183 1.299878 0.724333
H 8.347121 -0.470201 2.100308
H 6.774066 0.030147 2.694182
H 7.330815 -2.528954 1.100503
H 7.012256 -2.402028 2.832071
H 5.731573 -2.017787 1.678130
H 8.944630 -0.227949 -0.320543
H 7.758128 0.335020 -1.494750
H 7.651477 -1.308726 -0.829131
SCF Energy (B3LYP/6-31G**)= -1361.68980569
Number of imaginary frequencies = 0

1b_c402
MMFF Geometry
C -1.450437 -2.379544 -0.404614
C -2.362560 0.119190 0.155556
N -2.775656 -2.223985 -0.170225
C -0.508639 -1.346318 -0.377660
C -0.991963 -0.057166 -0.084928
C -3.220180 -0.977593 0.098251
C -0.093967 1.025411 -0.045122
C 1.275643 0.840291 -0.277341
C 1.751287 -0.442889 -0.556400
C 0.866810 -1.529467 -0.623781
C 3.204305 -0.624052 -0.778432
C 4.170426 0.447780 -0.217539
C 3.512227 1.830897 -0.289725
C 2.176727 1.993544 -0.292983
O 1.303829 -2.797713 -0.922328
O 3.572036 -1.657455 -1.340379
O 5.368251 0.473433 -0.993290
C 4.503771 0.110116 1.244639
C 5.736005 -0.750451 1.449312
C 5.804976 -2.095233 0.776675
O 6.631697 -0.386110 2.216304
O 4.436595 2.858733 -0.349860
C 3.969449 4.203378 -0.339960
C -4.675623 -0.854453 0.350904
C -5.254262 -1.958659 1.194567
C -5.380981 0.168556 -0.177589
C -6.870981 0.415800 -0.058924
C -7.109915 1.668170 0.803636
C -8.581379 1.936671 1.088024
C -7.471470 0.551028 -1.461682
H -1.151609 -3.402758 -0.622884
H -2.756244 1.101199 0.399200

H -0.474910 2.023862 0.167802
H   1.711583 2.972674 -0.330265
H   2.237884 -2.744876 -1.230672
H   5.345253 1.274946 -1.546138
H   3.665053 -0.378932 1.755101
H   4.698323 1.036674 1.803137
H   6.605547 -2.686318 1.231587
H   4.864091 -2.631117 0.923424
H   6.022846 -1.979112 -0.286080
H   3.364084 4.412102 -1.227731
H   3.409917 4.416104 0.576595
H   4.842104 4.862638 -0.363986
H -4.583818 -2.205407 2.026250
H -6.209466 -1.686346 1.650726
H -5.407819 -2.861610 0.594337
H -4.861783 0.901970 -0.795038
H -7.366410 -0.435272 0.418906
H -6.594063 1.549082 1.765418
H -6.673667 2.552497 0.321759
H -9.059850 1.063264 1.542087
H -8.681328 2.776668 1.783075
H -9.126065 2.197076 0.175947
H -8.563565 0.611181 -1.420213
H -7.218666 -0.317142 -2.081217
H -7.102701 1.447704 -1.972427
SCF Energy (B3LYP/6-31G**)= -1361.69882943
Number of imaginary frequencies = 0

1b_c403
MMFF Geometry
C -1.521207 -2.220888 -0.612102
C -2.374292 0.357587 -0.726353
N -2.856552 -1.992307 -0.617406
C -0.540998 -1.224912 -0.655941
C -0.994429 0.106885 -0.716296
C -3.270579 -0.708057 -0.667302
C -0.057634 1.155166 -0.773435
C   1.318148 0.895977 -0.749515
C   1.762617 -0.426685 -0.682973
C   0.844021 -1.484708 -0.650117
C   3.221397 -0.677325 -0.636287
C   4.146149 0.468681 -0.152985
C   3.578214 1.814897 -0.603175
C   2.275298 1.992139 -0.868137
O   1.255951 -2.794636 -0.600220
O   3.624539 -1.814010 -0.890388
O   5.436290 0.298245 -0.737044
C   4.234346 0.397326 1.383890
C   4.746298 -0.946715 1.862669
C   4.010746 -1.596311 3.003291
O   5.741066 -1.466880 1.357716
O   4.427944 2.873919 -0.901993
C   5.345445 3.239034 0.120868
C -4.739810 -0.501406 -0.680750
C -5.491499 -1.478956 -1.544767
C -5.297520 0.470773 0.072897
C -6.764570 0.823172 0.193249
C -7.376224 0.292099 1.504670
C -6.790482 0.869320 2.787621
C -6.952373 2.335339 0.035827
H -1.247454 -3.273101 -0.570197
H -2.747242 1.375074 -0.794192
H -0.409738 2.184269 -0.839647
H   1.918691 2.953341 -1.228705
H   2.222402 -2.829126 -0.789740
H   5.752941 -0.590369 -0.464500
H   3.251465 0.576135 1.836565
H   4.927022 1.139806 1.792369
H   4.482053 -2.551810 3.249286
H   4.048011 -0.945708 3.880340
H   2.973637 -1.780251 2.711731
H   4.828459 3.396364 1.072985
H   6.144285 2.501032 0.228024
H   5.808457 4.187143 -0.168926
H -5.432752 -2.490256 -1.127638
H -6.551243 -1.239256 -1.651119
H -5.070964 -1.496293 -2.556776
H -4.646428 1.077083 0.703023
H -7.329664 0.364753 -0.624569
H -8.455044 0.493090 1.497870
H -7.268327 -0.799867 1.534157
H -6.938027 1.951486 2.845667
H -7.287051 0.422480 3.655385
H -5.720832 0.657850 2.874790
H -8.007815 2.607971 0.142097
H -6.622711 2.663724 -0.956581
H -6.378945 2.902898 0.776480
SCF Energy (B3LYP/6-31G**)= -1361.69927537
Number of imaginary frequencies = 0

1b_c404
MMFF Geometry
C -1.517034 -2.305407 -0.562945
C -2.349587 0.282216 -0.583085
N -2.850500 -2.066308 -0.556121
C -0.528990 -1.316047 -0.577605
C -0.971675 0.020025 -0.590697
C -3.253819 -0.777709 -0.557284
C -0.025953 1.061489 -0.620476
C   1.349367 0.792271 -0.617103
C   1.782996 -0.535085 -0.589092
C   0.854117 -1.586103 -0.587073
C   3.239724 -0.801047 -0.564446
C   4.181749 0.324970 -0.073017
C   3.620899 1.690641 -0.487608
C   2.309776 1.892678 -0.711608
O   1.252950 -2.901202 -0.590062
O   3.619442 -1.932245 -0.872867
O   5.473061 0.166681 -0.660105
C   4.293773 0.255974 1.458568
C   5.434430 -0.590805 1.990292
C   5.515075 -2.039079 1.588260
O   6.237722 -0.118902 2.799807
O   4.603106 2.658762 -0.599473
C   4.217957 3.995616 -0.900933
C -4.720601 -0.562639 -0.554132
C -5.485497 -1.496432 -1.453445
C -5.273359 0.386772 0.231047
C -6.745313 0.707240 0.392457
C -7.132084 0.589409 1.879567
C -8.623063 0.765189 2.133884
C -7.016780 2.104151 -0.170289
H -1.251493 -3.360561 -0.556368
H -2.715207 1.304092 -0.611460
H -0.373507 2.093870 -0.647945
H   1.911589 2.861943 -0.991652
H   2.221939 -2.936505 -0.763635
H   5.571335 0.850303 -1.346831
H   3.366505 -0.101439 1.922909
H   4.464907 1.263792 1.862669
H   6.210589 -2.560515 2.252793
H   4.533231 -2.507223 1.692154
H   5.881417 -2.130654 0.564634
H   3.751276 4.053178 -1.889400
H   3.553053 4.394575 -0.128188
H   5.122727 4.610274 -0.918650
H -5.596329 -2.479112 -0.983030
H -6.482471 -1.124217 -1.701943
H -4.966546 -1.627126 -2.410370
H -4.621858 0.989219 0.864684
H -7.359983 -0.004984 -0.166517
H -6.837365 -0.400206 2.252553
H -6.580938 1.326569 2.477379
H -9.210158 0.065365 1.530980
H -8.848136 0.574569 3.188253
H -8.952586 1.782792 1.905324
H -8.087762 2.329614 -0.171177
H -6.669261 2.183198 -1.206841
H -6.508339 2.878870 0.414682
SCF Energy (B3LYP/6-31G**)= -1361.69889615
Number of imaginary frequencies = 0

1b_c405
MMFF Geometry
C -1.469728 -2.188773 -0.549591
C -2.337998 0.367062 -0.218344
N -2.802880 -1.983068 -0.419343
C -0.499935 -1.181265 -0.527913
C -0.961632 0.139254 -0.357692
C -3.220316 -0.710871 -0.246844
C -0.038122 1.200947 -0.343977
C 1.334871 0.962647 -0.473764
C 1.788022 -0.352966 -0.623491
C 0.881433 -1.419132 -0.673077
C 3.246700 -0.572672 -0.753605
C 4.188229 0.504247 -0.153812
C 3.594090 1.885165 -0.411144
C 2.274543 2.081996 -0.550264
O 1.297503 -2.713265 -0.869432
O 3.646586 -1.605866 -1.291633
O 5.470116 0.423965 -0.782910
C 4.394846 0.259482 1.345520
C 4.552490 -1.208334 1.673735
C 5.884734 -1.855625 1.404215
O 3.599999 -1.849646 2.119718
O 4.443857 2.951458 -0.670262
C 5.267452 3.311487 0.435498
C -4.684533 -0.528528 -0.110134
C -5.491630 -1.257292 -1.145830
C -5.174128 0.235228 0.890205
C -6.596705 0.538720 1.319369
C -7.030631 1.956948 0.898510
C -7.135562 2.184583 -0.603908
C -7.662929 -0.514339 1.007633
H -1.187620 -3.231799 -0.677805
H -2.722023 1.375871 -0.101804
H -0.399728 2.222918 -0.236404
H 1.884823 3.071061 -0.774655
H 2.265045 -2.713245 -1.045397
H 5.334640 0.646417 -1.722430
H 3.539357 0.624069 1.929138
H 5.281574 0.775732 1.729913
H 5.920947 -2.831591 1.896500
H 6.024061 -1.993567 0.330291
H 6.686856 -1.235721 1.812398
H 4.691772 3.356316 1.366264
H 6.109538 2.621218 0.534147
H 5.674171 4.308067 0.239673
H -5.825982 -2.227233 -0.764698
H -6.358502 -0.673035 -1.463357
H -4.912054 -1.434700 -2.059615
H -4.451515 0.698700 1.565619
H -6.549133 0.556061 2.419097
H -8.002313 2.184183 1.354892
H -6.321896 2.687769 1.309379
H -7.877146 1.523799 -1.061156
H -7.446539 3.215767 -0.801778
H -6.174878 2.028874 -1.103253
H -8.572023 -0.307556 1.584311
H -7.321151 -1.516348 1.288434
H -7.955968 -0.531536 -0.043922
SCF Energy (B3LYP/6-31G**)= -1361.68441995
Number of imaginary frequencies = 0

1b_c406
MMFF Geometry
C -1.608880 -1.614508 -1.171820
C -2.279337 0.863732 -0.275611
N -2.925112 -1.305626 -1.083125
C -0.561374 -0.750769 -0.836472
C -0.920710 0.529481 -0.372690
C -3.246250 -0.070211 -0.639690
C 0.086919 1.446830 -0.022078
C 1.442157 1.104977 -0.116456
C 1.791673 -0.173823 -0.564365
C 0.802146 -1.092791 -0.939574
C 3.227635 -0.510915 -0.667484
C 4.248010 0.325984 0.129723
C 3.762866 1.754540 0.286457
C 2.467570 2.097070 0.210165
O 1.116645 -2.341412 -1.418808
O 3.586885 -1.448793 -1.385843
O 5.464687 0.332284 -0.637344
C 4.548743 -0.264279 1.509331
C 4.623164 -1.771876 1.492506
C 5.952768 -2.406623 1.185511
O 3.612634 -2.449184 1.684707
O 4.706146 2.685438 0.694892
C 5.165390 3.470981 -0.405722
C -4.694227 0.241105 -0.555069
C -5.130942 1.546908 -1.150508
C -5.495097 -0.653264 0.063514
C -6.985111 -0.674281 0.340122
C -7.266150 -0.493866 1.847458
C -6.886734 0.868796 2.414715
C -7.881571 0.227019 -0.509894
H -1.408587 -2.620460 -1.534754
H -2.581356 1.838640 0.094405
H -0.194552 2.440045 0.326316
H 2.157174 3.118205 0.411660
H 2.093058 -2.415633 -1.502251
H 5.411334 -0.425740 -1.254436
H 3.765867 -0.002537 2.233738
H 5.493054 0.120703 1.913482
H 5.889054 -3.485710 1.351686
H 6.221930 -2.223476 0.143476
H 6.718404 -1.999817 1.850722
H 5.598070 2.847466 -1.194218
H 4.353717 4.083675 -0.812306
H 5.945534 4.142772 -0.035927
H -4.307286 2.091280 -1.625049
H -5.871853 1.387891 -1.939147
H -5.549110 2.202639 -0.380328
H -5.007729 -1.552462 0.453834
H -7.301007 -1.698835 0.093057
H -8.333053 -0.666701 2.037156
H -6.727532 -1.266210 2.411918
H -7.451075 1.674931 1.937639
H -7.109994 0.899582 3.486321
H -5.818945 1.073348 2.293694
H -8.936716 0.022404 -0.294889
H -7.728776 0.035108 -1.577180
H -7.715775 1.290312 -0.319985
SCF Energy (B3LYP/6-31G**)= -1361.68876141
Number of imaginary frequencies = 0

1b_c407
MMFF Geometry
C -1.721999 -2.468639 -0.151099
C -2.453774 0.140293 0.073728
N -3.031274 -2.191103 0.059080
C -0.710809 -1.509150 -0.259758
C -1.100846 -0.161068 -0.140160
C -3.385611 -0.892233 0.160535
C -0.130298 0.853285 -0.243799
C 1.220400 0.543866 -0.448028
C 1.600143 -0.796989 -0.551585
C 0.645576 -1.819646 -0.479082
C 3.031939 -1.098597 -0.758283
C 4.060725 -0.039543 -0.295998
C 3.527333 1.364438 -0.575476
C 2.204758 1.616907 -0.620825
O 0.994410 -3.141285 -0.614056
O 3.332910 -2.181368 -1.261869
O 5.260112 -0.262548 -1.039795
C 4.324569 -0.264441 1.202044
C 5.757095 -0.082369 1.660542
C 6.431006 1.244108 1.434872
O 6.334804 -0.980591 2.279064
O 4.517616 2.310404 -0.753604
C 4.146214 3.649991 -1.056390
C -4.826418 -0.634498 0.394468
C -5.469607 -1.568853 1.383799
C -5.463188 0.352673 -0.271396

C -6.927613 0.726007 -0.186048
C -7.114430 2.148233 0.374019
C -6.646728 2.291588 1.817049
C -7.554807 0.624915 -1.580396
H -1.496442 -3.530063 -0.231681
H -2.777075 1.170677 0.185581
H -0.439808 1.895091 -0.166524
H 1.809619 2.611473 -0.797552
H 1.928123 -3.194845 -0.922459
H 5.015244 -0.320267 -1.980015
H 4.057649 -1.296527 1.470821
H 3.686045 0.370283 1.829323
H 6.743167 1.341779 0.394107
H 5.755446 2.055716 1.715579
H 7.319391 1.310328 2.070283
H 3.586226 3.697992 -1.995781
H 3.573613 4.090973 -0.234266
H 5.063350 4.233490 -1.179435
H -4.814858 -1.729805 2.248350
H -6.408377 -1.180973 1.786590
H -5.674669 -2.539308 0.919661
H -4.899913 0.950792 -0.988126
H -7.475617 0.030737 0.457358
H -6.581418 2.877714 -0.248935
H -8.177676 2.415699 0.330436
H -5.567050 2.139003 1.907815
H -6.871995 3.297426 2.185896
H -7.154224 1.571582 2.466869
H -8.625626 0.852358 -1.540212
H -7.445202 -0.386645 -1.987315
H -7.089602 1.324471 -2.284250
SCF Energy (B3LYP/6-31G**)= -1361.69682372
Number of imaginary frequencies = 0

1b_c408
MMFF Geometry
C -1.780446 -1.240452 -0.709604
C -2.122870 1.337586 0.075491
N -3.040957 -0.807104 -0.468103
C -0.631242 -0.457828 -0.579264
C -0.819869 0.875295 -0.170174
C -3.209747 0.478294 -0.080287
C 0.298354 1.716740 -0.021282
C 1.595280 1.243949 -0.256524
C 1.776077 -0.081367 -0.658808
C 0.672895 -0.927663 -0.833503
C 3.157136 -0.567471 -0.880823
C 4.320002 0.191296 -0.192284
C 3.995980 1.684097 -0.127882
C 2.737528 2.147574 -0.154235
O 0.822852 -2.231373 -1.240713
O 3.311093 -1.608582 -1.522313
O 5.507181 0.013917 -0.962853
C 4.501130 -0.391902 1.222611
C 4.772638 -1.882920 1.192840
C 4.004353 -2.741496 2.160622
O 5.609724 -2.363418 0.428949
O 5.013145 2.629633 -0.192924
C 6.054490 2.485633 0.764284
C -4.614276 0.922119 0.155459
C -4.903199 2.390432 -0.006509
C -5.558431 0.005171 0.463254
C -7.022533 0.243988 0.758723
C -7.914217 -0.091493 -0.452386
C -7.891727 -1.547257 -0.902534
C -7.437267 -0.528110 2.015424
H -1.717198 -2.280030 -1.024102
H -2.276432 2.361867 0.399054
H 0.151326 2.753846 0.279041
H 2.551415 3.218278 -0.151380
H 1.747456 -2.359038 -1.556793
H 5.662754 -0.953882 -1.020645
H 3.606893 -0.213538 1.832092
H 5.352931 0.053030 1.746441
H 4.295971 -3.788030 2.036745
H 4.227742 -2.429278 3.183611
H 2.933807 -2.648542 1.961302
H 5.647068 2.404560 1.777193
H 6.699018 1.633764 0.533687
H 6.672365 3.387766 0.723577
H -4.390960 2.795645 -0.886748
H -5.965066 2.599657 -0.161789
H -4.576493 2.947276 0.877821
H -5.257490 -1.042601 0.524639
H -7.184902 1.302332 0.992795
H -8.950495 0.180625 -0.215208
H -7.618656 0.538628 -1.301163
H -8.253418 -2.216558 -0.116751
H -8.545251 -1.676585 -1.771672
H -6.886507 -1.864287 -1.195235
H -8.500352 -0.375827 2.230488
H -6.869196 -0.181409 2.886329
H -7.261822 -1.604634 1.916560
SCF Energy (B3LYP/6-31G**)= -1361.69982614
Number of imaginary frequencies = 0

1b_c409
MMFF Geometry
C -1.864674 -2.141201 0.027457
C -2.438677 0.502662 0.286036
N -3.157426 -1.788265 0.227414
C -0.794999 -1.243710 -0.054117
C -1.104052 0.121593 0.083290
C -3.432784 -0.471857 0.345044
C -0.070636 1.073321 0.012422
C 1.263679 0.687637 -0.185216
C 1.566162 -0.669418 -0.315844
C 0.544504 -1.631375 -0.261774
C 2.974892 -1.068441 -0.522840
C 4.096476 -0.040937 -0.282076
C 3.608908 1.398461 -0.327079
C 2.302394 1.714855 -0.279302
O 0.804573 -2.974017 -0.402316
O 3.219055 -2.232419 -0.856119
O 5.000623 -0.244289 -1.379005
C 4.762826 -0.356437 1.066788
C 6.268538 -0.457588 0.990796
C 7.066392 0.816034 0.916293
O 6.821778 -1.558855 0.964718
O 4.638962 2.320067 -0.389402
C 4.322467 3.687456 -0.620574
C -4.858929 -0.129510 0.564803
C -5.552356 -1.018647 1.559257
C -5.426077 0.885940 -0.121972
C -6.855649 1.388019 -0.112495
C -7.942626 0.334634 -0.401475
C -7.736823 -0.399990 -1.720254
C -7.163977 2.174378 1.164299
H -1.703132 -3.212947 -0.068440
H -2.699824 1.549144 0.410024
H -0.317130 2.130103 0.112169
H 1.961985 2.744460 -0.296852
H 1.750236 -3.090754 -0.650330
H 5.169685 -1.207643 -1.428867
H 4.407164 -1.316313 1.465848
H 4.499854 0.374344 1.842030
H 8.096629 0.616430 1.224800
H 7.067459 1.195792 -0.107121
H 6.647103 1.560404 1.597755
H 3.761593 3.809128 -1.552887
H 3.772470 4.108101 0.227098
H 5.262779 4.238122 -0.717523
H -4.909670 -1.207692 2.427171
H -6.467973 -0.577027 1.955655
H -5.808840 -1.980745 1.103263
H -4.805938 1.429612 -0.836375
H -6.916639 2.115778 -0.935414
H -8.017130 -0.397377 0.408304
H -8.916923 0.838625 -0.442595
H -6.828627 -1.009750 -1.704863
H -8.582042 -1.068840 -1.912216
H -7.667659 0.304356 -2.555313

H -8.137343 2.670706 1.083175
H -6.411154 2.951236 1.339104
H -7.194744 1.531441 2.048715
SCF Energy (B3LYP/6-31G**)= -1361.69769613
Number of imaginary frequencies = 0

1b_c410
MMFF Geometry
C -1.799819 -2.387434 -0.222673
C -2.518320 0.233013 -0.166750
N -3.120367 -2.092093 -0.153744
C -0.770676 -1.441147 -0.265735
C -1.154873 -0.088329 -0.238500
C -3.465788 -0.787571 -0.117968
C -0.166032 0.910812 -0.289741
C 1.197010 0.585111 -0.354701
C 1.573949 -0.759433 -0.372485
C 0.598491 -1.769129 -0.339872
C 3.012531 -1.094947 -0.439786
C 4.059576 0.003251 -0.176422
C 3.509167 1.409344 -0.354135
C 2.189579 1.658327 -0.432024
O 0.933109 -3.102107 -0.375591
O 3.339147 -2.262282 -0.676812
O 5.061324 -0.218231 -1.181182
C 4.624657 -0.193908 1.239470
C 6.134552 -0.219058 1.292148
C 6.873642 1.088385 1.199578
O 6.740375 -1.288789 1.382687
O 4.495532 2.378758 -0.393733
C 4.134482 3.710404 -0.739769
C -4.920616 -0.507180 -0.049966
C -5.747596 -1.359391 -0.972125
C -5.390491 0.428455 0.803297
C -6.814410 0.865512 1.080785
C -7.452434 1.688844 -0.053583
C -6.663913 2.944270 -0.406212
C -7.724134 -0.286674 1.517901
H -1.580139 -3.452903 -0.244019
H -2.840439 1.269849 -0.163356
H -0.470303 1.957137 -0.278811
H 1.802926 2.665414 -0.544065
H 1.901025 -3.183126 -0.536779
H 5.279643 -1.172562 -1.154695
H 4.283350 -1.145442 1.669819
H 4.262605 0.567459 1.941917
H 7.882641 0.963227 1.602893
H 6.942626 1.404475 0.157023
H 6.363129 1.849538 1.794812
H 3.648409 3.743959 -1.720176
H 3.496009 4.153115 0.031169
H 5.052204 4.303127 -0.797066
H -5.853388 -2.372648 -0.570173
H -6.748333 -0.958413 -1.138603
H -5.280438 -1.426739 -1.961676
H -4.671284 0.950293 1.436516
H -6.751627 1.532192 1.953765
H -7.576067 1.083107 -0.957369
H -8.460941 1.994095 0.253093
H -5.680571 2.699219 -0.818945
H -7.204231 3.526946 -1.159281
H -6.522289 3.579054 0.474234
H -8.661986 0.106092 1.926741
H -7.249899 -0.888449 2.301042
H -7.987162 -0.951324 0.690860
SCF Energy (B3LYP/6-31G**)= -1361.69782417
Number of imaginary frequencies = 0

1b_c411
MMFF Geometry
C -1.808790 -2.322469 -0.301153
C -2.417278 0.307022 -0.629388
N -3.116744 -1.970132 -0.323594
C -0.740231 -1.430554 -0.433370
C -1.066961 -0.071424 -0.605706
C -3.408325 -0.660844 -0.477955
C -0.036473 0.874979 -0.760686
C 1.309963 0.490656 -0.725205
C 1.624965 -0.857624 -0.536159
C 0.613680 -1.818874 -0.411303
C 3.052528 -1.237228 -0.493839
C 4.072543 -0.144088 -0.095840
C 3.656574 1.200345 -0.690213
C 2.365965 1.481759 -0.955002
O 0.902830 -3.153562 -0.264145
O 3.353651 -2.403213 -0.751490
O 5.341170 -0.538209 -0.621905
C 4.134617 -0.097067 1.439810
C 5.506573 0.121358 2.044419
C 6.275682 1.361367 1.676344
O 5.951997 -0.664796 2.885060
O 4.712126 2.065323 -0.902040
C 4.456097 3.336500 -1.487437
C -4.851125 -0.321409 -0.500126
C -5.703178 -1.286700 -1.279683
C -5.312313 0.757060 0.168793
C -6.748650 1.217828 0.294376
C -7.178735 1.326614 1.770226
C -7.185102 -0.015152 2.492521
C -6.903156 2.573707 -0.399264
H -1.634217 -3.388249 -0.168411
H -2.694962 1.345851 -0.779064
H -0.295098 1.922428 -0.912646
H 2.051507 2.438536 -1.357779
H 1.863840 -3.291874 -0.428864
H 5.213312 -0.757251 -1.561563
H 3.779798 -1.052996 1.850887
H 3.458769 0.662190 1.853113
H 6.720163 1.255191 0.685720
H 5.616307 2.231779 1.714980
H 7.080164 1.515748 2.401837
H 4.021149 3.226988 -2.486020
H 3.810735 3.940182 -0.841457
H 5.412291 3.857840 -1.590583
H -5.889413 -2.194000 -0.695373
H -6.669014 -0.863986 -1.567025
H -5.209643 -1.572770 -2.215971
H -4.605631 1.370141 0.728628
H -7.432367 0.517999 -0.195923
H -6.522978 2.021216 2.310376
H -8.190792 1.747582 1.820138
H -6.178882 -0.439058 2.563856
H -7.566401 0.109217 3.511155
H -7.828088 -0.735047 1.976193
H -7.942084 2.917474 -0.350549
H -6.624906 2.507062 -1.457081
H -6.273749 3.339817 0.067794
SCF Energy (B3LYP/6-31G**)= -1361.69691939
Number of imaginary frequencies = 0

1b_c412
MMFF Geometry
C -1.512507 -2.429205 -0.396354
C -2.438432 0.060084 0.182803
N -2.837081 -2.284413 -0.151612
C -0.577992 -1.389324 -0.371119
C -1.068424 -0.105157 -0.068483
C -3.288558 -1.042601 0.126142
C -0.178025 0.983704 -0.029783
C 1.191148 0.809665 -0.272851
C 1.673990 -0.468647 -0.561739
C 0.796911 -1.561273 -0.628113
C 3.126622 -0.638170 -0.795202
C 4.089044 0.437866 -0.236002
C 3.420374 1.816512 -0.296530
C 2.083747 1.969485 -0.289289
O 1.240886 -2.824830 -0.936124
O 3.497696 -1.666098 -1.364938
O 5.280970 0.476022 -1.020278
C 4.435461 0.095432 1.222023
C 5.675328 -0.757177 1.413475
C 5.749100 -2.098092 0.733666

O 6.573927 -0.390138 2.175767
O 4.336838 2.851308 -0.358254
C 3.860086 4.192465 -0.338286
C -4.742729 -0.931094 0.390066
C -5.309164 -2.043829 1.230889
C -5.459321 0.092543 -0.121860
C -6.949399 0.321984 0.014445
C -7.266052 1.417256 1.051139
C -6.734030 2.807177 0.723220
C -7.561652 0.626505 -1.356445
H -1.207898 -3.449146 -0.621834
H -2.837287 1.038036 0.434332
H -0.564614 1.978302 0.190864
H 1.611290 2.945387 -0.318325
H 2.172263 -2.763700 -1.250970
H 5.248142 1.280037 -1.568980
H 3.604034 -0.402188 1.736114
H 4.627389 1.020617 1.783699
H 6.557208 -2.685591 1.179813
H 4.813201 -2.641502 0.884559
H 5.958380 -1.975158 -0.330050
H 3.246778 4.401155 -1.220596
H 3.305728 4.396620 0.583348
H 4.727762 4.858147 -0.365364
H -4.634458 -2.287660 2.059925
H -6.265954 -1.780494 1.689742
H -5.456801 -2.945741 0.627650
H -4.949205 0.833749 -0.736852
H -7.436287 -0.596257 0.362001
H -8.354425 1.484250 1.174144
H -6.865494 1.113842 2.027037
H -7.162508 3.192558 -0.206360
H -7.002206 3.504963 1.523468
H -5.643841 2.811762 0.632932
H -8.637416 0.812085 -1.267746
H -7.425902 -0.222146 -2.036681
H -7.104010 1.502210 -1.828834
SCF Energy (B3LYP/6-31G**)= -1361.69783969
Number of imaginary frequencies = 0

1b_c413
MMFF Geometry
C -1.714591 -2.105990 -0.200572
C -2.268197 0.507505 0.295928
N -3.005302 -1.762232 0.026045
C -0.636956 -1.214040 -0.197746
C -0.936220 0.134999 0.062521
C -3.270523 -0.459592 0.262494
C 0.104946 1.079244 0.085817
C 1.435714 0.703067 -0.148979
C 1.732406 -0.637916 -0.409186
C 0.701466 -1.592899 -0.433299
C 3.142866 -1.035264 -0.644692
C 4.255996 0.030336 -0.640084
C 3.761818 1.429771 -0.356476
C 2.480354 1.720854 -0.102364
O 0.947298 -2.923509 -0.679307
O 3.393635 -2.223042 -0.876226
O 4.727529 -0.002228 -2.006652
C 5.413295 -0.422040 0.262632
C 5.190070 -0.201947 1.744350
C 4.137924 -1.027794 2.436149
O 5.858290 0.616802 2.376241
O 4.697764 2.444610 -0.212385
C 5.362154 2.771535 -1.431638
C -4.694696 -0.126715 0.508129
C -5.399911 -1.097910 1.413484
C -5.250177 0.953391 -0.082859
C -6.675128 1.465863 -0.030951
C -7.770536 0.454189 -0.419859
C -7.566392 -0.156543 -1.800633
C -6.981146 2.132946 1.312545
H -1.561916 -3.166014 -0.392301
H -2.520247 1.540639 0.515195
H -0.129977 2.123852 0.290290
H 2.200932 2.741017 0.148211
H 1.907325 -3.044041 -0.854567
H 4.967729 -0.928546 -2.201585
H 6.331820 0.111840 -0.016168
H 5.654337 -1.481873 0.112427
H 4.348915 -1.057381 3.509119
H 3.153269 -0.584396 2.280589
H 4.158596 -2.053604 2.060091
H 6.164734 2.057579 -1.636800
H 4.662562 2.824738 -2.272989
H 5.818813 3.758114 -1.308193
H -4.762487 -1.372478 2.262225
H -6.313274 -0.686725 1.846156
H -5.662879 -2.011192 0.869323
H -4.622161 1.556289 -0.740688
H -6.726364 2.267519 -0.782795
H -7.854798 -0.349209 0.318099
H -8.740041 0.968975 -0.416807
H -6.663849 -0.773620 -1.839334
H -8.416925 -0.796657 -2.056469
H -7.487630 0.621710 -2.566354
H -7.949660 2.643771 1.275097
H -6.221979 2.883089 1.561056
H -7.021078 1.410864 2.133223
SCF Energy (B3LYP/6-31G**)= -1361.69216454
Number of imaginary frequencies = 0

1b_c414
MMFF Geometry
C 2.013047 1.942484 -0.098552
C 2.426625 -0.727020 -0.371857
N 3.291379 1.496647 -0.153872
C 0.879989 1.127768 -0.169443
C 1.106668 -0.251661 -0.309380
C 3.493773 0.166324 -0.292027
C 0.006614 -1.124254 -0.383420
C -1.311088 -0.645796 -0.319528
C -1.535727 0.726558 -0.179323
C -0.444545 1.610271 -0.107816
C -2.929627 1.232310 -0.103872
C -4.111252 0.239119 -0.121738
C -3.691464 -1.196937 -0.347520
C -2.415173 -1.595934 -0.403654
O -0.615842 2.968399 0.027593
O -3.103228 2.453356 -0.020498
O -4.888134 0.661697 -1.247857
C -4.855146 0.413740 1.214513
C -6.297537 -0.030521 1.161670
C -6.707692 -1.174866 2.049433
O -7.118517 0.568128 0.464617
O -4.683594 -2.169182 -0.363972
C -5.398009 -2.188730 -1.602349
C 4.913374 -0.286359 -0.367917
C 5.177150 -1.526046 -1.175982
C 5.867954 0.442277 0.252796
C 7.360975 0.219895 0.370619
C 7.804367 -1.165286 0.880396
C 7.191418 -1.539556 2.224052
C 8.083594 0.595922 -0.925479
H 1.920110 3.021085 0.009307
H 2.611078 -1.791988 -0.468230
H 0.184639 -2.193736 -0.494269
H -2.183911 -2.651555 -0.517471
H -1.579645 3.168425 0.006610
H -5.437925 1.417147 -0.961623
H -4.890753 1.468366 1.518590
H -4.334125 -0.109058 2.026227
H -7.706337 -1.518690 1.766321
H -6.011474 -2.008814 1.939328
H -6.722267 -0.838431 3.088914
H -6.193826 -1.439169 -1.598097
H -4.733889 -2.045245 -2.461897
H -5.868804 -3.171771 -1.697831
H 4.570350 -1.536896 -2.088804
H 6.212530 -1.599125 -1.512897
H 4.947109 -2.423412 -0.592303
H 5.546967 1.343495 0.781547

H 7.705586 0.940209 1.127590
H 7.581791 -1.951740 0.153175
H 8.896085 -1.162741 0.995307
H 6.106674 -1.662058 2.152731
H 7.609798 -2.488387 2.575184
H 7.405421 -0.776634 2.979279
H 7.802056 1.602359 -1.255088
H 7.859049 -0.099182 -1.739827
H 9.168855 0.589612 -0.775472
SCF Energy (B3LYP/6-31G**)= -1361.69746687
Number of imaginary frequencies = 0

1b_c415
MMFF Geometry
C -1.638634 -2.353466 -0.310934
C -2.344956 0.268434 -0.211346
N -2.958525 -2.053211 -0.253830
C -0.603856 -1.412141 -0.322027
C -0.982461 -0.058846 -0.272414
C -3.298081 -0.747741 -0.195801
C 0.011341 0.935287 -0.285902
C 1.373043 0.604056 -0.345913
C 1.748927 -0.741475 -0.393027
C 0.765649 -1.745690 -0.381824
C 3.190840 -1.089441 -0.443051
C 4.253285 0.026058 -0.479267
C 3.676257 1.421885 -0.437427
C 2.365099 1.674162 -0.344319
O 1.089046 -3.081743 -0.424771
O 3.512010 -2.281961 -0.488727
O 4.861215 -0.161408 -1.777795
C 5.331080 -0.239674 0.582061
C 4.947951 0.157094 1.992563
C 3.874227 -0.636945 2.688462
O 5.505113 1.093105 2.566628
O 4.550710 2.496223 -0.348879
C 5.318237 2.693636 -1.534940
C -4.752262 -0.461350 -0.140030
C -5.572278 -1.291132 -1.088377
C -5.227825 0.460339 0.725176
C -6.652634 0.900113 0.993575
C -7.272122 1.748517 -0.132560
C -6.472543 3.005987 -0.451379
C -7.573915 -0.255056 1.397343
H -1.424541 -3.419463 -0.350199
H -2.661021 1.307042 -0.189779
H -0.285452 1.983462 -0.247829
H 2.020526 2.701912 -0.263103
H 2.065828 -3.172110 -0.492731
H 5.156023 -1.091457 -1.821074
H 6.249296 0.304677 0.323218
H 5.631994 -1.294708 0.598960
H 3.979064 -0.516225 3.770627
H 2.889320 -0.278564 2.385334
H 3.982280 -1.699165 2.455760
H 6.166648 2.004210 -1.564234
H 4.703912 2.595199 -2.436630
H 5.718353 3.711608 -1.507417
H -5.688120 -2.311375 -0.707413
H -6.568860 -0.881699 -1.259142
H -5.093467 -1.341891 -2.073337
H -4.513731 0.966831 1.376536
H -6.597025 1.549631 1.879876
H -7.387813 1.160832 -1.049228
H -8.282690 2.053527 0.167502
H -5.485500 2.763330 -0.856623
H -7.000347 3.605955 -1.199730
H -6.338414 3.623050 0.442734
H -8.514620 0.135120 1.802088
H -7.112671 -0.874332 2.174578
H -7.830270 -0.902310 0.554560
SCF Energy (B3LYP/6-31G**)= -1361.69229127
Number of imaginary frequencies = 0

1b_c416
MMFF Geometry
C -1.604598 -2.424918 -0.115936
C -2.440417 0.151850 0.115154
N -2.920677 -2.201541 0.115013
C -0.635572 -1.424991 -0.242402
C -1.079627 -0.093987 -0.118771
C -3.327328 -0.917934 0.218789
C -0.154155 0.959197 -0.238686
C 1.204049 0.703787 -0.461501
C 1.640825 -0.619742 -0.574004
C 0.729436 -1.680042 -0.482437
C 3.087232 -0.865926 -0.787876
C 4.078918 0.242074 -0.350745
C 3.463475 1.609934 -0.622115
C 2.138205 1.809059 -0.663847
O 1.130065 -2.987577 -0.614743
O 3.433823 -1.965064 -1.224082
O 5.262637 0.093700 -1.134278
C 4.364502 -0.006257 1.143143
C 5.830406 -0.027515 1.507964
C 6.597946 -1.300975 1.260647
O 6.357759 0.941246 2.058546
O 4.283832 2.657567 -1.016904
C 5.267061 3.027613 -0.052351
C -4.774384 -0.720534 0.472855
C -5.367142 -1.684488 1.465376
C -5.461520 0.242175 -0.178615
C -6.941980 0.546170 -0.073653
C -7.131947 1.909897 0.614609
C -8.590410 2.256747 0.880621
C -7.563487 0.508115 -1.473252
H -1.337045 -3.476267 -0.198924
H -2.803419 1.168749 0.229138
H -0.502413 1.988826 -0.161162
H 1.737260 2.789758 -0.905697
H 2.059330 -3.002068 -0.940173
H 5.065109 0.443063 -2.022516
H 3.947618 -0.963034 1.486108
H 3.870479 0.746010 1.772129
H 7.670492 -1.088481 1.273307
H 6.360338 -2.021147 2.047441
H 6.343754 -1.726408 0.287944
H 4.854232 3.040315 0.961513
H 6.134179 2.367088 -0.121288
H 5.603403 4.040831 -0.291987
H -4.689095 -1.836246 2.313488
H -6.306809 -1.326403 1.893672
H -5.554794 -2.655147 0.994437
H -4.933817 0.871140 -0.896102
H -7.451426 -0.217896 0.521962
H -6.602416 1.906880 1.576324
H -6.680868 2.708102 0.011325
H -9.083892 1.466846 1.455497
H -8.655474 3.185442 1.456795
H -9.144105 2.407234 -0.050591
H -8.652669 0.604908 -1.425033
H -7.345097 -0.442700 -1.972963
H -7.179965 1.316571 -2.105858
SCF Energy (B3LYP/6-31G**)= -1361.69106673
Number of imaginary frequencies = 0

1b_c417
MMFF Geometry
C 2.063442 1.696540 -0.259424
C 2.362710 -0.982971 0.060581
N 3.320789 1.201692 -0.158862
C 0.897297 0.928437 -0.209167
C 1.064871 -0.456632 -0.044205
C 3.466594 -0.133794 0.000191
C -0.070777 -1.283922 0.010188
C -1.366232 -0.754275 -0.092996
C -1.532122 0.624142 -0.253956
C -0.404685 1.461886 -0.315408
C -2.902817 1.185737 -0.355184
C -4.127795 0.255620 -0.223690
C -3.766613 -1.210316 -0.123495
C -2.509105 -1.659218 -0.031658

O -0.517706 2.823228 -0.477154
O -3.022509 2.402481 -0.537526
O -4.837771 0.459882 -1.450250
C -4.916422 0.735391 1.008101
C -6.374693 0.343996 0.977690
C -6.873660 -0.571893 2.063074
O -7.137797 0.812795 0.131425
O -4.801545 -2.126736 0.014138
C -5.464767 -2.380467 -1.226881
C   4.865018 -0.640131 0.123452
C   5.055099 -1.885174 0.943987
C   5.866347 0.050437 -0.466306
C   7.354158 -0.223729 -0.528190
C   8.100927 0.076895 0.784594
C   7.937710 1.516281 1.257461
C   7.694647 -1.619982 -1.058777
H   2.016777 2.776008 -0.386757
H   2.501384 -2.053154 0.174089
H   0.061505 -2.358612 0.133946
H -2.322904 -2.722975 0.088859
H -1.469547 3.049969 -0.586483
H -5.362479 1.278830 -1.355870
H -4.914948 1.830985 1.083185
H -4.453898 0.376311 1.935890
H -7.874864 -0.930366 1.808622
H -6.213188 -1.435542 2.164048
H -6.914935 -0.023922 3.007503
H -6.224306 -1.617965 -1.418918
H -4.760044 -2.446013 -2.063263
H -5.976542 -3.343466 -1.138273
H   4.795713 -2.775005 0.361051
H   6.078850 -2.005319 1.301820
H   4.429928 -1.861322 1.844072
H   5.596730 0.959512 -1.010202
H   7.749059 0.474110 -1.281591
H   7.774995 -0.597189 1.583866
H   9.171334 -0.114462 0.636637
H   6.899296 1.737501 1.522221
H   8.551492 1.690317 2.147155
H   8.256525 2.222072 0.483893
H   8.758496 -1.676873 -1.315903
H   7.124175 -1.847810 -1.965929
H   7.499692 -2.407804 -0.326578
SCF Energy (B3LYP/6-31G**)= -1361.69768794
Number of imaginary frequencies = 0

1b_c418
MMFF Geometry
C -1.626068 -1.638639 -1.125264
C -2.298423 0.862858 -0.298085
N -2.942551 -1.328274 -1.045895
C -0.579214 -0.765376 -0.813179
C -0.939566 0.526720 -0.385120
C -3.264632 -0.081237 -0.636926
C   0.067547 1.453403 -0.058818
C   1.423299 1.110087 -0.143310
C   1.774099 -0.179240 -0.556107
C   0.784756 -1.108907 -0.905946
C   3.209804 -0.517082 -0.653163
C   4.238641 0.351288 0.100298
C   3.744191 1.780089 0.242010
C   2.446336 2.109260 0.164944
O   1.099729 -2.370437 -1.350172
O   3.565819 -1.480991 -1.338783
O   5.429511 0.338120 -0.706497
C   4.584035 -0.205068 1.484014
C   4.647969 -1.713139 1.509590
C   5.964662 -2.365852 1.185113
O   3.639345 -2.377239 1.751046
O   4.647069 2.743037 0.671813
C   5.466293 3.229655 -0.391126
C -4.712841 0.231559 -0.561915
C -5.149616 1.520637 -1.192732
C -5.513850 -0.646137 0.079939
C -7.004058 -0.660296 0.355921
C -7.286252 -0.439617 1.857677
C -6.907754 0.937926 2.388436
C -7.900244 0.217545 -0.518579
H -1.424963 -2.654043 -1.460317
H -2.601000 1.847481 0.044767
H -0.214505 2.455360 0.263220
H   2.131921 3.130821 0.359782
H   2.076232 -2.446377 -1.430734
H   5.370314 -0.451485 -1.282862
H   3.828838 0.082348 2.227902
H   5.544121 0.184732 1.844035
H   5.894519 -3.441389 1.370550
H   6.213233 -2.202836 0.134654
H   6.747944 -1.955028 1.826895
H   6.363756 2.613467 -0.491842
H   4.925807 3.281871 -1.342969
H   5.787147 4.242018 -0.127535
H -4.325924 2.052088 -1.681638
H -5.890218 1.340133 -1.977020
H -5.568183 2.196934 -0.440768
H -5.026405 -1.534232 0.494798
H -7.319403 -1.691229 0.136204
H -8.353227 -0.607719 2.051188
H -6.747754 -1.196326 2.443040
H -7.472061 1.730746 1.889507
H -7.131787 0.997363 3.458673
H -5.839957 1.139582 2.262715
H -8.955465 0.018453 -0.298822
H -7.746713 -0.002944 -1.580224
H -7.734879 1.285611 -0.357212
SCF Energy (B3LYP/6-31G**)= -1361.68801551
Number of imaginary frequencies = 0

1b_c419
MMFF Geometry
C -1.542785 -2.379796 -0.214538
C -2.433640 0.165341 0.130695
N -2.865018 -2.194446 0.014857
C -0.593610 -1.355486 -0.284875
C -1.066180 -0.041586 -0.102337
C -3.299086 -0.925839 0.175570
C -0.161455 1.034179 -0.163176
C   1.203946 0.817854 -0.386104
C   1.669845 -0.489159 -0.558707
C   0.778598 -1.570701 -0.524278
C   3.122781 -0.699063 -0.772008
C   4.096070 0.414443 -0.310604
C   3.442001 1.780693 -0.488506
C   2.113468 1.953060 -0.519688
O   1.205329 -2.863171 -0.712842
O   3.493549 -1.781469 -1.230188
O   5.268313 0.367167 -1.121078
C   4.427282 0.127158 1.165686
C   5.808805 -0.445678 1.393656
C   6.085308 -1.845219 0.908431
O   6.666078 0.206486 1.993822
O   4.245030 2.868490 -0.808280
C   5.199935 3.205234 0.197738
C -4.751883 -0.770192 0.425777
C -5.332564 -1.792514 1.365674
C -5.453589 0.208571 -0.184974
C -6.940852 0.476952 -0.078816
C -7.164603 1.801183 0.673434
C -8.632047 2.104924 0.942735
C -7.550081 0.494890 -1.484207
H -1.252956 -3.420409 -0.344740
H -2.818505 1.168085 0.289484
H -0.531931 2.051428 -0.038685
H   1.691688 2.938698 -0.697613
H   2.138979 -2.846130 -1.025128
H   5.053217 0.793397 -1.970517
H   3.705147 -0.556757 1.630106
H   4.363439 1.041354 1.769083
H   7.020156 -2.203693 1.348993
H   5.279245 -2.511075 1.227012
H   6.180588 -1.862041 -0.178398
H   4.716255 3.328698 1.172131

H 5.999723 2.462369 0.251691
H 5.651543 4.162532 -0.078931
H -4.658332 -1.971106 2.211601
H -6.282798 -1.474463 1.802188
H -5.496405 -2.743021 0.847146
H -4.933396 0.881875 -0.866802
H -7.439162 -0.325587 0.474097
H -6.642960 1.761787 1.638651
H -6.725253 2.636970 0.113983
H -9.113732 1.277940 1.473795
H -8.721006 3.002736 1.562977
H -9.181142 2.289635 0.014966
H -8.641391 0.567035 -1.441232
H -7.307960 -0.425655 -2.027737
H -7.178317 1.341009 -2.073066
SCF Energy (B3LYP/6-31G**)= -1361.69299307
Number of imaginary frequencies = 0

1b_c420
MMFF Geometry
C -1.602661 -2.352483 -0.161025
C -2.422312 0.194098 -0.644328
N -2.934698 -2.106661 -0.183829
C -0.609677 -1.389612 -0.365867
C -1.046030 -0.074579 -0.617422
C -3.331483 -0.836997 -0.416420
C -0.096368 0.938110 -0.845633
C 1.276141 0.663533 -0.805168
C 1.704912 -0.641393 -0.543481
C 0.771847 -1.667731 -0.340403
C 3.163077 -0.907336 -0.483291
C 4.111759 0.287309 -0.213299
C 3.546551 1.552091 -0.851239
C 2.242941 1.713423 -1.116311
O 1.164475 -2.963702 -0.107069
O 3.545842 -2.075732 -0.568951
O 5.379572 0.012096 -0.805056
C 4.228062 0.421545 1.316440
C 5.540761 -0.069063 1.886366
C 5.820826 -1.549216 1.849884
O 6.336003 0.722075 2.398345
O 4.431028 2.504988 -1.341225
C 5.255145 3.105266 -0.342321
C -4.797087 -0.614789 -0.437832
C -5.576727 -1.692161 -1.142948
C -5.336234 0.466496 0.165141
C -6.804584 0.821792 0.279420
C -7.175397 0.982561 1.766574
C -8.662514 1.212878 1.999022
C -7.074998 2.093122 -0.528252
H -1.342708 -3.390730 0.034671
H -2.782706 1.196303 -0.855415
H -0.436534 1.951004 -1.059827
H 1.891421 2.611795 -1.616722
H 2.131992 -3.036742 -0.274641
H 5.299481 0.185562 -1.760557
H 3.421668 -0.103958 1.844151
H 4.121782 1.467781 1.629521
H 6.672015 -1.773335 2.499334
H 4.951918 -2.097381 2.222858
H 6.060580 -1.868824 0.834538
H 4.650299 3.496784 0.482001
H 6.008028 2.404088 0.026365
H 5.780237 3.946142 -0.805054
H -5.687160 -2.571225 -0.499296
H -6.574577 -1.364852 -1.445296
H -5.069267 -1.999581 -2.064942
H -4.674596 1.170700 0.670374
H -7.429146 0.023247 -0.132628
H -6.881727 0.076445 2.312492
H -6.613786 1.813204 2.212860
H -9.259935 0.418255 1.541212
H -8.876836 1.221265 3.072653
H -8.989231 2.173346 1.590248
H -8.144731 2.322253 -0.559967
H -6.738628 1.977470 -1.565044
H -6.556039 2.958628 -0.101082
SCF Energy (B3LYP/6-31G**)= -1361.69307717
Number of imaginary frequencies = 0

1b_c421
MMFF Geometry
C -1.579319 -1.890281 -0.394204
C -2.246587 0.627561 0.388660
N -2.875480 -1.645151 -0.083766
C -0.552018 -0.942262 -0.342550
C -0.909380 0.358616 0.065452
C -3.192700 -0.391159 0.300804
C 0.075943 1.361139 0.130289
C 1.410181 1.080571 -0.185565
C 1.761150 -0.217339 -0.574056
C 0.788936 -1.220236 -0.674632
C 3.181678 -0.483627 -0.896691
C 4.246452 0.444649 -0.255987
C 3.717621 1.875143 -0.242755
C 2.405904 2.152945 -0.203671
O 1.100262 -2.488786 -1.099228
O 3.457533 -1.441576 -1.619945
O 5.448908 0.399541 -1.028960
C 4.590700 -0.033000 1.159632
C 4.688047 -1.539343 1.252137
C 5.941682 -2.200890 0.744924
O 3.747960 -2.194816 1.703838
O 4.601947 2.926850 -0.437942
C 5.556171 3.076823 0.609732
C -4.616308 -0.152384 0.628009
C -5.212485 -1.123706 1.605406
C -5.277137 0.838561 -0.008710
C -6.709952 1.324189 0.097308
C -7.532116 0.925184 -1.145723
C -7.750969 -0.572077 -1.323084
C -7.460304 1.030018 1.397788
H -1.377474 -2.915306 -0.698378
H -2.550667 1.617882 0.713874
H -0.207200 2.371010 0.424808
H 2.058531 3.181860 -0.237382
H 2.041904 -2.507539 -1.382163
H 5.231579 0.767261 -1.905364
H 3.824811 0.279464 1.881557
H 5.543267 0.376795 1.513532
H 5.966634 -3.240539 1.083556
H 5.960403 -2.181740 -0.346422
H 6.819288 -1.688285 1.146591
H 5.083404 3.008192 1.595392
H 6.358512 2.340446 0.513241
H 6.002864 4.071122 0.516071
H -4.527748 -1.938649 1.864774
H -5.462345 -0.619044 2.543798
H -6.110161 -1.593059 1.191799
H -4.718747 1.409308 -0.754799
H -6.630805 2.421418 0.066366
H -8.511278 1.418532 -1.101356
H -7.036829 1.311167 -2.046226
H -8.301844 -1.001436 -0.481669
H -8.337359 -0.756293 -2.229382
H -6.803431 -1.108325 -1.428792
H -8.404029 1.587050 1.422110
H -6.875519 1.346996 2.267920
H -7.711407 -0.026501 1.515742
SCF Energy (B3LYP/6-31G**)= -1361.68418270
Number of imaginary frequencies = 0

1b_c422
MMFF Geometry
C -1.765756 -1.431756 -0.725132
C -2.143063 1.140241 0.062404
N -3.033632 -1.010605 -0.501137
C -0.625879 -0.638210 -0.578273
C -0.832578 0.691632 -0.169578
C -3.219609 0.271109 -0.109916
C 0.275851 1.543847 -0.008358
C 1.581763 1.086732 -0.232129

C 1.780702 -0.238168 -0.626263
C 0.686272 -1.094381 -0.816001
C 3.168139 -0.710859 -0.838082
C 4.319592 0.068725 -0.157512
C 3.977859 1.562760 -0.106325
C 2.709858 2.012294 -0.118363
O 0.850760 -2.394453 -1.229760
O 3.326493 -1.743815 -1.491403
O 5.524256 -0.096585 -0.904963
C 4.514944 -0.465673 1.270545
C 5.524431 -1.588873 1.412666
C 5.328447 -2.849428 0.613773
O 6.447354 -1.499586 2.227231
O 5.103440 2.365409 -0.048680
C 4.936797 3.772900 0.083818
C -4.631064 0.700503 0.110634
C -4.933176 2.165069 -0.059017
C -5.566244 -0.226325 0.415932
C -7.037532 -0.010244 0.699289
C -7.867985 -0.556809 -0.475702
C -9.359105 -0.277883 -0.345616
C -7.397255 -0.681218 2.028850
H -1.688186 -2.470538 -1.039167
H -2.311292 2.161215 0.388977
H 0.111404 2.577707 0.294019
H 2.468311 3.068501 -0.068434
H 1.785745 -2.519206 -1.513526
H 5.695947 0.737683 -1.377294
H 3.571367 -0.804991 1.715194
H 4.882030 0.341227 1.920497
H 5.607381 -2.687338 -0.428498
H 5.964682 -3.639696 1.023552
H 4.289281 -3.178398 0.690492
H 4.425909 4.188780 -0.790372
H 4.400648 4.017962 1.006254
H 5.931195 4.225334 0.140416
H -4.408261 2.575153 -0.929523
H -5.993543 2.362873 -0.236313
H -4.629661 2.726523 0.830644
H -5.254776 -1.271080 0.483880
H -7.254140 1.056569 0.815048
H -7.517230 -0.100124 -1.410416
H -7.716192 -1.638793 -0.579657
H -9.548599 0.791354 -0.208597
H -9.881001 -0.600445 -1.252402
H -9.797427 -0.819892 0.497284
H -8.423099 -0.443069 2.327113
H -6.737966 -0.335119 2.833229
H -7.307963 -1.771634 1.966308
SCF Energy (B3LYP/6-31G**)= -1361.69976533
Number of imaginary frequencies = 0

1b_c423
MMFF Geometry
C -1.742083 -1.601266 -0.310436
C -2.151329 0.998350 0.362184
N -3.004635 -1.207285 -0.017532
C -0.622963 -0.765899 -0.291053
C -0.846393 0.578473 0.056858
C -3.206242 0.087730 0.318182
C 0.239272 1.473744 0.083036
C 1.540652 1.045587 -0.212741
C 1.757602 -0.293729 -0.543593
C 0.683667 -1.193851 -0.600775
C 3.141781 -0.734037 -0.832468
C 4.311305 0.121075 -0.286758
C 3.920736 1.603967 -0.297236
C 2.639898 2.011608 -0.239180
O 0.863708 -2.510223 -0.951739
O 3.288772 -1.798559 -1.435841
O 5.463360 -0.051452 -1.111460
C 4.629465 -0.319075 1.151334
C 5.685293 -1.400201 1.282768
C 5.476599 -2.711883 0.574551
O 6.660603 -1.234008 2.020634
O 5.017748 2.443838 -0.372208
C 4.810563 3.850851 -0.311251
C -4.607632 0.478406 0.648084
C -4.788046 1.649283 1.576186
C -5.633018 -0.237453 0.135353
C -7.118298 -0.008137 0.317613
C -7.793687 0.078125 -1.065255
C -9.271132 0.440288 -0.996884
C -7.697336 -1.132923 1.178233
H -1.650943 -2.653439 -0.571837
H -2.335520 2.036541 0.618292
H 0.060387 2.518281 0.336581
H 2.364675 3.060721 -0.232945
H 1.779273 -2.622123 -1.297417
H 5.565781 0.754468 -1.648614
H 3.733478 -0.659271 1.684867
H 5.014834 0.536839 1.723337
H 6.168496 -3.455988 0.980542
H 4.458051 -3.067795 0.747338
H 5.673262 -2.604069 -0.493203
H 4.221902 4.195477 -1.167337
H 4.335385 4.135469 0.632994
H 5.789419 4.337125 -0.357136
H -4.659933 2.592638 1.035317
H -5.770844 1.666358 2.054154
H -4.062753 1.613768 2.397286
H -5.396892 -1.090438 -0.504916
H -7.301943 0.940410 0.831788
H -7.283973 0.839733 -1.669804
H -7.686171 -0.872544 -1.603057
H -9.422858 1.370346 -0.440204
H -9.669079 0.581975 -2.006960
H -9.857165 -0.350433 -0.519747
H -8.747554 -0.944697 1.422015
H -7.155942 -1.218326 2.127464
H -7.636494 -2.100998 0.668320
SCF Energy (B3LYP/6-31G**)= -1361.69979331
Number of imaginary frequencies = 0

1b_c424
MMFF Geometry
C -1.673381 -2.465864 -0.141953
C -2.517004 0.101784 0.153203
N -2.988335 -2.252948 0.104786
C -0.709263 -1.459501 -0.254205
C -1.157329 -0.133344 -0.097392
C -3.398955 -0.973610 0.240318
C -0.236963 0.925966 -0.201523
C 1.120298 0.680997 -0.441098
C 1.561181 -0.637966 -0.586506
C 0.654664 -1.703690 -0.511060
C 3.006721 -0.873512 -0.817490
C 3.997616 0.228385 -0.363452
C 3.374873 1.599429 -0.599866
C 2.048558 1.794179 -0.626594
O 1.059214 -3.006338 -0.675655
O 3.353945 -1.961163 -1.281113
O 5.175861 0.101957 -1.158972
C 4.295540 -0.051864 1.122352
C 5.764161 -0.073366 1.476050
C 6.536393 -1.337020 1.195289
O 6.290573 0.885859 2.043952
O 4.188069 2.658598 -0.978325
C 5.177133 3.011871 -0.013459
C -4.844305 -0.787556 0.510952
C -5.426949 -1.776215 1.485078
C -5.540451 0.190708 -0.106936
C -7.020438 0.480576 0.023677
C -7.286029 1.698811 0.929211
C -6.720064 3.023726 0.432478
C -7.648773 0.641544 -1.364202
H -1.402504 -3.514032 -0.250448
H -2.882793 1.114488 0.292929
H -0.588514 1.952218 -0.098316
H 1.641932 2.778319 -0.843717
H 1.985772 -3.009853 -1.009018
H 4.970166 0.469869 -2.037819

H 3.886308 -1.018413 1.446634
H 3.802133 0.683330 1.771681
H 6.277770 -1.741999 0.215056
H 7.607922 -1.119325 1.205566
H 6.307747 -2.075746 1.967400
H 4.772019 3.000954 1.003533
H 6.046342 2.356620 -0.103231
H 5.507554 4.031422 -0.233678
H -4.744562 -1.941660 2.327073
H -6.367356 -1.431875 1.923715
H -5.611225 -2.737696 0.994427
H -5.020465 0.839073 -0.812058
H -7.527259 -0.375216 0.483870
H -8.369550 1.813387 1.059712
H -6.876517 1.499314 1.927902
H -7.154232 3.310857 -0.529439
H -6.953119 3.818250 1.149162
H -5.632048 2.983982 0.326058
H -8.716882 0.869448 -1.281874
H -7.550264 -0.284611 -1.942036
H -7.174709 1.441418 -1.942917
SCF Energy (B3LYP/6-31G**)= -1361.69006370
Number of imaginary frequencies = 0

1b_c425
MMFF Geometry
C -1.679632 -1.514552 -0.646771
C -2.153946 1.069020 0.052380
N -2.963274 -1.138447 -0.429803
C -0.571793 -0.669494 -0.539037
C -0.829363 0.667331 -0.177712
C -3.191142 0.147356 -0.079049
C 0.241517 1.573908 -0.067406
C 1.560528 1.161578 -0.289216
C 1.810251 -0.172658 -0.629476
C 0.754948 -1.081212 -0.776347
C 3.216533 -0.579243 -0.852937
C 4.322118 0.260725 -0.161467
C 3.932149 1.734436 -0.209522
C 2.653022 2.134964 -0.261754
O 0.969642 -2.383044 -1.157811
O 3.445259 -1.576655 -1.538293
O 5.561264 0.083820 -0.853322
C 4.529219 -0.211765 1.282305
C 4.475659 -1.717746 1.409514
C 5.689484 -2.507311 0.997550
O 3.450512 -2.269826 1.811634
O 4.923117 2.693503 -0.365750
C 5.819951 2.778217 0.738397
C -4.609105 0.527793 0.168095
C -4.848650 1.478161 1.305591
C -5.571900 -0.030016 -0.598173
C -7.072855 0.170036 -0.651607
C -7.834414 -0.994000 0.012694
C -7.626704 -1.133700 1.514972
C -7.610438 1.539497 -0.228350
H -1.559461 -2.560525 -0.921155
H -2.372843 2.098727 0.316872
H 0.038336 2.613092 0.188615
H 2.408157 3.190791 -0.337525
H 1.921534 -2.497767 -1.376478
H 5.435473 0.447483 -1.749126
H 3.752899 0.189633 1.946840
H 5.492511 0.114943 1.689608
H 5.592855 -3.536067 1.356006
H 5.779097 -2.516704 -0.090397
H 6.585072 -2.070500 1.446340
H 5.282109 2.777918 1.692581
H 6.552990 1.967675 0.707070
H 6.364276 3.723653 0.655946
H -4.067728 1.397134 2.070667
H -4.872611 2.512465 0.948577
H -5.780867 1.257189 1.829700
H -5.242283 -0.750343 -1.353027
H -7.313655 0.107903 -1.724122
H -8.907921 -0.876335 -0.181846
H -7.539135 -1.937189 -0.465236
H -7.963617 -0.243531 2.053234
H -8.204149 -1.984570 1.891525
H -6.576348 -1.313856 1.761461
H -8.646190 1.655038 -0.568488
H -7.027543 2.349128 -0.680537
H -7.619026 1.683409 0.853800
SCF Energy (B3LYP/6-31G**)= -1361.68445435
Number of imaginary frequencies = 0

1b_c426
MMFF Geometry
C 1.802497 2.376998 -0.031077
C 2.475653 -0.250871 -0.186424
N 3.119470 2.058048 -0.036649
C 0.755164 1.452037 -0.097898
C 1.116440 0.095818 -0.179891
C 3.442560 0.749008 -0.106259
C 0.108727 -0.881368 -0.256276
C -1.249956 -0.530775 -0.247264
C -1.609394 0.817316 -0.161777
C -0.610891 1.804900 -0.091248
C -3.047121 1.187726 -0.144176
C -4.126907 0.084670 -0.161267
C -3.564459 -1.310441 -0.324512
C -2.254676 -1.585557 -0.329901
O -0.916238 3.143810 -0.011293
O -3.339584 2.388253 -0.108567
O -4.904268 0.393703 -1.323383
C -4.926534 0.228967 1.146110
C -6.317770 -0.353092 1.066673
C -6.645862 -1.502999 1.980962
O -7.168945 0.141836 0.325840
O -4.457924 -2.374130 -0.335479
C -5.126081 -2.503111 -1.592970
C 4.893794 0.444269 -0.116047
C 5.701271 1.348524 -1.005430
C 5.383370 -0.558438 0.645172
C 6.814894 -1.018656 0.842843
C 7.298489 -1.862600 -0.350323
C 8.649902 -2.523818 -0.114545
C 7.774974 0.110859 1.228099
H 1.601299 3.444497 0.030576
H 2.777907 -1.290482 -0.267520
H 0.392500 -1.931416 -0.324532
H -1.919721 -2.616896 -0.400201
H -1.893786 3.248155 -0.065248
H -5.533568 1.100380 -1.079445
H -5.071887 1.284323 1.412682
H -4.384589 -0.215658 1.990066
H -7.597766 -1.949707 1.680977
H -5.870501 -2.269411 1.919837
H -6.725715 -1.136993 3.007405
H -5.989594 -1.834217 -1.639006
H -4.450829 -2.324275 -2.437079
H -5.497379 -3.529640 -1.667183
H 6.658070 0.909398 -1.293794
H 5.170634 1.550153 -1.943482
H 5.897590 2.304002 -0.507817
H 4.681966 -1.124401 1.260086
H 6.784038 -1.687092 1.716073
H 6.563110 -2.650604 -0.558487
H 7.363058 -1.250828 -1.257099
H 8.637221 -3.131223 0.795862
H 8.898249 -3.179590 -0.955325
H 9.448932 -1.781712 -0.026845
H 8.681372 -0.296935 1.688916
H 7.318914 0.787118 1.959648
H 8.091352 0.702744 0.365088
SCF Energy (B3LYP/6-31G**)= -1361.69616955
Number of imaginary frequencies = 0

1b_c427
MMFF Geometry
C -1.606141 -2.294604 -0.569954
C -2.416125 0.298367 -0.656410

N -2.937526 -2.044079 -0.580173
C -0.609446 -1.314350 -0.598529
C -1.040462 0.024651 -0.646030
C -3.330206 -0.752866 -0.613838
C -0.085616 1.057193 -0.691399
C 1.287259 0.776581 -0.669877
C 1.709195 -0.553381 -0.607716
C 0.771311 -1.596214 -0.589750
C 3.163314 -0.830959 -0.564294
C 4.110793 0.298092 -0.090633
C 3.565251 1.658555 -0.541213
C 2.257867 1.866415 -0.781168
O 1.158777 -2.914351 -0.559168
O 3.535876 -1.972114 -0.843367
O 5.405619 0.115512 -0.662737
C 4.209242 0.263340 1.443044
C 5.338056 -0.580527 2.003876
C 5.409694 -2.038275 1.635812
O 6.138469 -0.096903 2.809315
O 4.556667 2.615562 -0.666652
C 4.185613 3.948333 -1.001973
C -4.795658 -0.521524 -0.629173
C -5.560894 -1.474319 -1.508770
C -5.339505 0.448714 0.136992
C -6.800983 0.823185 0.258682
C -7.425950 0.279911 1.558762
C -6.835120 0.825134 2.853324
C -6.963341 2.340725 0.126643
H -1.349882 -3.351564 -0.537081
H -2.772577 1.322458 -0.711438
H -0.424056 2.091547 -0.745519
H 1.870429 2.832313 -1.086763
H 2.128874 -2.961801 -0.723415
H 5.515628 0.782406 -1.363980
H 3.275045 -0.075544 1.907387
H 4.385572 1.278701 1.825445
H 6.095078 -2.550114 2.318096
H 4.423035 -2.495680 1.741903
H 5.783877 -2.156394 0.617757
H 3.727868 3.987101 -1.995522
H 3.517619 4.370508 -0.244377
H 5.095757 4.554793 -1.025884
H -5.518898 -2.492461 -1.106528
H -6.616556 -1.216478 -1.613330
H -5.138640 -1.483355 -2.520164
H -4.680586 1.034148 0.778687
H -7.370530 0.388306 -0.568870
H -8.501250 0.498953 1.551996
H -7.336321 -0.814048 1.569756
H -6.964894 1.908447 2.929581
H -7.342000 0.371750 3.711680
H -5.769426 0.594427 2.940177
H -8.014544 2.628859 0.234026
H -6.624705 2.680587 -0.858856
H -6.383379 2.885980 0.878873
SCF Energy (B3LYP/6-31G**)= -1361.69841691
Number of imaginary frequencies = 0

1b_c428
MMFF Geometry
C -1.758378 -1.437104 -0.250245
C -2.141279 1.176970 0.381808
N -3.012142 -1.031591 0.063867
C -0.635138 -0.607660 -0.269329
C -0.844719 0.744354 0.059585
C -3.201459 0.271339 0.376709
C 0.245777 1.633962 0.051511
C 1.537622 1.191320 -0.258564
C 1.739921 -0.153103 -0.578824
C 0.662228 -1.048597 -0.598880
C 3.117149 -0.605018 -0.882080
C 4.300032 0.244101 -0.351191
C 3.920335 1.725044 -0.366194
C 2.645719 2.140129 -0.318571
O 0.832151 -2.372649 -0.924436
O 3.261208 -1.685193 -1.457933
O 5.426022 0.052116 -1.205547
C 4.621393 -0.225041 1.081108
C 4.951607 -1.703499 1.134705
C 4.300887 -2.514749 2.222109
O 5.743190 -2.209183 0.339217
O 4.888308 2.698764 -0.584817
C 6.010279 2.663573 0.287709
C -4.595407 0.674840 0.727311
C -4.748309 1.872772 1.625508
C -5.631621 -0.049337 0.248256
C -7.113562 0.171958 0.456335
C -7.830406 0.535573 -0.860705
C -7.841148 -0.546293 -1.933874
C -7.731646 -1.052247 1.136139
H -1.678293 -2.493880 -0.496102
H -2.314012 2.221300 0.620386
H 0.080738 2.684811 0.287913
H 2.415928 3.200743 -0.376560
H 1.732506 -2.490650 -1.307229
H 5.616771 -0.911097 -1.207056
H 3.773794 -0.033677 1.750358
H 5.494140 0.287805 1.497313
H 4.624914 -3.556506 2.148834
H 4.593329 -2.119672 3.197958
H 3.214755 -2.475141 2.107292
H 5.691672 2.643707 1.334915
H 6.668548 1.820387 0.064130
H 6.584491 3.581589 0.130234
H -4.605351 2.798864 1.058927
H -5.726165 1.926702 2.109073
H -4.017493 1.844282 2.442022
H -5.401515 -0.912352 -0.379745
H -7.280086 1.018627 1.129226
H -8.870736 0.800130 -0.631951
H -7.370437 1.438922 -1.281815
H -8.351829 -1.449950 -1.589203
H -8.375237 -0.184198 -2.818754
H -6.828958 -0.815496 -2.248509
H -8.811557 -0.921596 1.263599
H -7.295707 -1.202230 2.130535
H -7.565507 -1.970853 0.563258
SCF Energy (B3LYP/6-31G**)= -1361.69965517
Number of imaginary frequencies = 0

1b_c429
MMFF Geometry
C -1.932984 -1.930234 -0.330740
C -2.390998 0.746580 -0.355345
N -3.218018 -1.501633 -0.307545
C -0.814358 -1.093799 -0.363904
C -1.063297 0.289532 -0.375190
C -3.442744 -0.167851 -0.321530
C 0.022281 1.183949 -0.408879
C 1.347560 0.723490 -0.422811
C 1.588758 -0.651767 -0.405642
C 0.516691 -1.558321 -0.387219
C 2.987914 -1.129960 -0.419555
C 4.130843 -0.136170 -0.140729
C 3.729308 1.313942 -0.358998
C 2.444262 1.691171 -0.484467
O 0.717013 -2.918408 -0.386671
O 3.203321 -2.328549 -0.625696
O 5.135451 -0.475453 -1.109100
C 4.630231 -0.360857 1.295628
C 6.127740 -0.535102 1.397038
C 6.996343 0.690371 1.307770
O 6.620529 -1.657918 1.523446
O 4.808295 2.179698 -0.381407
C 4.593007 3.534010 -0.759326
C -4.869982 0.266368 -0.313521
C -5.177312 1.565408 -1.004362
C -5.793379 -0.527893 0.272868
C -7.284651 -0.341057 0.456546
C -7.730155 0.988803 1.095444
C -7.078389 1.258949 2.445782
C -8.044388 -0.618683 -0.843131

H -1.822117 -3.012529 -0.321127
H -2.593757 1.812553 -0.352240
H -0.175498 2.255438 -0.425392
H 2.163894 2.729354 -0.625220
H 1.676233 -3.098946 -0.515851
H 5.257005 -1.445969 -1.059572
H 4.182555 -1.265084 1.730444
H 4.325097 0.446357 1.973487
H 7.975071 0.473057 1.745114
H 7.128156 0.978415 0.263141
H 6.546700 1.509566 1.874358
H 4.143524 3.597613 -1.755574
H 3.978198 4.052066 -0.016619
H 5.566669 4.031499 -0.795449
H -4.600181 1.664067 -1.931053
H -6.223892 1.649017 -1.301962
H -4.941655 2.413817 -0.353571
H -5.442811 -1.465335 0.712394
H -7.594002 -1.128095 1.160450
H -7.542707 1.837188 0.430607
H -8.817336 0.958441 1.244355
H -5.998402 1.405033 2.350665
H -7.498076 2.167818 2.888991
H -7.256696 0.431793 3.140214
H -9.124093 -0.642946 -0.658796
H -7.760016 -1.589048 -1.265439
H -7.856419 0.146057 -1.602476
SCF Energy (B3LYP/6-31G**)= -1361.69805349
Number of imaginary frequencies = 0

1b_c430
MMFF Geometry
C 1.897280 2.139430 0.183789
C 2.339473 -0.392158 -0.704798
N 3.180981 1.732654 0.037818
C 0.774048 1.351865 -0.088259
C 1.015500 0.042895 -0.547167
C 3.389014 0.473886 -0.408152
C -0.072858 -0.798354 -0.841725
C -1.393143 -0.360020 -0.675712
C -1.627696 0.936326 -0.207330
C -0.554622 1.795335 0.071109
C -3.028878 1.378700 -0.021189
C -4.150324 0.321741 0.041109
C -3.770444 -0.909654 -0.753392
C -2.502260 -1.239038 -1.039907
O -0.753323 3.083398 0.507461
O -3.268106 2.581581 0.127405
O -5.302327 0.943962 -0.549180
C -4.426832 0.058140 1.524708
C -5.349914 -1.109497 1.794856
C -6.803193 -0.966285 1.423496
O -4.927008 -2.129691 2.342556
O -4.802822 -1.772587 -1.086123
C -5.197838 -1.612262 -2.448946
C 4.804454 0.057194 -0.561829
C 5.165498 -0.612343 -1.854925
C 5.644582 0.288822 0.469933
C 7.119000 0.002867 0.674200
C 7.321756 -1.110680 1.724217
C 6.815948 -2.487064 1.309118
C 7.979190 -0.233462 -0.567856
H 1.790257 3.160975 0.542416
H 2.550388 -1.402571 -1.041622
H 0.116531 -1.807743 -1.206282
H -2.280711 -2.170953 -1.552307
H -1.711983 3.294765 0.450292
H -5.255364 1.893605 -0.317263
H -4.872402 0.939739 2.003260
H -3.489086 -0.142090 2.060370
H -7.370817 -1.797482 1.851864
H -7.197036 -0.033352 1.834608
H -6.926720 -0.981456 0.339805
H -5.494843 -0.580754 -2.663078
H -4.395352 -1.922494 -3.126444
H -6.061632 -2.259639 -2.626054
H 4.324144 -0.661220 -2.554684
H 5.950007 -0.056723 -2.376456
H 5.498278 -1.640062 -1.678645
H 5.211501 0.777719 1.348365
H 7.522252 0.921984 1.124932
H 8.389801 -1.188384 1.963766
H 6.820349 -0.824495 2.657988
H 7.336706 -2.856600 0.421317
H 6.990926 -3.205530 2.116782
H 5.741705 -2.477054 1.102816
H 9.036844 -0.301706 -0.288323
H 7.886729 0.600611 -1.271451
H 7.724898 -1.159264 -1.089752
SCF Energy (B3LYP/6-31G**)= -1361.69104774
Number of imaginary frequencies = 0

1b_c431
MMFF Geometry
C -1.607696 -2.424788 -0.225180
C -2.509968 0.109769 0.165134
N -2.929123 -2.250461 0.017175
C -0.664827 -1.394131 -0.287469
C -1.143271 -0.085832 -0.081545
C -3.368959 -0.986909 0.200165
C -0.245117 0.995892 -0.133169
C 1.119681 0.790743 -0.369881
C 1.591528 -0.510852 -0.565729
C 0.706651 -1.597875 -0.540683
C 3.043904 -0.709221 -0.793395
C 4.014520 0.402787 -0.322835
C 3.351437 1.767790 -0.475097
C 2.021757 1.933007 -0.493403
O 1.139130 -2.884910 -0.751959
O 3.417136 -1.782497 -1.270657
O 5.180634 0.374377 -1.142974
C 4.358872 0.095287 1.146359
C 5.745324 -0.472993 1.354991
C 6.025862 -1.863492 0.846638
O 6.603596 0.174987 1.958253
O 4.145836 2.864834 -0.784671
C 5.106681 3.191903 0.218881
C -4.820358 -0.843163 0.464012
C -5.389886 -1.883065 1.391445
C -5.532264 0.144256 -0.120455
C -7.019386 0.396527 0.007302
C -7.319740 1.570804 0.958873
C -6.778615 2.926781 0.521876
C -7.638711 0.600352 -1.378994
H -1.313065 -3.461662 -0.373217
H -2.899071 1.107848 0.342108
H -0.620313 2.009021 0.009472
H 1.593084 2.918776 -0.653210
H 2.070198 -2.857846 -1.071166
H 4.956517 0.812065 -1.984214
H 3.644236 -0.599637 1.606010
H 4.294629 0.999948 1.763918
H 6.966121 -2.223182 1.274512
H 5.226059 -2.538652 1.161390
H 6.112733 -1.863462 -0.241025
H 4.629953 3.297969 1.198735
H 5.911013 2.452898 0.255461
H 5.550742 4.155810 -0.046840
H -4.711545 -2.068055 2.232642
H -6.341444 -1.577191 1.834520
H -5.548741 -2.827446 0.860360
H -5.020441 0.831855 -0.793644
H -7.511363 -0.488063 0.427400
H -8.406636 1.656543 1.083291
H -6.914861 1.339635 1.952613
H -7.210400 3.243461 -0.431804
H -7.035443 3.686202 1.267892
H -5.689083 2.914908 0.424593
H -8.712263 0.801587 -1.297958
H -7.514724 -0.299123 -1.992939
H -7.177203 1.433214 -1.920102
SCF Energy (B3LYP/6-31G**)= -1361.69199949

Number of imaginary frequencies = 0

1b_c432
MMFF Geometry
C 1.776692 1.922044 -0.285534
C 2.205468 -0.754880 -0.436147
N 3.057278 1.480391 -0.303214
C 0.648209 1.099682 -0.338050
C 0.882820 -0.283608 -0.414721
C 3.267605 0.146156 -0.379685
C -0.211985 -1.164372 -0.466113
C -1.532021 -0.690065 -0.444808
C -1.763669 0.686237 -0.369275
C -0.678958 1.578084 -0.316046
C -3.161033 1.184930 -0.333777
C -4.336972 0.192666 -0.417834
C -3.910994 -1.254144 -0.509808
C -2.631948 -1.647937 -0.488870
O -0.858741 2.939220 -0.236424
O -3.354373 2.403912 -0.270500
O -4.960022 0.549733 -1.672900
C -5.348008 0.479506 0.702108
C -4.968261 -0.072578 2.060313
C -3.793854 0.541633 2.775269
O -5.607023 -0.988646 2.578882
O -4.891784 -2.235659 -0.472540
C -5.712960 -2.252399 -1.638820
C 4.690199 -0.302327 -0.414237
C 4.970949 -1.578230 -1.157769
C 5.632863 0.459813 0.184101
C 7.124971 0.250319 0.333564
C 7.566468 -1.106296 0.916550
C 6.935768 -1.417479 2.268060
C 7.864642 0.566215 -0.968951
H 1.678357 3.003819 -0.225319
H 2.395562 -1.822319 -0.482289
H -0.028262 -2.237281 -0.523595
H -2.396625 -2.709065 -0.503093
H -1.821812 3.136296 -0.259130
H -5.155561 1.505632 -1.630368
H -6.326686 0.058035 0.435680
H -5.533095 1.555048 0.815570
H -3.877981 0.341313 3.847389
H -2.863423 0.108190 2.405420
H -3.793020 1.625208 2.633844
H -6.483187 -1.478353 -1.579131
H -5.120239 -2.143593 -2.553680
H -6.218690 -3.221903 -1.676451
H 4.378406 -1.635407 -2.078198
H 6.011640 -1.662982 -1.474960
H 4.735175 -2.447574 -0.535256
H 5.300836 1.383493 0.665017
H 7.455880 1.008238 1.059292
H 7.357459 -1.928293 0.225514
H 8.656405 -1.093081 1.046630
H 5.852655 -1.548366 2.187446
H 7.352816 -2.346108 2.670967
H 7.135870 -0.617664 2.988108
H 8.947645 0.572406 -0.803414
H 7.583783 1.554052 -1.351202
H 7.654732 -0.168771 -1.751558
SCF Energy (B3LYP/6-31G**)= -1361.69258691
Number of imaginary frequencies = 0

1b_c433
MMFF Geometry
C -1.987121 -1.683278 -0.365346
C -2.325727 0.981345 0.035411
N -3.249538 -1.214786 -0.215957
C -0.834666 -0.894454 -0.327010
C -1.021932 0.483156 -0.120233
C -3.415065 0.112893 -0.016469
C 0.099733 1.331567 -0.078984
C 1.401197 0.829147 -0.228532
C 1.581465 -0.541431 -0.425558
C 0.471974 -1.399814 -0.486191
C 2.955908 -1.064131 -0.581156
C 4.153308 -0.166232 -0.218269
C 3.807306 1.314213 -0.201471
C 2.535971 1.753633 -0.204353
O 0.611214 -2.751140 -0.697129
O 3.108303 -2.225923 -0.971693
O 5.097632 -0.395666 -1.275472
C 4.705788 -0.619875 1.142586
C 6.197126 -0.863014 1.141543
C 7.114451 0.328891 1.187484
O 6.644722 -2.009842 1.078467
O 4.921639 2.132821 -0.152631
C 4.750069 3.535035 -0.319111
C -4.818269 0.588958 0.160026
C -5.007978 1.804740 1.023339
C -5.822854 -0.102455 -0.423111
C -7.316577 0.145121 -0.436010
C -8.020987 -0.210296 0.886456
C -7.818701 -1.660347 1.308955
C -7.697046 1.550458 -0.912722
H -1.924485 -2.757959 -0.523066
H -2.481611 2.045191 0.181343
H -0.050678 2.400282 0.071698
H 2.296015 2.810964 -0.177509
H 1.554802 -2.946194 -0.899554
H 5.177326 -1.366661 -1.375189
H 4.238226 -1.561231 1.462495
H 4.467308 0.088737 1.945823
H 8.101161 0.013029 1.538256
H 7.212042 0.762696 0.190548
H 6.727470 1.071806 1.889343
H 4.259396 3.760907 -1.271379
H 4.193066 3.961216 0.521188
H 5.742305 3.995582 -0.332568
H -4.782783 2.716657 0.460683
H -6.022932 1.893477 1.413939
H -4.356323 1.766447 1.903932
H -5.552098 -0.989748 -1.001373
H -7.719425 -0.536352 -1.200105
H -7.685128 0.444655 1.697448
H -9.098378 -0.034717 0.773853
H -6.769495 -1.870003 1.538198
H -8.404244 -1.873560 2.209095
H -8.146116 -2.347640 0.522432
H -8.768429 1.594920 -1.139192
H -7.156009 1.817377 -1.827216
H -7.496528 2.318643 -0.161407
SCF Energy (B3LYP/6-31G**)= -1361.69819142
Number of imaginary frequencies = 0

1b_c434
MMFF Geometry
C -1.861785 -1.349738 -0.629064
C -2.169531 1.253210 0.083414
N -3.116690 -0.891809 -0.404341
C -0.701989 -0.579531 -0.518908
C -0.872687 0.766636 -0.148924
C -3.268185 0.405241 -0.050426
C 0.257255 1.596699 -0.025962
C 1.549399 1.102016 -0.249778
C 1.712599 -0.238610 -0.605250
C 0.596241 -1.073865 -0.756772
C 3.086007 -0.750441 -0.817526
C 4.263249 0.021811 -0.173713
C 3.957308 1.524430 -0.164646
C 2.700158 2.003470 -0.176693
O 0.725340 -2.389453 -1.132269
O 3.212488 -1.806252 -1.440559
O 5.454966 -0.194792 -0.928774
C 4.462515 -0.473442 1.267852
C 5.446925 -1.615536 1.433377
C 5.212162 -2.894759 0.675674
O 6.381052 -1.523551 2.234765
O 5.102017 2.301418 -0.143858
C 4.970016 3.715856 -0.052785
C -4.666679 0.874324 0.171821

C -4.935514 2.341774 -0.027411
C -5.623501 -0.021480 0.501943
C -7.084388 0.244505 0.790114
C -7.980028 -0.110221 -0.412562
C -7.976918 -1.577321 -0.824867
C -7.509933 -0.489162 2.066076
H -1.812332 -2.399119 -0.911993
H -2.309650 2.287452 0.380062
H 0.120654 2.642898 0.246344
H 2.484073 3.066162 -0.156551
H 1.653791 -2.545021 -1.422199
H 5.640673 0.620375 -1.428365
H 3.516441 -0.776511 1.732909
H 4.855948 0.343864 1.888739
H 5.834366 -3.687142 1.102558
H 4.166516 -3.196396 0.773713
H 5.482744 -2.771108 -0.374041
H 4.458947 4.117021 -0.933719
H 4.450484 4.001551 0.867468
H 5.975351 4.145983 -0.020884
H -4.417238 2.717609 -0.917099
H -5.994325 2.561459 -0.188975
H -4.601782 2.916300 0.842874
H -5.336860 -1.071335 0.590990
H -7.232634 1.310635 0.996582
H -9.012650 0.181825 -0.183269
H -7.675697 0.493695 -1.277142
H -8.347878 -2.221141 -0.022293
H -8.631779 -1.720325 -1.690850
H -6.975933 -1.915284 -1.108519
H -8.570953 -0.317096 2.276362
H -6.937573 -0.127695 2.928124
H -7.348946 -1.570145 1.995232
SCF Energy (B3LYP/6-31G**)= -1361.69897683
Number of imaginary frequencies = 0

1b_c435
MMFF Geometry
C 1.846255 2.236175 -0.038125
C 2.431752 -0.396627 0.289656
N 3.146551 1.889777 0.121495
C 0.774128 1.337652 -0.045102
C 1.089852 -0.021337 0.127795
C 3.427353 0.578030 0.273508
C 0.054920 -0.972694 0.135535
C -1.286324 -0.593285 -0.026090
C -1.600482 0.757906 -0.197649
C -0.574189 1.719099 -0.210074
C -3.020386 1.160210 -0.360743
C -4.136187 0.096306 -0.282688
C -3.615523 -1.319233 -0.162334
C -2.320516 -1.622423 -0.013084
O -0.834698 3.059416 -0.377389
O -3.269520 2.356075 -0.550233
O -4.808207 0.221498 -1.540865
C -5.029508 0.479839 0.910797
C -6.431197 -0.073870 0.814344
C -6.872958 -1.042850 1.878194
O -7.202493 0.307643 -0.067609
O -4.544760 -2.347607 -0.068511
C -5.117598 -2.673030 -1.337490
C 4.861664 0.241263 0.448147
C 5.594221 1.168320 1.378150
C 5.398600 -0.802196 -0.220956
C 6.824735 -1.317285 -0.242575
C 7.839751 -0.262493 -0.722322
C 9.214193 -0.846886 -1.023647
C 7.208063 -1.985681 1.079936
H 1.680401 3.304004 -0.164901
H 2.697147 -1.438564 0.440311
H 0.303537 -2.025521 0.267944
H -2.020082 -2.658341 0.118862
H -1.800274 3.176315 -0.530361
H -5.428291 0.973606 -1.471923
H -5.155466 1.568309 0.983774
H -4.572224 0.172368 1.859487
H -7.814777 -1.511092 1.579219
H -6.124765 -1.826914 2.011467
H -7.018770 -0.505418 2.818314
H -5.948903 -2.001092 -1.566879
H -4.372476 -2.657251 -2.140615
H -5.520697 -3.687873 -1.268652
H 5.008034 1.347808 2.287031
H 6.553095 0.768894 1.710792
H 5.783869 2.131775 0.892990
H 4.749389 -1.367235 -0.891828
H 6.829138 -2.116984 -0.998073
H 7.462734 0.214264 -1.636206
H 7.962878 0.533710 0.018496
H 9.145199 -1.660969 -1.751958
H 9.866485 -0.073587 -1.442232
H 9.694015 -1.229655 -0.118179
H 8.114992 -2.588042 0.963275
H 6.416010 -2.660155 1.424662
H 7.397651 -1.256483 1.872475
SCF Energy (B3LYP/6-31G**)= -1361.69644788
Number of imaginary frequencies = 0

1b_c436
MMFF Geometry
C 1.961582 -2.178409 -0.247767
C 2.473197 0.486016 -0.407736
N 3.256278 -1.783125 -0.311331
C 0.860923 -1.315629 -0.259464
C 1.137906 0.061233 -0.345987
C 3.498946 -0.456729 -0.379844
C 0.072461 0.979498 -0.377308
C -1.260346 0.549270 -0.307186
C -1.529295 -0.817423 -0.205196
C -0.479105 -1.748621 -0.197357
C -2.942155 -1.253500 -0.131817
C -4.002458 -0.219241 0.309165
C -3.620913 1.191025 -0.131461
C -2.345637 1.528340 -0.400094
O -0.712658 -3.102162 -0.133733
O -3.191822 -2.436820 -0.378121
O -3.960040 -0.256169 1.742884
C -5.365476 -0.701156 -0.229610
C -6.550026 0.015546 0.383245
C -7.747283 0.234800 -0.501570
O -6.556600 0.354038 1.568677
O -4.692196 2.063217 -0.196540
C -4.435554 3.450024 -0.387545
C 4.928208 -0.066616 -0.448993
C 5.746502 -0.914805 -1.382258
C 5.389866 0.952461 0.307840
C 6.795751 1.498684 0.451359
C 7.881917 0.474071 0.832226
C 7.568446 -0.291534 2.111598
C 7.205347 2.318377 -0.775004
H 1.824893 -3.255955 -0.184804
H 2.711287 1.541961 -0.490287
H 0.290918 2.043790 -0.456380
H -2.063558 2.532760 -0.694635
H -1.684084 -3.252005 -0.186900
H -4.816754 0.101349 2.062206
H -5.530033 -1.762328 -0.004240
H -5.397436 -0.588292 -1.320105
H -8.523134 0.768888 0.053850
H -7.458269 0.836965 -1.366527
H -8.142660 -0.730262 -0.827429
H -3.791393 3.843306 0.405519
H -4.000915 3.633003 -1.375274
H -5.392452 3.978004 -0.338362
H 5.200986 -1.106428 -2.313786
H 6.682746 -0.437983 -1.676565
H 5.986578 -1.877323 -0.918286
H 4.683114 1.462549 0.964373
H 6.749616 2.212142 1.287653
H 8.061417 -0.239747 0.022668
H 8.830148 1.007030 0.979754
H 6.686800 -0.928634 1.994679

H 8.411413 -0.937508 2.377545
H 7.392594 0.394314 2.946402
H 8.148829 2.842933 -0.587795
H 6.448989 3.074771 -1.012911
H 7.346111 1.693643 -1.661799
SCF Energy (B3LYP/6-31G**)= -1361.70565261
Number of imaginary frequencies = 0

1b_c437
MMFF Geometry
C 1.894529 -2.415443 0.026902
C 2.542899 0.223830 0.047252
N 3.207043 -2.084619 0.093397
C 0.840859 -1.497697 -0.032948
C 1.188540 -0.134270 -0.020716
C 3.517756 -0.770786 0.095450
C 0.173513 0.838032 -0.081540
C -1.179288 0.473871 -0.142810
C -1.519593 -0.880661 -0.142347
C -0.518557 -1.863790 -0.103231
C -2.952533 -1.246924 -0.207468
C -3.996345 -0.184789 0.205672
C -3.513579 1.225963 -0.118701
C -2.206020 1.511221 -0.264167
O -0.820019 -3.205012 -0.133527
O -3.235952 -2.401112 -0.540337
O -4.076486 -0.302285 1.633432
C -5.330276 -0.565474 -0.469675
C -6.527365 0.176536 0.086031
C -7.634111 0.505934 -0.878855
O -6.618013 0.448909 1.284972
O -4.533544 2.154721 -0.217218
C -4.196989 3.534946 -0.302631
C 4.963703 -0.450525 0.177257
C 5.713807 -1.280769 1.181427
C 5.493062 0.498809 -0.624471
C 6.925222 0.975564 -0.754173
C 7.419605 1.814801 0.438726
C 6.564426 3.047451 0.706023
C 7.906815 -0.150411 -1.093082
H 1.702174 -3.486310 0.024394
H 2.834865 1.269066 0.076515
H 0.447497 1.892369 -0.080795
H -1.853709 2.514491 -0.475128
H -1.789408 -3.301758 -0.274533
H -4.939412 0.080732 1.902496
H -5.562746 -1.627529 -0.321516
H -5.265032 -0.391365 -1.550587
H -8.428127 1.047908 -0.357810
H -7.245232 1.139224 -1.680104
H -8.045439 -0.417920 -1.292561
H -3.603073 3.848826 0.561839
H -3.673765 3.750730 -1.239472
H -5.128771 4.108181 -0.298329
H 5.888228 -2.290325 0.794358
H 6.680665 -0.852745 1.449699
H 5.148879 -1.361942 2.117508
H 4.828385 1.001402 -1.328878
H 6.934473 1.641337 -1.630044
H 7.465919 1.211545 1.351402
H 8.445562 2.148262 0.237396
H 5.550867 2.774730 1.015348
H 7.007715 3.643899 1.509959
H 6.497003 3.679201 -0.185347
H 8.870682 0.268706 -1.403376
H 7.532939 -0.764071 -1.920114
H 8.101198 -0.808599 -0.242231
SCF Energy (B3LYP/6-31G**)= -1361.70580916
Number of imaginary frequencies = 0

1b_c438
MMFF Geometry
C -1.832197 -1.461761 -0.617681
C -2.214880 1.165090 -0.041446
N -3.099928 -1.028952 -0.416366
C -0.694828 -0.654304 -0.548097
C -0.904505 0.704331 -0.247876
C -3.288605 0.280177 -0.130977
C 0.200305 1.572287 -0.167664
C 1.504861 1.102493 -0.364208
C 1.707867 -0.250217 -0.653512
C 0.617086 -1.123868 -0.761066
C 3.098491 -0.732887 -0.836965
C 4.248761 0.109449 -0.230842
C 3.892756 1.591311 -0.290003
C 2.629518 2.034519 -0.349159
O 0.784811 -2.452874 -1.067048
O 3.268964 -1.820801 -1.390464
O 5.429785 -0.101321 -1.001583
C 4.434895 -0.376429 1.218629
C 5.660633 -1.236495 1.434241
C 5.678945 -2.612014 0.819224
O 6.596988 -0.830335 2.126122
O 4.913804 2.516560 -0.468327
C 5.861554 2.553624 0.598313
C -4.700130 0.720533 0.066655
C -5.010388 2.165263 -0.219179
C -5.628391 -0.181863 0.455013
C -7.098098 0.051123 0.733123
C -7.936310 -0.592258 -0.386006
C -9.427462 -0.309196 -0.265575
C -7.443287 -0.510835 2.116018
H -1.752739 -2.522488 -0.846091
H -2.384767 2.209082 0.200842
H 0.035916 2.627850 0.047503
H 2.424480 3.097924 -0.439408
H 1.718640 -2.599168 -1.342785
H 5.350553 0.436666 -1.810138
H 3.567078 -0.940192 1.584660
H 4.520552 0.470856 1.910507
H 6.499745 -3.191632 1.251503
H 4.741231 -3.127287 1.042343
H 5.825655 -2.547189 -0.260050
H 5.360986 2.680412 1.563702
H 6.492291 1.661128 0.601254
H 6.509914 3.420074 0.437657
H -4.495482 2.504754 -1.125326
H -6.073232 2.343996 -0.401792
H -4.701146 2.798680 0.618678
H -5.311611 -1.216359 0.605100
H -7.318418 1.123001 0.763757
H -7.595719 -0.211807 -1.357879
H -7.780643 -1.678543 -0.403030
H -9.620483 0.766934 -0.214222
H -9.955789 -0.706362 -1.138393
H -9.856040 -0.782480 0.622514
H -8.467542 -0.253009 2.403179
H -6.778566 -0.098038 2.883622
H -7.349731 -1.602389 2.141513
SCF Energy (B3LYP/6-31G**)= -1361.69398408
Number of imaginary frequencies = 0

1b_c439
MMFF Geometry
C -1.811402 -1.597738 -0.159363
C -2.221141 1.057258 0.243055
N -3.072527 -1.177155 0.100773
C -0.693721 -0.763751 -0.234832
C -0.917552 0.609641 -0.025821
C -3.274508 0.145664 0.301334
C 0.165767 1.504845 -0.099352
C 1.464631 1.048812 -0.356648
C 1.682791 -0.318189 -0.552747
C 0.611314 -1.220761 -0.508713
C 3.068798 -0.784127 -0.802493
C 4.237125 0.128986 -0.353863
C 3.837145 1.593227 -0.502688
C 2.561279 2.002944 -0.500688
O 0.792116 -2.566034 -0.722181
O 3.227780 -1.909332 -1.279588
O 5.363260 -0.117999 -1.192964
C 4.543162 -0.234063 1.111139

C 5.805062 -1.046691 1.300516
C 5.816472 -2.466699 0.796440
O 6.778104 -0.565418 1.885315
O 4.816387 2.522790 -0.830425
C 5.839307 2.665966 0.154684
C -4.674547 0.566659 0.598647
C -4.851176 1.830695 1.396183
C -5.701942 -0.202315 0.174416
C -7.186409 0.042565 0.342548
C -7.872588 -0.022428 -1.036172
C -9.350452 0.342211 -0.995678
C -7.755765 -0.983727 1.324209
H -1.720383 -2.671156 -0.311404
H -2.405171 2.116390 0.390931
H -0.011239 2.570601 0.043361
H 2.320951 3.050900 -0.658990
H 1.706976 -2.714009 -1.054752
H 5.209512 0.350733 -2.033280
H 3.720836 -0.785434 1.585150
H 4.655766 0.668004 1.725572
H 6.683060 -2.991142 1.209380
H 4.912758 -2.982866 1.130311
H 5.880887 -2.486162 -0.292609
H 5.408478 2.859242 1.142434
H 6.492855 1.790394 0.179741
H 6.449859 3.530429 -0.122529
H -4.728298 2.710325 0.755848
H -5.830886 1.898249 1.875989
H -4.120602 1.884759 2.211568
H -5.468493 -1.118809 -0.372173
H -7.368779 1.040762 0.752627
H -7.369594 0.670337 -1.723492
H -7.766593 -1.025452 -1.468919
H -9.500458 1.326704 -0.541593
H -9.756495 0.373136 -2.011977
H -9.930658 -0.393408 -0.431267
H -8.804609 -0.772273 1.554490
H -7.206916 -0.964998 2.272815
H -7.696143 -2.001124 0.921429
SCF Energy (B3LYP/6-31G**)= -1361.69401988
Number of imaginary frequencies = 0

1b_c440
MMFF Geometry
C -1.708410 -2.377422 -0.177687
C -2.434419 0.241191 -0.225587
N -3.030283 -2.083601 -0.131727
C -0.681444 -1.430346 -0.246529
C -1.069468 -0.078599 -0.272960
C -3.379576 -0.779598 -0.147871
C -0.082758 0.920847 -0.352139
C 1.281682 0.596992 -0.392566
C 1.662280 -0.746163 -0.357109
C 0.689201 -1.756822 -0.295995
C 3.102291 -1.079695 -0.398823
C 4.143993 0.030579 -0.166766
C 3.591577 1.427502 -0.401447
C 2.272117 1.669535 -0.500533
O 1.027611 -3.089217 -0.279241
O 3.434124 -2.254033 -0.589475
O 5.155547 -0.224980 -1.153449
C 4.696493 -0.112378 1.260555
C 6.205899 -0.131093 1.327910
C 6.942386 1.174210 1.193729
O 6.813660 -1.194942 1.463535
O 4.575731 2.397692 -0.467928
C 4.214409 3.714548 -0.866319
C -4.835719 -0.503074 -0.102244
C -5.654263 -1.393185 -0.995828
C -5.319318 0.464010 0.707346
C -6.752240 0.888890 0.965215
C -7.289537 1.762176 -0.183016
C -8.644908 2.388236 0.117206
C -7.678102 -0.271615 1.342028
H -1.485764 -3.442293 -0.157408
H -2.759063 1.276501 -0.263311
H -0.389819 1.965965 -0.382740
H 1.883887 2.670641 -0.653318
H 1.997177 -3.173295 -0.428507
H 5.376094 -1.177032 -1.089651
H 4.353722 -1.048342 1.722710
H 4.325979 0.673387 1.930993
H 7.947940 1.067052 1.610602
H 7.020169 1.451695 0.140855
H 6.424404 1.955359 1.755674
H 3.737310 3.710362 -1.851687
H 3.567702 4.183601 -0.118158
H 5.131070 4.307451 -0.937160
H -5.816942 -2.368180 -0.524638
H -6.627701 -0.963562 -1.239331
H -5.150094 -1.553421 -1.956196
H -4.609479 1.022621 1.319239
H -6.706032 1.528490 1.859120
H -6.576137 2.571101 -0.387382
H -7.371567 1.179664 -1.107476
H -8.614533 2.965240 1.046767
H -8.932311 3.066473 -0.692743
H -9.426630 1.627905 0.204786
H -8.576781 0.102601 1.844565
H -7.186228 -0.962579 2.035649
H -8.010777 -0.840712 0.469866
SCF Energy (B3LYP/6-31G**)= -1361.69673382
Number of imaginary frequencies = 0

1b_c441
MMFF Geometry
C -1.692034 -2.345767 -0.121491
C -2.489624 0.182485 -0.721479
N -3.022096 -2.092369 -0.167286
C -0.690534 -1.399247 -0.358202
C -1.115484 -0.093572 -0.670211
C -3.408479 -0.831622 -0.457494
C -0.156854 0.902155 -0.932971
C 1.213307 0.620676 -0.867891
C 1.630680 -0.674503 -0.546225
C 0.688747 -1.685023 -0.307852
C 3.086367 -0.947087 -0.461011
C 4.040801 0.251898 -0.233945
C 3.490118 1.491632 -0.931018
C 2.190100 1.649856 -1.214937
O 1.070252 -2.972252 -0.015239
O 3.461805 -2.120563 -0.492616
O 5.311960 -0.056850 -0.801481
C 4.144240 0.451253 1.289606
C 5.448369 -0.022749 1.892313
C 5.718614 -1.504873 1.922274
O 6.244366 0.784609 2.377075
O 4.385538 2.416730 -1.453337
C 5.204749 3.054151 -0.473593
C -4.872859 -0.597350 -0.503716
C -5.653442 -1.703101 -1.163296
C -5.402510 0.512438 0.054842
C -6.860387 0.915063 0.113168
C -7.472302 0.653993 1.503622
C -6.862970 1.448035 2.652731
C -7.016333 2.374706 -0.324820
H -1.441159 -3.376342 0.121027
H -2.841065 1.177045 -0.979026
H -0.488101 1.907044 -1.193781
H 1.849324 2.528071 -1.756840
H 2.038723 -3.058764 -0.170438
H 5.241693 0.075777 -1.764269
H 3.329556 -0.045795 1.831951
H 4.042286 1.510683 1.556325
H 6.562402 -1.706228 2.588619
H 4.842671 -2.030805 2.310494
H 5.965254 -1.869566 0.923913
H 4.595224 3.484773 0.327453
H 5.949431 2.364643 -0.068083
H 5.739770 3.870860 -0.967206
H -5.612482 -2.618449 -0.562764
H -6.708773 -1.463693 -1.307485

H -5.242915 -1.920944 -2.155926
H -4.732870 1.211446 0.556523
H -7.442320 0.324456 -0.601583
H -8.546453 0.875152 1.463826
H -7.388226 -0.415227 1.737286
H -6.986158 2.524988 2.507063
H -7.361755 1.183706 3.591136
H -5.797519 1.231896 2.773442
H -8.064605 2.686774 -0.267212
H -6.687999 2.503190 -1.362643
H -6.424330 3.057831 0.293550
SCF Energy (B3LYP/6-31G**)= -1361.69255420
Number of imaginary frequencies = 0

1b_c442
MMFF Geometry
C 1.544203 2.353253 -0.205967
C 2.260516 -0.266986 -0.275349
N 2.865563 2.055258 -0.179524
C 0.512626 1.410313 -0.265966
C 0.896340 0.058065 -0.303638
C 3.210230 0.749982 -0.205878
C -0.094210 -0.937244 -0.369504
C -1.457543 -0.608353 -0.396502
C -1.838427 0.736006 -0.356887
C -0.858505 1.741493 -0.292543
C -3.281918 1.080673 -0.372156
C -4.340855 -0.034664 -0.467623
C -3.758718 -1.428106 -0.517427
C -2.445897 -1.680444 -0.452043
O -1.186827 3.076352 -0.249799
O -3.607552 2.272451 -0.341061
O -4.961489 0.230282 -1.746496
C -5.409615 0.160688 0.617856
C -5.012065 -0.321053 1.997569
C -3.934481 0.432537 2.731444
O -5.560828 -1.292868 2.517697
O -4.628647 -2.509326 -0.487513
C -5.406537 -2.636113 -1.676473
C 4.665878 0.468359 -0.179857
C 5.476551 1.360277 -1.078679
C 5.156015 -0.505024 0.618169
C 6.590318 -0.937005 0.855664
C 7.109612 -1.805996 -0.304049
C 8.466133 -2.439055 -0.024401
C 7.525492 0.217747 1.227115
H 1.326134 3.418774 -0.177130
H 2.580103 -1.303693 -0.320714
H 0.206460 -1.984589 -0.399032
H -2.097088 -2.709872 -0.437672
H -2.164501 3.166871 -0.303144
H -5.259852 1.160049 -1.729498
H -6.328366 -0.370211 0.334164
H -5.713895 1.211468 0.702743
H -4.028804 0.244712 3.804977
H -2.951286 0.097374 2.397746
H -4.048175 1.506694 2.565920
H -6.257518 -1.949562 -1.655361
H -4.800991 -2.479713 -2.575908
H -5.802923 -3.655418 -1.708297
H 5.648718 2.332074 -0.604266
H 6.445273 0.928433 -1.336630
H 4.961093 1.527636 -2.031812
H 4.451763 -1.065150 1.235270
H 6.552799 -1.581351 1.746561
H 6.390459 -2.610954 -0.503867
H 7.182330 -1.218663 -1.226238
H 8.445146 -3.021101 0.902271
H 8.740596 -3.113898 -0.841640
H 9.251938 -1.682309 0.057510
H 8.428924 -0.162775 1.716213
H 7.045165 0.906795 1.930662
H 7.849430 0.790345 0.353956
SCF Energy (B3LYP/6-31G**)= -1361.69120907
Number of imaginary frequencies = 0

1b_c443
MMFF Geometry
C 1.965835 1.801874 -0.111541
C 2.313796 -0.886331 0.046131
N 3.231814 1.325470 -0.032086
C 0.814170 1.010427 -0.117006
C 1.006959 -0.378876 -0.035934
C 3.401287 -0.014232 0.046466
C -0.112996 -1.228987 -0.040943
C -1.417607 -0.718204 -0.121208
C -1.608692 0.664180 -0.198604
C -0.497054 1.524997 -0.200133
C -2.989172 1.205301 -0.275524
C -4.197020 0.246212 -0.210475
C -3.808626 -1.216128 -0.197653
C -2.543405 -1.646329 -0.124071
O -0.634994 2.891358 -0.279024
O -3.130868 2.428505 -0.383855
O -4.902989 0.512202 -1.427477
C -5.002417 0.634623 1.042479
C -6.452742 0.218730 0.977228
C -6.941002 -0.771283 2.000631
O -7.219290 0.724362 0.155640
O -4.826766 -2.158388 -0.124251
C -5.477147 -2.347677 -1.383435
C 4.807166 -0.502405 0.147509
C 5.018712 -1.780845 0.909384
C 5.798500 0.231549 -0.405606
C 7.289796 -0.025309 -0.487236
C 7.989607 0.344547 0.832994
C 9.509298 0.304111 0.743732
C 7.649182 -1.421683 -1.004356
H 1.899288 2.886052 -0.173055
H 2.473238 -1.958639 0.094273
H 0.038979 -2.306594 0.017687
H -2.337743 -2.711829 -0.067617
H -1.590273 3.106596 -0.381399
H -5.443732 1.313929 -1.287006
H -5.022295 1.723372 1.184595
H -4.539007 0.227905 1.949943
H -7.933553 -1.132058 1.717182
H -6.264823 -1.627033 2.053395
H -6.998703 -0.283257 2.976553
H -6.249853 -1.589103 -1.534032
H -4.766014 -2.348541 -2.216955
H -5.971021 -3.323673 -1.357903
H 4.812936 -2.648039 0.273525
H 6.034663 -1.876087 1.297287
H 4.365513 -1.828699 1.788392
H 5.517013 1.165071 -0.899742
H 7.659602 0.672653 -1.253023
H 7.690700 1.358140 1.130233
H 7.669419 -0.323047 1.640913
H 9.872893 0.943469 -0.066801
H 9.949553 0.661704 1.680173
H 9.873582 -0.713506 0.574535
H 8.679392 -1.438418 -1.376824
H 7.001527 -1.715446 -1.837827
H 7.576708 -2.187093 -0.227138
SCF Energy (B3LYP/6-31G**)= -1361.69656070
Number of imaginary frequencies = 0

1b_c444
MMFF Geometry
C -1.545754 -2.364413 -0.349317
C -2.442420 0.163366 0.094520
N -2.872132 -2.188216 -0.138374
C -0.595267 -1.338818 -0.353394
C -1.070694 -0.034138 -0.120909
C -3.308753 -0.927855 0.071905
C -0.163906 1.042168 -0.114701
C 1.206498 0.836377 -0.321311
C 1.671516 -0.461726 -0.538785
C 0.781016 -1.543368 -0.575041
C 3.120366 -0.657109 -0.745438
C 4.099492 0.420873 -0.237627

C 3.458069 1.806795 -0.337730
C 2.122567 1.977980 -0.361545
O 1.212619 -2.824545 -0.819451
O 3.509353 -1.689589 -1.297658
O 5.262311 0.397748 -1.069792
C 4.472066 0.141397 1.227853
C 5.676802 -0.753225 1.447001
C 5.664886 -2.141833 0.867090
O 6.620326 -0.380226 2.148380
O 4.383698 2.834836 -0.354055
C 3.928478 4.174681 -0.486954
C -4.765759 -0.781192 0.301341
C -5.362248 -1.839288 1.190169
C -5.454284 0.221971 -0.284280
C -6.940112 0.496183 -0.192663
C -7.222389 1.846756 0.491130
C -6.783591 1.884624 1.949775
C -7.541540 0.486066 -1.601920
H -1.253720 -3.398353 -0.520960
H -2.830653 1.157955 0.291941
H -0.538164 2.051862 0.052448
H 1.669143 2.962177 -0.406652
H 2.141201 -2.791490 -1.141541
H 5.185992 -0.371341 -1.666366
H 3.630783 -0.289418 1.784902
H 4.716767 1.085137 1.735540
H 6.424519 -2.749381 1.368203
H 4.692508 -2.609110 1.040492
H 5.895056 -2.108724 -0.198801
H 3.380052 4.310920 -1.424564
H 3.315416 4.465373 0.372041
H 4.805677 4.827929 -0.511070
H -4.710149 -2.038044 2.048836
H -6.328660 -1.547816 1.608579
H -5.500086 -2.774130 0.636699
H -4.920295 0.916635 -0.933219
H -7.452128 -0.287465 0.374274
H -6.727599 2.661556 -0.052562
H -8.299543 2.052051 0.452086
H -5.697814 1.790113 2.046589
H -7.075783 2.836658 2.404476
H -7.253898 1.078340 2.521559
H -8.624881 0.643290 -1.561212
H -7.363844 -0.475140 -2.097262
H -7.111660 1.274511 -2.230227
SCF Energy (B3LYP/6-31G**)= -1361.70276250
Number of imaginary frequencies = 0

1b_c445
MMFF Geometry
C -1.639484 -2.130630 -0.757244
C -2.406621 0.469099 -0.548920
N -2.966302 -1.859630 -0.727244
C -0.626910 -1.168950 -0.686362
C -1.035793 0.174219 -0.581614
C -3.337126 -0.566042 -0.614793
C -0.064293 1.190724 -0.521163
C 1.303529 0.888075 -0.543093
C 1.700712 -0.447289 -0.630765
C 0.748664 -1.471686 -0.722360
C 3.146985 -0.744253 -0.643153
C 4.128703 0.299982 -0.073510
C 3.602004 1.714161 -0.328485
C 2.294466 1.966025 -0.529432
O 1.117155 -2.789424 -0.845668
O 3.528286 -1.830312 -1.087355
O 5.380929 0.153286 -0.748442
C 4.297600 0.089206 1.440275
C 5.400588 -0.863924 1.858316
C 5.364172 -2.281788 1.355311
O 6.273208 -0.508610 2.654312
O 4.591718 2.679751 -0.283904
C 4.250145 4.034257 -0.546073
C -4.797882 -0.315457 -0.589494
C -5.586968 -1.148706 -1.563389
C -5.326194 0.573761 0.278499
C -6.788342 0.913640 0.472963
C -7.229056 0.692608 1.933051
C -7.156052 -0.766553 2.366187
C -7.022873 2.371240 0.068225
H -1.401013 -3.188650 -0.843824
H -2.746414 1.498443 -0.486890
H -0.385400 2.230052 -0.456516
H 1.919161 2.971686 -0.684588
H 2.078557 -2.833555 -1.047771
H 5.323856 -0.642040 -1.311839
H 3.366102 -0.252598 1.908393
H 4.543519 1.045462 1.923359
H 6.012386 -2.903740 1.980023
H 4.347840 -2.676377 1.428129
H 5.725711 -2.327901 0.327028
H 3.831098 4.145090 -1.551339
H 3.558416 4.415779 0.211668
H 5.166893 4.629039 -0.495106
H -5.719311 -2.166033 -1.180211
H -6.575895 -0.733843 -1.773152
H -5.073925 -1.206780 -2.530582
H -4.660110 1.100178 0.962577
H -7.426564 0.289854 -0.160432
H -6.618576 1.299411 2.613752
H -8.265028 1.034054 2.051971
H -6.126124 -1.136028 2.365240
H -7.547516 -0.875935 3.382684
H -7.752335 -1.402189 1.703852
H -8.080960 2.635915 0.169791
H -6.737630 2.538957 -0.976452
H -6.442816 3.061319 0.691557
SCF Energy (B3LYP/6-31G**)= -1361.70293127
Number of imaginary frequencies = 0

1b_c446
MMFF Geometry
C -1.754975 -2.229545 -0.102215
C -2.389287 0.391357 0.235535
N -3.057176 -1.911140 0.094588
C -0.704331 -1.307165 -0.142659
C -1.044554 0.045921 0.035520
C -3.362728 -0.605571 0.251555
C -0.031451 1.021427 0.007278
C 1.312977 0.671161 -0.187760
C 1.646367 -0.674082 -0.359012
C 0.645429 -1.658997 -0.347719
C 3.065572 -1.035785 -0.563000
C 4.161864 0.007132 -0.276387
C 3.643499 1.436433 -0.280054
C 2.329960 1.722971 -0.236713
O 0.936192 -2.990432 -0.528852
O 3.338575 -2.182726 -0.931146
O 5.081727 -0.140739 -1.369125
C 4.820711 -0.337977 1.068907
C 6.328988 -0.404241 1.006577
C 7.099601 0.887971 0.982487
O 6.906370 -1.491934 0.950916
O 4.653794 2.381487 -0.300796
C 4.309986 3.748602 -0.490940
C -4.798405 -0.300445 0.467677
C -5.481599 -1.239594 1.422863
C -5.378697 0.727383 -0.189306
C -6.816376 1.209582 -0.170388
C -7.820706 0.129579 -0.615261
C -9.216567 0.680967 -0.877839
C -7.175415 1.875386 1.160223
H -1.569107 -3.293884 -0.230944
H -2.674280 1.427400 0.390819
H -0.301991 2.068839 0.138543
H 1.967366 2.745053 -0.224610
H 1.886740 -3.078672 -0.769873
H 5.272304 -1.098089 -1.448360
H 4.481901 -1.317815 1.432544
H 4.533714 0.361221 1.864489
H 7.103147 1.300977 -0.027961
H 6.657022 1.600451 1.683042

H 8.130645 0.700531 1.295888
H 3.756448 3.888667 -1.425053
H 3.742036 4.129360 0.363720
H 5.239026 4.322208 -0.559355
H -4.864507 -1.401728 2.314382
H -6.438861 -0.860350 1.782656
H -5.663815 -2.209255 0.947318
H -4.763044 1.303644 -0.881837
H -6.861670 2.005342 -0.928696
H -7.460185 -0.342840 -1.538011
H -7.903424 -0.665648 0.132189
H -9.187991 1.492886 -1.611255
H -9.863086 -0.109041 -1.273555
H -9.677903 1.057047 0.039954
H -8.099022 2.456297 1.068230
H -6.389161 2.569431 1.478348
H -7.324513 1.145864 1.961070
SCF Energy (B3LYP/6-31G**)= -1361.69692887
Number of imaginary frequencies = 0

1b_c447
MMFF Geometry
C -1.926192 -1.373894 -0.526558
C -2.242383 1.277883 -0.029694
N -3.181470 -0.905346 -0.327389
C -0.769842 -0.591571 -0.492748
C -0.945090 0.780296 -0.233499
C -3.337193 0.416109 -0.081452
C 0.180253 1.624202 -0.190762
C 1.471593 1.118065 -0.384347
C 1.640393 -0.247407 -0.632784
C 0.528685 -1.098186 -0.702824
C 3.017600 -0.768023 -0.813667
C 4.192975 0.065665 -0.244905
C 3.870772 1.552889 -0.347402
C 2.617599 2.023503 -0.408918
O 0.662580 -2.439664 -0.968376
O 3.157387 -1.876399 -1.334187
O 5.361131 -0.196694 -1.019416
C 4.382140 -0.378919 1.217371
C 5.589765 -1.260257 1.448718
C 5.570227 -2.654382 0.877166
O 6.542043 -0.854679 2.118864
O 4.911132 2.447944 -0.564079
C 5.869996 2.496079 0.492136
C -4.736291 0.895028 0.114812
C -5.015023 2.339893 -0.201806
C -5.685134 0.024861 0.526503
C -7.144770 0.306829 0.805668
C -8.049294 -0.149413 -0.355413
C -8.041975 -1.645336 -0.646188
C -7.554847 -0.321767 2.141389
H -1.873613 -2.442799 -0.722252
H -2.385384 2.332705 0.180869
H 0.042468 2.689537 -0.007429
H 2.436330 3.088081 -0.530702
H 1.590010 -2.616350 -1.247886
H 5.286365 0.317464 -1.843745
H 3.505195 -0.910528 1.608873
H 4.494155 0.487329 1.881443
H 6.381677 -3.239235 1.319920
H 4.623129 -3.140364 1.125001
H 5.707685 -2.626774 -0.204916
H 5.382072 2.664629 1.457611
H 6.479928 1.589584 0.517350
H 6.536594 3.341671 0.298384
H -4.507427 2.643583 -1.124587
H -6.076517 2.540640 -0.370244
H -4.676080 2.985664 0.614865
H -5.391934 -1.012715 0.698767
H -7.296892 1.385590 0.925534
H -9.081368 0.155641 -0.141080
H -7.756041 0.383030 -1.269381
H -8.402213 -2.222937 0.209876
H -8.703822 -1.861863 -1.491347
H -7.041772 -2.000753 -0.910616
H -8.614838 -0.137949 2.346866
H -6.976691 0.111767 2.965503
H -7.388699 -1.404165 2.157804
SCF Energy (B3LYP/6-31G**)= -1361.69316081
Number of imaginary frequencies = 0

1b_c448
MMFF Geometry
C -1.614239 -2.492846 -0.208073
C -2.379015 0.092976 0.139792
N -2.925098 -2.243053 0.025739
C -0.617114 -1.515610 -0.282505
C -1.024155 -0.179779 -0.099291
C -3.296164 -0.955056 0.188599
C -0.068330 0.851529 -0.166067
C 1.284430 0.570285 -0.395822
C 1.681200 -0.759225 -0.562495
C 0.741206 -1.797221 -0.527389
C 3.114927 -1.031440 -0.794994
C 4.133628 0.020149 -0.294868
C 3.578801 1.427634 -0.506859
C 2.252578 1.663503 -0.529272
O 1.106493 -3.106567 -0.724371
O 3.425740 -2.086402 -1.349087
O 5.328969 -0.152937 -1.058212
C 4.414332 -0.267450 1.189319
C 5.848438 -0.086012 1.643102
C 6.502279 1.258406 1.470876
O 6.443895 -1.002678 2.216055
O 4.554535 2.394218 -0.651436
C 4.162286 3.740571 -0.891387
C -4.738482 -0.729063 0.445503
C -5.362957 -1.721033 1.389931
C -5.395250 0.281421 -0.163562
C -6.867451 0.620798 -0.050694
C -7.023892 1.955385 0.700262
C -8.473784 2.329419 0.976097
C -7.482008 0.665693 -1.453159
H -1.375257 -3.546396 -0.338095
H -2.714741 1.112861 0.300319
H -0.391069 1.884472 -0.039608
H 1.842432 2.659355 -0.658039
H 2.037912 -3.133387 -1.043026
H 5.076171 -0.172491 -1.997902
H 4.163903 -1.314088 1.414086
H 3.773107 0.329791 1.849770
H 6.803509 1.406471 0.432842
H 5.818435 2.047300 1.793196
H 7.395494 1.308711 2.100954
H 3.592923 3.822192 -1.822786
H 3.591522 4.136854 -0.045573
H 5.070273 4.341607 -0.996533
H -4.693700 -1.931018 2.232594
H -6.294369 -1.356819 1.830922
H -5.575308 -2.663118 0.873635
H -4.846690 0.927622 -0.849252
H -7.400871 -0.156119 0.506047
H -6.500004 1.892569 1.663019
H -6.547804 2.768319 0.137206
H -8.991876 1.527258 1.510949
H -8.516642 3.231419 1.595159
H -9.017957 2.538756 0.050660
H -8.568392 0.790042 -1.405048
H -7.286873 -0.266223 -1.996210
H -7.073081 1.492141 -2.045308
SCF Energy (B3LYP/6-31G**)= -1361.69571178
Number of imaginary frequencies = 0

1b_c449
MMFF Geometry
C -1.832229 -1.535631 -0.161775
C -2.182822 1.096293 0.410934
N -3.082145 -1.106502 0.135837
C -0.698100 -0.721272 -0.195220
C -0.891172 0.640005 0.101273
C -3.254736 0.204688 0.421452

C 0.210771 1.515095 0.072852
C 1.498869 1.051032 -0.226898
C 1.685829 -0.304389 -0.506480
C 0.594996 -1.185807 -0.509002
C 3.056592 -0.781874 -0.800229
C 4.250695 0.072563 -0.309460
C 3.886799 1.560872 -0.373790
C 2.614730 1.994440 -0.311006
O 0.745105 -2.518439 -0.808974
O 3.173805 -1.872518 -1.362040
O 5.384855 -0.154687 -1.145625
C 4.585964 -0.314985 1.139839
C 5.624325 -1.409438 1.297502
C 5.379713 -2.744559 0.646922
O 6.615241 -1.231782 2.011518
O 4.997306 2.376335 -0.501216
C 4.816622 3.788297 -0.494165
C -4.643727 0.634106 0.758946
C -4.782422 1.841987 1.645947
C -5.688360 -0.078071 0.280384
C -7.167624 0.169408 0.477722
C -7.872769 0.522890 -0.848394
C -7.896831 -0.576207 -1.903689
C -7.807750 -1.033869 1.174174
H -1.764660 -2.599065 -0.381427
H -2.343442 2.147252 0.627672
H 0.055213 2.572202 0.286562
H 2.358623 3.047919 -0.342907
H 1.652347 -2.661372 -1.165007
H 5.492594 0.626963 -1.716546
H 3.693464 -0.616561 1.701691
H 4.996586 0.555996 1.669996
H 6.065117 -3.484433 1.071246
H 4.358109 -3.074105 0.851115
H 5.559562 -2.683701 -0.427485
H 4.219426 4.109005 -1.353627
H 4.363200 4.119692 0.445507
H 5.803139 4.253902 -0.576157
H -4.628812 2.761026 1.070767
H -5.759558 1.910969 2.129206
H -4.051797 1.812863 2.462617
H -5.468194 -0.951361 -0.336865
H -7.323758 1.029679 1.135773
H -8.909577 0.808148 -0.628691
H -7.396268 1.411535 -1.282216
H -8.423759 -1.465565 -1.546502
H -8.421207 -0.220047 -2.796749
H -6.887928 -0.867124 -2.209400
H -8.886109 -0.884602 1.294090
H -7.378919 -1.174303 2.173045
H -7.653041 -1.964123 0.617104
SCF Energy (B3LYP/6-31G**)= -1361.69871282
Number of imaginary frequencies = 0

1b_c450
MMFF Geometry
C 1.678272 2.457930 -0.033683
C 2.368097 -0.106354 -0.617421
N 2.996519 2.149615 -0.084676
C 0.637933 1.551306 -0.258484
C 1.006698 0.226515 -0.562819
C 3.328635 0.871523 -0.366288
C 0.006125 -0.730883 -0.815534
C -1.351576 -0.393215 -0.748591
C -1.708303 0.919490 -0.428632
C -0.727341 1.894058 -0.204548
C -3.146958 1.249306 -0.355725
C -4.133442 0.091537 -0.072543
C -3.675252 -1.174137 -0.795001
C -2.376142 -1.388452 -1.080255
O -1.057742 3.198182 0.072488
O -3.483469 2.425322 -0.498291
O -5.412753 0.496909 -0.562976
C -4.196537 -0.109143 1.450621
C -5.562103 -0.428055 2.024244
C -6.292034 -1.648434 1.531793
O -6.032923 0.257114 2.936379
O -4.703290 -2.045698 -1.096280
C -4.407241 -3.244244 -1.803369
C 4.781411 0.580345 -0.415828
C 5.603302 1.643019 -1.094824
C 5.275220 -0.545325 0.143148
C 6.726156 -0.973542 0.225562
C 7.106950 -1.201102 1.701429
C 8.584045 -1.509393 1.905734
C 6.925440 -2.228728 -0.626807
H 1.470708 3.499522 0.202943
H 2.677850 -1.116278 -0.867978
H 0.297163 -1.749766 -1.069114
H -2.031675 -2.290615 -1.574200
H -2.022276 3.322743 -0.082305
H -5.290116 0.812076 -1.475619
H -3.871985 0.811762 1.955876
H -3.498309 -0.884635 1.789962
H -6.737958 -1.458354 0.554451
H -5.606215 -2.497877 1.487266
H -7.092559 -1.898318 2.234703
H -3.974241 -3.023156 -2.784171
H -3.744636 -3.888960 -1.217273
H -5.346740 -3.781734 -1.962065
H 5.763956 2.493020 -0.423315
H 6.580375 1.279042 -1.422089
H 5.099871 2.006022 -1.998639
H 4.586752 -1.233549 0.634298
H 7.383208 -0.192334 -0.168809
H 6.863726 -0.300713 2.280614
H 6.511660 -2.018511 2.128065
H 9.213231 -0.729259 1.465914
H 8.810800 -1.563647 2.975466
H 8.859346 -2.470171 1.461374
H 7.982493 -2.507241 -0.680714
H 6.582373 -2.062821 -1.654541
H 6.370841 -3.082326 -0.220993
SCF Energy (B3LYP/6-31G**)= -1361.69581993
Number of imaginary frequencies = 0

1b_c451
MMFF Geometry
C -1.601249 -2.175692 -0.371409
C -2.220366 0.397520 0.243259
N -2.902324 -1.871742 -0.146991
C -0.544154 -1.261603 -0.309813
C -0.877270 0.066187 0.011285
C -3.200334 -0.588376 0.148318
C 0.141918 1.030739 0.095492
C 1.483864 0.695735 -0.138044
C 1.814211 -0.624100 -0.458775
C 0.805510 -1.599107 -0.544131
C 3.236304 -0.978476 -0.692899
C 4.324927 0.109750 -0.620455
C 3.795530 1.482486 -0.276361
C 2.504539 1.732661 -0.026947
O 1.084578 -2.910404 -0.850879
O 3.516897 -2.147739 -0.978206
O 4.815284 0.154375 -1.980039
C 5.479986 -0.360763 0.275836
C 5.232083 -0.218309 1.763120
C 4.189696 -1.099820 2.398720
O 5.873160 0.582999 2.443621
O 4.706313 2.509321 -0.069629
C 5.379383 2.909941 -1.261834
C -4.634981 -0.298488 0.390729
C -5.329041 -1.331071 1.235155
C -5.205140 0.799343 -0.151935
C -6.639291 1.287737 -0.085334
C -7.649620 0.270643 -0.649033
C -9.040272 0.858237 -0.854532
C -6.999067 1.806874 1.308862
H -1.422158 -3.221511 -0.612059
H -2.498364 1.412925 0.509094
H -0.119247 2.058703 0.347060
H 2.198829 2.732987 0.268832

H 2.049337 -3.001177 -1.017904
H 5.078930 -0.755821 -2.216253
H 6.389865 0.206096 0.036591
H 5.746879 -1.406452 0.077822
H 4.386947 -1.177194 3.471921
H 3.197450 -0.671011 2.250673
H 4.238538 -2.105323 1.973617
H 6.200548 2.224617 -1.489826
H 4.690072 2.988884 -2.109624
H 5.811962 3.899067 -1.084018
H -4.717195 -1.594046 2.106009
H -6.285289 -0.986280 1.630545
H -5.515801 -2.241705 0.655971
H -4.582136 1.444009 -0.774093
H -6.675541 2.161928 -0.752263
H -7.288661 -0.100878 -1.616660
H -7.741348 -0.600847 0.006709
H -9.002626 1.745204 -1.494689
H -9.690881 0.121249 -1.336452
H -9.502733 1.135320 0.097290
H -7.918031 2.401440 1.277809
H -6.209128 2.455930 1.703605
H -7.156815 0.995361 2.024650
SCF Energy (B3LYP/6-31G**)= -1361.69143608
Number of imaginary frequencies = 0

1b_c452
MMFF Geometry
C 1.931770 1.922542 -0.166295
C 2.347740 -0.758142 -0.275369
N 3.210465 1.474733 -0.186826
C 0.799341 1.104552 -0.194760
C 1.027206 -0.280592 -0.249808
C 3.414663 0.138597 -0.242921
C -0.072269 -1.156639 -0.277917
C -1.390441 -0.675941 -0.251128
C -1.616192 0.702235 -0.195310
C -0.525625 1.589222 -0.170508
C -3.010616 1.210857 -0.159092
C -4.191932 0.217746 -0.124314
C -3.770499 -1.229079 -0.260521
C -2.493813 -1.630032 -0.284630
O -0.697957 2.952953 -0.118335
O -3.184939 2.434613 -0.150574
O -4.961925 0.571239 -1.278616
C -4.944091 0.472202 1.194377
C -6.386043 0.024749 1.159553
C -6.801440 -1.064233 2.112150
O -7.202827 0.579827 0.422653
O -4.762334 -2.201096 -0.224448
C -5.469087 -2.295640 -1.463767
C 4.835840 -0.316241 -0.283692
C 5.101755 -1.612934 -0.996387
C 5.789099 0.459253 0.280064
C 7.284894 0.253423 0.410573
C 7.663056 -1.074169 1.095000
C 9.127677 -1.136545 1.511116
C 8.002339 0.484205 -0.921428
H 1.838102 3.005646 -0.124377
H 2.532012 -1.826983 -0.306635
H 0.106602 -2.230765 -0.322990
H -2.261664 -2.690467 -0.333177
H -1.661643 3.150842 -0.157273
H -5.513576 1.342294 -1.041846
H -4.981775 1.543223 1.434082
H -4.427988 -0.000407 2.039326
H -7.798249 -1.425072 1.844142
H -6.104387 -1.902903 2.056805
H -6.822501 -0.665755 3.129348
H -6.265058 -1.547632 -1.509589
H -4.799702 -2.203841 -2.326299
H -5.939096 -3.282912 -1.502724
H 4.512434 -1.681977 -1.918060
H 6.142498 -1.722560 -1.304975
H 4.850438 -2.464313 -0.355336
H 5.465428 1.395527 0.742179
H 7.625930 1.054079 1.083581
H 7.046465 -1.208637 1.992905
H 7.459405 -1.928869 0.442703
H 9.389730 -0.293758 2.158397
H 9.320444 -2.060728 2.065581
H 9.791938 -1.127537 0.642089
H 9.081026 0.594879 -0.770106
H 7.649278 1.402153 -1.405041
H 7.850500 -0.343317 -1.620295
SCF Energy (B3LYP/6-31G**)= -1361.69686324
Number of imaginary frequencies = 0

1b_c453
MMFF Geometry
C -2.008346 -1.966049 0.005929
C -2.408553 0.706400 -0.265843
N -3.281240 -1.521683 -0.130462
C -0.875448 -1.148667 0.019114
C -1.094556 0.233337 -0.119776
C -3.476211 -0.190178 -0.268431
C 0.005385 1.110831 -0.101361
C 1.316218 0.632010 0.037127
C 1.528464 -0.742893 0.158893
C 0.441247 -1.630267 0.166470
C 2.918627 -1.230924 0.303443
C 4.067529 -0.306656 -0.159310
C 3.710295 1.164271 0.034089
C 2.431623 1.578368 0.110416
O 0.614478 -2.985682 0.319423
O 3.084117 -2.373538 0.739632
O 4.172809 -0.549437 -1.569524
C 5.342331 -0.749658 0.588327
C 6.617304 -0.168417 0.014735
C 7.725350 0.136915 0.986023
O 6.763296 -0.005229 -1.198495
O 4.809615 2.001240 0.093712
C 4.601490 3.408728 0.058939
C -4.888283 0.261294 -0.435730
C -5.099490 1.503610 -1.254997
C -5.880507 -0.471067 0.117855
C -7.378324 -0.251148 0.137676
C -7.856641 1.132095 0.620424
C -7.334358 1.503861 2.002524
C -8.013158 -0.624733 -1.204286
H -1.919473 -3.045484 0.108363
H -2.589374 1.771904 -0.362097
H -0.168139 2.181965 -0.197460
H 2.168987 2.623518 0.228044
H 1.566555 -3.158150 0.500313
H 5.074418 -0.270474 -1.839761
H 5.478250 -1.837091 0.533807
H 5.266015 -0.481051 1.648971
H 8.579553 0.559285 0.449716
H 7.377437 0.866965 1.721050
H 8.037724 -0.783391 1.485334
H 4.063319 3.704078 -0.847538
H 4.075414 3.746713 0.957277
H 5.582588 3.892457 0.041972
H -4.431618 1.519867 -2.124051
H -6.109635 1.575267 -1.661690
H -4.912428 2.399126 -0.653403
H -5.593316 -1.374096 0.662760
H -7.770942 -0.973909 0.868474
H -7.587833 1.920686 -0.088641
H -8.953559 1.127667 0.663007
H -6.247470 1.628098 2.003310
H -7.776530 2.451203 2.327551
H -7.596551 0.738733 2.740122
H -9.105970 -0.620128 -1.126373
H -7.709040 -1.629966 -1.517078
H -7.736162 0.072774 -2.000235
SCF Energy (B3LYP/6-31G**)= -1361.70587411
Number of imaginary frequencies = 0

Cartesian Coordinates of all conformations found for each isomer at the B3LYP/6-31G* level of theory, with SCF energies computed at the PCM/mPW1PW91/6-31+G** level of theory.

1a_c001
B3LYP/6-31G* Geometry
C -1.582748 -2.501345 -0.213254
C -2.382593 0.107251 -0.257065
N -2.876361 -2.250253 -0.156132
C -0.582143 -1.504610 -0.283081
C -0.994032 -0.140209 -0.315655
C -3.285680 -0.944314 -0.152630
C -0.021262 0.892995 -0.418240
C 1.324693 0.600239 -0.472034
C 1.757907 -0.772646 -0.428476
C 0.811564 -1.810636 -0.342809
C 3.160812 -1.090636 -0.479090
C 4.224106 0.013936 -0.639209
C 3.639754 1.409377 -0.645449
C 2.316258 1.657193 -0.581563
O 1.169502 -3.094322 -0.314735
O 3.598424 -2.261993 -0.495789
O 4.811328 -0.188276 -1.934150
C 5.326277 -0.146543 0.431723
C 4.855973 0.042011 1.864539
C 5.932339 0.259654 2.910732
O 3.673257 0.006651 2.153735
O 4.621066 2.340434 -0.726998
C 4.242866 3.708601 -0.814174
C -4.756995 -0.729245 -0.057559
C -5.609848 -1.829234 -0.645616
C -5.236304 0.368646 0.560792
C -6.666297 0.792626 0.782404
C -6.932416 2.185106 0.161302
C -6.814199 2.223468 -1.365116
C -6.983585 0.804639 2.291565
H -1.286689 -3.548118 -0.208564
H -2.739319 1.130649 -0.315999
H -0.354398 1.926954 -0.450806
H 1.941875 2.674784 -0.599165
H 2.166274 -3.114108 -0.371913
H 4.995313 -1.143758 -1.985471
H 6.147086 0.536854 0.194102
H 5.722540 -1.168116 0.347648
H 5.491397 0.233091 3.909181
H 6.718781 -0.500314 2.828322
H 6.412285 1.233883 2.750970
H 3.623077 3.887734 -1.701111
H 3.695310 4.025610 0.082478
H 5.173352 4.272277 -0.895733
H -5.384792 -2.781112 -0.155166
H -6.677967 -1.625172 -0.549443
H -5.381923 -1.970837 -1.709495
H -4.510650 1.054170 1.002813
H -7.346971 0.080689 0.300285
H -7.939777 2.508661 0.457825
H -6.236399 2.911681 0.606049
H -7.532279 1.539240 -1.833828
H -7.013817 3.229623 -1.751076
H -5.812930 1.926787 -1.696309
H -6.839348 -0.186709 2.733988
H -8.020904 1.112979 2.469582
H -6.329891 1.506439 2.825034
SCF Energy (PCM/mPW1PW91/6-31+G**)= -1361.46589757
Number of imaginary frequencies = 0

1a_c002
B3LYP/6-31G* Geometry
C 1.605481 2.463805 -0.271187
C 2.399859 -0.147095 -0.253940
N 2.899035 2.210411 -0.222960
C 0.601972 1.467965 -0.301548
C 1.012269 0.102916 -0.301494
C 3.307073 0.904981 -0.189105
C 0.035245 -0.931740 -0.363889
C -1.308257 -0.633906 -0.407422
C -1.740203 0.739276 -0.394533
C -0.792216 1.776761 -0.352394
C -3.144247 1.055253 -0.431356
C -4.216351 -0.053347 -0.516376
C -3.630321 -1.449892 -0.565069
C -2.309202 -1.685580 -0.494238
O -1.146132 3.061910 -0.357517
O -3.583068 2.224452 -0.491028
O -4.923728 0.187585 -1.744437
C -5.216119 0.082464 0.659584
C -4.607321 -0.098555 2.043325
C -5.536888 -0.577016 3.139896
O -3.433393 0.147753 2.251969
O -4.496364 -2.511514 -0.522257
C -5.376265 -2.709289 -1.640870
C 4.778939 0.690320 -0.106861
C 5.626225 1.781271 -0.719376
C 5.264483 -0.398971 0.522040
C 6.696330 -0.821246 0.733757
C 6.955985 -2.218999 0.121449
C 6.824816 -2.269241 -1.403534
C 7.027361 -0.820901 2.239971
H 1.312250 3.511130 -0.291477
H 2.752715 -1.172806 -0.289856
H 0.363901 -1.967387 -0.374834
H -1.969877 -2.717508 -0.490401
H -2.142557 3.084323 -0.403875
H -5.059122 1.153246 -1.772383
H -6.044200 -0.614481 0.501114
H -5.634100 1.098411 0.620150
H -5.038152 -0.509149 4.108683
H -6.465190 0.007162 3.152772
H -5.817780 -1.620803 2.947670
H -6.111696 -1.906037 -1.729804
H -4.802372 -2.771494 -2.572600
H -5.876205 -3.661834 -1.452192
H 5.411984 2.739461 -0.236381
H 6.694879 1.574544 -0.636373
H 5.383327 1.912029 -1.781248
H 4.543705 -1.078202 0.981405
H 7.372661 -0.113564 0.239490
H 7.965617 -2.540748 0.412052
H 6.263379 -2.941514 0.577891
H 7.539593 -1.589503 -1.883679
H 7.020186 -3.278620 -1.783091
H 5.821030 -1.974338 -1.728664
H 6.887258 0.174022 2.675665
H 8.066312 -1.127703 2.410694
H 6.378816 -1.518414 2.785180
SCF Energy (PCM/mPW1PW91/6-31+G**)= -1361.45632325
Number of imaginary frequencies = 0

1a_c003
B3LYP/6-31G* Geometry
C -1.628146 -2.328904 -0.104203
C -2.316708 0.264252 0.393670
N -2.893360 -2.064966 0.158932
C -0.606897 -1.352152 -0.157973
C -0.958721 0.003195 0.109927
C -3.252150 -0.763702 0.385508
C 0.042003 1.014179 0.088517
C 1.356881 0.707602 -0.189512
C 1.729207 -0.657111 -0.460468
C 0.754238 -1.671745 -0.449184
C 3.100162 -0.990031 -0.746004
C 4.185030 0.101507 -0.833305
C 3.668279 1.484761 -0.503716
C 2.375202 1.744095 -0.224643
O 1.054529 -2.945053 -0.704635
O 3.478882 -2.146225 -1.034934
O 4.598291 0.137837 -2.208317
C 5.402265 -0.283466 0.036922
C 5.117960 -0.372435 1.527204
C 6.320943 -0.375123 2.450847
O 3.978921 -0.450580 1.951624
O 4.673096 2.393537 -0.537256

C 4.348601 3.761457 -0.322658
C -4.699566 -0.526103 0.647151
C -5.432641 -1.666647 1.313460
C -5.267505 0.634053 0.260866
C -6.686370 1.110951 0.442408
C -7.373903 1.352036 -0.923162
C -7.561374 0.084339 -1.761480
C -6.697230 2.401657 1.287065
H -1.372845 -3.370790 -0.285035
H -2.620286 1.276810 0.639720
H -0.245180 2.042374 0.292021
H 2.048380 2.754087 -0.003404
H 2.035496 -2.975409 -0.889052
H 4.729828 -0.793966 -2.461026
H 6.215331 0.419501 -0.167600
H 5.743467 -1.275113 -0.291670
H 6.004733 -0.592951 3.472912
H 7.065044 -1.111573 2.124044
H 6.810646 0.606903 2.423239
H 3.631325 4.115928 -1.072836
H 3.932425 3.916874 0.680814
H 5.285903 4.310929 -0.421364
H -4.996564 -1.888350 2.295894
H -6.494862 -1.455766 1.450448
H -5.325652 -2.577723 0.717357
H -4.636454 1.350430 -0.269029
H -7.269948 0.354074 0.980280
H -8.351562 1.819087 -0.740338
H -6.785669 2.087747 -1.491209
H -8.187877 -0.647602 -1.236736
H -8.046958 0.312048 -2.717225
H -6.600981 -0.396523 -1.976373
H -6.249115 2.234583 2.272531
H -7.722237 2.762676 1.433773
H -6.129988 3.200354 0.792066
SCF Energy (PCM/mPW1PW91/6-31+G**)= -1361.46592309
Number of imaginary frequencies = 0

1a_c005
B3LYP/6-31G* Geometry
C -1.878634 -1.529718 -0.604098
C -2.124055 1.138009 -0.094865
N -3.088368 -1.037273 -0.459423
C -0.689362 -0.764046 -0.511904
C -0.813363 0.629061 -0.246104
C -3.229990 0.302812 -0.202520
C 0.350870 1.439116 -0.148055
C 1.610030 0.896469 -0.299126
C 1.751790 -0.511278 -0.563121
C 0.609749 -1.328351 -0.674290
C 3.059822 -1.089473 -0.720314
C 4.329369 -0.216547 -0.669852
C 4.044216 1.235900 -0.357174
C 2.798482 1.727997 -0.204912
O 0.696919 -2.634107 -0.930807
O 3.248850 -2.293666 -1.000349
O 4.882521 -0.247905 -1.995026
C 5.359022 -0.827194 0.306996
C 4.916918 -0.863365 1.760505
C 5.999256 -1.107011 2.794859
O 3.748856 -0.714817 2.072295
O 5.196543 1.942703 -0.261741
C 5.107474 3.343487 -0.033390
C -4.617902 0.814126 -0.045118
C -4.778410 2.289859 0.239957
C -5.650448 -0.046341 -0.165328
C -7.129638 0.215157 -0.068085
C -7.775597 -0.678483 1.017881
C -7.283347 -0.391981 2.439463
C -7.792785 -0.039487 -1.438323
H -1.804091 -2.596216 -0.806654
H -2.245536 2.196149 0.107803
H 0.237413 2.501688 0.050113
H 2.640235 2.779435 0.008041
H 1.669058 -2.843164 -1.019607
H 4.865143 -1.186224 -2.257219
H 6.305929 -0.289984 0.197586
H 5.538683 -1.864539 -0.008173
H 5.547433 -1.260715 3.776833
H 6.612490 -1.975230 2.524667
H 6.672894 -0.241287 2.836154
H 4.554152 3.837864 -0.841096
H 4.619356 3.557126 0.925970
H 6.134846 3.709976 -0.013618
H -4.343201 2.901336 -0.561260
H -5.826888 2.575640 0.336488
H -4.271832 2.575574 1.171040
H -5.386116 -1.081499 -0.377535
H -7.319743 1.260100 0.207895
H -8.864952 -0.542245 0.972328
H -7.587478 -1.732327 0.767016
H -7.491870 0.645694 2.729182
H -7.778875 -1.045129 3.166747
H -6.202786 -0.549528 2.526528
H -7.378032 0.619732 -2.208370
H -8.874534 0.133417 -1.384208
H -7.633427 -1.074709 -1.764165
SCF Energy (PCM/mPW1PW91/6-31+G**)= -1361.46786143
Number of imaginary frequencies = 0

1a_c006
B3LYP/6-31G* Geometry
C -1.886344 -1.352759 -0.879368
C -2.132606 1.162743 0.145781
N -3.096313 -0.893159 -0.652463
C -0.696689 -0.625509 -0.624313
C -0.822496 0.688586 -0.091949
C -3.239297 0.368590 -0.134621
C 0.343401 1.461346 0.171487
C 1.599888 0.950157 -0.069705
C 1.744048 -0.381367 -0.595021
C 0.603184 -1.154984 -0.878505
C 3.054452 -0.924535 -0.835827
C 4.330274 -0.096600 -0.567000
C 4.040724 1.304264 -0.066478
C 2.793685 1.745693 0.170767
O 0.689226 -2.386283 -1.383428
O 3.248607 -2.045505 -1.354834
O 5.002298 -0.000541 -1.833855
C 5.256481 -0.851273 0.420474
C 4.669204 -1.083858 1.805826
C 5.654378 -1.224334 2.948426
O 3.466500 -1.168338 1.974507
O 5.102405 2.086534 0.306977
C 6.029120 2.498806 -0.710746
C -4.627661 0.841390 0.111635
C -4.787560 2.218074 0.714948
C -5.660495 0.035502 -0.212472
C -7.140241 0.278882 -0.083163
C -7.809298 -0.838564 0.752578
C -7.341731 -0.894508 2.209849
C -7.777521 0.356795 -1.486676
H -1.812019 -2.358244 -1.288490
H -2.253975 2.160385 0.552336
H 0.232095 2.467179 0.567271
H 2.669702 2.746575 0.574320
H 1.660535 -2.580778 -1.500012
H 4.932428 -0.892263 -2.223531
H 6.210789 -0.320469 0.483403
H 5.462887 -1.839908 -0.013741
H 5.135010 -1.549253 3.852070
H 6.451445 -1.934623 2.696621
H 6.135757 -0.255165 3.133651
H 6.591774 1.655162 -1.117443
H 5.500799 3.001770 -1.528768
H 6.704043 3.203511 -0.220077
H -4.358769 2.992740 0.065719
H -5.835540 2.473286 0.879172
H -4.274463 2.291933 1.682460
H -5.395710 -0.928789 -0.644293
H -7.331340 1.231937 0.426026
H -8.897193 -0.687305 0.721537

H -7.619904 -1.806095 0.265825
H -7.554810 0.047873 2.730194
H -7.849912 -1.697246 2.756118
H -6.262817 -1.072652 2.275575
H -7.343710 1.172932 -2.074279
H -8.859077 0.523256 -1.413264
H -7.617524 -0.575805 -2.041653
SCF Energy (PCM/mPW1PW91/6-31+G**)= -1361.45816628
Number of imaginary frequencies = 0

1a_c010
B3LYP/6-31G* Geometry
C -1.502875 -2.422988 -0.243533
C -2.271153 0.128255 0.345747
N -2.777720 -2.205710 0.015917
C -0.509947 -1.416087 -0.249006
C -0.903659 -0.082685 0.066687
C -3.176398 -0.924966 0.288333
C   0.067459 0.956721 0.094609
C   1.393324 0.698988 -0.180496
C   1.807826 -0.643059 -0.499449
C   0.862638 -1.684881 -0.537756
C   3.190514 -0.925076 -0.782998
C   4.243940 0.199733 -0.816447
C   3.684214 1.553564 -0.438465
C   2.381370 1.764816 -0.164541
O   1.202335 -2.938092 -0.839253
O   3.605543 -2.057720 -1.112591
O   4.668056 0.302418 -2.184932
C   5.464211 -0.184126 0.049742
C   5.169753 -0.341117 1.532346
C   6.364752 -0.352491 2.466293
O   4.029523 -0.463986 1.942767
O   4.662654 2.491253 -0.425151
C   4.296374 3.840029 -0.161989
C -4.632365 -0.740213 0.543764
C -5.339730 -1.931709 1.146087
C -5.229990 0.419431 0.204360
C -6.670167 0.832758 0.374607
C -7.334657 1.004586 -1.014477
C -8.835835 1.313484 -0.973647
C -6.741975 2.122122 1.218223
H -1.215756 -3.449420 -0.461304
H -2.606261 1.121494 0.627050
H -0.251571 1.967622 0.334146
H   2.023056 2.755688 0.091637
H   2.185324 -2.932886 -1.015051
H   4.827956 -0.614452 -2.473374
H   6.257728 0.550220 -0.118248
H   5.837923 -1.151135 -0.314907
H   6.045530 -0.620305 3.475463
H   7.129828 -1.056097 2.116385
H   6.829890 0.641645 2.484019
H   3.578573 4.203249 -0.907502
H   3.863223 3.944219 0.840919
H   5.218657 4.419182 -0.226562
H -4.894120 -2.201110 2.111992
H -6.405149 -1.750019 1.300090
H -5.218950 -2.805580 0.498852
H -4.615833 1.177845 -0.284487
H -7.221227 0.050601 0.910726
H -6.809591 1.799180 -1.564637
H -7.173595 0.081902 -1.586473
H -9.046984 2.281075 -0.505369
H -9.252363 1.342953 -1.986946
H -9.383430 0.545388 -0.413194
H -6.266433 1.976349 2.194198
H -7.777906 2.431702 1.392556
H -6.225109 2.949144 0.713909
SCF Energy (PCM/mPW1PW91/6-31+G**)= -1361.46503572
Number of imaginary frequencies = 0

1a_c011
B3LYP/6-31G* Geometry
C   1.496418 2.530261 -0.110534
C   2.332119 -0.052861 -0.390630
N   2.794681 2.297717 -0.124005
C   0.508296 1.524503 -0.220372
C   0.939902 0.174989 -0.376862
C   3.223583 1.003516 -0.238588
C -0.021964 -0.864738 -0.526947
C -1.370630 -0.588572 -0.506812
C -1.823758 0.766882 -0.333419
C -0.891359 1.811103 -0.201918
C -3.232885 1.058562 -0.301403
C -4.289259 -0.053409 -0.483147
C -3.682796 -1.424803 -0.701887
C -2.356684 -1.642371 -0.687063
O -1.265374 3.082430 -0.057490
O -3.691636 2.218905 -0.220950
O -5.032577 0.309569 -1.658936
C -5.260374 -0.067375 0.724048
C -4.615394 -0.387263 2.065533
C -5.513332 -0.991871 3.125799
O -3.439274 -0.150136 2.272042
O -4.529718 -2.500922 -0.759067
C -5.446253 -2.580986 -1.862876
C   4.699593 0.805897 -0.217131
C   5.514049 1.955250 -0.763707
C   5.215996 -0.323939 0.306094
C   6.660065 -0.740269 0.429670
C   6.870740 -2.102193 -0.277539
C   8.328245 -2.574415 -0.333821
C   7.063214 -0.786363 1.918202
H   1.185938 3.568063 -0.011326
H   2.701622 -1.061768 -0.543932
H   0.322546 -1.886872 -0.658037
H -2.000861 -2.661823 -0.805810
H -2.262885 3.091332 -0.075338
H -5.181859 1.270040 -1.576354
H -6.080645 -0.758613 0.509549
H -5.697001 0.938217 0.806087
H -4.993308 -1.018421 4.085367
H -6.448402 -0.426280 3.219855
H -5.785277 -2.013939 2.831193
H -6.201607 -1.792366 -1.825409
H -4.906637 -2.511767 -2.814263
H -5.918233 -3.562190 -1.777412
H   5.284872 2.871917 -0.211996
H   6.588274 1.768741 -0.708276
H   5.253635 2.153602 -1.811132
H   4.517728 -1.046258 0.732543
H   7.302851 -0.006835 -0.072094
H   6.256499 -2.863786 0.225120
H   6.480538 -2.022001 -1.300636
H   8.737770 -2.766944 0.663543
H   8.412311 -3.504171 -0.907926
H   8.968334 -1.826756 -0.818679
H   6.904379 0.187393 2.393426
H   8.118111 -1.053180 2.043486
H   6.461995 -1.525330 2.463543
SCF Energy (PCM/mPW1PW91/6-31+G**)= -1361.45548508
Number of imaginary frequencies = 0

1a_c014
B3LYP/6-31G* Geometry
C   1.581189 2.553608 -0.126714
C   2.417013 -0.036374 -0.329481
N   2.879175 2.319383 -0.118052
C   0.593135 1.546424 -0.222802
C   1.025037 0.192899 -0.337564
C   3.308425 1.022518 -0.194813
C   0.064018 -0.849966 -0.470057
C -1.284551 -0.572547 -0.473281
C -1.738163 0.787651 -0.344144
C -0.806351 1.834490 -0.230342
C -3.147322 1.081137 -0.337845
C -4.202635 -0.034857 -0.500795
C -3.595072 -1.412874 -0.669153
C -2.269376 -1.630595 -0.633503
O -1.180779 3.109663 -0.126724
O -3.605772 2.243648 -0.296173

O -4.927834 0.292543 -1.698302
C -5.190830 -0.009969 0.692054
C -4.565492 -0.290690 2.051418
C -5.482380 -0.850054 3.120278
O -3.389616 -0.058240 2.264528
O -4.441761 -2.490036 -0.701980
C -5.351320 -2.601798 -1.808604
C   4.784464 0.825718 -0.153597
C   5.603399 1.967176 -0.709769
C   5.296716 -0.298644 0.386977
C   6.744056 -0.704458 0.521961
C   7.138443 -1.791479 -0.516967
C   6.362195 -3.111501 -0.438584
C   7.044900 -1.148254 1.966804
H   1.271021 3.593993 -0.058285
H   2.785568 -1.049697 -0.452852
H   0.409026 -1.875645 -0.568066
H -1.913278 -2.653391 -0.717541
H -2.177971 3.118771 -0.157640
H -5.078289 1.254991 -1.647234
H -6.010315 -0.705208 0.487496
H -5.625524 0.998755 0.737556
H -4.974546 -0.847934 4.086717
H -6.413163 -0.273170 3.182865
H -5.760135 -1.879335 2.857995
H -6.105757 -1.811400 -1.799709
H -4.805776 -2.561867 -2.758267
H -5.825468 -3.579298 -1.696690
H   5.364294 2.894821 -0.181235
H   6.677194 1.783559 -0.639169
H   5.356718 2.142348 -1.764628
H   4.591645 -1.016541 0.806388
H   7.382332 0.160796 0.309719
H   7.016394 -1.364773 -1.520952
H   8.211295 -1.998205 -0.396175
H   5.290192 -2.961439 -0.610126
H   6.720751 -3.811451 -1.201783
H   6.480231 -3.601086 0.534698
H   6.866461 -0.328249 2.670841
H   8.090734 -1.462466 2.068771
H   6.411233 -1.988543 2.274022
SCF Energy (PCM/mPW1PW91/6-31+G**)= -1361.45405843
Number of imaginary frequencies = 0

1a_c016
B3LYP/6-31G* Geometry
C -1.812309 -1.440769 -0.704304
C -2.060074 1.160429 0.077402
N -3.022400 -0.963081 -0.519465
C -0.623109 -0.690777 -0.523460
C -0.749874 0.667021 -0.115762
C -3.166531 0.342618 -0.125771
C   0.415827 1.461826 0.073477
C   1.672282 0.932408 -0.124083
C   1.817262 -0.440663 -0.527926
C   0.676770 -1.238942 -0.734130
C   3.127671 -1.000144 -0.729295
C   4.402365 -0.147262 -0.548708
C   4.112070 1.290298 -0.167858
C   2.865890 1.748153 0.039783
O   0.763211 -2.510685 -1.126002
O   3.320986 -2.162739 -1.147018
O   5.058141 -0.156663 -1.827808
C   5.344707 -0.810491 0.487214
C   4.773697 -0.930263 1.893407
C   5.771477 -0.970104 3.032929
O   3.573331 -1.007003 2.081720
O   5.177464 2.102975 0.122861
C   6.055659 2.468235 -0.954538
C -4.555132 0.836600 0.072039
C -4.716365 2.274502 0.509151
C -5.586379 -0.006389 -0.143934
C -7.065054 0.242943 -0.013852
C -7.648067 -0.674721 1.091378
C -9.128539 -0.431903 1.406887
C -7.746692 0.013915 -1.379217
H -1.737529 -2.480631 -1.015748
H -2.181698 2.191852 0.388453
H   0.304210 2.499400 0.376367
H   2.741896 2.779991 0.356025
H   1.734460 -2.712355 -1.231195
H   4.993074 -1.079616 -2.137060
H   6.295584 -0.269907 0.494159
H   5.553045 -1.830900 0.135236
H   5.264222 -1.225120 3.965421
H   6.572616 -1.691559 2.830697
H   6.245770 0.014864 3.134143
H   6.608133 1.608399 -1.340830
H   5.486528 2.923809 -1.773191
H   6.744270 3.203195 -0.531969
H -4.275297 2.966937 -0.219758
H -5.765087 2.550571 0.629082
H -4.216008 2.459715 1.468613
H -5.320988 -1.015148 -0.456429
H -7.255622 1.280962 0.286662
H -7.498941 -1.723591 0.797790
H -7.058419 -0.528445 2.005557
H -9.776512 -0.662698 0.554377
H -9.453356 -1.060168 2.244117
H -9.307886 0.613418 1.688522
H -7.319151 0.671990 -2.143291
H -8.822719 0.211787 -1.329156
H -7.608167 -1.021384 -1.715327
SCF Energy (PCM/mPW1PW91/6-31+G**)= -1361.45728155
Number of imaginary frequencies = 0

1a_c017
B3LYP/6-31G* Geometry
C -1.805267 -1.546270 -0.529807
C -2.048015 1.134153 -0.091792
N -3.014293 -1.049874 -0.392759
C -0.615407 -0.778732 -0.463796
C -0.738167 0.621013 -0.234922
C -3.154702 0.296690 -0.171503
C   0.426511 1.433191 -0.162945
C   1.685005 0.886445 -0.303777
C   1.825636 -0.527847 -0.530894
C   0.683051 -1.347368 -0.616026
C   3.133104 -1.110318 -0.676300
C   4.402931 -0.236641 -0.652302
C   4.119042 1.223681 -0.377571
C   2.873841 1.719951 -0.235160
O   0.769293 -2.659602 -0.837582
O   3.321294 -2.321589 -0.924687
O   4.952797 -0.303199 -1.977607
C   5.434989 -0.821412 0.337777
C   4.995839 -0.820832 1.792632
C   6.080236 -1.039045 2.830547
O   3.828475 -0.664231 2.103060
O   5.271848 1.932389 -0.303777
C   5.183607 3.338603 -0.111508
C -4.541504 0.812644 -0.020602
C -4.699120 2.297103 0.217364
C -5.574257 -0.052117 -0.100105
C -7.050729 0.213828 0.024104
C -7.611593 -0.551573 1.250004
C -9.084738 -0.264200 1.562727
C -7.759477 -0.188537 -1.286106
H -1.731780 -2.617881 -0.703792
H -2.168210 2.197351 0.083180
H   0.313840 2.500705 0.007171
H   2.716262 2.776723 -0.049878
H   1.741113 -2.871168 -0.923707
H   4.934489 -1.248096 -2.214907
H   6.381283 -0.286530 0.212794
H   5.614814 -1.866363 0.048937
H   5.630343 -1.168234 3.816932
H   6.692571 -1.913935 2.580647
H   6.754287 -0.172846 2.849101
H   4.628217 3.812113 -0.930223
H   4.698036 3.576998 0.843303
H   6.211074 3.705291 -0.103912

H -4.263902 2.882147 -0.603320
H -5.746754 2.588520 0.306763
H -4.190426 2.611432 1.138111
H -5.311529 -1.094067 -0.276373
H -7.235341 1.282444 0.191521
H -7.470943 -1.630048 1.089974
H -7.002284 -0.292137 2.125375
H -9.750402 -0.595990 0.758613
H -9.393645 -0.782103 2.477896
H -9.256003 0.808845 1.715793
H -7.345143 0.361351 -2.137934
H -8.833883 0.019716 -1.242221
H -7.630587 -1.259722 -1.486018
SCF Energy (PCM/mPW1PW91/6-31+G**)= -1361.46696887
Number of imaginary frequencies = 0

1a_c019
B3LYP/6-31G* Geometry
C -1.585254 -2.401637 -0.294104
C -2.329501 0.122517 0.428075
N -2.854715 -2.190463 -0.004857
C -0.586551 -1.400859 -0.266311
C -0.967709 -0.081908 0.117935
C -3.241527 -0.922415 0.335742
C 0.009763 0.950003 0.180371
C 1.330258 0.698362 -0.124647
C 1.732207 -0.629359 -0.511954
C 0.780311 -1.662990 -0.586751
C 3.109526 -0.905416 -0.826051
C 4.170267 0.213063 -0.816324
C 3.622788 1.551670 -0.372209
C 2.324591 1.757160 -0.072934
O 1.108169 -2.902322 -0.952083
O 3.513379 -2.023034 -1.215236
O 4.585562 0.375390 -2.181558
C 5.393996 -0.217839 0.022772
C 5.108374 -0.442905 1.498345
C 6.308844 -0.499336 2.423543
O 3.970433 -0.583047 1.909631
O 4.606697 2.482628 -0.328755
C 4.251073 3.820468 -0.002520
C -4.691068 -0.744438 0.627711
C -5.396072 -1.963957 1.174084
C -5.291080 0.434637 0.366975
C -6.729310 0.827629 0.605099
C -7.577616 0.754608 -0.694605
C -7.108436 1.644235 -1.852193
C -6.798376 2.219389 1.263778
H -1.307576 -3.417891 -0.565282
H -2.653870 1.102854 0.762237
H -0.299972 1.950283 0.471214
H 1.975180 2.737337 0.232086
H 2.088651 -2.894368 -1.141004
H 4.734531 -0.528605 -2.513353
H 6.190929 0.518907 -0.116232
H 5.759515 -1.168828 -0.389391
H 5.995339 -0.814545 3.420740
H 7.071467 -1.185776 2.036172
H 6.774774 0.492670 2.485794
H 3.526764 4.219605 -0.722949
H 3.830046 3.882882 1.008989
H 5.175787 4.397167 -0.052514
H -4.927703 -2.300358 2.107571
H -6.455154 -1.781322 1.366777
H -5.304256 -2.795837 0.469167
H -4.683450 1.214563 -0.092404
H -7.183054 0.117212 1.306137
H -7.594454 -0.289326 -1.033113
H -8.614113 1.014293 -0.436174
H -6.098275 1.376528 -2.181563
H -7.775490 1.532735 -2.714447
H -7.103050 2.705559 -1.578673
H -6.284863 2.218862 2.231698
H -7.839593 2.519569 1.432801
H -6.326017 2.988058 0.640747
SCF Energy (PCM/mPW1PW91/6-31+G**)= -1361.46366046
Number of imaginary frequencies = 0

1a_c021
B3LYP/6-31G* Geometry
C -1.900222 -1.404495 -0.673273
C -2.089888 1.239641 -0.034350
N -3.099416 -0.894932 -0.502356
C -0.695145 -0.668978 -0.546137
C -0.790201 0.711455 -0.211526
C -3.213170 0.434060 -0.181348
C 0.390682 1.490897 -0.072738
C 1.638249 0.929993 -0.250497
C 1.750815 -0.465095 -0.585894
C 0.591927 -1.251277 -0.738368
C 3.046532 -1.061682 -0.773188
C 4.334201 -0.219413 -0.677984
C 4.079187 1.220428 -0.289720
C 2.843855 1.730230 -0.113019
O 0.651754 -2.543575 -1.061972
O 3.210335 -2.253337 -1.115778
O 4.886931 -0.192736 -2.003423
C 5.350340 -0.902073 0.264597
C 4.905564 -1.007645 1.713891
C 5.981380 -1.326744 2.734388
O 3.739987 -0.854084 2.032502
O 5.245939 1.897071 -0.157446
C 5.185744 3.286005 0.141996
C -4.589656 0.965764 0.004183
C -4.719105 2.432872 0.344321
C -5.639795 0.128599 -0.129948
C -7.109165 0.427417 0.019798
C -7.657525 -0.079242 1.383948
C -7.514324 -1.583460 1.646362
C -7.894656 -0.156639 -1.171087
H -1.847927 -2.461227 -0.927488
H -2.188649 2.288483 0.221917
H 0.299531 2.544196 0.179175
H 2.707678 2.772332 0.154064
H 1.619299 -2.767941 -1.162863
H 4.850051 -1.115479 -2.314392
H 6.308129 -0.379134 0.184471
H 5.509289 -1.924424 -0.105844
H 5.525031 -1.524631 3.706316
H 6.579081 -2.190394 2.418516
H 6.670761 -0.477079 2.822789
H 4.644875 3.832367 -0.640263
H 4.699945 3.460938 1.110342
H 6.220546 3.629260 0.181760
H -4.257662 3.065259 -0.425248
H -5.761632 2.740347 0.440600
H -4.219604 2.668842 1.293138
H -5.394646 -0.902822 -0.373372
H -7.270270 1.512262 0.012501
H -7.145378 0.471732 2.183551
H -8.719020 0.200370 1.444857
H -6.463856 -1.893735 1.643909
H -7.933654 -1.841416 2.625498
H -8.042978 -2.182582 0.896407
H -7.560566 0.288867 -2.114549
H -8.968262 0.040516 -1.063562
H -7.757518 -1.240795 -1.253241
SCF Energy (PCM/mPW1PW91/6-31+G**)= -1361.46555508
Number of imaginary frequencies = 0

1a_c022
B3LYP/6-31G* Geometry
C -1.909185 -1.280594 -0.807390
C -2.096332 1.256926 0.175109
N -3.107668 -0.792534 -0.579265
C -0.703066 -0.572880 -0.575662
C -0.798059 0.752234 -0.064792
C -3.221248 0.481302 -0.084003
C 0.385440 1.505584 0.175689
C 1.629227 0.965571 -0.068527
C 1.742178 -0.376930 -0.572882
C 0.583576 -1.132452 -0.832488

C 3.039173 -0.949551 -0.818045
C 4.333375 -0.141338 -0.579279
C 4.076304 1.272340 -0.097415
C 2.840822 1.741405 0.146909
O 0.640447 -2.373487 -1.317253
O 3.205842 -2.083046 -1.319106
O 4.989328 -0.077058 -1.856543
C 5.259085 -0.897395 0.407206
C 4.685904 -1.098839 1.803221
C 5.683420 -1.247496 2.934058
O 3.484032 -1.153580 1.989298
O 5.157770 2.039395 0.250345
C 6.071653 2.427550 -0.788359
C -4.598019 0.987011 0.160983
C -4.726339 2.376523 0.741843
C -5.649058 0.194961 -0.138563
C -7.120281 0.478076 0.019040
C -7.718378 -0.276820 1.240014
C -7.605888 -1.805871 1.205200
C -7.867817 0.145868 -1.287981
H -1.858551 -2.294795 -1.198205
H -2.193928 2.263443 0.566028
H 0.297923 2.519985 0.555269
H 2.740654 2.750709 0.535881
H 1.606629 -2.589015 -1.439731
H 4.900170 -0.973849 -2.230248
H 6.223350 -0.382739 0.449807
H 5.442475 -1.895957 -0.014436
H 5.169566 -1.550277 3.848475
H 6.461261 -1.977977 2.680014
H 6.188262 -0.287014 3.101147
H 6.612404 1.571281 -1.198463
H 5.535994 2.932610 -1.600336
H 6.767514 3.124946 -0.316844
H -4.279970 3.132066 0.081991
H -5.768124 2.657157 0.904774
H -4.211519 2.452387 1.708305
H -5.403012 -0.782726 -0.546791
H -7.275261 1.545612 0.216859
H -7.225133 0.096081 2.147115
H -8.777219 0.006603 1.324005
H -6.561122 -2.133186 1.171990
H -8.060128 -2.241646 2.102358
H -8.119749 -2.235268 0.337806
H -7.504842 0.767678 -2.113663
H -8.944351 0.323871 -1.176070
H -7.727896 -0.901154 -1.579430
SCF Energy (PCM/mPW1PW91/6-31+G**)= -1361.45592545
Number of imaginary frequencies = 0

1a_c023
B3LYP/6-31G* Geometry
C -1.619394 -2.488424 -0.183360
C -2.442689 0.113093 -0.205941
N -2.916162 -2.249253 -0.139403
C -0.627349 -1.482351 -0.231459
C -1.051747 -0.122089 -0.251094
C -3.337752 -0.947717 -0.125648
C -0.085688 0.920698 -0.318347
C 1.262915 0.638691 -0.369443
C 1.709336 -0.730904 -0.362785
C 0.771114 -1.775976 -0.274838
C 3.122800 -1.043225 -0.418249
C 4.139357 0.080774 -0.729460
C 3.574111 1.467122 -0.485891
C 2.248331 1.708606 -0.404785
O 1.139643 -3.055125 -0.236367
O 3.555395 -2.206894 -0.341121
O 4.474009 -0.040962 -2.128234
C 5.456075 -0.177859 0.005463
C 5.326236 -0.096722 1.515328
C 6.612760 -0.178683 2.313774
O 4.239907 0.022255 2.054601
O 4.553094 2.403454 -0.472580
C 4.176612 3.767255 -0.322335
C -4.812110 -0.747185 -0.046096
C -5.647708 -1.852853 -0.648096
C -5.309329 0.343207 0.571463
C -6.745687 0.752820 0.778250
C -7.018553 2.143410 0.155707
C -6.885515 2.184172 -1.369422
C -7.079054 0.760086 2.283945
H -1.314030 -3.532497 -0.186157
H -2.807344 1.134209 -0.255190
H -0.425407 1.953122 -0.316301
H 1.869805 2.721780 -0.324847
H 2.140845 -3.060787 -0.249248
H 3.681056 0.193445 -2.639611
H 6.213797 0.525458 -0.353205
H 5.793297 -1.184668 -0.267190
H 6.385653 -0.195661 3.381471
H 7.182177 -1.075383 2.040574
H 7.250620 0.685635 2.089116
H 3.552856 4.098260 -1.162133
H 3.634128 3.923101 0.618065
H 5.107484 4.336023 -0.311648
H -5.423179 -2.803797 -0.155540
H -6.718579 -1.656746 -0.567532
H -5.402895 -1.991617 -1.708537
H -4.595755 1.034187 1.024593
H -7.414292 0.034918 0.288191
H -8.031738 2.457412 0.442455
H -6.333741 2.875987 0.607969
H -7.593216 1.494458 -1.845805
H -7.089627 3.188981 -1.756543
H -5.878566 1.895992 -1.690822
H -6.929767 -0.230245 2.726952
H -8.121192 1.057952 2.451223
H -6.438070 1.467755 2.825048
SCF Energy (PCM/mPW1PW91/6-31+G**)= -1361.46554033
Number of imaginary frequencies = 0

1a_c024
B3LYP/6-31G* Geometry
C 1.662793 -2.328906 0.001964
C 2.378299 0.269005 -0.428451
N 2.934916 -2.068170 -0.231013
C 0.647818 -1.346247 0.058994
C 1.013887 0.011037 -0.175213
C 3.306875 -0.765535 -0.423452
C 0.016707 1.026186 -0.162372
C -1.303535 0.722906 0.094489
C -1.687931 -0.640859 0.355141
C -0.722307 -1.663663 0.314941
C -3.070618 -0.974962 0.628457
C -4.090938 0.159003 0.885186
C -3.619623 1.501889 0.360828
C -2.323992 1.760074 0.082605
O -1.037642 -2.941679 0.516623
O -3.458769 -2.146645 0.782821
O -4.250545 0.254049 2.316654
C -5.473214 -0.246617 0.369383
C -5.527457 -0.405289 -1.139142
C -6.893468 -0.656996 -1.747433
O -4.520281 -0.340160 -1.822097
O -4.633575 2.399166 0.321421
C -4.337095 3.731475 -0.080707
C 4.760336 -0.532547 -0.654032
C 5.497999 -1.664403 -1.330041
C 5.328520 0.616082 -0.235083
C 6.752861 1.087795 -0.383949
C 7.419541 1.298816 0.996598
C 7.587818 0.014573 1.813660
C 6.784567 2.394322 -1.203575
H 1.396885 -3.372613 0.154657
H 2.692771 1.283990 -0.649679
H 0.309217 2.053204 -0.364758
H -2.002259 2.756332 -0.200435
H -2.029618 -2.969674 0.650376
H -3.412831 0.585321 2.682351
H -6.213455 0.485975 0.705318
H -5.730608 -1.204600 0.835823

H -6.795212 -0.826262 -2.821481
H -7.371802 -1.524864 -1.277267
H -7.549931 0.204408 -1.570652
H -3.635686 4.206048 0.616911
H -3.914244 3.751424 -1.092504
H -5.287063 4.267631 -0.065815
H 5.078579 -1.863101 -2.324575
H 6.563922 -1.458161 -1.443269
H 5.374134 -2.586655 -0.754761
H 4.692710 1.326085 0.297802
H 7.340683 0.337868 -0.926892
H 8.402430 1.763750 0.838239
H 6.826301 2.026853 1.569349
H 8.216944 -0.711410 1.283820
H 8.062135 0.221731 2.779688
H 6.622010 -0.464522 2.007373
H 6.352045 2.248568 -2.199349
H 7.813683 2.752610 -1.326477
H 6.213278 3.186573 -0.702845
SCF Energy (PCM/mPW1PW91/6-31+G**)= -1361.46554346
Number of imaginary frequencies = 0

1a_c028
B3LYP/6-31G* Geometry
C -1.911285 -1.549835 -0.486021
C -2.193335 1.132389 -0.082961
N -3.128565 -1.065283 -0.380063
C -0.732145 -0.767715 -0.407435
C -0.875547 0.632187 -0.195684
C -3.288784 0.281511 -0.177179
C 0.279634 1.456138 -0.101639
C 1.545956 0.922557 -0.225262
C 1.708080 -0.489025 -0.456541
C 0.576436 -1.324955 -0.524592
C 3.030309 -1.063884 -0.590807
C 4.254504 -0.129142 -0.736176
C 3.977446 1.280223 -0.248849
C 2.726094 1.764726 -0.100392
O 0.680765 -2.640931 -0.704848
O 3.220569 -2.287167 -0.712824
O 4.566850 -0.075120 -2.144680
C 5.487410 -0.766691 -0.092970
C 5.367401 -0.923326 1.411836
C 6.596279 -1.429158 2.142423
O 4.332192 -0.660705 1.998608
O 5.124137 1.985572 -0.100171
C 5.027566 3.351571 0.286078
C -4.684535 0.783123 -0.062581
C -4.864759 2.267020 0.162168
C -5.705824 -0.092811 -0.165860
C -7.189037 0.155988 -0.101539
C -7.842115 -0.699728 1.010430
C -7.375157 -0.348688 2.426186
C -7.828807 -0.160091 -1.469984
H -1.822235 -2.622626 -0.644508
H -2.328957 2.195779 0.079390
H 0.153815 2.520508 0.079189
H 2.557123 2.804460 0.157634
H 1.660265 -2.844709 -0.735765
H 3.839424 0.394589 -2.586860
H 6.372847 -0.175452 -0.346309
H 5.619550 -1.757050 -0.543729
H 6.367696 -1.564470 3.201333
H 6.937481 -2.378768 1.712489
H 7.421791 -0.714313 2.033963
H 4.494297 3.938293 -0.472384
H 4.515180 3.451389 1.250629
H 6.053592 3.712004 0.372709
H -4.420730 2.851718 -0.654103
H -5.917504 2.545669 0.228512
H -4.378207 2.593610 1.090483
H -5.426923 -1.132431 -0.332843
H -7.393717 1.208975 0.129576
H -8.932033 -0.578018 0.942889
H -7.638551 -1.760744 0.806545
H -7.600972 0.697187 2.669671
H -7.873516 -0.977815 3.172441
H -6.294168 -0.488454 2.535377
H -7.409773 0.473068 -2.259374
H -8.913016 0.002532 -1.438502
H -7.653612 -1.205448 -1.752572
SCF Energy (PCM/mPW1PW91/6-31+G**)= -1361.46743169
Number of imaginary frequencies = 0

1a_c030
B3LYP/6-31G* Geometry
C 1.642831 -2.260471 0.178837
C 2.531213 0.227392 -0.507423
N 2.939966 -2.091018 0.008365
C 0.683532 -1.230945 0.041661
C 1.142352 0.067264 -0.324538
C 3.398625 -0.843104 -0.313076
C 0.204052 1.124520 -0.506883
C -1.142974 0.916260 -0.316702
C -1.626053 -0.387561 0.063723
C -0.717307 -1.450591 0.226654
C -3.041935 -0.622997 0.239806
C -4.050194 0.549330 0.180686
C -3.420482 1.846361 -0.287983
C -2.103735 1.994266 -0.504709
O -1.115364 -2.678890 0.550504
O -3.505462 -1.759094 0.471159
O -4.512841 0.791009 1.506961
C -5.216311 0.150988 -0.766445
C -6.098599 -0.967231 -0.217999
C -6.434790 -2.112245 -1.141116
O -6.552139 -0.902472 0.917636
O -4.303953 2.875501 -0.490029
C -4.429550 3.816661 0.588194
C 4.874083 -0.717155 -0.471237
C 5.586799 -1.974638 -0.911653
C 5.481731 0.454086 -0.193425
C 6.937716 0.827119 -0.311154
C 7.505843 1.295122 1.049532
C 7.535406 0.203363 2.123190
C 7.105692 1.931595 -1.375600
H 1.309761 -3.263084 0.437660
H 2.914356 1.191877 -0.824800
H 0.567004 2.106322 -0.798657
H -1.727073 2.961461 -0.827221
H -2.113713 -2.646444 0.612241
H -5.282861 0.195348 1.648308
H -4.819538 -0.107653 -1.753658
H -5.845999 1.040516 -0.884949
H -7.160726 -2.776012 -0.667807
H -6.829528 -1.740384 -2.094814
H -5.515679 -2.668369 -1.361643
H -4.781463 3.316269 1.494057
H -3.470245 4.310657 0.786239
H -5.158557 4.558576 0.255009
H 5.189498 -2.331426 -1.870067
H 6.662366 -1.825136 -1.022012
H 5.413292 -2.778827 -0.190248
H 4.857507 1.270439 0.175030
H 7.522343 -0.042369 -0.634431
H 8.523204 1.676299 0.885931
H 6.913463 2.150519 1.406398
H 8.155734 -0.644368 1.806750
H 7.949879 0.585765 3.062812
H 6.530754 -0.181602 2.329479
H 6.744427 1.597689 -2.354205
H 8.159806 2.215907 -1.478035
H 6.542641 2.832394 -1.099808
SCF Energy (PCM/mPW1PW91/6-31+G**)= -1361.45850473
Number of imaginary frequencies = 0

1a_c032
B3LYP/6-31G* Geometry
C 1.543361 -2.425188 0.128363
C 2.332527 0.132307 -0.403648
N 2.823899 -2.208646 -0.103907
C 0.555292 -1.413987 0.136445

C 0.959878 -0.078093 -0.151556
C 3.232796 -0.925503 -0.346136
C -0.009388 0.963288 -0.188865
C -1.338767 0.707162 0.071893
C -1.761759 -0.633429 0.387438
C -0.824454 -1.682939 0.396099
C -3.154151 -0.917371 0.668079
C -4.141881 0.254853 0.875897
C -3.633769 1.559440 0.292439
C -2.330640 1.770002 0.009052
O -1.176052 -2.942204 0.649421
O -3.575580 -2.070417 0.868533
O -4.294166 0.417030 2.302242
C -5.536451 -0.133444 0.381012
C -5.600380 -0.355986 -1.119014
C -6.975011 -0.597409 -1.711953
O -4.594549 -0.347064 -1.806932
O -4.623025 2.480823 0.207095
C -4.289700 3.783441 -0.258136
C 4.694445 -0.742269 -0.568011
C 5.409984 -1.925480 -1.176905
C 5.289114 0.408047 -0.193544
C 6.735196 0.816646 -0.319833
C 7.364424 0.955783 1.089063
C 8.867880 1.256211 1.093973
C 6.835460 2.122497 -1.134641
H 1.247951 -3.454147 0.321571
H 2.676344 1.128384 -0.663770
H 0.312345 1.972482 -0.432405
H -1.980780 2.743507 -0.316540
H -2.169000 -2.937209 0.778465
H -3.446054 0.739932 2.651102
H -6.254457 0.634023 0.685935
H -5.819714 -1.062342 0.889333
H -6.885558 -0.816782 -2.777666
H -7.474935 -1.430089 -1.202306
H -7.607086 0.288650 -1.571892
H -3.578205 4.272937 0.418637
H -3.863132 3.742249 -1.267729
H -5.225002 4.344772 -0.273947
H 4.981954 -2.177146 -2.155440
H 6.478560 -1.744188 -1.307816
H 5.274952 -2.809962 -0.547110
H 4.667104 1.160154 0.295236
H 7.295304 0.042211 -0.857756
H 6.829577 1.741920 1.641992
H 7.183658 0.022421 1.637420
H 9.096673 2.232245 0.652262
H 9.257727 1.261510 2.118223
H 9.425616 0.496863 0.531630
H 6.386726 1.998873 -2.126254
H 7.877066 2.430970 -1.273690
H 6.308004 2.941449 -0.628069
SCF Energy (PCM/mPW1PW91/6-31+G**)= -1361.46467530
Number of imaginary frequencies = 0

1a_c033
B3LYP/6-31G* Geometry
C 1.806834 2.567982 0.053940
C 2.490003 -0.059857 -0.224296
N 3.086544 2.254096 0.096189
C 0.763281 1.627099 -0.109800
C 1.114755 0.252857 -0.264359
C 3.438702 0.935949 -0.018683
C 0.100891 -0.723943 -0.473400
C -1.232351 -0.373841 -0.496232
C -1.604098 1.008503 -0.303986
C -0.613327 1.997935 -0.151930
C -2.991085 1.383135 -0.315767
C -4.082075 0.299855 -0.265582
C -3.583918 -1.046655 -0.743968
C -2.269028 -1.357478 -0.765020
O -0.919075 3.289943 -0.030672
O -3.395255 2.566112 -0.312559
O -5.174295 0.729489 -1.055561
C -4.490148 0.187681 1.251306
C -5.515559 -0.912727 1.547557
C -6.955455 -0.640611 1.171760
O -5.161651 -1.948126 2.080859
O -4.610566 -1.875456 -1.012694
C -4.324505 -3.252433 -1.244503
C 4.897999 0.649792 0.060182
C 5.803658 1.780443 -0.369305
C 5.326005 -0.539173 0.531453
C 6.734680 -1.047662 0.704687
C 6.952815 -2.353273 -0.098062
C 6.851163 -2.179994 -1.616356
C 7.030331 -1.277975 2.200799
H 1.557981 3.622616 0.150661
H 2.800607 -1.088072 -0.378642
H 0.392778 -1.759818 -0.623329
H -1.940707 -2.362802 -1.003306
H -1.907902 3.366121 -0.123159
H -5.155376 1.704381 -0.986886
H -4.884572 1.171235 1.534716
H -3.600951 -0.025864 1.851733
H -7.568498 -1.515951 1.395993
H -7.334669 0.224463 1.731377
H -7.013042 -0.385793 0.108546
H -3.690475 -3.377567 -2.130330
H -3.836592 -3.695029 -0.368685
H -5.289529 -3.731206 -1.412761
H 5.629663 2.661833 0.255452
H 6.859443 1.509679 -0.311690
H 5.582711 2.086618 -1.399131
H 4.569251 -1.247936 0.873297
H 7.450049 -0.305944 0.329511
H 7.942940 -2.753652 0.159089
H 6.223608 -3.104500 0.239601
H 7.601966 -1.469560 -1.983779
H 7.013696 -3.132207 -2.133928
H 5.866709 -1.799567 -1.910305
H 6.916791 -0.351852 2.774165
H 8.053161 -1.646611 2.343725
H 6.344461 -2.021032 2.627154
SCF Energy (PCM/mPW1PW91/6-31+G**)= -1361.46436761
Number of imaginary frequencies = 0

1a_c035
B3LYP/6-31G* Geometry
C -1.594063 -2.570710 -0.097601
C -2.458833 0.010700 -0.283137
N -2.894994 -2.351649 -0.086963
C -0.617753 -1.552305 -0.189931
C -1.063660 -0.202852 -0.294662
C -3.337781 -1.058811 -0.155003
C -0.113693 0.850517 -0.408976
C 1.239909 0.588903 -0.425548
C 1.708119 -0.770328 -0.332878
C 0.785660 -1.824268 -0.196662
C 3.127030 -1.061377 -0.350806
C 4.129474 0.059565 -0.714663
C 3.539126 1.447777 -0.557740
C 2.208549 1.671360 -0.509244
O 1.174096 -3.092583 -0.077967
O 3.577274 -2.210836 -0.198277
O 4.482507 -0.136680 -2.100522
C 5.441291 -0.135992 0.048031
C 5.293498 0.031301 1.549185
C 6.570925 -0.000515 2.365656
O 4.200700 0.177369 2.068330
O 4.502805 2.399407 -0.584236
C 4.102777 3.763001 -0.514977
C -4.816078 -0.877524 -0.109416
C -5.623692 -2.019151 -0.681431
C -5.338901 0.233434 0.448268
C -6.790152 0.622654 0.590887
C -7.195341 1.725933 -0.426411
C -6.431253 3.051352 -0.322586
C -7.095593 1.035031 2.044060
H -1.271693 -3.607761 -0.034882
H -2.839534 1.020718 -0.397291

H -0.469966 1.875454 -0.471072
H 1.812978 2.681017 -0.492449
H 2.175286 -3.082118 -0.076125
H 3.692179 0.055366 -2.633176
H 6.192878 0.556882 -0.342663
H 5.796196 -1.151558 -0.162276
H 6.331495 0.045450 3.429798
H 7.143875 -0.911142 2.152610
H 7.210855 0.849768 2.097563
H 3.486358 4.036812 -1.380388
H 3.544095 3.961312 0.407767
H 5.024342 4.346761 -0.522871
H -5.367976 -2.952795 -0.171646
H -6.699430 -1.851767 -0.600468
H -5.381834 -2.171460 -1.741032
H -4.640246 0.951773 0.877653
H -7.419971 -0.244401 0.361421
H -7.069803 1.320195 -1.438619
H -8.269992 1.920453 -0.301353
H -5.358183 2.914408 -0.498546
H -6.797479 3.763336 -1.070906
H -6.552102 3.519887 0.660663
H -6.907729 0.203586 2.732022
H -8.144851 1.335511 2.152397
H -6.471254 1.876254 2.367505
SCF Energy (PCM/mPW1PW91/6-31+G**)= -1361.46319706
Number of imaginary frequencies = 0

1a_c041
B3LYP/6-31G* Geometry
C 1.815829 2.560851 -0.016926
C 2.510680 -0.069726 -0.231241
N 3.098018 2.253991 0.022040
C 0.775964 1.611573 -0.149161
C 1.134066 0.237425 -0.270010
C 3.455842 0.935781 -0.060622
C 0.120112 -0.746421 -0.442777
C -1.212341 -0.395973 -0.470801
C -1.595248 0.986781 -0.324325
C -0.604650 1.978425 -0.188275
C -2.991476 1.376181 -0.322217
C -4.077522 0.281663 -0.275171
C -3.551540 -1.075434 -0.695021
C -2.244389 -1.395413 -0.706462
O -0.907791 3.270805 -0.083911
O -3.363210 2.559646 -0.269760
O -5.144317 0.696784 -1.117141
C -4.537475 0.241732 1.228502
C -5.599366 -0.813431 1.538668
C -7.043491 -0.413023 1.313327
O -5.286418 -1.907554 1.970917
O -4.591398 -1.893248 -1.000487
C -4.332086 -3.281786 -1.200964
C 4.917246 0.656973 0.013906
C 5.813705 1.767127 -0.482749
C 5.353444 -0.505809 0.538875
C 6.766127 -1.000401 0.722067
C 6.966833 -2.368982 0.028403
C 6.824647 -2.326290 -1.496115
C 7.096131 -1.103932 2.225320
H 1.562398 3.616225 0.055130
H 2.825274 -1.100550 -0.358958
H 0.411073 -1.786942 -0.559503
H -1.915918 -2.404354 -0.930768
H -1.904781 3.335410 -0.148808
H -5.544457 -0.116361 -1.468923
H -4.911784 1.247945 1.445659
H -3.667865 0.032660 1.859248
H -7.692162 -1.289831 1.368583
H -7.344440 0.302793 2.090345
H -7.155929 0.103030 0.354187
H -3.889839 -3.715395 -0.297968
H -5.300846 -3.741885 -1.397457
H -3.667055 -3.434673 -2.058929
H 5.629802 2.682284 0.088176
H 6.872128 1.510845 -0.408135
H 5.591101 2.008665 -1.529496
H 4.602268 -1.196366 0.927186
H 7.472087 -0.293812 0.269204
H 7.964269 -2.746184 0.292251
H 6.248602 -3.088802 0.448081
H 7.564151 -1.650308 -1.942892
H 6.974951 -3.319359 -1.934618
H 5.832191 -1.971371 -1.795270
H 6.997851 -0.131972 2.720247
H 8.121180 -1.463468 2.375910
H 6.418449 -1.804976 2.728925
SCF Energy (PCM/mPW1PW91/6-31+G**)= -1361.46131224
Number of imaginary frequencies = 0

1a_c042
B3LYP/6-31G* Geometry
C -1.840057 -1.555134 -0.418407
C -2.117011 1.134032 -0.061502
N -3.056215 -1.067391 -0.314367
C -0.659612 -0.773162 -0.360337
C -0.800471 0.630370 -0.172358
C -3.214055 0.282990 -0.134742
C 0.356108 1.454501 -0.098468
C 1.621164 0.917410 -0.219109
C 1.780661 -0.498134 -0.426981
C 0.647739 -1.333808 -0.474793
C 3.101557 -1.076648 -0.557968
C 4.325926 -0.145788 -0.725383
C 4.052900 1.271751 -0.260092
C 2.802817 1.760203 -0.114079
O 0.749801 -2.652821 -0.632897
O 3.290055 -2.302073 -0.659967
O 4.630693 -0.115533 -2.136297
C 5.561643 -0.773948 -0.078318
C 5.449772 -0.905484 1.429522
C 6.681634 -1.402159 2.161413
O 4.418603 -0.630678 2.017814
O 5.201123 1.978051 -0.128136
C 5.107818 3.349920 0.237429
C -4.608465 0.788386 -0.021840
C -4.785046 2.277284 0.170708
C -5.630739 -0.089291 -0.094143
C -7.111869 0.163722 -0.003582
C -7.685127 -0.567948 1.237108
C -9.167791 -0.291835 1.512943
C -7.793404 -0.289066 -1.311739
H -1.753029 -2.630698 -0.558210
H -2.250390 2.200259 0.083107
H 0.232329 2.521994 0.064538
H 2.636181 2.804321 0.127255
H 1.728919 -2.858199 -0.665323
H 3.901457 0.347912 -2.582095
H 6.446274 -0.187872 -0.346113
H 5.690476 -1.771753 -0.513329
H 6.458595 -1.519829 3.223613
H 7.018464 -2.359351 1.745042
H 7.508077 -0.691005 2.036920
H 4.572454 3.925719 -0.527910
H 4.599221 3.465178 1.202267
H 6.134638 3.710256 0.314636
H -4.341645 2.843421 -0.658863
H -5.836913 2.560272 0.234045
H -4.295434 2.622933 1.090569
H -5.354109 -1.133014 -0.235150
H -7.310881 1.235033 0.125789
H -7.526464 -1.649060 1.116635
H -7.096086 -0.270178 2.114148
H -9.813660 -0.661518 0.709193
H -9.486052 -0.782077 2.440048
H -9.357297 0.783215 1.625051
H -7.370358 0.237579 -2.173930
H -8.870564 -0.091698 -1.292809
H -7.649468 -1.364667 -1.474389
SCF Energy (PCM/mPW1PW91/6-31+G**)= -1361.46652326
Number of imaginary frequencies = 0

1a_c043
B3LYP/6-31G* Geometry
C -1.843660 -2.387657 0.134389
C -2.439793 0.267135 0.348359
N -3.098379 -2.051328 0.362329
C -0.787751 -1.459167 -0.017525
C -1.092629 -0.072073 0.100706
C -3.411914 -0.722033 0.448329
C -0.055697 0.894507 -0.027933
C 1.248739 0.511122 -0.252980
C 1.578903 -0.888884 -0.358835
C 0.562196 -1.858469 -0.263472
C 2.948487 -1.317247 -0.563741
C 4.083599 -0.277928 -0.460855
C 3.583767 1.143618 -0.615698
C 2.297976 1.502285 -0.444298
O 0.812963 -3.160511 -0.385431
O 3.267222 -2.505448 -0.731352
O 5.048766 -0.589796 -1.455776
C 4.681513 -0.486455 0.979164
C 5.810908 0.478460 1.340989
C 7.210024 0.079596 0.917083
O 5.584602 1.500867 1.961808
O 4.626713 1.965249 -0.900077
C 4.414473 3.375686 -0.863540
C -4.849320 -0.410407 0.684461
C -5.624864 -1.466866 1.436330
C -5.375063 0.736005 0.207458
C -6.774864 1.277639 0.350613
C -7.445297 1.465843 -1.031230
C -7.676132 0.160343 -1.797820
C -6.738441 2.614553 1.119836
H -1.626234 -3.451340 0.066429
H -2.705252 1.310727 0.483429
H -0.307322 1.949233 0.044561
H 1.996227 2.543524 -0.478285
H 1.793671 -3.245625 -0.567746
H 5.445882 0.256438 -1.722660
H 5.031012 -1.524272 1.001490
H 3.883781 -0.354881 1.716752
H 7.897578 0.918304 1.045539
H 7.550436 -0.759506 1.538947
H 7.211878 -0.278770 -0.117592
H 3.678002 3.678899 -1.616798
H 4.081635 3.679835 0.134312
H 5.380395 3.829488 -1.086887
H -5.181762 -1.645727 2.424059
H -6.672203 -1.192975 1.575480
H -5.576068 -2.420115 0.901584
H -4.719419 1.386656 -0.374781
H -7.390503 0.576662 0.926914
H -8.405681 1.978244 -0.882115
H -6.827306 2.145964 -1.635841
H -8.331177 -0.517184 -1.236226
H -8.149457 0.351391 -2.767585
H -6.733529 -0.367143 -1.979961
H -6.304882 2.486596 2.117572
H -7.748815 3.024659 1.235654
H -6.135314 3.359676 0.585346
SCF Energy (PCM/mPW1PW91/6-31+G**)= -1361.46126960
Number of imaginary frequencies = 0

1a_c044
B3LYP/6-31G* Geometry
C 1.624785 -2.410042 0.166304
C 2.392797 0.127558 -0.480155
N 2.900755 -2.197111 -0.092975
C 0.631416 -1.404183 0.146545
C 1.024920 -0.079038 -0.200293
C 3.299152 -0.923118 -0.394131
C 0.049740 0.955296 -0.266485
C -1.275072 0.703025 0.020077
C -1.686910 -0.625982 0.393665
C -0.743653 -1.669422 0.433724
C -3.074673 -0.905836 0.700410
C -4.067534 0.268147 0.870621
C -3.572221 1.551689 0.231868
C -2.273276 1.757622 -0.073282
O -1.085099 -2.919220 0.741877
O -3.487194 -2.052018 0.952095
O -4.209016 0.485545 2.290651
C -5.463695 -0.146953 0.402861
C -5.537516 -0.432507 -1.086009
C -6.915027 -0.706627 -1.657688
O -4.536893 -0.446657 -1.781420
O -4.567353 2.463915 0.120062
C -4.246293 3.747460 -0.403188
C 4.755218 -0.743443 -0.650401
C 5.466830 -1.949584 -1.217366
C 5.353763 0.424456 -0.340676
C 6.799168 0.817142 -0.531219
C 7.612930 0.701517 0.787489
C 7.126421 1.569672 1.954283
C 6.891418 2.226797 -1.147343
H 1.337674 -3.431593 0.405810
H 2.727349 1.113875 -0.785652
H 0.363010 1.955611 -0.553777
H -1.932213 2.719441 -0.440388
H -2.076519 -2.914310 0.882150
H -3.360399 0.827976 2.619044
H -6.183159 0.629380 0.680613
H -5.738802 -1.055017 0.951726
H -6.832376 -0.967938 -2.714460
H -7.404829 -1.521751 -1.111147
H -7.552477 0.179856 -1.547948
H -3.531201 4.269283 0.245028
H -3.829297 3.665491 -1.414280
H -5.185022 4.302437 -0.433515
H 5.015169 -2.257790 -2.168636
H 6.529854 -1.765193 -1.385027
H 5.359337 -2.800751 -0.538113
H 4.738331 1.193173 0.127321
H 7.267956 0.124780 -1.240353
H 7.607043 -0.350425 1.100662
H 8.659227 0.952650 0.562259
H 6.102072 1.313589 2.246987
H 7.765460 1.422375 2.832253
H 7.149496 2.637756 1.709476
H 6.407661 2.255274 -2.130030
H 7.937945 2.528609 -1.275753
H 6.401733 2.978587 -0.517221
SCF Energy (PCM/mPW1PW91/6-31+G**)= -1361.46332914
Number of imaginary frequencies = 0

1a_c051
B3LYP/6-31G* Geometry
C -1.502468 -2.335618 -0.678852
C -2.445610 0.183057 -0.213695
N -2.806476 -2.173829 -0.569355
C -0.559280 -1.288968 -0.554796
C -1.046807 0.030948 -0.320010
C -3.288411 -0.918551 -0.313227
C -0.136774 1.122231 -0.232220
C 1.223774 0.922896 -0.329721
C 1.731709 -0.413011 -0.529418
C 0.846839 -1.495749 -0.686072
C 3.149559 -0.626434 -0.636868
C 4.124733 0.481143 -0.204472
C 3.498354 1.859056 -0.310422
C 2.158426 2.038267 -0.296979
O 1.276002 -2.730194 -0.944897
O 3.666466 -1.687764 -1.039659
O 5.287361 0.398563 -1.005116
C 4.482206 0.265518 1.315468
C 5.119992 -1.091555 1.622982
C 6.624266 -1.193667 1.479475
O 4.431750 -2.026059 1.986766
O 4.437072 2.827188 -0.293849
C 4.004122 4.182389 -0.334625
C -4.768022 -0.809573 -0.180889
C -5.563890 -1.841666 -0.945238
C -5.304159 0.139086 0.613455

C -6.754166 0.436932 0.900209
C -7.095145 1.905439 0.549085
C -6.970620 2.238353 -0.940683
C -7.068238 0.149470 2.382648
H -1.149186 -3.344756 -0.879142
H -2.858528 1.177118 -0.075322
H -0.531302 2.125409 -0.094379
H 1.730825 3.034331 -0.267656
H 2.264132 -2.685334 -1.045993
H 5.284336 -0.518022 -1.346793
H 3.569175 0.348991 1.911794
H 5.162276 1.083403 1.576731
H 6.939200 -2.236342 1.556159
H 6.945443 -0.753937 0.529521
H 7.107349 -0.612303 2.276682
H 3.412379 4.376999 -1.237176
H 3.408985 4.433762 0.552473
H 4.912807 4.785276 -0.351639
H -5.278075 -2.848506 -0.626340
H -6.640006 -1.719957 -0.809091
H -5.344601 -1.785995 -2.018773
H -4.615999 0.780883 1.167083
H -7.397011 -0.206627 0.287638
H -8.120354 2.111231 0.885780
H -6.443247 2.571156 1.133949
H -7.647111 1.618262 -1.541721
H -7.222445 3.287422 -1.133193
H -5.952579 2.062431 -1.305160
H -6.871709 -0.898353 2.633143
H -8.119870 0.364537 2.607059
H -6.451116 0.771880 3.043080
SCF Energy (PCM/mPW1PW91/6-31+G**)= -1361.46329990
Number of imaginary frequencies = 0

1a_c052
B3LYP/6-31G* Geometry
C 1.623751 -2.278107 -0.169013
C 2.511038 0.282163 -0.492063
N 2.922067 -2.085647 -0.302483
C 0.663227 -1.240663 -0.166625
C 1.120138 0.097720 -0.344414
C 3.378796 -0.803318 -0.440131
C 0.182262 1.168140 -0.385769
C -1.166946 0.937873 -0.227434
C -1.644665 -0.407080 -0.027535
C -0.737669 -1.483476 -0.022197
C -3.058382 -0.660231 0.121714
C -4.043629 0.514602 0.327100
C -3.445941 1.851371 -0.078462
C -2.125720 2.029910 -0.289724
O -1.138524 -2.746065 0.117989
O -3.532360 -1.815084 0.158200
O -4.277338 0.609431 1.729052
C -5.349360 0.219213 -0.455204
C -6.136848 -0.968197 0.094078
C -6.659318 -1.979800 -0.896272
O -6.382638 -1.053590 1.290502
O -4.391540 2.818689 -0.116670
C -3.976404 4.160703 -0.339596
C 4.855842 -0.650882 -0.558345
C 5.579930 -1.822157 -1.180161
C 5.453747 0.464399 -0.092769
C 6.909960 0.853903 -0.126197
C 7.463865 1.063426 1.303081
C 7.481687 -0.205906 2.159691
C 7.086064 2.135714 -0.966640
H 1.290635 -3.307579 -0.056985
H 2.896076 1.281446 -0.668190
H 0.546517 2.179834 -0.543528
H -1.726697 3.014315 -0.508642
H -2.136122 -2.723150 0.173064
H -5.015865 -0.009262 1.927922
H -5.123147 0.082735 -1.517835
H -5.991800 1.101890 -0.363639
H -7.293381 -2.708987 -0.388428
H -7.224033 -1.484001 -1.695855
H -5.808528 -2.490096 -1.362421
H -3.283138 4.490015 0.443692
H -3.496457 4.268687 -1.320576
H -4.884322 4.764451 -0.306326
H 5.210468 -2.012688 -2.195690
H 6.658564 -1.663459 -1.233143
H 5.383231 -2.732824 -0.606578
H 4.819984 1.210779 0.390403
H 7.499744 0.059524 -0.599220
H 8.483052 1.466063 1.222878
H 6.867543 1.840058 1.804274
H 8.107864 -0.982203 1.702639
H 7.883189 -0.001086 3.158594
H 6.474993 -0.620970 2.278850
H 6.737726 1.987369 -1.994491
H 8.140144 2.436494 -1.002165
H 6.515964 2.969204 -0.536486
SCF Energy (PCM/mPW1PW91/6-31+G**)= -1361.46708401
Number of imaginary frequencies = 0

1a_c053
B3LYP/6-31G* Geometry
C -1.934139 -1.430336 -0.540502
C -2.160437 1.237029 -0.020791
N -3.141050 -0.926042 -0.411134
C -0.739023 -0.676715 -0.430508
C -0.853341 0.715183 -0.157025
C -3.273415 0.413983 -0.150635
C 0.318819 1.509812 -0.026748
C 1.573748 0.956016 -0.175146
C 1.706342 -0.446638 -0.470896
C 0.557552 -1.255032 -0.574915
C 3.016109 -1.041743 -0.634730
C 4.258369 -0.126021 -0.742336
C 4.012376 1.263025 -0.184903
C 2.771542 1.766176 -0.011502
O 0.634560 -2.563293 -0.815091
O 3.180985 -2.261954 -0.812208
O 4.562973 -0.008998 -2.148829
C 5.481449 -0.819785 -0.140309
C 5.365454 -1.052909 1.354883
C 6.582135 -1.635909 2.047522
O 4.342264 -0.790949 1.962632
O 5.173825 1.935635 -0.003496
C 5.106574 3.282178 0.451418
C -4.658301 0.938457 -0.011621
C -4.808505 2.415963 0.269819
C -5.697241 0.085856 -0.135013
C -7.172418 0.373491 -0.027871
C -7.746723 -0.095003 1.339424
C -7.596830 -1.588694 1.652326
C -7.923991 -0.257956 -1.216605
H -1.867318 -2.496881 -0.745362
H -2.273776 2.294615 0.189634
H 0.215306 2.567188 0.202704
H 2.624871 2.795468 0.296900
H 1.609677 -2.785321 -0.857405
H 3.844062 0.499176 -2.561413
H 6.377091 -0.233026 -0.366882
H 5.592431 -1.787188 -0.643339
H 6.360187 -1.808684 3.102383
H 6.879723 -2.578755 1.572584
H 7.434390 -0.950426 1.958894
H 4.585070 3.917557 -0.275352
H 4.597449 3.343859 1.420877
H 6.140090 3.616056 0.553981
H -4.340833 3.023732 -0.515745
H -5.855667 2.715343 0.336946
H -4.327420 2.693199 1.216972
H -5.436453 -0.951093 -0.334793
H -7.344439 1.455689 -0.074394
H -7.257347 0.485730 2.132199
H -8.811616 0.176714 1.367726
H -6.544200 -1.890217 1.680042
H -8.033118 -1.818940 2.630961
H -8.106253 -2.215692 0.911989

H -7.573816 0.161270 -2.166259
H -9.001991 -0.070744 -1.139382
H -7.772231 -1.342425 -1.260604
SCF Energy (PCM/mPW1PW91/6-31+G**)= -1361.46512855
Number of imaginary frequencies = 0

1a_c055
B3LYP/6-31G* Geometry
C -1.817339 -1.348808 -0.809246
C -2.197508 1.207995 0.062296
N -3.050084 -0.940450 -0.611646
C -0.667977 -0.546748 -0.594850
C -0.862381 0.789320 -0.140626
C -3.260054 0.342407 -0.172974
C 0.258150 1.639382 0.073053
C 1.545187 1.181297 -0.119166
C 1.755389 -0.179521 -0.547150
C 0.657258 -1.016643 -0.824630
C 3.093370 -0.661481 -0.754490
C 4.287872 0.136489 -0.205485
C 3.966175 1.612330 -0.063148
C 2.694763 2.059501 0.042686
O 0.811753 -2.256134 -1.289759
O 3.368246 -1.728507 -1.339704
O 5.392690 -0.052394 -1.067708
C 4.618230 -0.392483 1.241964
C 4.951240 -1.885136 1.304413
C 6.393275 -2.279935 1.061546
O 4.084703 -2.698394 1.563349
O 5.088726 2.349360 0.061219
C 4.952138 3.751551 0.263195
C -4.672066 0.758556 0.036215
C -4.908453 2.171628 0.517899
C -5.658656 -0.128232 -0.212624
C -7.149768 0.041993 -0.100971
C -7.746543 -0.980959 0.896107
C -7.281879 -0.789791 2.342790
C -7.794496 -0.125128 -1.493223
H -1.688764 -2.371442 -1.157984
H -2.373627 2.220854 0.406434
H 0.087241 2.666800 0.383434
H 2.487457 3.103816 0.248664
H 1.785127 -2.400757 -1.430580
H 5.190520 -0.881938 -1.545055
H 3.756774 -0.214569 1.891964
H 5.464937 0.209791 1.588528
H 6.474104 -3.364127 0.960355
H 6.782542 -1.771352 0.173484
H 7.009169 -1.951033 1.909757
H 4.402383 4.214894 -0.564884
H 4.436563 3.965884 1.207948
H 5.967603 4.147798 0.299521
H -4.507086 2.908927 -0.189686
H -5.970213 2.386317 0.647461
H -4.416661 2.353384 1.482386
H -5.340418 -1.111482 -0.556537
H -7.398473 1.045745 0.265857
H -8.841812 -0.906965 0.849766
H -7.492684 -1.995458 0.556975
H -7.555493 0.204525 2.717889
H -7.741479 -1.532100 3.005122
H -6.194292 -0.889350 2.428612
H -7.411639 0.618783 -2.200079
H -8.883615 -0.011924 -1.432268
H -7.582066 -1.118828 -1.906236
SCF Energy (PCM/mPW1PW91/6-31+G**)= -1361.46517618
Number of imaginary frequencies = 0

1a_c056
B3LYP/6-31G* Geometry
C -1.544908 -2.226915 -0.365566
C -2.382180 0.256200 0.398735
N -2.823570 -2.063659 -0.088374
C -0.580177 -1.194446 -0.300712
C -1.010183 0.103741 0.104936
C -3.257360 -0.816808 0.273217
C -0.072090 1.170947 0.192684
C 1.263583 0.967835 -0.081960
C 1.716304 -0.348160 -0.463136
C 0.796777 -1.402548 -0.613447
C 3.107741 -0.565832 -0.751687
C 4.149312 0.485886 -0.334825
C 3.544820 1.874429 -0.241404
C 2.219357 2.065430 -0.055710
O 1.167313 -2.612023 -1.031490
O 3.552884 -1.590848 -1.305980
O 5.211943 0.464343 -1.267323
C 4.671980 0.126813 1.108085
C 5.299233 -1.264557 1.221184
C 6.773726 -1.384798 0.896337
O 4.628745 -2.213646 1.580475
O 4.499751 2.826605 -0.244564
C 4.094576 4.183777 -0.104260
C -4.716001 -0.694908 0.549222
C -5.382513 -1.947895 1.067698
C -5.350120 0.469255 0.302957
C -6.794362 0.837129 0.531069
C -7.479921 1.253220 -0.791792
C -7.587056 0.125233 -1.822121
C -6.883307 1.976246 1.567950
H -1.230220 -3.227119 -0.655238
H -2.744187 1.218390 0.746521
H -0.426560 2.160577 0.468323
H 1.818855 3.058585 0.115129
H 2.138234 -2.568523 -1.241383
H 5.151244 -0.417512 -1.686291
H 3.836226 0.174274 1.812024
H 5.401418 0.904586 1.358367
H 7.064398 -2.436446 0.854259
H 6.998250 -0.876864 -0.047515
H 7.361761 -0.879491 1.674623
H 3.407098 4.472197 -0.908513
H 3.611374 4.353123 0.866370
H 5.008159 4.775877 -0.169415
H -4.917991 -2.274726 2.006496
H -6.450250 -1.807638 1.245514
H -5.246145 -2.768015 0.356479
H -4.761495 1.281335 -0.128586
H -7.340853 -0.024463 0.933075
H -8.483937 1.632261 -0.556265
H -6.928430 2.100095 -1.226287
H -8.176457 -0.713037 -1.430481
H -8.074882 0.474140 -2.739307
H -6.598876 -0.263302 -2.091492
H -6.438842 1.678233 2.523644
H -7.927839 2.257115 1.748616
H -6.353543 2.870873 1.216285
SCF Energy (PCM/mPW1PW91/6-31+G**)= -1361.46330816
Number of imaginary frequencies = 0

1a_c057
B3LYP/6-31G* Geometry
C -1.496204 -2.395260 -0.445526
C -2.510894 0.123871 -0.175088
N -2.807438 -2.250513 -0.460256
C -0.582003 -1.328328 -0.287782
C -1.107802 -0.011062 -0.152073
C -3.327811 -0.995339 -0.303029
C -0.219899 1.094493 -0.007937
C 1.143963 0.911220 -0.003579
C 1.695716 -0.413107 -0.144976
C 0.834938 -1.520202 -0.272198
C 3.126758 -0.618198 -0.120384
C 4.096123 0.587732 -0.102419
C 3.388584 1.910254 0.117139
C 2.052610 2.038233 0.153968
O 1.294101 -2.764974 -0.380268
O 3.638005 -1.757329 -0.141130
O 4.702103 0.677146 -1.389068
C 5.153427 0.355095 1.012270
C 6.118964 -0.788914 0.714125
C 6.373679 -1.796267 1.807963

O 6.698632 -0.849482 -0.362933
O 4.224373 2.985133 0.278946
C 4.419903 3.808414 -0.881345
C -4.814258 -0.904920 -0.310448
C -5.523404 -1.982260 -1.097610
C -5.431012 0.071119 0.385886
C -6.903405 0.374589 0.500627
C -7.188267 1.771665 -0.108294
C -8.671666 2.154444 -0.166266
C -7.346428 0.278046 1.974315
H -1.113743 -3.406071 -0.568644
H -2.946385 1.116023 -0.115157
H -0.635144 2.092488 0.103375
H 1.619158 3.024705 0.297002
H 2.291741 -2.704813 -0.327430
H 5.498759 0.100428 -1.365179
H 4.651113 0.207181 1.973890
H 5.745106 1.275158 1.085050
H 7.166200 -2.484362 1.507909
H 6.645155 -1.294022 2.744775
H 5.449351 -2.356782 1.992376
H 4.857700 3.223669 -1.694769
H 3.471392 4.250406 -1.210616
H 5.103248 4.603031 -0.573902
H -5.267159 -2.969360 -0.700976
H -6.608462 -1.864727 -1.078304
H -5.196096 -1.978628 -2.144674
H -4.803741 0.748760 0.967591
H -7.481354 -0.358185 -0.075106
H -6.634617 2.529938 0.464857
H -6.772718 1.793316 -1.124054
H -9.111856 2.253386 0.831630
H -8.803576 3.114208 -0.678645
H -9.253442 1.403381 -0.715039
H -7.127631 -0.714802 2.381466
H -8.421063 0.457329 2.084870
H -6.817265 1.016083 2.591094
SCF Energy (PCM/mPW1PW91/6-31+G**)= -1361.45763424
Number of imaginary frequencies = 0

1a_c059
B3LYP/6-31G* Geometry
C 1.504137 -2.374455 -0.037300
C 2.457614 0.148768 -0.450218
N 2.807656 -2.219659 -0.169095
C 0.570118 -1.314094 -0.080872
C 1.061801 0.004892 -0.304882
C 3.297618 -0.955414 -0.352444
C 0.151418 1.096170 -0.392810
C -1.204197 0.905662 -0.236601
C -1.716948 -0.418727 0.008506
C -0.837399 -1.516413 0.061115
C -3.137646 -0.631021 0.154673
C -4.094300 0.574759 0.310095
C -3.460481 1.880460 -0.139414
C -2.134758 2.018159 -0.346786
O -1.270985 -2.762605 0.244427
O -3.640976 -1.771617 0.228450
O -4.334443 0.726303 1.706039
C -5.402313 0.283895 -0.470064
C -6.222060 -0.863522 0.115674
C -6.763415 -1.896952 -0.841462
O -6.477226 -0.899864 1.312633
O -4.381256 2.868884 -0.220518
C -3.931007 4.190898 -0.489786
C 4.778342 -0.845790 -0.470312
C 5.474463 -2.064361 -1.030340
C 5.403332 0.274435 -0.055507
C 6.872086 0.613910 -0.091619
C 7.412283 0.766116 1.352325
C 8.924096 1.001429 1.448325
C 7.084335 1.890141 -0.932277
H 1.144498 -3.390301 0.111174
H 2.868387 1.130815 -0.661514
H 0.542175 2.091868 -0.585180
H -1.709505 2.983483 -0.598901
H -2.268092 -2.712870 0.289571
H -5.088930 0.133080 1.921890
H -5.173541 0.104566 -1.525764
H -6.023207 1.184771 -0.413940
H -7.419629 -2.590691 -0.312550
H -7.309427 -1.416617 -1.663168
H -5.922663 -2.445462 -1.281719
H -3.234456 4.531579 0.285681
H -3.442597 4.250545 -1.470721
H -4.823550 4.818033 -0.485147
H 5.096303 -2.302306 -2.032459
H 6.556140 -1.932387 -1.095737
H 5.261480 -2.938516 -0.407668
H 4.788842 1.058459 0.390618
H 7.431055 -0.199312 -0.570048
H 6.878658 1.590570 1.847483
H 7.154268 -0.142212 1.911720
H 9.224103 1.951258 0.992659
H 9.246159 1.024683 2.495576
H 9.482634 0.200391 0.948055
H 6.691916 1.758008 -1.946400
H 8.145733 2.147067 -1.014675
H 6.566021 2.746644 -0.481539
SCF Energy (PCM/mPW1PW91/6-31+G**)= -1361.46623841
Number of imaginary frequencies = 0

1a_c062
B3LYP/6-31G* Geometry
C -1.544617 -2.244490 -0.819946
C -2.485046 0.234491 -0.169971
N -2.848672 -2.086671 -0.706320
C -0.599446 -1.213098 -0.610737
C -1.086824 0.085599 -0.278988
C -3.330637 -0.854033 -0.358499
C -0.173549 1.165161 -0.102261
C 1.185220 0.965622 -0.204190
C 1.693370 -0.351465 -0.501730
C 0.807382 -1.415040 -0.748790
C 3.113491 -0.558471 -0.616016
C 4.082315 0.510002 -0.086604
C 3.463962 1.898322 -0.111042
C 2.128992 2.069456 -0.085429
O 1.233383 -2.627506 -1.100515
O 3.629294 -1.582103 -1.107855
O 5.275231 0.450006 -0.852133
C 4.396234 0.200584 1.426460
C 5.046134 -1.165346 1.658702
C 6.558911 -1.225412 1.618802
O 4.357449 -2.143605 1.878599
O 4.298711 2.960448 0.096749
C 5.058987 3.425803 -1.032529
C -4.810875 -0.752684 -0.229523
C -5.601233 -1.732734 -1.065143
C -5.352280 0.142886 0.621004
C -6.803656 0.426170 0.914325
C -7.150018 1.903107 0.603088
C -7.027401 2.276681 -0.877034
C -7.117665 0.096611 2.387628
H -1.193090 -3.236647 -1.094232
H -2.894940 1.216863 0.041267
H -0.564180 2.156947 0.108435
H 1.734135 3.074440 0.033546
H 2.221811 -2.579224 -1.193121
H 5.262638 -0.441624 -1.256609
H 3.462706 0.229298 1.995202
H 5.048679 1.012766 1.764136
H 6.895416 -2.263318 1.660689
H 6.930531 -0.729875 0.715375
H 6.972314 -0.675561 2.475052
H 5.730239 2.650906 -1.406312
H 4.383832 3.753546 -1.832950
H 5.630798 4.281150 -0.666893
H -5.326511 -2.758925 -0.803074
H -6.678574 -1.612134 -0.938022
H -5.365631 -1.614783 -2.130034
H -4.667941 0.748395 1.218322

H -7.442999 -0.202926 0.283544
H -8.175852 2.095513 0.945851
H -6.500333 2.555045 1.205592
H -7.701868 1.670698 -1.494508
H -7.283282 3.329632 -1.040667
H -6.008734 2.114829 -1.246046
H -6.914990 -0.956421 2.609931
H -8.170907 0.298848 2.615973
H -6.505601 0.705011 3.065572
SCF Energy (PCM/mPW1PW91/6-31+G**)= -1361.45397507
Number of imaginary frequencies = 0

1a_c064
B3LYP/6-31G* Geometry
C -1.569115 -2.429563 -0.438022
C -2.588422 0.089125 -0.180732
N -2.880574 -2.287486 -0.450621
C -0.656782 -1.359912 -0.287931
C -1.184875 -0.042790 -0.160475
C -3.403238 -1.032659 -0.298203
C -0.298785 1.065350 -0.025433
C 1.065408 0.884296 -0.019592
C 1.619389 -0.440196 -0.150394
C 0.760454 -1.549405 -0.270872
C 3.050757 -0.643205 -0.121277
C 4.018718 0.563831 -0.106740
C 3.308475 1.887598 0.096100
C 1.972217 2.013976 0.128986
O 1.221440 -2.794250 -0.370185
O 3.563482 -1.781707 -0.133979
O 4.635540 0.641857 -1.388872
C 5.066877 0.342559 1.019063
C 6.035333 -0.803809 0.739919
C 6.279800 -1.801610 1.844809
O 6.625000 -0.873488 -0.331091
O 4.141736 2.965553 0.250139
C 4.345267 3.773504 -0.919711
C -4.890094 -0.947534 -0.293984
C -5.601552 -2.024777 -1.079000
C -5.505629 0.024453 0.410048
C -6.984468 0.296667 0.544006
C -7.435225 1.485027 -0.351192
C -6.767904 2.834884 -0.061498
C -7.359071 0.509773 2.023328
H -1.184852 -3.440287 -0.556279
H -3.025716 1.080815 -0.125293
H -0.715610 2.063488 0.078500
H 1.537287 3.001211 0.261924
H 2.218923 -2.732335 -0.316928
H 5.432225 0.065868 -1.353068
H 4.556521 0.203703 1.977817
H 5.657370 1.263709 1.087701
H 7.075455 -2.491890 1.558357
H 6.541880 -1.291281 2.779930
H 5.353934 -2.361016 2.025008
H 4.790978 3.178828 -1.721513
H 3.398433 4.208734 -1.262550
H 5.024287 4.573746 -0.617316
H -5.324857 -3.013640 -0.701185
H -6.687385 -1.919639 -1.039810
H -5.295075 -2.002279 -2.132277
H -4.873509 0.705615 0.979970
H -7.544529 -0.576765 0.190934
H -7.249780 1.212923 -1.398266
H -8.524128 1.592026 -0.246553
H -5.681773 2.785701 -0.199767
H -7.150434 3.604451 -0.741373
H -6.960694 3.177532 0.961407
H -7.130588 -0.383100 2.615515
H -8.429968 0.721513 2.128554
H -6.807181 1.347558 2.465582
SCF Energy (PCM/mPW1PW91/6-31+G**)= -1361.45613444
Number of imaginary frequencies = 0

1a_c065
B3LYP/6-31G* Geometry
C 1.584777 -2.164956 0.539950
C 2.431021 0.235297 -0.448073
N 2.866575 -2.025385 0.264968
C 0.620305 -1.144854 0.365633
C 1.056489 0.108244 -0.157475
C 3.306256 -0.818713 -0.207771
C 0.118335 1.162079 -0.356672
C -1.218281 0.980983 -0.077906
C -1.677530 -0.291832 0.422220
C -0.760056 -1.324920 0.683360
C -3.073227 -0.478379 0.720789
C -4.105737 0.529179 0.191359
C -3.500598 1.909042 -0.010364
C -2.178518 2.068775 -0.208995
O -1.132940 -2.489104 1.213387
O -3.520776 -1.438072 1.379952
O -5.202083 0.556653 1.091283
C -4.589516 0.054335 -1.231689
C -5.250831 -1.325774 -1.231660
C -6.749679 -1.372629 -1.020128
O -4.582937 -2.326728 -1.409556
O -4.357027 2.948000 -0.242823
C -5.029974 3.518127 0.893998
C 4.769286 -0.722715 -0.469101
C 5.448629 -2.023891 -0.827552
C 5.397206 0.464019 -0.343519
C 6.846316 0.807693 -0.577270
C 7.485171 1.416186 0.693415
C 7.546314 0.457013 1.885699
C 6.964429 1.788016 -1.763079
H 1.267369 -3.133983 0.918722
H 2.794599 1.160990 -0.882261
H 0.473494 2.122502 -0.720082
H -1.808360 3.052087 -0.484844
H -2.104705 -2.426537 1.412341
H -5.133223 -0.284867 1.587388
H -3.728713 0.014180 -1.904984
H -5.283078 0.826606 -1.580753
H -7.082190 -2.406914 -0.910794
H -7.022934 -0.779454 -0.140609
H -7.258368 -0.917210 -1.880474
H -5.693526 2.794172 1.369286
H -4.296141 3.885186 1.622263
H -5.602943 4.360054 0.500086
H 4.997649 -2.462535 -1.726429
H 6.517656 -1.898566 -1.008700
H 5.308438 -2.755741 -0.026434
H 4.799607 1.318906 -0.021074
H 7.409503 -0.097756 -0.833009
H 8.499878 1.753831 0.441608
H 6.924097 2.319971 0.973726
H 8.140523 -0.433695 1.646861
H 8.006329 0.939255 2.755633
H 6.546167 0.118037 2.177039
H 6.558743 1.350533 -2.681570
H 8.012638 2.054678 -1.944337
H 6.414037 2.716023 -1.562332
SCF Energy (PCM/mPW1PW91/6-31+G**)= -1361.45383909
Number of imaginary frequencies = 0

1a_c071
B3LYP/6-31G* Geometry
C 1.693447 2.631473 0.088194
C 2.426253 0.035229 -0.327701
N 2.980063 2.344370 0.091530
C 0.666923 1.676527 -0.100944
C 1.044125 0.319306 -0.328107
C 3.357260 1.040927 -0.091975
C 0.047075 -0.668143 -0.566016
C -1.293385 -0.346480 -0.547581
C -1.690319 1.016450 -0.281336
C -0.717650 2.018800 -0.099351
C -3.084680 1.360872 -0.249204
C -4.151953 0.253070 -0.232676
C -3.634838 -1.057946 -0.783762
C -2.314370 -1.338451 -0.844466

O -1.047894 3.296401 0.090892
O -3.513369 2.533200 -0.180316
O -5.266598 0.696520 -0.982882
C -4.530798 0.060615 1.283615
C -5.530743 -1.071699 1.544888
C -6.982126 -0.809117 1.208267
O -5.148821 -2.124168 2.022497
O -4.649013 -1.895060 -1.073192
C -4.338542 -3.251489 -1.381491
C 4.822789 0.780714 -0.053461
C 5.697132 1.938881 -0.474421
C 5.280722 -0.413071 0.374764
C 6.702030 -0.904336 0.481162
C 6.886160 -2.153944 -0.417098
C 8.326859 -2.670751 -0.504095
C 7.047707 -1.190872 1.956663
H 1.424109 3.674546 0.239863
H 2.756717 -0.977008 -0.536833
H 0.357525 -1.689022 -0.771282
H -1.969845 -2.323822 -1.136987
H -2.039757 3.355372 0.021318
H -5.266902 1.667100 -0.866840
H -4.937929 1.021990 1.620224
H -3.627320 -0.164299 1.857978
H -7.574440 -1.706493 1.398502
H -7.366819 0.018793 1.818191
H -7.064622 -0.501760 0.160726
H -3.719987 -3.316686 -2.284568
H -3.823688 -3.727842 -0.539423
H -5.296332 -3.742418 -1.556008
H 5.503980 2.807081 0.162865
H 6.760158 1.695530 -0.426997
H 5.461888 2.252171 -1.499229
H 4.543881 -1.142498 0.715675
H 7.391070 -0.133361 0.115789
H 6.228348 -2.956153 -0.051424
H 6.533408 -1.906290 -1.426762
H 8.698004 -3.024793 0.463678
H 8.395744 -3.508433 -1.207423
H 9.008313 -1.886209 -0.856014
H 6.914587 -0.292561 2.568720
H 8.084580 -1.524636 2.069946
H 6.395977 -1.973429 2.366400
SCF Energy (PCM/mPW1PW91/6-31+G**)= -1361.46348187
Number of imaginary frequencies = 0

1a_c080
B3LYP/6-31G* Geometry
C -1.727686 -2.484123 0.081159
C -2.376621 0.153999 0.347779
N -2.988655 -2.178392 0.313541
C -0.690682 -1.531002 -0.051041
C -1.021847 -0.150956 0.096361
C -3.328906 -0.856531 0.422591
C -0.007954 0.841752 -0.015690
C 1.306174 0.495568 -0.248573
C 1.659032 -0.899322 -0.373944
C 0.663761 -1.894013 -0.311204
C 3.026103 -1.272315 -0.609245
C 4.146112 -0.227056 -0.470372
C 3.637034 1.191057 -0.608586
C 2.335932 1.507080 -0.425475
O 0.946002 -3.186373 -0.476639
O 3.395722 -2.435402 -0.881061
O 5.127677 -0.493985 -1.454489
C 4.727595 -0.426375 0.979060
C 5.820876 0.577551 1.363677
C 7.197329 0.365031 0.772929
O 5.570014 1.488227 2.131308
O 4.652511 2.051312 -0.813259
C 4.382419 3.448202 -0.728281
C -4.774195 -0.579394 0.648028
C -5.537525 -1.665054 1.370277
C -5.319765 0.559423 0.175268
C -6.744966 1.042180 0.275095
C -7.414737 0.979665 -1.121440
C -8.905207 1.338670 -1.134137
C -6.771707 2.462923 0.873516
H -1.488432 -3.541731 -0.007126
H -2.665036 1.188748 0.502051
H -0.285968 1.888532 0.072101
H 2.011987 2.541714 -0.420488
H 1.917587 -3.248561 -0.687614
H 5.090440 -1.462883 -1.578016
H 5.114850 -1.452135 1.013973
H 3.920793 -0.325793 1.710992
H 7.862745 1.173273 1.083227
H 7.607763 -0.595556 1.111057
H 7.123109 0.316569 -0.318218
H 3.665535 3.756459 -1.498587
H 3.997973 3.704464 0.265489
H 5.338279 3.945365 -0.895382
H -5.095686 -1.863893 2.354604
H -6.589234 -1.409323 1.512131
H -5.474755 -2.603683 0.811409
H -4.672746 1.230416 -0.392484
H -7.314165 0.385661 0.944360
H -6.869536 1.643525 -1.808131
H -7.286922 -0.036391 -1.515885
H -9.081651 2.382446 -0.853062
H -9.329034 1.193397 -2.134262
H -9.471452 0.705558 -0.439527
H -6.285621 2.481815 1.855061
H -7.796673 2.826695 1.000532
H -6.242387 3.172052 0.223628
SCF Energy (PCM/mPW1PW91/6-31+G**)= -1361.46341322
Number of imaginary frequencies = 0

1a_c083
B3LYP/6-31G* Geometry
C 1.584613 -2.374146 -0.051032
C 2.524265 0.140733 -0.540741
N 2.886079 -2.218551 -0.201010
C 0.646389 -1.318325 -0.111958
C 1.130485 -0.004169 -0.377173
C 3.369131 -0.957596 -0.421943
C 0.214951 1.080911 -0.485462
C -1.138060 0.890034 -0.308219
C -1.642637 -0.428563 -0.019076
C -0.758565 -1.521369 0.052863
C -3.060583 -0.641006 0.152582
C -4.017687 0.566530 0.290760
C -3.396751 1.859124 -0.211766
C -2.074423 1.995214 -0.440764
O -1.185185 -2.763457 0.276284
O -3.559147 -1.780548 0.264241
O -4.227346 0.760818 1.686532
C -5.341423 0.248446 -0.450653
C -6.144917 -0.881730 0.188576
C -6.707514 -1.943969 -0.723776
O -6.371118 -0.882889 1.391918
O -4.322868 2.840657 -0.313038
C -3.882130 4.154760 -0.632174
C 4.847968 -0.845758 -0.555186
C 5.544666 -2.074519 -1.091474
C 5.474376 0.284135 -0.168336
C 6.947079 0.610250 -0.228018
C 7.631288 0.466695 1.159625
C 7.087158 1.369467 2.273454
C 7.156736 2.011601 -0.835107
H 1.230193 -3.386923 0.127816
H 2.929585 1.118158 -0.782091
H 0.599567 2.072115 -0.710607
H -1.656450 2.953601 -0.728859
H -2.181723 -2.715912 0.332791
H -4.974388 0.171855 1.937430
H -5.135746 0.036513 -1.505028
H -5.963638 1.149221 -0.409278
H -7.350793 -2.621162 -0.158770
H -7.272591 -1.489353 -1.547210
H -5.876567 -2.505769 -1.165921
H -3.177098 4.524021 0.122256

H -3.406871 4.183514 -1.620924
H -4.777369 4.778044 -0.636743
H 5.143803 -2.353135 -2.073950
H 6.622896 -1.932487 -1.187631
H 5.359470 -2.928680 -0.432905
H 4.859922 1.072745 0.266482
H 7.447562 -0.105750 -0.890244
H 7.544409 -0.580682 1.475894
H 8.704847 0.663252 1.028297
H 6.028472 1.170257 2.473596
H 7.635368 1.196745 3.206437
H 7.189144 2.432665 2.027488
H 6.769252 2.055061 -1.859058
H 8.222590 2.268040 -0.864840
H 6.642422 2.786787 -0.254885
SCF Energy (PCM/mPW1PW91/6-31+G**)= -1361.46489271
Number of imaginary frequencies = 0

1a_c086
B3LYP/6-31G* Geometry
C 1.783062 2.635631 0.082115
C 2.503576 0.024216 -0.251496
N 3.067178 2.339536 0.114463
C 0.752775 1.683078 -0.097202
C 1.123783 0.317552 -0.280610
C 3.438473 1.029276 -0.027898
C 0.123221 -0.669702 -0.504644
C -1.215107 -0.338902 -0.513996
C -1.606138 1.033847 -0.293216
C -0.629184 2.034557 -0.126390
C -2.998416 1.388215 -0.290473
C -4.073146 0.288296 -0.254796
C -3.558090 -1.041656 -0.760738
C -2.238893 -1.332465 -0.795567
O -0.953053 3.319357 0.021584
O -3.419800 2.564864 -0.262533
O -5.175840 0.716503 -1.030936
C -4.471231 0.142424 1.261907
C -5.480845 -0.976339 1.543456
C -6.926391 -0.715426 1.181562
O -5.110785 -2.016854 2.055386
O -4.574128 -1.880442 -1.038584
C -4.269253 -3.248409 -1.298048
C 4.901766 0.764146 0.045092
C 5.790259 1.917348 -0.359621
C 5.351302 -0.429611 0.483916
C 6.780185 -0.897391 0.627514
C 7.234453 -1.752177 -0.588596
C 6.425652 -3.028191 -0.851550
C 6.971253 -1.640998 1.962812
H 1.519247 3.684389 0.200689
H 2.828139 -0.996155 -0.427817
H 0.429365 -1.698139 -0.675859
H -1.897371 -2.328266 -1.054413
H -1.943342 3.382883 -0.064480
H -5.170651 1.690120 -0.944106
H -4.876749 1.115381 1.565536
H -3.575999 -0.070616 1.853473
H -6.993983 -0.440541 0.123956
H -7.526852 -1.602763 1.392282
H -7.312986 0.133567 1.760461
H -3.638861 -3.347435 -2.189766
H -3.769544 -3.700100 -0.433593
H -5.228113 -3.738232 -1.469753
H 5.592087 2.789792 0.270297
H 6.850500 1.665377 -0.296194
H 5.575179 2.228286 -1.389437
H 4.606894 -1.162446 0.795457
H 7.442518 -0.023818 0.646746
H 7.199178 -1.117886 -1.483744
H 8.290166 -2.018732 -0.438220
H 5.373049 -2.802321 -1.057511
H 6.823668 -3.560136 -1.722981
H 6.460548 -3.720326 -0.002598
H 6.743649 -0.985398 2.810487
H 8.005595 -1.988538 2.071044
H 6.314888 -2.516033 2.038277
SCF Energy (PCM/mPW1PW91/6-31+G**)= -1361.46204103
Number of imaginary frequencies = 0

1a_c090
B3LYP/6-31G* Geometry
C 1.702441 2.620303 0.039856
C 2.445493 0.018014 -0.313091
N 2.990719 2.337706 0.048085
C 0.679509 1.657934 -0.125961
C 1.062825 0.298854 -0.318539
C 3.373233 1.032658 -0.104468
C 0.065825 -0.695864 -0.524932
C -1.273436 -0.371551 -0.517416
C -1.681349 0.994659 -0.299967
C -0.708365 1.998419 -0.129349
C -3.084582 1.355277 -0.260245
C -4.148696 0.238457 -0.251844
C -3.601993 -1.087730 -0.738492
C -2.289149 -1.379934 -0.784016
O -1.035042 3.277939 0.041409
O -3.478603 2.527131 -0.145547
O -5.232245 0.670295 -1.063780
C -4.591247 0.121556 1.252996
C -5.628706 -0.967234 1.527273
C -7.082768 -0.588680 1.328957
O -5.289970 -2.070923 1.913267
O -4.630254 -1.912609 -1.064165
C -4.346370 -3.285107 -1.330651
C 4.840423 0.779289 -0.063119
C 5.708186 1.930601 -0.515303
C 5.305068 -0.401528 0.392335
C 6.729047 -0.883953 0.505394
C 6.922588 -2.140482 -0.381283
C 8.366407 -2.650119 -0.459600
C 7.073953 -1.155253 1.983802
H 1.429472 3.665568 0.166967
H 2.778604 -0.998409 -0.496648
H 0.375212 -1.723616 -0.695106
H -1.944480 -2.370910 -1.058436
H -2.033927 3.325586 -0.006493
H -5.622242 -0.134153 -1.445614
H -4.982768 1.109370 1.518894
H -3.710702 -0.098013 1.864727
H -7.711676 -1.481292 1.347668
H -7.393789 0.083239 2.140482
H -7.213642 -0.031296 0.395706
H -3.692724 -3.385856 -2.204947
H -3.880465 -3.749147 -0.455079
H -5.308740 -3.756259 -1.532344
H 5.507339 2.815277 0.096355
H 6.772810 1.696134 -0.459031
H 5.472882 2.212741 -1.549166
H 4.572638 -1.126173 0.752359
H 7.414262 -0.112323 0.134292
H 6.268093 -2.943066 -0.010459
H 6.571345 -1.903558 -1.394012
H 8.736762 -2.993346 0.512362
H 8.442135 -3.493615 -1.155277
H 9.044430 -1.864845 -0.816500
H 6.933653 -0.252540 2.587744
H 8.112776 -1.481119 2.102264
H 6.426586 -1.938499 2.399206
SCF Energy (PCM/mPW1PW91/6-31+G**)= -1361.46039771
Number of imaginary frequencies = 0

1a_c093
B3LYP/6-31G* Geometry
C 1.816143 2.533969 0.008307
C 2.519852 -0.093424 -0.225041
N 3.099162 2.231736 0.034873
C 0.779015 1.580248 -0.117955
C 1.142681 0.207528 -0.249973
C 3.463025 0.915649 -0.056443
C 0.132832 -0.783050 -0.425396
C -1.200501 -0.441797 -0.431077

C -1.585731 0.939057 -0.259034
C -0.602314 1.939341 -0.144229
C -2.978954 1.300735 -0.255814
C -4.057150 0.207455 -0.165612
C -3.550775 -1.140742 -0.630768
C -2.239788 -1.434889 -0.670122
O -0.915695 3.231114 -0.044851
O -3.390205 2.480877 -0.275591
O -5.166603 0.613039 -0.954364
C -4.447829 0.133003 1.354836
C -5.458347 -0.961447 1.725377
C -6.896033 -0.766958 1.291575
O -5.101657 -1.914604 2.389761
O -4.505496 -2.097821 -0.837049
C -5.011684 -2.199001 -2.177914
C 4.925615 0.645009 0.005434
C 5.813549 1.771030 -0.470795
C 5.371607 -0.525625 0.504999
C 6.787893 -1.013649 0.674653
C 6.996697 -2.365033 -0.049887
C 6.852411 -2.289164 -1.572901
C 7.120447 -1.148380 2.174973
H 1.559552 3.588154 0.087098
H 2.837001 -1.122106 -0.361889
H 0.429620 -1.819229 -0.561988
H -1.936519 -2.452571 -0.898681
H -1.906557 3.297860 -0.122473
H -5.163672 1.589035 -0.901105
H -4.851012 1.120726 1.614623
H -3.549205 -0.048164 1.951786
H -6.944272 -0.553261 0.220183
H -7.477103 -1.657264 1.540229
H -7.324352 0.100658 1.811510
H -5.469210 -1.258747 -2.496601
H -4.206925 -2.475741 -2.870525
H -5.760349 -2.993263 -2.153022
H 5.640994 2.668276 0.131408
H 6.872666 1.511891 -0.421693
H 5.573964 2.045339 -1.505455
H 4.627004 -1.229642 0.881448
H 7.488012 -0.292016 0.236814
H 7.997085 -2.740649 0.204528
H 6.284219 -3.099110 0.354491
H 7.586246 -1.597810 -2.005272
H 7.009731 -3.271024 -2.033459
H 5.856847 -1.935688 -1.863438
H 7.014939 -0.188815 2.692095
H 8.148726 -1.502396 2.315608
H 6.449720 -1.866626 2.663281
SCF Energy (PCM/mPW1PW91/6-31+G**)= -1361.45505276
Number of imaginary frequencies = 0

1a_c097
B3LYP/6-31G* Geometry
C 1.851291 2.363249 0.059343
C 2.456574 -0.278989 0.384992
N 3.108706 2.039652 0.287414
C 0.795994 1.426069 -0.037522
C 1.106412 0.045384 0.139220
C 3.427838 0.716294 0.428382
C 0.072419 -0.932573 0.060741
C -1.235282 -0.564353 -0.160542
C -1.569121 0.832057 -0.312759
C -0.556065 1.808811 -0.288599
C -2.935859 1.223724 -0.536587
C -4.070223 0.200810 -0.353667
C -3.584046 -1.227198 -0.473396
C -2.291631 -1.557354 -0.307193
O -0.815172 3.101921 -0.481749
O -3.284746 2.386029 -0.836136
O -5.063705 0.463097 -1.335055
C -4.630045 0.453638 1.092667
C -5.732453 -0.510234 1.553067
C -7.101791 -0.363224 0.923385
O -5.502837 -1.317246 2.432295
O -4.557466 -2.181401 -0.583260
C -4.929667 -2.547195 -1.921883
C 4.868675 0.419704 0.657681
C 5.652296 1.510855 1.349235
C 5.390540 -0.745231 0.222449
C 6.794907 -1.274685 0.362978
C 7.448233 -1.497999 -1.022222
C 7.664140 -0.213171 -1.827035
C 6.775627 -2.589508 1.169754
H 1.630764 3.422474 -0.053645
H 2.726510 -1.314860 0.562741
H 0.331008 -1.982417 0.167336
H -2.016431 -2.608008 -0.288250
H -1.788309 3.178121 -0.680672
H -5.020147 1.429000 -1.478082
H -5.005873 1.485339 1.097587
H -3.813742 0.373608 1.816480
H -7.749725 -1.174252 1.261825
H -7.542286 0.597799 1.221862
H -7.020804 -0.353208 -0.167039
H -5.311594 -1.683784 -2.472802
H -4.071907 -2.978535 -2.453006
H -5.710589 -3.302811 -1.816267
H 5.222490 1.734894 2.333548
H 6.701729 1.244560 1.486717
H 5.595769 2.438220 0.771458
H 4.728145 -1.422122 -0.320803
H 7.413565 -0.554127 0.911067
H 8.412379 -2.002539 -0.871120
H 6.825457 -2.196821 -1.599851
H 8.323419 0.482025 -1.292685
H 8.126167 -0.429203 -2.796951
H 6.717282 0.305598 -2.012246
H 6.352843 -2.436380 2.168537
H 7.790030 -2.989277 1.285248
H 6.171727 -3.353347 0.663424
SCF Energy (PCM/mPW1PW91/6-31+G**)= -1361.45501673
Number of imaginary frequencies = 0

1a_c105
B3LYP/6-31G* Geometry
C -1.993807 -1.666843 -0.186341
C -2.156279 1.055328 -0.254817
N -3.186062 -1.118228 -0.138772
C -0.781942 -0.934911 -0.268924
C -0.862793 0.487012 -0.307027
C -3.286970 0.250092 -0.171874
C 0.322633 1.266089 -0.409129
C 1.566404 0.670160 -0.447418
C 1.663453 -0.767820 -0.375467
C 0.496986 -1.558207 -0.323622
C 2.953071 -1.398843 -0.407730
C 4.229760 -0.553326 -0.257946
C 4.008318 0.900604 -0.612877
C 2.777251 1.457820 -0.615019
O 0.548811 -2.890996 -0.311959
O 3.125236 -2.633609 -0.503538
O 5.233681 -1.113300 -1.083202
C 4.624005 -0.657419 1.263098
C 5.840203 0.187352 1.659664
C 7.205279 -0.329284 1.261445
O 5.687656 1.219479 2.286705
O 5.179822 1.538082 -0.798052
C 5.165892 2.958954 -0.907784
C -4.656705 0.824547 -0.117273
C -4.771449 2.331595 -0.144247
C -5.714004 -0.011766 -0.051292
C -7.182448 0.311500 0.013119
C -7.775101 -0.246543 1.332500
C -9.242763 0.122483 1.577582
C -7.889926 -0.263508 -1.232177
H -1.952248 -2.753886 -0.159103
H -2.245689 2.135355 -0.282769
H 0.237388 2.347782 -0.468692
H 2.651435 2.524534 -0.762394
H 1.507330 -3.146987 -0.397912
H 5.028030 -2.068772 -1.100529

H 4.811571 -1.720728 1.456771
H 3.782909 -0.326586 1.879485
H 7.974555 0.382011 1.569049
H 7.394547 -1.300823 1.736493
H 7.233367 -0.490561 0.178912
H 4.587071 3.278773 -1.782471
H 4.751447 3.409603 0.001204
H 6.207802 3.257192 -1.027379
H -4.334341 2.750157 -1.060242
H -5.809702 2.663438 -0.097265
H -4.241002 2.789310 0.700860
H -5.481451 -1.075431 -0.043370
H -7.337383 1.397683 0.016463
H -7.662111 -1.340033 1.338507
H -7.167527 0.126469 2.167141
H -9.908762 -0.310788 0.823636
H -9.575070 -0.242137 2.556203
H -9.385970 1.210320 1.563609
H -7.456357 0.146578 -2.150571
H -8.959387 -0.027559 -1.230793
H -7.784064 -1.354956 -1.271714
SCF Energy (PCM/mPW1PW91/6-31+G**)= -1361.46543339
Number of imaginary frequencies = 0

1a_c109
B3LYP/6-31G* Geometry
C -1.845740 -1.213141 -0.938143
C -2.237335 1.217278 0.237613
N -3.080481 -0.831058 -0.703850
C -0.698813 -0.444134 -0.614793
C -0.901008 0.825728 -0.002631
C -3.297246 0.387577 -0.112574
C 0.216913 1.644865 0.323435
C 1.503707 1.210770 0.089088
C 1.722879 -0.088114 -0.496482
C 0.629567 -0.884203 -0.886481
C 3.066385 -0.538623 -0.747165
C 4.248773 0.190565 -0.089582
C 3.926386 1.646569 0.204612
C 2.655117 2.062427 0.357094
O 0.788844 -2.057828 -1.498294
O 3.348882 -1.519481 -1.464199
O 5.378786 0.059388 -0.937442
C 4.540696 -0.479695 1.306777
C 4.911736 -1.961834 1.218854
C 6.380302 -2.296591 1.060749
O 4.049250 -2.816525 1.290654
O 4.962063 2.462956 0.562959
C 5.793673 2.974566 -0.493855
C -4.711036 0.775536 0.133401
C -4.951237 2.116542 0.788359
C -5.695249 -0.070885 -0.236451
C -7.187131 0.086375 -0.116664
C -7.799072 -1.086207 0.687140
C -7.347394 -1.145162 2.149263
C -7.814902 0.160321 -1.524646
H -1.713181 -2.185146 -1.408716
H -2.416458 2.180463 0.701579
H 0.043824 2.627776 0.753110
H 2.476284 3.077505 0.700089
H 1.762952 -2.185077 -1.647263
H 5.170363 -0.711018 -1.505008
H 3.649624 -0.393009 1.934783
H 5.352348 0.105903 1.751418
H 6.505139 -3.365860 0.878249
H 6.813595 -1.707553 0.245179
H 6.922139 -2.019153 1.974987
H 6.308421 2.169630 -1.020647
H 5.189955 3.556949 -1.201011
H 6.514542 3.632526 -0.004223
H -4.542308 2.936391 0.183416
H -6.013982 2.316125 0.932865
H -4.468797 2.173890 1.772723
H -5.373931 -1.001507 -0.702408
H -7.436382 1.015185 0.411736
H -8.893455 -0.998424 0.644068
H -7.546769 -2.030235 0.183291
H -7.625284 -0.229774 2.686787
H -7.811481 -1.989738 2.670982
H -6.260436 -1.257735 2.226319
H -7.421394 1.011544 -2.090419
H -8.904385 0.266302 -1.457834
H -7.599981 -0.749814 -2.098031
SCF Energy (PCM/mPW1PW91/6-31+G**)= -1361.45574351
Number of imaginary frequencies = 0

1a_c116
B3LYP/6-31G* Geometry
C -2.084139 -1.568991 -0.314115
C -2.197350 1.155257 -0.229426
N -3.266784 -1.002380 -0.243101
C -0.858927 -0.855644 -0.349921
C -0.913885 0.567379 -0.306685
C -3.342796 0.367405 -0.201528
C 0.285839 1.329309 -0.358264
C 1.518896 0.713980 -0.425911
C 1.589520 -0.727353 -0.436615
C 0.408866 -1.497976 -0.434852
C 2.867780 -1.378998 -0.499310
C 4.158446 -0.566767 -0.296155
C 3.965218 0.908719 -0.569893
C 2.744498 1.487707 -0.544048
O 0.436652 -2.829951 -0.499571
O 3.018134 -2.609220 -0.664664
O 5.157292 -1.097873 -1.146119
C 4.541271 -0.762528 1.218661
C 5.768763 0.038466 1.667688
C 7.127491 -0.477469 1.247524
O 5.629228 1.036771 2.350088
O 5.148951 1.533667 -0.715232
C 5.161980 2.958655 -0.744579
C -4.702487 0.962936 -0.131501
C -4.791560 2.470744 -0.070490
C -5.776717 0.145307 -0.127002
C -7.237338 0.511468 -0.071900
C -7.819988 0.332157 1.358637
C -7.735427 -1.081944 1.946097
C -8.025628 -0.295458 -1.122362
H -2.062008 -2.656467 -0.346185
H -2.267415 2.236558 -0.195851
H 0.220273 2.413999 -0.356277
H 2.638567 2.563210 -0.630587
H 1.390875 -3.097935 -0.595957
H 4.933926 -2.046705 -1.219319
H 4.710032 -1.837969 1.353918
H 3.701994 -0.452199 1.848034
H 7.906599 0.201490 1.600152
H 7.297591 -1.478265 1.665549
H 7.159464 -0.575362 0.157515
H 4.591744 3.338229 -1.600777
H 4.753672 3.365178 0.187684
H 6.209668 3.243292 -0.845355
H -4.333111 2.937765 -0.951996
H -5.824926 2.816557 -0.015143
H -4.267115 2.865535 0.809335
H -5.562241 -0.920150 -0.168160
H -7.362803 1.572685 -0.319252
H -7.299430 1.028043 2.029440
H -8.871270 0.652476 1.334891
H -6.697485 -1.420678 2.036372
H -8.178637 -1.104806 2.948119
H -8.275046 -1.813286 1.333973
H -7.668584 -0.072736 -2.133869
H -9.094111 -0.052154 -1.078166
H -7.918749 -1.374954 -0.966700
SCF Energy (PCM/mPW1PW91/6-31+G**)= -1361.46406391
Number of imaginary frequencies = 0

1a_c123
B3LYP/6-31G* Geometry
C -1.446651 -2.316121 -0.763826
C -2.413872 0.207130 -0.374030

N -2.754041 -2.156114 -0.706191
C -0.511375 -1.264658 -0.618599
C -1.012269 0.057060 -0.427268
C -3.248947 -0.899745 -0.483875
C -0.107765 1.153582 -0.327057
C 1.254139 0.951961 -0.362632
C 1.774496 -0.385635 -0.508886
C 0.899753 -1.472217 -0.685056
C 3.198130 -0.595906 -0.544971
C 4.142236 0.524961 -0.082623
C 3.526995 1.900722 -0.283932
C 2.191603 2.066845 -0.326932
O 1.340393 -2.710956 -0.901789
O 3.735029 -1.659439 -0.913946
O 5.372669 0.384733 -0.773885
C 4.379554 0.379004 1.468698
C 5.021217 -0.950737 1.871727
C 6.533392 -0.994540 1.938424
O 4.325458 -1.913512 2.133703
O 4.349029 2.985074 -0.159605
C 5.182650 3.311941 -1.285981
C -4.731367 -0.795392 -0.392671
C -5.503519 -1.817477 -1.193854
C -5.287300 0.139164 0.404327
C -6.745466 0.425421 0.659424
C -7.061202 1.897437 0.293232
C -8.543448 2.275645 0.395088
C -7.086515 0.103904 2.129285
H -1.083665 -3.327026 -0.935565
H -2.835694 1.201239 -0.266129
H -0.507086 2.159345 -0.229098
H 1.791938 3.076927 -0.331528
H 2.331524 -2.667156 -0.960164
H 5.373113 -0.543274 -1.087177
H 3.418796 0.459228 1.984863
H 5.011731 1.227213 1.751789
H 6.873143 -2.016885 2.116318
H 6.959578 -0.597759 1.010425
H 6.887020 -0.347275 2.752215
H 5.892871 2.512200 -1.501360
H 4.562967 3.508573 -2.169765
H 5.710361 4.224761 -1.002159
H -5.216131 -2.828357 -0.889519
H -6.583316 -1.707178 -1.078095
H -5.263078 -1.737472 -2.261517
H -4.613054 0.772755 0.983335
H -7.367513 -0.212906 0.020393
H -6.466766 2.560830 0.938649
H -6.714743 2.078611 -0.732651
H -8.916437 2.207284 1.422567
H -8.704700 3.305080 0.055466
H -9.163181 1.620013 -0.229277
H -6.853328 -0.940340 2.362450
H -8.148331 0.266761 2.342221
H -6.505174 0.736296 2.812740
SCF Energy (PCM/mPW1PW91/6-31+G**)= -1361.45301913
Number of imaginary frequencies = 0

1a_c124
B3LYP/6-31G* Geometry
C -1.468303 -2.235844 -0.629053
C -2.380831 0.136416 0.367205
N -2.756729 -2.126578 -0.371546
C -0.528859 -1.196754 -0.431801
C -1.000078 0.041915 0.095294
C -3.229552 -0.934225 0.105517
C -0.088305 1.114340 0.317050
C 1.255823 0.964799 0.056943
C 1.750453 -0.293492 -0.446402
C 0.859779 -1.344009 -0.730005
C 3.154401 -0.447234 -0.724501
C 4.156172 0.578002 -0.170621
C 3.517888 1.942632 0.031782
C 2.189855 2.072275 0.211158
O 1.266058 -2.495291 -1.263695
O 3.632967 -1.391264 -1.384513
O 5.266014 0.637110 -1.052325
C 4.627419 0.102522 1.256367
C 5.323316 -1.260517 1.255790
C 6.826301 -1.267277 1.070299
O 4.678123 -2.279637 1.413110
O 4.348971 2.997708 0.283737
C 5.015252 3.596136 -0.842400
C -4.697311 -0.872325 0.349192
C -5.354196 -2.192584 0.678534
C -5.347857 0.302871 0.232396
C -6.809384 0.607170 0.442464
C -7.439390 1.094799 -0.886467
C -8.954411 1.322211 -0.829858
C -6.961539 1.643539 1.575381
H -1.124491 -3.194402 -1.011453
H -2.770525 1.050061 0.804245
H -0.469847 2.063681 0.682853
H 1.794023 3.045142 0.488440
H 2.239141 -2.409932 -1.447211
H 5.223452 -0.201184 -1.556784
H 3.756554 0.034894 1.914375
H 5.295461 0.888615 1.623890
H 7.187220 -2.291717 0.958374
H 7.099056 -0.659695 0.200525
H 7.308069 -0.806230 1.943061
H 5.691282 2.889982 -1.326895
H 4.277209 3.962719 -1.566713
H 5.574080 4.440862 -0.434399
H -4.906113 -2.636958 1.576020
H -6.428072 -2.092449 0.846933
H -5.188504 -2.908306 -0.132297
H -4.765563 1.173632 -0.073907
H -7.340260 -0.302814 0.746516
H -6.938203 2.022776 -1.198492
H -7.216763 0.352664 -1.663829
H -9.225409 2.128346 -0.139570
H -9.342454 1.593101 -1.818291
H -9.479078 0.414756 -0.505522
H -6.502597 1.279312 2.500808
H -8.014599 1.858283 1.785578
H -6.472889 2.589704 1.308452
SCF Energy (PCM/mPW1PW91/6-31+G**)= -1361.45295372
Number of imaginary frequencies = 0

1a_c135
B3LYP/6-31G* Geometry
C -1.750490 -1.350657 -0.754511
C -2.116312 1.220717 0.079231
N -2.980542 -0.934829 -0.555913
C -0.596913 -0.549884 -0.558640
C -0.783962 0.793912 -0.124534
C -3.183446 0.355257 -0.135380
C 0.340976 1.642743 0.070372
C 1.625275 1.176300 -0.119847
C 1.828071 -0.191997 -0.526948
C 0.725326 -1.028730 -0.786804
C 3.163196 -0.682989 -0.731213
C 4.362814 0.118174 -0.198067
C 4.048228 1.597664 -0.079501
C 2.779177 2.052110 0.023360
O 0.872903 -2.276065 -1.232953
O 3.431598 -1.760263 -1.300425
O 5.464970 -0.090471 -1.059107
C 4.693608 -0.388144 1.257319
C 5.019342 -1.881153 1.344113
C 6.459175 -2.287079 1.106504
O 4.149083 -2.685757 1.617217
O 5.174449 2.331709 0.028994
C 5.044452 3.737534 0.208326
C -4.592306 0.778420 0.079557
C -4.820495 2.200998 0.536633
C -5.582679 -0.112256 -0.137466
C -7.070233 0.060674 0.012572
C -7.590123 -0.892293 1.119724
C -9.076704 -0.727358 1.455958
C -7.757182 -0.195460 -1.345203

H -1.627706 -2.378906 -1.088531
H -2.286633 2.239314 0.409059
H 0.175623 2.675654 0.365145
H 2.577086 3.100564 0.212786
H 1.845101 -2.426903 -1.375225
H 5.257492 -0.926505 -1.522703
H 3.834454 -0.195187 1.906065
H 5.544013 0.215559 1.592149
H 6.534697 -3.373266 1.024441
H 6.849685 -1.796276 0.209011
H 7.077811 -1.946088 1.947907
H 4.493649 4.189575 -0.625288
H 4.533178 3.969531 1.151248
H 6.061672 4.130067 0.234555
H -4.415273 2.923479 -0.183856
H -5.880896 2.424790 0.662506
H -4.327057 2.397160 1.497562
H -5.270581 -1.102629 -0.465056
H -7.309450 1.085783 0.322379
H -7.391430 -1.930358 0.817314
H -6.996305 -0.721236 2.026894
H -9.722936 -0.984009 0.609596
H -9.358403 -1.377876 2.291770
H -9.305033 0.305279 1.748662
H -7.374191 0.487693 -2.110896
H -8.841120 -0.053164 -1.279985
H -7.570441 -1.220494 -1.689245
SCF Energy (PCM/mPW1PW91/6-31+G**)= -1361.46428648
Number of imaginary frequencies = 0

1a_c153
B3LYP/6-31G* Geometry
C -1.779376 -1.251667 -0.828670
C -2.155794 1.218560 0.266243
N -3.011196 -0.859530 -0.595728
C -0.628234 -0.473752 -0.543958
C -0.822615 0.817524 0.024547
C -3.220401 0.378791 -0.044054
C 0.299625 1.647079 0.306928
C 1.583277 1.203755 0.072895
C 1.795007 -0.116455 -0.466077
C 0.696662 -0.925193 -0.813848
C 3.135147 -0.577437 -0.715287
C 4.325346 0.175197 -0.099580
C 4.007168 1.642220 0.139838
C 2.738130 2.064613 0.292700
O 0.848185 -2.120730 -1.383700
O 3.408776 -1.584849 -1.398327
O 5.446489 0.008539 -0.952784
C 4.632129 -0.438884 1.319071
C 5.000997 -1.923859 1.285926
C 6.467703 -2.266341 1.127229
O 4.138453 -2.774000 1.399218
O 5.047992 2.471626 0.450507
C 5.861480 2.942641 -0.638749
C -4.631017 0.777333 0.202482
C -4.861213 2.129867 0.837137
C -5.620668 -0.070364 -0.148849
C -7.110220 0.094066 -0.010566
C -7.671416 -1.033752 0.892640
C -9.166835 -0.914172 1.208468
C -7.758915 0.096345 -1.410915
H -1.652865 -2.239709 -1.266385
H -2.329155 2.197579 0.698158
H 0.132070 2.645445 0.701766
H 2.563827 3.092585 0.597546
H 1.820475 -2.254390 -1.539029
H 5.230113 -0.781619 -1.489360
H 3.748394 -0.325954 1.953220
H 5.449404 0.163162 1.730181
H 6.589883 -3.341999 0.984855
H 6.893753 -1.709627 0.285575
H 7.018232 -1.954614 2.025087
H 6.369032 2.118407 -1.142260
H 5.245314 3.496370 -1.358102
H 6.589153 3.619909 -0.186946
H -5.921661 2.332751 0.994042
H -4.364051 2.203649 1.813045
H -4.461248 2.939413 0.212563
H -5.306494 -1.010565 -0.599410
H -7.348514 1.050613 0.471366
H -7.470251 -2.003883 0.416328
H -7.105608 -1.033816 1.833239
H -9.788122 -1.016466 0.312286
H -9.476447 -1.695547 1.911766
H -9.399756 0.055122 1.667125
H -7.342095 0.896528 -2.031847
H -8.841988 0.247073 -1.350584
H -7.577983 -0.855089 -1.926744
SCF Energy (PCM/mPW1PW91/6-31+G**)= -1361.45484613
Number of imaginary frequencies = 0

1a_c156
B3LYP/6-31G* Geometry
C 1.704783 2.592431 0.085188
C 2.455500 -0.001010 -0.319159
N 2.993664 2.315377 0.074665
C 0.684399 1.627209 -0.083821
C 1.072546 0.272809 -0.305294
C 3.381369 1.014805 -0.102120
C 0.079345 -0.725605 -0.525645
C -1.260247 -0.411942 -0.489447
C -1.669418 0.947779 -0.227528
C -0.703801 1.959107 -0.066979
C -3.069034 1.280029 -0.180567
C -4.124438 0.161696 -0.140648
C -3.599710 -1.145974 -0.693105
C -2.284011 -1.409882 -0.771203
O -1.040377 3.235834 0.116200
O -3.503537 2.450538 -0.121958
O -5.253890 0.592113 -0.886979
C -4.490031 -0.010652 1.378175
C -5.475693 -1.143385 1.696853
C -6.924081 -0.946981 1.300908
O -5.091869 -2.129598 2.294511
O -4.538145 -2.109414 -0.940215
C -5.071653 -2.136921 -2.274084
C 4.849167 0.768526 -0.077686
C 5.708681 1.933376 -0.510739
C 5.322164 -0.419361 0.350880
C 6.748828 -0.896302 0.447817
C 6.937241 -2.149089 -0.445192
C 8.382324 -2.651698 -0.541908
C 7.109089 -1.171466 1.922021
H 1.428927 3.634415 0.231945
H 2.791154 -1.012345 -0.524026
H 0.393768 -1.745358 -0.729686
H -1.965010 -2.405830 -1.064835
H -2.033491 3.287124 0.058626
H -5.268085 1.563028 -0.774780
H -4.906285 0.952447 1.701866
H -3.579273 -0.210632 1.950168
H -7.485575 -1.861829 1.500624
H -7.357287 -0.123089 1.884126
H -6.995788 -0.664472 0.246896
H -5.551870 -1.187367 -2.524114
H -4.277412 -2.355463 -2.998912
H -5.805825 -2.944925 -2.283057
H 5.519304 2.800153 0.129677
H 6.773893 1.697378 -0.478896
H 5.456394 2.245318 -1.531833
H 4.595588 -1.154490 0.701312
H 7.427304 -0.120717 0.072671
H 6.290778 -2.955688 -0.069170
H 6.573278 -1.910766 -1.453132
H 8.765665 -2.995928 0.424624
H 8.453317 -3.493008 -1.240620
H 9.052512 -1.862482 -0.904822
H 6.972726 -0.271163 2.530417
H 8.149954 -1.494785 2.028185
H 6.468331 -1.957949 2.341336
SCF Energy (PCM/mPW1PW91/6-31+G**)= -1361.45413171

Number of imaginary frequencies = 0

1a_c160
B3LYP/6-31G* Geometry
C 1.729390 2.471124 -0.089255
C 2.409204 -0.134771 0.358878
N 2.996556 2.193345 0.145347
C 0.699967 1.501835 -0.134719
C 1.049475 0.140298 0.106586
C 3.353144 0.887385 0.346485
C 0.042746 -0.868508 0.080791
C -1.275483 -0.547760 -0.151209
C -1.648822 0.830114 -0.368021
C -0.663247 1.834476 -0.396532
C -3.026982 1.172600 -0.601820
C -4.130922 0.129087 -0.359784
C -3.606076 -1.289193 -0.419603
C -2.304176 -1.575568 -0.246397
O -0.959221 3.109107 -0.650403
O -3.410101 2.308647 -0.955435
O -5.142101 0.319264 -1.339289
C -4.681601 0.432688 1.080310
C -5.747547 -0.542080 1.599128
C -7.128840 -0.466115 0.982924
O -5.481605 -1.301377 2.510086
O -4.552848 -2.274080 -0.480881
C -4.925241 -2.705289 -1.799816
C 4.803530 0.641134 0.574133
C 5.564331 1.786235 1.201035
C 5.352607 -0.528313 0.187915
C 6.779559 -0.997371 0.316013
C 7.404128 -1.161682 -1.092484
C 8.895306 -1.517831 -1.094283
C 6.822563 -2.307483 1.129269
H 1.479086 3.517364 -0.250861
H 2.708724 -1.153163 0.584028
H 0.331027 -1.904485 0.235867
H -1.999594 -2.616260 -0.182150
H -1.934796 3.148679 -0.847733
H -5.124389 1.277979 -1.528944
H -5.089020 1.451589 1.041984
H -3.854785 0.411309 1.796265
H -7.747587 -1.278139 1.370082
H -7.594083 0.495215 1.239937
H -7.062915 -0.508053 -0.107827
H -5.334996 -1.876383 -2.382787
H -4.059955 -3.134916 -2.320023
H -5.684244 -3.476772 -1.656021
H 5.136397 2.050210 2.176186
H 6.621406 1.554363 1.343560
H 5.479661 2.681010 0.577037
H 4.703908 -1.248743 -0.313456
H 7.371066 -0.248030 0.855577
H 6.841060 -1.928379 -1.644541
H 7.259239 -0.223086 -1.642556
H 9.087332 -2.500036 -0.648852
H 9.285369 -1.541402 -2.118117
H 9.480261 -0.777491 -0.534345
H 6.378921 -2.166323 2.120766
H 7.850178 -2.659250 1.268484
H 6.261761 -3.102789 0.621106
SCF Energy (PCM/mPW1PW91/6-31+G**)= -1361.45409592
Number of imaginary frequencies = 0

1a_c178
B3LYP/6-31G* Geometry
C 1.993983 1.623780 -0.268875
C 2.184828 -1.096687 -0.190555
N 3.192099 1.089475 -0.205834
C 0.789009 0.876338 -0.296173
C 0.886570 -0.543864 -0.257537
C 3.308620 -0.276697 -0.164746
C -0.293833 -1.339636 -0.302386
C -1.540273 -0.755711 -0.351612
C -1.655517 0.682271 -0.355989
C -0.497609 1.484848 -0.366586
C -2.955127 1.298170 -0.399923
C -4.217669 0.448036 -0.176249
C -3.983630 -1.019206 -0.463104
C -2.752026 -1.557611 -0.462634
O -0.561058 2.815504 -0.428426
O -3.138763 2.523346 -0.567264
O -5.246475 0.954114 -1.015035
C -4.584870 0.641795 1.339530
C -5.775879 -0.184241 1.844218
C -7.160129 0.225039 1.386132
O -5.592088 -1.096141 2.626458
O -5.104566 -1.798191 -0.545611
C -5.654855 -1.966886 -1.862029
C 4.684503 -0.833565 -0.093339
C 4.814384 -2.338728 -0.043134
C 5.733182 0.016156 -0.078668
C 7.205175 -0.288138 -0.006970
C 7.795769 0.331489 1.285701
C 9.269411 -0.007135 1.538763
C 7.902321 0.241292 -1.277906
H 1.941879 2.710171 -0.300313
H 2.284753 -2.175661 -0.160314
H -0.200316 -2.422121 -0.306536
H -2.649685 -2.634835 -0.557776
H -1.523092 3.058375 -0.513305
H -5.057902 1.910628 -1.085803
H -4.788838 1.712957 1.468478
H -3.724444 0.370774 1.958319
H -7.893988 -0.498530 1.746741
H -7.403298 1.217521 1.789067
H -7.192165 0.306409 0.296055
H -5.931936 -1.003505 -2.297912
H -4.935752 -2.478289 -2.514199
H -6.540716 -2.592275 -1.735089
H 4.369186 -2.808951 -0.929672
H 5.856508 -2.657783 0.006197
H 4.300583 -2.756610 0.832370
H 5.489424 1.076436 -0.122856
H 7.371607 -1.371482 0.041731
H 7.667990 1.422683 1.247525
H 7.196888 -0.015210 2.137762
H 9.926550 0.405531 0.765746
H 9.599717 0.400486 2.500907
H 9.428120 -1.092518 1.566996
H 7.468544 -0.209579 -2.176886
H 8.973596 0.014018 -1.271426
H 7.787665 1.329345 -1.361521
SCF Energy (PCM/mPW1PW91/6-31+G**)= -1361.45609522
Number of imaginary frequencies = 0

1a_c260
B3LYP/6-31G* Geometry
C 1.921187 -2.374995 -0.210876
C 2.552102 0.267860 -0.464336
N 3.192521 -2.053432 -0.355122
C 0.864980 -1.436330 -0.165041
C 1.187813 -0.055638 -0.306283
C 3.521303 -0.729377 -0.458779
C 0.149720 0.918328 -0.302197
C -1.168785 0.552220 -0.139905
C -1.513022 -0.837732 0.021850
C -0.503279 -1.819379 -0.008857
C -2.890362 -1.240441 0.197355
C -3.997626 -0.170444 0.397548
C -3.522199 1.228635 0.037452
C -2.227982 1.548669 -0.159486
O -0.774223 -3.117574 0.106992
O -3.237328 -2.433745 0.249664
O -4.275727 -0.221340 1.791939
C -5.228648 -0.590213 -0.448218
C -6.527463 0.111713 -0.077943
C -7.423175 0.556371 -1.210351
O -6.854891 0.266835 1.091380
O -4.564115 2.099112 0.011388
C -4.287061 3.486882 -0.143973
C 4.974749 -0.432415 -0.595607

C 5.794733 -1.512466 -1.261960
C 5.472685 0.721972 -0.108428
C 6.883791 1.250738 -0.157492
C 7.445519 1.475950 1.266343
C 7.607849 0.192666 2.086153
C 6.919085 2.564661 -0.965496
H 1.690834 -3.434723 -0.125965
H 2.835121 1.305018 -0.613045
H 0.411007 1.965604 -0.429217
H -1.928056 2.574680 -0.343822
H -1.768377 -3.193227 0.192358
H -5.226878 0.001095 1.893920
H -5.372982 -1.662558 -0.263530
H -5.011395 -0.468133 -1.514689
H -8.361533 0.950559 -0.815566
H -6.911034 1.330992 -1.794256
H -7.625737 -0.279063 -1.892135
H -3.636679 3.846060 0.662170
H -3.813539 3.689315 -1.112902
H -5.252083 3.992903 -0.092381
H 5.423773 -1.713002 -2.274991
H 6.852014 -1.249947 -1.330718
H 5.697889 -2.451598 -0.709104
H 4.780314 1.390422 0.407215
H 7.536888 0.529099 -0.662684
H 8.418216 1.979291 1.178092
H 6.787063 2.176271 1.801079
H 8.295255 -0.506306 1.593637
H 8.012294 0.409998 3.081240
H 6.649990 -0.322549 2.216458
H 6.563805 2.410093 -1.990061
H 7.938830 2.965302 -1.013334
H 6.282126 3.328739 -0.501663
SCF Energy (PCM/mPW1PW91/6-31+G**)= -1361.46647563
Number of imaginary frequencies = 0

1a_c272
B3LYP/6-31G* Geometry
C -1.857654 -2.583796 0.039524
C -2.615575 0.031537 -0.138497
N -3.148677 -2.313365 0.016891
C -0.840524 -1.603406 -0.008936
C -1.230887 -0.236545 -0.108147
C -3.539987 -1.003985 -0.049063
C -0.237022 0.780664 -0.173581
C 1.104641 0.467646 -0.148270
C 1.518535 -0.909619 -0.056001
C 0.550628 -1.929828 0.025684
C 2.921299 -1.257814 -0.027782
C 4.003494 -0.169816 -0.264679
C 3.435328 1.238885 -0.191159
C 2.115712 1.512469 -0.182575
O 0.883398 -3.213999 0.135617
O 3.318031 -2.425495 0.134473
O 4.459885 -0.418297 -1.588475
C 5.131134 -0.383525 0.780317
C 6.439204 0.326652 0.461210
C 7.159371 1.002610 1.604396
O 6.911303 0.309595 -0.668116
O 4.432993 2.160138 -0.199279
C 4.086016 3.537103 -0.300024
C -5.011344 -0.771902 -0.042095
C -5.850040 -1.910379 -0.574401
C -5.514474 0.378721 0.449695
C -6.962338 0.802173 0.541717
C -7.400871 1.646112 -0.687343
C -6.617506 2.943316 -0.921499
C -7.225037 1.534072 1.870838
H -1.578381 -3.633619 0.095855
H -2.950180 1.057583 -0.253192
H -0.551382 1.819111 -0.237020
H 1.757403 2.536273 -0.198182
H 1.883416 -3.246734 0.159165
H 5.408428 -0.164809 -1.608541
H 5.338265 -1.461371 0.787717
H 4.774299 -0.111337 1.779351
H 8.125053 1.384582 1.267827
H 6.543955 1.828631 1.981414
H 7.303252 0.302607 2.436893
H 3.515858 3.728054 -1.216696
H 3.499601 3.861420 0.568881
H 5.030055 4.082716 -0.332269
H -5.655909 -2.824247 -0.005016
H -6.918629 -1.688970 -0.541354
H -5.581000 -2.135180 -1.613987
H -4.807863 1.105446 0.850286
H -7.596247 -0.092700 0.535877
H -7.318135 1.015280 -1.581733
H -8.468024 1.883439 -0.572257
H -5.552711 2.743805 -1.088313
H -6.996977 3.465141 -1.807238
H -6.701372 3.634095 -0.074719
H -7.003898 0.884154 2.724714
H -8.273707 1.845726 1.944737
H -6.601920 2.430843 1.969817
SCF Energy (PCM/mPW1PW91/6-31+G**)= -1361.46414184
Number of imaginary frequencies = 0

1a_c292
B3LYP/6-31G* Geometry
C -2.126371 -1.632563 -0.141724
C -2.353570 1.084826 -0.175193
N -3.333691 -1.113405 -0.160694
C -0.931269 -0.870946 -0.138305
C -1.046278 0.547082 -0.155624
C -3.466669 0.251355 -0.177498
C 0.126145 1.352290 -0.146373
C 1.381444 0.781623 -0.133129
C 1.517083 -0.651713 -0.124148
C 0.365639 -1.465643 -0.113233
C 2.823947 -1.268630 -0.108917
C 4.102643 -0.401239 -0.266008
C 3.817678 1.085449 -0.119246
C 2.576469 1.609955 -0.100917
O 0.439738 -2.794962 -0.081802
O 2.982942 -2.498968 -0.018681
O 4.539407 -0.661430 -1.593876
C 5.138186 -0.888449 0.783106
C 6.566108 -0.425876 0.530060
C 7.364040 0.048292 1.721762
O 7.060804 -0.479743 -0.588372
O 4.975352 1.793661 -0.072478
C 4.904698 3.215103 -0.105130
C -4.852080 0.791993 -0.196750
C -5.002685 2.295993 -0.202795
C -5.890487 -0.069777 -0.213042
C -7.368284 0.213395 -0.250467
C -8.087775 -0.466125 0.939296
C -7.671611 0.071696 2.311569
C -7.956914 -0.281149 -1.588856
H -2.059390 -2.718477 -0.128649
H -2.467431 2.162934 -0.189052
H 0.021731 2.434143 -0.147861
H 2.424031 2.683207 -0.060403
H 1.413197 -3.024355 -0.054723
H 5.519356 -0.600367 -1.582381
H 5.134487 -1.984538 0.725790
H 4.811127 -0.611379 1.791052
H 8.393926 0.258064 1.426727
H 6.901017 0.956205 2.127567
H 7.348943 -0.704745 2.519519
H 4.395766 3.558025 -1.013546
H 4.380596 3.605366 0.776356
H 5.937306 3.566795 -0.105773
H -4.524616 2.742324 -1.084611
H -6.049834 2.602353 -0.208879
H -4.532148 2.749497 0.679289
H -5.631623 -1.127797 -0.212872
H -7.556534 1.292045 -0.178469
H -9.170705 -0.335636 0.807083
H -7.901125 -1.548692 0.894165
H -7.881353 1.145561 2.395665

H -8.215746 -0.436630 3.115655
H -6.599771 -0.071413 2.486814
H -7.487789 0.224566 -2.439569
H -9.036769 -0.093918 -1.630911
H -7.797277 -1.359415 -1.712079
SCF Energy (PCM/mPW1PW91/6-31+G**)= -1361.46844782
Number of imaginary frequencies = 0

1a_c339
B3LYP/6-31G* Geometry
C -1.860710 -2.598442 0.041061
C -2.622115 0.010968 -0.198279
N -3.151997 -2.332471 -0.007434
C -0.845220 -1.616246 -0.012107
C -1.237088 -0.252624 -0.144015
C -3.544617 -1.025417 -0.104214
C -0.245016 0.765338 -0.219612
C 1.097017 0.456007 -0.174125
C 1.512758 -0.917798 -0.046442
C 0.546160 -1.938340 0.046882
C 2.915953 -1.261911 0.004268
C 3.998338 -0.177280 -0.247076
C 3.426298 1.231411 -0.215314
C 2.106106 1.502058 -0.224320
O 0.880476 -3.218918 0.189282
O 3.313441 -2.424482 0.197859
O 4.471124 -0.457394 -1.558620
C 5.114042 -0.361738 0.816343
C 6.423571 0.344678 0.494743
C 7.125819 1.056116 1.627397
O 6.910482 0.297518 -0.627422
O 4.422008 2.154445 -0.239301
C 4.073191 3.527272 -0.381876
C -5.015920 -0.791935 -0.129658
C -5.838236 -1.917420 -0.712447
C -5.523944 0.349724 0.377644
C -6.965551 0.792406 0.442981
C -7.292675 1.855876 -0.642800
C -6.487021 3.158528 -0.572577
C -7.311690 1.286831 1.860779
H -1.579478 -3.646060 0.122860
H -2.959489 1.033013 -0.338096
H -0.560904 1.801344 -0.309556
H 1.745826 2.524323 -0.268456
H 1.880181 -3.248744 0.225217
H 5.419926 -0.204503 -1.573083
H 5.324150 -1.438404 0.852695
H 4.744494 -0.065973 1.804000
H 8.093576 1.434037 1.292221
H 6.501336 1.888715 1.973824
H 7.263115 0.380200 2.480651
H 3.509829 3.690745 -1.308004
H 3.479411 3.875098 0.472815
H 5.016402 4.073765 -0.422377
H -5.640716 -2.847515 -0.171341
H -6.909537 -1.709233 -0.684580
H -5.554654 -2.103396 -1.756101
H -4.820839 1.055067 0.820995
H -7.615541 -0.064289 0.231383
H -7.141244 1.395701 -1.627767
H -8.364003 2.091466 -0.571784
H -5.412680 2.975156 -0.687968
H -6.791175 3.839816 -1.375230
H -6.637359 3.684880 0.376874
H -7.180754 0.484794 2.595336
H -8.352670 1.628199 1.912059
H -6.670183 2.121392 2.167656
SCF Energy (PCM/mPW1PW91/6-31+G**)= -1361.46423679
Number of imaginary frequencies = 0

1a_c367
B3LYP/6-31G* Geometry
C -1.883148 -1.517565 -0.254387
C -2.329160 1.167153 -0.079926
N -3.128146 -1.096387 -0.259985
C -0.753960 -0.665900 -0.166185
C -0.983204 0.735579 -0.072982
C -3.370963 0.250608 -0.174130
C 0.119289 1.628818 0.031041
C 1.416460 1.161510 0.021986
C 1.666091 -0.252477 -0.088753
C 0.586280 -1.154775 -0.158528
C 3.019894 -0.753030 -0.083041
C 4.212960 0.223450 -0.213437
C 3.823785 1.660188 0.091601
C 2.541546 2.073820 0.155930
O 0.769304 -2.473377 -0.221664
O 3.284091 -1.972976 -0.039818
O 4.599808 0.215106 -1.584272
C 5.360253 -0.259460 0.711451
C 5.978272 -1.587052 0.279051
C 6.212789 -2.629948 1.344266
O 6.324336 -1.765252 -0.881827
O 4.916962 2.449604 0.206058
C 4.725401 3.851221 0.353086
C -4.794771 0.680329 -0.185457
C -5.065009 2.164679 -0.094867
C -5.760678 -0.257602 -0.277998
C -7.255624 -0.090611 -0.326533
C -7.933466 -0.869742 0.826482
C -7.577149 -0.354549 2.223979
C -7.786740 -0.579075 -1.690863
H -1.728526 -2.592363 -0.323809
H -2.529743 2.230304 -0.010425
H -0.073690 2.694572 0.121342
H 2.302876 3.121129 0.305317
H 1.755455 -2.626269 -0.173951
H 5.233437 -0.530256 -1.687407
H 5.006778 -0.309705 1.746591
H 6.154243 0.494204 0.668600
H 6.766118 -2.203507 2.190378
H 5.242517 -2.970654 1.723917
H 6.762471 -3.475595 0.926663
H 5.724427 4.286618 0.401557
H 4.182654 4.263507 -0.505997
H 4.177051 4.081014 1.275542
H -4.604022 2.706932 -0.930740
H -6.132927 2.387602 -0.109532
H -4.652959 2.591216 0.828950
H -5.418434 -1.289777 -0.340610
H -7.530843 0.966099 -0.217839
H -9.021601 -0.819064 0.682895
H -7.662045 -1.931909 0.743619
H -7.873803 0.694826 2.346459
H -8.087152 -0.936197 3.000254
H -6.499167 -0.417988 2.408354
H -7.347386 -0.007592 -2.515446
H -8.877194 -0.474154 -1.743193
H -7.542419 -1.636532 -1.850119
SCF Energy (PCM/mPW1PW91/6-31+G**)= -1361.46889084
Number of imaginary frequencies = 0

1b_c001
B3LYP/6-31G* Geometry
C -1.573458 -2.491867 -0.086625
C -2.378953 0.111124 0.058808
N -2.861152 -2.250716 0.066322
C -0.584051 -1.487388 -0.193568
C -0.997653 -0.125613 -0.109682
C -3.277573 -0.947939 0.116174
C -0.031490 0.915863 -0.189222
C 1.308215 0.632791 -0.348140
C 1.742300 -0.737867 -0.434896
C 0.802878 -1.782969 -0.362823
C 3.138806 -1.046066 -0.599087
C 4.189807 0.071311 -0.749763
C 3.608587 1.461017 -0.608566
C 2.292379 1.698485 -0.439909
O 1.160851 -3.064053 -0.448722
O 3.572764 -2.211549 -0.729190
O 4.682942 -0.029433 -2.095051
C 5.365270 -0.165267 0.224592

C 4.994619 -0.097156 1.696797
C 6.138499 0.051192 2.681503
O 3.834900 -0.170958 2.061953
O 4.583865 2.399224 -0.681441
C 4.202817 3.768548 -0.634969
C -4.746275 -0.743729 0.262221
C -5.484489 -1.847740 0.982586
C -5.328936 0.349975 -0.269003
C -6.779810 0.761079 -0.252016
C -6.947042 2.175739 0.352458
C -6.554216 2.276596 1.829335
C -7.358361 0.713335 -1.681085
H -1.272518 -3.536477 -0.126991
H -2.730179 1.132600 0.164486
H -0.365168 1.948220 -0.126038
H 1.919159 2.713081 -0.355278
H 2.151937 -3.075807 -0.570804
H 4.863497 -0.977139 -2.231740
H 6.165007 0.542386 -0.013574
H 5.757700 -1.173303 0.030921
H 5.767371 -0.060163 3.702236
H 6.923710 -0.689099 2.485704
H 6.598509 1.041382 2.567788
H 3.526881 4.015291 -1.462708
H 3.715327 4.011124 0.317812
H 5.126853 4.340355 -0.731841
H -5.099556 -1.972146 2.002565
H -6.558250 -1.660349 1.042491
H -5.318845 -2.802289 0.474295
H -4.691931 1.039544 -0.826538
H -7.357770 0.066583 0.369644
H -6.352659 2.887856 -0.238993
H -7.995469 2.480789 0.231250
H -5.504635 2.002956 1.983287
H -6.696897 3.295450 2.207069
H -7.163427 1.604560 2.446346
H -8.413686 1.011756 -1.684848
H -7.285331 -0.294108 -2.104221
H -6.815394 1.395373 -2.347687
SCF Energy (PCM/mPW1PW91/6-31+G**)= -1361.46591582
Number of imaginary frequencies = 0

1b_c004
B3LYP/6-31G* Geometry
C 1.650403 2.369986 -0.152011
C 2.351160 -0.229904 -0.595946
N 2.934727 2.078922 -0.227129
C 0.611899 1.417891 -0.273901
C 0.973220 0.059830 -0.512601
C 3.297725 0.774843 -0.426530
C -0.041497 -0.927171 -0.670597
C -1.373662 -0.591950 -0.579977
C -1.755828 0.773009 -0.328211
C -0.771133 1.766663 -0.187414
C -3.147732 1.125979 -0.224829
C -4.260873 0.070672 -0.405880
C -3.728064 -1.314491 -0.711357
C -2.414288 -1.591672 -0.762806
O -1.078995 3.045547 0.028608
O -3.548077 2.300980 -0.076390
O -5.039732 0.517846 -1.528299
C -5.176890 0.043167 0.843536
C -4.487187 -0.360862 2.139315
C -5.361750 -0.976708 3.212519
O -3.294356 -0.179336 2.302042
O -4.628577 -2.345865 -0.780862
C -5.559736 -2.352177 -1.875309
C 4.762931 0.511180 -0.480187
C 5.610361 1.664114 -0.965470
C 5.245411 -0.683030 -0.081201
C 6.665499 -1.189675 -0.082819
C 7.110068 -1.610293 1.337599
C 7.179318 -0.454212 2.339751
C 6.792003 -2.376107 -1.061597
H 1.393973 3.414352 0.011877
H 2.663860 -1.245365 -0.816722
H 0.249046 -1.956362 -0.862884
H -2.111052 -2.619531 -0.940625
H -2.074900 3.100791 0.050242
H -5.142714 1.478972 -1.396857
H -6.033471 -0.605190 0.637217
H -5.567026 1.060776 0.987862
H -4.802110 -1.065435 4.145698
H -6.268599 -0.380778 3.372410
H -5.686838 -1.973647 2.887204
H -6.271890 -1.526036 -1.810729
H -5.026469 -2.289690 -2.830919
H -6.081791 -3.309105 -1.807256
H 5.447353 2.542225 -0.333607
H 6.675165 1.424092 -0.970302
H 5.320130 1.959049 -1.981777
H 4.529850 -1.411220 0.305454
H 7.344949 -0.399808 -0.424925
H 6.421871 -2.383134 1.710547
H 8.095642 -2.090258 1.263527
H 6.205963 0.035679 2.451885
H 7.495494 -0.807546 3.327773
H 7.897670 0.308263 2.013919
H 7.819021 -2.760466 -1.076473
H 6.522375 -2.079549 -2.080932
H 6.131160 -3.201402 -0.766944
SCF Energy (PCM/mPW1PW91/6-31+G**)= -1361.45623836
Number of imaginary frequencies = 0

1b_c009
B3LYP/6-31G* Geometry
C -1.459011 -2.538055 -0.150909
C -2.309633 0.042111 0.102243
N -2.749723 -2.326602 0.019588
C -0.488328 -1.512378 -0.224019
C -0.925811 -0.162550 -0.085311
C -3.188825 -1.034520 0.124533
C 0.020633 0.898920 -0.131573
C 1.364156 0.646809 -0.308647
C 1.822724 -0.711645 -0.447407
C 0.902799 -1.775867 -0.410563
C 3.223844 -0.987786 -0.626841
C 4.254089 0.153119 -0.738897
C 3.647545 1.526270 -0.551109
C 2.328049 1.733221 -0.368373
O 1.283884 -3.045980 -0.545647
O 3.678820 -2.139602 -0.800488
O 4.749882 0.106324 -2.086005
C 5.433548 -0.094313 0.228348
C 5.063047 -0.077341 1.702100
C 6.204340 0.069997 2.690010
O 3.905929 -0.189385 2.065718
O 4.604827 2.484556 -0.597411
C 4.198121 3.844204 -0.506192
C -4.658781 -0.860577 0.293949
C -5.369800 -2.001596 0.983472
C -5.263216 0.245102 -0.185156
C -6.713763 0.648528 -0.101448
C -6.835196 1.963864 0.710075
C -8.273914 2.414451 0.988211
C -7.306711 0.780236 -1.518559
H -1.139716 -3.574626 -0.234449
H -2.678493 1.051724 0.252097
H -0.331599 1.921820 -0.028174
H 1.936517 2.737241 -0.247867
H 2.274549 -3.034739 -0.671345
H 4.945334 -0.833311 -2.254485
H 6.221721 0.632908 0.011633
H 5.841590 -1.089721 0.003937
H 5.837522 -0.080339 3.707306
H 7.007270 -0.644619 2.471973
H 6.639614 1.074163 2.605058
H 3.513677 4.103850 -1.322944
H 3.710475 4.047298 0.455714
H 5.110661 4.436354 -0.588776
H -4.978813 -2.147172 1.998504
H -6.447290 -1.840300 1.052646

H -5.184525 -2.936824 0.446929
H -4.643551 0.965327 -0.722437
H -7.283839 -0.120142 0.434166
H -6.312674 1.826352 1.665632
H -6.295551 2.761672 0.178753
H -8.841476 1.630264 1.504685
H -8.284032 3.305023 1.627017
H -8.813759 2.665646 0.068892
H -8.368800 1.045699 -1.488331
H -7.210632 -0.161869 -2.068637
H -6.782295 1.556001 -2.091563
SCF Energy (PCM/mPW1PW91/6-31+G**)= -1361.46500251
Number of imaginary frequencies = 0

1b_c013
B3LYP/6-31G* Geometry
C -1.545002 -2.556854 -0.162114
C -2.391368 0.015183 0.175638
N -2.833439 -2.350210 0.029416
C -0.574956 -1.529459 -0.218012
C -1.009465 -0.184294 -0.032588
C -3.270702 -1.061294 0.173901
C -0.062614 0.877384 -0.057558
C 1.277909 0.630362 -0.263377
C 1.732803 -0.723023 -0.453860
C 0.812798 -1.787584 -0.434453
C 3.130007 -0.993550 -0.670257
C 4.156590 0.151220 -0.775071
C 3.557489 1.516170 -0.516516
C 2.242143 1.717376 -0.300606
O 1.190399 -3.052996 -0.615544
O 3.580251 -2.139790 -0.887152
O 4.602749 0.155528 -2.140798
C 5.369179 -0.132432 0.138752
C 5.049288 -0.172353 1.623783
C 6.225627 -0.082182 2.576844
O 3.903568 -0.283896 2.022059
O 4.515383 2.474499 -0.544151
C 4.113569 3.829700 -0.387401
C -4.740444 -0.891728 0.351263
C -5.444543 -2.039270 1.036665
C -5.348231 0.215106 -0.122419
C -6.807471 0.592906 -0.053370
C -7.058553 1.752721 0.950219
C -6.356161 3.079954 0.638469
C -7.343307 0.918902 -1.461595
H -1.227097 -3.590608 -0.279076
H -2.758871 1.020059 0.357795
H -0.412062 1.896610 0.084328
H 1.854709 2.716382 -0.134143
H 2.178770 -3.037944 -0.758172
H 4.800177 -0.775522 -2.349469
H 6.149901 0.603328 -0.076099
H 5.768846 -1.117904 -0.137961
H 5.891470 -0.270481 3.599062
H 7.009661 -0.797861 2.301303
H 6.672786 0.918651 2.519010
H 3.414101 4.124808 -1.178988
H 3.645041 3.992271 0.591520
H 5.025685 4.423497 -0.462465
H -5.056491 -2.179430 2.053698
H -6.523504 -1.887428 1.103665
H -5.247867 -2.973031 0.501704
H -4.725723 0.936665 -0.652130
H -7.383391 -0.260129 0.322946
H -8.143048 1.925240 0.999492
H -6.753747 1.413145 1.948592
H -6.656121 3.484274 -0.334857
H -6.606647 3.831515 1.395710
H -5.265627 2.971014 0.634259
H -8.396444 1.222690 -1.418721
H -7.267762 0.044959 -2.117547
H -6.778980 1.732175 -1.932703
SCF Energy (PCM/mPW1PW91/6-31+G**)= -1361.46360626
Number of imaginary frequencies = 0

1b_c018
B3LYP/6-31G* Geometry
C -1.812789 -1.504005 -0.489513
C -2.060530 1.111931 0.241076
N -3.019425 -1.038187 -0.257746
C -0.627161 -0.734321 -0.383133
C -0.753894 0.631058 -0.001629
C -3.163467 0.274709 0.111340
C 0.408172 1.444713 0.116144
C 1.661676 0.926274 -0.124723
C 1.806876 -0.453717 -0.504076
C 0.669151 -1.270624 -0.641027
C 3.114587 -1.001819 -0.749293
C 4.385069 -0.130517 -0.640661
C 4.092932 1.311485 -0.278532
C 2.850624 1.759426 -0.029733
O 0.755014 -2.549854 -1.008035
O 3.304958 -2.170937 -1.149737
O 4.987220 -0.160133 -1.945588
C 5.377500 -0.760357 0.369139
C 4.865303 -0.859076 1.799496
C 5.909210 -0.871554 2.897630
O 3.674342 -0.941065 2.038448
O 5.158312 2.142705 -0.047372
C 5.999911 2.482742 -1.161489
C -4.547867 0.754408 0.365781
C -4.709785 2.203051 0.765781
C -5.574803 -0.110852 0.233708
C -7.048683 0.121122 0.432703
C -7.794612 -0.126671 -0.903508
C -9.293443 0.193763 -0.866881
C -7.563646 -0.792200 1.564950
H -1.737942 -2.549976 -0.779779
H -2.181950 2.148973 0.532919
H 0.296268 2.487477 0.400569
H 2.727013 2.796460 0.269165
H 1.723134 -2.742006 -1.152138
H 4.919893 -1.090038 -2.232710
H 6.320532 -0.207584 0.327590
H 5.585774 -1.784869 0.029220
H 5.442727 -1.115405 3.854098
H 6.708065 -1.589369 2.674594
H 6.378040 0.119010 2.963677
H 6.549708 1.616830 -1.538031
H 5.402070 2.907337 -1.976266
H 6.693155 3.236831 -0.782444
H -4.316993 2.878137 -0.005736
H -5.755471 2.467633 0.929960
H -4.165076 2.426131 1.692499
H -5.309605 -1.125571 -0.059006
H -7.238435 1.160997 0.726983
H -7.317247 0.483077 -1.681466
H -7.646892 -1.173698 -1.204156
H -9.469907 1.230259 -0.552489
H -9.740036 0.068956 -1.859942
H -9.839270 -0.460351 -0.178477
H -8.629973 -0.632021 1.757076
H -7.021555 -0.599937 2.497042
H -7.420813 -1.849507 1.308455
SCF Energy (PCM/mPW1PW91/6-31+G**)= -1361.45728402
Number of imaginary frequencies = 0

1b_c020
B3LYP/6-31G* Geometry
C 1.623737 2.443451 0.219305
C 2.367270 -0.029275 -0.664451
N 2.912471 2.194077 0.089860
C 0.601751 1.505877 -0.056921
C 0.984900 0.215932 -0.527181
C 3.296667 0.949145 -0.328270
C -0.013762 -0.747934 -0.847516
C -1.350877 -0.459046 -0.691950
C -1.754419 0.834414 -0.205226
C -0.786231 1.807861 0.097526
C -3.150982 1.137363 -0.032033
C -4.246881 0.108464 -0.385180

C -3.693567 -1.193041 -0.929288
C -2.375575 -1.431577 -1.038441
O -1.114583 3.024682 0.531979
O -3.570795 2.261285 0.319672
O -5.043661 0.728576 -1.408656
C -5.150261 -0.150823 0.846836
C -4.439769 -0.754195 2.050445
C -5.293847 -1.547548 3.018502
O -3.246821 -0.587438 2.227565
O -4.575635 -2.214626 -1.169055
C -5.532276 -2.043859 -2.227566
C 4.765325 0.723229 -0.424459
C 5.592837 1.953540 -0.714438
C 5.270690 -0.509545 -0.215893
C 6.711044 -0.957324 -0.278324
C 7.335265 -1.112213 1.136014
C 6.649570 -2.120246 2.066238
C 6.829125 -2.251106 -1.108241
H 1.349516 3.440024 0.558303
H 2.697886 -0.986050 -1.055694
H 0.293249 -1.722900 -1.216114
H -2.056293 -2.406950 -1.394495
H -2.111051 3.056488 0.568950
H -5.160910 1.650663 -1.112654
H -5.995964 -0.772925 0.539278
H -5.560094 0.818413 1.164993
H -4.718811 -1.784943 3.915653
H -6.201169 -0.993677 3.289028
H -5.618703 -2.479444 2.537593
H -6.258165 -1.259670 -1.999616
H -5.023449 -1.796800 -3.166400
H -6.034258 -3.009079 -2.323762
H 5.430833 2.705808 0.063504
H 6.660321 1.732208 -0.772635
H 5.284011 2.418168 -1.659229
H 4.568681 -1.299096 0.053178
H 7.303817 -0.190965 -0.791351
H 8.389536 -1.396870 1.009527
H 7.335845 -0.126455 1.618631
H 6.647255 -3.132256 1.645479
H 7.171852 -2.168046 3.028415
H 5.610735 -1.838239 2.270649
H 7.869858 -2.594775 -1.148531
H 6.486381 -2.087967 -2.136058
H 6.225344 -3.062309 -0.684629
SCF Energy (PCM/mPW1PW91/6-31+G**)= -1361.45408868
Number of imaginary frequencies = 0

1b_c021
B3LYP/6-31G* Geometry
C -1.886694 -1.534179 -0.169214
C -2.085299 1.164233 0.169585
N -3.081005 -1.022979 0.029055
C -0.691130 -0.774085 -0.216444
C -0.791122 0.635390 -0.042489
C -3.198484 0.332537 0.204761
C 0.379222 1.441069 -0.089429
C 1.622394 0.878718 -0.291614
C 1.740690 -0.545617 -0.460859
C 0.591329 -1.359255 -0.429767
C 3.032933 -1.144421 -0.664913
C 4.306340 -0.281119 -0.763110
C 4.046928 1.193987 -0.549829
C 2.816361 1.705841 -0.347702
O 0.656136 -2.681075 -0.594681
O 3.198617 -2.367225 -0.868225
O 4.777155 -0.421705 -2.112508
C 5.392282 -0.820854 0.194932
C 5.033470 -0.754601 1.670251
C 6.173123 -0.926514 2.656289
O 3.884370 -0.584700 2.036728
O 5.204315 1.897366 -0.596024
C 5.133756 3.313277 -0.483706
C -4.567764 0.864420 0.435328
C -4.699788 2.354447 0.653245
C -5.612803 0.010426 0.437522
C -7.074058 0.315877 0.648494
C -7.836113 0.431737 -0.702117
C -7.805842 -0.813394 -1.596610
C -7.707869 -0.732915 1.582680
H -1.830734 -2.612577 -0.303169
H -2.188418 2.235028 0.304778
H 0.283448 2.516160 0.037979
H 2.675862 2.772019 -0.208226
H 1.618828 -2.904329 -0.735540
H 4.735271 -1.377093 -2.299697
H 6.329528 -0.293122 -0.006036
H 5.558259 -1.877957 -0.055548
H 5.777480 -1.013959 3.670137
H 6.774427 -1.810416 2.411374
H 6.844087 -0.059382 2.601788
H 4.522147 3.739802 -1.288040
H 4.718524 3.612826 0.486946
H 6.160055 3.672630 -0.571847
H -4.341382 2.918622 -0.217737
H -5.735026 2.651684 0.828747
H -4.107875 2.688742 1.515307
H -5.371773 -1.035107 0.259239
H -7.179945 1.291047 1.139876
H -8.880381 0.692689 -0.478530
H -7.416586 1.280155 -1.258407
H -8.253444 -1.683259 -1.103079
H -8.370732 -0.634631 -2.518626
H -6.782174 -1.079632 -1.881970
H -8.775728 -0.530080 1.729274
H -7.223094 -0.723527 2.565157
H -7.609523 -1.746808 1.178422
SCF Energy (PCM/mPW1PW91/6-31+G**)= -1361.46558278
Number of imaginary frequencies = 0

1b_c023
B3LYP/6-31G* Geometry
C -1.609135 -2.486990 -0.038344
C -2.438340 0.109145 0.094581
N -2.901656 -2.255987 0.091269
C -0.626419 -1.474750 -0.129155
C -1.052980 -0.117137 -0.051212
C -3.329599 -0.957163 0.135381
C -0.092358 0.931464 -0.104183
C 1.251130 0.658466 -0.250103
C 1.698048 -0.707395 -0.351687
C 0.767478 -1.759668 -0.269155
C 3.106113 -1.009886 -0.507240
C 4.100412 0.134616 -0.814714
C 3.550970 1.501649 -0.453833
C 2.232292 1.732482 -0.279254
O 1.138787 -3.037401 -0.324658
O 3.543786 -2.174139 -0.520911
O 4.344838 0.097462 -2.236956
C 5.461282 -0.161842 -0.182210
C 5.426492 -0.181903 1.334986
C 6.758515 -0.336421 2.043177
O 4.377923 -0.086173 1.948478
O 4.528600 2.439243 -0.443154
C 4.161247 3.790590 -0.191514
C -4.801948 -0.764329 0.258858
C -5.543492 -1.877428 0.961580
C -5.383981 0.328592 -0.274686
C -6.837238 0.731486 -0.275274
C -7.020212 2.140633 0.337680
C -6.647195 2.232235 1.820297
C -7.395680 0.691774 -1.712504
H -1.299154 -3.529132 -0.072746
H -2.798889 1.127711 0.197015
H -0.432188 1.960565 -0.022020
H 1.858857 2.737365 -0.115377
H 2.137611 -3.038100 -0.394964
H 3.521687 0.361826 -2.681659
H 6.192046 0.570558 -0.538876
H 5.785626 -1.144862 -0.542749
H 6.599249 -0.403917 3.121101
H 7.281608 -1.232950 1.688767

H 7.406854 0.520607 1.821090
H 3.488303 4.167260 -0.972003
H 3.675895 3.889759 0.787009
H 5.090922 4.361282 -0.203310
H -5.170635 -2.007773 1.985239
H -6.618827 -1.695683 1.009400
H -5.366985 -2.827442 0.448402
H -4.743356 1.025832 -0.818418
H -7.419997 0.029186 0.332973
H -6.421960 2.860394 -0.240528
H -8.068568 2.441084 0.204970
H -5.598528 1.961882 1.985763
H -6.799304 3.247603 2.203673
H -7.261383 1.552982 2.424301
H -8.452403 0.984747 -1.728575
H -7.311558 -0.311972 -2.142324
H -6.847218 1.381842 -2.366269
SCF Energy (PCM/mPW1PW91/6-31+G**)= -1361.46554871
Number of imaginary frequencies = 0

1b_c024
B3LYP/6-31G* Geometry
C -1.680303 -2.356279 -0.253339
C -2.399929 0.260984 -0.534268
N -2.967117 -2.071354 -0.312792
C -0.649169 -1.391071 -0.317041
C -1.018831 -0.023357 -0.471132
C -3.338142 -0.759521 -0.429730
C -0.011106 0.977462 -0.559787
C 1.326290 0.646979 -0.504071
C 1.718218 -0.731679 -0.359020
C 0.736862 -1.734009 -0.247324
C 3.119273 -1.094535 -0.298745
C 4.195224 -0.035621 -0.636600
C 3.670825 1.384410 -0.543643
C 2.352886 1.676282 -0.565102
O 1.052703 -3.017248 -0.082706
O 3.501255 -2.261204 -0.098261
O 4.607735 -0.286079 -1.997039
C 5.455063 -0.276283 0.197707
C 5.240260 -0.064073 1.685105
C 6.472208 -0.135858 2.566428
O 4.131178 0.146511 2.144073
O 4.682701 2.284986 -0.546736
C 4.351257 3.668538 -0.535593
C -4.805356 -0.502164 -0.465928
C -5.641651 -1.613731 -1.055687
C -5.296952 0.647590 0.037839
C -6.721364 1.139780 0.083899
C -7.178546 1.388295 1.540775
C -7.234764 0.124548 2.403880
C -6.853623 2.430701 -0.750593
H -1.415557 -3.406607 -0.152969
H -2.721969 1.285991 -0.688198
H -0.310343 2.017321 -0.661817
H 2.009589 2.704720 -0.591443
H 2.051779 -3.057471 -0.030109
H 3.856318 -0.069729 -2.574795
H 6.259299 0.368638 -0.169552
H 5.768416 -1.313141 0.029372
H 6.182838 -0.047492 3.615385
H 7.007301 -1.079859 2.406890
H 7.167636 0.673069 2.308971
H 3.791813 3.946412 -1.437638
H 3.760580 3.923441 0.352704
H 5.301296 4.204393 -0.514807
H -5.469455 -2.543862 -0.506027
H -6.709076 -1.385702 -1.040519
H -5.347732 -1.811576 -2.094353
H -4.586555 1.342118 0.490521
H -7.391549 0.389256 -0.352357
H -6.503187 2.122855 2.003828
H -8.170694 1.859894 1.515995
H -6.253729 -0.358422 2.468578
H -7.564432 0.357972 3.422642
H -7.937171 -0.607918 1.987091
H -7.884611 2.804257 -0.729905
H -6.573056 2.258319 -1.795278
H -6.204292 3.222529 -0.355490
SCF Energy (PCM/mPW1PW91/6-31+G**)= -1361.46554411
Number of imaginary frequencies = 0

1b_c026
B3LYP/6-31G* Geometry
C -1.901544 -1.485534 -0.265973
C -2.103595 1.186550 0.240879
N -3.098026 -0.985146 -0.053777
C -0.704696 -0.725959 -0.246738
C -0.807793 0.669065 0.016557
C -3.218549 0.356290 0.205039
C 0.365174 1.474760 0.040810
C 1.607746 0.921274 -0.177503
C 1.729754 -0.488522 -0.436754
C 0.580373 -1.299672 -0.479633
C 3.026267 -1.072294 -0.658400
C 4.307656 -0.210149 -0.653857
C 4.039024 1.261219 -0.411076
C 2.806935 1.744509 -0.178443
O 0.644821 -2.607351 -0.733401
O 3.196155 -2.274687 -0.956390
O 4.878462 -0.359176 -1.965115
C 5.317391 -0.762189 0.383920
C 4.840968 -0.721937 1.829336
C 5.912912 -0.659856 2.898474
O 3.655262 -0.754275 2.103585
O 5.116777 2.096305 -0.270202
C 5.958986 2.308990 -1.414807
C -4.590924 0.875057 0.445614
C -4.726387 2.351754 0.739088
C -5.634703 0.021159 0.390292
C -7.098891 0.312061 0.598056
C -7.851899 0.452381 -0.755483
C -7.810715 -0.773592 -1.675672
C -7.731477 -0.762846 1.503322
H -1.844833 -2.553335 -0.467192
H -2.207191 2.246672 0.442779
H 0.270495 2.539709 0.234958
H 2.700618 2.804961 0.031664
H 1.607689 -2.823991 -0.877633
H 4.795967 -1.309513 -2.169414
H 6.266312 -0.230048 0.270038
H 5.503952 -1.816223 0.133469
H 5.466795 -0.807461 3.883924
H 6.691582 -1.411837 2.721361
H 6.402372 0.322319 2.864485
H 6.493923 1.400982 -1.703421
H 5.365110 2.658163 -2.267316
H 6.665230 3.086341 -1.115114
H -4.350312 2.960217 -0.093745
H -5.764672 2.641102 0.909367
H -4.152156 2.639573 1.629442
H -5.389749 -1.013677 0.161955
H -7.215776 1.274906 1.110420
H -8.898735 0.703981 -0.533380
H -7.432604 1.314195 -1.290928
H -8.256480 -1.655882 -1.202922
H -8.372085 -0.578032 -2.596423
H -6.784572 -1.028897 -1.962082
H -8.801986 -0.571412 1.645888
H -7.254427 -0.772103 2.489536
H -7.622192 -1.766330 1.076616
SCF Energy (PCM/mPW1PW91/6-31+G**)= -1361.45598509
Number of imaginary frequencies = 0

1b_c031
B3LYP/6-31G* Geometry
C -1.497410 -2.533422 -0.094481
C -2.369357 0.040838 0.145260
N -2.792598 -2.330104 0.053446
C -0.532656 -1.501571 -0.152925
C -0.981761 -0.155466 -0.020356
C -3.242069 -1.041474 0.151863

C -0.039860 0.911094 -0.041155
C 1.306926 0.667365 -0.206731
C 1.776706 -0.685910 -0.360734
C 0.864926 -1.756663 -0.311807
C 3.188845 -0.957874 -0.534091
C 4.161851 0.213920 -0.804671
C 3.590175 1.557964 -0.395419
C 2.268915 1.759009 -0.204685
O 1.258068 -3.024761 -0.416531
O 3.646532 -2.113076 -0.592052
O 4.402252 0.229880 -2.227932
C 5.529864 -0.079920 -0.186319
C 5.501346 -0.148192 1.329601
C 6.839326 -0.293174 2.028548
O 4.453231 -0.096030 1.949066
O 4.551190 2.512066 -0.359336
C 4.161458 3.848049 -0.063581
C -4.715616 -0.876713 0.298087
C -5.430898 -2.023866 0.972789
C -5.318706 0.227204 -0.186756
C -6.772411 0.622937 -0.124042
C -6.913107 1.934842 0.689974
C -8.358279 2.376358 0.948466
C -7.344128 0.756886 -1.549619
H -1.170003 -3.567918 -0.171616
H -2.746669 1.047881 0.291390
H -0.396914 1.930282 0.081480
H 1.878813 2.750802 -0.003950
H 2.256350 -3.005200 -0.491700
H 3.572167 0.491499 -2.661216
H 6.247438 0.674756 -0.522685
H 5.868331 -1.046029 -0.578316
H 6.685362 -0.406824 3.103366
H 7.387005 -1.159567 1.638278
H 7.461942 0.590381 1.839049
H 3.476584 4.236375 -0.827808
H 3.681058 3.908394 0.920544
H 5.080665 4.435577 -0.063452
H -5.054000 -2.171451 1.992816
H -6.510050 -1.868047 1.026827
H -5.233334 -2.956232 0.435617
H -4.694745 0.952749 -0.711842
H -7.346811 -0.150501 0.399956
H -6.403949 1.797095 1.652686
H -6.370204 2.737447 0.169239
H -8.928499 1.587602 1.454928
H -8.382762 3.265260 1.589199
H -8.886506 2.626627 0.022131
H -8.407458 1.018556 -1.534767
H -7.236396 -0.183111 -2.101149
H -6.813891 1.536373 -2.112148
SCF Energy (PCM/mPW1PW91/6-31+G**)= -1361.46465819
Number of imaginary frequencies = 0

1b_c034
B3LYP/6-31G* Geometry
C 1.801852 2.547152 0.164727
C 2.485480 -0.092350 0.039411
N 3.076548 2.232503 0.283062
C 0.766164 1.604266 -0.033895
C 1.116419 0.222442 -0.093846
C 3.432747 0.913199 0.198070
C 0.106508 -0.762291 -0.283827
C -1.221734 -0.409396 -0.393690
C -1.593109 0.983460 -0.305152
C -0.604538 1.976635 -0.163514
C -2.975116 1.362100 -0.409730
C -4.075268 0.288395 -0.354232
C -3.562253 -1.087169 -0.720704
C -2.250301 -1.403842 -0.653667
O -0.907315 3.274617 -0.133808
O -3.370441 2.544930 -0.496579
O -5.117752 0.673130 -1.230371
C -4.569070 0.271531 1.140906
C -5.618901 -0.803486 1.445037
C -7.033043 -0.546496 0.973007
O -5.304637 -1.806105 2.059498
O -4.579524 -1.926910 -0.990903
C -4.291539 -3.316390 -1.125247
C 4.890313 0.630335 0.314424
C 5.682617 1.634846 1.118215
C 5.419168 -0.440479 -0.312009
C 6.848020 -0.920837 -0.341553
C 6.954947 -2.368374 0.197135
C 6.582951 -2.516290 1.675368
C 7.402324 -0.837209 -1.778322
H 1.550047 3.603439 0.231048
H 2.789078 -1.134187 0.037166
H 0.398631 -1.806400 -0.356350
H -1.917124 -2.423403 -0.811396
H -1.889978 3.348912 -0.279111
H -5.097185 1.650294 -1.218190
H -4.970484 1.272562 1.341298
H -3.716839 0.090864 1.802621
H -7.664492 -1.405325 1.209646
H -7.436760 0.349633 1.462279
H -7.028246 -0.349979 -0.103997
H -3.613547 -3.496408 -1.968001
H -3.852421 -3.708181 -0.200743
H -5.249961 -3.800523 -1.314495
H 5.288819 1.714145 2.139003
H 6.740825 1.374508 1.179207
H 5.587679 2.630952 0.675433
H 4.748109 -1.052002 -0.918344
H 7.470871 -0.283910 0.297937
H 6.315989 -3.023224 -0.413611
H 7.984766 -2.717923 0.041934
H 5.551964 -2.196172 1.861670
H 6.678149 -3.557463 2.003744
H 7.237278 -1.906275 2.310307
H 8.441631 -1.184788 -1.815571
H 7.371084 0.190310 -2.155939
H 6.815807 -1.462790 -2.463135
SCF Energy (PCM/mPW1PW91/6-31+G**)= -1361.46438635
Number of imaginary frequencies = 0

1b_c035
B3LYP/6-31G* Geometry
C 1.862466 2.447948 0.019624
C 2.445665 -0.145547 -0.600611
N 3.132048 2.102558 -0.061749
C 0.782256 1.555661 -0.177247
C 1.080511 0.201189 -0.512016
C 3.434820 0.798079 -0.347061
C 0.025939 -0.721410 -0.761153
C -1.294678 -0.341589 -0.648814
C -1.610308 1.016561 -0.272636
C -0.582185 1.959598 -0.079509
C -2.982975 1.420076 -0.140974
C -4.099397 0.361906 -0.134620
C -3.676828 -0.930329 -0.798685
C -2.376569 -1.264511 -0.952880
O -0.839717 3.235316 0.209962
O -3.350531 2.603150 0.026022
O -5.231254 0.903434 -0.788257
C -4.402586 0.083897 1.385514
C -5.439329 -1.018777 1.629637
C -6.892937 -0.669694 1.397525
O -5.081979 -2.117582 2.012377
O -4.744500 -1.699597 -1.083342
C -4.517396 -3.043840 -1.498957
C 4.885675 0.465861 -0.395476
C 5.796194 1.596861 -0.812902
C 5.300864 -0.768325 -0.045050
C 6.694076 -1.344234 -0.043896
C 7.110787 -1.794103 1.376556
C 7.238193 -0.647159 2.383377
C 6.765849 -2.529906 -1.028749
H 1.652387 3.489811 0.251853
H 2.716186 -1.155741 -0.890336
H 0.275550 -1.739073 -1.048922
H -2.095822 -2.242249 -1.327966

H -1.829663 3.344016 0.197627
H -5.177652 1.863293 -0.611402
H -4.743724 1.036993 1.808062
H -3.480368 -0.219287 1.889827
H -7.516577 -1.550327 1.564519
H -7.202101 0.132032 2.080923
H -7.019204 -0.289564 0.378561
H -3.952849 -3.073332 -2.438506
H -3.981067 -3.600377 -0.721954
H -5.505931 -3.478226 -1.650338
H 5.662602 2.452333 -0.144167
H 6.848174 1.305998 -0.809251
H 5.540954 1.950360 -1.819940
H 4.542754 -1.475317 0.297845
H 7.413363 -0.587256 -0.378959
H 6.380814 -2.530356 1.743915
H 8.069314 -2.326023 1.303033
H 6.293027 -0.103834 2.490532
H 7.527100 -1.020967 3.372154
H 8.000581 0.074793 2.065446
H 7.773780 -2.961474 -1.044407
H 6.512129 -2.216041 -2.047027
H 6.067095 -3.325157 -0.738959
SCF Energy (PCM/mPW1PW91/6-31+G**)= -1361.46434391
Number of imaginary frequencies = 0

1b_c036
B3LYP/6-31G* Geometry
C -1.579319 -2.555192 -0.096834
C -2.447868 0.013492 0.208259
N -2.872590 -2.355100 0.070126
C -0.615246 -1.522173 -0.144731
C -1.061806 -0.179292 0.024347
C -3.320563 -1.068618 0.198721
C -0.119232 0.886848 0.018144
C 1.225141 0.646479 -0.171327
C 1.691304 -0.702775 -0.366287
C 0.779637 -1.774002 -0.329745
C 3.100160 -0.970853 -0.569933
C 4.068233 0.207415 -0.829289
C 3.506807 1.539311 -0.369459
C 2.188817 1.736577 -0.152995
O 1.170122 -3.039321 -0.471286
O 3.555972 -2.124433 -0.663496
O 4.275275 0.263514 -2.256920
C 5.449760 -0.105294 -0.251755
C 5.456270 -0.217107 1.261877
C 6.808676 -0.396565 1.923987
O 4.423717 -0.172259 1.907501
O 4.470031 2.490275 -0.319773
C 4.088187 3.816766 0.025201
C -4.793966 -0.907143 0.352049
C -5.502024 -2.056315 1.030588
C -5.400793 0.194627 -0.134490
C -6.863075 0.564314 -0.088832
C -7.136565 1.723660 0.909520
C -6.434014 3.053394 0.609011
C -7.378566 0.886296 -1.505557
H -1.252934 -3.587759 -0.199752
H -2.824154 1.017114 0.379192
H -0.473633 1.902888 0.170899
H 1.803553 2.722604 0.082397
H 2.167097 -3.018300 -0.561737
H 3.435979 0.539321 -2.662818
H 6.160489 0.658377 -0.582409
H 5.777890 -1.059644 -0.679513
H 6.679587 -0.530025 2.999802
H 7.331649 -1.263874 1.502906
H 7.441291 0.480186 1.736319
H 3.392407 4.230991 -0.715181
H 3.623522 3.845926 1.018245
H 5.008727 4.402178 0.029889
H -5.126916 -2.192416 2.052977
H -6.582409 -1.908975 1.082845
H -5.294259 -2.990563 0.500668
H -4.774417 0.918422 -0.656615
H -7.440125 -0.291669 0.278939
H -8.222462 1.891769 0.939546
H -6.847928 1.386156 1.913365
H -6.717596 3.455132 -0.370259
H -6.701304 3.804935 1.360484
H -5.343181 2.948531 0.625013
H -8.434136 1.183345 -1.479278
H -7.287098 0.012607 -2.159807
H -6.812161 1.703122 -1.968003
SCF Energy (PCM/mPW1PW91/6-31+G**)= -1361.46319417
Number of imaginary frequencies = 0

1b_c038
B3LYP/6-31G* Geometry
C 1.901879 -1.505793 0.153376
C 2.346558 1.125005 -0.413831
N 3.145899 -1.102734 0.021825
C 0.772684 -0.659209 0.022846
C 1.002467 0.712655 -0.275519
C 3.389132 0.216245 -0.261782
C -0.102408 1.598684 -0.426313
C -1.395426 1.150639 -0.271781
C -1.647317 -0.232388 0.041649
C -0.567406 -1.128512 0.170743
C -3.001734 -0.716284 0.188015
C -4.195111 0.268864 0.179068
C -3.799333 1.671249 -0.239004
C -2.526926 2.053474 -0.431980
O -0.749317 -2.421977 0.430726
O -3.264111 -1.925503 0.356882
O -4.675130 0.380430 1.517003
C -5.287781 -0.287468 -0.775082
C -5.962771 -1.556887 -0.262963
C -6.119318 -2.706622 -1.227293
O -6.403212 -1.611330 0.878478
O -4.847997 2.538304 -0.409558
C -5.123015 3.415649 0.694022
C 4.812381 0.624778 -0.396266
C 5.084027 2.081695 -0.694012
C 5.777054 -0.308153 -0.252825
C 7.271682 -0.161119 -0.350529
C 7.957114 -0.657049 0.945499
C 7.613181 0.166408 2.190189
C 7.792383 -0.950449 -1.570470
H 1.748399 -2.559154 0.378422
H 2.546507 2.166100 -0.640696
H 0.086828 2.641412 -0.666861
H -2.320598 3.083597 -0.710752
H -1.738309 -2.565488 0.476779
H -5.330986 -0.342537 1.640496
H -4.864458 -0.441302 -1.773128
H -6.060757 0.485507 -0.859237
H -6.722686 -3.496414 -0.775840
H -6.579427 -2.369664 -2.164487
H -5.124451 -3.097483 -1.472398
H -5.375630 2.838553 1.587440
H -4.261295 4.062104 0.901505
H -5.971791 4.029579 0.384808
H 4.674218 2.734118 0.087909
H 6.152166 2.290688 -0.768320
H 4.622353 2.388859 -1.641537
H 5.434100 -1.321306 -0.047265
H 7.547828 0.891821 -0.488556
H 7.683271 -1.709032 1.110509
H 9.044121 -0.644429 0.786037
H 6.536947 0.150395 2.393899
H 8.129449 -0.223142 3.074863
H 7.912177 1.214891 2.066152
H 8.882799 -0.864811 -1.650704
H 7.349930 -0.580207 -2.501461
H 7.544171 -2.015383 -1.483154
SCF Energy (PCM/mPW1PW91/6-31+G**)= -1361.46021914
Number of imaginary frequencies = 0

1b_c041
B3LYP/6-31G* Geometry

C -1.806007 -2.541791 0.112063
C -2.502214 0.096242 0.048255
N -3.082615 -2.235089 0.237957
C -0.774749 -1.590628 -0.065661
C -1.132319 -0.211306 -0.092123
C -3.445093 -0.916663 0.183997
C -0.122926 0.779300 -0.252381
C 1.204345 0.428341 -0.372013
C 1.586818 -0.961633 -0.331586
C 0.599738 -1.957427 -0.201247
C 2.979152 -1.353447 -0.427942
C 4.071896 -0.266184 -0.368653
C 3.532307 1.115476 -0.675815
C 2.228001 1.438204 -0.599594
O 0.900994 -3.254425 -0.190340
O 3.346619 -2.538639 -0.468917
O 5.093200 -0.629779 -1.287216
C 4.606476 -0.321356 1.109666
C 5.688295 0.710040 1.430929
C 7.117115 0.321099 1.108901
O 5.403107 1.776258 1.944460
O 4.559312 1.948436 -0.983571
C 4.297478 3.347268 -1.084820
C -4.904112 -0.642210 0.308120
C -5.689947 -1.665176 1.094661
C -5.438874 0.437823 -0.296662
C -6.870262 0.911711 -0.314153
C -6.981859 2.353705 0.237650
C -6.602500 2.490757 1.715071
C -7.432478 0.837737 -1.748404
H -1.549642 -3.598086 0.153923
H -2.809777 1.136755 0.070616
H -0.414071 1.825605 -0.290788
H 1.893468 2.459888 -0.742934
H 1.892490 -3.317430 -0.313802
H 5.478379 0.203097 -1.608206
H 4.985657 -1.340024 1.244611
H 3.770792 -0.149972 1.795189
H 7.772861 1.190759 1.188215
H 7.452000 -0.444573 1.821842
H 7.178756 -0.131876 0.113926
H 3.589345 3.553935 -1.895709
H 3.905422 3.725893 -0.135129
H 5.257184 3.816282 -1.303368
H -5.299795 -1.754385 2.116137
H -6.751206 -1.417094 1.154320
H -5.582060 -2.653888 0.638623
H -4.771805 1.063242 -0.893238
H -7.486250 0.266134 0.323344
H -6.349716 3.017296 -0.370796
H -8.014303 2.699369 0.091338
H -5.568646 2.175101 1.892923
H -6.702118 3.528360 2.053379
H -7.249594 1.871068 2.348047
H -8.473595 1.180832 -1.777100
H -7.398461 -0.186405 -2.134789
H -6.852552 1.471647 -2.431177
SCF Energy (PCM/mPW1PW91/6-31+G**)= -1361.46131993
Number of imaginary frequencies = 0

1b_c043
B3LYP/6-31G* Geometry
C 1.866621 2.438978 -0.041838
C 2.465236 -0.168358 -0.582575
N 3.138649 2.097650 -0.112272
C 0.791545 1.536149 -0.213357
C 1.098842 0.175375 -0.504684
C 3.449393 0.787855 -0.358402
C 0.046392 -0.758290 -0.719668
C -1.273813 -0.374935 -0.623256
C -1.604012 0.992194 -0.304053
C -0.576828 1.938985 -0.126592
C -2.984886 1.412019 -0.168365
C -4.097358 0.343315 -0.159931
C -3.642145 -0.971434 -0.759346
C -2.348904 -1.318056 -0.895815
O -0.832778 3.219508 0.134033
O -3.316387 2.591572 0.031758
O -5.208736 0.869805 -0.871198
C -4.449865 0.150288 1.361122
C -5.525688 -0.901523 1.633103
C -6.965726 -0.434361 1.568200
O -5.223789 -2.047230 1.912418
O -4.724378 -1.726482 -1.078341
C -4.521596 -3.089410 -1.447852
C 4.902653 0.462830 -0.398311
C 5.806068 1.588708 -0.843902
C 5.326101 -0.759727 -0.018790
C 6.723030 -1.326950 -0.006585
C 7.140594 -1.751879 1.421118
C 7.257672 -0.589132 2.410915
C 6.804160 -2.527544 -0.972474
H 1.650893 3.486044 0.159084
H 2.740317 -1.185917 -0.840747
H 0.297098 -1.786531 -0.966347
H -2.066560 -2.303795 -1.249261
H -1.829236 3.315321 0.139530
H -5.655426 0.107875 -1.277476
H -4.769955 1.136515 1.714100
H -3.545745 -0.152945 1.898093
H -7.642583 -1.291263 1.581335
H -7.178383 0.202075 2.437919
H -7.129577 0.186148 0.680987
H -3.921041 -3.160459 -2.362193
H -4.032719 -3.631492 -0.631643
H -5.514946 -3.502619 -1.624831
H 5.663634 2.461045 -0.199270
H 6.860193 1.306036 -0.829242
H 5.551181 1.912778 -1.860904
H 4.573013 -1.463481 0.341403
H 7.438187 -0.570917 -0.352521
H 6.415537 -2.488016 1.798443
H 8.103305 -2.277571 1.357631
H 6.308125 -0.051841 2.509183
H 7.548666 -0.945857 3.405403
H 8.014478 0.134211 2.082953
H 7.814530 -2.953738 -0.979103
H 6.550986 -2.231047 -1.996071
H 6.109121 -3.322066 -0.671887
SCF Energy (PCM/mPW1PW91/6-31+G**)= -1361.46121424
Number of imaginary frequencies = 0

1b_c044
B3LYP/6-31G* Geometry
C -1.651484 -2.446286 -0.021933
C -2.415179 0.108564 -0.602260
N -2.942727 -2.193902 -0.120118
C -0.637306 -1.475299 -0.187589
C -1.029922 -0.140987 -0.498713
C -3.335913 -0.910409 -0.385918
C -0.039294 0.862135 -0.692556
C 1.303154 0.565312 -0.589236
C 1.718254 -0.780988 -0.287601
C 0.753859 -1.782335 -0.069983
C 3.124811 -1.109021 -0.179278
C 4.183892 -0.075260 -0.629495
C 3.634686 1.337199 -0.692540
C 2.312026 1.600663 -0.755573
O 1.090710 -3.032894 0.240388
O 3.525885 -2.238431 0.153645
O 4.603226 -0.465431 -1.954544
C 5.444759 -0.202549 0.227389
C 5.221178 0.164456 1.683006
C 6.448247 0.188809 2.573761
O 4.109051 0.418452 2.111634
O 4.630838 2.250192 -0.785405
C 4.275721 3.621186 -0.921876
C -4.806436 -0.685181 -0.453033
C -5.625128 -1.874739 -0.897282
C -5.322499 0.505851 -0.088031
C -6.768474 0.941551 -0.089677
C -7.407165 0.840020 1.323259

C -6.745710 1.684109 2.419400
C -6.897656 2.357532 -0.683648
H -1.368657 -3.473632 0.196977
H -2.754622 1.103263 -0.873238
H -0.355973 1.878197 -0.912956
H 1.951071 2.613702 -0.895580
H 2.089835 -3.049052 0.302776
H 3.850472 -0.321187 -2.552689
H 6.238938 0.413935 -0.204954
H 5.777712 -1.245327 0.171497
H 6.153784 0.398733 3.603857
H 6.977159 -0.770932 2.527726
H 7.151035 0.957938 2.229217
H 3.719352 3.792859 -1.851820
H 3.673168 3.957224 -0.069274
H 5.216283 4.173105 -0.950980
H -5.305949 -2.221024 -1.888251
H -5.465632 -2.714818 -0.214373
H -6.693419 -1.652698 -0.937652
H -4.627801 1.259797 0.282608
H -7.345538 0.268075 -0.734448
H -8.465789 1.123670 1.237599
H -7.392505 -0.214107 1.628668
H -6.773640 2.754935 2.187082
H -7.264455 1.544227 3.374492
H -5.698357 1.399337 2.569604
H -7.942967 2.689095 -0.677724
H -6.541278 2.380355 -1.719560
H -6.311348 3.090555 -0.117091
SCF Energy (PCM/mPW1PW91/6-31+G**)= -1361.46329259
Number of imaginary frequencies = 0

1b_c049
B3LYP/6-31G* Geometry
C -1.491709 -2.363626 -0.472750
C -2.436726 0.141418 0.058249
N -2.789430 -2.218026 -0.290105
C -0.558447 -1.302071 -0.420308
C -1.045325 0.008259 -0.135832
C -3.276144 -0.962051 -0.047202
C -0.140943 1.105445 -0.064124
C 1.214191 0.922398 -0.239056
C 1.722475 -0.403133 -0.497728
C 0.840501 -1.491479 -0.630591
C 3.134371 -0.599994 -0.683615
C 4.121532 0.507259 -0.278649
C 3.478356 1.880961 -0.318313
C 2.139454 2.046082 -0.229405
O 1.266563 -2.715681 -0.938088
O 3.638865 -1.647604 -1.135472
O 5.239932 0.454228 -1.142241
C 4.562830 0.261130 1.214301
C 5.218482 -1.099916 1.459772
C 6.712422 -1.197067 1.229756
O 4.552963 -2.042106 1.845462
O 4.407946 2.857909 -0.327600
C 3.961501 4.209251 -0.308647
C -4.752684 -0.863830 0.122538
C -5.426153 -2.105066 0.660007
C -5.396108 0.265420 -0.236691
C -6.866290 0.591862 -0.163842
C -7.109542 1.852146 0.701405
C -6.744917 1.677968 2.178613
C -7.431151 0.794554 -1.584549
H -1.134846 -3.372217 -0.669548
H -2.843130 1.114991 0.312740
H -0.536544 2.101068 0.118231
H 1.705056 3.036718 -0.153062
H 2.247665 -2.658811 -1.089510
H 5.227316 -0.454899 -1.502969
H 3.683905 0.329980 1.861610
H 5.254830 1.075536 1.454214
H 7.032177 -2.240132 1.274339
H 6.979856 -0.744191 0.269318
H 7.239145 -0.625564 2.006173
H 3.321470 4.422137 -1.173336
H 3.411652 4.430423 0.614949
H 4.862699 4.821628 -0.356681
H -5.006129 -2.384267 1.634175
H -6.503603 -1.974890 0.776794
H -5.241707 -2.952794 -0.006853
H -4.798989 1.067665 -0.674883
H -7.411872 -0.238130 0.301011
H -6.540872 2.691599 0.274646
H -8.169455 2.128836 0.616899
H -5.687372 1.419722 2.302460
H -6.936459 2.597515 2.743289
H -7.335747 0.876386 2.638831
H -8.500895 1.033137 -1.548342
H -7.300762 -0.105723 -2.194276
H -6.921397 1.620978 -2.096006
SCF Energy (PCM/mPW1PW91/6-31+G**)= -1361.46322389
Number of imaginary frequencies = 0

1b_c051
B3LYP/6-31G* Geometry
C -2.059451 -1.664002 0.038672
C -2.222594 1.058698 -0.002839
N -3.246289 -1.114836 0.159681
C -0.853576 -0.932346 -0.108546
C -0.934705 0.489855 -0.131945
C -3.347209 0.253722 0.141520
C 0.244281 1.268843 -0.292734
C 1.483758 0.672355 -0.400180
C 1.582550 -0.766263 -0.344684
C 0.419704 -1.556218 -0.237888
C 2.868408 -1.397917 -0.445216
C 4.150966 -0.554303 -0.345461
C 3.915114 0.901876 -0.681766
C 2.685802 1.460086 -0.622545
O 0.470394 -2.889141 -0.239704
O 3.034419 -2.631720 -0.561037
O 5.118972 -1.109798 -1.215063
C 4.608216 -0.666763 1.157528
C 5.841612 0.174343 1.506016
C 7.187604 -0.343924 1.049541
O 5.717394 1.205054 2.141552
O 5.077442 1.539345 -0.917234
C 5.060131 2.961067 -1.016113
C -4.710456 0.829213 0.282997
C -4.828087 2.335763 0.245341
C -5.759518 -0.005112 0.442463
C -7.217567 0.317175 0.629499
C -8.078110 -0.385172 -0.448006
C -7.807956 0.095083 -1.876950
C -7.665150 -0.115680 2.041950
H -2.017518 -2.751234 0.055749
H -2.312280 2.138962 -0.017715
H 0.157163 2.351015 -0.339441
H 2.554188 2.527807 -0.756942
H 1.422689 -3.145313 -0.377666
H 4.909866 -2.064566 -1.232198
H 4.801708 -1.731294 1.338258
H 3.794129 -0.337259 1.809822
H 7.970493 0.365782 1.324745
H 7.395251 -1.316286 1.515125
H 7.168852 -0.503944 -0.033377
H 4.441934 3.287696 -1.860804
H 4.688744 3.405324 -0.085654
H 6.095768 3.259180 -1.181683
H -4.439779 2.744280 -0.696637
H -5.862855 2.667136 0.343480
H -4.254828 2.802670 1.056903
H -5.525772 -1.068864 0.455741
H -7.389068 1.397079 0.538928
H -7.909274 -1.469734 -0.383984
H -9.136601 -0.224503 -0.200988
H -6.765752 -0.078865 -2.166476
H -8.448813 -0.428201 -2.595587
H -8.005440 1.169803 -1.977383
H -8.729612 0.100134 2.193931
H -7.094799 0.407526 2.816845

H -7.515931 -1.192667 2.187194
SCF Energy (PCM/mPW1PW91/6-31+G**)= -1361.46637599
Number of imaginary frequencies = 0

1b_c053
B3LYP/6-31G* Geometry
C -1.637797 -2.227556 -0.401919
C -2.512185 0.357471 -0.441271
N -2.935619 -2.015294 -0.506362
C -0.670970 -1.200995 -0.299707
C -1.120719 0.151064 -0.328564
C -3.385177 -0.723093 -0.502178
C -0.177649 1.214513 -0.246938
C 1.173606 0.961200 -0.152852
C 1.646382 -0.399847 -0.131169
C 0.728431 -1.465683 -0.182099
C 3.058940 -0.675824 -0.014202
C 4.092105 0.465598 -0.165887
C 3.466109 1.840907 -0.007993
C 2.133637 2.048278 -0.042344
O 1.117670 -2.738477 -0.126970
O 3.505404 -1.831095 0.145233
O 4.564134 0.412131 -1.508770
C 5.237314 0.245322 0.855992
C 6.080714 -0.995959 0.573875
C 6.406965 -1.899741 1.737529
O 6.523918 -1.207309 -0.547697
O 4.414495 2.801431 0.087916
C 3.999915 4.161953 0.091032
C -4.862119 -0.551181 -0.592959
C -5.592263 -1.627121 -1.362178
C -5.452771 0.493717 0.020990
C -6.908440 0.885596 0.051762
C -7.463191 0.857249 1.496607
C -7.480825 -0.535698 2.132316
C -7.084382 2.287514 -0.566715
H -1.309818 -3.264755 -0.401395
H -2.893728 1.371709 -0.502386
H -0.539447 2.239284 -0.254676
H 1.727018 3.052675 0.002970
H 2.110776 -2.727611 -0.015787
H 5.310587 -0.228635 -1.513356
H 4.829056 0.220119 1.871730
H 5.906361 1.110454 0.789289
H 7.107959 -2.675714 1.424360
H 6.831380 -1.323754 2.569432
H 5.480136 -2.361883 2.096869
H 3.454111 4.405449 -0.828425
H 3.366735 4.380912 0.960208
H 4.914723 4.753717 0.144823
H -5.390069 -2.606899 -0.919258
H -6.671351 -1.464168 -1.383143
H -5.231877 -1.676924 -2.397491
H -4.812802 1.165154 0.597109
H -7.498389 0.179476 -0.544980
H -6.866963 1.540903 2.118865
H -8.482227 1.267992 1.483184
H -6.474173 -0.965059 2.177520
H -7.879012 -0.498385 3.152652
H -8.109339 -1.225412 1.555374
H -8.138822 2.588264 -0.555623
H -6.733143 2.310696 -1.603982
H -6.517085 3.039896 -0.003818
SCF Energy (PCM/mPW1PW91/6-31+G**)= -1361.46702361
Number of imaginary frequencies = 0

1b_c054
B3LYP/6-31G* Geometry
C -1.934089 -1.499821 -0.044665
C -2.153469 1.211129 0.151520
N -3.135329 -0.985524 0.097364
C -0.741965 -0.736109 -0.106736
C -0.852567 0.678861 -0.002803
C -3.264184 0.376216 0.197570
C 0.317176 1.486014 -0.051819
C 1.565315 0.920696 -0.213456
C 1.692991 -0.507831 -0.337860
C 0.548445 -1.325307 -0.262211
C 2.995480 -1.115935 -0.512557
C 4.217154 -0.218142 -0.820587
C 3.994236 1.229674 -0.426916
C 2.762404 1.747933 -0.234964
O 0.623209 -2.653347 -0.339545
O 3.161499 -2.348371 -0.544003
O 4.422765 -0.279336 -2.248380
C 5.486594 -0.826395 -0.222169
C 5.480076 -0.859709 1.295192
C 6.751272 -1.338372 1.969614
O 4.498596 -0.524945 1.935159
O 5.157973 1.922245 -0.408925
C 5.107164 3.313948 -0.117644
C -4.643054 0.911642 0.354677
C -4.789941 2.412936 0.451050
C -5.679230 0.048079 0.399827
C -7.149535 0.341766 0.546310
C -7.903506 0.164674 -0.802481
C -7.822977 -1.228590 -1.437835
C -7.755525 -0.529494 1.664929
H -1.869885 -2.583463 -0.119155
H -2.264038 2.286666 0.232671
H 0.217776 2.563762 0.048226
H 2.625370 2.807660 -0.049683
H 1.595615 -2.877251 -0.417466
H 3.670731 0.169900 -2.670179
H 6.358120 -0.274819 -0.588196
H 5.570741 -1.851983 -0.599511
H 6.604670 -1.376843 3.050773
H 7.033831 -2.331184 1.598674
H 7.582701 -0.661435 1.735913
H 4.533851 3.855418 -0.880521
H 4.661172 3.491943 0.868396
H 6.141879 3.659590 -0.124020
H -4.398746 2.910588 -0.445942
H -5.832204 2.715967 0.562854
H -4.234558 2.816746 1.307708
H -5.419984 -1.005050 0.316490
H -7.293545 1.388899 0.838840
H -8.958009 0.426245 -0.634603
H -7.510400 0.903405 -1.513133
H -8.240785 -2.002211 -0.783988
H -8.389302 -1.253086 -2.375831
H -6.789044 -1.506832 -1.668499
H -8.830859 -0.338701 1.766948
H -7.279415 -0.314260 2.627777
H -7.620325 -1.598160 1.463556
SCF Energy (PCM/mPW1PW91/6-31+G**)= -1361.46511815
Number of imaginary frequencies = 0

1b_c056
B3LYP/6-31G* Geometry
C -1.567217 -2.146724 -0.880502
C -2.404121 0.424165 -0.506041
N -2.865228 -1.917969 -0.844697
C -0.579904 -1.146601 -0.720124
C -1.011061 0.200198 -0.533109
C -3.295200 -0.635780 -0.633238
C -0.052265 1.245579 -0.411527
C 1.299559 0.975782 -0.428071
C 1.748078 -0.387478 -0.578115
C 0.818769 -1.426641 -0.768722
C 3.156784 -0.674703 -0.600235
C 4.161692 0.389231 -0.128102
C 3.614982 1.795056 -0.291194
C 2.287556 2.042471 -0.358262
O 1.197382 -2.685645 -0.984573
O 3.640201 -1.767624 -0.957491
O 5.363078 0.232091 -0.856969
C 4.419615 0.183847 1.413333
C 4.962564 -1.199748 1.778668
C 6.465014 -1.385205 1.730570
O 4.203241 -2.089761 2.111323
O 4.600087 2.714360 -0.233723

C 4.240707 4.089006 -0.317013
C -4.771992 -0.449771 -0.578880
C -5.579241 -1.438982 -1.386614
C -5.296599 0.530836 0.183584
C -6.738534 0.930268 0.368488
C -7.156029 0.841033 1.855060
C -7.136367 -0.579248 2.427769
C -6.953898 2.361268 -0.167574
H -1.255424 -3.175585 -1.046465
H -2.773376 1.439788 -0.406183
H -0.401644 2.269776 -0.311607
H 1.911223 3.059454 -0.368222
H 2.190596 -2.692962 -1.030030
H 5.332238 -0.689619 -1.183248
H 3.480206 0.327932 1.954686
H 5.127706 0.968633 1.700332
H 6.717153 -2.441182 1.847143
H 6.867392 -0.985918 0.793595
H 6.930172 -0.812003 2.544168
H 3.712229 4.299177 -1.254665
H 3.609791 4.384326 0.530968
H 5.178802 4.644617 -0.289282
H -5.352541 -2.459813 -1.065135
H -6.653779 -1.269800 -1.296711
H -5.310679 -1.384467 -2.449182
H -4.603494 1.140006 0.767130
H -7.390155 0.257613 -0.202090
H -6.494545 1.489001 2.448920
H -8.165712 1.262388 1.956041
H -6.136723 -1.022856 2.364098
H -7.440739 -0.583142 3.480535
H -7.824700 -1.235411 1.880784
H -7.998461 2.670749 -0.041850
H -6.702672 2.428527 -1.231575
H -6.325527 3.082020 0.371286
SCF Energy (PCM/mPW1PW91/6-31+G**)= -1361.46323817
Number of imaginary frequencies = 0

1b_c062
B3LYP/6-31G* Geometry
C -1.534649 -2.290120 -0.622012
C -2.478094 0.173189 0.081835
N -2.833307 -2.152732 -0.440706
C -0.598700 -1.238568 -0.483501
C -1.086063 0.048425 -0.108841
C -3.320185 -0.917066 -0.110903
C -0.178079 1.135029 0.050878
C 1.176498 0.955901 -0.122212
C 1.685472 -0.348135 -0.470702
C 0.802033 -1.419081 -0.693601
C 3.100731 -0.534403 -0.655888
C 4.080894 0.536599 -0.152715
C 3.445584 1.917443 -0.122243
C 2.111137 2.069763 -0.029060
O 1.225628 -2.619494 -1.087032
O 3.605119 -1.542263 -1.190367
O 5.237315 0.503377 -0.973521
C 4.468542 0.204717 1.338307
C 5.138394 -1.159505 1.517455
C 6.648281 -1.207200 1.409106
O 4.467423 -2.146911 1.750895
O 4.273365 2.987901 0.068402
C 4.995820 3.473394 -1.077203
C -4.797723 -0.828756 0.053177
C -5.478486 -2.108206 0.480411
C -5.436238 0.327354 -0.219057
C -6.906935 0.648150 -0.135520
C -7.156293 1.857728 0.797055
C -6.796423 1.603124 2.263794
C -7.464922 0.928648 -1.545912
H -1.179301 -3.283059 -0.888464
H -2.882947 1.127652 0.402464
H -0.570080 2.117312 0.299980
H 1.710119 3.066955 0.128461
H 2.207811 -2.556661 -1.225639
H 5.214819 -0.381096 -1.393161
H 3.562821 0.216474 1.951003
H 5.129963 1.016681 1.658510
H 6.993746 -2.242871 1.424320
H 6.975638 -0.699619 0.495221
H 7.096015 -0.662993 2.251544
H 5.677393 2.716386 -1.468229
H 4.294597 3.785241 -1.861293
H 5.553887 4.342369 -0.722648
H -5.069809 -2.467672 1.432855
H -6.557077 -1.986164 0.595110
H -5.288026 -2.898764 -0.251769
H -4.834865 1.160732 -0.587471
H -7.453621 -0.207047 0.279612
H -6.588346 2.720923 0.419731
H -8.216576 2.136182 0.724039
H -5.737856 1.344343 2.377704
H -6.995369 2.488601 2.878069
H -7.384345 0.773748 2.676032
H -8.535010 1.164204 -1.501294
H -7.331092 0.063498 -2.203835
H -6.953720 1.782331 -2.008818
SCF Energy (PCM/mPW1PW91/6-31+G**)= -1361.45390337
Number of imaginary frequencies = 0

1b_c065
B3LYP/6-31G* Geometry
C -2.060640 -1.669058 -0.039652
C -2.242713 1.049481 0.084038
N -3.251648 -1.135350 0.113115
C -0.859895 -0.922468 -0.142392
C -0.952190 0.496231 -0.080297
C -3.362098 0.230171 0.179128
C 0.224297 1.287642 -0.191796
C 1.463703 0.701233 -0.339320
C 1.578365 -0.734725 -0.379931
C 0.419196 -1.533124 -0.304567
C 2.872828 -1.374849 -0.501311
C 4.149019 -0.516387 -0.382162
C 3.880722 0.956532 -0.612629
C 2.659517 1.513432 -0.514334
O 0.471981 -2.862681 -0.370083
O 3.012635 -2.603725 -0.610428
O 5.094850 -1.014795 -1.317827
C 4.647306 -0.748129 1.092034
C 5.897315 0.046579 1.469975
C 7.233376 -0.581019 1.127199
O 5.808317 1.117403 2.042193
O 5.048387 1.597094 -0.876853
C 5.054423 3.023510 -0.900499
C -4.729536 0.787432 0.356439
C -4.852496 2.291779 0.438405
C -5.776022 -0.060636 0.439612
C -7.237425 0.239679 0.637445
C -8.077832 -0.318866 -0.536469
C -7.784761 0.342430 -1.886401
C -7.713235 -0.364431 1.975458
H -2.011722 -2.754927 -0.087929
H -2.338691 2.128191 0.134622
H 0.135296 2.370503 -0.167163
H 2.523086 2.586008 -0.599376
H 1.434905 -3.103432 -0.498263
H 5.631850 -0.252427 -1.592568
H 4.829469 -1.825206 1.172627
H 3.849542 -0.460748 1.783883
H 8.037135 0.147084 1.255425
H 7.413889 -1.433421 1.796149
H 7.221346 -0.981893 0.108349
H 4.401906 3.403128 -1.695352
H 4.735696 3.416628 0.070515
H 6.086220 3.315303 -1.098249
H -4.470541 2.774880 -0.470364
H -5.887799 2.610823 0.567100
H -4.275940 2.694992 1.281192
H -5.537649 -1.120999 0.367055
H -7.407027 1.322943 0.679412
H -7.906790 -1.402310 -0.610698

H -9.140819 -0.192201 -0.288966
H -6.737213 0.207502 -2.177166
H -8.411744 -0.083605 -2.677839
H -7.983413 1.420997 -1.850033
H -8.778649 -0.160019 2.136158
H -7.153106 0.051148 2.819928
H -7.574396 -1.452505 1.985887
SCF Energy (PCM/mPW1PW91/6-31+G**)= -1361.46324192
Number of imaginary frequencies = 0

1b_c066
B3LYP/6-31G* Geometry
C -1.619841 -2.080260 -0.939603
C -2.459898 0.477273 -0.486210
N -2.917791 -1.852364 -0.904416
C -0.633431 -1.085923 -0.740255
C -1.066973 0.254072 -0.512188
C -3.350208 -0.578043 -0.653604
C -0.109243 1.295721 -0.350877
C 1.241820 1.024070 -0.366467
C 1.692275 -0.333073 -0.556765
C 0.765250 -1.365379 -0.789153
C 3.101219 -0.614123 -0.583364
C 4.103907 0.435493 -0.070309
C 3.561053 1.851910 -0.197035
C 2.233515 2.082140 -0.262297
O 1.145264 -2.616586 -1.045338
O 3.589630 -1.690419 -0.983790
O 5.316808 0.259383 -0.784263
C 4.333999 0.193659 1.470290
C 4.895595 -1.189894 1.807478
C 6.402952 -1.336640 1.834885
O 4.144018 -2.113349 2.055331
O 4.389070 2.909056 0.027887
C 5.594552 3.059265 -0.741833
C -4.827244 -0.395149 -0.602823
C -5.628648 -1.362243 -1.442623
C -5.357485 0.563251 0.183713
C -6.800833 0.955988 0.371021
C -7.220186 0.848543 1.855585
C -7.195463 -0.577774 2.413019
C -7.019568 2.392527 -0.148993
H -1.307035 -3.103197 -1.137196
H -2.829121 1.489312 -0.355417
H -0.457516 2.316603 -0.220390
H 1.886501 3.110438 -0.221463
H 2.138003 -2.622617 -1.087049
H 5.265507 -0.656789 -1.128063
H 3.378684 0.303150 1.991110
H 5.012451 0.986643 1.803028
H 6.675892 -2.386697 1.957567
H 6.837767 -0.925986 0.917128
H 6.815592 -0.755163 2.670340
H 6.371958 2.365603 -0.416655
H 5.398417 2.904621 -1.807378
H 5.911973 4.090072 -0.569897
H -5.409473 -2.391473 -1.143329
H -6.703521 -1.191145 -1.360831
H -5.348116 -1.283356 -2.500483
H -4.668549 1.157424 0.787164
H -7.449383 0.287704 -0.208067
H -6.562692 1.492902 2.457732
H -8.231887 1.264296 1.959262
H -6.193499 -1.016083 2.348210
H -7.503291 -0.594423 3.464640
H -7.878758 -1.231346 1.856684
H -8.065113 2.697596 -0.020510
H -6.767667 2.472538 -1.211908
H -6.393607 3.108782 0.398510
SCF Energy (PCM/mPW1PW91/6-31+G**)= -1361.45359658
Number of imaginary frequencies = 0

1b_c075
B3LYP/6-31G* Geometry
C -1.684888 -2.599724 0.112751
C -2.417147 0.029835 0.123393
N -2.963033 -2.314711 0.263232
C -0.669111 -1.629416 -0.054036
C -1.044559 -0.252986 -0.042586
C -3.343966 -0.999619 0.246339
C -0.054381 0.757839 -0.196924
C 1.278330 0.434715 -0.340054
C 1.675038 -0.953865 -0.325160
C 0.705792 -1.970358 -0.219507
C 3.061507 -1.302524 -0.466697
C 4.144309 -0.213816 -0.374986
C 3.602479 1.169405 -0.662061
C 2.286357 1.459059 -0.561938
O 1.031231 -3.262601 -0.257249
O 3.475503 -2.472680 -0.616872
O 5.176773 -0.535768 -1.287909
C 4.665208 -0.264022 1.110041
C 5.707728 0.808069 1.448363
C 7.116199 0.591299 0.940461
O 5.393310 1.775189 2.117218
O 4.601735 2.039568 -0.901417
C 4.289992 3.429313 -0.956006
C -4.804255 -0.749073 0.395467
C -5.564850 -1.799436 1.170882
C -5.361187 0.336155 -0.179227
C -6.796847 0.795589 -0.150262
C -6.877866 2.204969 0.489056
C -8.303155 2.725325 0.708575
C -7.384077 0.768914 -1.576256
H -1.413298 -3.653131 0.124649
H -2.739755 1.064647 0.175534
H -0.365407 1.798869 -0.214483
H 1.933955 2.479448 -0.662492
H 2.013000 -3.312130 -0.418638
H 5.172773 -1.512546 -1.324268
H 5.082433 -1.268369 1.253474
H 3.823223 -0.127239 1.794992
H 7.741281 1.446703 1.204792
H 7.539494 -0.320416 1.382189
H 7.094772 0.442875 -0.143985
H 3.598439 3.643934 -1.779408
H 3.856757 3.762921 -0.006245
H 5.238135 3.938798 -1.129828
H -5.167834 -1.895786 2.189193
H -6.630831 -1.574904 1.239536
H -5.438985 -2.778654 0.699392
H -4.711393 0.982588 -0.771816
H -7.392033 0.117841 0.473375
H -6.355933 2.175278 1.454403
H -6.319097 2.914366 -0.139045
H -8.887615 2.030422 1.324424
H -8.286983 3.691615 1.225402
H -8.841519 2.867711 -0.234688
H -8.436013 1.073450 -1.583631
H -7.321997 -0.237201 -2.004274
H -6.832658 1.448332 -2.239107
SCF Energy (PCM/mPW1PW91/6-31+G**)= -1361.46338689
Number of imaginary frequencies = 0

1b_c076
B3LYP/6-31G* Geometry
C 1.835245 -1.481248 0.207134
C 2.269916 1.154042 -0.346439
N 3.077915 -1.071647 0.083460
C 0.702478 -0.640065 0.072464
C 0.927226 0.734561 -0.217026
C 3.316241 0.249291 -0.195213
C -0.181208 1.615953 -0.369320
C -1.472924 1.160103 -0.227606
C -1.719994 -0.226465 0.073830
C -0.636139 -1.117434 0.206900
C -3.072810 -0.719163 0.203406
C -4.272833 0.257996 0.184554
C -3.879957 1.667432 -0.212183
C -2.607899 2.058251 -0.389497
O -0.813194 -2.413394 0.457427
O -3.329701 -1.930855 0.363233

O -4.778923 0.350023 1.513997
C -5.343534 -0.294585 -0.796795
C -6.018762 -1.574882 -0.312749
C -6.145521 -2.715132 -1.292621
O -6.482174 -1.645432 0.818645
O -4.930479 2.531550 -0.387263
C -5.235365 3.383793 0.728236
C 4.737775 0.663642 -0.328872
C 5.002562 2.119535 -0.637721
C 5.706332 -0.262995 -0.172473
C 7.200595 -0.102497 -0.255992
C 7.829989 -0.393782 1.130779
C 9.342032 -0.152390 1.208087
C 7.755881 -1.032658 -1.354782
H 1.685846 -2.536094 0.427946
H 2.465872 2.197276 -0.566845
H 0.004243 2.661249 -0.601524
H -2.405080 3.092053 -0.656965
H -1.801843 -2.562522 0.494016
H -5.431252 -0.379255 1.616766
H -4.900545 -0.433207 -1.788514
H -6.120292 0.474047 -0.885968
H -6.751263 -3.514945 -0.862501
H -6.589020 -2.371624 -2.235430
H -5.142482 -3.094931 -1.521308
H -5.504952 2.787204 1.603599
H -4.381767 4.029723 0.968477
H -6.080033 4.000088 0.412513
H 4.596074 2.774992 0.143530
H 6.069457 2.332312 -0.719627
H 4.533302 2.420329 -1.583518
H 5.368356 -1.274172 0.048568
H 7.461557 0.928681 -0.525449
H 7.327602 0.237292 1.875153
H 7.606223 -1.432729 1.411809
H 9.595492 0.874283 0.915314
H 9.705386 -0.304159 2.230818
H 9.902625 -0.832240 0.557610
H 8.838800 -0.916794 -1.469617
H 7.290600 -0.814284 -2.322014
H 7.551483 -2.084000 -1.115972
SCF Energy (PCM/mPW1PW91/6-31+G**)= -1361.45932441
Number of imaginary frequencies = 0

1b_c078
B3LYP/6-31G* Geometry
C 1.741910 2.536220 0.143952
C 2.390808 -0.013546 -0.583529
N 3.019868 2.226916 0.049792
C 0.684770 1.625844 -0.091440
C 1.017306 0.294799 -0.483297
C 3.355536 0.943203 -0.288359
C -0.013842 -0.642113 -0.774165
C -1.343632 -0.300522 -0.648224
C -1.693448 1.031749 -0.214000
C -0.689322 1.990852 0.021711
C -3.075691 1.394981 -0.067828
C -4.165499 0.310132 -0.108747
C -3.710511 -0.940479 -0.828901
C -2.402200 -1.235396 -0.995144
O -0.978749 3.246013 0.365920
O -3.472848 2.560317 0.149046
O -5.310650 0.851571 -0.738827
C -4.461252 -0.041722 1.397273
C -5.472744 -1.177097 1.592439
C -6.934016 -0.851533 1.374452
O -5.090520 -2.283120 1.927360
O -4.758644 -1.721818 -1.150867
C -4.498072 -3.038664 -1.629476
C 4.813966 0.649693 -0.344430
C 5.696910 1.819405 -0.711891
C 5.259802 -0.584532 -0.034820
C 6.674231 -1.106604 -0.020473
C 7.122270 -1.367537 1.440240
C 8.588140 -1.789131 1.593711
C 6.768597 -2.373564 -0.894418
H 1.505287 3.561879 0.418882
H 2.687570 -1.003646 -0.914212
H 0.261355 -1.639773 -1.105375
H -2.097153 -2.188171 -1.413042
H -1.971053 3.330711 0.356153
H -5.279946 1.804035 -0.521160
H -4.823727 0.883766 1.861375
H -3.532087 -0.345757 1.888120
H -7.537574 -1.751931 1.506036
H -7.260526 -0.084570 2.088944
H -7.069432 -0.434171 0.371325
H -3.931121 -3.009390 -2.567595
H -3.949888 -3.618753 -0.878405
H -5.475441 -3.489146 -1.804544
H 5.555931 2.635160 0.003622
H 6.754693 1.550758 -0.736196
H 5.421662 2.221819 -1.694845
H 4.520266 -1.321798 0.282687
H 7.351987 -0.355701 -0.444499
H 6.949938 -0.451187 2.018985
H 6.469128 -2.134053 1.882259
H 9.262131 -1.050657 1.141630
H 8.856250 -1.876430 2.652726
H 8.792269 -2.757598 1.124253
H 7.794067 -2.754927 -0.939902
H 6.442533 -2.165989 -1.919449
H 6.130983 -3.173214 -0.495169
SCF Energy (PCM/mPW1PW91/6-31+G**)= -1361.46351488
Number of imaginary frequencies = 0

1b_c084
B3LYP/6-31G* Geometry
C -1.626181 -2.279017 -0.542001
C -2.548634 0.288921 -0.471206
N -2.927876 -2.085601 -0.634942
C -0.678192 -1.240168 -0.396244
C -1.154964 0.102081 -0.366892
C -3.403047 -0.804292 -0.575768
C -0.230369 1.179323 -0.240667
C 1.122659 0.944245 -0.150877
C 1.625143 -0.406529 -0.182146
C 0.726731 -1.485147 -0.290688
C 3.043248 -0.664861 -0.065605
C 4.059520 0.501732 -0.070055
C 3.397405 1.861325 0.035270
C 2.067880 2.042857 -0.009756
O 1.138175 -2.751170 -0.298419
O 3.506459 -1.821803 0.012278
O 4.727014 0.490973 -1.329515
C 5.054371 0.296478 1.105352
C 5.983831 -0.900156 0.921351
C 6.148810 -1.846488 2.084773
O 6.607752 -1.049372 -0.121787
O 4.270240 2.908865 0.177472
C 4.536917 3.669737 -1.010691
C -4.882260 -0.658075 -0.658762
C -5.598929 -1.743045 -1.428180
C -5.492702 0.369419 -0.033898
C -6.963481 0.708291 -0.002910
C -7.638462 0.252962 1.320439
C -7.070934 0.862761 2.607999
C -7.168064 2.212390 -0.270212
H -1.279645 -3.309108 -0.587123
H -2.946533 1.298423 -0.490389
H -0.608832 2.197514 -0.210914
H 1.668626 3.051981 0.051060
H 2.133019 -2.726759 -0.193067
H 5.497229 -0.113431 -1.233539
H 4.503502 0.226390 2.049021
H 5.679804 1.195654 1.151936
H 6.922435 -2.583803 1.862335
H 6.403342 -1.297568 2.999973
H 5.194146 -2.355831 2.262939
H 4.974821 3.030349 -1.782080
H 3.619801 4.136064 -1.391208
H 5.244368 4.448048 -0.716339

H -5.402645 -2.718251 -0.972194
H -6.678353 -1.583250 -1.463812
H -5.224110 -1.805134 -2.457389
H -4.865552 1.045283 0.547788
H -7.473372 0.165900 -0.807682
H -8.709855 0.488857 1.250836
H -7.565290 -0.840472 1.382183
H -7.151545 1.955746 2.615686
H -7.618595 0.489283 3.480584
H -6.015735 0.602033 2.746876
H -8.232792 2.472786 -0.235791
H -6.783356 2.489043 -1.258071
H -6.648643 2.831052 0.470985
SCF Energy (PCM/mPW1PW91/6-31+G**)= -1361.45631848
Number of imaginary frequencies = 0

1b_c089
B3LYP/6-31G* Geometry
C -1.774901 -2.620531 0.093619
C -2.499017 0.010990 0.126706
N -3.051728 -2.332759 0.249880
C -0.756481 -1.652063 -0.067941
C -1.127595 -0.274633 -0.044362
C -3.428703 -1.016445 0.243877
C -0.134687 0.734418 -0.191999
C 1.196791 0.408431 -0.340257
C 1.589136 -0.981435 -0.338519
C 0.616977 -1.995835 -0.239601
C 2.974233 -1.333135 -0.486150
C 4.060704 -0.248778 -0.386658
C 3.522720 1.138883 -0.659103
C 2.207647 1.431638 -0.554197
O 0.938262 -3.288679 -0.289428
O 3.384119 -2.503175 -0.647914
O 5.089871 -0.565126 -1.305272
C 4.584997 -0.315047 1.096511
C 5.632052 0.750111 1.442749
C 7.038499 0.533591 0.929157
O 5.322650 1.711535 2.122068
O 4.524282 2.008272 -0.891337
C 4.217028 3.399578 -0.930312
C -4.888264 -0.762929 0.396788
C -5.648161 -1.811621 1.174917
C -5.444241 0.325297 -0.174321
C -6.884974 0.773314 -0.142031
C -7.097559 1.966785 0.830894
C -6.324005 3.250095 0.504528
C -7.379679 1.090595 -1.566651
H -1.506582 -3.674839 0.096378
H -2.817954 1.046482 0.187553
H -0.442469 1.776527 -0.199935
H 1.858285 2.454037 -0.644409
H 1.919499 -3.339856 -0.453604
H 5.083051 -1.541494 -1.350757
H 4.999061 -1.322172 1.229203
H 3.745175 -0.182033 1.784855
H 7.667003 1.384499 1.199804
H 7.460014 -0.383487 1.361397
H 7.013885 0.395248 -0.156547
H 3.525220 3.625489 -1.750464
H 3.786038 3.724111 0.023600
H 5.166646 3.907806 -1.099666
H -5.249916 -1.902511 2.193295
H -6.713644 -1.585551 1.246761
H -5.522231 -2.793075 0.708125
H -4.789981 0.971155 -0.760158
H -7.507271 -0.042890 0.242286
H -8.172925 2.193111 0.854357
H -6.828865 1.635144 1.842161
H -6.586474 3.648174 -0.482094
H -6.549554 4.029187 1.241428
H -5.240274 3.087016 0.523028
H -8.418730 1.441149 -1.549987
H -7.331817 0.199923 -2.202498
H -6.771664 1.867902 -2.044079
SCF Energy (PCM/mPW1PW91/6-31+G**)= -1361.46196071
Number of imaginary frequencies = 0

1b_c093
B3LYP/6-31G* Geometry
C -1.688824 -2.602662 0.055275
C -2.434495 0.022312 0.084431
N -2.970159 -2.324462 0.197970
C -0.676321 -1.626834 -0.094783
C -1.060221 -0.254565 -0.074522
C -3.357765 -1.012038 0.192470
C -0.070639 0.759900 -0.207798
C 1.262438 0.438493 -0.343761
C 1.671868 -0.944431 -0.348028
C 0.704474 -1.962667 -0.246113
C 3.071251 -1.306080 -0.459265
C 4.142785 -0.199971 -0.369045
C 3.575505 1.179490 -0.633412
C 2.265541 1.474526 -0.543839
O 1.030632 -3.253332 -0.276370
O 3.461488 -2.482153 -0.537106
O 5.169175 -0.515350 -1.299644
C 4.681695 -0.288605 1.106145
C 5.740450 0.756863 1.457357
C 7.176953 0.412432 1.119118
O 5.432123 1.799117 2.005605
O 4.585398 2.040940 -0.919193
C 4.295654 3.436596 -0.980857
C -4.819692 -0.767753 0.341842
C -5.579005 -1.833945 1.096569
C -5.377593 0.329860 -0.207554
C -6.809992 0.797702 -0.153401
C -6.883303 2.137019 0.624588
C -8.303719 2.661259 0.865345
C -7.379161 0.919570 -1.580840
H -1.412619 -3.654805 0.059820
H -2.760587 1.055592 0.145114
H -0.381880 1.801129 -0.211915
H 1.910911 2.493452 -0.655111
H 2.022856 -3.293309 -0.403514
H 5.535131 0.334525 -1.597766
H 5.083917 -1.302162 1.209120
H 3.843843 -0.157013 1.797793
H 7.811881 1.294820 1.223497
H 7.532801 -0.366626 1.806982
H 7.245744 -0.007804 0.110353
H 3.582152 3.651689 -1.784836
H 3.897650 3.780699 -0.020542
H 5.245490 3.930572 -1.187282
H -5.189603 -1.940769 2.116852
H -6.647171 -1.618116 1.159787
H -5.441401 -2.805854 0.613496
H -4.728249 0.991121 -0.784086
H -7.419799 0.068318 0.393103
H -6.384565 1.998208 1.592518
H -6.297332 2.895520 0.084915
H -8.918165 1.915861 1.385568
H -8.282570 3.563979 1.486509
H -8.813362 2.919564 -0.069076
H -8.430013 1.227525 -1.570431
H -7.314399 -0.038112 -2.108194
H -6.817384 1.660627 -2.164341
SCF Energy (PCM/mPW1PW91/6-31+G**)= -1361.46037558
Number of imaginary frequencies = 0

1b_c096
B3LYP/6-31G* Geometry
C 1.810157 2.516429 0.098093
C 2.515399 -0.120389 0.051297
N 3.088749 2.215210 0.210099
C 0.780281 1.559026 -0.055646
C 1.143573 0.180040 -0.074229
C 3.456868 0.897949 0.164813
C 0.137637 -0.819562 -0.218778
C -1.191872 -0.477452 -0.318088
C -1.577490 0.913081 -0.271233
C -0.595907 1.917459 -0.178925

C -2.966802 1.276739 -0.368186
C -4.055348 0.195753 -0.255687
C -3.532867 -1.185230 -0.586835
C -2.223330 -1.483984 -0.533660
O -0.906730 3.213528 -0.190504
O -3.369165 2.453165 -0.496791
O -5.115188 0.542133 -1.135884
C -4.532223 0.239527 1.240953
C -5.571951 -0.818230 1.636578
C -6.980040 -0.653681 1.104275
O -5.263373 -1.715891 2.395607
O -4.481093 -2.154068 -0.767117
C -4.915053 -2.363162 -2.120648
C 4.918013 0.633029 0.273147
C 5.712326 1.680780 1.017706
C 5.448428 -0.460716 -0.311186
C 6.881884 -0.926699 -0.341105
C 7.010005 -2.356784 0.237269
C 6.653856 -2.466463 1.722792
C 7.421099 -0.877732 -1.785423
H 1.550784 3.572306 0.132974
H 2.826661 -1.159485 0.079819
H 0.435330 -1.863570 -0.262334
H -1.914527 -2.517090 -0.665082
H -1.891891 3.276289 -0.324546
H -5.110887 1.519353 -1.154757
H -4.940700 1.246124 1.401196
H -3.670492 0.100898 1.900434
H -7.581164 -1.520877 1.384862
H -7.432041 0.251977 1.530985
H -6.963521 -0.521528 0.018957
H -5.353892 -1.452696 -2.537204
H -4.074938 -2.691841 -2.745224
H -5.664979 -3.155170 -2.072432
H 5.327770 1.809722 2.036838
H 6.772446 1.429124 1.081126
H 5.608515 2.652990 0.526404
H 4.775511 -1.105925 -0.879356
H 7.503666 -0.265048 0.273756
H 6.373105 -3.036092 -0.348358
H 8.042287 -2.698769 0.082110
H 5.619805 -2.156872 1.910155
H 6.768063 -3.495902 2.080568
H 7.304667 -1.828094 2.332982
H 8.463745 -1.215092 -1.822939
H 7.375011 0.138287 -2.191548
H 6.834958 -1.528812 -2.446330
SCF Energy (PCM/mPW1PW91/6-31+G**)= -1361.45505374
Number of imaginary frequencies = 0

1b_c101
B3LYP/6-31G* Geometry
C 1.463268 -2.454930 -0.035117
C 2.484424 0.062335 -0.295528
N 2.773685 -2.324168 -0.112864
C 0.554295 -1.372368 -0.061228
C 1.081211 -0.056161 -0.208346
C 3.296300 -1.064004 -0.216829
C 0.197559 1.058052 -0.279483
C -1.166341 0.891603 -0.176312
C -1.714569 -0.430006 -0.004578
C -0.861072 -1.548743 0.026067
C -3.143280 -0.615591 0.090987
C -4.075822 0.604515 0.276926
C -3.403434 1.911861 -0.106600
C -2.069485 2.028105 -0.270031
O -1.326878 -2.791715 0.139607
O -3.673639 -1.746198 0.097896
O -4.338921 0.705381 1.673628
C -5.375019 0.374545 -0.537350
C -6.229922 -0.777083 -0.013550
C -6.780910 -1.754346 -1.022794
O -6.504569 -0.859208 1.176836
O -4.300602 2.922572 -0.175668
C -3.815230 4.243449 -0.382383
C 4.782595 -0.980824 -0.264932
C 5.478923 -2.174965 -0.874654
C 5.409318 0.092952 0.256226
C 6.885506 0.395035 0.317070
C 7.165852 1.753336 -0.372823
C 8.650444 2.118510 -0.486801
C 7.361585 0.375444 1.784460
H 1.076812 -3.468027 0.052258
H 2.924886 1.043034 -0.444465
H 0.615502 2.052051 -0.415194
H -1.616626 2.992824 -0.471407
H -2.323524 -2.721532 0.155879
H -5.109061 0.119624 1.851634
H -5.130670 0.232474 -1.595221
H -5.977595 1.285870 -0.455972
H -7.462926 -2.453085 -0.534673
H -7.300904 -1.225048 -1.831163
H -5.946992 -2.304823 -1.473467
H -3.134712 4.540063 0.424770
H -3.297218 4.328321 -1.346099
H -4.694257 4.889401 -0.379747
H 5.158732 -2.324299 -1.913642
H 6.565847 -2.074014 -0.863234
H 5.202007 -3.085413 -0.334737
H 4.789780 0.855729 0.731396
H 7.445358 -0.372950 -0.230392
H 6.725544 1.724992 -1.378190
H 6.632878 2.546443 0.172155
H 9.211956 1.335097 -1.010958
H 8.778191 3.050105 -1.049775
H 9.115674 2.262295 0.494155
H 8.439108 0.555773 1.862576
H 7.148429 -0.593449 2.248110
H 6.847690 1.146746 2.372685
SCF Energy (PCM/mPW1PW91/6-31+G**)= -1361.46624187
Number of imaginary frequencies = 0

1b_c109
B3LYP/6-31G* Geometry
C 1.833449 2.529887 0.211708
C 2.468003 0.008504 -0.619560
N 3.109666 2.218528 0.101527
C 0.771235 1.634724 -0.056886
C 1.096398 0.318619 -0.501630
C 3.438440 0.948242 -0.289936
C 0.060146 -0.601922 -0.824974
C -1.267661 -0.259552 -0.681881
C -1.610126 1.057068 -0.196885
C -0.600913 2.001691 0.073075
C -2.990362 1.421366 -0.034899
C -4.085627 0.344177 -0.113620
C -3.638015 -0.881565 -0.879838
C -2.331470 -1.176088 -1.060265
O -0.883541 3.244298 0.465115
O -3.381249 2.579904 0.225407
O -5.228958 0.913829 -0.721871
C -4.380932 -0.060558 1.379073
C -5.399279 -1.195872 1.534936
C -6.858703 -0.853593 1.330563
O -5.023597 -2.315480 1.829685
O -4.690458 -1.645813 -1.227735
C -4.437028 -2.946220 -1.753076
C 4.895233 0.651840 -0.366560
C 5.782511 1.834895 -0.675891
C 5.338609 -0.598772 -0.124932
C 6.755278 -1.118034 -0.163651
C 7.357406 -1.276605 1.259820
C 6.612972 -2.230137 2.202379
C 6.816512 -2.431885 -0.967981
H 1.602593 3.544859 0.528073
H 2.758219 -0.968725 -0.991701
H 0.329954 -1.587373 -1.194914
H -2.031919 -2.114125 -1.513992
H -1.875403 3.334363 0.459647
H -5.193455 1.857561 -0.469699
H -4.736883 0.849755 1.877019
H -3.452830 -0.387774 1.856879

H -7.467924 -1.754103 1.431849
H -7.179331 -0.109560 2.071459
H -6.992435 -0.401037 0.342589
H -3.871396 -2.886506 -2.690527
H -3.890536 -3.555001 -1.023813
H -5.416874 -3.385494 -1.942218
H 5.655037 2.608823 0.087147
H 6.838179 1.561075 -0.725320
H 5.500121 2.295883 -1.630602
H 4.596487 -1.346549 0.155460
H 7.390230 -0.392060 -0.684744
H 8.396793 -1.616879 1.149169
H 7.404051 -0.282927 1.723532
H 6.562328 -3.248779 1.801192
H 7.124086 -2.285785 3.170076
H 5.588227 -1.891674 2.391962
H 7.839669 -2.825873 -0.992546
H 6.490293 -2.272261 -2.001699
H 6.171041 -3.204706 -0.534319
SCF Energy (PCM/mPW1PW91/6-31+G**)= -1361.46216641
Number of imaginary frequencies = 0

1b_c116
B3LYP/6-31G* Geometry
C 1.921064 -1.432659 0.070948
C 2.317667 1.226019 -0.392582
N 3.157114 -1.003868 -0.053646
C 0.776953 -0.600801 -0.018258
C 0.981773 0.786084 -0.259933
C 3.376193 0.329020 -0.288223
C -0.138672 1.659360 -0.362697
C -1.422860 1.183399 -0.218286
C -1.649640 -0.216410 0.033841
C -0.554016 -1.098832 0.117028
C -2.994780 -0.729667 0.164800
C -4.205363 0.233457 0.202655
C -3.834718 1.661837 -0.144525
C -2.570141 2.073905 -0.327344
O -0.712435 -2.405446 0.320604
O -3.235669 -1.949417 0.281524
O -4.690875 0.267094 1.542637
C -5.285780 -0.292098 -0.782832
C -5.939259 -1.598247 -0.339748
C -6.070559 -2.699211 -1.363021
O -6.382822 -1.719549 0.795252
O -4.897585 2.519469 -0.268747
C -5.194574 3.323207 0.884431
C 4.790889 0.764641 -0.423833
C 5.036930 2.231613 -0.692592
C 5.773703 -0.152130 -0.298526
C 7.264833 0.048668 -0.388532
C 7.915656 0.135565 1.021225
C 7.716505 -1.085674 1.926997
C 7.899568 -1.059513 -1.251144
H 1.786752 -2.496452 0.255546
H 2.498694 2.279045 -0.576307
H 0.031470 2.714746 -0.558030
H -2.383069 3.119499 -0.557865
H -1.698550 -2.567848 0.365675
H -5.333029 -0.473502 1.626750
H -4.857373 -0.386283 -1.786110
H -6.072062 0.470586 -0.828912
H -6.658337 -3.523626 -0.954999
H -6.536219 -2.323069 -2.282418
H -5.067616 -3.056376 -1.626048
H -5.442338 2.689125 1.739673
H -4.345179 3.970160 1.136471
H -6.051786 3.940500 0.606793
H 4.642974 2.858057 0.118356
H 6.100407 2.455821 -0.788728
H 4.544579 2.559068 -1.617554
H 5.448753 -1.170985 -0.099639
H 7.480187 1.004815 -0.881407
H 8.991529 0.314708 0.884199
H 7.515650 1.023462 1.527974
H 8.138567 -1.996167 1.487080
H 8.211905 -0.929465 2.891979
H 6.655146 -1.270121 2.125916
H 8.987032 -0.930738 -1.311367
H 7.498672 -1.038586 -2.270524
H 7.700293 -2.055702 -0.840248
SCF Energy (PCM/mPW1PW91/6-31+G**)= -1361.45794920
Number of imaginary frequencies = 0

1b_c117
B3LYP/6-31G* Geometry
C 1.532435 -2.497982 -0.006261
C 2.565013 0.014077 -0.269584
N 2.843477 -2.373054 -0.082559
C 0.628124 -1.411609 -0.036620
C 1.161156 -0.097998 -0.184079
C 3.372058 -1.115613 -0.189181
C 0.282772 1.020119 -0.258386
C -1.082191 0.859774 -0.159708
C -1.636864 -0.459337 0.010449
C -0.788336 -1.581749 0.045780
C -3.066835 -0.638839 0.099037
C -3.995760 0.585125 0.277668
C -3.315030 1.889990 -0.099494
C -1.979796 2.000348 -0.256478
O -1.259889 -2.822572 0.158662
O -3.601793 -1.767263 0.104689
O -4.270908 0.686286 1.671885
C -5.288588 0.361168 -0.548608
C -6.153689 -0.786547 -0.033067
C -6.699161 -1.761369 -1.047689
O -6.440033 -0.867472 1.154611
O -4.207140 2.904946 -0.171027
C -3.714645 4.224272 -0.371049
C 4.859000 -1.039342 -0.240495
C 5.545892 -2.236835 -0.854196
C 5.491936 0.034317 0.275047
C 6.971842 0.323784 0.323628
C 7.371275 1.455774 -0.664054
C 6.720266 2.823195 -0.421697
C 7.411317 0.635072 1.768139
H 1.141485 -3.509209 0.082892
H 3.009182 0.992843 -0.420255
H 0.705556 2.012182 -0.393290
H -1.521650 2.963267 -0.454493
H -2.256317 -2.748132 0.170650
H -5.045290 0.104005 1.842606
H -5.035093 0.218219 -1.604219
H -5.887680 1.275267 -0.472569
H -7.390603 -2.455797 -0.566727
H -7.206770 -1.229857 -1.862433
H -5.863319 -2.317098 -1.488326
H -3.036451 4.514891 0.440221
H -3.191911 4.310071 -1.332130
H -4.590668 4.874296 -0.370170
H 5.231332 -2.371829 -1.896945
H 6.633796 -2.148405 -0.837177
H 5.253787 -3.148820 -0.325157
H 4.872779 0.798445 0.745466
H 7.524563 -0.565871 0.001209
H 8.464019 1.567362 -0.622091
H 7.133194 1.121851 -1.682263
H 6.959536 3.221034 0.570808
H 7.074565 3.551322 -1.160103
H 5.628845 2.773782 -0.508362
H 8.481827 0.870653 1.807895
H 7.227327 -0.222755 2.423872
H 6.864564 1.489474 2.183835
SCF Energy (PCM/mPW1PW91/6-31+G**)= -1361.46479316
Number of imaginary frequencies = 0

1b_c124
B3LYP/6-31G* Geometry
C -1.992357 -1.661113 -0.075167
C -2.171928 1.059144 0.004392
N -3.184420 -1.123639 0.054300
C -0.789292 -0.917758 -0.174665

C -0.880323 0.501801 -0.135011
C -3.293866 0.242907 0.097231
C 0.298610 1.290004 -0.243917
C 1.538758 0.699965 -0.368468
C 1.651896 -0.736554 -0.386931
C 0.490732 -1.532315 -0.312831
C 2.946587 -1.379950 -0.485229
C 4.222961 -0.521519 -0.367140
C 3.958940 0.948557 -0.619246
C 2.737572 1.508205 -0.541275
O 0.542479 -2.862757 -0.358039
O 3.085906 -2.610566 -0.574144
O 5.175105 -1.033420 -1.289252
C 4.709680 -0.734523 1.113655
C 5.957782 0.063211 1.491444
C 7.295678 -0.568749 1.163995
O 5.865950 1.139721 2.052465
O 5.130236 1.584311 -0.879486
C 5.138880 3.010352 -0.918603
C -4.662598 0.804700 0.245968
C -4.783776 2.310470 0.300921
C -5.712070 -0.040081 0.322838
C -7.176847 0.272209 0.473189
C -7.939622 -0.205818 -0.789052
C -9.428376 0.160081 -0.811589
C -7.713039 -0.385797 1.761638
H -1.944411 -2.747671 -0.105860
H -2.266807 2.138647 0.036792
H 0.210883 2.373224 -0.236082
H 2.603506 2.579725 -0.642110
H 1.506491 -3.106512 -0.471652
H 5.716121 -0.275624 -1.568703
H 4.889706 -1.810715 1.209584
H 3.906931 -0.437261 1.795509
H 8.098852 0.160691 1.288263
H 7.471979 -1.412906 1.844434
H 7.289737 -0.982075 0.150107
H 4.495724 3.382376 -1.724606
H 4.810199 3.414399 0.044577
H 6.173252 3.298426 -1.108193
H -4.392021 2.777004 -0.612430
H -5.819665 2.633946 0.413533
H -4.214955 2.727903 1.141995
H -5.475509 -1.101528 0.267531
H -7.331374 1.355093 0.560083
H -7.450518 0.228279 -1.670634
H -7.824841 -1.295070 -0.882689
H -9.572657 1.242485 -0.703433
H -9.885853 -0.141454 -1.760728
H -9.986965 -0.332218 -0.008326
H -8.769709 -0.148130 1.924157
H -7.152825 -0.040831 2.637321
H -7.615627 -1.477616 1.712221
SCF Energy (PCM/mPW1PW91/6-31+G**)= -1361.46234147
Number of imaginary frequencies = 0

1b_c126
B3LYP/6-31G* Geometry
C 2.056970 1.643599 -0.066783
C 2.253451 -1.071667 0.112821
N 3.251276 1.118753 0.085626
C 0.858704 0.888591 -0.140337
C 0.959290 -0.529103 -0.049627
C 3.370173 -0.244544 0.179914
C -0.213253 -1.332815 -0.137629
C -1.457231 -0.757978 -0.276213
C -1.577371 0.678577 -0.332769
C -0.424075 1.487039 -0.304259
C -2.875257 1.285437 -0.465542
C -4.143422 0.435641 -0.276820
C -3.889891 -1.037722 -0.512056
C -2.657967 -1.569443 -0.430362
O -0.489070 2.814703 -0.412751
O -3.054886 2.503261 -0.682717
O -5.135733 0.913782 -1.173168
C -4.578145 0.667512 1.215846
C -5.785513 -0.153740 1.688227
C -7.150988 0.238030 1.163263
O -5.630695 -1.047722 2.496948
O -5.001660 -1.824758 -0.631333
C -5.487674 -2.026315 -1.968291
C 4.741142 -0.790820 0.356039
C 4.873412 -2.292594 0.465316
C 5.783134 0.065368 0.415249
C 7.247537 -0.222509 0.607153
C 8.076256 0.329635 -0.578048
C 7.778867 -0.347214 -1.919314
C 7.726690 0.399743 1.935773
H 2.002568 2.727996 -0.137866
H 2.355869 -2.148420 0.184790
H -0.115276 -2.414323 -0.102018
H -2.546557 -2.648336 -0.489248
H -1.445606 3.049318 -0.559417
H -4.945188 1.868198 -1.264820
H -4.793384 1.740322 1.307072
H -3.744254 0.418182 1.878472
H -7.895459 -0.484213 1.504223
H -7.417480 1.235453 1.538381
H -7.136389 0.300883 0.071567
H -5.755055 -1.075538 -2.436626
H -4.732957 -2.541880 -2.575354
H -6.371310 -2.659912 -1.869791
H 4.485582 -2.795257 -0.430215
H 5.911752 -2.603058 0.589798
H 4.307967 -2.682904 1.321553
H 5.537996 1.122836 0.325525
H 7.425014 -1.303946 0.659732
H 7.897815 1.411196 -0.661954
H 9.141477 0.212273 -0.336053
H 6.728900 -0.221337 -2.205546
H 8.398681 0.074333 -2.718701
H 7.984010 -1.424148 -1.873211
H 8.794473 0.204665 2.091711
H 7.175085 -0.010850 2.788212
H 7.580119 1.486831 1.935267
SCF Energy (PCM/mPW1PW91/6-31+G**)= -1361.45700756
Number of imaginary frequencies = 0

1b_c137
B3LYP/6-31G* Geometry
C -2.088057 -1.554734 0.049788
C -2.205551 1.169635 -0.003249
N -3.266456 -0.984315 0.162223
C -0.869482 -0.844924 -0.095619
C -0.928049 0.576577 -0.124701
C -3.344616 0.384955 0.139016
C 0.267412 1.331271 -0.279968
C 1.492842 0.707485 -0.384587
C 1.573180 -0.730483 -0.335719
C 0.394969 -1.494797 -0.214569
C 2.852025 -1.407519 -0.414572
C 4.148674 -0.574399 -0.347588
C 3.916032 0.888637 -0.662785
C 2.708286 1.479098 -0.602294
O 0.416067 -2.826721 -0.196956
O 2.962393 -2.643852 -0.446837
O 5.078969 -1.148711 -1.255313
C 4.646290 -0.732512 1.136545
C 5.915679 0.052520 1.467221
C 7.235795 -0.621970 1.152558
O 5.853699 1.153011 1.984089
O 5.099576 1.485259 -0.958090
C 5.141815 2.908173 -1.051935
C -4.699066 0.984291 0.270568
C -4.788108 2.492959 0.237987
C -5.765712 0.168568 0.407664
C -7.220080 0.532348 0.554216
C -8.011379 0.260571 -0.756618
C -8.006860 -1.189482 -1.255767
C -7.832730 -0.205005 1.761938
H -2.064869 -2.642306 0.071589
H -2.275721 2.251328 -0.023571

H 0.204407 2.415381 -0.322931
H 2.597594 2.547768 -0.750787
H 1.373160 -3.097756 -0.307225
H 5.635592 -0.417117 -1.571471
H 4.803424 -1.807241 1.278204
H 3.857586 -0.387638 1.812378
H 8.055566 0.094823 1.235073
H 7.402841 -1.438582 1.868054
H 7.208809 -1.079709 0.158315
H 4.503126 3.264599 -1.868474
H 4.828168 3.355775 -0.103161
H 6.181627 3.163980 -1.257301
H -4.400030 2.895660 -0.706731
H -5.815580 2.844501 0.343452
H -4.198954 2.947735 1.045015
H -5.548631 -0.897249 0.411749
H -7.317400 1.607104 0.749245
H -9.049266 0.584769 -0.594979
H -7.603302 0.909240 -1.542714
H -8.442915 -1.877134 -0.522376
H -8.595638 -1.277175 -2.175913
H -6.992057 -1.536243 -1.478591
H -8.897173 0.038230 1.866298
H -7.326732 0.080402 2.690689
H -7.743919 -1.292417 1.660285
SCF Energy (PCM/mPW1PW91/6-31+G**)= -1361.46092419
Number of imaginary frequencies = 0

1b_c169
B3LYP/6-31G* Geometry
C 1.906240 -1.438981 -0.126982
C 2.294644 1.259275 -0.200439
N 3.141543 -0.993921 -0.183804
C 0.759542 -0.606726 -0.099781
C 0.958638 0.801882 -0.138151
C 3.355565 0.360223 -0.222361
C -0.163005 1.676968 -0.122246
C -1.448754 1.184002 -0.050653
C -1.666915 -0.238397 0.002431
C -0.569830 -1.121290 -0.044141
C -3.009549 -0.765161 0.058059
C -4.209100 0.178668 0.311911
C -3.863287 1.637831 0.067365
C -2.595011 2.079511 -0.058845
O -0.726068 -2.444822 -0.035568
O -3.255251 -1.986514 -0.030099
O -4.516860 0.085162 1.699276
C -5.399325 -0.274154 -0.573401
C -5.968159 -1.636828 -0.184516
C -6.240551 -2.623939 -1.292972
O -6.247353 -1.886031 0.981231
O -4.973918 2.411000 0.072292
C -4.814192 3.822260 -0.001457
C 4.769245 0.816383 -0.288672
C 5.008362 2.307928 -0.339777
C 5.755518 -0.105091 -0.295883
C 7.245756 0.109028 -0.354755
C 7.904556 -0.070443 1.042382
C 7.717047 -1.441788 1.702678
C 7.873736 -0.819470 -1.412983
H 1.774586 -2.518689 -0.098329
H 2.472418 2.328376 -0.229422
H 0.005777 2.749583 -0.169631
H -2.383653 3.138017 -0.164129
H -1.710489 -2.615105 -0.025958
H -5.131020 -0.677236 1.794657
H -5.104950 -0.257681 -1.627987
H -6.202352 0.459405 -0.441716
H -6.749808 -3.503688 -0.895051
H -6.847845 -2.163708 -2.082505
H -5.286234 -2.920970 -1.743163
H -4.219880 4.192508 0.842544
H -4.334046 4.117372 -0.943106
H -5.820607 4.240577 0.044479
H 4.610292 2.806759 0.553537
H 6.070513 2.550482 -0.401222
H 4.514403 2.764770 -1.207266
H 5.433855 -1.143077 -0.249405
H 7.461379 1.139949 -0.660859
H 8.979010 0.136174 0.935558
H 7.505145 0.703927 1.710236
H 8.134600 -2.251088 1.093211
H 8.224807 -1.469179 2.673499
H 6.658853 -1.664850 1.877217
H 8.962101 -0.688518 -1.448730
H 7.472909 -0.602872 -2.409304
H 7.668007 -1.874293 -1.198442
SCF Energy (PCM/mPW1PW91/6-31+G**)= -1361.46667837
Number of imaginary frequencies = 0

1b_c204
B3LYP/6-31G* Geometry
C 1.879588 -2.520702 -0.142058
C 2.605393 0.109637 -0.143060
N 3.166425 -2.233802 -0.191968
C 0.852208 -1.551957 -0.074450
C 1.225207 -0.176724 -0.083250
C 3.541644 -0.918387 -0.169109
C 0.219581 0.830272 -0.051222
C -1.116723 0.499835 0.010575
C -1.512666 -0.885459 0.035498
C -0.534038 -1.896910 -0.023605
C -2.909672 -1.251505 0.101567
C -3.993543 -0.162737 0.328169
C -3.453979 1.243817 0.119900
C -2.141166 1.532064 0.021384
O -0.851931 -3.189576 -0.031625
O -3.296302 -2.431837 0.034971
O -4.360288 -0.321041 1.693416
C -5.181214 -0.464975 -0.623747
C -6.478847 0.248399 -0.271568
C -7.280905 0.833309 -1.410195
O -6.877788 0.305858 0.884357
O -4.464420 2.151075 0.115439
C -4.134530 3.535804 0.097835
C 5.009234 -0.663779 -0.204180
C 5.833265 -1.726102 -0.893610
C 5.515168 0.434854 0.391695
C 6.947537 0.896790 0.484875
C 7.108760 2.317389 -0.108443
C 6.825070 2.404185 -1.611001
C 7.416720 0.868413 1.953737
H 1.612235 -3.575010 -0.157587
H 2.928097 1.144811 -0.189859
H 0.520508 1.874347 -0.075287
H -1.798683 2.558495 -0.055291
H -1.851233 -3.235953 -0.006215
H -5.310059 -0.079341 1.756652
H -5.368735 -1.543354 -0.542243
H -4.893652 -0.259938 -1.660426
H -8.231487 1.222518 -1.040524
H -6.705296 1.641383 -1.878229
H -7.461452 0.076747 -2.183952
H -3.519724 3.799878 0.966258
H -3.600349 3.802910 -0.822665
H -5.084090 4.070942 0.141714
H 5.518187 -1.846464 -1.937672
H 6.900618 -1.497498 -0.882606
H 5.673511 -2.695609 -0.412356
H 4.816830 1.090030 0.916175
H 7.595721 0.224116 -0.089557
H 6.445793 3.006984 0.434771
H 8.133248 2.660188 0.091076
H 5.801634 2.089337 -1.842588
H 6.955737 3.428193 -1.978994
H 7.505054 1.757220 -2.178908
H 8.457446 1.203919 2.037381
H 7.347593 -0.141409 2.371872
H 6.800841 1.530150 2.575947
SCF Energy (PCM/mPW1PW91/6-31+G**)= -1361.46655651
Number of imaginary frequencies = 0

1b_c226
B3LYP/6-31G* Geometry
C 2.126886 -1.633021 0.024696
C 2.359143 1.066660 -0.286651
N 3.333467 -1.124964 -0.092023
C 0.934541 -0.867118 0.000578
C 1.053658 0.540975 -0.161971
C 3.469923 0.230015 -0.249591
C -0.117480 1.349866 -0.196155
C -1.369504 0.789915 -0.068117
C -1.510921 -0.633700 0.099160
C -0.362436 -1.450953 0.125308
C -2.819214 -1.241030 0.224604
C -4.091633 -0.355328 0.284267
C -3.800672 1.109621 0.035615
C -2.567326 1.617387 -0.110924
O -0.438510 -2.772919 0.262154
O -2.978789 -2.471630 0.311504
O -4.596851 -0.496704 1.609989
C -5.095316 -0.903967 -0.769824
C -6.515320 -0.384563 -0.588851
C -7.217325 0.196283 -1.790964
O -7.072065 -0.479987 0.499000
O -4.931233 1.890341 -0.037276
C -5.212632 2.698597 1.115455
C 4.854376 0.757442 -0.378300
C 5.006433 2.250255 -0.560531
C 5.890300 -0.106363 -0.331668
C 7.366204 0.162029 -0.452782
C 8.119700 -0.333379 0.805426
C 7.749705 0.412968 2.090546
C 7.916228 -0.530701 -1.717427
H 2.058287 -2.711887 0.147440
H 2.474673 2.137398 -0.411399
H -0.013105 2.423841 -0.325561
H -2.442025 2.686004 -0.266077
H -1.413021 -2.995889 0.321525
H -5.569392 -0.611783 1.535413
H -5.121068 -1.992272 -0.632427
H -4.722263 -0.700028 -1.778479
H -8.252765 0.437455 -1.542813
H -6.689027 1.104179 -2.105776
H -7.187805 -0.507709 -2.631886
H -5.311465 2.069883 2.004979
H -4.423651 3.443339 1.273821
H -6.156408 3.206422 0.906548
H 4.575105 2.802986 0.284211
H 6.052841 2.547063 -0.644856
H 4.491628 2.597289 -1.466018
H 5.630459 -1.155856 -0.199283
H 7.556456 1.238285 -0.550067
H 7.926970 -1.408220 0.933359
H 9.198378 -0.234869 0.621249
H 6.682721 0.311321 2.316916
H 8.314266 0.026753 2.946723
H 7.970346 1.484356 2.003472
H 8.993563 -0.352853 -1.819452
H 7.420450 -0.159533 -2.620790
H 7.757257 -1.615079 -1.670981
SCF Energy (PCM/mPW1PW91/6-31+G**)= -1361.45991261
Number of imaginary frequencies = 0

1b_c259
B3LYP/6-31G* Geometry
C -1.926084 -2.388030 -0.064258
C -2.560290 0.237754 -0.451943
N -3.200655 -2.071161 -0.188660
C -0.867175 -1.452339 -0.109433
C -1.192197 -0.080749 -0.317859
C -3.530287 -0.754518 -0.361588
C -0.153947 0.890388 -0.388200
C 1.169683 0.526969 -0.266321
C 1.518292 -0.856204 -0.061827
C 0.504480 -1.829909 0.029309
C 2.901472 -1.256667 0.066412
C 4.040120 -0.231302 -0.185483
C 3.530540 1.200650 -0.234944
C 2.225189 1.526338 -0.313298
O 0.775004 -3.116040 0.240953
O 3.238471 -2.425034 0.327599
O 4.548726 -0.587607 -1.464996
C 5.105541 -0.418118 0.927705
C 6.457086 0.215237 0.629213
C 7.148972 0.941094 1.759060
O 6.982385 0.100349 -0.470515
O 4.566203 2.078658 -0.252874
C 4.284630 3.457963 -0.465466
C -4.986532 -0.461433 -0.477484
C -5.824352 -1.574264 -1.062657
C -5.469863 0.720937 -0.046136
C -6.879850 1.252881 -0.095571
C -7.400819 1.586944 1.322186
C -7.536979 0.370384 2.242244
C -6.932176 2.503299 -0.997977
H -1.695034 -3.441620 0.076663
H -2.845879 1.265576 -0.651718
H -0.419287 1.933737 -0.537139
H 1.912561 2.560046 -0.415221
H 1.769691 -3.186715 0.324247
H 5.507912 -0.377353 -1.452197
H 5.264841 -1.500383 1.017480
H 4.713173 -0.063887 1.886956
H 8.145787 1.259211 1.447794
H 6.552307 1.815890 2.045515
H 7.221086 0.296911 2.644258
H 3.768946 3.606544 -1.421428
H 3.671125 3.866035 0.347612
H 5.251958 3.961761 -0.485956
H -5.704546 -2.485457 -0.469179
H -6.884445 -1.318084 -1.108224
H -5.487064 -1.821921 -2.077100
H -4.763590 1.415785 0.412517
H -7.549623 0.499913 -0.528173
H -6.727888 2.325642 1.782388
H -8.376135 2.083339 1.224109
H -6.575379 -0.136319 2.378391
H -7.907767 0.663449 3.231024
H -8.240532 -0.362037 1.827293
H -7.951388 2.904915 -1.048184
H -6.605215 2.270646 -2.017117
H -6.280044 3.296049 -0.609430
SCF Energy (PCM/mPW1PW91/6-31+G**)= -1361.46647703
Number of imaginary frequencies = 0

1b_c319
B3LYP/6-31G* Geometry
C 1.769437 -2.570332 -0.108856
C 2.534115 0.044408 -0.269136
N 3.059063 -2.307290 -0.199259
C 0.758158 -1.582940 -0.074718
C 1.151065 -0.216422 -0.168711
C 3.453846 -0.998453 -0.254311
C 0.160799 0.806151 -0.175725
C -1.178567 0.500510 -0.068721
C -1.593924 -0.874616 0.045882
C -0.631377 -1.902814 0.024960
C -2.994072 -1.214358 0.164448
C -4.056402 -0.097133 0.350743
C -3.502070 1.285574 0.044247
C -2.187861 1.547208 -0.099239
O -0.967726 -3.188743 0.099137
O -3.398957 -2.390364 0.176373
O -4.390863 -0.166991 1.731693
C -5.272075 -0.438167 -0.551167
C -6.549124 0.317929 -0.213269
C -7.371102 0.842689 -1.366822
O -6.917552 0.455023 0.946070
O -4.499638 2.206193 0.004902
C -4.151042 3.581841 -0.107292
C 4.923906 -0.768239 -0.323123
C 5.719623 -1.865188 -0.991231
C 5.454857 0.340568 0.229989

C 6.896549 0.779607 0.280905
C 7.038636 2.181761 -0.362037
C 8.482170 2.682713 -0.488008
C 7.396466 0.754051 1.740239
H 1.486017 -3.619455 -0.060875
H 2.872765 1.069698 -0.378947
H 0.475970 1.842311 -0.266373
H -1.832938 2.561691 -0.245855
H -1.966669 -3.218099 0.149496
H -5.334780 0.094618 1.803158
H -5.473917 -1.505939 -0.396966
H -5.007615 -0.303838 -1.605425
H -8.304103 1.274034 -0.999172
H -6.794043 1.606396 -1.902610
H -7.586486 0.040365 -2.083514
H -3.514237 3.891028 0.729832
H -3.632883 3.782079 -1.053573
H -5.091960 4.133014 -0.078107
H 5.391009 -2.008195 -2.028421
H 6.791519 -1.658380 -0.997832
H 5.545818 -2.817319 -0.481063
H 4.774820 1.021502 0.745010
H 7.516769 0.087387 -0.301261
H 6.581846 2.150554 -1.359933
H 6.448563 2.903426 0.221981
H 9.098256 1.975995 -1.057899
H 8.514185 3.645509 -1.010661
H 8.955867 2.824306 0.489322
H 8.452538 1.035754 1.811026
H 7.286126 -0.246777 2.170781
H 6.819953 1.450330 2.363015
SCF Energy (PCM/mPW1PW91/6-31+G**)= -1361.46563990
Number of imaginary frequencies = 0

1b_c336
B3LYP/6-31G* Geometry
C 1.856891 -2.595297 -0.091880
C 2.618042 0.022395 -0.208992
N 3.146943 -2.328377 -0.163384
C 0.843176 -1.610555 -0.056227
C 1.234750 -0.242231 -0.126021
C 3.540356 -1.018469 -0.197805
C 0.243136 0.779067 -0.126913
C -1.096717 0.470103 -0.037577
C -1.511217 -0.907274 0.049445
C -0.546994 -1.933833 0.022295
C -2.912101 -1.250663 0.146990
C -3.978066 -0.138258 0.341442
C -3.422072 1.250707 0.067954
C -2.106886 1.516168 -0.058467
O -0.882369 -3.221156 0.071367
O -3.315451 -2.427213 0.133836
O -4.329160 -0.234406 1.716548
C -5.182254 -0.463172 -0.581802
C -6.464176 0.285014 -0.244549
C -7.270187 0.836730 -1.396760
O -6.848247 0.395509 0.912576
O -4.420062 2.171187 0.038062
C -4.071697 3.549306 -0.039473
C 5.011102 -0.786437 -0.249117
C 5.816755 -1.886351 -0.900008
C 5.535415 0.329983 0.296546
C 6.980838 0.762808 0.348855
C 7.298744 1.847875 -0.718070
C 6.497492 3.150740 -0.611069
C 7.351712 1.223227 1.771668
H 1.575377 -3.645592 -0.061488
H 2.953646 1.050290 -0.302372
H 0.557739 1.816981 -0.197314
H -1.751638 2.533611 -0.182115
H -1.881851 -3.252533 0.109538
H -5.274879 0.022338 1.781341
H -5.384580 -1.533934 -0.450795
H -4.905080 -0.307929 -1.629891
H -8.208167 1.259711 -1.032059
H -6.685641 1.612571 -1.906339
H -7.475645 0.051495 -2.134960
H -3.442624 3.839461 0.810211
H -3.545358 3.771615 -0.976253
H -5.013321 4.099023 -0.006024
H 5.503424 -2.030927 -1.941665
H 6.888139 -1.676860 -0.894490
H 5.636072 -2.838082 -0.391600
H 4.846012 1.017964 0.786266
H 7.621800 -0.093272 0.108484
H 8.371575 2.078852 -0.654792
H 7.134317 1.409069 -1.710717
H 6.658720 3.655662 0.348155
H 6.795171 3.848857 -1.401617
H 5.421586 2.972031 -0.719229
H 8.396476 1.553814 1.815215
H 7.223684 0.406783 2.490690
H 6.722503 2.056771 2.105612
SCF Energy (PCM/mPW1PW91/6-31+G**)= -1361.46417274
Number of imaginary frequencies = 0

1b_c353
B3LYP/6-31G* Geometry
C -1.905908 -2.458811 0.075782
C -2.573739 0.116249 -0.533791
N -3.183856 -2.172261 -0.082961
C -0.860178 -1.514135 -0.038055
C -1.202346 -0.169971 -0.364772
C -3.530217 -0.878737 -0.364224
C -0.177033 0.806862 -0.508463
C 1.150150 0.475192 -0.342436
C 1.515927 -0.880007 -0.016527
C 0.515000 -1.857765 0.146303
C 2.902869 -1.246977 0.160801
C 4.030165 -0.229463 -0.163394
C 3.500711 1.185017 -0.341916
C 2.191947 1.482776 -0.463107
O 0.801187 -3.116991 0.469554
O 3.253238 -2.383840 0.523868
O 4.562283 -0.685478 -1.400972
C 5.081720 -0.304992 0.975708
C 6.428400 0.321613 0.643466
C 7.091844 1.151238 1.717495
O 6.971586 0.123343 -0.435491
O 4.524029 2.073916 -0.423441
C 4.225701 3.425531 -0.756787
C -4.989438 -0.615426 -0.501188
C -5.814828 -1.781020 -0.993843
C -5.491907 0.586297 -0.152604
C -6.924849 1.058325 -0.219142
C -7.629826 0.968686 1.162736
C -6.999801 1.793876 2.291324
C -6.991681 2.478503 -0.813333
H -1.660956 -3.493797 0.304082
H -2.873526 1.119556 -0.819386
H -0.455488 1.829773 -0.747883
H 1.866112 2.499078 -0.656998
H 1.795782 -3.165463 0.568901
H 5.517615 -0.458279 -1.394288
H 5.254804 -1.373386 1.158422
H 4.670218 0.122465 1.896355
H 8.089987 1.455128 1.396407
H 6.479085 2.039157 1.916463
H 7.155807 0.587339 2.656426
H 3.719210 3.483888 -1.727317
H 3.597054 3.892634 0.011741
H 5.186038 3.940151 -0.810498
H -5.705708 -2.630868 -0.313183
H -6.874760 -1.533610 -1.080140
H -5.459247 -2.125211 -1.973082
H -4.796674 1.320109 0.255749
H -7.488181 0.400913 -0.891933
H -8.676271 1.278147 1.029863
H -7.654528 -0.086248 1.464731
H -6.989927 2.865448 2.061154
H -7.566491 1.665309 3.220430
H -5.968164 1.482665 2.490117

H -8.027575 2.836444 -0.852503
H -6.589159 2.494491 -1.832271
H -6.412756 3.195220 -0.218984
SCF Energy (PCM/mPW1PW91/6-31+G**)= -1361.46425998
Number of imaginary frequencies = 0

1b_c367
B3LYP/6-31G* Geometry
C 1.564231 -2.420186 -0.028440
C 2.555188 0.117980 -0.176741
N 2.874024 -2.269117 -0.074598
C 0.640819 -1.349731 -0.034091
C 1.152504 -0.022260 -0.121828
C 3.381408 -0.999479 -0.123376
C 0.255356 1.082343 -0.168894
C -1.107859 0.894526 -0.099553
C -1.641482 -0.439599 0.010929
C -0.773559 -1.547600 0.017141
C -3.069210 -0.646982 0.069931
C -4.022204 0.553072 0.281185
C -3.359275 1.883090 -0.034714
C -2.024070 2.022355 -0.166677
O -1.224410 -2.799895 0.074191
O -3.584025 -1.783768 0.023564
O -4.319469 0.595299 1.673869
C -5.298535 0.339177 -0.572977
C -6.151503 -0.841469 -0.114951
C -6.665860 -1.785196 -1.174265
O -6.453457 -0.973062 1.064318
O -4.268001 2.884731 -0.080185
C -3.795671 4.219196 -0.218988
C 4.867455 -0.897596 -0.142942
C 5.589136 -2.075093 -0.755716
C 5.474094 0.175484 0.403520
C 6.944842 0.491484 0.507376
C 7.263117 1.856386 -0.149267
C 7.024172 1.894504 -1.661575
C 7.380234 0.489042 1.986980
H 1.190602 -3.440934 0.014320
H 2.982901 1.109983 -0.280936
H 0.662148 2.086179 -0.258070
H -1.580220 3.000001 -0.319946
H -2.222060 -2.743397 0.072680
H -5.086095 -0.006053 1.809912
H -5.026971 0.240632 -1.629157
H -5.914410 1.239606 -0.471932
H -7.351213 -2.509717 -0.730418
H -7.171919 -1.231057 -1.974792
H -5.814544 -2.308828 -1.624463
H -3.135434 4.488043 0.614319
H -3.259435 4.352493 -1.167114
H -4.682845 4.853769 -0.206328
H 5.270207 -2.229586 -1.794015
H 6.673261 -1.948308 -0.746159
H 5.334965 -2.993393 -0.217724
H 4.838281 0.926738 0.876265
H 7.530239 -0.272547 -0.018253
H 6.661314 2.635536 0.341554
H 8.312338 2.105163 0.061470
H 5.980835 1.667457 -1.906978
H 7.261798 2.881679 -2.074000
H 7.651685 1.158260 -2.178910
H 8.448138 0.720751 2.079681
H 7.199342 -0.486210 2.451138
H 6.823834 1.240448 2.561589
SCF Energy (PCM/mPW1PW91/6-31+G**)= -1361.46706957
Number of imaginary frequencies = 0

1b_c376
B3LYP/6-31G* Geometry
C 2.141624 -1.533888 -0.228743
C 2.319355 1.185058 -0.116536
N 3.338156 -0.990904 -0.260996
C 0.934215 -0.797328 -0.138052
C 1.023478 0.621428 -0.078986
C 3.446188 0.375141 -0.206272
C -0.162211 1.402256 0.008146
C -1.405111 0.806525 0.050639
C -1.514262 -0.628204 0.000195
C -0.350649 -1.417407 -0.107765
C -2.807895 -1.271106 0.043219
C -4.089354 -0.436338 0.313198
C -3.842665 1.059887 0.198115
C -2.615185 1.610482 0.119779
O -0.402092 -2.746126 -0.181408
O -2.949443 -2.500533 -0.082285
O -4.430308 -0.748504 1.657972
C -5.184114 -0.910675 -0.680030
C -6.600095 -0.486668 -0.316115
C -7.486772 0.006155 -1.435391
O -7.016310 -0.585350 0.830848
O -5.014060 1.744856 0.248926
C -4.968427 3.165484 0.329018
C 4.819732 0.943235 -0.246962
C 4.942741 2.448889 -0.192517
C 5.874258 0.104315 -0.324250
C 7.342663 0.438865 -0.373989
C 8.026063 0.217467 1.005106
C 7.950481 -1.205983 1.570866
C 8.032652 -0.361651 -1.496214
H 2.094266 -2.619983 -0.273988
H 2.413666 2.264246 -0.071965
H -0.077963 2.485393 0.040516
H -2.485744 2.687439 0.106032
H -1.371122 -2.994098 -0.159243
H -5.409732 -0.706026 1.714565
H -5.155674 -2.007725 -0.660538
H -4.931688 -0.593484 -1.697462
H -8.496798 0.188708 -1.063559
H -7.068152 0.933005 -1.846485
H -7.515577 -0.724423 -2.253467
H -4.408641 3.486644 1.215228
H -4.509831 3.598151 -0.569063
H -6.005394 3.494736 0.408235
H 4.513233 2.851121 0.734308
H 5.982687 2.776288 -0.237361
H 4.409397 2.926424 -1.024854
H 5.634626 -0.956398 -0.349465
H 7.473460 1.502176 -0.609447
H 9.080294 0.514003 0.909097
H 7.573573 0.911201 1.725662
H 8.423518 -1.937202 0.905821
H 8.467024 -1.260114 2.535904
H 6.913888 -1.521041 1.732682
H 9.106288 -0.139710 -1.528503
H 7.604837 -0.111580 -2.473285
H 7.915906 -1.441813 -1.353556
SCF Energy (PCM/mPW1PW91/6-31+G**)= -1361.46617786
Number of imaginary frequencies = 0

1b_c384
B3LYP/6-31G* Geometry
C 1.880775 -1.537829 -0.078367
C 2.327749 1.147677 -0.238358
N 3.125561 -1.121537 -0.145709
C 0.752472 -0.680517 -0.080843
C 0.981959 0.721440 -0.165719
C 3.368962 0.225670 -0.226017
C -0.120740 1.620332 -0.184201
C -1.416924 1.157672 -0.099748
C -1.665515 -0.256983 0.003283
C -0.587757 -1.164278 -0.010770
C -3.019226 -0.752396 0.076653
C -4.196705 0.226028 0.300561
C -3.821501 1.666223 -0.005431
C -2.544091 2.076134 -0.145744
O -0.772539 -2.483084 0.042842
O -3.291395 -1.970476 0.030583
O -4.492984 0.196088 1.693542
C -5.404221 -0.237406 -0.554898
C -5.997973 -1.570356 -0.105409
C -6.305490 -2.594611 -1.170075

O -6.268204 -1.767080 1.072493
O -4.915961 2.461588 -0.035273
C -4.727125 3.864938 -0.168485
C 4.792804 0.648934 -0.300050
C 5.064678 2.132958 -0.391693
C 5.757365 -0.294862 -0.286682
C 7.252054 -0.137381 -0.365884
C 7.937815 -0.774478 0.867013
C 7.605491 -0.084339 2.193218
C 7.772883 -0.784906 -1.666384
H 1.725614 -2.612972 -0.015999
H 2.528686 2.211058 -0.303467
H 0.071125 2.686749 -0.268900
H -2.310483 3.124954 -0.292724
H -1.760406 -2.631525 0.056541
H -5.121097 -0.549359 1.825672
H -5.119585 -0.270658 -1.611727
H -6.190439 0.517936 -0.447132
H -6.832246 -3.444452 -0.731955
H -6.908668 -2.152179 -1.972781
H -5.362934 -2.933555 -1.615493
H -4.126963 4.258635 0.660623
H -4.239309 4.109821 -1.120549
H -5.724851 4.305244 -0.143136
H 4.662444 2.668422 0.478115
H 6.132484 2.350423 -0.444827
H 4.595269 2.571272 -1.282083
H 5.413983 -1.326467 -0.221382
H 7.528743 0.924335 -0.385380
H 7.655059 -1.835495 0.921146
H 9.024381 -0.754357 0.705010
H 6.529723 -0.112223 2.397950
H 8.120648 -0.569665 3.029900
H 7.913923 0.968646 2.178999
H 8.862692 -0.685855 -1.739490
H 7.325973 -0.316168 -2.549688
H 7.529526 -1.854071 -1.696580
SCF Energy (PCM/mPW1PW91/6-31+G**)= -1361.46888358
Number of imaginary frequencies = 0

1b_c422
B3LYP/6-31G* Geometry
C 1.819389 -1.509480 -0.044539
C 2.248665 1.180348 -0.176189
N 3.061726 -1.083883 -0.096625
C 0.685042 -0.660165 -0.048742
C 0.905412 0.744043 -0.119586
C 3.296453 0.265720 -0.161607
C -0.203481 1.635317 -0.139561
C -1.496955 1.162959 -0.069082
C -1.736445 -0.254214 0.020245
C -0.652213 -1.153811 0.006680
C -3.087082 -0.759576 0.079584
C -4.272903 0.208800 0.303550
C -3.905663 1.653993 0.011704
C -2.630196 2.073901 -0.116027
O -0.828118 -2.474323 0.047692
O -3.350519 -1.979057 0.021168
O -4.578425 0.165449 1.694181
C -5.471391 -0.256141 -0.563623
C -6.058142 -1.597127 -0.129113
C -6.350929 -2.614875 -1.204111
O -6.335007 -1.805432 1.045278
O -5.005453 2.442004 -0.019836
C -4.825335 3.847542 -0.141091
C 4.717800 0.699939 -0.214519
C 4.979313 2.186907 -0.285942
C 5.688853 -0.236840 -0.196026
C 7.183313 -0.062896 -0.232781
C 7.799704 -0.634708 1.069747
C 9.310897 -0.418618 1.209752
C 7.750335 -0.747456 -1.494256
H 1.671335 -2.586247 0.006102
H 2.442512 2.245696 -0.229629
H -0.018495 2.703742 -0.213656
H -2.402905 3.125477 -0.252857
H -1.814940 -2.629848 0.052629
H -5.202027 -0.585552 1.815938
H -5.179757 -0.278441 -1.618834
H -6.263836 0.492488 -0.454616
H -5.403182 -2.942067 -1.647218
H -6.872989 -3.472731 -0.776114
H -6.953486 -2.170663 -2.006323
H -4.233930 4.239072 0.695308
H -4.332255 4.102912 -1.087676
H -5.826217 4.280851 -0.119764
H 4.564748 2.708976 0.586314
H 6.045864 2.413570 -0.326385
H 4.515082 2.632678 -1.175339
H 5.352774 -1.271288 -0.144839
H 7.446609 1.001309 -0.281100
H 7.290400 -0.169354 1.923532
H 7.572525 -1.708700 1.128433
H 9.568475 0.645756 1.138855
H 9.664457 -0.779925 2.182182
H 9.877674 -0.950261 0.437837
H 8.835401 -0.617840 -1.567891
H 7.299409 -0.328974 -2.400469
H 7.538208 -1.823963 -1.482719
SCF Energy (PCM/mPW1PW91/6-31+G**)= -1361.46798125
Number of imaginary frequencies = 0
